# Characteristics of Serum Metabolites and Gut Microbiota in Diabetic Kidney Disease

**DOI:** 10.3389/fphar.2022.872988

**Published:** 2022-04-14

**Authors:** Bo Zhang, Yuzhou Wan, Xuefeng Zhou, Haojun Zhang, Hailing Zhao, Liang Ma, Xi Dong, Meihua Yan, Tingting Zhao, Ping Li

**Affiliations:** ^1^ Graduate School of Peking Union Medical College, Chinese Academy of Medical Sciences and Peking Union Medical College, Beijing, China; ^2^ Beijing Key Laboratory for Immune-Mediated Inflammatory Diseases, Institute of Clinical Medical Sciences, China–Japan Friendship Hospital, Beijing, China

**Keywords:** gut microbiota, diabetic kidney disease, carbohydrate metabolism, lipid metabolism, serum metabolites, amino acid metabolism

## Abstract

Disturbance of circulating metabolites and disorders of the gut microbiota are involved in the progression of diabetic kidney disease (DKD). However, there is limited research on the relationship between serum metabolites and gut microbiota, and their involvement in DKD. In this study, using an experimental DKD rat model induced by combining streptozotocin injection and unilateral nephrectomy, we employed untargeted metabolomics and 16S rRNA gene sequencing to explore the relationship between the metabolic profile and the structure and function of gut microbiota. Striking alterations took place in 140 serum metabolites, as well as in the composition and function of rat gut microbiota. These changes were mainly associated with carbohydrate, lipid, and amino acid metabolism. In these pathways, isomaltose, D-mannose, galactonic acid, citramalic acid, and prostaglandin B2 were significantly upregulated. 3-(2-Hydroxyethyl)indole, 3-methylindole, and indoleacrylic acid were downregulated and were the critical metabolites in the DKD model. Furthermore, the levels of these three indoles were restored after treatment with the traditional Chinese herbal medicine Tangshen Formula. At the genera level, *g_Eubacterium_nodatum_*group, *g_Lactobacillus*, and *g_Faecalibaculum* were most involved in metabolic disorders in the progression of DKD. Notably, the circulating lipid metabolites had a strong relationship with DKD-related parameters and were especially negatively related to the mesangial matrix area. Serum lipid indices (TG and TC) and UACR were directly associated with certain microbial genera. In conclusion, the present research verified the anomalous circulating metabolites and gut microbiota in DKD progression. We also identified the potential metabolic and microbial targets for the treatment of DKD.

## Introduction

Diabetic kidney disease (DKD) is one of the most prevalent microvascular complications of diabetes mellitus and is the main cause of end-stage renal disease in developed countries and the developed regions of China (Ma, 2018; Johansen et al., 2021). The poor prognosis of DKD severely reduces the quality of life and imposes a substantial financial burden on patients. The pathogenesis of DKD remains unclear, and there is a lack of comprehensive awareness of the progression of DKD (Winther et al., 2020; Jin and Ma, 2021), which severely restricts its prevention and diagnosis.

Metabolites are both the substrates and products of cellular basal metabolism. With advances in metabolomics, abnormal biological pathways can now be detected in the disease state, especially in DKD, which involves various metabolic pathways and complicated pathogenic mechanisms (Johnson et al., 2016; Rinschen et al., 2019). In addition, the metabolites are produced not only by the host organism but also by the gut microbiota in large portions, such as short chain fatty acids (Li et al., 2020a; Hu et al., 2020) and uremic toxins (Kikuchi et al., 2019; Winther et al., 2019), which are highly involved in DKD. The number of genes encoded by gut microorganisms is high in humans. Furthermore, the plethora of active metabolites, particularly those uniquely produced by gut microbiota, have profound implications on the health of the host.

In the present research, we used untargeted metabolomics and 16S rRNA gene sequencing to explore the relationship and possible mechanisms between the shift in host serum metabolic profile and intestinal flora disorder in the experimental DKD rat model induced by combining streptozotocin injection and unilateral nephrectomy. The results indicated that disorders in the serum metabolites and gut microbiota principally involved carbohydrate, lipid, and amino acid metabolism. This was verified by the restoration of the serum metabolites and gut microbiota after treatment with the traditional Chinese herbal medicine Tangshen Formula (TSF).

## Methods

### Animals and Experimental Design

This study was conducted in the Experimental Animal Center of the Institute of Clinical Medicine, China–Japan Friendship Hospital [Beijing, China, permit number: SYXK (Jing) 2016-0043]. Adult (6- to 8-weeks old) male Wistar rats (180–220 g) were purchased from Beijing Huafukang Biotechnology Co. Ltd. [Beijing, China, license number: SCXK (Jing) 2019-0008]. The animals of each experimental group were raised together in specific-pathogen-free (SPF) environment with controlled temperature and humidity (20–25°C, 65–75% humidity) and maintained under light–dark cycle (12 h light/12 h dark). The rats were given free access to standard laboratory animal feed and water.

TSF was extracted from the following seven herbs: astragalus root [*Astragalus membranaceus* (Fisch.) Bge.], burning bush twig [*Euonymus alatus* (Thunb.) Sieb.], rehmannia root (*Rehmannia glutinosa* Libosch.), bitter orange (*Citrus aurantium* L.), cornus fruit (*Cornus officinalis* Sieb. et Zucc), rhubarb root and rhizome (*Rheum palmatum* L.), and notoginseng root [*Panax notoginseng* (Burk.) F.H. Chen] (Li et al., 2015). TSF preparation was conducted by the Beijing Institute of Clinical Pharmacy (Beijing, China) and quality control performed according to the criteria published in the *Chinese Pharmacopoeia* (2015 edition).

Following an adaptation period of 1 week, 30 rats were randomly divided into a sham group and a DKD-model group based on weight (10 and 20 rats, respectively). The DKD-model group underwent unilateral nephrectomy. The sham group was subjected to sham surgery that involved opening the abdominal cavity and exposing the kidney through stripping the renal capsule. Following a period of 1 week after surgery, the DKD rats were administered streptozotocin intraperitoneally (STZ; 40 mg/kg; Sigma-Aldrich, St. Louis, MO, United States) as previously described (Tang et al., 2019). The sham rats were injected with the same volume of citrate buffer solution (0.1 mol/L, pH = 4.2). Blood glucose was detected after 3 days and animals with blood glucose levels >16.7 mmol/L were designated as successful models. A total of 20 rats met the criteria for model building and were separated into two equal groups (DKD and TSF).

A dose of TSF of 4.8 g/kg/d was equivalent to 85 g/d of the raw herbs for DKD patients (Zhang et al., 2011). The sham and DKD rats received gavages of solvent (CMC-Na aqueous solution) only. After 20 weeks, all rats were housed in metabolic cages (Suzhou Fengshi Laboratory Animal Equipment, Jiangsu Province, China), and their urine was collected for 24 h under fasting condition except for access to water. All animals were then anesthetized by intraperitoneal injection of 1% sodium pentobarbital (1 ml/kg; Sigma-Aldrich), and then sacrificed. The colon contents, serum, and renal tissues were collected for subsequent examinations. The protocol in this study was approved by the Ethics Committee of the China–Japan Friendship Hospital (No. 180115).

### Histology and Pathologic Analysis of Renal Tissue

The renal tissues preserved in 10% neutral formalin were dehydrated, embedded in paraffin, and sectioned to 3-μm thick sections. They were subjected to periodic acid–Schiff (PAS), Masson trichrome, and hematoxylin and eosin (H&E) staining. Under ×400 magnification, 20 glomeruli from each rat were randomly chosen to view the mesangial matrices that had been stained with PAS. This was followed by semiquantitative analysis using the Image-Pro Plus 6.0 software (Media Cybernetics, Rockville, MD, United States). Under ×200 magnification, 10 areas were randomly selected from each sample, and a semiquantitative analysis of the area of collagen fiber, which had been stained with Masson trichrome, was performed. Inflammatory infiltration, which was highlighted using H&E staining, was assessed using Image-Pro Plus 6.0 software. The scoring criteria of inflammatory infiltration was: 0, no inflammatory infiltration; 1, <25%; 2, 25–50%; 3, 50–75%; and 4, >75% of renal tubular injury.

### Untargeted Analysis of Serum Metabolites

The serum samples were added to the extract solvent, and the extraction was diluted after grinding, ultrasonic processing, and centrifugation. The supernatant was further separated using an Agilent 1290 Infinity II series UHPLC System (Agilent Technologies, Santa Clara, CA, United States), equipped with a Waters ACQUITY UPLC BEH Amide column (100 × 2.1 mm, 1.7 μm). Then, mass spectrometry data were obtained using a Triple TOF 6600 mass spectrometer (SCIEX, Redwood City, CA, United States). Data management was conducted as follows: The metabolite features that were only detected in < 20% of the experimental samples or in <50% of the QC samples were removed from subsequent analysis. The missing values of raw data were filled by half of the minimum value, and data normalization performed by internal standardization. Features with relative standard deviation (RSD) >30% were excluded. Then, the differential metabolites were filtered from statistical analysis. Markedly altered metabolites were determined based on the following criteria: 1) VIP value >1 using OPLS-DA analysis, and 2) *p*-value < 0.05 obtained by Student’s unpaired t-test. The metabolite pathways were searched using databases, including KEGG (http://www.kegg.jp), HMDB (http://www.hmdb.ca), and MetaboAnalyst (http://www.metaboanalyst.ca).

### Exploration of Gut Microbial Communities

Fresh colon contents were collected and immediately frozen at −80°C until tested. Microbial DNA was extracted using the PowerSoil DNA Isolation Kit (MO BIO Laboratories, Carlsbad, CA, United States), according to the manufacturer's instructions. The V3-4 regions of the 16S rRNA gene were amplified, and the PCR products were purified using an AMPure XP kit (Beckman Coulter Life Sciences, Indianapolis, IN, United States).

High-quality PCR products were sequenced on a MiSeq PE300 high-throughput sequencing system (Illumina, San Diego, CA, United States), and technical support was provided by Beijing Allwegene Technology (Beijing, China). Quality control processing of the raw data included removing sequences that were shorter than 230 bp using the UPARSE-OTU algorithm (v2.7.1; USEARCH software, https://drive5.com/uparse) and deleting chimeric sequences using the UCHIME algorithm (USEARCH; https://www.drive5.com/usearch/manual/uchime_algo.html) based on the “Gold database.” With a 97% sequence similarity level, clean tags were clustered into operational taxonomic units (OTUs) using UPARSE. The alpha diversity indices, namely, Chao1, Shannon, and Simpson were analyzed by the QIIME (v1.8.0; http://qiime.org/1.8.0) software to clarify diversity and abundance of the microbial community. Partial least-squares discrimination analysis (PLS-DA) was conducted for beta diversity to delineate the differences in intestinal flora between the groups.

### Statistical Analyses

The data involved in the present study were presented as means ± SEM. Statistical analyses were conducted using GraphPad Prism (v8.0.2; https://www.graphpad.com/scientific-software/prism), Origin software (2021; OrginLab, Northampton, MA, United States), and R software (v4.1.1; R Foundation, https://www.r-project.org/foundation). The significant differences between the two groups were analyzed by Student’s unpaired t-test or Mann–Whitney U test. The significant differences of genera were assessed using Wilcoxon rank-sum test and LEfSe analysis. The differential concentration of the serum metabolites was determined by the Student’s unpaired t-test and OPLS-DA models (VIP>1). *p*-value <0.05 was regarded as statistically significant.

## Results

### Physiologic and Biochemical Parameters Were Altered in Diabetic Kidney Disease Rats

**FIGURE 1:**
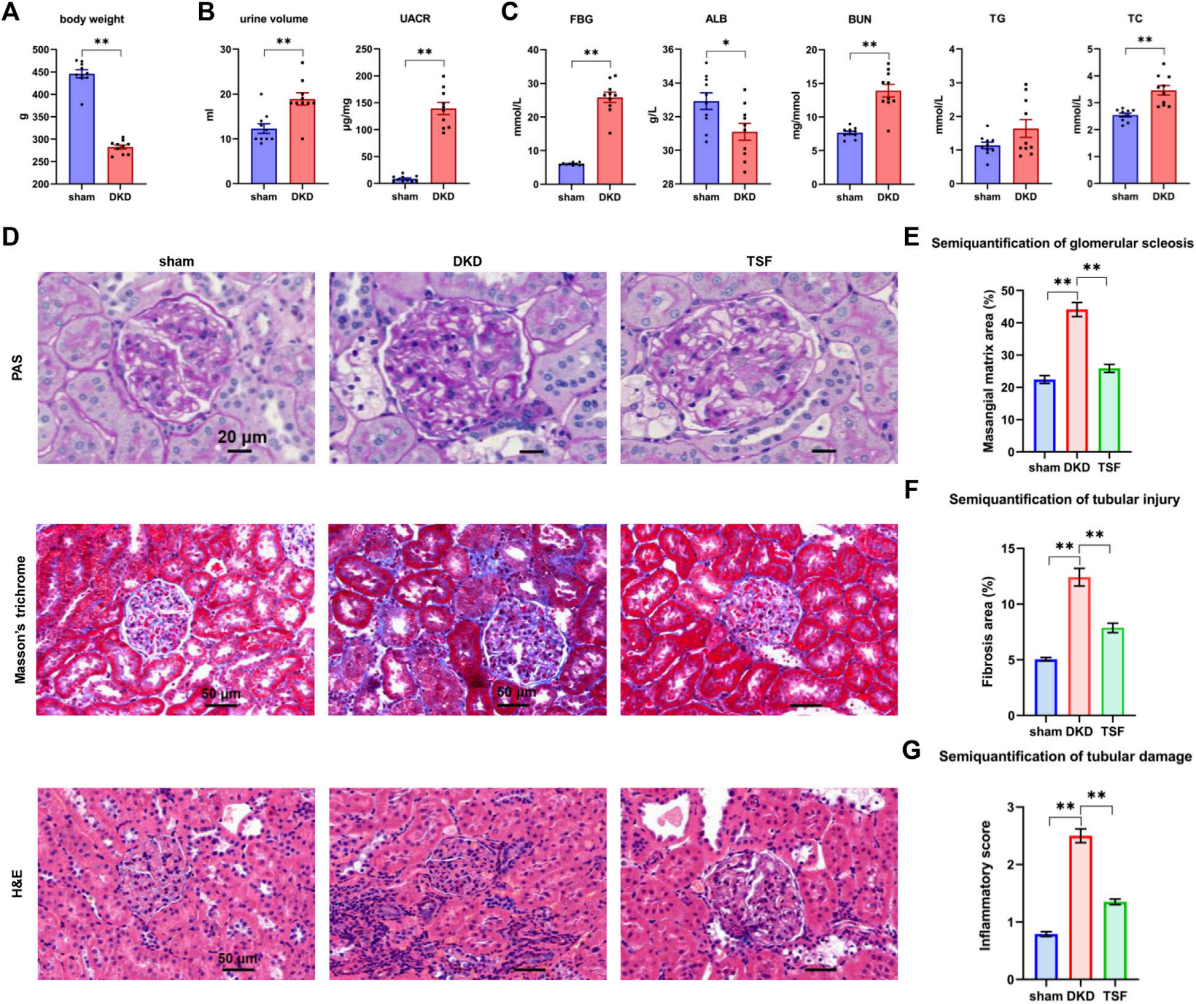
Significant shift in physiologic and biochemical parameters and kidney pathology in DKD rats, and the effects of TSF treatment on renal damage. **(A)** Body weight of rats in the sham and DKD groups. *p < 0.05, **p < 0.01, versus sham rats. **(B)** Urinary parameters of sham and DKD rats. **(C)** Biochemical parameters in rat serum in the sham and DKD groups. **(D)** H&E, Masson trichrome, and PAS staining. **(E–G)** Semi-quantification of mesangial matrix, fibrosis area, and inflammatory scores based on PAS, Masson trichrome, and H&E staining, respectively. *p < 0.05, **p < 0.01.

The body weight in the DKD rats was lower than that in the sham group after 20 weeks of TSF treatment (Figure 1A). Compared to the sham rats, the urine volume and urinary albumin to creatinine ratio (UACR) were markedly increased in the DKD group (Figure 1B). These outcomes were in line with typical characteristics of the diabetic rat model, including polyuria and emaciation (Committee of the Japan Diabetes Society on the Diagnostic Criteria of Diabetes Mellitus et al., 2010). Moreover, the rise in the UACR was a typical manifestation of renal impairment. The serum biochemical parameters, such as the fasting blood glucose (FBG), blood urea nitrogen (BUN), and total cholesterol (TC) levels were significantly increased in the DKD group. Albumin was reduced in DKD rats when compared with that in sham rats (Figure 1C).

### Renal Injury in Diabetic Kidney Disease Rats and the Effects of Tangshen Formula Treatment

PAS, Masson trichrome, and H&E staining of the renal tissue are shown in Figure 1D. In the sham group, the tubular epithelial cells were tightly arranged; there was no obvious inflammatory cell infiltration although a small amount of collagen fiber deposition was noted. In the DKD group, there were clear pathologic phenomena in the kidneys, including collagen fiber deposition, glomerular mesangial matrix expansion, glomerular basement membrane thickening, and moderate inflammatory cell infiltration. The aforementioned pathologies were significantly improved in the TSF group. Semi-quantification of renal damage showed that TSF treatment could significantly improve kidney damage in rats with DKD (Figures 1E–G).

### Changes in Serum Metabolite Profiling Between the Diabetic Kidney Disease and Sham Rats

**FIGURE 2:**
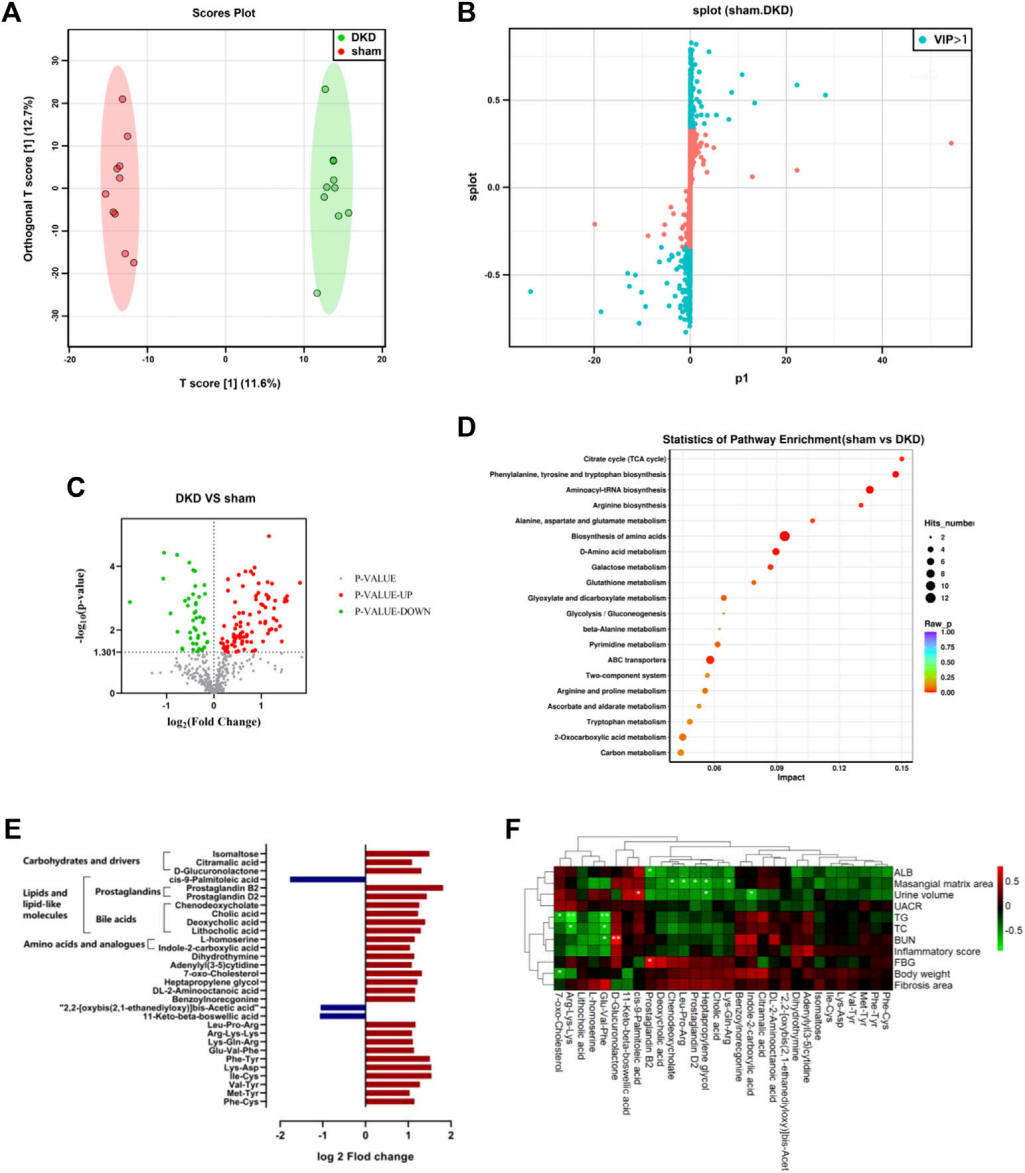
Significantly altered circulating metabolic profile in the DKD group. **(A)** OPLS-DA plots with scores of the first two principal components. **(B)** S-plots of the OPLS-DA model from sham and DKD rats. **(C)** Volcano plot of the different metabolites obtained according to the screening criteria from sham and DKD rats. **(D)** Bubble plot of the KEGG pathway enrichment analysis of 315 markedly different metabolites. **(E)** Further screening from differential serum metabolites according to the following condition: log2 fold change>1 or < −1. **(F)** Correlation analysis between base parameters related to DKD, semi-quantification of renal damage, and 30 differential serum metabolites using Spearman’s rank correlation (*p < 0.05, **p < 0.01).

The serum samples from the DKD and sham rats underwent untargeted metabolic analysis using UHPLC-QE-MS. A total of 476 metabolites were detected after data management. The OPLS-DA model was established between the sham and DKD groups. The R^2^Y value of this model was 0.994 and showed a good interpretation rate. The Q^2^ parameter of the model was 0.606 (*p* < 0.05), indicating that the model has good predictive ability. The result of the OPLS-DA score plots showed that the samples could be divided into two clusters (Figure 2A). The S-plot from the OPLS-DA analysis and 205 metabolites was selected with a VIP score >1 (Figure 2B). Among them, 140 differential metabolites were identified according to the selection criteria, which included 95 significantly upregulated and 45 downregulated substances (Figure 2C). Overall, these compounds mainly contained carbohydrates, lipids, amino acids, nucleosides, nucleotides, and their derivatives. Notably, all the carbohydrates, nucleosides, and nucleotides showed significantly elevated characteristics; 17 of 26 lipids and lipid-like molecules were increased; and 16 of 29 amino acids were decreased in the DKD serum (Table 1).

**TABLE 1:** Distribution and changes of differential metabolites in DKD serum.

		Total	Upregulated	Downregulated
Carbohydrates and drivers		15	15	0
Lipids and lipid-like molecules		26	17	9
Amino acids	Basic amino acid	7	2	5
	Other amino acid	11	7	4
	Tryptophan derivatives	8	2	6
	Amino acid derivatives	3	2	1
Nucleosides, nucleotides, and analogs		11	11	0

The KEGG pathway differential enrichment analysis of the metabolites was conducted, and the top 20 pathways based on the *p*-values were revealed (Figure 2D). The citric acid cycle had the highest impact value (0.15) among the 55 pathways enriched. The impact value of phenylalanine, tyrosine, and tryptophan biosynthesis, aminoacyl-tRNA biosynthesis, and arginine biosynthesis was 0.1471, 0.1346, and 0.1304, respectively. A total of 12 differentially expressed metabolites were involved in the biosynthesis of amino acids, and 8 differential metabolites were in ABC transporters.

Further filtering of the different metabolites was conducted based on the condition log2 fold change >1 or <−1, and 30 metabolites were ultimately screened out. The relative content of 27 substances was elevated in the DKD serum, which included products from carbohydrate metabolism (isomaltose, citramalic acid, and D-glucuronolactone), prostaglandins (B2 and D2) and bile acids (cholic acid, chenodeoxycholate, deoxycholic acid, and lithocholic acid), amino acids and analogs (L-homoserine and indole-2-carboxylic acid), among others. While three substances decreased in the DKD serum, cis-9-palmitoleic acid was greatly decreased among all serum metabolites in DKD rats (Figure 2E).

The Spearman correlation heat map showed the correlation between base parameters related to DKD, semi-quantification of renal damage, and serum differences. Prostaglandin B2, chenodeoxycholate, 7-oxo-cholesterol, indole-2-carboxylic acid, heptapropylene glycol, prostaglandin D2, and cis-9-palmitoleic acid had a strong correlation with DKD-related base parameters. In particular, lipid metabolites had conspicuous association with DKD-related parameters, including prostaglandin D2 and chenodeoxycholate, which were negatively related to the mesangial matrix area. Furthermore, 7-oxo-cholesterol was negatively related to body weight, while cis-9-palmitoleic acid was positively related to urine volume (Figure 2F).

### Analysis of Metabolites in Carbohydrate and Amino Acid Metabolism

**FIGURE 3:**
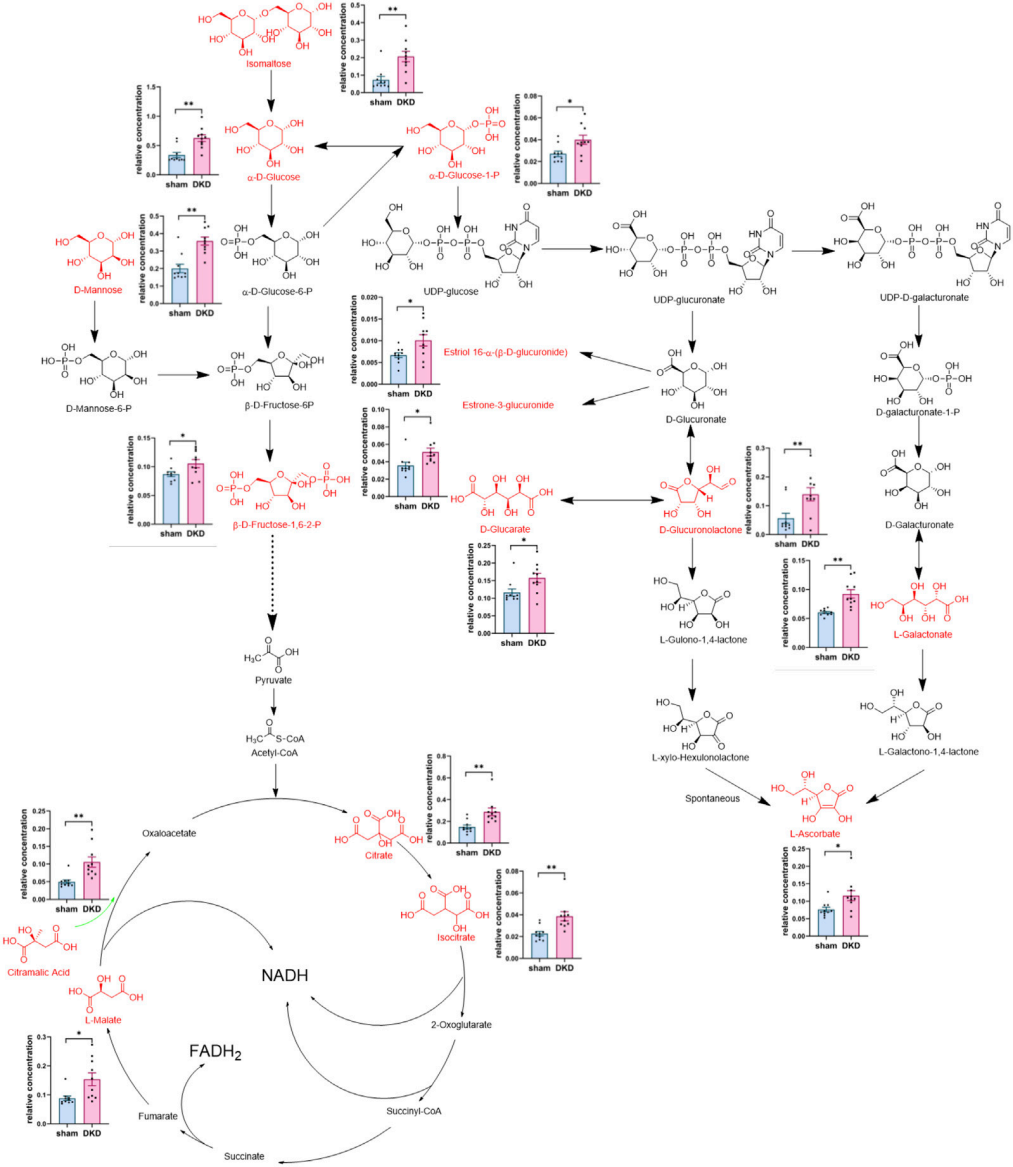
Analysis of 15 markedly elevated metabolites in carbohydrate metabolism. Note: Carbohydrates highlighted in red and their relative concentrations shown in column scatter plot (*p < 0.05, **p < 0.01).

**FIGURE 4:**
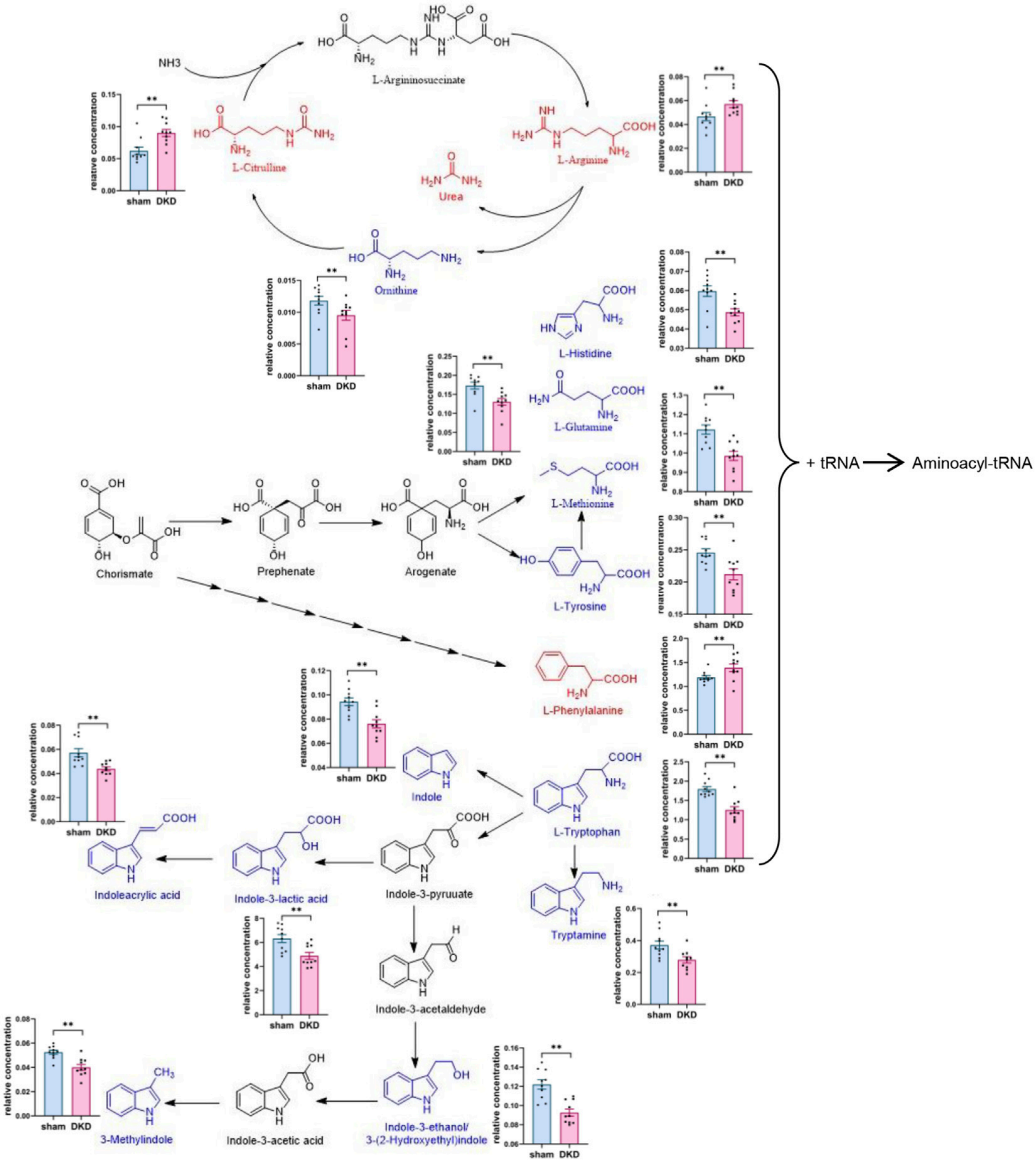
Analysis of amino acid biosynthesis and metabolism. Note: Elevated metabolites are highlighted in red, reduced metabolites are shown in blue, and their relative concentrations are shown in column scatter plot (*p < 0.05, **p < 0.01).

We further analyzed the metabolic pathways of significantly increased serum carbohydrates and found α-D-glucose, β-D-fructose-1,6-bisphosphate, and α-D-glucose-1-phosphate were involved in glycolysis/gluconeogenesis; citrate, isocitrate, L-malate, and citramalic acid participated in the citric acid cycle; isomaltose was involved in starch metabolism; D-mannose was involved in mannose metabolism; and D-glucuronolactone, D-glucarate, estriol 16α-(β-D-glucuronide), estrone-3-glucuronide, L-galactonate, and L-ascorbate were associated with ascorbate and aldarate metabolism (Figure 3). Similarly, we demonstrated the relationship between amino acid biosynthesis and metabolism, which had a higher impact value (Figure 4). The results indicated that L-arginine and L-citrulline were elevated, while ornithine declined in the ornithine cycle. Tryptophan and its derivatives, namely, tryptamine, indole, DL-indole-3-lactic acid, indoleacrylic acid, 3-(2-hydroxyethyl) indole, and 3-methylindole, were all decreased in the DKD serum. Among the aromatic amino acids, L-phenylalanine was raised, whereas tyrosine and methionine were reduced. L-glutamine and L-histidine were diminished in the DKD rats when compared with those in the sham rats.

### Comparison of Gut Microbiome Between Sham and Diabetic Kidney Disease Rats

**FIGURE 5:**
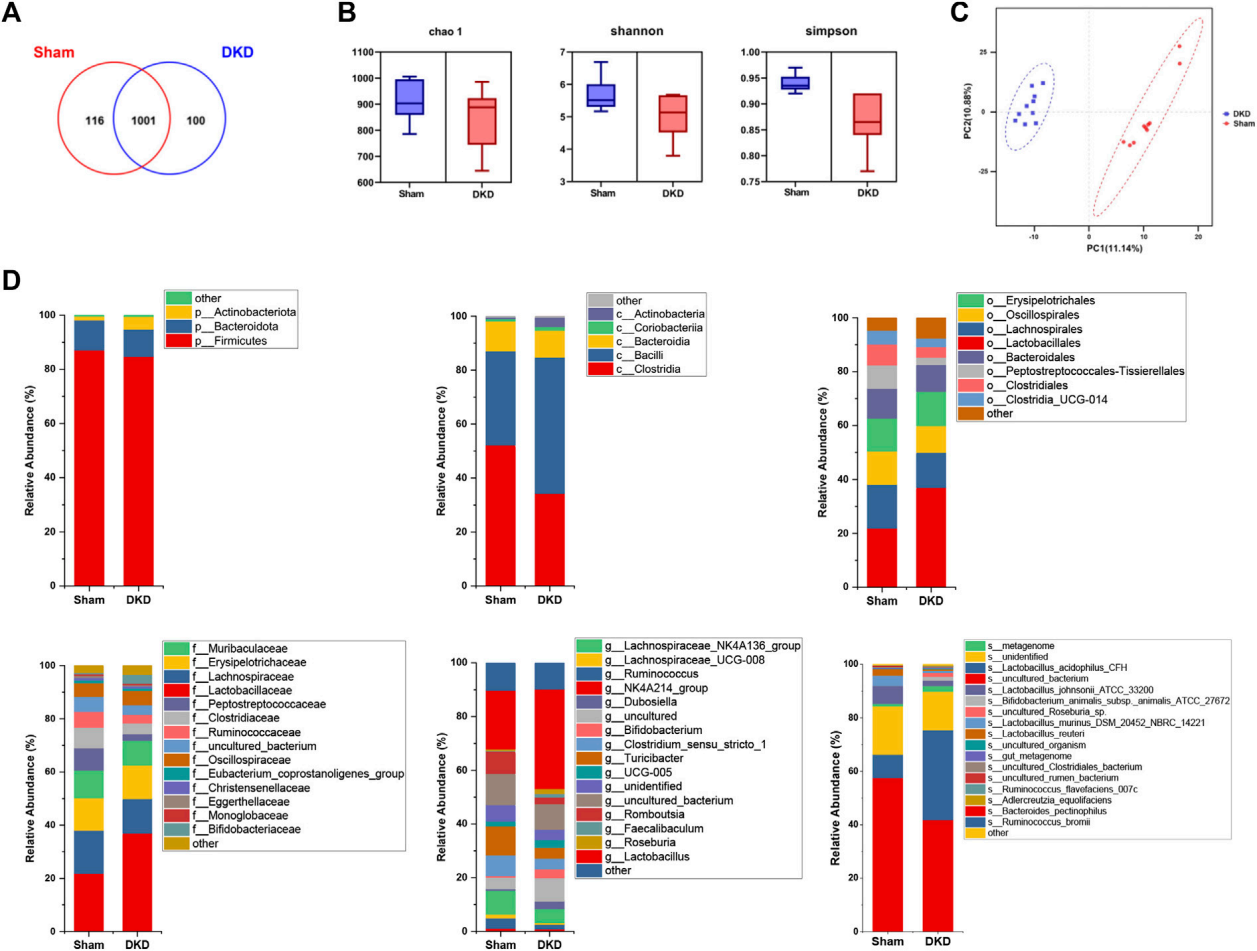
Altered bacterial taxa in DKD rats. **(A)** Venn diagram of OTU distribution between samples from sham and DKD rats. **(B)** Alpha-diversity indices of Chao1, Shannon, and Simpson of gut microbiota between sham and DKD rats. **(C)** PLS-DA of gut bacterial communities from the sham and DKD groups. **(D)** Taxonomic distributions of bacteria from colon content 16S rDNA sequencing data at the phylum to genus level between sham and DKD rats.

DNA was extracted from fresh colon contents and sequenced, obtaining an average of 61,776 quality-controlled sequences per sample. A total of 1,227 OTUs were obtained with cluster analysis, and 1,217 OTUs remained after pumping analysis. A total of 116 OTUs were unique to the sham group and 100 OTUs belonged specifically to the DKD group (Figure 5A). Chao1, Shannon, and Simpson indices characterized the α-diversity of microbial communities and were 910.60 ± 23.25, 5.68 ± 0.16, and 0.94 ± 0.01 in sham rats and 843.00 ± 38.10, 5.06 ± 0.20, and 0.87 ± 0.02 in the DKD rats, respectively (Figure 5B). The sham and DKD groups were markedly separated based on PLS-DA (Figure 5C).

Annotation of OTUs and microbial composition analysis were presented for each classification level. At the phylum level, 1,217 OTUs were assigned to 8 phyla. *Firmicutes* was the most dominant phylum and accounted for more than 80%. At the class level, *Clostridia*, *Bacilli*, and *Bacteroidia* occupied more than 90% of the total detected classes. *Lactobacillales* and *Lactobacillaceae* were the predominant bacteria at the order and family levels, respectively. At the genus level, the *NK4A214_group* had the highest relative abundance. The relative genus-level abundance of gut microbiota varied significantly among the DKD and sham groups (Figure 5D). We conclude that DKD dramatically alters the structure of the gut microbiota.

### Analysis of Significantly Altered Gut Taxa in Diabetic Kidney Disease Rats

**FIGURE 6:**
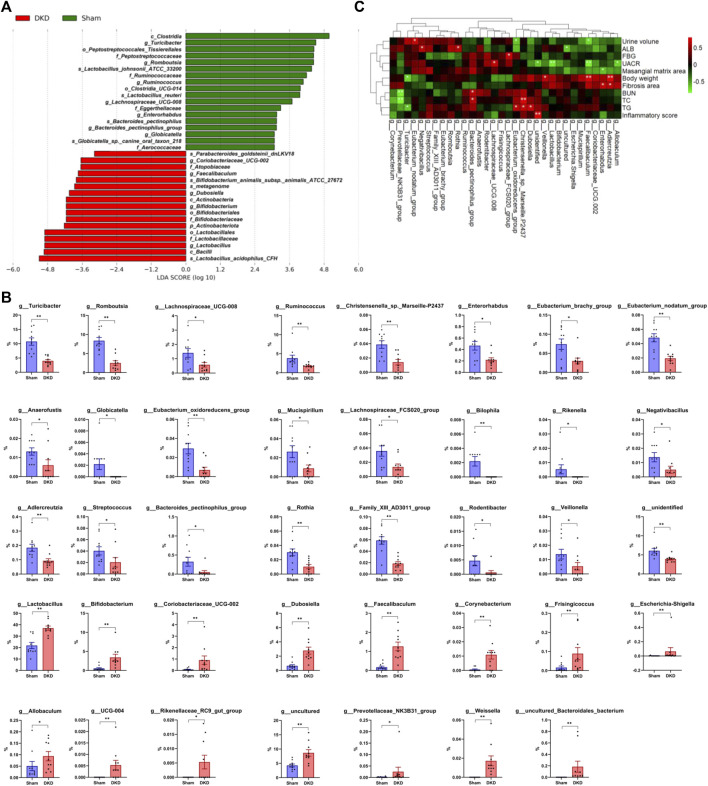
Differential gut microbiome in DKD rats compared to sham rats. **(A)** LDA score of the significantly discriminant taxa between the two groups (LDA score >3.0, Wilcoxon rank-sum test, *p* < 0.05). **(B)** Bar graphs of the significant differences in the relative abundance of 39 genera between sham and DKD rats selected by Wilcoxon test analysis and *p*-value < 0.05. *p < 0.05, **p < 0.01, versus sham rats. **(C)** Spearman rank correlation between DKD-related base parameters and differential genera (selected from Wilcoxon rank-sum test, *p* < 0.05) displayed in heat map cluster analysis (*p < 0.05, **p < 0.01).

Based on LEfSe analysis, 35 taxa were screened out, including 18 significantly decreased and 17 increased taxa (*p* < 0.05). *p_Actinobacteriota* was markedly increased in the DKD group (4.76 ± 0.89%) when compared to the sham group (1.43 ± 0.18%). *c_Clostridia* was detected at an average of 34.3% in DKD rats when compared to the 52.2% in sham rats (*p* < 0.05), whereas *c_Bacilli* (50.44 ± 2.92% in the DKD group and 34.89 ± 2.57% in the sham group, *p* < 0.05) and *c_Actinobacteria* (3.43 ± 0.84% and 0.56 ± 0.22% in the DKD and sham groups, respectively) had the opposite shift (Figure 6A). There were 39 genera that were significantly different among the sham and DKD groups (Figure 6B). *g_Turicibacter*, *g_Romboutsia*, *g_Ruminococcus*, *g_Lachnospiraceae_UCG-008*, etc. were markedly reduced in DKD rats, while *g_Lactobacillus*, *g_Bifidobacterium*, *g_Dubosiella*, *g_Coriobacteriaceae_UCG-002*, and *g_Faecalibaculum* were significantly elevated in the DKD group compared with the sham group.

The correlation between DKD-related base parameters and markedly differential genera was analyzed (Figure 6C). *g_Allobaculum*, *g_Adlercreutzia*, *g_Enterorhabdus*, *g_Coriobacteriaceae_UCG-002*, *g_Faecalibaculum*, *g_Mucispirillum*, *g_Escherichia–Shigella*, *g_uncultured*, *g_Bifidobacterium*, *g_Lactobacillus*, *g_Veillonella*, *g_unidentified*, *g_Dubosiella*, *g_Christensenella_sp._Marseille-P2437*, and *g_Eubacterium_oxidoreducens_group* were clustered together and had a negative correlation with urine volume, ALB, FBG, UACR, and mesangial matrix area and had a positive correlation with body weight, fibrosis area, BUN, TC, TG, and inflammatory score. In addition, *g_Lachnospiraceae_FCS020_group* had a positive correlation with FBG, *g_Lachnospiraceae_UCG-008* had a positive correlation with UACR, *g_Bacteroides_pectinophilus_group* had a positive correlation with BUN and TC, *g_Rothia* and *g_Negativibacillus* had positive correlations with ALB, *g_Eubacterium_nodatum_group* had a positive correlation with urine volume, while *g_Turicibacter* had a negative correlation with body weight and BUN, and *g_Prevotellaceae_NK3B31_group* had a negative correlation with BUN and TC.

### Prediction of Functional Changes in Intestinal Flora in Diabetic Kidney Disease Rats

**FIGURE 7:**
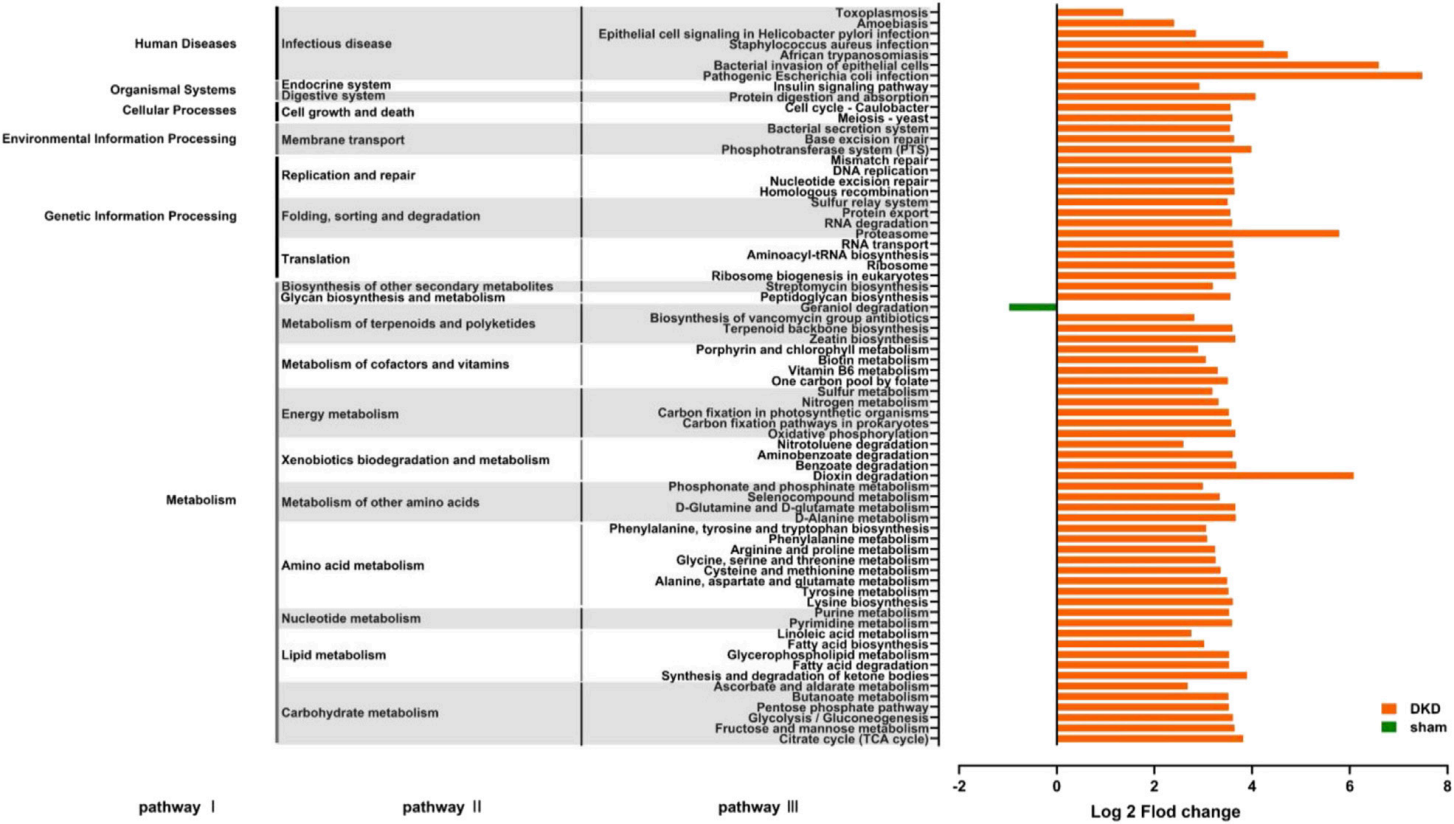
Predicting the effects of changes in the structure and abundance of intestinal flora on functional pathways. A total of 70 predictive pathways were screened out using Wilcoxon rank-sum test (*p* < 0.05).

Functional prediction was conducted based on the 16S rRNA gene sequencing data using PICRUSt analysis. There were 70 significantly altered pathways, including 45 metabolic pathways (Figure 7) (*p* < 0.05, Wilcoxon rank-sum test). In carbohydrate metabolism, the citric acid cycle, fructose and mannose metabolism, glycolysis/gluconeogenesis, the pentose phosphate pathway, and ascorbate and aldarate metabolism were significantly enriched, which were consistent with the abnormal accumulation of circulating carbohydrates. In lipid metabolism, fatty acid biosynthesis, metabolism, and enrichment were all improved, and correspondingly, disturbance of circulating fatty acids and their metabolites occurred. In amino acid metabolism, phenylalanine, tyrosine and tryptophan biosynthesis, phenylalanine metabolism, tyrosine metabolism, and arginine and proline metabolism were enriched, which coincided with the results that the concentration of L-phenylalanine, L-arginine, and L-citrulline were elevated, while L-tyrosine, L-tryptamine, and ornithine decreased in the DKD serum. This demonstrated a strong relationship between metabolites and gut microbiota in the DKD rats. In addition to the metabolic pathways, there were 25 pathways involved in human diseases, organismal systems, cellular processes, environmental information processing, and genetic information processing (Figure 7).

There were four metabolic pathways obtained by the KEGG enrichment analysis. These were dioxin degradation, proteasome, pathogenic *Escherichia coli* infection, and bacterial invasion of the epithelial cells. The number of differential genes annotated to the abovementioned pathways was extremely elevated (log2 fold change value >5) in the DKD group. Notably, the number of differential genes annotated to geraniol degradation, the exclusive pathway, was decreased.

### Analysis of the Interaction Between Serum Metabolites and Gut Microbiota in Diabetic Kidney Disease Rats

**FIGURE 8:**
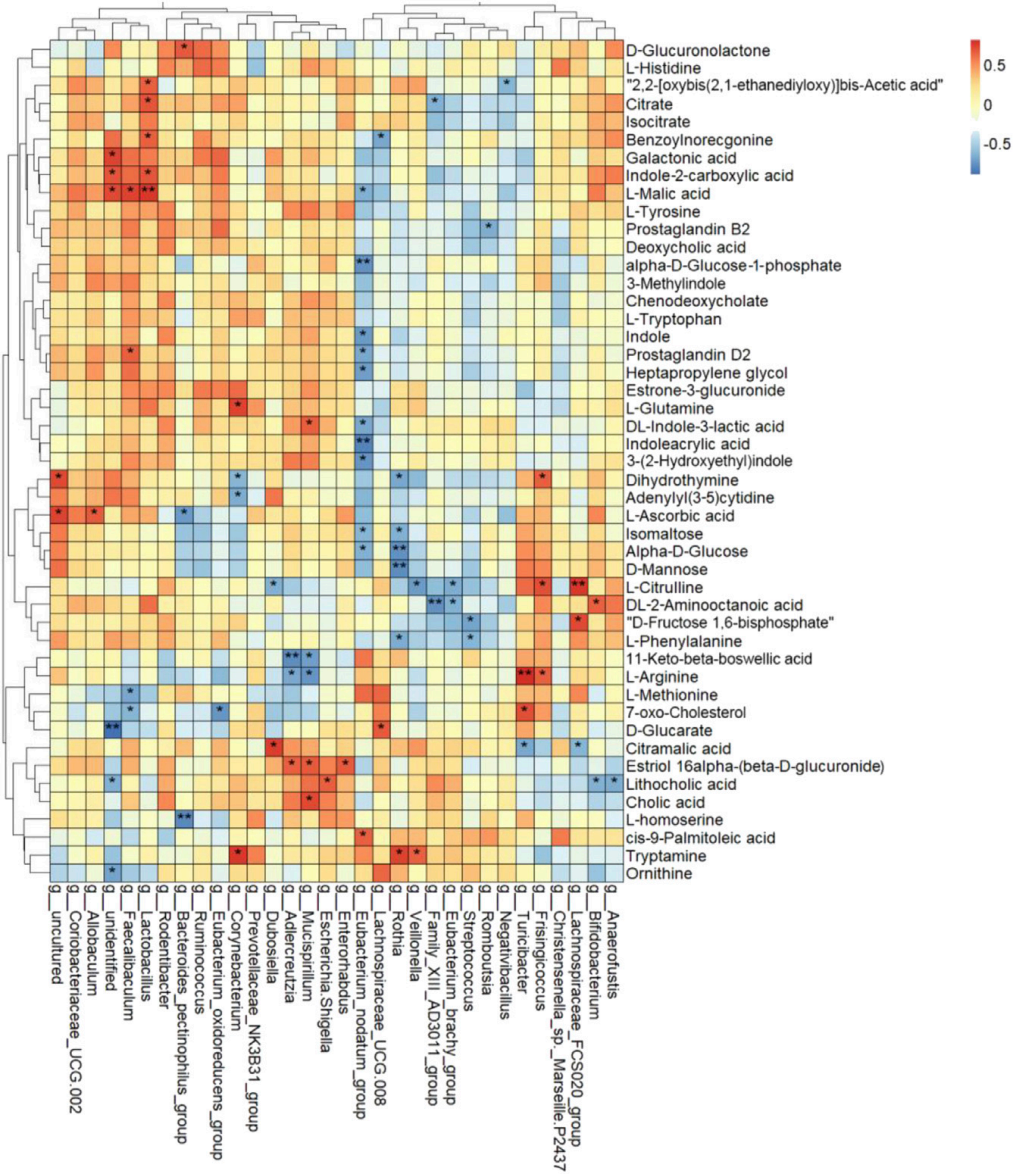
Heat map cluster analysis of the correlation among differential serum metabolites and differential genera using Spearman rank correlation (*p < 0.05, **p < 0.01).

To further study the correlations between serum metabolites and gut microbiota, Spearman correlation was performed (Figure 8). There was a complex interaction between serum metabolites and gut microbiota. A total of 27 of 32 genera had notable relationships with circulating differential metabolites. *g_Eubacterium_nodatum_group*, *g_Rothia*, *g_Lactobacillus*, *g_Mucispirillum*, *g_Faecalibaculum*, and *g_Corynebacterium* were all significantly correlated with at least 4 serum metabolites. More importantly, *g_Eubacterium_nodatum_group* had a significantly negative relationship with 10 metabolites and a positive relationship with 1 metabolite, indicating the potential impact on DKD metabolism. *g_Lactobacillus* and *g_Faecalibaculum* were high in abundance and were correlated with four and five serum differences, respectively. The above three genera showed a significant effect on DKD.

Furthermore, D-mannose, alpha-D-glucose, and isomaltose were clustered together and had strong negative correlations with *g_Eubacterium_nodatum_group* and *g_Rothia*. Galactonic acid, L-malic acid, and indole-2-carboxylic acid were clustered and positively correlated with *g_Lactobacillus* and *g_Faecalibaculum* and negatively correlated with *g_Eubacterium_nodatum_group* and *g__Lachnospiraceae_UCG-008*. Lithocholic acid, cholic acid, and estriol-16-alpha-(beta-D-glucuronide) were clustered and had positive correlations with *g_Enterorhabdus*, *g_Escherichia–Shigella*, *g_Mucispirillum,* and *g_Adlercreutzia*. DL-indole-3-lactic acid, indoleacrylic acid, 3-(2-hydroxyethyl)indole were clustered, and indole, prostaglandin D2, and heptapropylene glycol were clustered. All of them had a negative correlation with *g_Eubacterium_nodatum_group*.

### Effect of Tangshen Formula Treatment on Serum Metabolites in Diabetic Kidney Disease Rats

**FIGURE 9:**
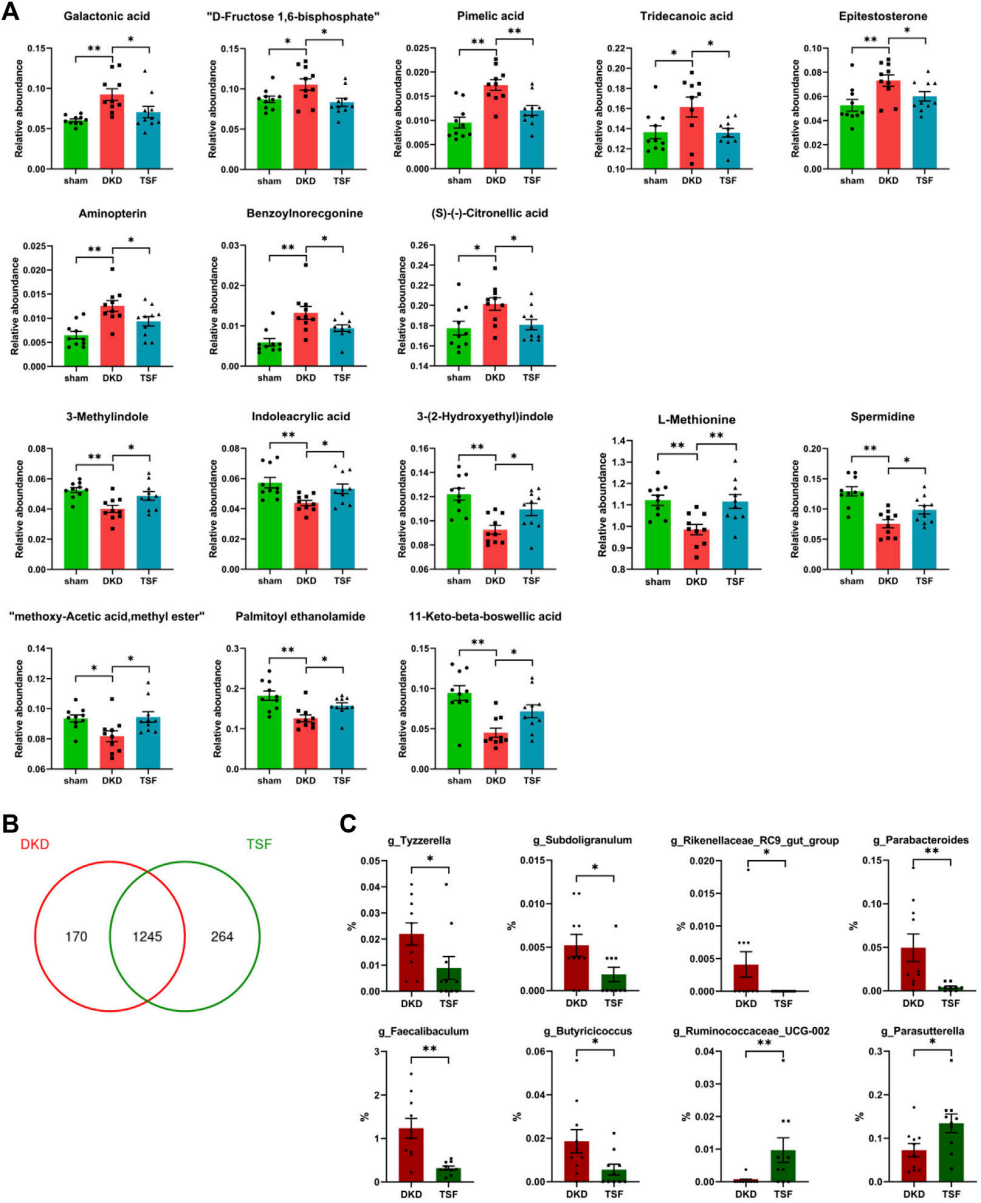
Effects of TSF treatment on serum metabolites and gut microbiota. **(A)** TSF treatment significantly improved levels of 16 metabolites in DKD rats as determined using Wilcoxon rank-sum test (*p < 0.05, **p < 0.01). **(B)** Effect of TSF treatment on gut microbiota at the OTU level. **(C)** Relative abundance of genera changes in the TSF group compared with the DKD group.

After 20 weeks of TSF treatment, disturbances of 16 serum metabolites in DKD rats were significantly attenuated. Eight of the metabolites that were excessively accumulated in the serum of DKD rats were reduced in the TSF group. These metabolites included galactonic acid, D-fructose-1, 6-bisphosphate, medium- and long-chain fatty acids (pimelic acid and tridecanoic acid), androgen (epitestosterone), among others. Eight metabolites that were markedly reduced in DKD rats and whose relative abundances were significantly elevated after TSF treatment were tryptophan [3-methylindole, indoleacrylic acid, and 3-(2-hydroxyethyl)indole], amino acids and derivatives (L-methionine and spermidine), and other substances (Figure 9A).

Finally, TSF treatment greatly altered the structure and composition of gut microbiota. At the OTU level, the number of unique OTUs in TSF rats was 264 compared with 170 OTUs in DKD rats (Figure 9B). There were eight genera that were altered in the TSF group, and of note, TSF treatment restored the OTU relative abundance of g_Faecalibaculum and g_Rikenellaceae_RC9_gut_group (Figure 9C).

## Discussion

**FIGURE 10:**
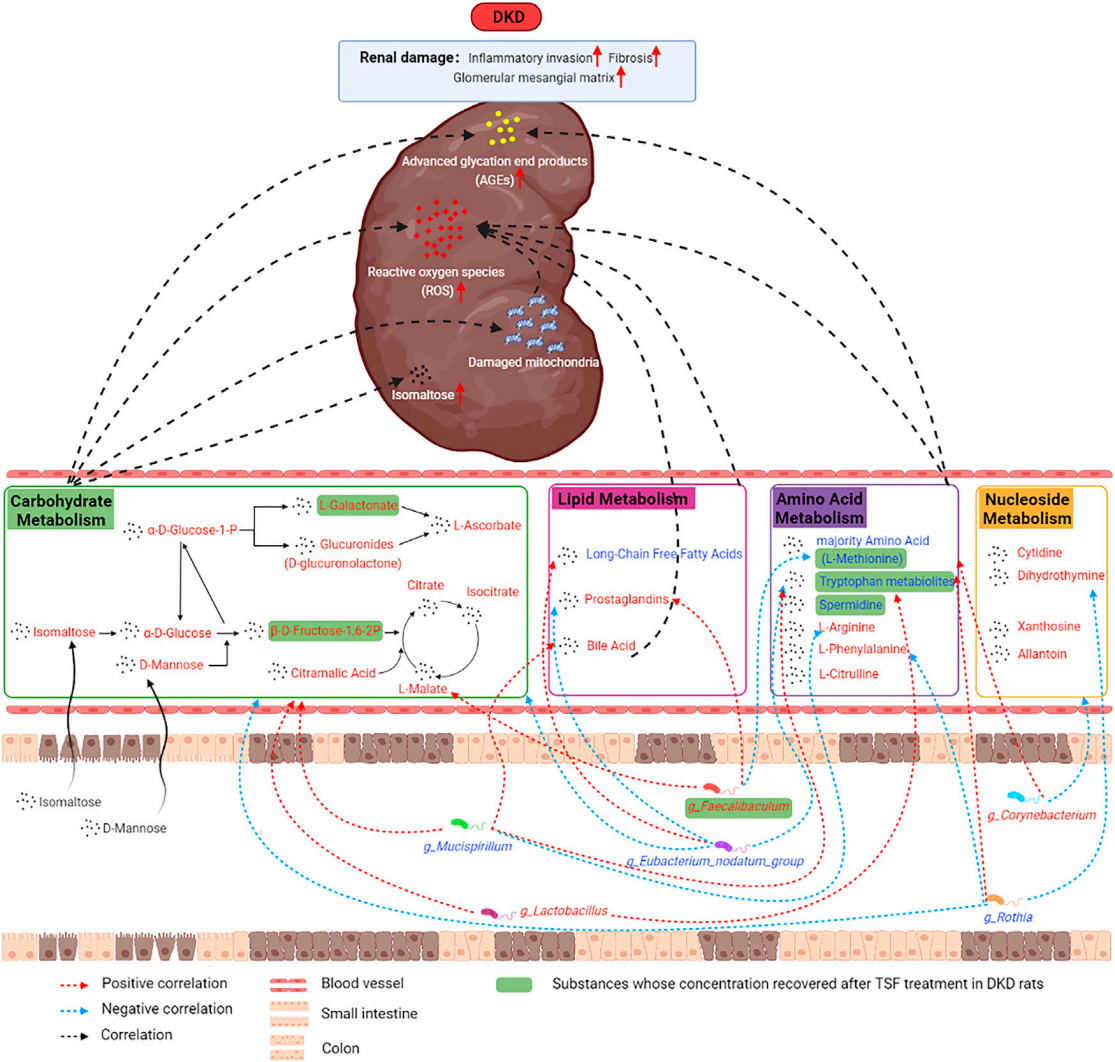
The mechanism diagram illustrated the potential mechanisms between altered serum metabolic profiles, disturbances in gut microbiota, and kidney damage in DKD.

In the current research, we used untargeted metabolomics to study the shift in the metabolic profile of the DKD rat serum and utilized 16S rRNA gene sequencing to analyze the changes in the structure and function of the gut microbiota. We further explored the relationship and role of the microbiota in the progression of DKD. Our results indicate that the metabolic profile of DKD rats and the composition and structure of their gut microbiota were altered. These changes were associated with the metabolism of carbohydrates, lipids, amino acids, and nucleotides. In these metabolic pathways, isomaltose, D-mannose, galactonic acid, citramalic acid, and prostaglandin B2 were significantly upregulated. 3-(2-hydroxyethyl)indole, 3-methylindole, and indoleacrylic acid were downregulated and were the critical metabolites, and *g_Eubacterium_nodatum_group*, *g_Lactobacillus*, and *g_Faecalibaculum* were important functional bacteria in the DKD model. Furthermore, the levels of these three indoles were restored after treatment with the traditional Chinese herbal medicine Tangshen Formula (Figure 10).

We found that alpha-D-glucose, alpha-D-glucose 1-phosphate, and “D-Fructose 1,6-bisphosphate”—which are involved in glycolysis/gluconeogenesis—and citrate, isocitrate, and L-malate—which are involved in the citric acid cycle—were all elevated in the DKD serum. Studies have pointed out that the glycolysis and citric acid cycle metabolites accumulate in the kidneys in the early stage of diabetes (Hasegawa et al., 2020) and abnormal glycolysis and accumulation of toxic glucose metabolites are linked to kidney damage in diabetic patients and models (Qi et al., 2017; Srivastava et al., 2018; Li et al., 2020b; Liu et al., 2021). Moreover, glycolysis and the citric acid cycle in the DKD serum are disturbed, and abnormal increases in dihydroxyacetone phosphate and succinyl-CoA are positively related to mitochondria damage (Jiang et al., 2019). Citramalic acid is a metabolite of gut microbiota and an analog of L-malate that competitively inhibits the metabolism of L-malate. In our research, citramalic acid was elevated in DKD rats, which concurs with the results of O'Kell et al. (2021) in their study on diabetic dogs. We further confirmed that the disorder of glycolysis/gluconeogenesis and citrate cycle and the abnormal accumulation of intermediate metabolites in the serum play an outstanding role in DKD progression.

In our study, we discovered that isomaltose and D-mannose were elevated in the DKD serum. We had previously studied the renal cortex metabolomics in DKD rats and demonstrated the abnormal accumulation of maltose and mannose in the kidneys (the fold change of DKD/sham was 498.56 for maltose and 7.43 for mannose) (Zhao et al., 2012). Isomaltose is the end product of starch digestion and is hydrolyzed into glucose, which is then absorbed in the small intestine. Therefore, an abnormal elevation of isomaltose in the DKD serum might be due to the downregulation of activity or content of isomaltose, and intestinal barrier impairment. The toxic effect of isomaltose on the kidneys is not well understood and needs further study. D-mannose is also derived from food and is absorbed directly into the blood and excreted intact in the urine without metabolization. Any remaining mannose in the system is mainly utilized for nonenzymatic glycation, the activity of which has been found to be five times that of glucose (Sharma et al., 2014). Advanced glycation end products participate in the progression of DKD through multiple modes of action (Vlassara et al., 2012; Azegami et al., 2021). Moreover, in our study, we found that isomaltose, D-mannose, and glucose were highly associated with differential genera of the microbiota.

Another interesting discovery was that the circulating oxidation products of carbohydrates, namely, D-glucuronolactone, D-glucarate, and galactonic acid, were greatly elevated in the DKD group when compared with the sham group, which might indicate excessive oxidative stress in DKD rats. In line with this viewpoint, we found a marked decline in serum L-methionine, which could be methylated to generate L-(+)-cysteine in the body. The latter is one of the three amino acids that synthesize glutathione (GSH). GSH is an important antioxidant that participates in a variety of redox reactions and can effectively improve oxidative stress (Fahrmann et al., 2015). In the present investigation, the elevation in circulating D-glucuronolactone was correlated positively with the serum level of BUN. Moreover, *g_Bacteroides_pectinophilus_group* had a positive association with D-glucuronolactone and BUN. We also found that the concentration of the three glucuronides in the renal cortex of DKD rats was more than five times that in the sham group. Of note, 2-phenylethanol glucuronide in the renal cortex was 27 times that in sham rats in our previous study (Zhao et al., 2012). Thus, it appears that excessive accumulation of glucuronides in the renal cortex was involved in kidney injury. However, the role of *g_Bacteroides_pectinophilus_group* in this process needs more exploration. In addition, we found that L-ascorbate, another well-known endogenous antioxidant (Al-Shamsi et al., 2006; Kazmierczak-Baranska et al., 2020), was elevated in the DKD serum. This might be a self-regulation response to the oxidative stress state.

Our results indicate that there were several DKD-associated changes in lipid metabolism. DKD rats displayed decreased levels of long-chain free fatty acids, such as arachidonic acid, cis-9-palmitoleic acid, palmitic acid, “all cis-(6,9,12)-linolenic acid,” and myristic acid. Free fatty acids have beneficial effects against DKD. One fatty acid, cis-9-palmitoleic acid, has been found to attenuate hyperglycemia by promoting β-cell proliferation under low glucose concentration, improving β-cell secretion and inhibiting the expression of pro-inflammatory genes (Maedler et al., 2001; Yang et al., 2011).

Consistent with this finding, the metabolites of arachidonic acid, namely, prostaglandin B2 and prostaglandin D2, were markedly elevated in the DKD group when compared with the sham group. Prostaglandins have many physiological effects, such as responding to inflammation, and regulating the immune system and blood pressure (Yao and Narumiya, 2019). Prostaglandin E2 contributes to the increase in renal blood flow through afferent arteriole dilation (Ren et al., 2013; Asirvatham-Jeyaraj et al., 2019) and is involved in insulin resistance (Robertson, 1983). The resulting changes in the DKD kidney include renal hemodynamic alteration, glomerular hypertension, and decrease in glomerular filtration rate (Alicic et al., 2017). Prostaglandin B2 has been found to reduce mean arterial pressure and increase renal blood flow (Marchand et al., 1973; Hall and Jaitly, 1976). In our study, prostaglandin B2 was the most elevated among all the metabolites and was positively correlated with the FBG level and negatively correlated with the ALB level. Therefore, prostaglandin B2 and E2 may act similarly, that is, their accumulation can lead to insulin resistance and accelerate kidney damage, but in our study, prostaglandin B2 was elevated, whereas prostaglandin E2 was not elevated.

The interaction between prostaglandins and gut microbiota is being studied intensely. Prostaglandin E2 was found to be synthesized by the commensal fungus *Meyerozyma guilliermondii*, and prostaglandin E2 production in the liver has been shown to be positively correlated with excessive growth of gut fungi (Sun et al., 2020). Our results indicate that prostaglandin B2 has a negative correlation with *g_Romboutsia*, and prostaglandin D2 was positively correlated with *g_Faecalibaculum* and negatively correlated with *g_Eubacterium_nodatum_group.*

Bile acids are derived from cholesterol through oxidation of liver enzymes and aid in fat digestion and absorption. Bile acids are being investigated for their regulatory effects on the metabolism of lipids, glucose, and energy, as well as their involvement in immunologic processes such as signaling molecules (McGlone and Bloom, 2019). In the present study, the levels of bile acid in the DKD rats' serum, namely, chenodeoxycholate, lithocholic acid, cholic acid, and deoxycholic acid, were significantly higher than in the sham group. Elevated levels of bile acids have also been found in the feces of DKD mice (Zhao et al., 2019) and in patients with end-stage renal disease (Wang et al., 2020). Proposed mechanisms of bile acids causing kidney damage such as disruption of the intestinal barrier and allowing bacterial toxins to enter the systemic circulation (Ramezani and Raj, 2014), oxidative stress, and promoting the release of inflammatory cells (Chávez-Talavera et al., 2017). The gut microbiota metabolize primary bile acids into secondary bile acids through deconjugation, dehydrogenation, and dihydroxylation (Caesar, 2019). Chenodeoxycholic acid undergoes 7-dehydroxylation to produce lithocholic acid with *Clostridium* and *Eubacterium* participating in 7-dehydroxylation (Jia et al., 2018). In our study, we found lithocholic acid was positively correlated with *g_Escherichia–Shigella* and was negatively correlated with *g_Bifidobacterium* and *g_Anaerofustis*, which implies that the three genera are involved in 7-dehydroxylation.

DKD rats were treated with TSF, a traditional Chinese medicine compound with verified therapeutic action against DKD in animal experiments (Zhang et al., 2011) and clinical trials (Li et al., 2015). Following TSF treatment, metabolite disorders in the serum and gut microbiota imbalance were partially restored, and damage to the renal cortex was ameliorated. Recovery of circulating galactonic acid and L-methionine levels following TSF treatment further demonstrated the important role of oxidative stress in DKD. We also found that levels of indole derivatives [namely, 3-(2-hydroxyethyl)indole, 3-methylindole, and indoleacrylic acid], as well as spermidine (an amino acid metabolite), were recovered after TSF treatment but were decreased in the DKD group. Indole and derivatives are converted from tryptophan under the action of gut microbiota and have a wide range of biologic functions. Indoleacrylic acid improves the integrity of the intestinal barrier through promoting the differentiation of the intestinal goblet cells and mucus secretion from the intestinal goblet cells. Furthermore, indoleacrylic acid possesses antioxidant and anti-inflammatory functions (Wlodarska et al., 2017). Studies have found that 3-methylindole is an antioxidant that prevents lipid peroxidation in lung tissue (Adams et al., 1987; Kiorpes et al., 1988). Research indicates that 3-(2-hydroxyethyl)indole shows antimicrobial activity against *Staphylococcus aureus, Salmonella enterica*, and *Lactobacillus plantarum* (Roager and Licht, 2018)*.* Spermidine is a polyamine that has diverse metabolic functions such as inhibiting hemoglobin glycosylation and lipid peroxidation (Méndez and Balderas, 2006) and has the potential to prevent DKD complications. Therefore, amino acids, especially tryptophan and its metabolites, are highly involved in the development of DKD.

Taken together, the metabolite disturbances that we identified were mainly concentrated in the metabolism of carbohydrates, lipids, and amino acids, as well as in related functional bacteria. Our finding that disruption of amino acid metabolism, especially disordered tryptophan metabolism, was in line with the results of studies in DKD patients (Winther et al., 2020; Zhang et al., 2021). However, we did not find abnormal accumulation of typical uremic toxins in middle-stage DKD rat serum, which might be due to the fact that the most significant accumulation of uremic toxins occurred during progression to end-stage renal disease. Zhang et al. (2021) compared end-stage (eGFR <15 ml/min/1.73 m^2^) and non–end-stage renal disease (eGFR ≥15 ml/min/1.73 m^2^) with type 2 DKD patients and demonstrated an anomalously elevated hippurate. Similarly, Winther et al. (2020) in comparing type 1 diabetic patients with macroalbuminuria and those with normo- or microalbuminuria found that serum indoxyl sulphate was elevated, while those abnormal rises did not occur in individuals with microalbuminuria and those with normoalbuminuria.

We discovered novel metabolites and gut genera involved in carbohydrate metabolism and lipid metabolism. TSF treatment attenuated the abnormal levels of metabolites and gut microbiota dysbiosis. In the future, we should validate our results in samples from DKD patients and confirm the mechanism for potential functional molecules *in vivo* and *in vitro*. In addition, fecal microbiota transplantation to identify specific bacterial species will support exploration of the underlying mechanisms in DKD and TSF treatment.

## Conclusion

In the present investigation, disturbances in the metabolic profile in the serum and gut microbiota were the main cause of imbalances in carbohydrate, lipid, and amino acid metabolism, as well as of renal damage, thus the importance of the aforementioned in the progression of DKD. Anomalously upregulated isomaltose, D-mannose, galactonic acid, citramalic acid, and prostaglandin B2 and downregulated 3-(2-hydroxyethyl)indole, 3-methylindole, and indoleacrylic acid were the critical metabolites in these pathways. Additionally, *g_Eubacterium_nodatum_group*, *g_Lactobacillus*, and *g_Faecalibaculum* were the most involved in metabolic disorders in DKD rats.

## Data Availability

The datasets presented in this study can be found in online repositories. The names of the repository/repositories and accession number(s) can be found below: National Center for Biotechnology Information (NCBI) BioProject database under accession number PRJNA812954.
