# Supplementary figures and images for: Characteristics of Serum Metabolites and Gut Microbiota in Diabetic Kidney Disease (part 1 of 13)
Source: Front Pharmacol. 2022 Apr 14;13:872988. doi: 10.3389/fphar.2022.872988 (PMC9084235; doi:10.3389/fphar.2022.872988)

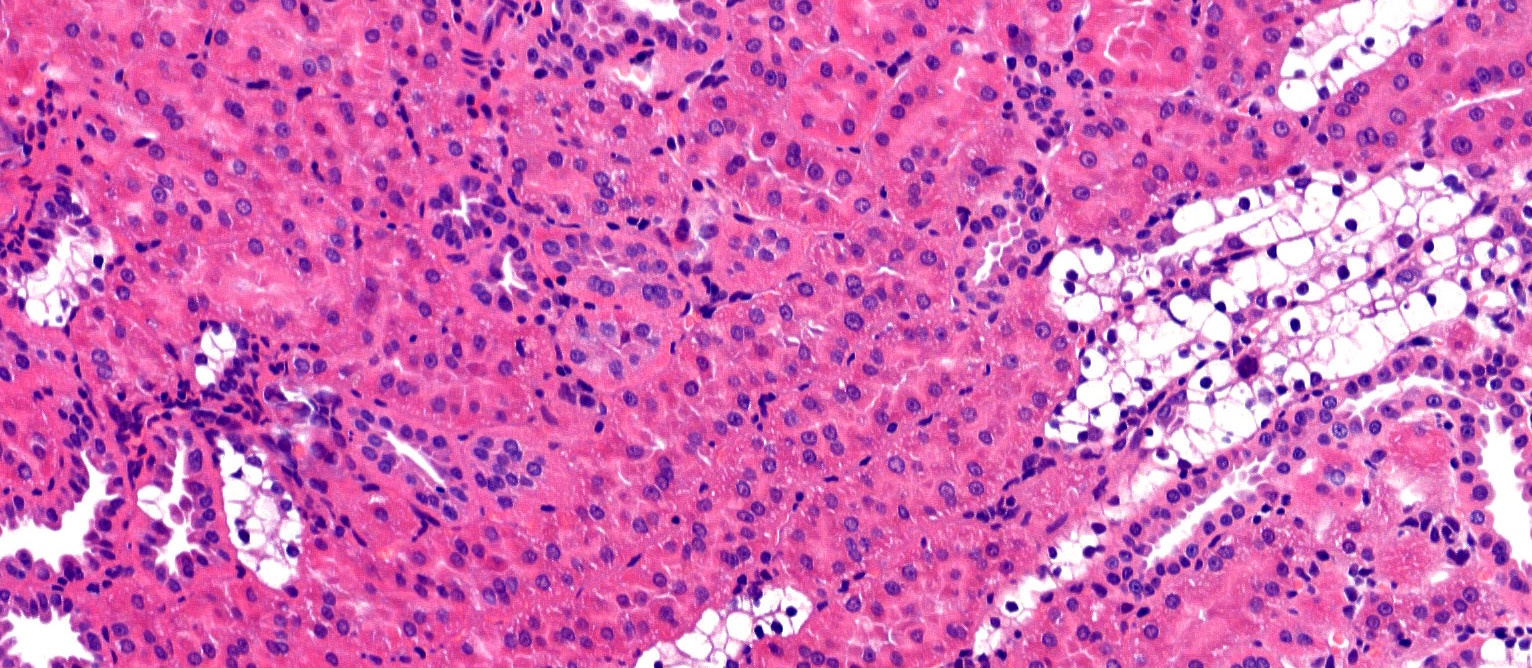

Supplement: Supplementary file 1 [file DataSheet3.ZIP › Fig 1D-HE-DKD-23(2)/23-10.jpeg]

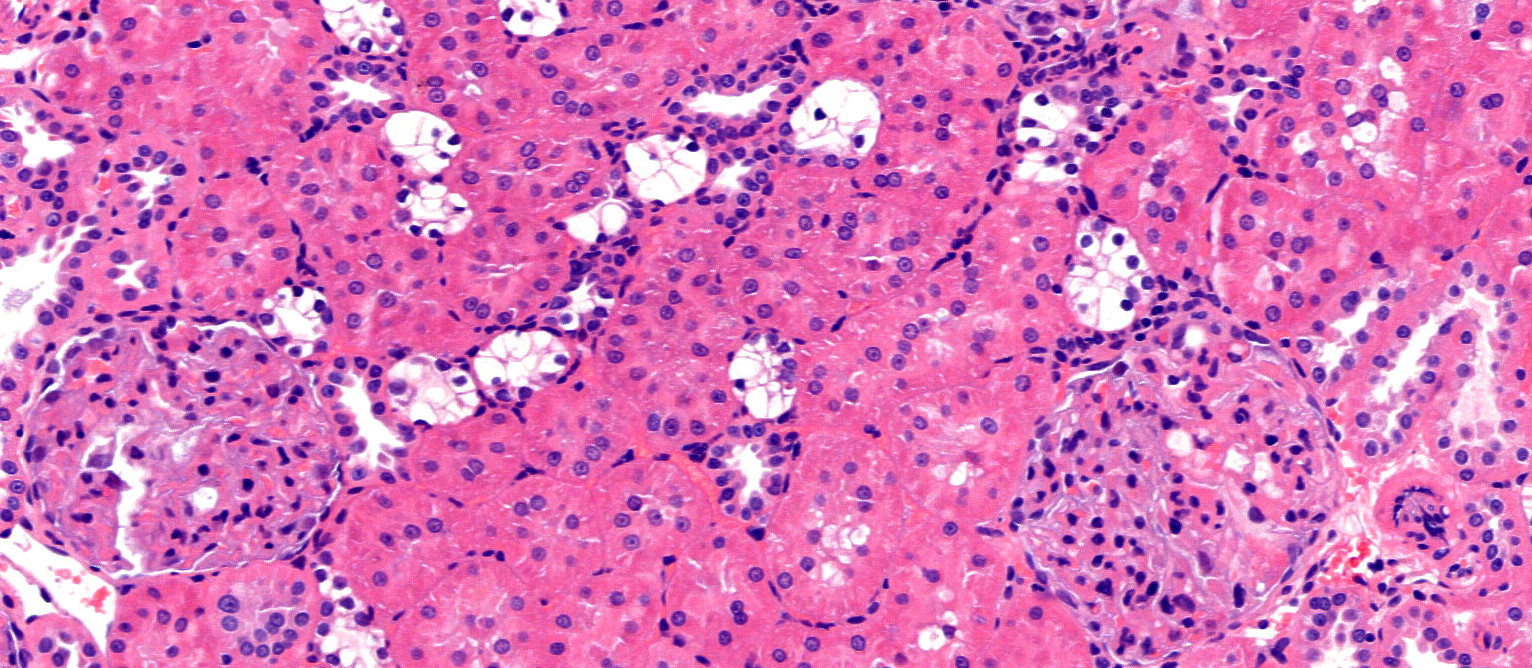

Supplement: Supplementary file 1 [file DataSheet3.ZIP › Fig 1D-HE-DKD-23(2)/23-3.jpeg]

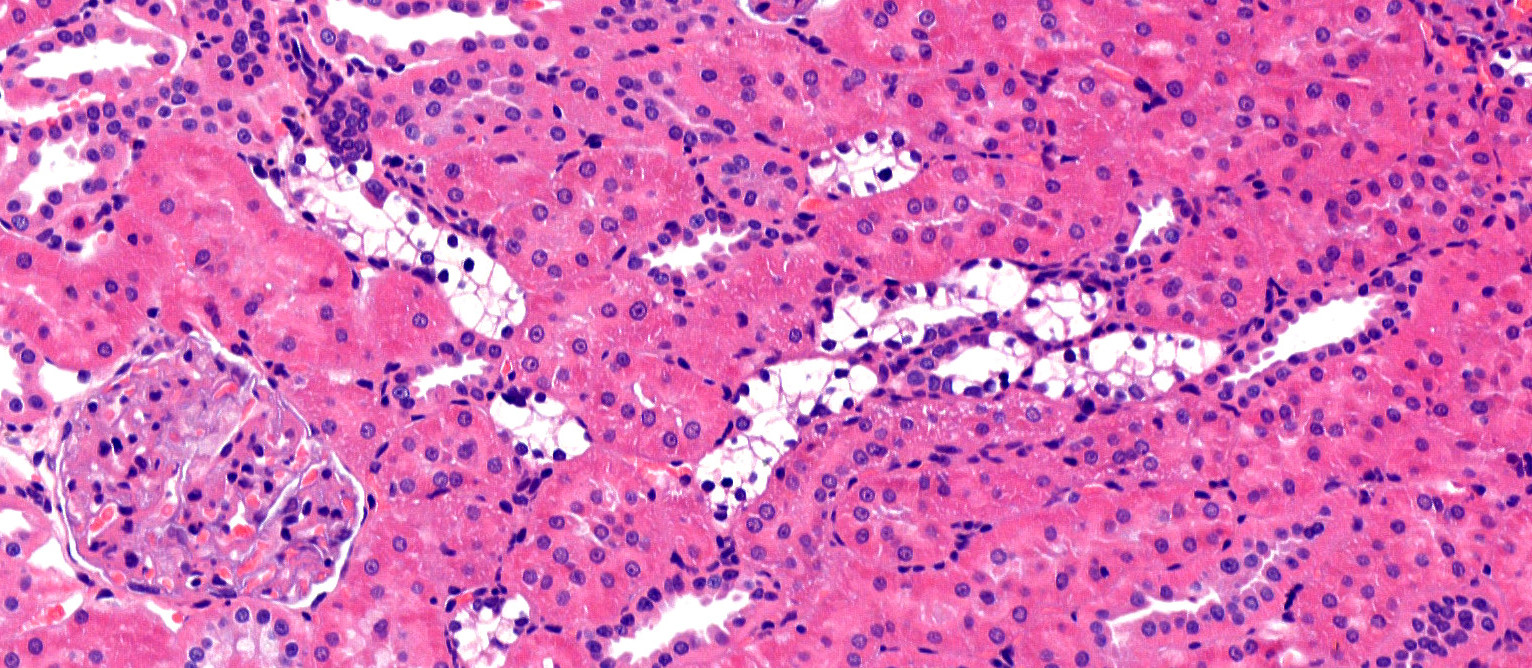

Supplement: Supplementary file 1 [file DataSheet3.ZIP › Fig 1D-HE-DKD-23(2)/23-4.jpeg]

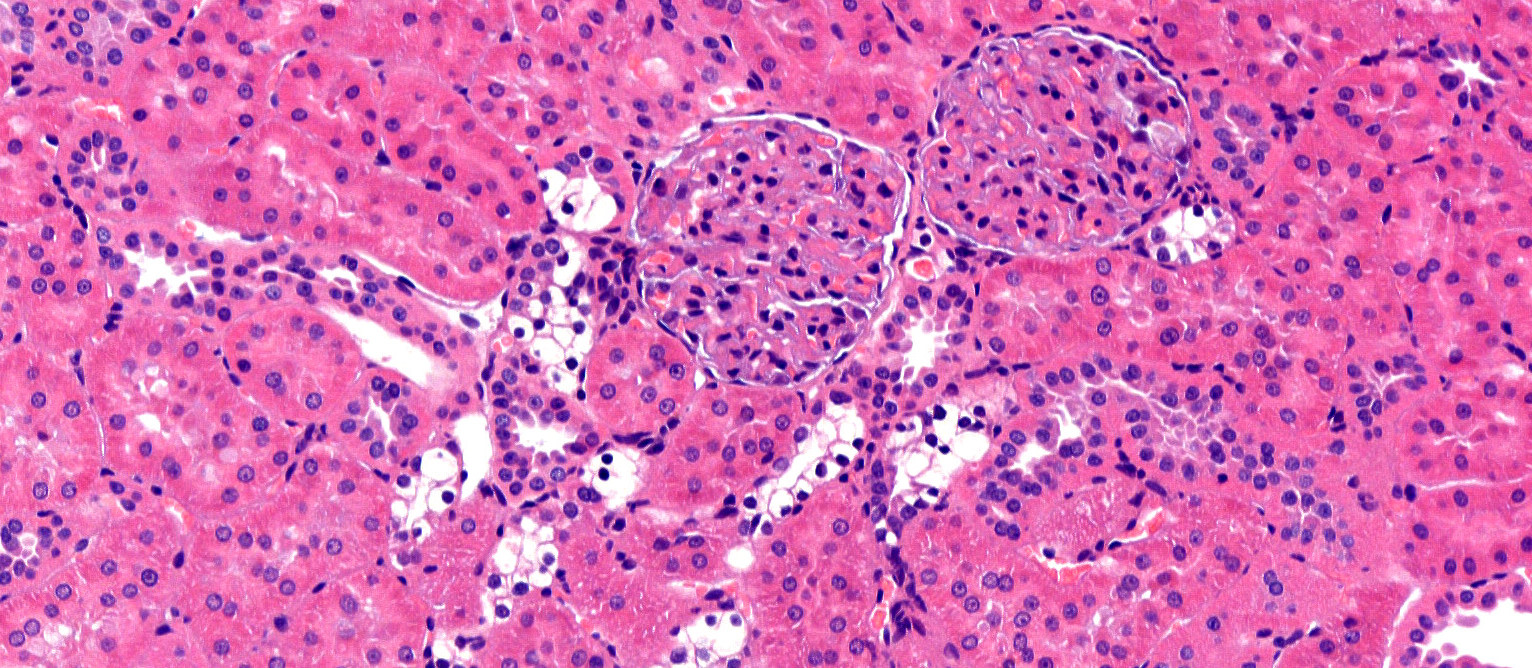

Supplement: Supplementary file 1 [file DataSheet3.ZIP › Fig 1D-HE-DKD-23(2)/23-5.jpeg]

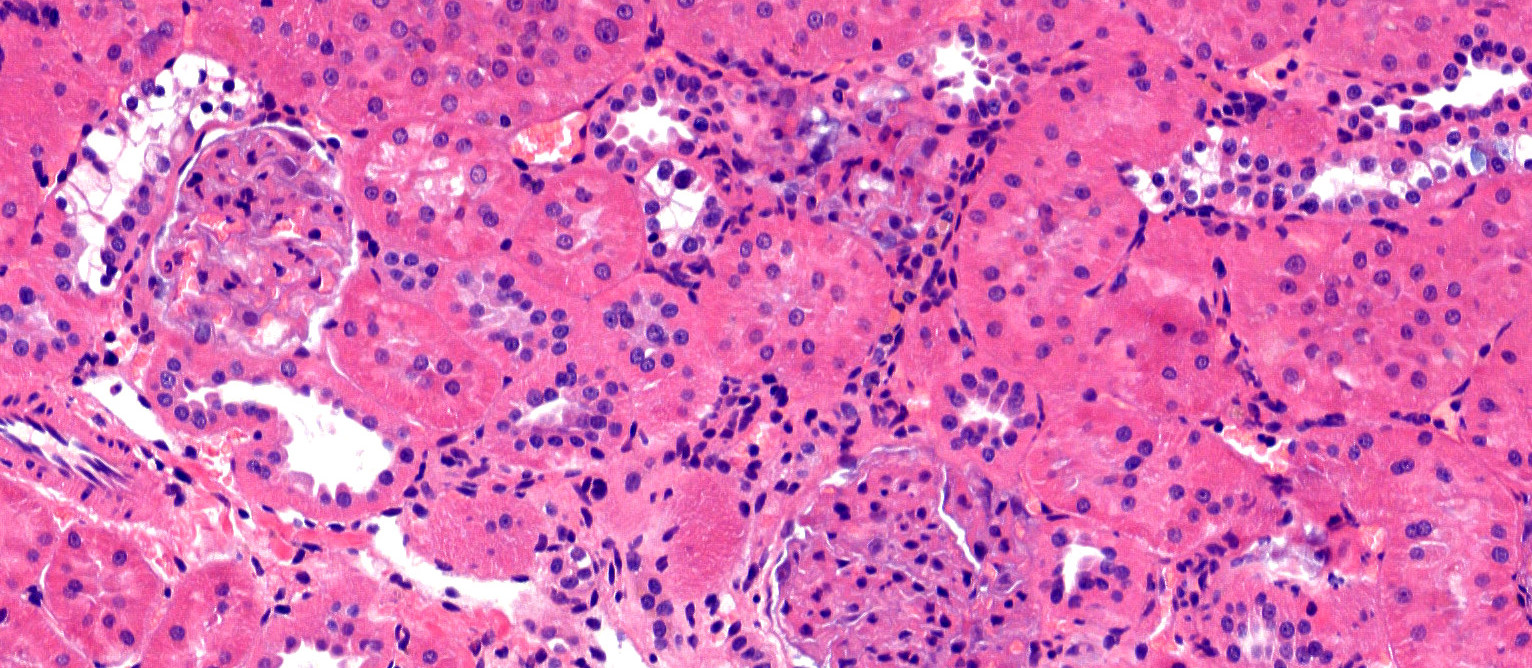

Supplement: Supplementary file 1 [file DataSheet3.ZIP › Fig 1D-HE-DKD-23(2)/23-6.jpeg]

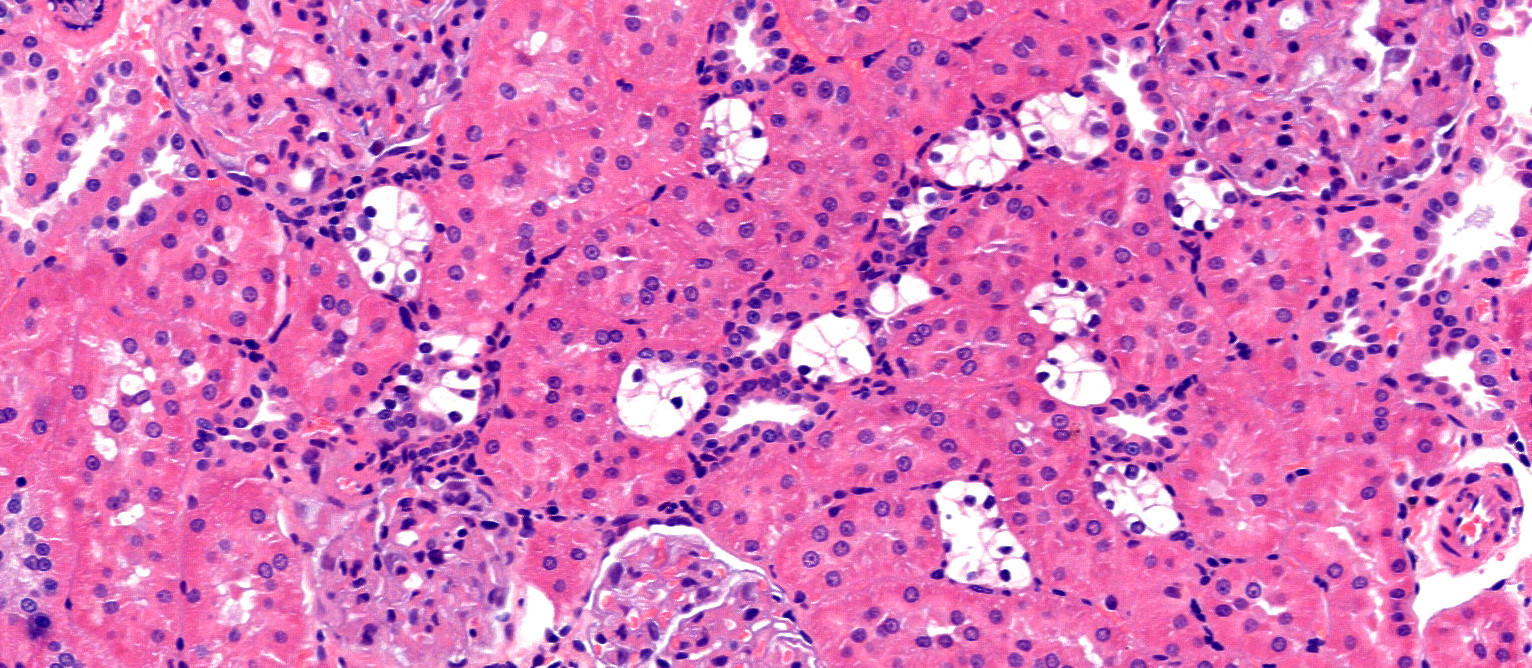

Supplement: Supplementary file 1 [file DataSheet3.ZIP › Fig 1D-HE-DKD-23(2)/23-7.jpeg]

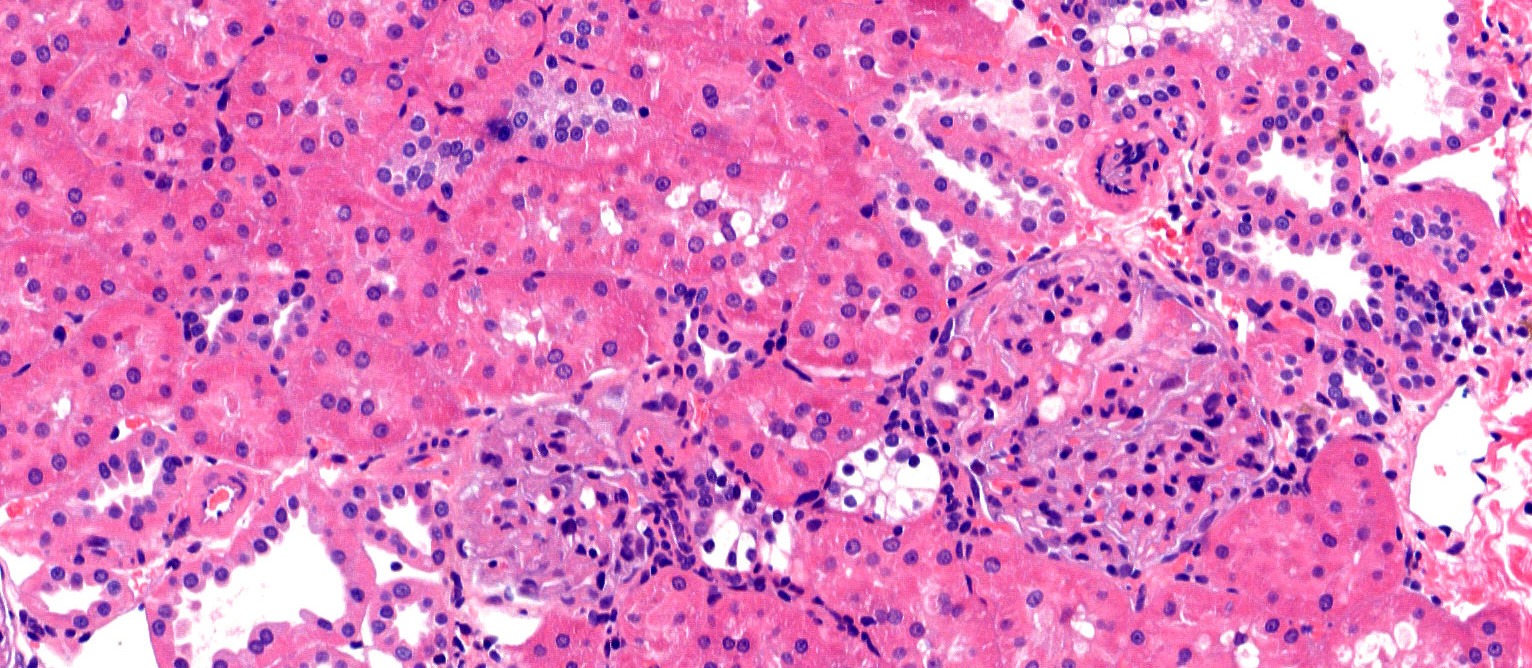

Supplement: Supplementary file 1 [file DataSheet3.ZIP › Fig 1D-HE-DKD-23(2)/23-8.jpeg]

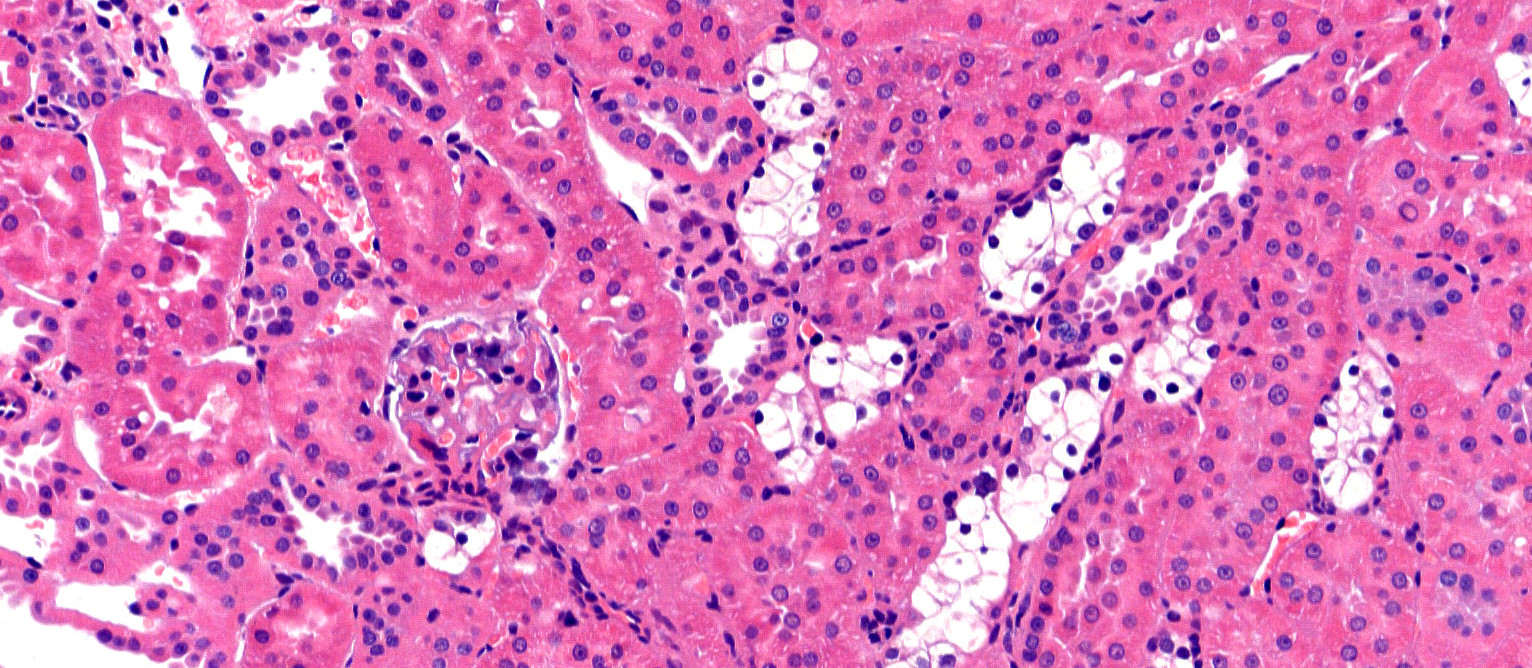

Supplement: Supplementary file 1 [file DataSheet3.ZIP › Fig 1D-HE-DKD-23(2)/23-9.jpeg]

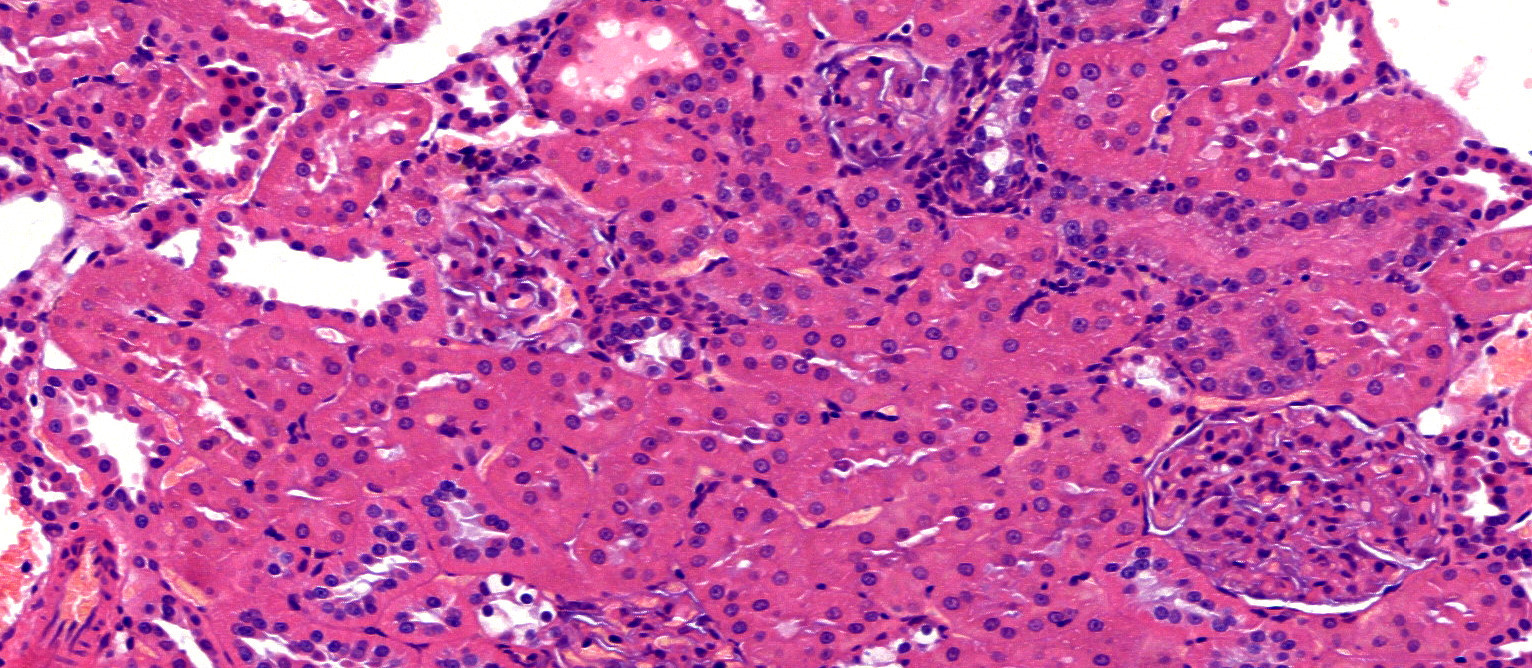

Supplement: Supplementary file 1 [file DataSheet3.ZIP › Fig 1D-HE-DKD-24/24-1.jpeg]

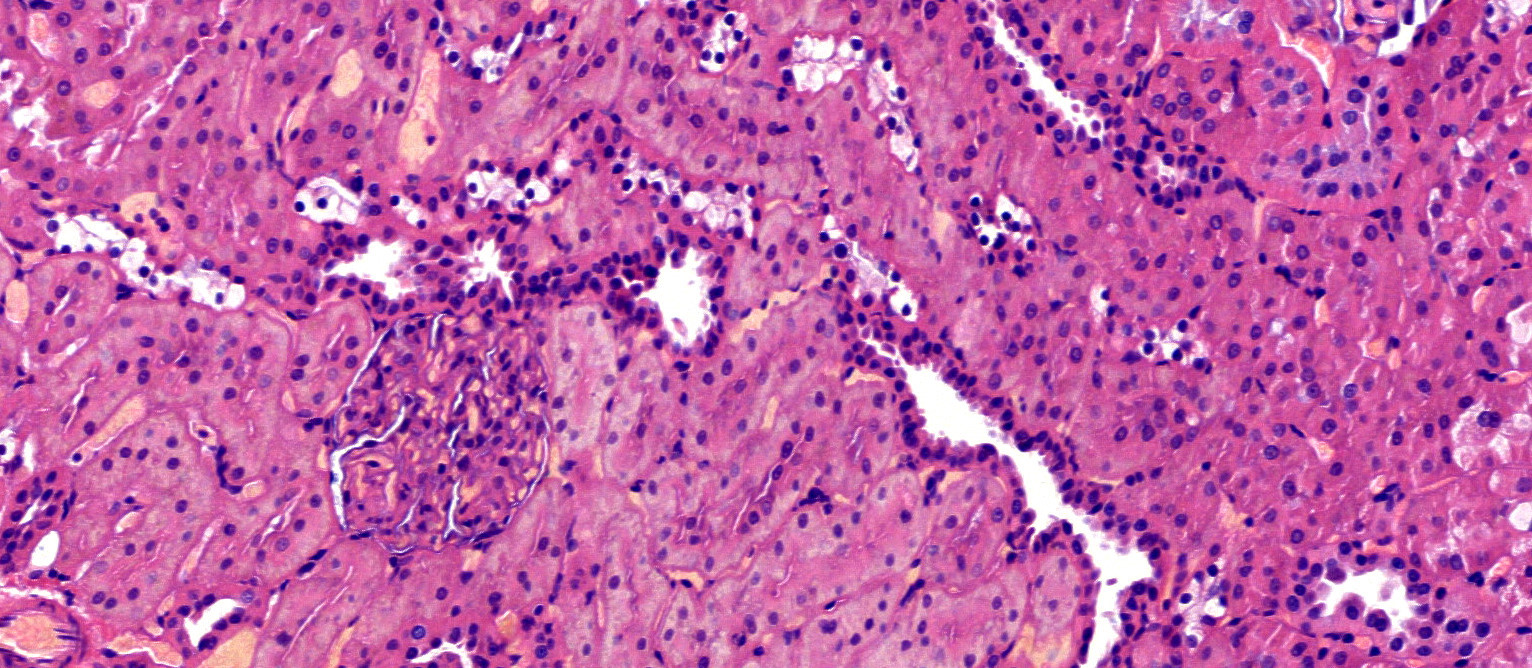

Supplement: Supplementary file 1 [file DataSheet3.ZIP › Fig 1D-HE-DKD-24/24-10.jpeg]

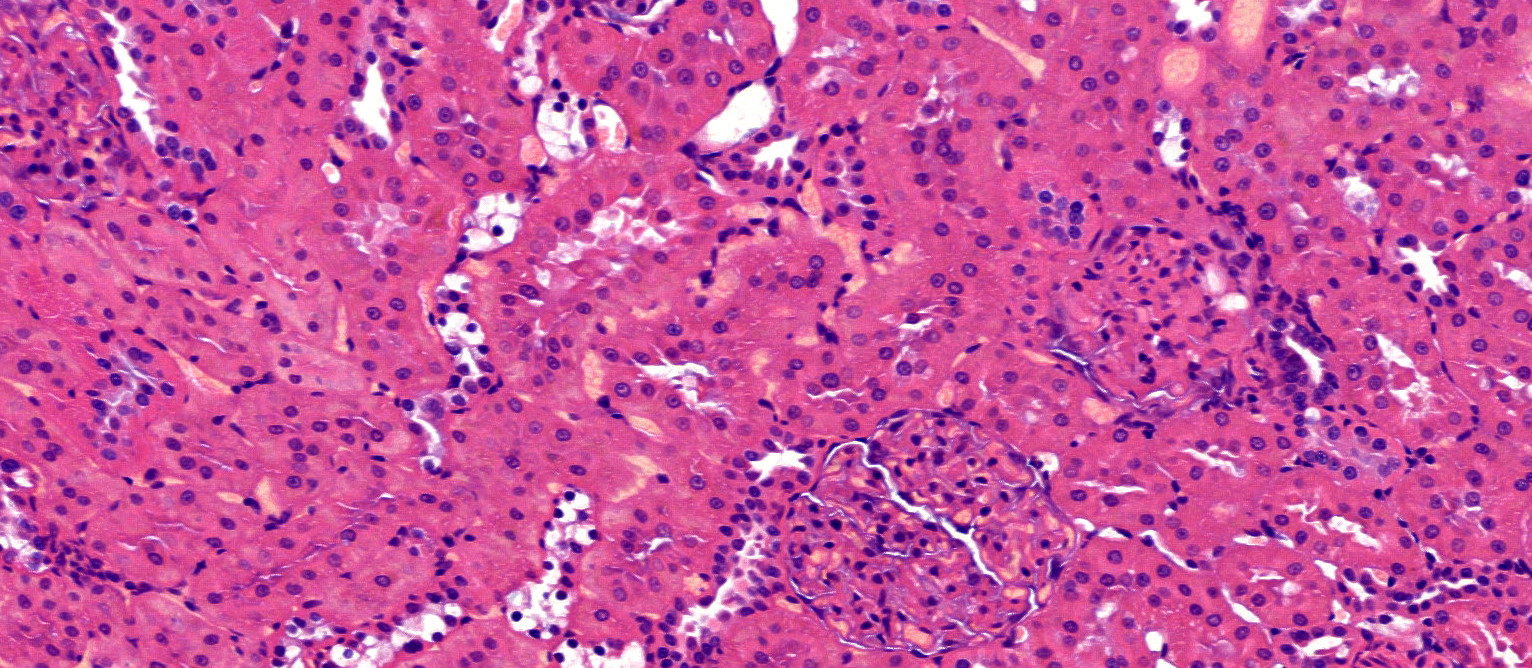

Supplement: Supplementary file 1 [file DataSheet3.ZIP › Fig 1D-HE-DKD-24/24-2.jpeg]

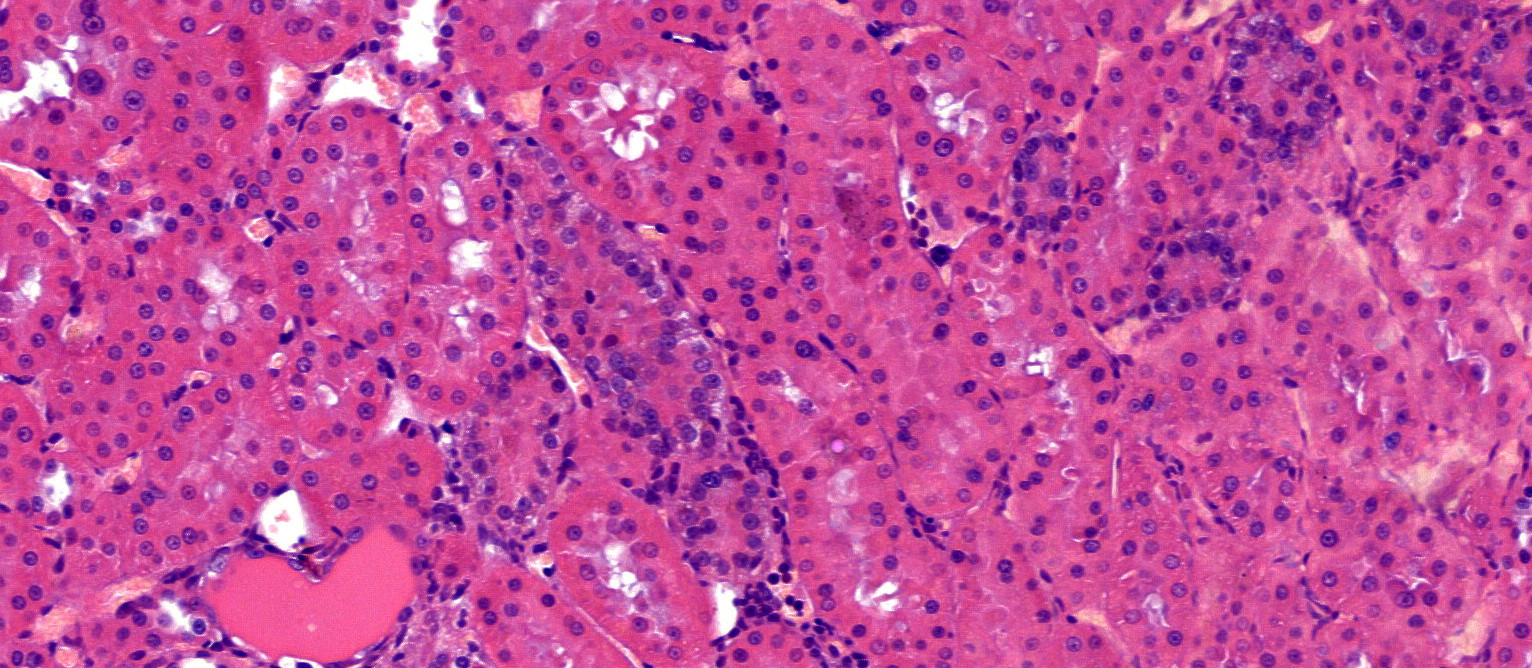

Supplement: Supplementary file 1 [file DataSheet3.ZIP › Fig 1D-HE-DKD-24/24-3.jpeg]

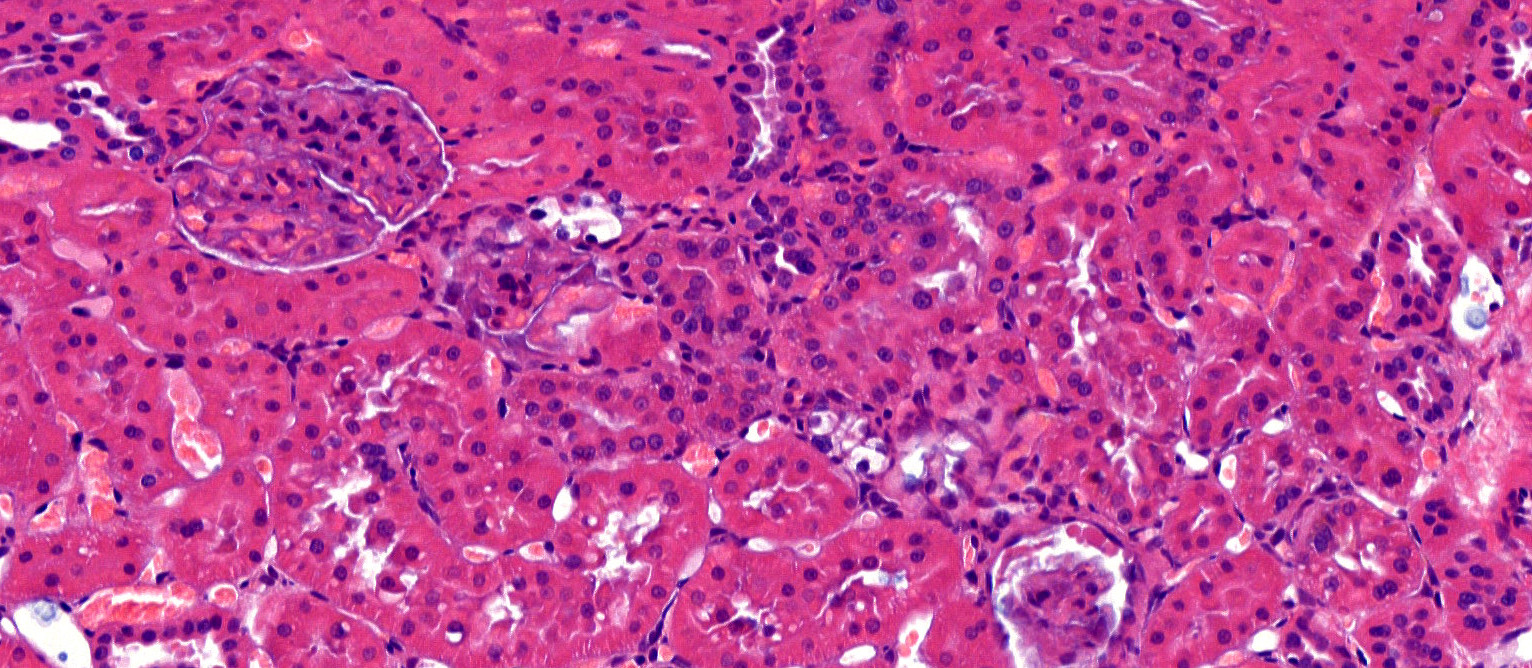

Supplement: Supplementary file 1 [file DataSheet3.ZIP › Fig 1D-HE-DKD-24/24-4.jpeg]

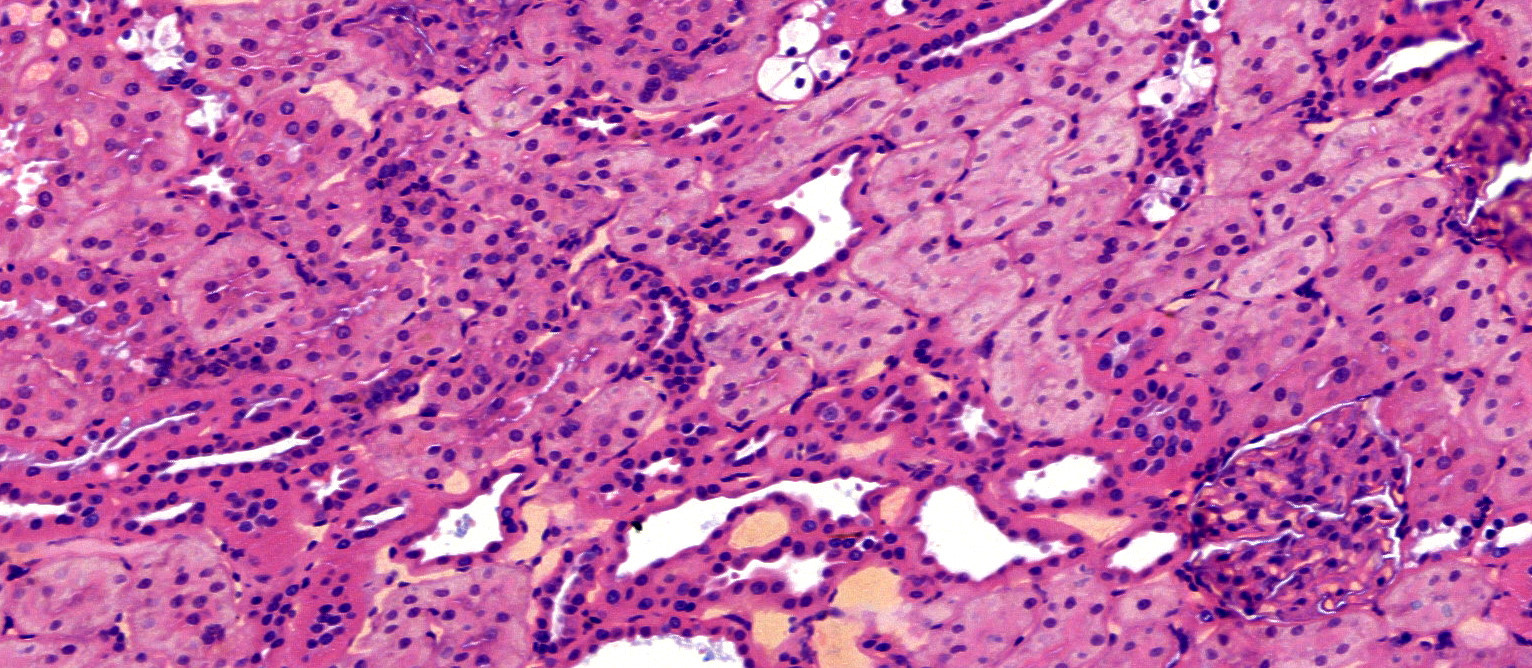

Supplement: Supplementary file 1 [file DataSheet3.ZIP › Fig 1D-HE-DKD-24/24-5.jpeg]

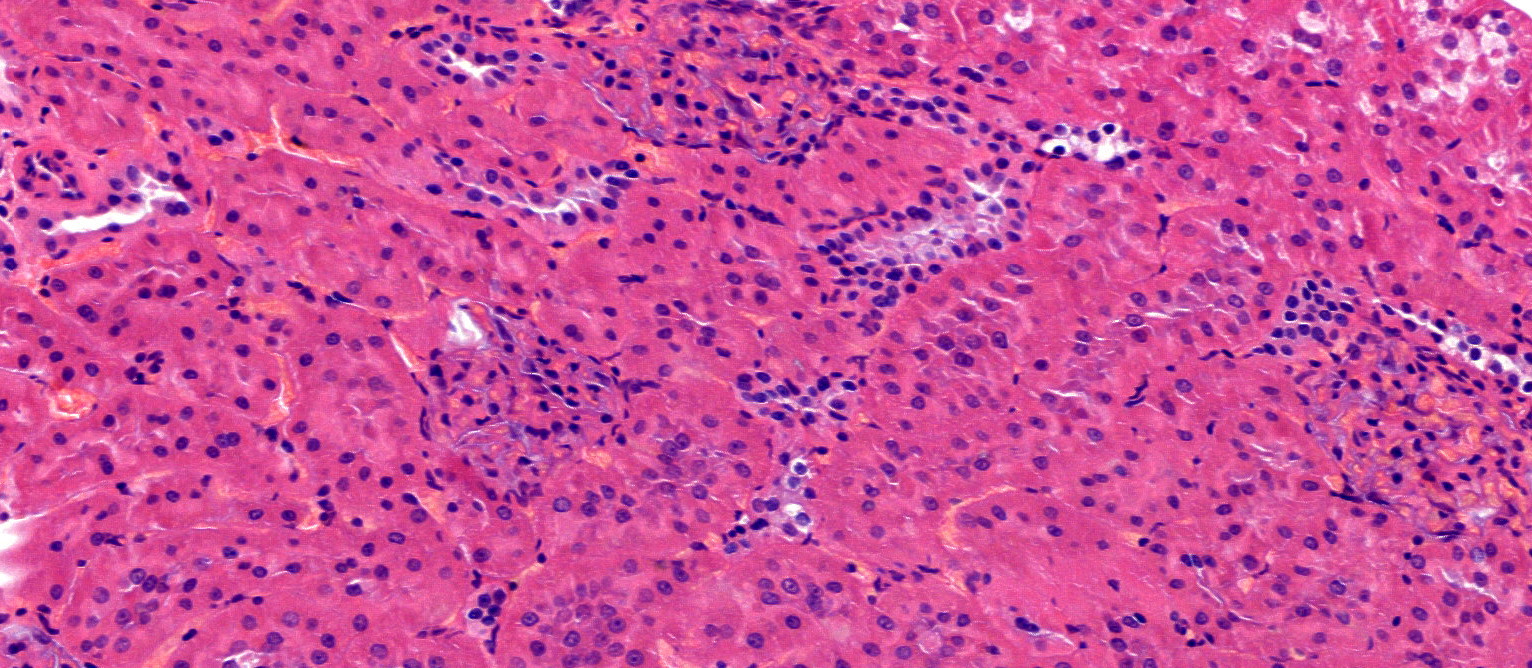

Supplement: Supplementary file 1 [file DataSheet3.ZIP › Fig 1D-HE-DKD-24/24-6.jpeg]

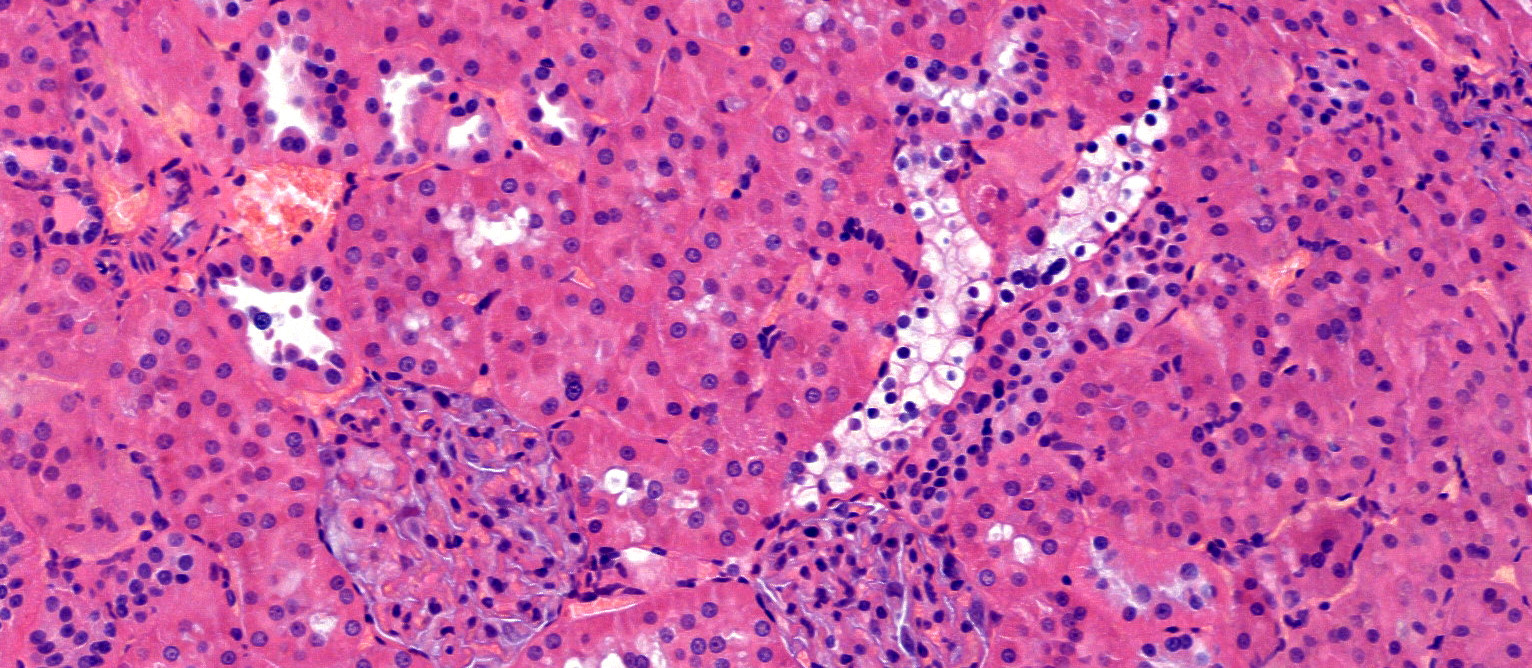

Supplement: Supplementary file 1 [file DataSheet3.ZIP › Fig 1D-HE-DKD-24/24-7.jpeg]

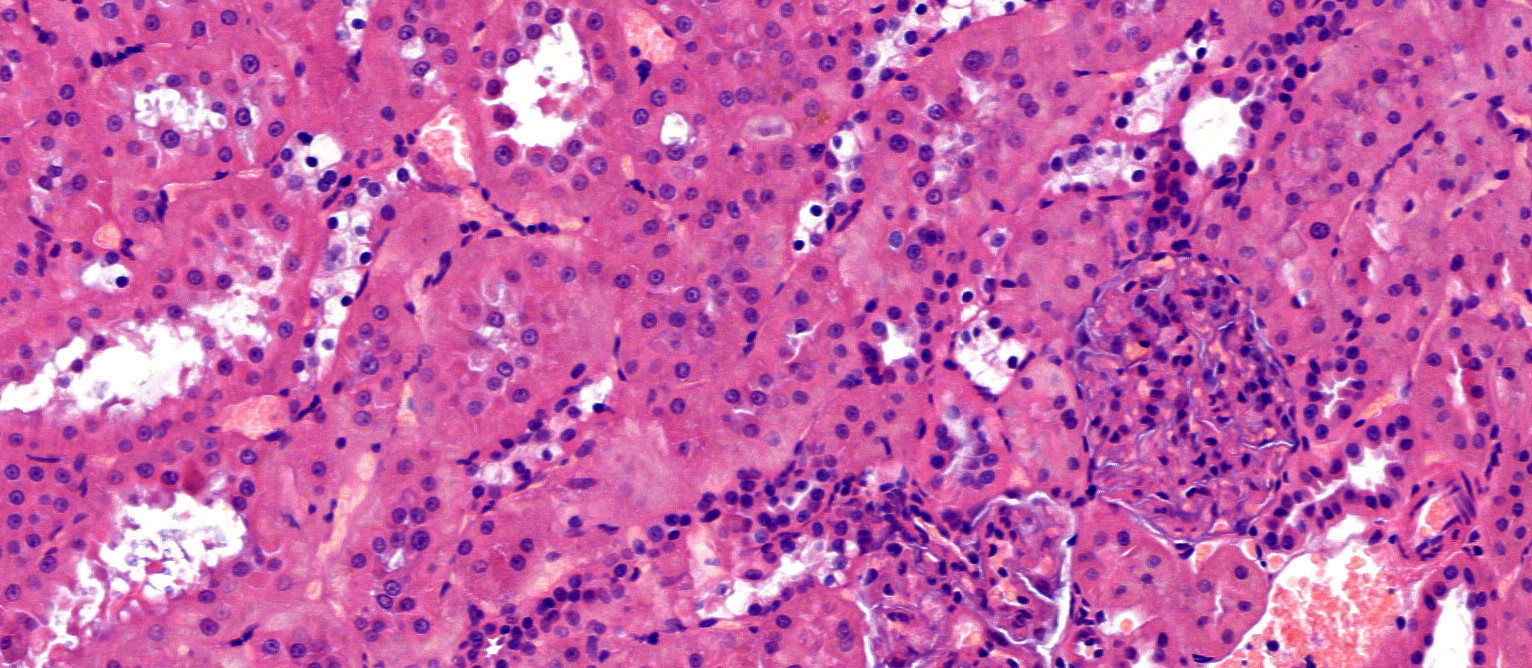

Supplement: Supplementary file 1 [file DataSheet3.ZIP › Fig 1D-HE-DKD-24/24-8.jpeg]

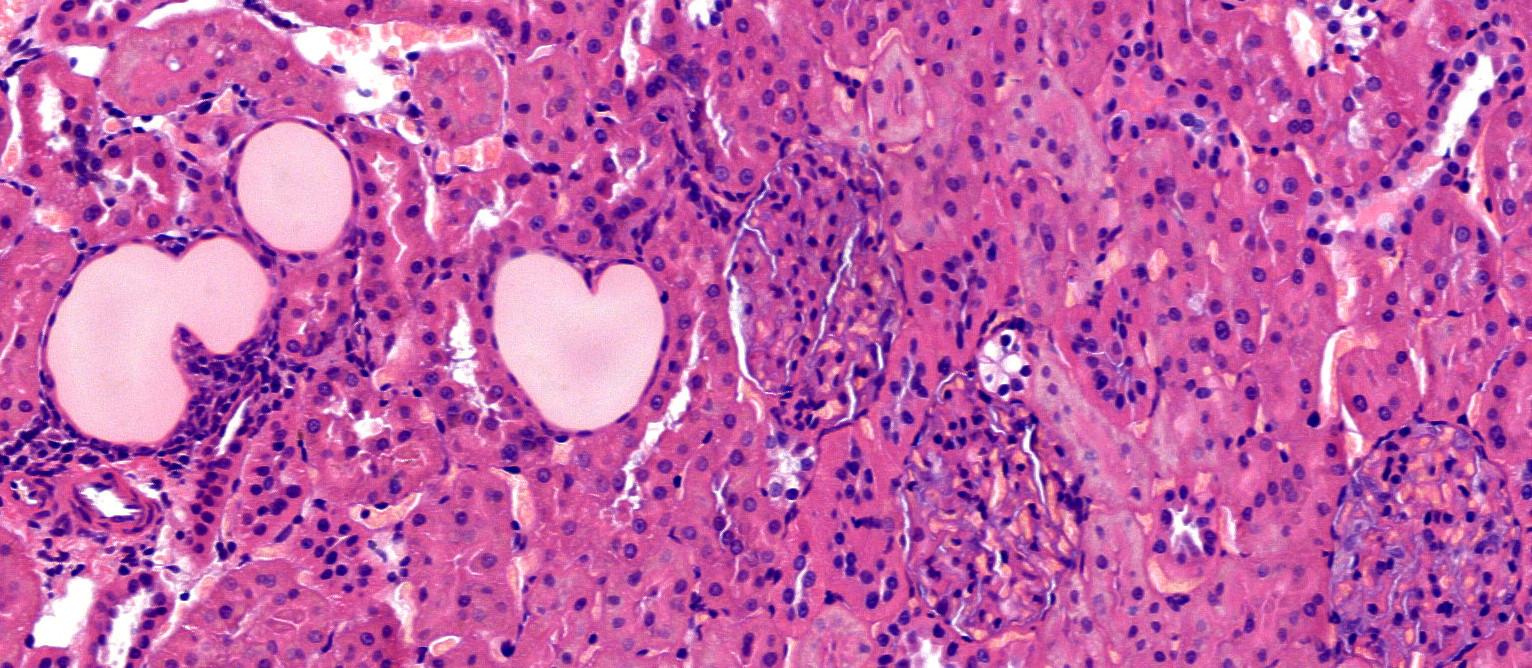

Supplement: Supplementary file 1 [file DataSheet3.ZIP › Fig 1D-HE-DKD-24/24-9.jpeg]

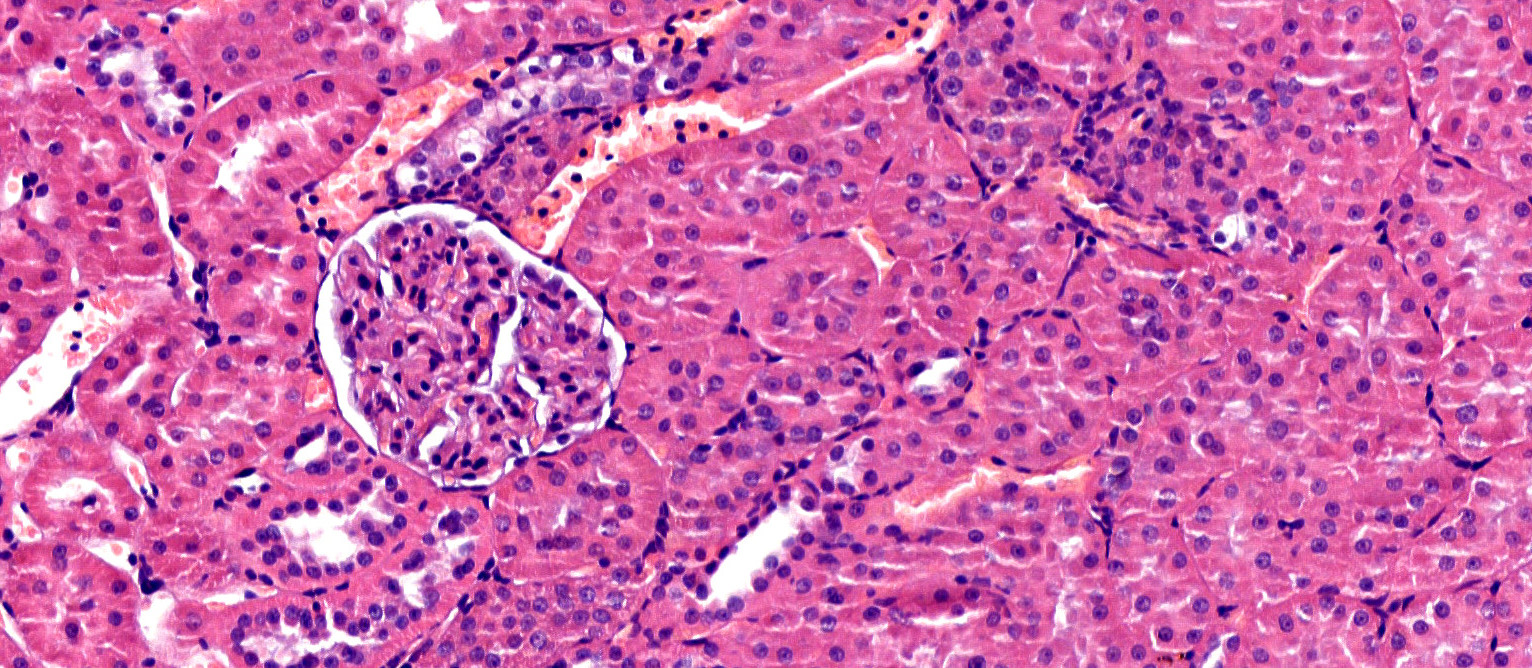

Supplement: Supplementary file 1 [file DataSheet3.ZIP › Fig 1D-HE-DKD-25/25-1.jpeg]

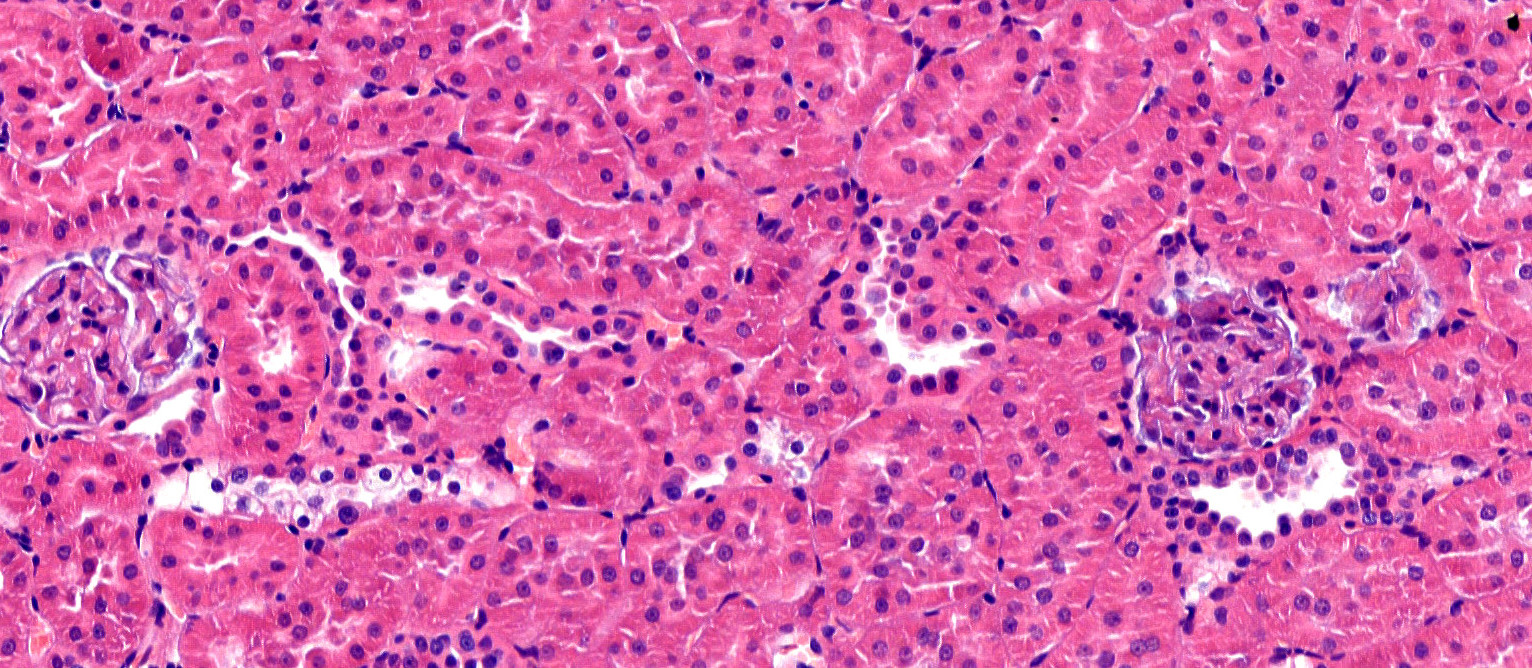

Supplement: Supplementary file 1 [file DataSheet3.ZIP › Fig 1D-HE-DKD-25/25-10.jpeg]

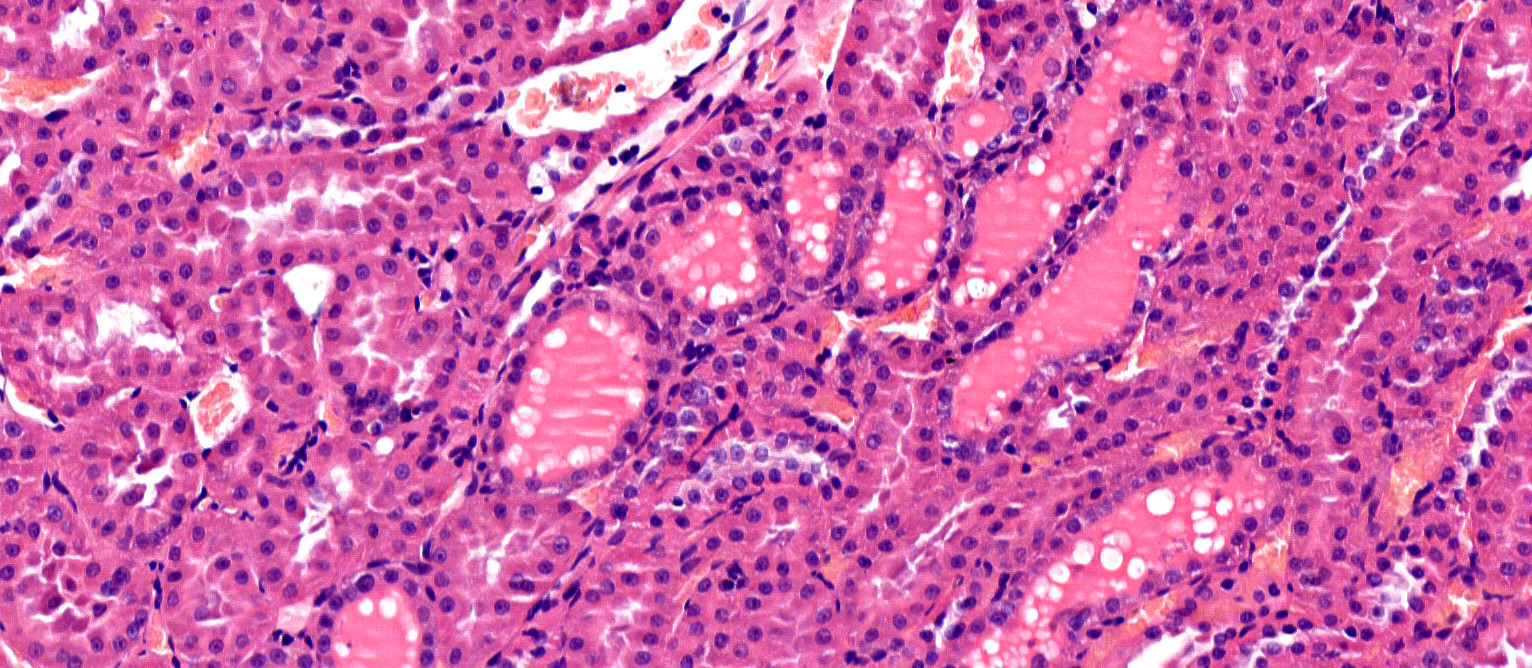

Supplement: Supplementary file 1 [file DataSheet3.ZIP › Fig 1D-HE-DKD-25/25-2.jpeg]

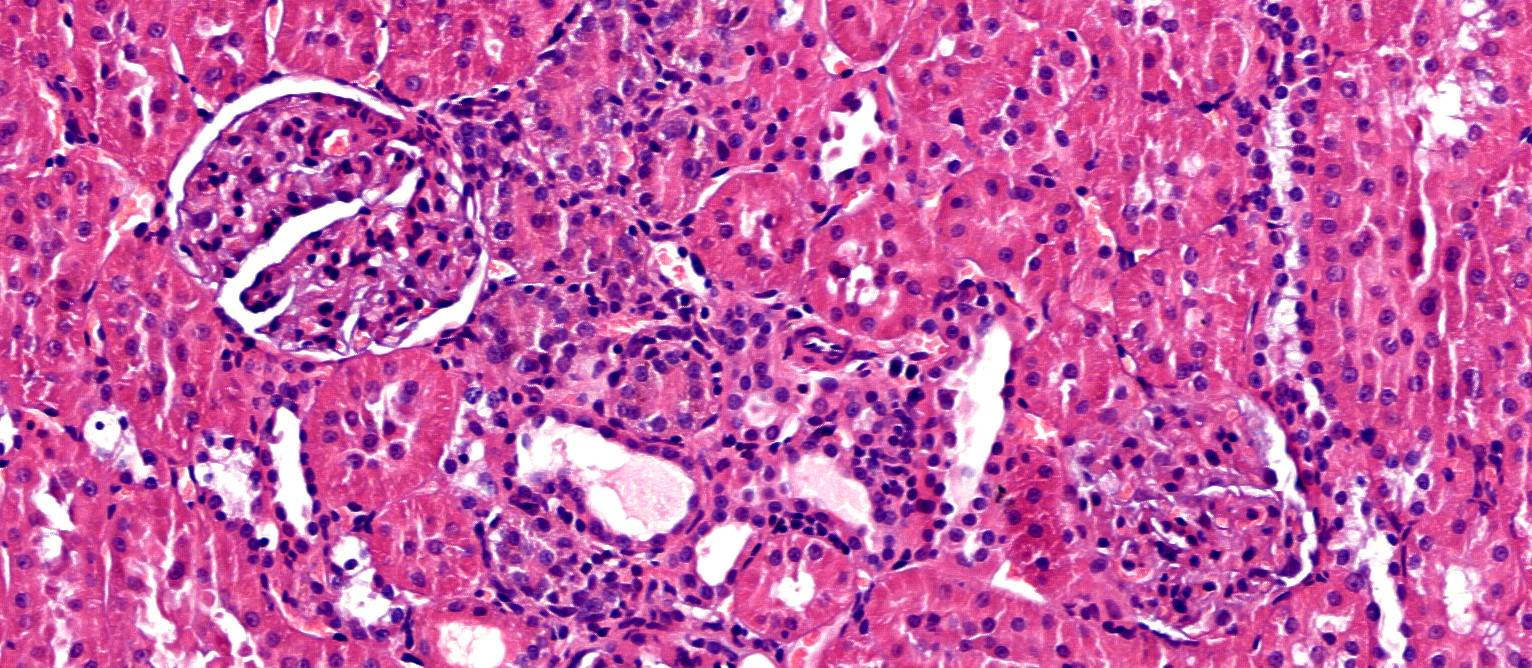

Supplement: Supplementary file 1 [file DataSheet3.ZIP › Fig 1D-HE-DKD-25/25-3.jpeg]

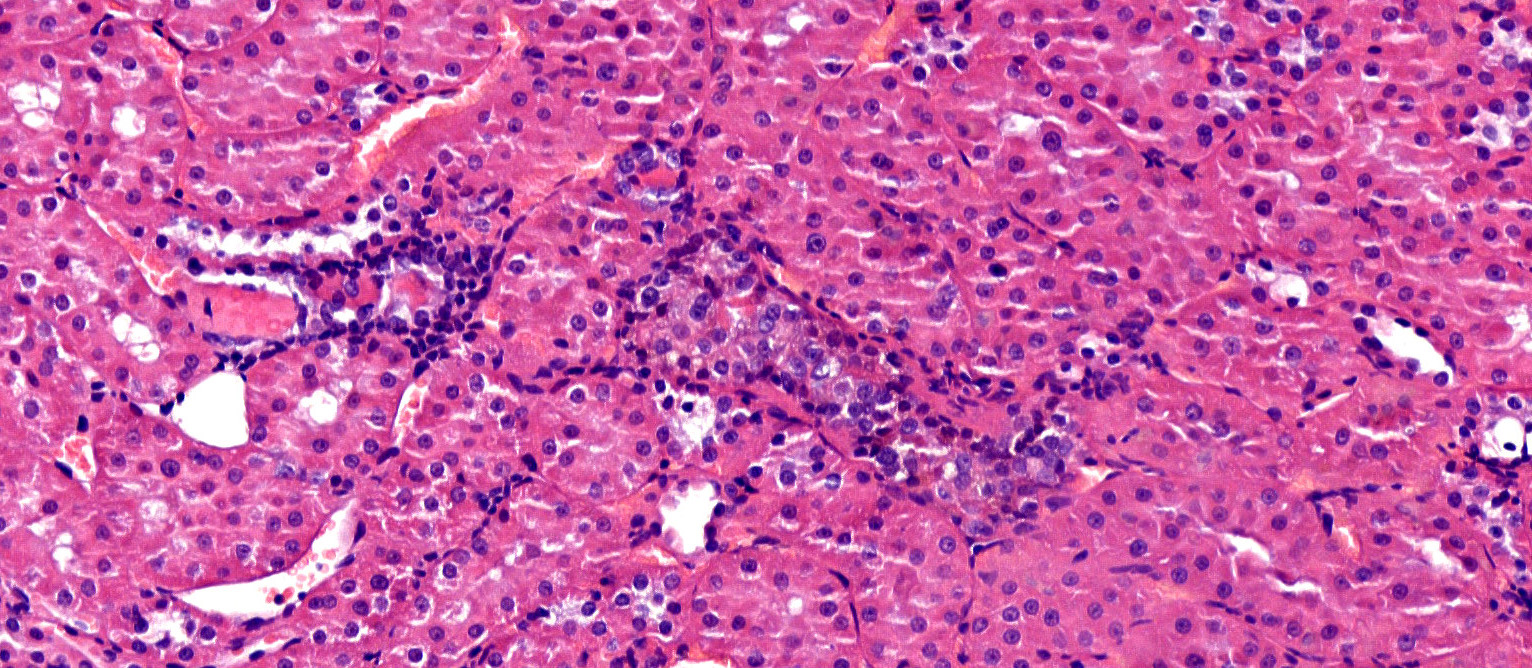

Supplement: Supplementary file 1 [file DataSheet3.ZIP › Fig 1D-HE-DKD-25/25-4.jpeg]

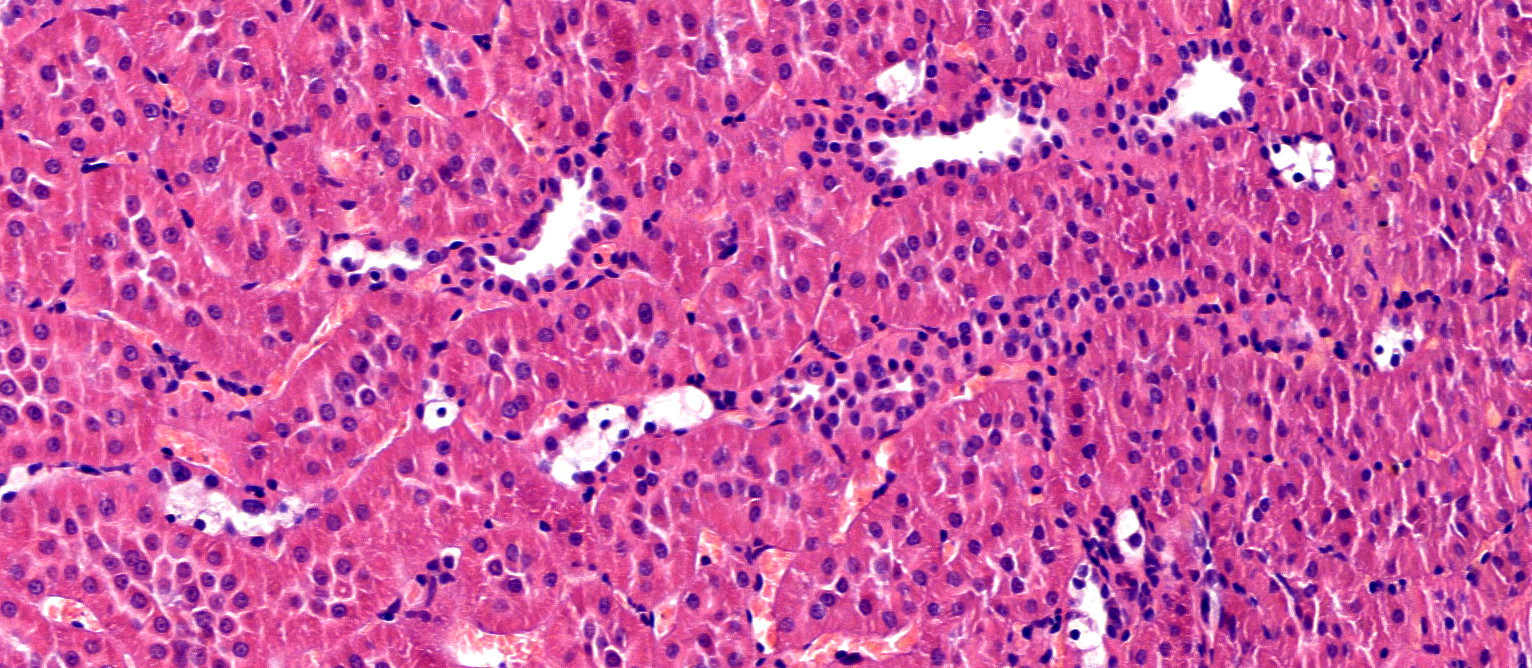

Supplement: Supplementary file 1 [file DataSheet3.ZIP › Fig 1D-HE-DKD-25/25-5.jpeg]

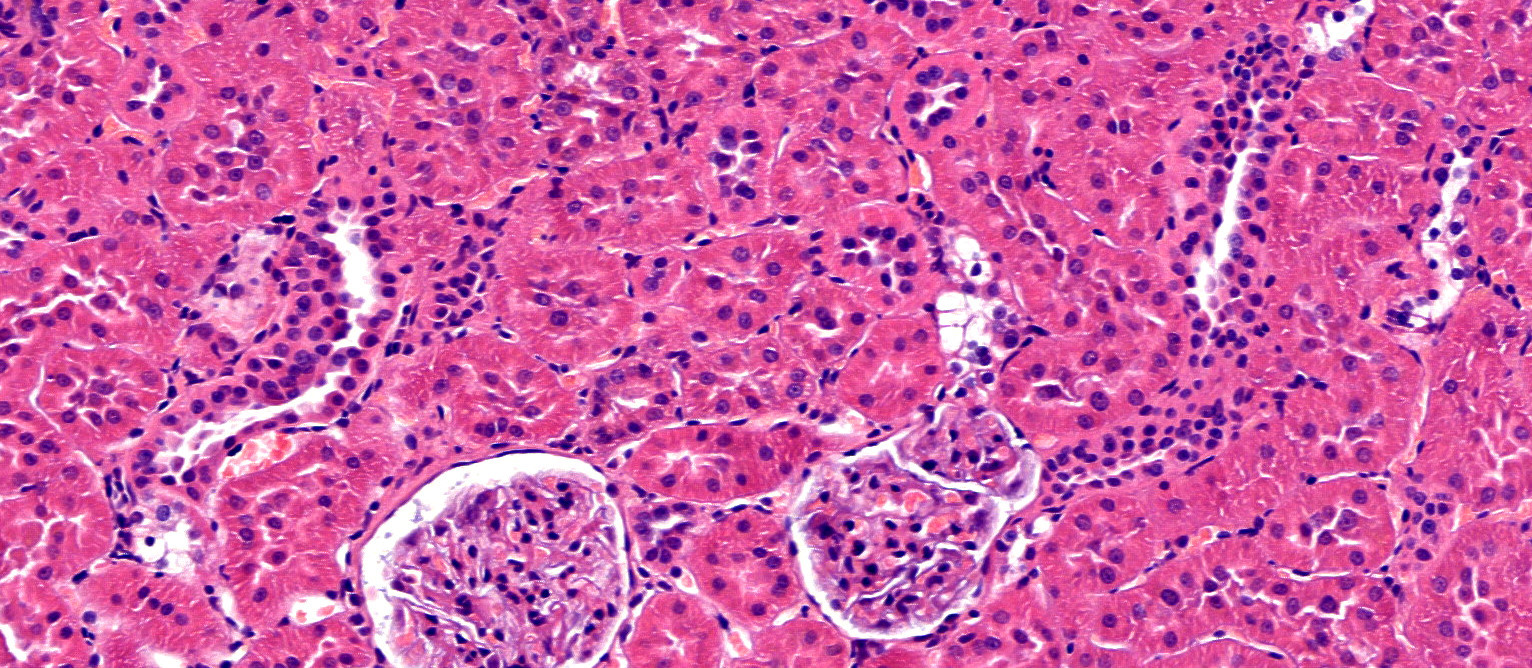

Supplement: Supplementary file 1 [file DataSheet3.ZIP › Fig 1D-HE-DKD-25/25-6.jpeg]

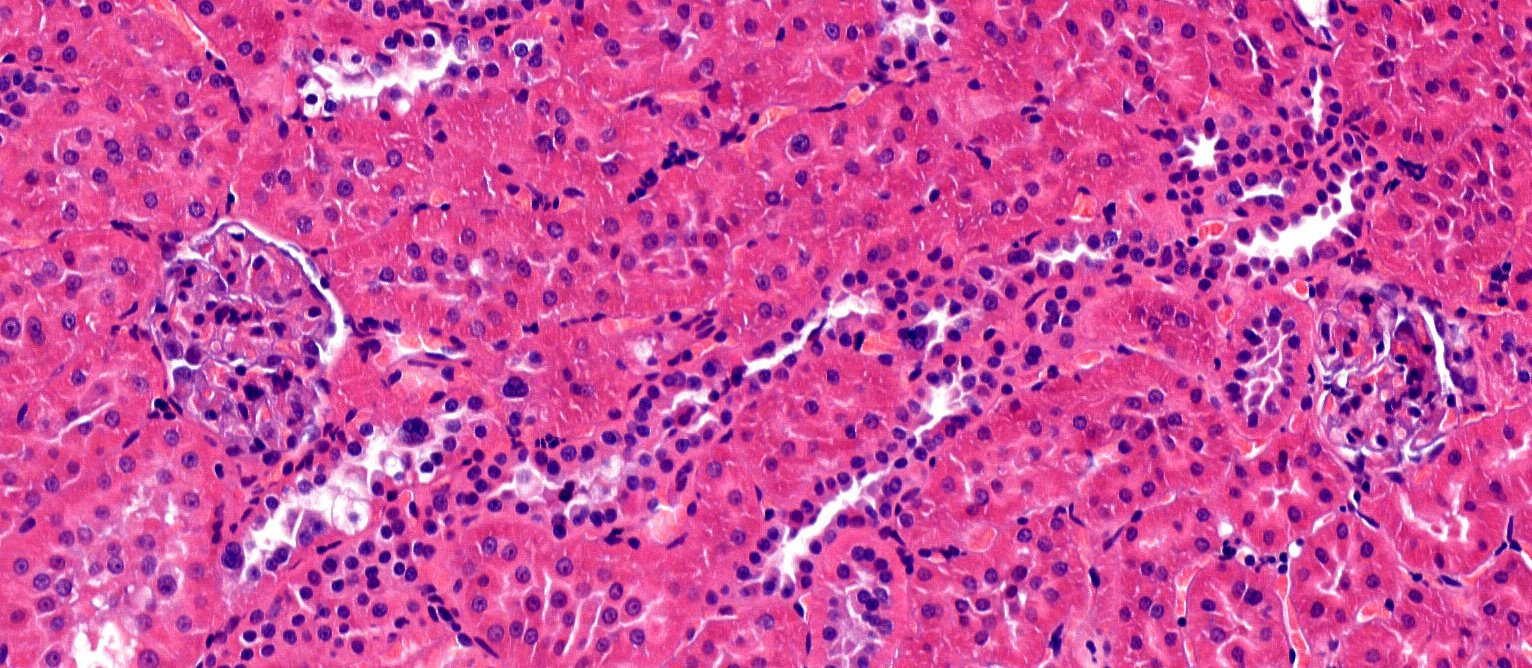

Supplement: Supplementary file 1 [file DataSheet3.ZIP › Fig 1D-HE-DKD-25/25-7.jpeg]

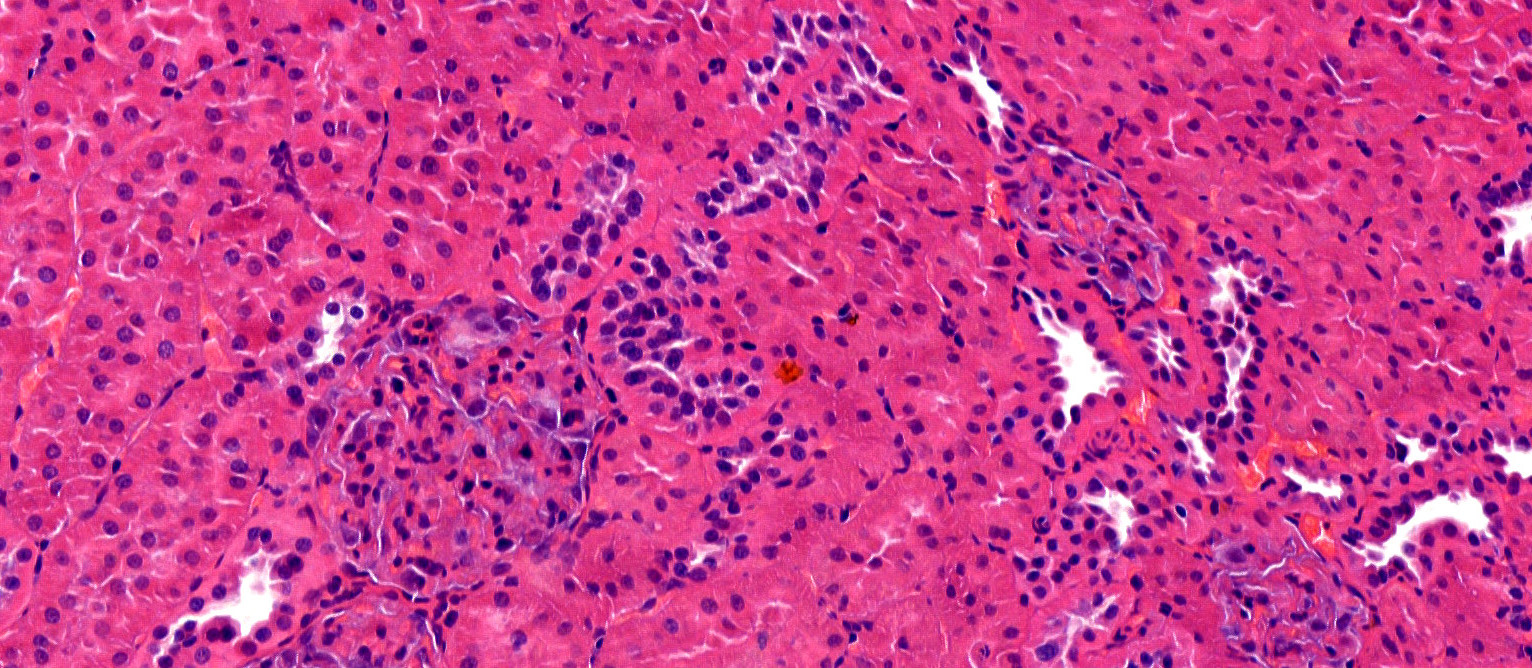

Supplement: Supplementary file 1 [file DataSheet3.ZIP › Fig 1D-HE-DKD-25/25-8.jpeg]

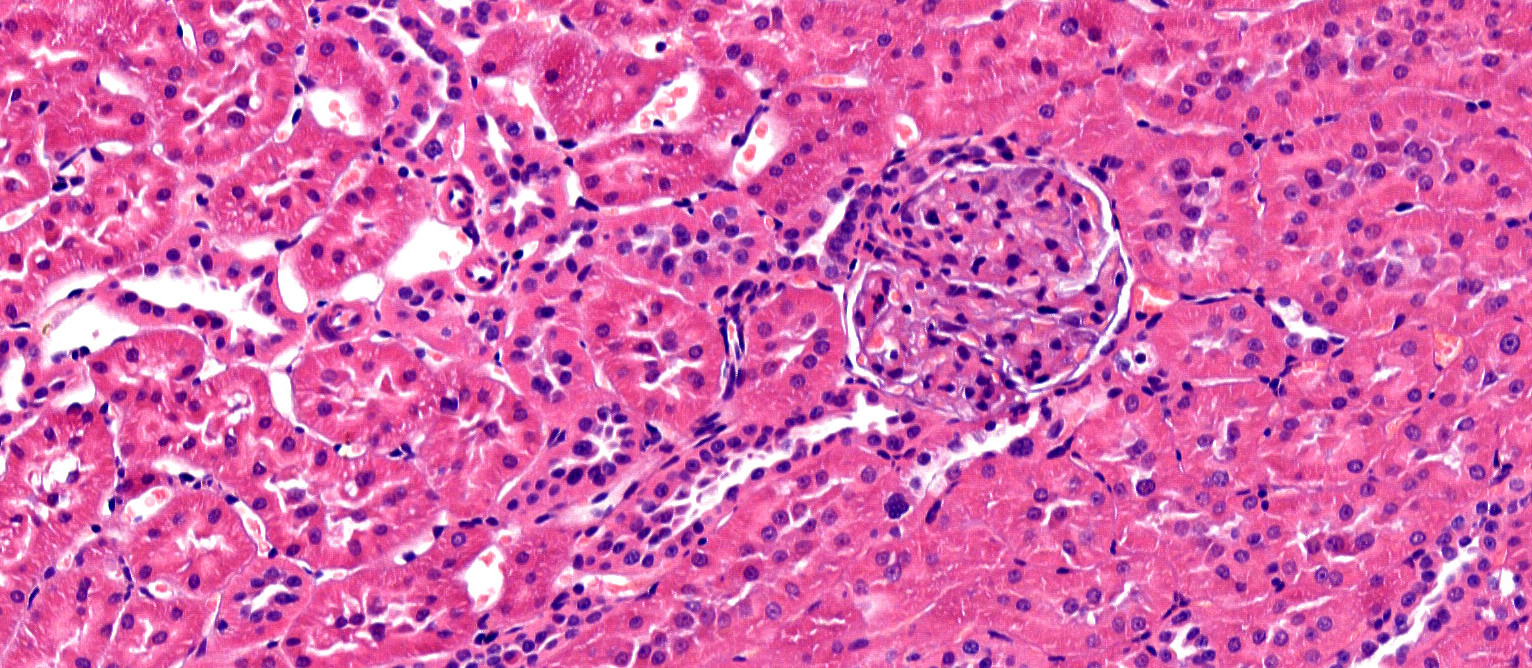

Supplement: Supplementary file 1 [file DataSheet3.ZIP › Fig 1D-HE-DKD-25/25-9.jpeg]

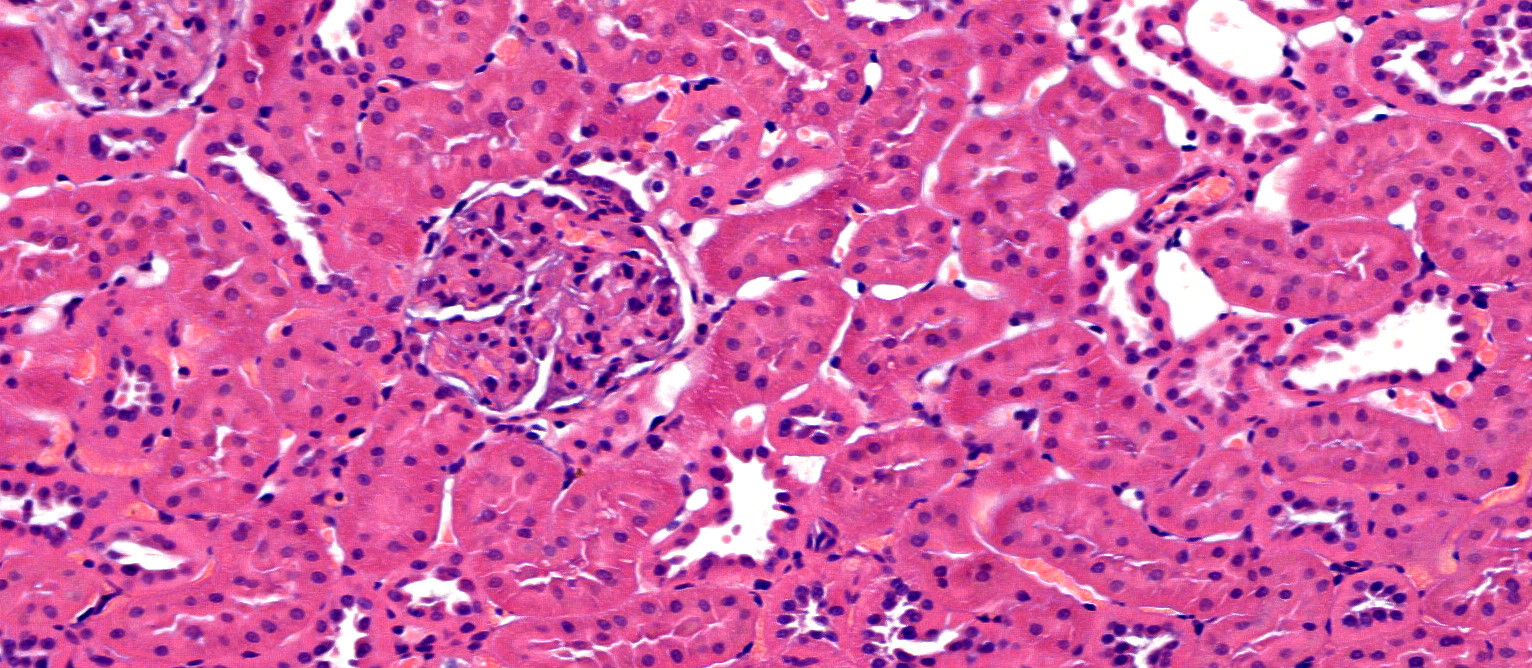

Supplement: Supplementary file 1 [file DataSheet3.ZIP › Fig 1D-HE-TSF-53/53-1.jpeg]

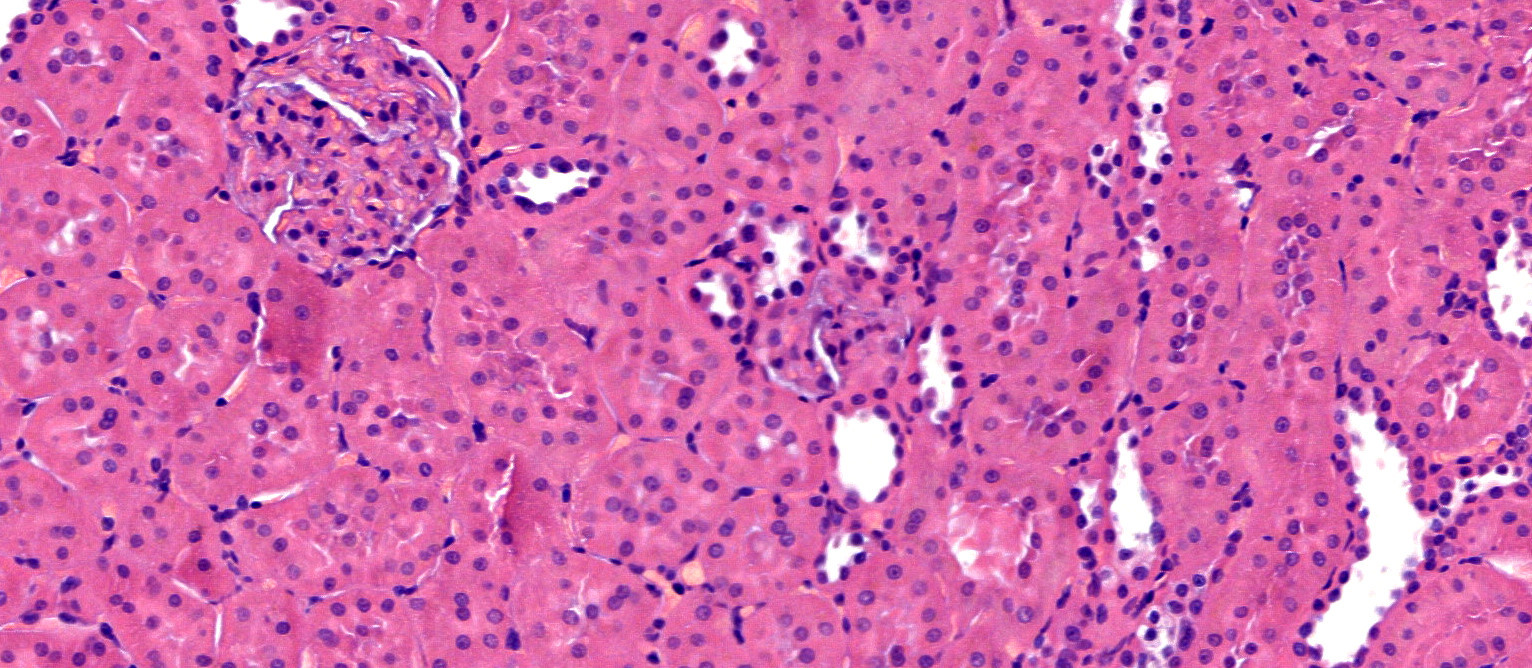

Supplement: Supplementary file 1 [file DataSheet3.ZIP › Fig 1D-HE-TSF-53/53-10.jpeg]

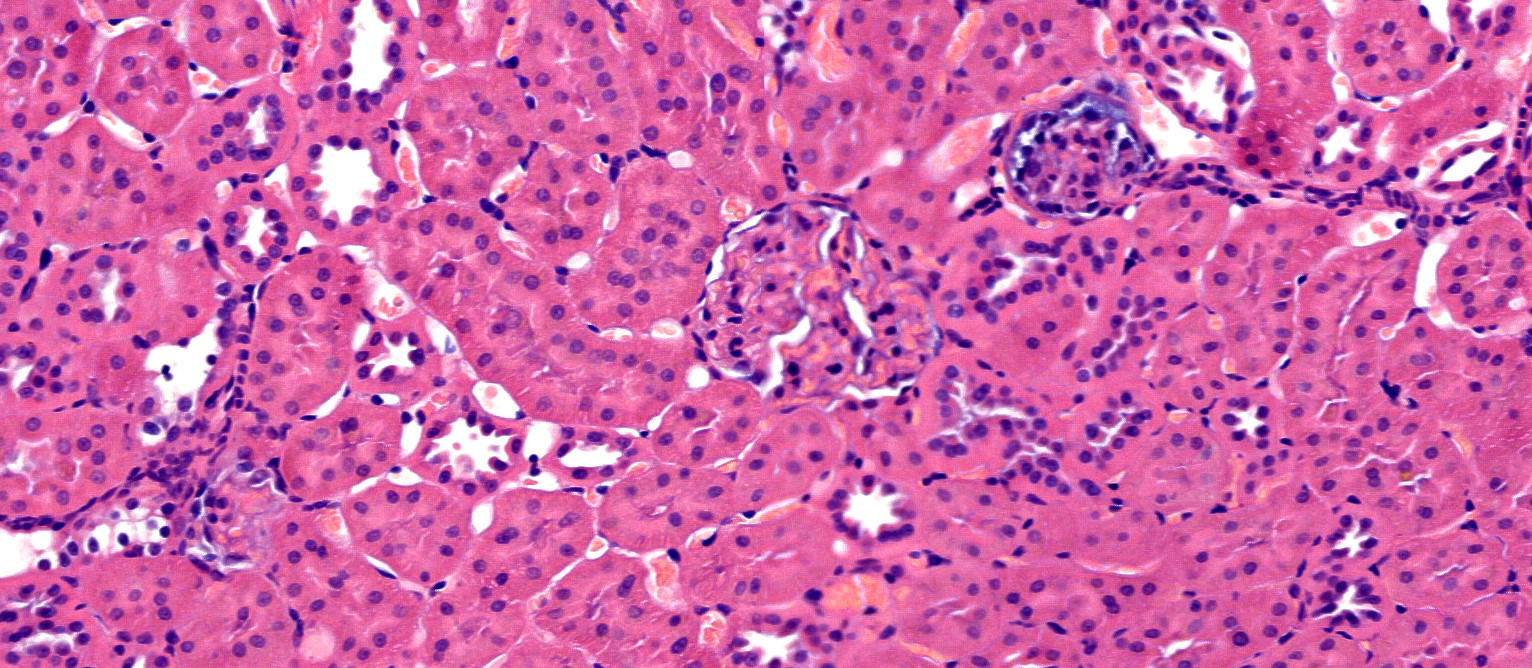

Supplement: Supplementary file 1 [file DataSheet3.ZIP › Fig 1D-HE-TSF-53/53-2.jpeg]

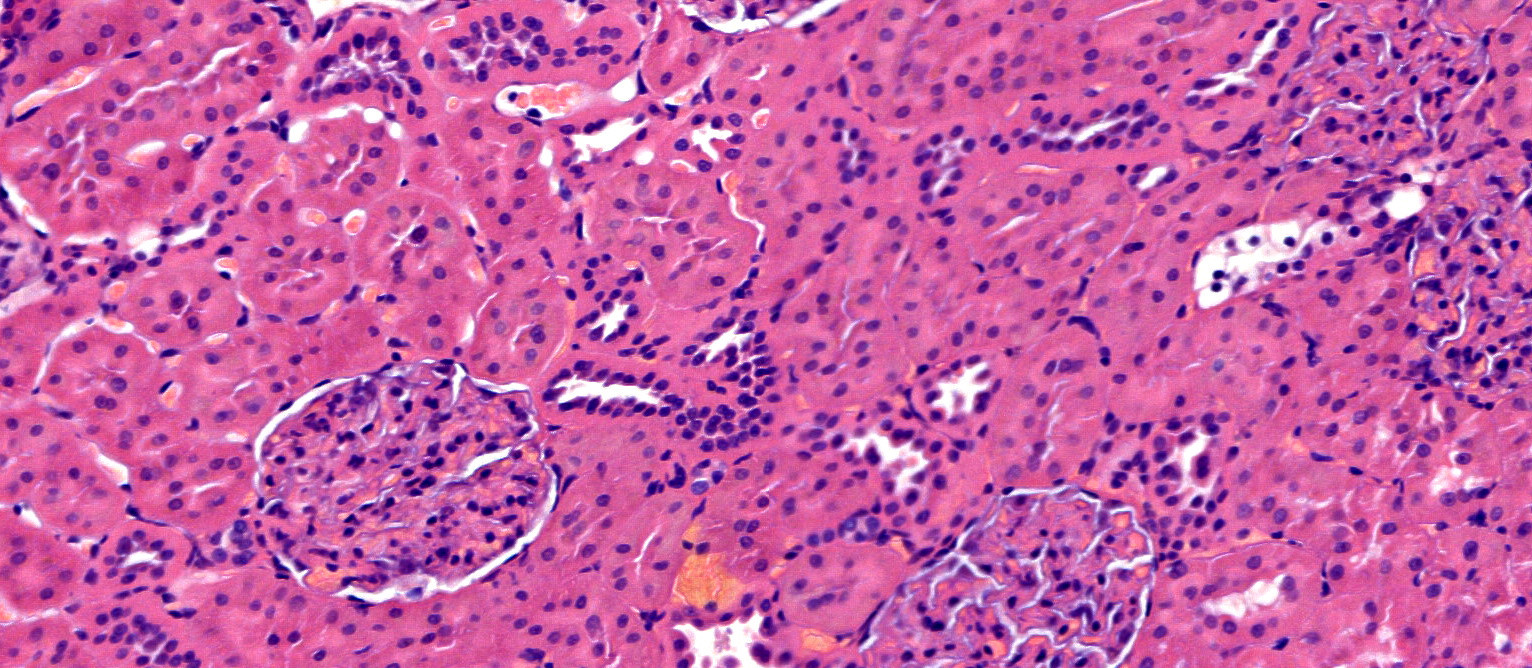

Supplement: Supplementary file 1 [file DataSheet3.ZIP › Fig 1D-HE-TSF-53/53-3.jpeg]

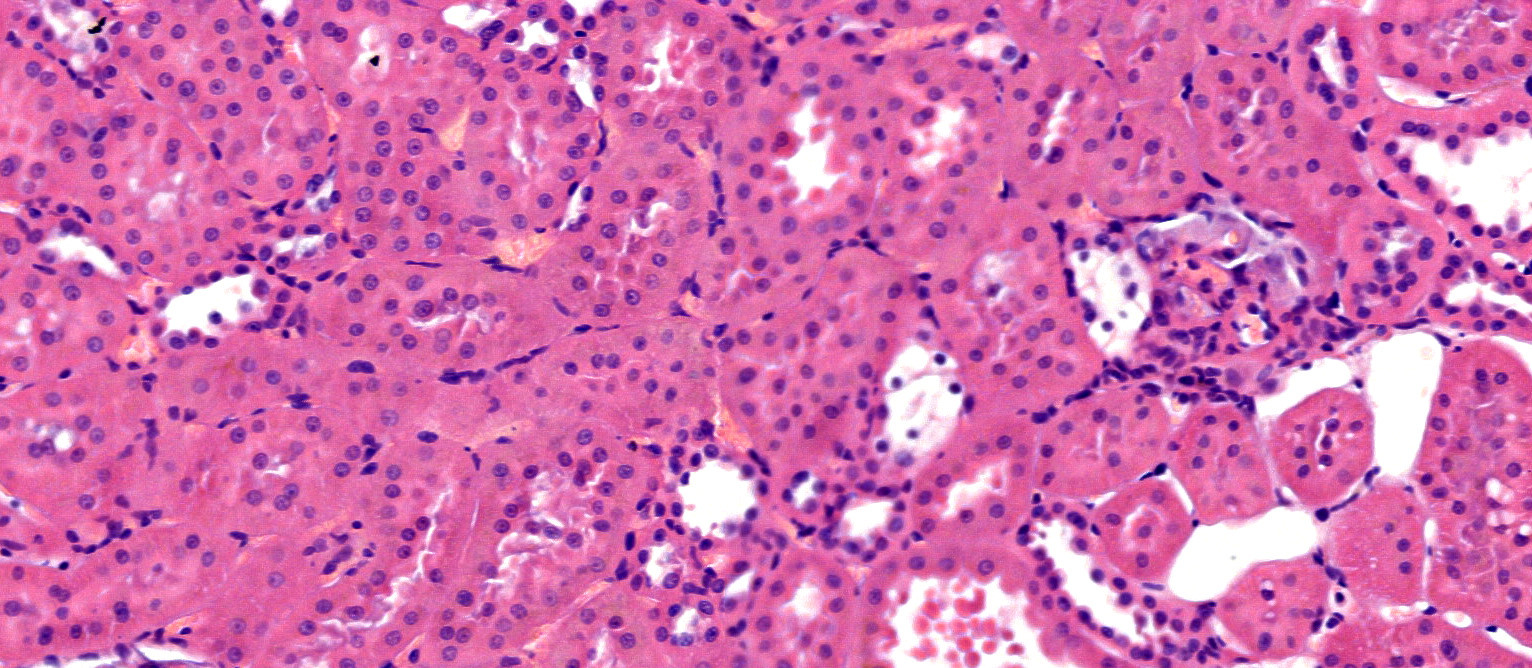

Supplement: Supplementary file 1 [file DataSheet3.ZIP › Fig 1D-HE-TSF-53/53-4.jpeg]

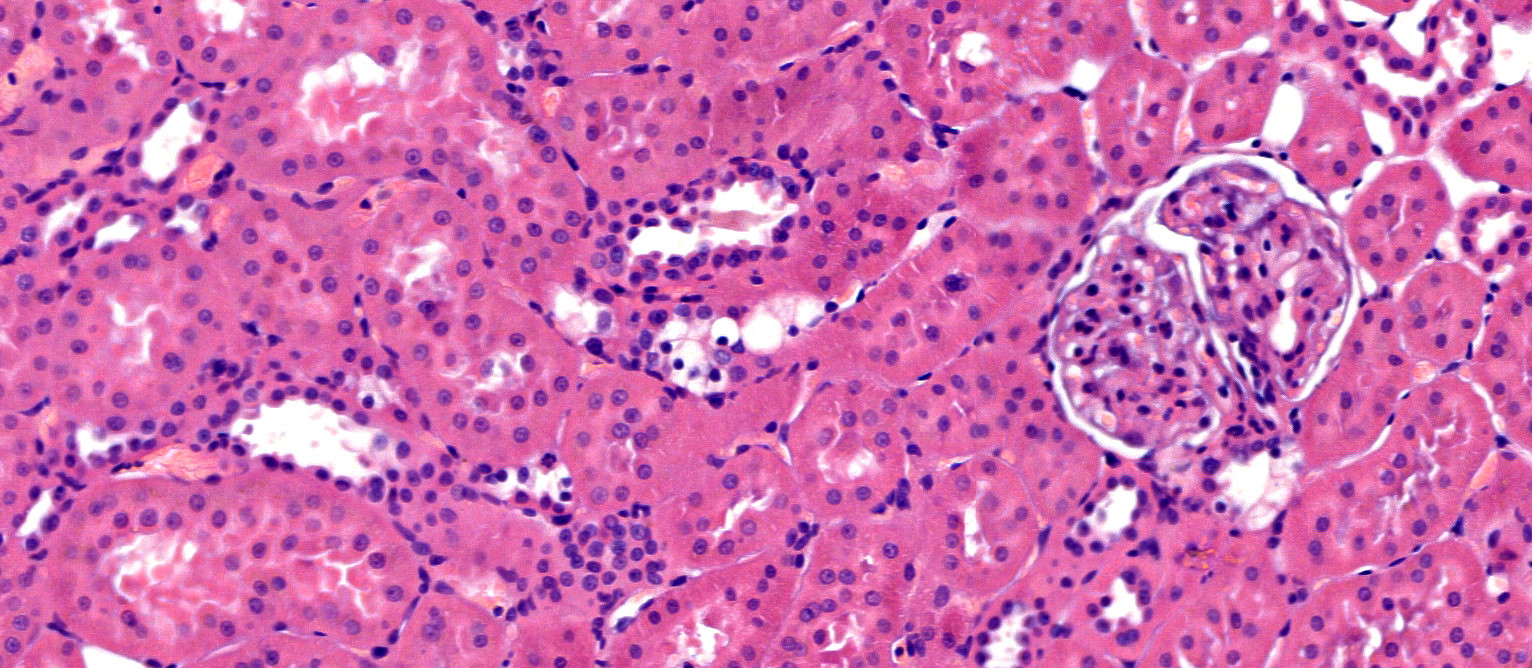

Supplement: Supplementary file 1 [file DataSheet3.ZIP › Fig 1D-HE-TSF-53/53-5.jpeg]

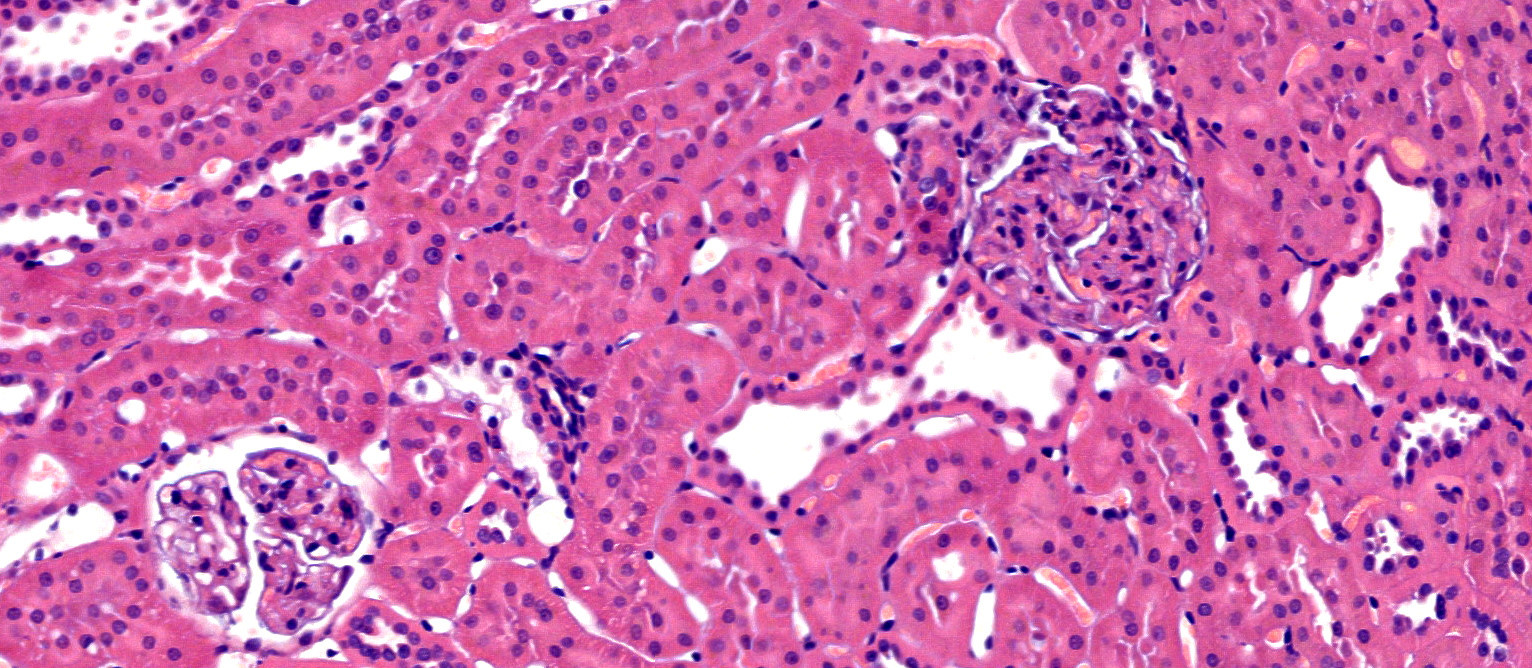

Supplement: Supplementary file 1 [file DataSheet3.ZIP › Fig 1D-HE-TSF-53/53-6.jpeg]

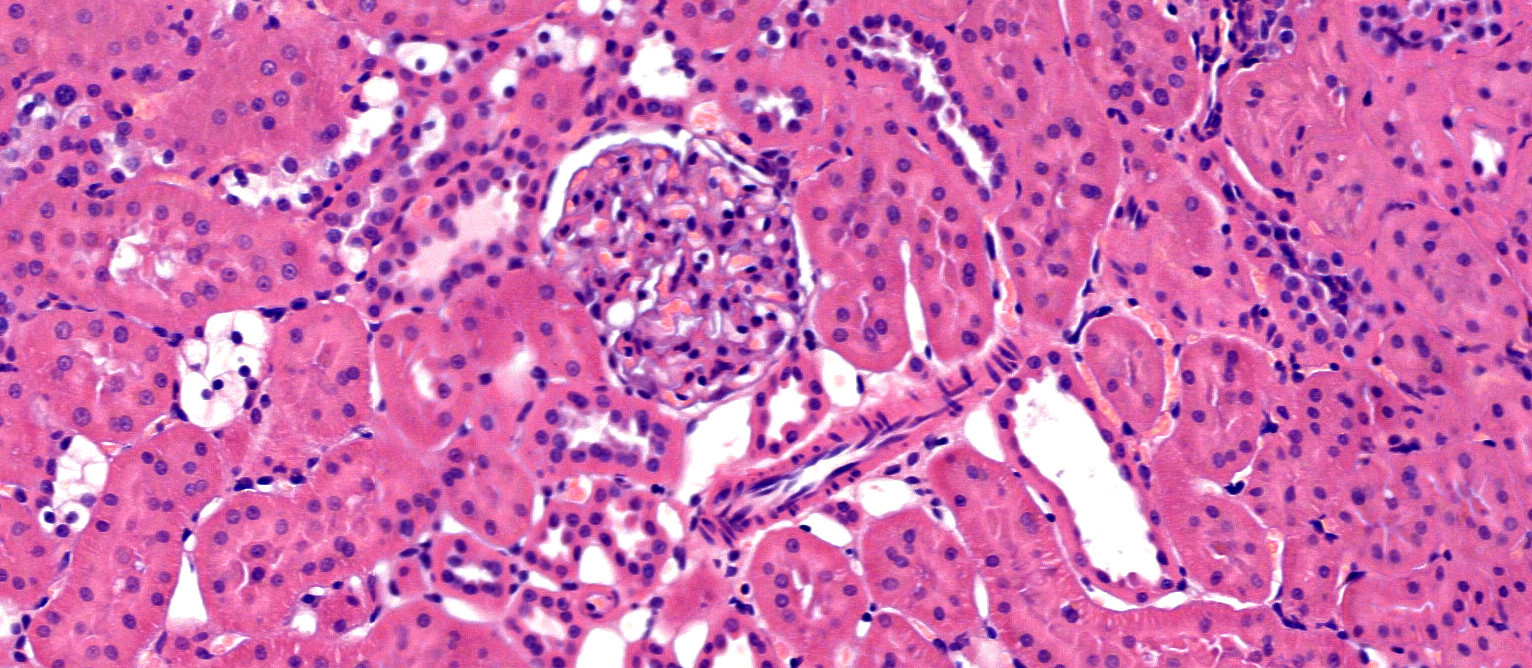

Supplement: Supplementary file 1 [file DataSheet3.ZIP › Fig 1D-HE-TSF-53/53-7.jpeg]

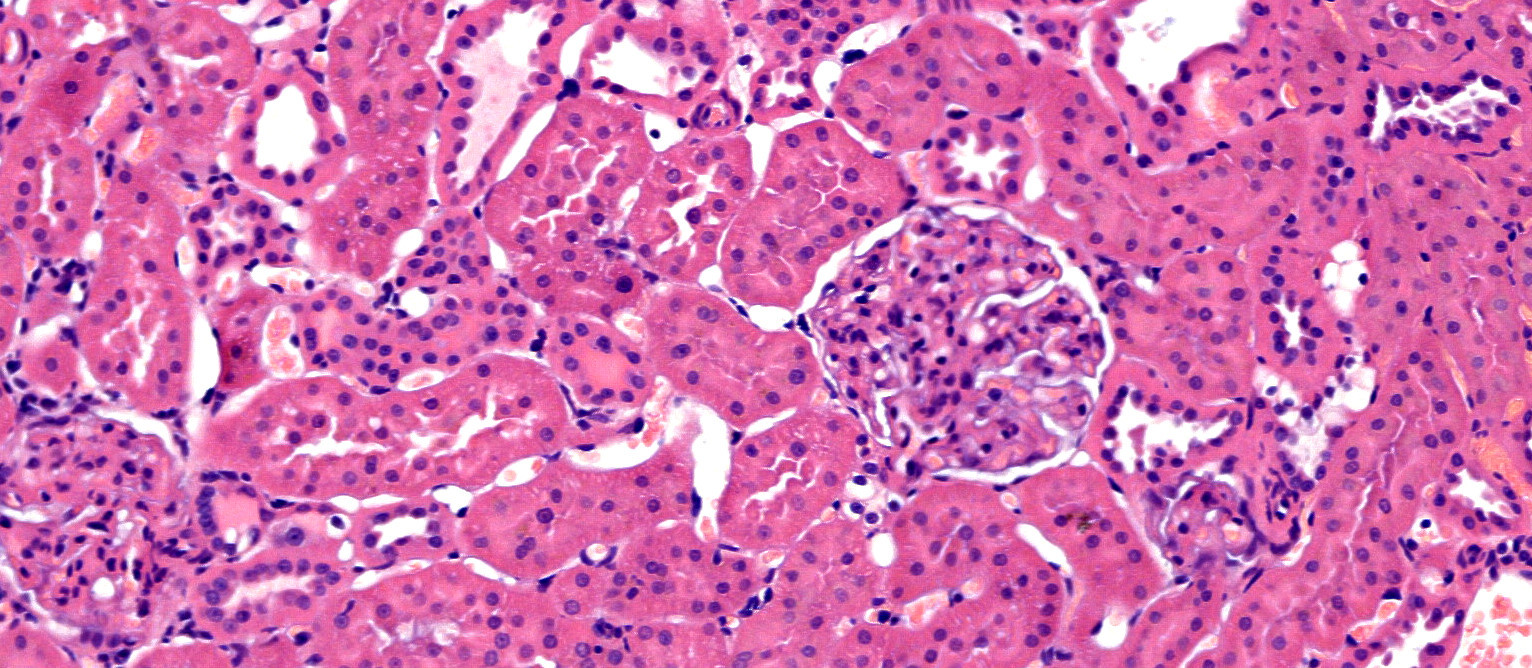

Supplement: Supplementary file 1 [file DataSheet3.ZIP › Fig 1D-HE-TSF-53/53-8.jpeg]

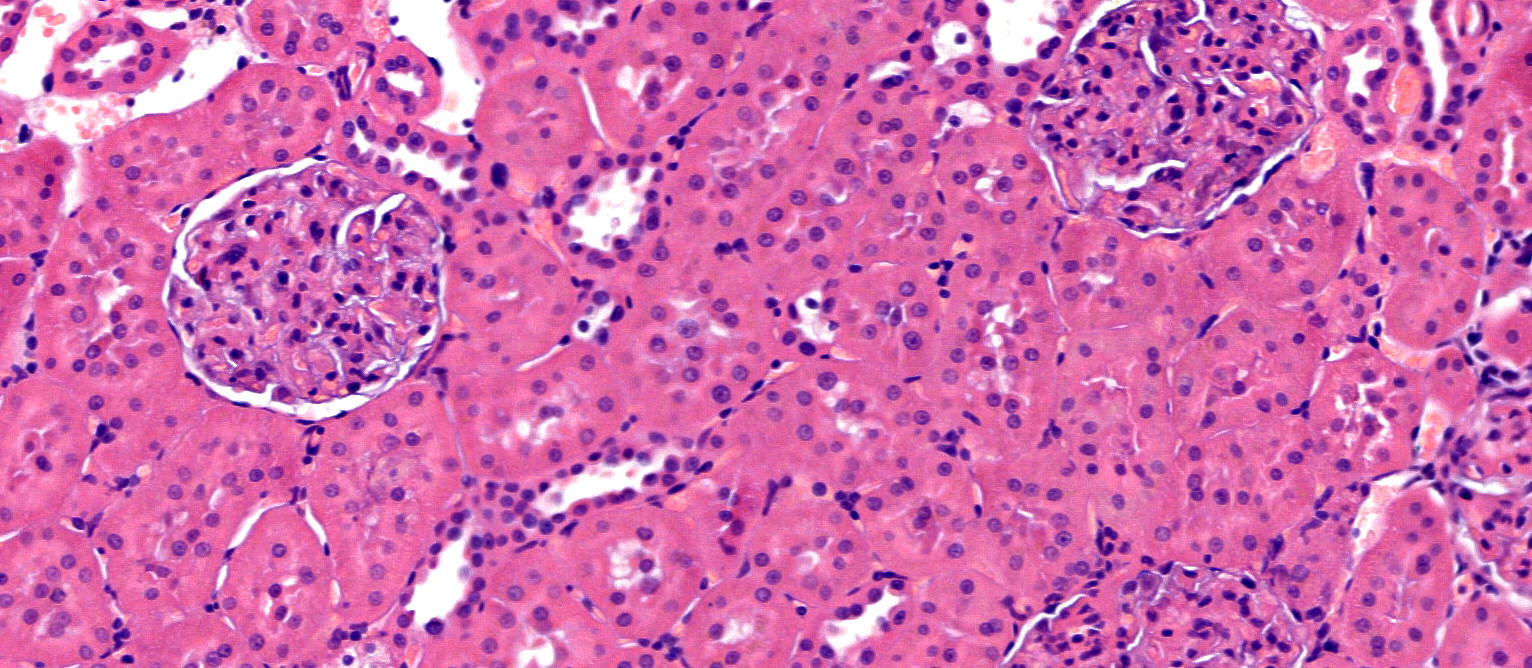

Supplement: Supplementary file 1 [file DataSheet3.ZIP › Fig 1D-HE-TSF-53/53-9.jpeg]

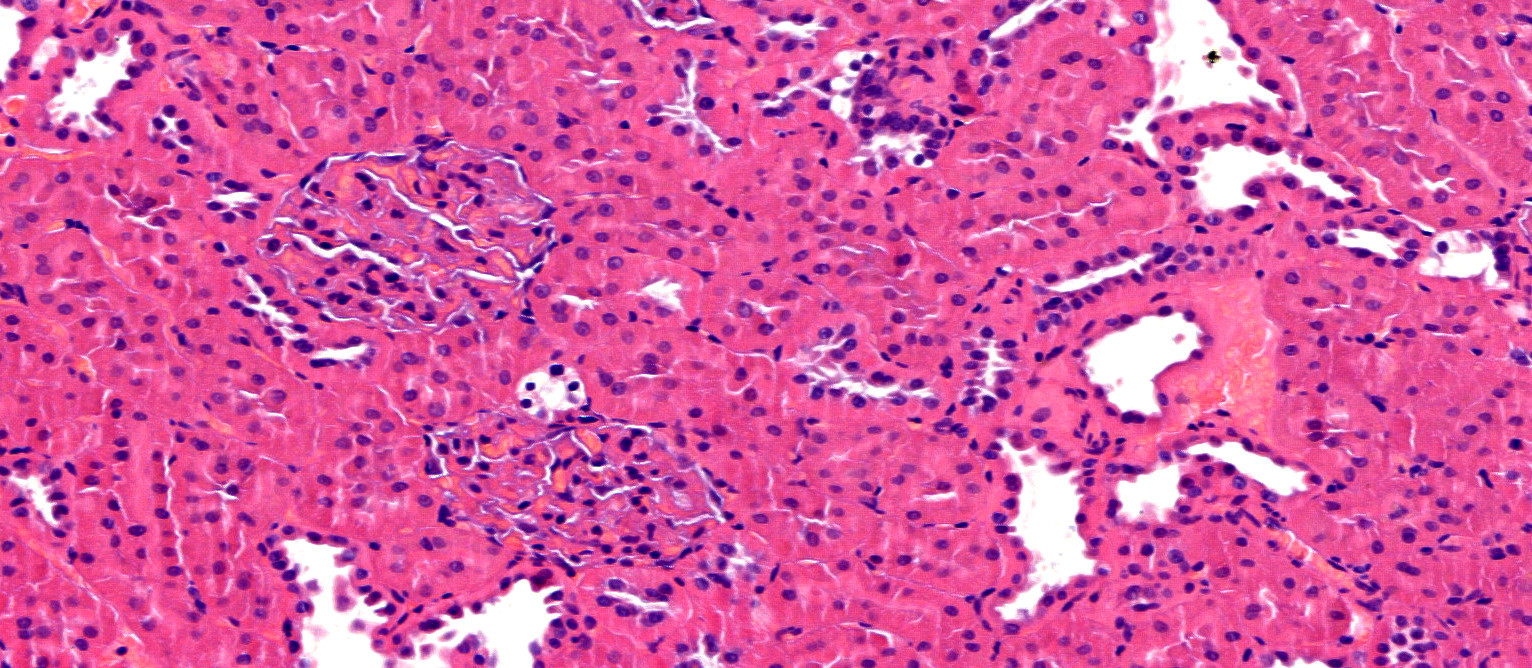

Supplement: Supplementary file 1 [file DataSheet3.ZIP › Fig 1D-HE-TSF-56/56-1.jpeg]

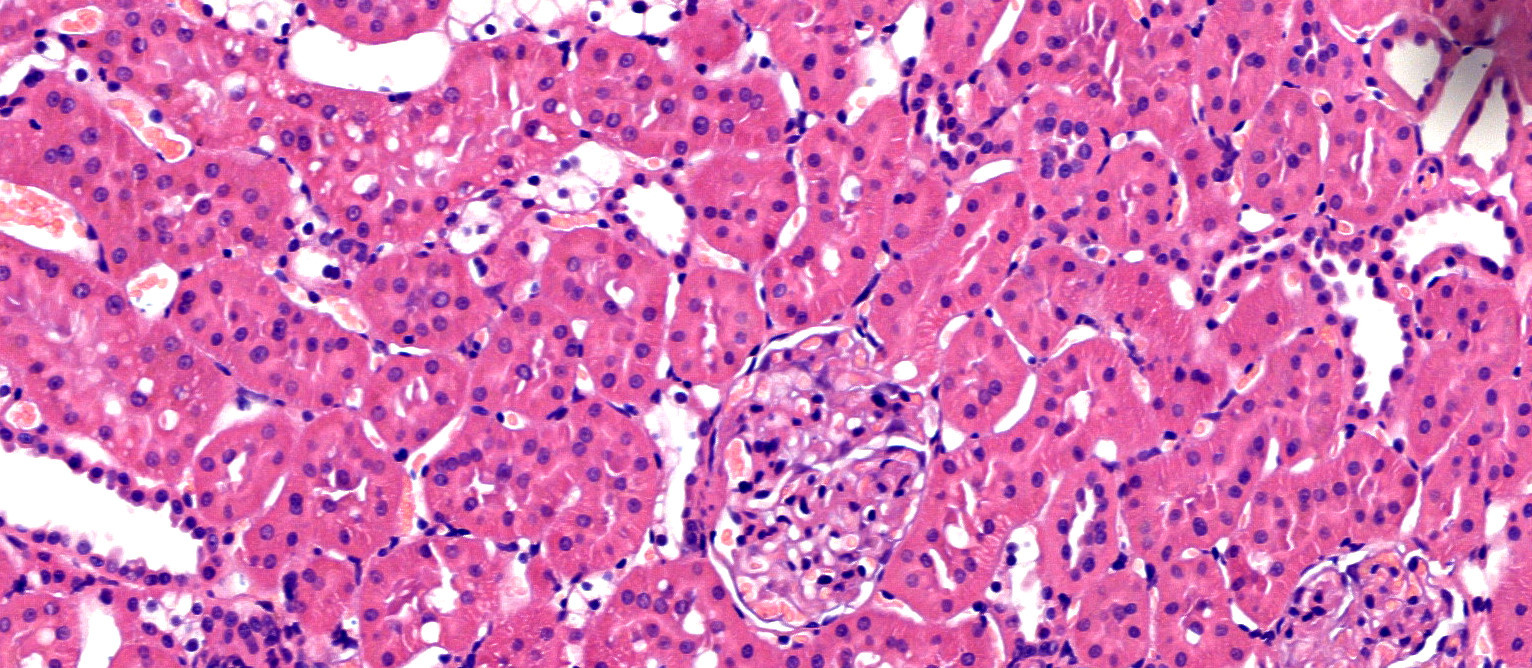

Supplement: Supplementary file 1 [file DataSheet3.ZIP › Fig 1D-HE-TSF-56/56-10.jpeg]

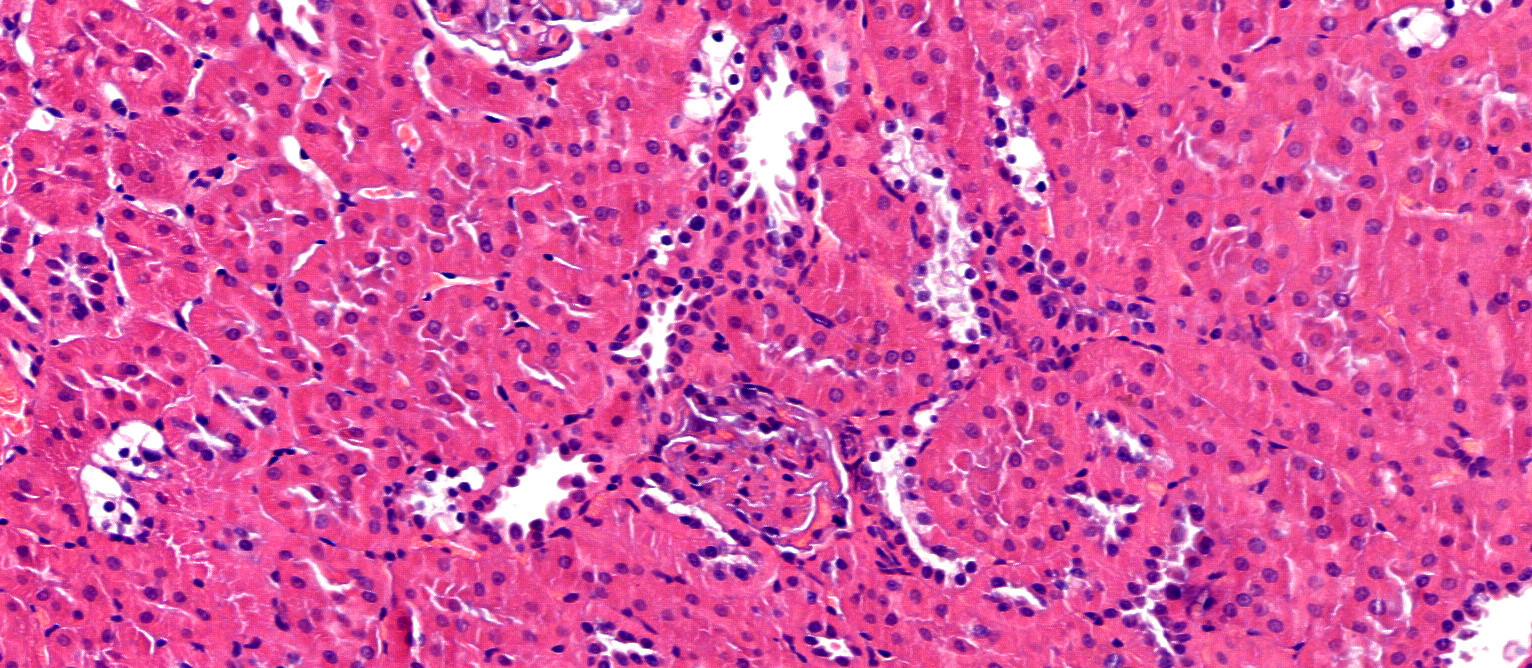

Supplement: Supplementary file 1 [file DataSheet3.ZIP › Fig 1D-HE-TSF-56/56-2.jpeg]

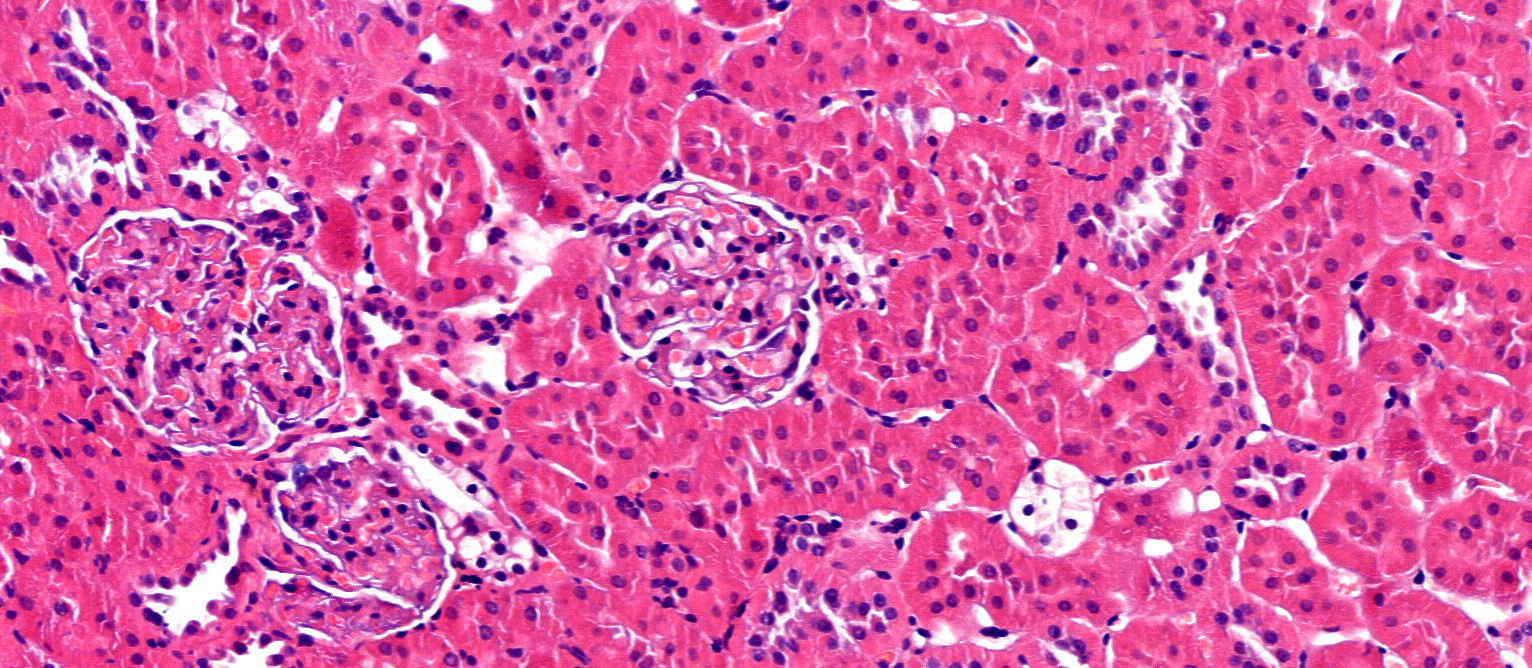

Supplement: Supplementary file 1 [file DataSheet3.ZIP › Fig 1D-HE-TSF-56/56-3.jpeg]

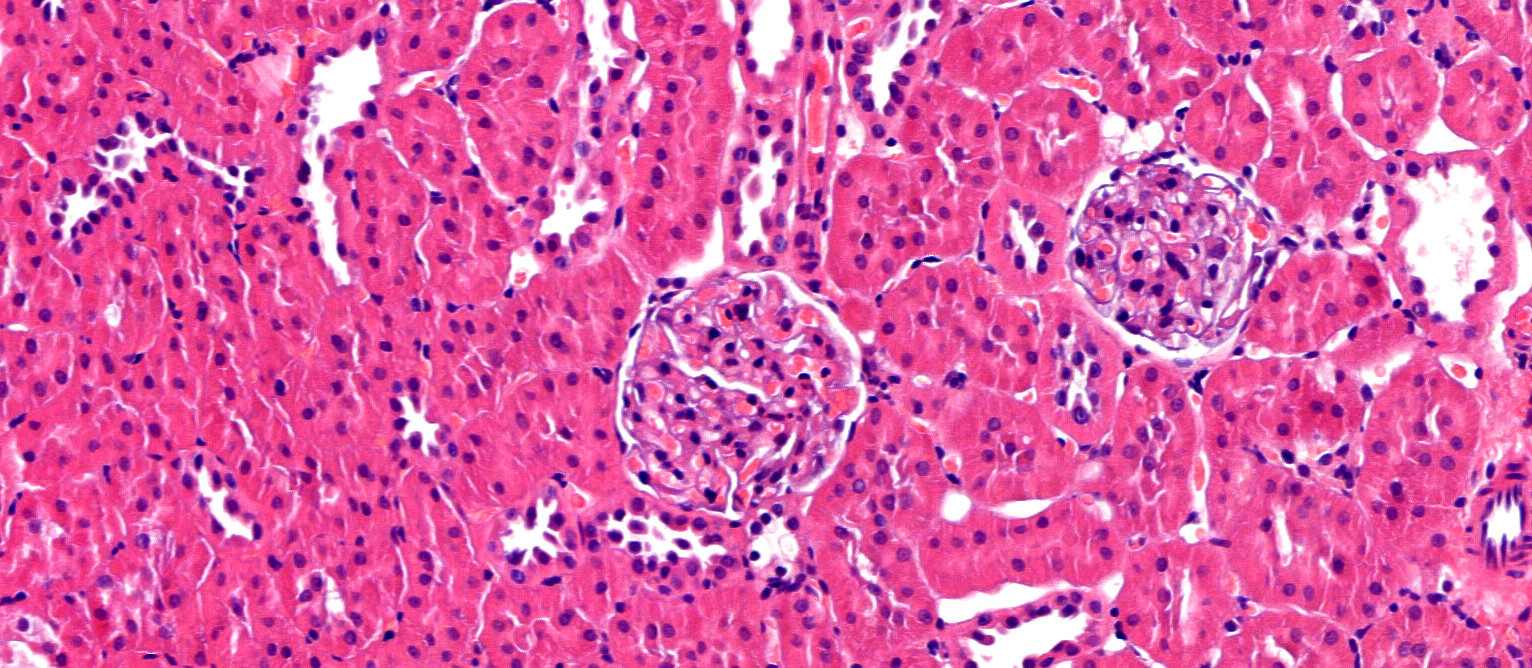

Supplement: Supplementary file 1 [file DataSheet3.ZIP › Fig 1D-HE-TSF-56/56-4.jpeg]

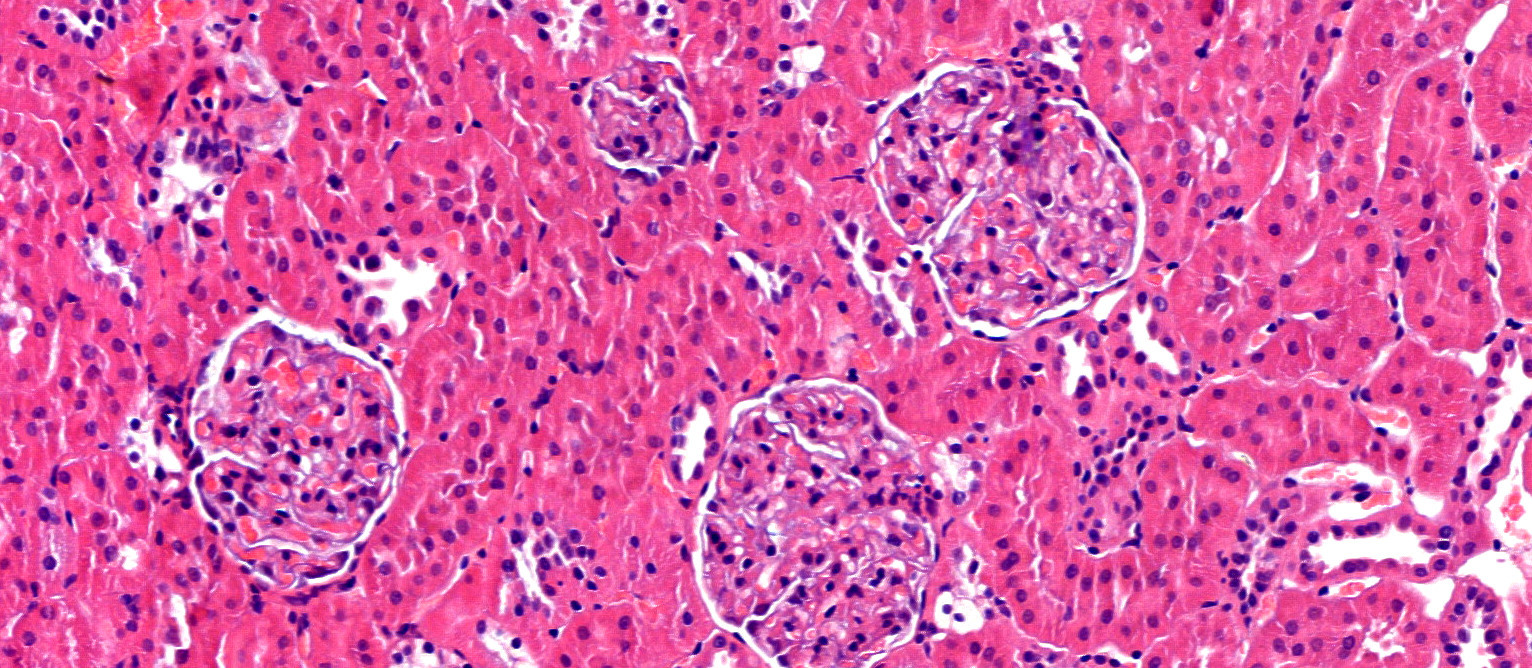

Supplement: Supplementary file 1 [file DataSheet3.ZIP › Fig 1D-HE-TSF-56/56-5.jpeg]

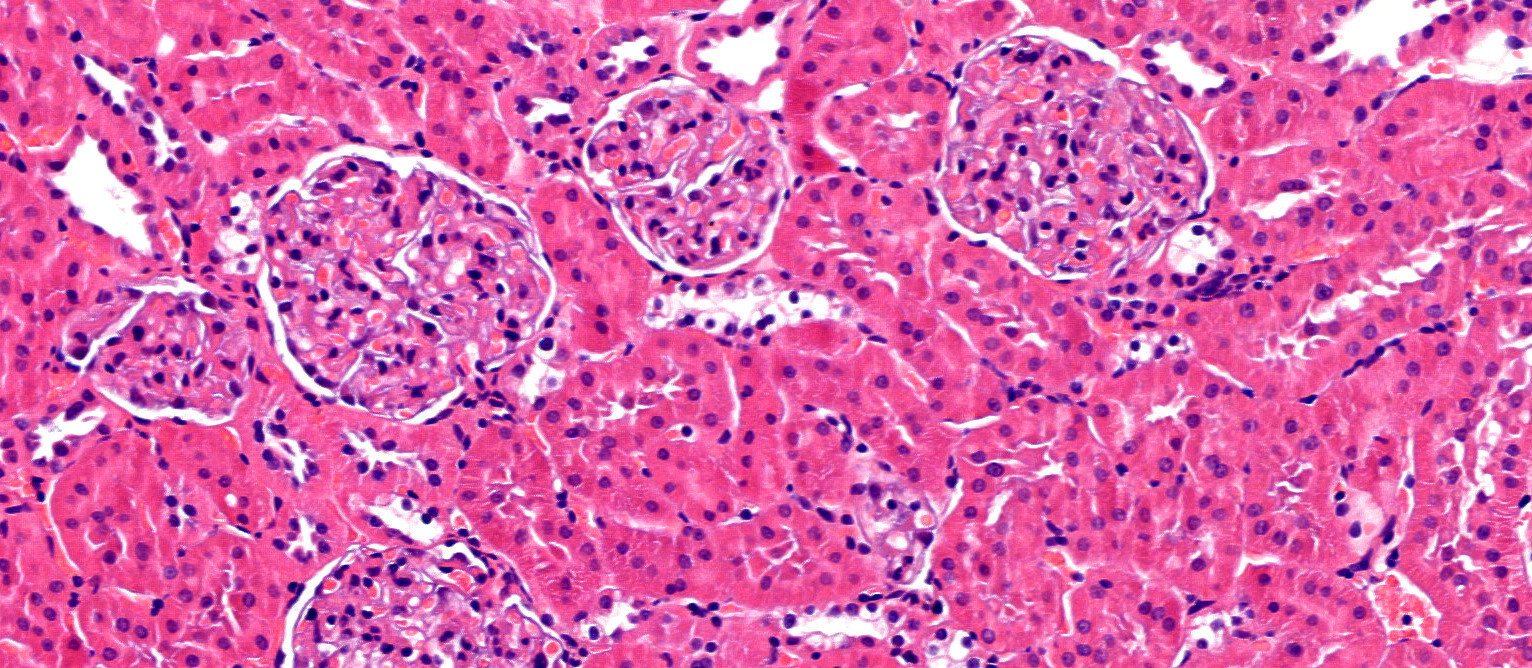

Supplement: Supplementary file 1 [file DataSheet3.ZIP › Fig 1D-HE-TSF-56/56-6.jpeg]

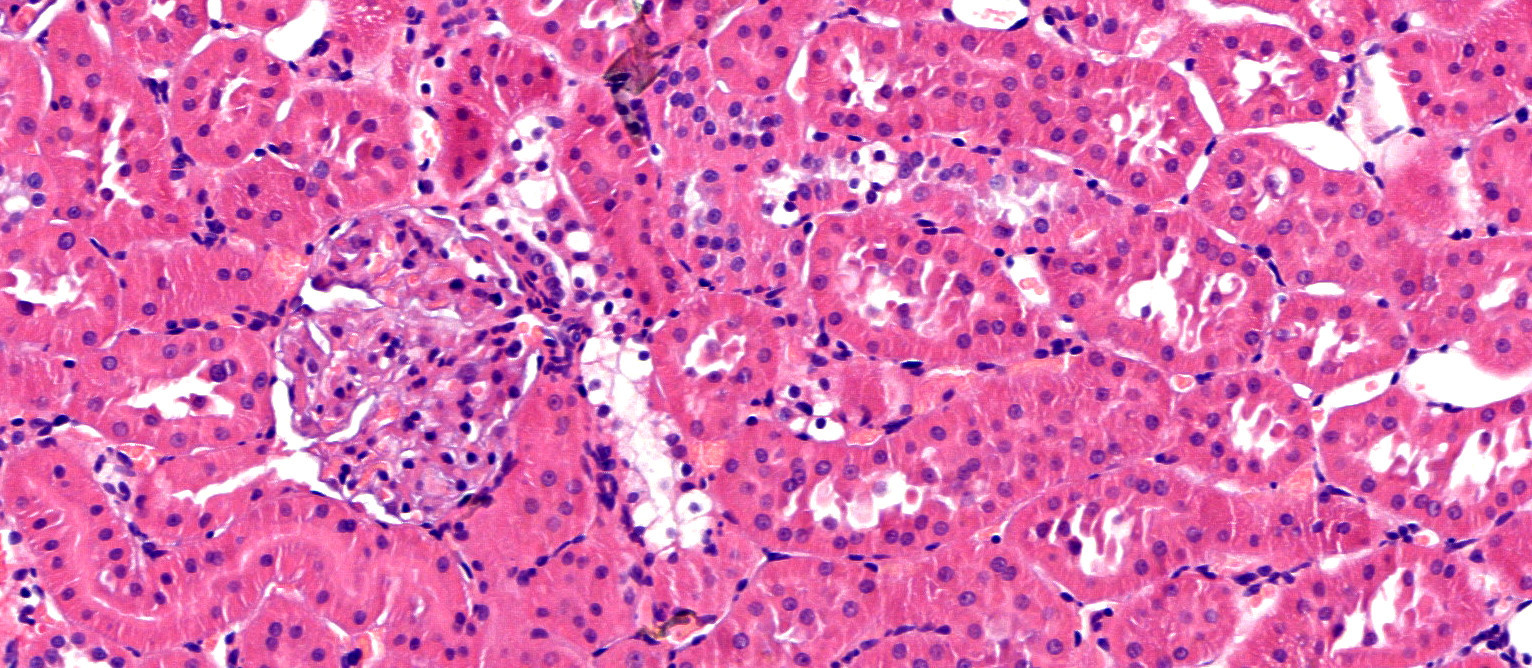

Supplement: Supplementary file 1 [file DataSheet3.ZIP › Fig 1D-HE-TSF-56/56-7.jpeg]

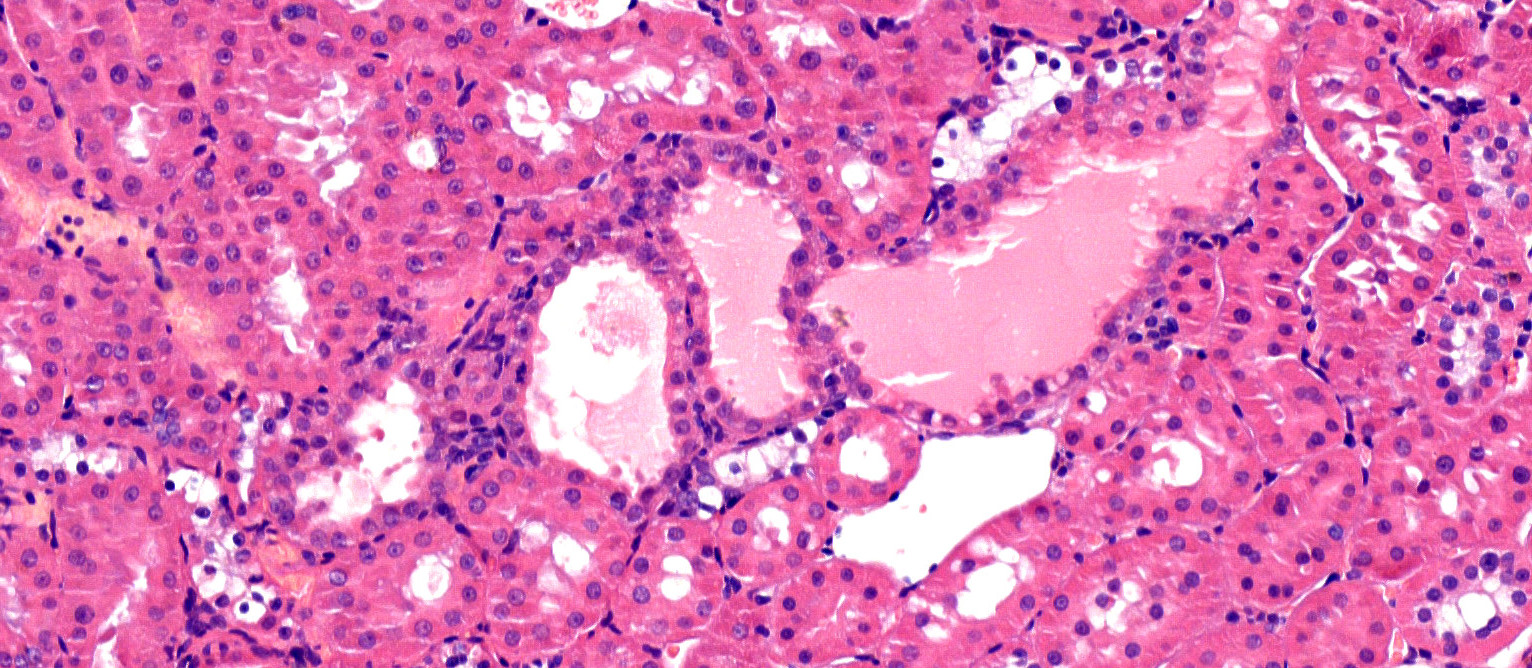

Supplement: Supplementary file 1 [file DataSheet3.ZIP › Fig 1D-HE-TSF-56/56-8.jpeg]

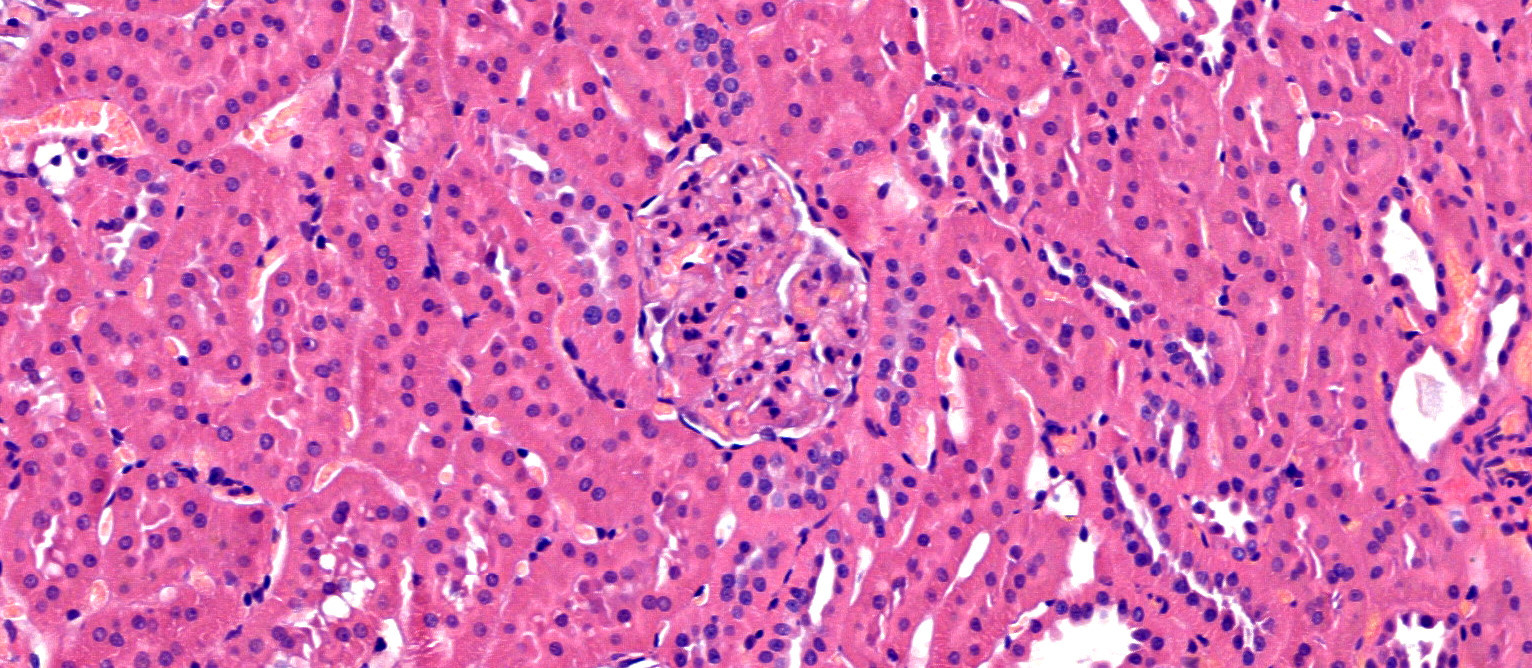

Supplement: Supplementary file 1 [file DataSheet3.ZIP › Fig 1D-HE-TSF-56/56-9.jpeg]

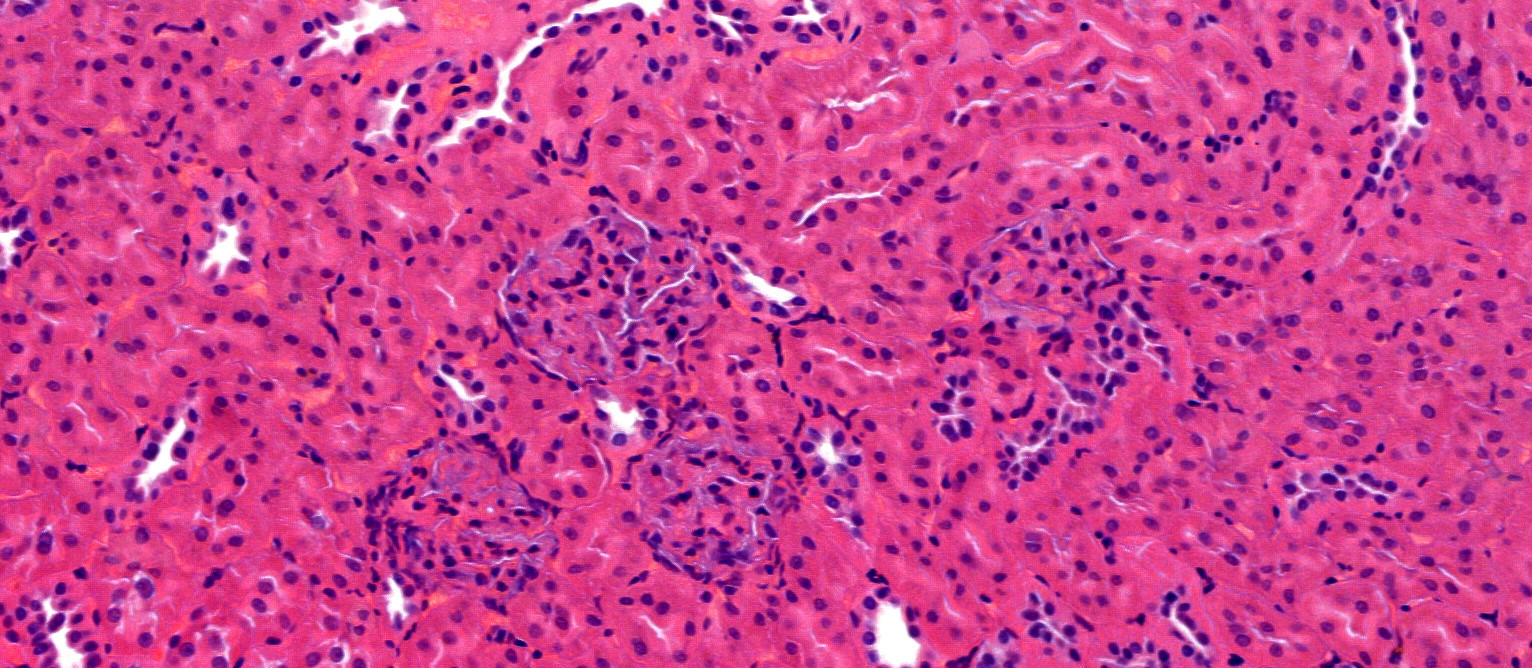

Supplement: Supplementary file 1 [file DataSheet3.ZIP › Fig 1D-HE-TSF-57/57-1.jpeg]

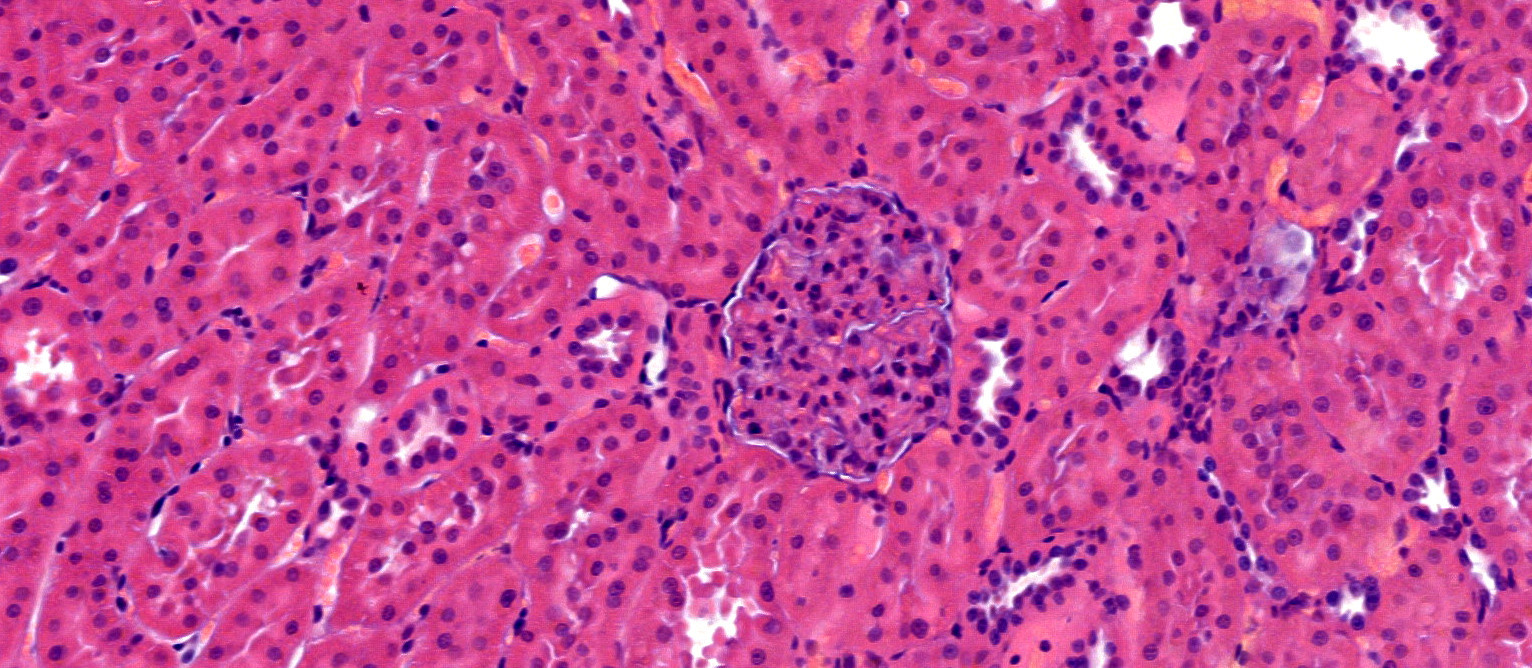

Supplement: Supplementary file 1 [file DataSheet3.ZIP › Fig 1D-HE-TSF-57/57-10.jpeg]

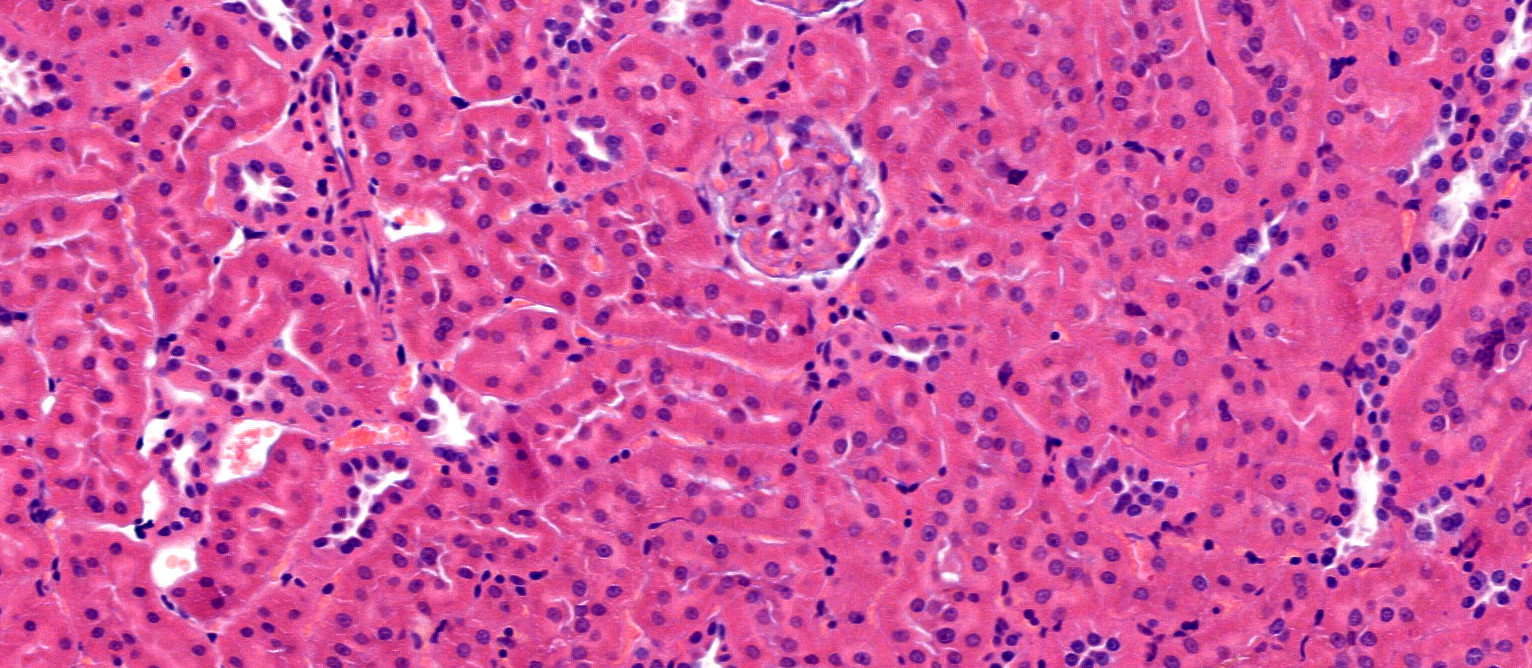

Supplement: Supplementary file 1 [file DataSheet3.ZIP › Fig 1D-HE-TSF-57/57-2.jpeg]

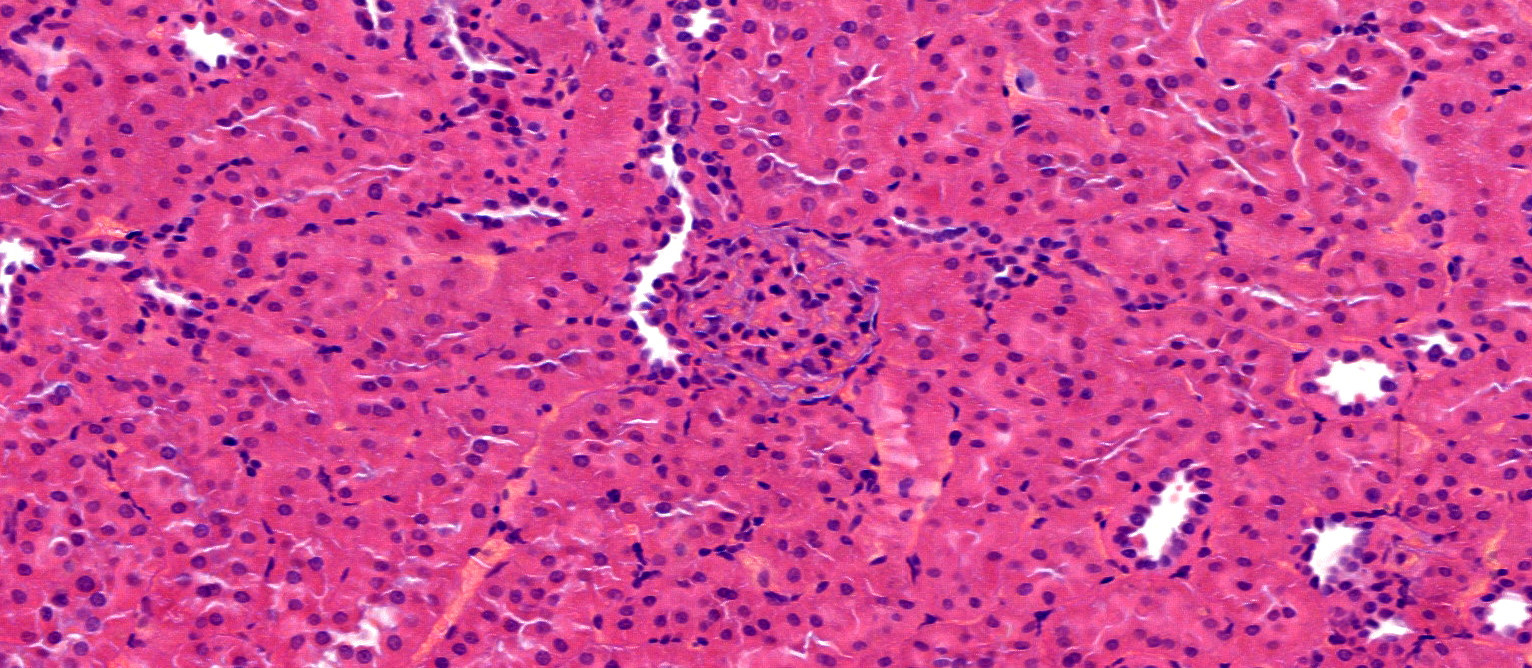

Supplement: Supplementary file 1 [file DataSheet3.ZIP › Fig 1D-HE-TSF-57/57-3.jpeg]

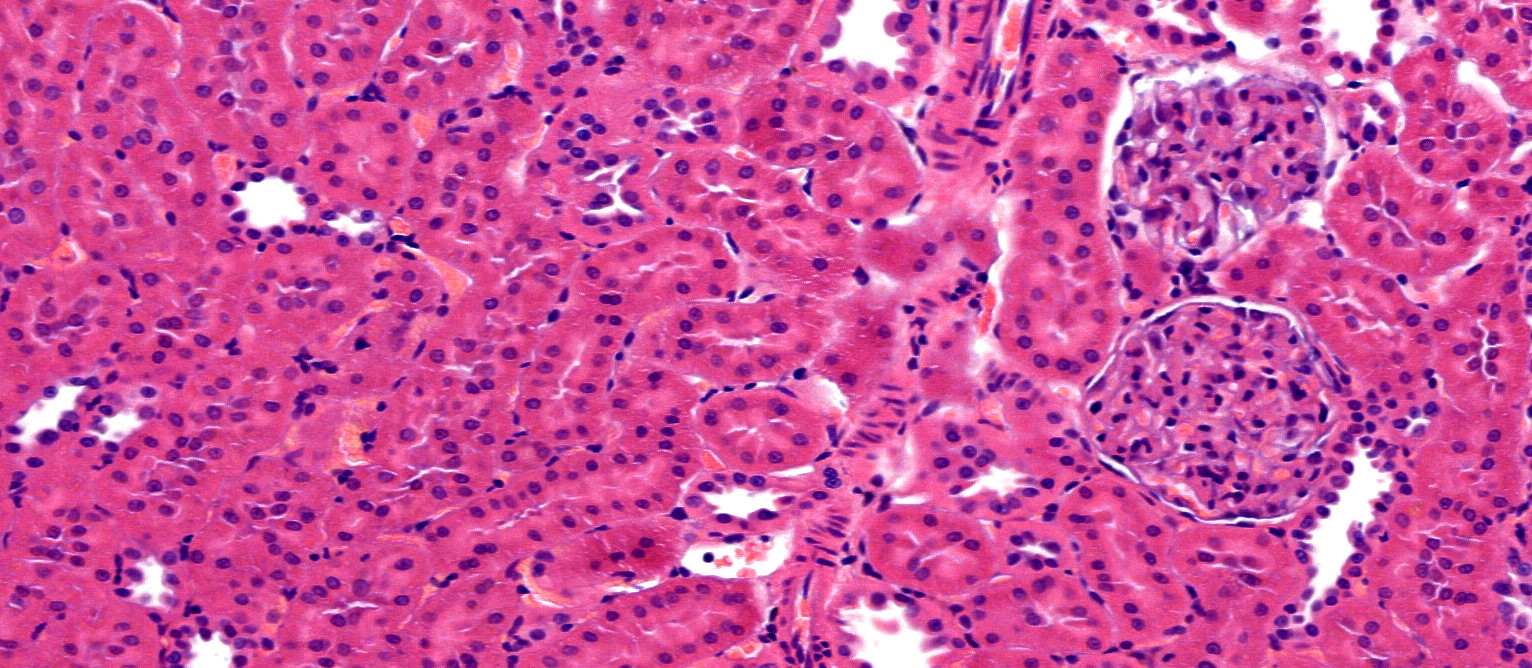

Supplement: Supplementary file 1 [file DataSheet3.ZIP › Fig 1D-HE-TSF-57/57-4.jpeg]

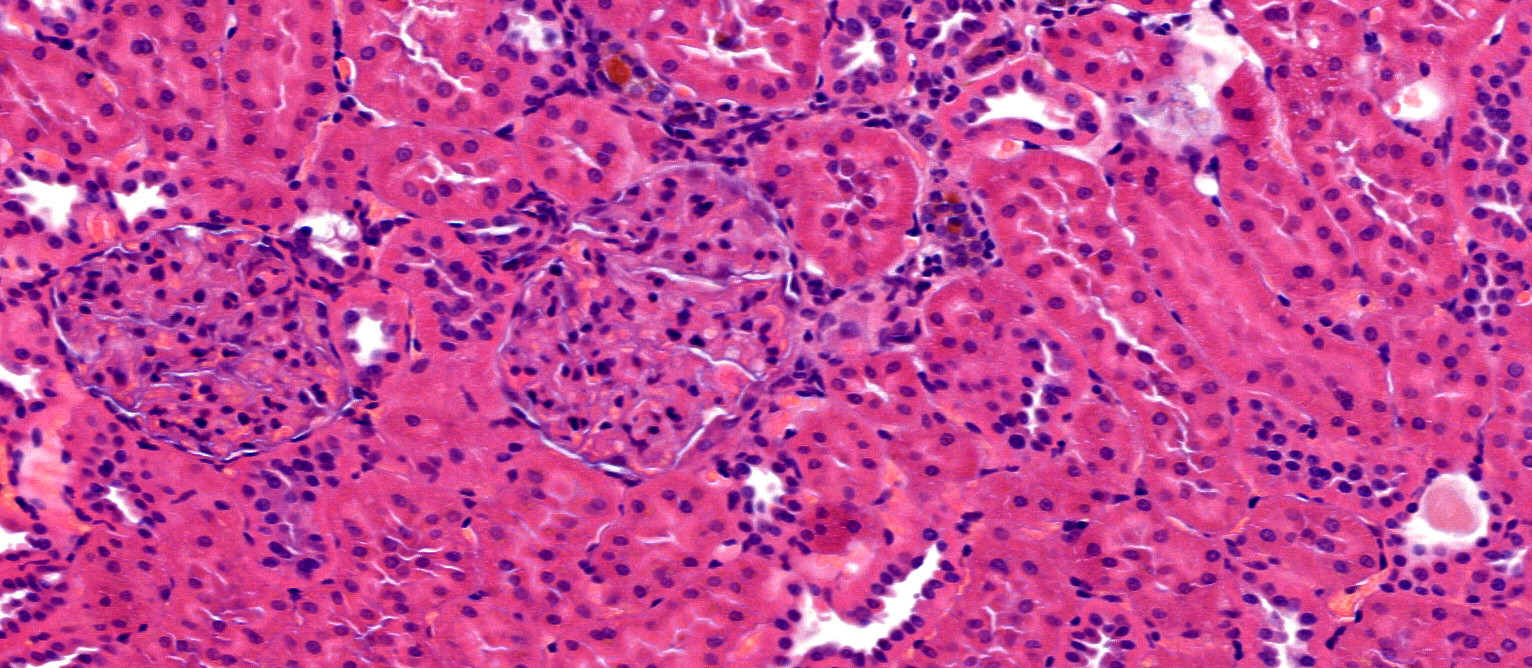

Supplement: Supplementary file 1 [file DataSheet3.ZIP › Fig 1D-HE-TSF-57/57-5.jpeg]

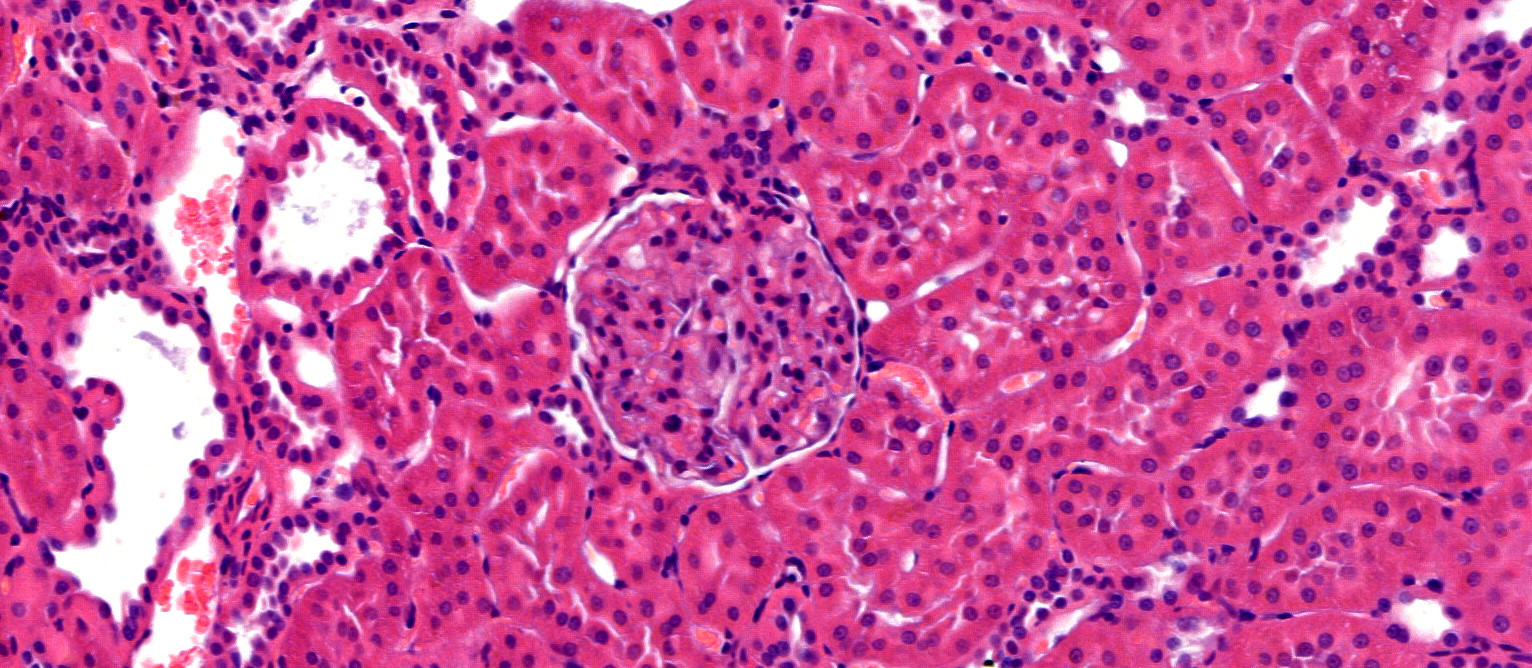

Supplement: Supplementary file 1 [file DataSheet3.ZIP › Fig 1D-HE-TSF-57/57-6.jpeg]

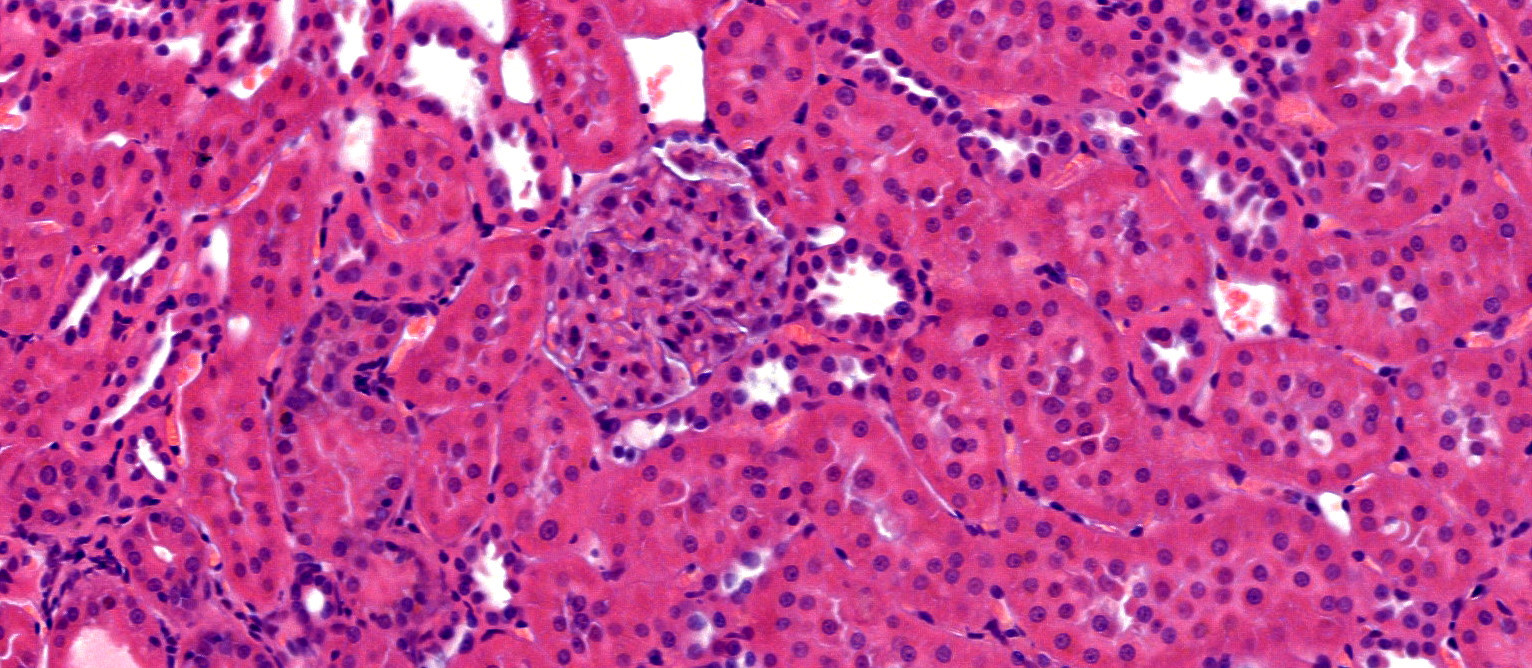

Supplement: Supplementary file 1 [file DataSheet3.ZIP › Fig 1D-HE-TSF-57/57-7.jpeg]

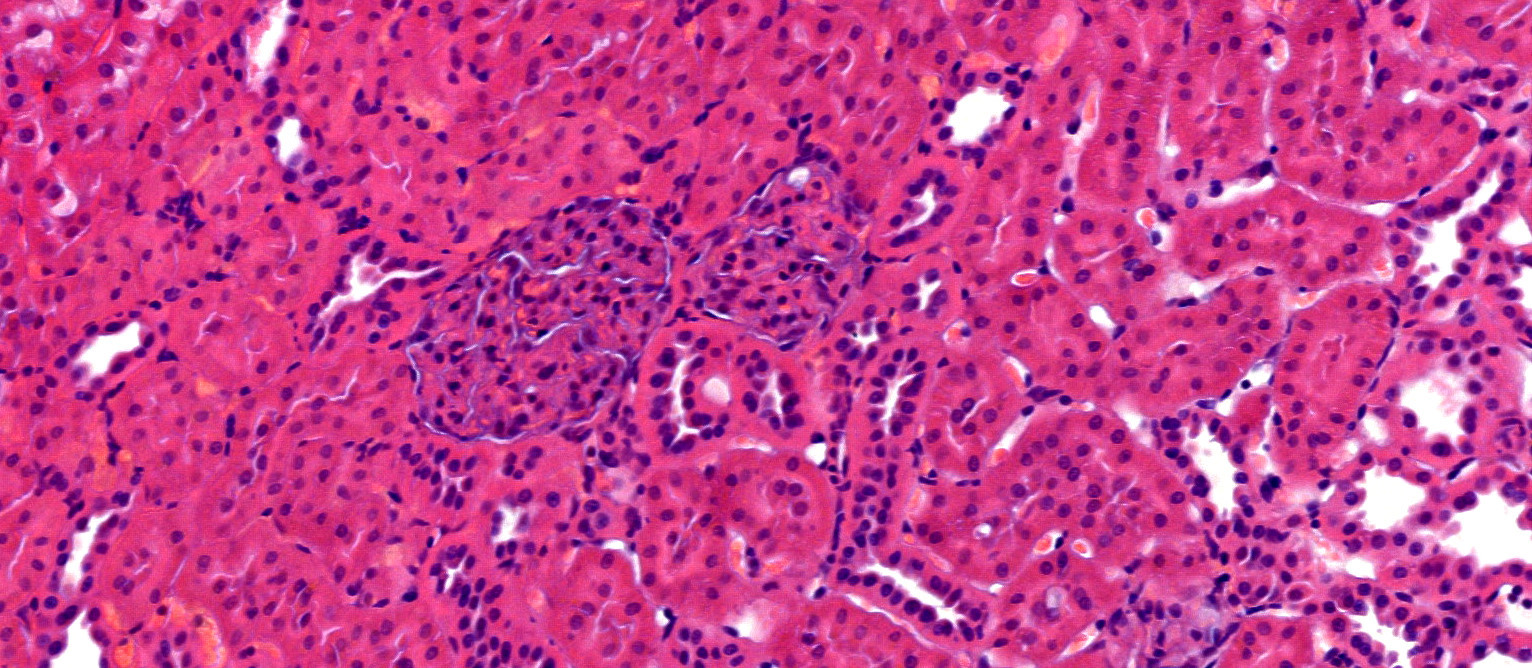

Supplement: Supplementary file 1 [file DataSheet3.ZIP › Fig 1D-HE-TSF-57/57-8.jpeg]

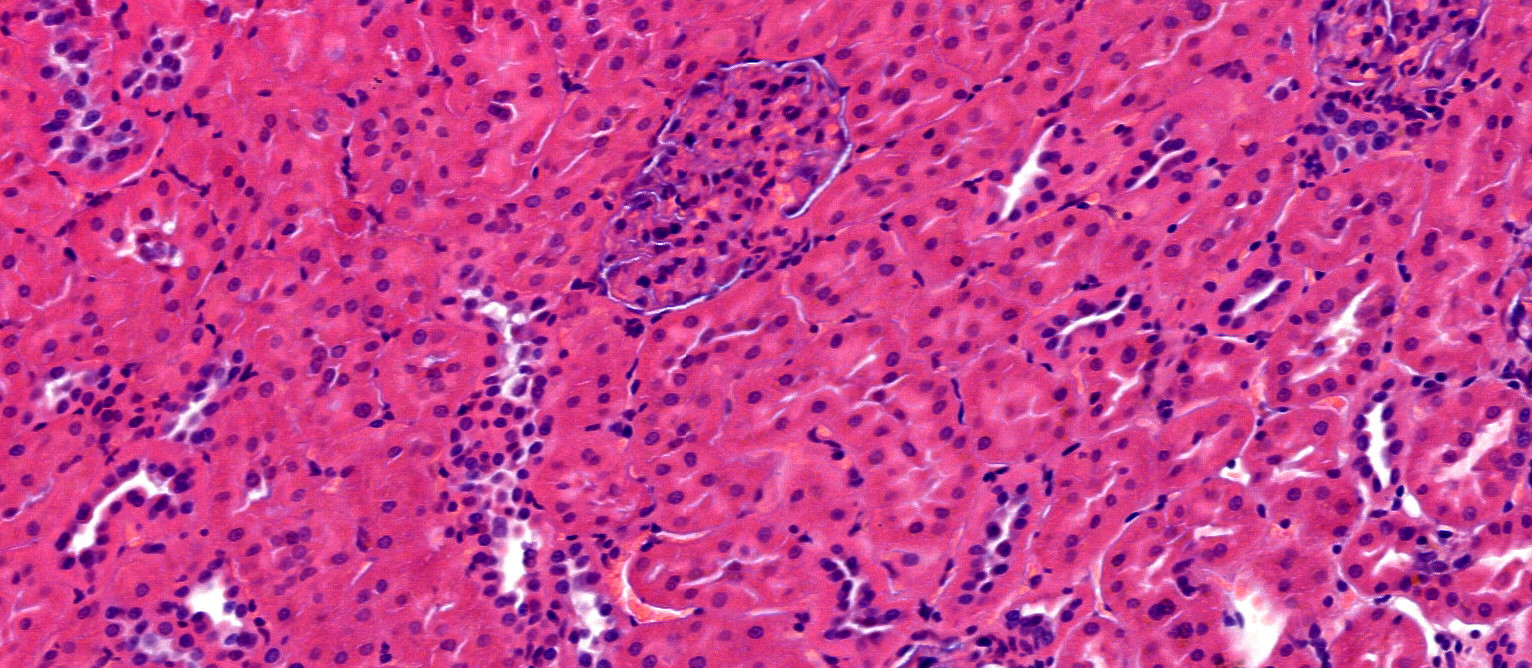

Supplement: Supplementary file 1 [file DataSheet3.ZIP › Fig 1D-HE-TSF-57/57-9.jpeg]

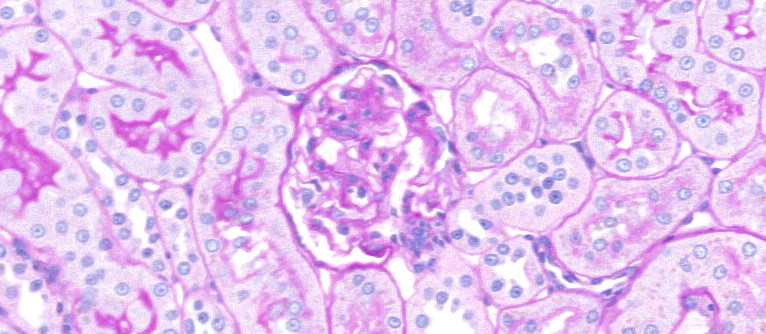

Supplement: Supplementary file 2 [file DataSheet14.ZIP › sham/Fig 1D-PAS-sham-1/1-1.jpeg]

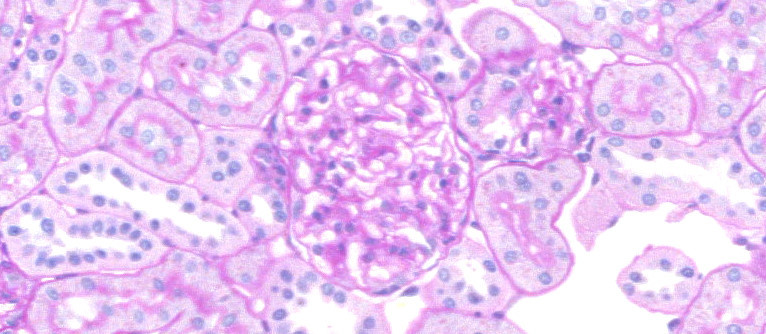

Supplement: Supplementary file 2 [file DataSheet14.ZIP › sham/Fig 1D-PAS-sham-1/1-10.jpeg]

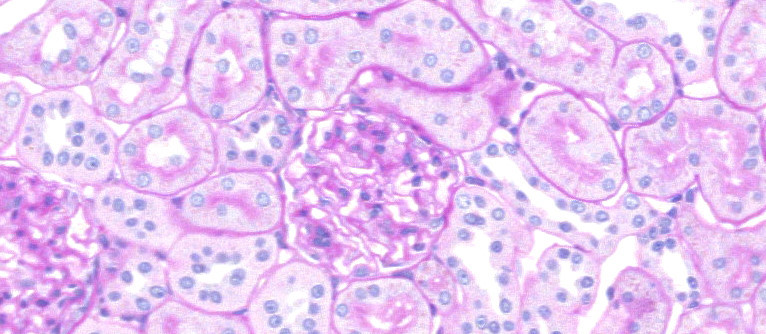

Supplement: Supplementary file 2 [file DataSheet14.ZIP › sham/Fig 1D-PAS-sham-1/1-11.jpeg]

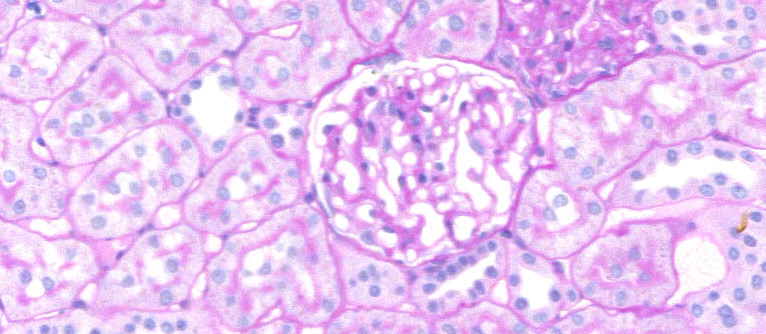

Supplement: Supplementary file 2 [file DataSheet14.ZIP › sham/Fig 1D-PAS-sham-1/1-12.jpeg]

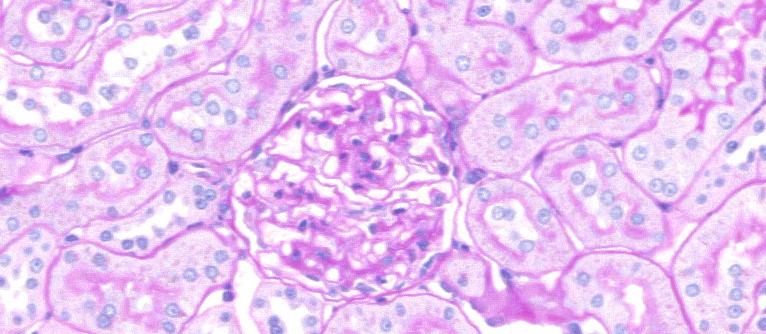

Supplement: Supplementary file 2 [file DataSheet14.ZIP › sham/Fig 1D-PAS-sham-1/1-13.jpeg]

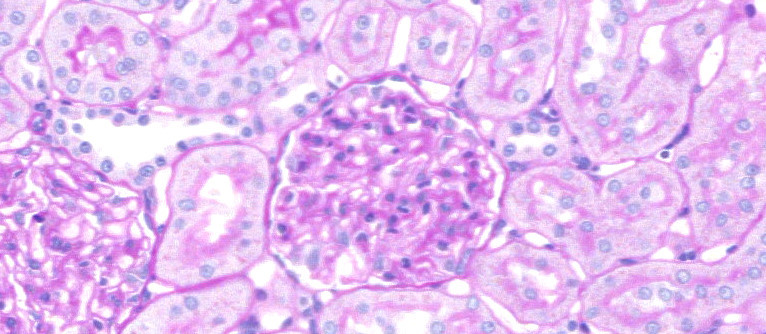

Supplement: Supplementary file 2 [file DataSheet14.ZIP › sham/Fig 1D-PAS-sham-1/1-14.jpeg]

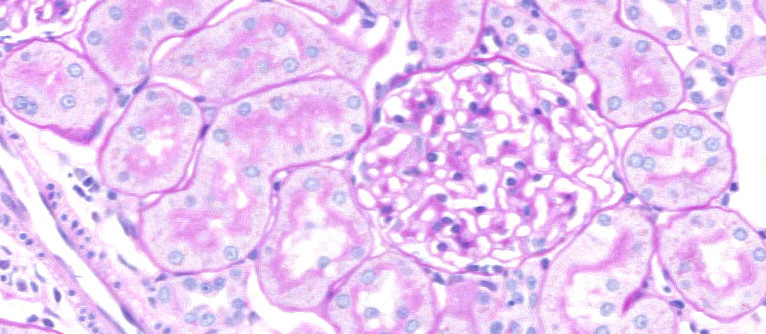

Supplement: Supplementary file 2 [file DataSheet14.ZIP › sham/Fig 1D-PAS-sham-1/1-15.jpeg]

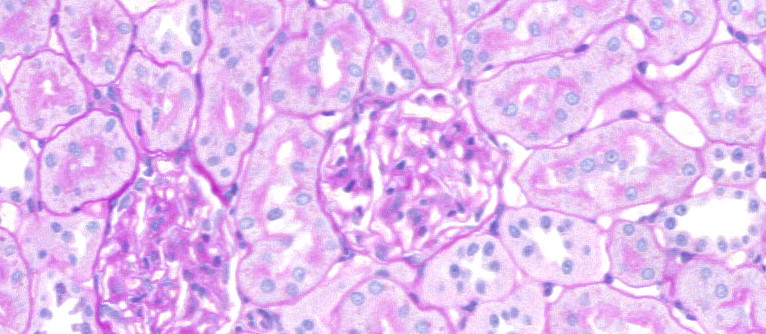

Supplement: Supplementary file 2 [file DataSheet14.ZIP › sham/Fig 1D-PAS-sham-1/1-16.jpeg]

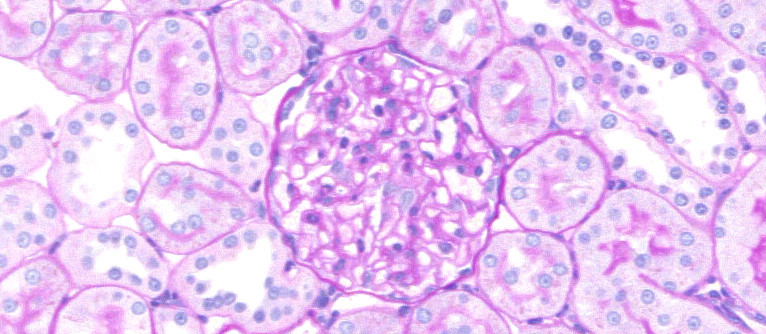

Supplement: Supplementary file 2 [file DataSheet14.ZIP › sham/Fig 1D-PAS-sham-1/1-17.jpeg]

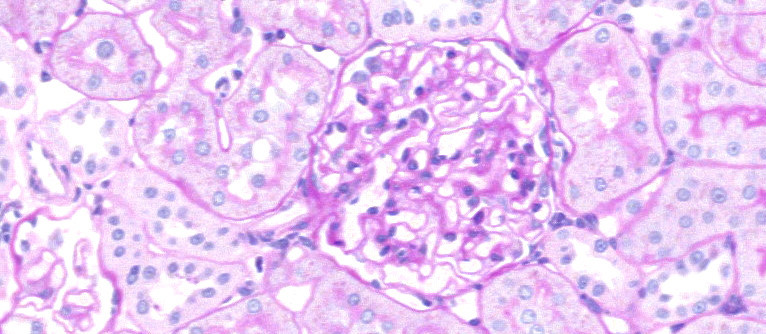

Supplement: Supplementary file 2 [file DataSheet14.ZIP › sham/Fig 1D-PAS-sham-1/1-18.jpeg]

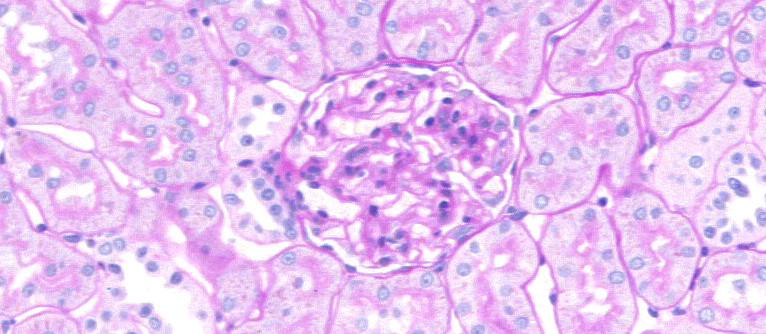

Supplement: Supplementary file 2 [file DataSheet14.ZIP › sham/Fig 1D-PAS-sham-1/1-19.jpeg]

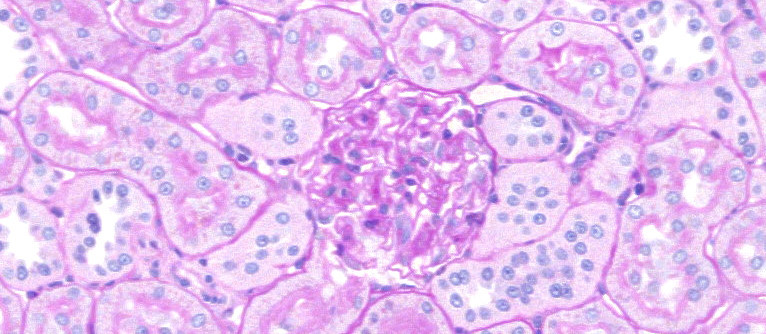

Supplement: Supplementary file 2 [file DataSheet14.ZIP › sham/Fig 1D-PAS-sham-1/1-2.jpeg]

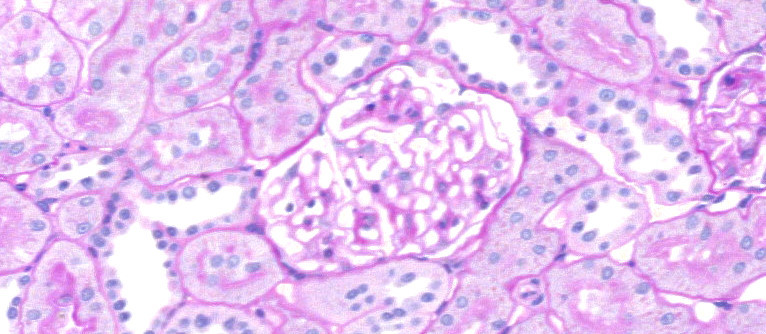

Supplement: Supplementary file 2 [file DataSheet14.ZIP › sham/Fig 1D-PAS-sham-1/1-20.jpeg]

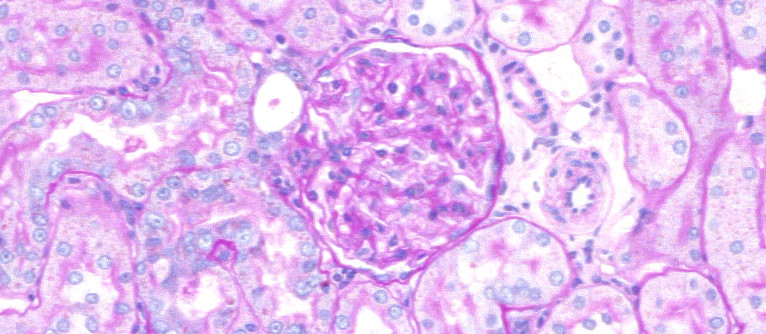

Supplement: Supplementary file 2 [file DataSheet14.ZIP › sham/Fig 1D-PAS-sham-1/1-3.jpeg]

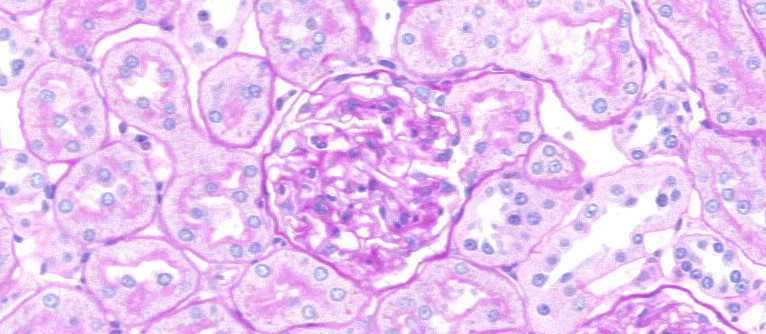

Supplement: Supplementary file 2 [file DataSheet14.ZIP › sham/Fig 1D-PAS-sham-1/1-4.jpeg]

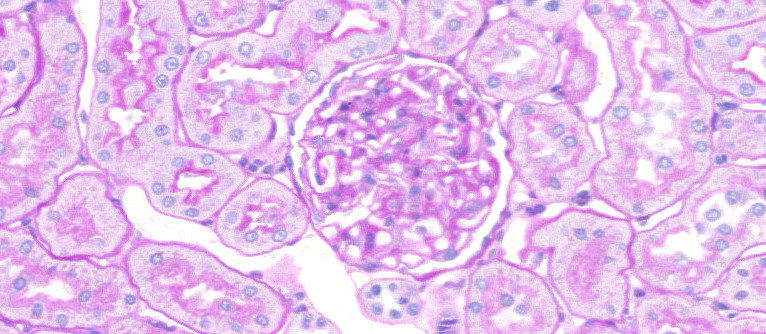

Supplement: Supplementary file 2 [file DataSheet14.ZIP › sham/Fig 1D-PAS-sham-1/1-5.jpeg]

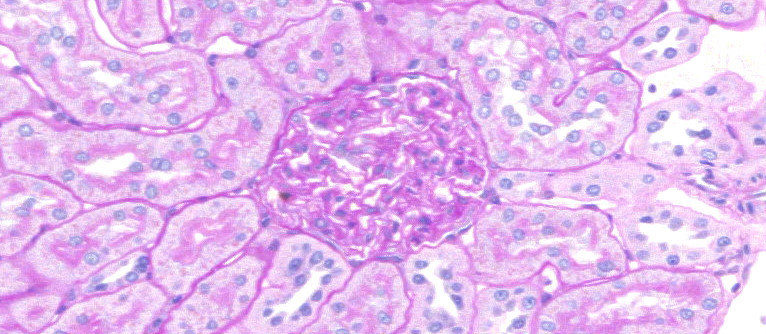

Supplement: Supplementary file 2 [file DataSheet14.ZIP › sham/Fig 1D-PAS-sham-1/1-6.jpeg]

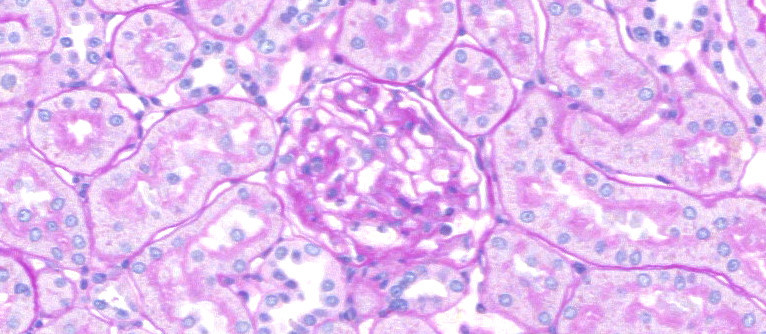

Supplement: Supplementary file 2 [file DataSheet14.ZIP › sham/Fig 1D-PAS-sham-1/1-7.jpeg]

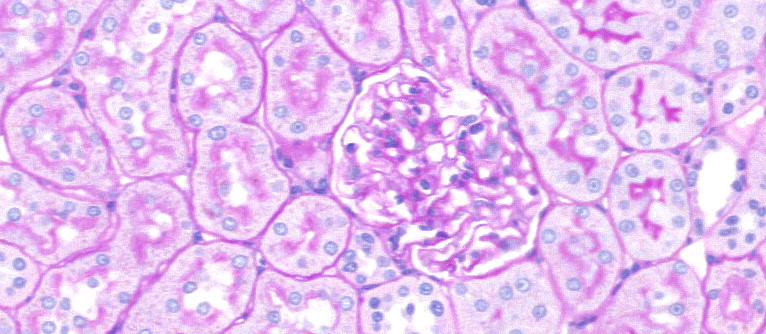

Supplement: Supplementary file 2 [file DataSheet14.ZIP › sham/Fig 1D-PAS-sham-1/1-8.jpeg]

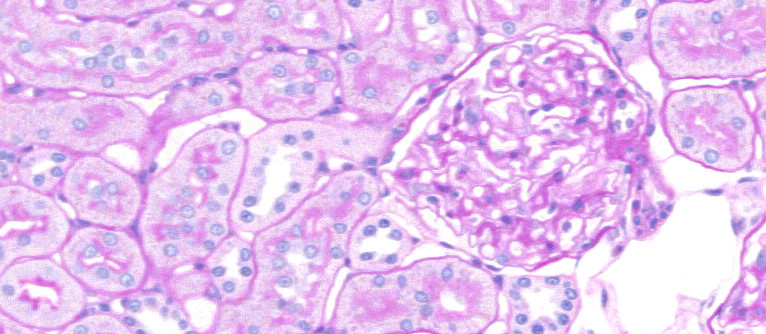

Supplement: Supplementary file 2 [file DataSheet14.ZIP › sham/Fig 1D-PAS-sham-1/1-9.jpeg]

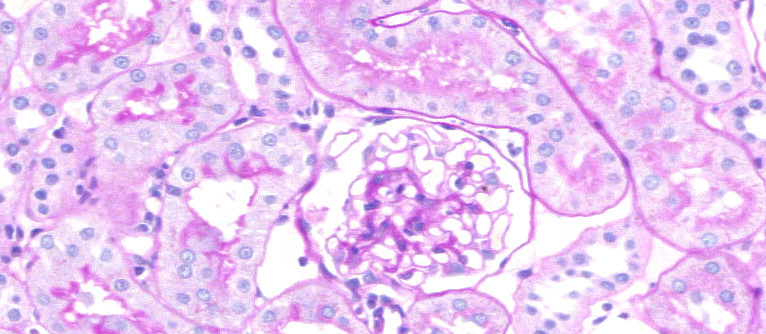

Supplement: Supplementary file 2 [file DataSheet14.ZIP › sham/Fig 1D-PAS-sham-10/10-1.jpeg]

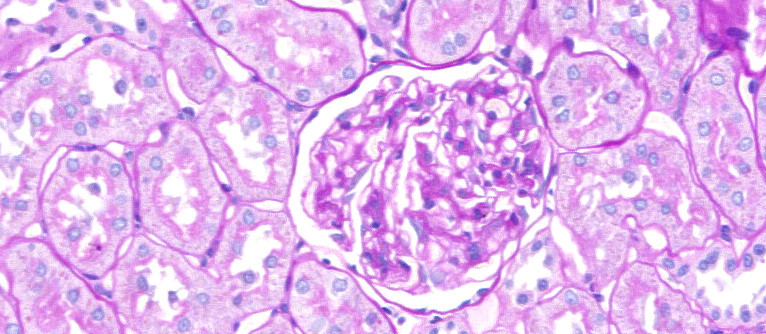

Supplement: Supplementary file 2 [file DataSheet14.ZIP › sham/Fig 1D-PAS-sham-10/10-10.jpeg]

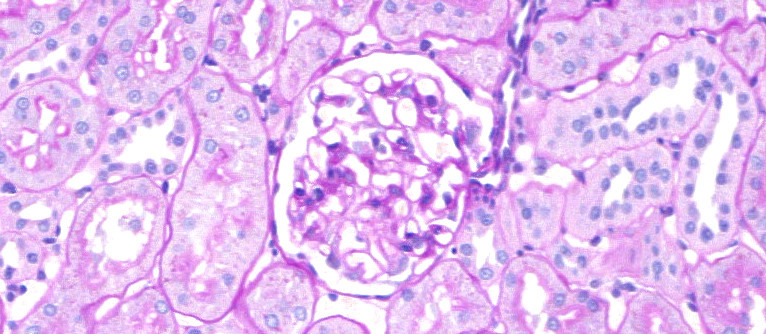

Supplement: Supplementary file 2 [file DataSheet14.ZIP › sham/Fig 1D-PAS-sham-10/10-11.jpeg]

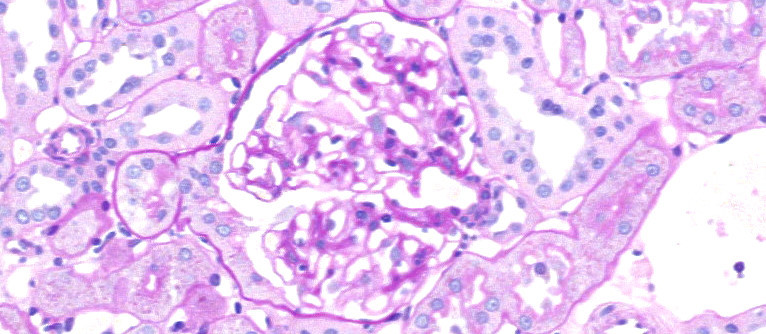

Supplement: Supplementary file 2 [file DataSheet14.ZIP › sham/Fig 1D-PAS-sham-10/10-12.jpeg]

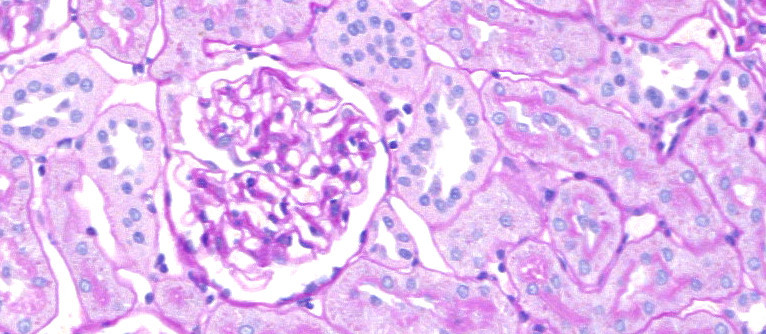

Supplement: Supplementary file 2 [file DataSheet14.ZIP › sham/Fig 1D-PAS-sham-10/10-13.jpeg]

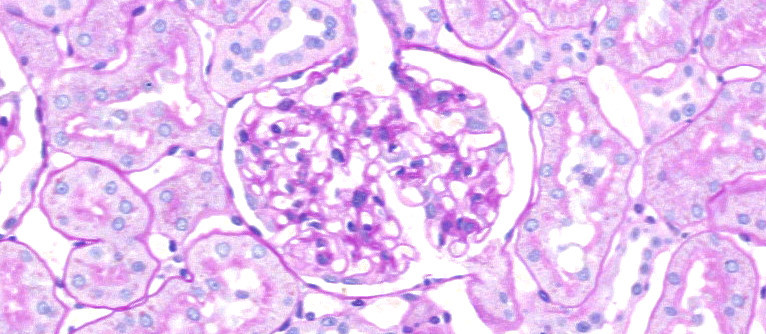

Supplement: Supplementary file 2 [file DataSheet14.ZIP › sham/Fig 1D-PAS-sham-10/10-14.jpeg]

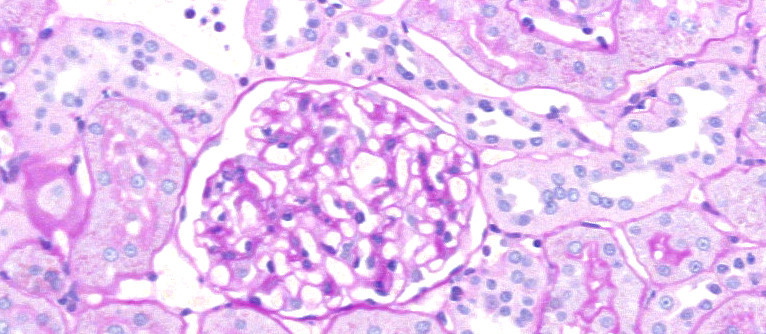

Supplement: Supplementary file 2 [file DataSheet14.ZIP › sham/Fig 1D-PAS-sham-10/10-15.jpeg]

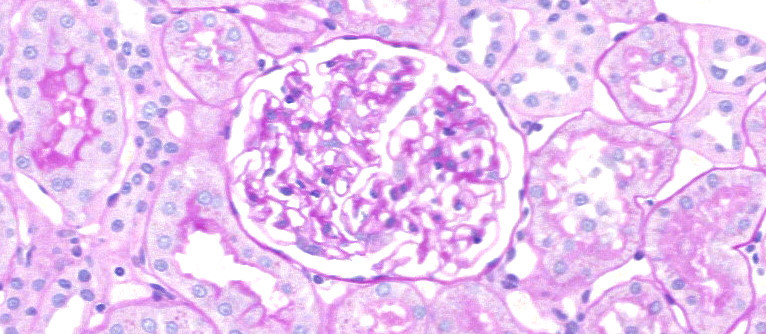

Supplement: Supplementary file 2 [file DataSheet14.ZIP › sham/Fig 1D-PAS-sham-10/10-16.jpeg]

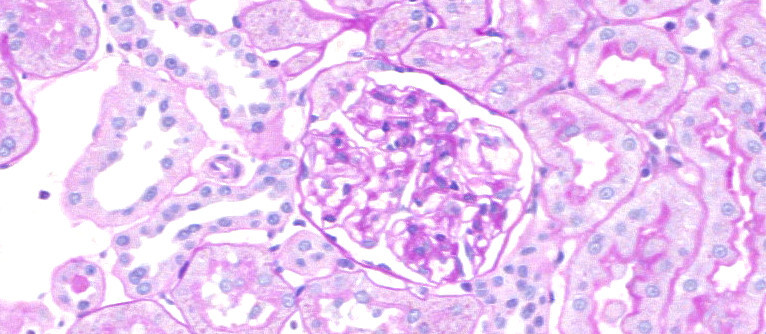

Supplement: Supplementary file 2 [file DataSheet14.ZIP › sham/Fig 1D-PAS-sham-10/10-17.jpeg]

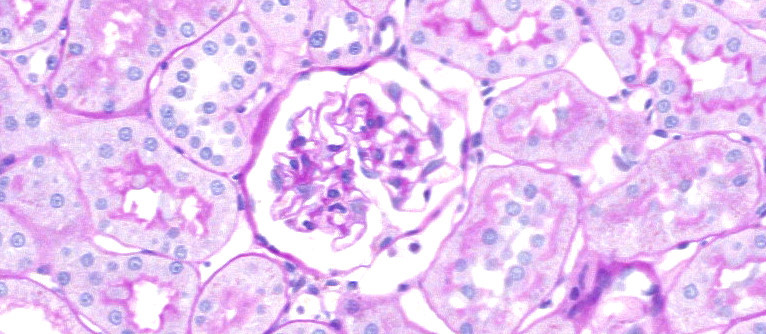

Supplement: Supplementary file 2 [file DataSheet14.ZIP › sham/Fig 1D-PAS-sham-10/10-18.jpeg]

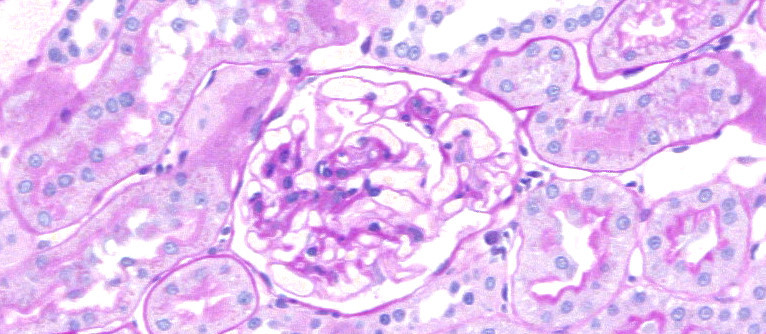

Supplement: Supplementary file 2 [file DataSheet14.ZIP › sham/Fig 1D-PAS-sham-10/10-19.jpeg]

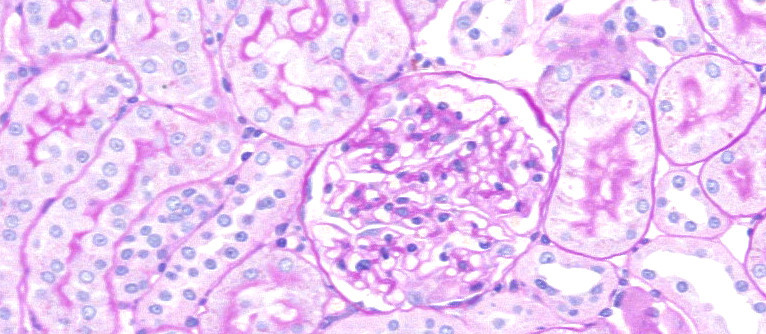

Supplement: Supplementary file 2 [file DataSheet14.ZIP › sham/Fig 1D-PAS-sham-10/10-2.jpeg]

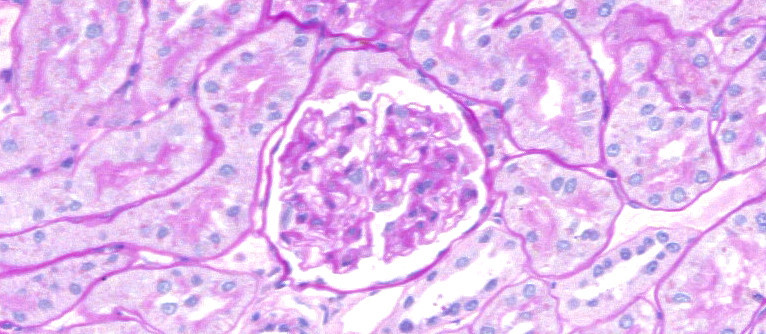

Supplement: Supplementary file 2 [file DataSheet14.ZIP › sham/Fig 1D-PAS-sham-10/10-20.jpeg]

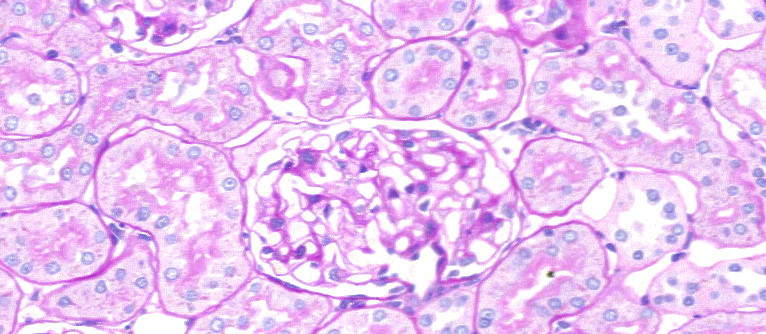

Supplement: Supplementary file 2 [file DataSheet14.ZIP › sham/Fig 1D-PAS-sham-10/10-3.jpeg]

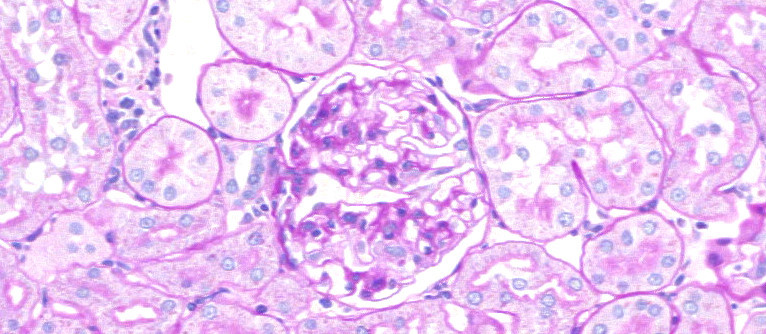

Supplement: Supplementary file 2 [file DataSheet14.ZIP › sham/Fig 1D-PAS-sham-10/10-4.jpeg]

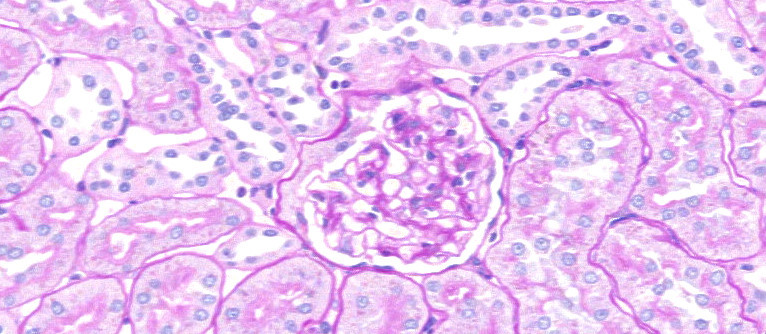

Supplement: Supplementary file 2 [file DataSheet14.ZIP › sham/Fig 1D-PAS-sham-10/10-5.jpeg]

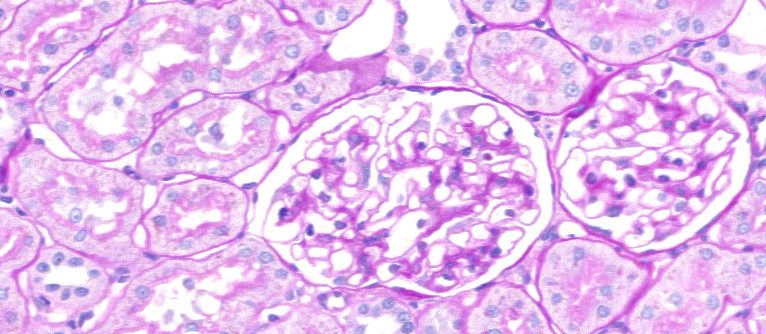

Supplement: Supplementary file 2 [file DataSheet14.ZIP › sham/Fig 1D-PAS-sham-10/10-6.jpeg]

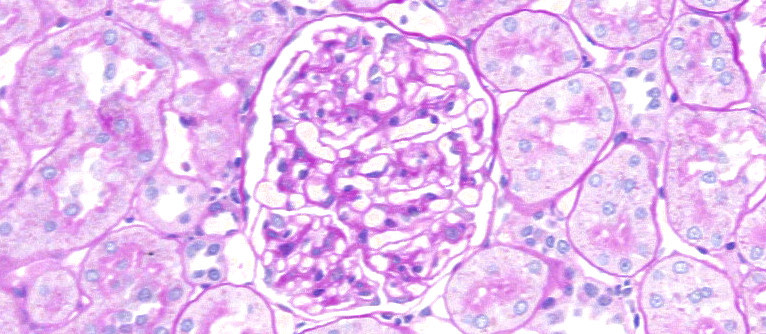

Supplement: Supplementary file 2 [file DataSheet14.ZIP › sham/Fig 1D-PAS-sham-10/10-7.jpeg]

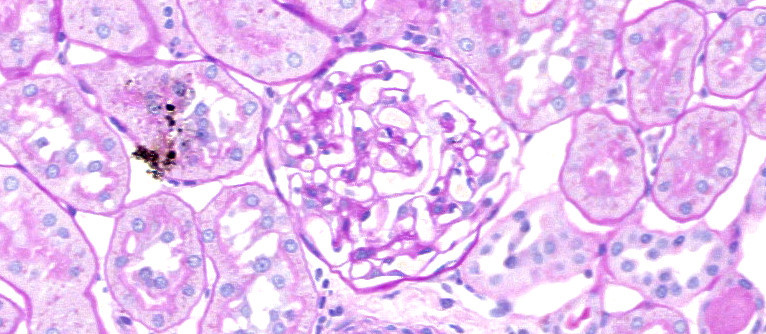

Supplement: Supplementary file 2 [file DataSheet14.ZIP › sham/Fig 1D-PAS-sham-10/10-8.jpeg]

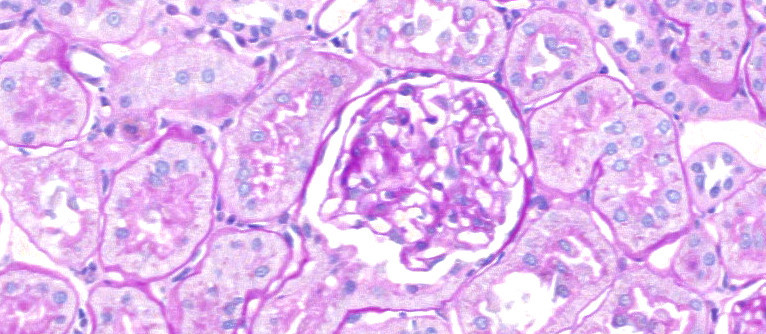

Supplement: Supplementary file 2 [file DataSheet14.ZIP › sham/Fig 1D-PAS-sham-10/10-9.jpeg]

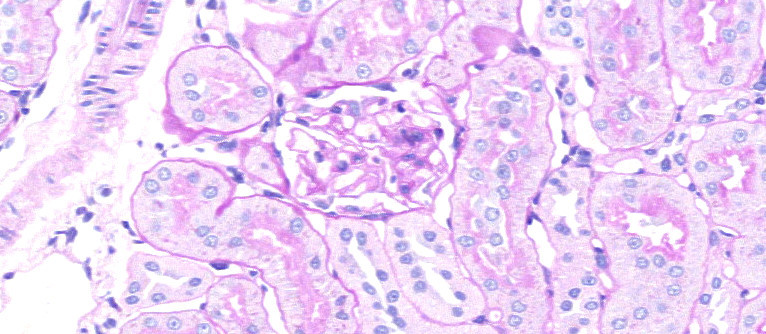

Supplement: Supplementary file 2 [file DataSheet14.ZIP › sham/Fig 1D-PAS-sham-2/2-1.jpeg]

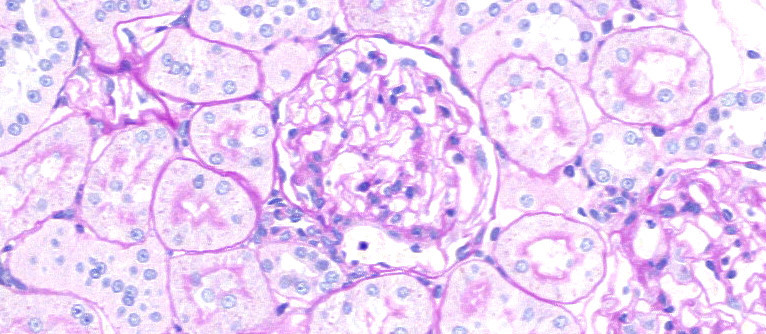

Supplement: Supplementary file 2 [file DataSheet14.ZIP › sham/Fig 1D-PAS-sham-2/2-10.jpeg]
