# Supplementary figures and images for: Characteristics of Serum Metabolites and Gut Microbiota in Diabetic Kidney Disease (part 3 of 13)
Source: Front Pharmacol. 2022 Apr 14;13:872988. doi: 10.3389/fphar.2022.872988 (PMC9084235; doi:10.3389/fphar.2022.872988)

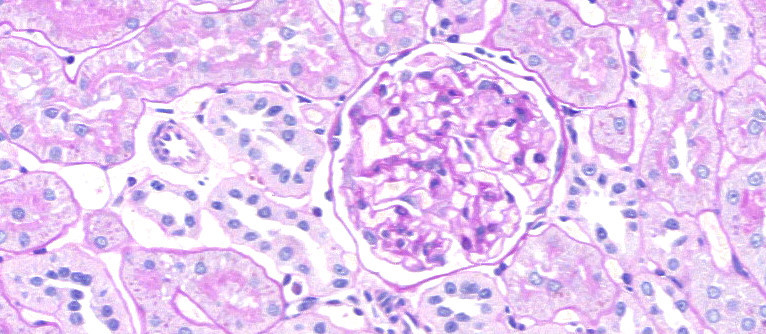

Supplement: Supplementary file 2 [file DataSheet14.ZIP › sham/Fig 1D-PAS-sham-7/7-11.jpeg]

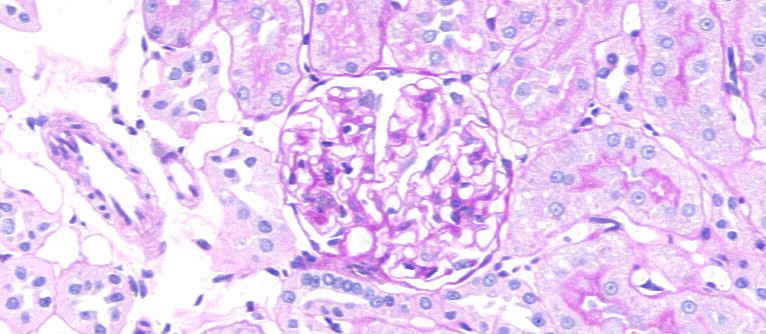

Supplement: Supplementary file 2 [file DataSheet14.ZIP › sham/Fig 1D-PAS-sham-7/7-12.jpeg]

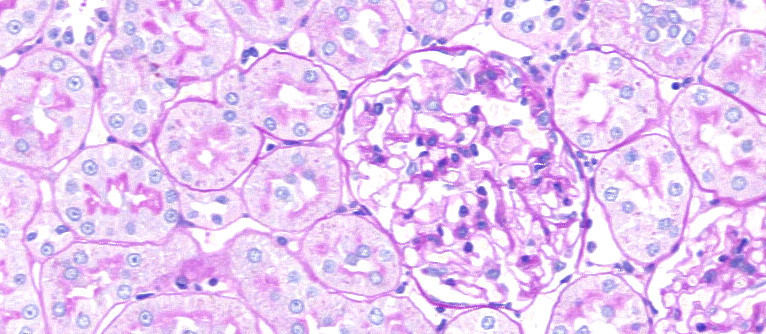

Supplement: Supplementary file 2 [file DataSheet14.ZIP › sham/Fig 1D-PAS-sham-7/7-13.jpeg]

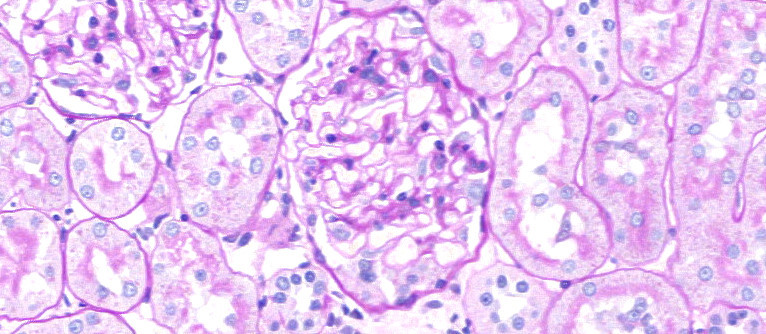

Supplement: Supplementary file 2 [file DataSheet14.ZIP › sham/Fig 1D-PAS-sham-7/7-14.jpeg]

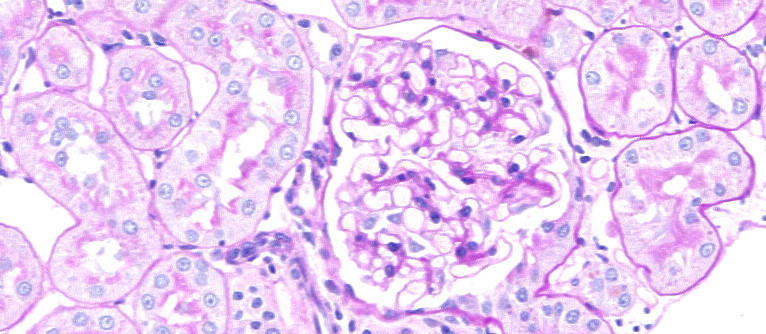

Supplement: Supplementary file 2 [file DataSheet14.ZIP › sham/Fig 1D-PAS-sham-7/7-15.jpeg]

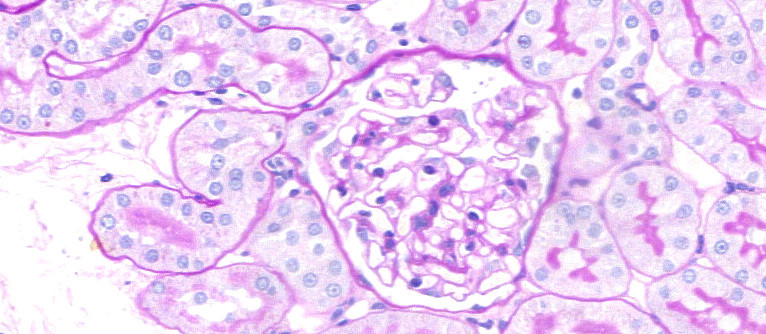

Supplement: Supplementary file 2 [file DataSheet14.ZIP › sham/Fig 1D-PAS-sham-7/7-16.jpeg]

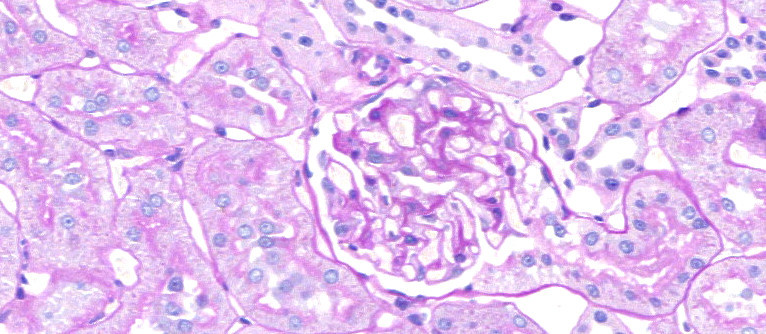

Supplement: Supplementary file 2 [file DataSheet14.ZIP › sham/Fig 1D-PAS-sham-7/7-17.jpeg]

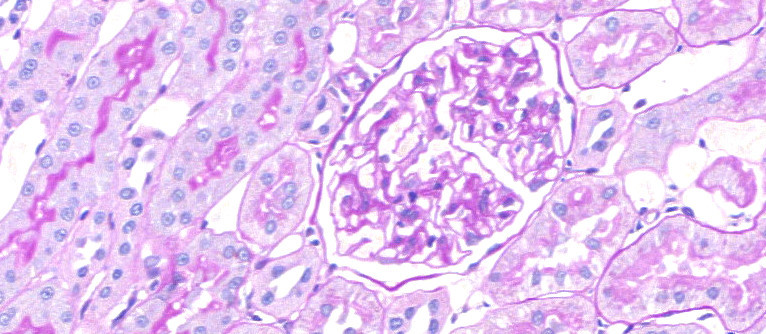

Supplement: Supplementary file 2 [file DataSheet14.ZIP › sham/Fig 1D-PAS-sham-7/7-18.jpeg]

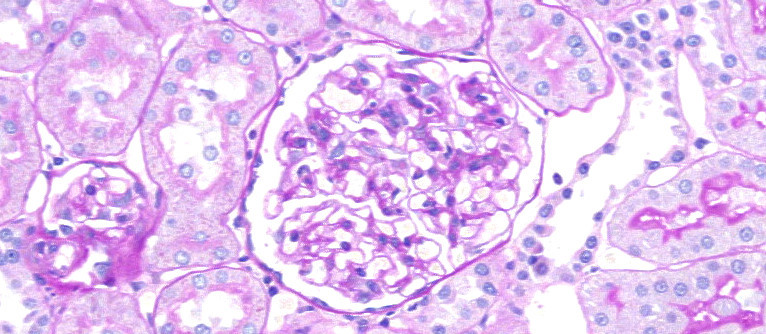

Supplement: Supplementary file 2 [file DataSheet14.ZIP › sham/Fig 1D-PAS-sham-7/7-19.jpeg]

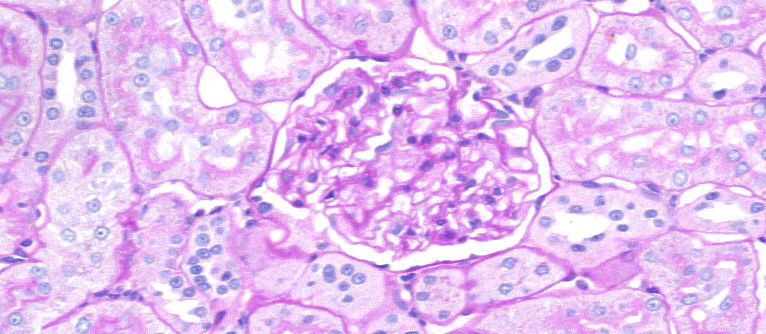

Supplement: Supplementary file 2 [file DataSheet14.ZIP › sham/Fig 1D-PAS-sham-7/7-2.jpeg]

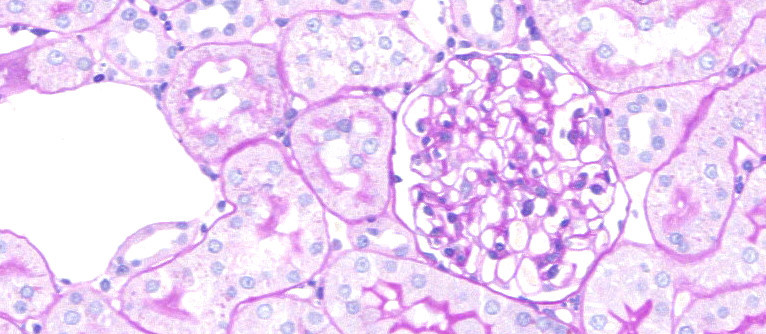

Supplement: Supplementary file 2 [file DataSheet14.ZIP › sham/Fig 1D-PAS-sham-7/7-20.jpeg]

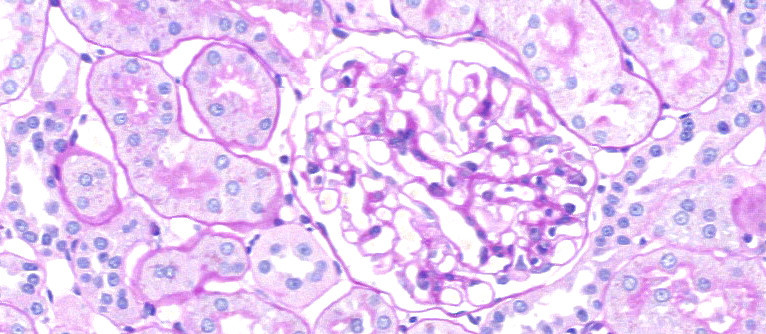

Supplement: Supplementary file 2 [file DataSheet14.ZIP › sham/Fig 1D-PAS-sham-7/7-3.jpeg]

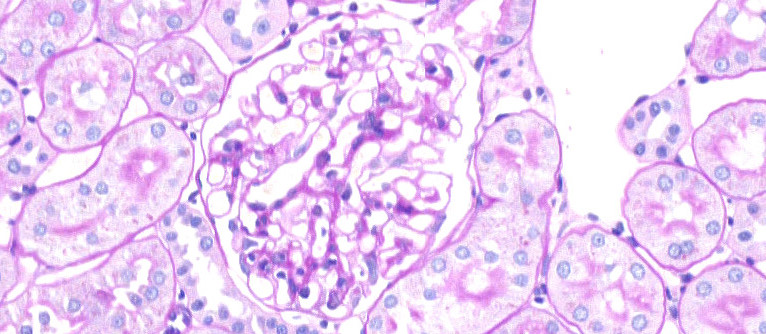

Supplement: Supplementary file 2 [file DataSheet14.ZIP › sham/Fig 1D-PAS-sham-7/7-4.jpeg]

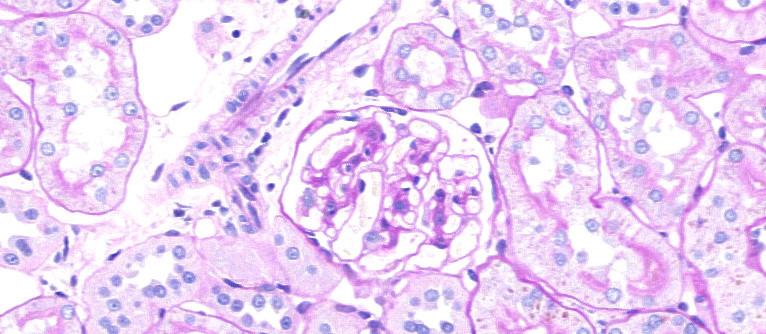

Supplement: Supplementary file 2 [file DataSheet14.ZIP › sham/Fig 1D-PAS-sham-7/7-5.jpeg]

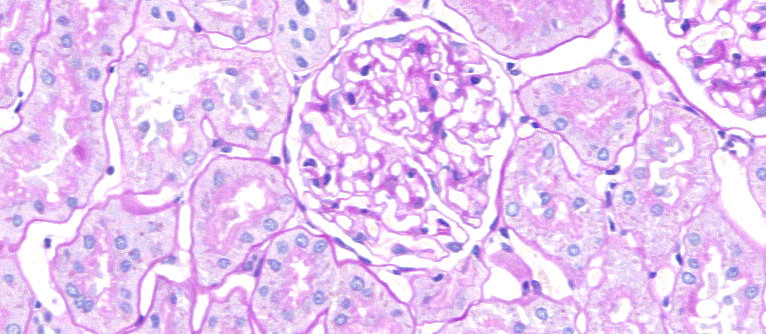

Supplement: Supplementary file 2 [file DataSheet14.ZIP › sham/Fig 1D-PAS-sham-7/7-6.jpeg]

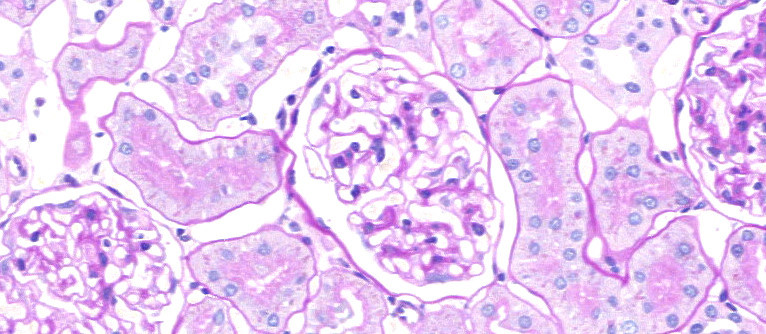

Supplement: Supplementary file 2 [file DataSheet14.ZIP › sham/Fig 1D-PAS-sham-7/7-7.jpeg]

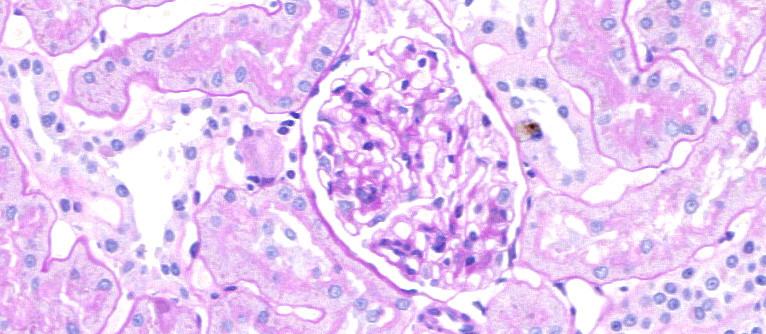

Supplement: Supplementary file 2 [file DataSheet14.ZIP › sham/Fig 1D-PAS-sham-7/7-8.jpeg]

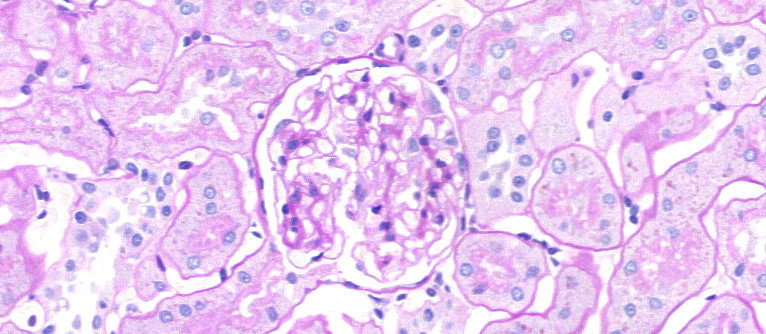

Supplement: Supplementary file 2 [file DataSheet14.ZIP › sham/Fig 1D-PAS-sham-7/7-9.jpeg]

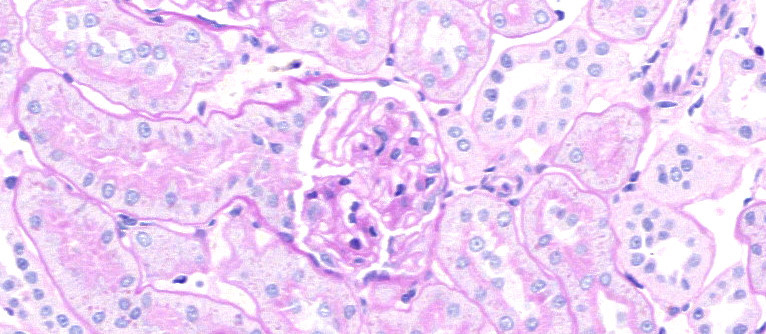

Supplement: Supplementary file 2 [file DataSheet14.ZIP › sham/Fig 1D-PAS-sham-8/8-1.jpeg]

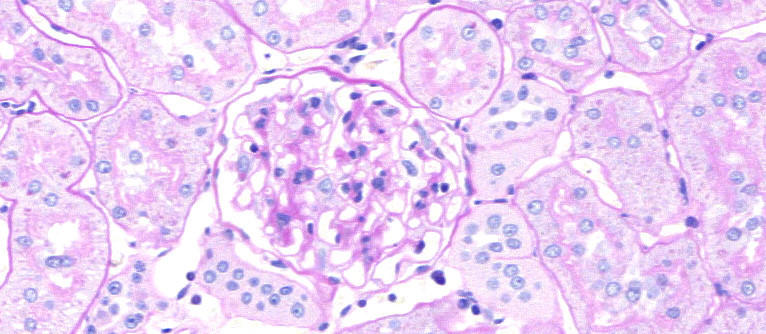

Supplement: Supplementary file 2 [file DataSheet14.ZIP › sham/Fig 1D-PAS-sham-8/8-10.jpeg]

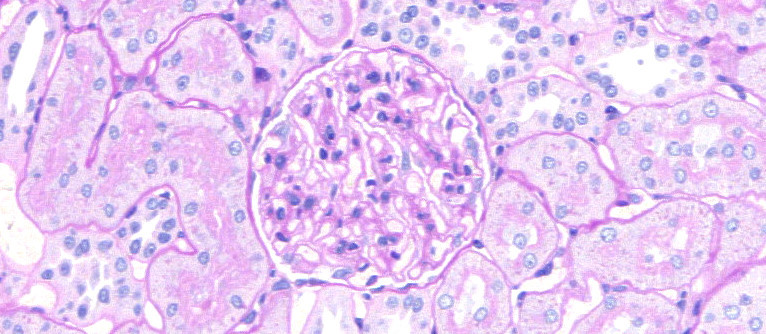

Supplement: Supplementary file 2 [file DataSheet14.ZIP › sham/Fig 1D-PAS-sham-8/8-11.jpeg]

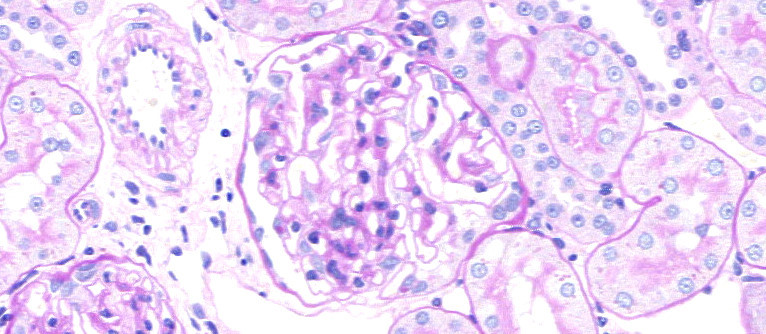

Supplement: Supplementary file 2 [file DataSheet14.ZIP › sham/Fig 1D-PAS-sham-8/8-12.jpeg]

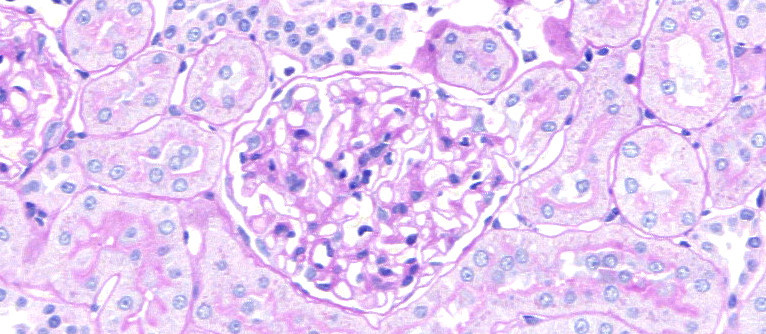

Supplement: Supplementary file 2 [file DataSheet14.ZIP › sham/Fig 1D-PAS-sham-8/8-13.jpeg]

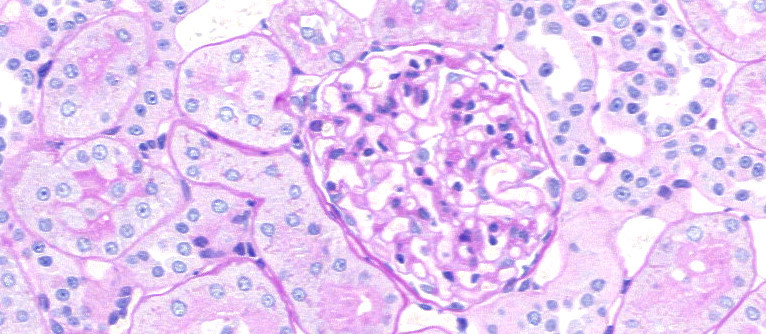

Supplement: Supplementary file 2 [file DataSheet14.ZIP › sham/Fig 1D-PAS-sham-8/8-14.jpeg]

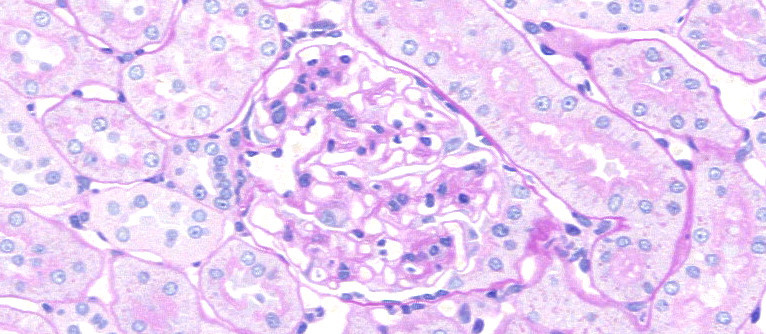

Supplement: Supplementary file 2 [file DataSheet14.ZIP › sham/Fig 1D-PAS-sham-8/8-15.jpeg]

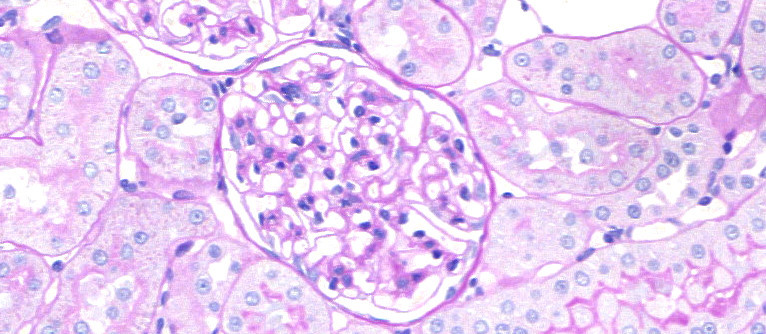

Supplement: Supplementary file 2 [file DataSheet14.ZIP › sham/Fig 1D-PAS-sham-8/8-16.jpeg]

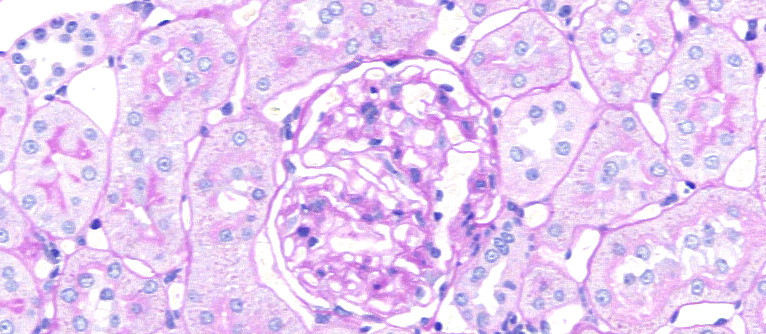

Supplement: Supplementary file 2 [file DataSheet14.ZIP › sham/Fig 1D-PAS-sham-8/8-17.jpeg]

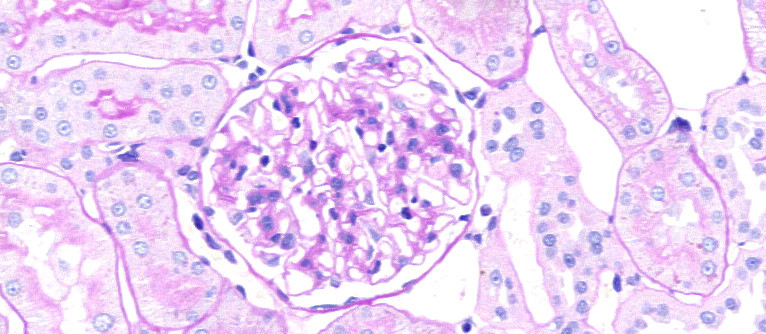

Supplement: Supplementary file 2 [file DataSheet14.ZIP › sham/Fig 1D-PAS-sham-8/8-18.jpeg]

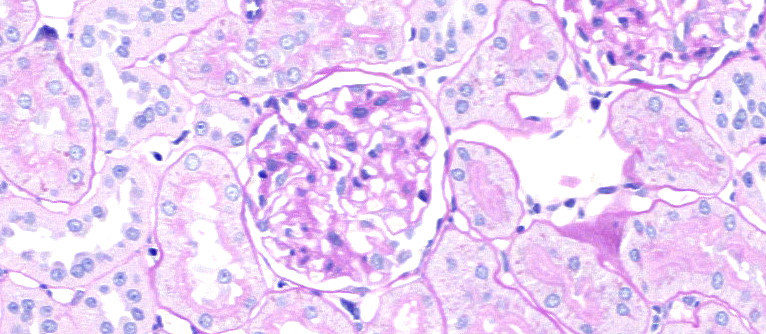

Supplement: Supplementary file 2 [file DataSheet14.ZIP › sham/Fig 1D-PAS-sham-8/8-19.jpeg]

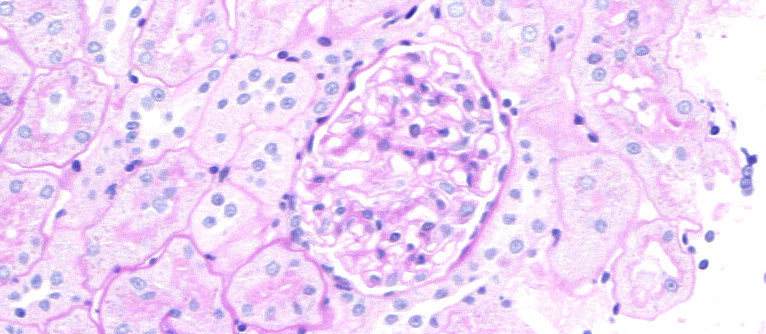

Supplement: Supplementary file 2 [file DataSheet14.ZIP › sham/Fig 1D-PAS-sham-8/8-2.jpeg]

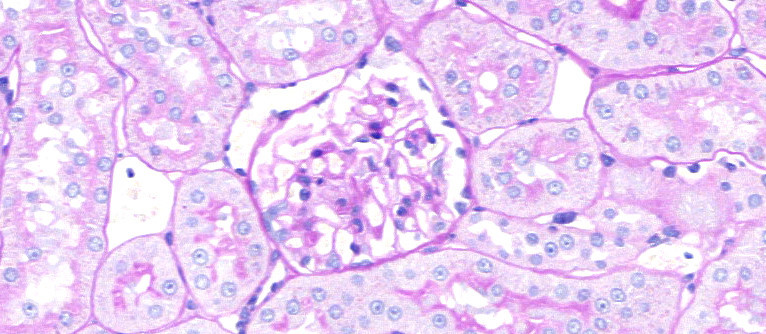

Supplement: Supplementary file 2 [file DataSheet14.ZIP › sham/Fig 1D-PAS-sham-8/8-20.jpeg]

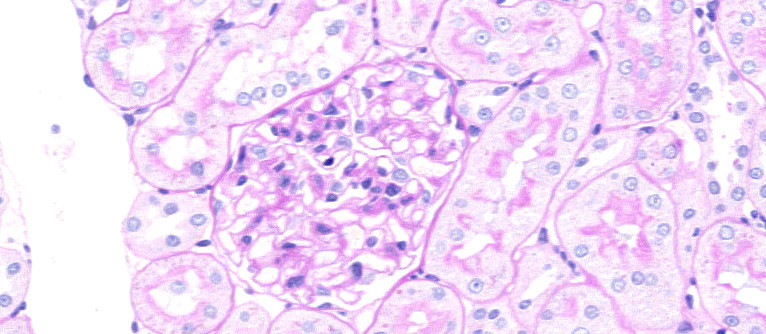

Supplement: Supplementary file 2 [file DataSheet14.ZIP › sham/Fig 1D-PAS-sham-8/8-3.jpeg]

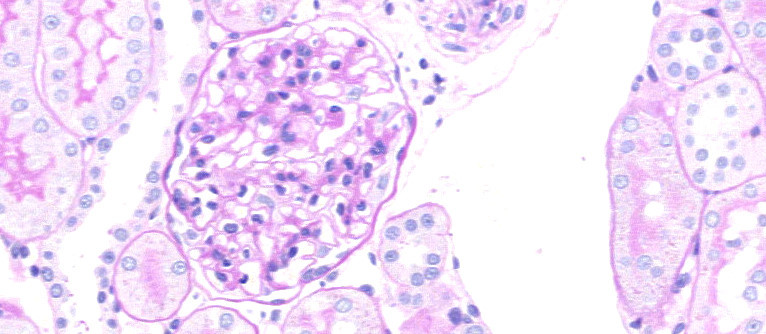

Supplement: Supplementary file 2 [file DataSheet14.ZIP › sham/Fig 1D-PAS-sham-8/8-4.jpeg]

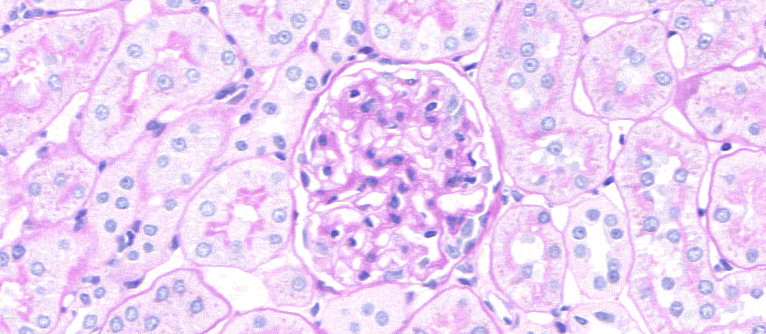

Supplement: Supplementary file 2 [file DataSheet14.ZIP › sham/Fig 1D-PAS-sham-8/8-5.jpeg]

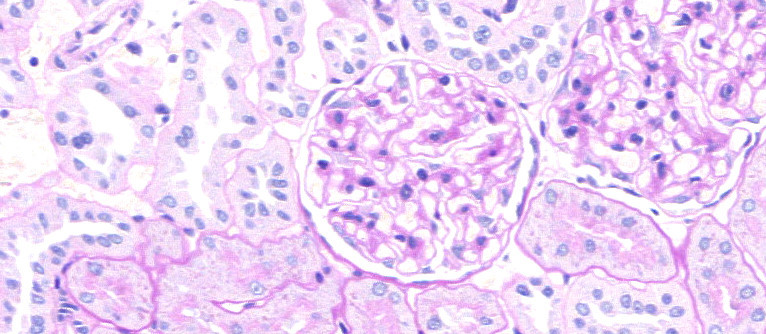

Supplement: Supplementary file 2 [file DataSheet14.ZIP › sham/Fig 1D-PAS-sham-8/8-6.jpeg]

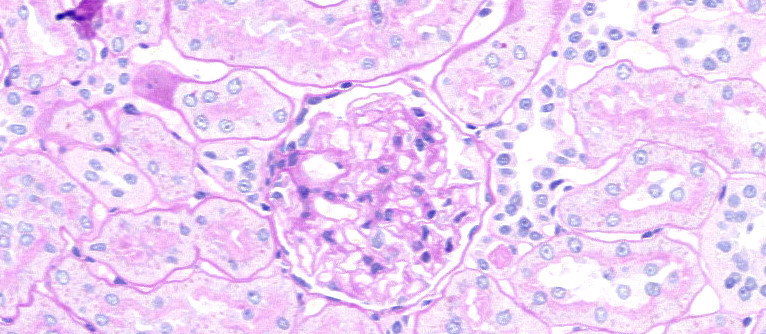

Supplement: Supplementary file 2 [file DataSheet14.ZIP › sham/Fig 1D-PAS-sham-8/8-7.jpeg]

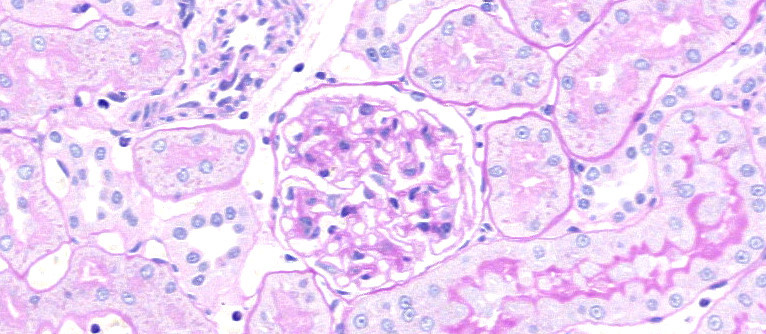

Supplement: Supplementary file 2 [file DataSheet14.ZIP › sham/Fig 1D-PAS-sham-8/8-8.jpeg]

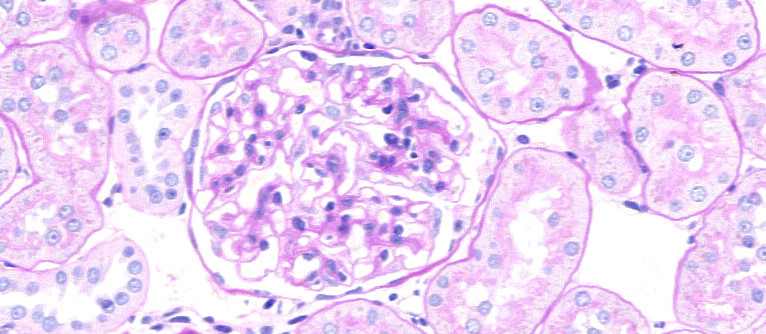

Supplement: Supplementary file 2 [file DataSheet14.ZIP › sham/Fig 1D-PAS-sham-8/8-9.jpeg]

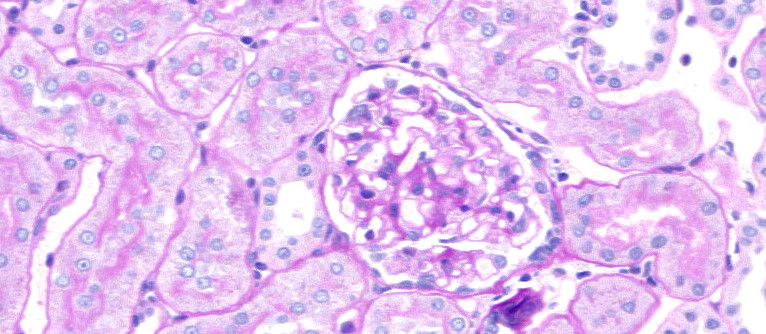

Supplement: Supplementary file 2 [file DataSheet14.ZIP › sham/Fig 1D-PAS-sham-9/9-1.jpeg]

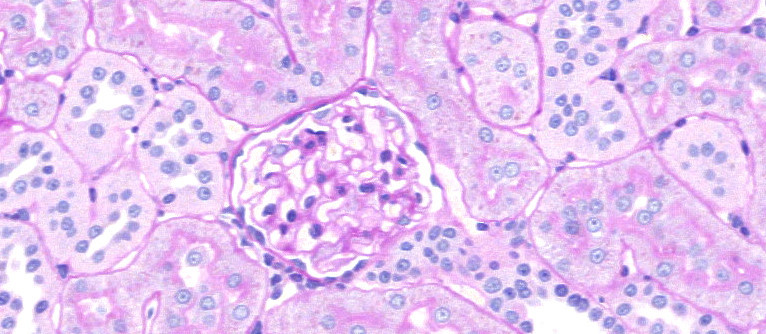

Supplement: Supplementary file 2 [file DataSheet14.ZIP › sham/Fig 1D-PAS-sham-9/9-10.jpeg]

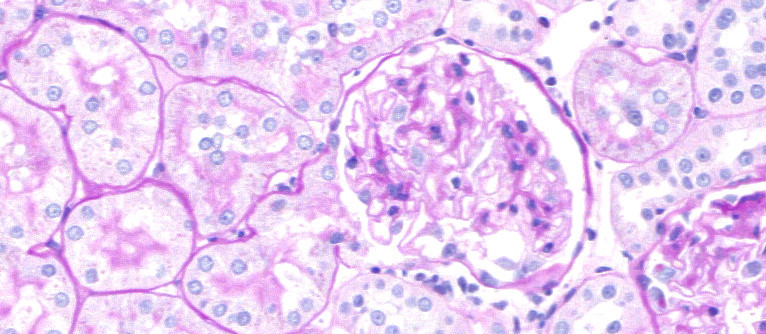

Supplement: Supplementary file 2 [file DataSheet14.ZIP › sham/Fig 1D-PAS-sham-9/9-11.jpeg]

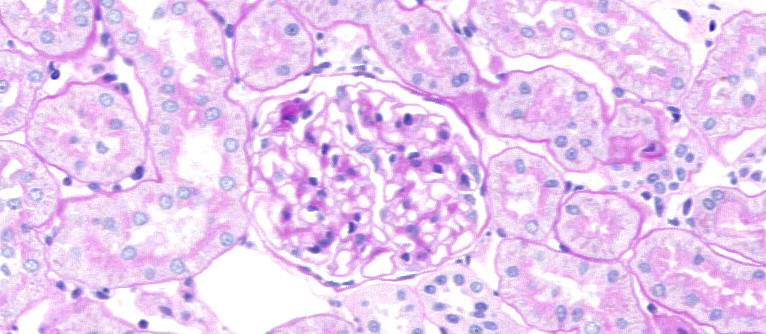

Supplement: Supplementary file 2 [file DataSheet14.ZIP › sham/Fig 1D-PAS-sham-9/9-12.jpeg]

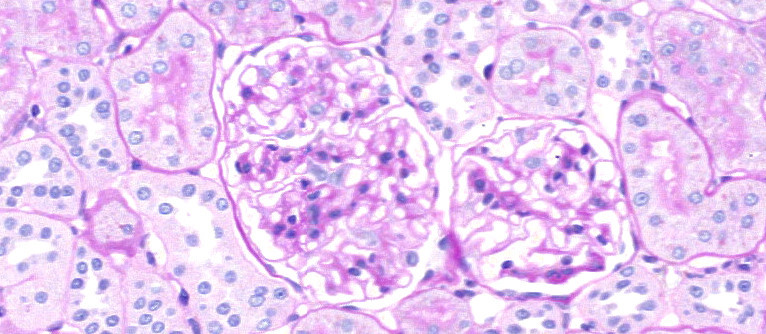

Supplement: Supplementary file 2 [file DataSheet14.ZIP › sham/Fig 1D-PAS-sham-9/9-13.jpeg]

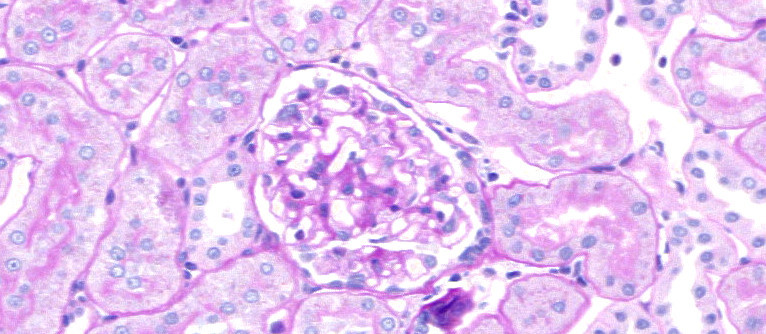

Supplement: Supplementary file 2 [file DataSheet14.ZIP › sham/Fig 1D-PAS-sham-9/9-14.jpeg]

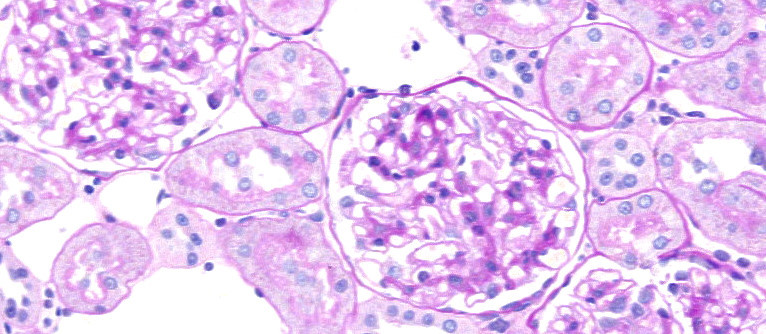

Supplement: Supplementary file 2 [file DataSheet14.ZIP › sham/Fig 1D-PAS-sham-9/9-15.jpeg]

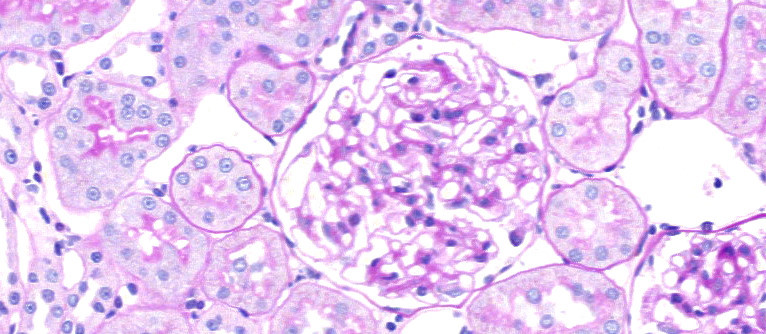

Supplement: Supplementary file 2 [file DataSheet14.ZIP › sham/Fig 1D-PAS-sham-9/9-16.jpeg]

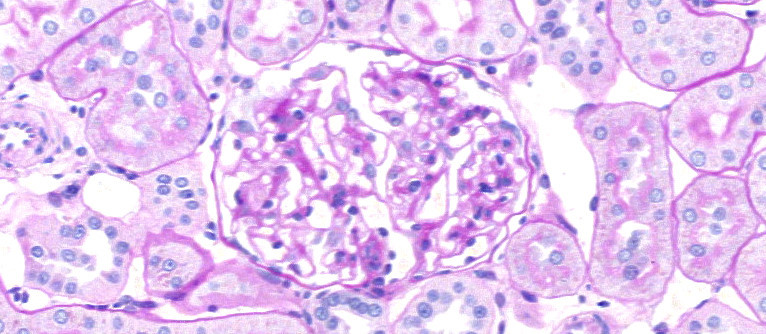

Supplement: Supplementary file 2 [file DataSheet14.ZIP › sham/Fig 1D-PAS-sham-9/9-17.jpeg]

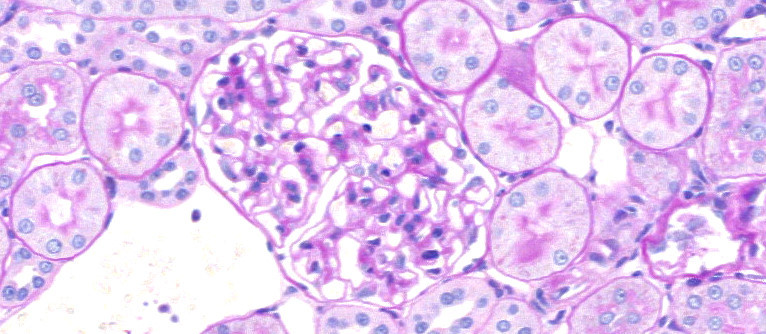

Supplement: Supplementary file 2 [file DataSheet14.ZIP › sham/Fig 1D-PAS-sham-9/9-18.jpeg]

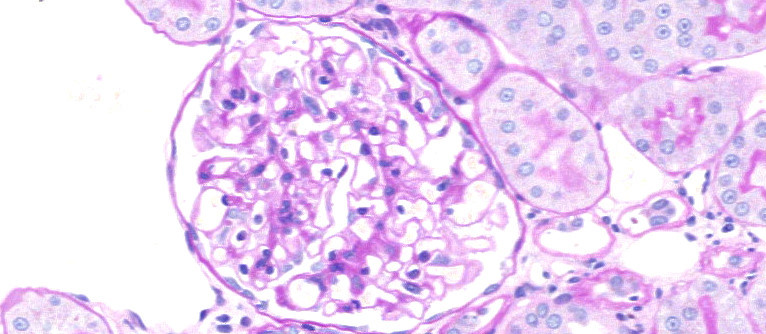

Supplement: Supplementary file 2 [file DataSheet14.ZIP › sham/Fig 1D-PAS-sham-9/9-19.jpeg]

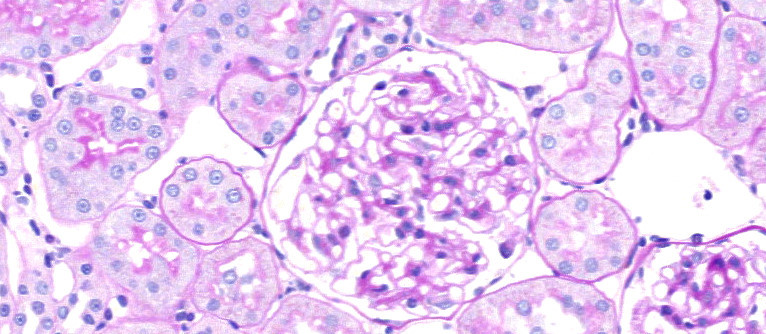

Supplement: Supplementary file 2 [file DataSheet14.ZIP › sham/Fig 1D-PAS-sham-9/9-2.jpeg]

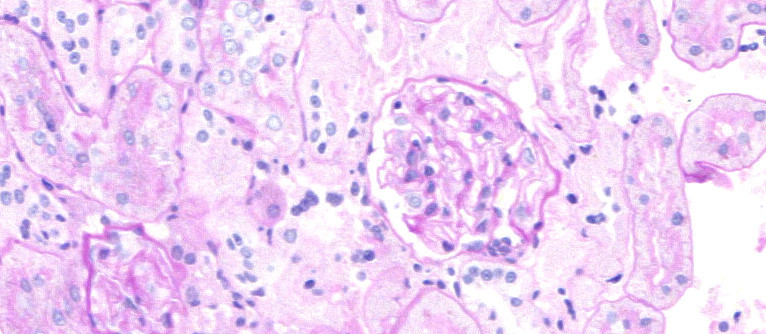

Supplement: Supplementary file 2 [file DataSheet14.ZIP › sham/Fig 1D-PAS-sham-9/9-20.jpeg]

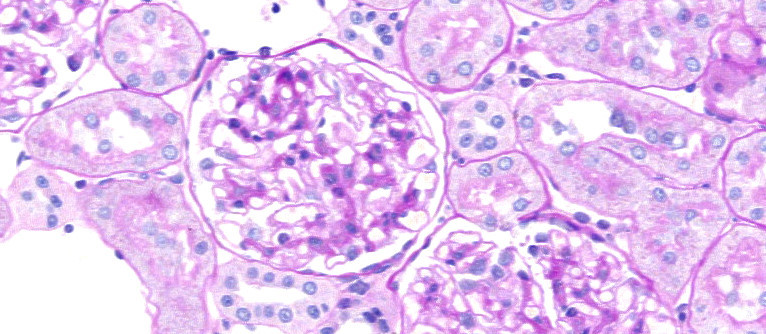

Supplement: Supplementary file 2 [file DataSheet14.ZIP › sham/Fig 1D-PAS-sham-9/9-3.jpeg]

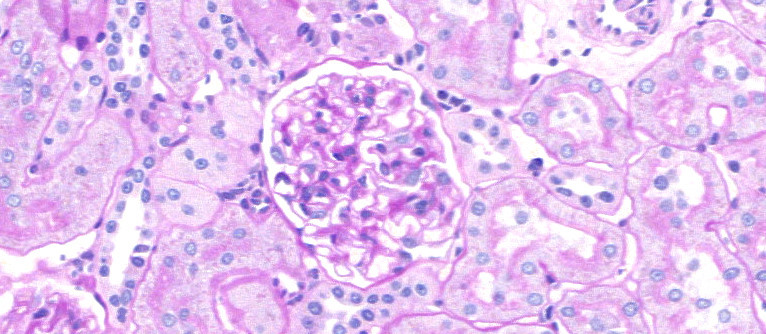

Supplement: Supplementary file 2 [file DataSheet14.ZIP › sham/Fig 1D-PAS-sham-9/9-4.jpeg]

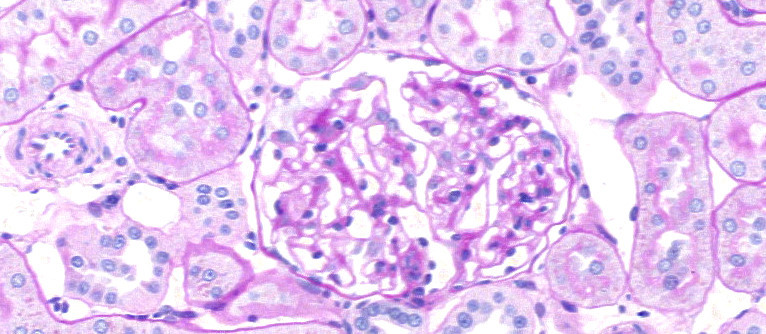

Supplement: Supplementary file 2 [file DataSheet14.ZIP › sham/Fig 1D-PAS-sham-9/9-5.jpeg]

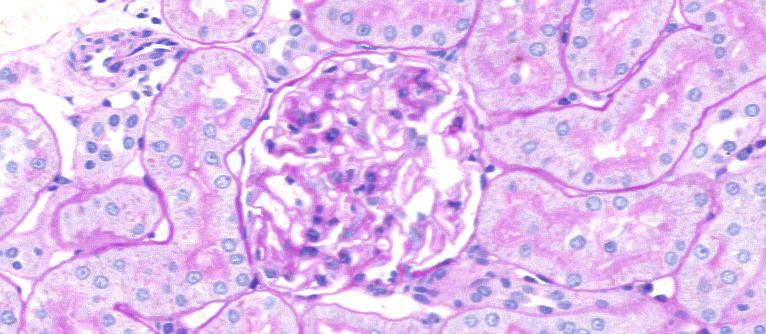

Supplement: Supplementary file 2 [file DataSheet14.ZIP › sham/Fig 1D-PAS-sham-9/9-6.jpeg]

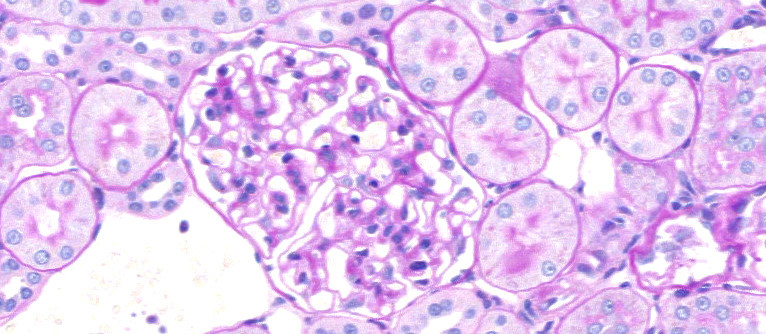

Supplement: Supplementary file 2 [file DataSheet14.ZIP › sham/Fig 1D-PAS-sham-9/9-7.jpeg]

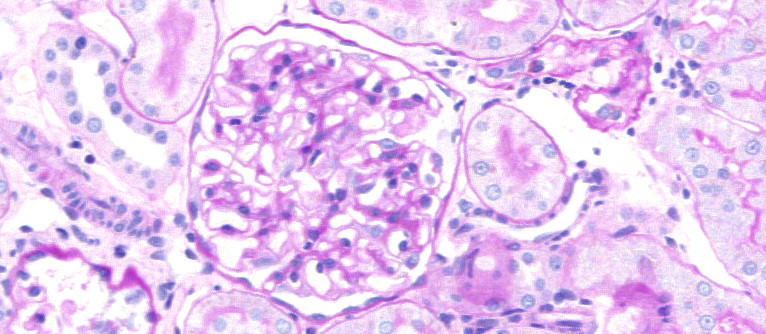

Supplement: Supplementary file 2 [file DataSheet14.ZIP › sham/Fig 1D-PAS-sham-9/9-8.jpeg]

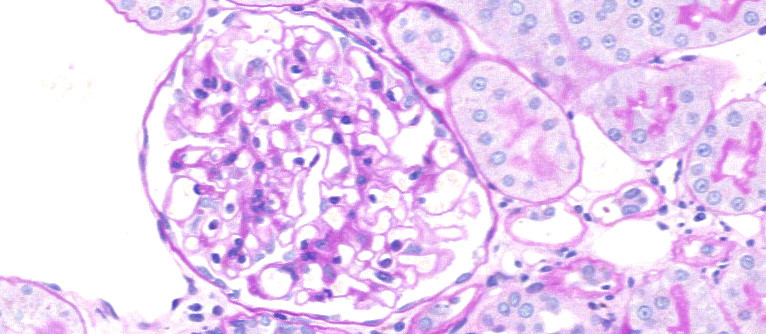

Supplement: Supplementary file 2 [file DataSheet14.ZIP › sham/Fig 1D-PAS-sham-9/9-9.jpeg]

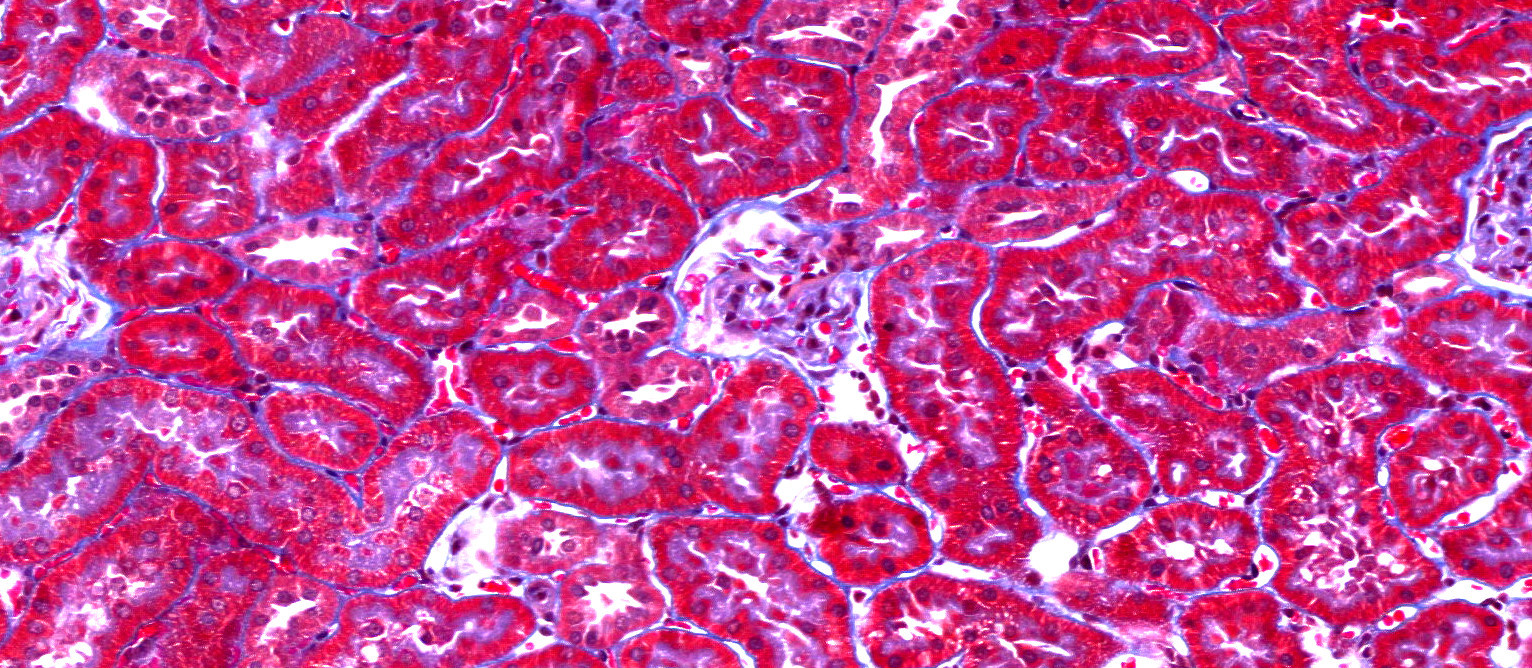

Supplement: Supplementary file 3 [file DataSheet11.ZIP › Fig 1D-masson-TSF-57(2)/57-10.jpeg]

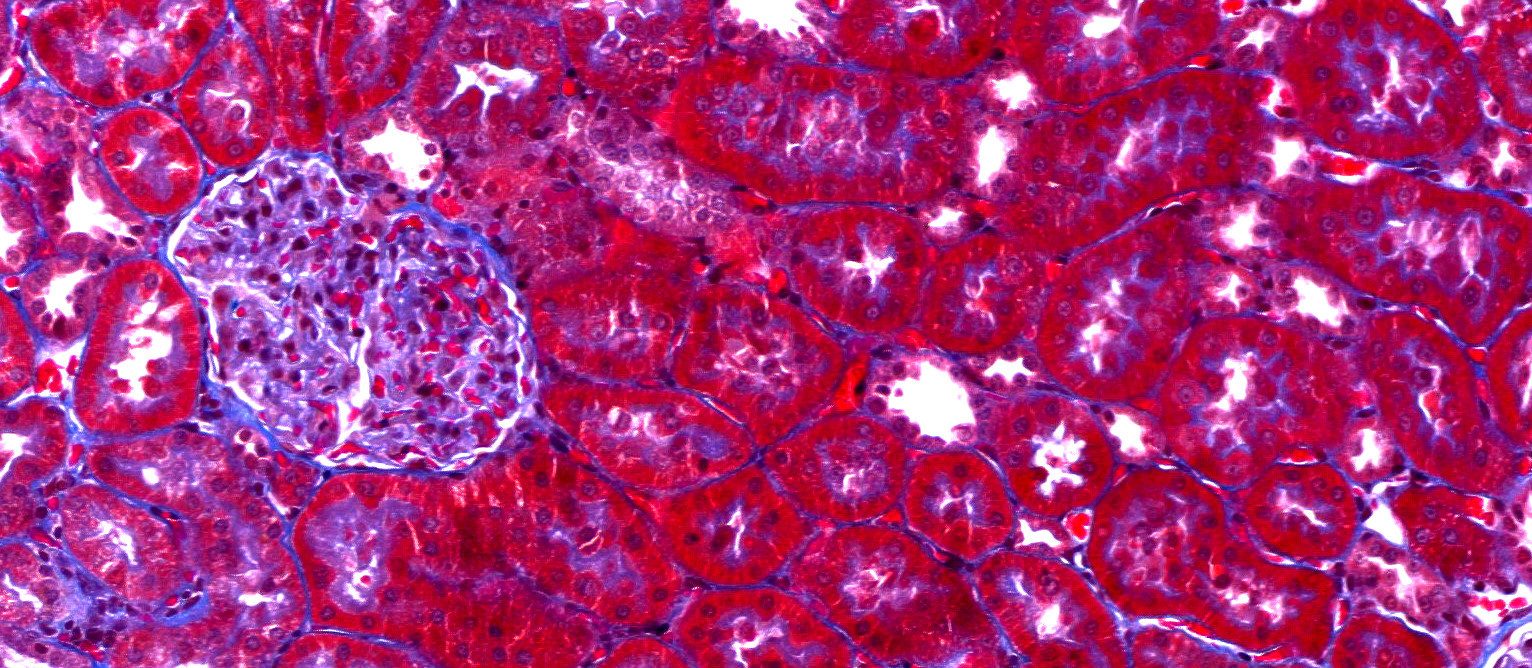

Supplement: Supplementary file 3 [file DataSheet11.ZIP › Fig 1D-masson-TSF-57(2)/57-5.jpeg]

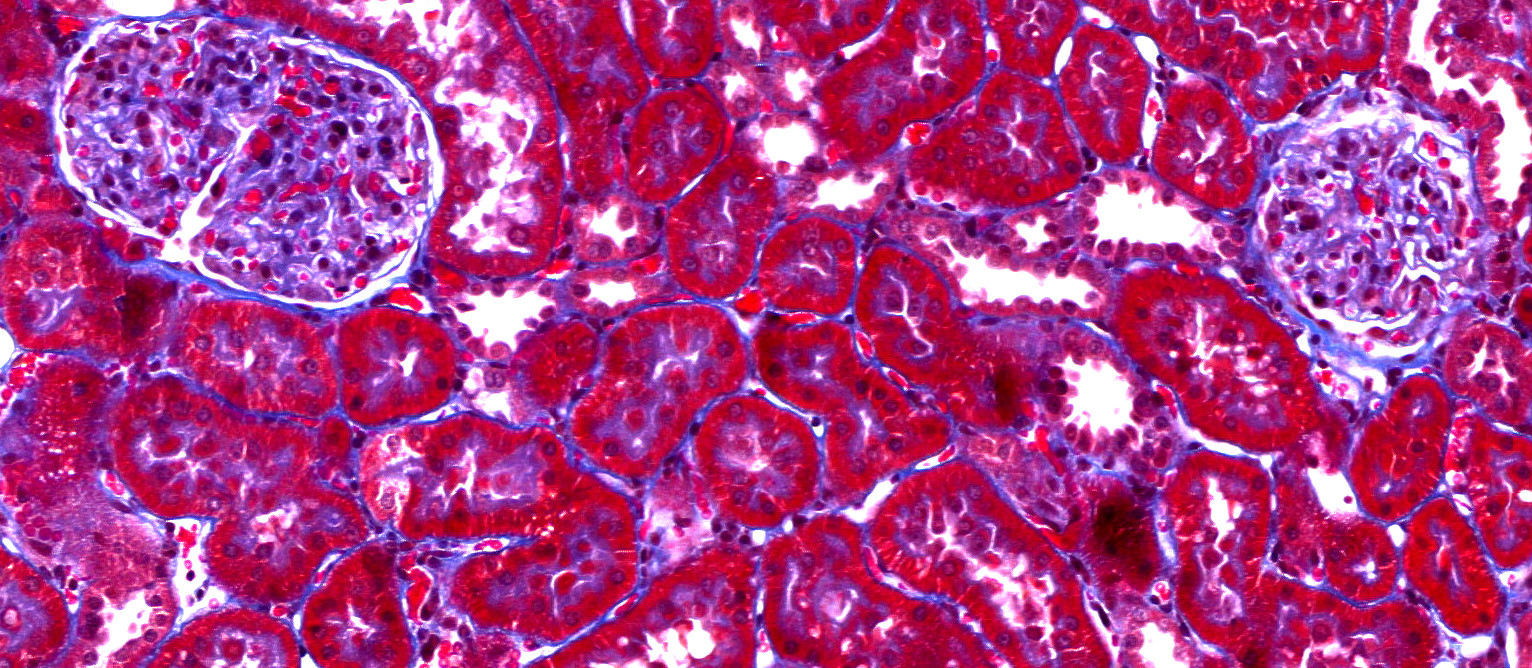

Supplement: Supplementary file 3 [file DataSheet11.ZIP › Fig 1D-masson-TSF-57(2)/57-6.jpeg]

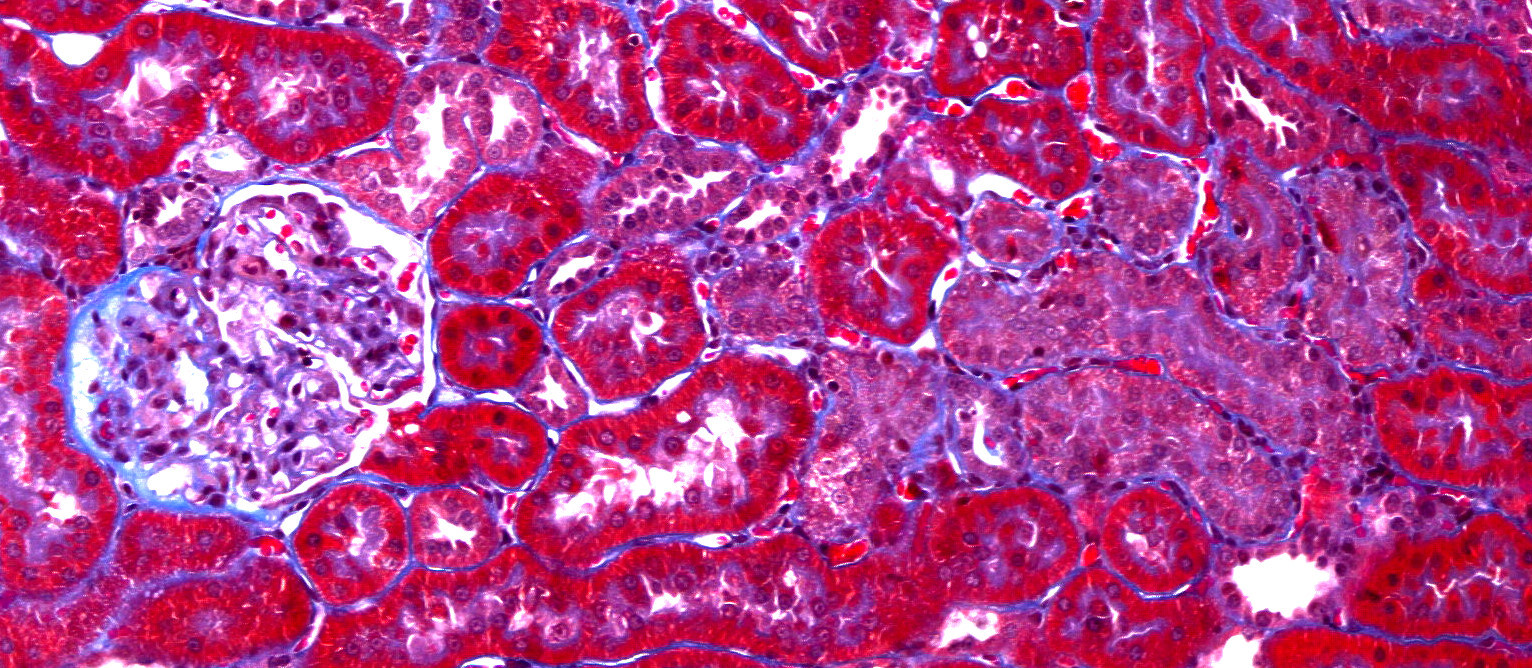

Supplement: Supplementary file 3 [file DataSheet11.ZIP › Fig 1D-masson-TSF-57(2)/57-7.jpeg]

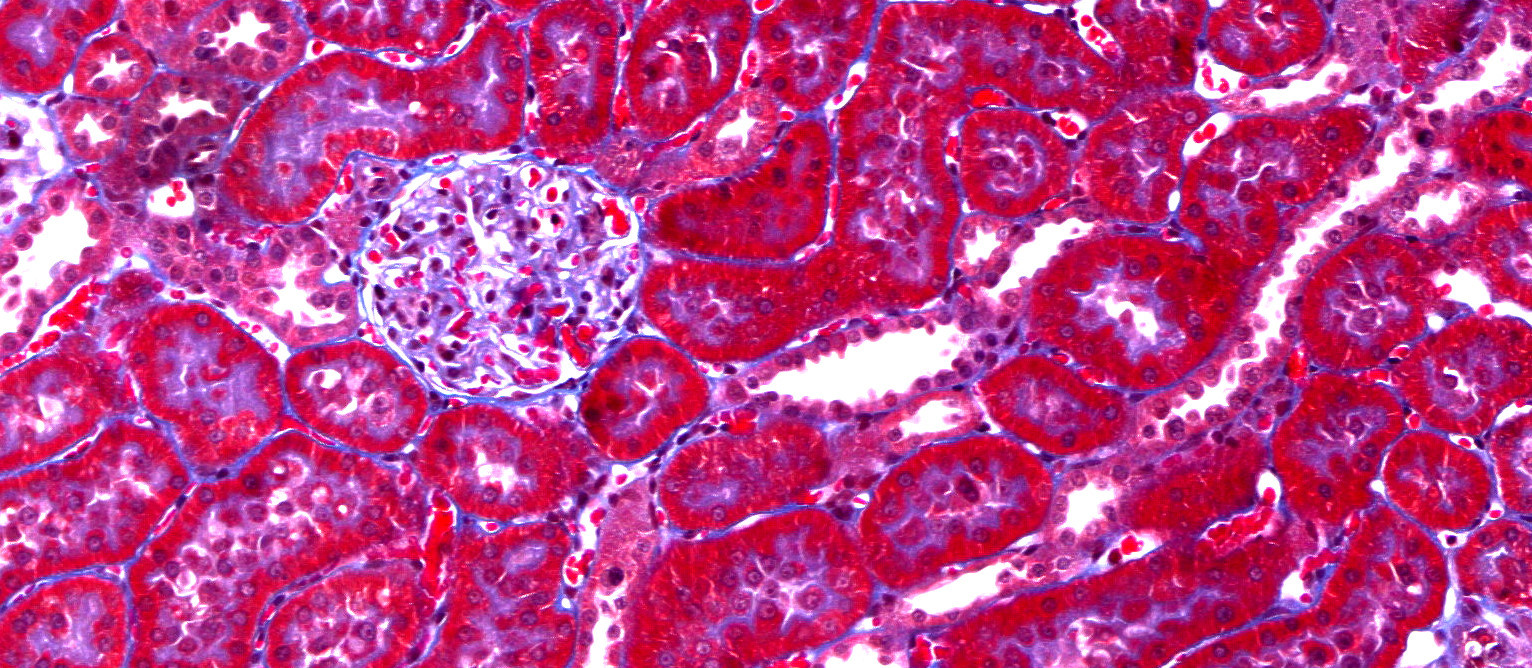

Supplement: Supplementary file 3 [file DataSheet11.ZIP › Fig 1D-masson-TSF-57(2)/57-8.jpeg]

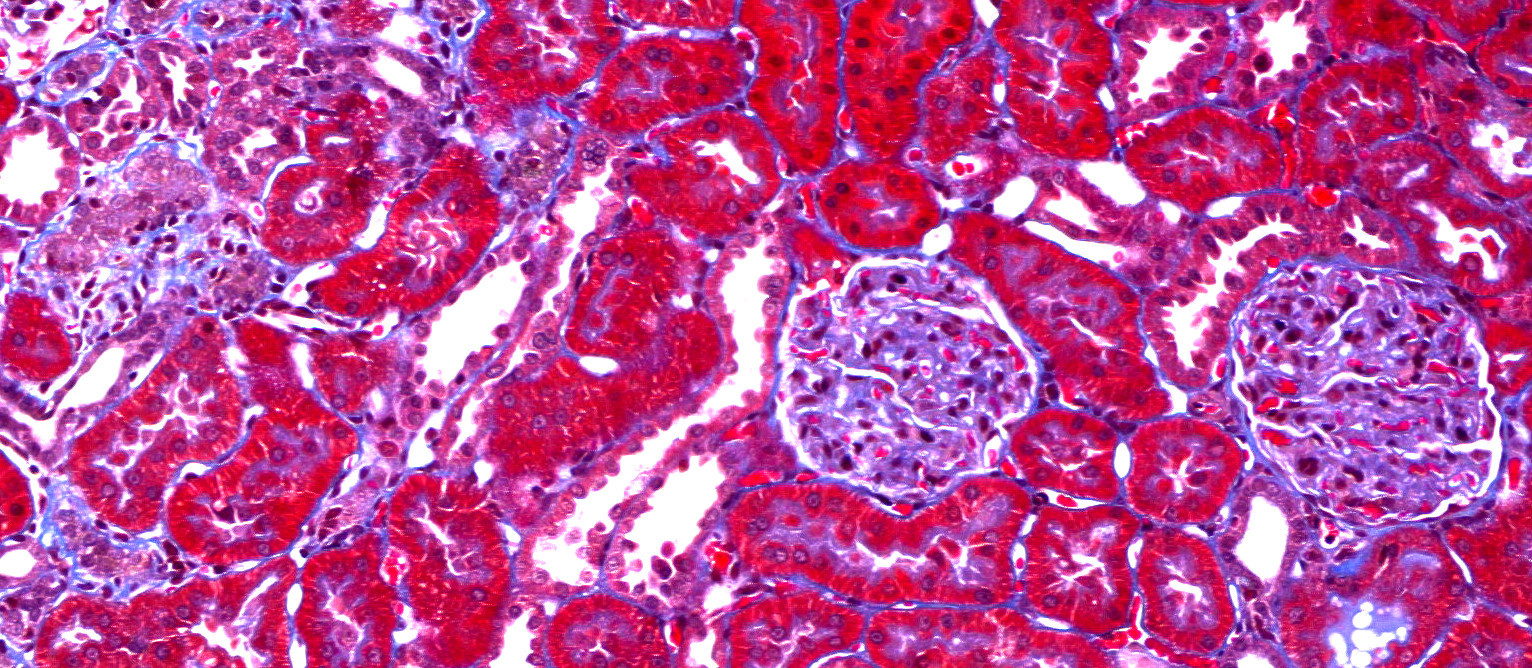

Supplement: Supplementary file 3 [file DataSheet11.ZIP › Fig 1D-masson-TSF-57(2)/57-9.jpeg]

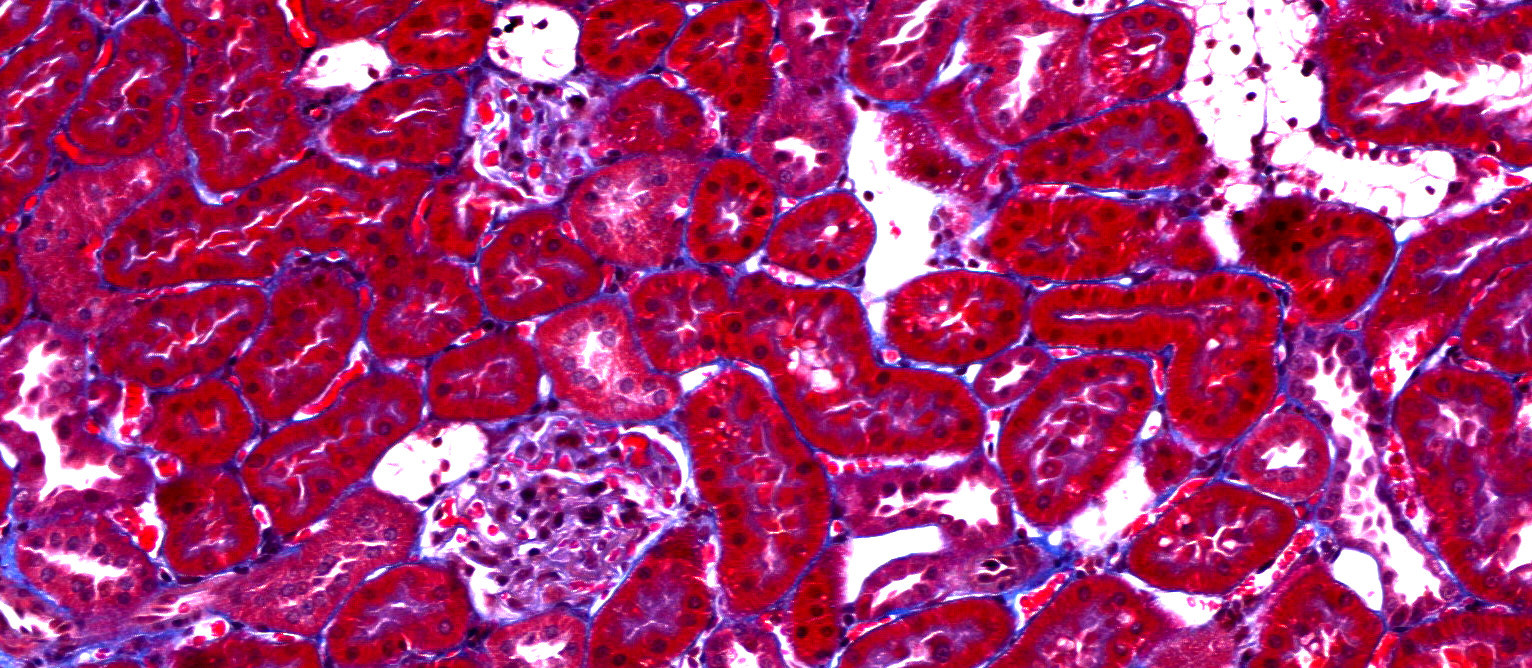

Supplement: Supplementary file 3 [file DataSheet11.ZIP › Fig 1D-masson-TSF-58/58-1.jpeg]

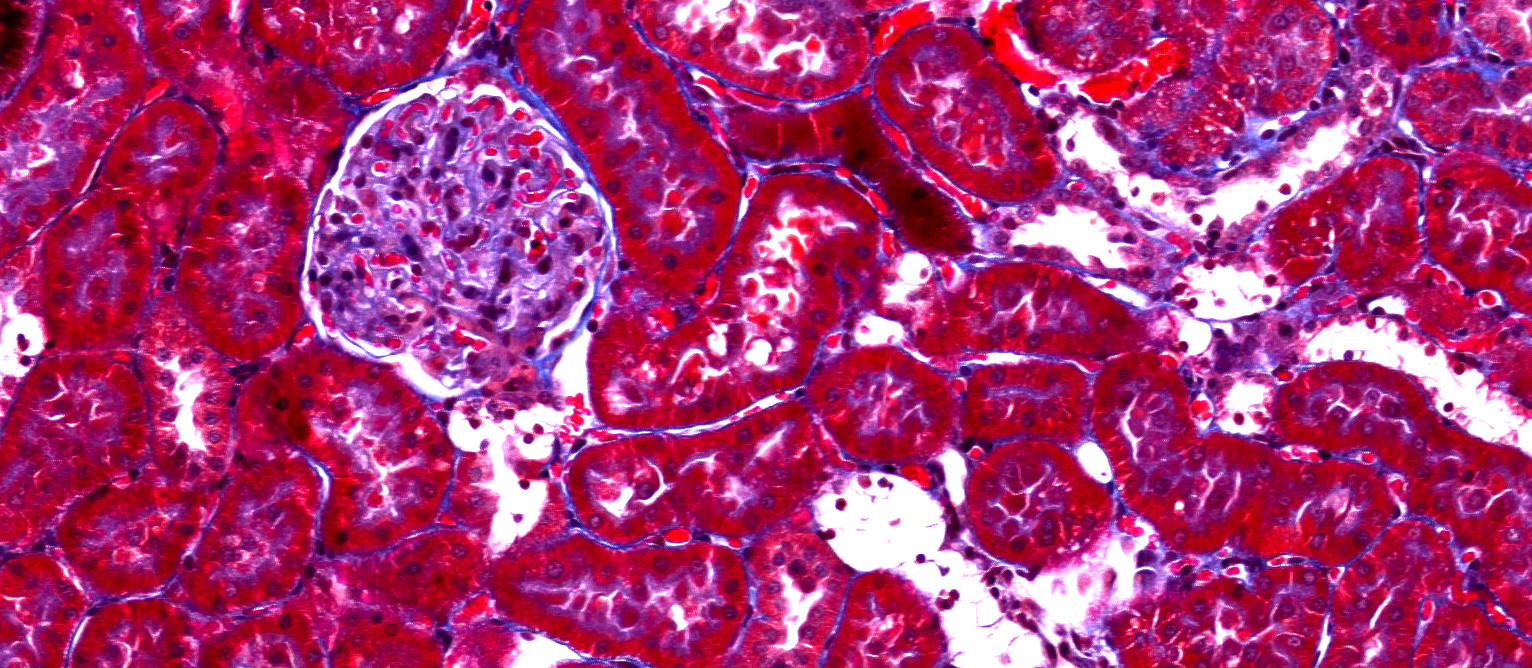

Supplement: Supplementary file 3 [file DataSheet11.ZIP › Fig 1D-masson-TSF-58/58-10.jpeg]

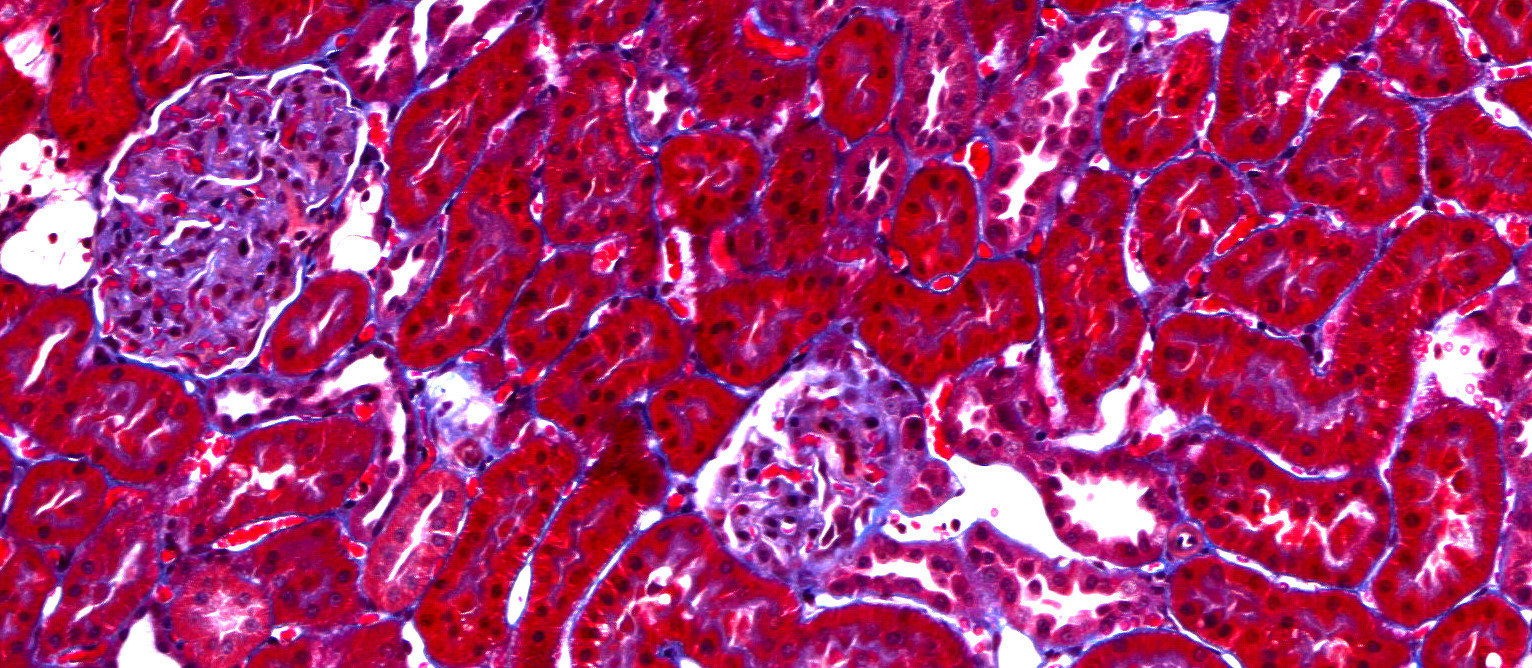

Supplement: Supplementary file 3 [file DataSheet11.ZIP › Fig 1D-masson-TSF-58/58-2.jpeg]

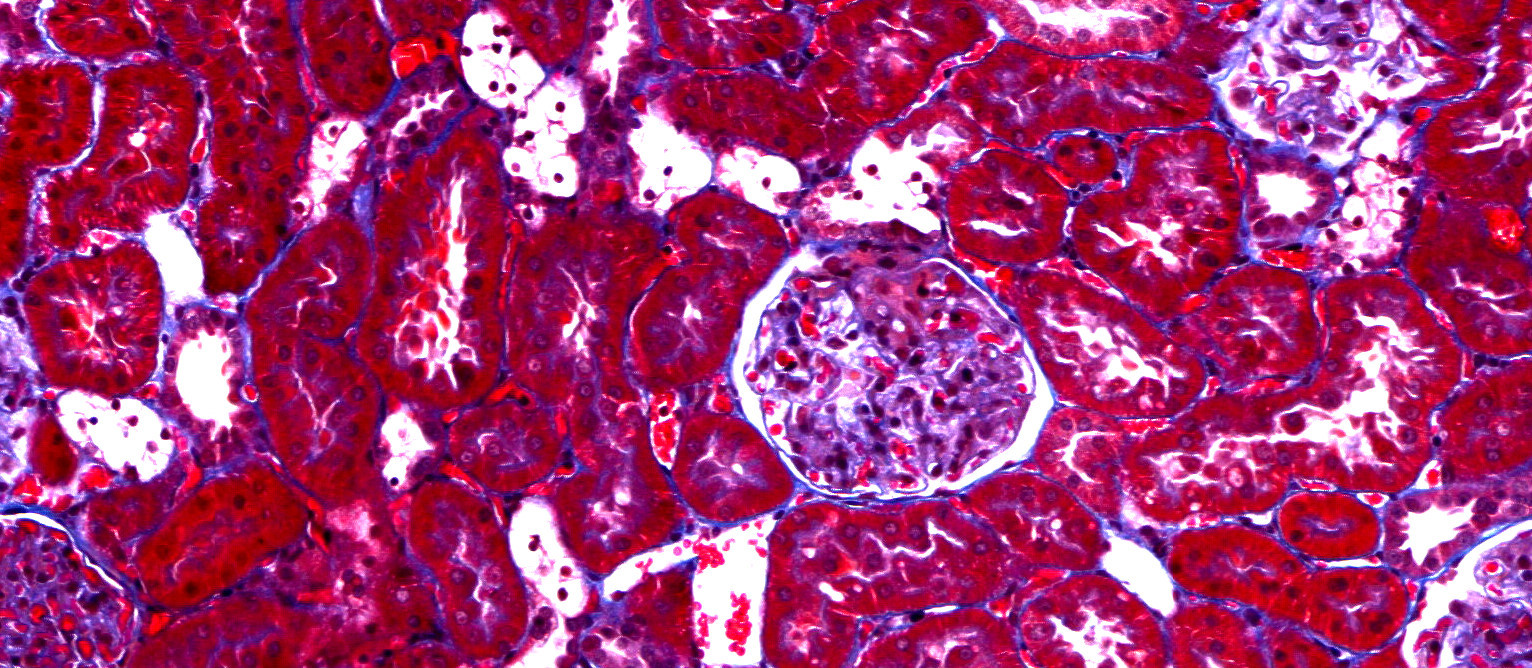

Supplement: Supplementary file 3 [file DataSheet11.ZIP › Fig 1D-masson-TSF-58/58-3.jpeg]

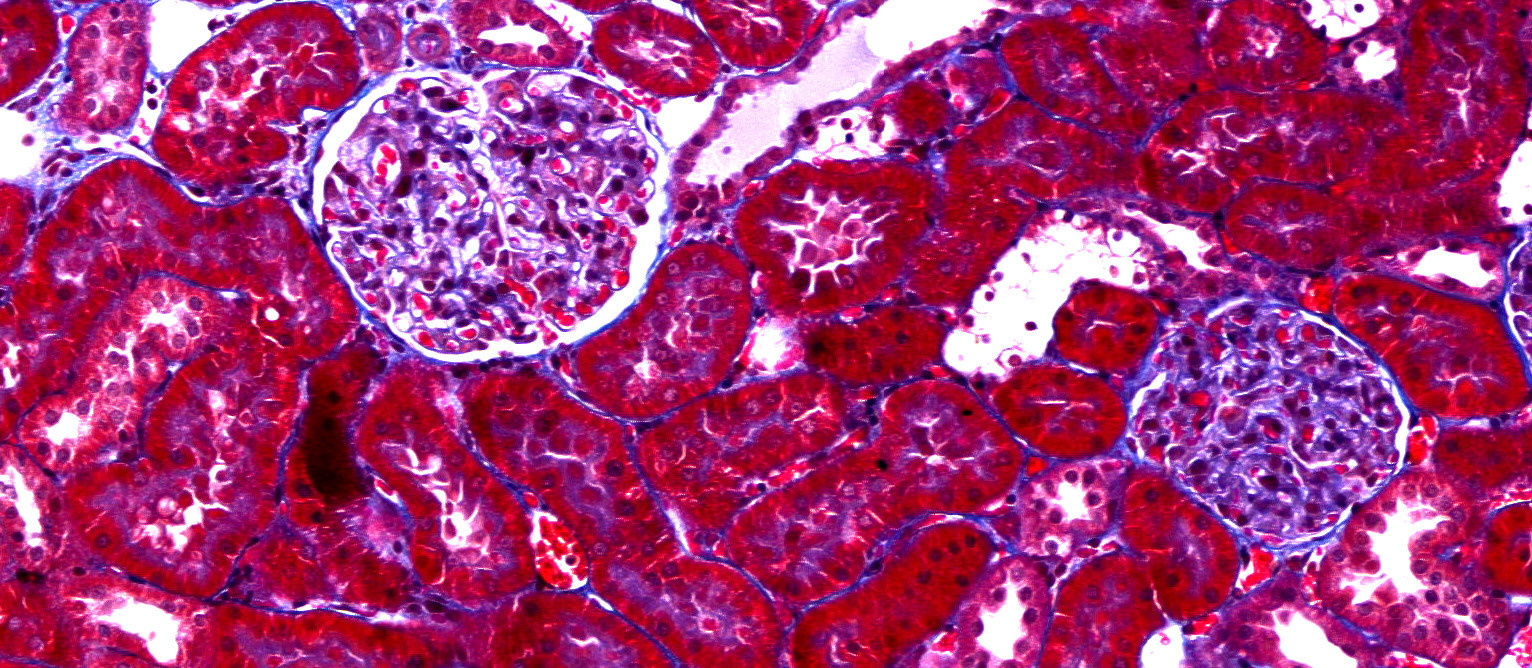

Supplement: Supplementary file 3 [file DataSheet11.ZIP › Fig 1D-masson-TSF-58/58-4.jpeg]

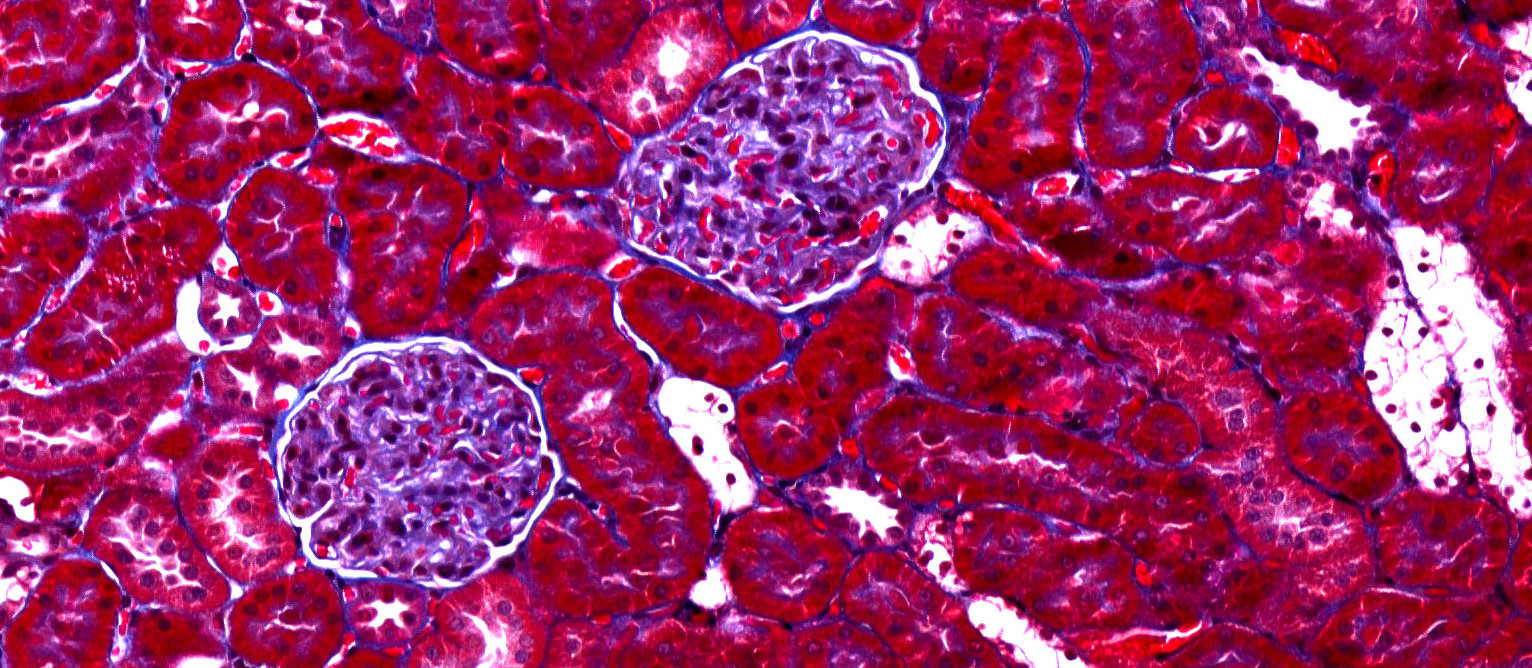

Supplement: Supplementary file 3 [file DataSheet11.ZIP › Fig 1D-masson-TSF-58/58-5.jpeg]

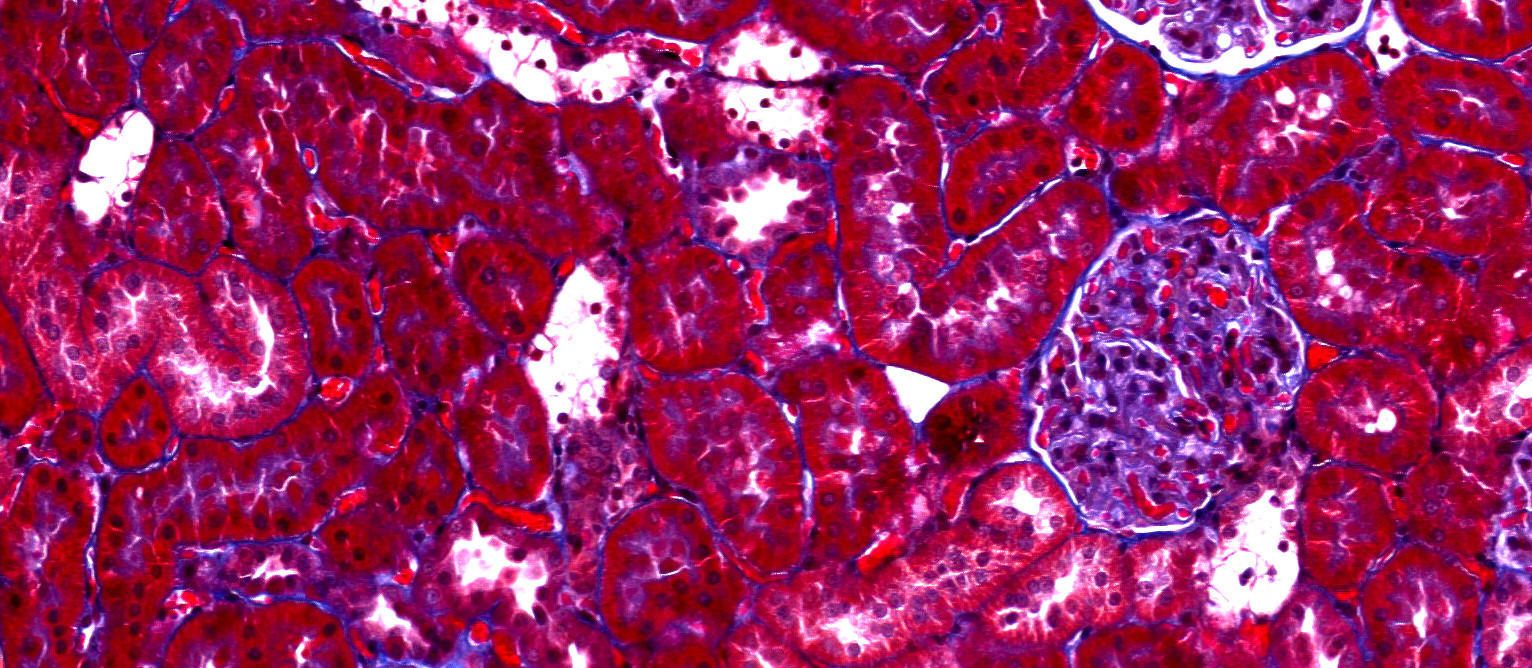

Supplement: Supplementary file 3 [file DataSheet11.ZIP › Fig 1D-masson-TSF-58/58-6.jpeg]

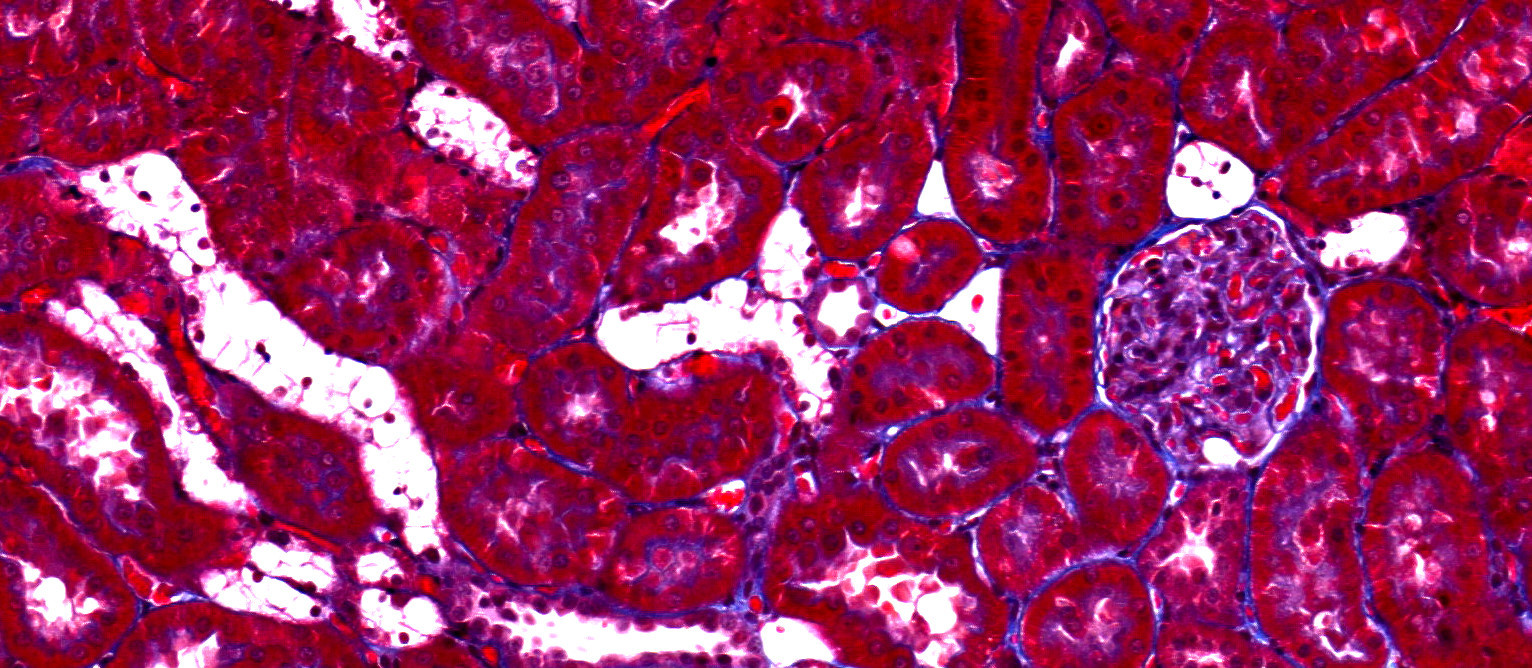

Supplement: Supplementary file 3 [file DataSheet11.ZIP › Fig 1D-masson-TSF-58/58-7.jpeg]

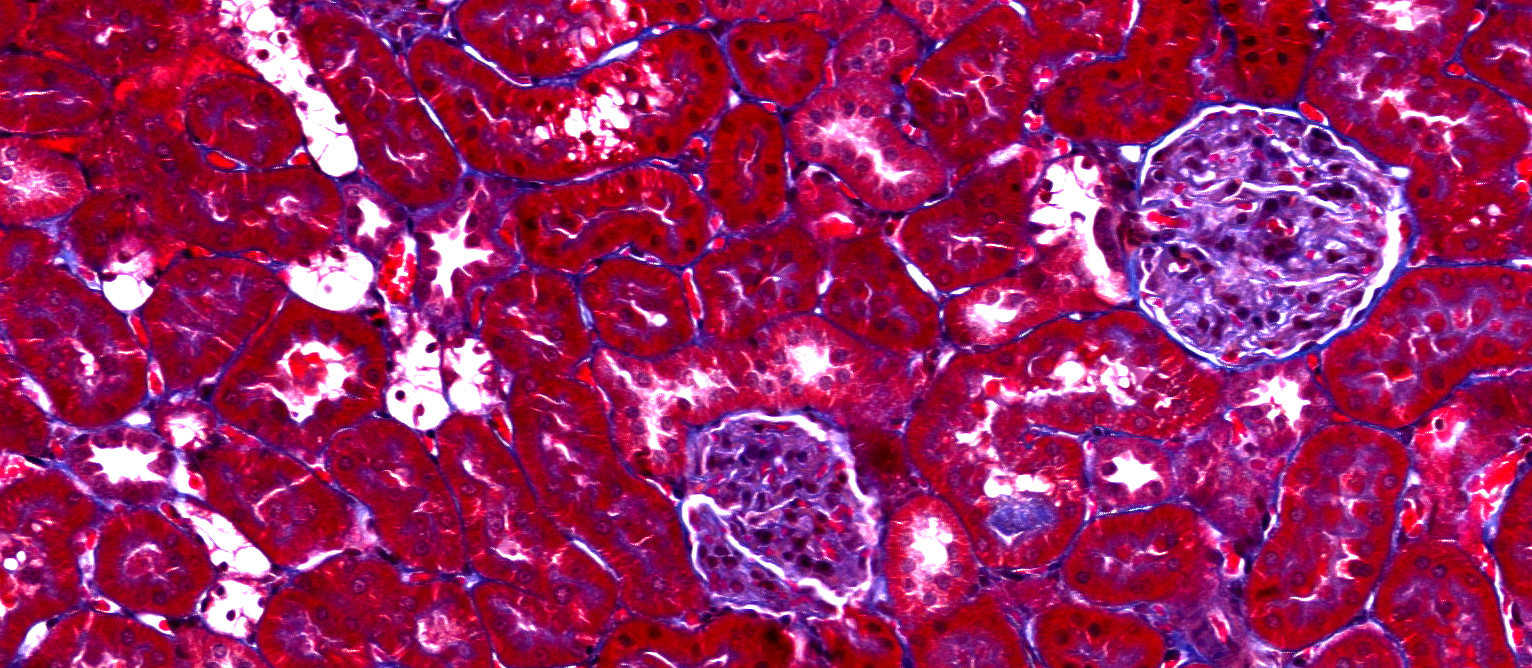

Supplement: Supplementary file 3 [file DataSheet11.ZIP › Fig 1D-masson-TSF-58/58-8.jpeg]

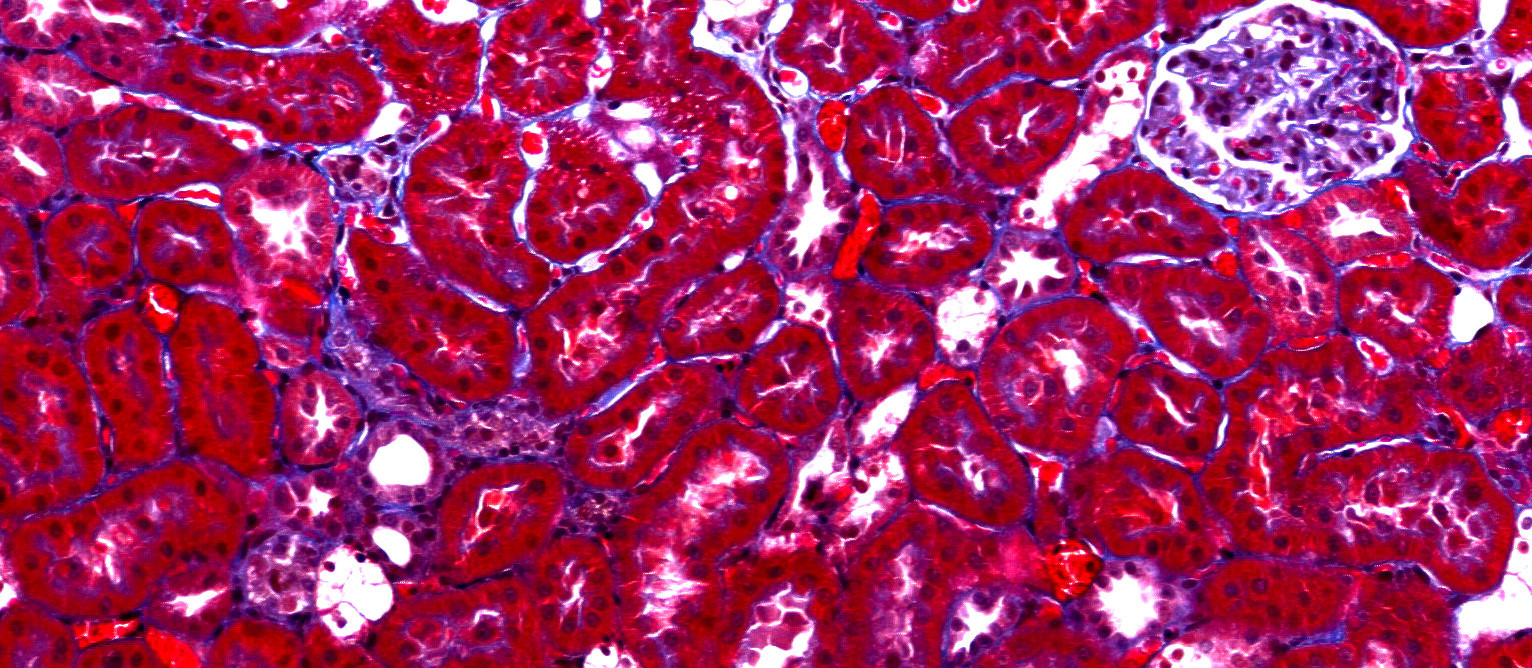

Supplement: Supplementary file 3 [file DataSheet11.ZIP › Fig 1D-masson-TSF-58/58-9.jpeg]

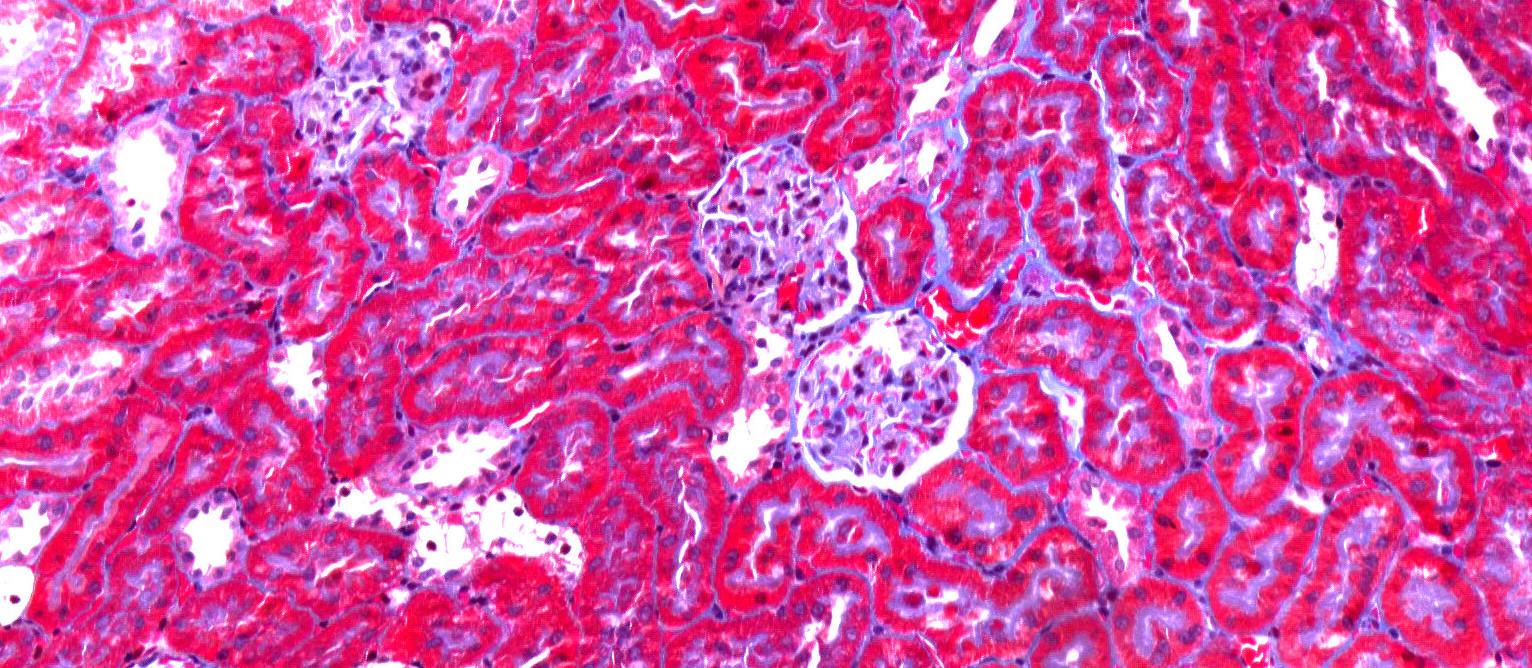

Supplement: Supplementary file 3 [file DataSheet11.ZIP › Fig 1D-masson-TSF-59/59-1.jpeg]

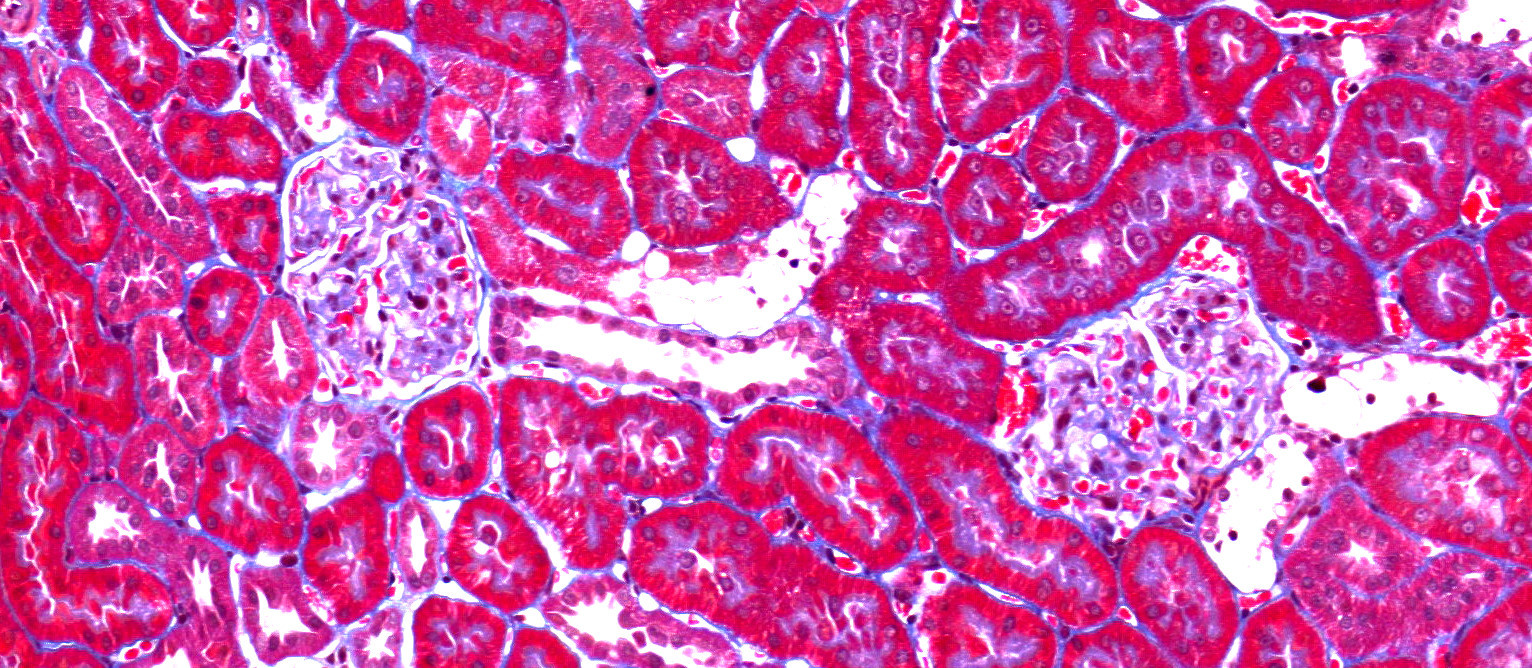

Supplement: Supplementary file 3 [file DataSheet11.ZIP › Fig 1D-masson-TSF-59/59-10.jpeg]

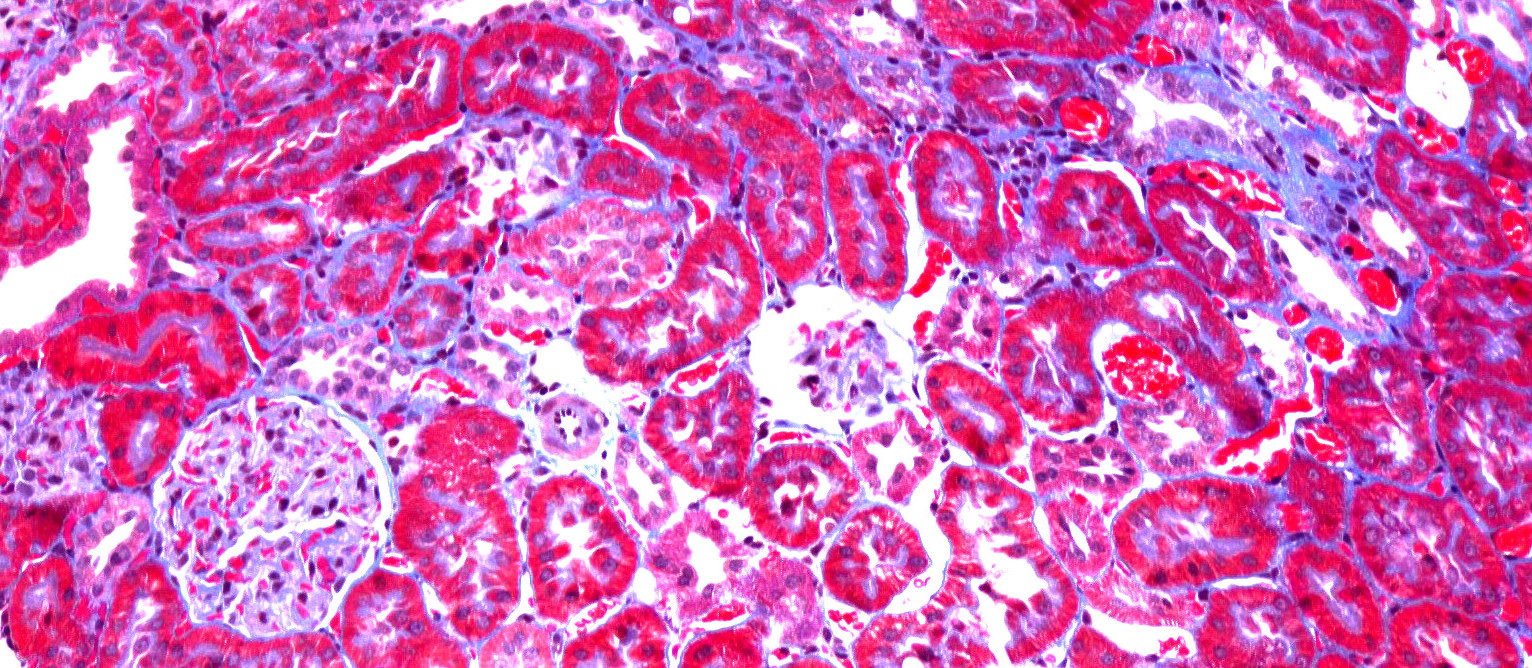

Supplement: Supplementary file 3 [file DataSheet11.ZIP › Fig 1D-masson-TSF-59/59-2.jpeg]

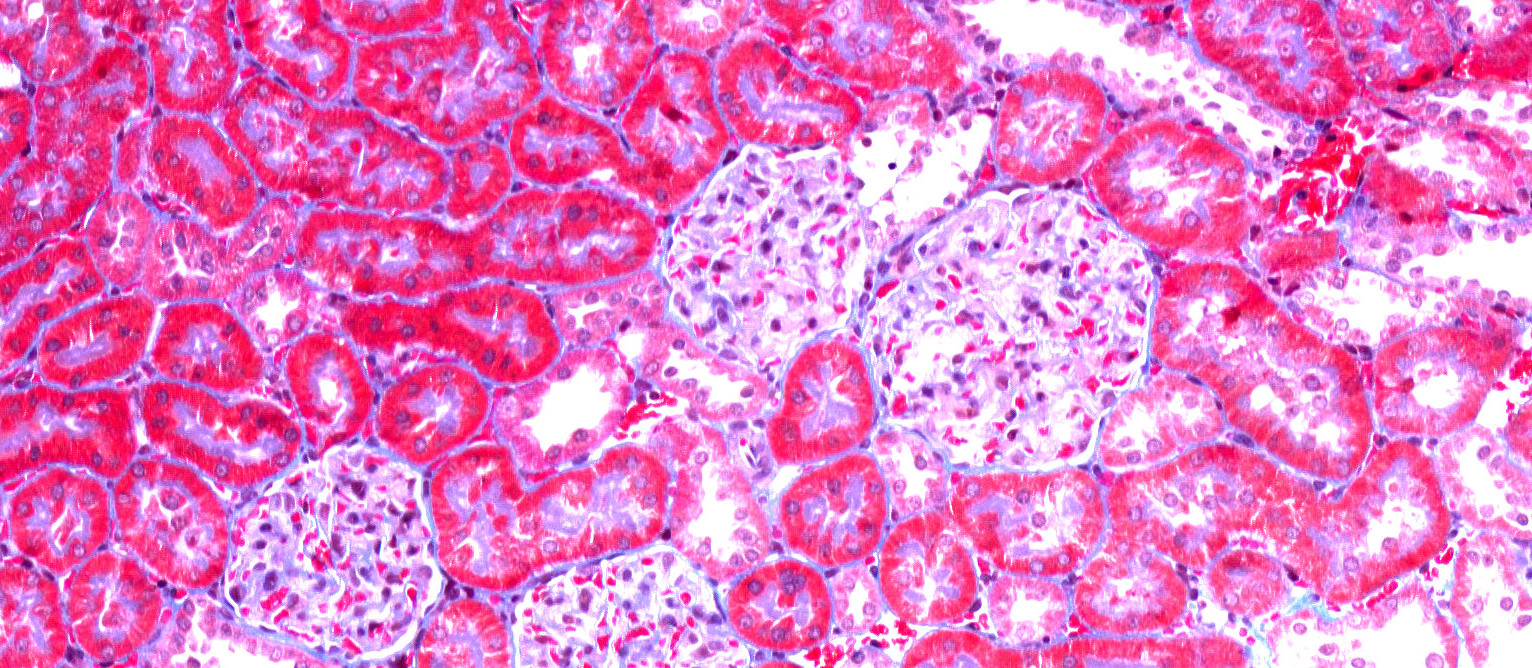

Supplement: Supplementary file 3 [file DataSheet11.ZIP › Fig 1D-masson-TSF-59/59-3.jpeg]

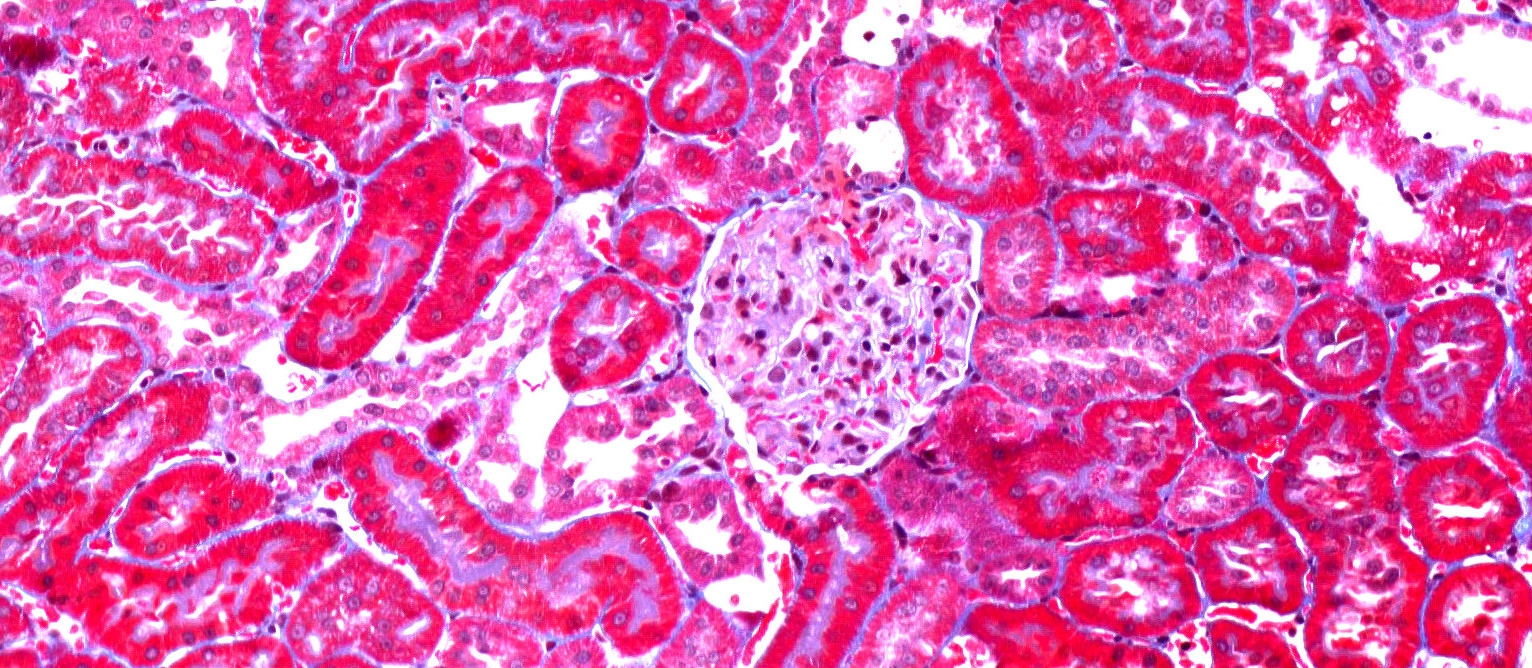

Supplement: Supplementary file 3 [file DataSheet11.ZIP › Fig 1D-masson-TSF-59/59-4.jpeg]

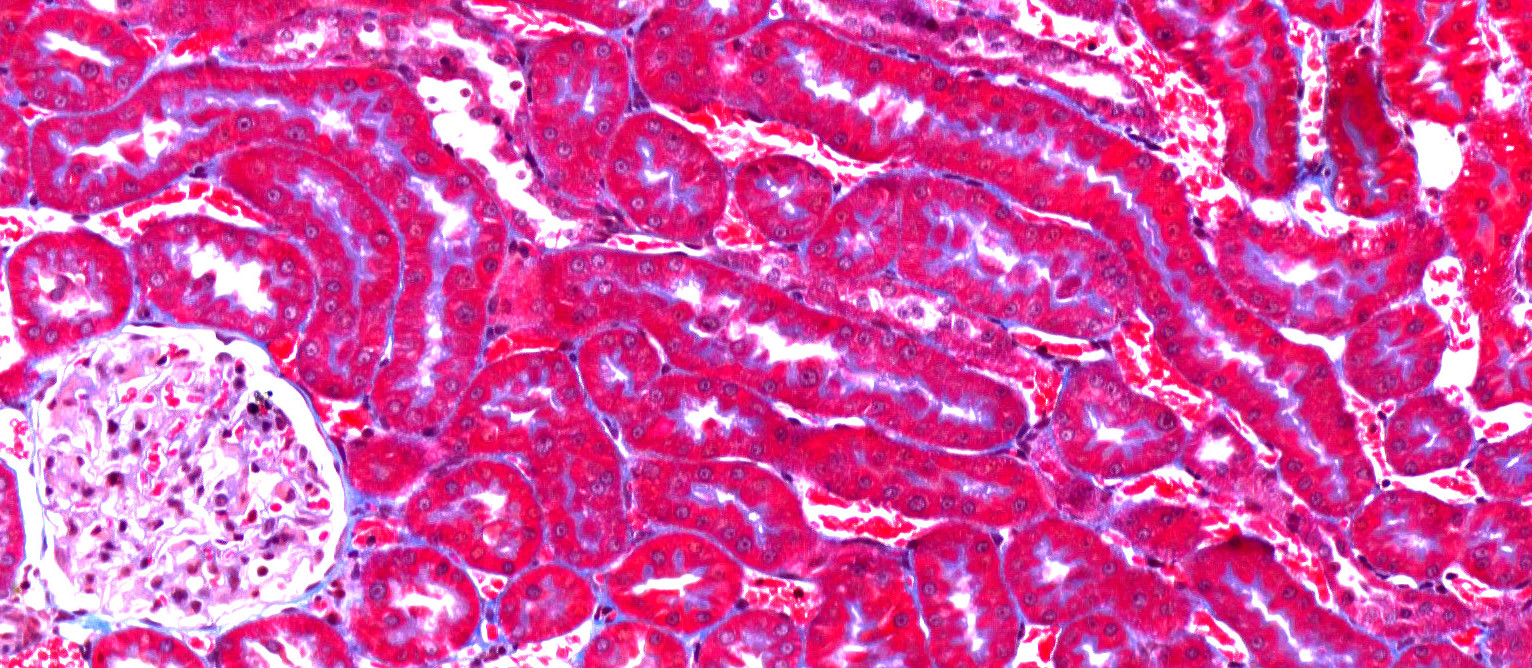

Supplement: Supplementary file 3 [file DataSheet11.ZIP › Fig 1D-masson-TSF-59/59-5.jpeg]

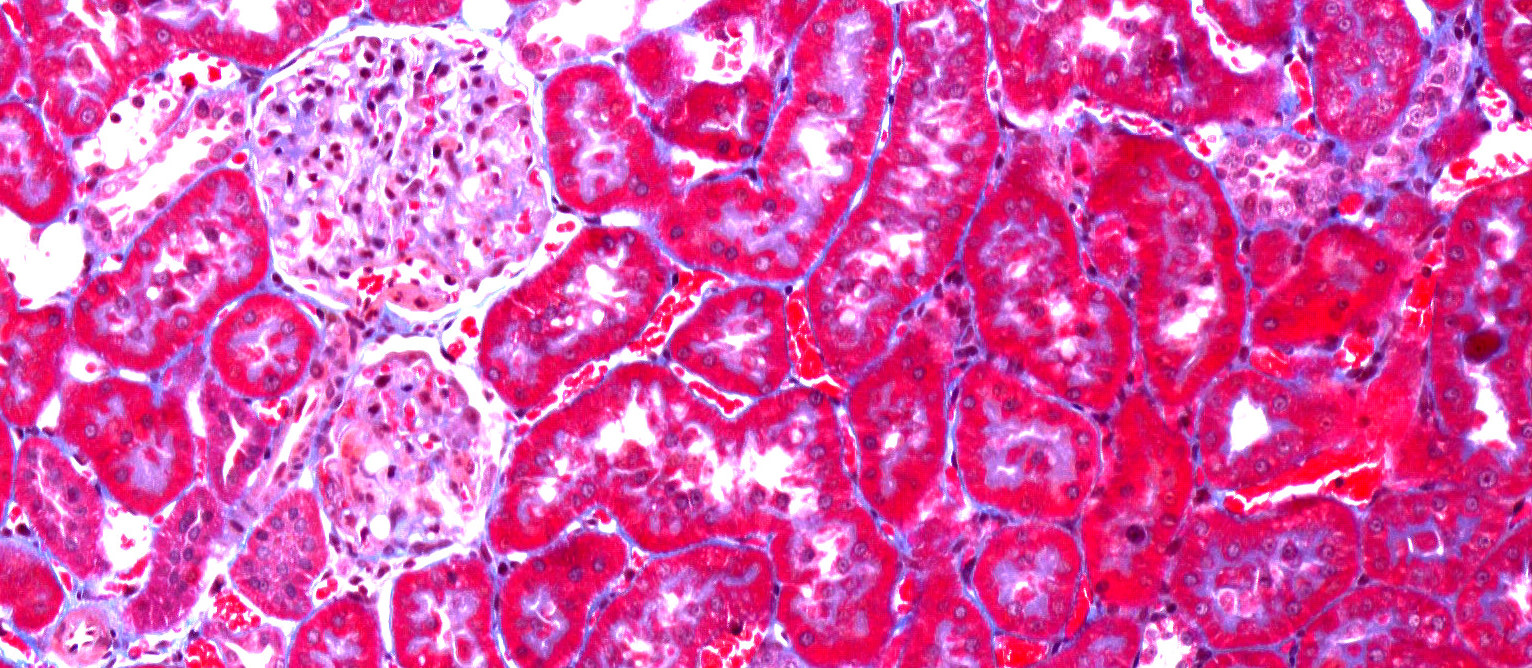

Supplement: Supplementary file 3 [file DataSheet11.ZIP › Fig 1D-masson-TSF-59/59-6.jpeg]

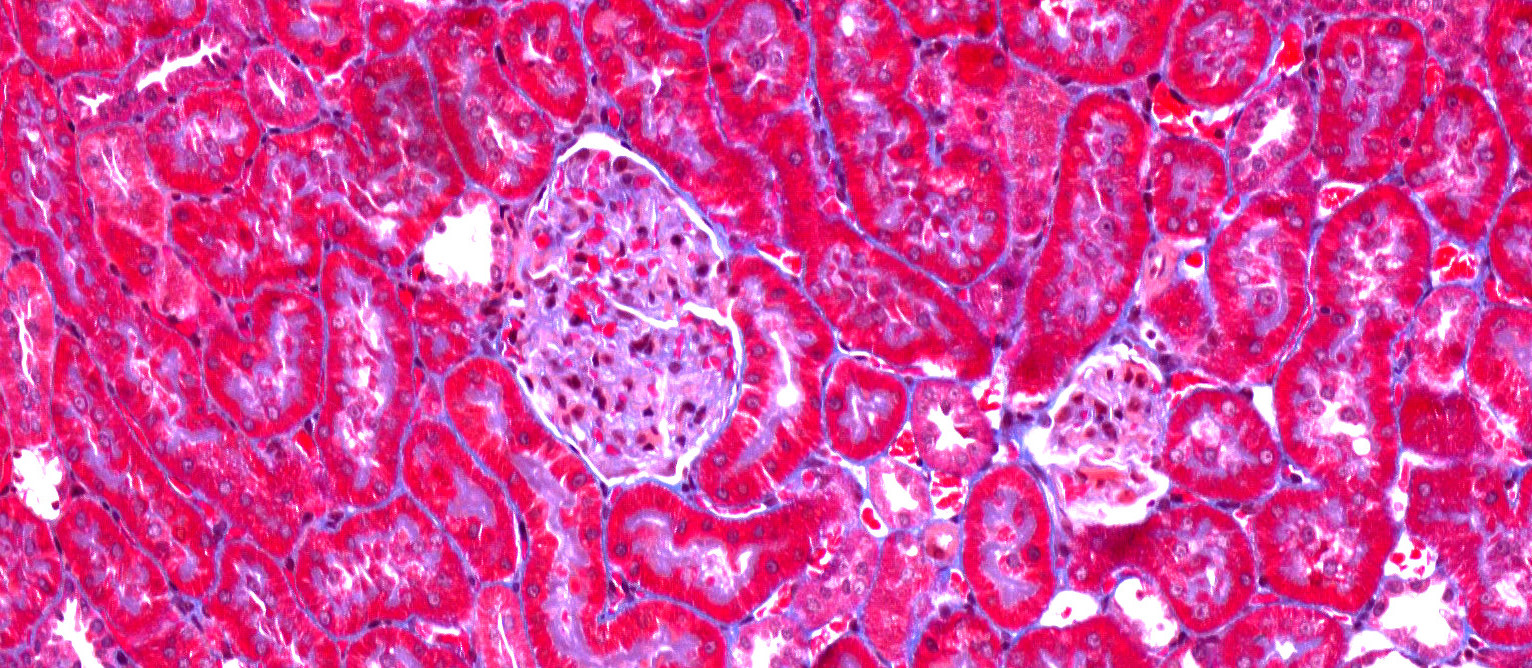

Supplement: Supplementary file 3 [file DataSheet11.ZIP › Fig 1D-masson-TSF-59/59-7.jpeg]

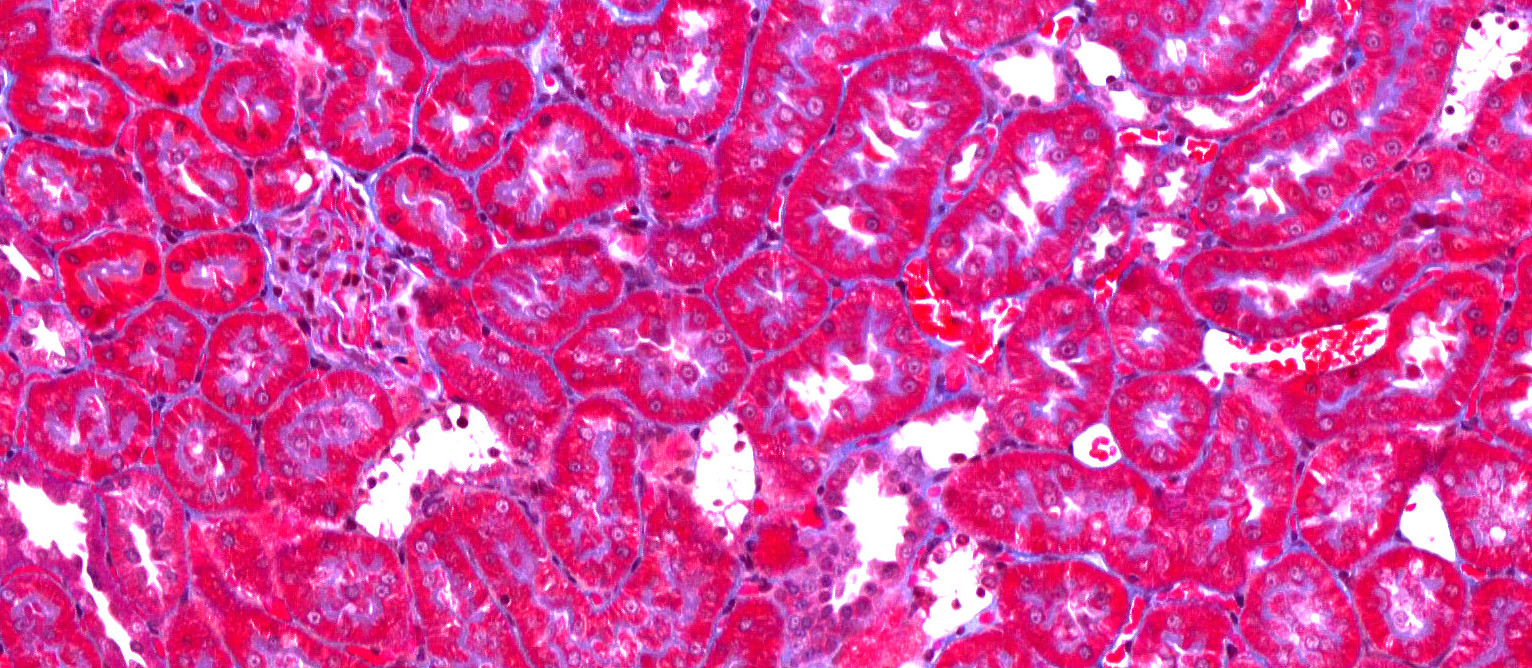

Supplement: Supplementary file 3 [file DataSheet11.ZIP › Fig 1D-masson-TSF-59/59-8.jpeg]

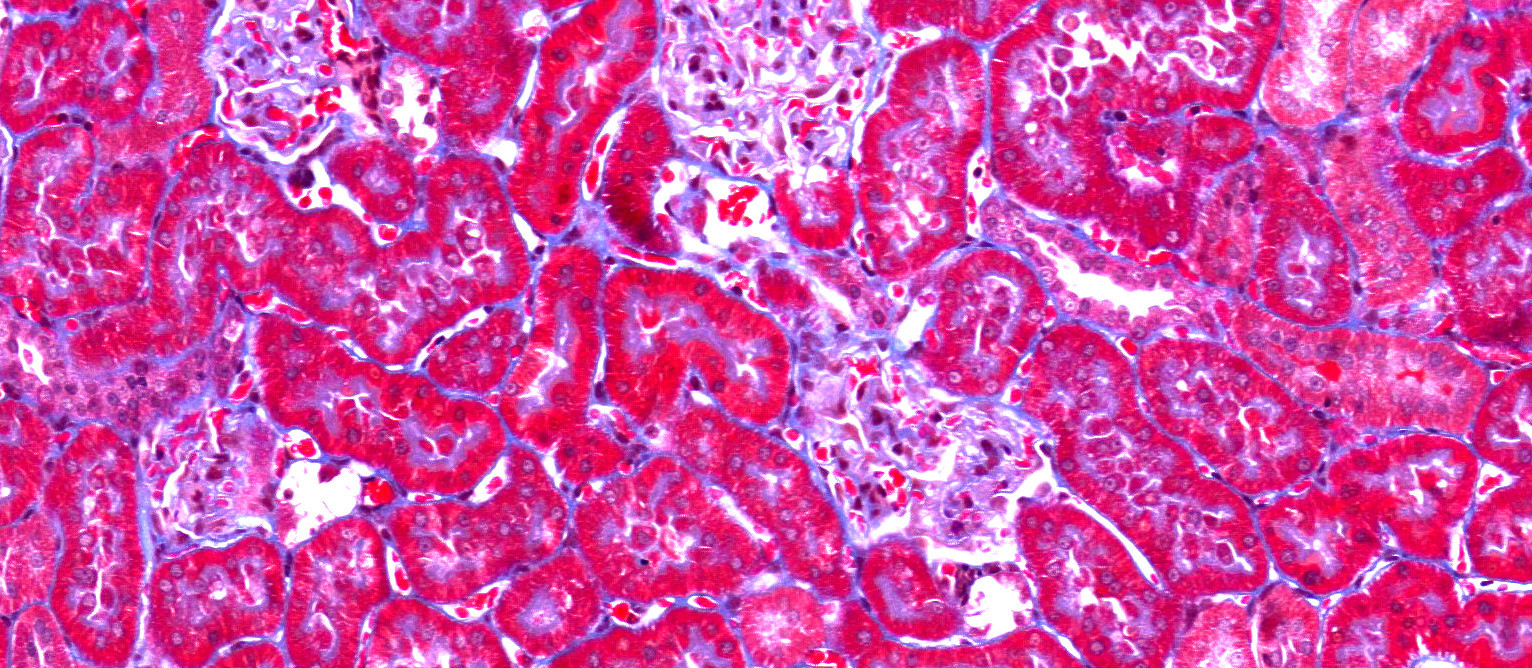

Supplement: Supplementary file 3 [file DataSheet11.ZIP › Fig 1D-masson-TSF-59/59-9.jpeg]

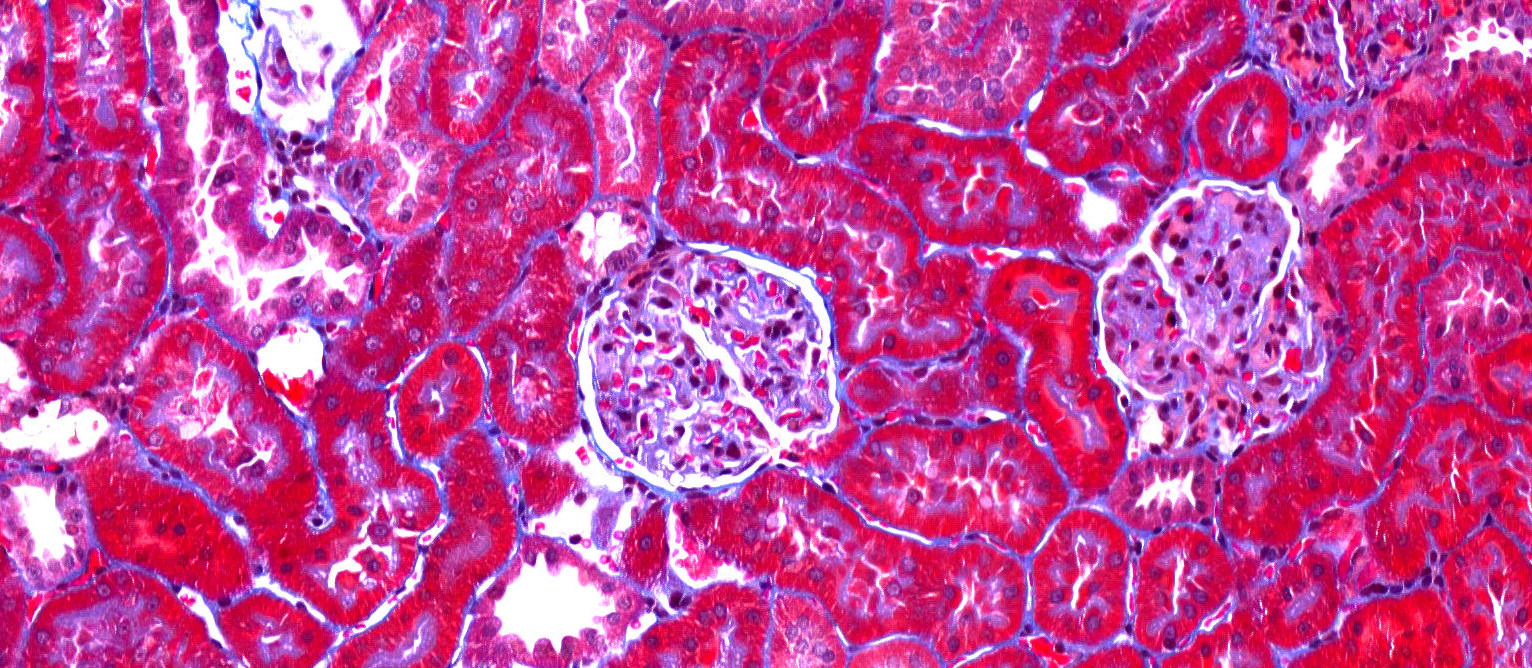

Supplement: Supplementary file 3 [file DataSheet11.ZIP › Fig 1D-masson-TSF-60/60-1.jpeg]

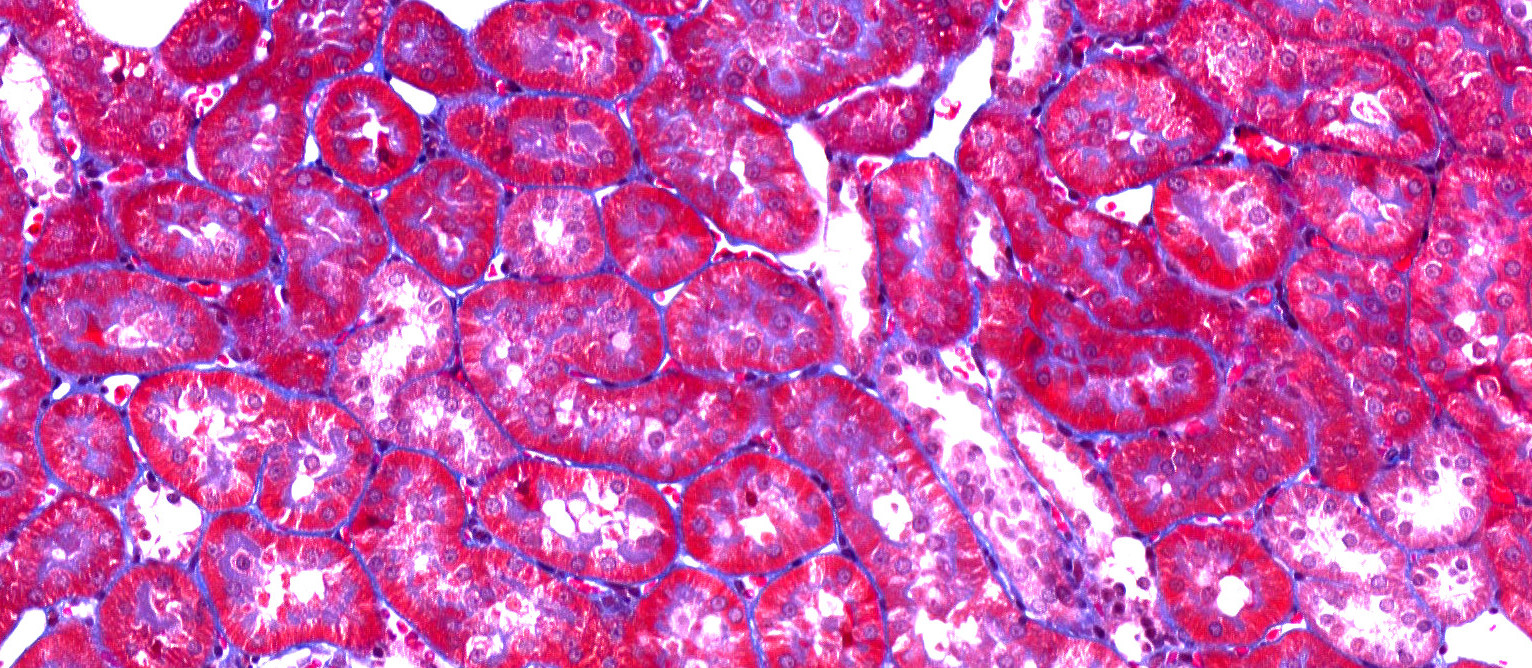

Supplement: Supplementary file 3 [file DataSheet11.ZIP › Fig 1D-masson-TSF-60/60-10.jpeg]

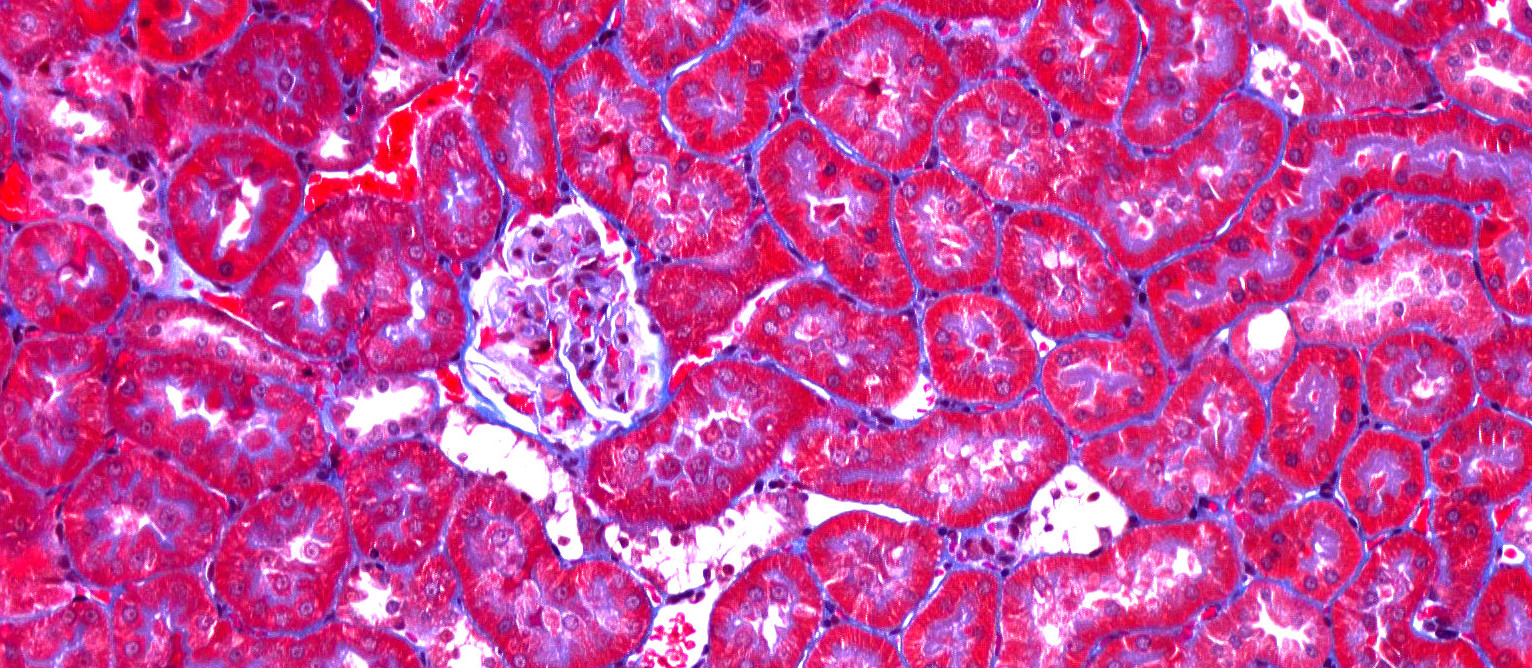

Supplement: Supplementary file 3 [file DataSheet11.ZIP › Fig 1D-masson-TSF-60/60-2.jpeg]

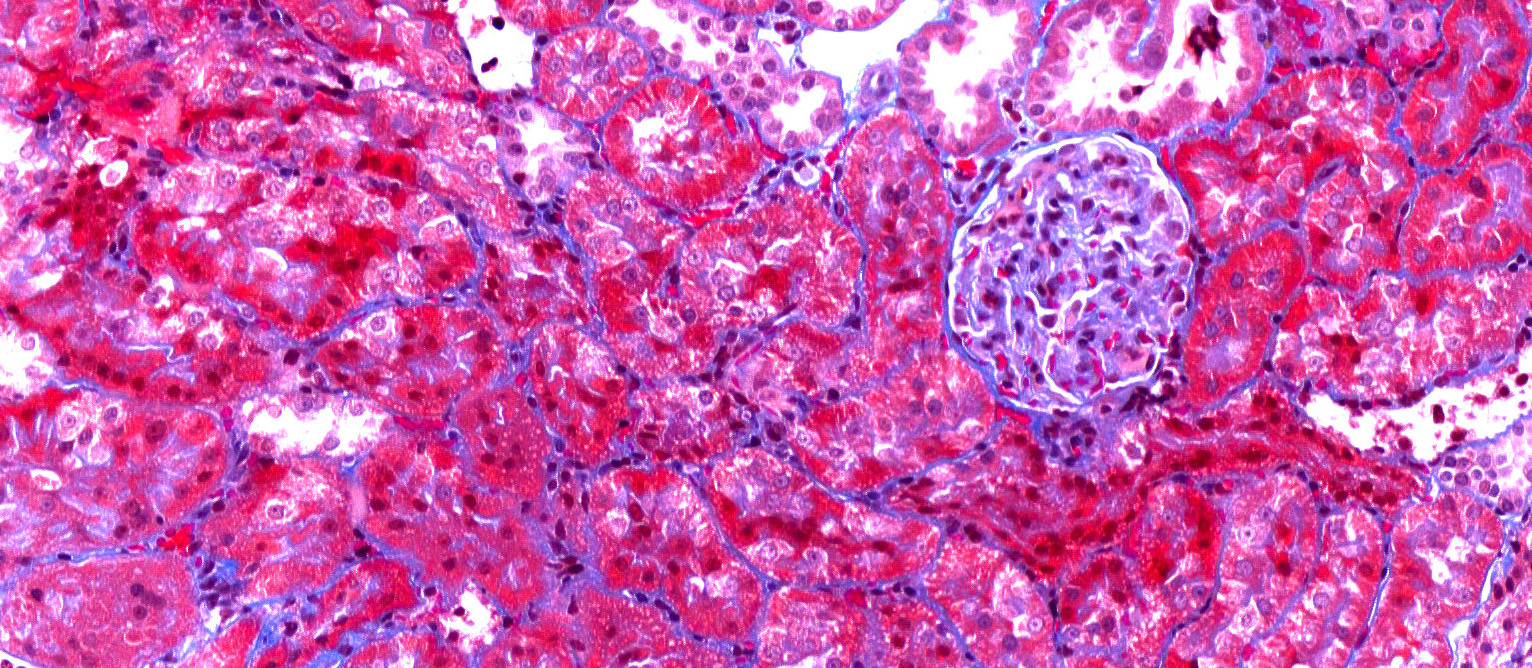

Supplement: Supplementary file 3 [file DataSheet11.ZIP › Fig 1D-masson-TSF-60/60-3.jpeg]

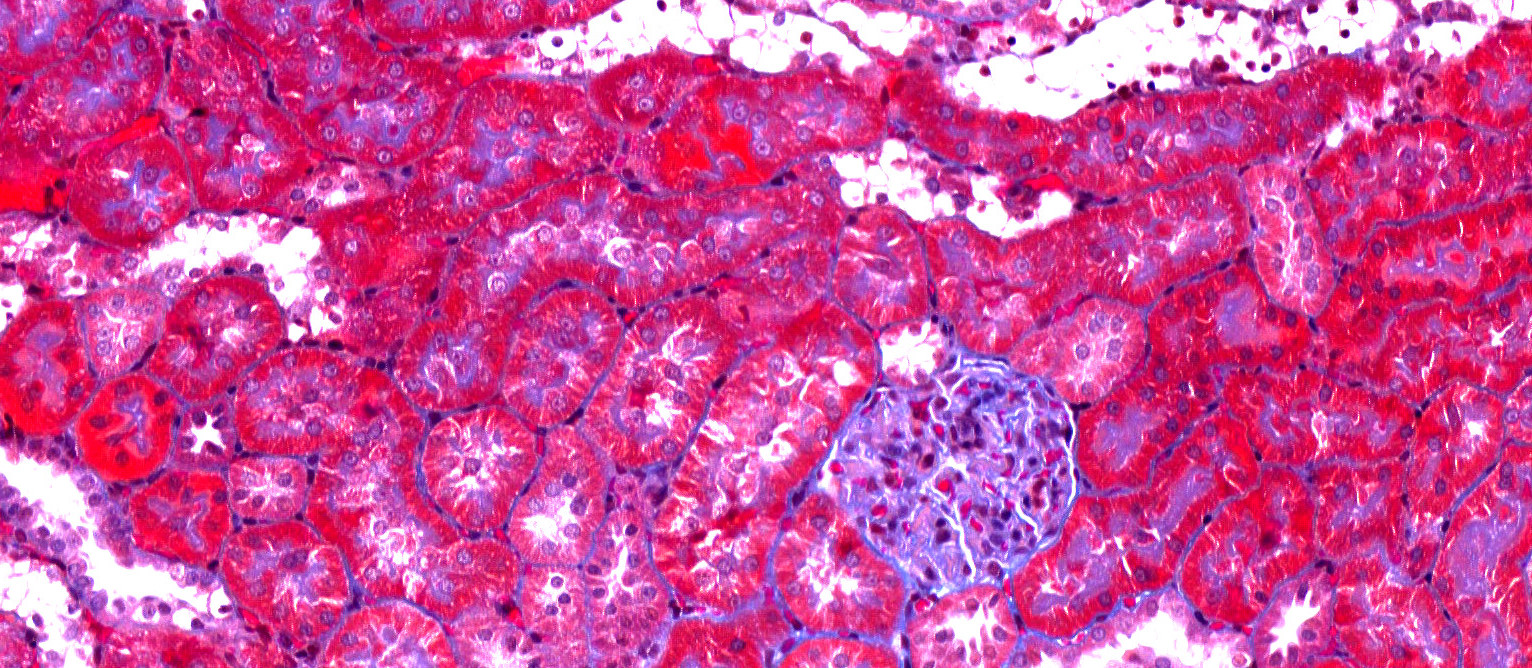

Supplement: Supplementary file 3 [file DataSheet11.ZIP › Fig 1D-masson-TSF-60/60-4.jpeg]

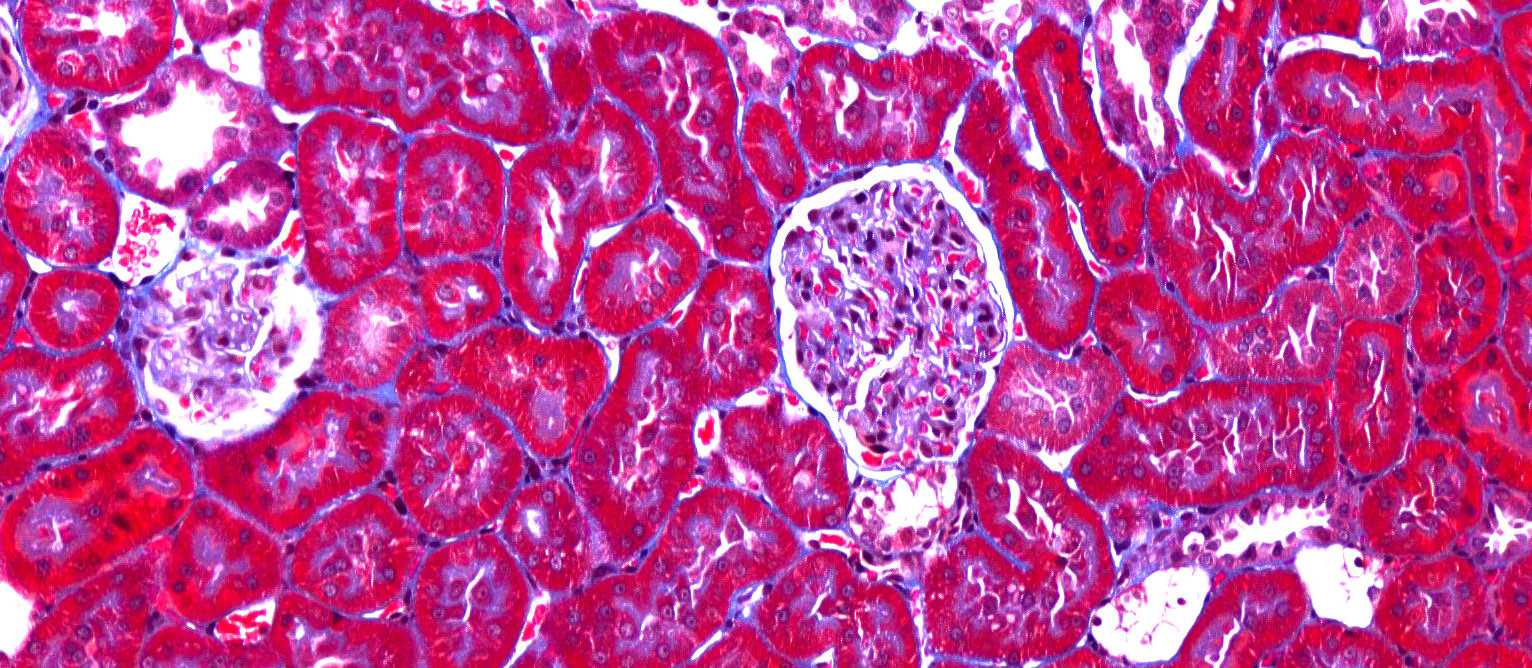

Supplement: Supplementary file 3 [file DataSheet11.ZIP › Fig 1D-masson-TSF-60/60-5.jpeg]

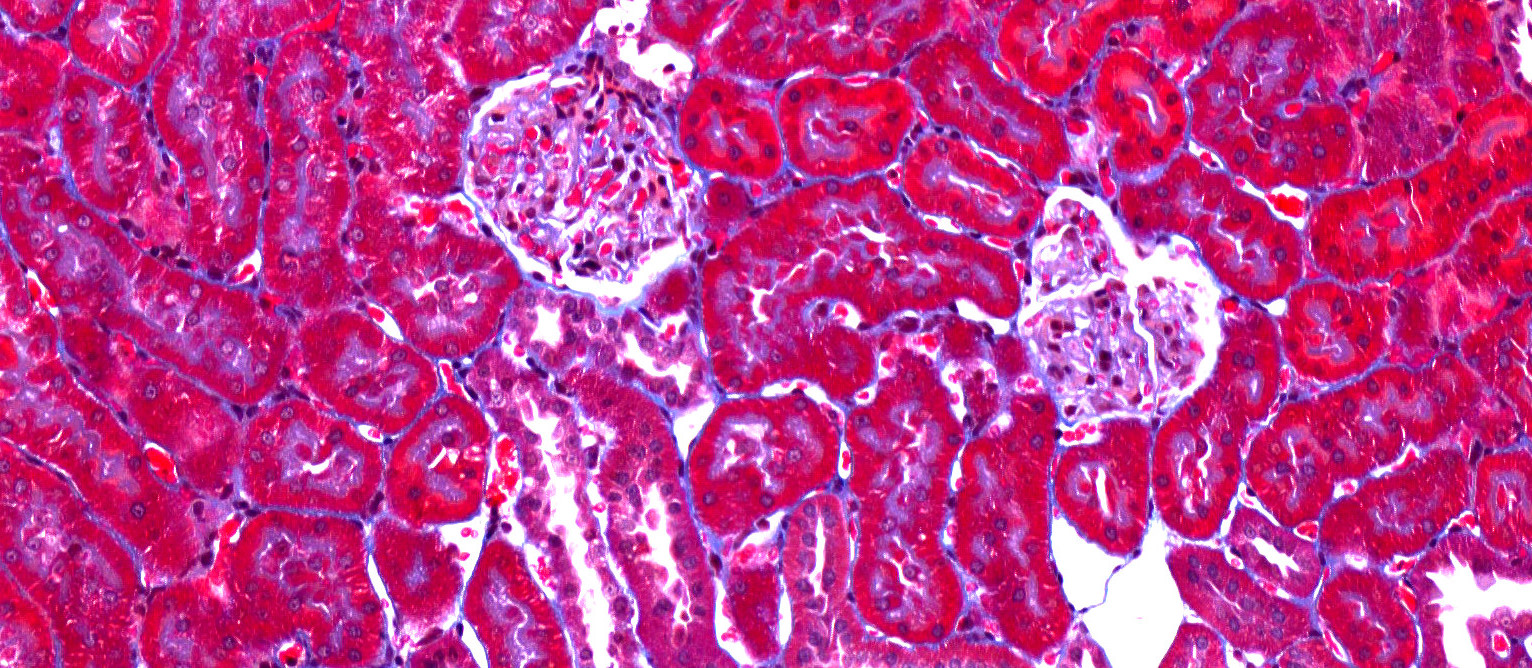

Supplement: Supplementary file 3 [file DataSheet11.ZIP › Fig 1D-masson-TSF-60/60-6.jpeg]

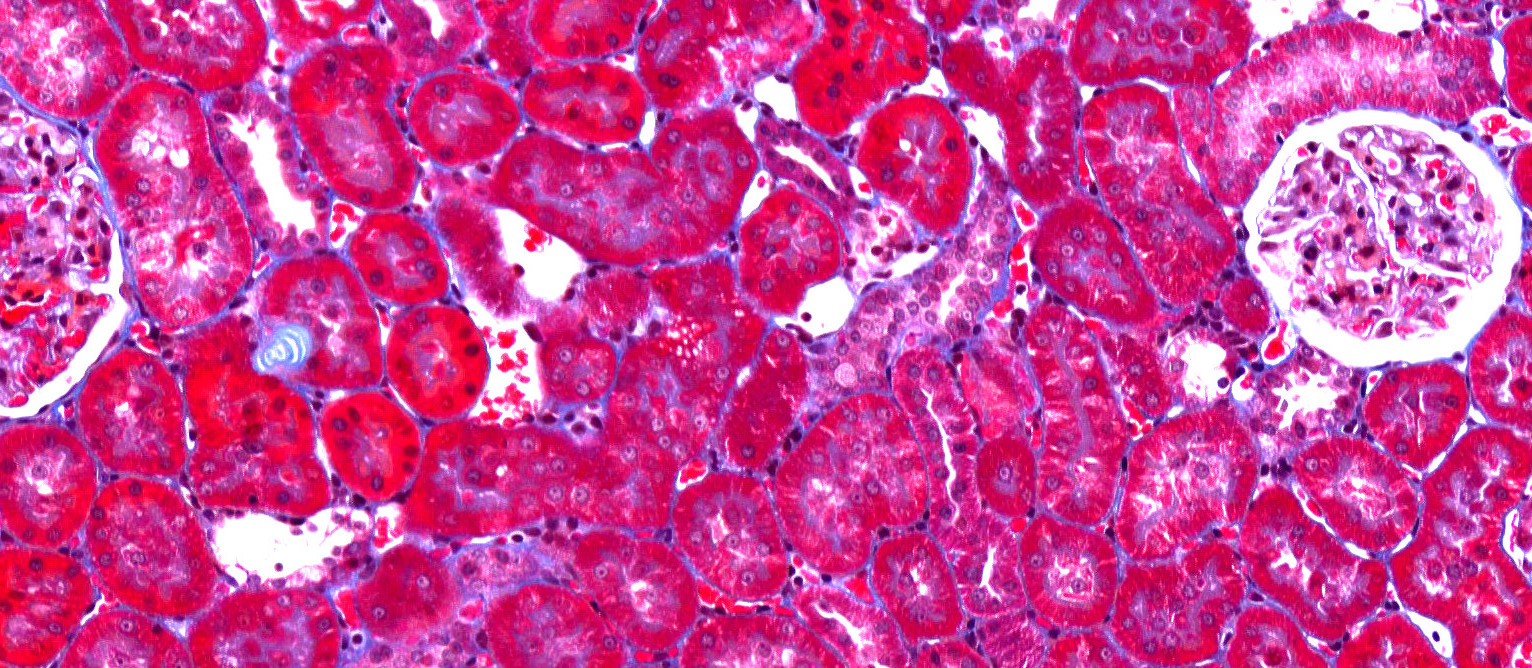

Supplement: Supplementary file 3 [file DataSheet11.ZIP › Fig 1D-masson-TSF-60/60-7.jpeg]

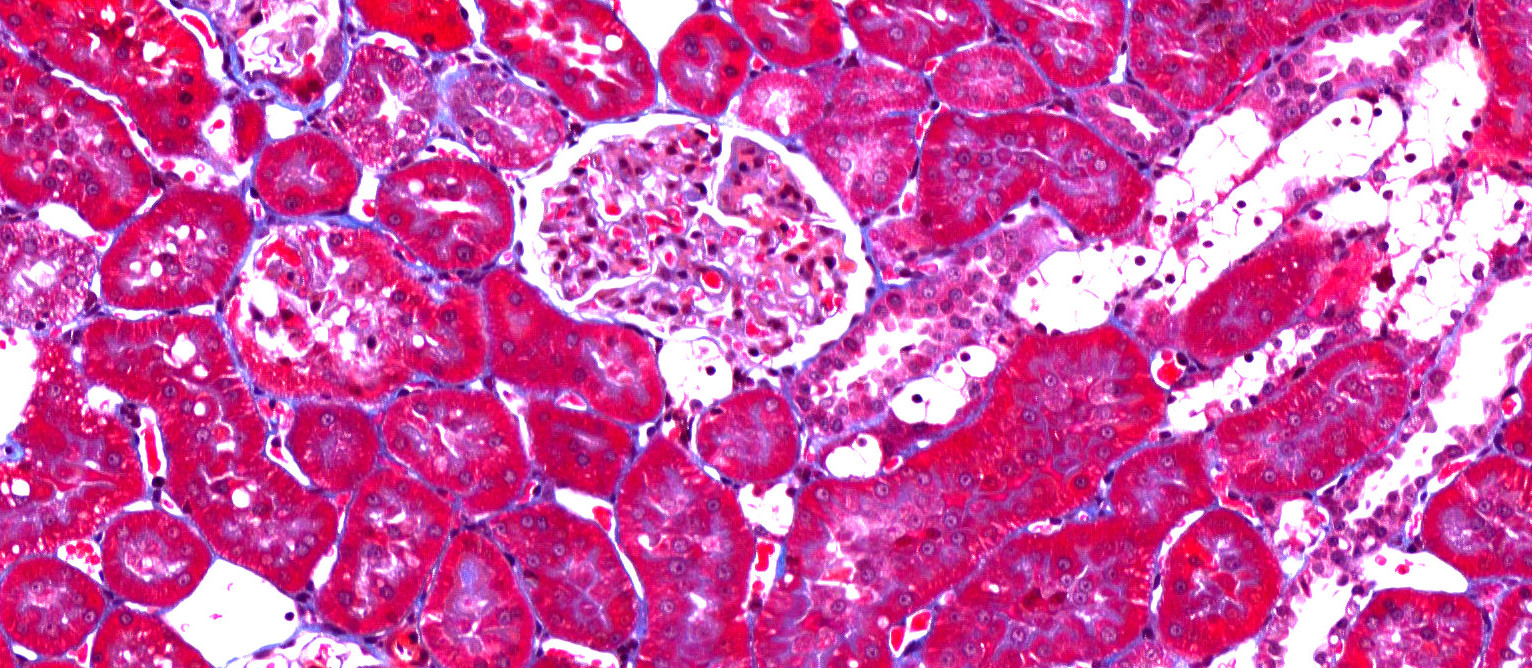

Supplement: Supplementary file 3 [file DataSheet11.ZIP › Fig 1D-masson-TSF-60/60-8.jpeg]

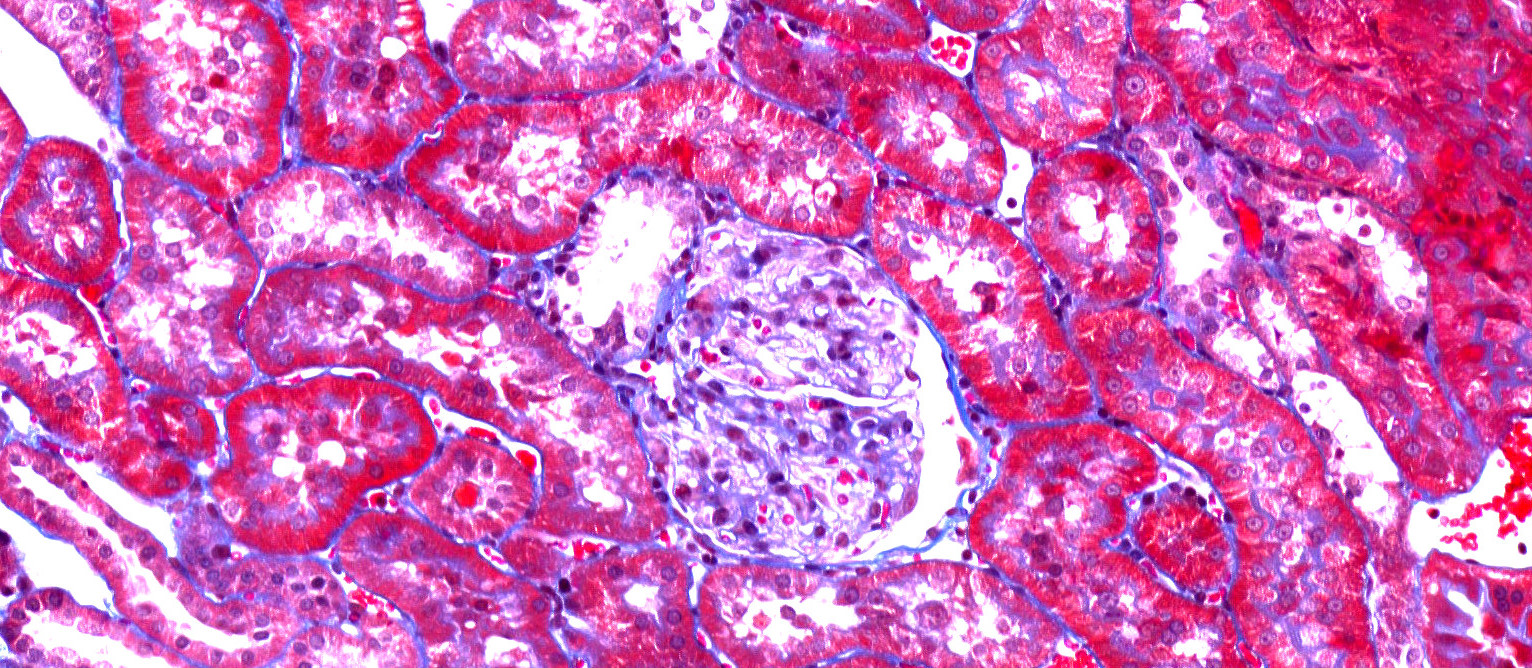

Supplement: Supplementary file 3 [file DataSheet11.ZIP › Fig 1D-masson-TSF-60/60-9.jpeg]

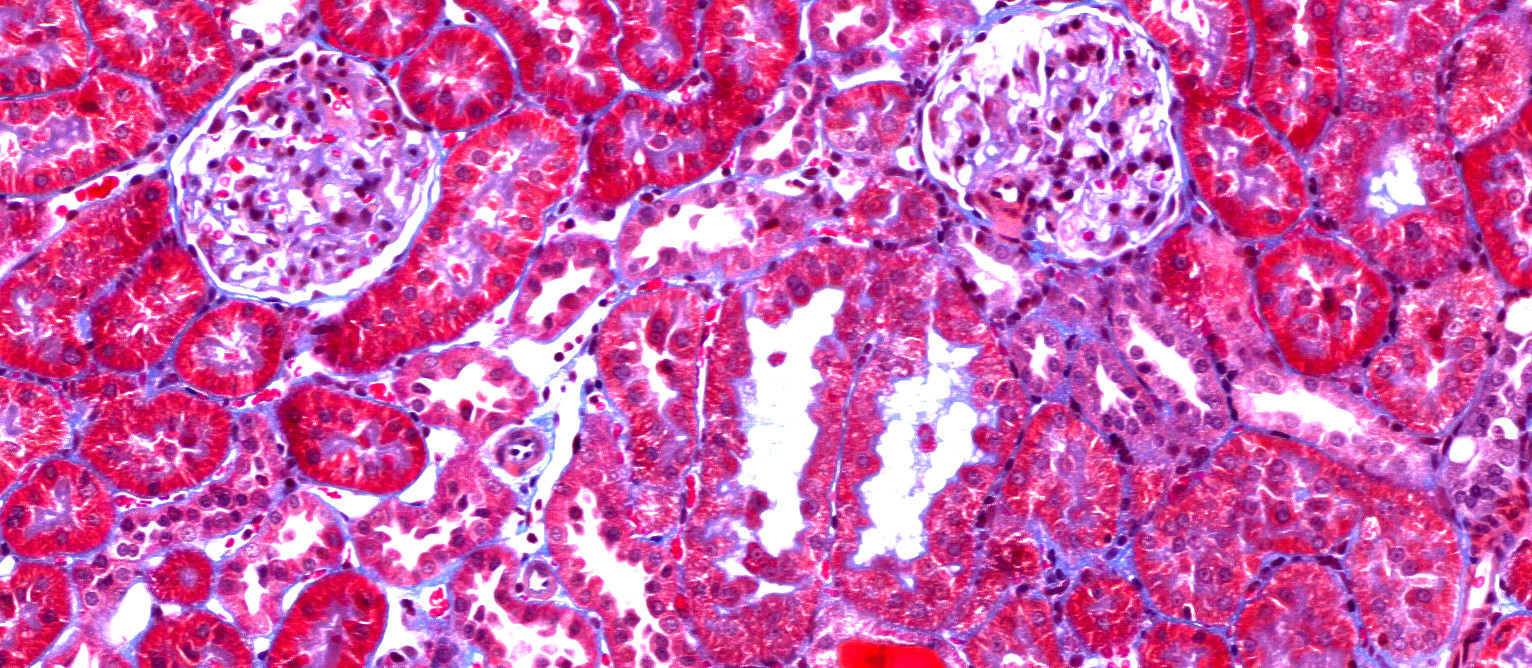

Supplement: Supplementary file 3 [file DataSheet11.ZIP › Fig 1D-masson-TSF-61/61-1.jpeg]

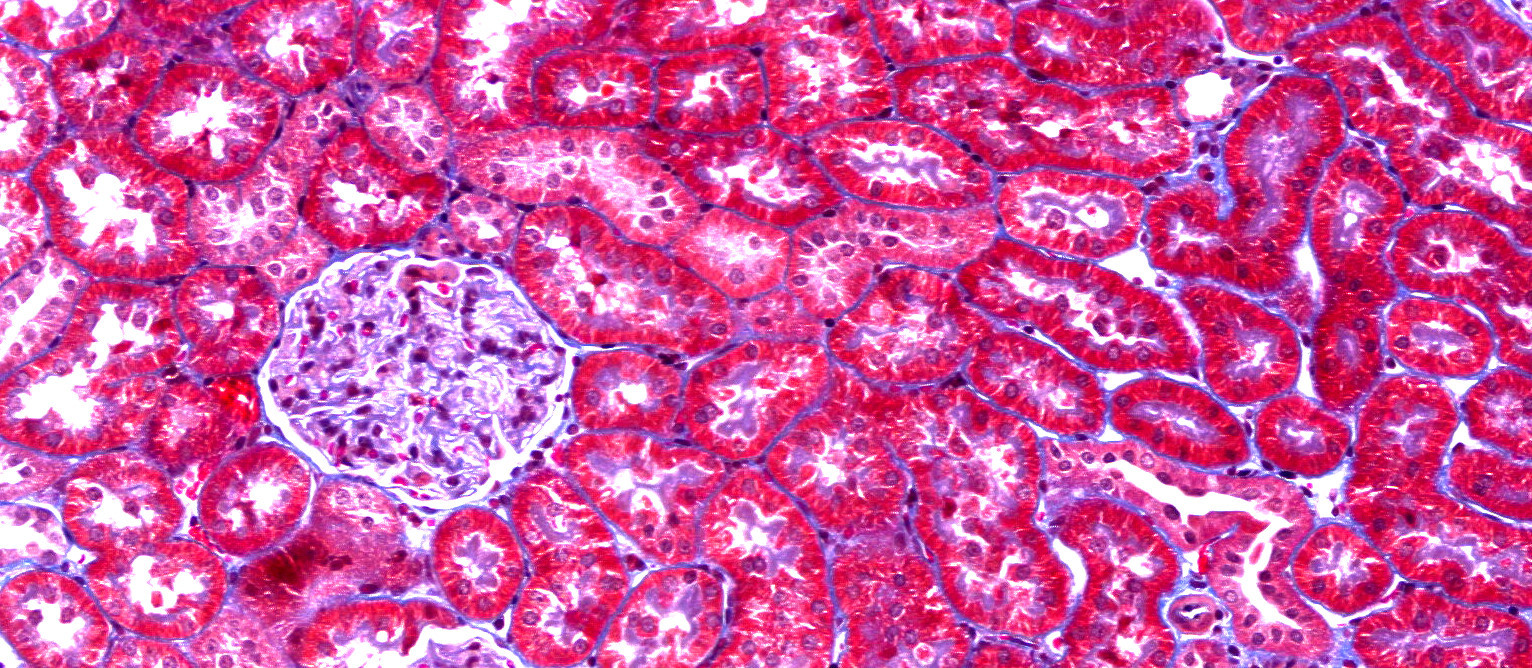

Supplement: Supplementary file 3 [file DataSheet11.ZIP › Fig 1D-masson-TSF-61/61-10.jpeg]

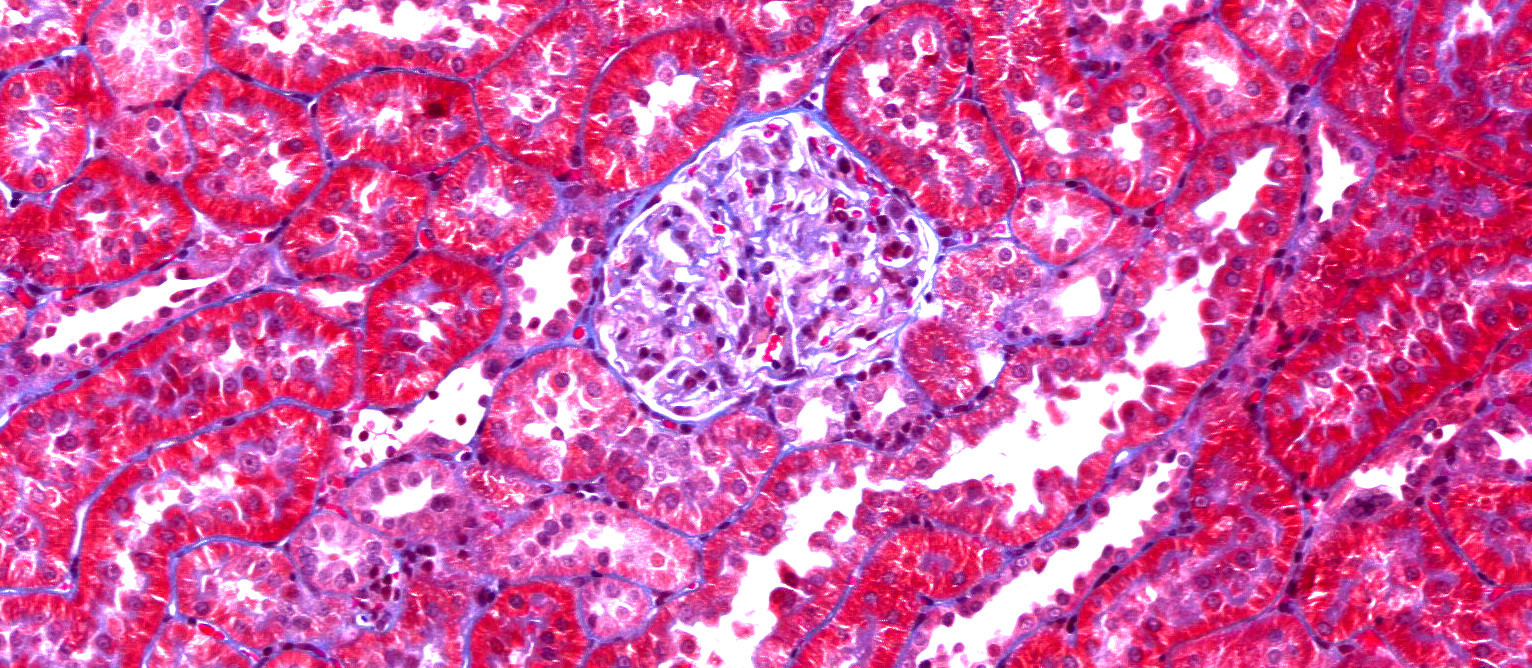

Supplement: Supplementary file 3 [file DataSheet11.ZIP › Fig 1D-masson-TSF-61/61-2.jpeg]

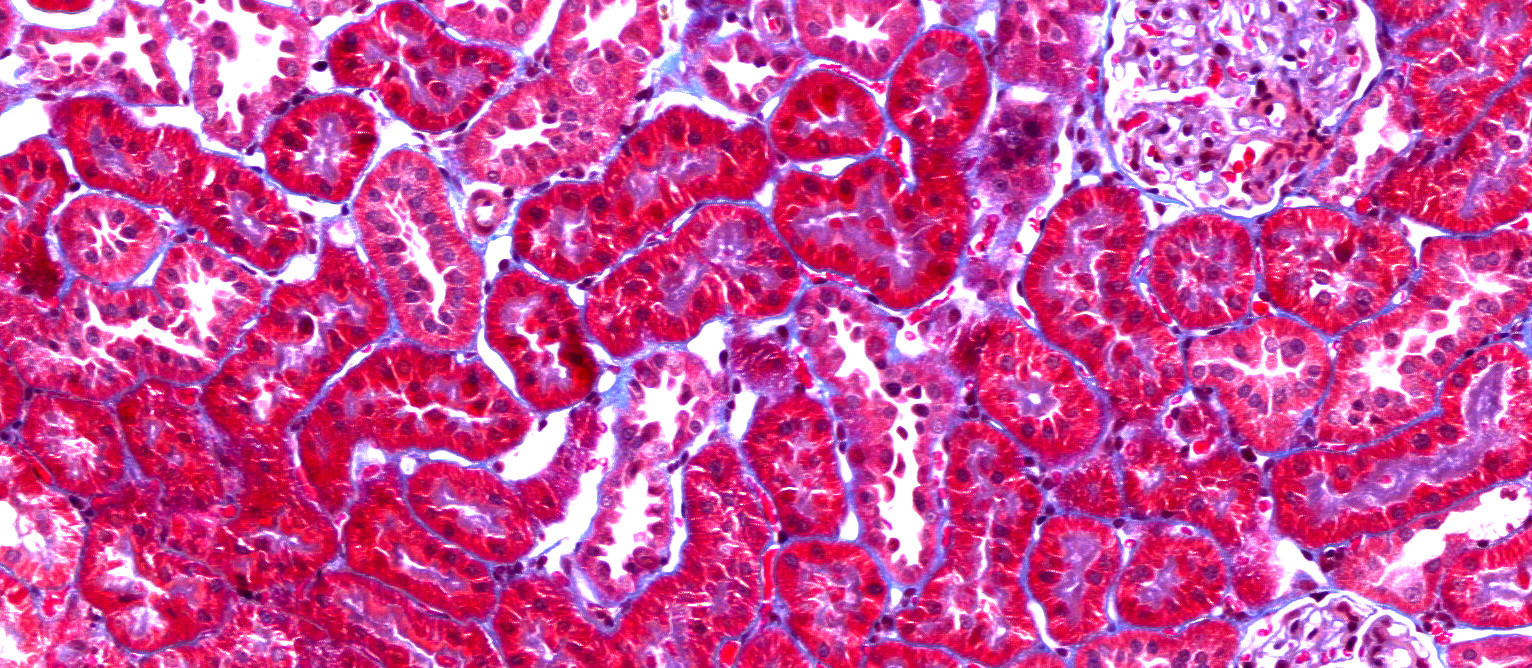

Supplement: Supplementary file 3 [file DataSheet11.ZIP › Fig 1D-masson-TSF-61/61-3.jpeg]

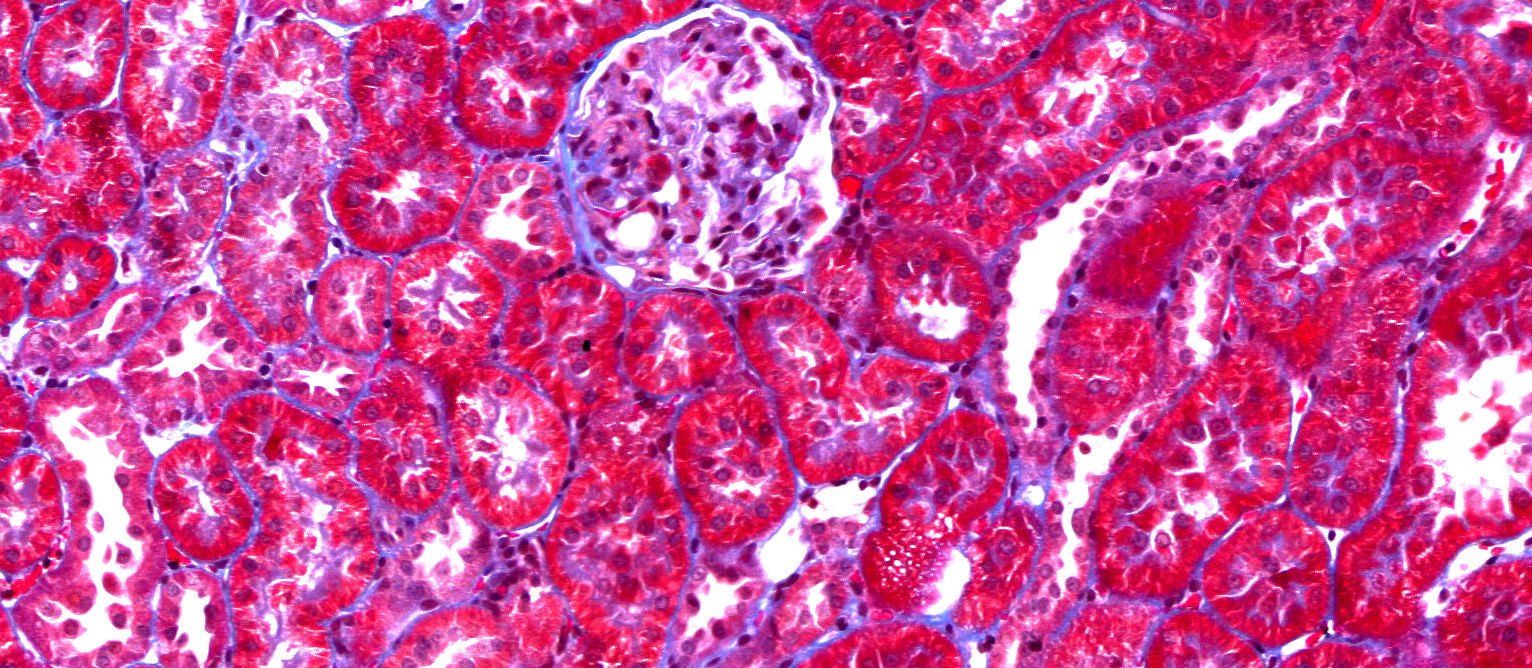

Supplement: Supplementary file 3 [file DataSheet11.ZIP › Fig 1D-masson-TSF-61/61-4.jpeg]

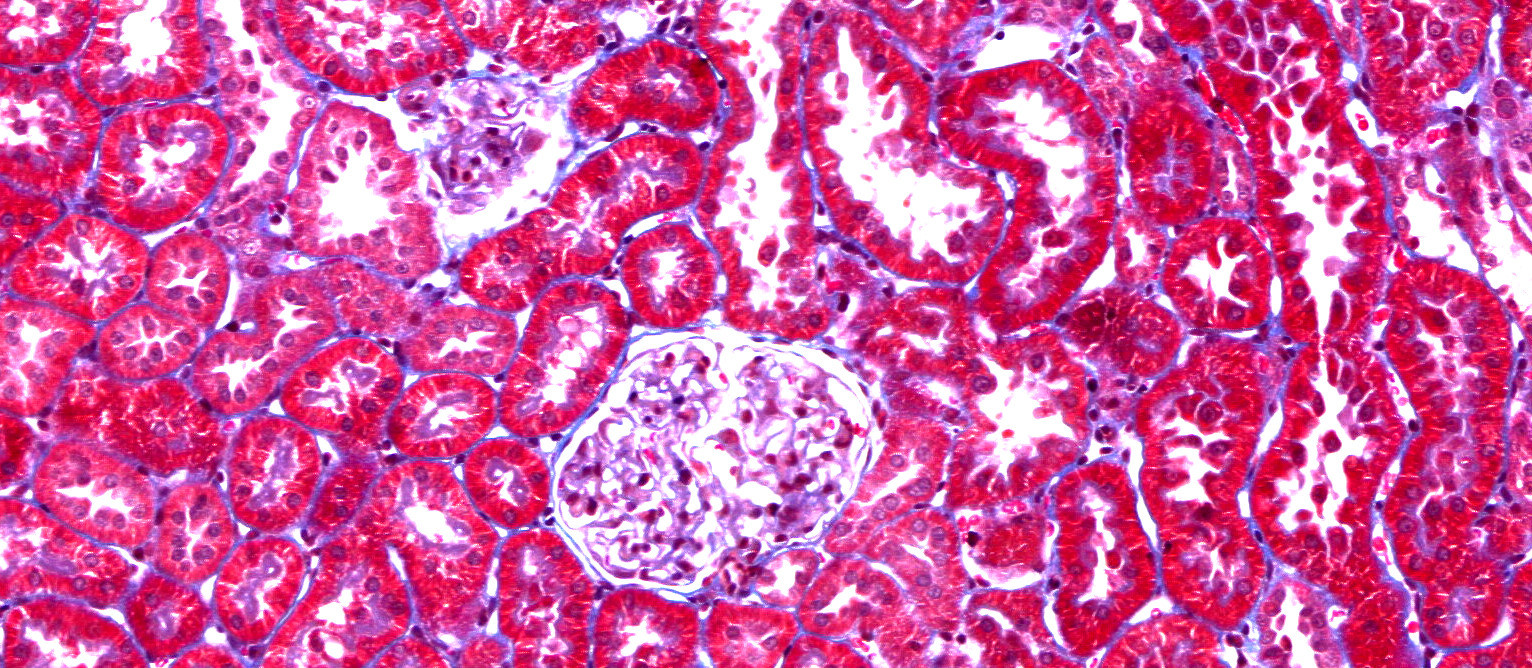

Supplement: Supplementary file 3 [file DataSheet11.ZIP › Fig 1D-masson-TSF-61/61-5.jpeg]
