# Supplementary figures and images for: Characteristics of Serum Metabolites and Gut Microbiota in Diabetic Kidney Disease (part 2 of 13)
Source: Front Pharmacol. 2022 Apr 14;13:872988. doi: 10.3389/fphar.2022.872988 (PMC9084235; doi:10.3389/fphar.2022.872988)

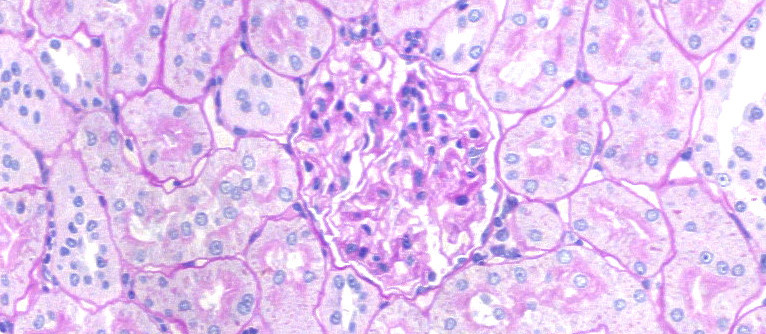

Supplement: Supplementary file 2 [file DataSheet14.ZIP › sham/Fig 1D-PAS-sham-2/2-11.jpeg]

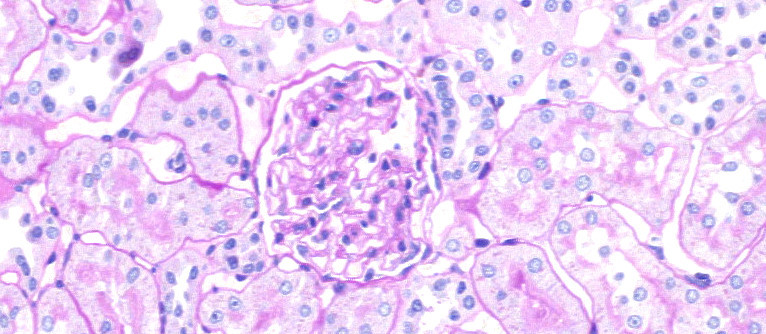

Supplement: Supplementary file 2 [file DataSheet14.ZIP › sham/Fig 1D-PAS-sham-2/2-12.jpeg]

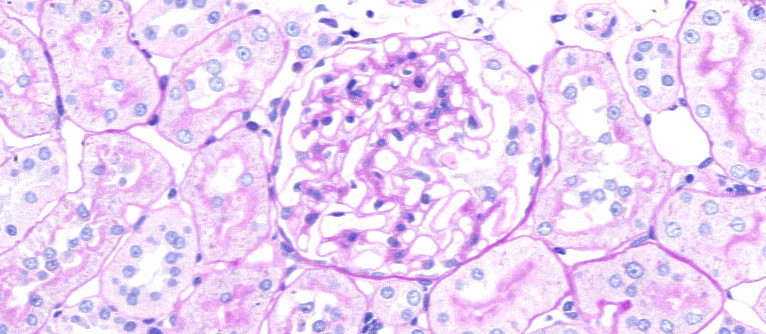

Supplement: Supplementary file 2 [file DataSheet14.ZIP › sham/Fig 1D-PAS-sham-2/2-13.jpeg]

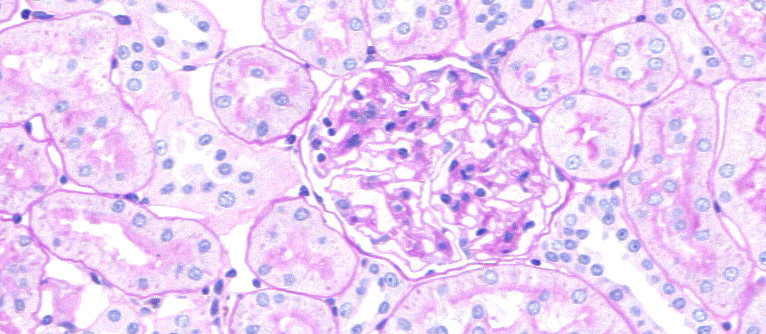

Supplement: Supplementary file 2 [file DataSheet14.ZIP › sham/Fig 1D-PAS-sham-2/2-14.jpeg]

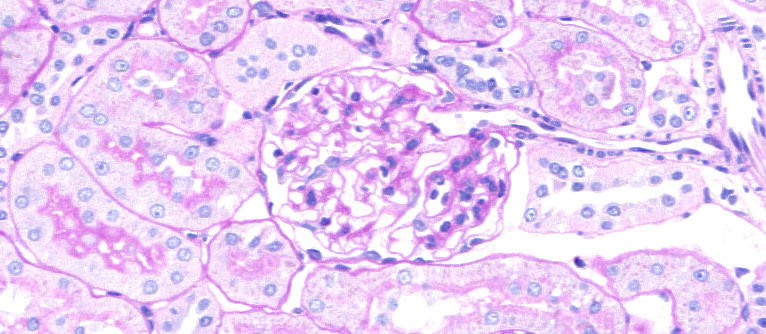

Supplement: Supplementary file 2 [file DataSheet14.ZIP › sham/Fig 1D-PAS-sham-2/2-15.jpeg]

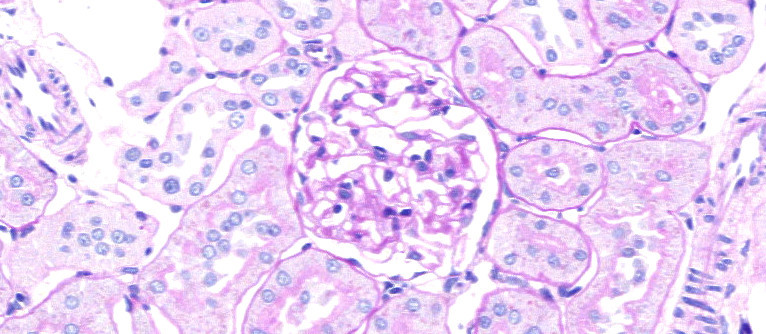

Supplement: Supplementary file 2 [file DataSheet14.ZIP › sham/Fig 1D-PAS-sham-2/2-16.jpeg]

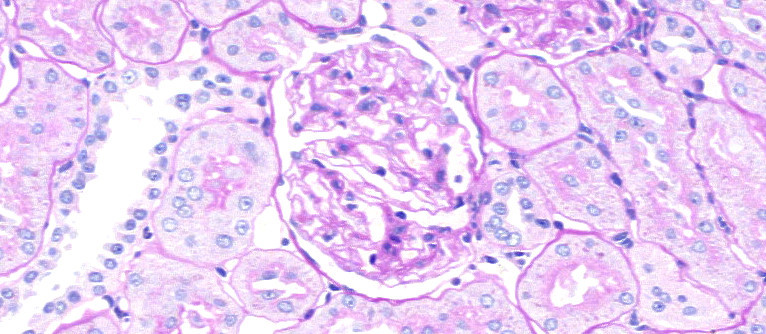

Supplement: Supplementary file 2 [file DataSheet14.ZIP › sham/Fig 1D-PAS-sham-2/2-17.jpeg]

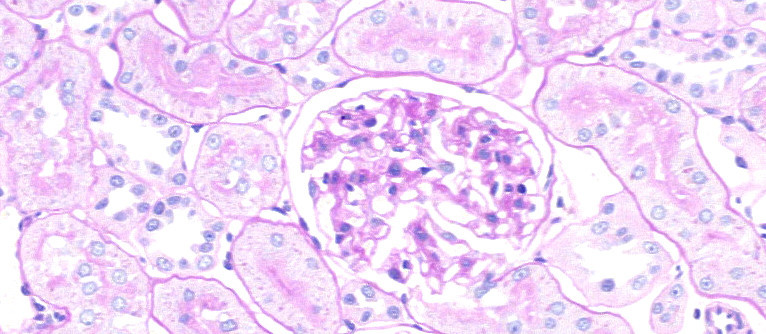

Supplement: Supplementary file 2 [file DataSheet14.ZIP › sham/Fig 1D-PAS-sham-2/2-18.jpeg]

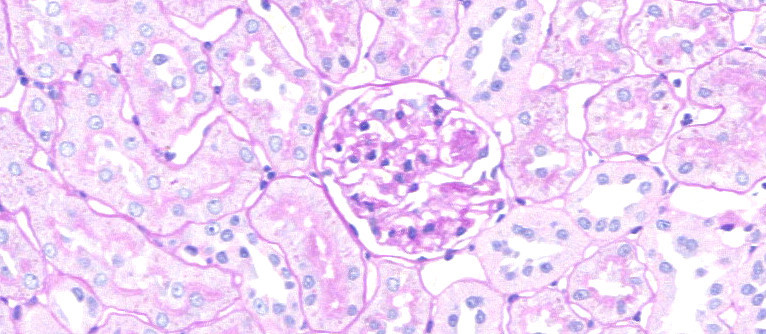

Supplement: Supplementary file 2 [file DataSheet14.ZIP › sham/Fig 1D-PAS-sham-2/2-19.jpeg]

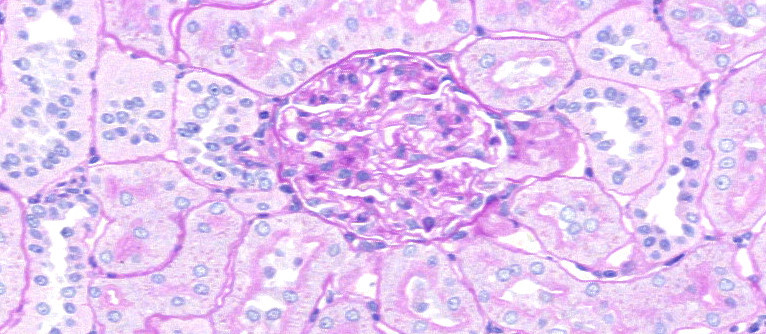

Supplement: Supplementary file 2 [file DataSheet14.ZIP › sham/Fig 1D-PAS-sham-2/2-2.jpeg]

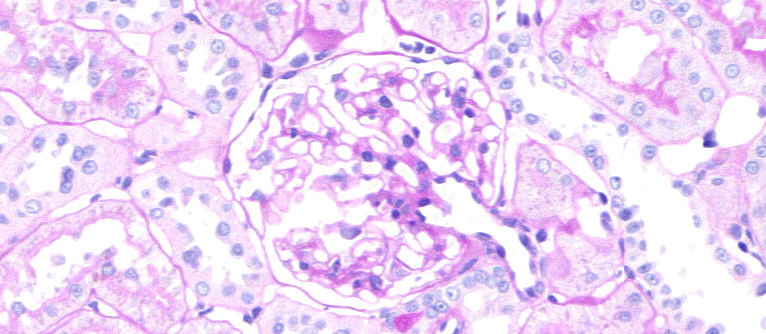

Supplement: Supplementary file 2 [file DataSheet14.ZIP › sham/Fig 1D-PAS-sham-2/2-20.jpeg]

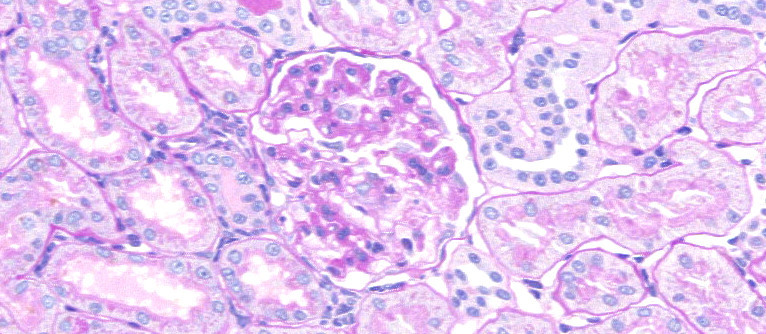

Supplement: Supplementary file 2 [file DataSheet14.ZIP › sham/Fig 1D-PAS-sham-2/2-3.jpeg]

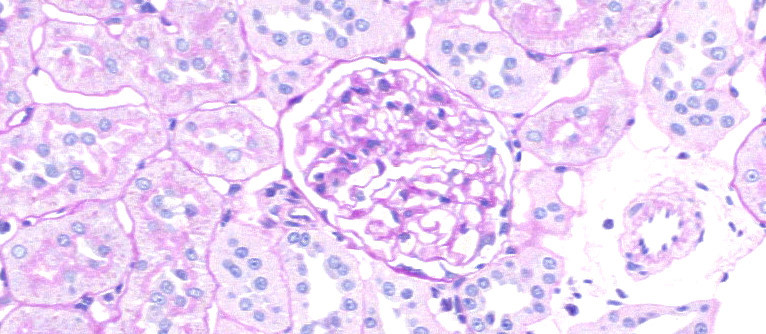

Supplement: Supplementary file 2 [file DataSheet14.ZIP › sham/Fig 1D-PAS-sham-2/2-4.jpeg]

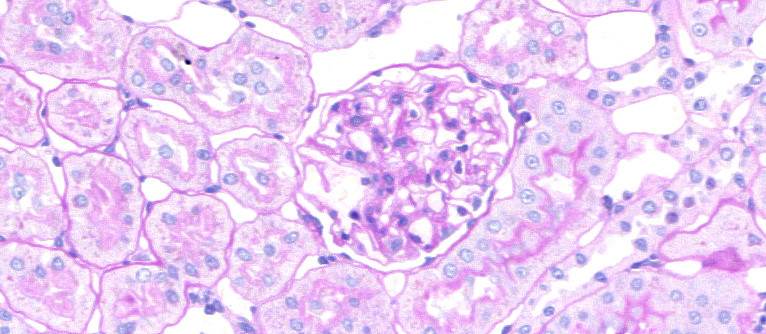

Supplement: Supplementary file 2 [file DataSheet14.ZIP › sham/Fig 1D-PAS-sham-2/2-5.jpeg]

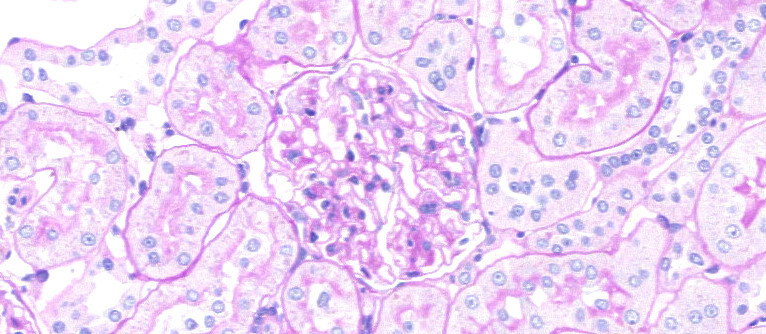

Supplement: Supplementary file 2 [file DataSheet14.ZIP › sham/Fig 1D-PAS-sham-2/2-6.jpeg]

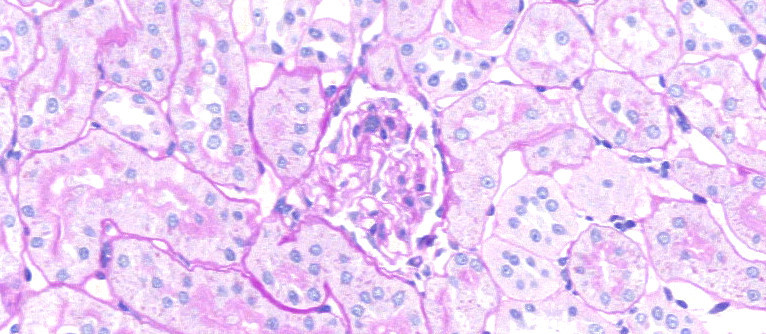

Supplement: Supplementary file 2 [file DataSheet14.ZIP › sham/Fig 1D-PAS-sham-2/2-7.jpeg]

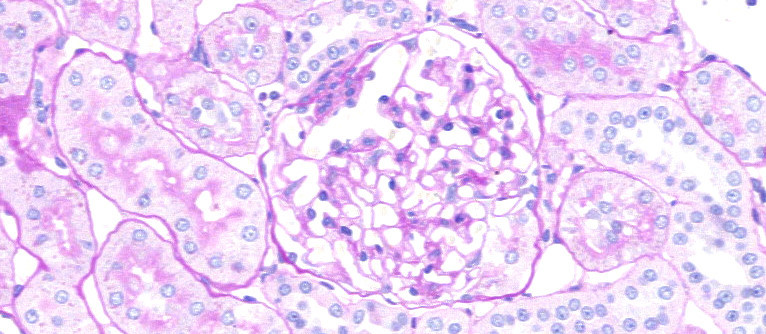

Supplement: Supplementary file 2 [file DataSheet14.ZIP › sham/Fig 1D-PAS-sham-2/2-8.jpeg]

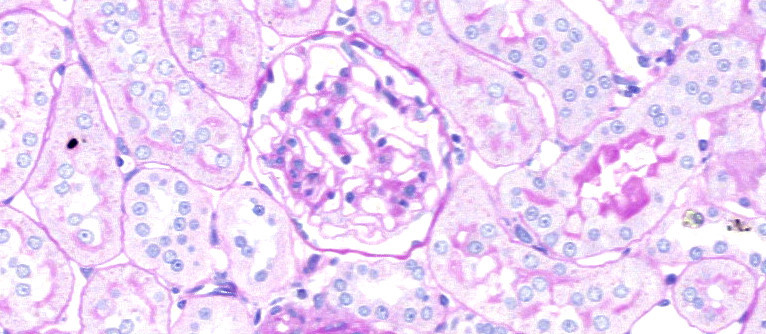

Supplement: Supplementary file 2 [file DataSheet14.ZIP › sham/Fig 1D-PAS-sham-2/2-9.jpeg]

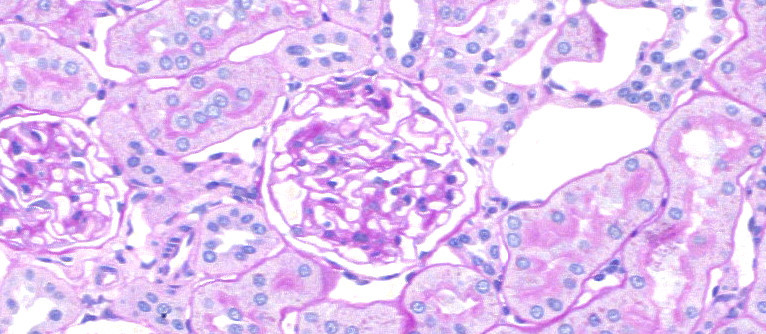

Supplement: Supplementary file 2 [file DataSheet14.ZIP › sham/Fig 1D-PAS-sham-3/3-1.jpeg]

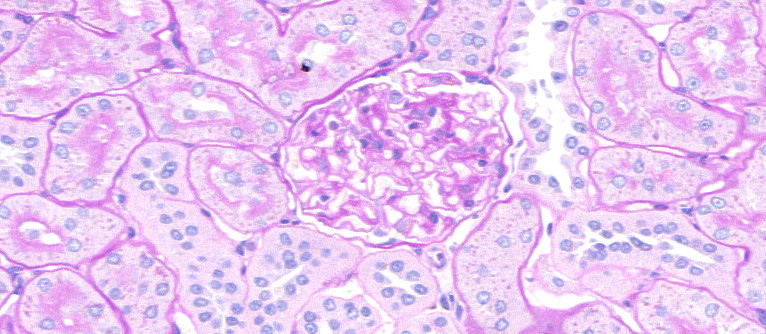

Supplement: Supplementary file 2 [file DataSheet14.ZIP › sham/Fig 1D-PAS-sham-3/3-10.jpeg]

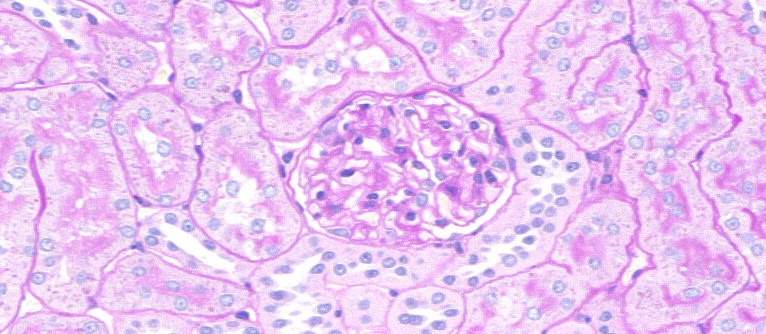

Supplement: Supplementary file 2 [file DataSheet14.ZIP › sham/Fig 1D-PAS-sham-3/3-11.jpeg]

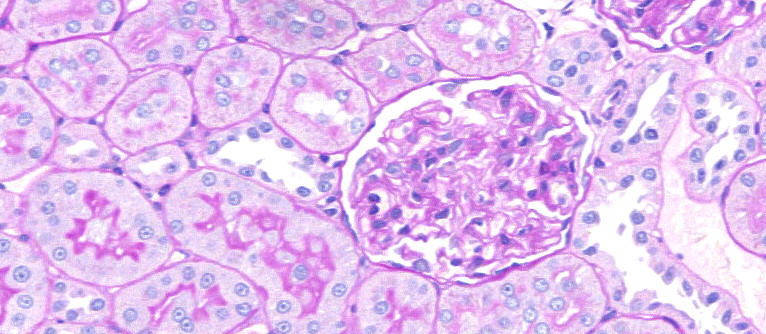

Supplement: Supplementary file 2 [file DataSheet14.ZIP › sham/Fig 1D-PAS-sham-3/3-12.jpeg]

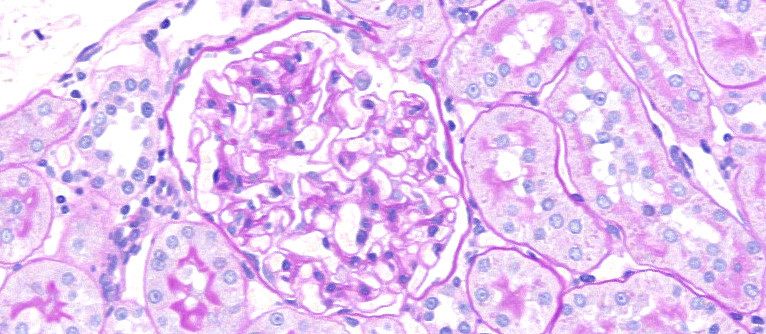

Supplement: Supplementary file 2 [file DataSheet14.ZIP › sham/Fig 1D-PAS-sham-3/3-13.jpeg]

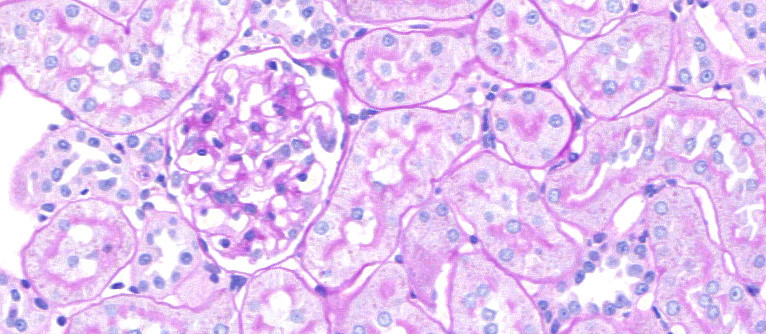

Supplement: Supplementary file 2 [file DataSheet14.ZIP › sham/Fig 1D-PAS-sham-3/3-14.jpeg]

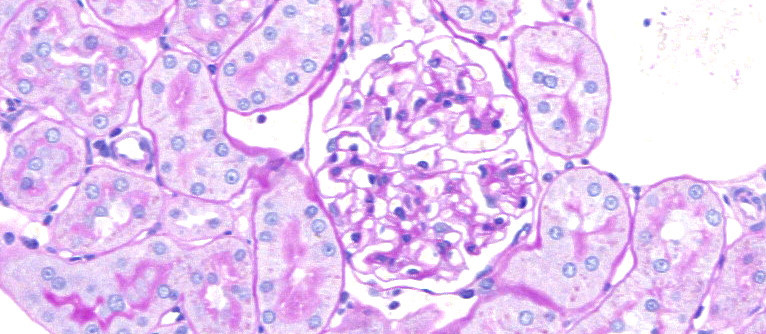

Supplement: Supplementary file 2 [file DataSheet14.ZIP › sham/Fig 1D-PAS-sham-3/3-15.jpeg]

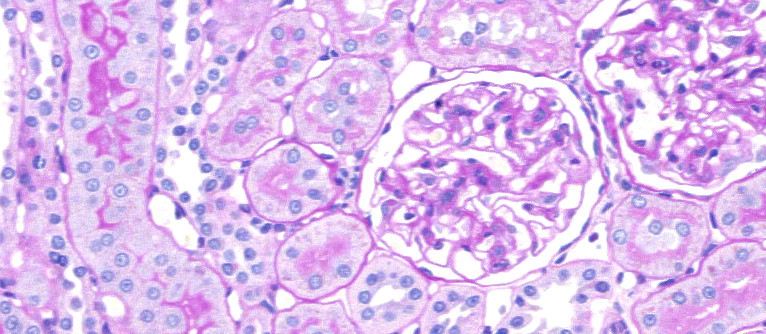

Supplement: Supplementary file 2 [file DataSheet14.ZIP › sham/Fig 1D-PAS-sham-3/3-16.jpeg]

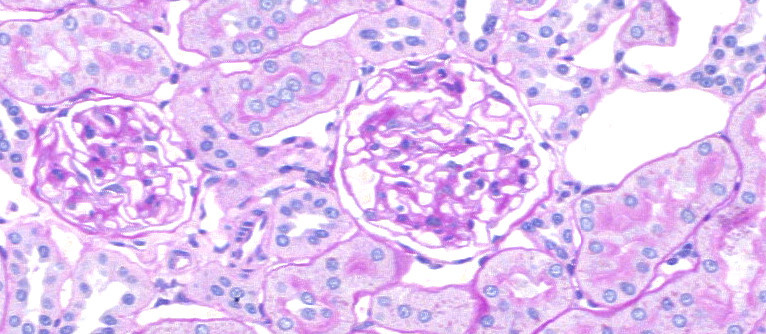

Supplement: Supplementary file 2 [file DataSheet14.ZIP › sham/Fig 1D-PAS-sham-3/3-17.jpeg]

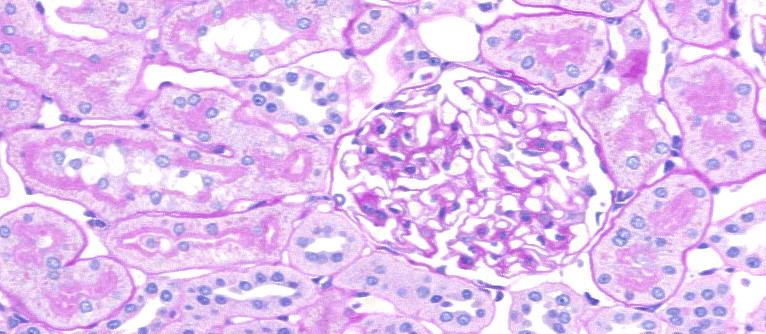

Supplement: Supplementary file 2 [file DataSheet14.ZIP › sham/Fig 1D-PAS-sham-3/3-18.jpeg]

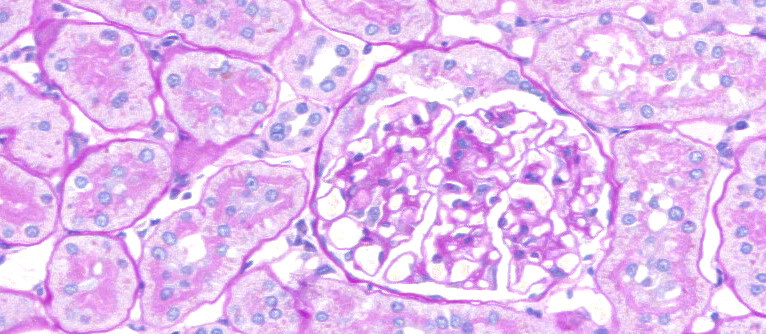

Supplement: Supplementary file 2 [file DataSheet14.ZIP › sham/Fig 1D-PAS-sham-3/3-19.jpeg]

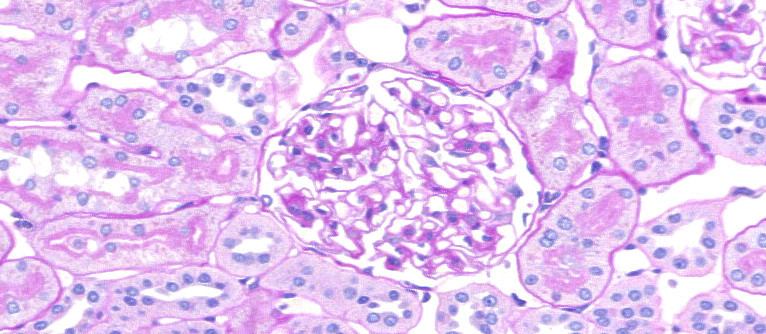

Supplement: Supplementary file 2 [file DataSheet14.ZIP › sham/Fig 1D-PAS-sham-3/3-2.jpeg]

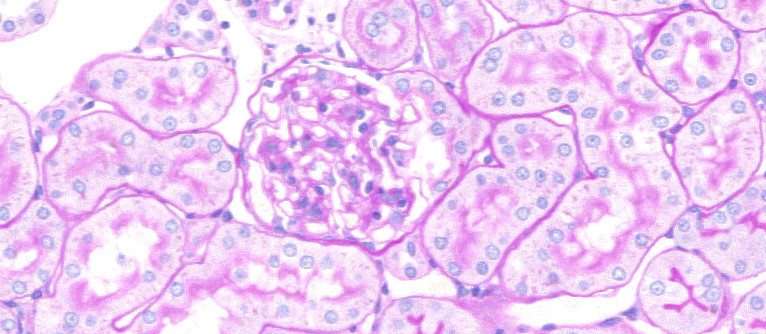

Supplement: Supplementary file 2 [file DataSheet14.ZIP › sham/Fig 1D-PAS-sham-3/3-20.jpeg]

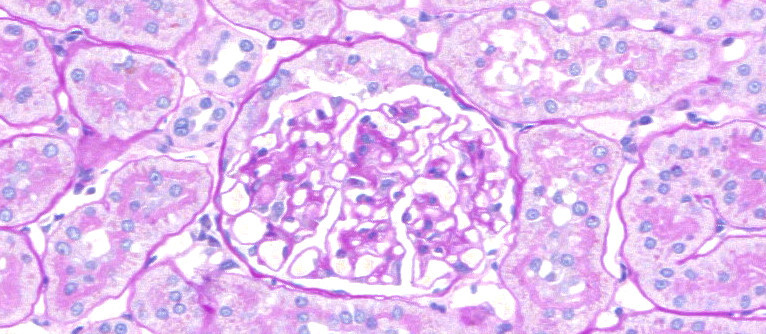

Supplement: Supplementary file 2 [file DataSheet14.ZIP › sham/Fig 1D-PAS-sham-3/3-3.jpeg]

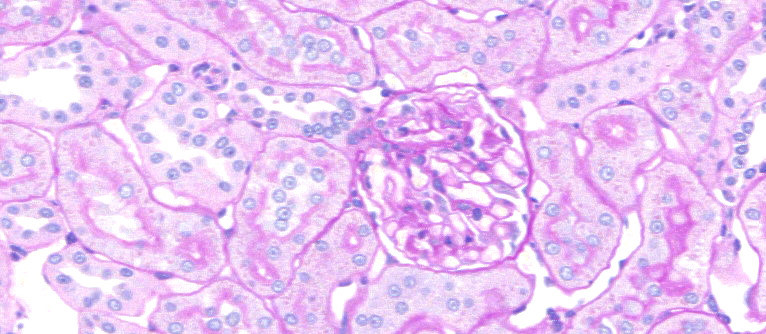

Supplement: Supplementary file 2 [file DataSheet14.ZIP › sham/Fig 1D-PAS-sham-3/3-4.jpeg]

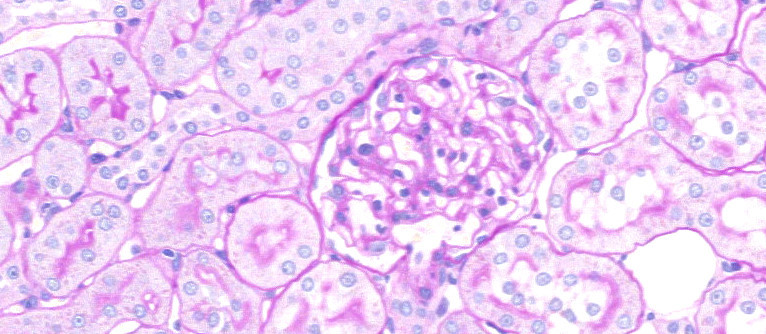

Supplement: Supplementary file 2 [file DataSheet14.ZIP › sham/Fig 1D-PAS-sham-3/3-5.jpeg]

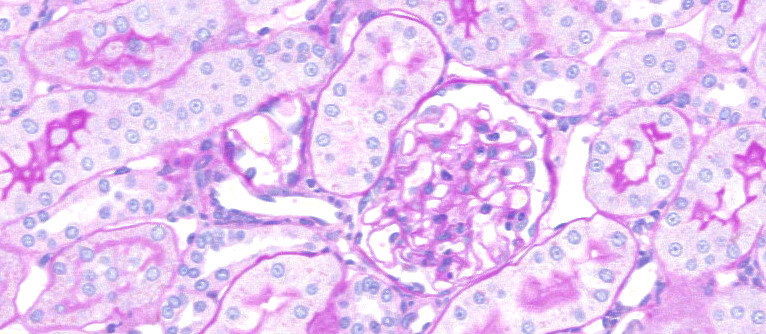

Supplement: Supplementary file 2 [file DataSheet14.ZIP › sham/Fig 1D-PAS-sham-3/3-6.jpeg]

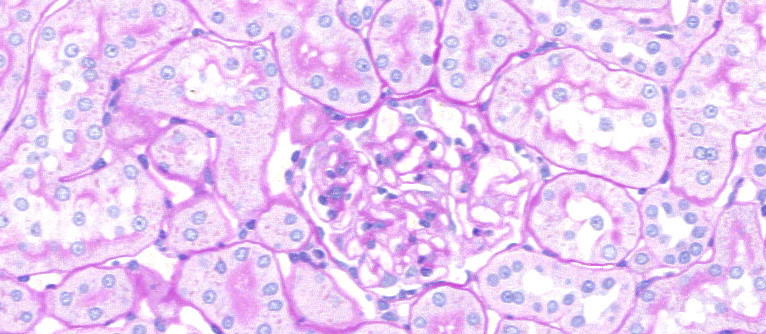

Supplement: Supplementary file 2 [file DataSheet14.ZIP › sham/Fig 1D-PAS-sham-3/3-7.jpeg]

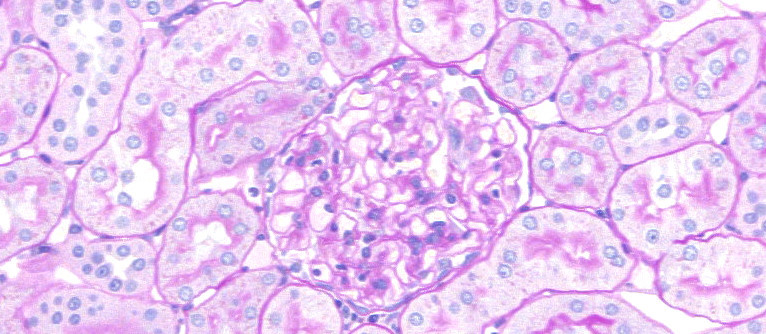

Supplement: Supplementary file 2 [file DataSheet14.ZIP › sham/Fig 1D-PAS-sham-3/3-8.jpeg]

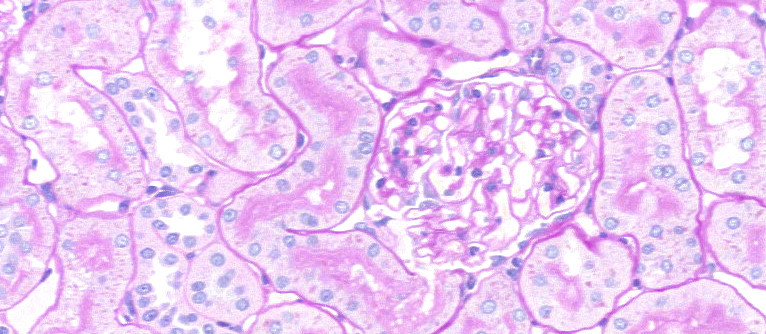

Supplement: Supplementary file 2 [file DataSheet14.ZIP › sham/Fig 1D-PAS-sham-3/3-9.jpeg]

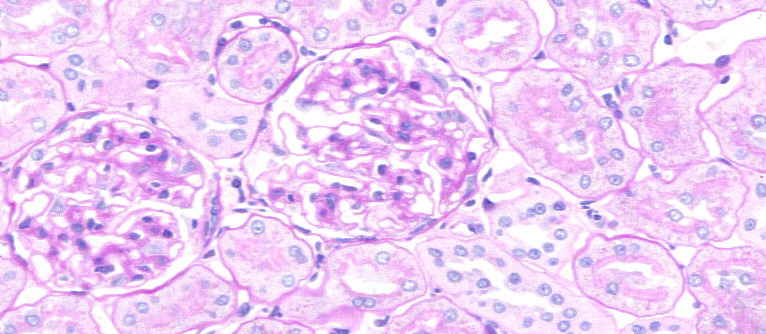

Supplement: Supplementary file 2 [file DataSheet14.ZIP › sham/Fig 1D-PAS-sham-4/4-1.jpeg]

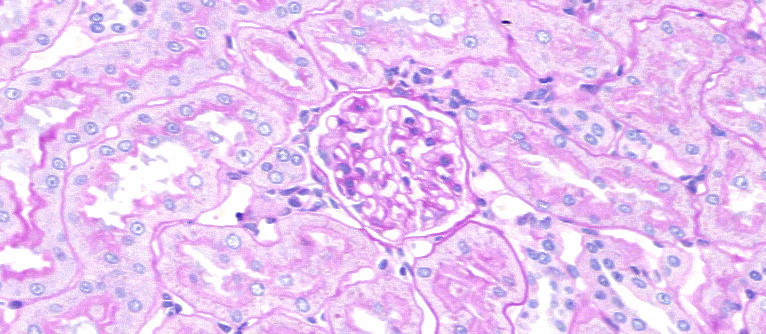

Supplement: Supplementary file 2 [file DataSheet14.ZIP › sham/Fig 1D-PAS-sham-4/4-10.jpeg]

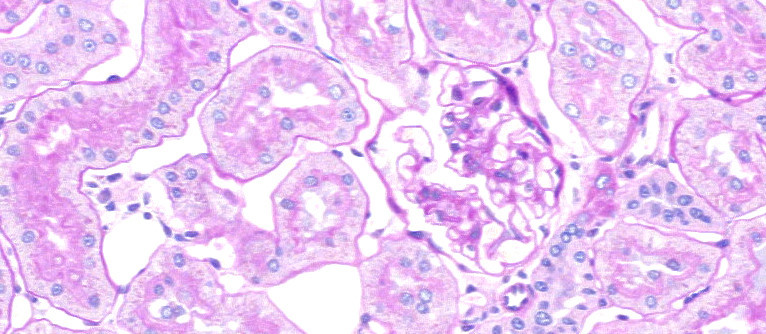

Supplement: Supplementary file 2 [file DataSheet14.ZIP › sham/Fig 1D-PAS-sham-4/4-11.jpeg]

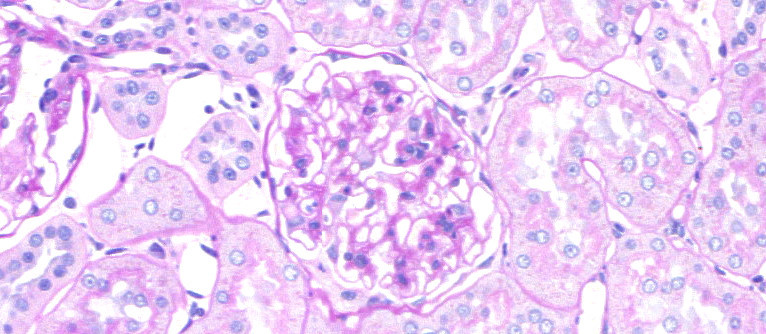

Supplement: Supplementary file 2 [file DataSheet14.ZIP › sham/Fig 1D-PAS-sham-4/4-12.jpeg]

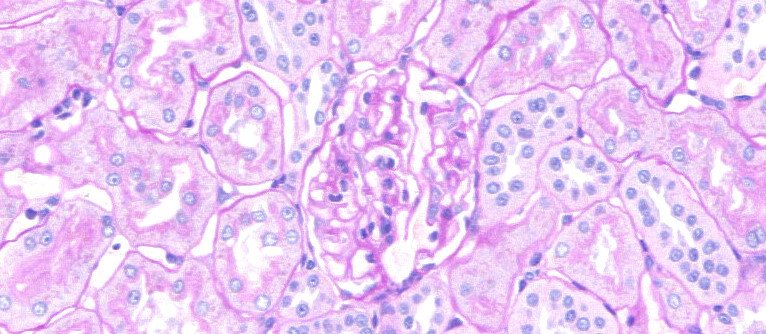

Supplement: Supplementary file 2 [file DataSheet14.ZIP › sham/Fig 1D-PAS-sham-4/4-13.jpeg]

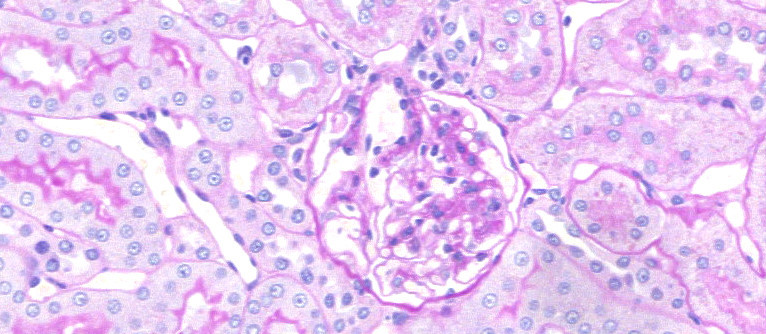

Supplement: Supplementary file 2 [file DataSheet14.ZIP › sham/Fig 1D-PAS-sham-4/4-14.jpeg]

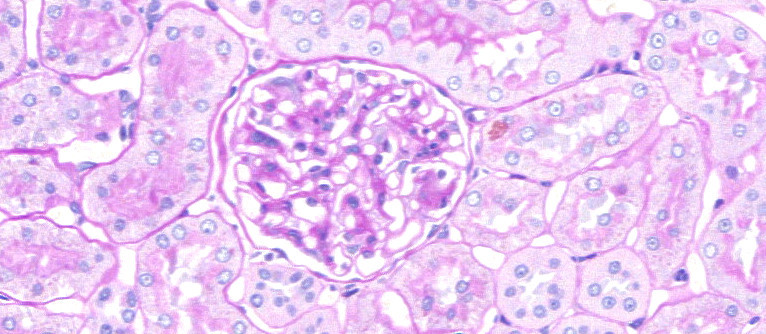

Supplement: Supplementary file 2 [file DataSheet14.ZIP › sham/Fig 1D-PAS-sham-4/4-15.jpeg]

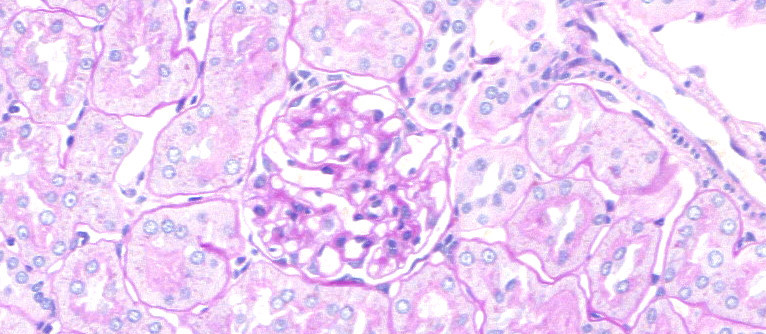

Supplement: Supplementary file 2 [file DataSheet14.ZIP › sham/Fig 1D-PAS-sham-4/4-16.jpeg]

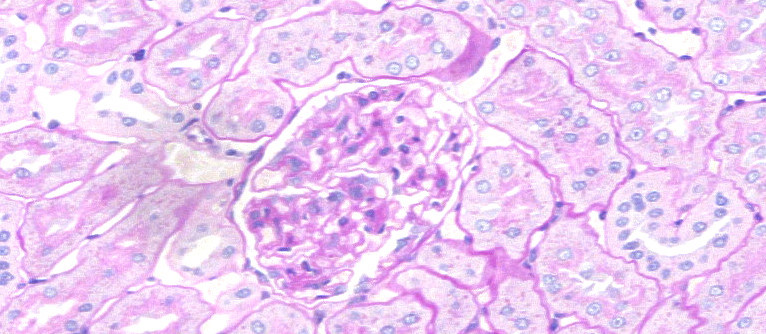

Supplement: Supplementary file 2 [file DataSheet14.ZIP › sham/Fig 1D-PAS-sham-4/4-17.jpeg]

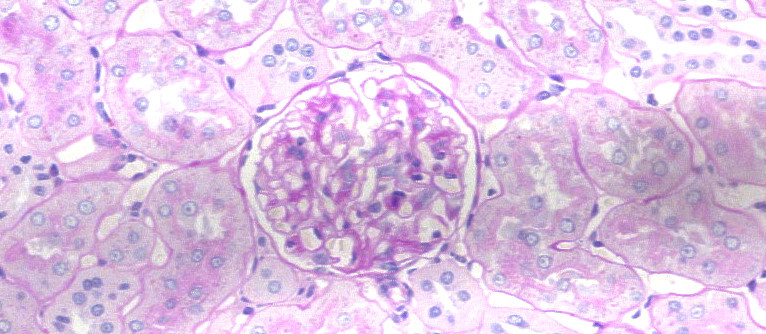

Supplement: Supplementary file 2 [file DataSheet14.ZIP › sham/Fig 1D-PAS-sham-4/4-18.jpeg]

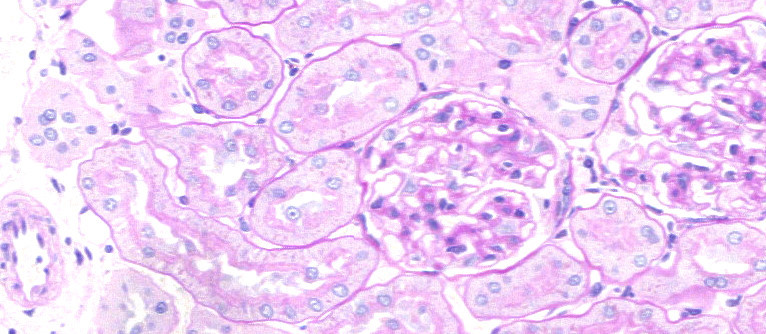

Supplement: Supplementary file 2 [file DataSheet14.ZIP › sham/Fig 1D-PAS-sham-4/4-19.jpeg]

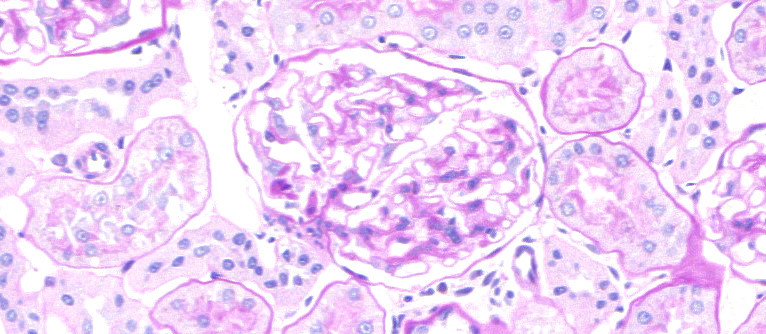

Supplement: Supplementary file 2 [file DataSheet14.ZIP › sham/Fig 1D-PAS-sham-4/4-2.jpeg]

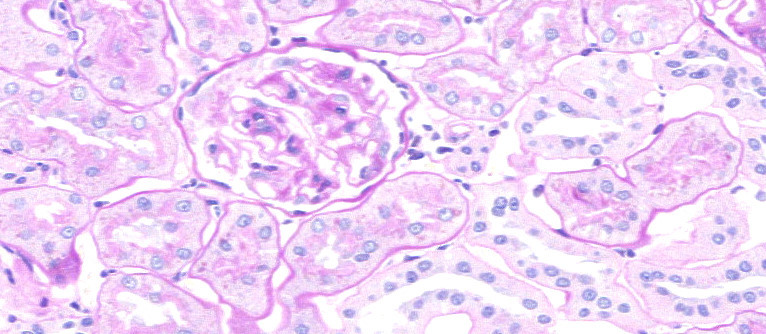

Supplement: Supplementary file 2 [file DataSheet14.ZIP › sham/Fig 1D-PAS-sham-4/4-20.jpeg]

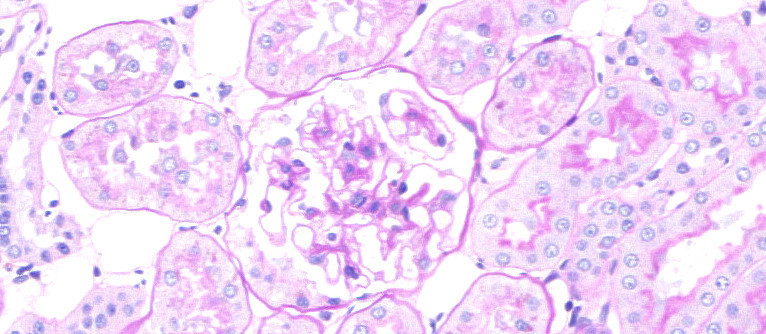

Supplement: Supplementary file 2 [file DataSheet14.ZIP › sham/Fig 1D-PAS-sham-4/4-3.jpeg]

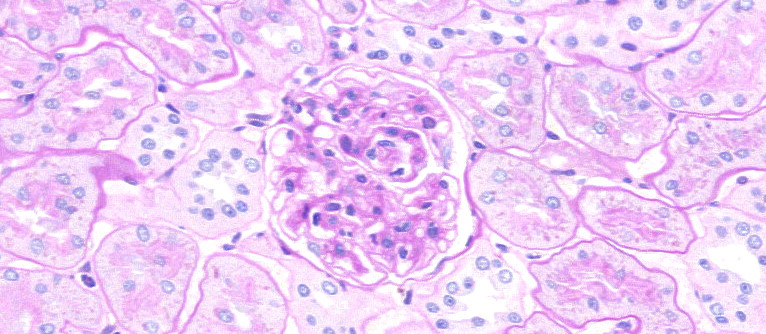

Supplement: Supplementary file 2 [file DataSheet14.ZIP › sham/Fig 1D-PAS-sham-4/4-4.jpeg]

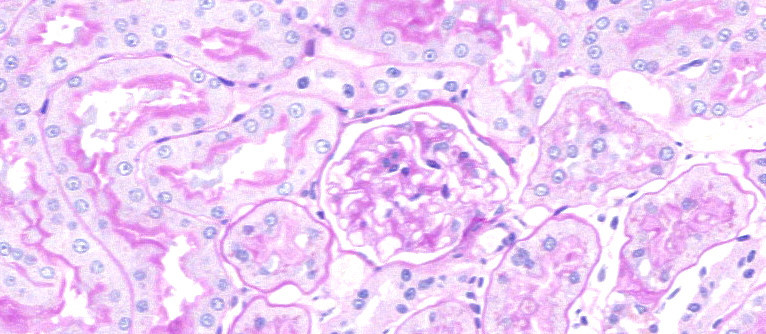

Supplement: Supplementary file 2 [file DataSheet14.ZIP › sham/Fig 1D-PAS-sham-4/4-5.jpeg]

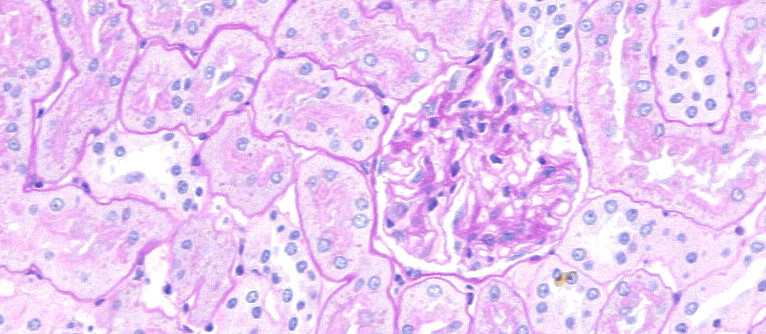

Supplement: Supplementary file 2 [file DataSheet14.ZIP › sham/Fig 1D-PAS-sham-4/4-6.jpeg]

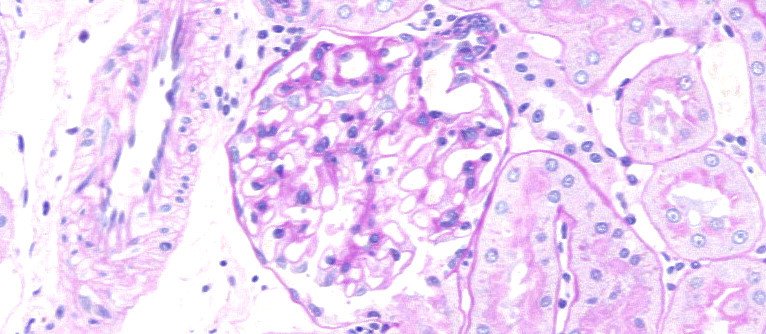

Supplement: Supplementary file 2 [file DataSheet14.ZIP › sham/Fig 1D-PAS-sham-4/4-7.jpeg]

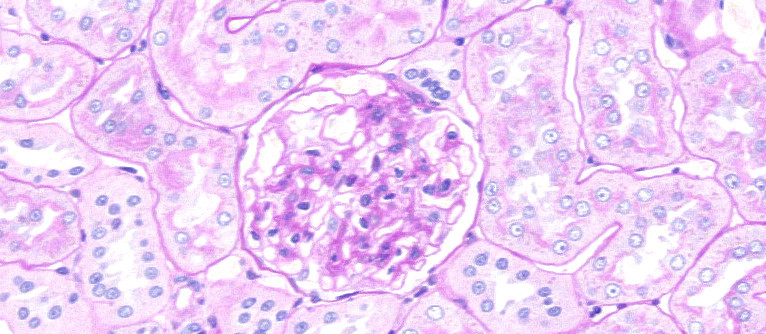

Supplement: Supplementary file 2 [file DataSheet14.ZIP › sham/Fig 1D-PAS-sham-4/4-8.jpeg]

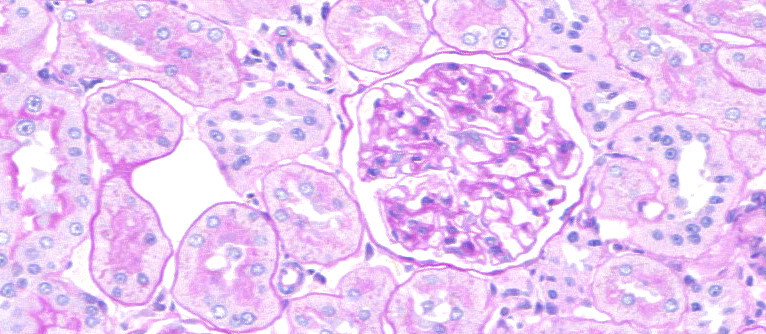

Supplement: Supplementary file 2 [file DataSheet14.ZIP › sham/Fig 1D-PAS-sham-4/4-9.jpeg]

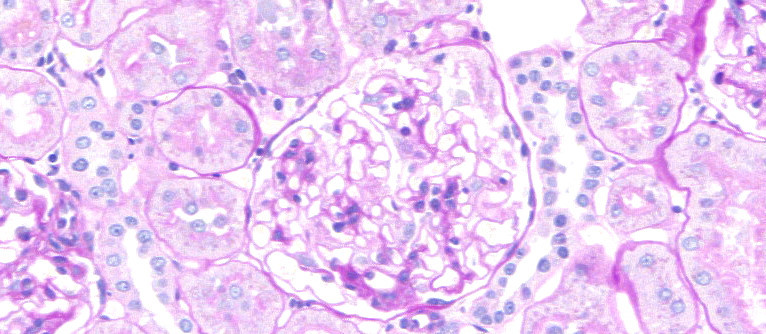

Supplement: Supplementary file 2 [file DataSheet14.ZIP › sham/Fig 1D-PAS-sham-5/5-1.jpeg]

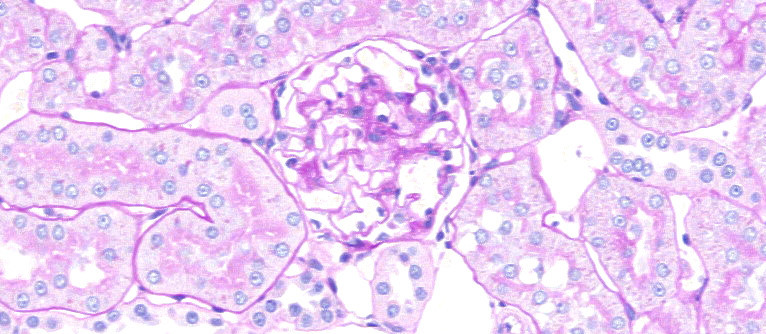

Supplement: Supplementary file 2 [file DataSheet14.ZIP › sham/Fig 1D-PAS-sham-5/5-10.jpeg]

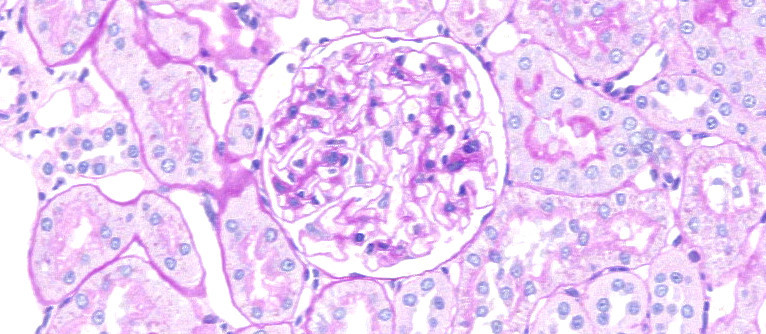

Supplement: Supplementary file 2 [file DataSheet14.ZIP › sham/Fig 1D-PAS-sham-5/5-11.jpeg]

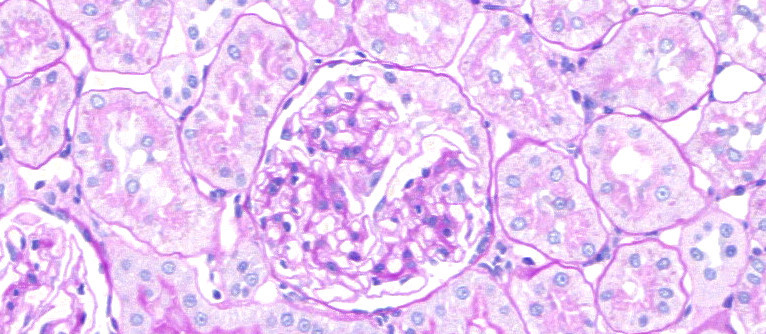

Supplement: Supplementary file 2 [file DataSheet14.ZIP › sham/Fig 1D-PAS-sham-5/5-12.jpeg]

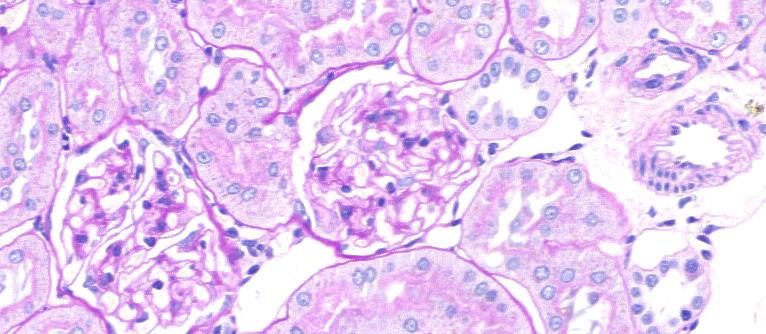

Supplement: Supplementary file 2 [file DataSheet14.ZIP › sham/Fig 1D-PAS-sham-5/5-13.jpeg]

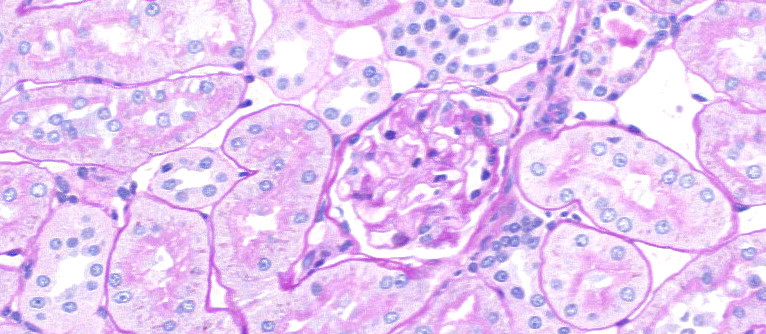

Supplement: Supplementary file 2 [file DataSheet14.ZIP › sham/Fig 1D-PAS-sham-5/5-14.jpeg]

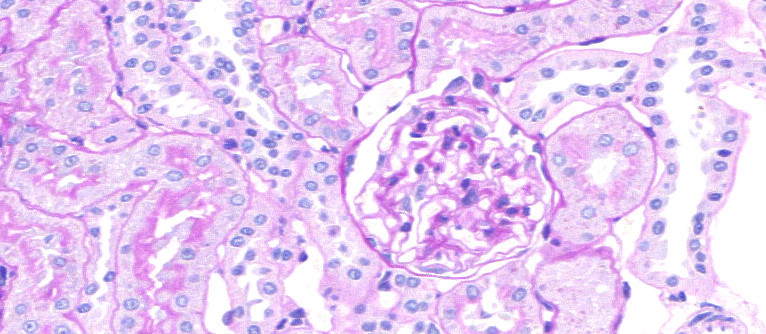

Supplement: Supplementary file 2 [file DataSheet14.ZIP › sham/Fig 1D-PAS-sham-5/5-15.jpeg]

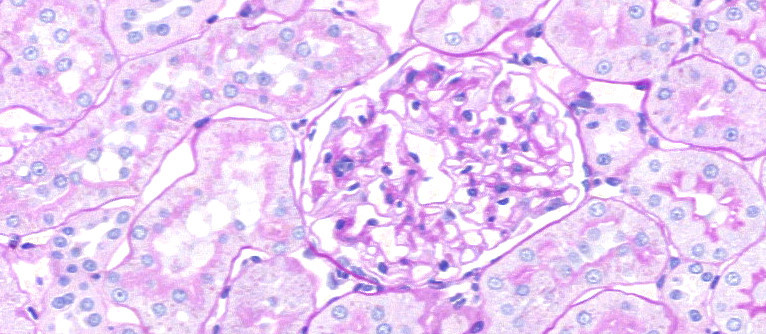

Supplement: Supplementary file 2 [file DataSheet14.ZIP › sham/Fig 1D-PAS-sham-5/5-16.jpeg]

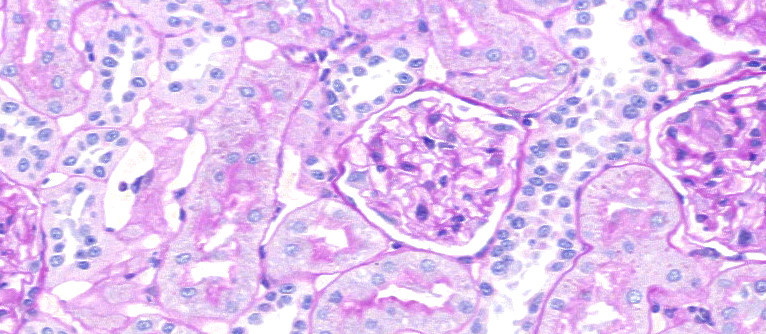

Supplement: Supplementary file 2 [file DataSheet14.ZIP › sham/Fig 1D-PAS-sham-5/5-17.jpeg]

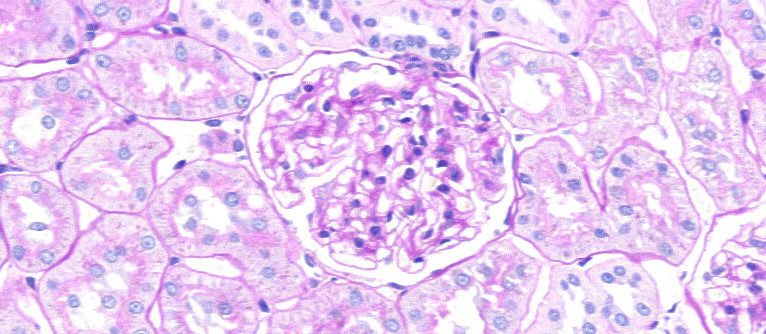

Supplement: Supplementary file 2 [file DataSheet14.ZIP › sham/Fig 1D-PAS-sham-5/5-18.jpeg]

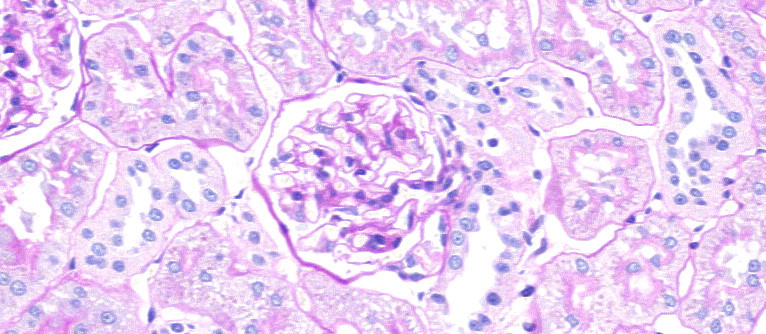

Supplement: Supplementary file 2 [file DataSheet14.ZIP › sham/Fig 1D-PAS-sham-5/5-19.jpeg]

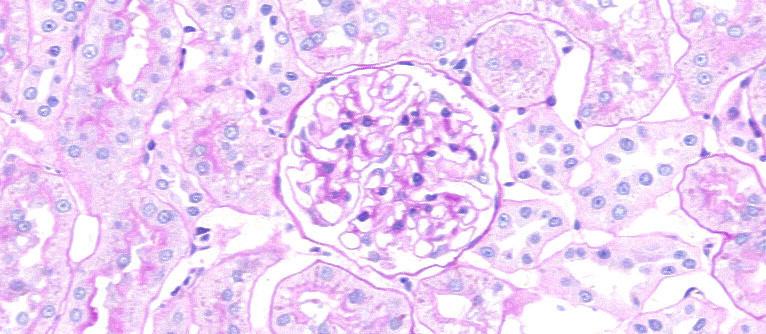

Supplement: Supplementary file 2 [file DataSheet14.ZIP › sham/Fig 1D-PAS-sham-5/5-2.jpeg]

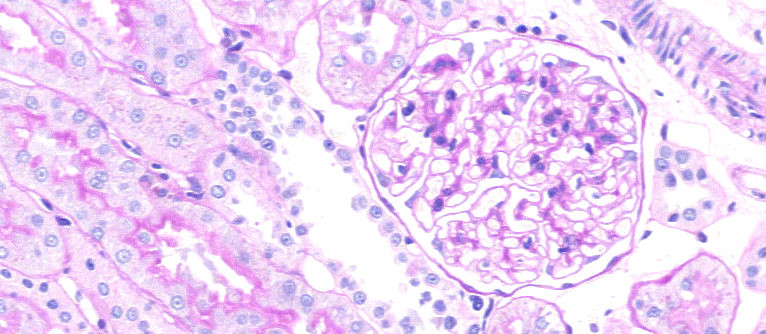

Supplement: Supplementary file 2 [file DataSheet14.ZIP › sham/Fig 1D-PAS-sham-5/5-20.jpeg]

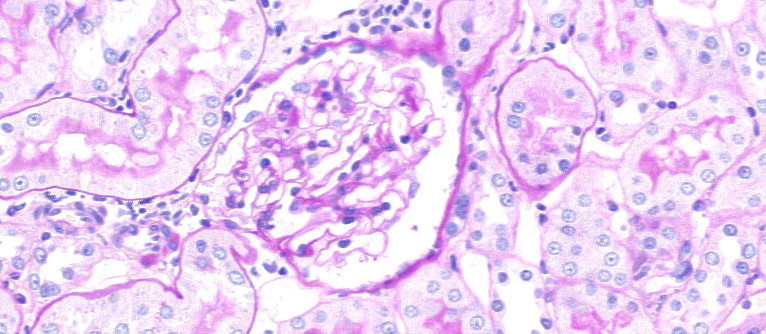

Supplement: Supplementary file 2 [file DataSheet14.ZIP › sham/Fig 1D-PAS-sham-5/5-3.jpeg]

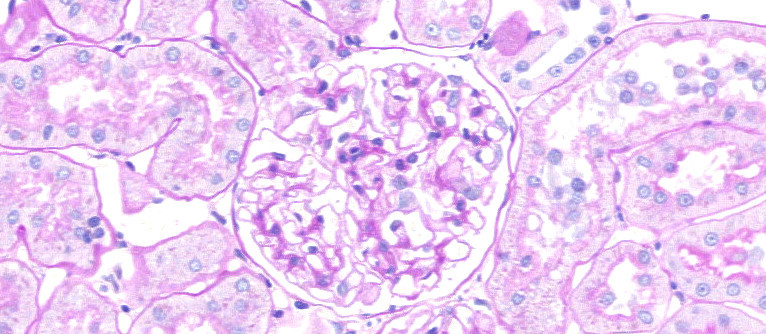

Supplement: Supplementary file 2 [file DataSheet14.ZIP › sham/Fig 1D-PAS-sham-5/5-4.jpeg]

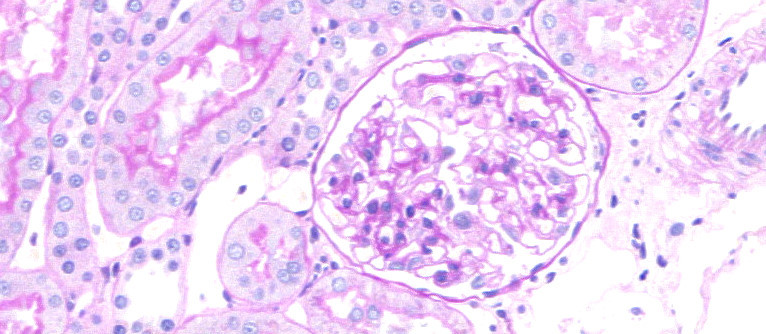

Supplement: Supplementary file 2 [file DataSheet14.ZIP › sham/Fig 1D-PAS-sham-5/5-5.jpeg]

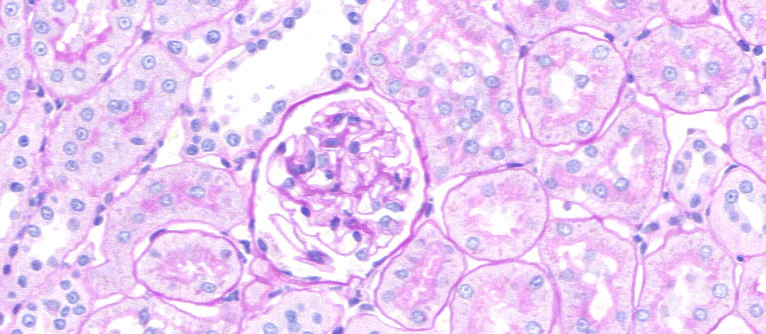

Supplement: Supplementary file 2 [file DataSheet14.ZIP › sham/Fig 1D-PAS-sham-5/5-6.jpeg]

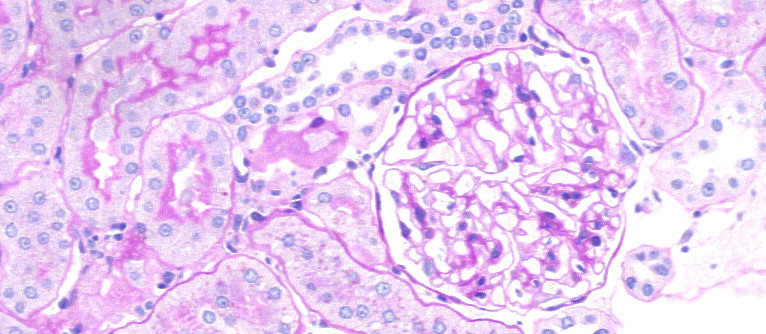

Supplement: Supplementary file 2 [file DataSheet14.ZIP › sham/Fig 1D-PAS-sham-5/5-7.jpeg]

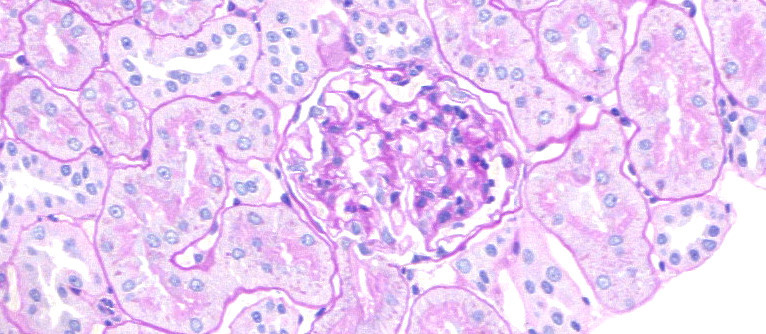

Supplement: Supplementary file 2 [file DataSheet14.ZIP › sham/Fig 1D-PAS-sham-5/5-8.jpeg]

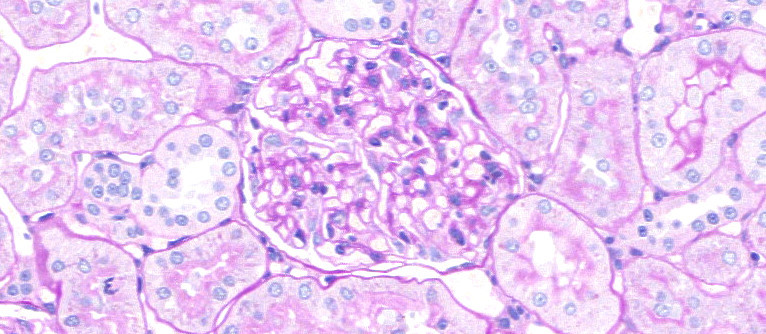

Supplement: Supplementary file 2 [file DataSheet14.ZIP › sham/Fig 1D-PAS-sham-5/5-9.jpeg]

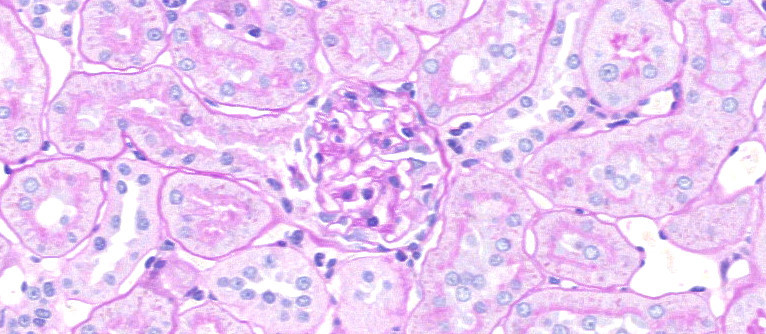

Supplement: Supplementary file 2 [file DataSheet14.ZIP › sham/Fig 1D-PAS-sham-6/6-1.jpeg]

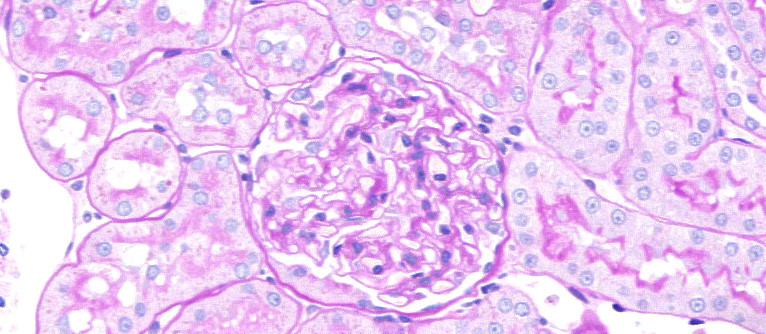

Supplement: Supplementary file 2 [file DataSheet14.ZIP › sham/Fig 1D-PAS-sham-6/6-10.jpeg]

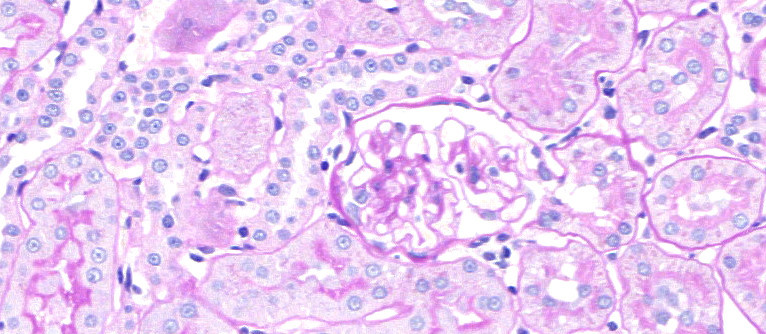

Supplement: Supplementary file 2 [file DataSheet14.ZIP › sham/Fig 1D-PAS-sham-6/6-11.jpeg]

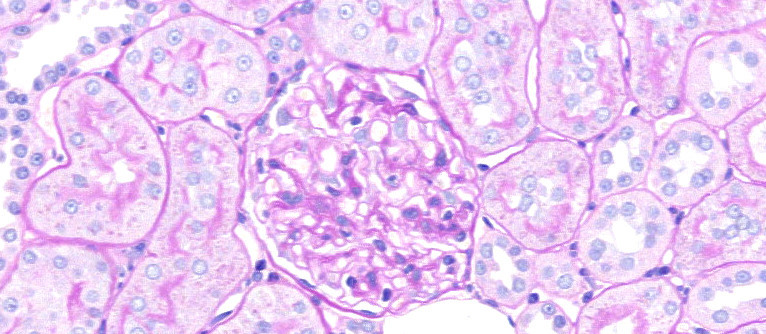

Supplement: Supplementary file 2 [file DataSheet14.ZIP › sham/Fig 1D-PAS-sham-6/6-12.jpeg]

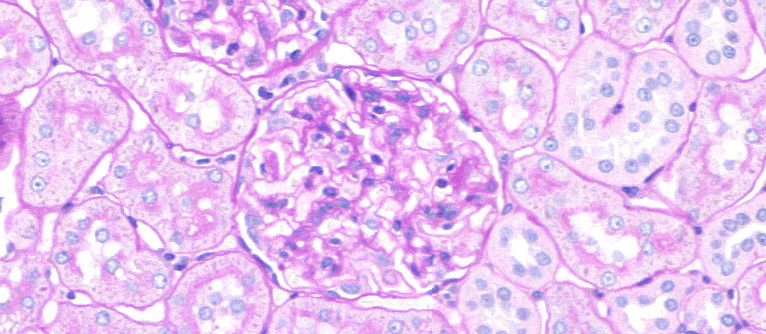

Supplement: Supplementary file 2 [file DataSheet14.ZIP › sham/Fig 1D-PAS-sham-6/6-13.jpeg]

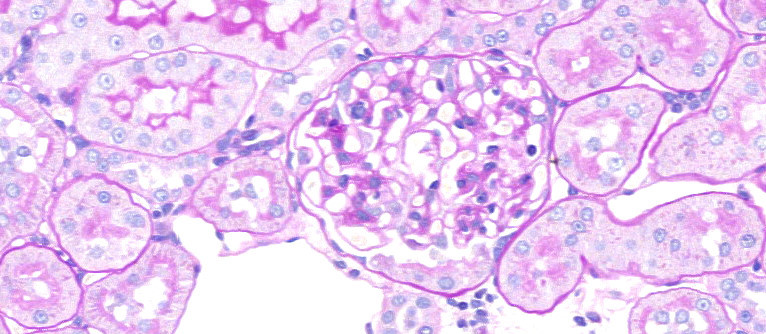

Supplement: Supplementary file 2 [file DataSheet14.ZIP › sham/Fig 1D-PAS-sham-6/6-14.jpeg]

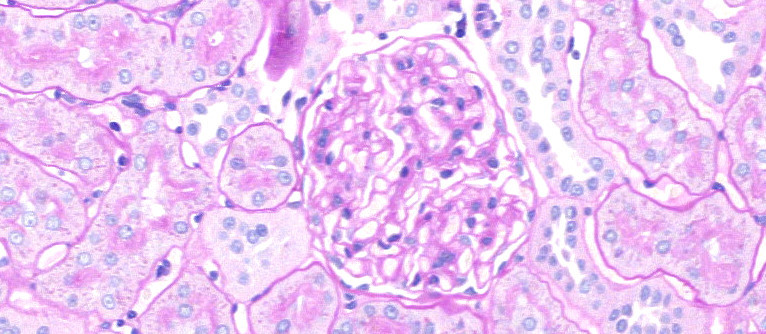

Supplement: Supplementary file 2 [file DataSheet14.ZIP › sham/Fig 1D-PAS-sham-6/6-15.jpeg]

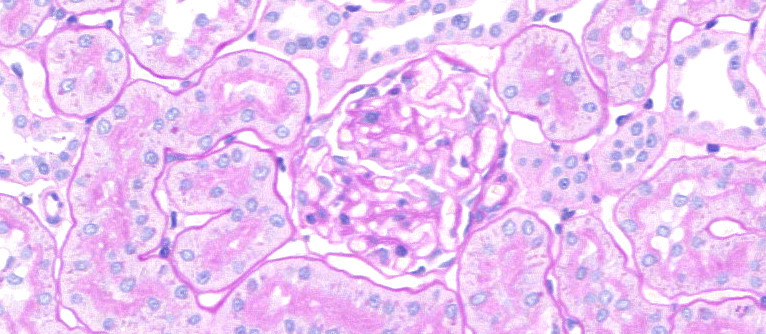

Supplement: Supplementary file 2 [file DataSheet14.ZIP › sham/Fig 1D-PAS-sham-6/6-16.jpeg]

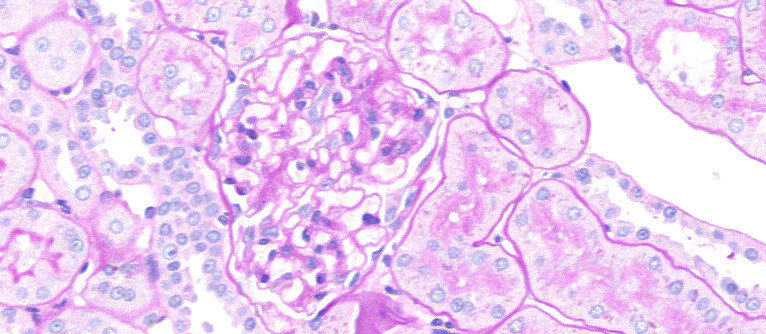

Supplement: Supplementary file 2 [file DataSheet14.ZIP › sham/Fig 1D-PAS-sham-6/6-17.jpeg]

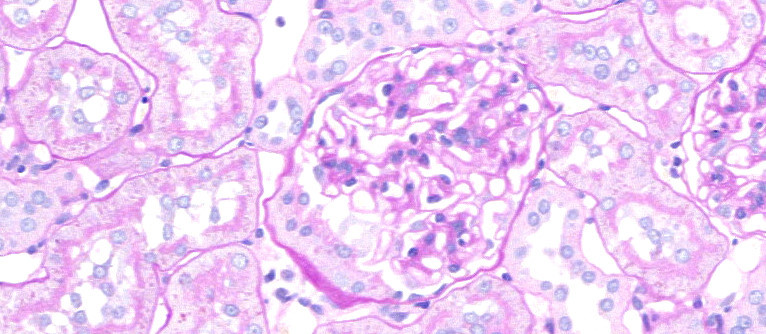

Supplement: Supplementary file 2 [file DataSheet14.ZIP › sham/Fig 1D-PAS-sham-6/6-18.jpeg]

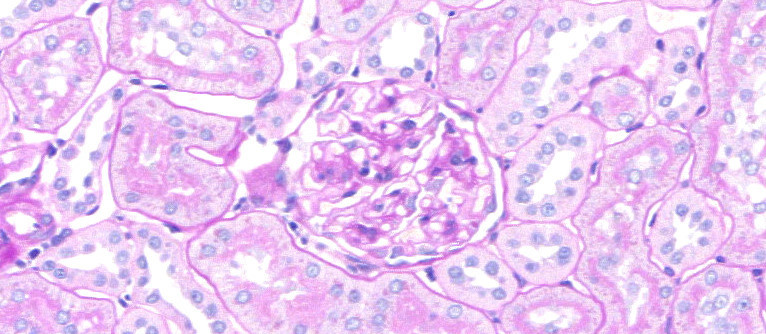

Supplement: Supplementary file 2 [file DataSheet14.ZIP › sham/Fig 1D-PAS-sham-6/6-19.jpeg]

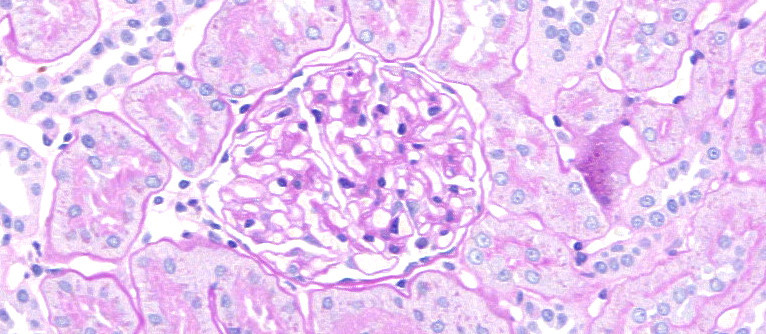

Supplement: Supplementary file 2 [file DataSheet14.ZIP › sham/Fig 1D-PAS-sham-6/6-2.jpeg]

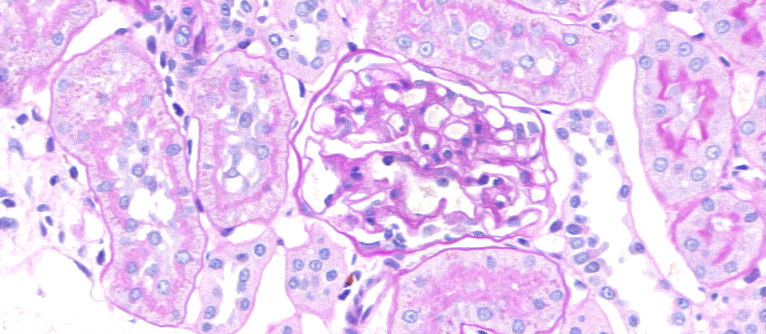

Supplement: Supplementary file 2 [file DataSheet14.ZIP › sham/Fig 1D-PAS-sham-6/6-20.jpeg]

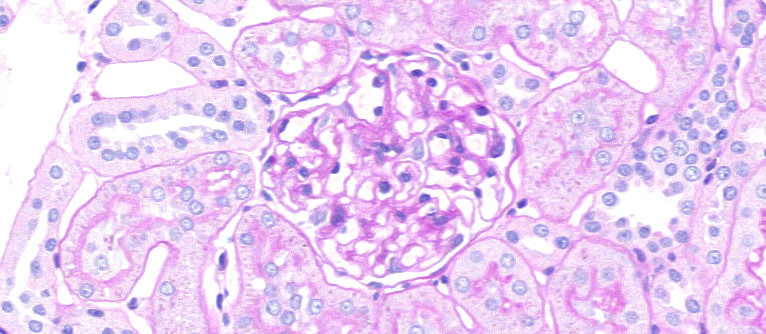

Supplement: Supplementary file 2 [file DataSheet14.ZIP › sham/Fig 1D-PAS-sham-6/6-3.jpeg]

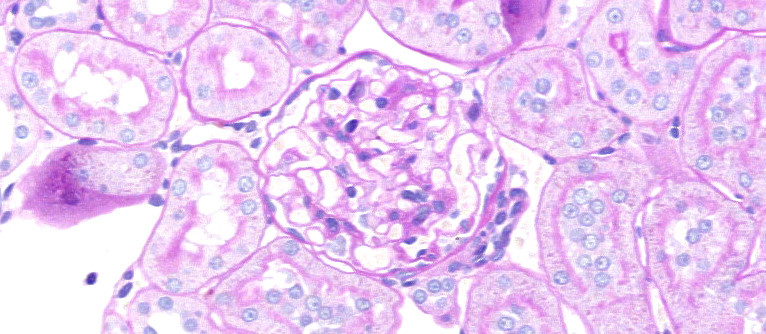

Supplement: Supplementary file 2 [file DataSheet14.ZIP › sham/Fig 1D-PAS-sham-6/6-4.jpeg]

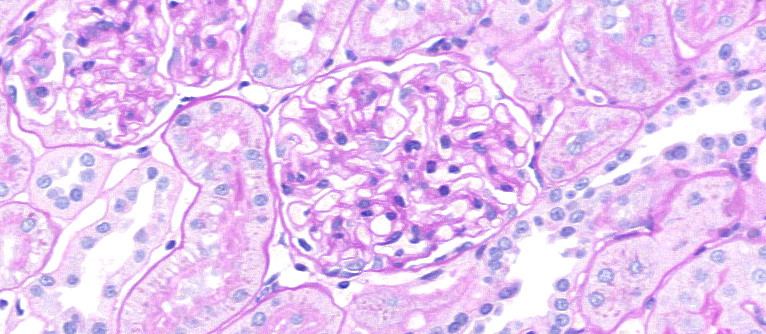

Supplement: Supplementary file 2 [file DataSheet14.ZIP › sham/Fig 1D-PAS-sham-6/6-5.jpeg]

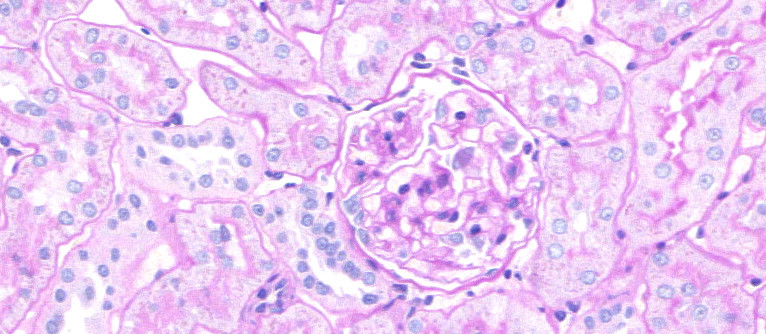

Supplement: Supplementary file 2 [file DataSheet14.ZIP › sham/Fig 1D-PAS-sham-6/6-6.jpeg]

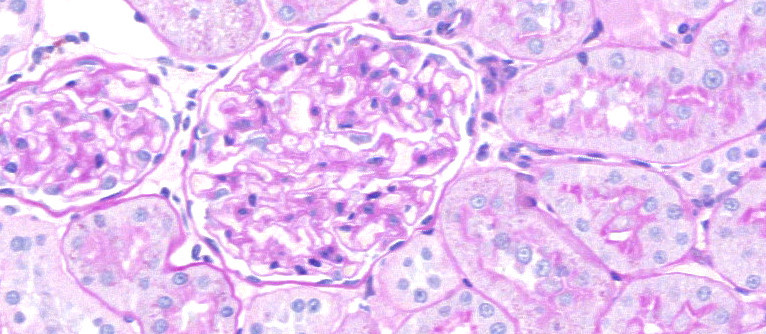

Supplement: Supplementary file 2 [file DataSheet14.ZIP › sham/Fig 1D-PAS-sham-6/6-7.jpeg]

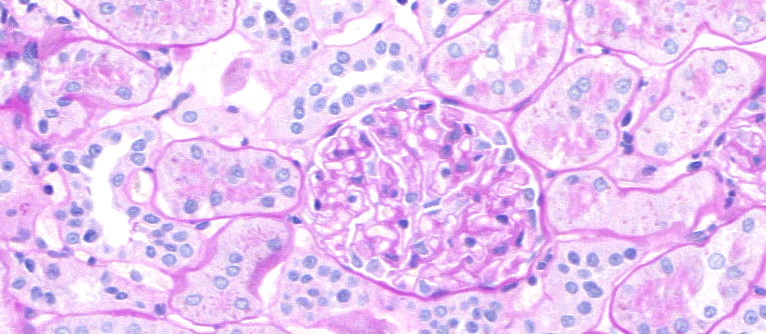

Supplement: Supplementary file 2 [file DataSheet14.ZIP › sham/Fig 1D-PAS-sham-6/6-8.jpeg]

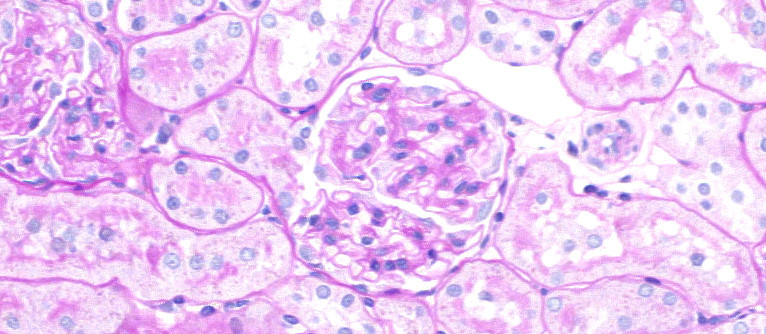

Supplement: Supplementary file 2 [file DataSheet14.ZIP › sham/Fig 1D-PAS-sham-6/6-9.jpeg]

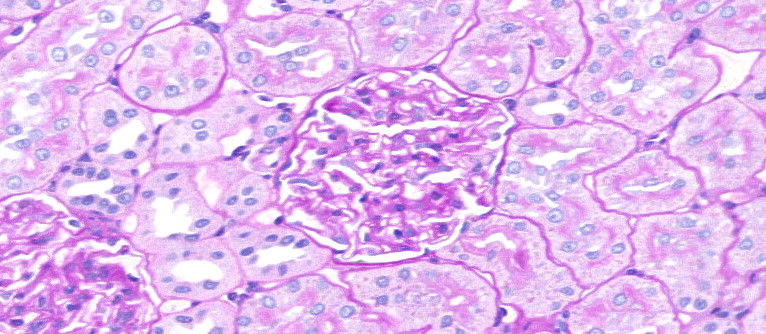

Supplement: Supplementary file 2 [file DataSheet14.ZIP › sham/Fig 1D-PAS-sham-7/7-1.jpeg]

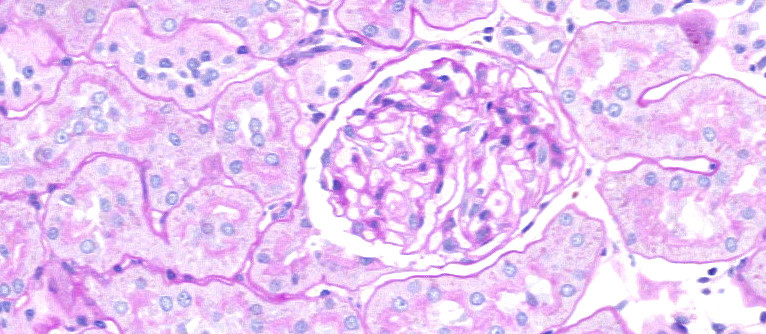

Supplement: Supplementary file 2 [file DataSheet14.ZIP › sham/Fig 1D-PAS-sham-7/7-10.jpeg]
