# Supplementary figures and images for: Characteristics of Serum Metabolites and Gut Microbiota in Diabetic Kidney Disease (part 4 of 13)
Source: Front Pharmacol. 2022 Apr 14;13:872988. doi: 10.3389/fphar.2022.872988 (PMC9084235; doi:10.3389/fphar.2022.872988)

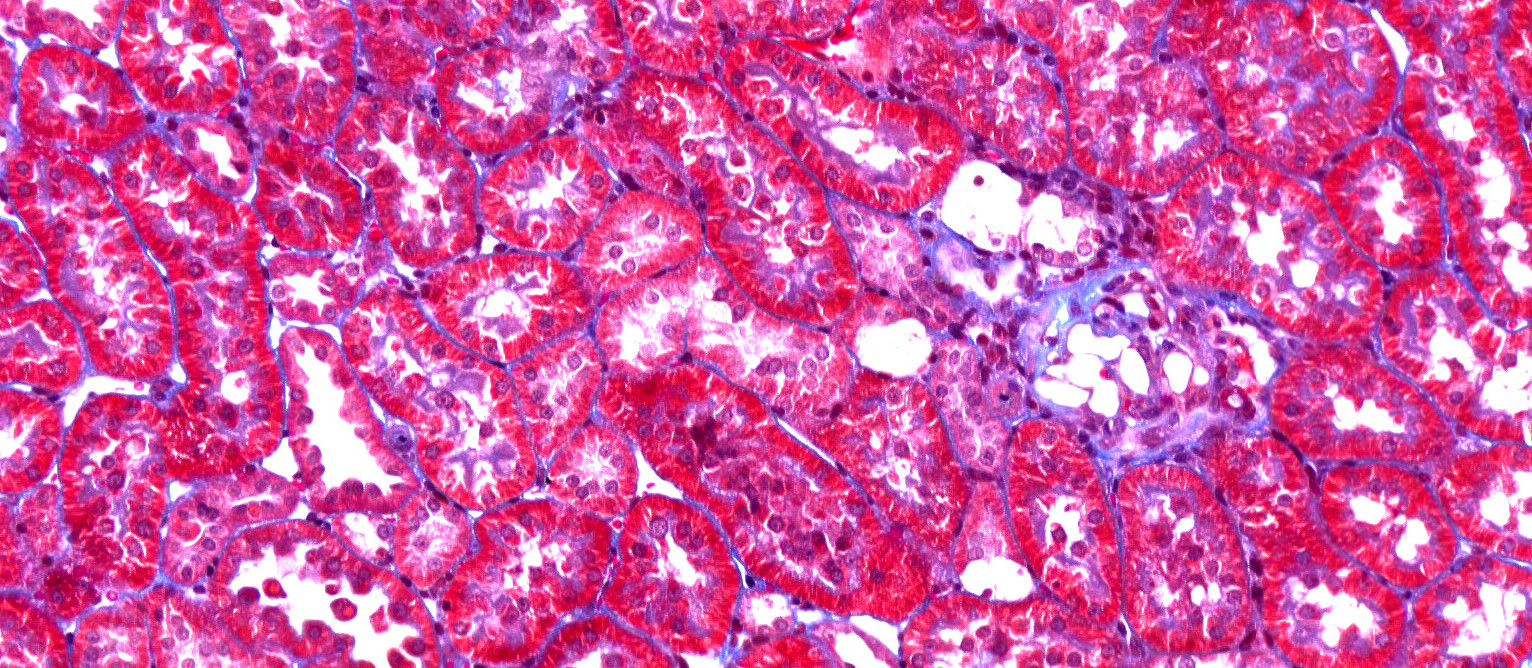

Supplement: Supplementary file 3 [file DataSheet11.ZIP › Fig 1D-masson-TSF-61/61-6.jpeg]

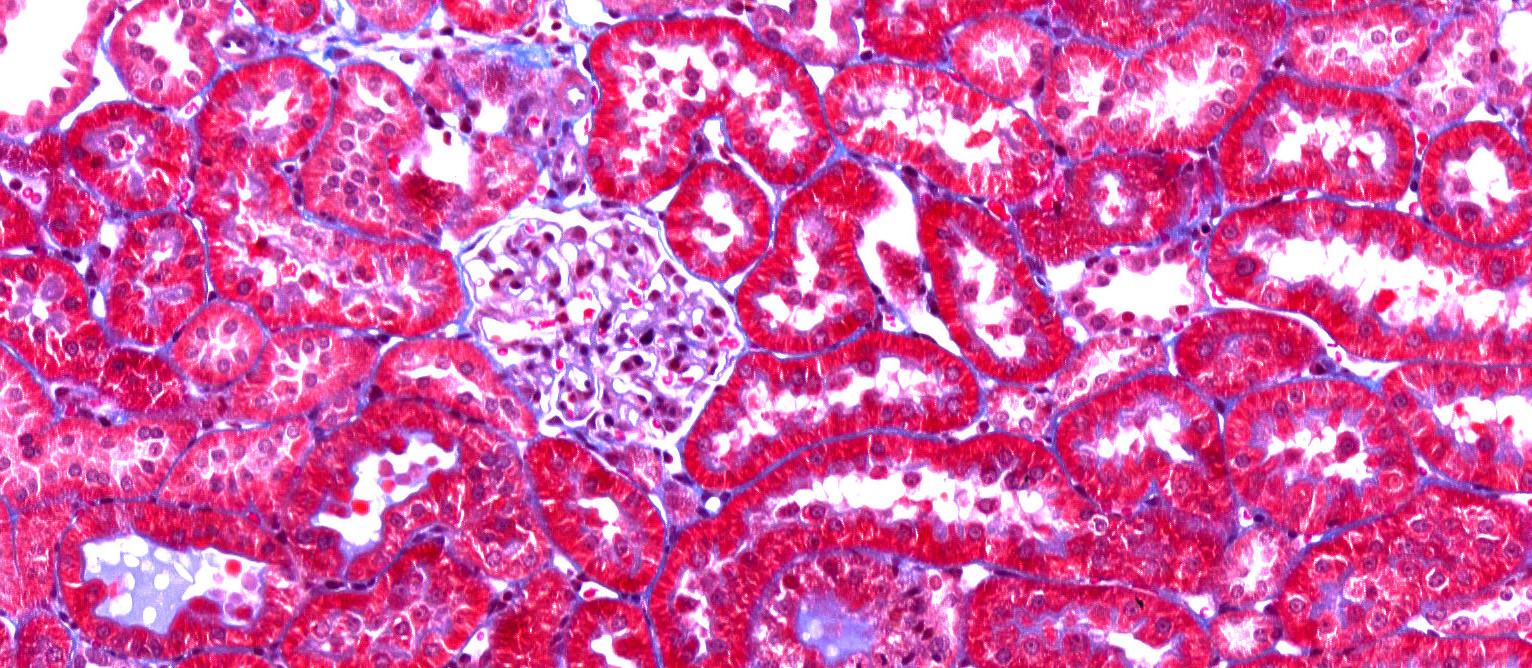

Supplement: Supplementary file 3 [file DataSheet11.ZIP › Fig 1D-masson-TSF-61/61-7.jpeg]

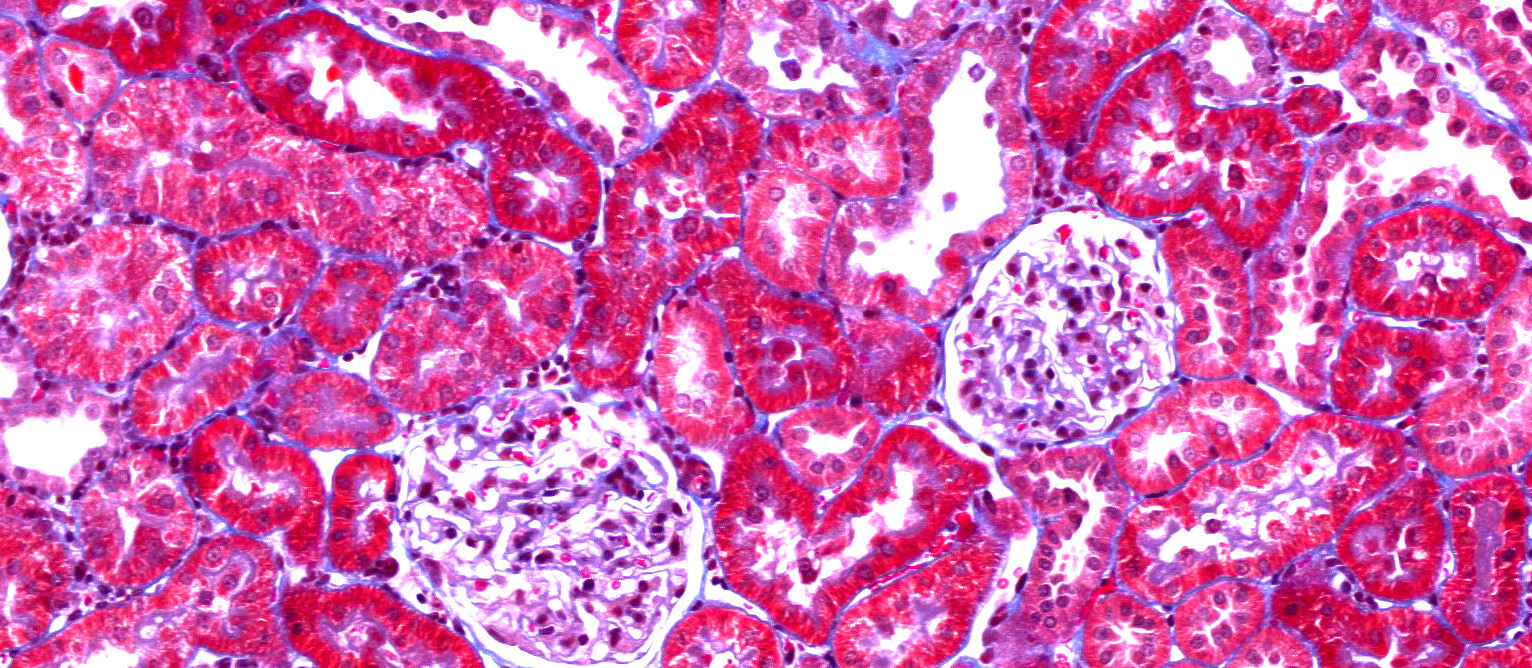

Supplement: Supplementary file 3 [file DataSheet11.ZIP › Fig 1D-masson-TSF-61/61-8.jpeg]

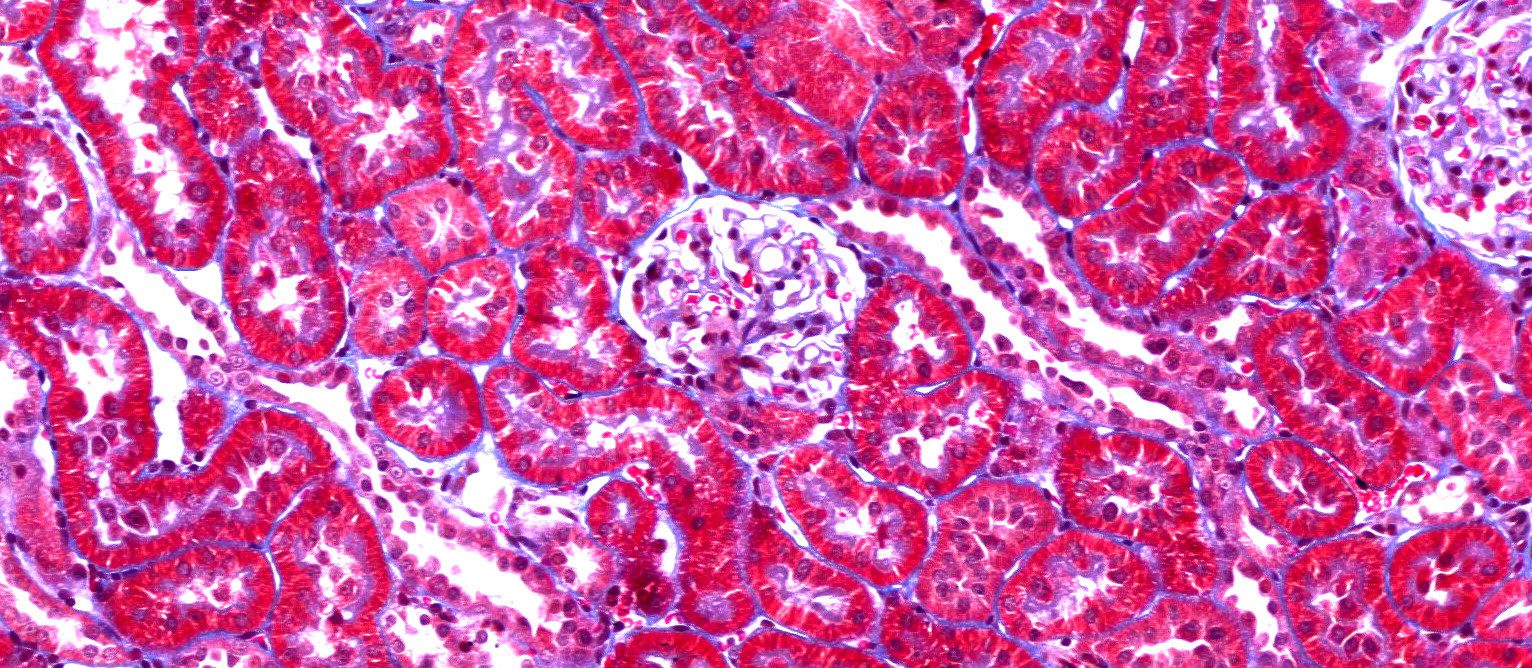

Supplement: Supplementary file 3 [file DataSheet11.ZIP › Fig 1D-masson-TSF-61/61-9.jpeg]

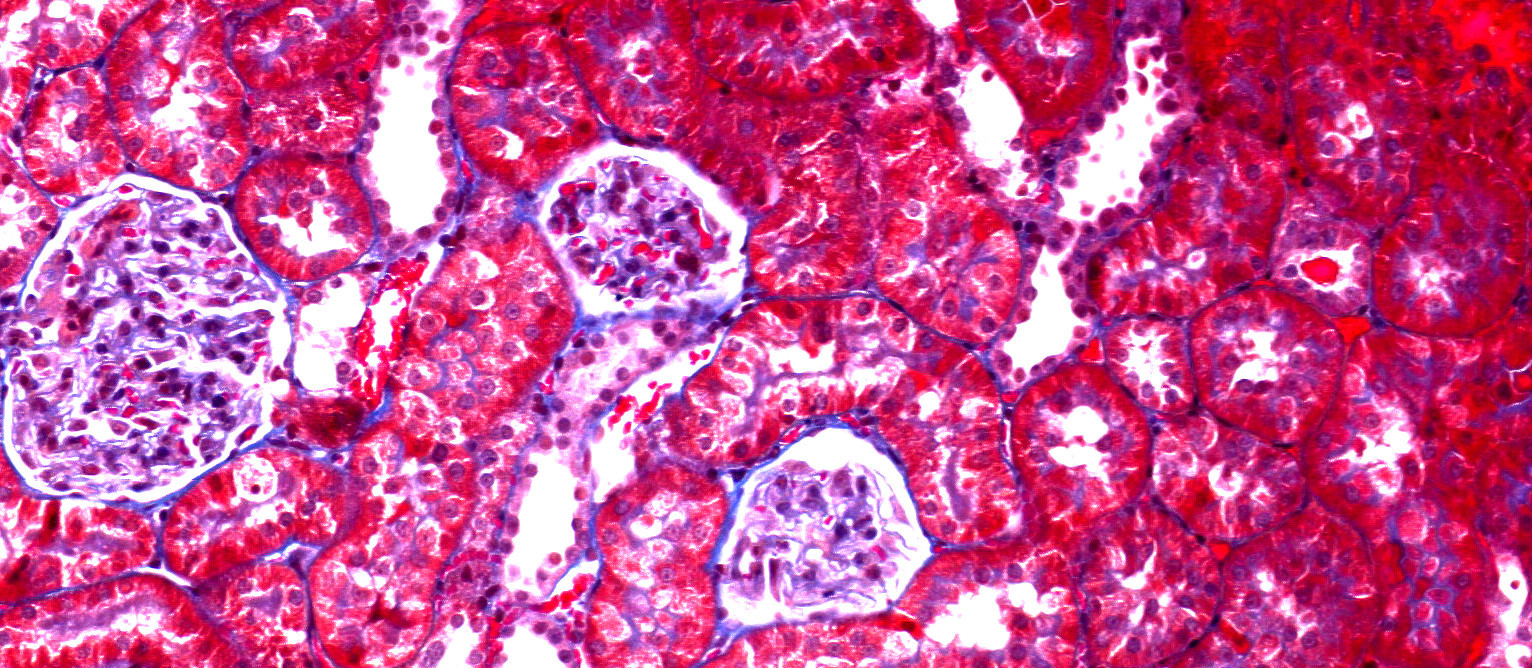

Supplement: Supplementary file 3 [file DataSheet11.ZIP › Fig 1D-masson-TSF-62(1)/62-1.jpeg]

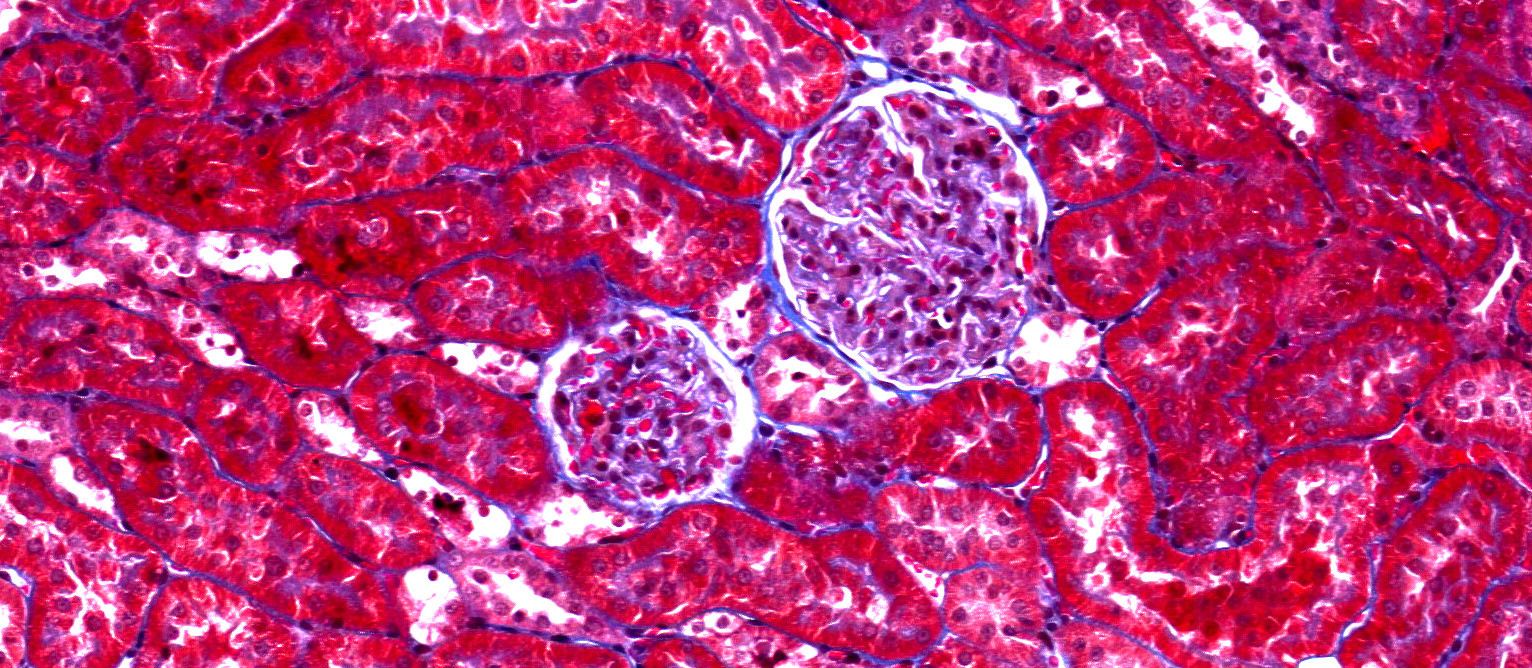

Supplement: Supplementary file 3 [file DataSheet11.ZIP › Fig 1D-masson-TSF-62(1)/62-2.jpeg]

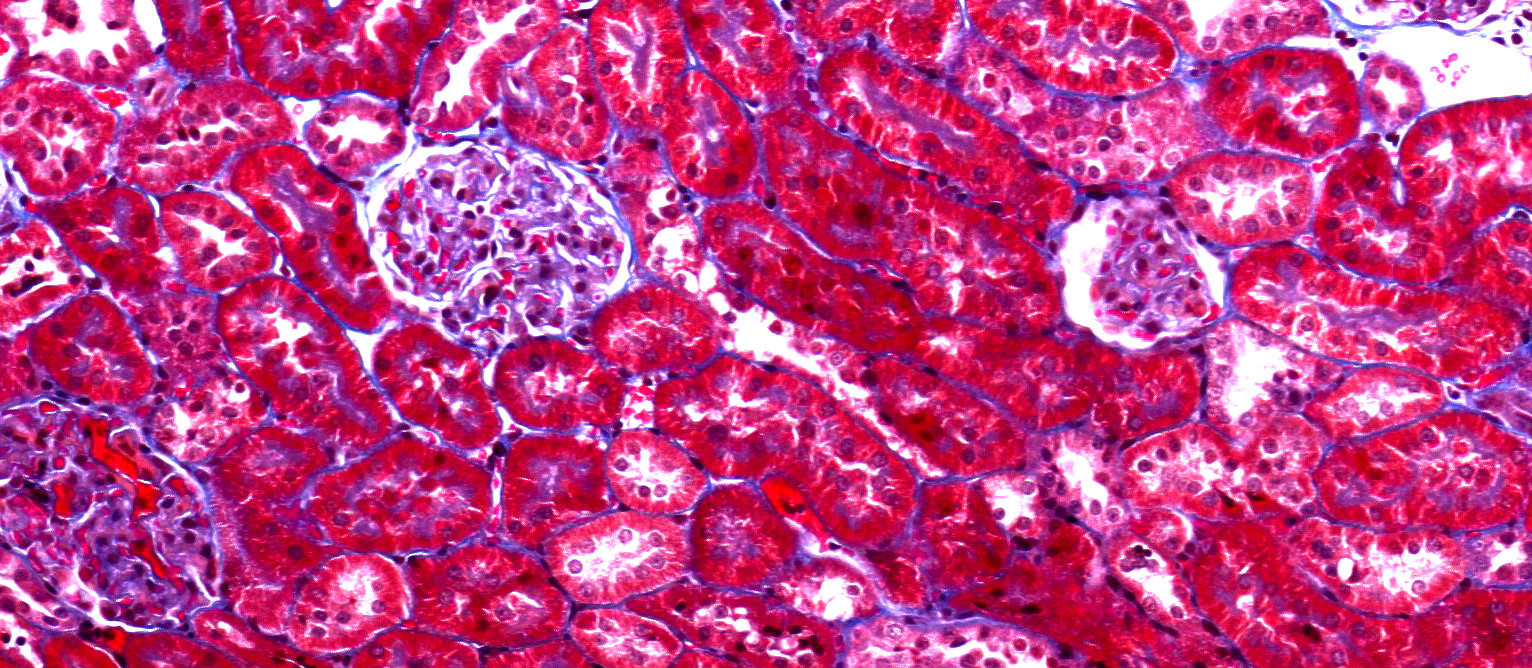

Supplement: Supplementary file 3 [file DataSheet11.ZIP › Fig 1D-masson-TSF-62(1)/62-3.jpeg]

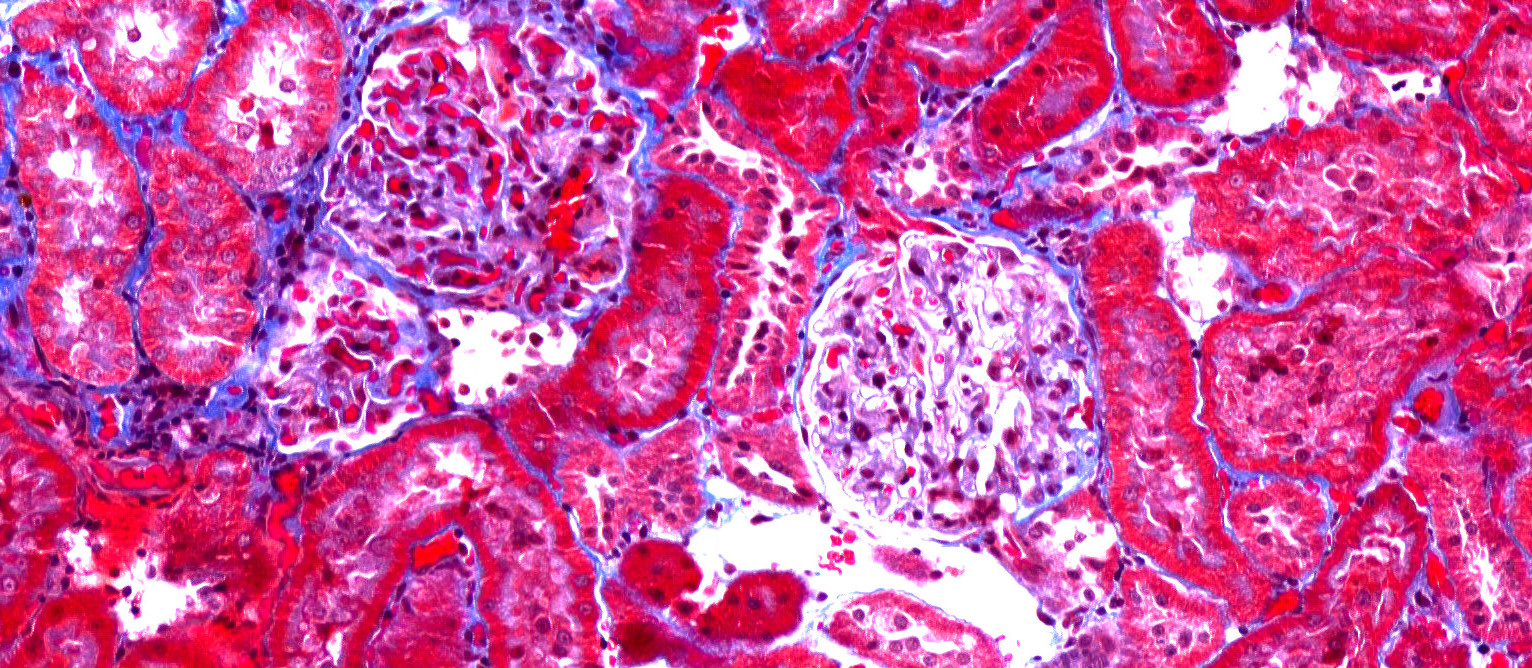

Supplement: Supplementary file 4 [file DataSheet8.ZIP › Fig 1D-masson-DKD-24/24-1.jpeg]

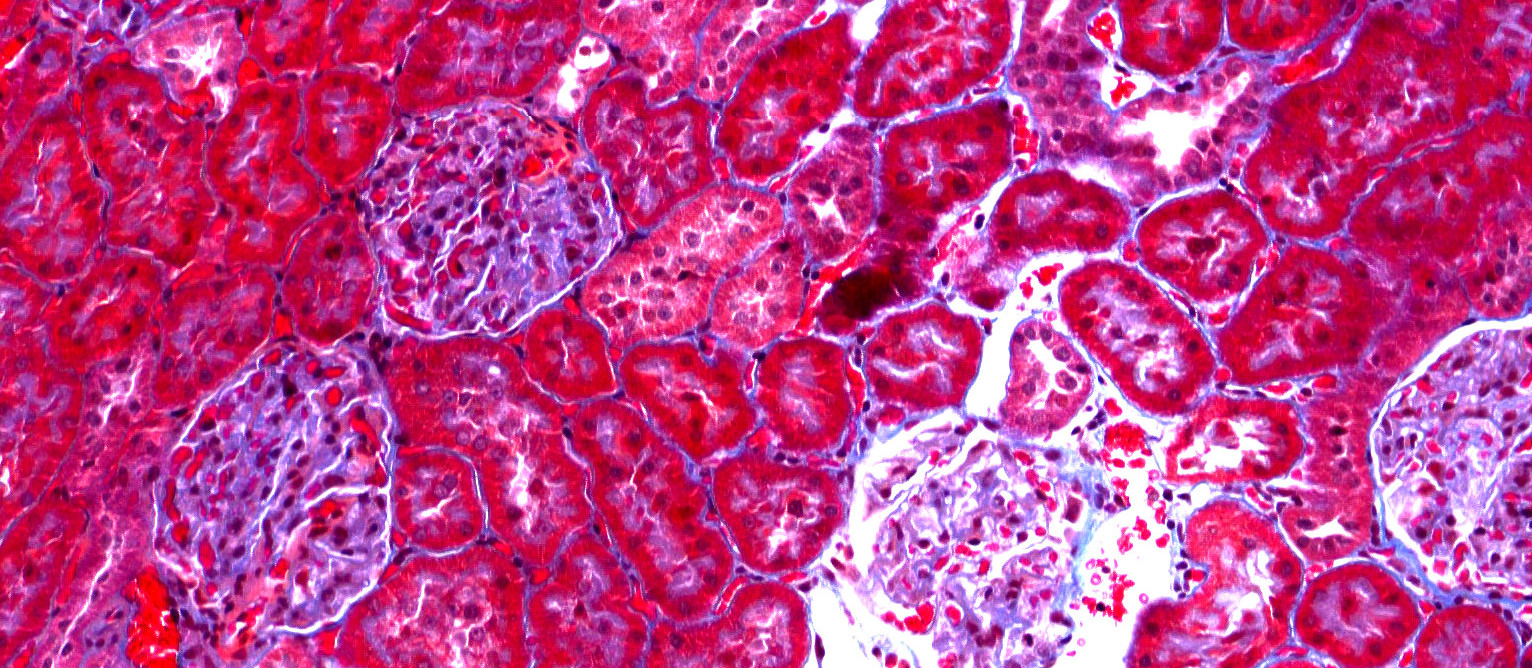

Supplement: Supplementary file 4 [file DataSheet8.ZIP › Fig 1D-masson-DKD-24/24-10.jpeg]

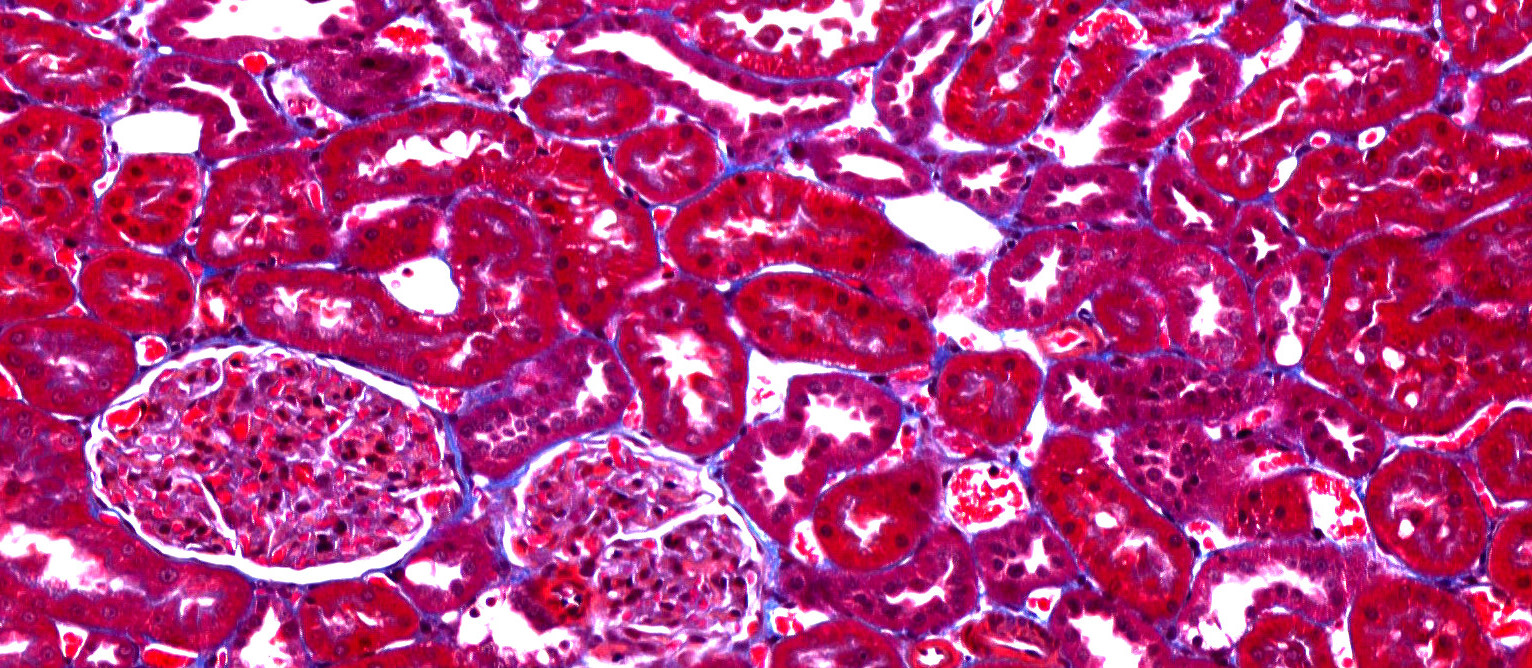

Supplement: Supplementary file 4 [file DataSheet8.ZIP › Fig 1D-masson-DKD-24/24-2.jpeg]

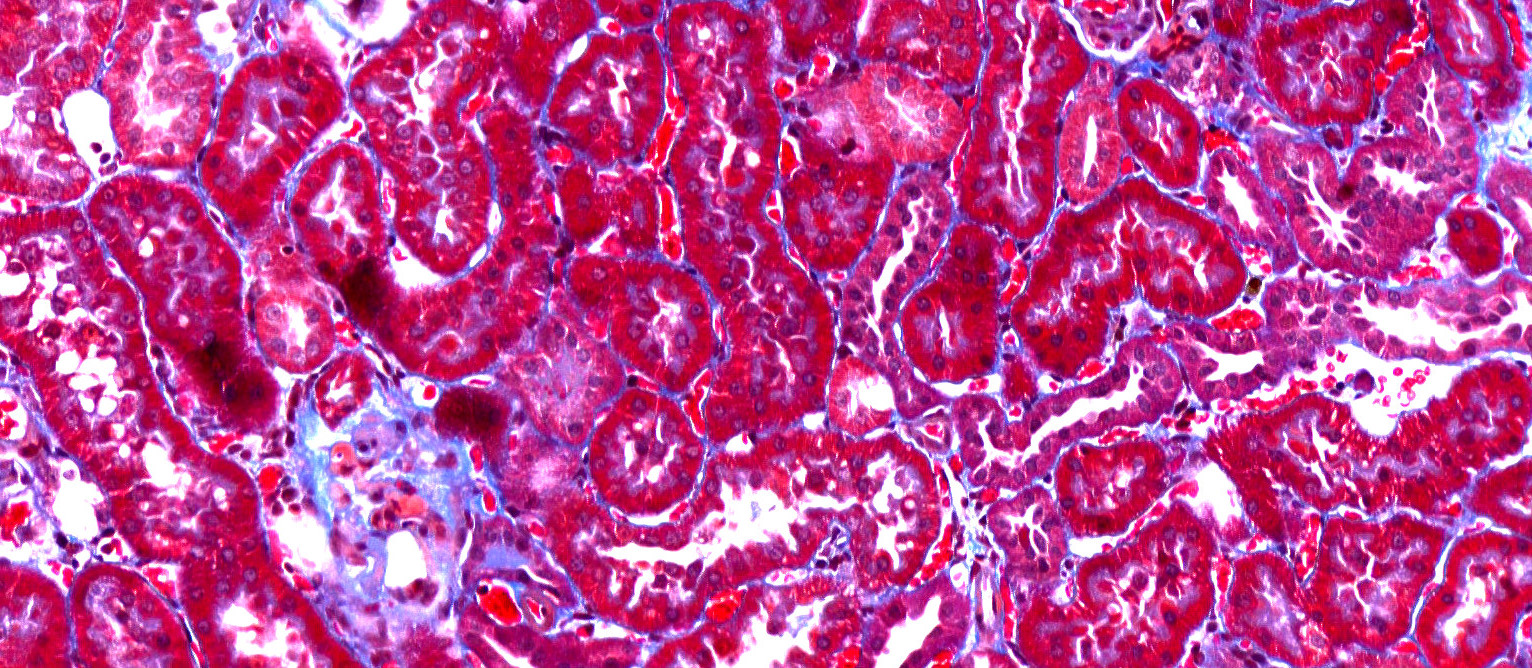

Supplement: Supplementary file 4 [file DataSheet8.ZIP › Fig 1D-masson-DKD-24/24-3.jpeg]

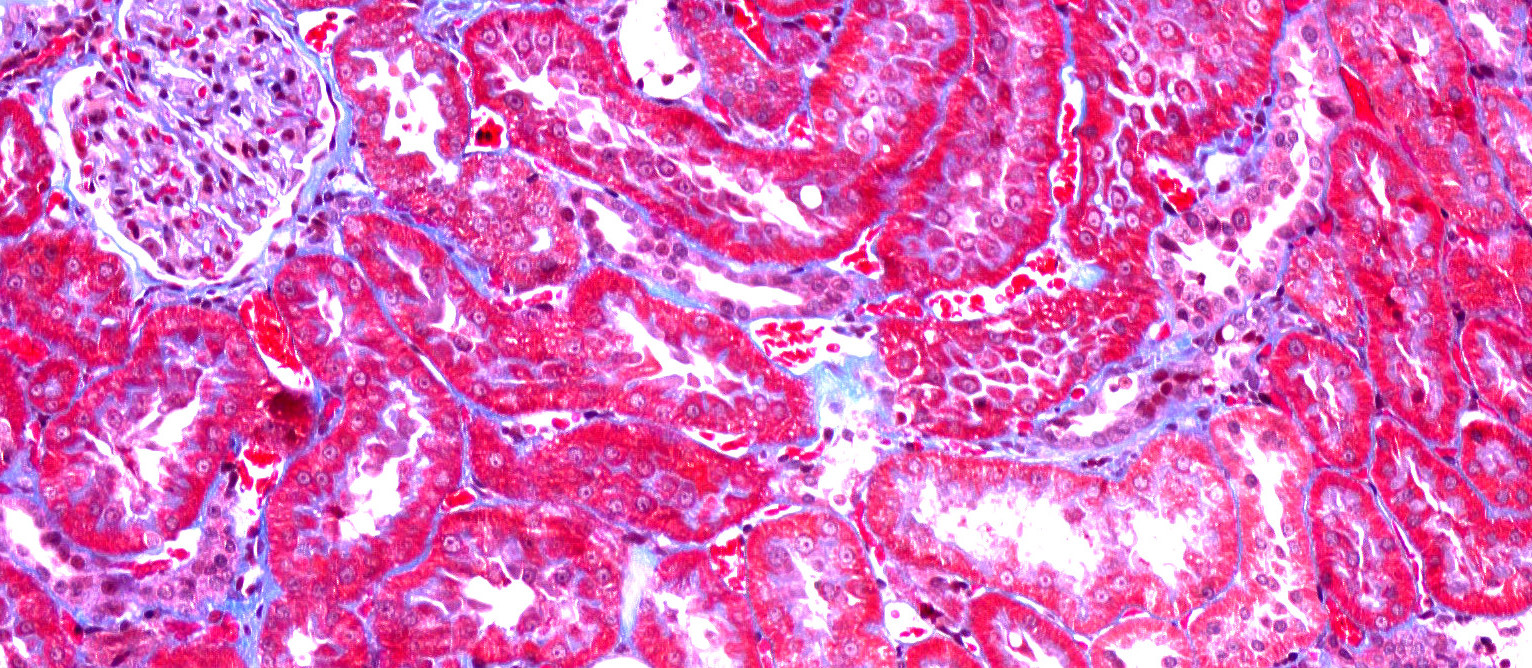

Supplement: Supplementary file 4 [file DataSheet8.ZIP › Fig 1D-masson-DKD-24/24-4.jpeg]

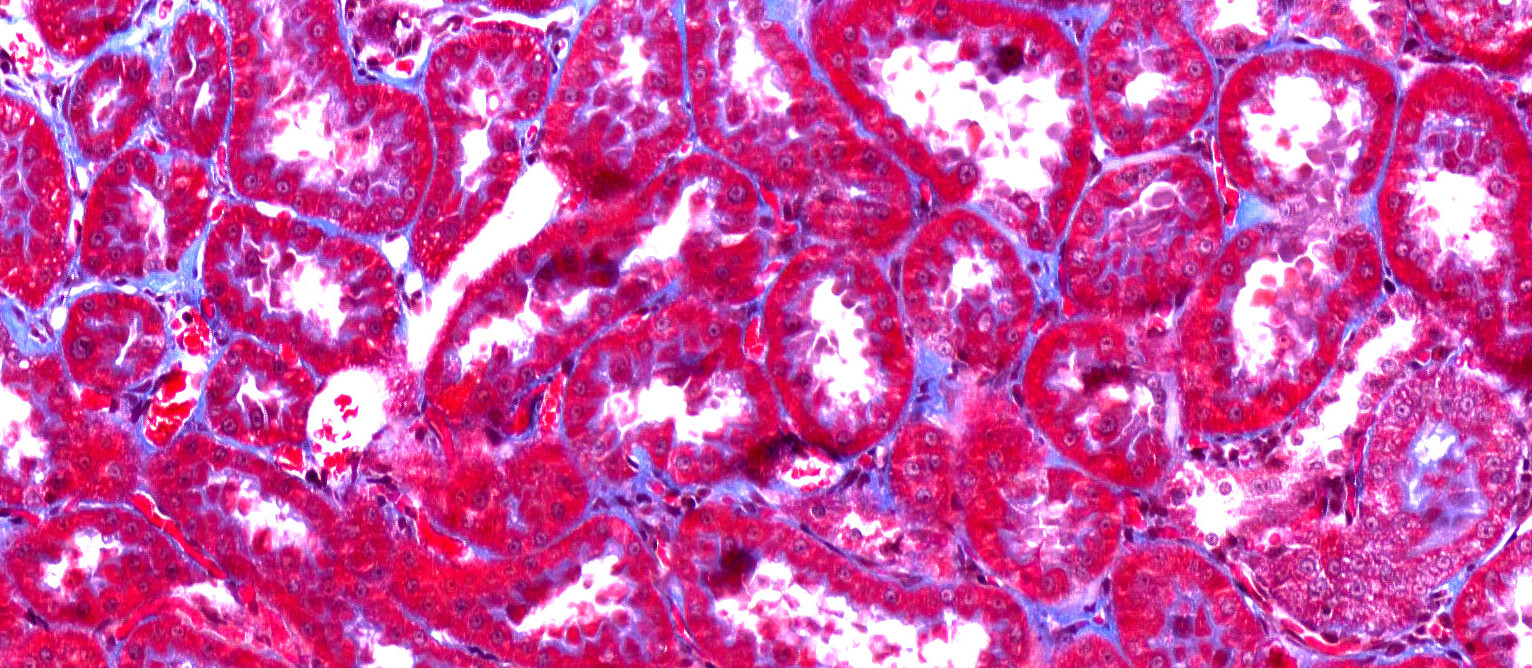

Supplement: Supplementary file 4 [file DataSheet8.ZIP › Fig 1D-masson-DKD-24/24-5.jpeg]

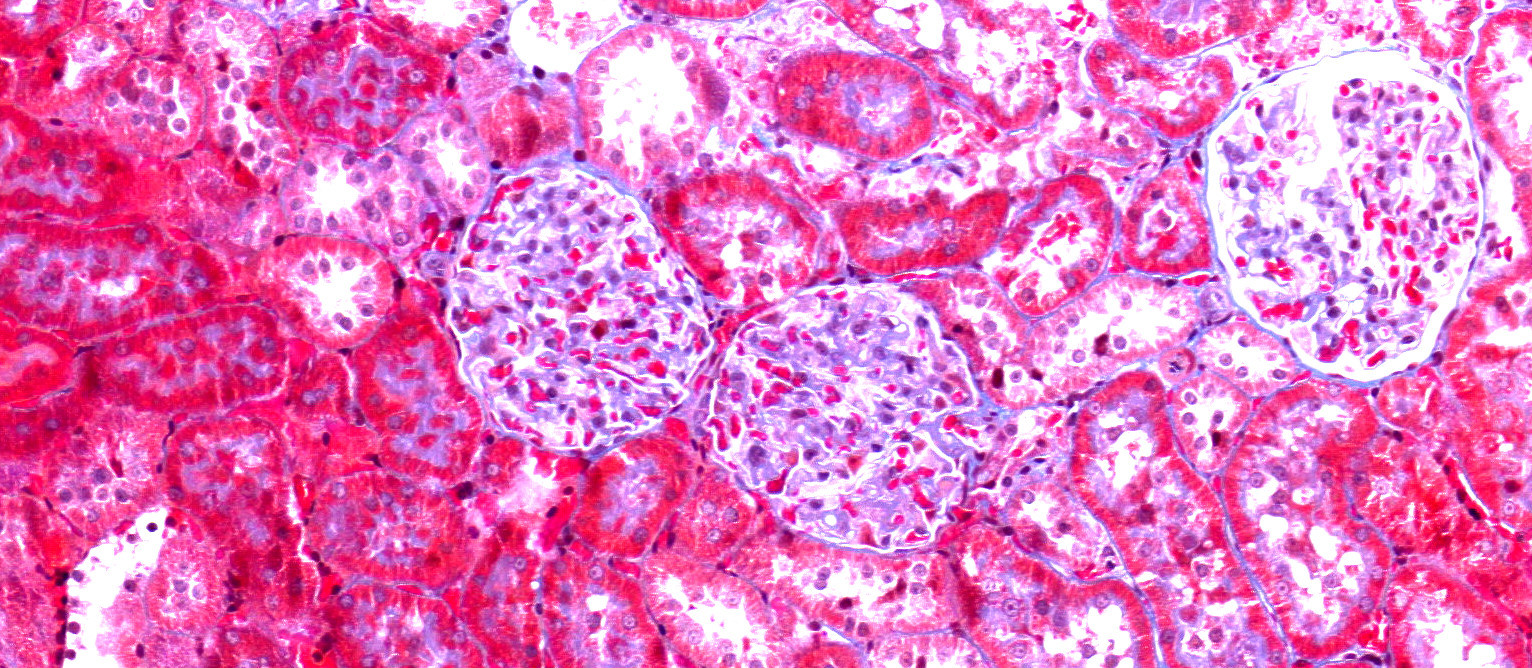

Supplement: Supplementary file 4 [file DataSheet8.ZIP › Fig 1D-masson-DKD-24/24-6.jpeg]

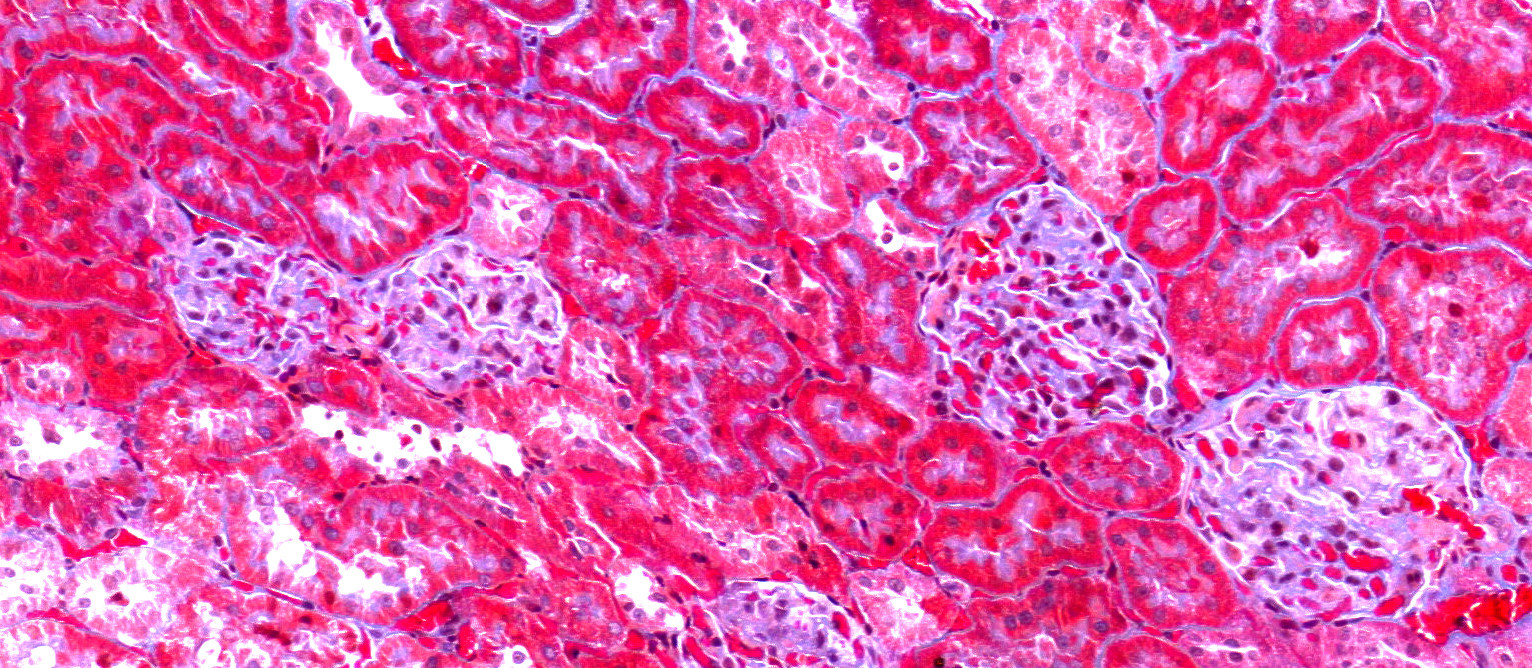

Supplement: Supplementary file 4 [file DataSheet8.ZIP › Fig 1D-masson-DKD-24/24-7.jpeg]

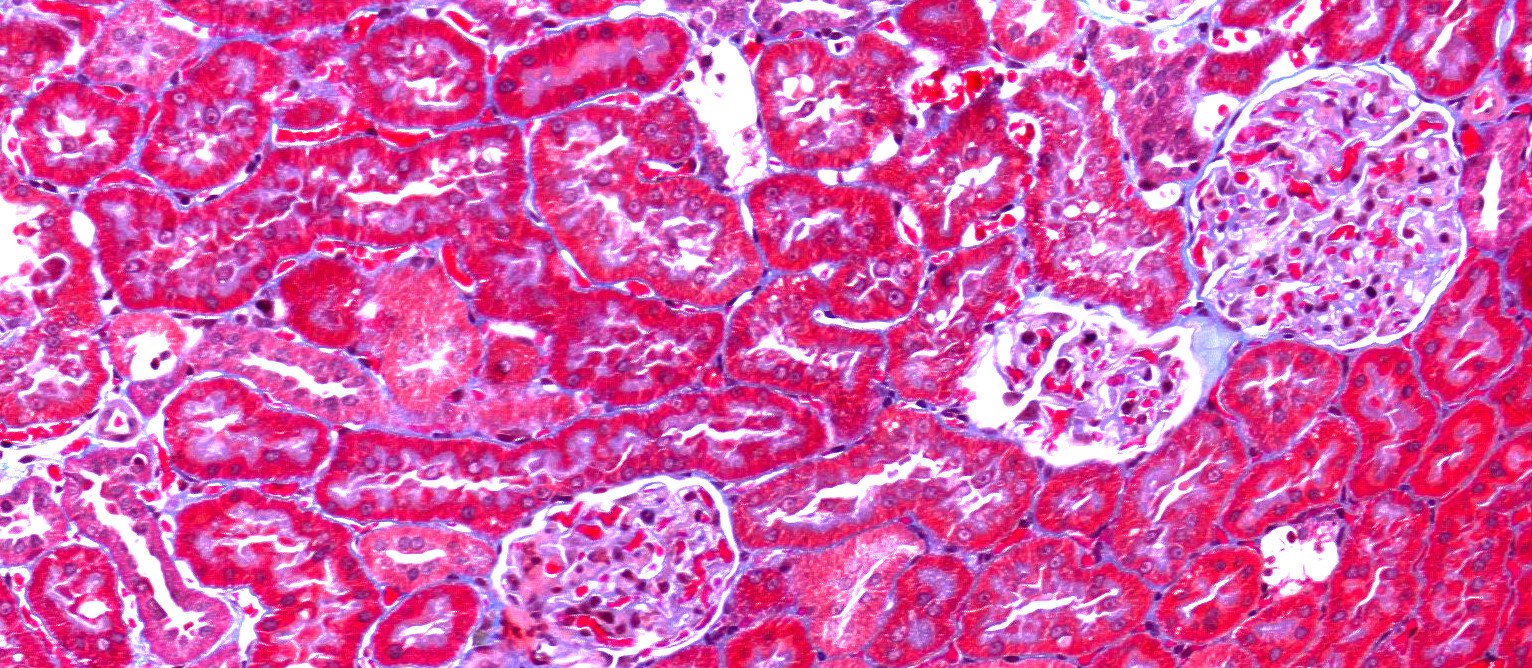

Supplement: Supplementary file 4 [file DataSheet8.ZIP › Fig 1D-masson-DKD-24/24-8.jpeg]

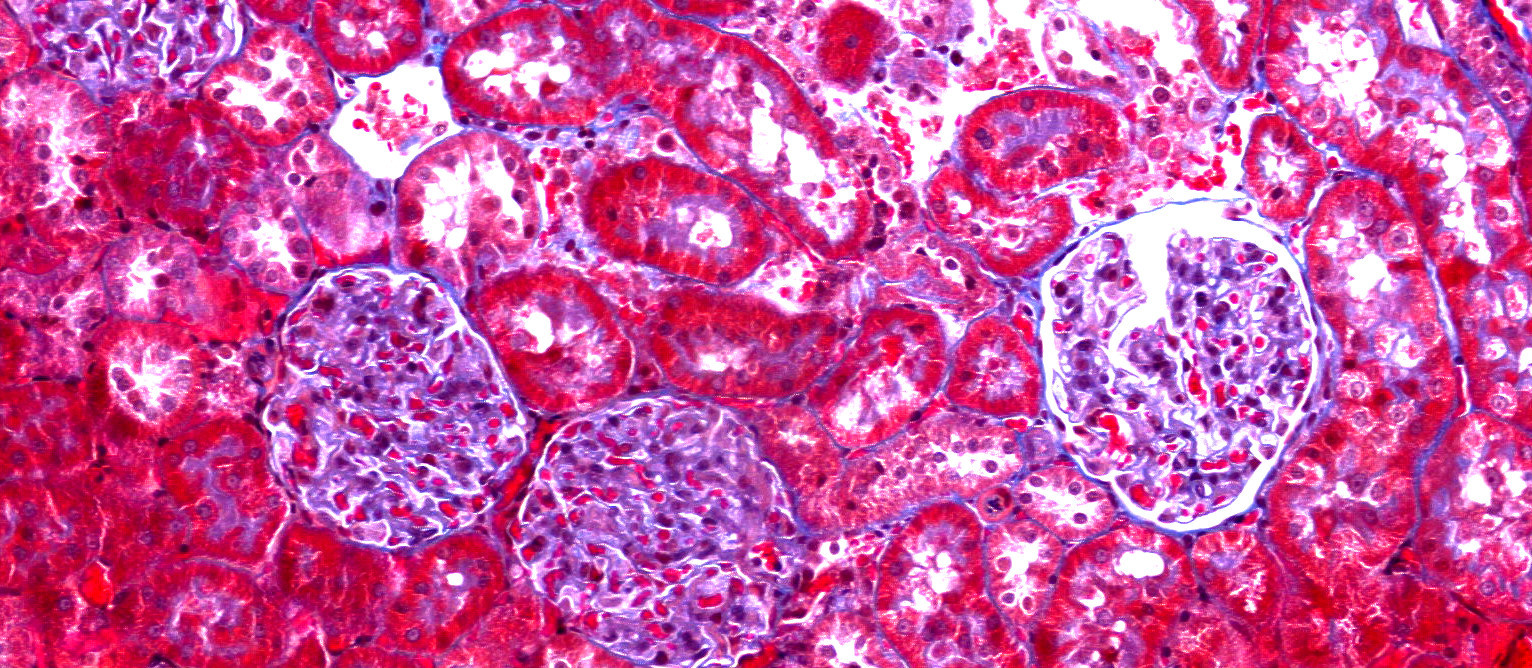

Supplement: Supplementary file 4 [file DataSheet8.ZIP › Fig 1D-masson-DKD-24/24-9.jpeg]

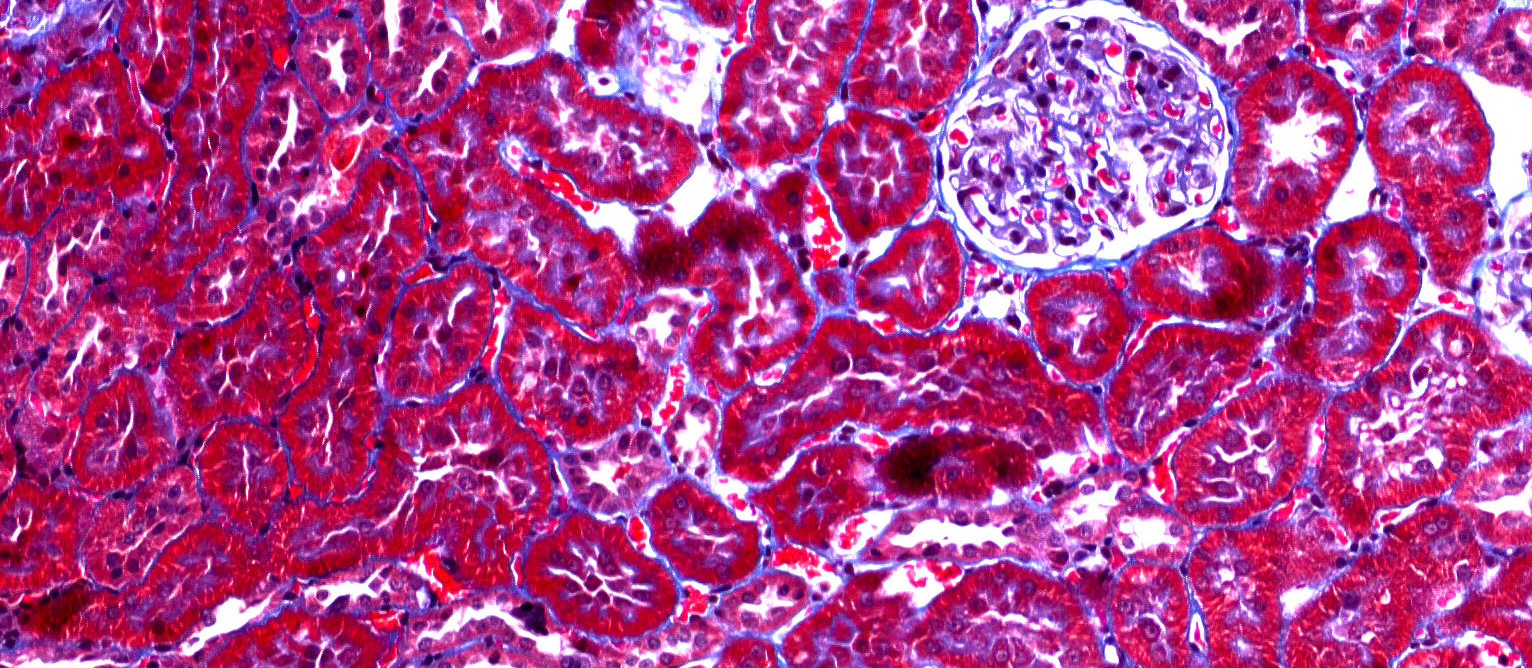

Supplement: Supplementary file 4 [file DataSheet8.ZIP › Fig 1D-masson-DKD-25/25-1.jpeg]

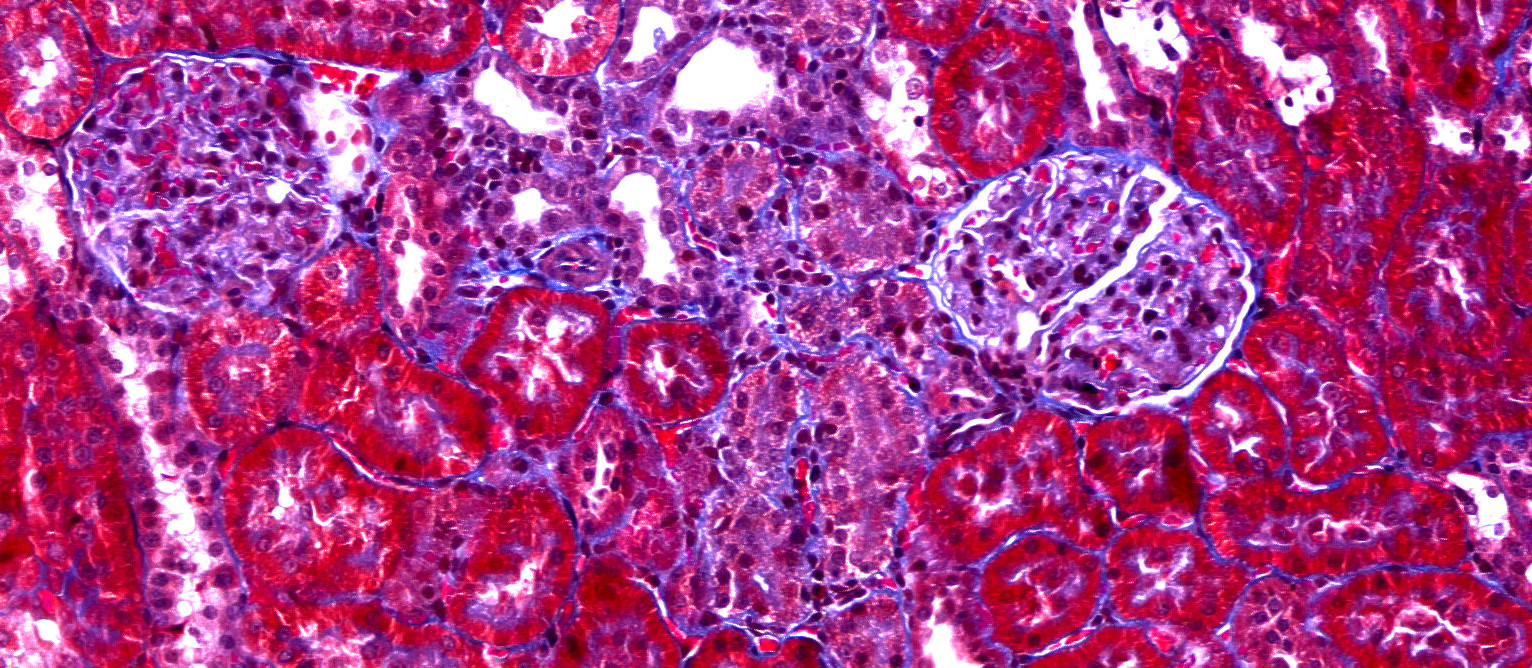

Supplement: Supplementary file 4 [file DataSheet8.ZIP › Fig 1D-masson-DKD-25/25-10.jpeg]

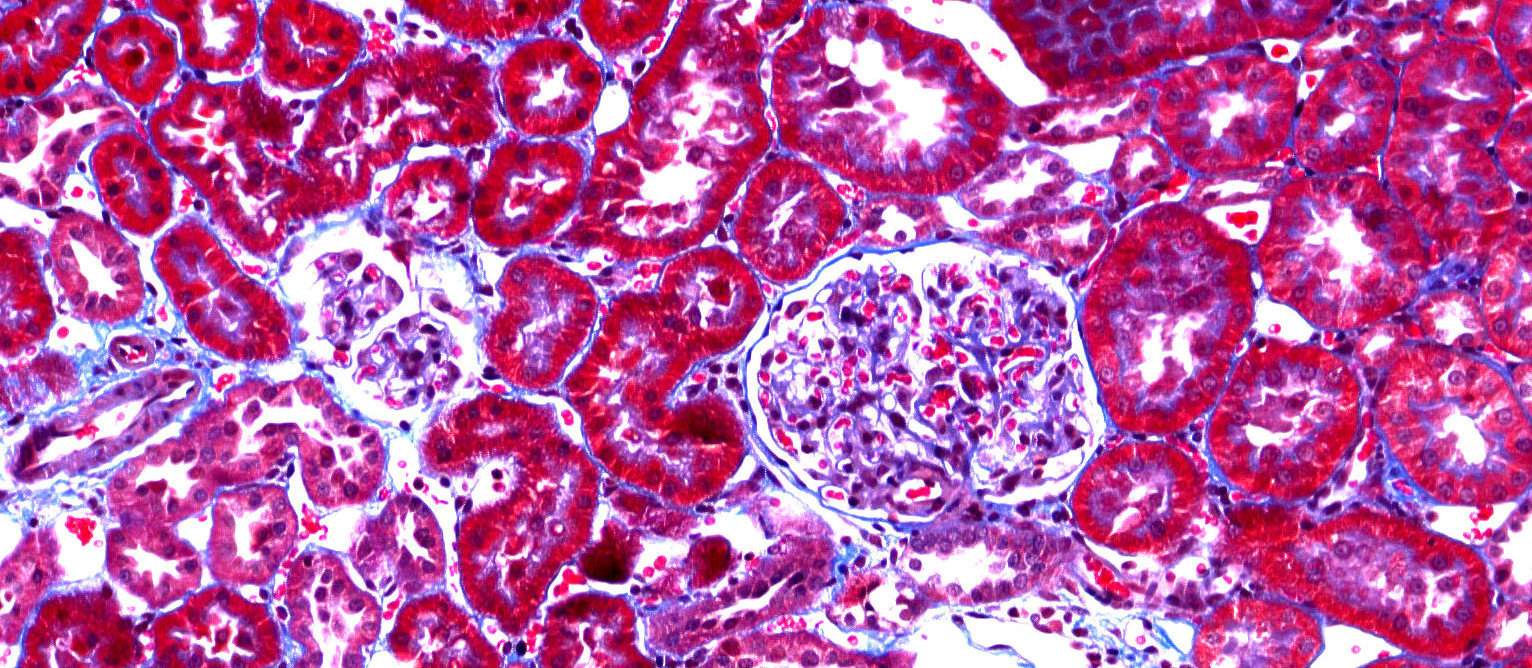

Supplement: Supplementary file 4 [file DataSheet8.ZIP › Fig 1D-masson-DKD-25/25-2.jpeg]

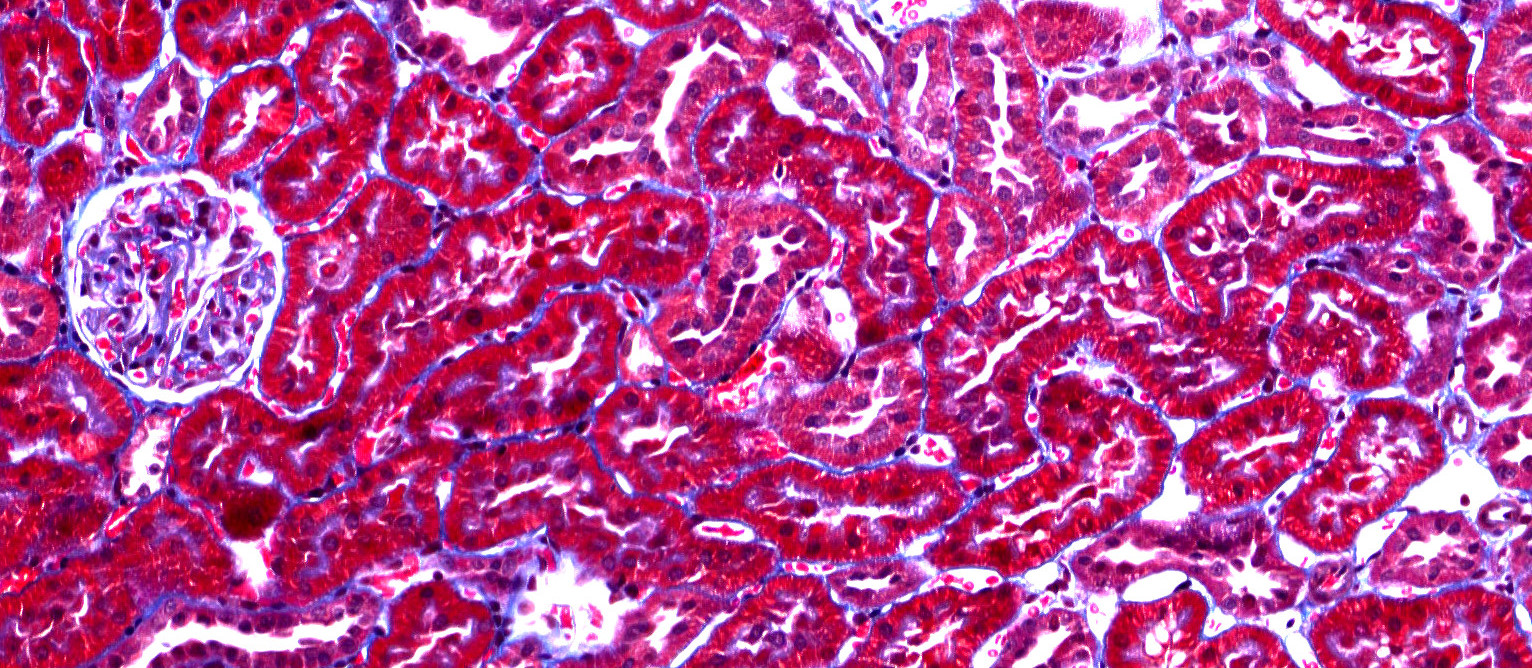

Supplement: Supplementary file 4 [file DataSheet8.ZIP › Fig 1D-masson-DKD-25/25-3.jpeg]

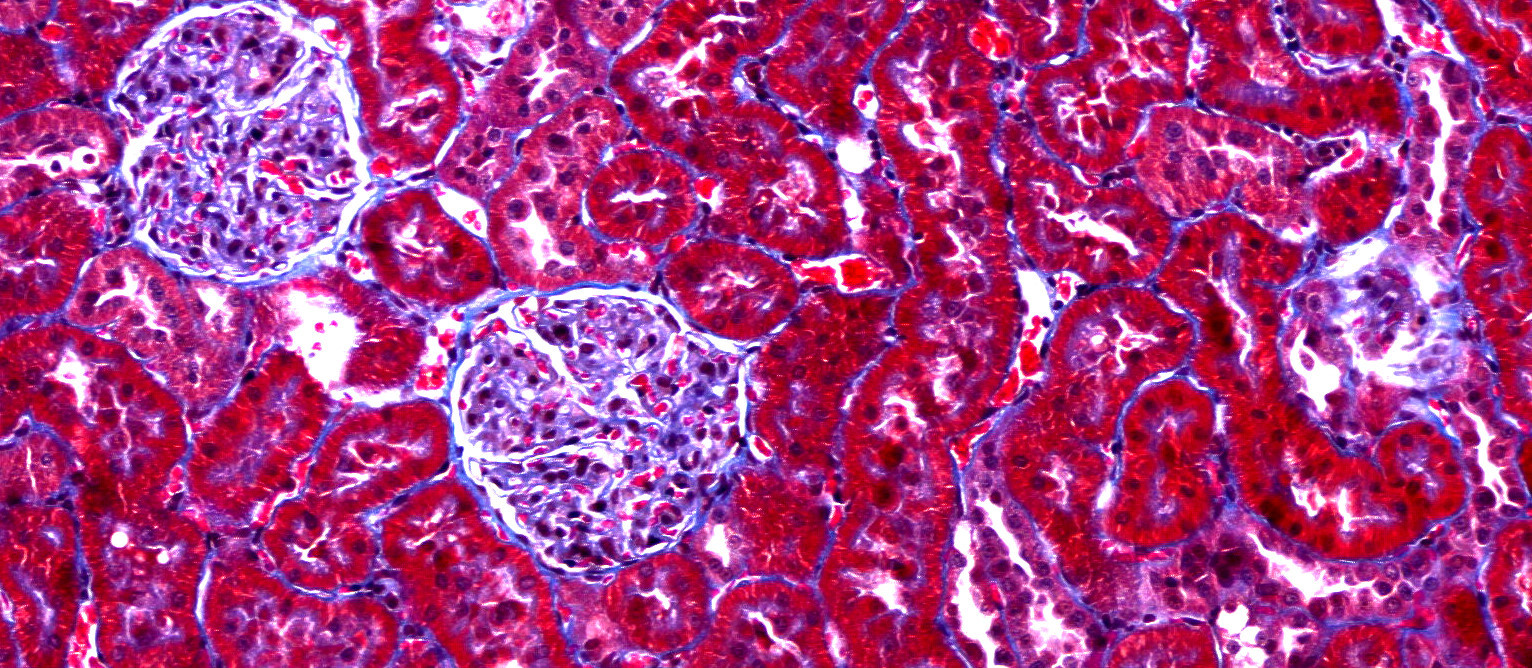

Supplement: Supplementary file 4 [file DataSheet8.ZIP › Fig 1D-masson-DKD-25/25-4.jpeg]

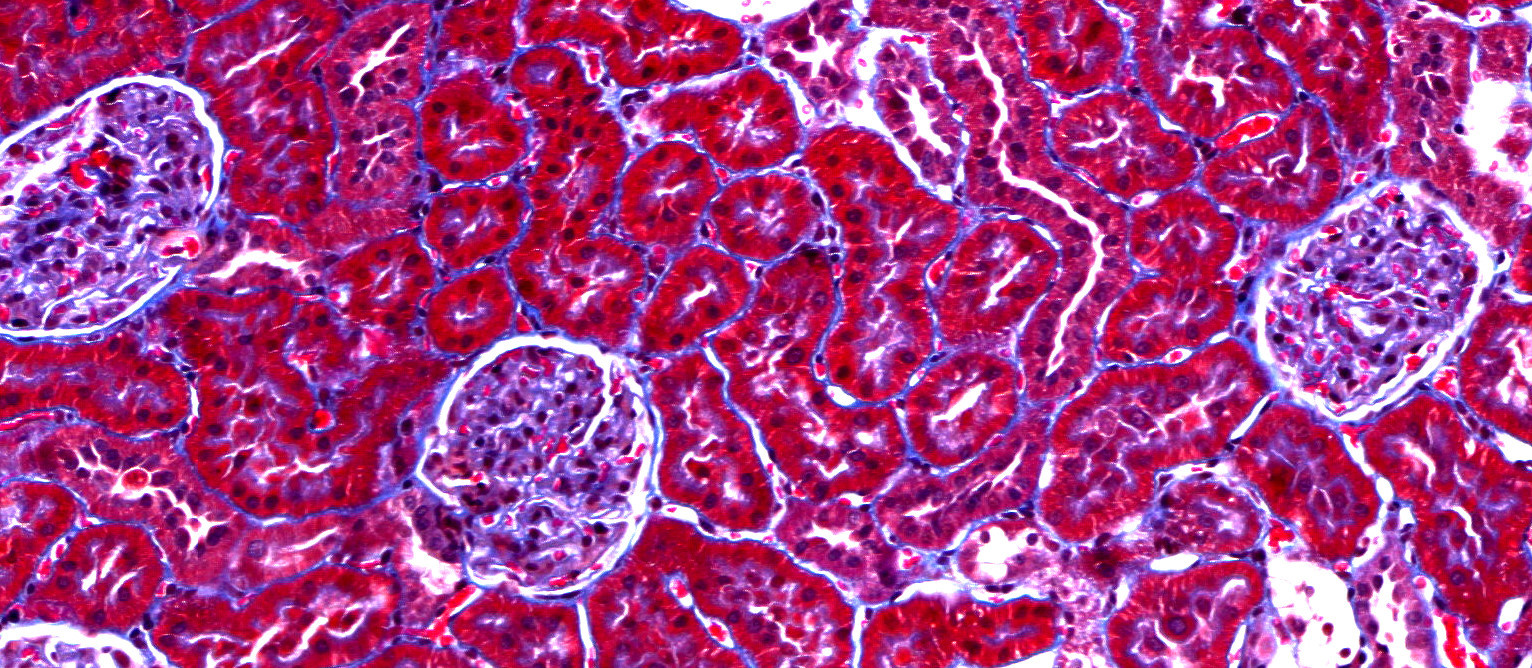

Supplement: Supplementary file 4 [file DataSheet8.ZIP › Fig 1D-masson-DKD-25/25-5.jpeg]

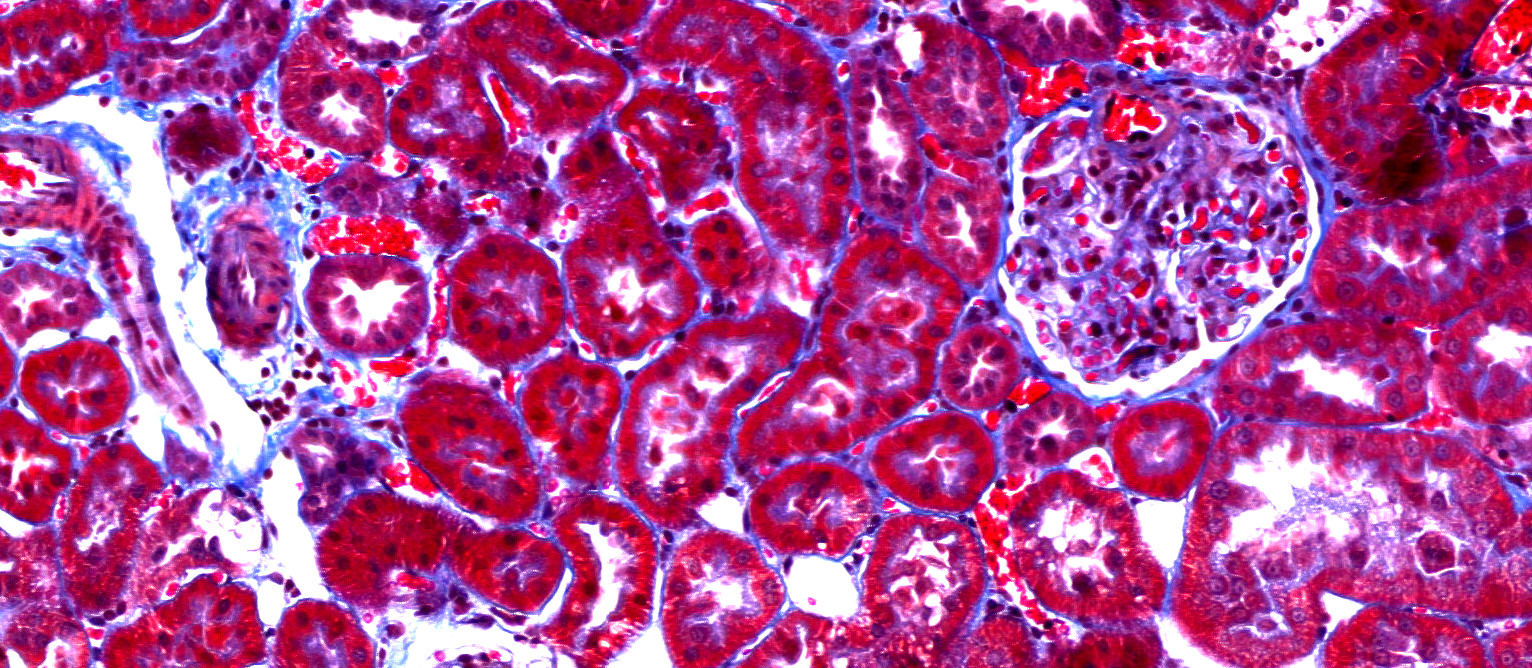

Supplement: Supplementary file 4 [file DataSheet8.ZIP › Fig 1D-masson-DKD-25/25-6.jpeg]

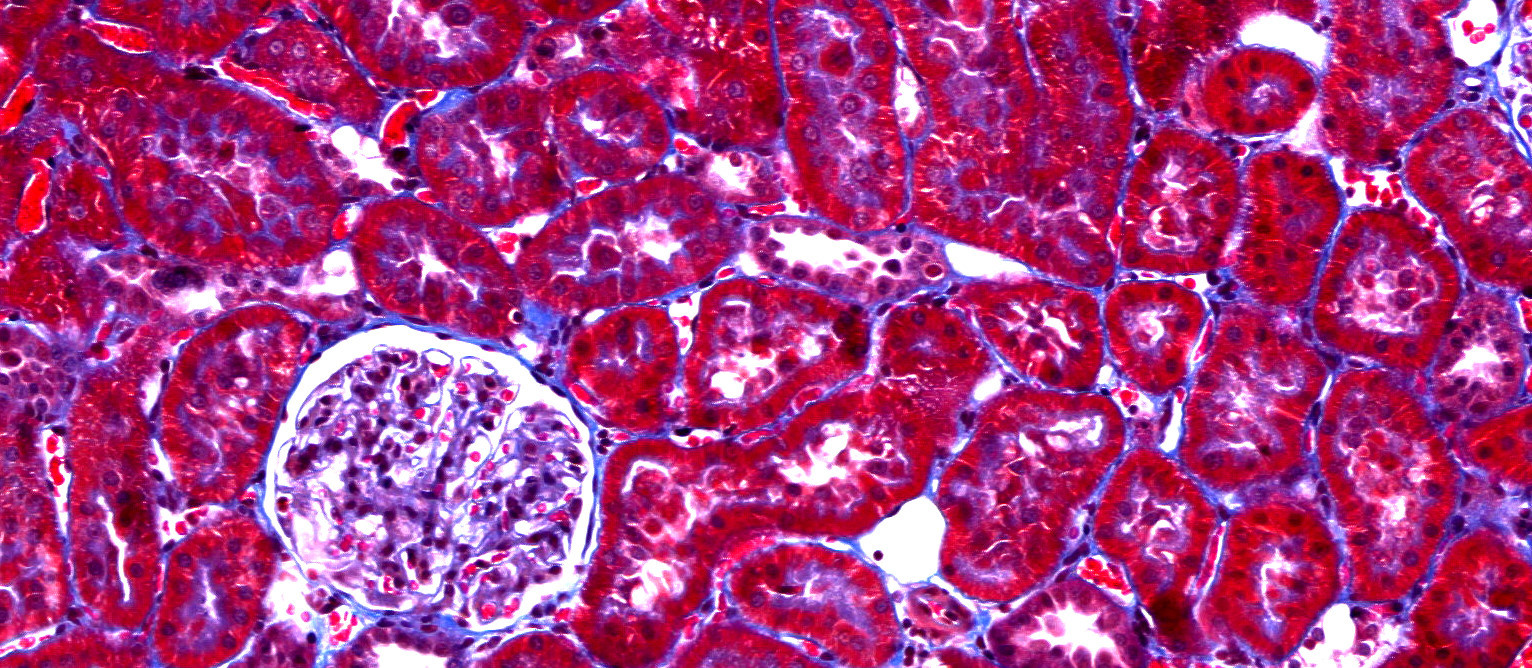

Supplement: Supplementary file 4 [file DataSheet8.ZIP › Fig 1D-masson-DKD-25/25-7.jpeg]

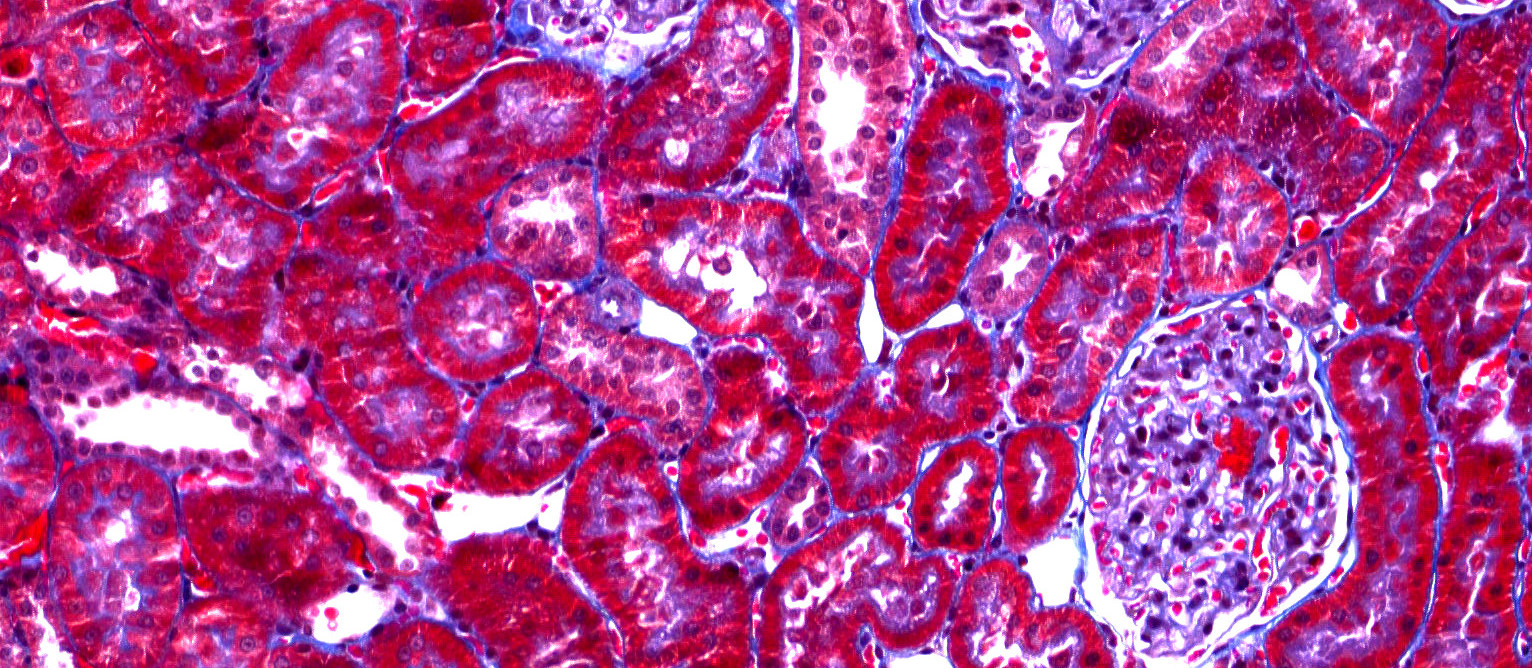

Supplement: Supplementary file 4 [file DataSheet8.ZIP › Fig 1D-masson-DKD-25/25-8.jpeg]

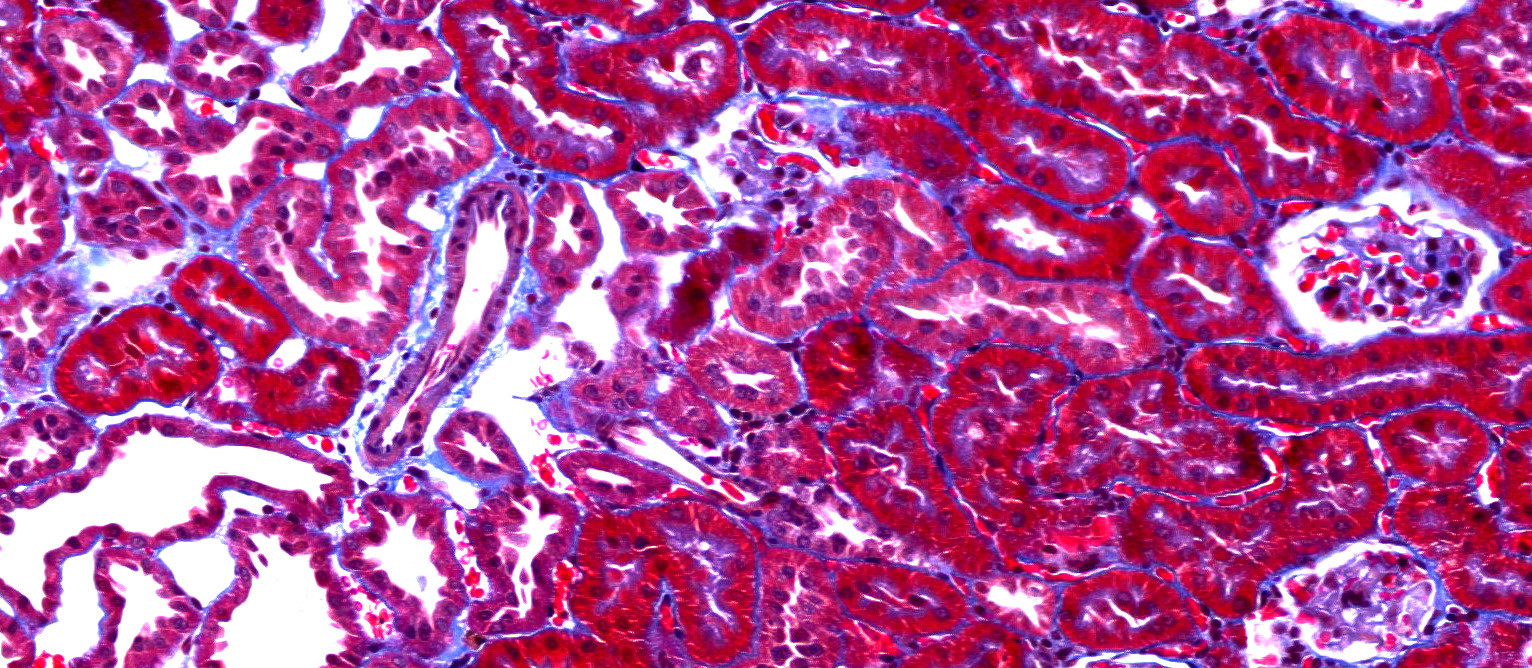

Supplement: Supplementary file 4 [file DataSheet8.ZIP › Fig 1D-masson-DKD-25/25-9.jpeg]

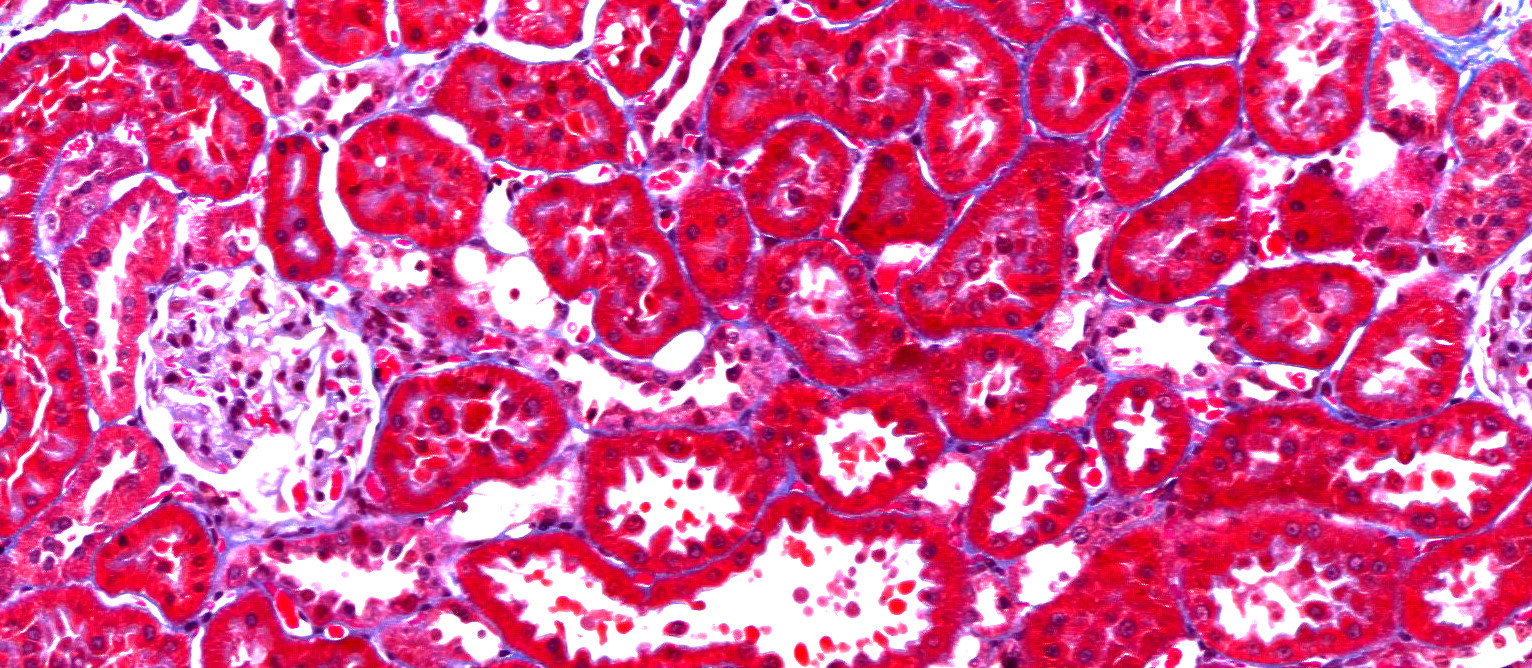

Supplement: Supplementary file 4 [file DataSheet8.ZIP › Fig 1D-masson-TSF-53/53-1.jpeg]

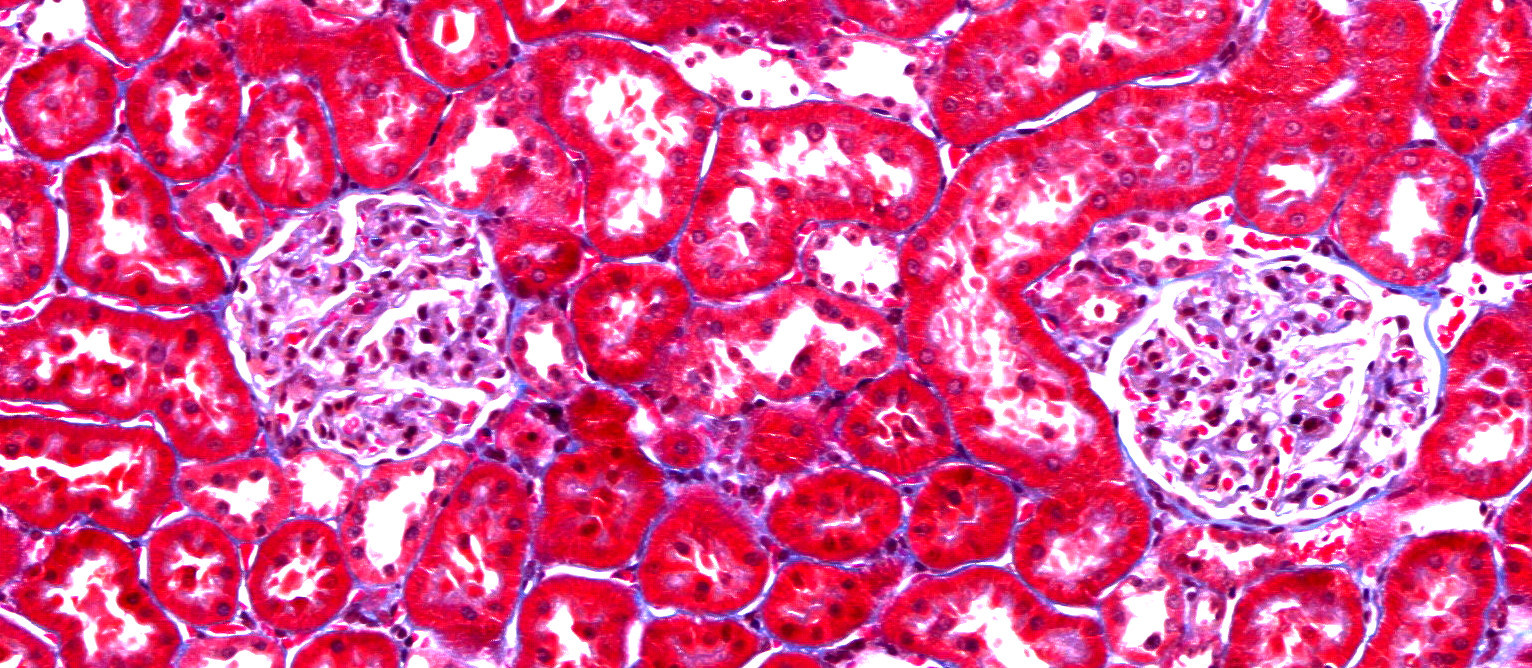

Supplement: Supplementary file 4 [file DataSheet8.ZIP › Fig 1D-masson-TSF-53/53-10.jpeg]

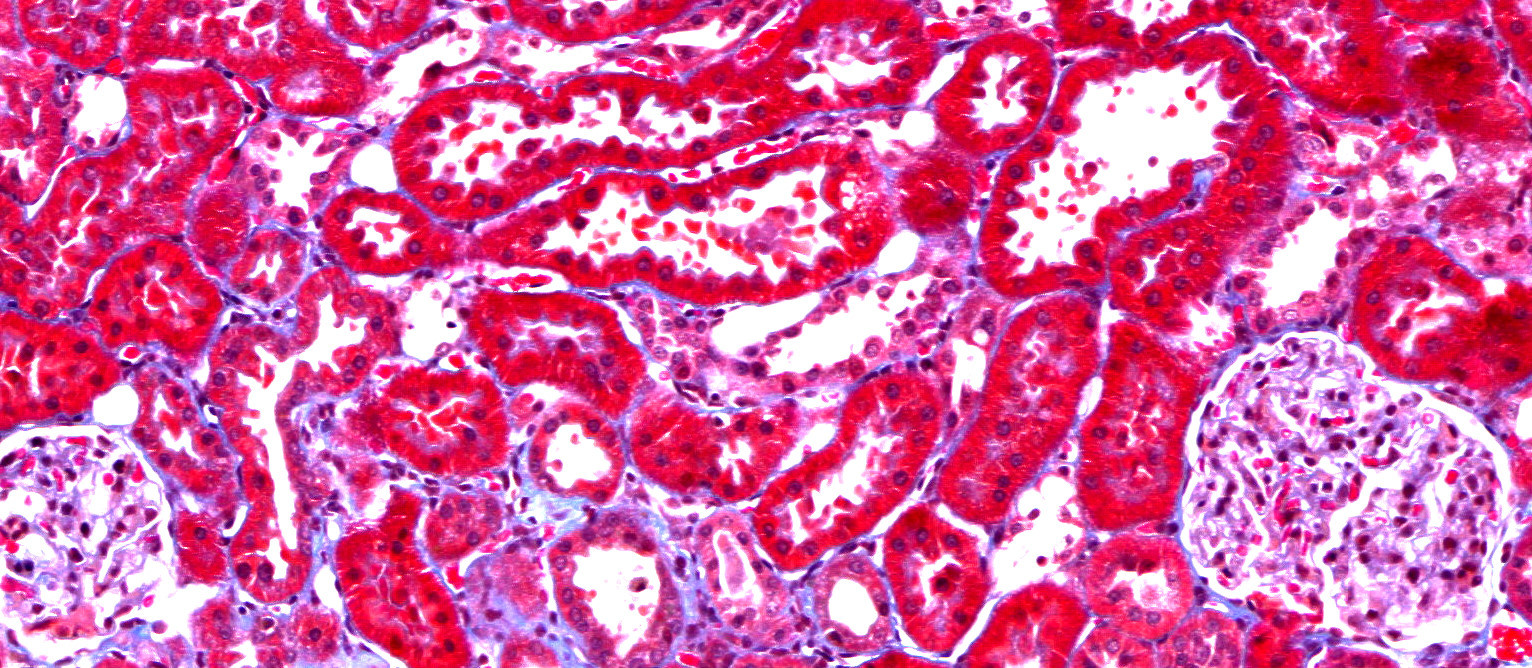

Supplement: Supplementary file 4 [file DataSheet8.ZIP › Fig 1D-masson-TSF-53/53-2.jpeg]

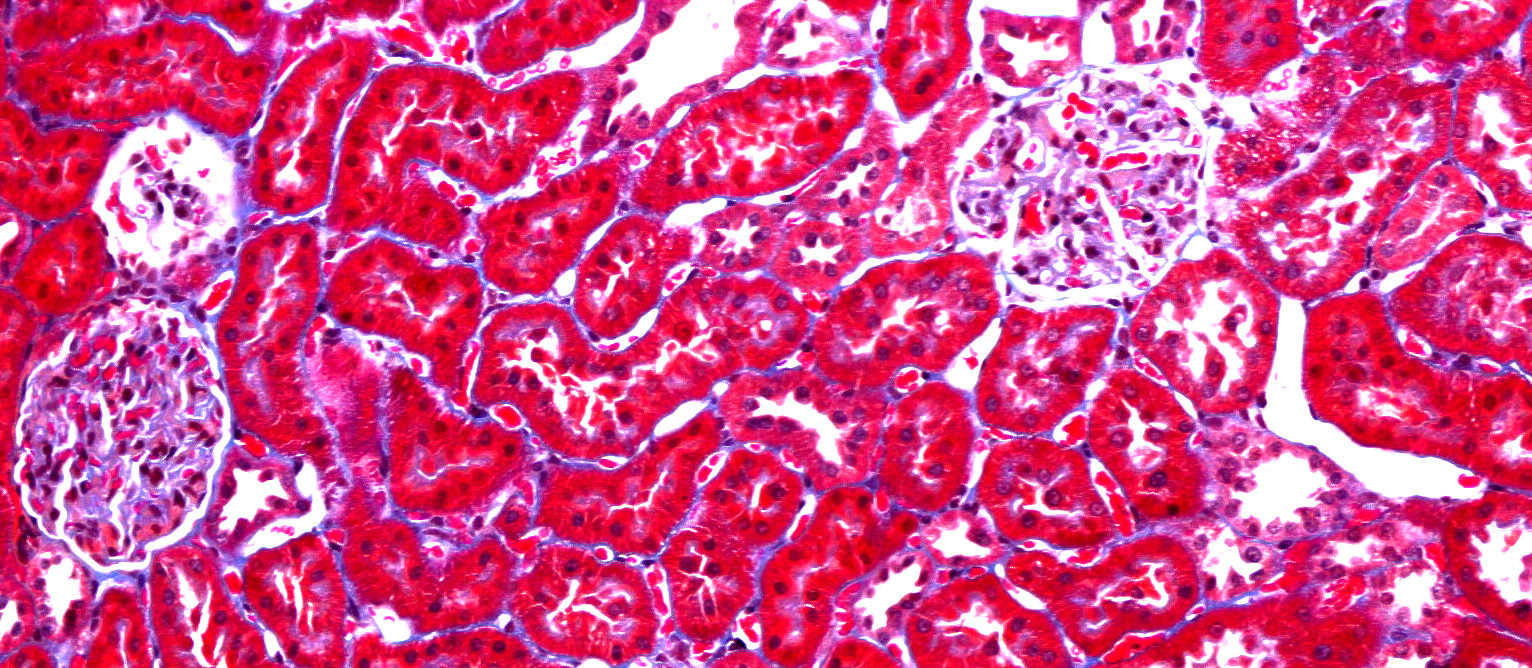

Supplement: Supplementary file 4 [file DataSheet8.ZIP › Fig 1D-masson-TSF-53/53-3.jpeg]

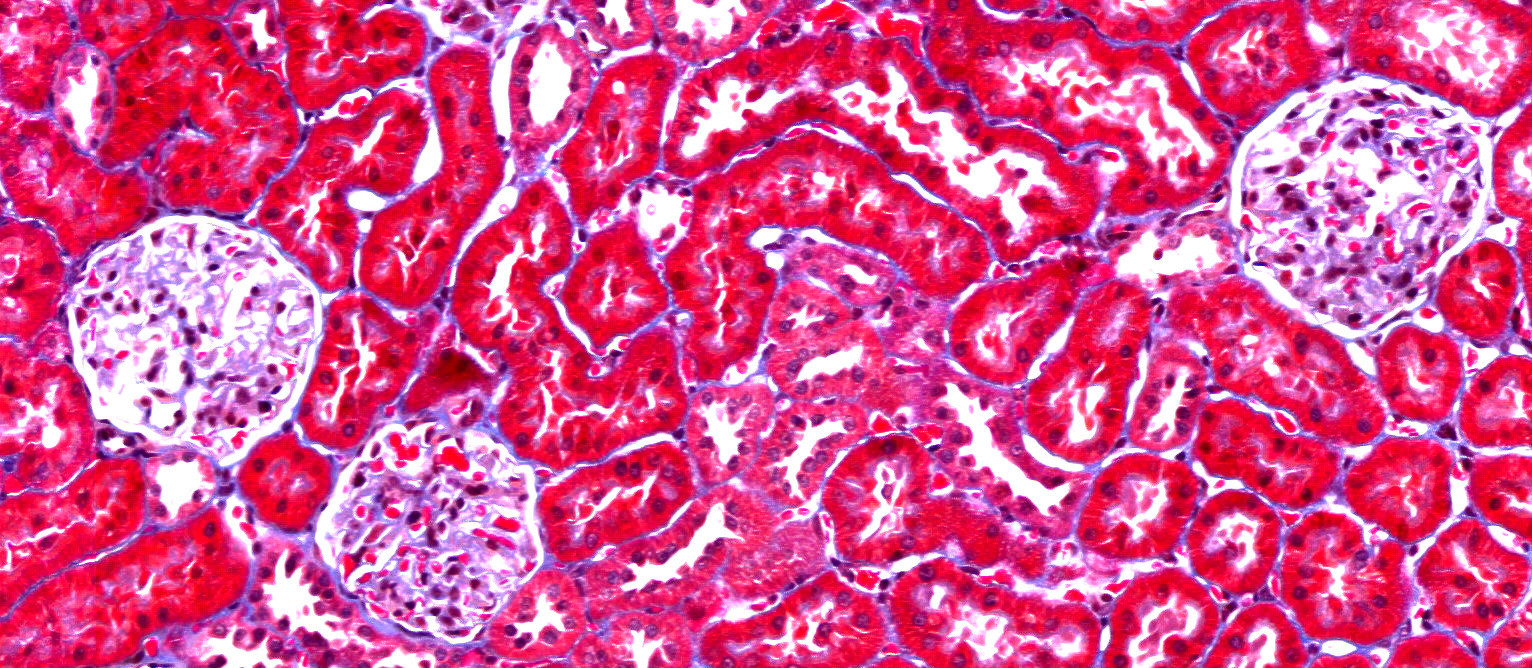

Supplement: Supplementary file 4 [file DataSheet8.ZIP › Fig 1D-masson-TSF-53/53-4.jpeg]

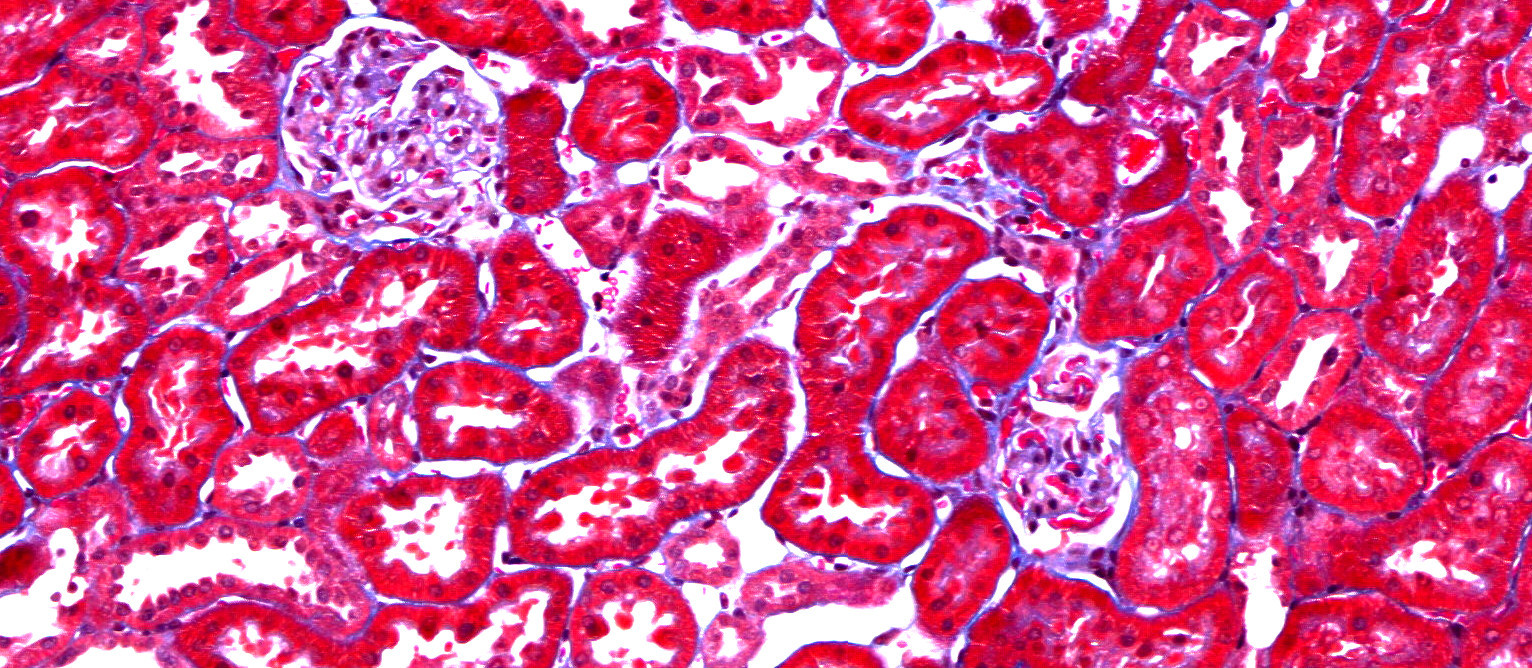

Supplement: Supplementary file 4 [file DataSheet8.ZIP › Fig 1D-masson-TSF-53/53-5.jpeg]

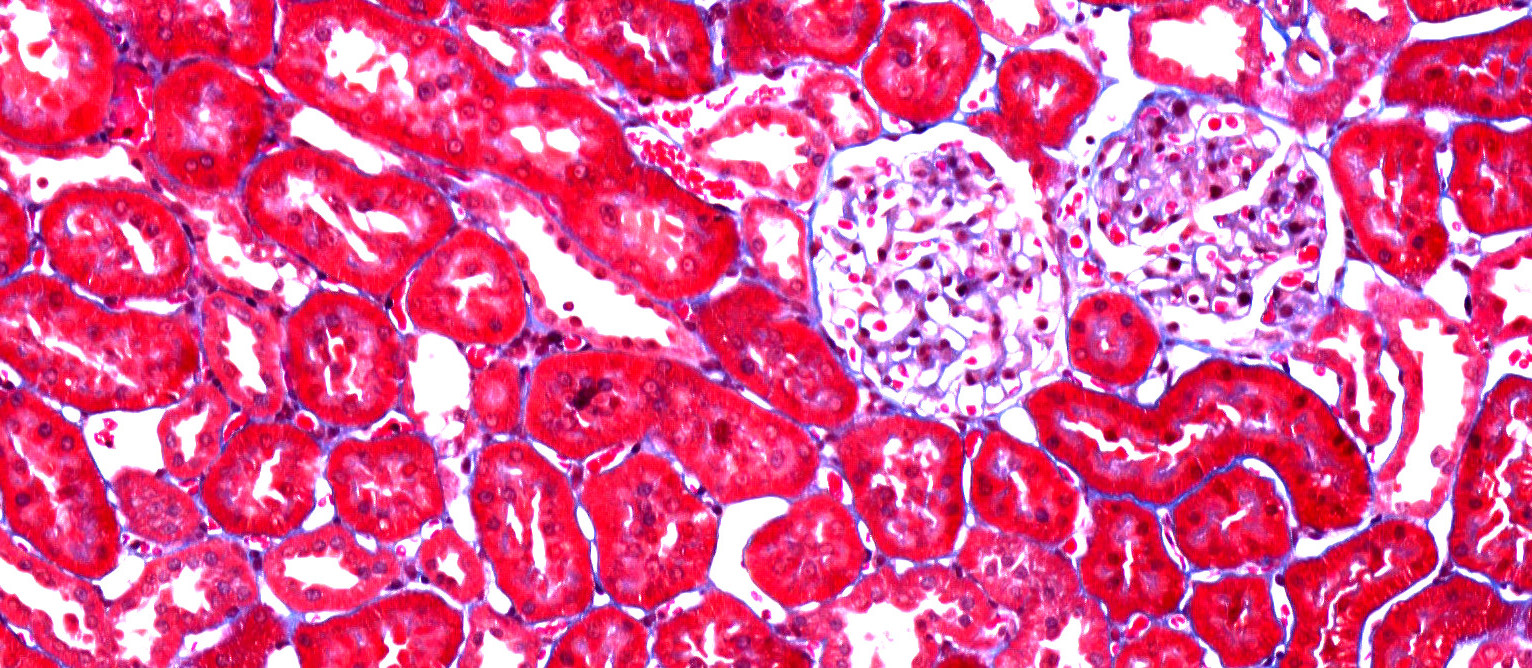

Supplement: Supplementary file 4 [file DataSheet8.ZIP › Fig 1D-masson-TSF-53/53-6.jpeg]

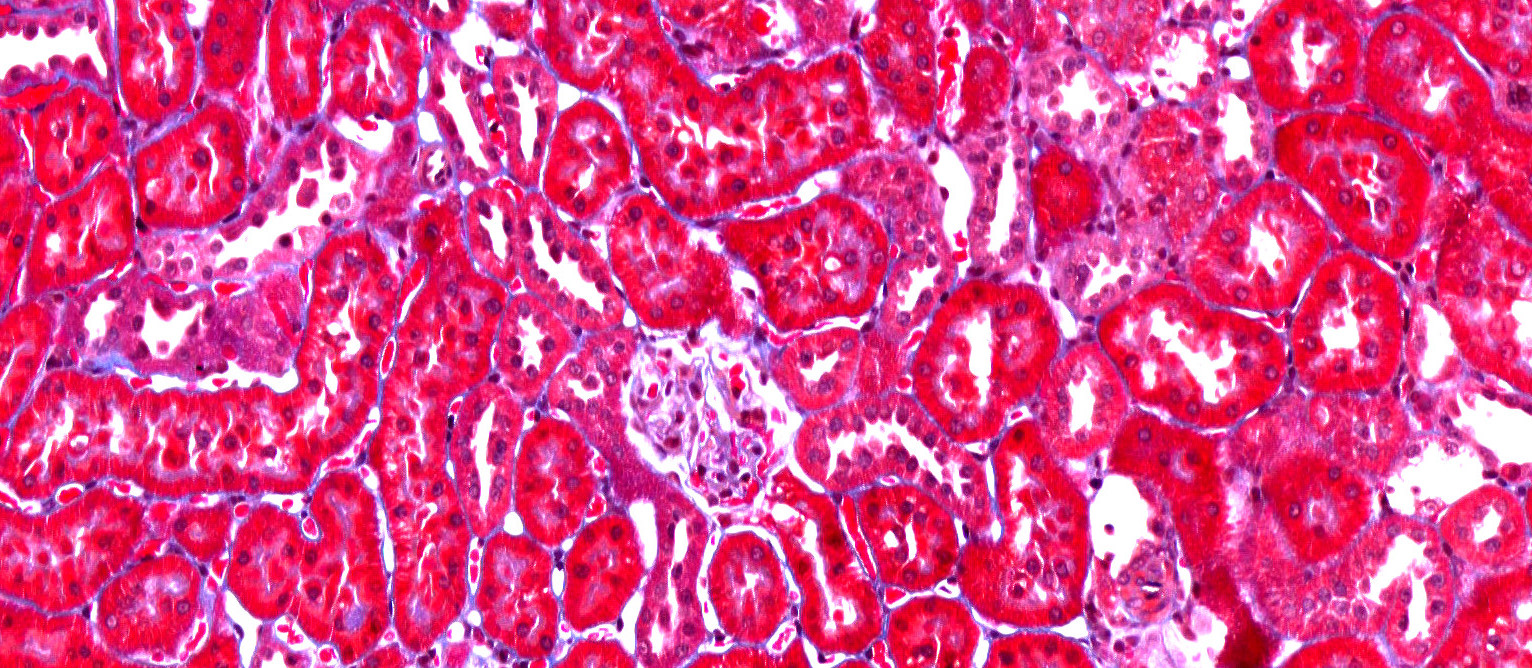

Supplement: Supplementary file 4 [file DataSheet8.ZIP › Fig 1D-masson-TSF-53/53-7.jpeg]

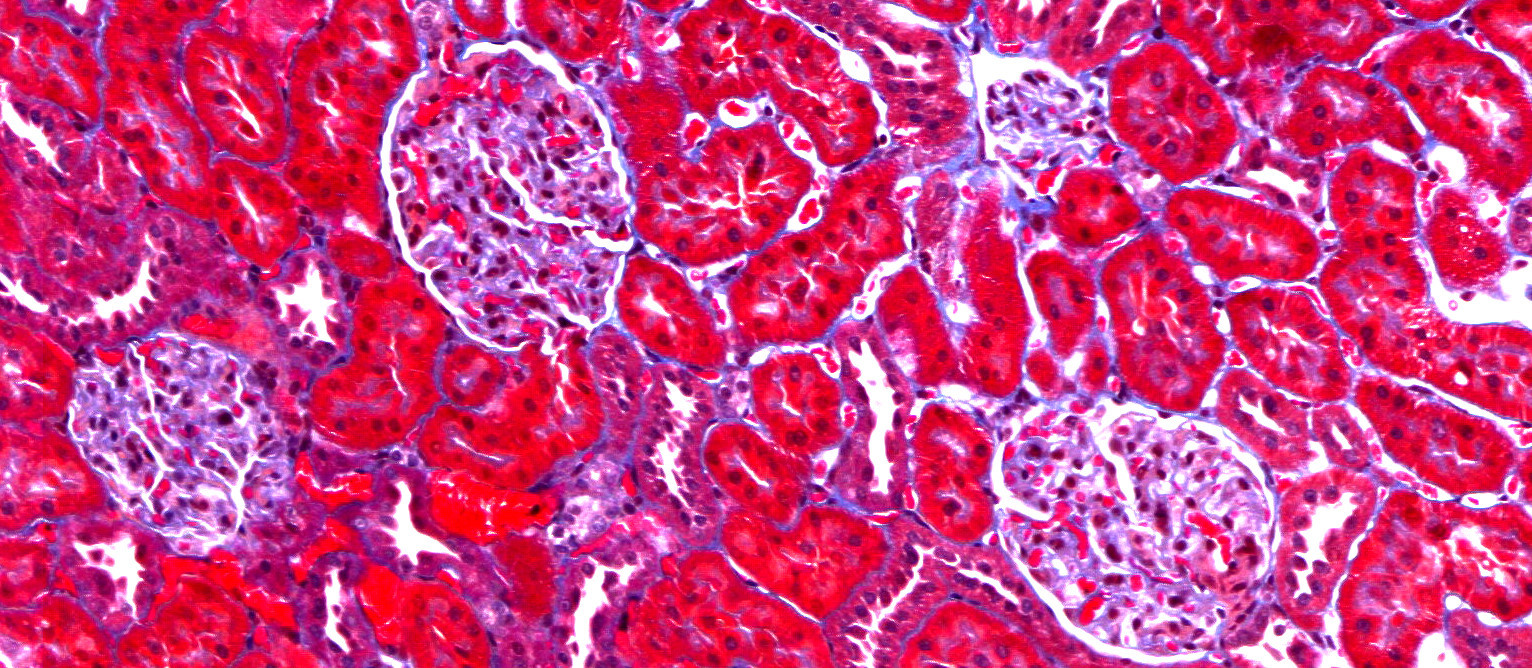

Supplement: Supplementary file 4 [file DataSheet8.ZIP › Fig 1D-masson-TSF-53/53-8.jpeg]

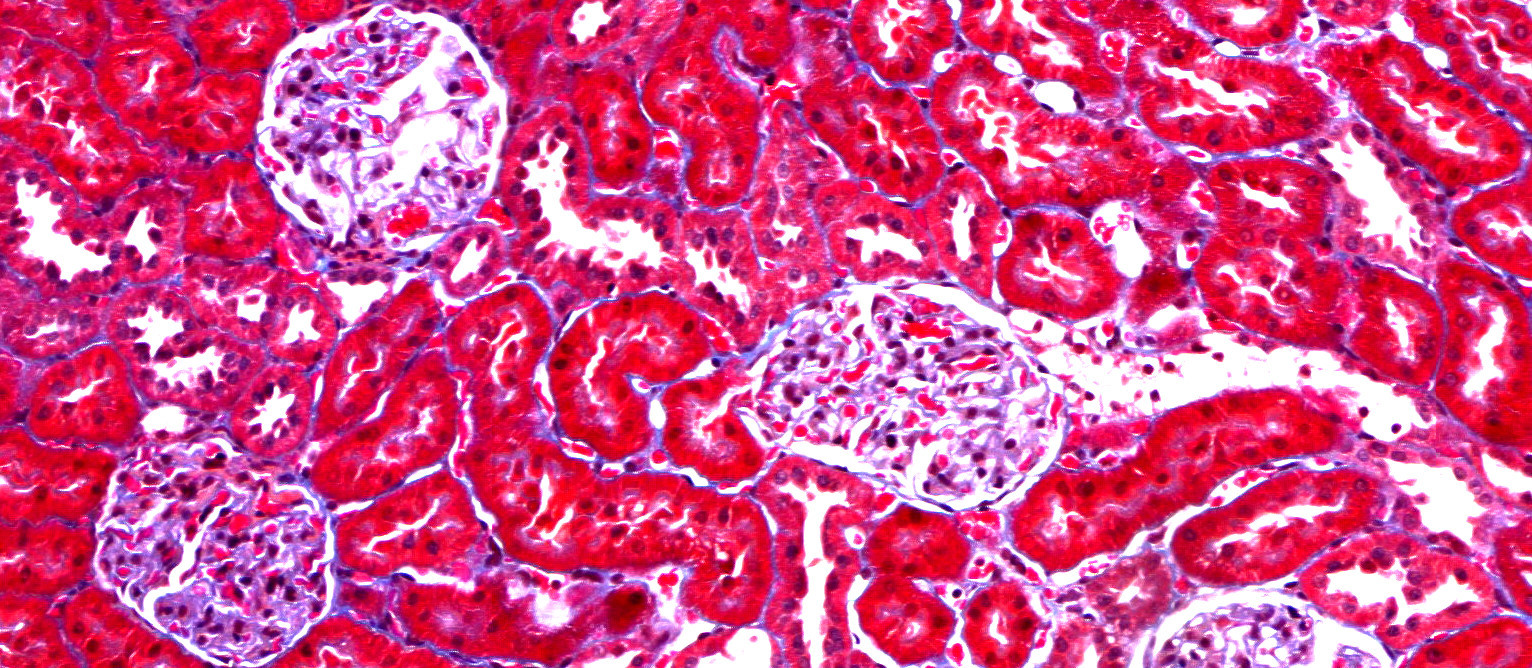

Supplement: Supplementary file 4 [file DataSheet8.ZIP › Fig 1D-masson-TSF-53/53-9.jpeg]

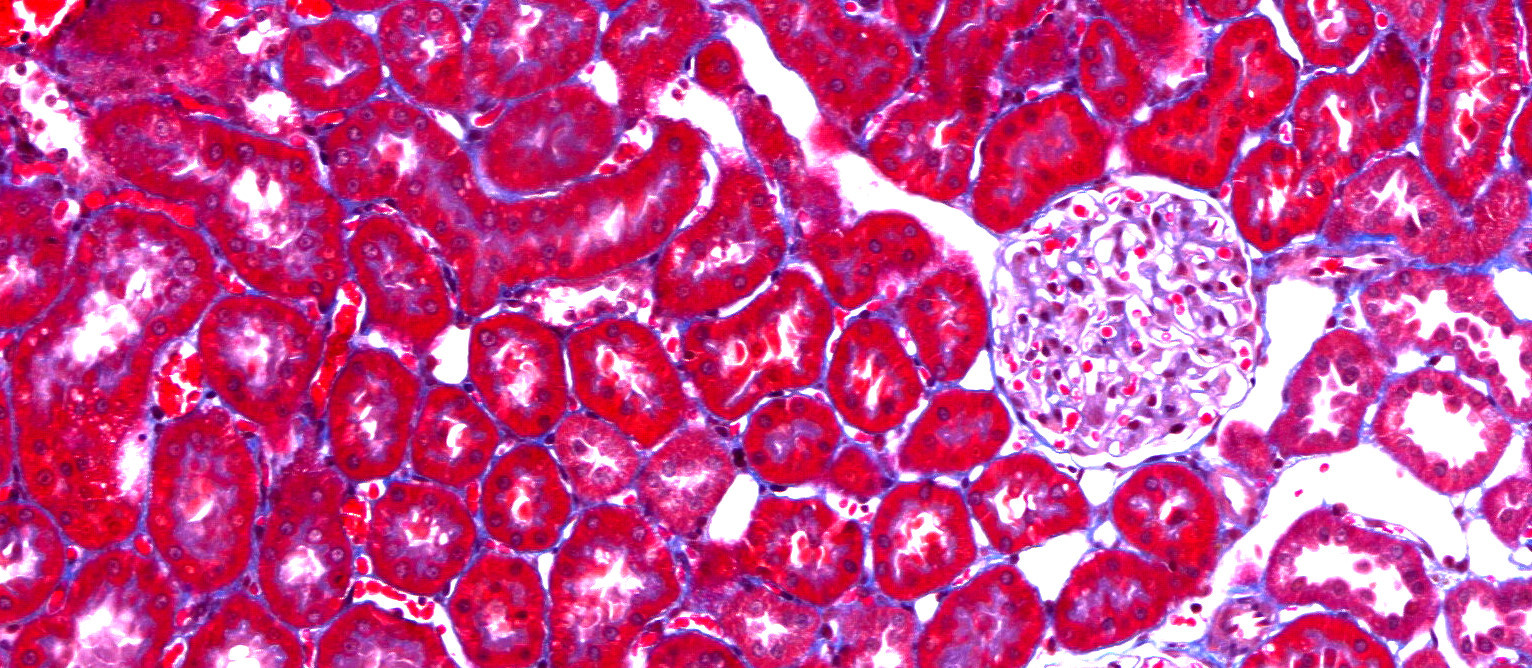

Supplement: Supplementary file 4 [file DataSheet8.ZIP › Fig 1D-masson-TSF-56/56-1.jpeg]

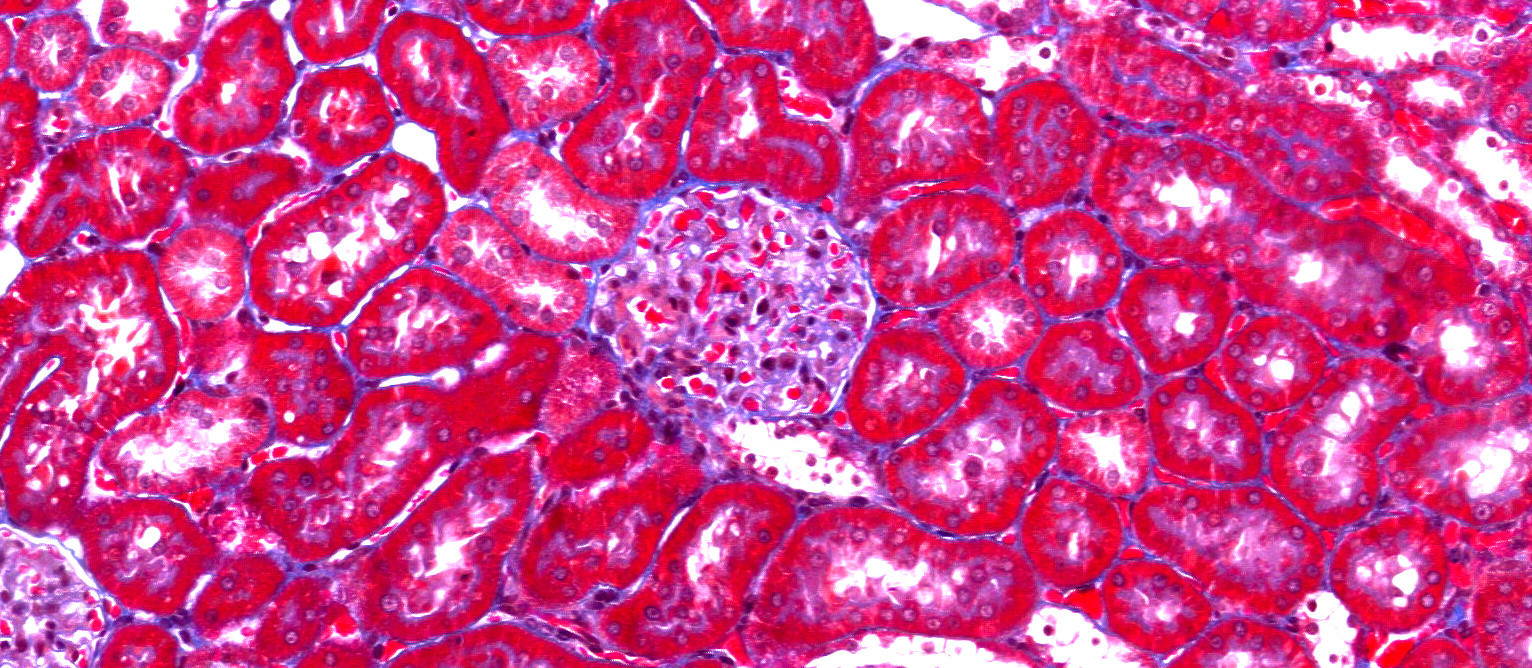

Supplement: Supplementary file 4 [file DataSheet8.ZIP › Fig 1D-masson-TSF-56/56-10.jpeg]

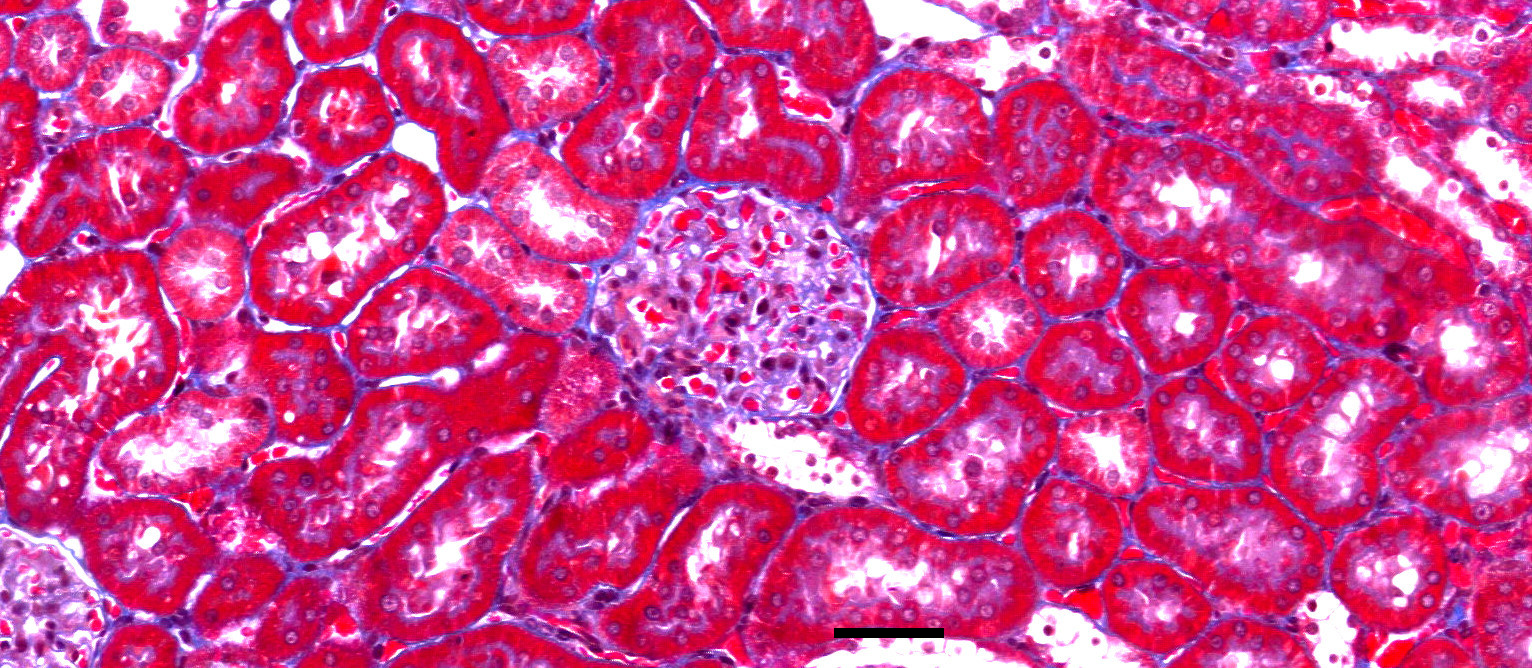

Supplement: Supplementary file 4 [file DataSheet8.ZIP › Fig 1D-masson-TSF-56/56-10-1.png]

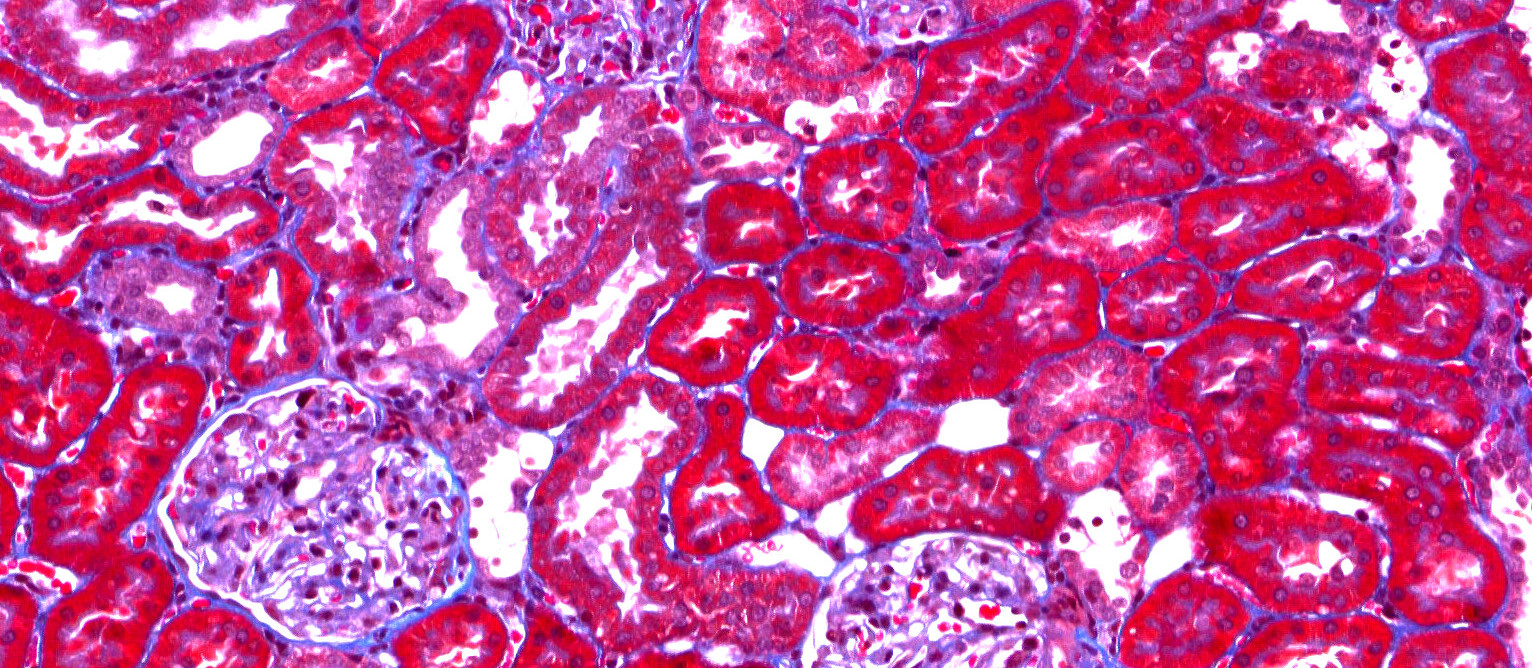

Supplement: Supplementary file 4 [file DataSheet8.ZIP › Fig 1D-masson-TSF-56/56-2.jpeg]

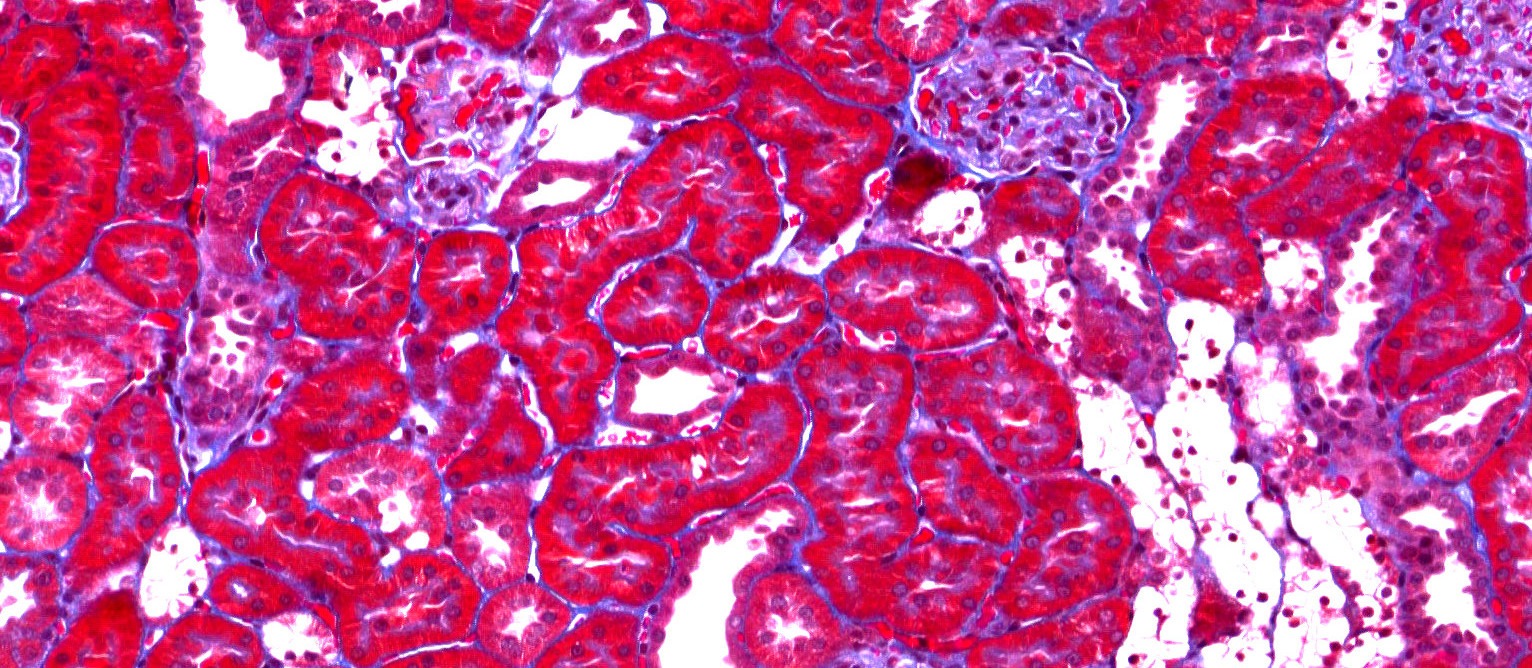

Supplement: Supplementary file 4 [file DataSheet8.ZIP › Fig 1D-masson-TSF-56/56-3.jpeg]

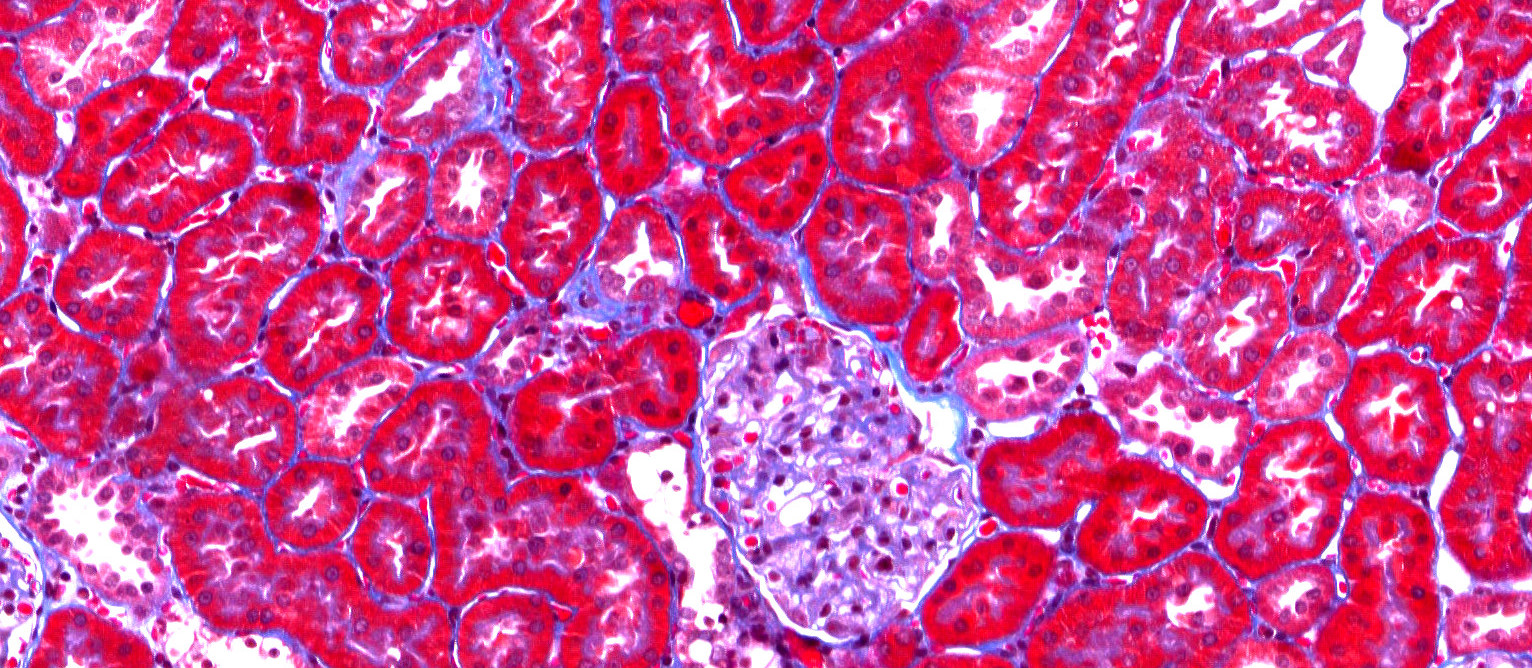

Supplement: Supplementary file 4 [file DataSheet8.ZIP › Fig 1D-masson-TSF-56/56-4.jpeg]

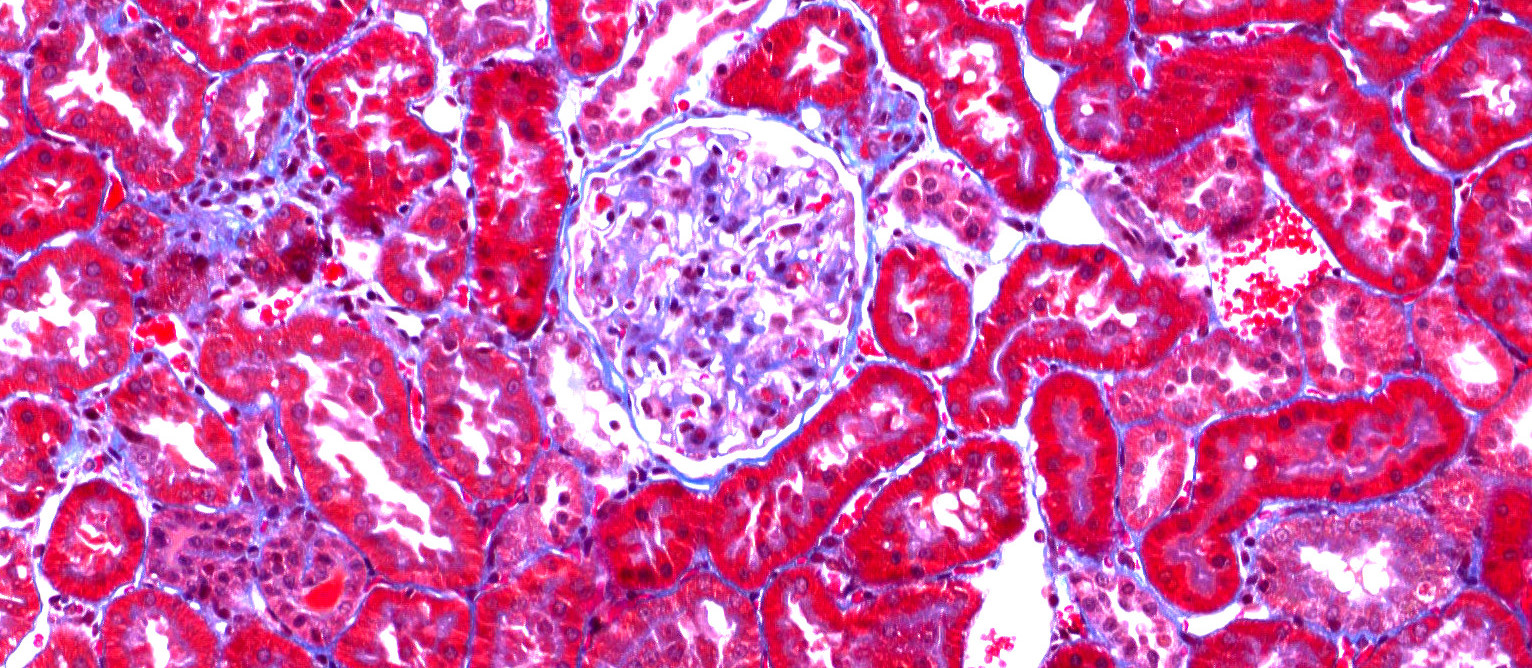

Supplement: Supplementary file 4 [file DataSheet8.ZIP › Fig 1D-masson-TSF-56/56-5.jpeg]

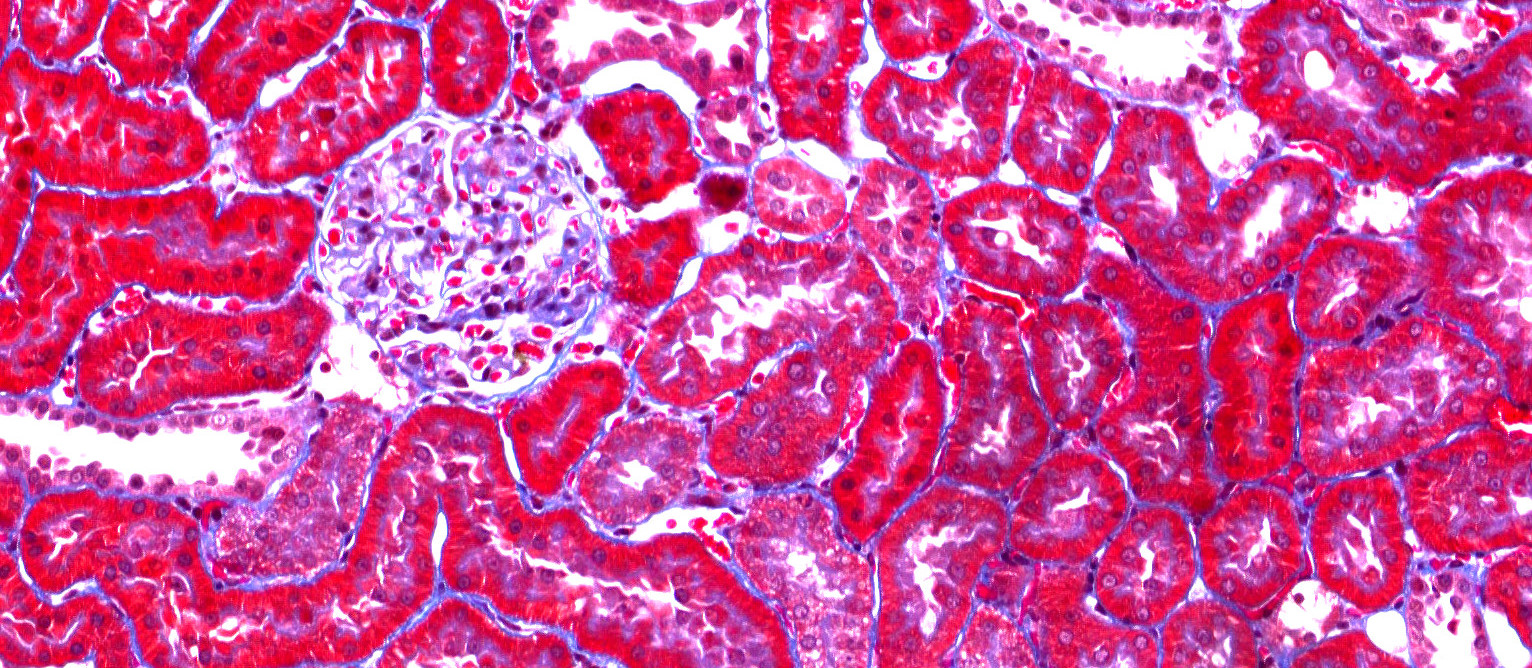

Supplement: Supplementary file 4 [file DataSheet8.ZIP › Fig 1D-masson-TSF-56/56-6.jpeg]

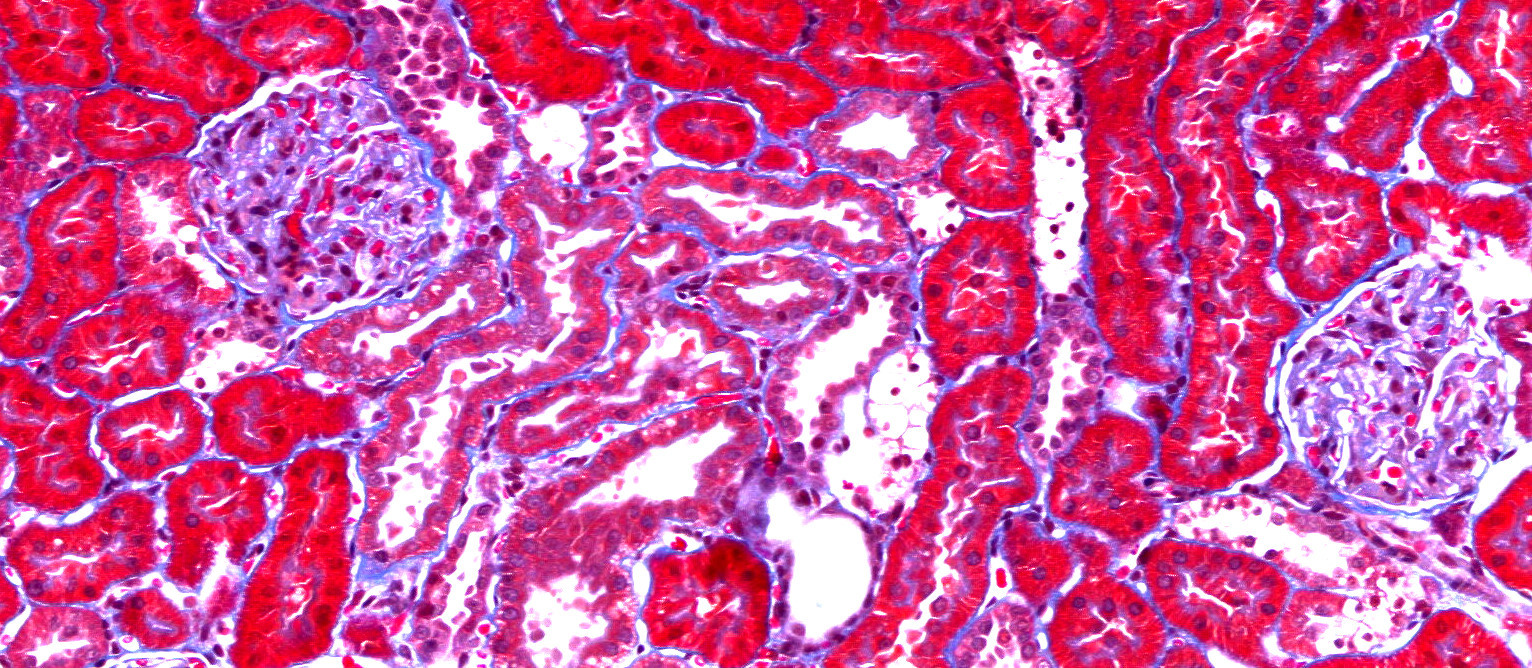

Supplement: Supplementary file 4 [file DataSheet8.ZIP › Fig 1D-masson-TSF-56/56-7.jpeg]

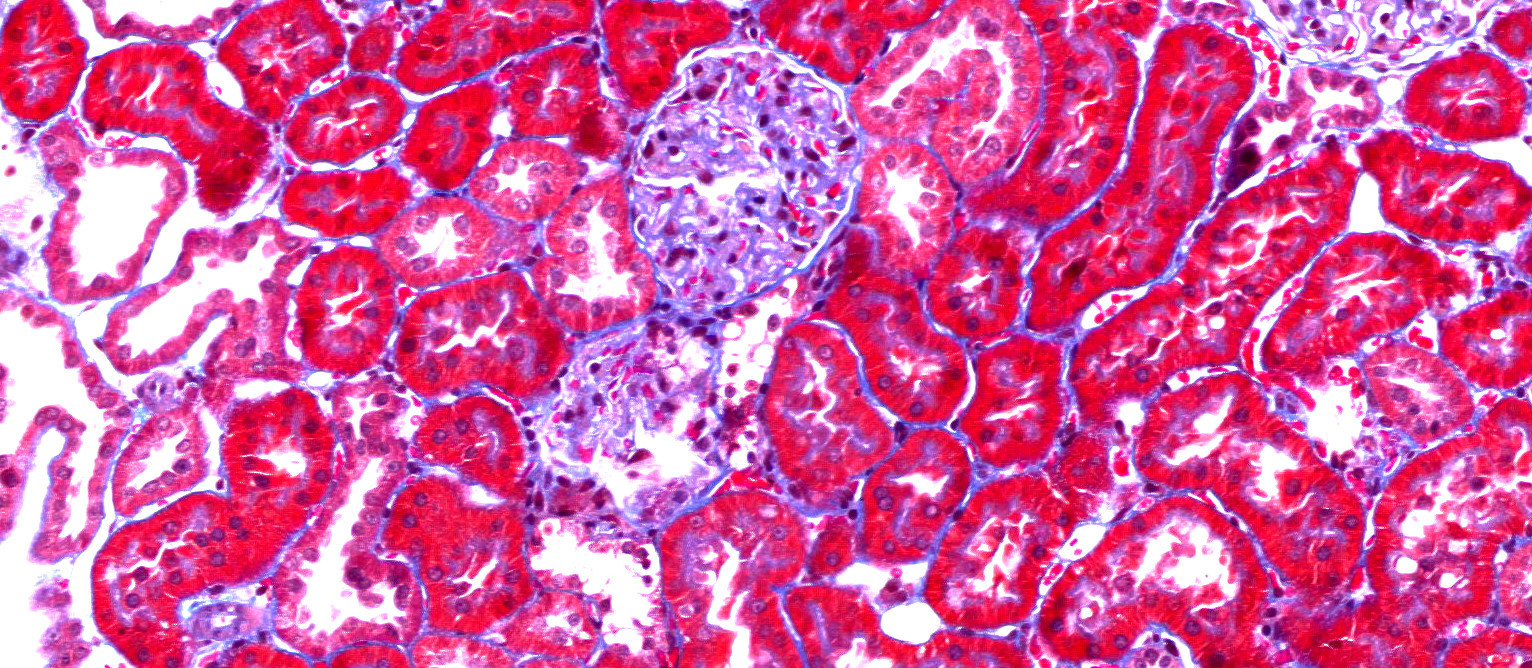

Supplement: Supplementary file 4 [file DataSheet8.ZIP › Fig 1D-masson-TSF-56/56-8.jpeg]

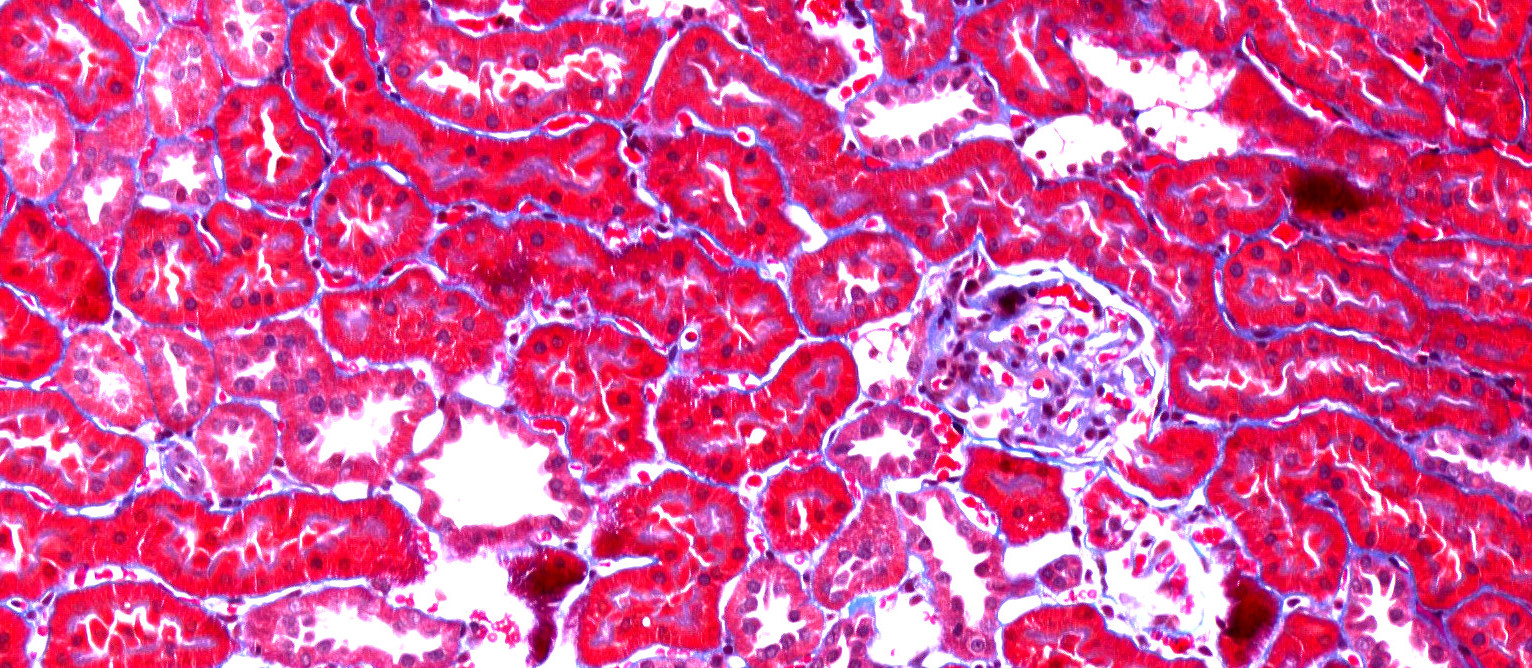

Supplement: Supplementary file 4 [file DataSheet8.ZIP › Fig 1D-masson-TSF-56/56-9.jpeg]

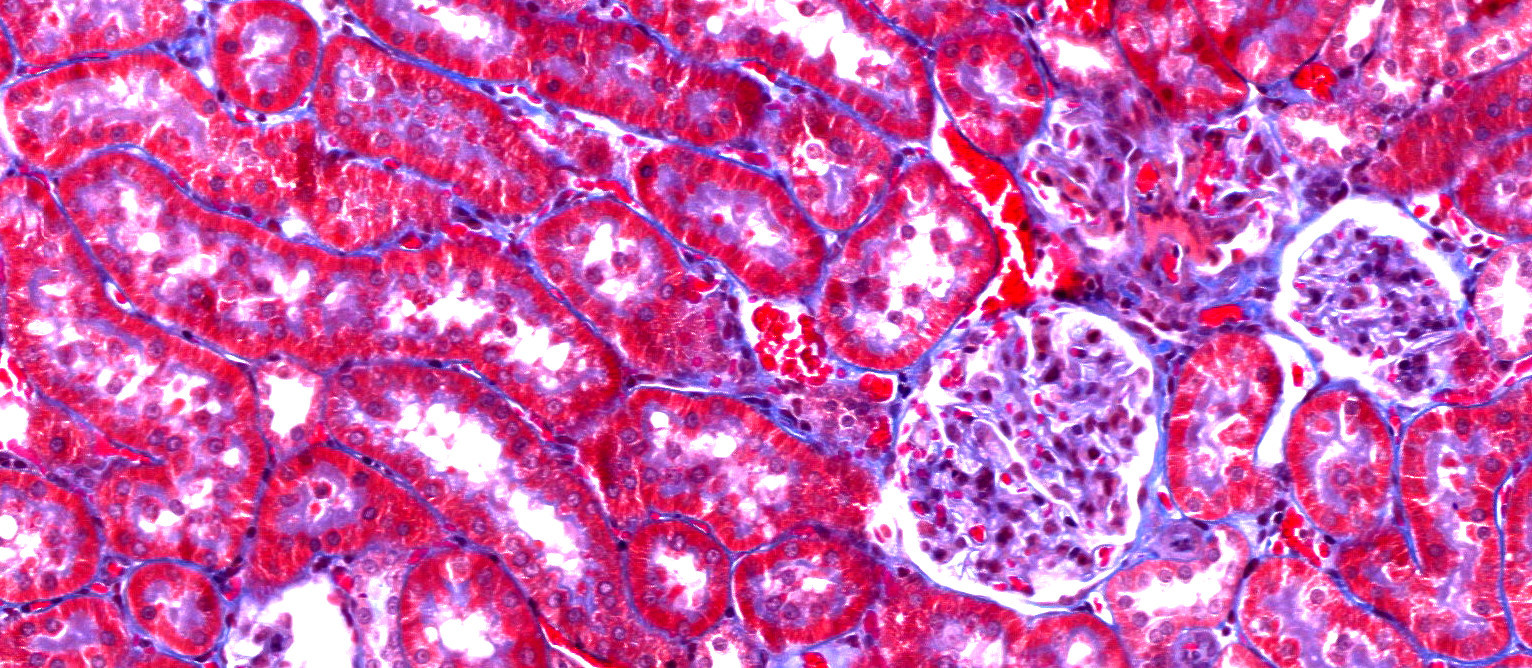

Supplement: Supplementary file 4 [file DataSheet8.ZIP › Fig 1D-masson-TSF-57(1)/57-1.jpeg]

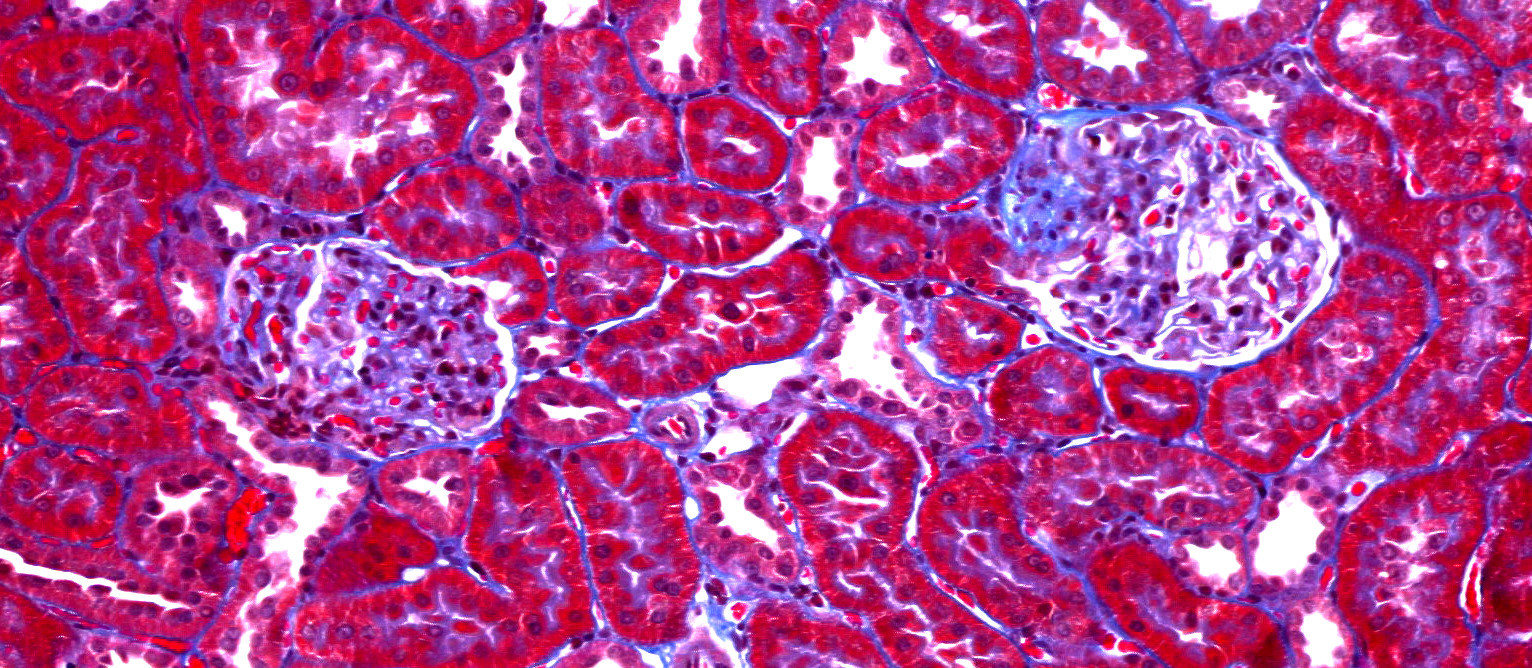

Supplement: Supplementary file 4 [file DataSheet8.ZIP › Fig 1D-masson-TSF-57(1)/57-2.jpeg]

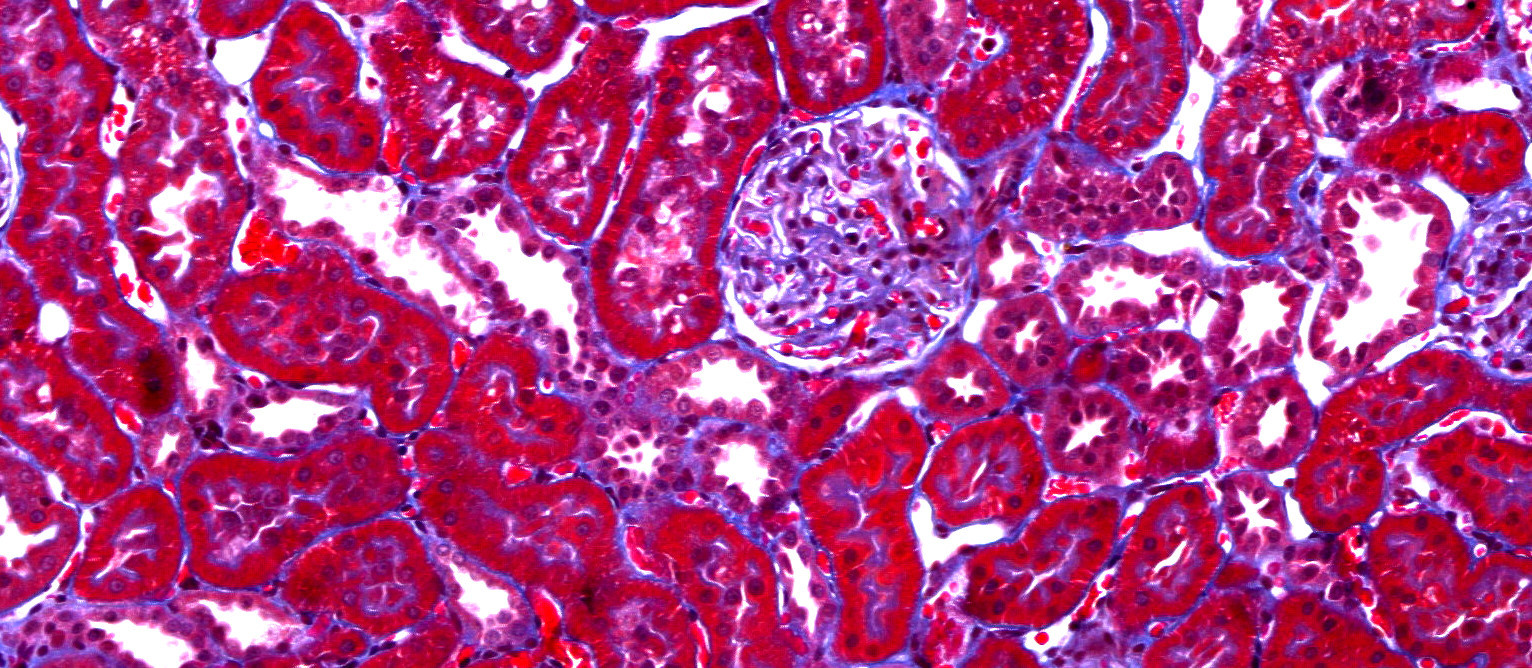

Supplement: Supplementary file 4 [file DataSheet8.ZIP › Fig 1D-masson-TSF-57(1)/57-3.jpeg]

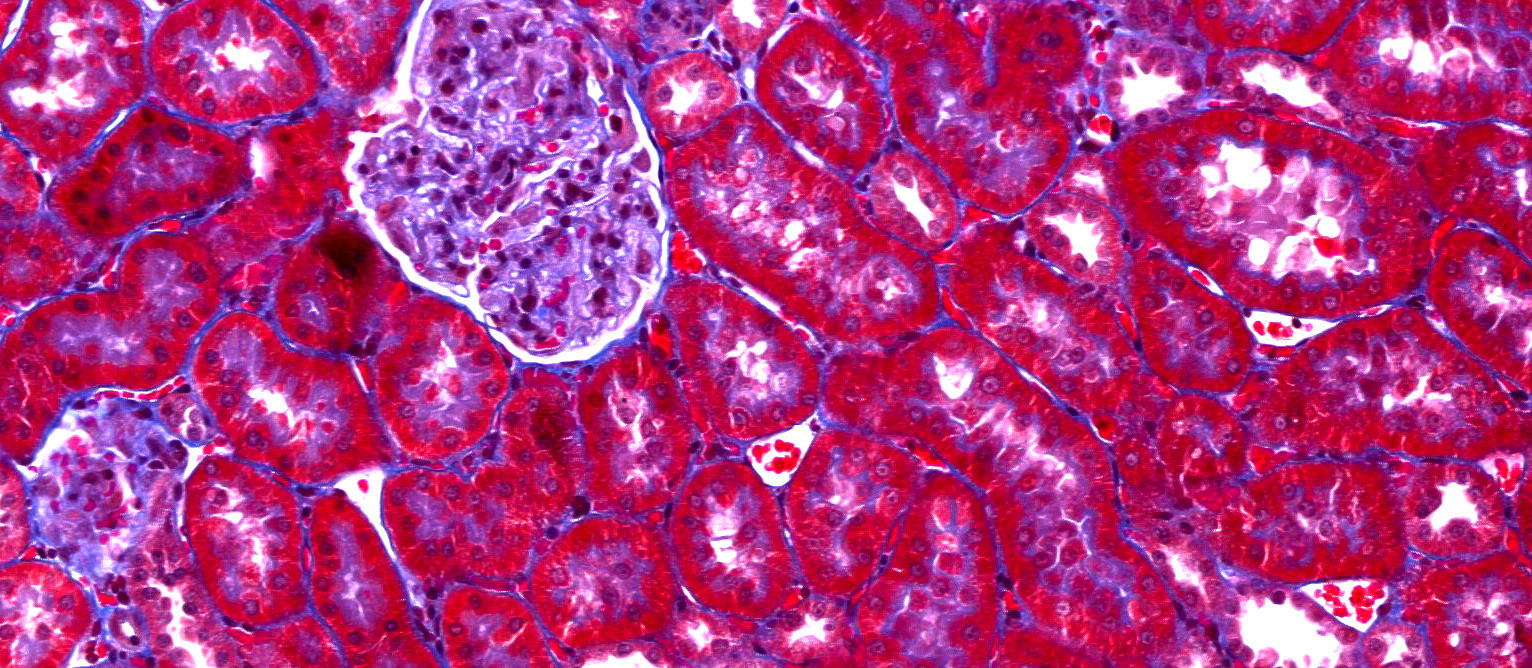

Supplement: Supplementary file 4 [file DataSheet8.ZIP › Fig 1D-masson-TSF-57(1)/57-4.jpeg]

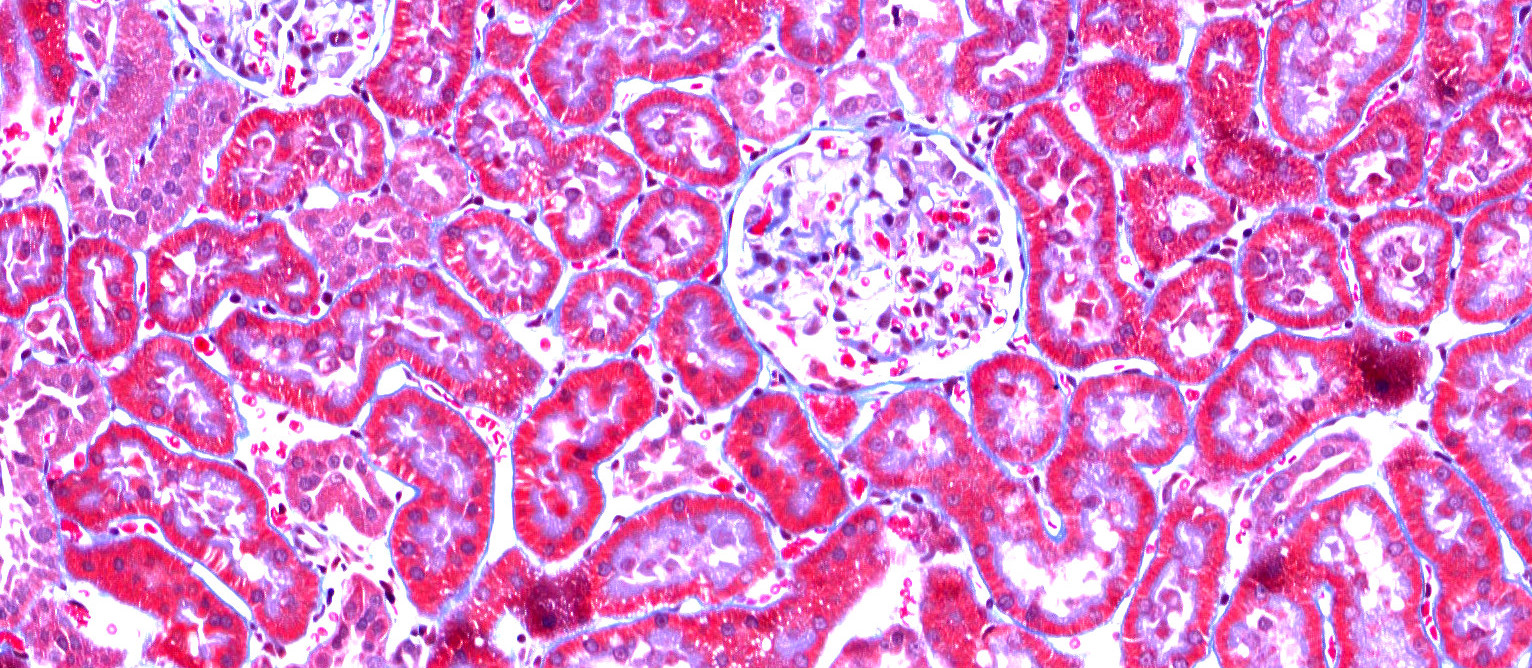

Supplement: Supplementary file 5 [file DataSheet9.ZIP › Fig 1D-masson-sham-5(2)/5-10.jpeg]

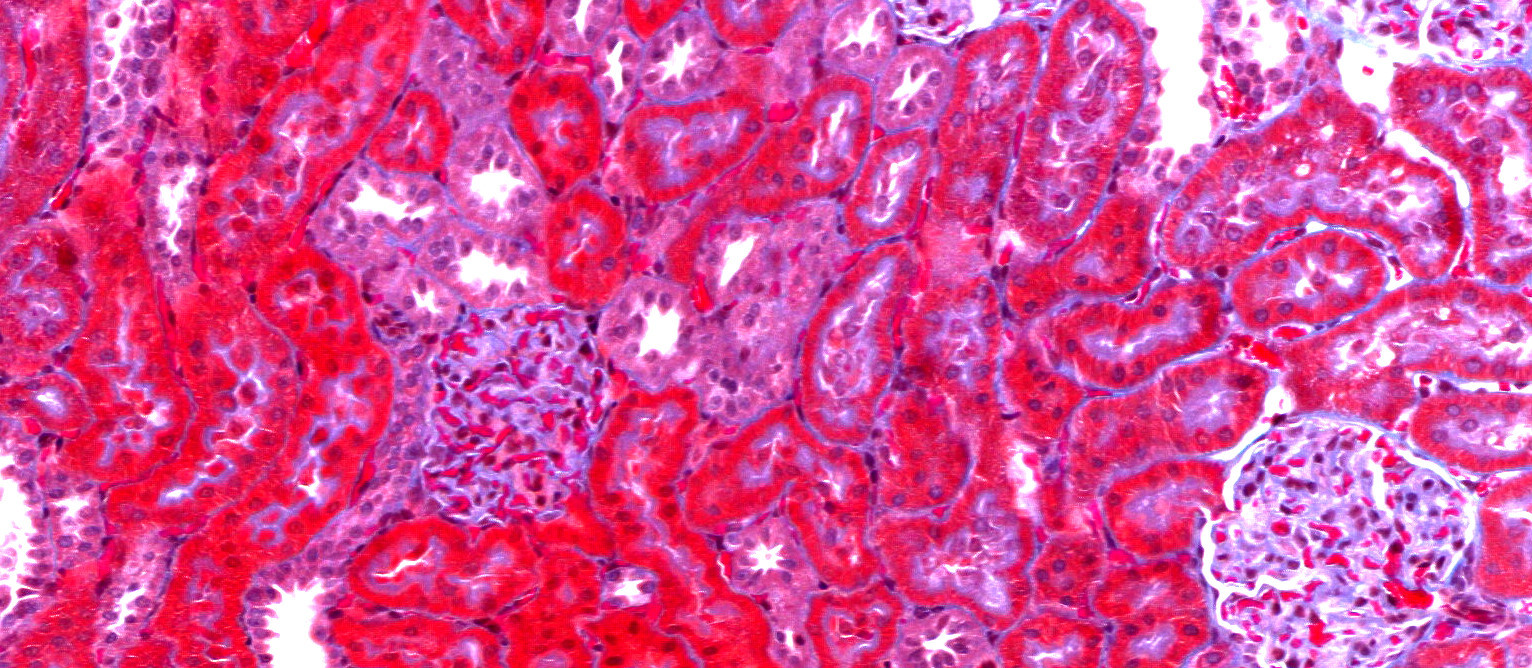

Supplement: Supplementary file 5 [file DataSheet9.ZIP › Fig 1D-masson-sham-5(2)/5-2.jpeg]

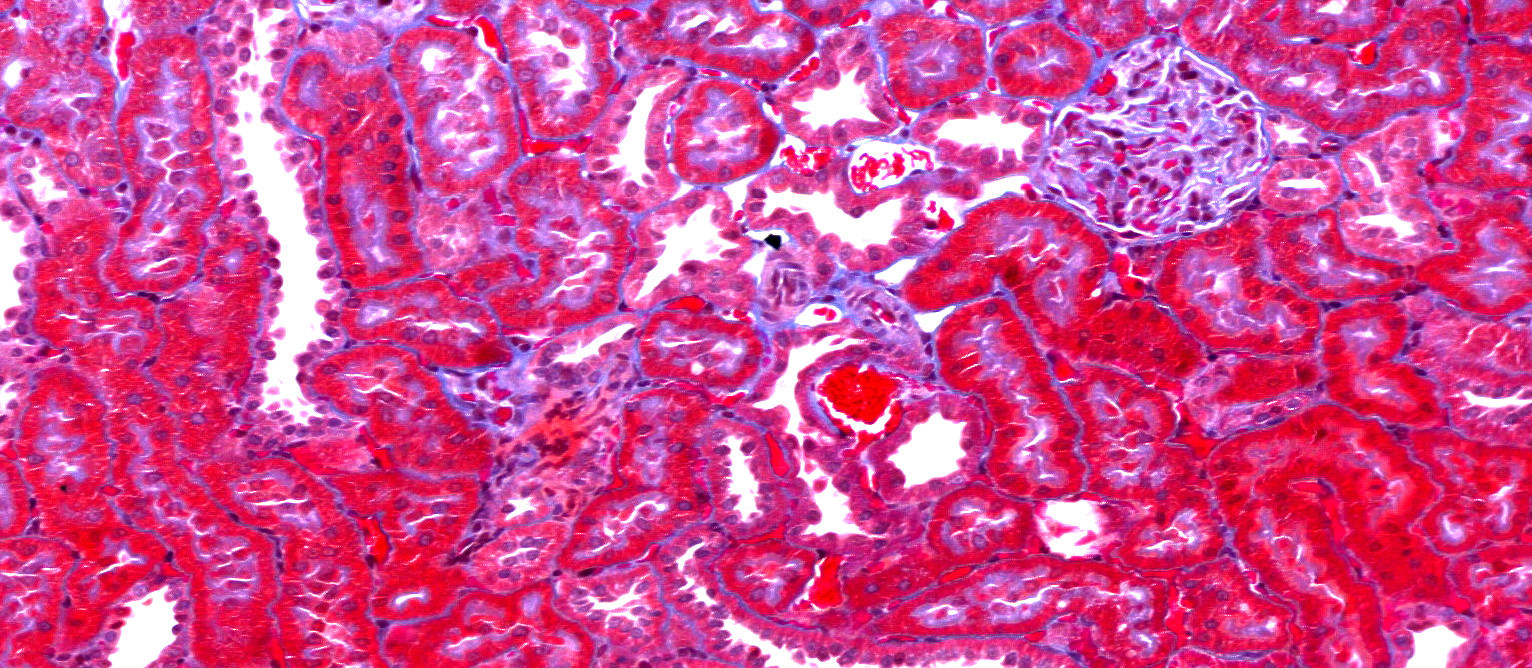

Supplement: Supplementary file 5 [file DataSheet9.ZIP › Fig 1D-masson-sham-5(2)/5-3.jpeg]

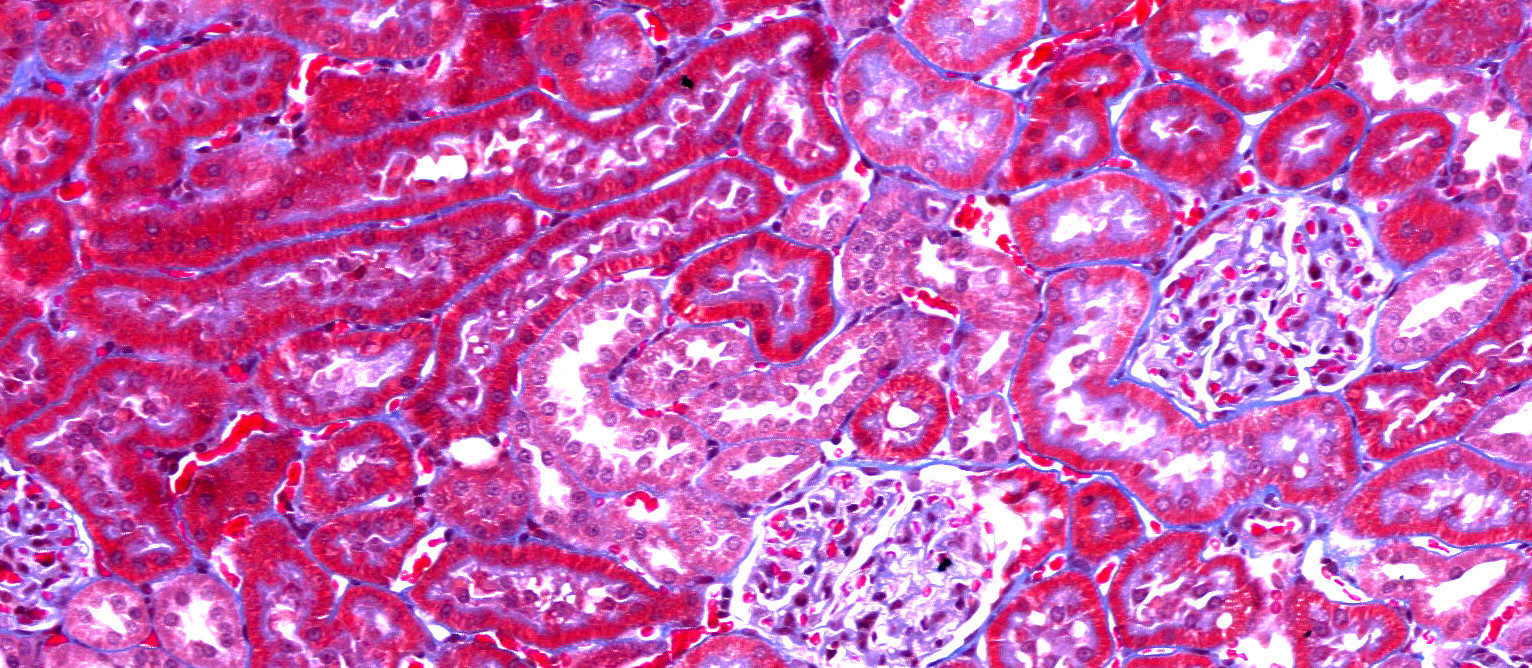

Supplement: Supplementary file 5 [file DataSheet9.ZIP › Fig 1D-masson-sham-5(2)/5-4.jpeg]

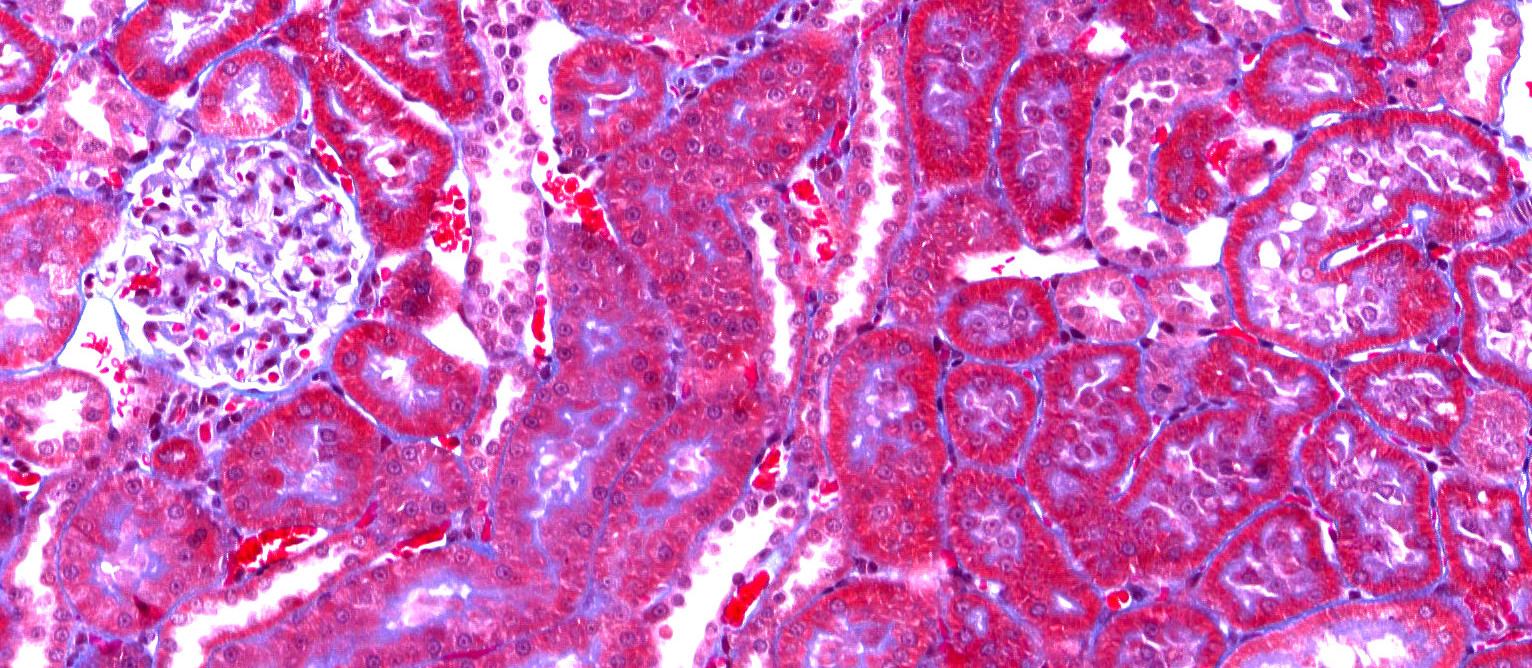

Supplement: Supplementary file 5 [file DataSheet9.ZIP › Fig 1D-masson-sham-5(2)/5-5.jpeg]

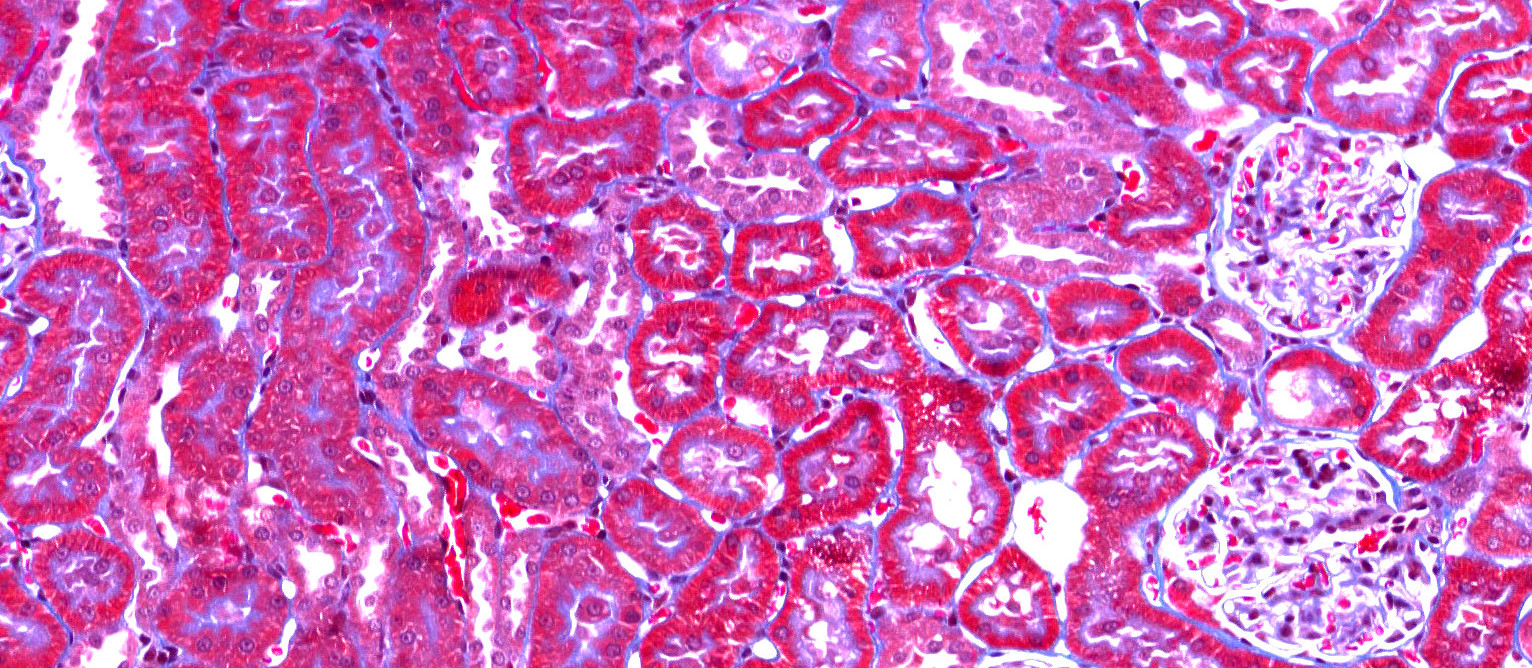

Supplement: Supplementary file 5 [file DataSheet9.ZIP › Fig 1D-masson-sham-5(2)/5-6.jpeg]

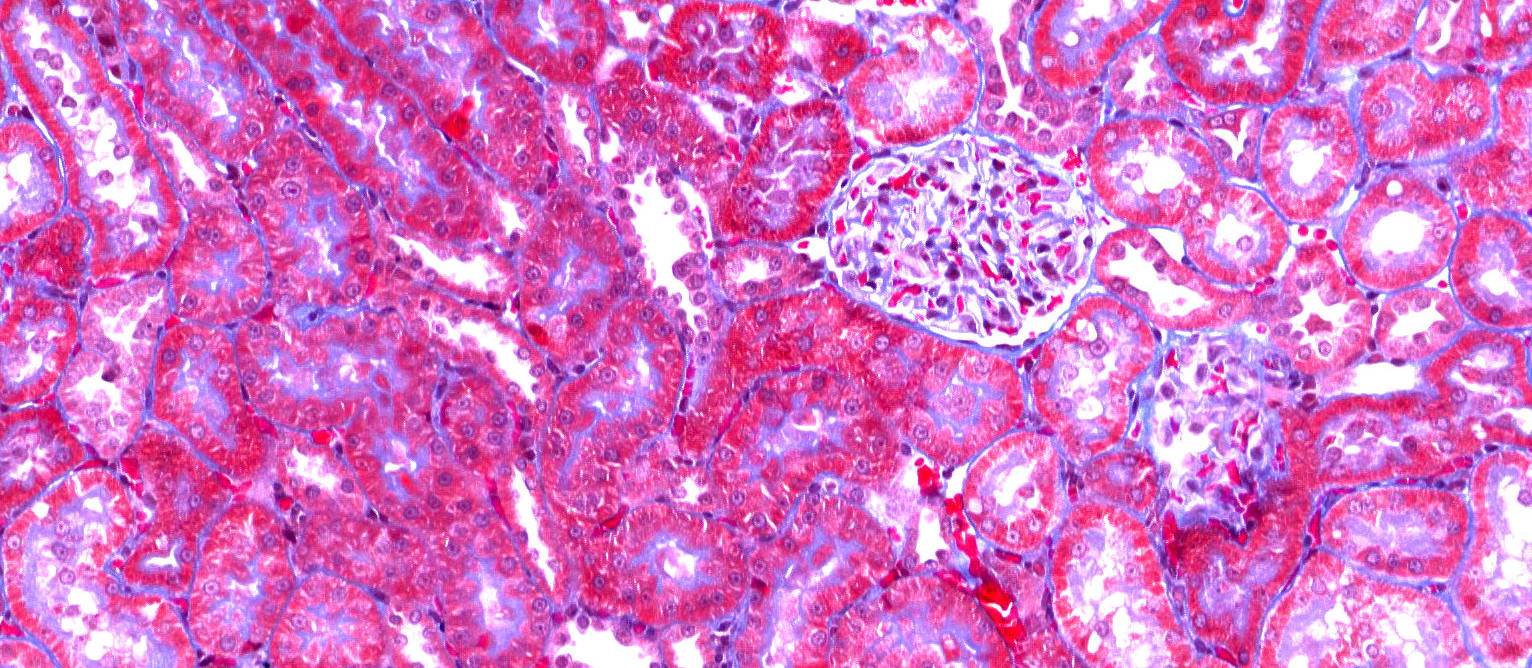

Supplement: Supplementary file 5 [file DataSheet9.ZIP › Fig 1D-masson-sham-5(2)/5-7.jpeg]

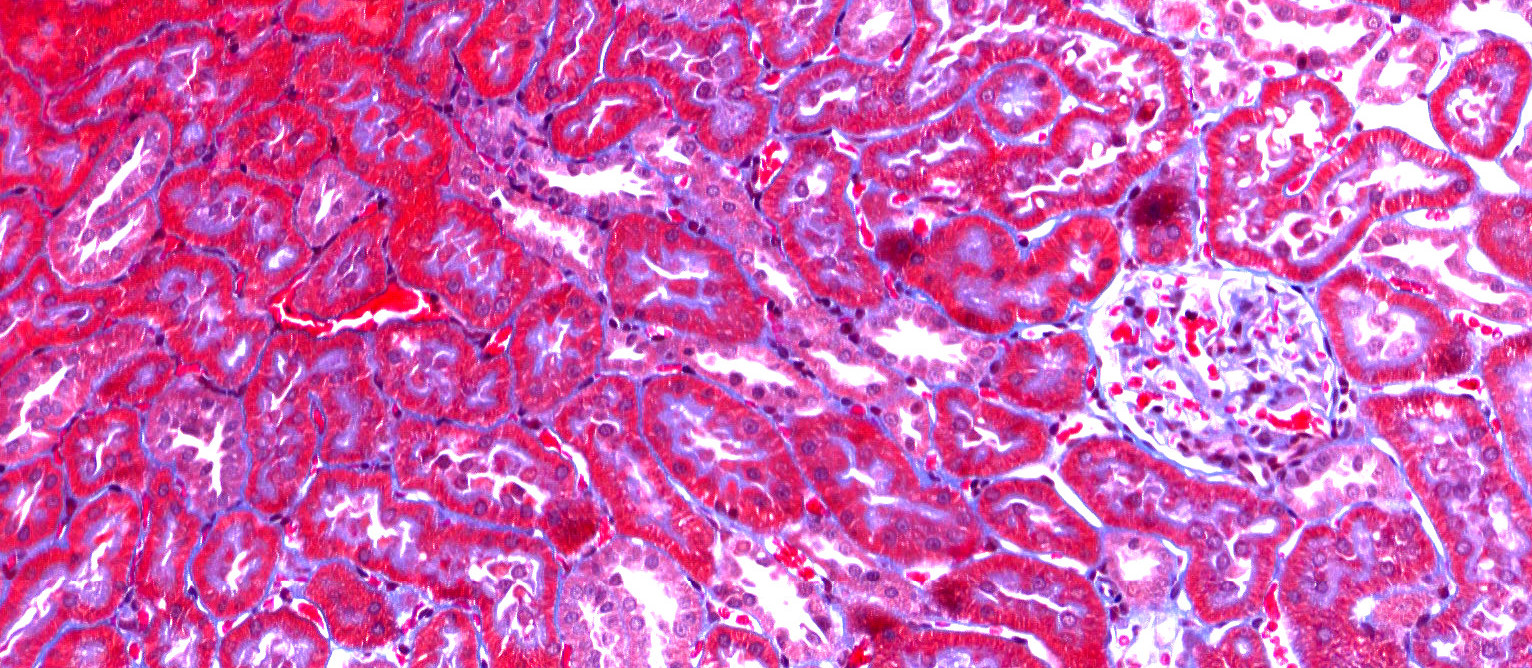

Supplement: Supplementary file 5 [file DataSheet9.ZIP › Fig 1D-masson-sham-5(2)/5-8.jpeg]

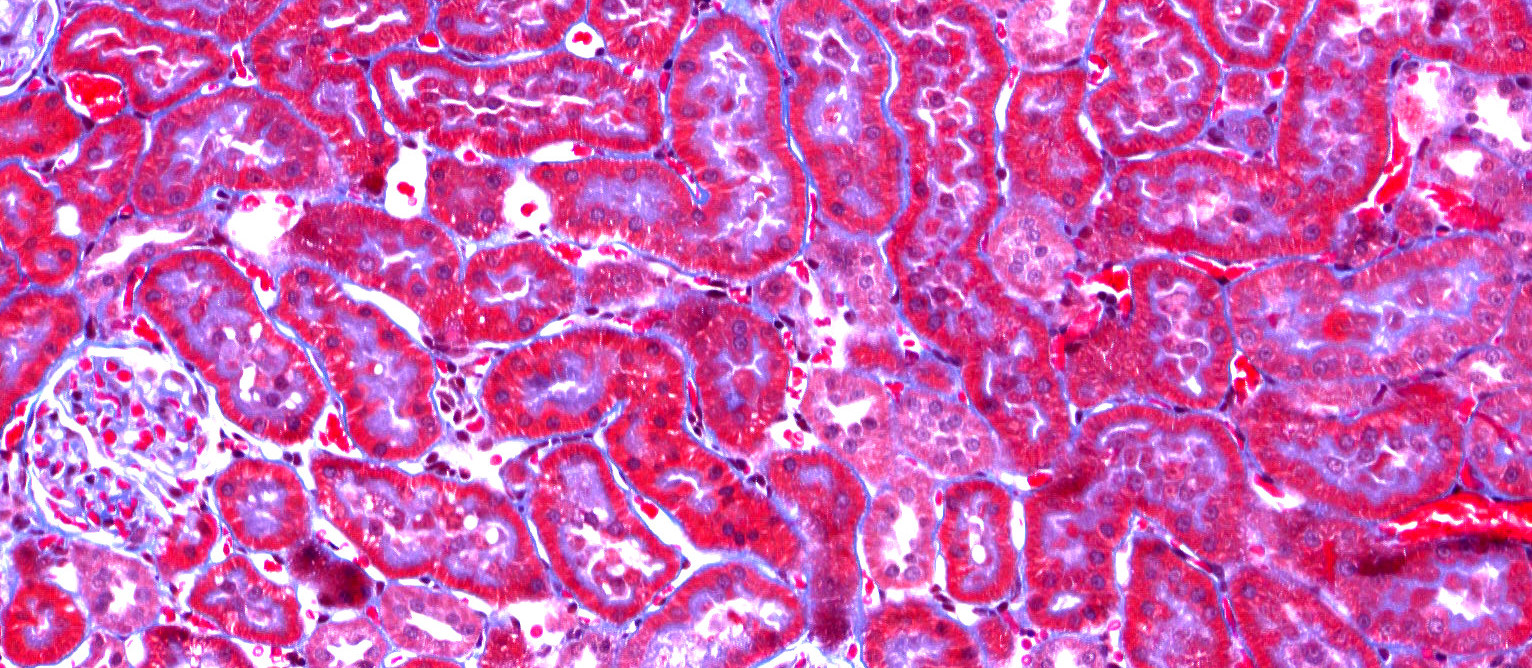

Supplement: Supplementary file 5 [file DataSheet9.ZIP › Fig 1D-masson-sham-5(2)/5-9.jpeg]

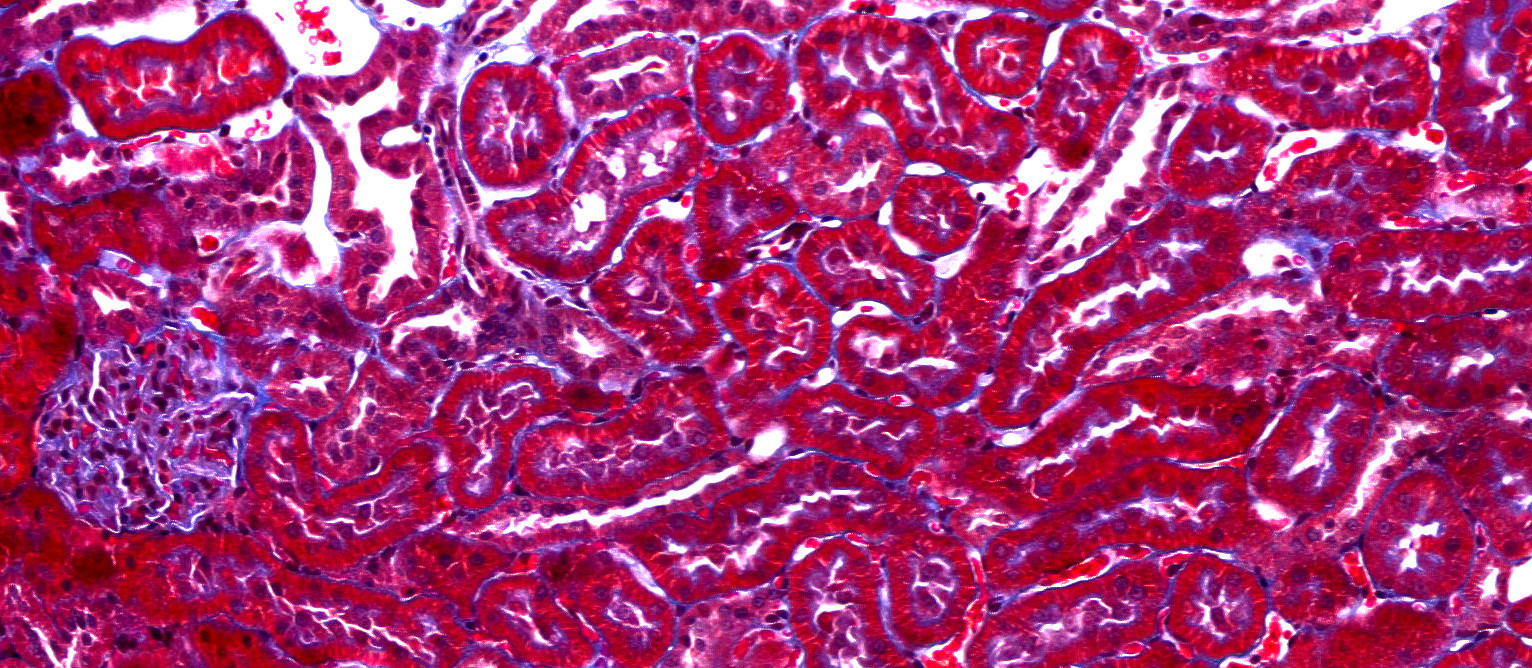

Supplement: Supplementary file 5 [file DataSheet9.ZIP › Fig 1D-masson-sham-6/6-1.jpeg]

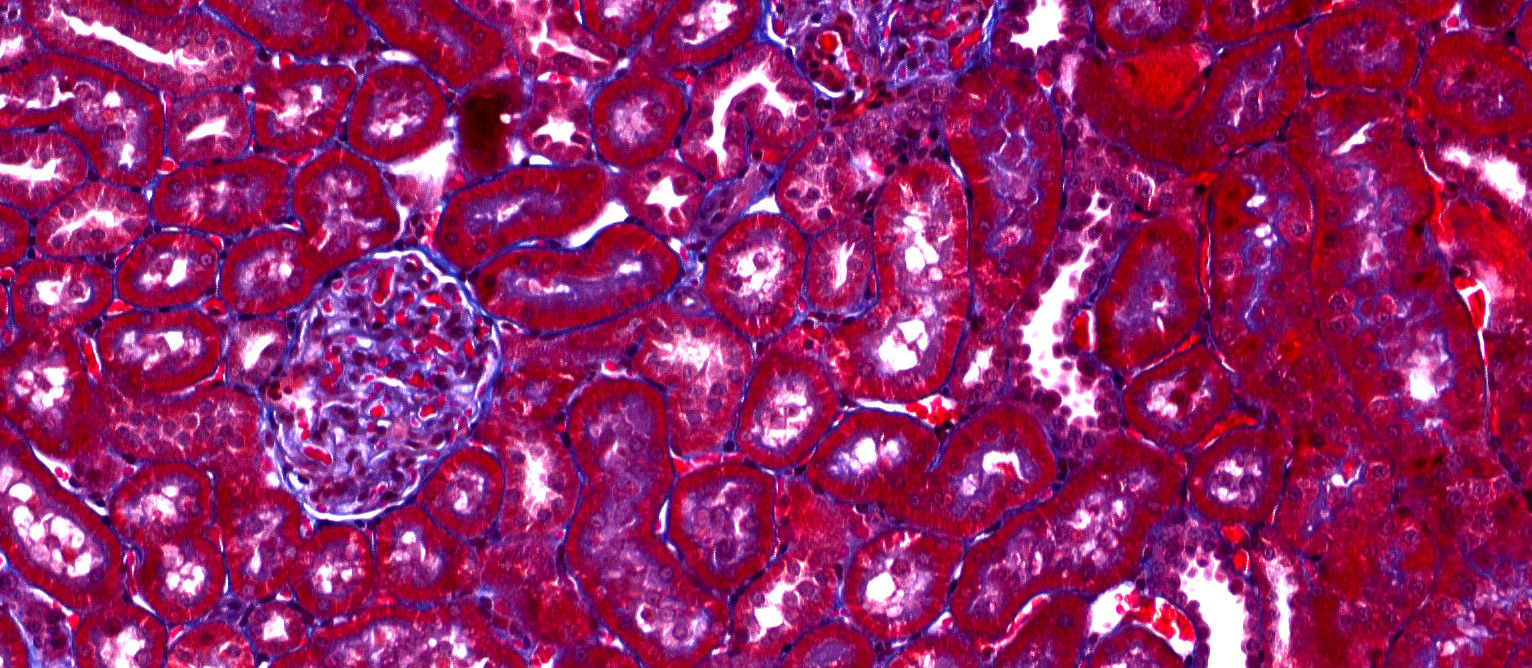

Supplement: Supplementary file 5 [file DataSheet9.ZIP › Fig 1D-masson-sham-6/6-10.jpeg]

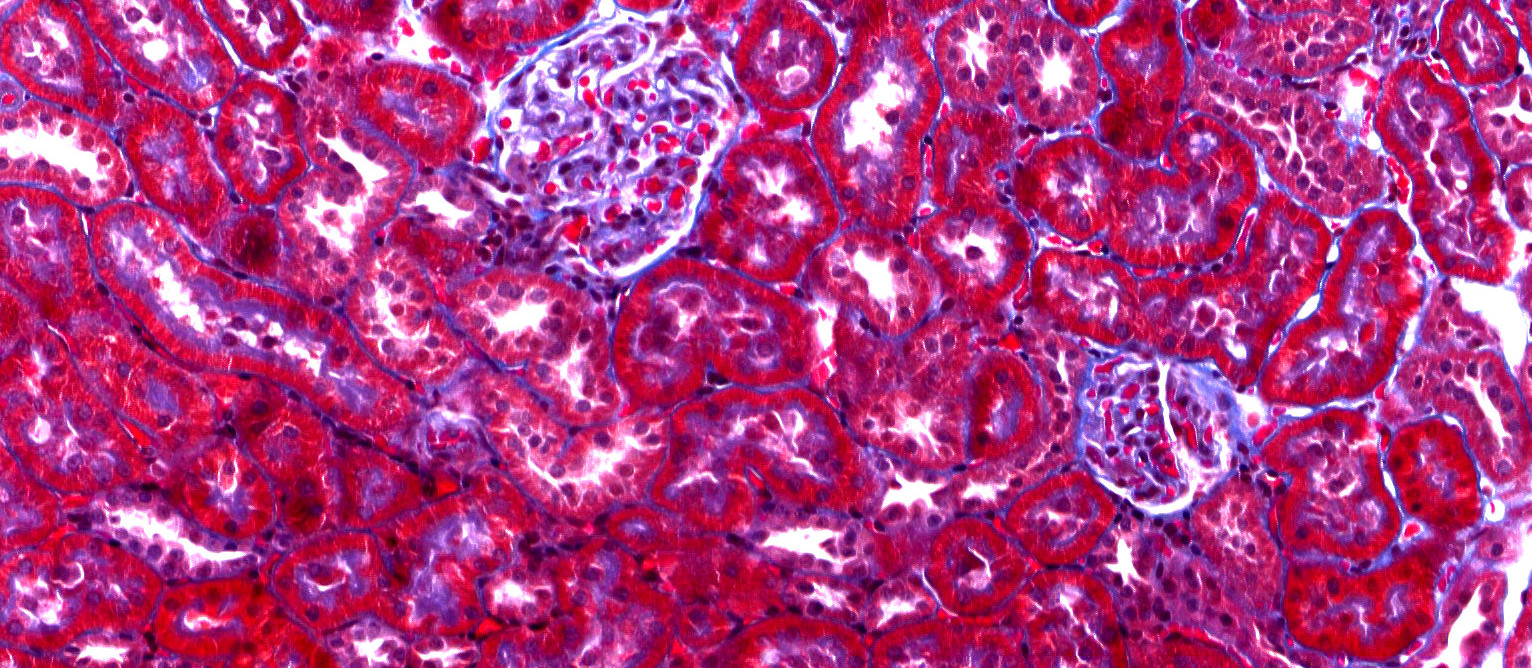

Supplement: Supplementary file 5 [file DataSheet9.ZIP › Fig 1D-masson-sham-6/6-2.jpeg]

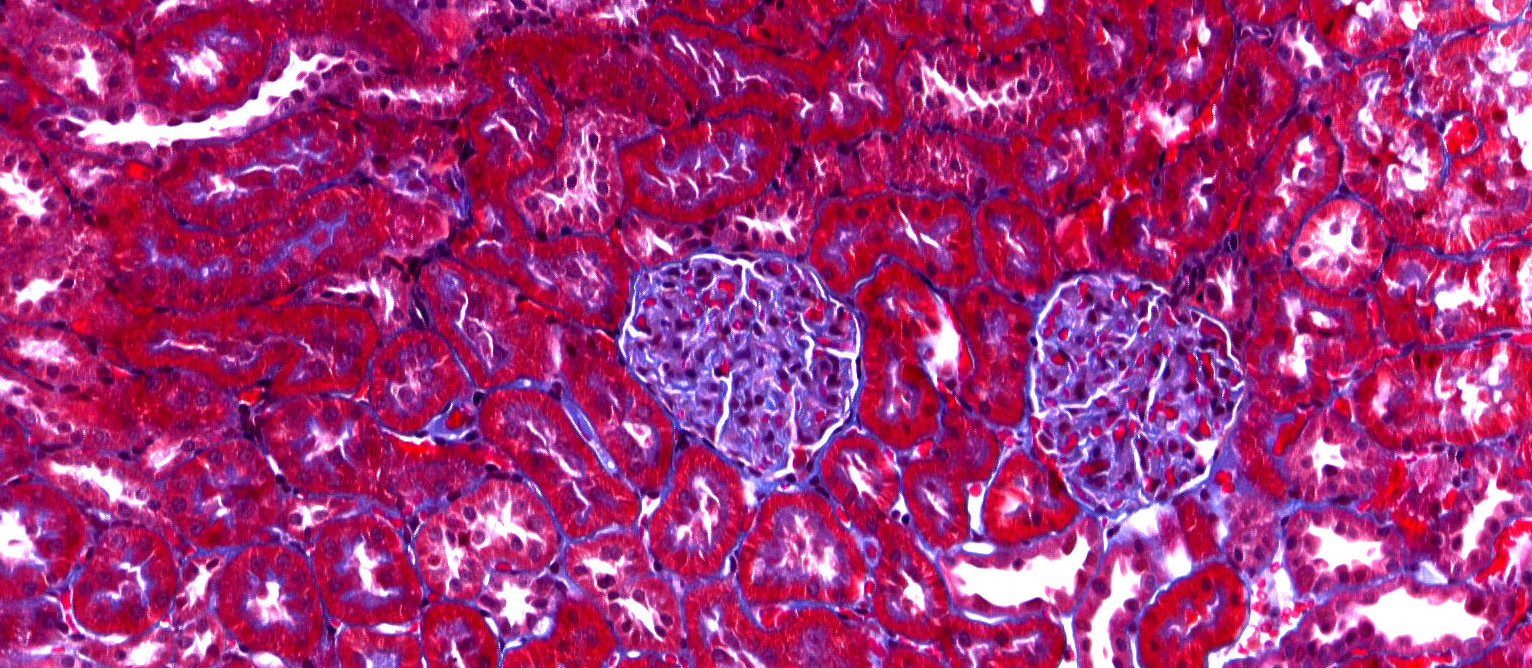

Supplement: Supplementary file 5 [file DataSheet9.ZIP › Fig 1D-masson-sham-6/6-3.jpeg]

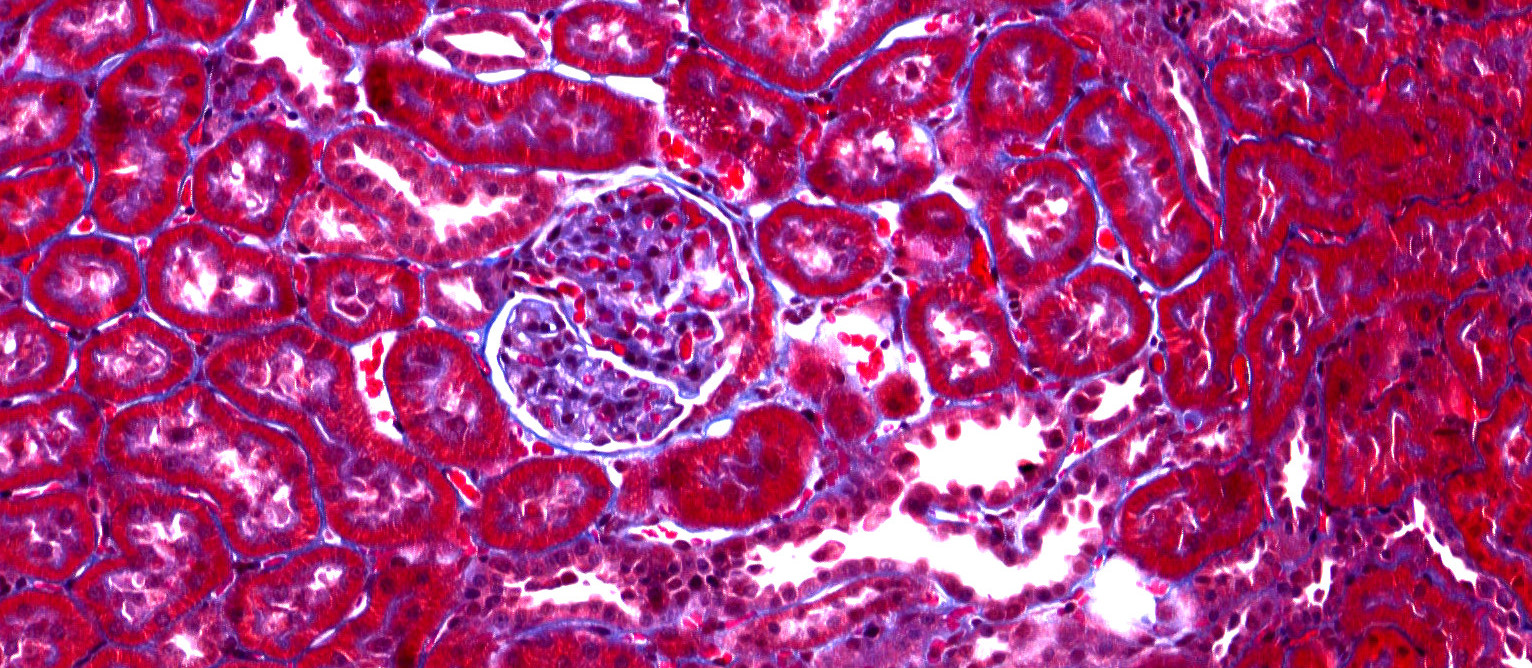

Supplement: Supplementary file 5 [file DataSheet9.ZIP › Fig 1D-masson-sham-6/6-4.jpeg]

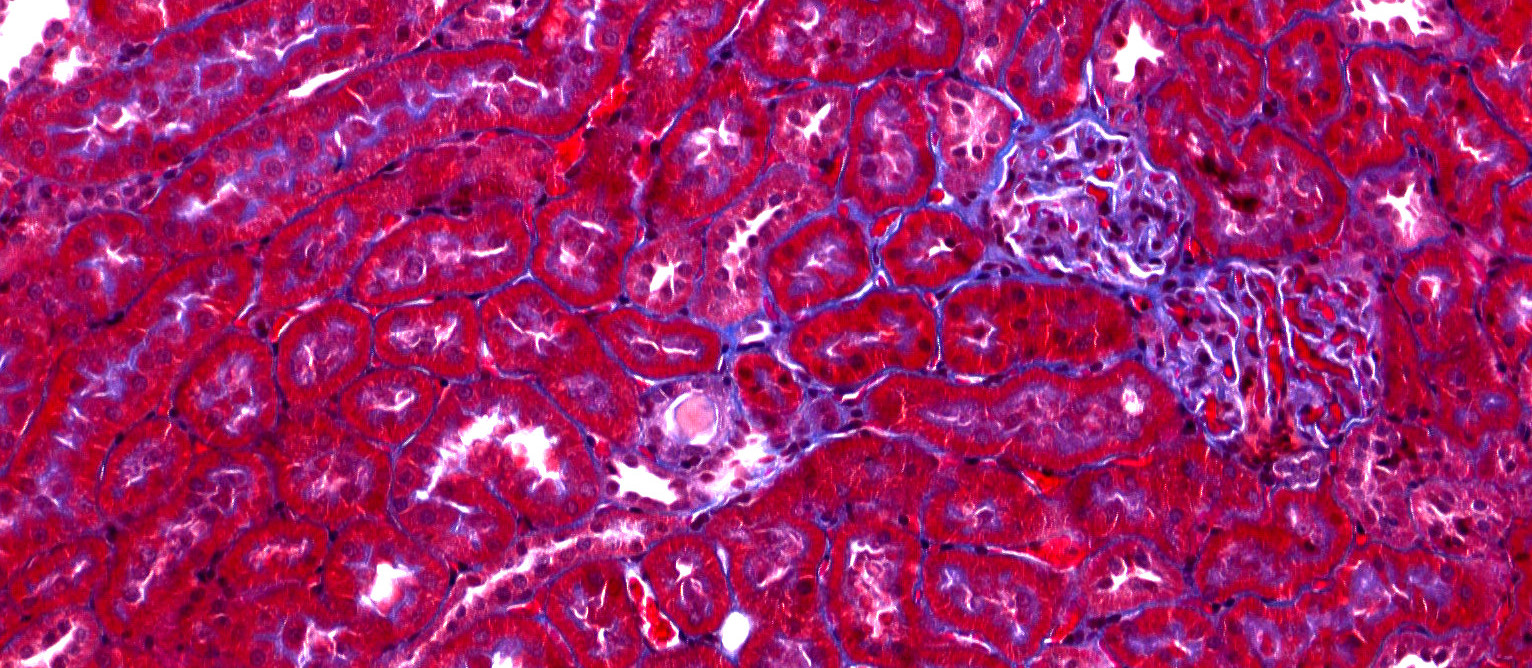

Supplement: Supplementary file 5 [file DataSheet9.ZIP › Fig 1D-masson-sham-6/6-5.jpeg]

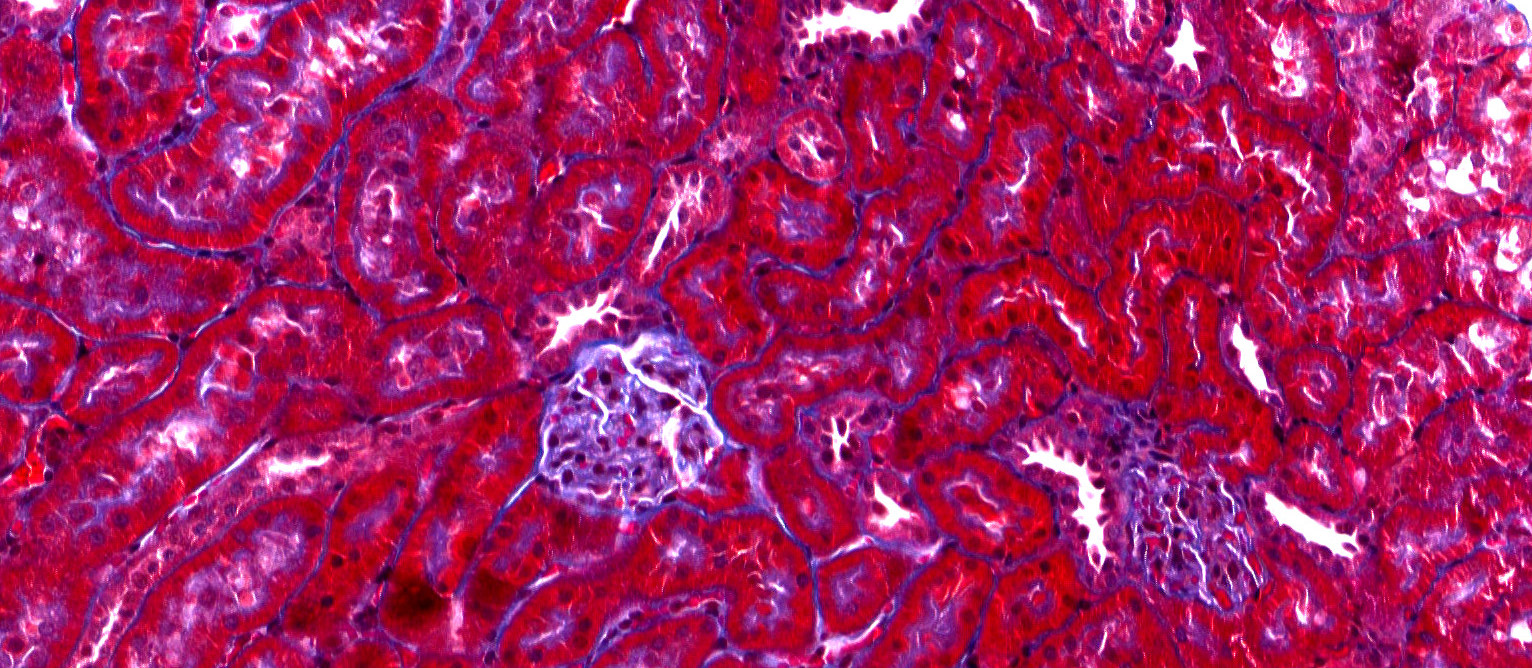

Supplement: Supplementary file 5 [file DataSheet9.ZIP › Fig 1D-masson-sham-6/6-6.jpeg]

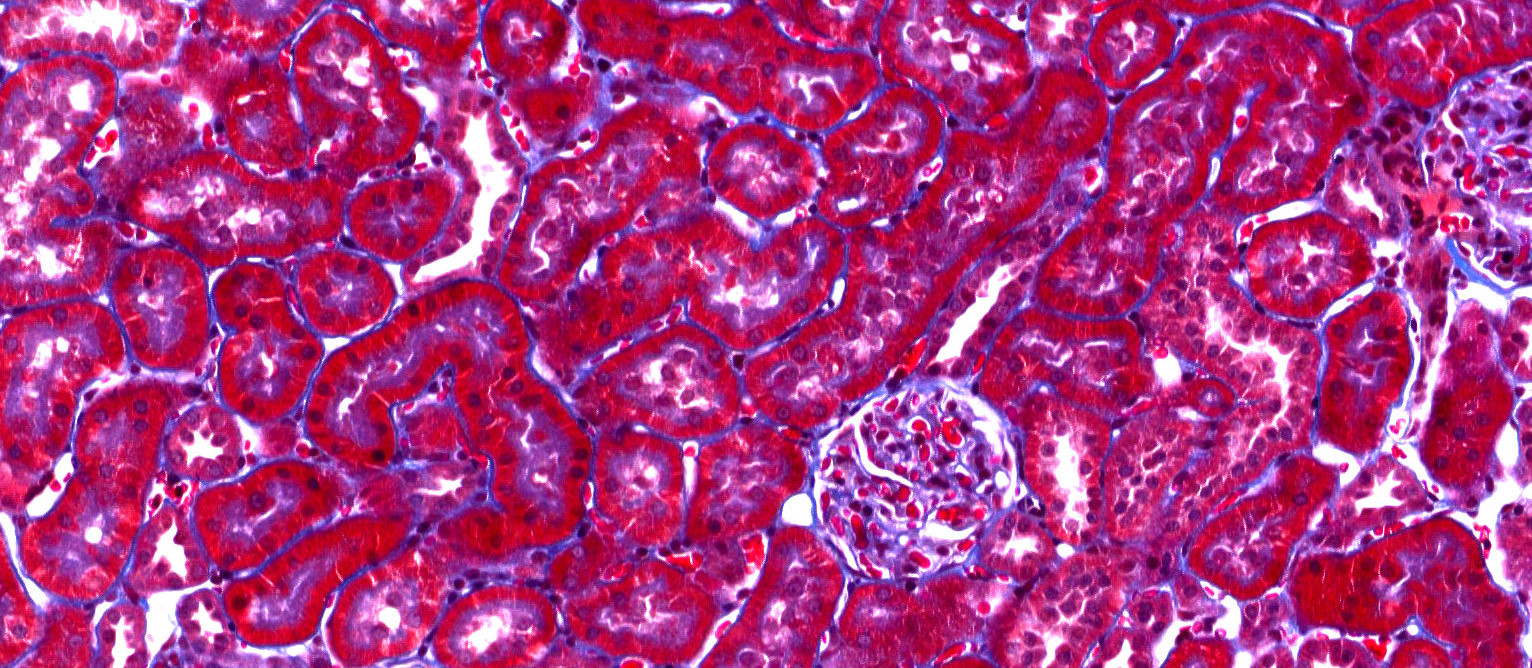

Supplement: Supplementary file 5 [file DataSheet9.ZIP › Fig 1D-masson-sham-6/6-7.jpeg]

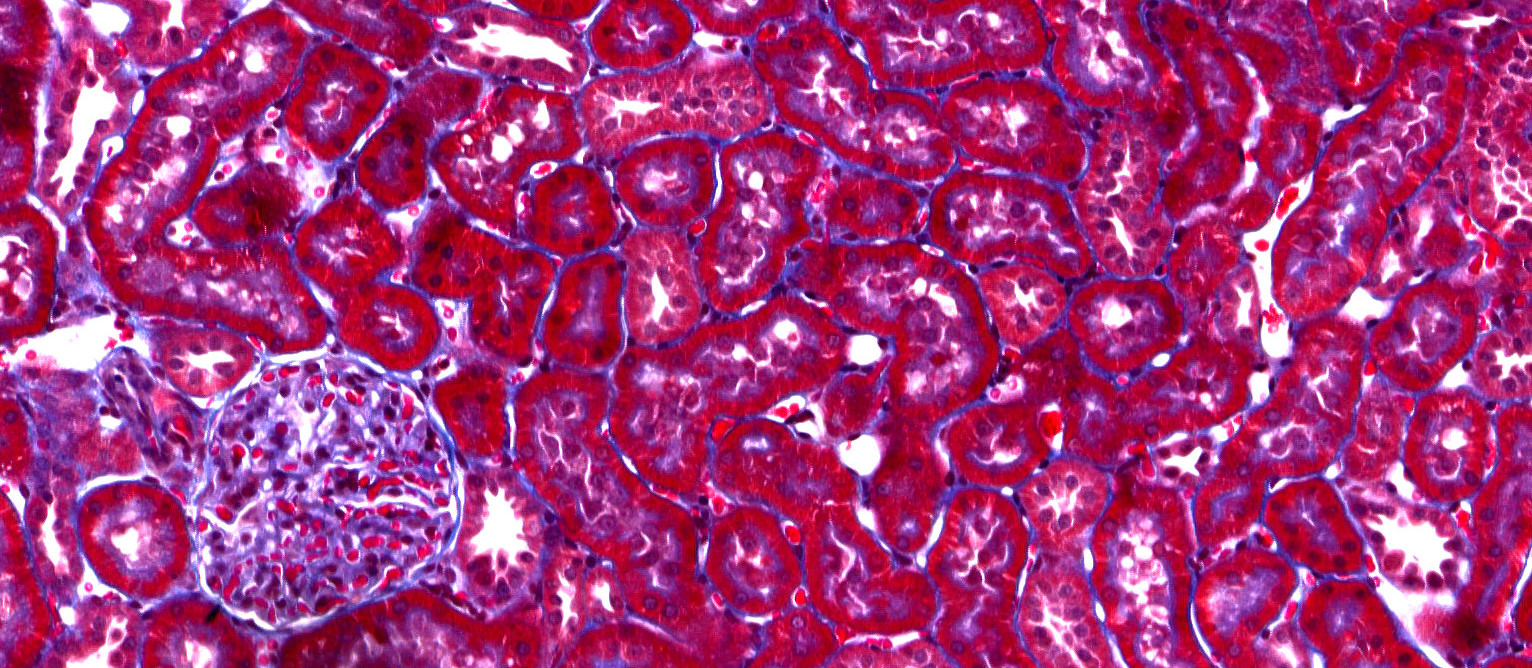

Supplement: Supplementary file 5 [file DataSheet9.ZIP › Fig 1D-masson-sham-6/6-8.jpeg]

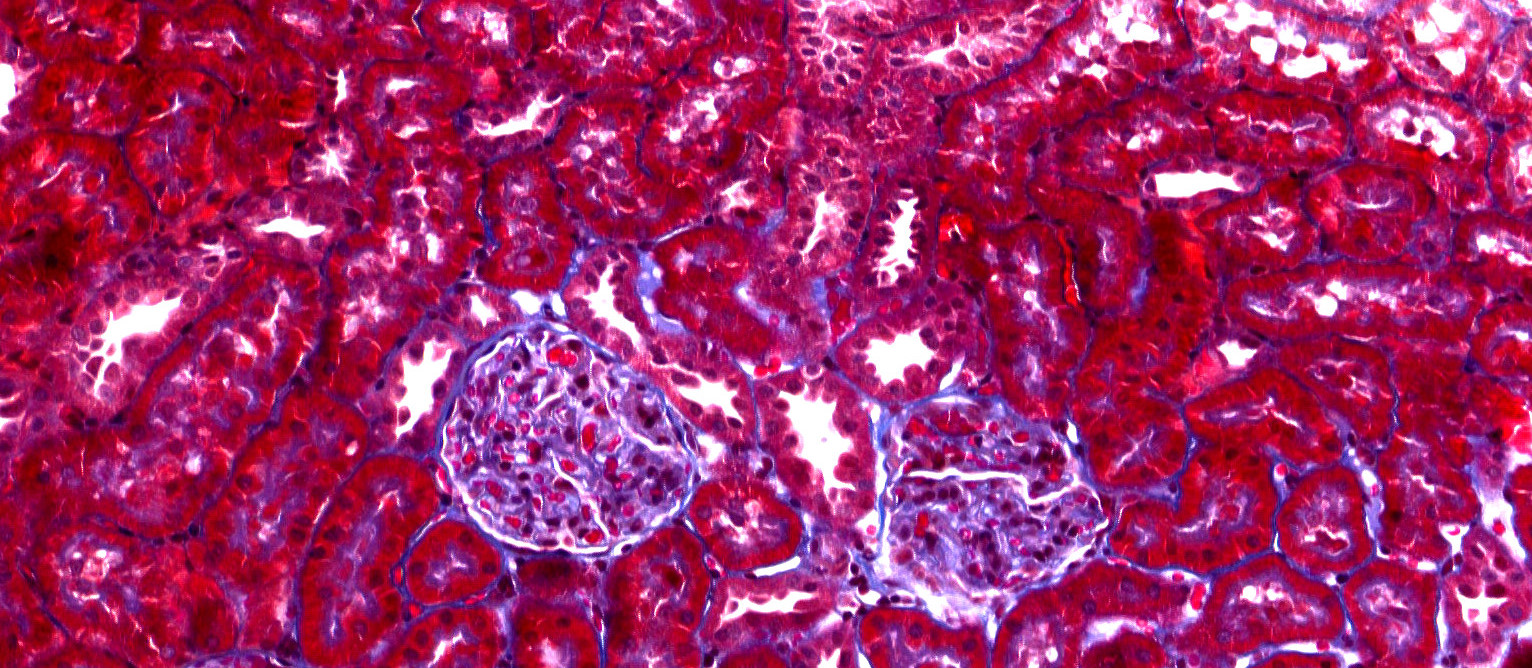

Supplement: Supplementary file 5 [file DataSheet9.ZIP › Fig 1D-masson-sham-6/6-9.jpeg]

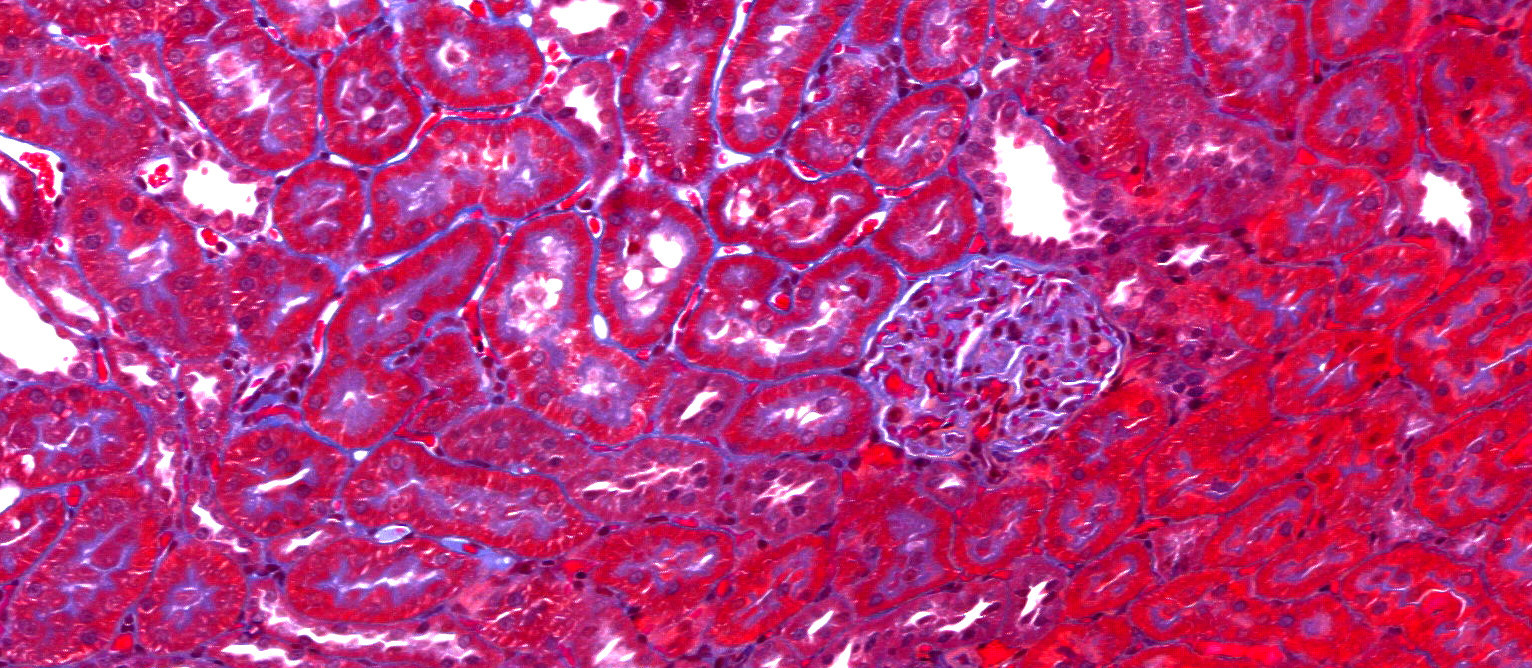

Supplement: Supplementary file 5 [file DataSheet9.ZIP › Fig 1D-masson-sham-7/7-1.jpeg]

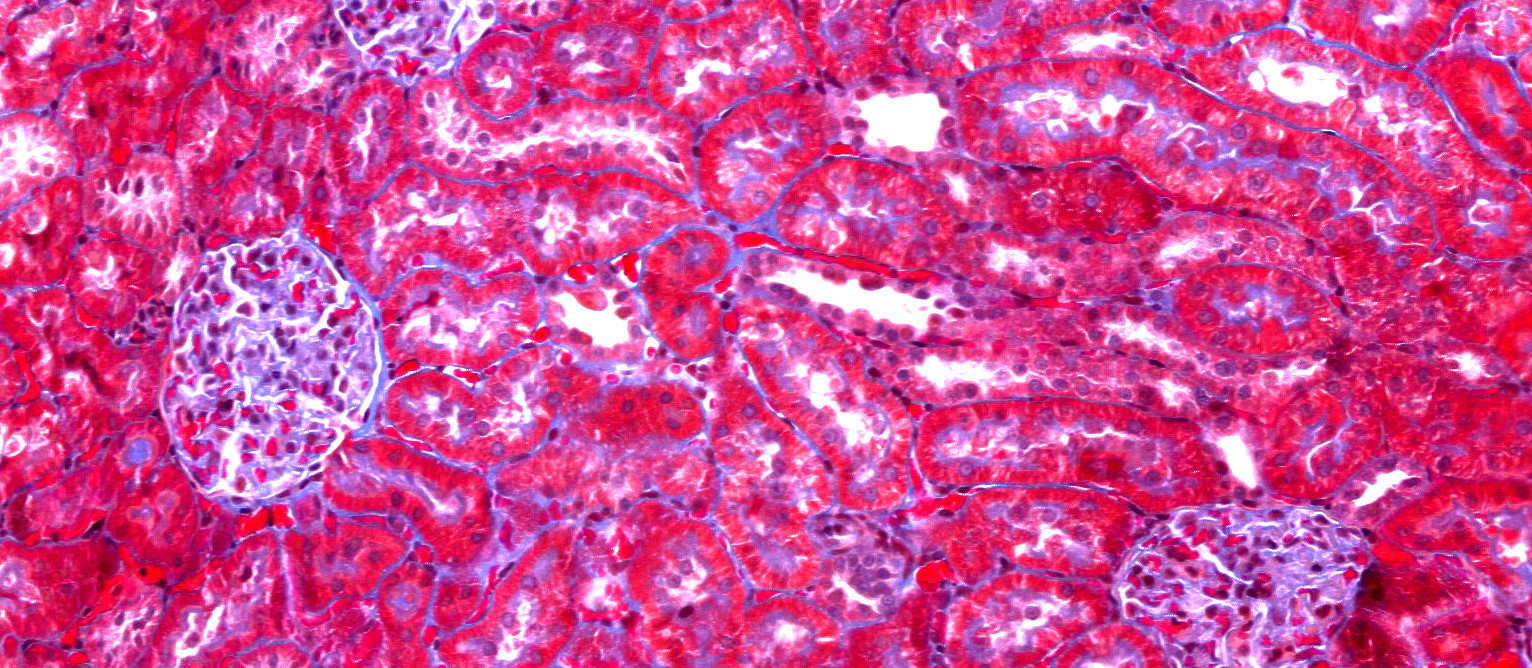

Supplement: Supplementary file 5 [file DataSheet9.ZIP › Fig 1D-masson-sham-7/7-10.jpeg]

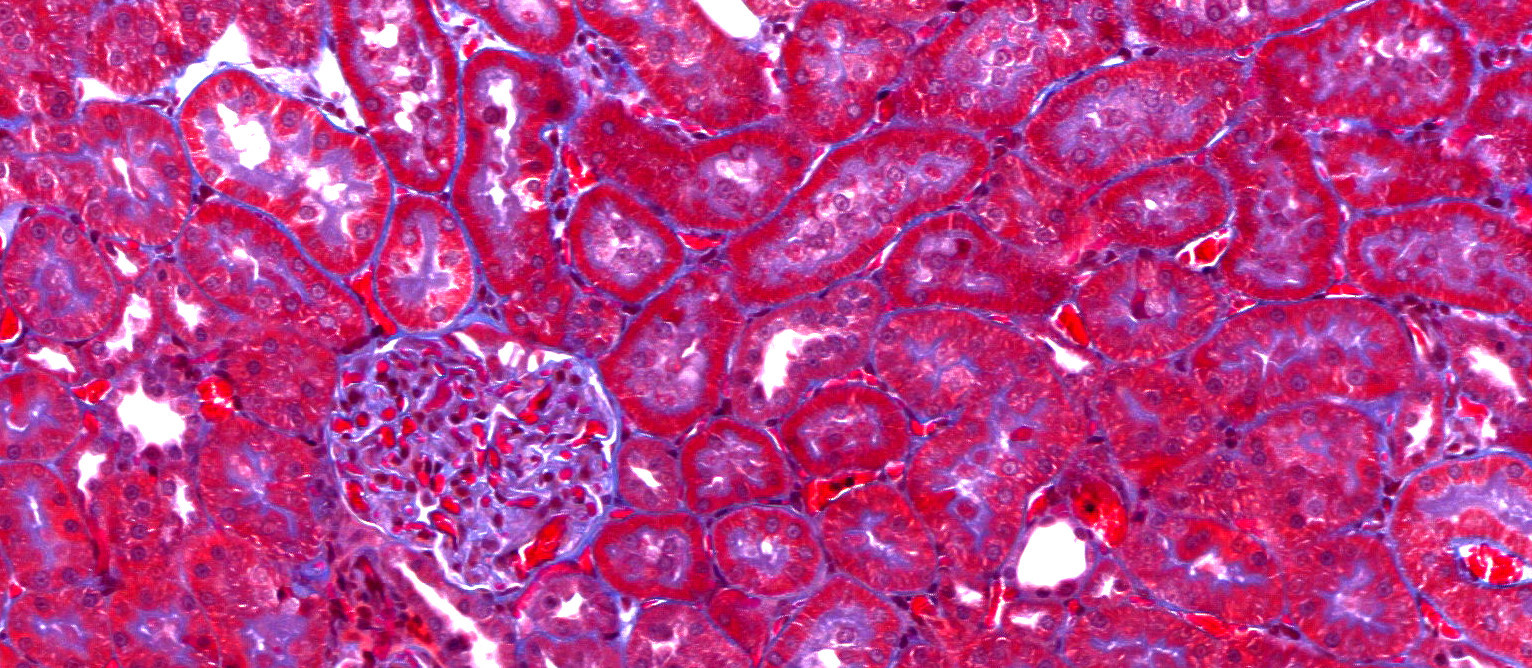

Supplement: Supplementary file 5 [file DataSheet9.ZIP › Fig 1D-masson-sham-7/7-2.jpeg]

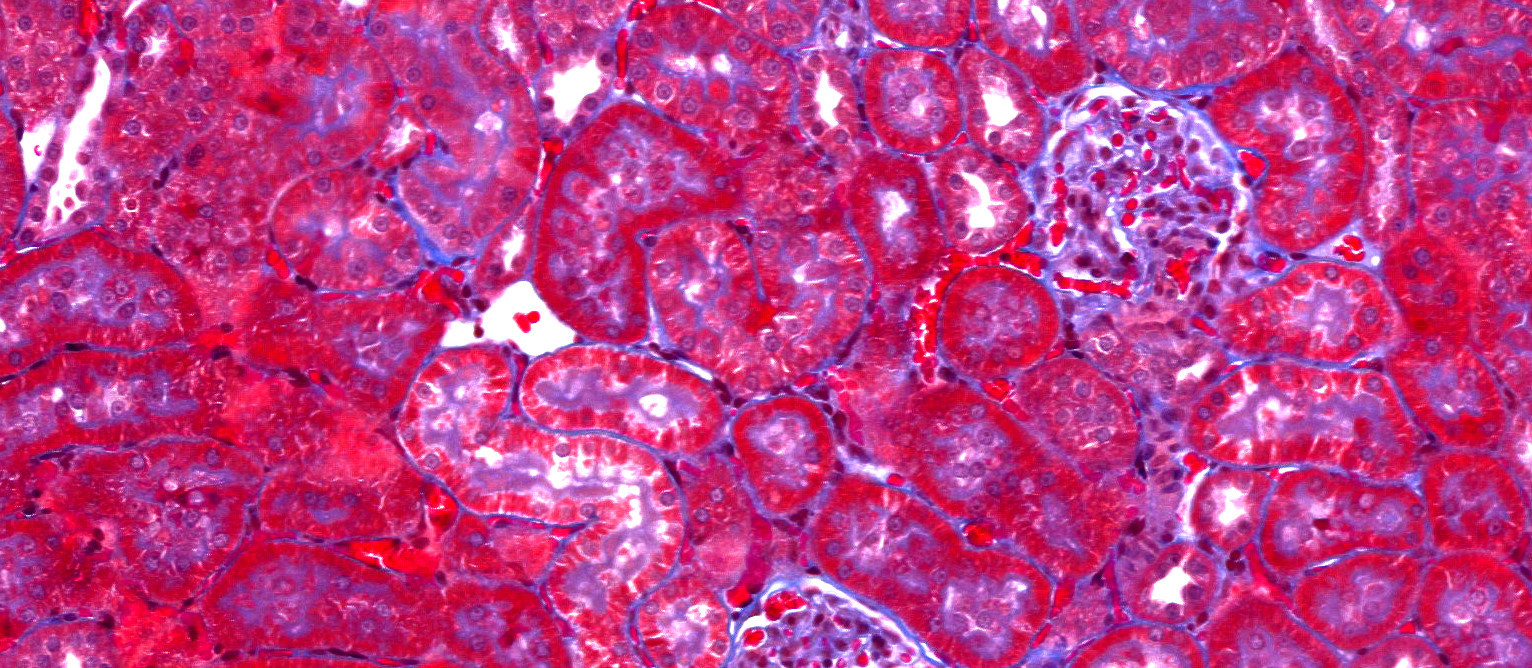

Supplement: Supplementary file 5 [file DataSheet9.ZIP › Fig 1D-masson-sham-7/7-3.jpeg]

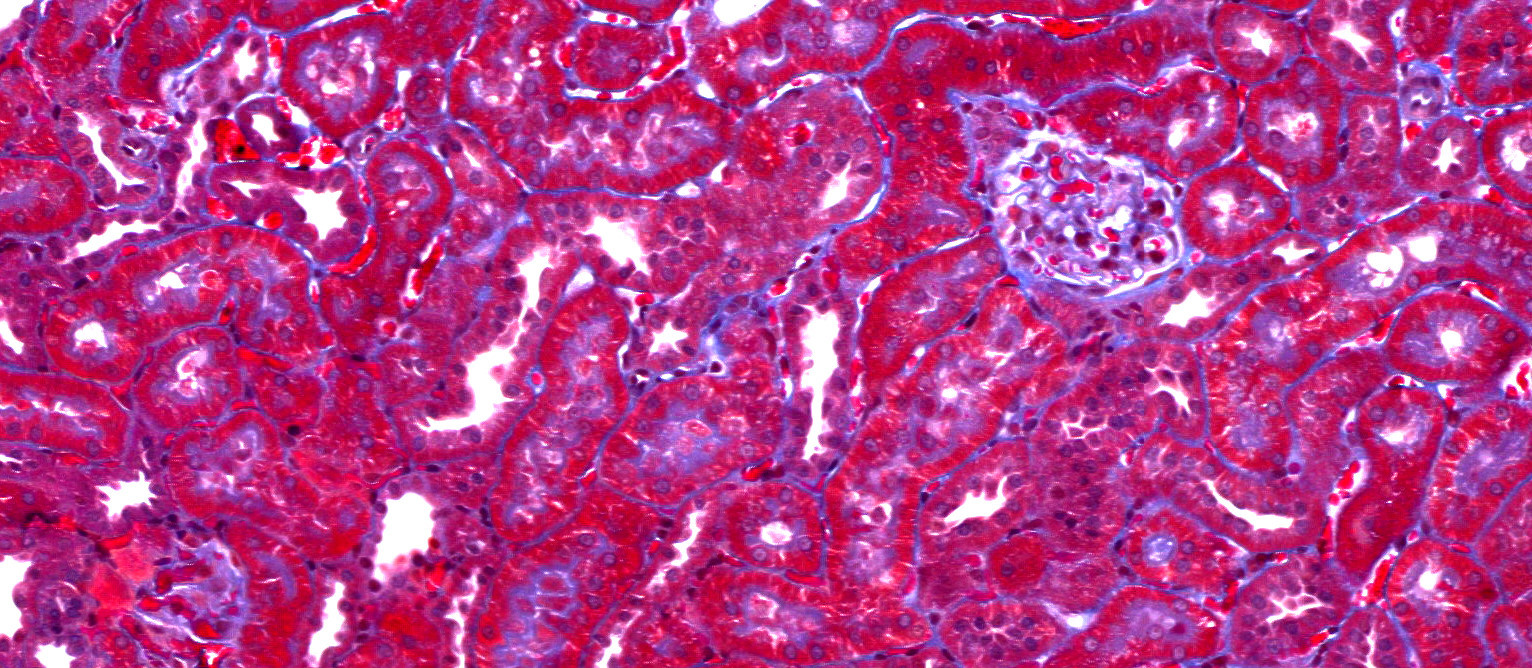

Supplement: Supplementary file 5 [file DataSheet9.ZIP › Fig 1D-masson-sham-7/7-4.jpeg]

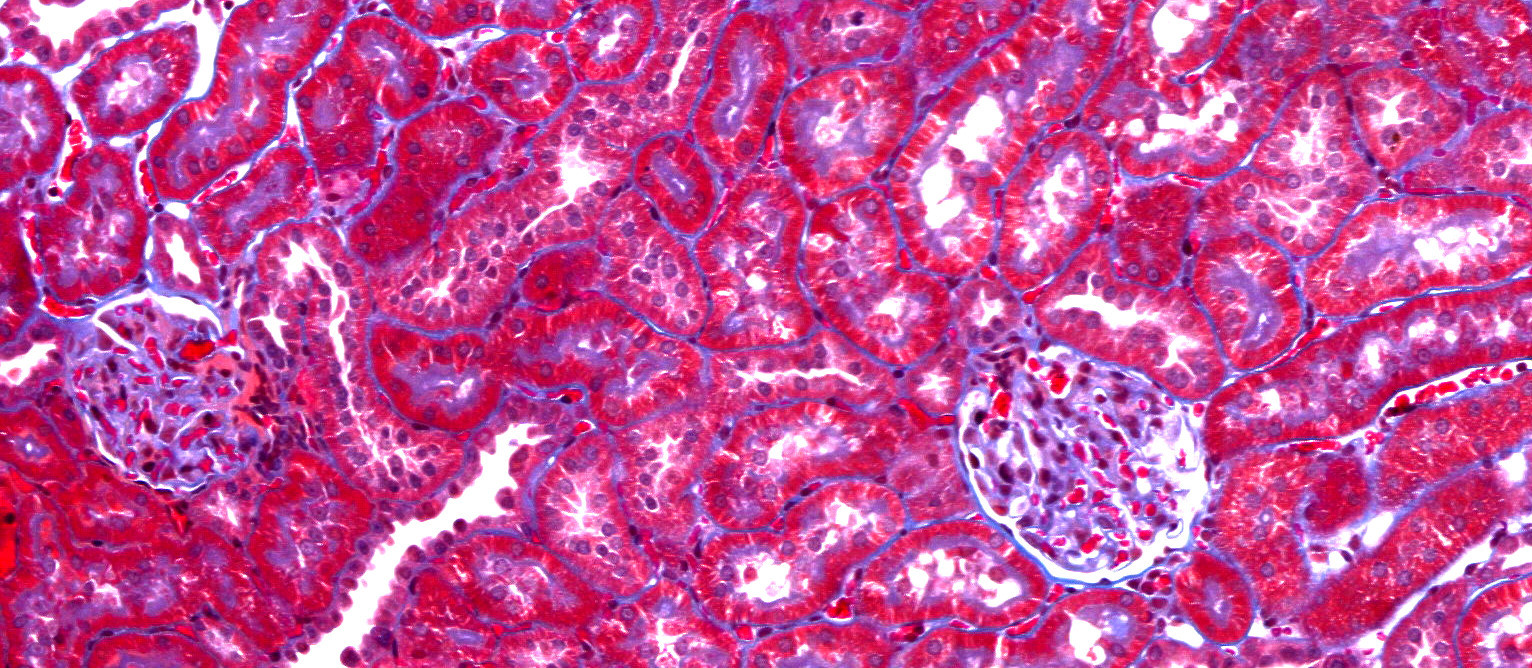

Supplement: Supplementary file 5 [file DataSheet9.ZIP › Fig 1D-masson-sham-7/7-5.jpeg]

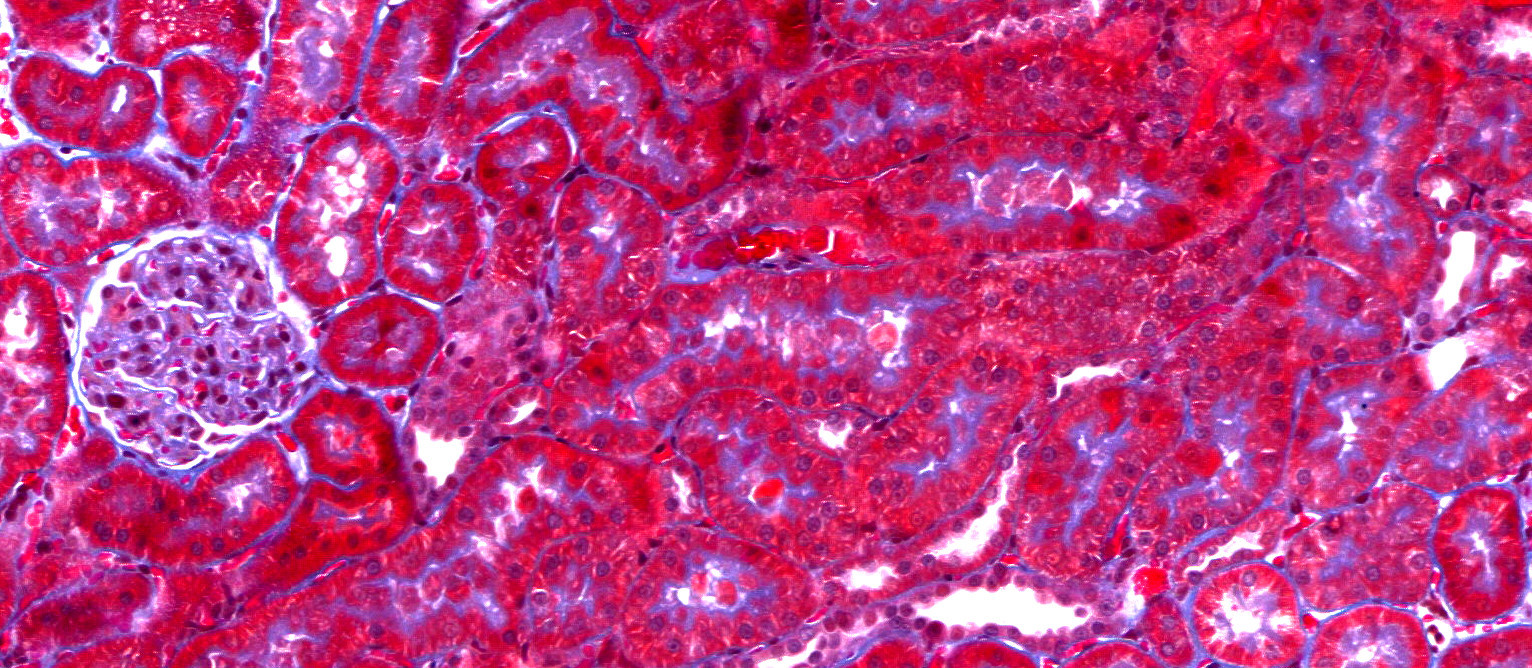

Supplement: Supplementary file 5 [file DataSheet9.ZIP › Fig 1D-masson-sham-7/7-6.jpeg]

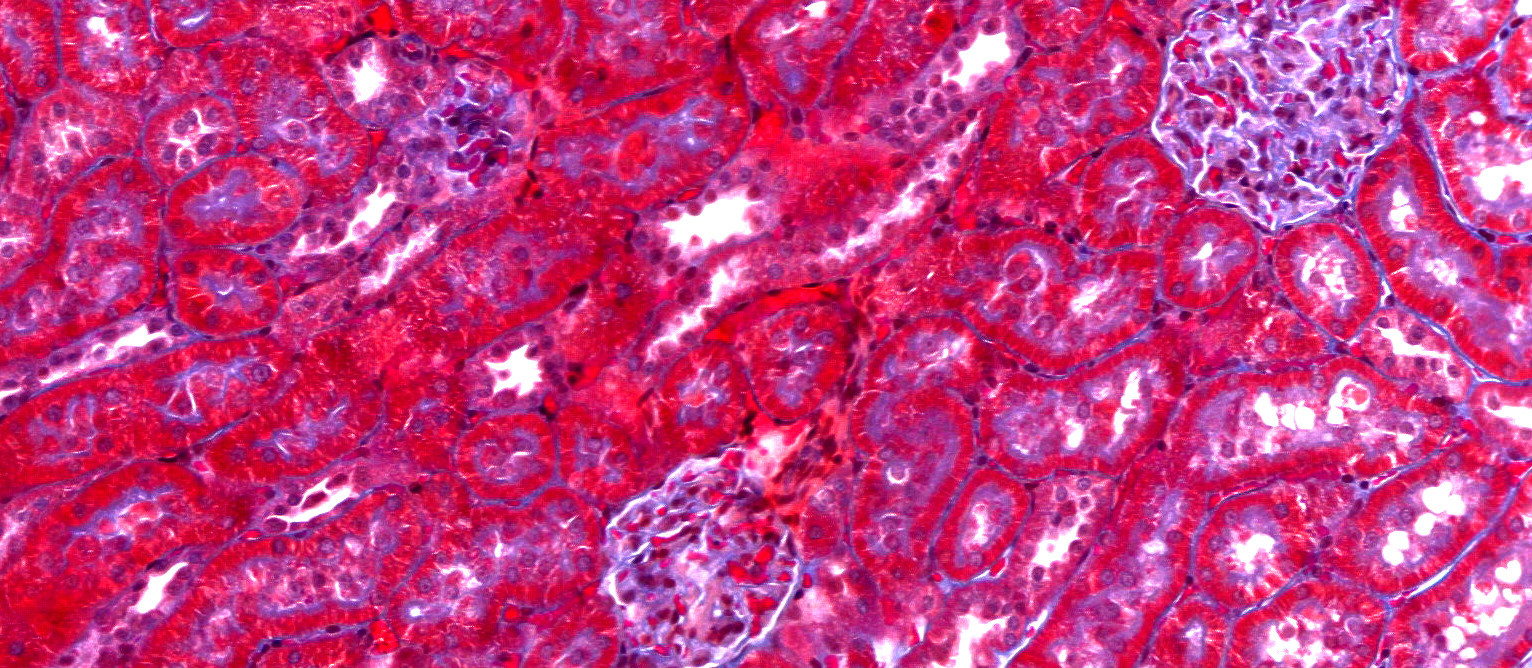

Supplement: Supplementary file 5 [file DataSheet9.ZIP › Fig 1D-masson-sham-7/7-7.jpeg]

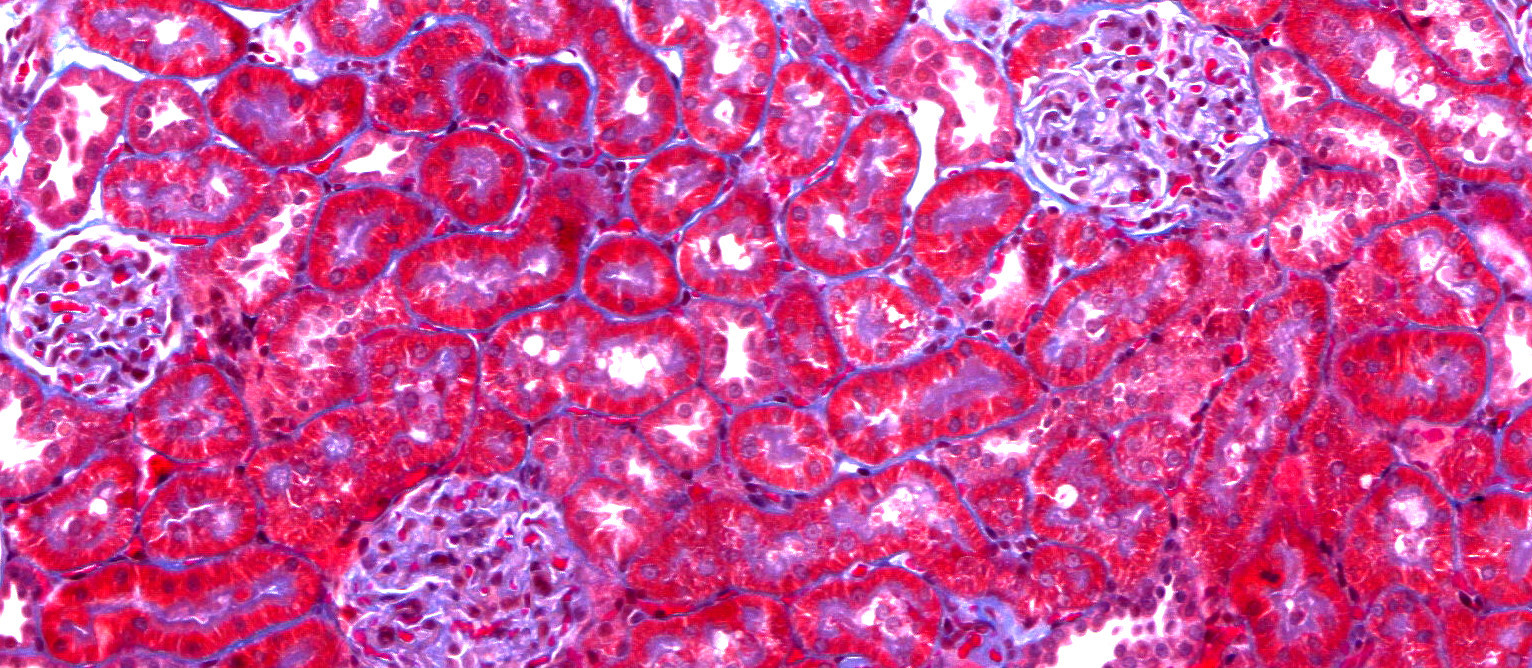

Supplement: Supplementary file 5 [file DataSheet9.ZIP › Fig 1D-masson-sham-7/7-8.jpeg]

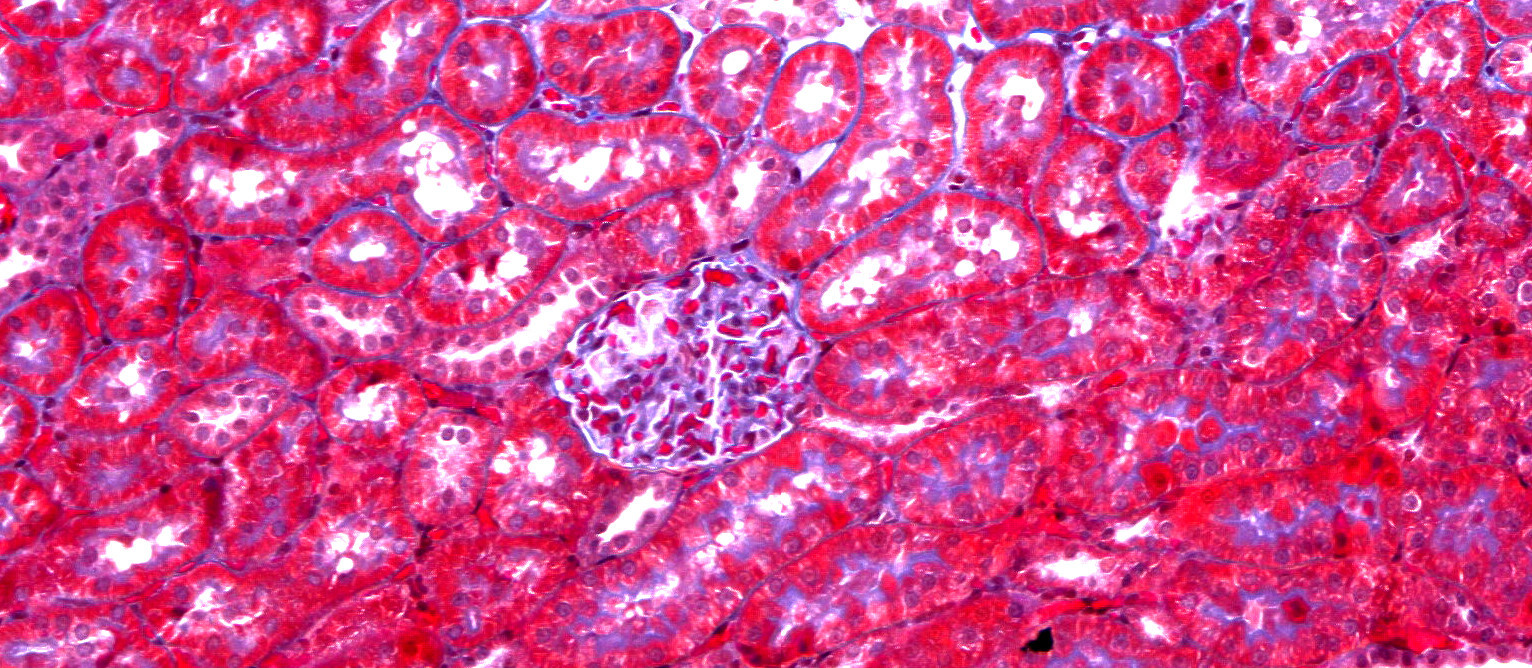

Supplement: Supplementary file 5 [file DataSheet9.ZIP › Fig 1D-masson-sham-7/7-9.jpeg]

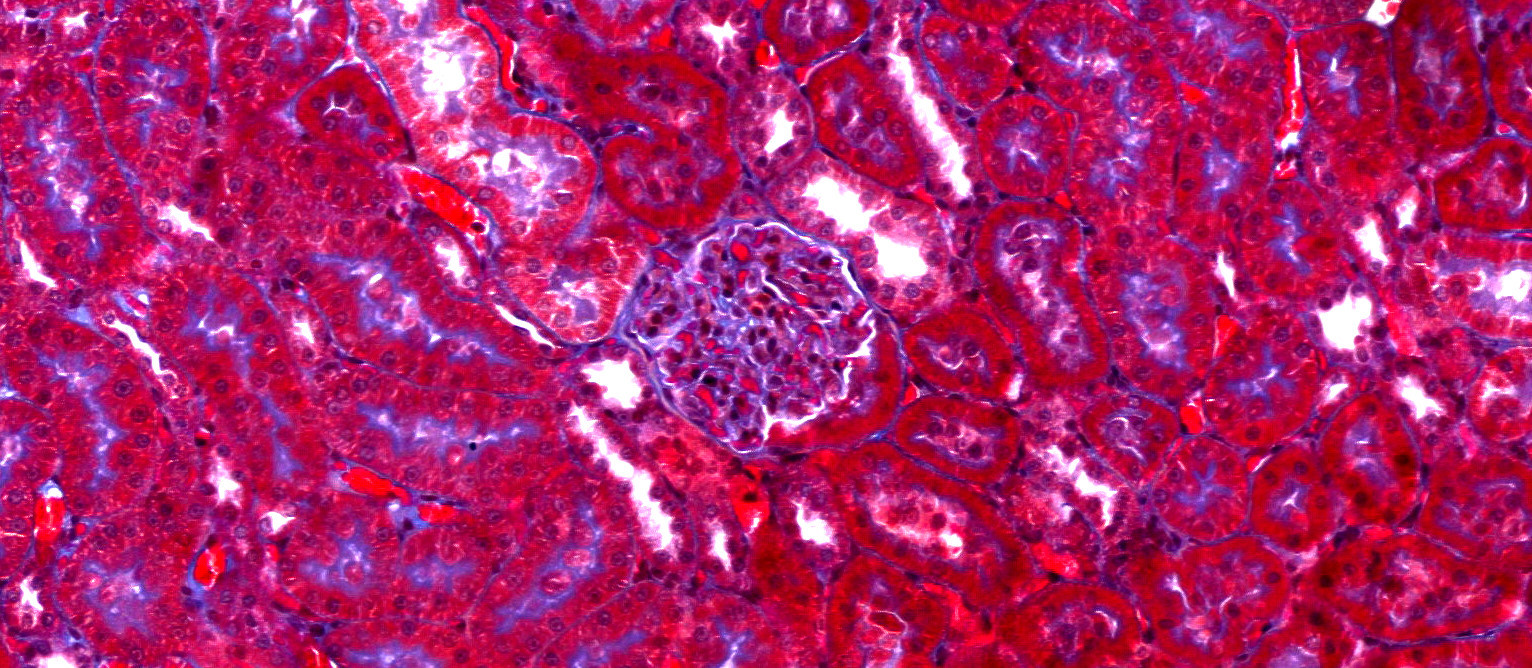

Supplement: Supplementary file 5 [file DataSheet9.ZIP › Fig 1D-masson-sham-8/8-1.jpeg]

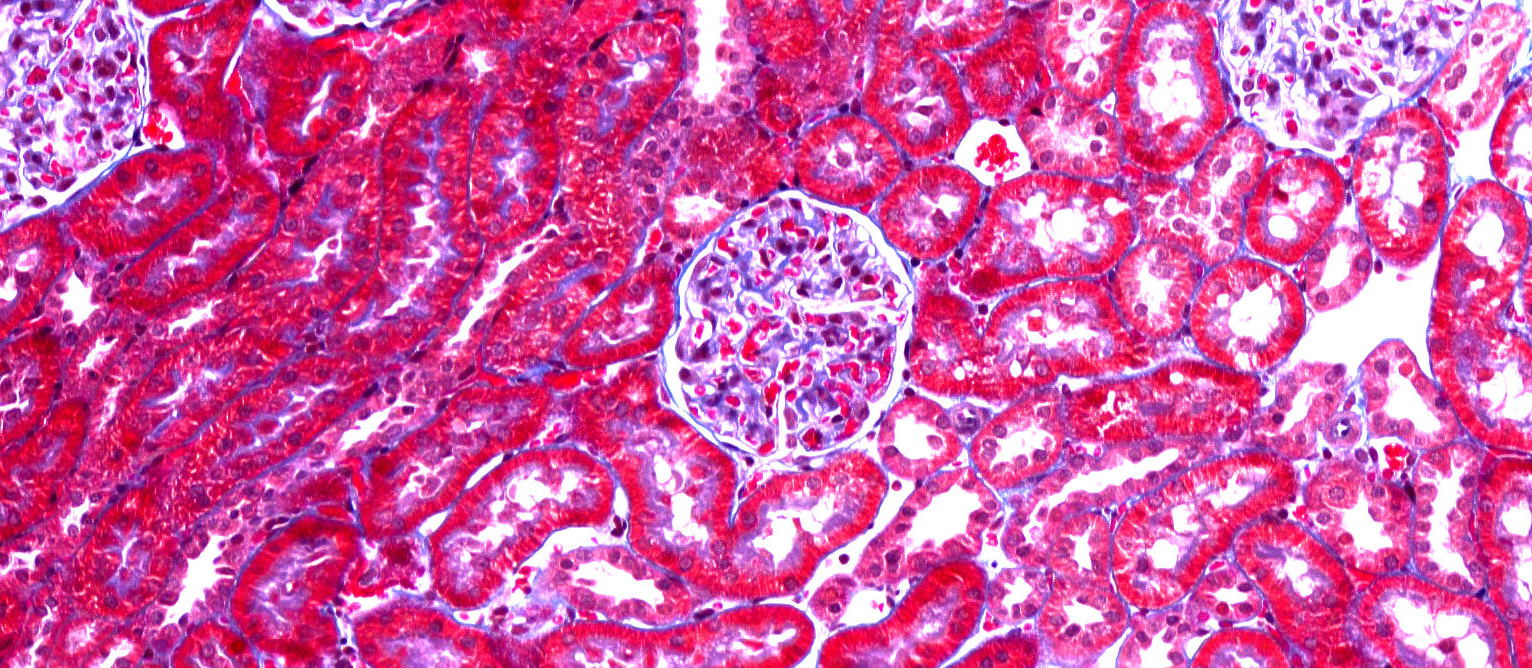

Supplement: Supplementary file 5 [file DataSheet9.ZIP › Fig 1D-masson-sham-8/8-10.jpeg]

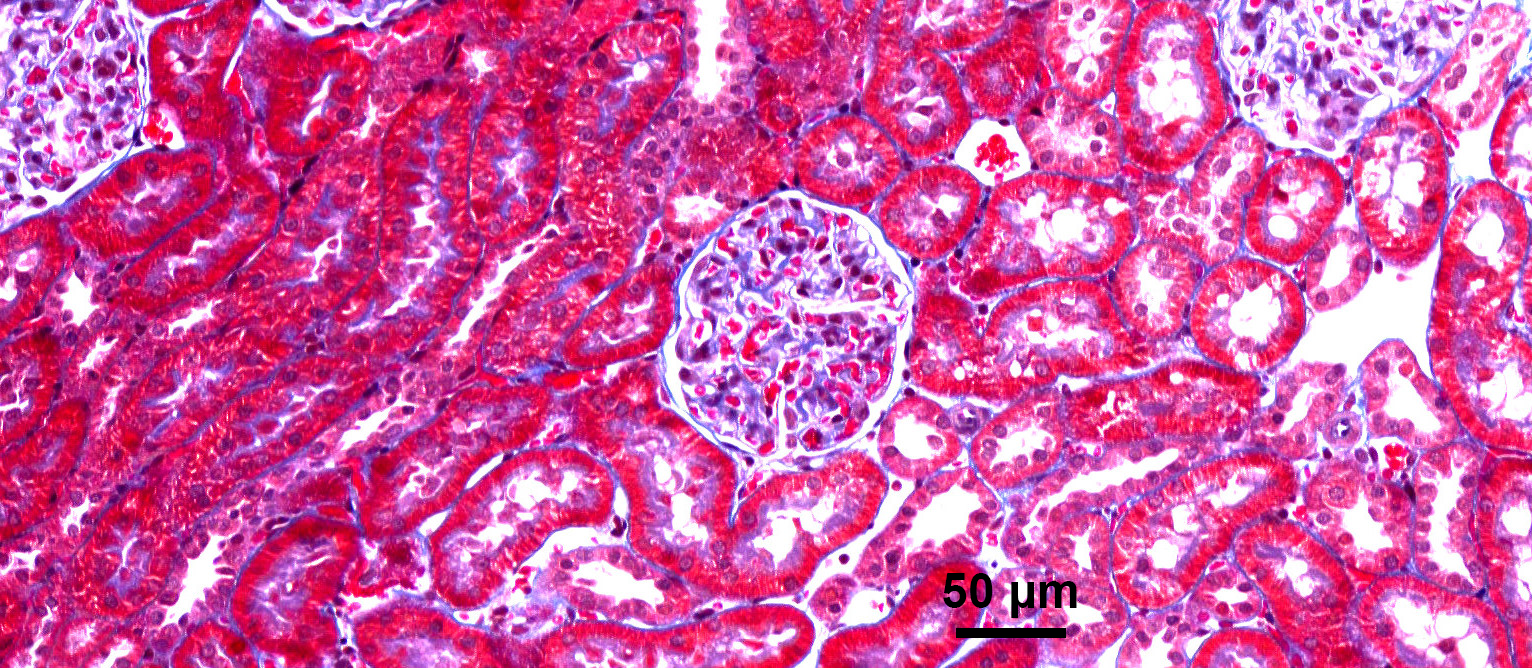

Supplement: Supplementary file 5 [file DataSheet9.ZIP › Fig 1D-masson-sham-8/8-10-1.png]

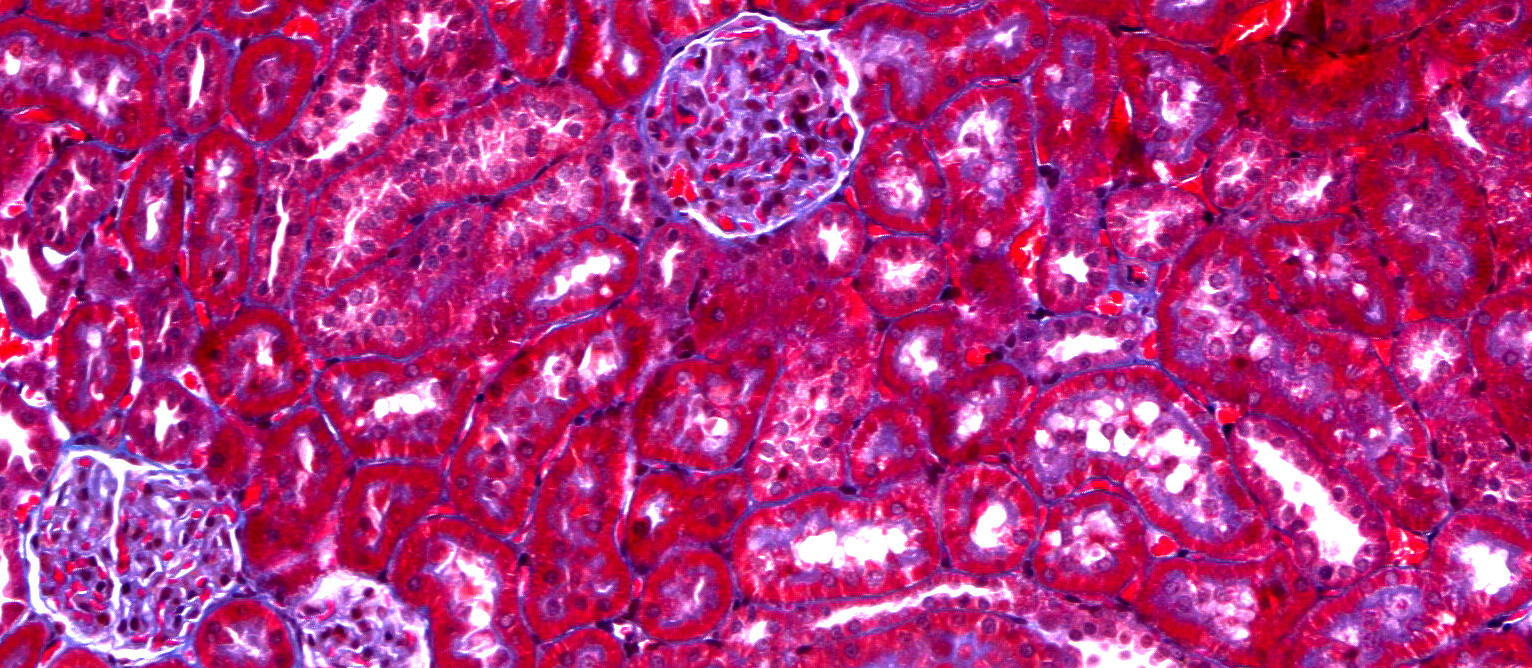

Supplement: Supplementary file 5 [file DataSheet9.ZIP › Fig 1D-masson-sham-8/8-2.jpeg]

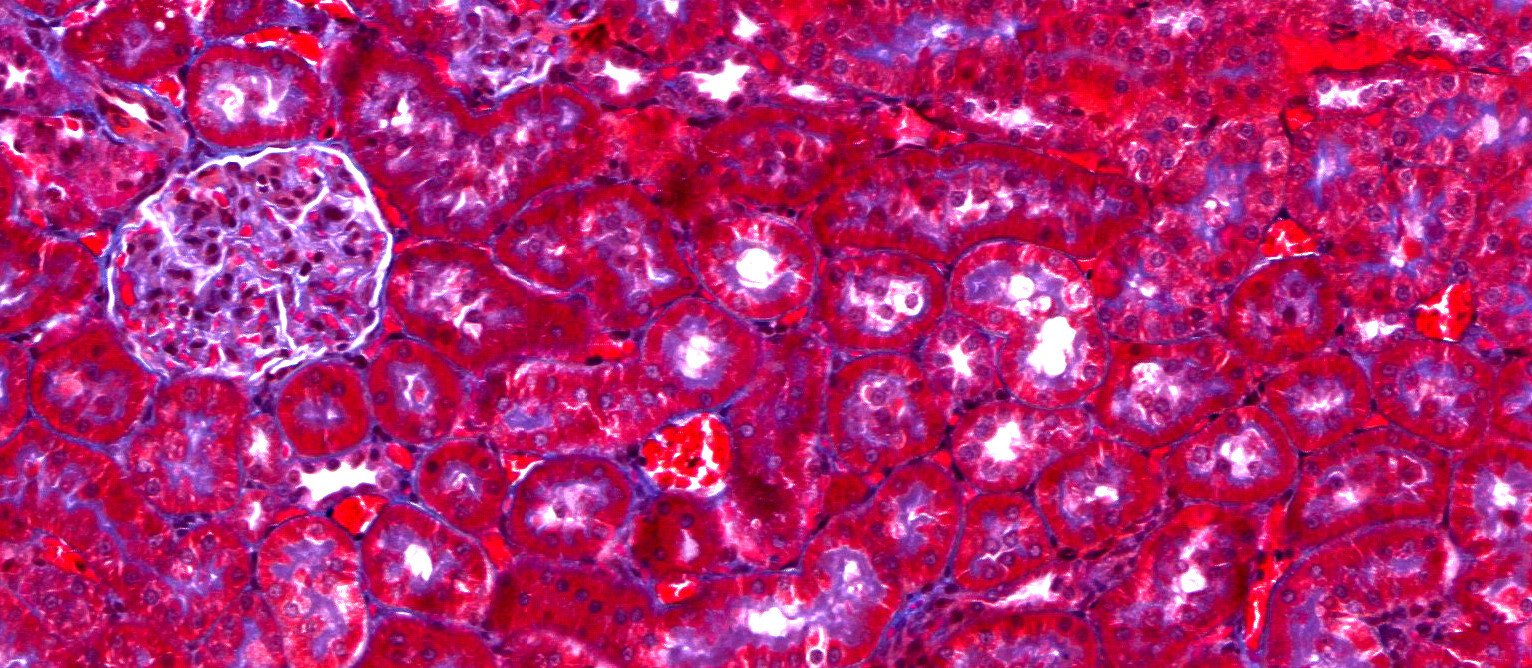

Supplement: Supplementary file 5 [file DataSheet9.ZIP › Fig 1D-masson-sham-8/8-3.jpeg]

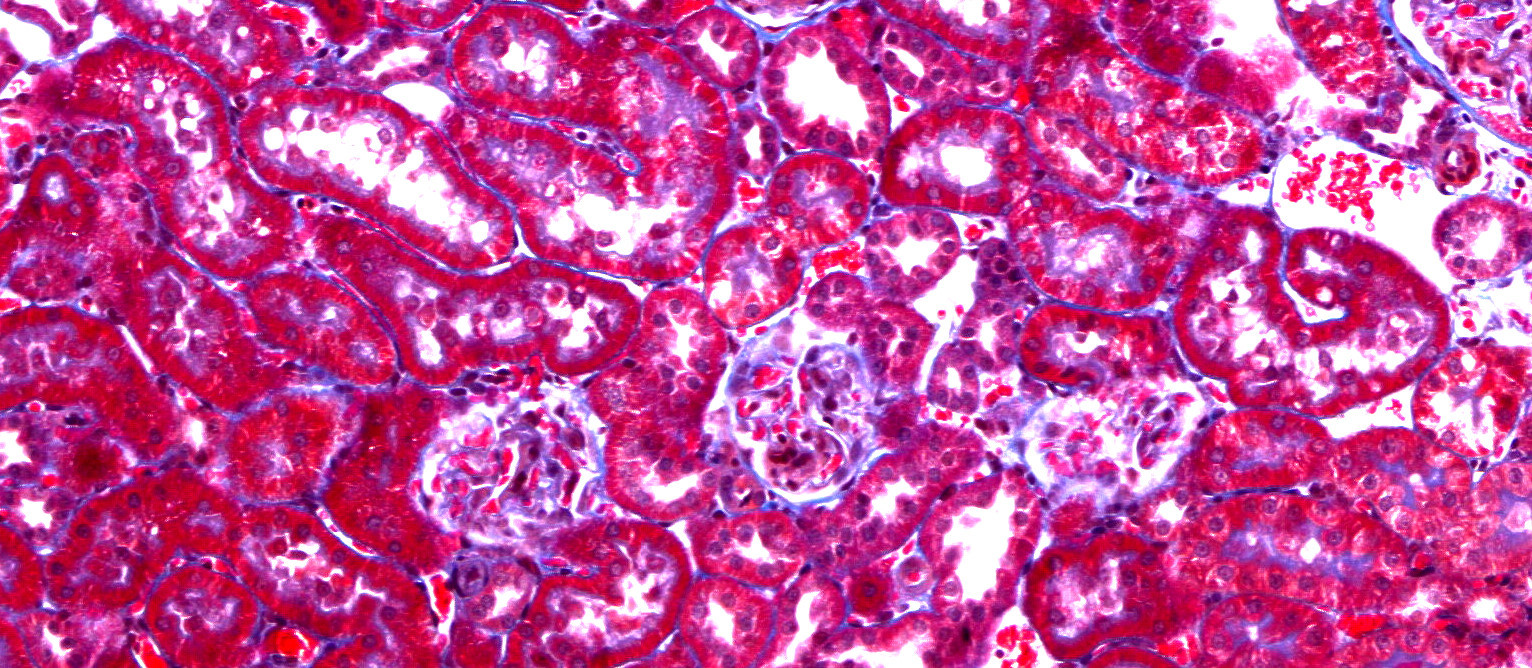

Supplement: Supplementary file 5 [file DataSheet9.ZIP › Fig 1D-masson-sham-8/8-4.jpeg]

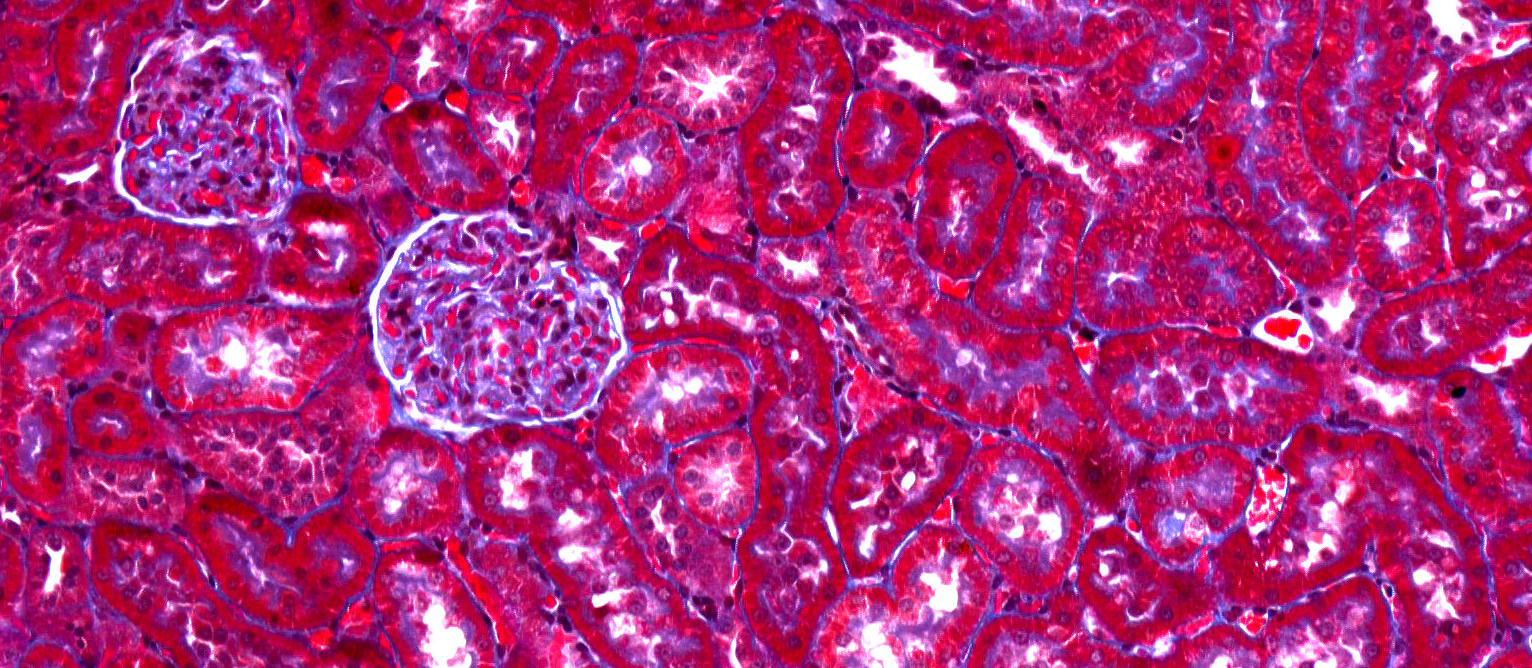

Supplement: Supplementary file 5 [file DataSheet9.ZIP › Fig 1D-masson-sham-8/8-5.jpeg]

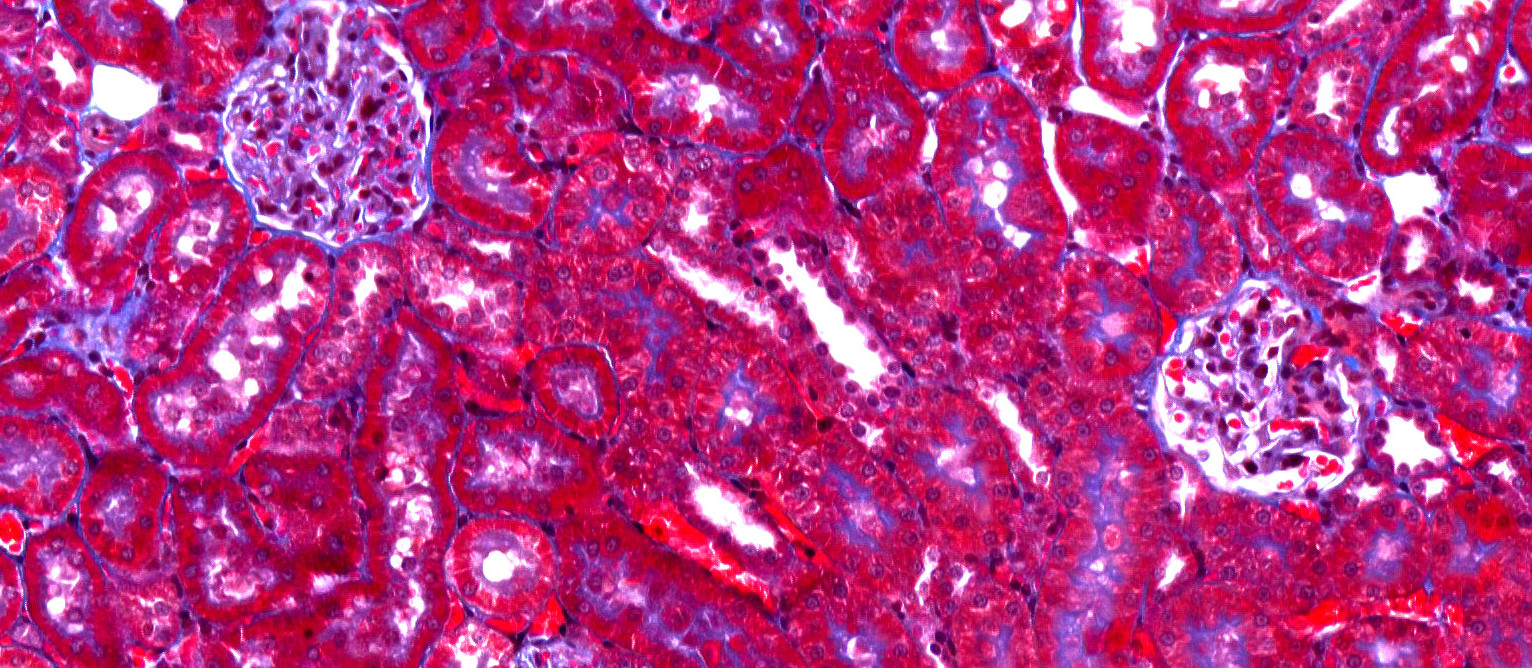

Supplement: Supplementary file 5 [file DataSheet9.ZIP › Fig 1D-masson-sham-8/8-6.jpeg]

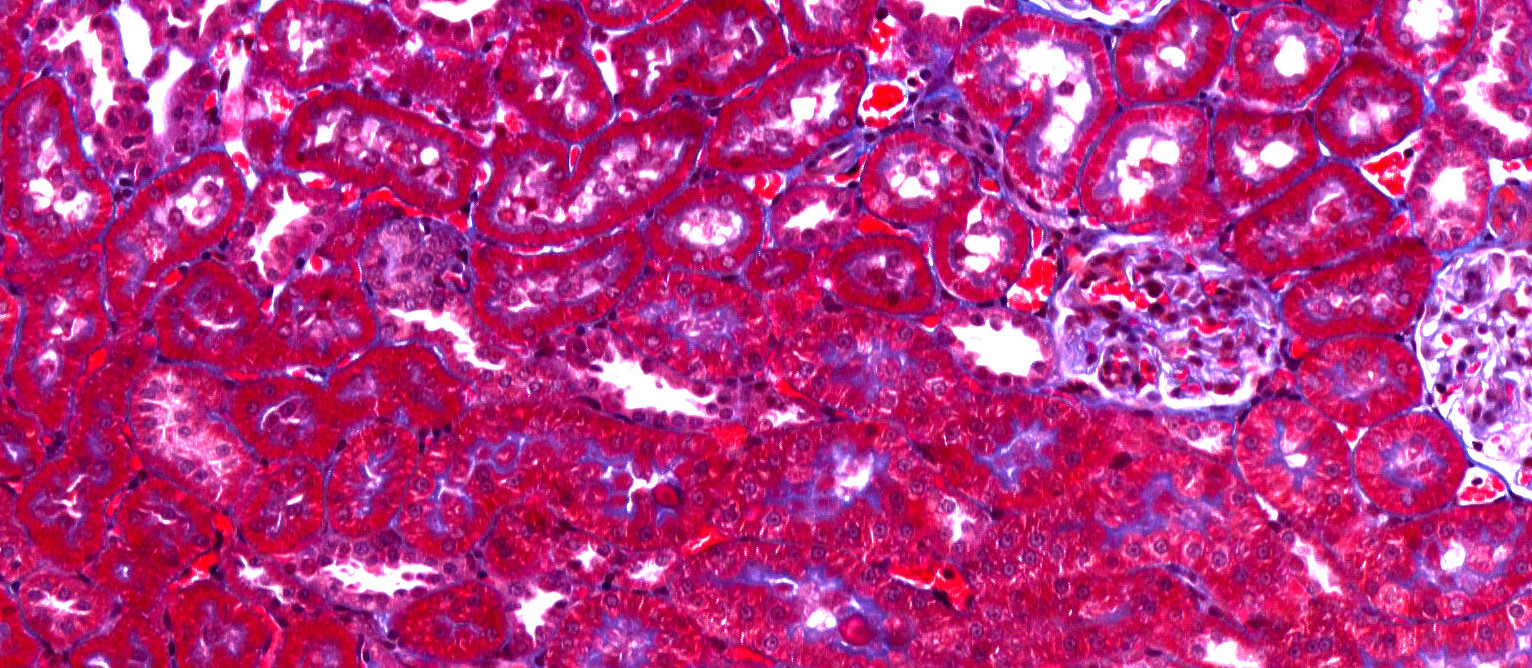

Supplement: Supplementary file 5 [file DataSheet9.ZIP › Fig 1D-masson-sham-8/8-7.jpeg]

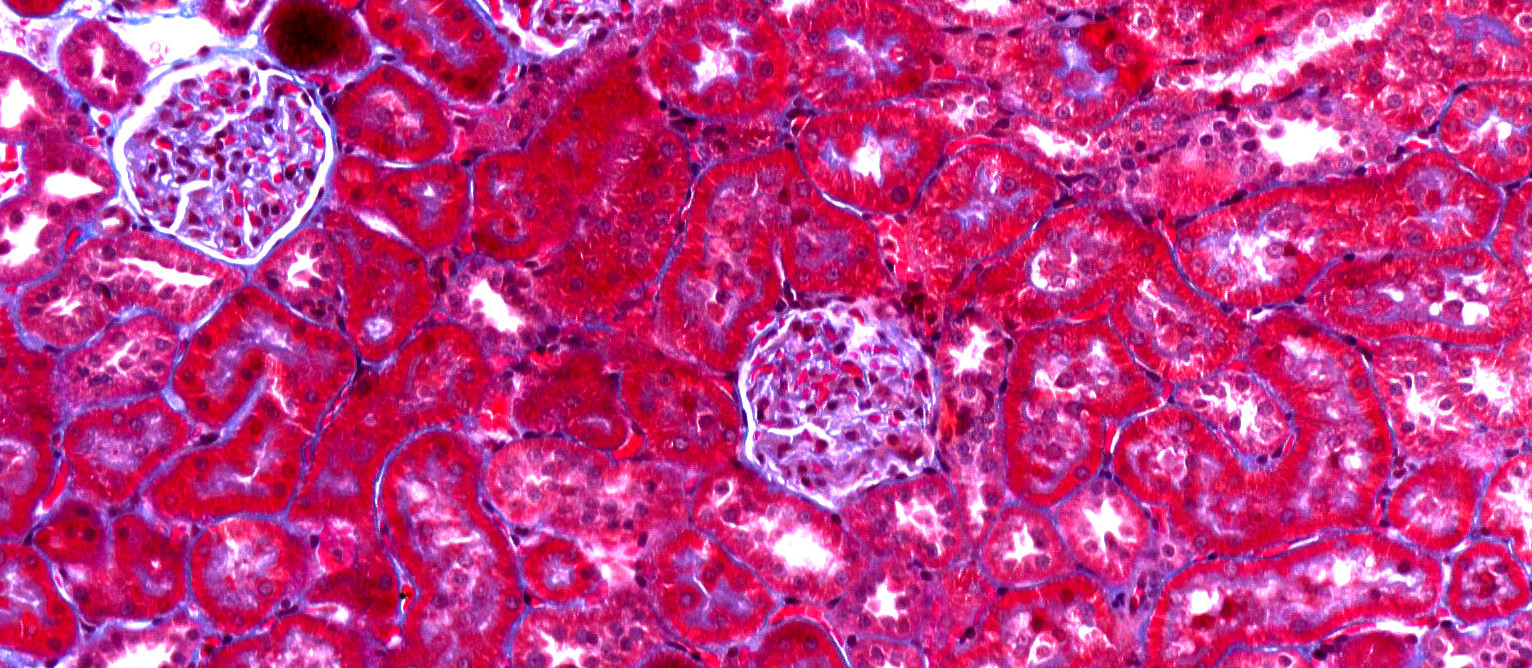

Supplement: Supplementary file 5 [file DataSheet9.ZIP › Fig 1D-masson-sham-8/8-8.jpeg]

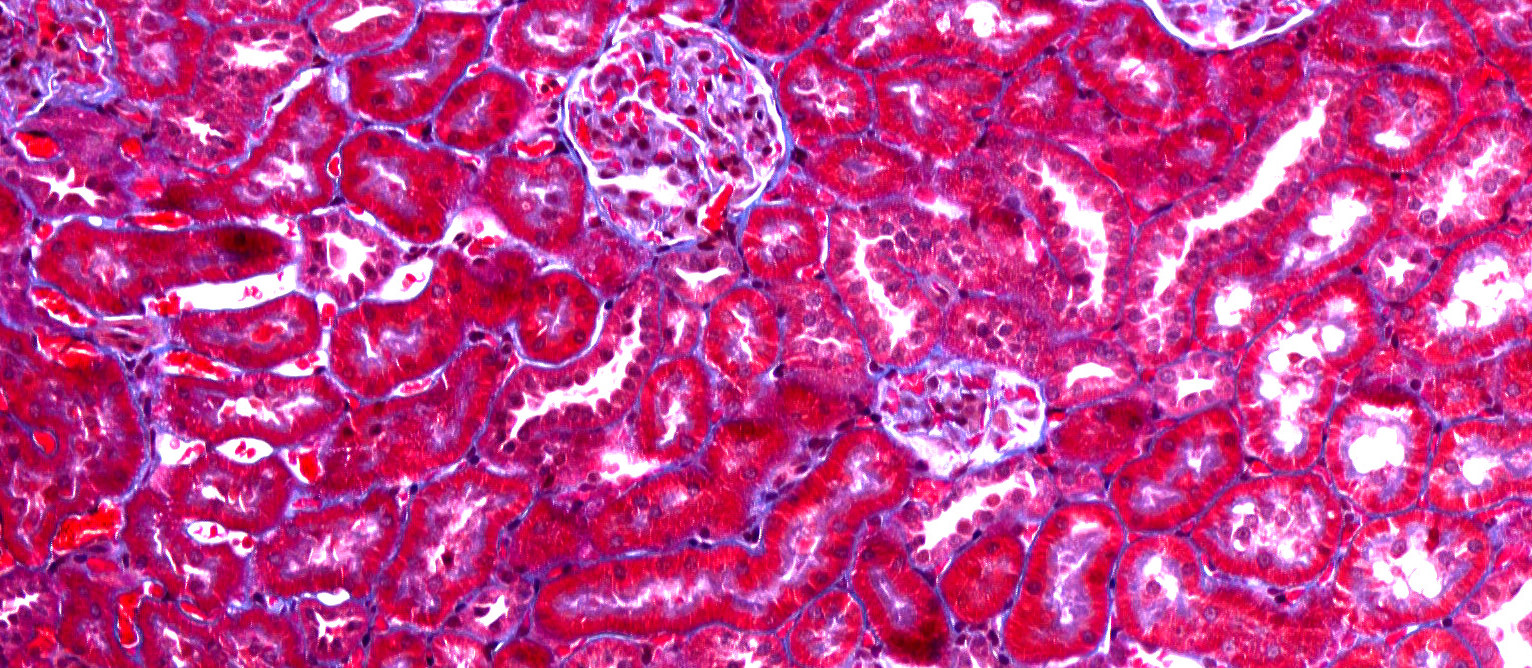

Supplement: Supplementary file 5 [file DataSheet9.ZIP › Fig 1D-masson-sham-8/8-9.jpeg]

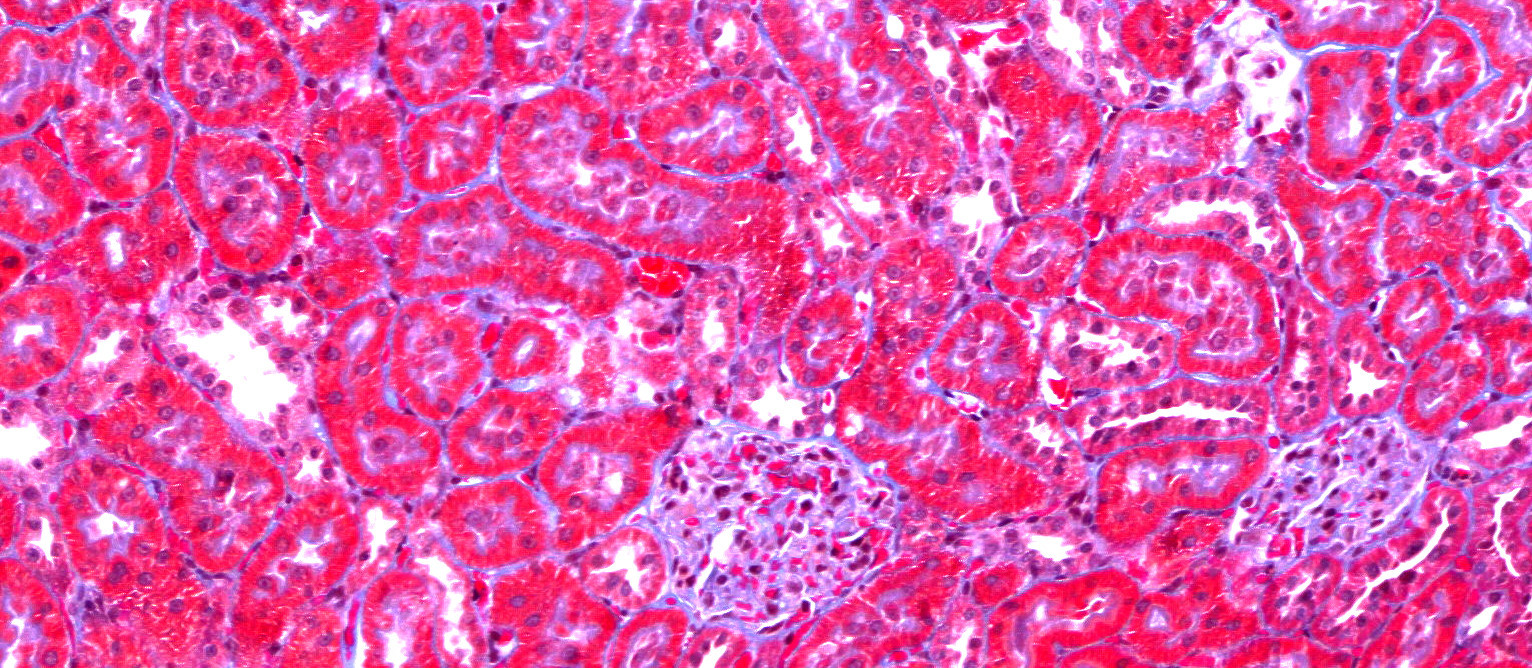

Supplement: Supplementary file 5 [file DataSheet9.ZIP › Fig 1D-masson-sham-9(1)/9-1.jpeg]

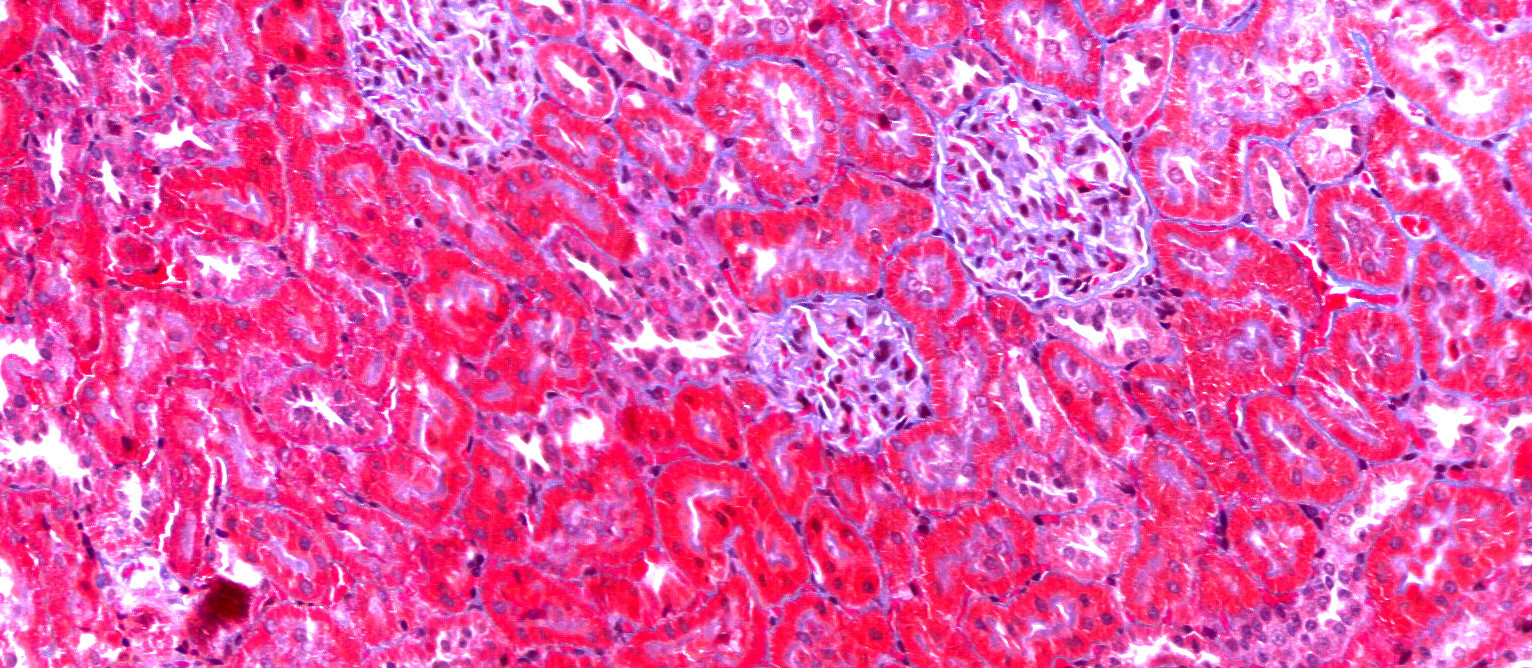

Supplement: Supplementary file 5 [file DataSheet9.ZIP › Fig 1D-masson-sham-9(1)/9-2.jpeg]

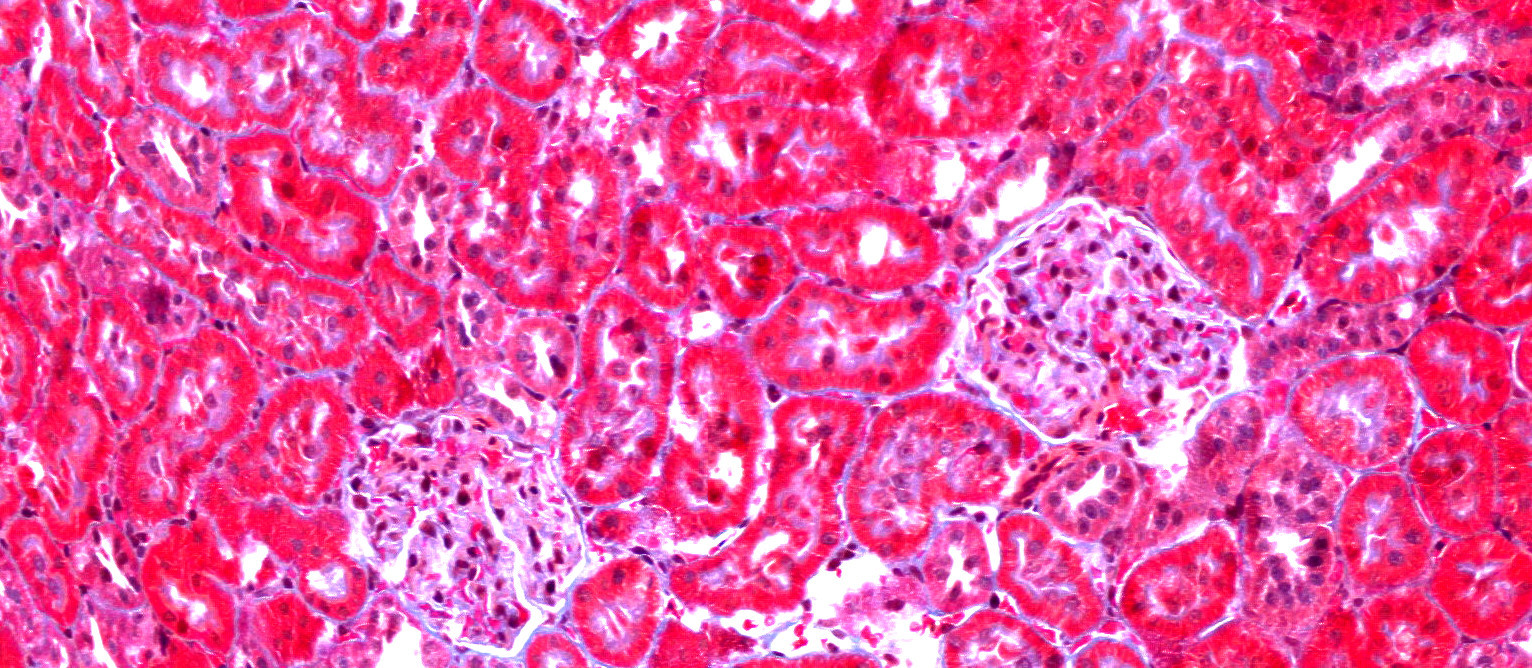

Supplement: Supplementary file 5 [file DataSheet9.ZIP › Fig 1D-masson-sham-9(1)/9-3.jpeg]

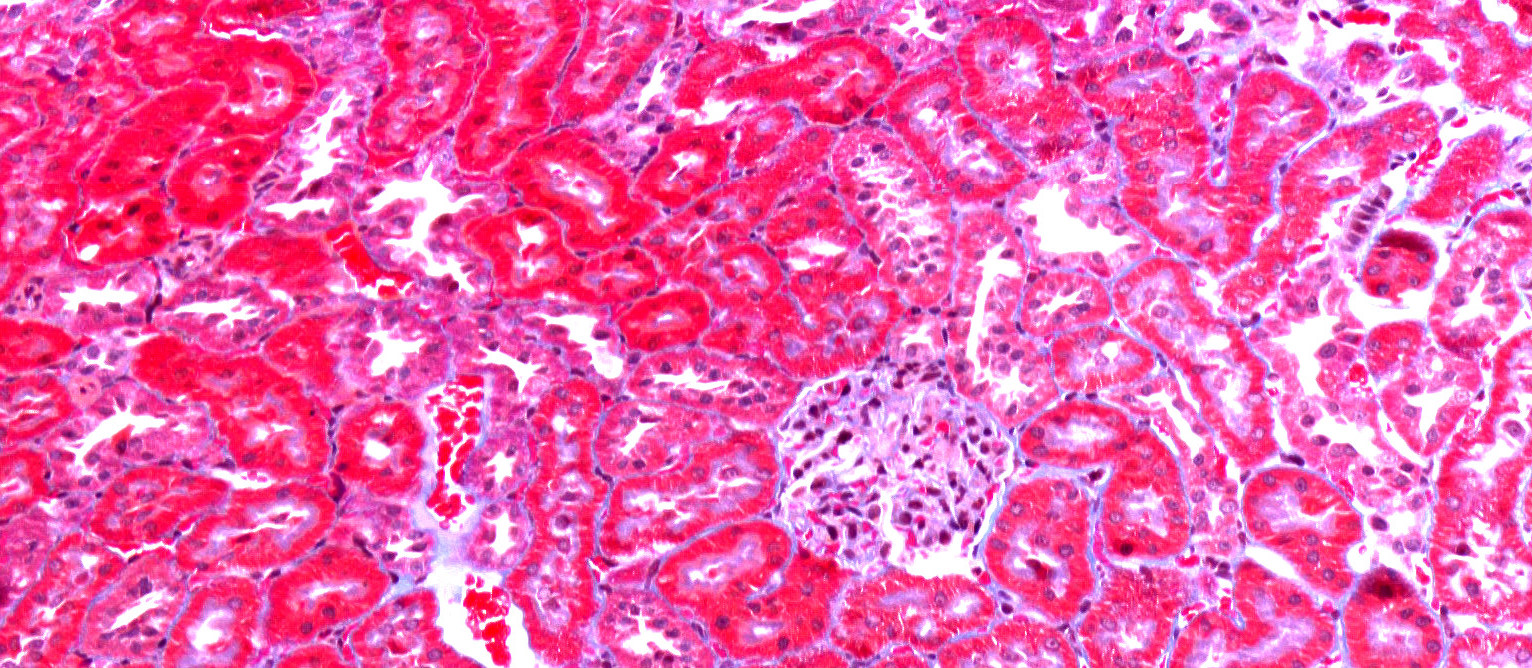

Supplement: Supplementary file 5 [file DataSheet9.ZIP › Fig 1D-masson-sham-9(1)/9-4.jpeg]

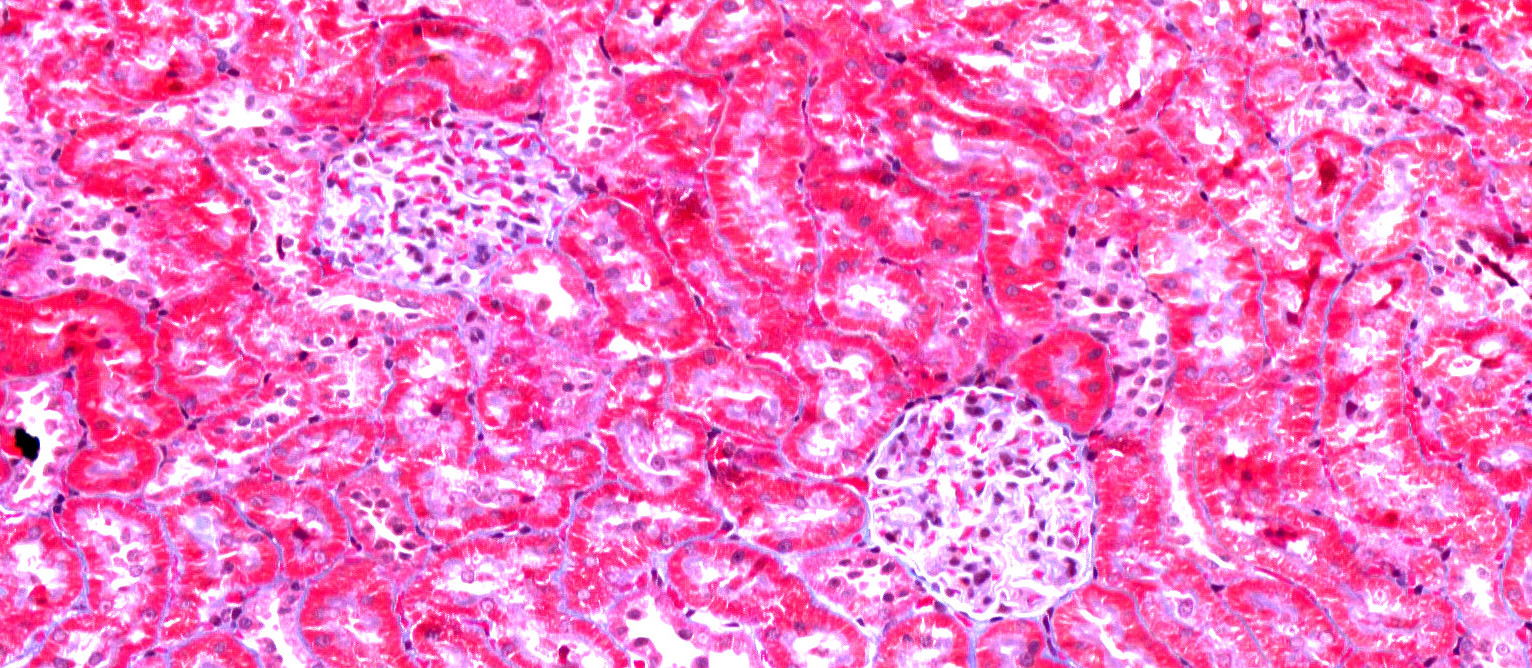

Supplement: Supplementary file 5 [file DataSheet9.ZIP › Fig 1D-masson-sham-9(1)/9-5.jpeg]

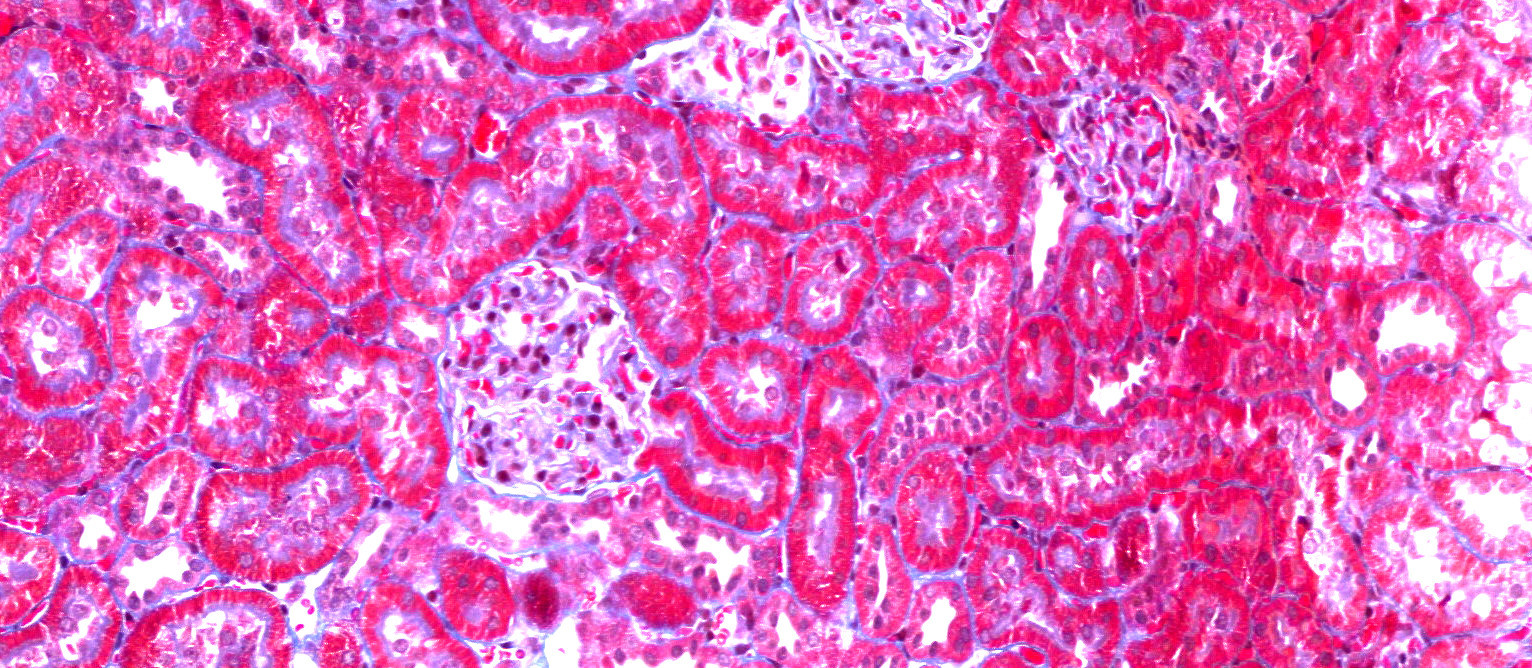

Supplement: Supplementary file 5 [file DataSheet9.ZIP › Fig 1D-masson-sham-9(1)/9-6.jpeg]

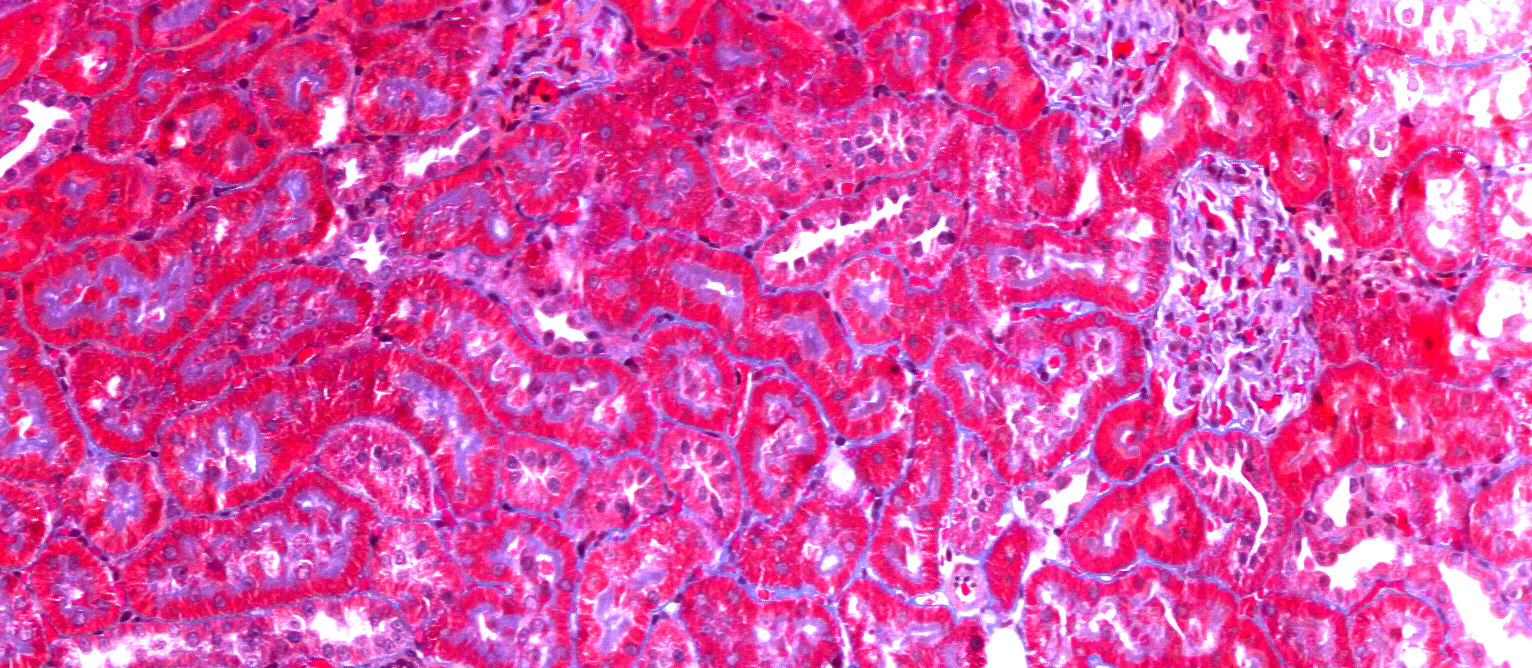

Supplement: Supplementary file 5 [file DataSheet9.ZIP › Fig 1D-masson-sham-9(1)/9-7.jpeg]

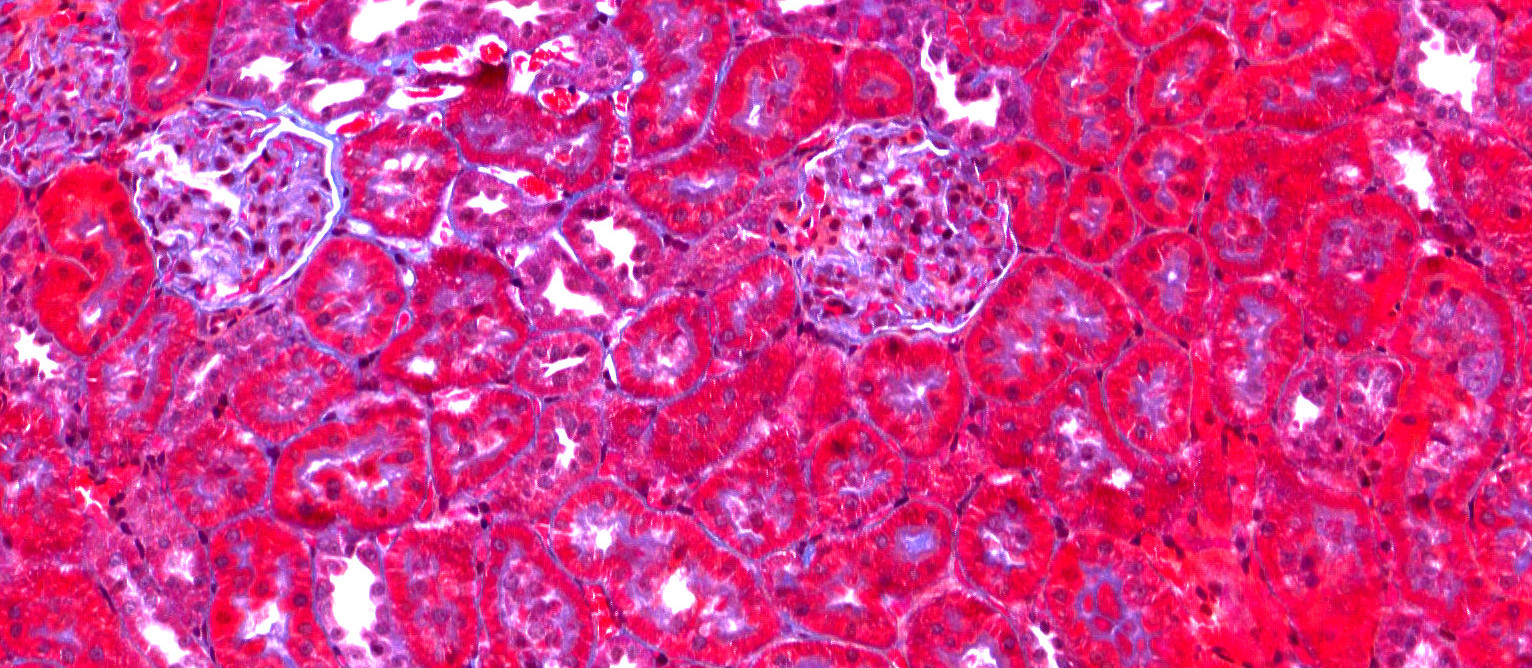

Supplement: Supplementary file 5 [file DataSheet9.ZIP › Fig 1D-masson-sham-9(1)/9-8.jpeg]
