# Supplementary figures and images for: Characteristics of Serum Metabolites and Gut Microbiota in Diabetic Kidney Disease (part 5 of 13)
Source: Front Pharmacol. 2022 Apr 14;13:872988. doi: 10.3389/fphar.2022.872988 (PMC9084235; doi:10.3389/fphar.2022.872988)

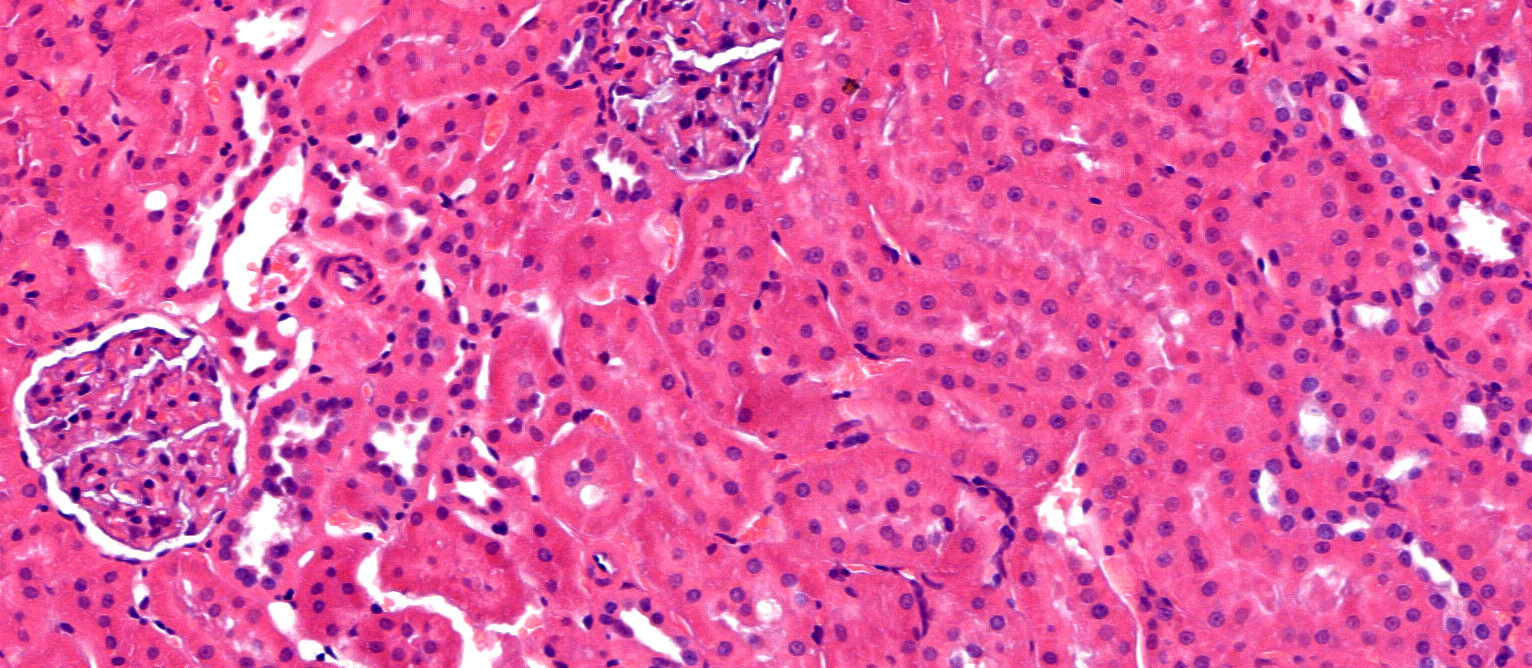

Supplement: Supplementary file 6 [file DataSheet4.ZIP › Fid 1D-HE-sham-6(2)/6-10.jpeg]

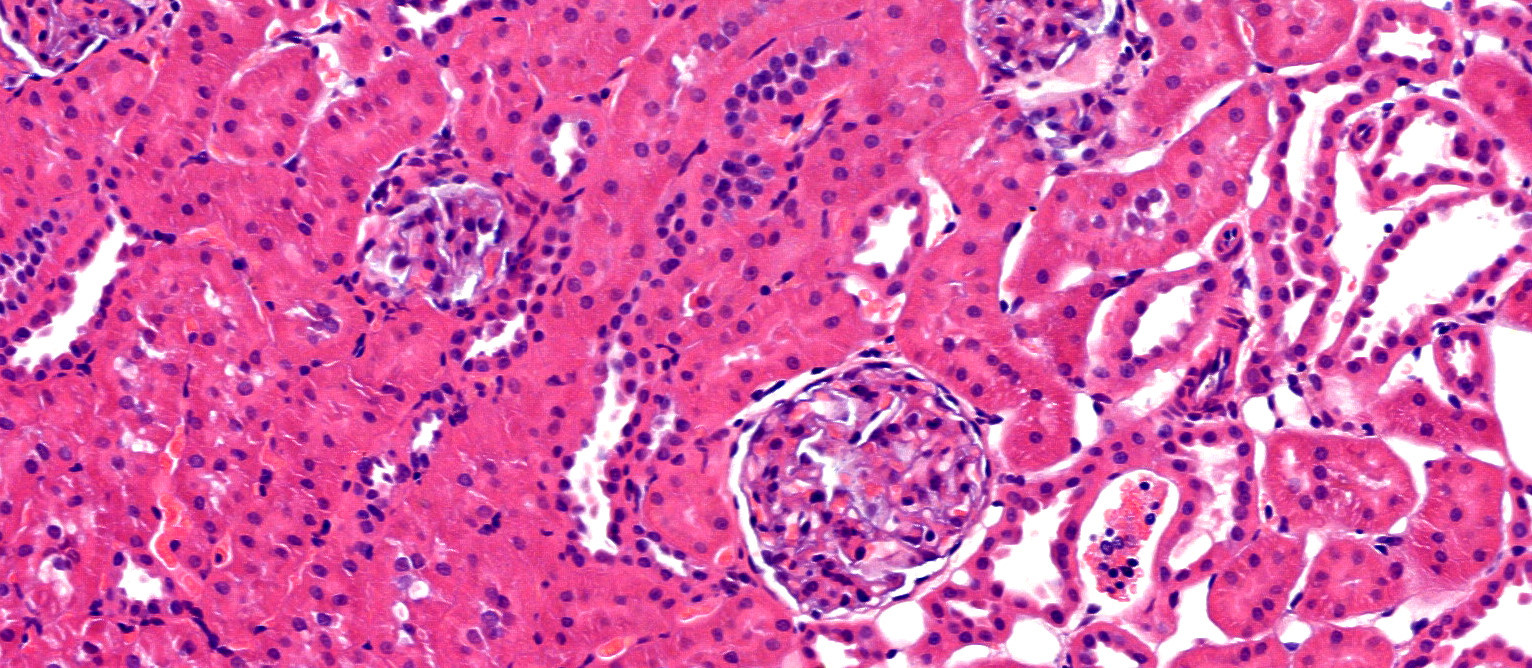

Supplement: Supplementary file 6 [file DataSheet4.ZIP › Fid 1D-HE-sham-6(2)/6-6.jpeg]

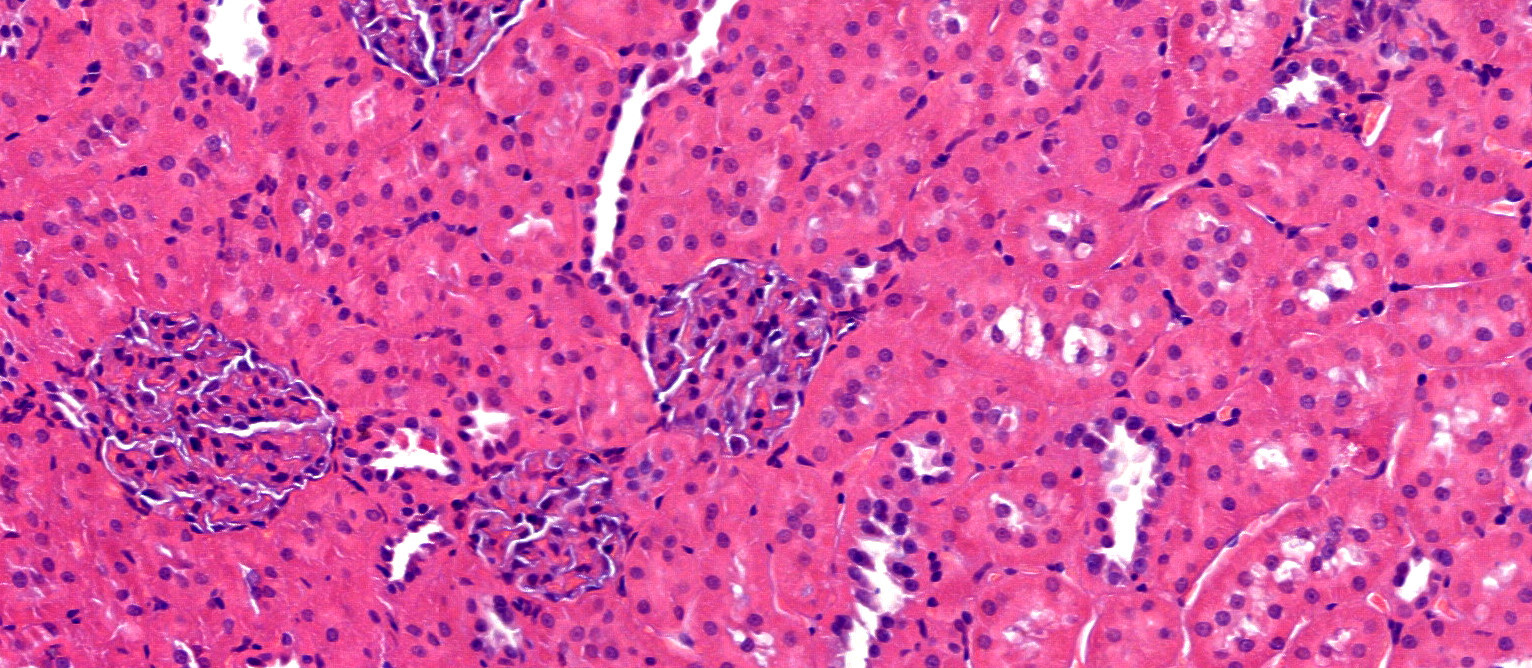

Supplement: Supplementary file 6 [file DataSheet4.ZIP › Fid 1D-HE-sham-6(2)/6-7.jpeg]

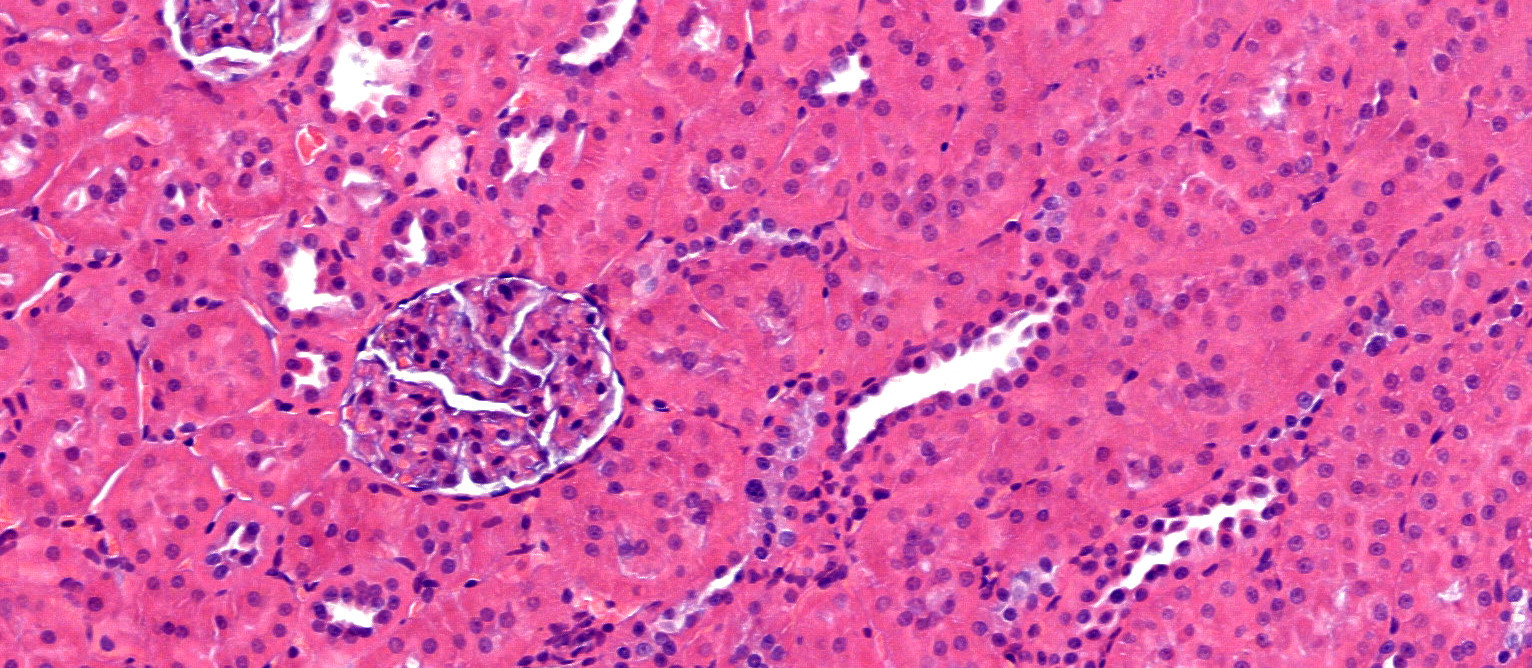

Supplement: Supplementary file 6 [file DataSheet4.ZIP › Fid 1D-HE-sham-6(2)/6-8.jpeg]

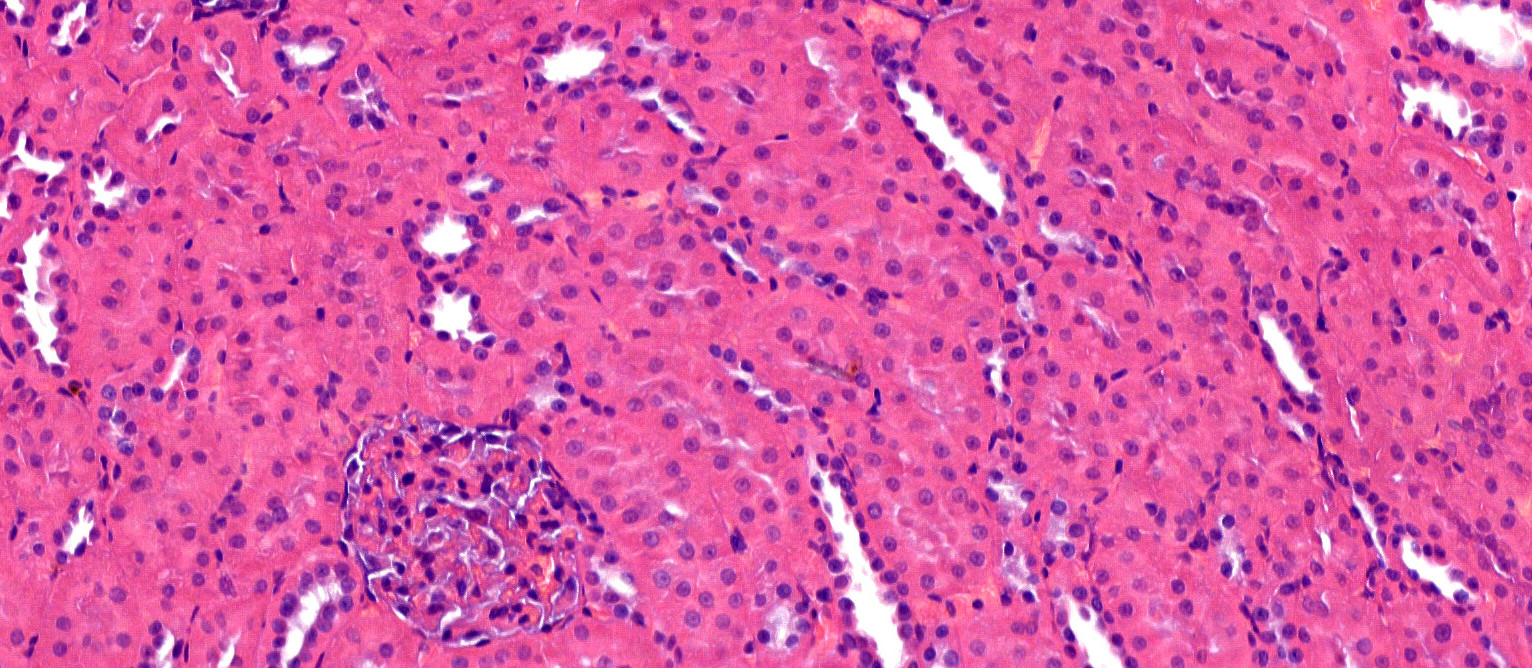

Supplement: Supplementary file 6 [file DataSheet4.ZIP › Fid 1D-HE-sham-6(2)/6-9.jpeg]

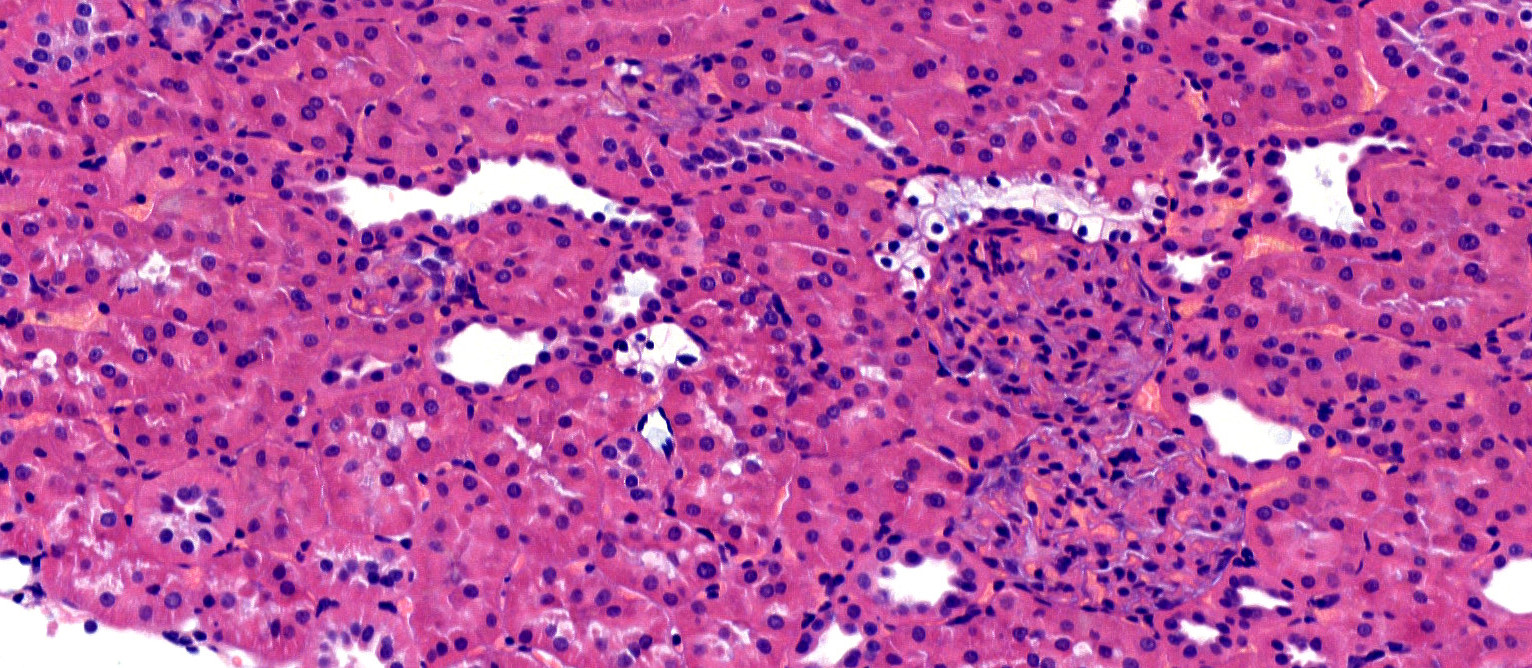

Supplement: Supplementary file 6 [file DataSheet4.ZIP › Fig 1D-HE-DKD-14/14-1.jpeg]

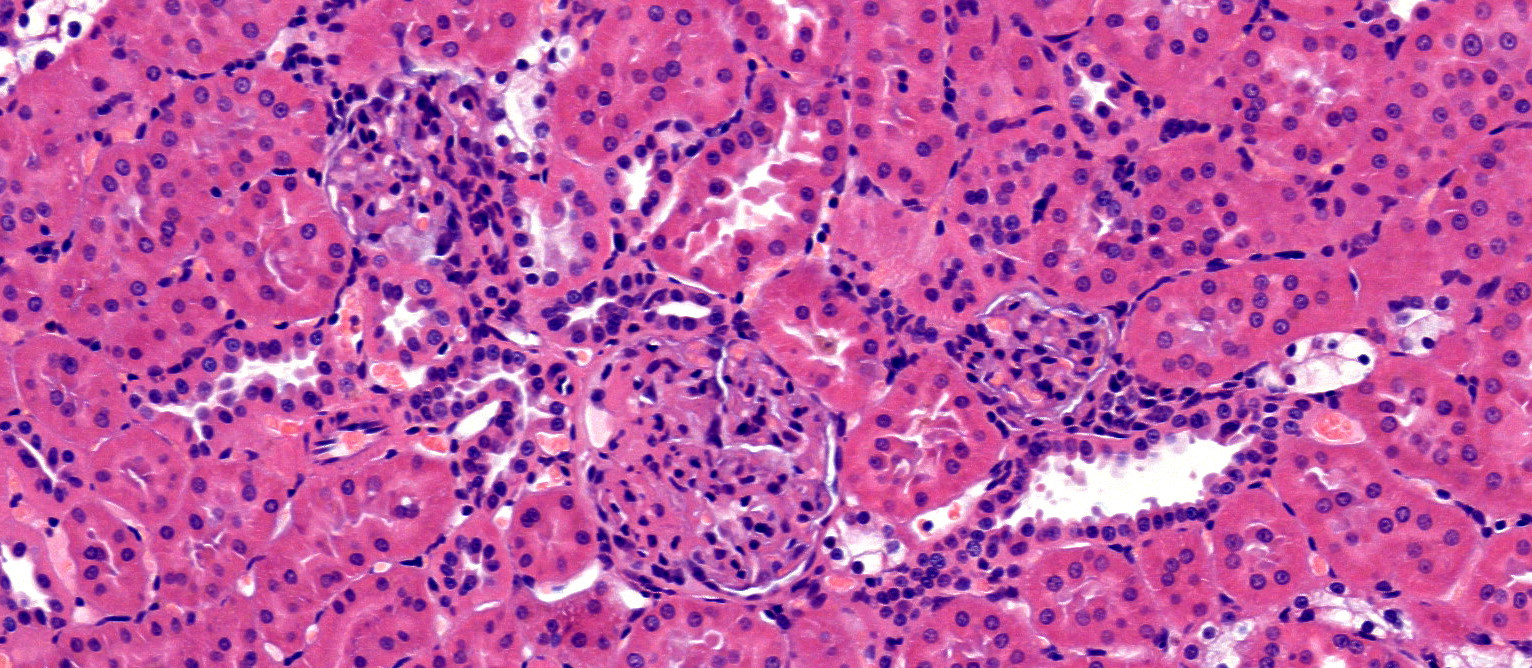

Supplement: Supplementary file 6 [file DataSheet4.ZIP › Fig 1D-HE-DKD-14/14-10.jpeg]

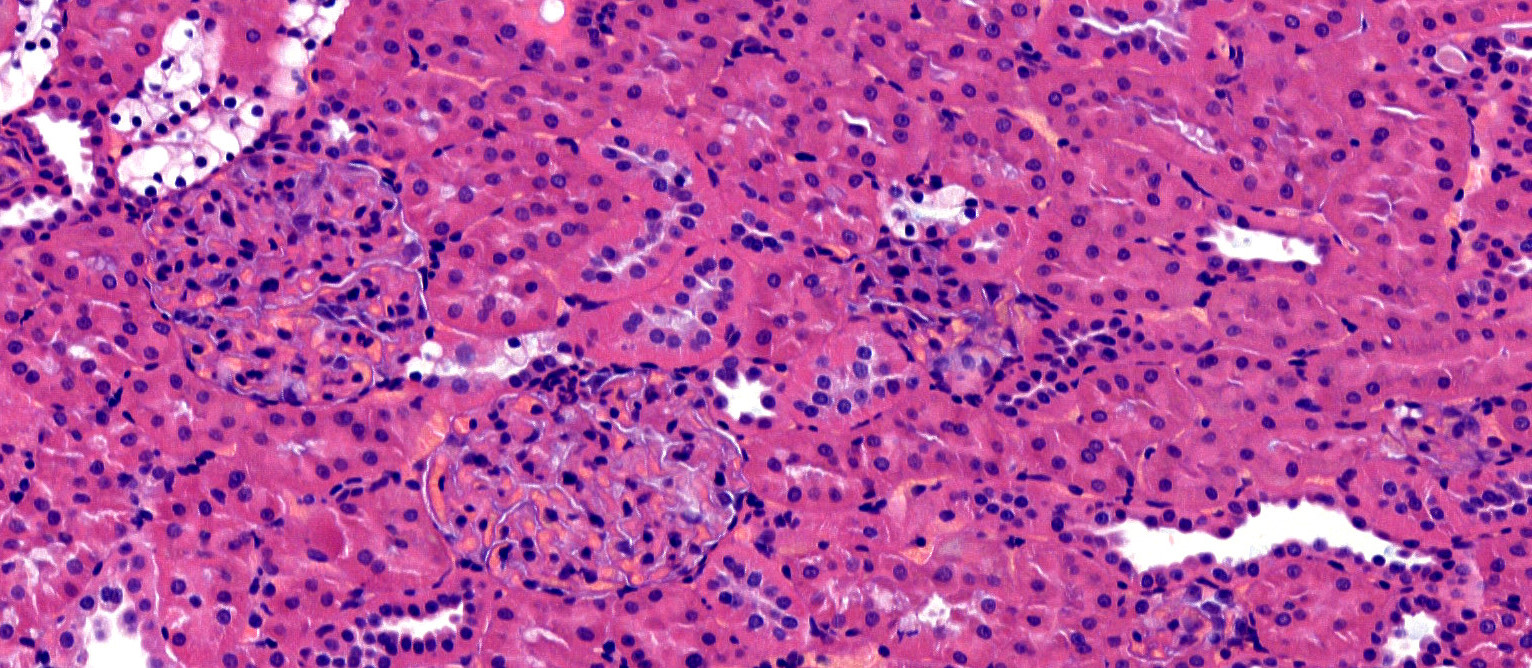

Supplement: Supplementary file 6 [file DataSheet4.ZIP › Fig 1D-HE-DKD-14/14-2.jpeg]

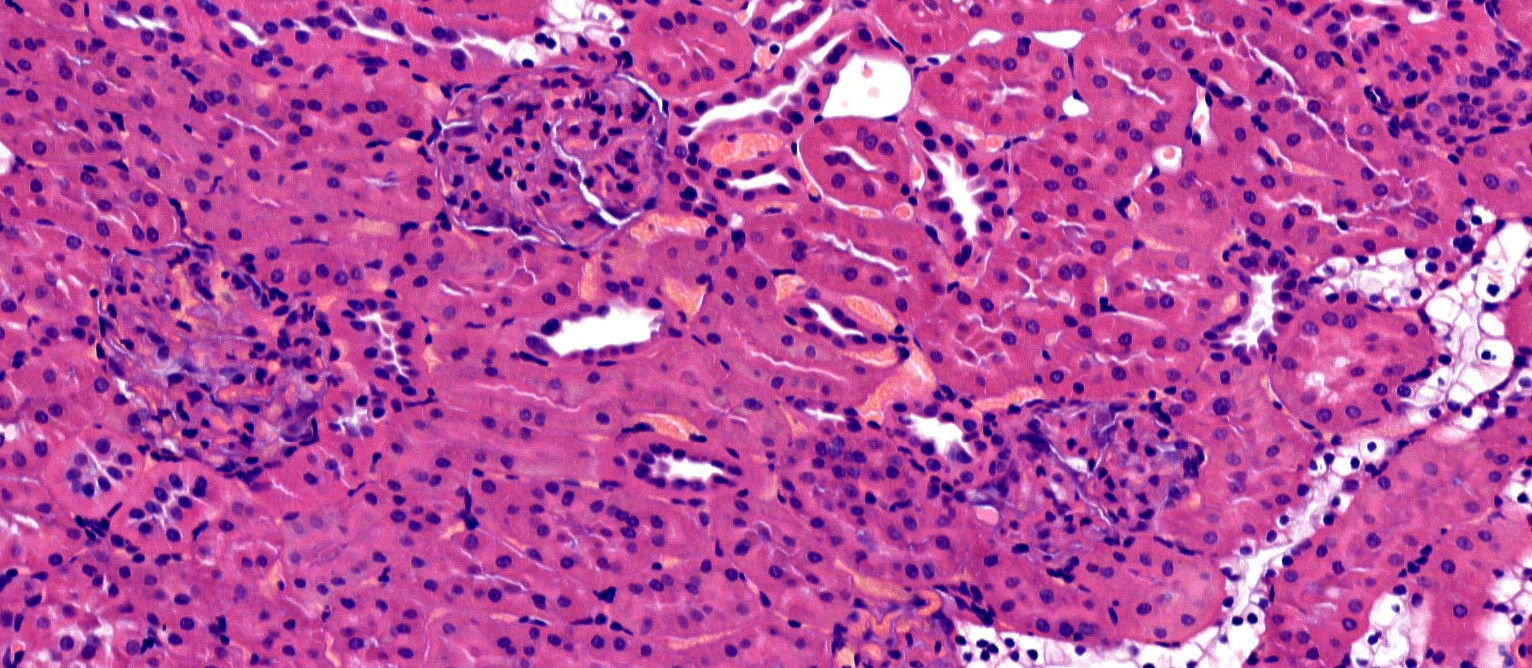

Supplement: Supplementary file 6 [file DataSheet4.ZIP › Fig 1D-HE-DKD-14/14-3.jpeg]

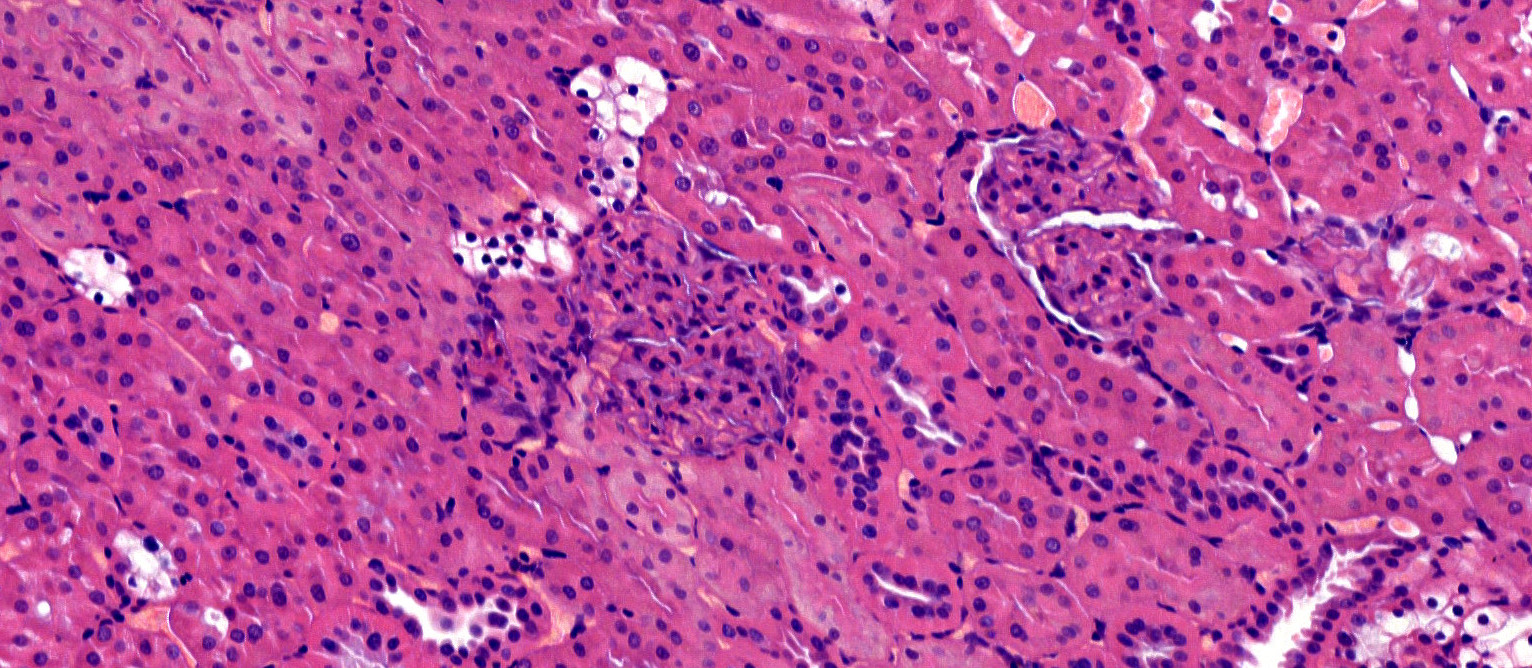

Supplement: Supplementary file 6 [file DataSheet4.ZIP › Fig 1D-HE-DKD-14/14-4.jpeg]

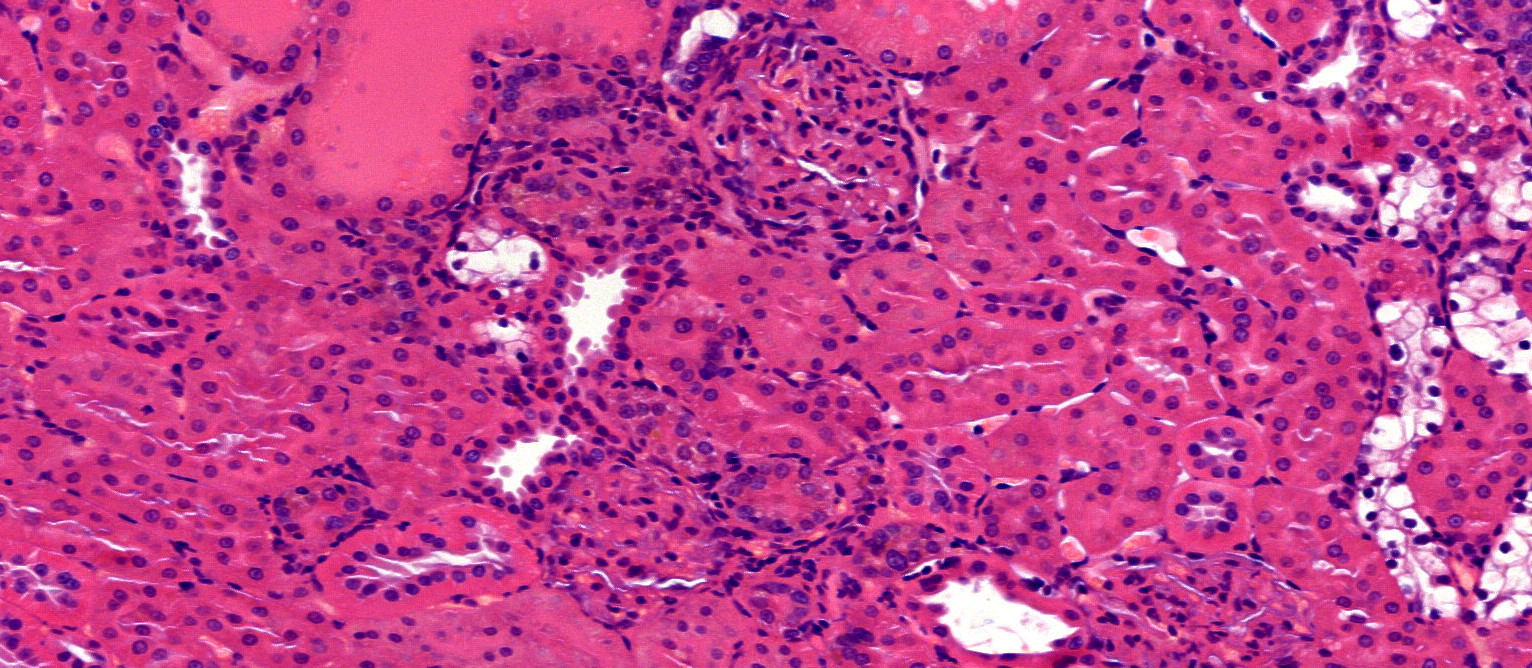

Supplement: Supplementary file 6 [file DataSheet4.ZIP › Fig 1D-HE-DKD-14/14-5.jpeg]

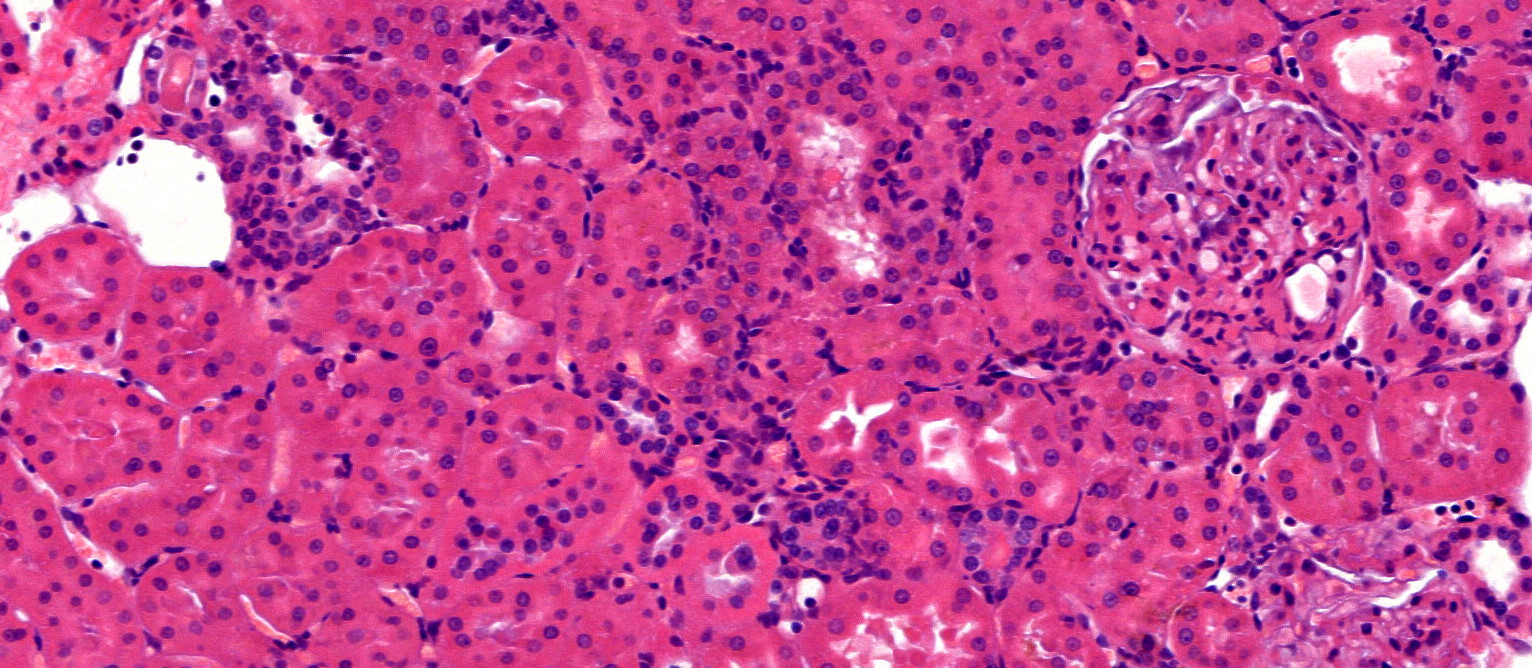

Supplement: Supplementary file 6 [file DataSheet4.ZIP › Fig 1D-HE-DKD-14/14-6.jpeg]

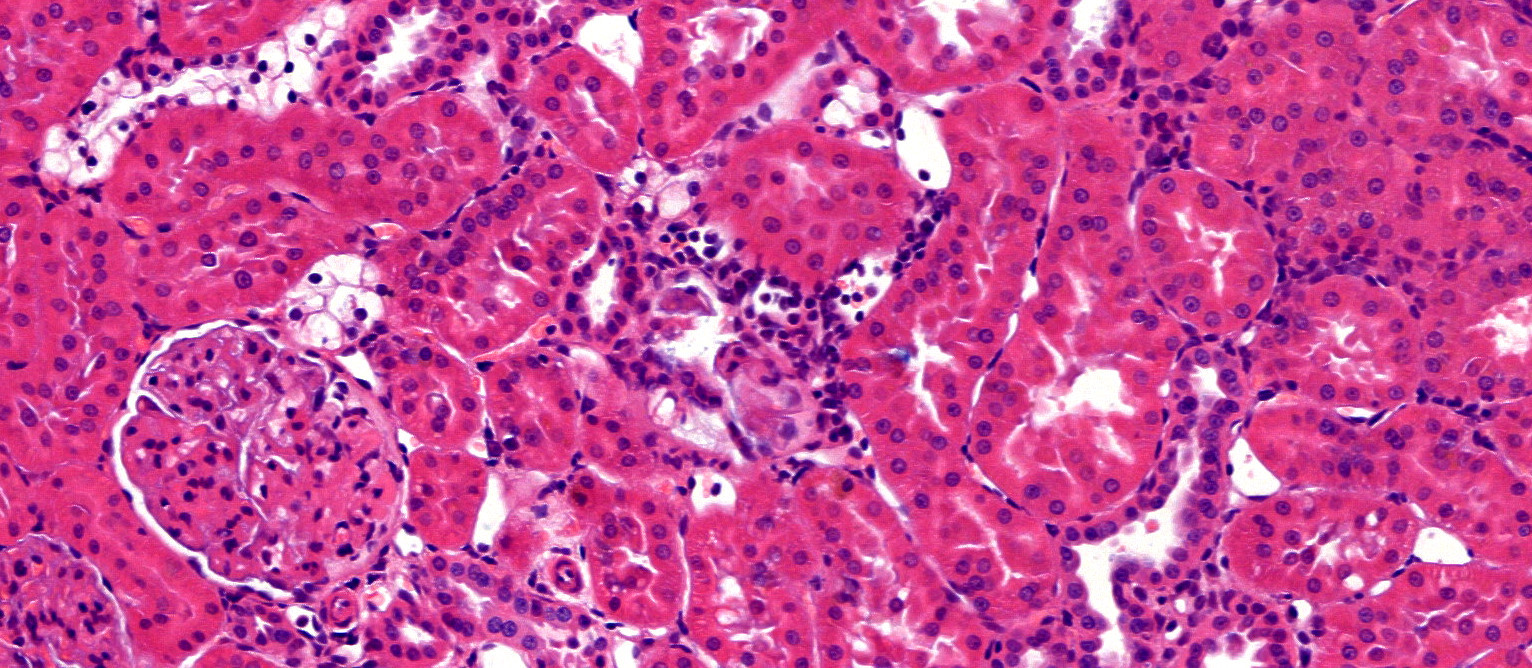

Supplement: Supplementary file 6 [file DataSheet4.ZIP › Fig 1D-HE-DKD-14/14-7.jpeg]

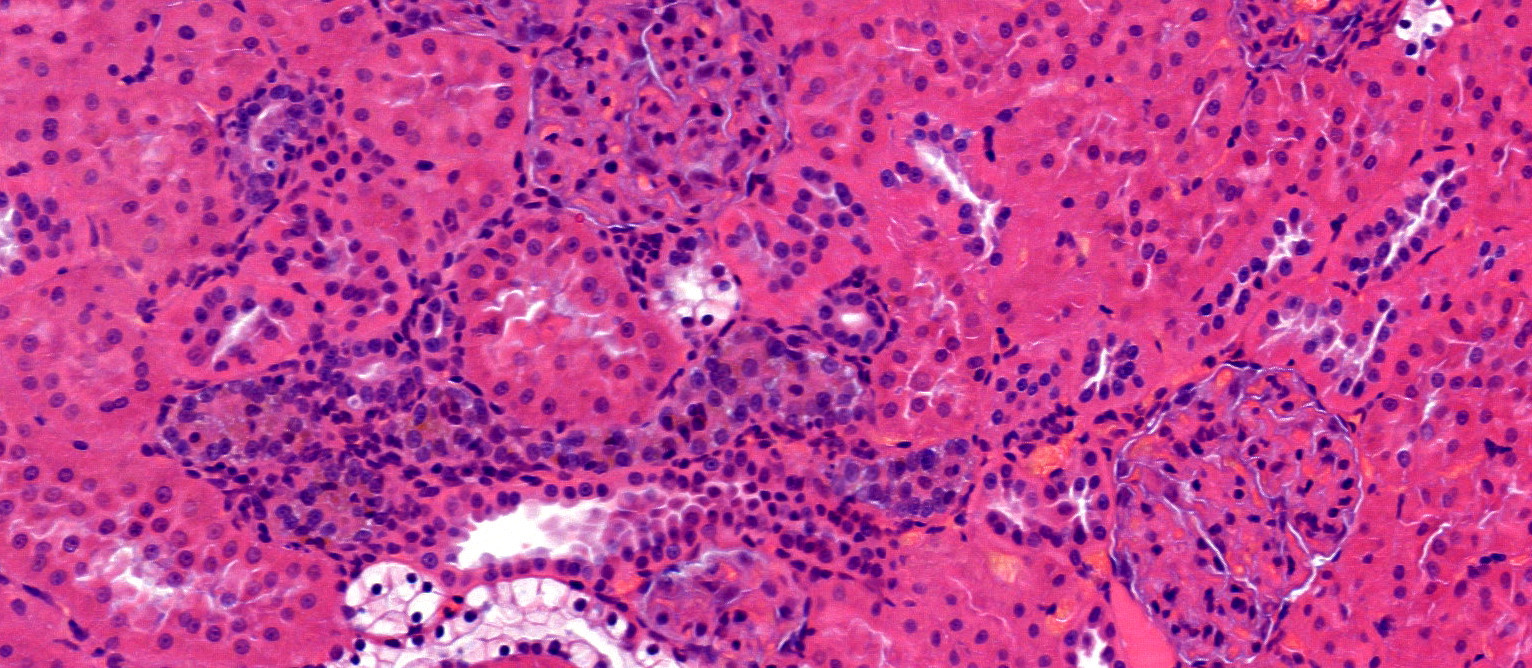

Supplement: Supplementary file 6 [file DataSheet4.ZIP › Fig 1D-HE-DKD-14/14-8.jpeg]

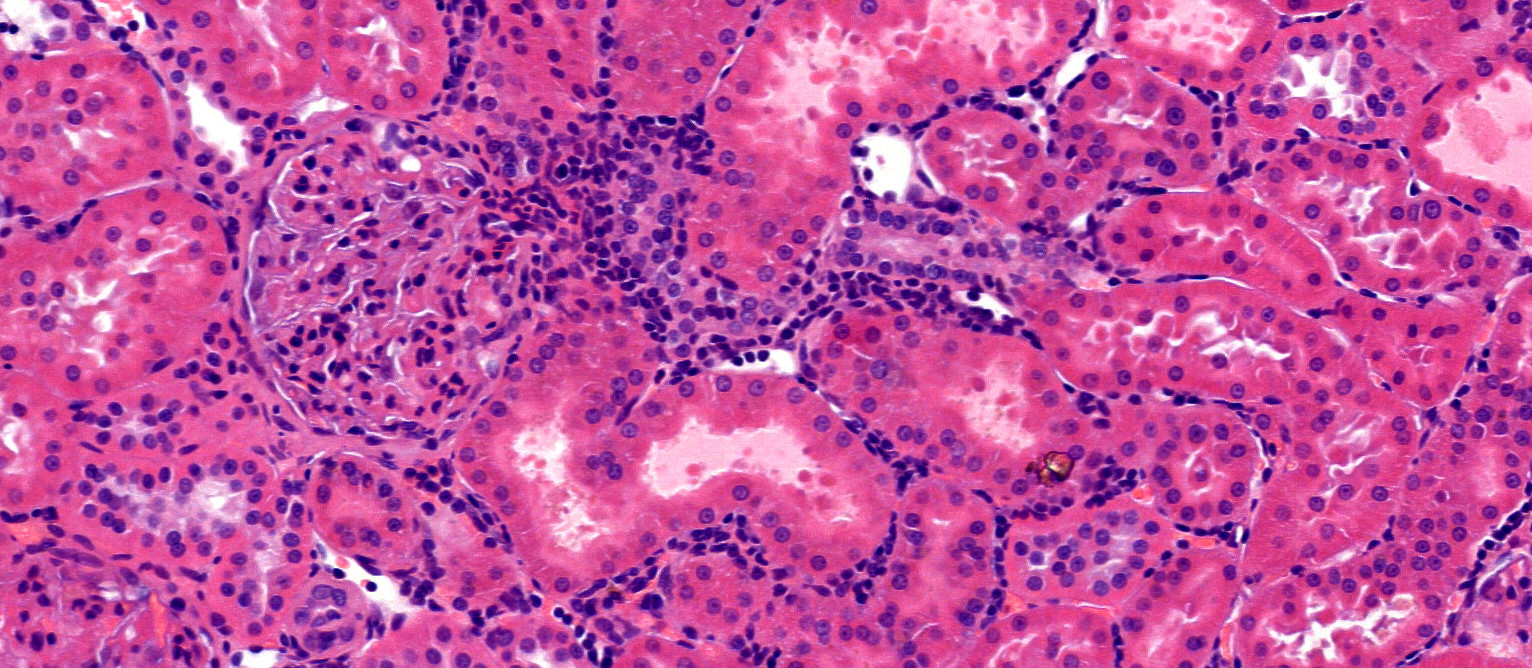

Supplement: Supplementary file 6 [file DataSheet4.ZIP › Fig 1D-HE-DKD-14/14-9.jpeg]

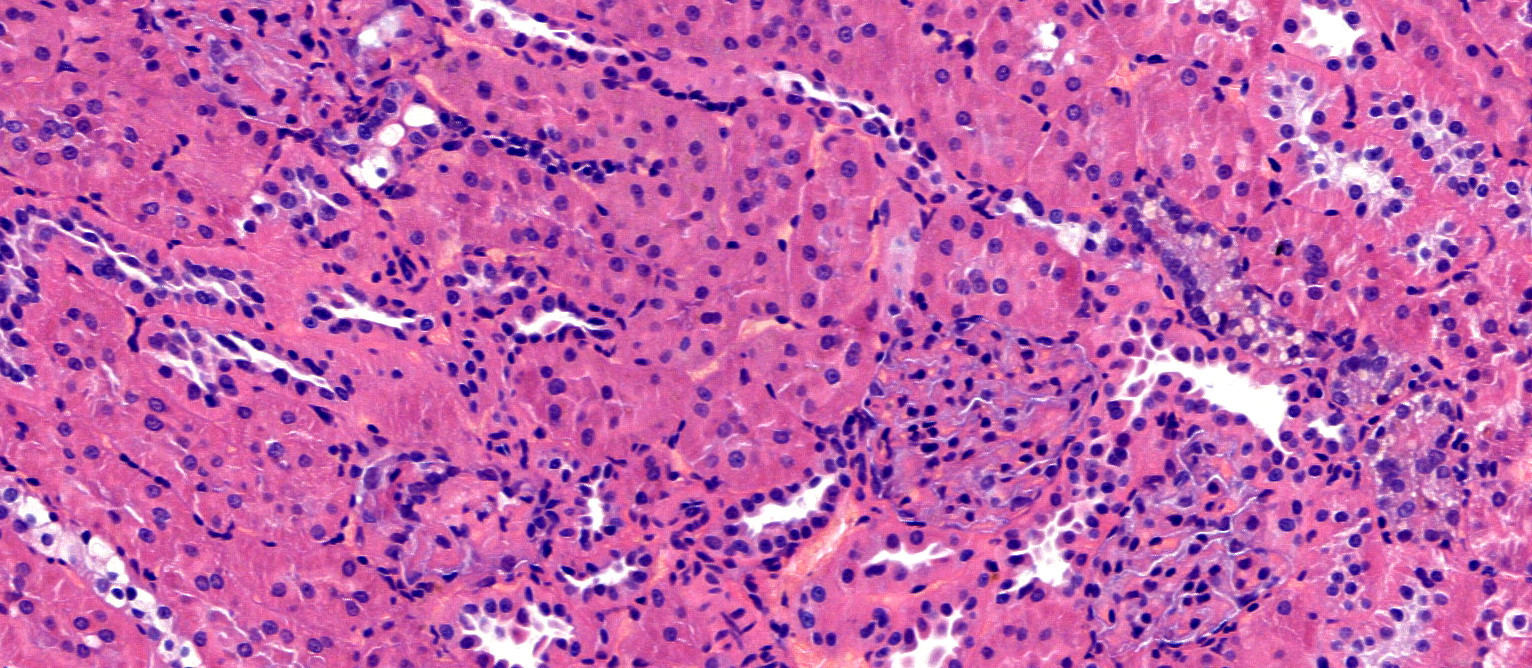

Supplement: Supplementary file 6 [file DataSheet4.ZIP › Fig 1D-HE-DKD-15(1)/15-1.jpeg]

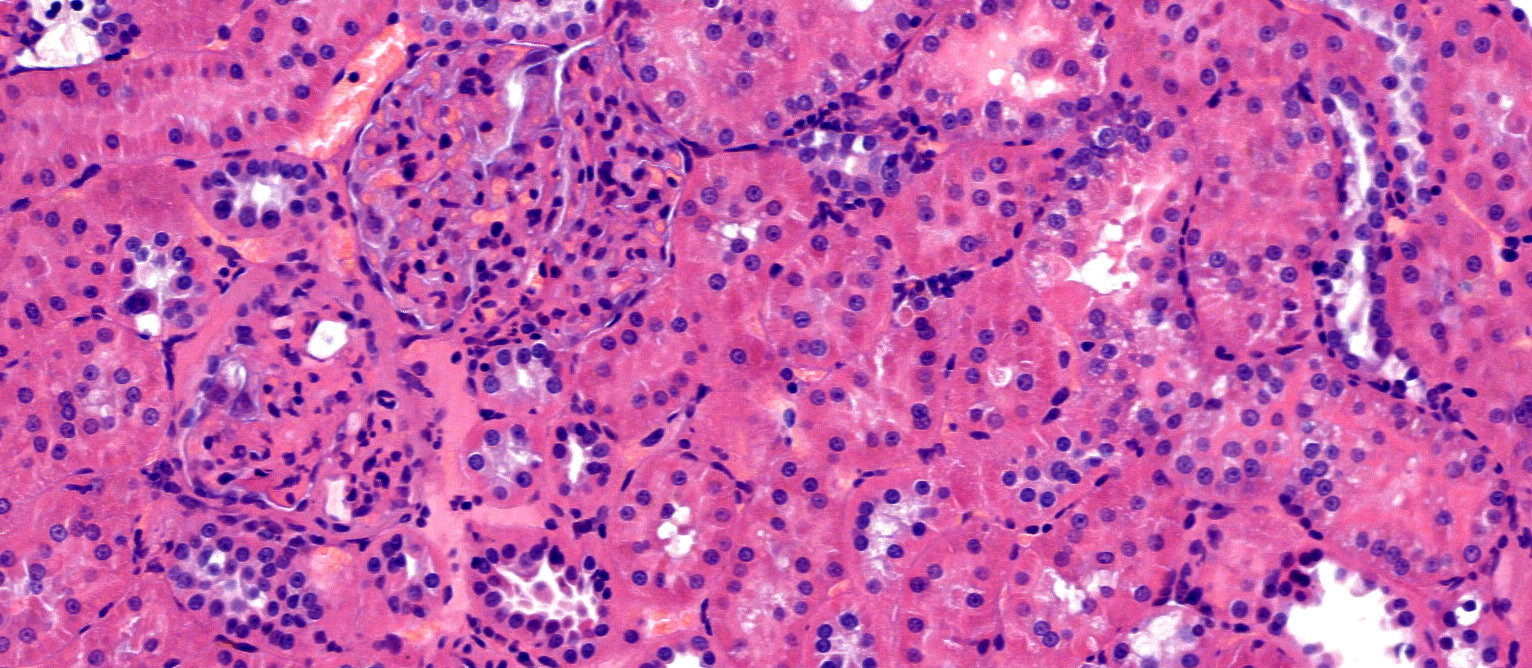

Supplement: Supplementary file 6 [file DataSheet4.ZIP › Fig 1D-HE-DKD-15(1)/15-2.jpeg]

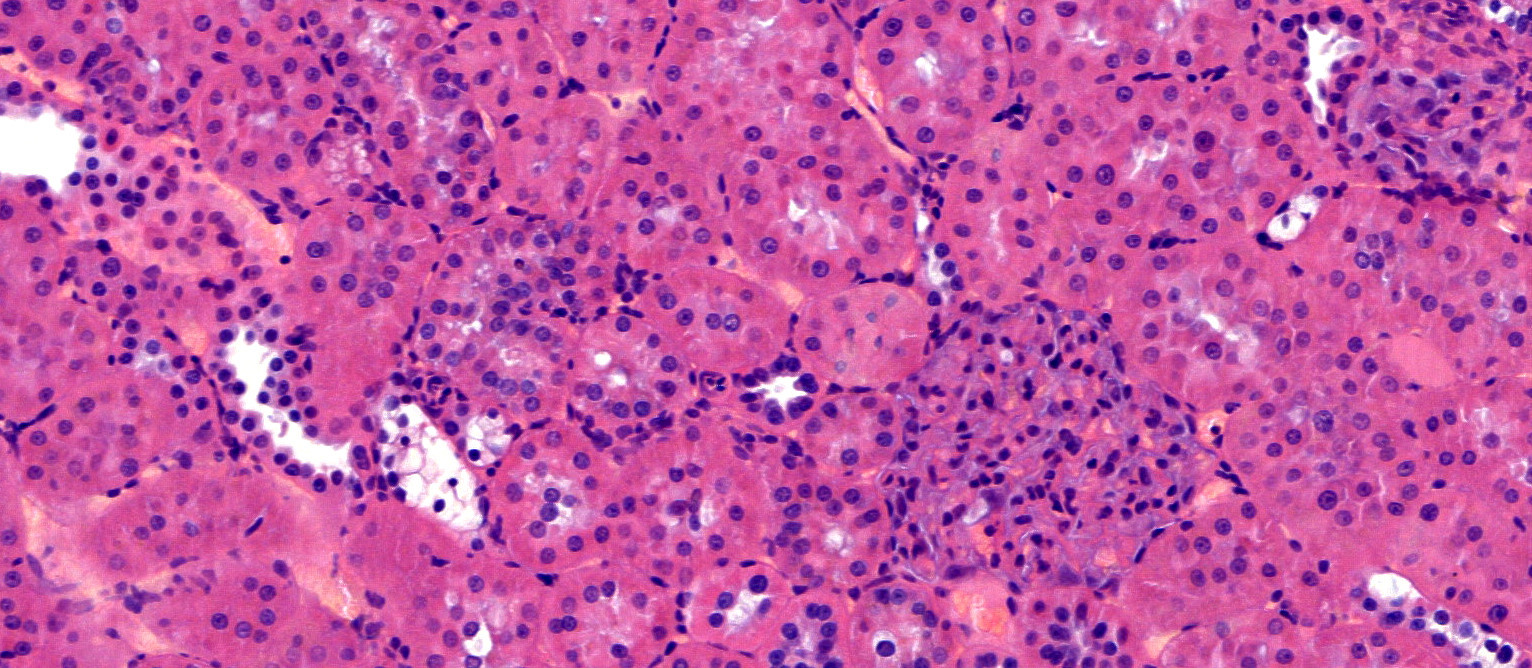

Supplement: Supplementary file 6 [file DataSheet4.ZIP › Fig 1D-HE-DKD-15(1)/15-3.jpeg]

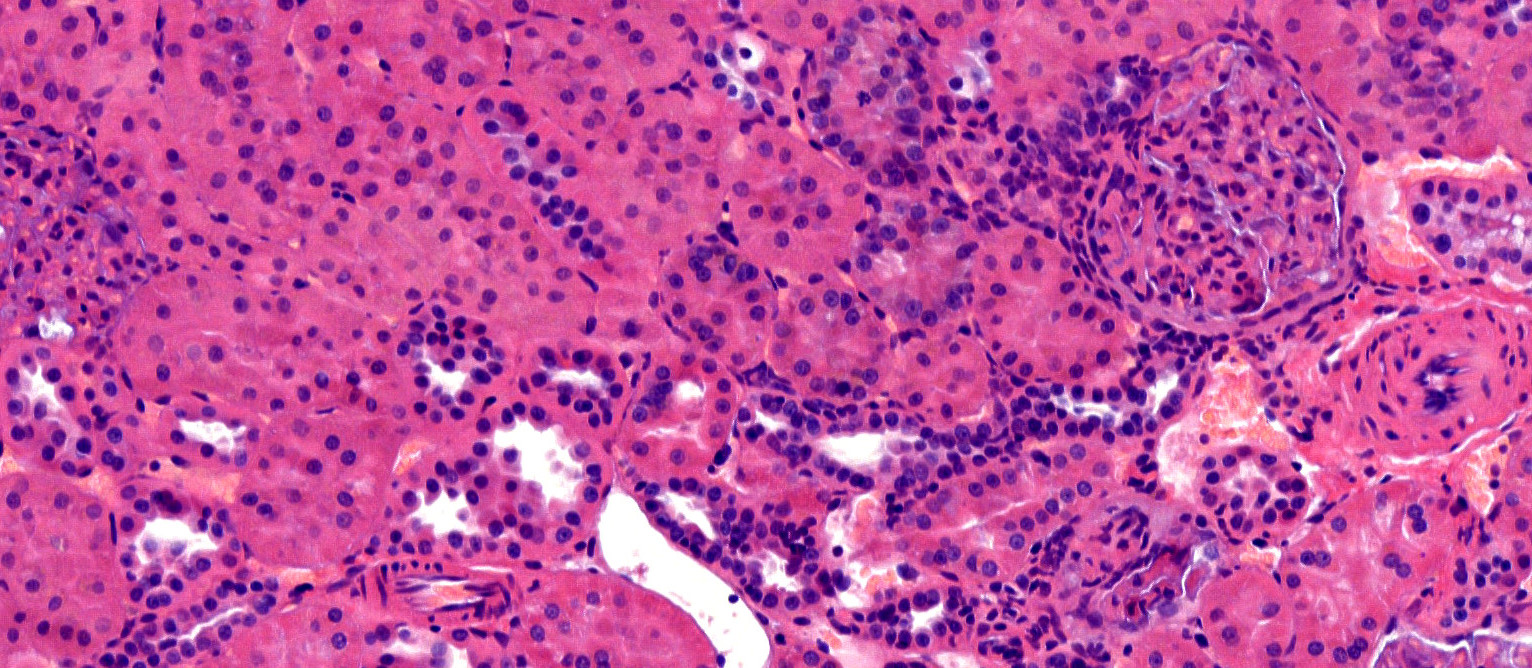

Supplement: Supplementary file 6 [file DataSheet4.ZIP › Fig 1D-HE-DKD-15(1)/15-4.jpeg]

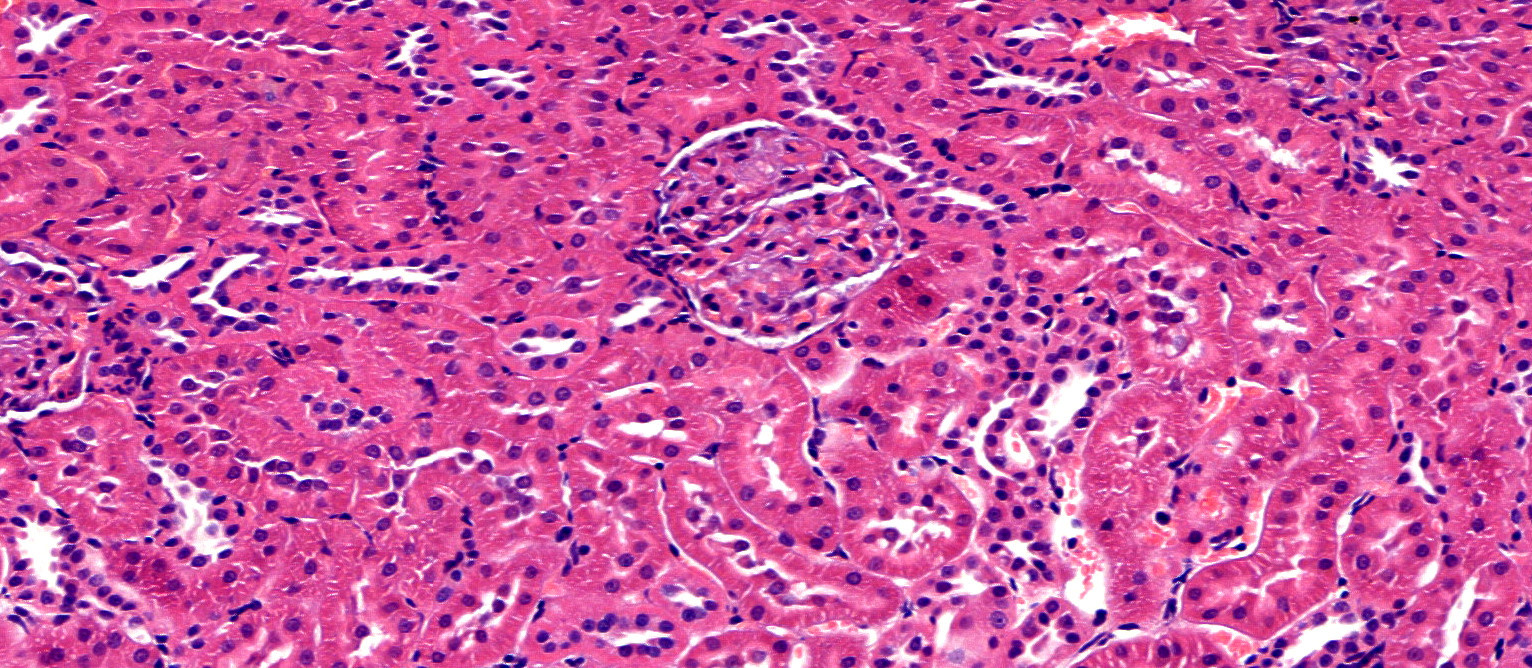

Supplement: Supplementary file 6 [file DataSheet4.ZIP › Fig 1D-HE-sham-10/10-1.jpeg]

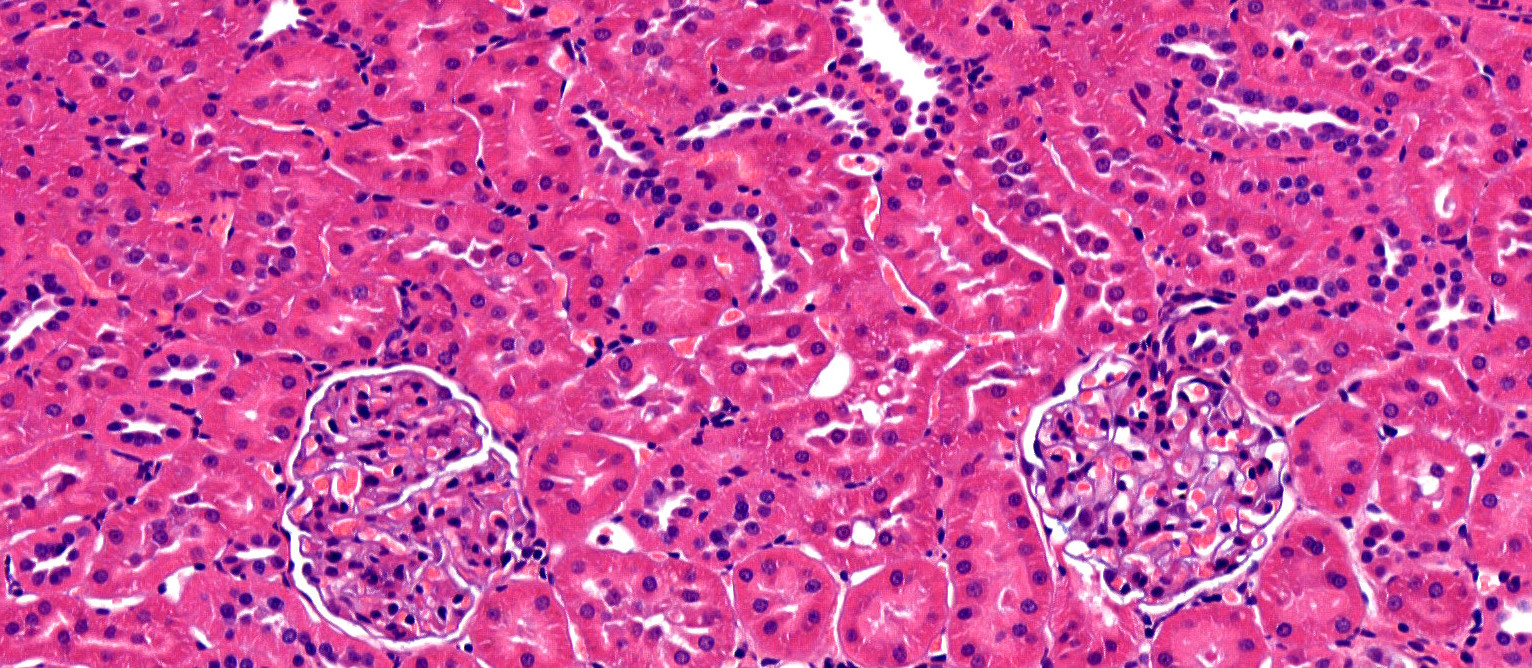

Supplement: Supplementary file 6 [file DataSheet4.ZIP › Fig 1D-HE-sham-10/10-10.jpeg]

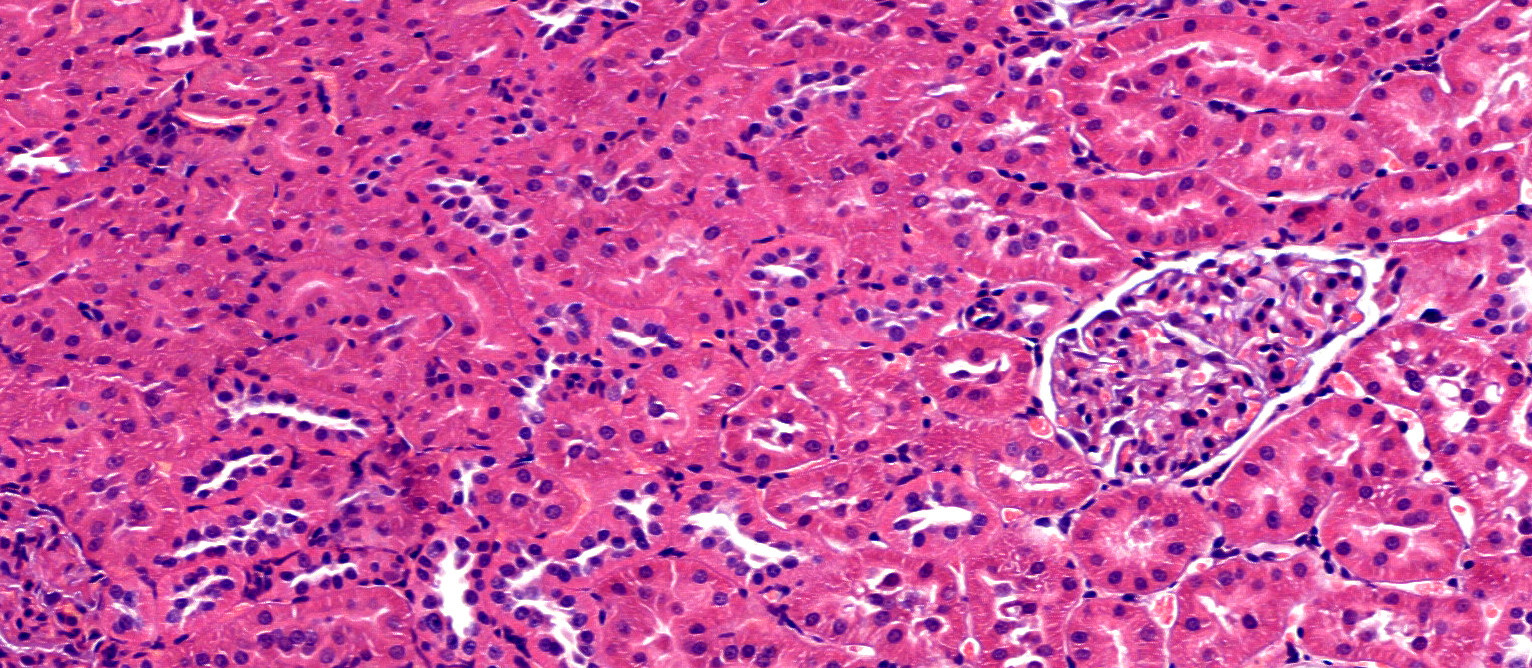

Supplement: Supplementary file 6 [file DataSheet4.ZIP › Fig 1D-HE-sham-10/10-2.jpeg]

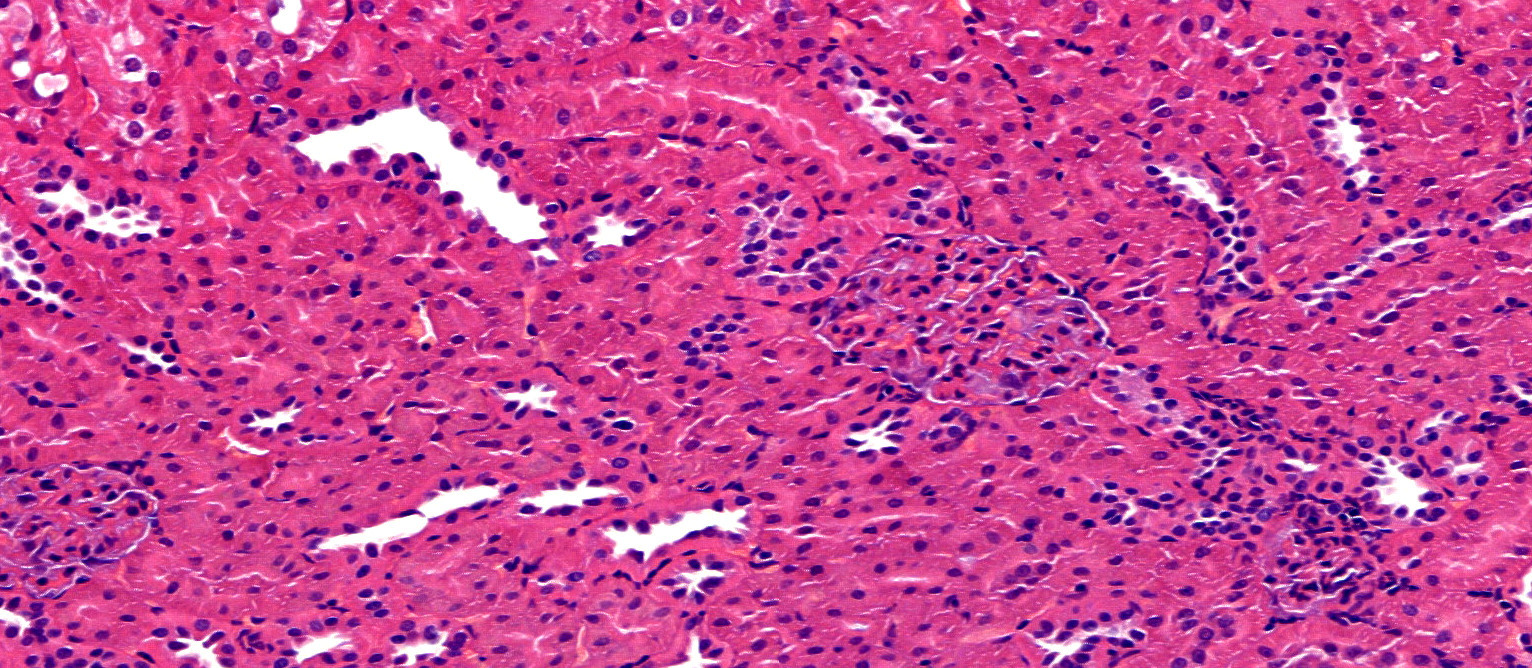

Supplement: Supplementary file 6 [file DataSheet4.ZIP › Fig 1D-HE-sham-10/10-3.jpeg]

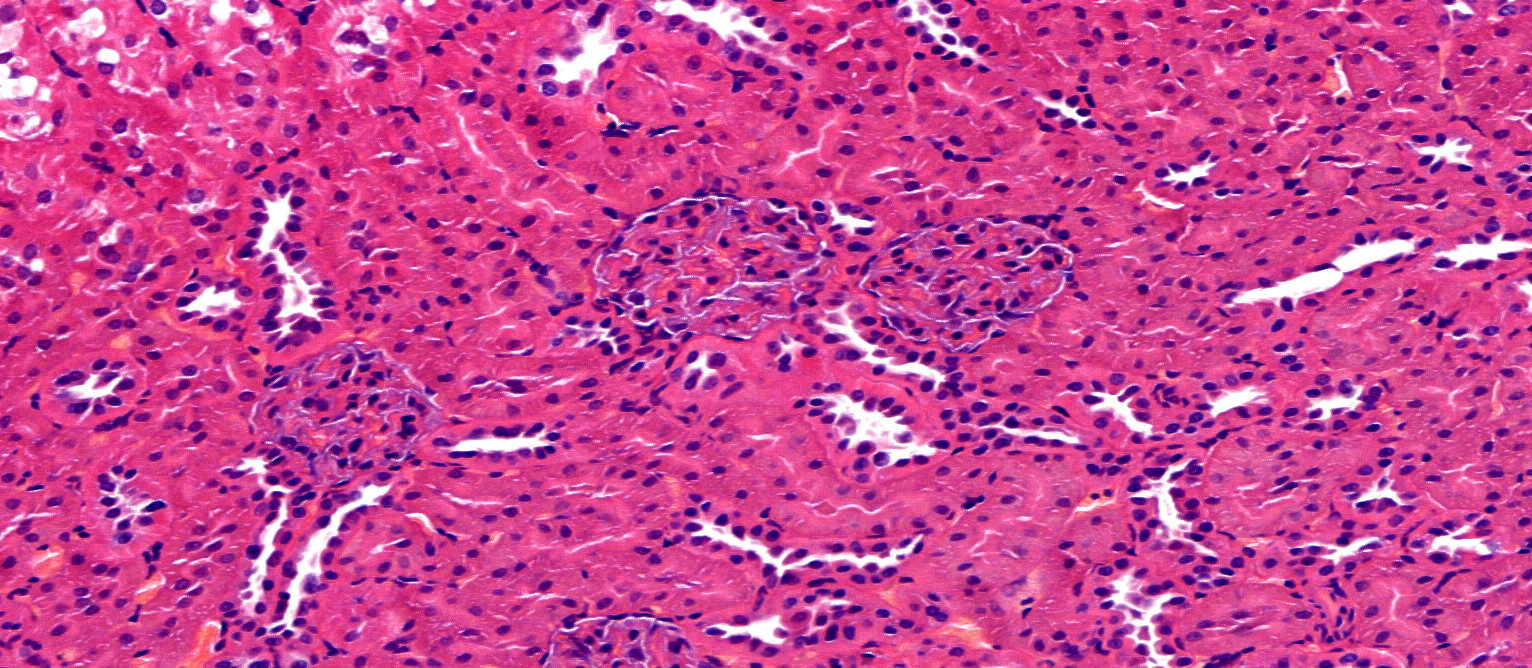

Supplement: Supplementary file 6 [file DataSheet4.ZIP › Fig 1D-HE-sham-10/10-4.jpeg]

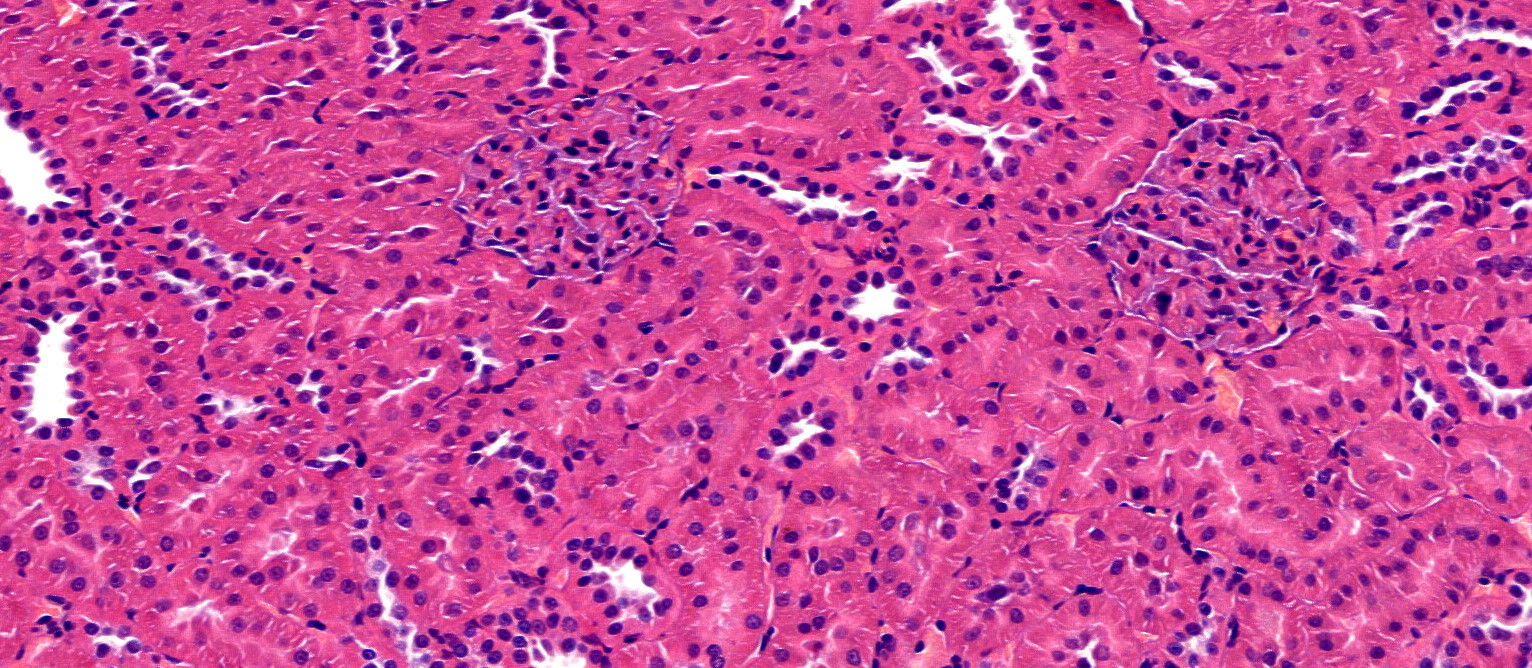

Supplement: Supplementary file 6 [file DataSheet4.ZIP › Fig 1D-HE-sham-10/10-5.jpeg]

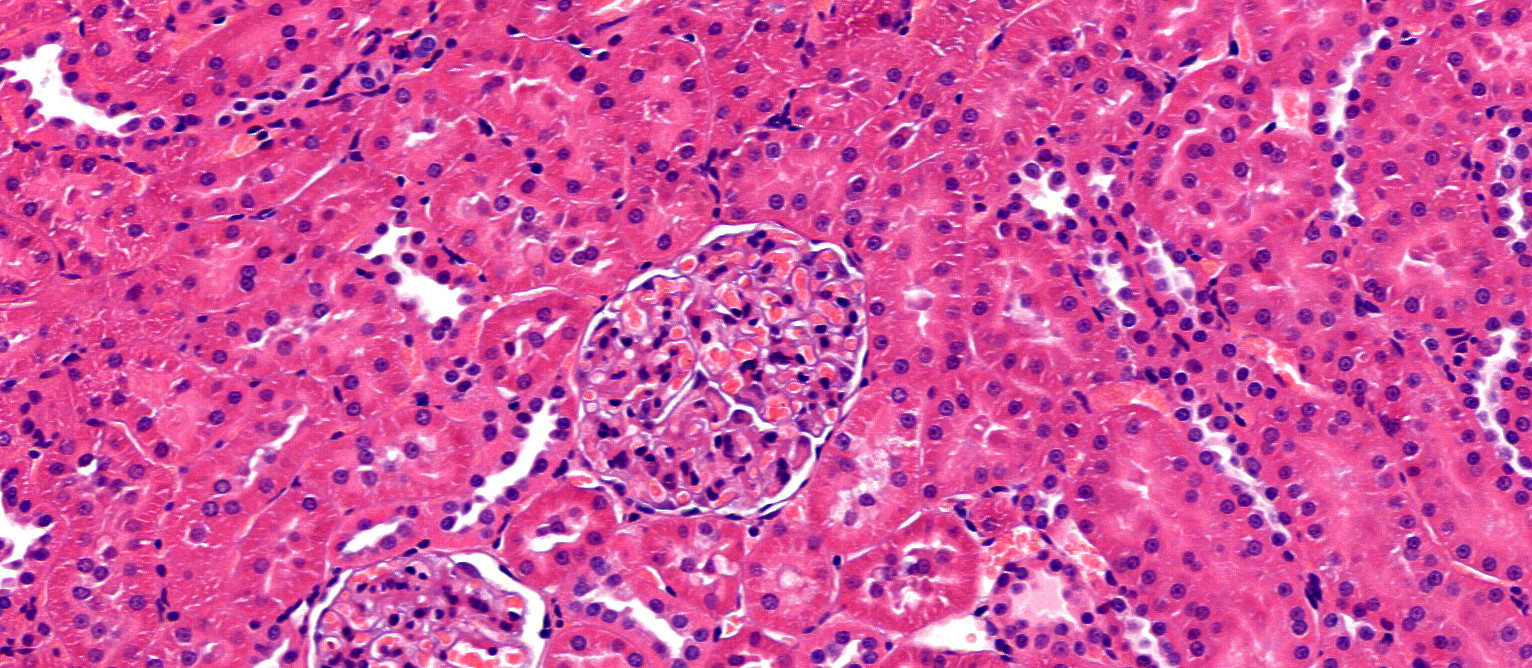

Supplement: Supplementary file 6 [file DataSheet4.ZIP › Fig 1D-HE-sham-10/10-6.jpeg]

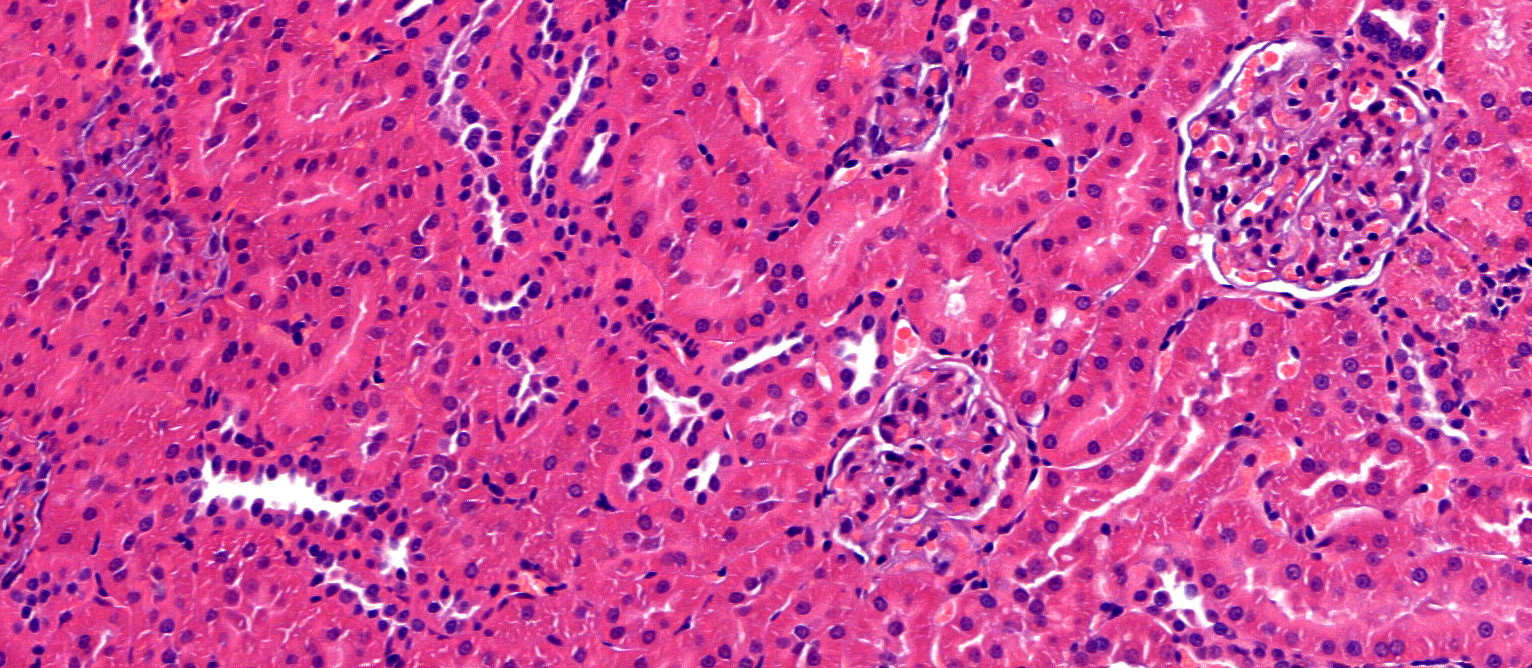

Supplement: Supplementary file 6 [file DataSheet4.ZIP › Fig 1D-HE-sham-10/10-7.jpeg]

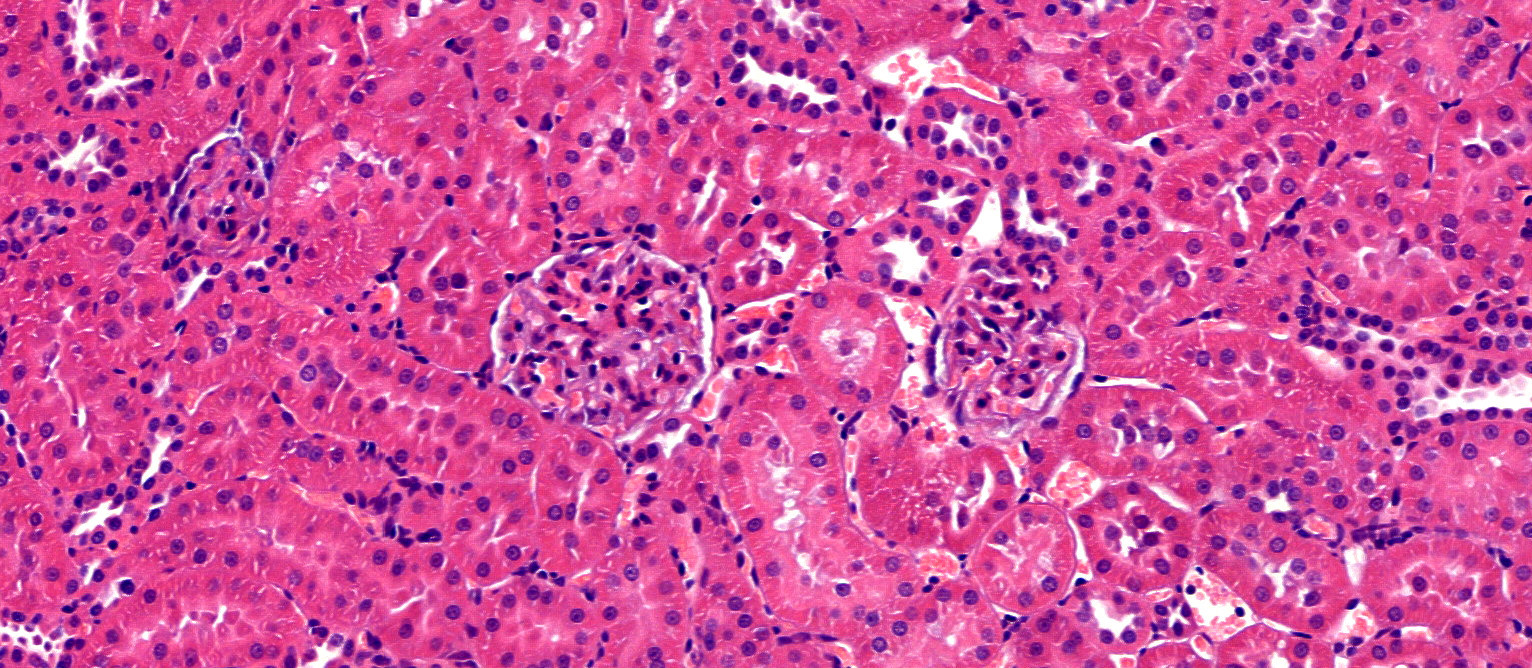

Supplement: Supplementary file 6 [file DataSheet4.ZIP › Fig 1D-HE-sham-10/10-8.jpeg]

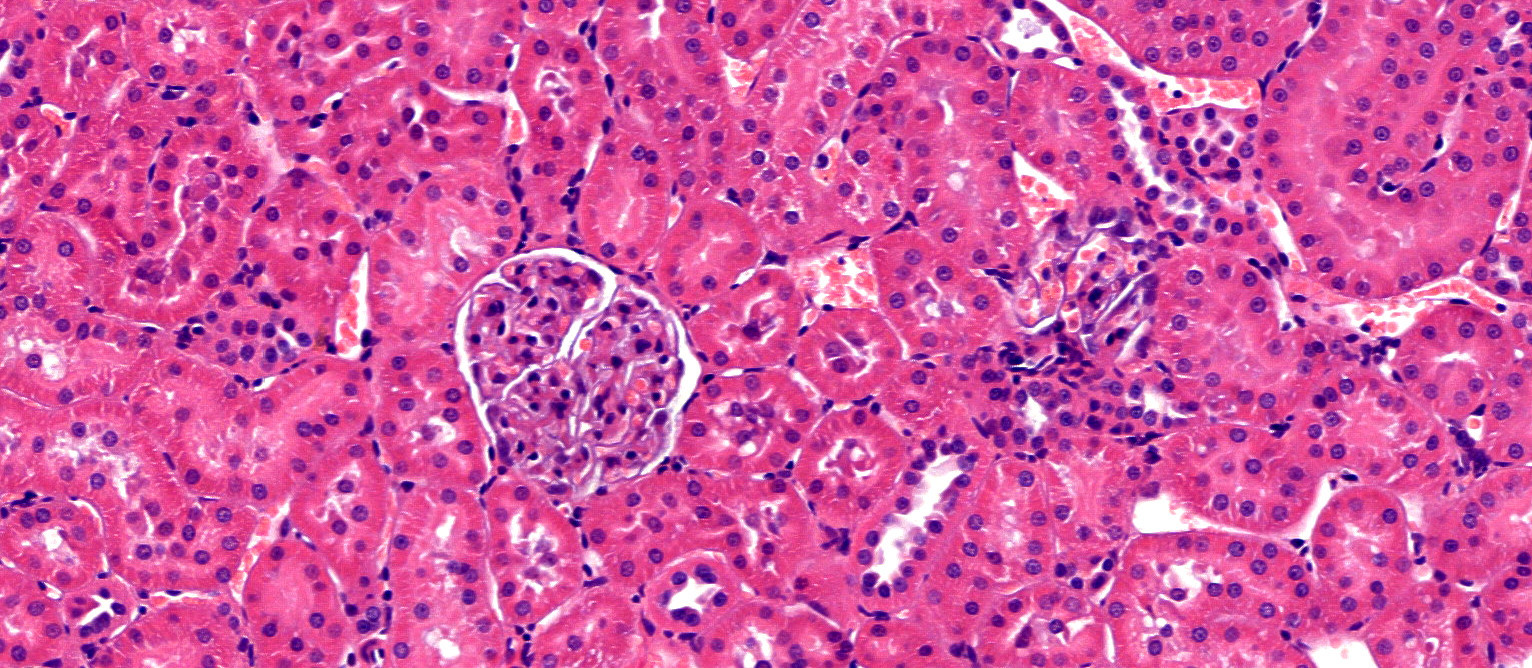

Supplement: Supplementary file 6 [file DataSheet4.ZIP › Fig 1D-HE-sham-10/10-9.jpeg]

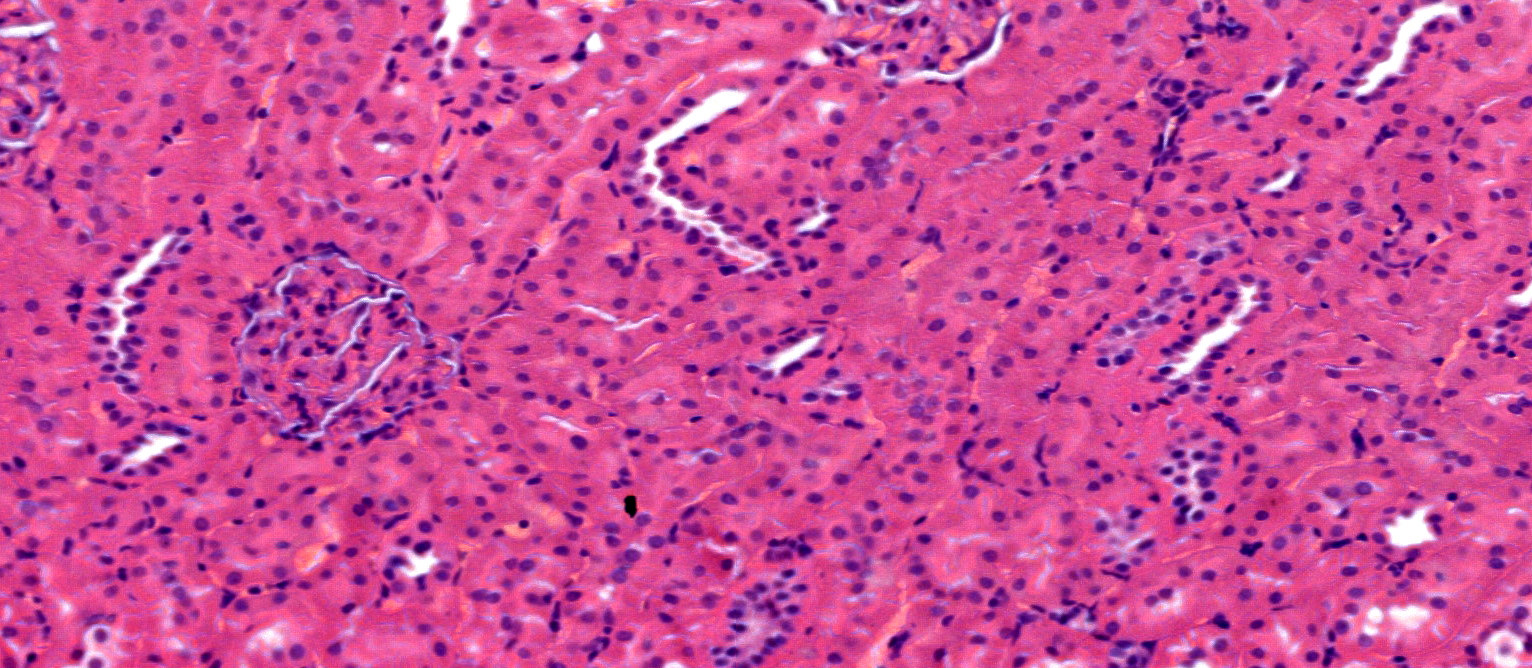

Supplement: Supplementary file 6 [file DataSheet4.ZIP › Fig 1D-HE-sham-7/7-1.jpeg]

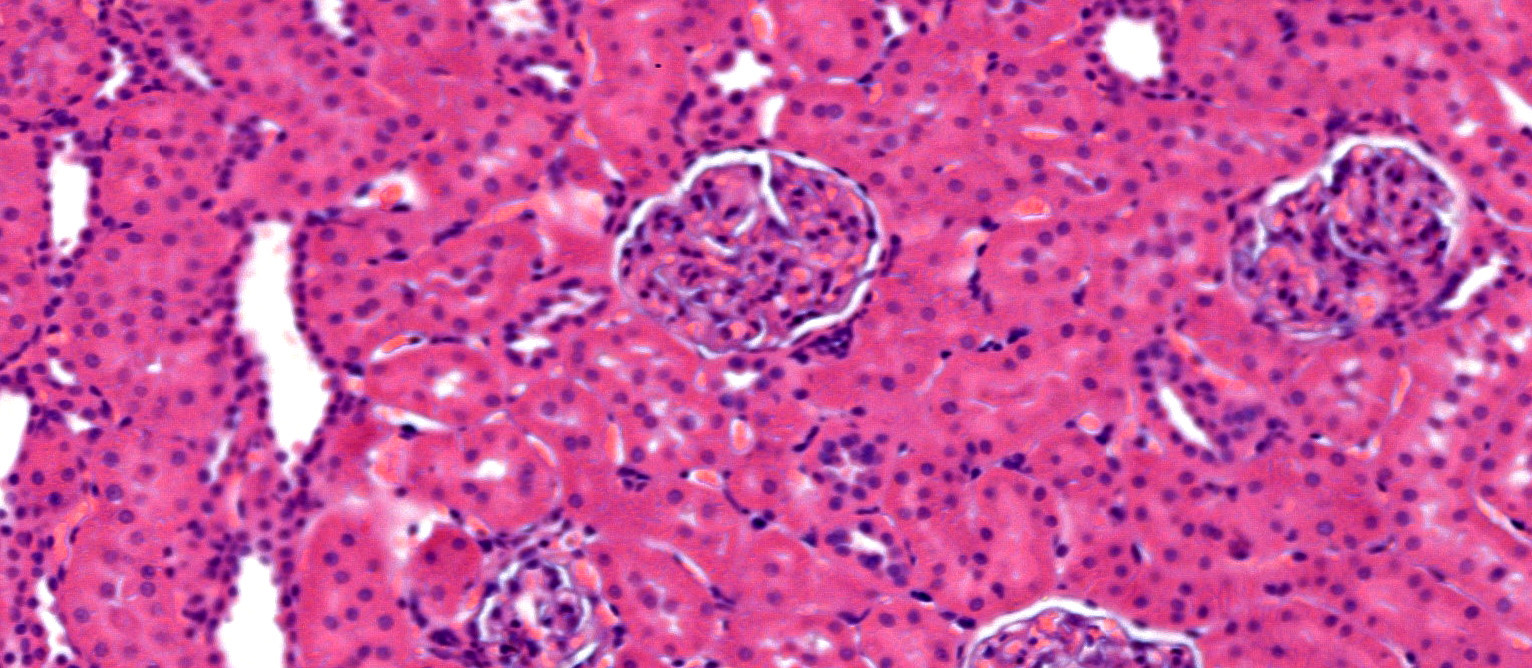

Supplement: Supplementary file 6 [file DataSheet4.ZIP › Fig 1D-HE-sham-7/7-10.jpeg]

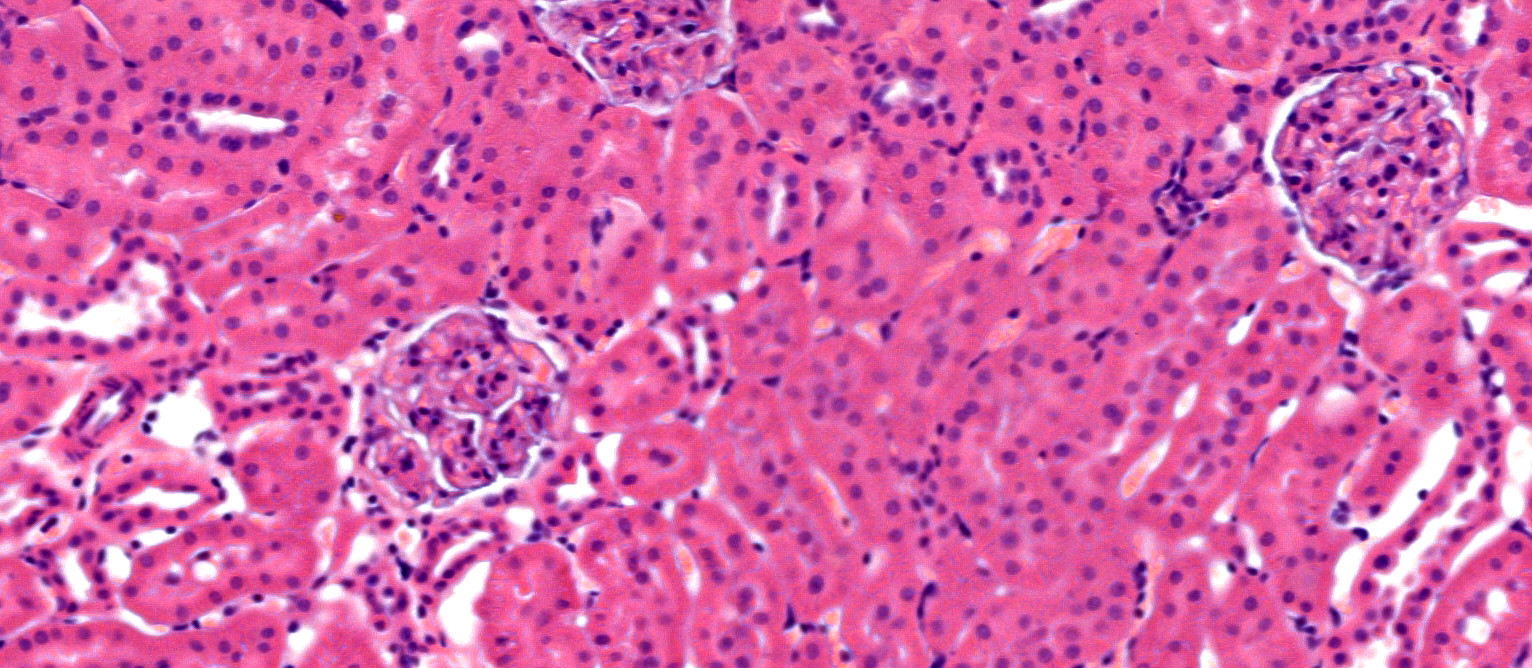

Supplement: Supplementary file 6 [file DataSheet4.ZIP › Fig 1D-HE-sham-7/7-2.jpeg]

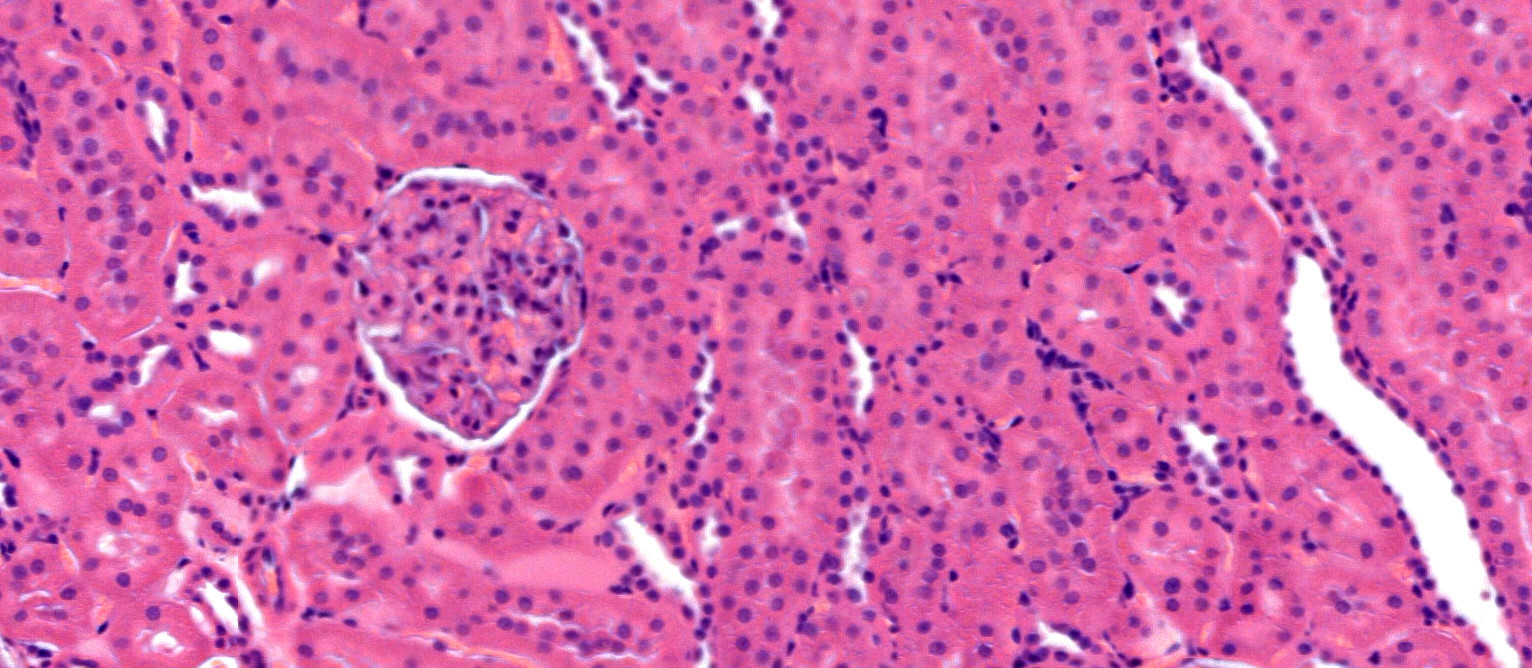

Supplement: Supplementary file 6 [file DataSheet4.ZIP › Fig 1D-HE-sham-7/7-3.jpeg]

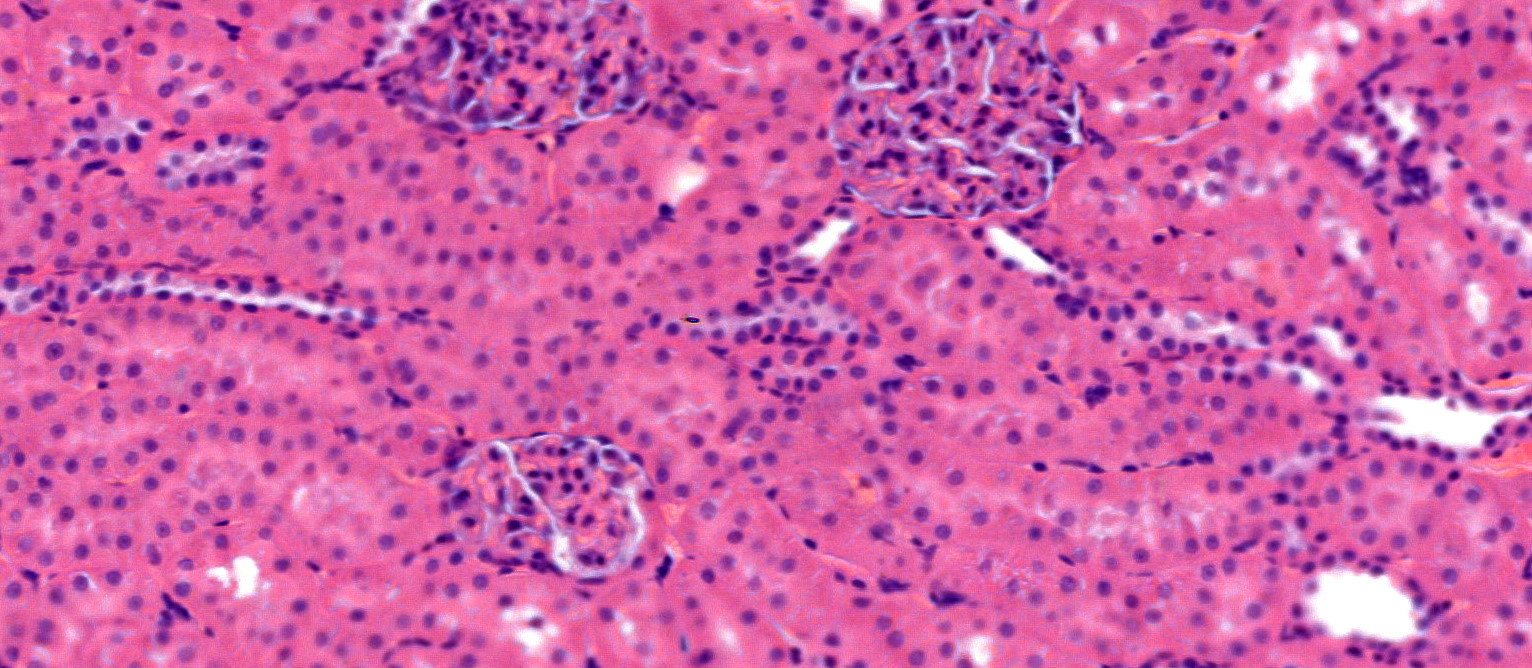

Supplement: Supplementary file 6 [file DataSheet4.ZIP › Fig 1D-HE-sham-7/7-4.jpeg]

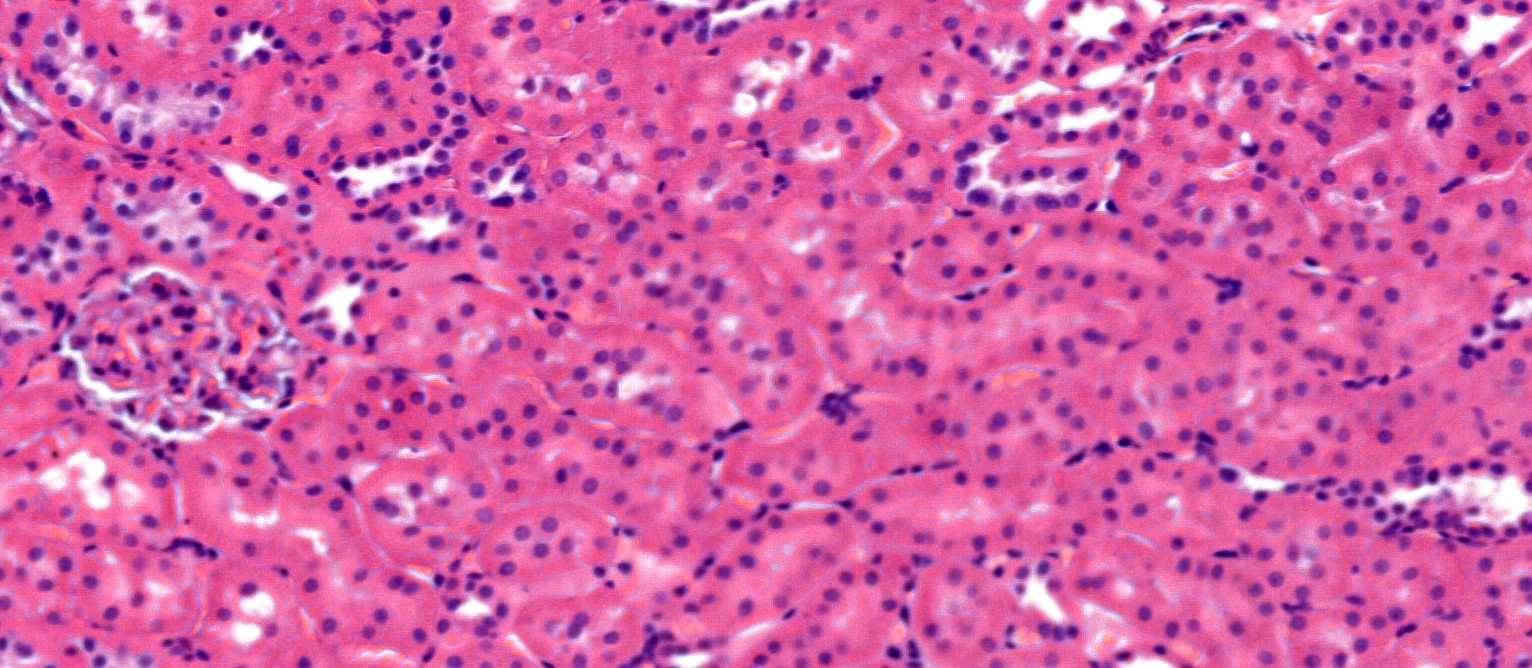

Supplement: Supplementary file 6 [file DataSheet4.ZIP › Fig 1D-HE-sham-7/7-5.jpeg]

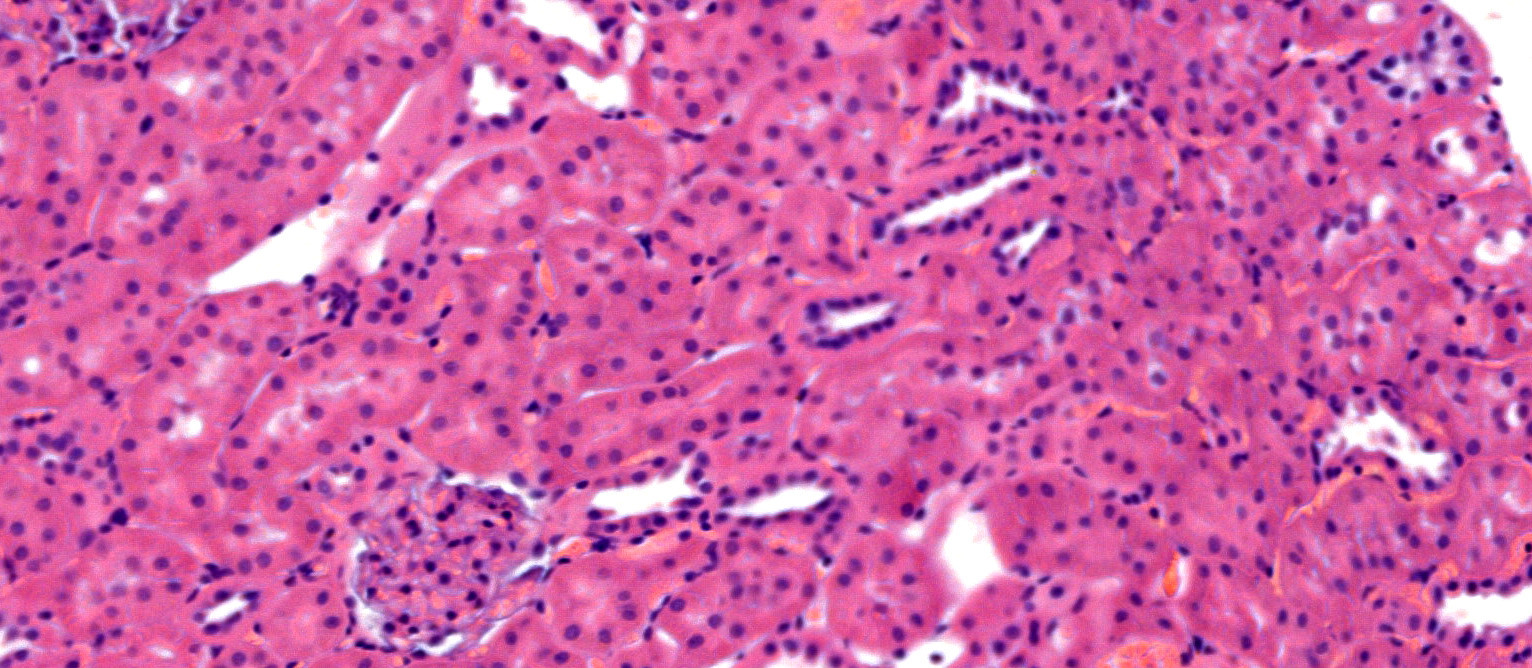

Supplement: Supplementary file 6 [file DataSheet4.ZIP › Fig 1D-HE-sham-7/7-6.jpeg]

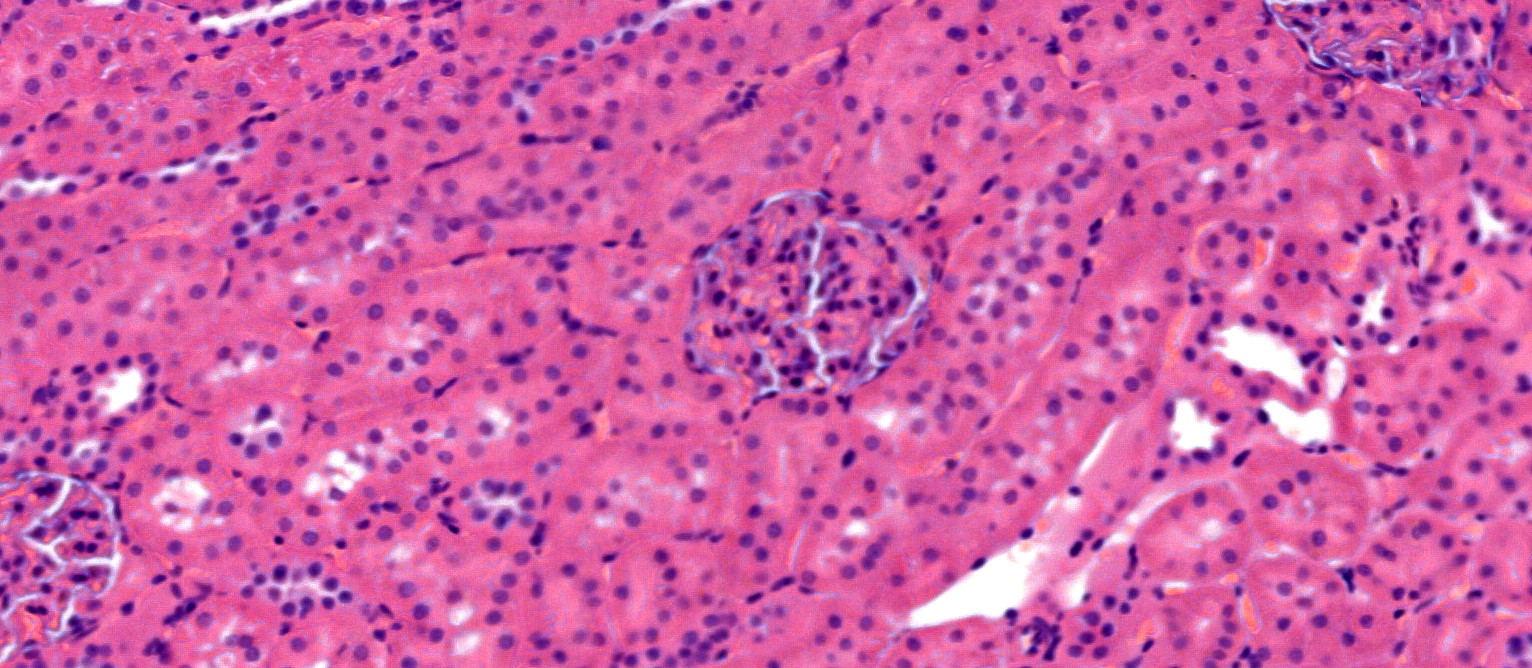

Supplement: Supplementary file 6 [file DataSheet4.ZIP › Fig 1D-HE-sham-7/7-7.jpeg]

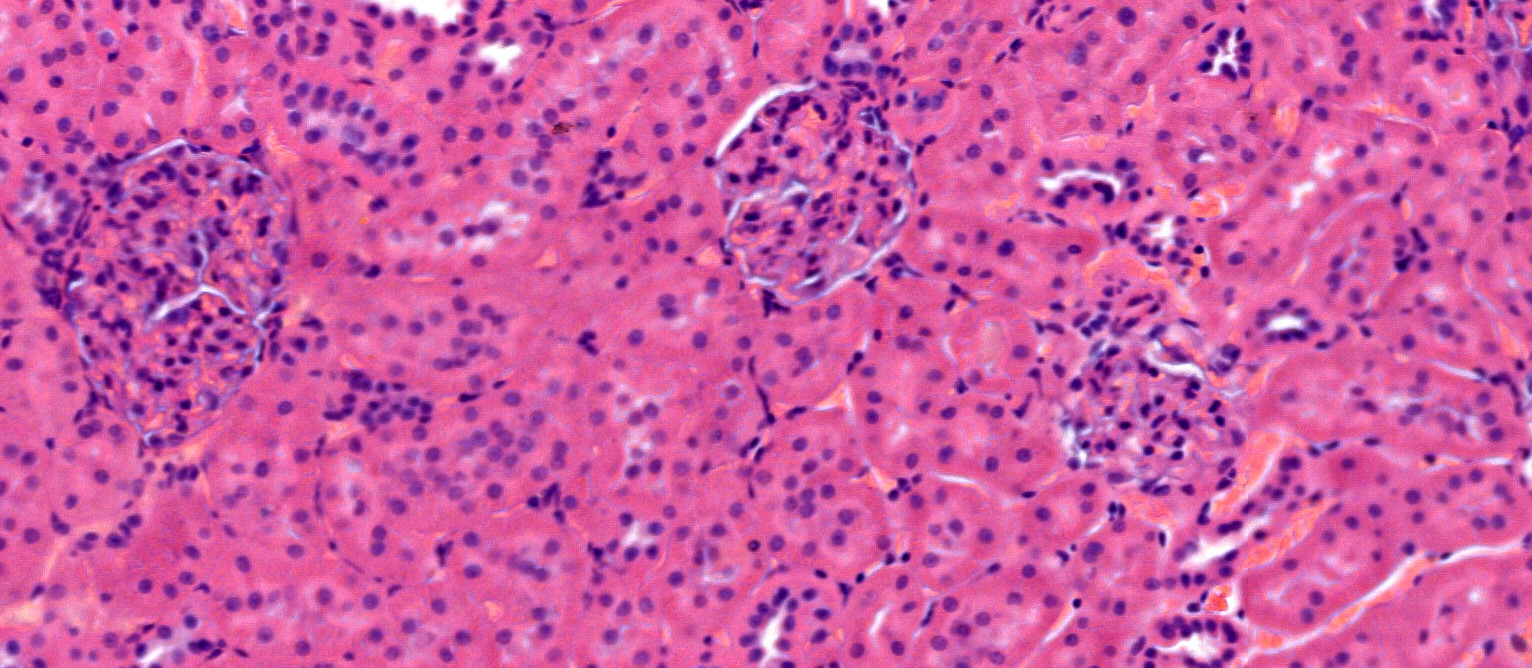

Supplement: Supplementary file 6 [file DataSheet4.ZIP › Fig 1D-HE-sham-7/7-8.jpeg]

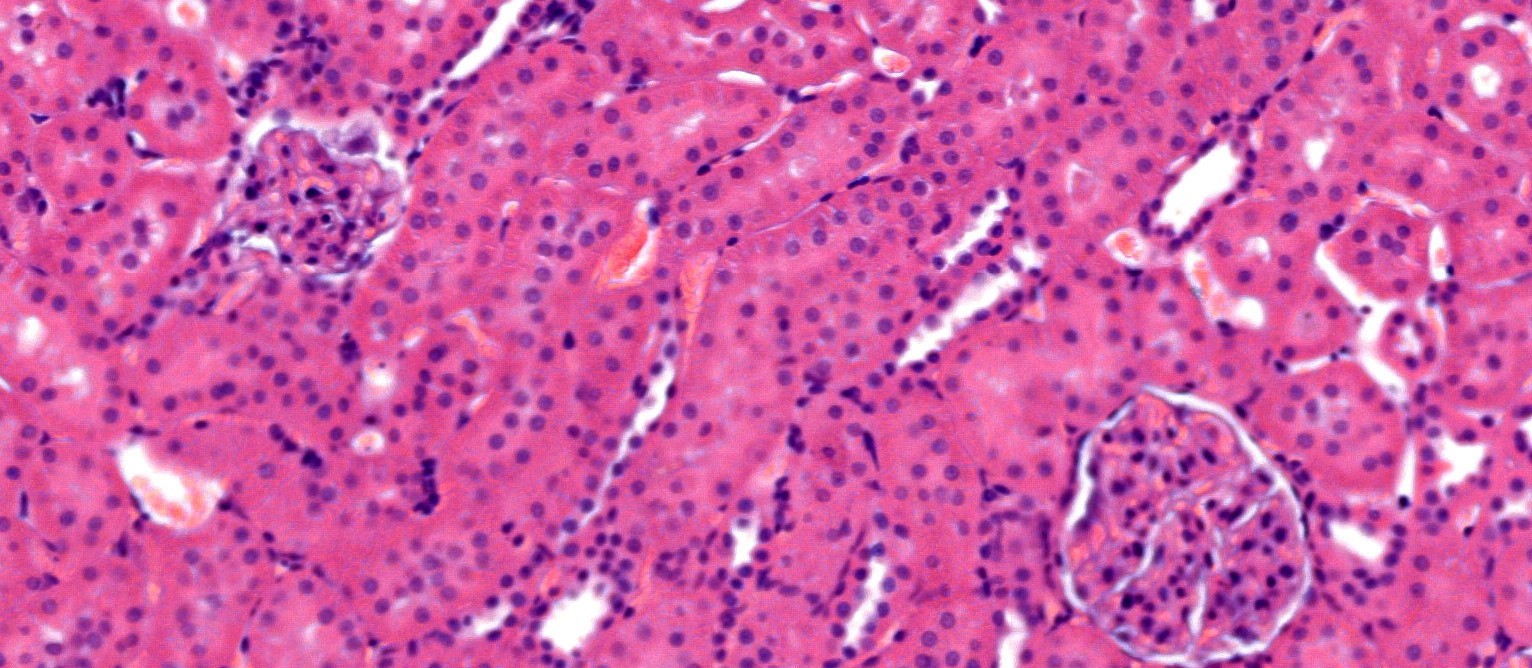

Supplement: Supplementary file 6 [file DataSheet4.ZIP › Fig 1D-HE-sham-7/7-9.jpeg]

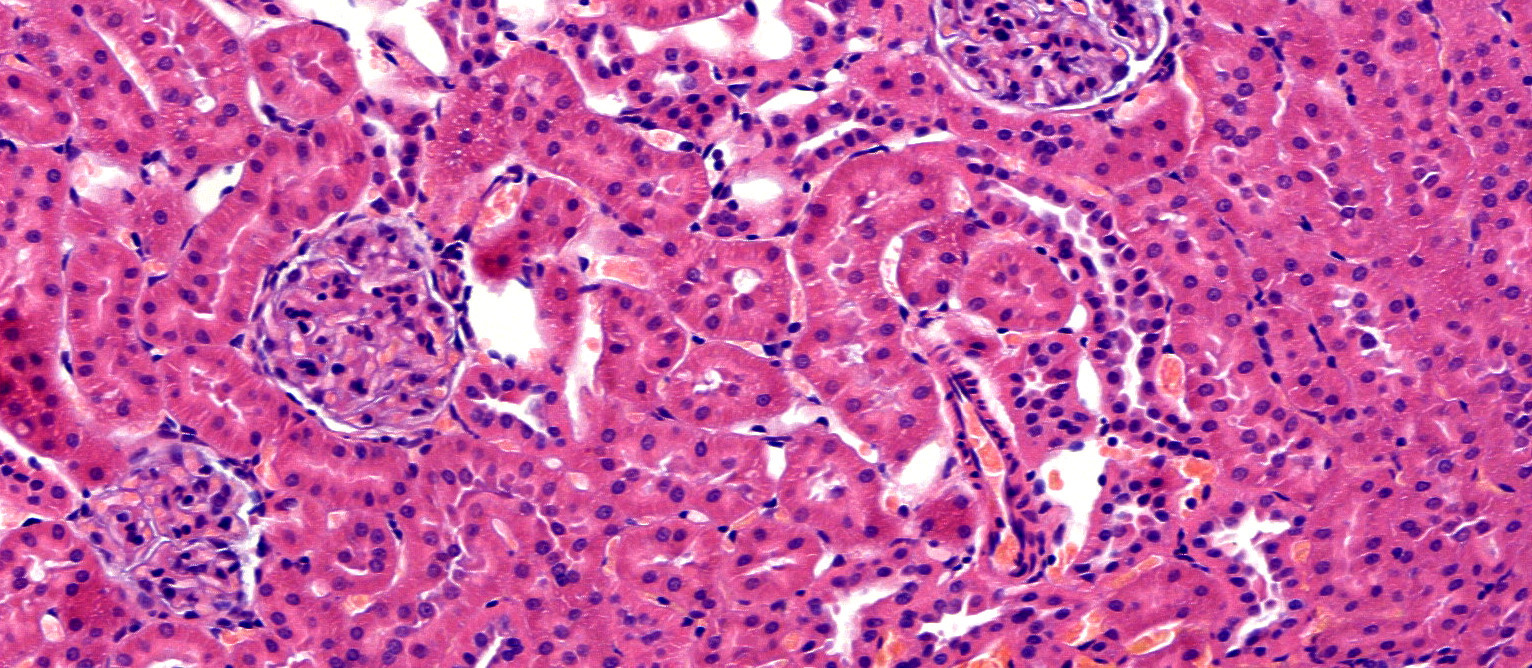

Supplement: Supplementary file 6 [file DataSheet4.ZIP › Fig 1D-HE-sham-8/8-1.jpeg]

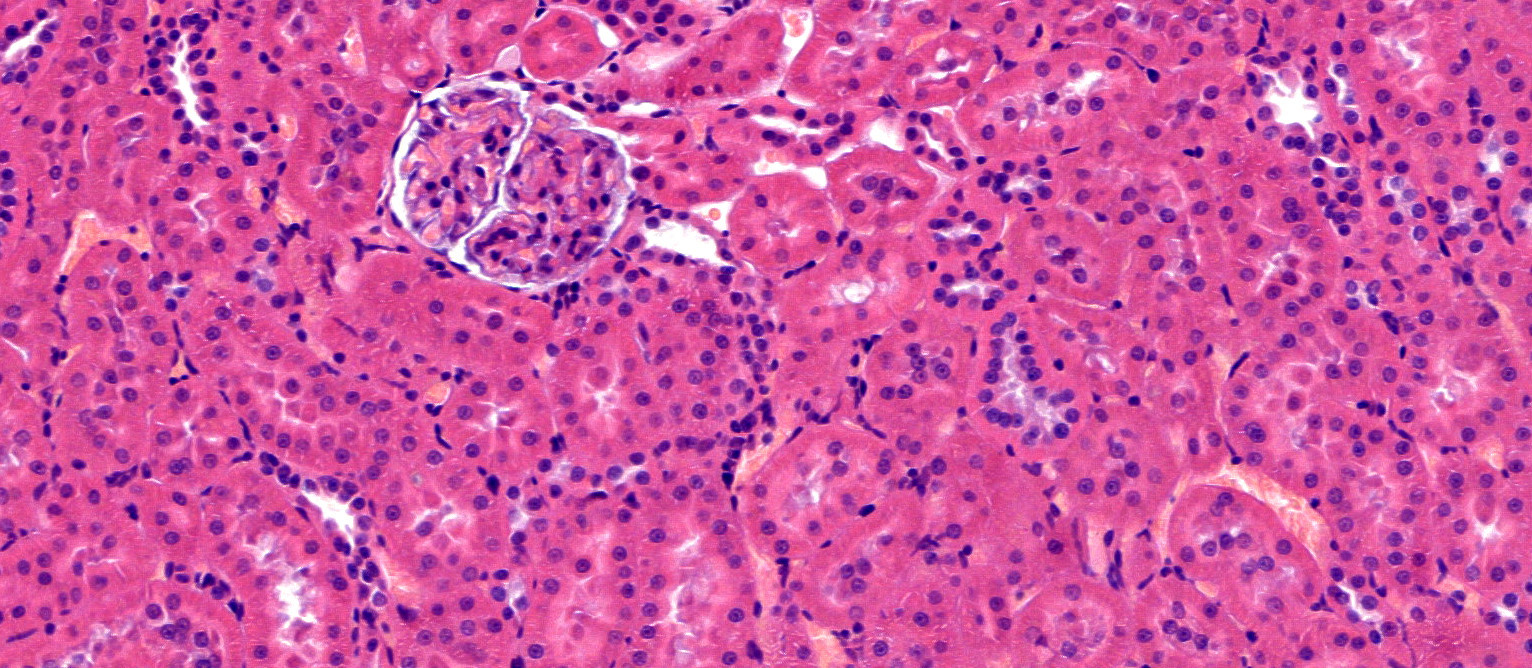

Supplement: Supplementary file 6 [file DataSheet4.ZIP › Fig 1D-HE-sham-8/8-10.jpeg]

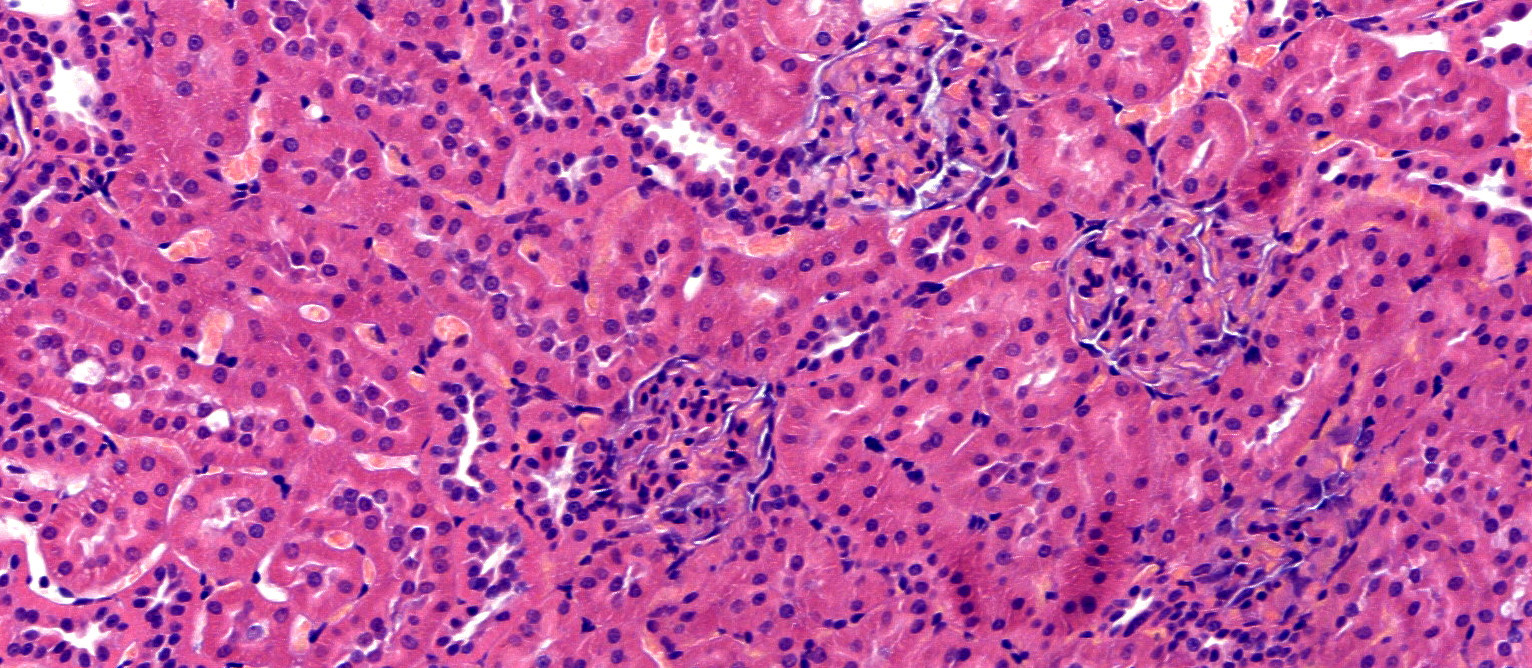

Supplement: Supplementary file 6 [file DataSheet4.ZIP › Fig 1D-HE-sham-8/8-2.jpeg]

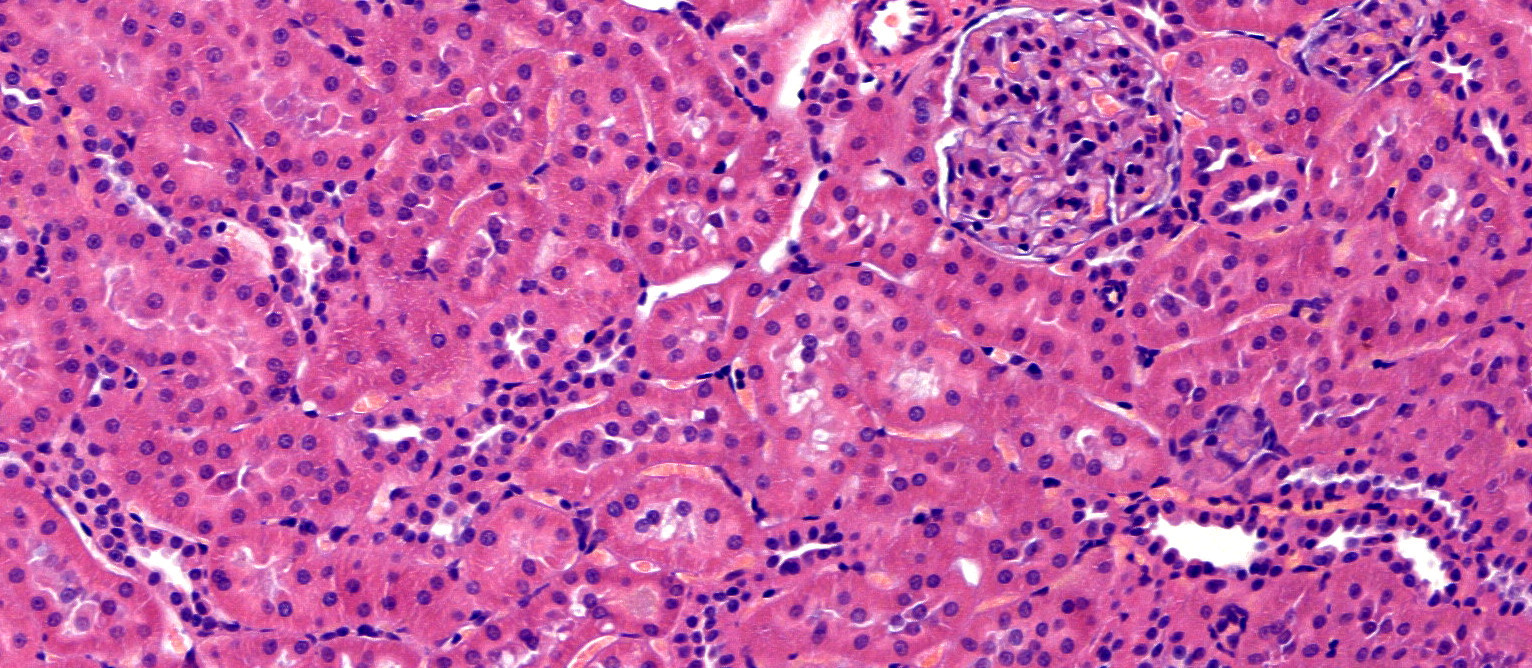

Supplement: Supplementary file 6 [file DataSheet4.ZIP › Fig 1D-HE-sham-8/8-3.jpeg]

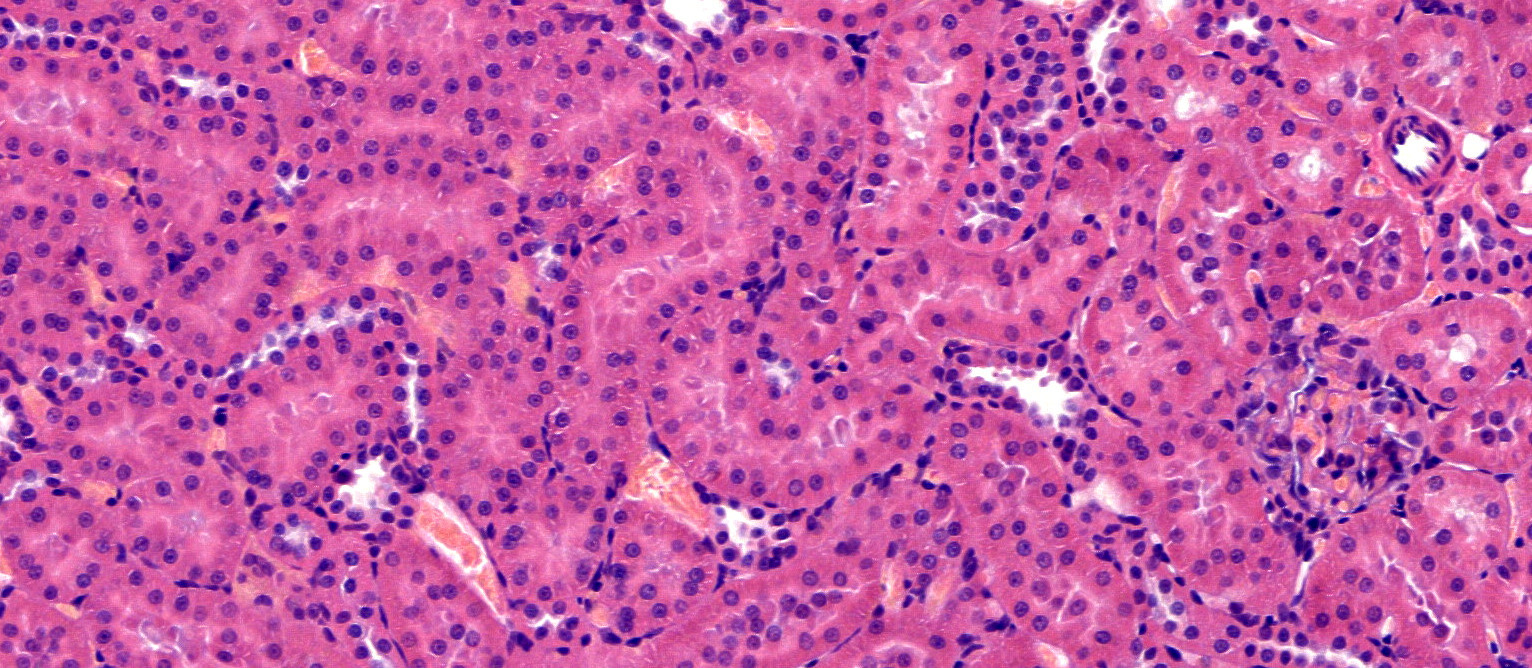

Supplement: Supplementary file 6 [file DataSheet4.ZIP › Fig 1D-HE-sham-8/8-4.jpeg]

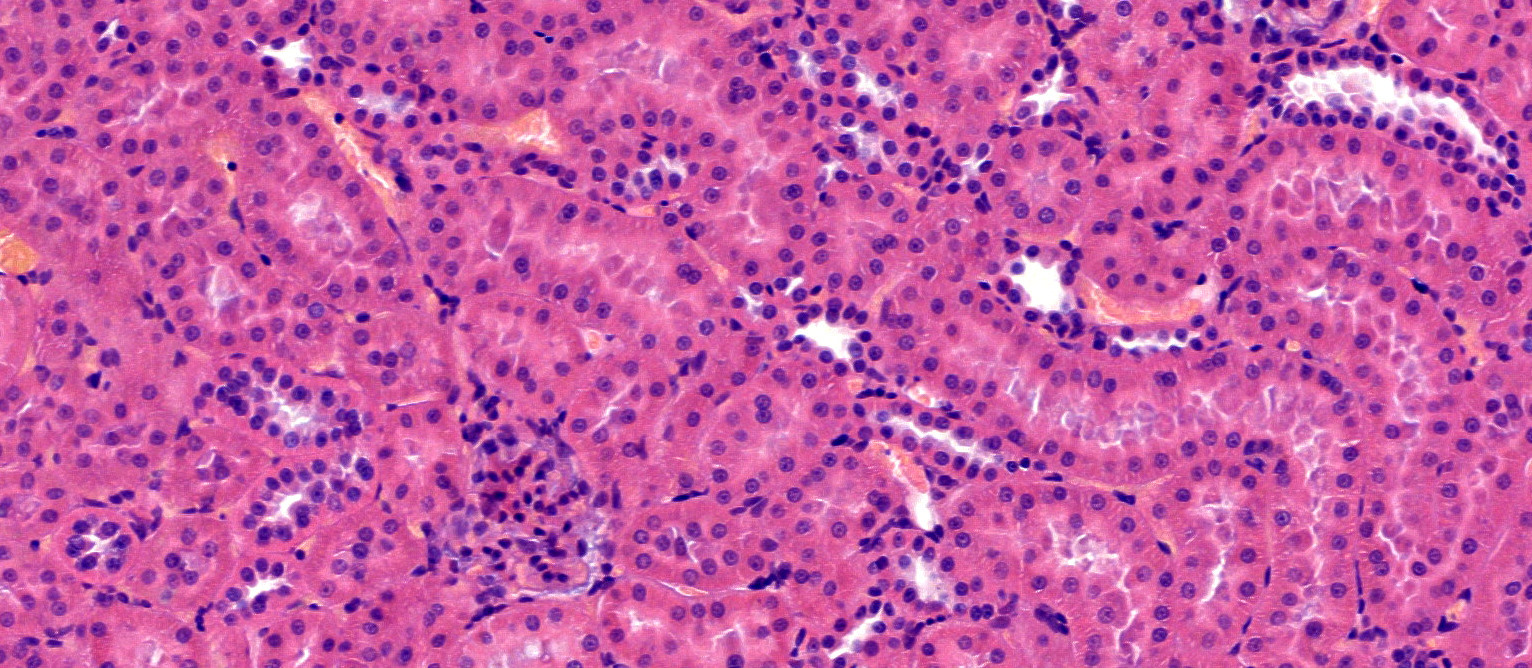

Supplement: Supplementary file 6 [file DataSheet4.ZIP › Fig 1D-HE-sham-8/8-5.jpeg]

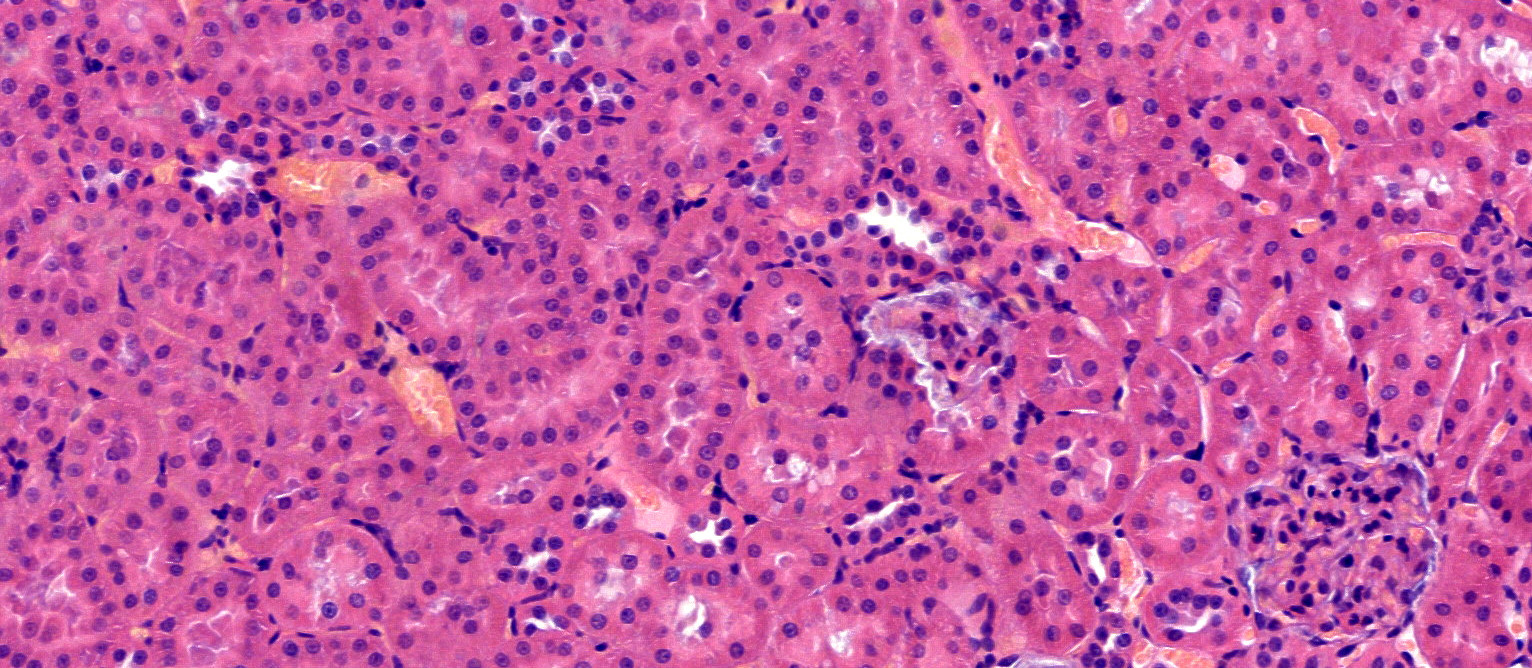

Supplement: Supplementary file 6 [file DataSheet4.ZIP › Fig 1D-HE-sham-8/8-6.jpeg]

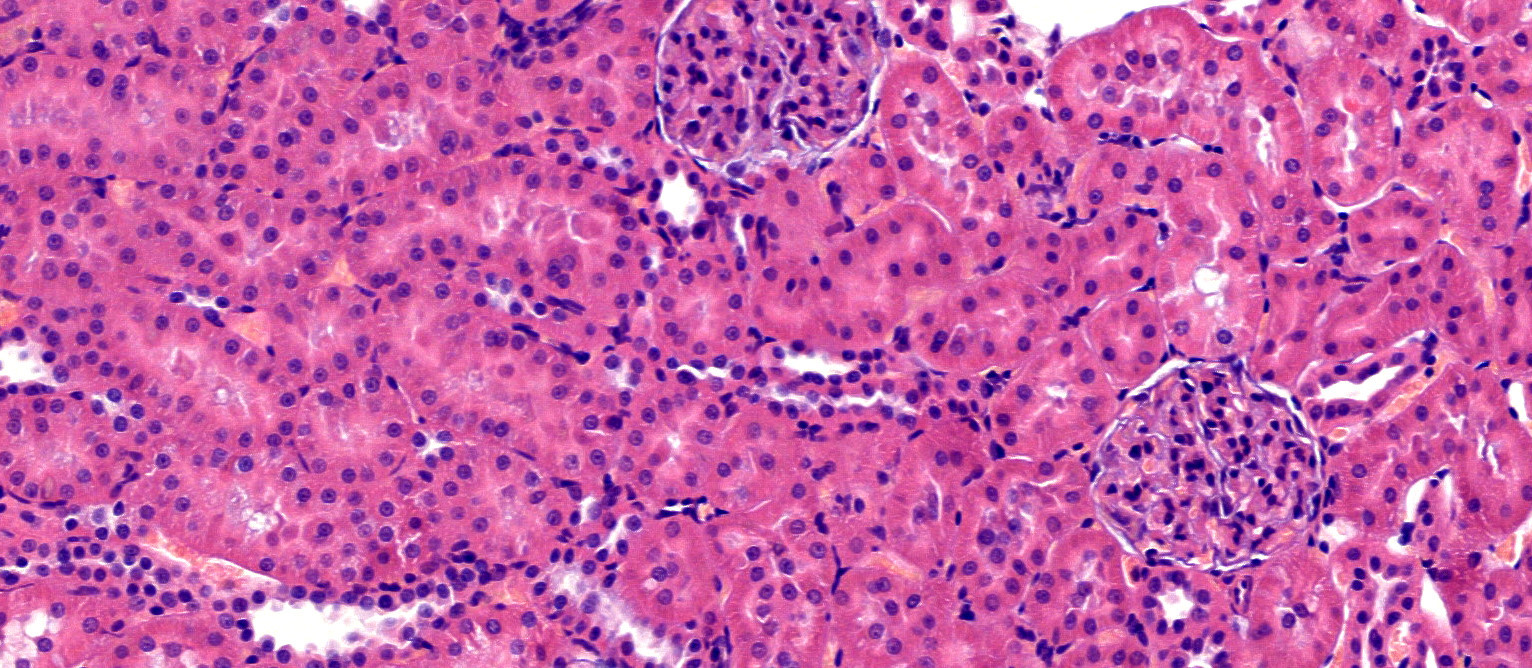

Supplement: Supplementary file 6 [file DataSheet4.ZIP › Fig 1D-HE-sham-8/8-7.jpeg]

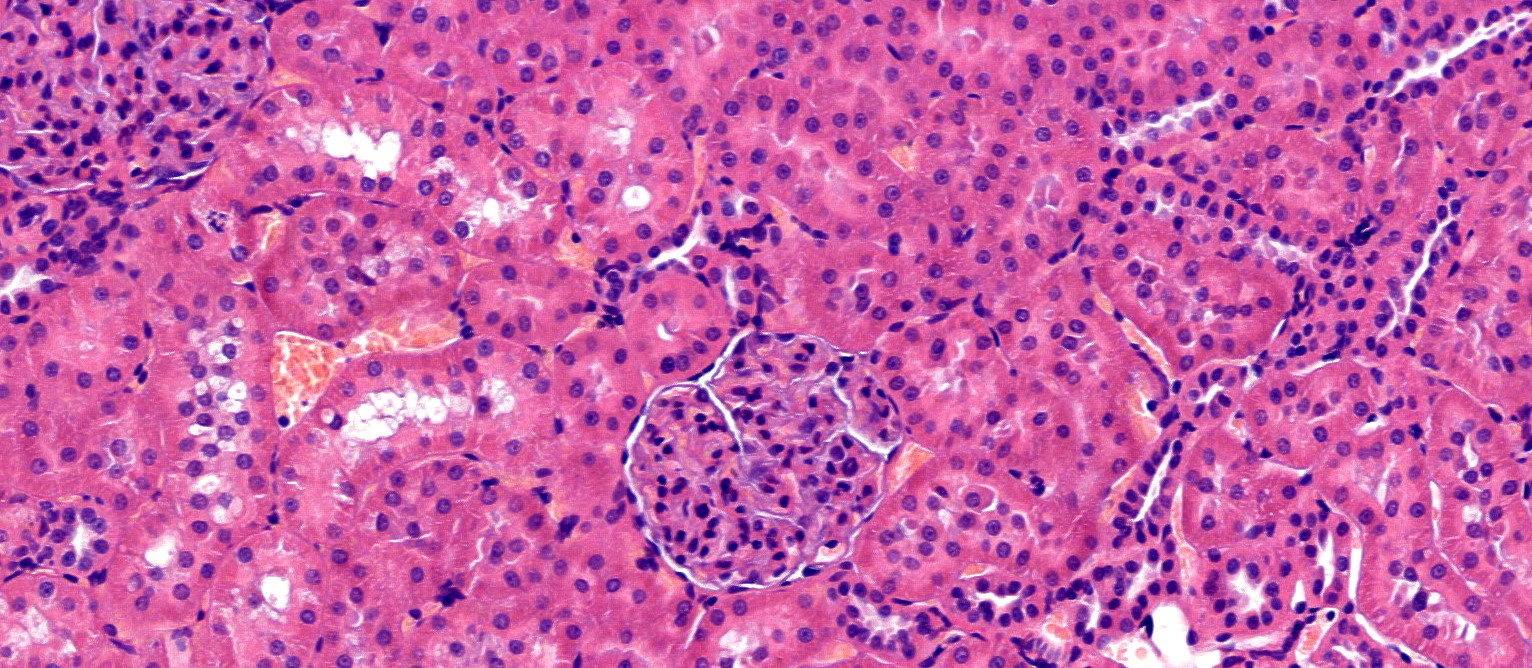

Supplement: Supplementary file 6 [file DataSheet4.ZIP › Fig 1D-HE-sham-8/8-8.jpeg]

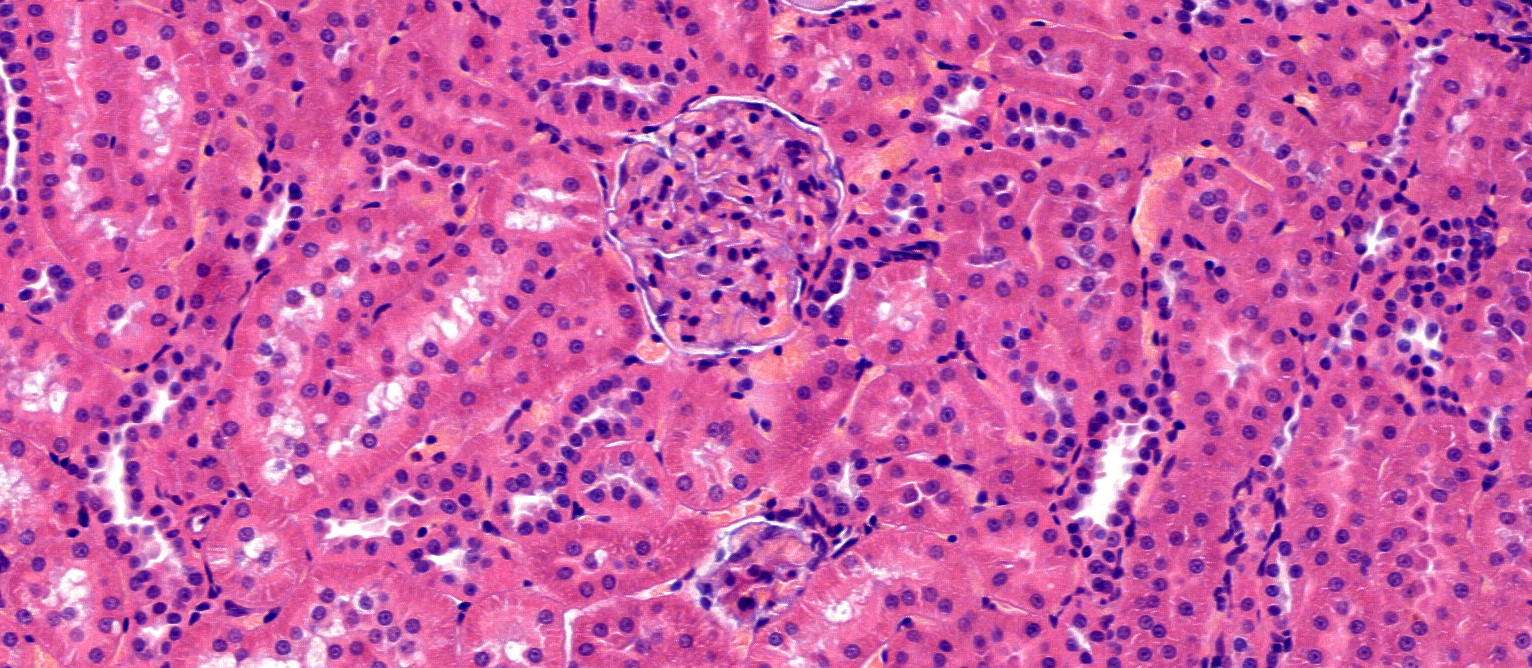

Supplement: Supplementary file 6 [file DataSheet4.ZIP › Fig 1D-HE-sham-8/8-9.jpeg]

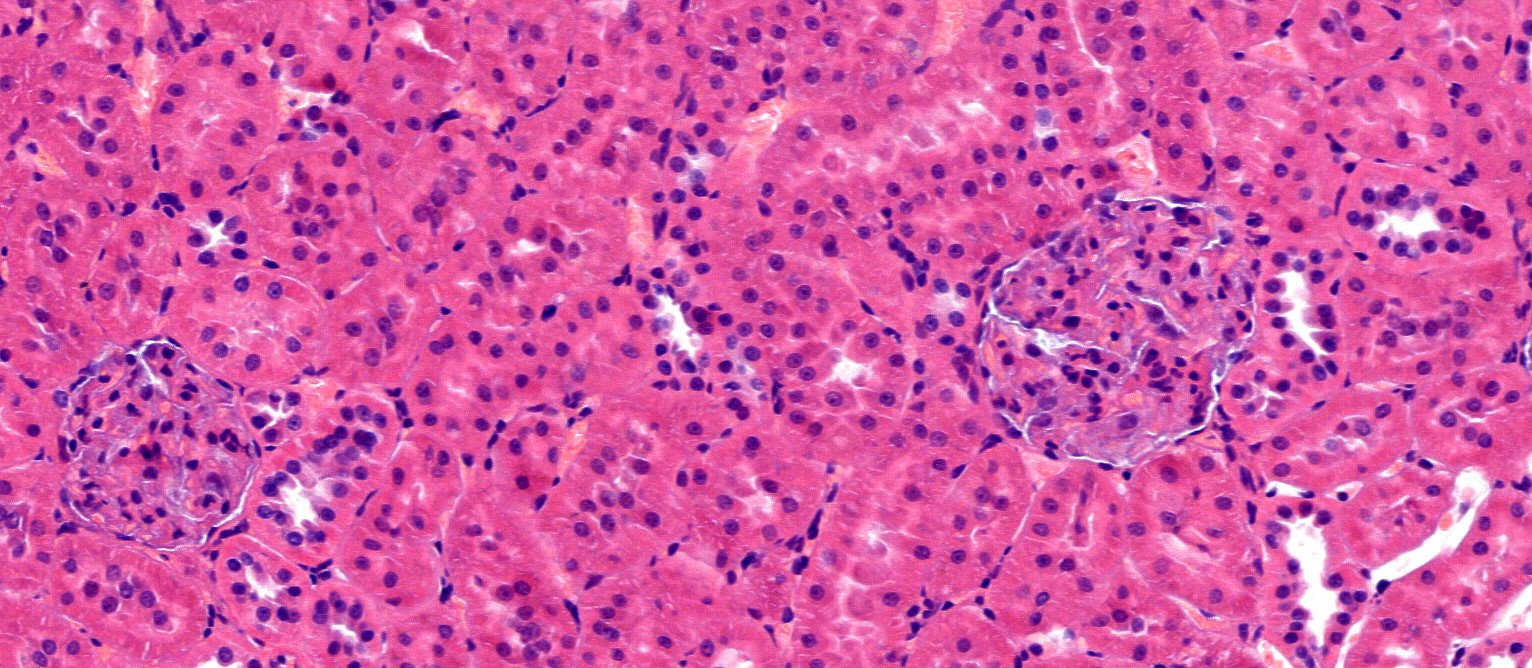

Supplement: Supplementary file 6 [file DataSheet4.ZIP › Fig 1D-HE-sham-9/9-1.jpeg]

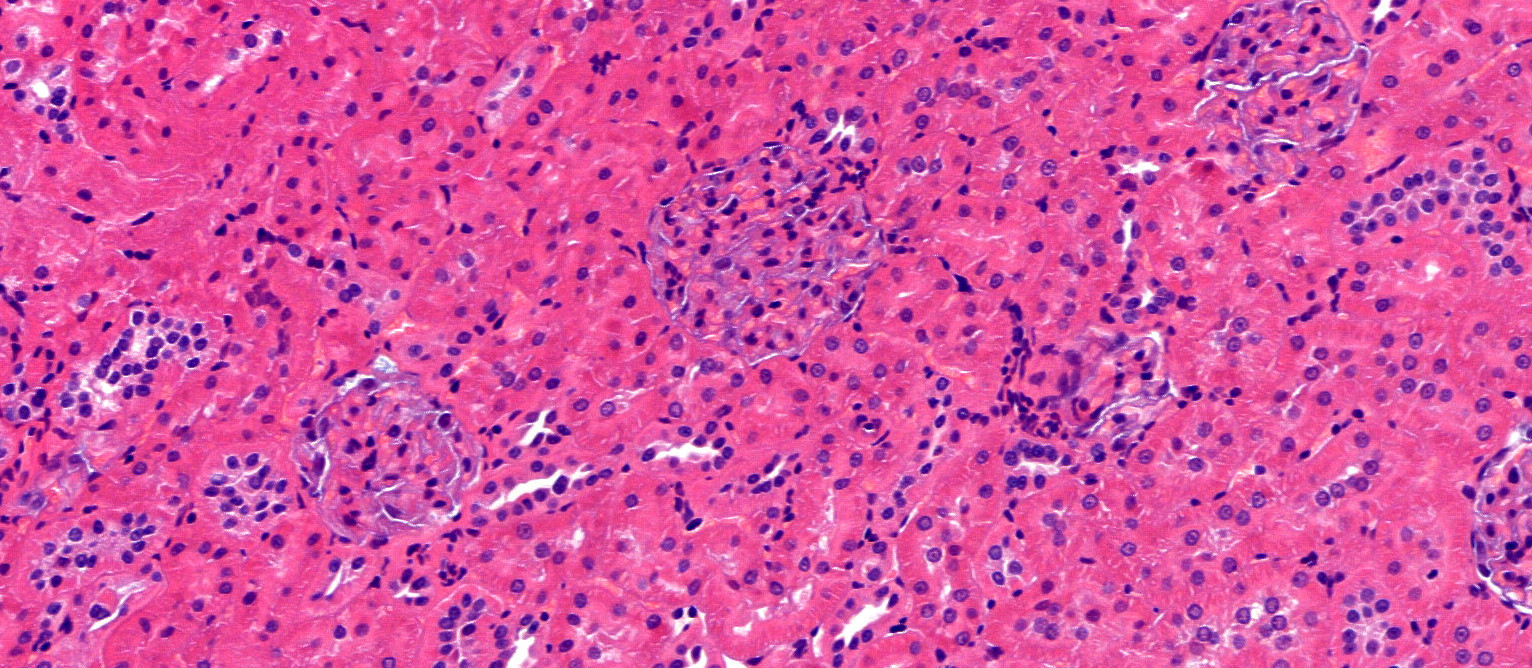

Supplement: Supplementary file 6 [file DataSheet4.ZIP › Fig 1D-HE-sham-9/9-10.jpeg]

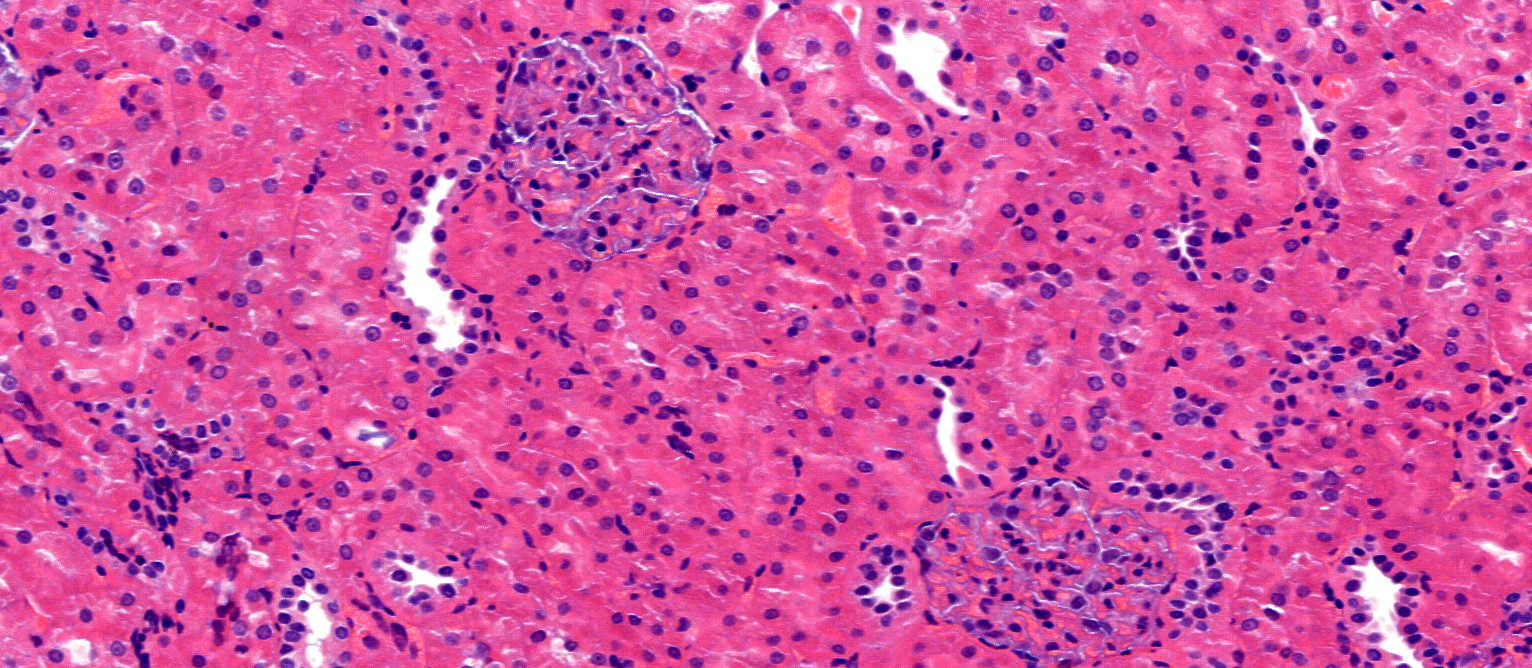

Supplement: Supplementary file 6 [file DataSheet4.ZIP › Fig 1D-HE-sham-9/9-2.jpeg]

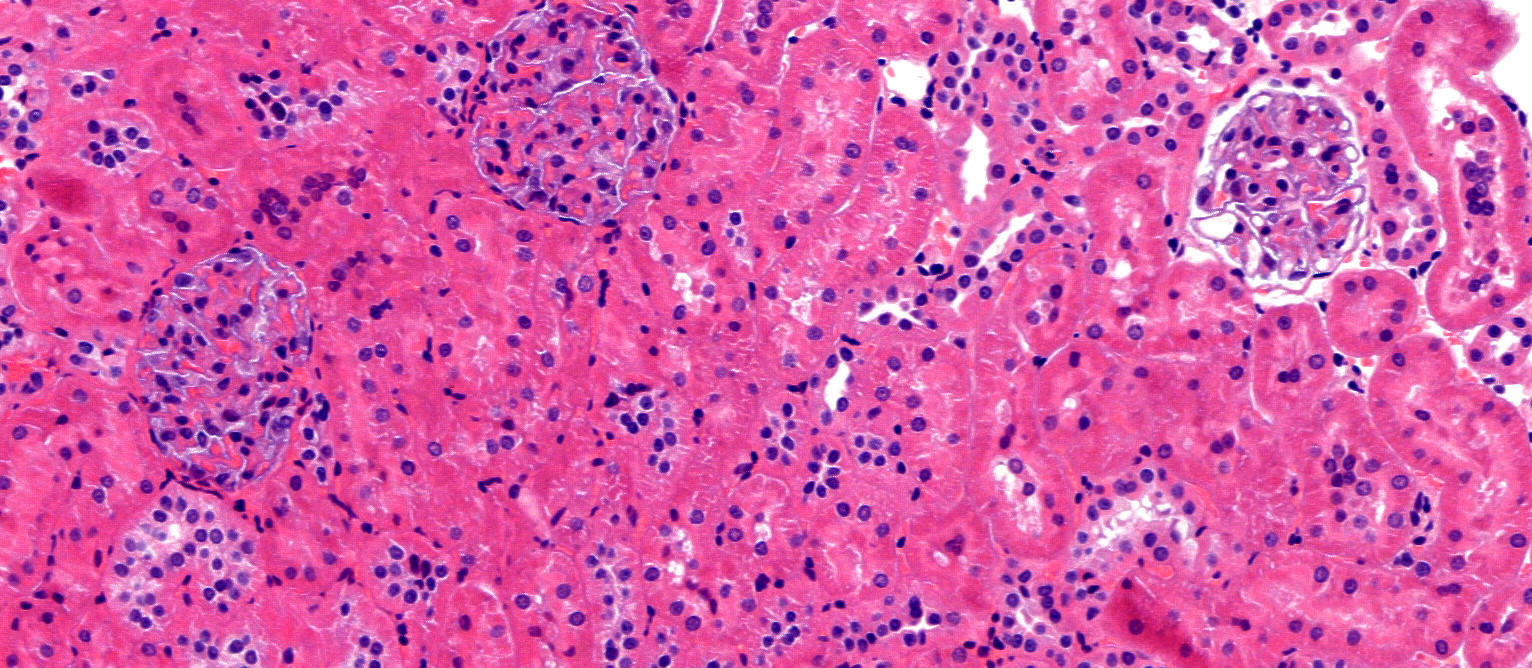

Supplement: Supplementary file 6 [file DataSheet4.ZIP › Fig 1D-HE-sham-9/9-3.jpeg]

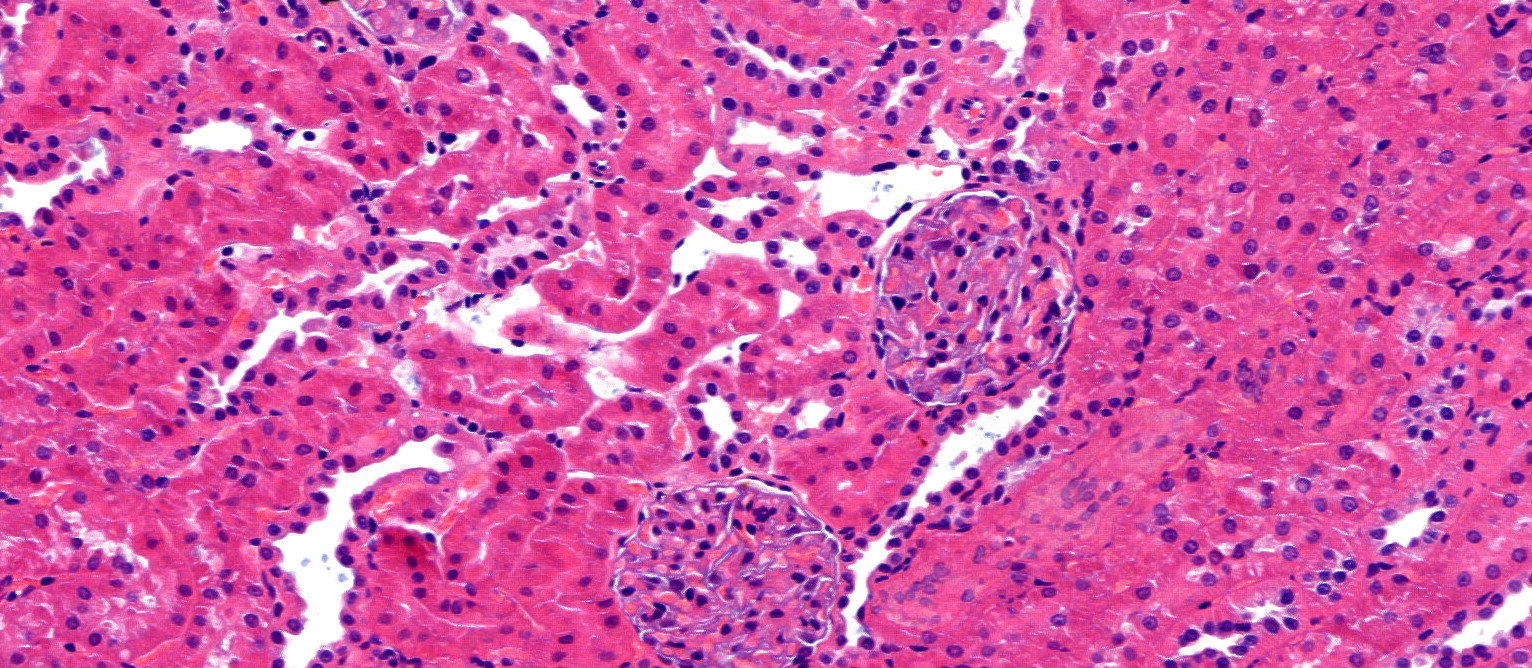

Supplement: Supplementary file 6 [file DataSheet4.ZIP › Fig 1D-HE-sham-9/9-4.jpeg]

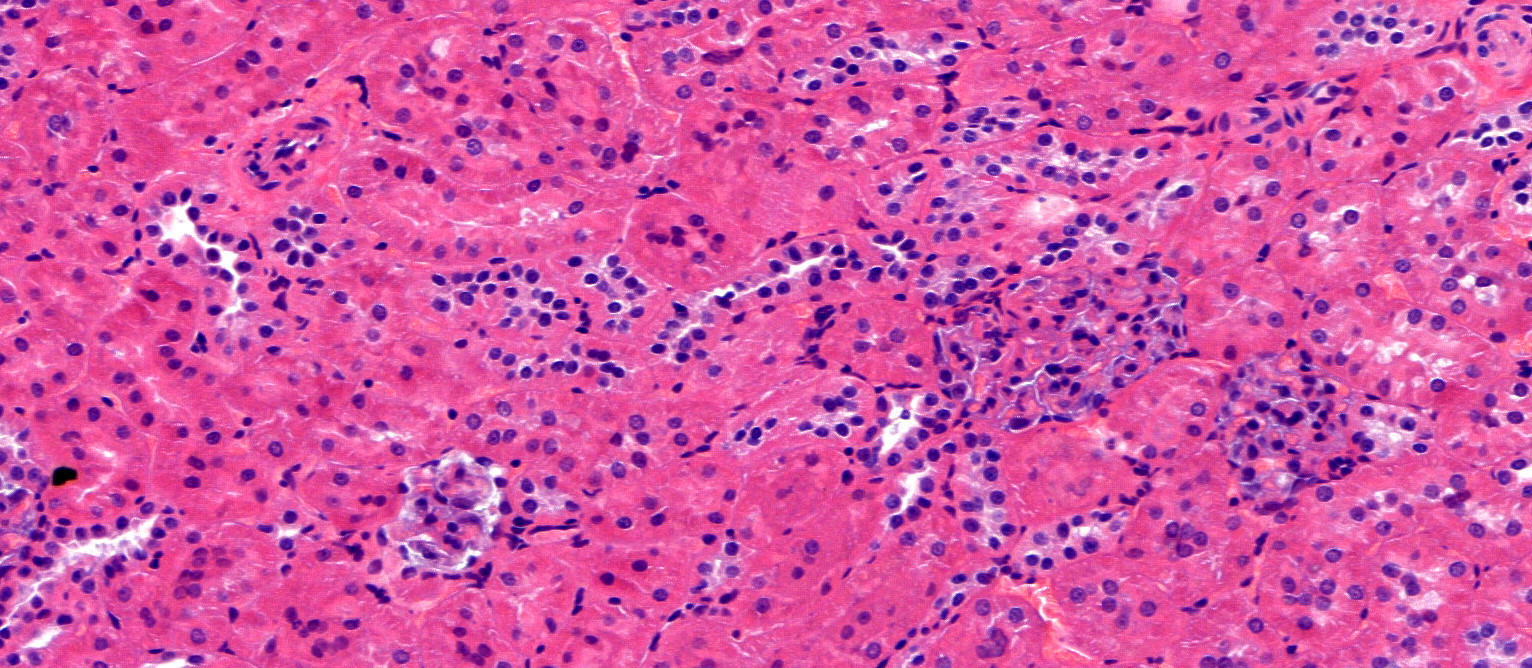

Supplement: Supplementary file 6 [file DataSheet4.ZIP › Fig 1D-HE-sham-9/9-5.jpeg]

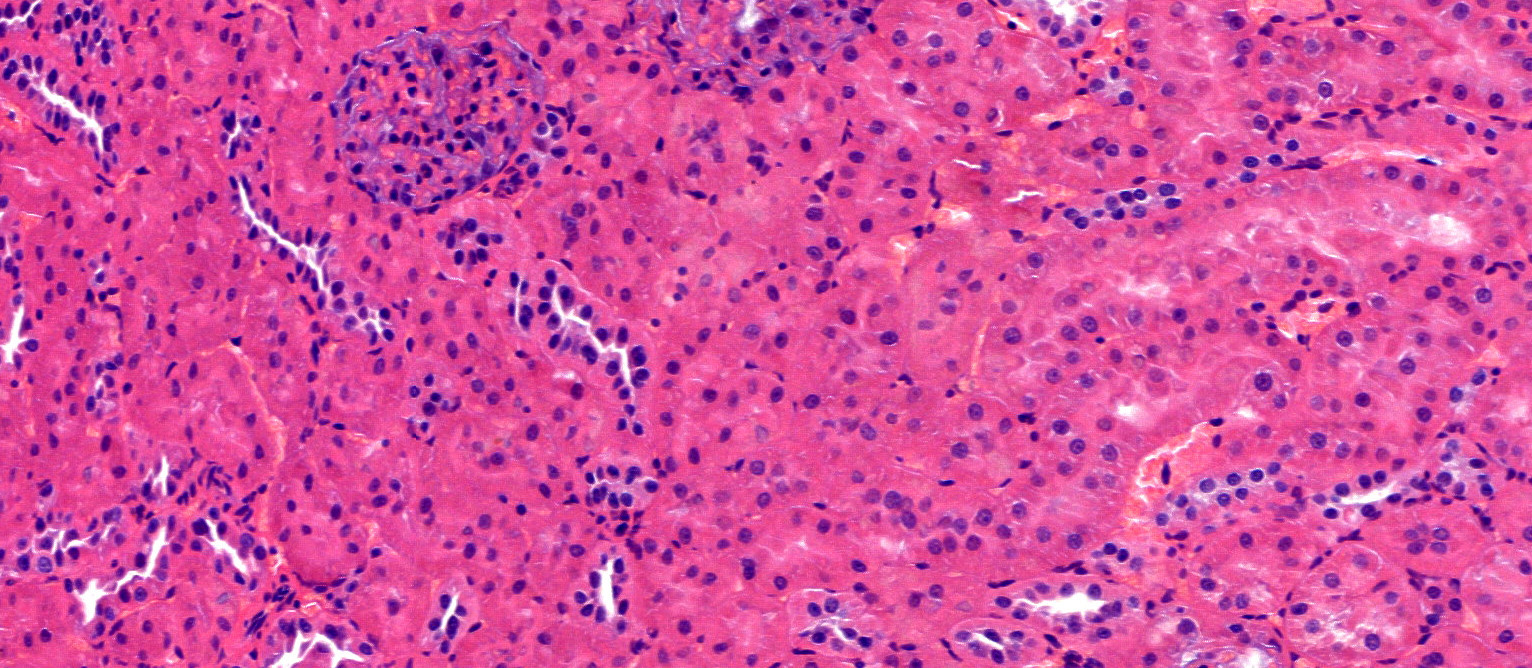

Supplement: Supplementary file 6 [file DataSheet4.ZIP › Fig 1D-HE-sham-9/9-6.jpeg]

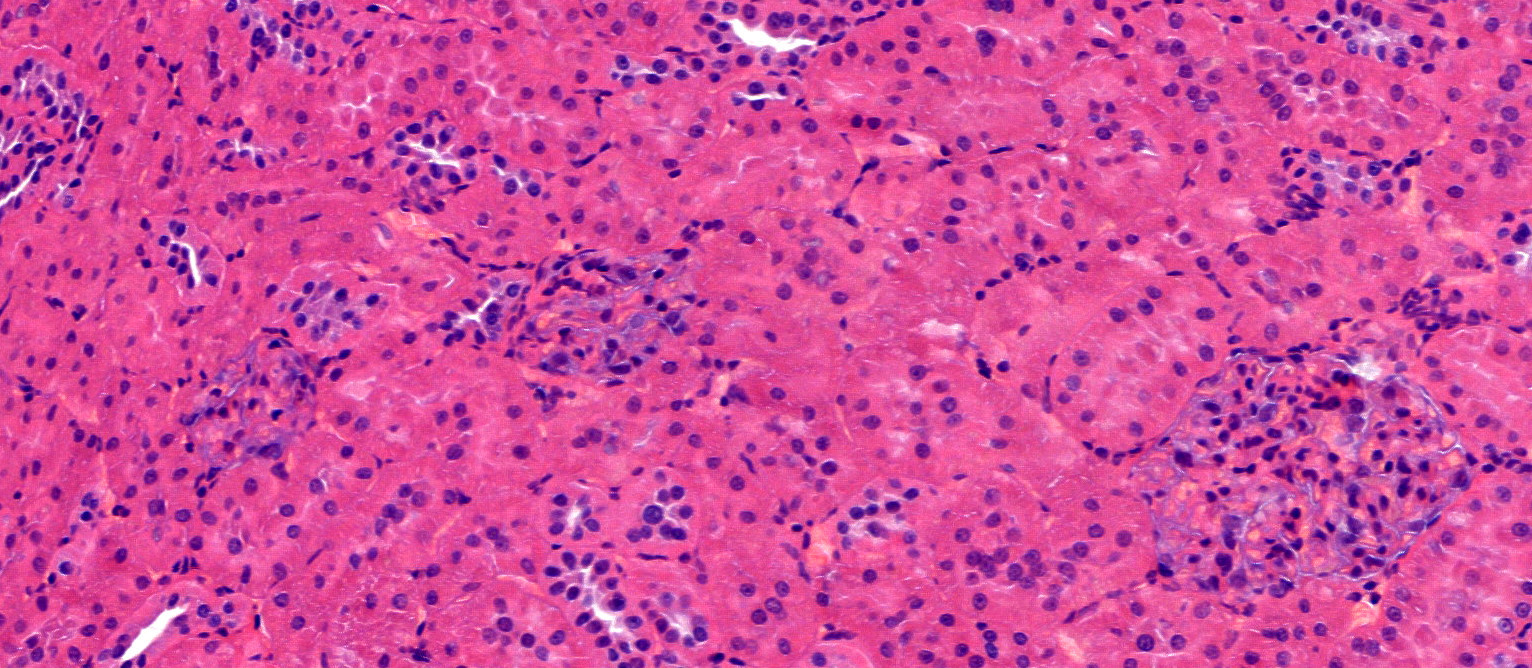

Supplement: Supplementary file 6 [file DataSheet4.ZIP › Fig 1D-HE-sham-9/9-7.jpeg]

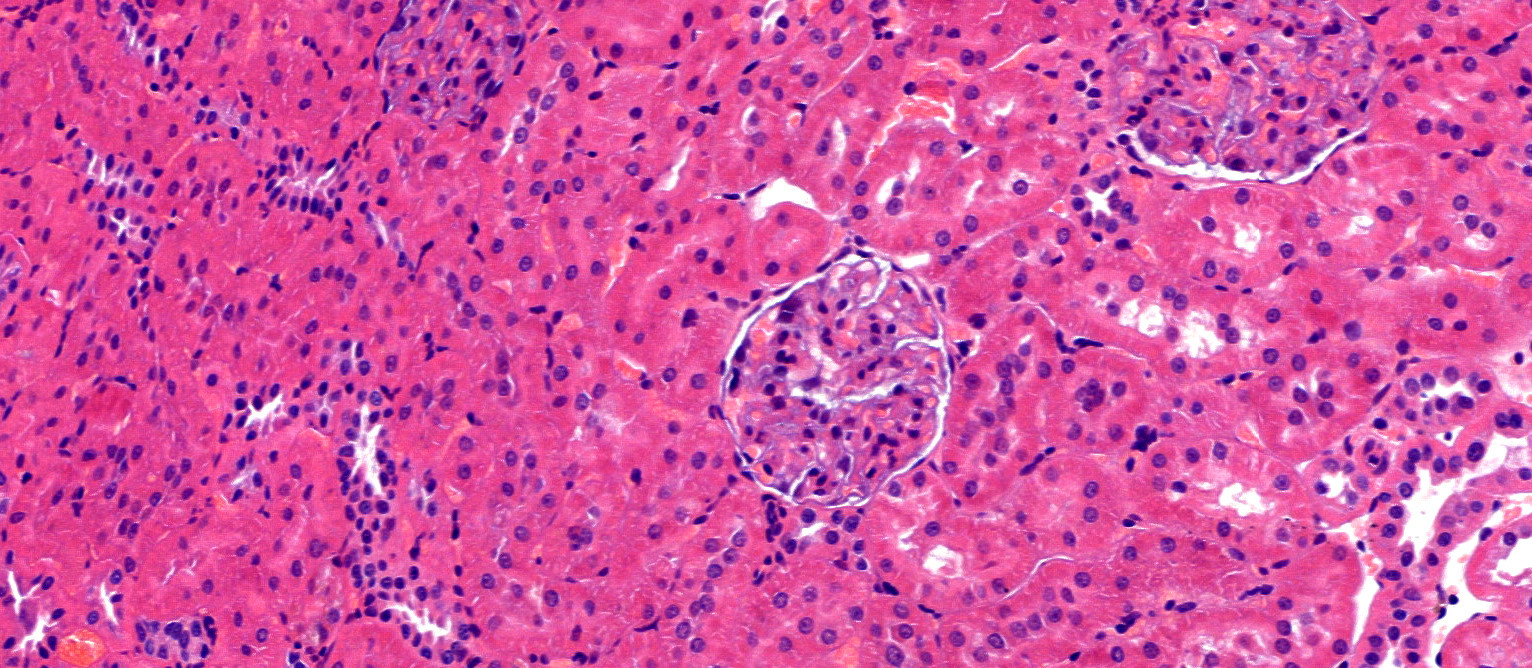

Supplement: Supplementary file 6 [file DataSheet4.ZIP › Fig 1D-HE-sham-9/9-8.jpeg]

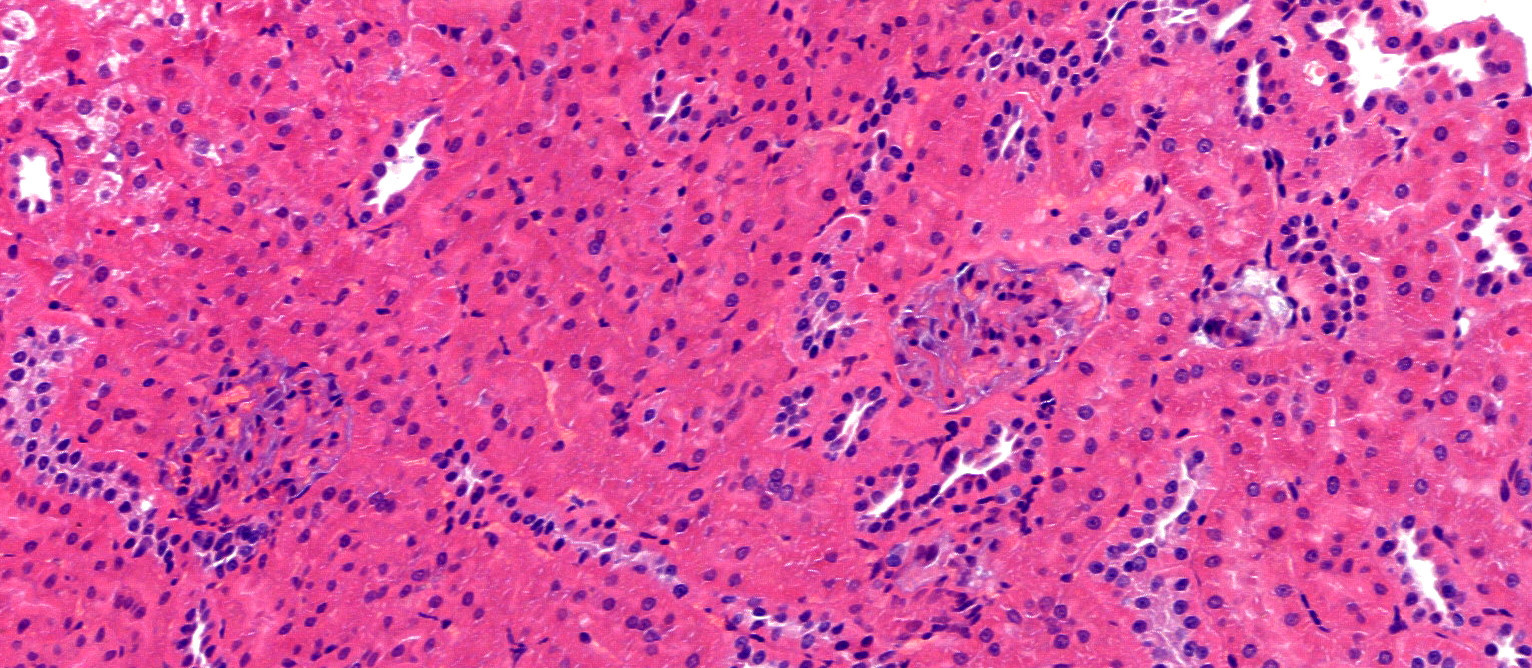

Supplement: Supplementary file 6 [file DataSheet4.ZIP › Fig 1D-HE-sham-9/9-9.jpeg]

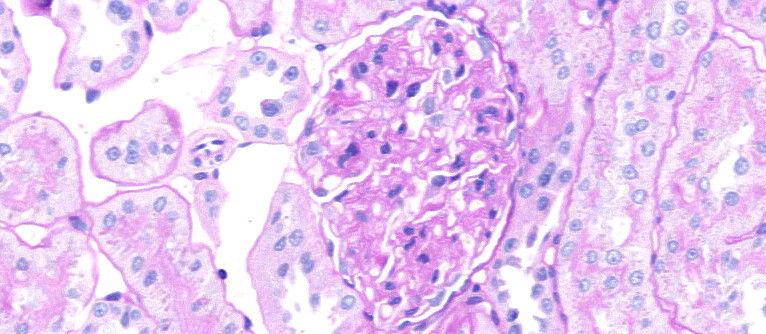

Supplement: Supplementary file 7 [file DataSheet13.ZIP › DKD/Fig 1D-PAS-DKD-14/14-1.jpeg]

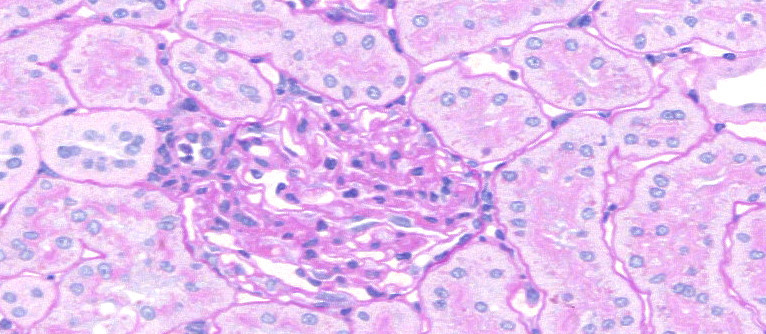

Supplement: Supplementary file 7 [file DataSheet13.ZIP › DKD/Fig 1D-PAS-DKD-14/14-10.jpeg]

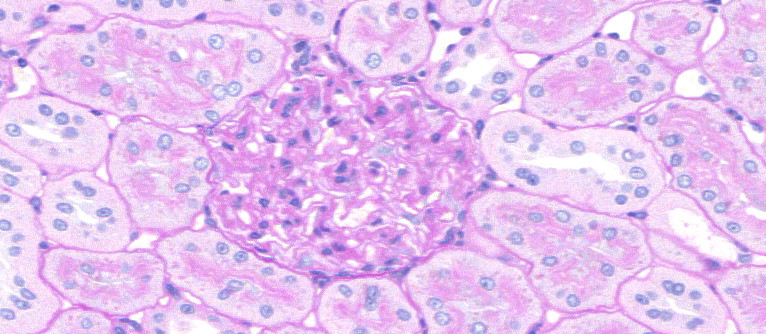

Supplement: Supplementary file 7 [file DataSheet13.ZIP › DKD/Fig 1D-PAS-DKD-14/14-11.jpeg]

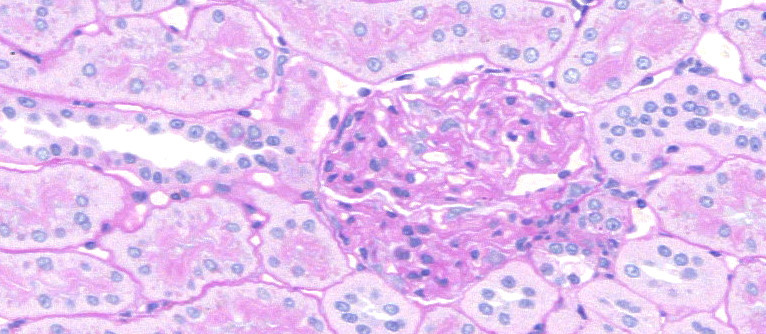

Supplement: Supplementary file 7 [file DataSheet13.ZIP › DKD/Fig 1D-PAS-DKD-14/14-12.jpeg]

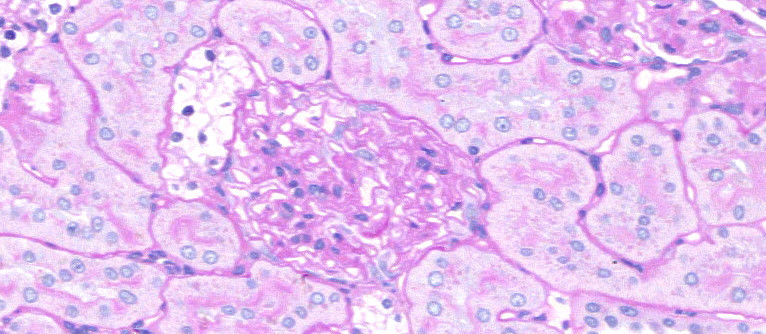

Supplement: Supplementary file 7 [file DataSheet13.ZIP › DKD/Fig 1D-PAS-DKD-14/14-13.jpeg]

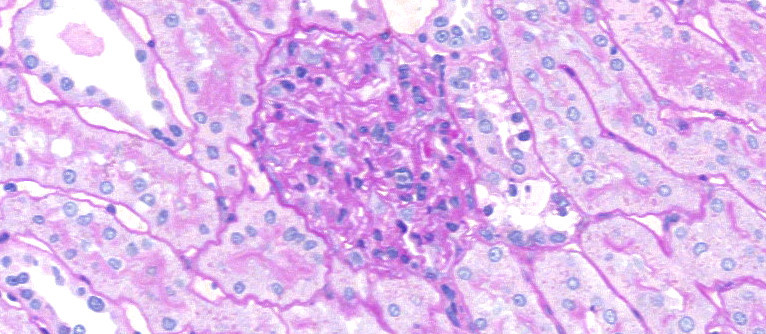

Supplement: Supplementary file 7 [file DataSheet13.ZIP › DKD/Fig 1D-PAS-DKD-14/14-14.jpeg]

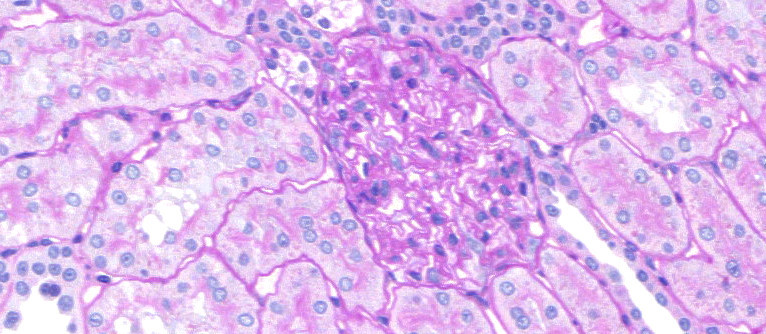

Supplement: Supplementary file 7 [file DataSheet13.ZIP › DKD/Fig 1D-PAS-DKD-14/14-15.jpeg]

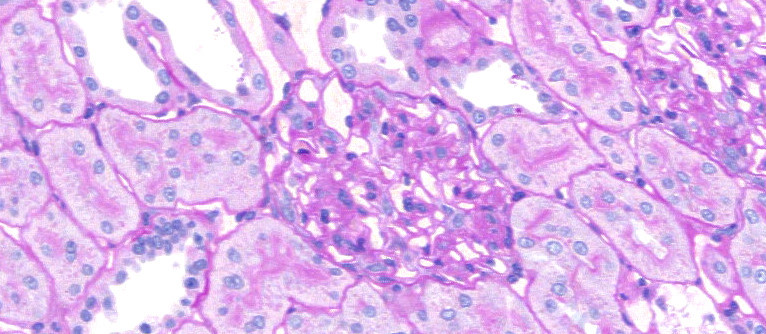

Supplement: Supplementary file 7 [file DataSheet13.ZIP › DKD/Fig 1D-PAS-DKD-14/14-16.jpeg]

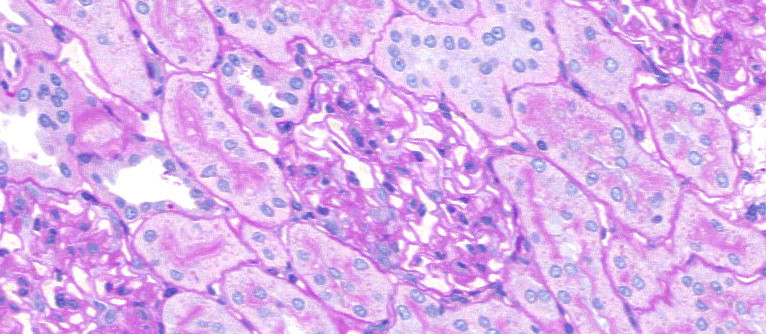

Supplement: Supplementary file 7 [file DataSheet13.ZIP › DKD/Fig 1D-PAS-DKD-14/14-17.jpeg]

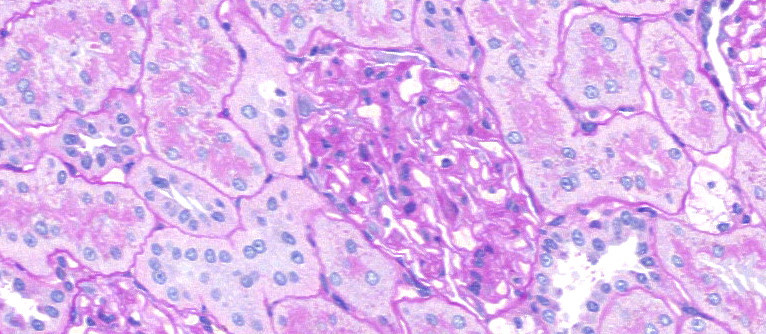

Supplement: Supplementary file 7 [file DataSheet13.ZIP › DKD/Fig 1D-PAS-DKD-14/14-18.jpeg]

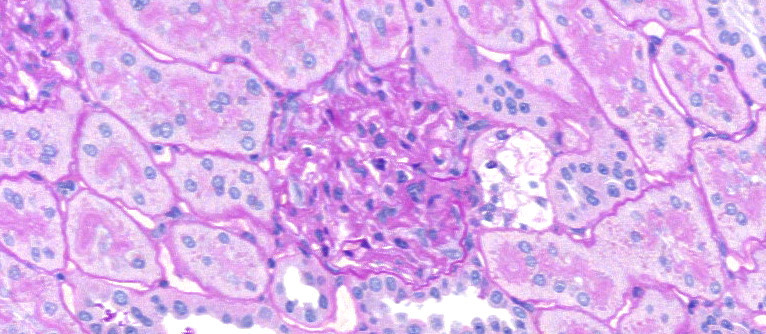

Supplement: Supplementary file 7 [file DataSheet13.ZIP › DKD/Fig 1D-PAS-DKD-14/14-19.jpeg]

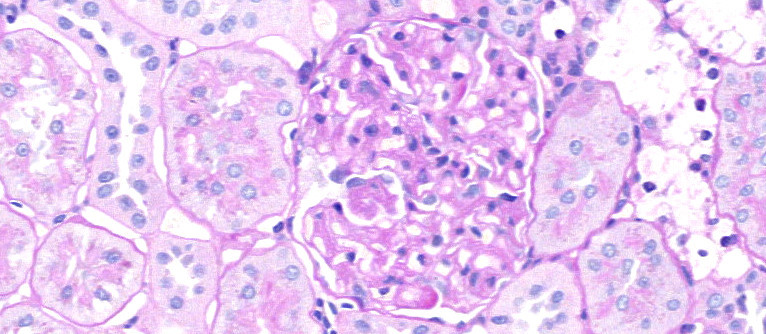

Supplement: Supplementary file 7 [file DataSheet13.ZIP › DKD/Fig 1D-PAS-DKD-14/14-2.jpeg]

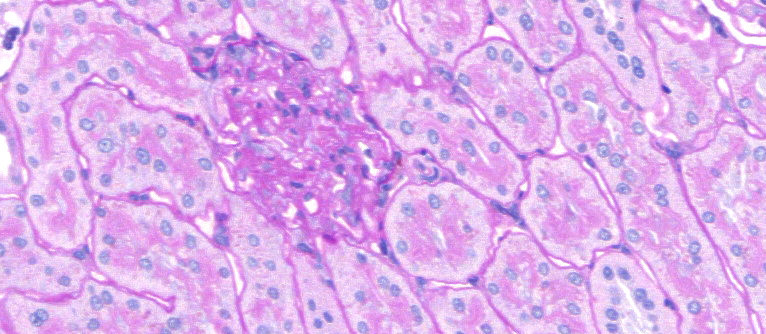

Supplement: Supplementary file 7 [file DataSheet13.ZIP › DKD/Fig 1D-PAS-DKD-14/14-20.jpeg]

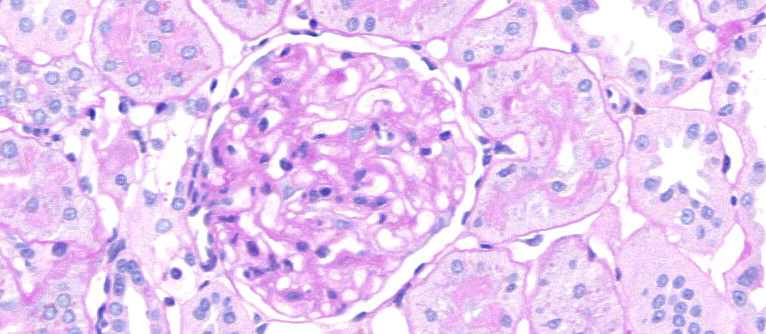

Supplement: Supplementary file 7 [file DataSheet13.ZIP › DKD/Fig 1D-PAS-DKD-14/14-3.jpeg]

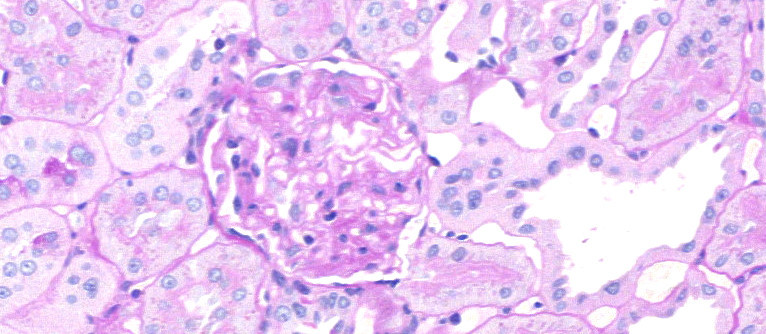

Supplement: Supplementary file 7 [file DataSheet13.ZIP › DKD/Fig 1D-PAS-DKD-14/14-4.jpeg]

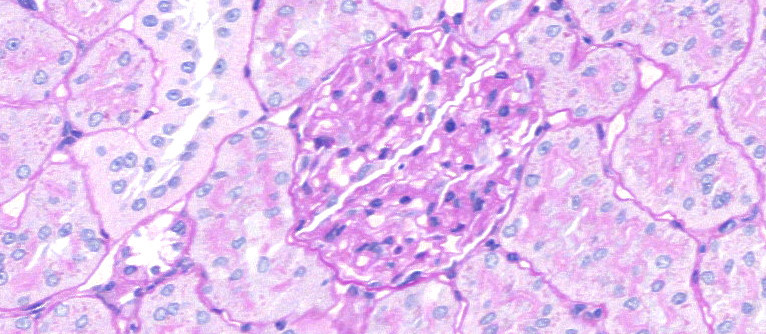

Supplement: Supplementary file 7 [file DataSheet13.ZIP › DKD/Fig 1D-PAS-DKD-14/14-5.jpeg]

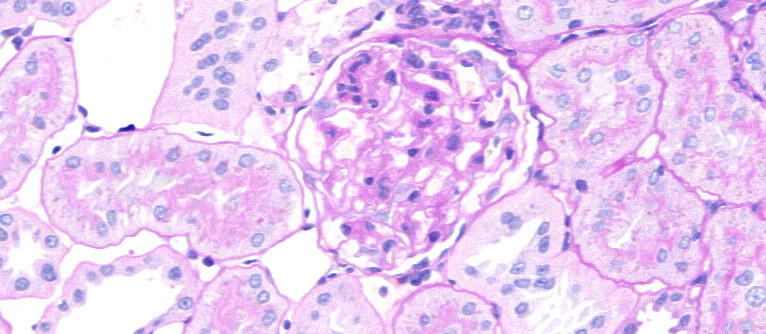

Supplement: Supplementary file 7 [file DataSheet13.ZIP › DKD/Fig 1D-PAS-DKD-14/14-6.jpeg]

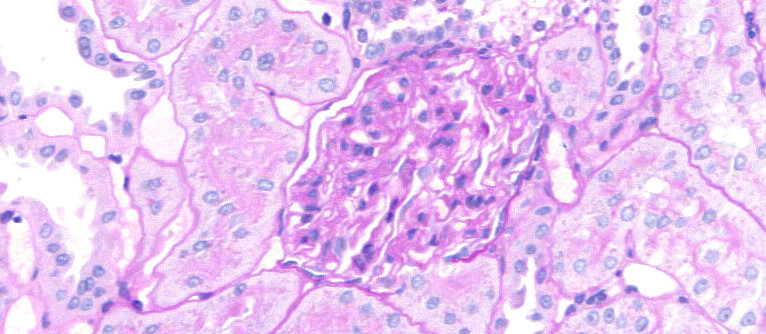

Supplement: Supplementary file 7 [file DataSheet13.ZIP › DKD/Fig 1D-PAS-DKD-14/14-7.jpeg]

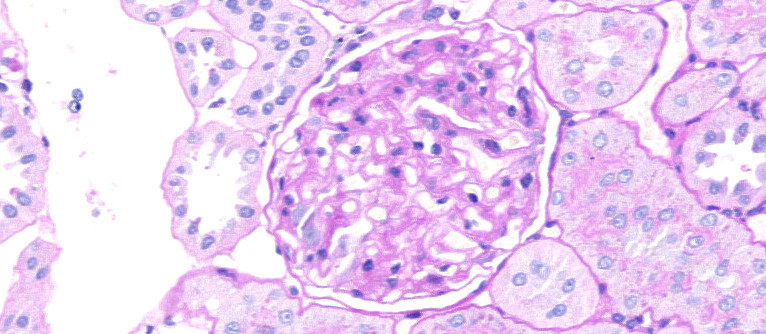

Supplement: Supplementary file 7 [file DataSheet13.ZIP › DKD/Fig 1D-PAS-DKD-14/14-8.jpeg]

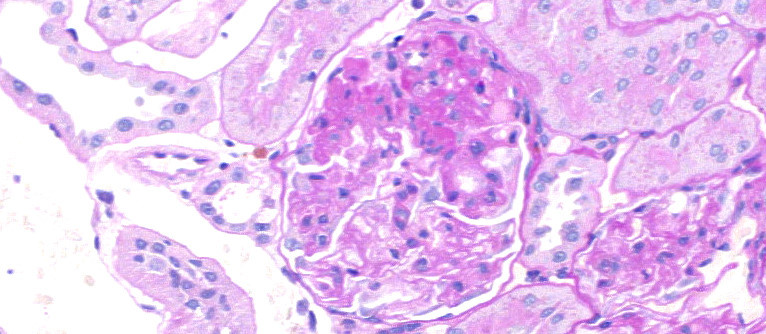

Supplement: Supplementary file 7 [file DataSheet13.ZIP › DKD/Fig 1D-PAS-DKD-14/14-9.jpeg]

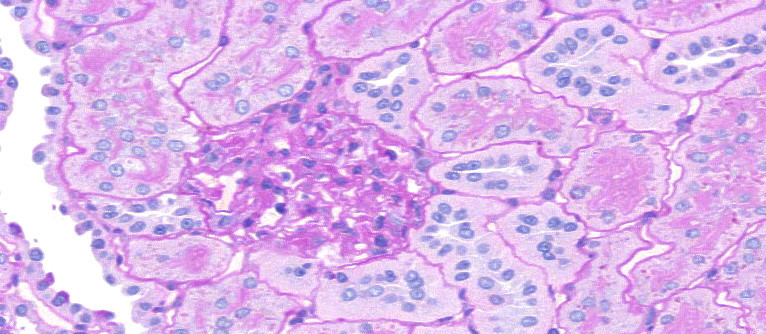

Supplement: Supplementary file 7 [file DataSheet13.ZIP › DKD/Fig 1D-PAS-DKD-15/15-1.jpeg]

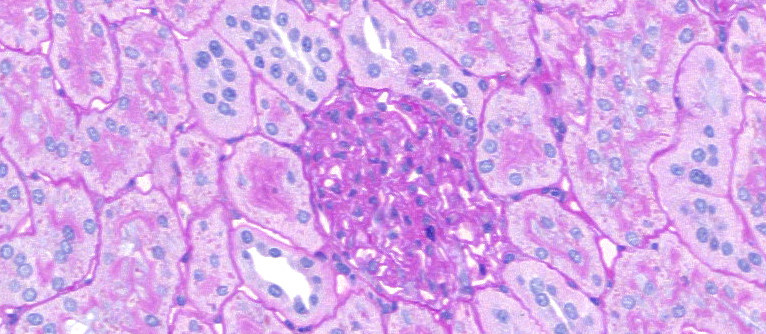

Supplement: Supplementary file 7 [file DataSheet13.ZIP › DKD/Fig 1D-PAS-DKD-15/15-10.jpeg]

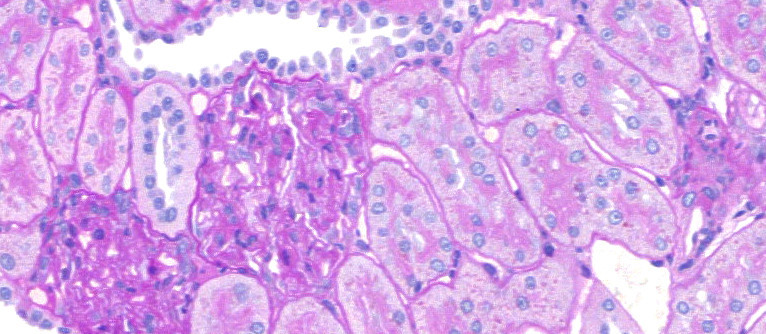

Supplement: Supplementary file 7 [file DataSheet13.ZIP › DKD/Fig 1D-PAS-DKD-15/15-11.jpeg]

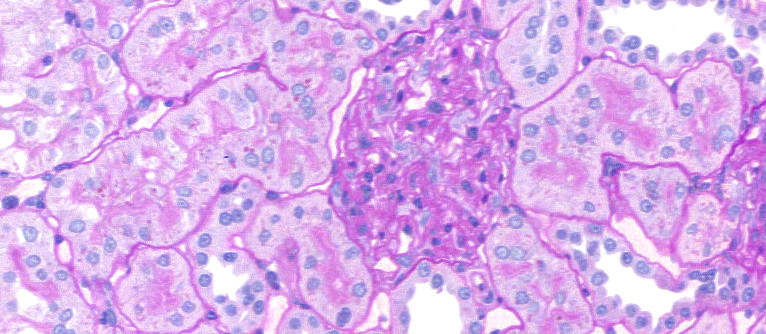

Supplement: Supplementary file 7 [file DataSheet13.ZIP › DKD/Fig 1D-PAS-DKD-15/15-12.jpeg]

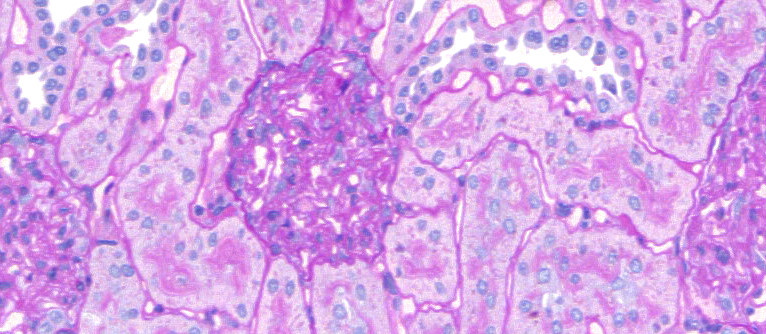

Supplement: Supplementary file 7 [file DataSheet13.ZIP › DKD/Fig 1D-PAS-DKD-15/15-13.jpeg]

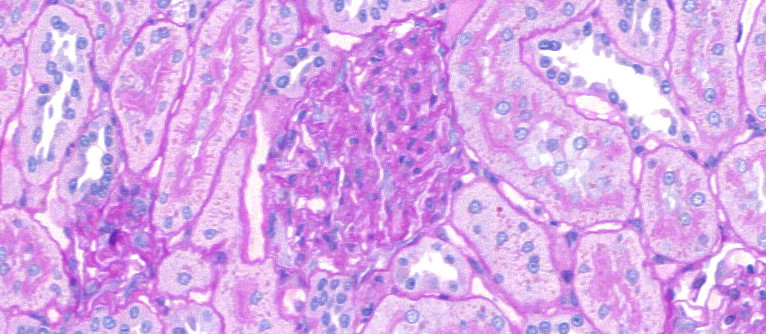

Supplement: Supplementary file 7 [file DataSheet13.ZIP › DKD/Fig 1D-PAS-DKD-15/15-14.jpeg]

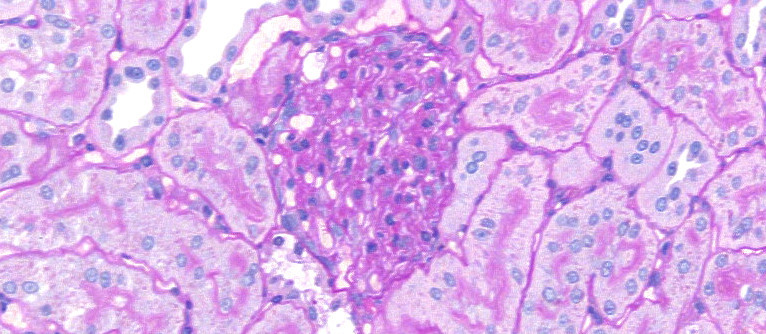

Supplement: Supplementary file 7 [file DataSheet13.ZIP › DKD/Fig 1D-PAS-DKD-15/15-15.jpeg]

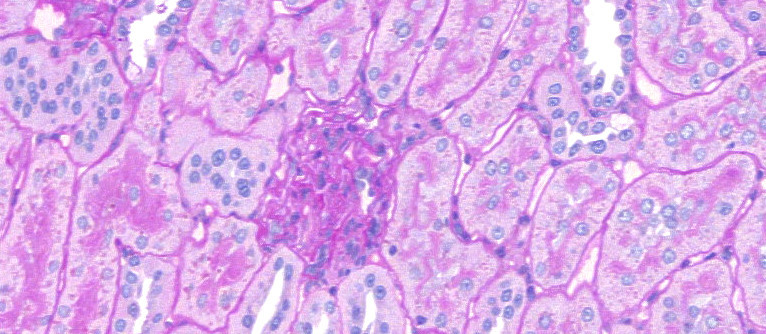

Supplement: Supplementary file 7 [file DataSheet13.ZIP › DKD/Fig 1D-PAS-DKD-15/15-16.jpeg]

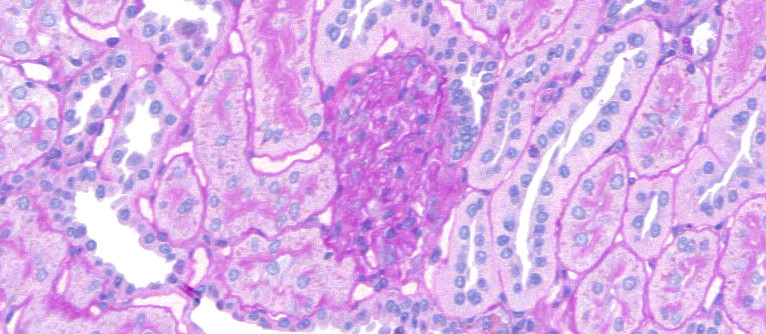

Supplement: Supplementary file 7 [file DataSheet13.ZIP › DKD/Fig 1D-PAS-DKD-15/15-17.jpeg]

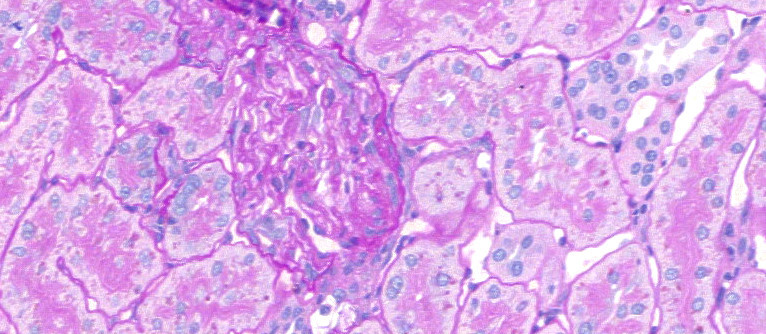

Supplement: Supplementary file 7 [file DataSheet13.ZIP › DKD/Fig 1D-PAS-DKD-15/15-18.jpeg]

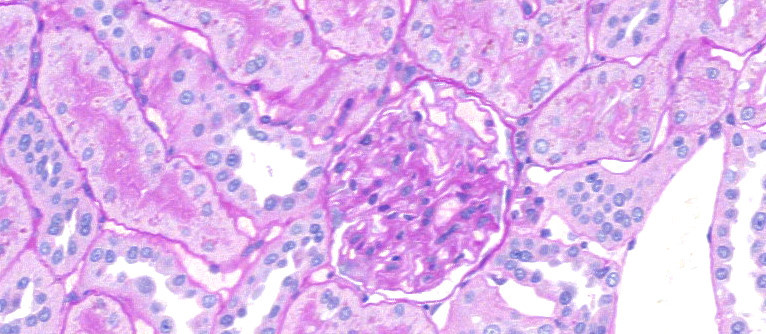

Supplement: Supplementary file 7 [file DataSheet13.ZIP › DKD/Fig 1D-PAS-DKD-15/15-19.jpeg]

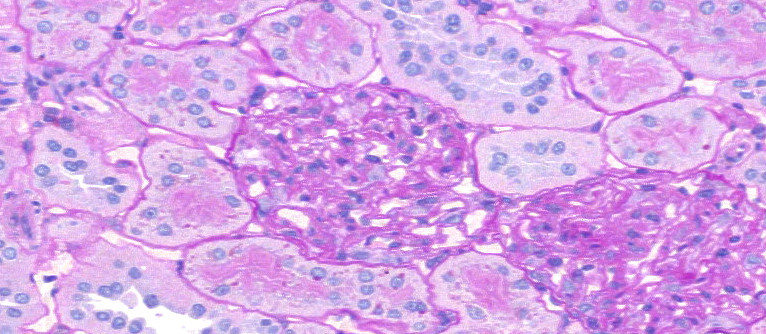

Supplement: Supplementary file 7 [file DataSheet13.ZIP › DKD/Fig 1D-PAS-DKD-15/15-2.jpeg]

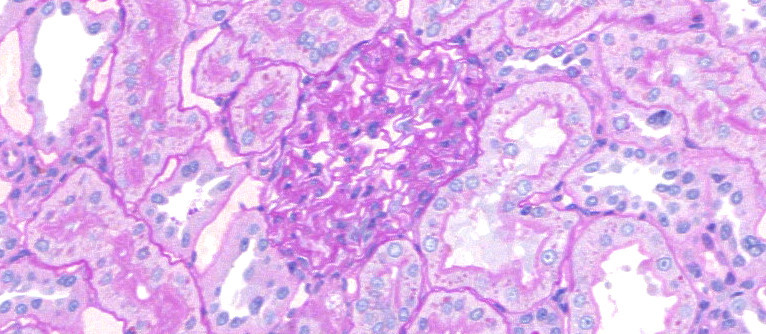

Supplement: Supplementary file 7 [file DataSheet13.ZIP › DKD/Fig 1D-PAS-DKD-15/15-20.jpeg]

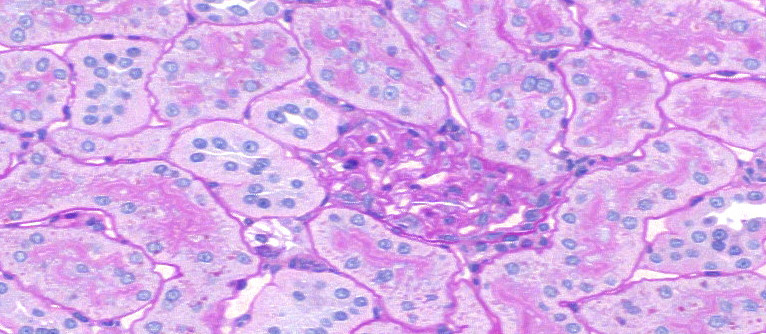

Supplement: Supplementary file 7 [file DataSheet13.ZIP › DKD/Fig 1D-PAS-DKD-15/15-3.jpeg]

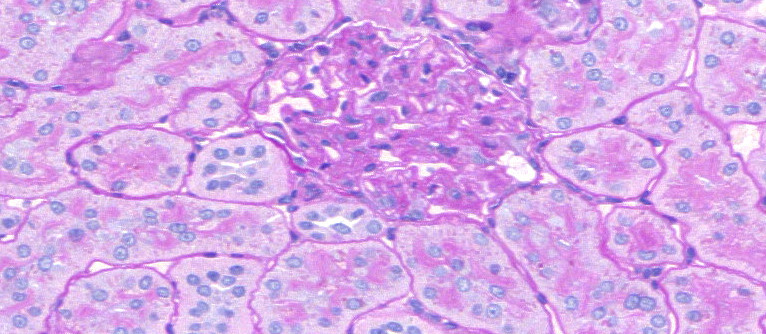

Supplement: Supplementary file 7 [file DataSheet13.ZIP › DKD/Fig 1D-PAS-DKD-15/15-4.jpeg]

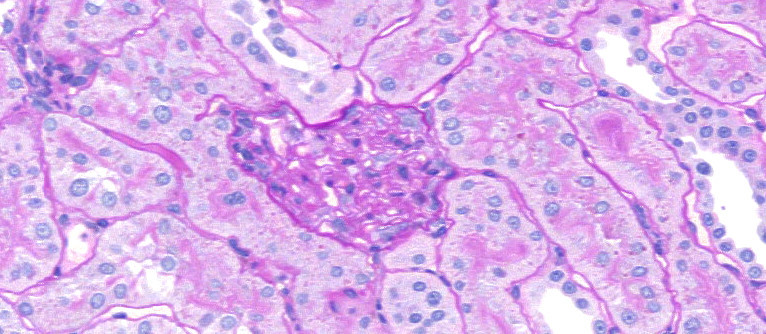

Supplement: Supplementary file 7 [file DataSheet13.ZIP › DKD/Fig 1D-PAS-DKD-15/15-5.jpeg]

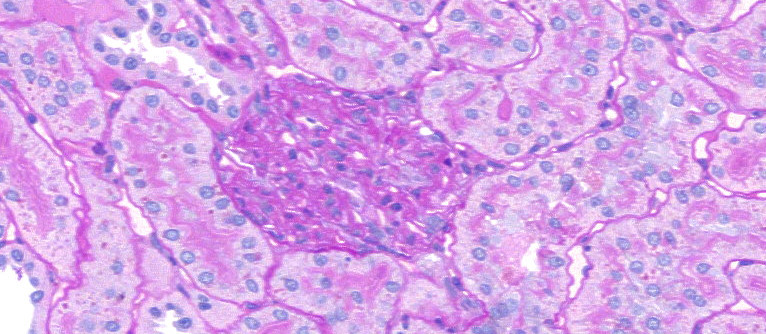

Supplement: Supplementary file 7 [file DataSheet13.ZIP › DKD/Fig 1D-PAS-DKD-15/15-6.jpeg]

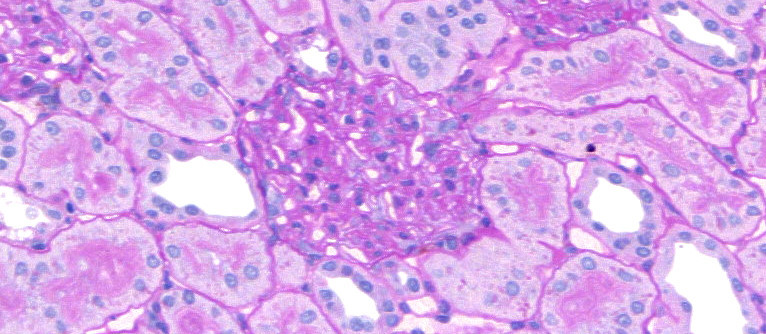

Supplement: Supplementary file 7 [file DataSheet13.ZIP › DKD/Fig 1D-PAS-DKD-15/15-7.jpeg]

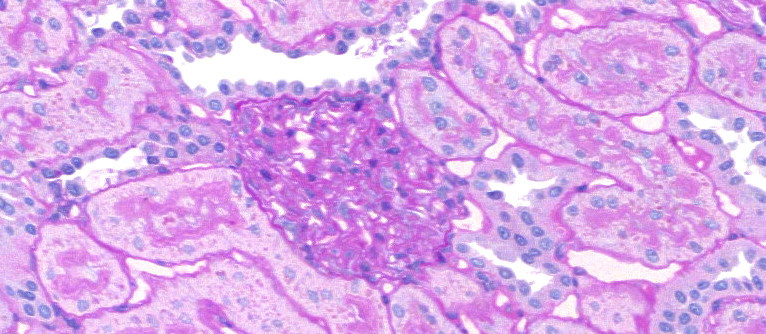

Supplement: Supplementary file 7 [file DataSheet13.ZIP › DKD/Fig 1D-PAS-DKD-15/15-8.jpeg]

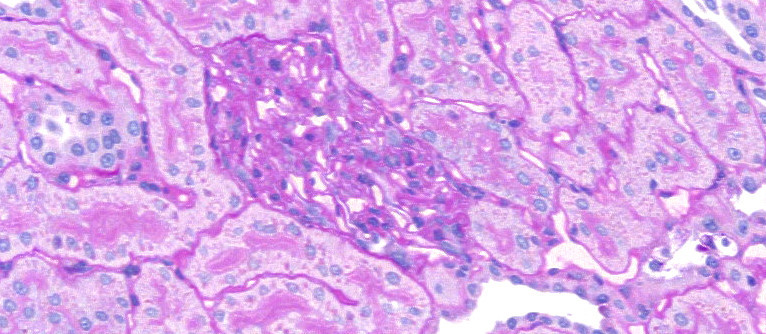

Supplement: Supplementary file 7 [file DataSheet13.ZIP › DKD/Fig 1D-PAS-DKD-15/15-9.jpeg]

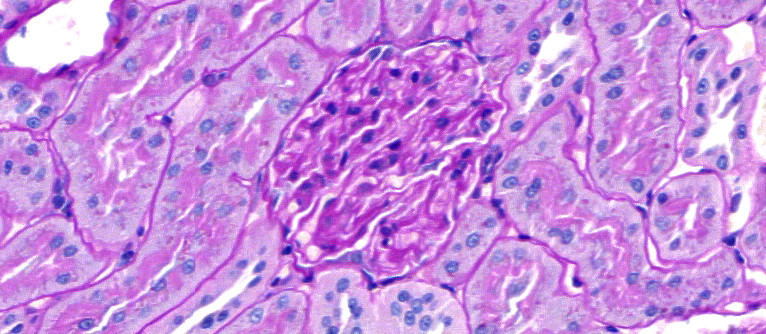

Supplement: Supplementary file 7 [file DataSheet13.ZIP › DKD/Fig 1D-PAS-DKD-17/17-1.jpeg]
