# Supplementary figures and images for: Characteristics of Serum Metabolites and Gut Microbiota in Diabetic Kidney Disease (part 7 of 13)
Source: Front Pharmacol. 2022 Apr 14;13:872988. doi: 10.3389/fphar.2022.872988 (PMC9084235; doi:10.3389/fphar.2022.872988)

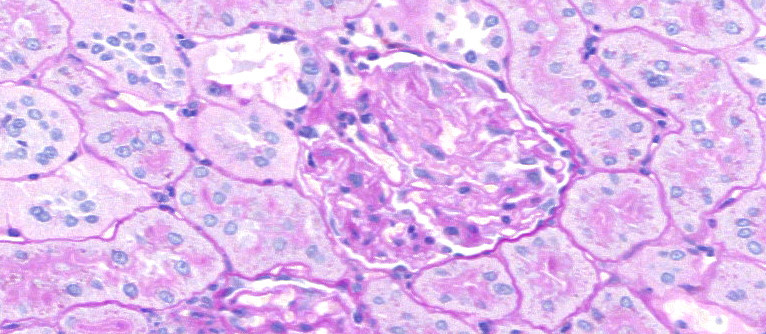

Supplement: Supplementary file 7 [file DataSheet13.ZIP › DKD/Fig 1D-PAS-DKD-23/23-10.jpeg]

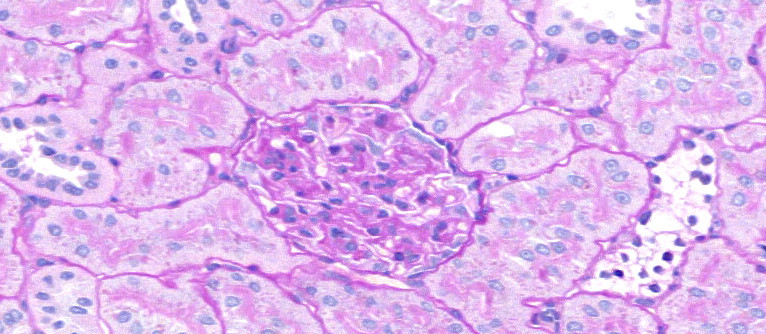

Supplement: Supplementary file 7 [file DataSheet13.ZIP › DKD/Fig 1D-PAS-DKD-23/23-11.jpeg]

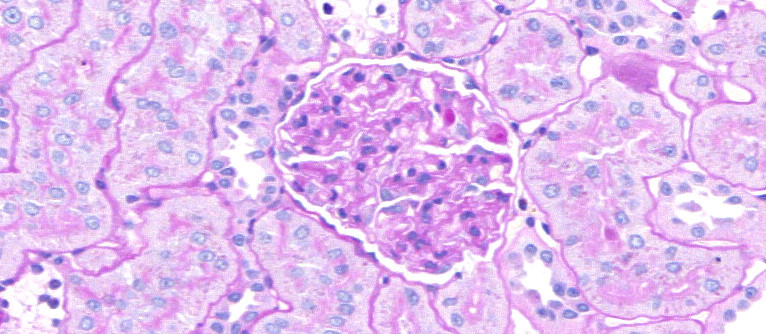

Supplement: Supplementary file 7 [file DataSheet13.ZIP › DKD/Fig 1D-PAS-DKD-23/23-12.jpeg]

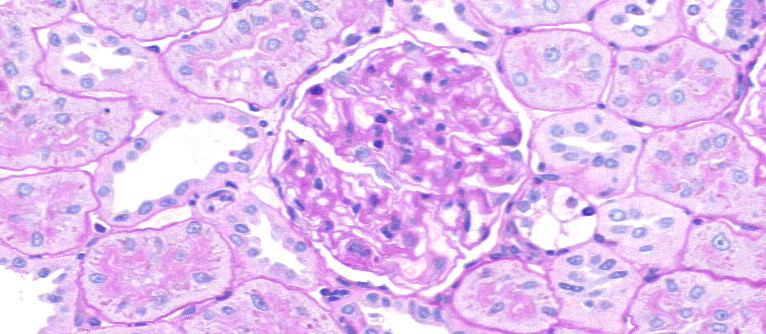

Supplement: Supplementary file 7 [file DataSheet13.ZIP › DKD/Fig 1D-PAS-DKD-23/23-13.jpeg]

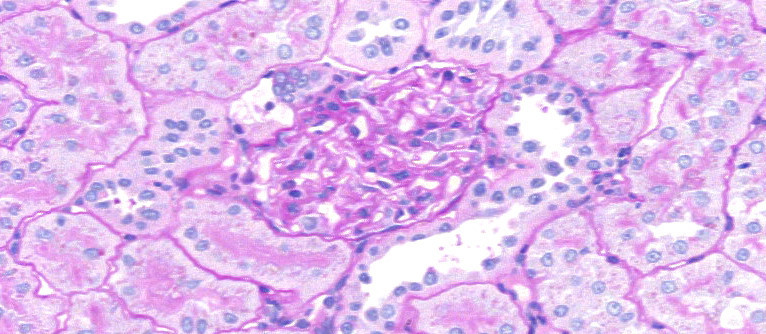

Supplement: Supplementary file 7 [file DataSheet13.ZIP › DKD/Fig 1D-PAS-DKD-23/23-14.jpeg]

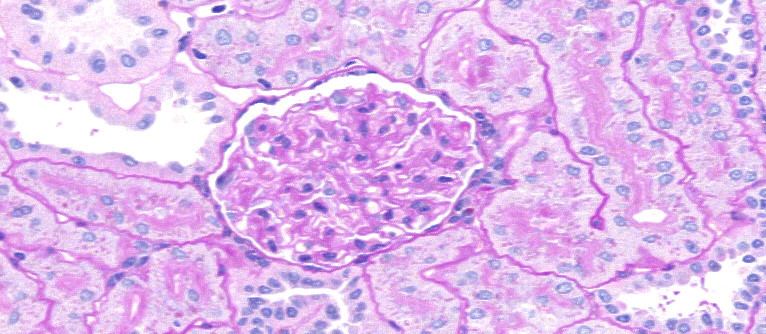

Supplement: Supplementary file 7 [file DataSheet13.ZIP › DKD/Fig 1D-PAS-DKD-23/23-15.jpeg]

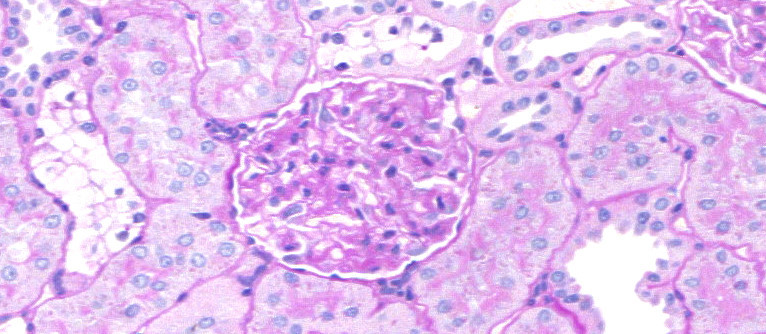

Supplement: Supplementary file 7 [file DataSheet13.ZIP › DKD/Fig 1D-PAS-DKD-23/23-16.jpeg]

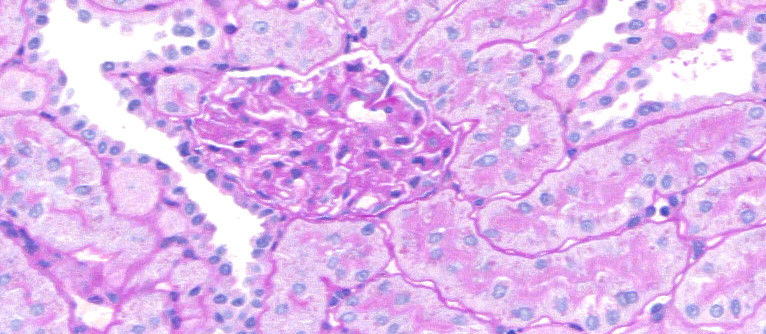

Supplement: Supplementary file 7 [file DataSheet13.ZIP › DKD/Fig 1D-PAS-DKD-23/23-17.jpeg]

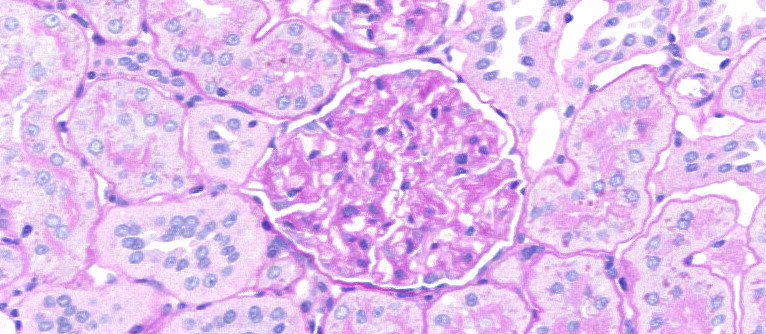

Supplement: Supplementary file 7 [file DataSheet13.ZIP › DKD/Fig 1D-PAS-DKD-23/23-18.jpeg]

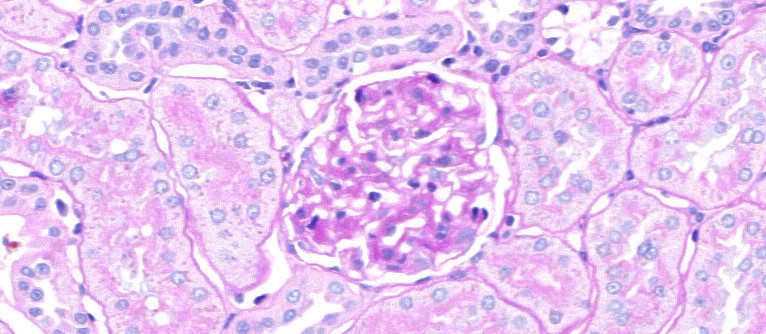

Supplement: Supplementary file 7 [file DataSheet13.ZIP › DKD/Fig 1D-PAS-DKD-23/23-19.jpeg]

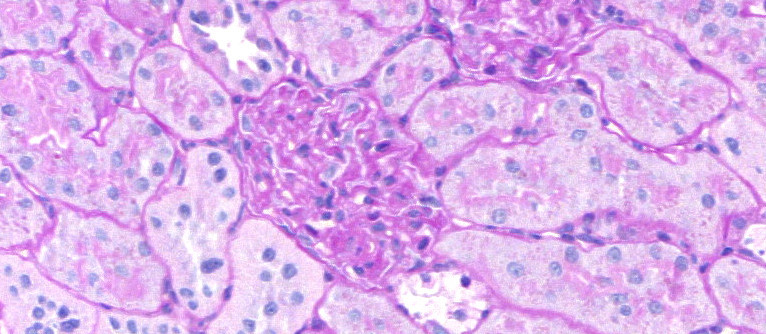

Supplement: Supplementary file 7 [file DataSheet13.ZIP › DKD/Fig 1D-PAS-DKD-23/23-2.jpeg]

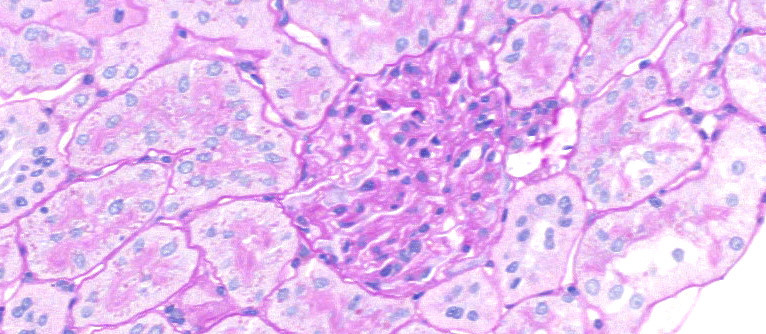

Supplement: Supplementary file 7 [file DataSheet13.ZIP › DKD/Fig 1D-PAS-DKD-23/23-20.jpeg]

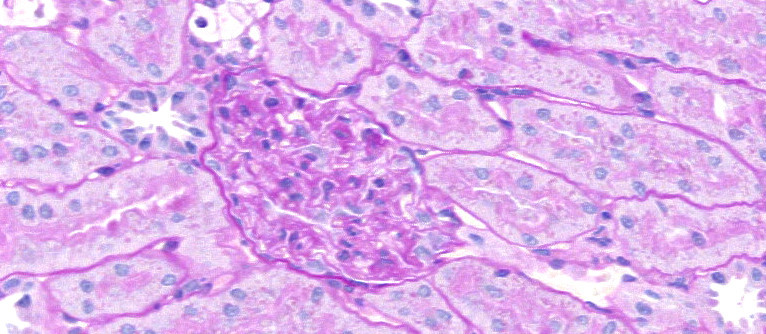

Supplement: Supplementary file 7 [file DataSheet13.ZIP › DKD/Fig 1D-PAS-DKD-23/23-3.jpeg]

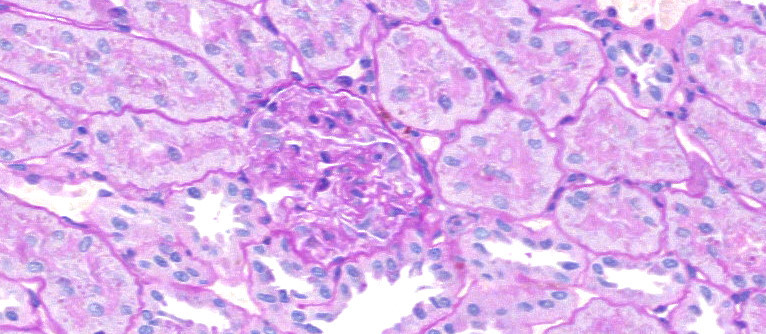

Supplement: Supplementary file 7 [file DataSheet13.ZIP › DKD/Fig 1D-PAS-DKD-23/23-4.jpeg]

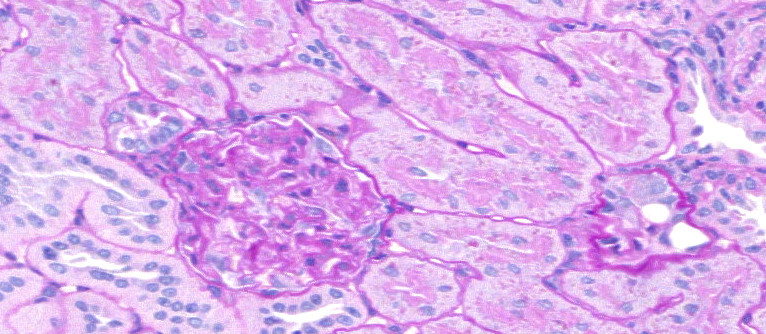

Supplement: Supplementary file 7 [file DataSheet13.ZIP › DKD/Fig 1D-PAS-DKD-23/23-5.jpeg]

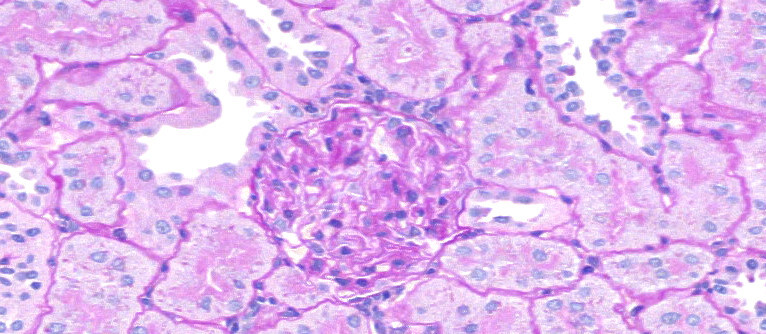

Supplement: Supplementary file 7 [file DataSheet13.ZIP › DKD/Fig 1D-PAS-DKD-23/23-6.jpeg]

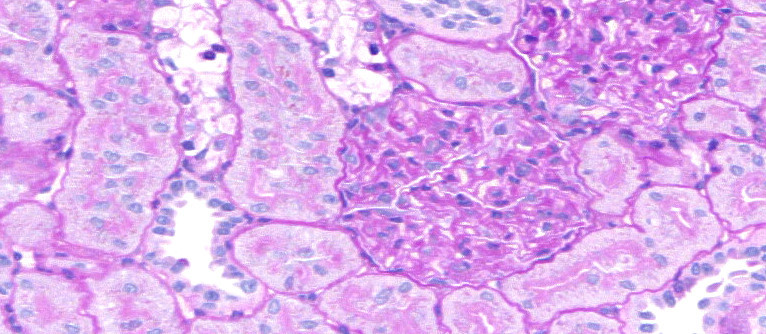

Supplement: Supplementary file 7 [file DataSheet13.ZIP › DKD/Fig 1D-PAS-DKD-23/23-7.jpeg]

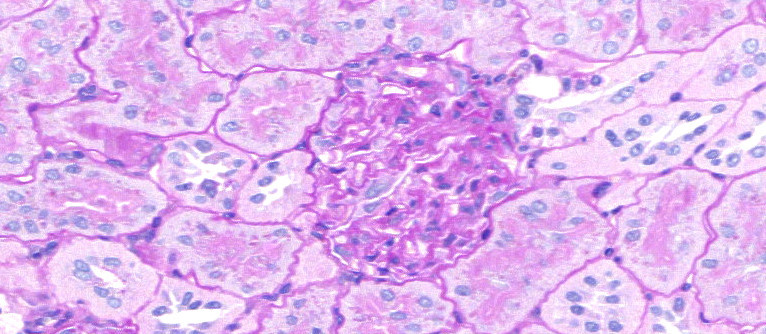

Supplement: Supplementary file 7 [file DataSheet13.ZIP › DKD/Fig 1D-PAS-DKD-23/23-8.jpeg]

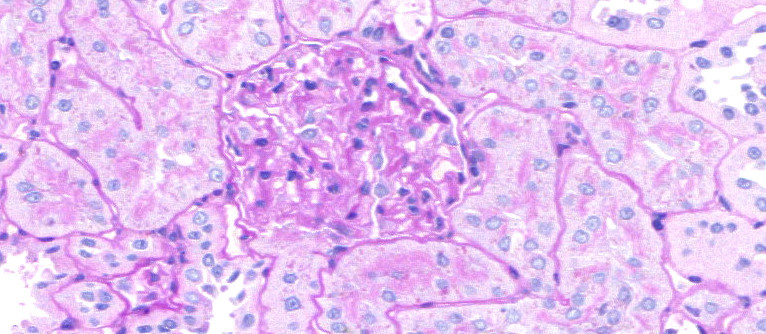

Supplement: Supplementary file 7 [file DataSheet13.ZIP › DKD/Fig 1D-PAS-DKD-23/23-9.jpeg]

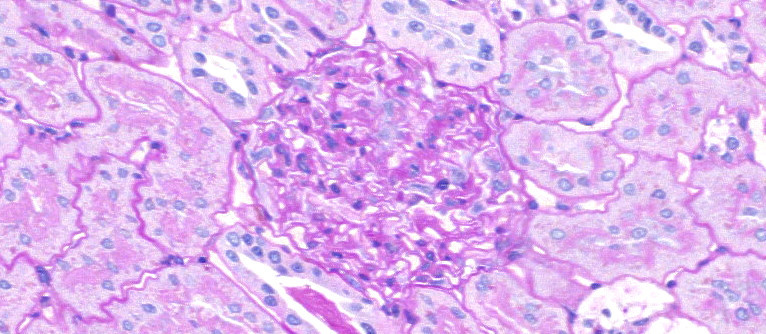

Supplement: Supplementary file 7 [file DataSheet13.ZIP › DKD/Fig 1D-PAS-DKD-24/24-1.jpeg]

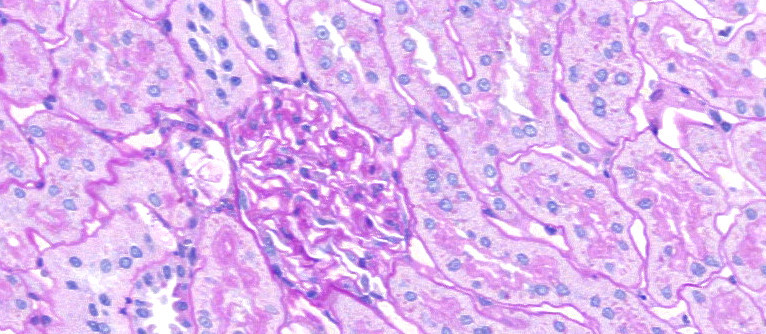

Supplement: Supplementary file 7 [file DataSheet13.ZIP › DKD/Fig 1D-PAS-DKD-24/24-10.jpeg]

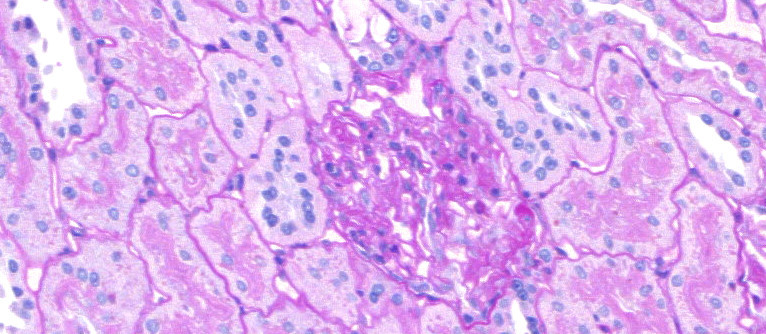

Supplement: Supplementary file 7 [file DataSheet13.ZIP › DKD/Fig 1D-PAS-DKD-24/24-11.jpeg]

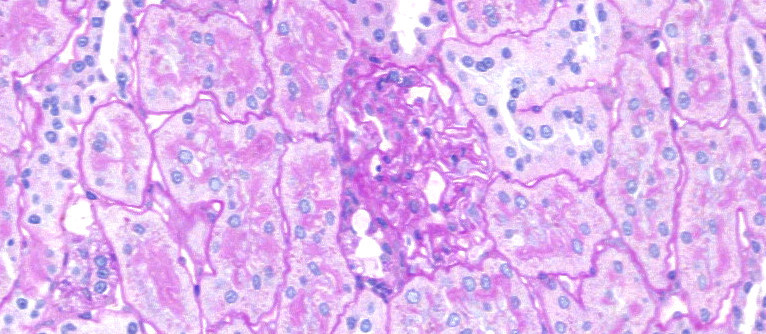

Supplement: Supplementary file 7 [file DataSheet13.ZIP › DKD/Fig 1D-PAS-DKD-24/24-12.jpeg]

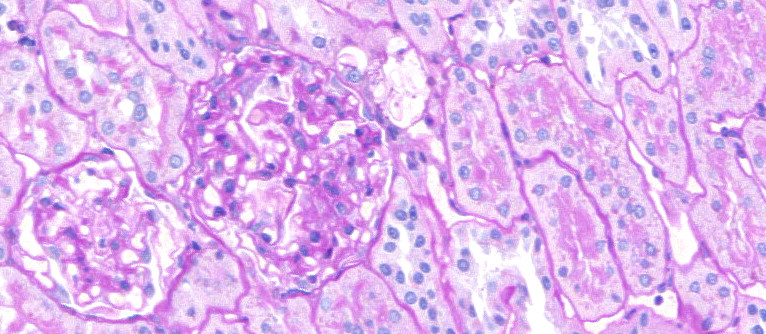

Supplement: Supplementary file 7 [file DataSheet13.ZIP › DKD/Fig 1D-PAS-DKD-24/24-13.jpeg]

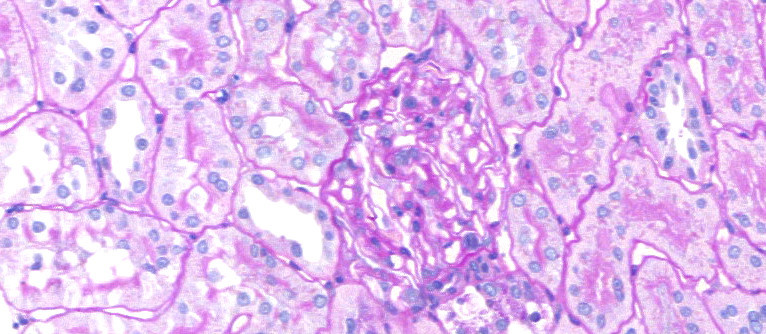

Supplement: Supplementary file 7 [file DataSheet13.ZIP › DKD/Fig 1D-PAS-DKD-24/24-14.jpeg]

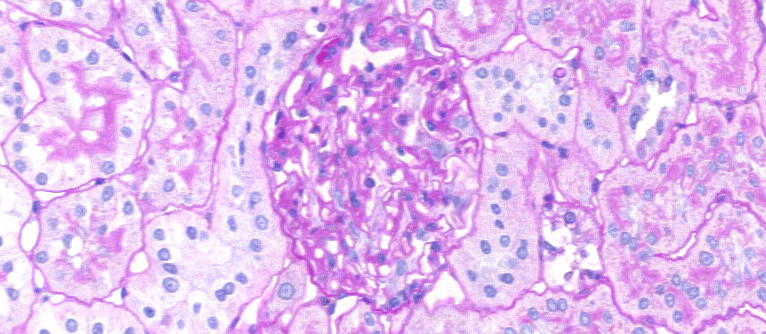

Supplement: Supplementary file 7 [file DataSheet13.ZIP › DKD/Fig 1D-PAS-DKD-24/24-15.jpeg]

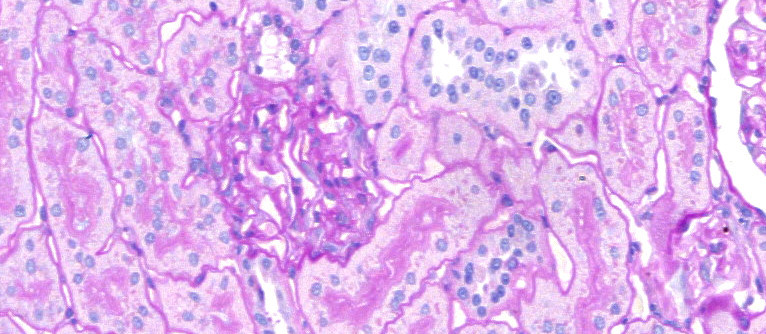

Supplement: Supplementary file 7 [file DataSheet13.ZIP › DKD/Fig 1D-PAS-DKD-24/24-16.jpeg]

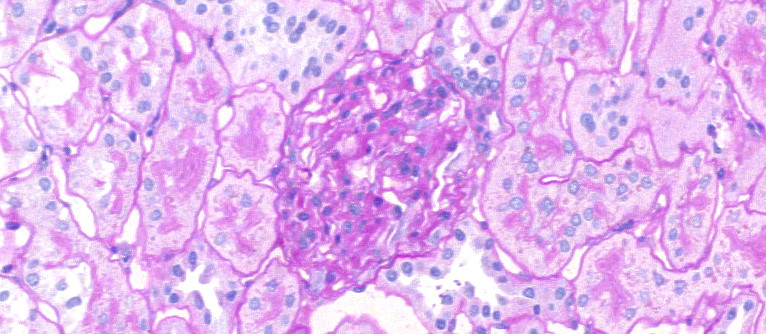

Supplement: Supplementary file 7 [file DataSheet13.ZIP › DKD/Fig 1D-PAS-DKD-24/24-17.jpeg]

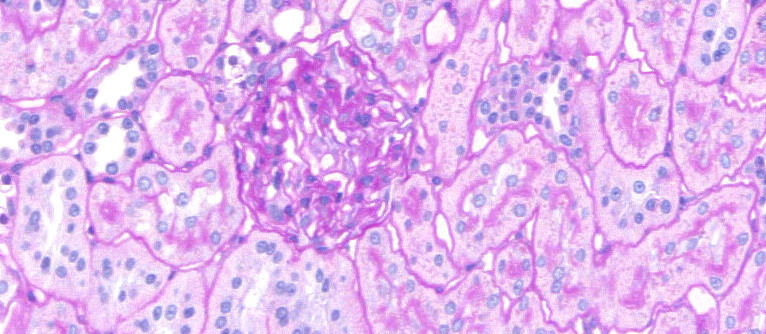

Supplement: Supplementary file 7 [file DataSheet13.ZIP › DKD/Fig 1D-PAS-DKD-24/24-18.jpeg]

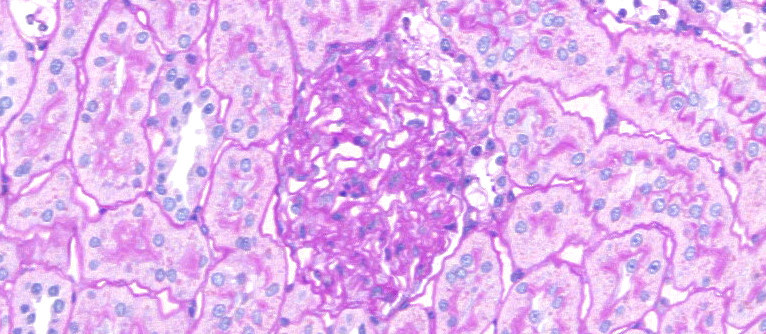

Supplement: Supplementary file 7 [file DataSheet13.ZIP › DKD/Fig 1D-PAS-DKD-24/24-19.jpeg]

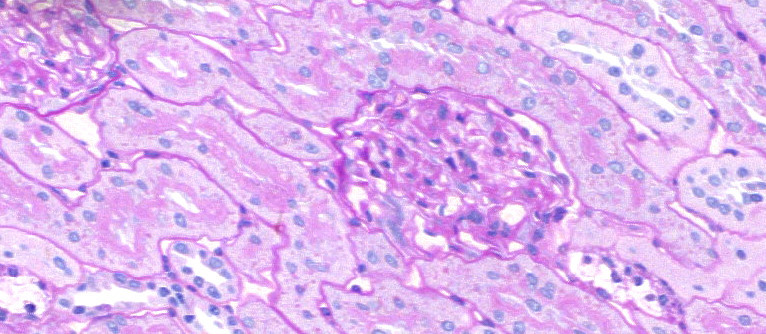

Supplement: Supplementary file 7 [file DataSheet13.ZIP › DKD/Fig 1D-PAS-DKD-24/24-2.jpeg]

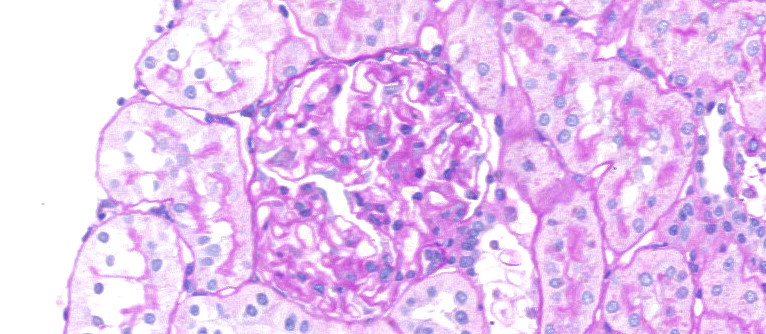

Supplement: Supplementary file 7 [file DataSheet13.ZIP › DKD/Fig 1D-PAS-DKD-24/24-20.jpeg]

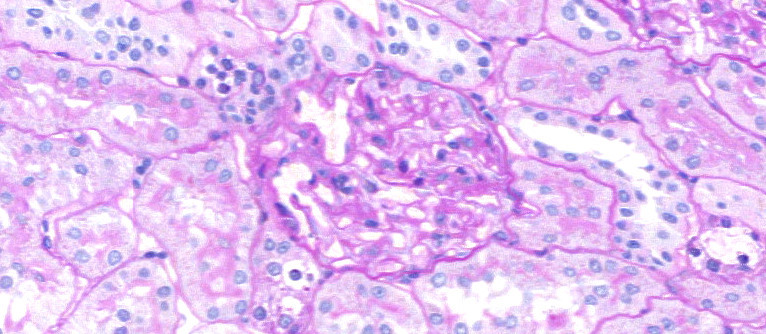

Supplement: Supplementary file 7 [file DataSheet13.ZIP › DKD/Fig 1D-PAS-DKD-24/24-3.jpeg]

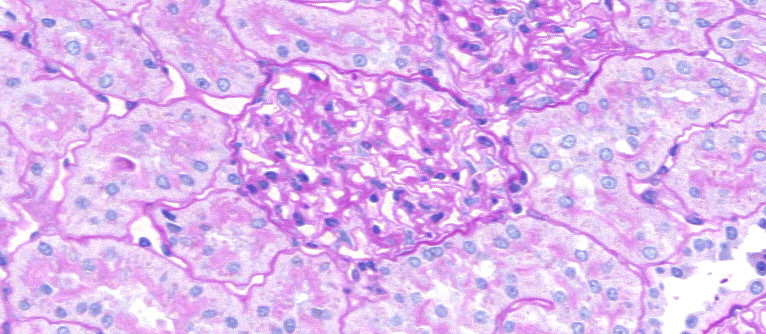

Supplement: Supplementary file 7 [file DataSheet13.ZIP › DKD/Fig 1D-PAS-DKD-24/24-4.jpeg]

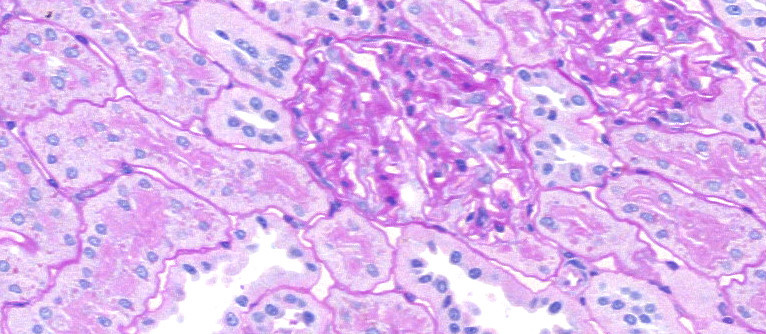

Supplement: Supplementary file 7 [file DataSheet13.ZIP › DKD/Fig 1D-PAS-DKD-24/24-5.jpeg]

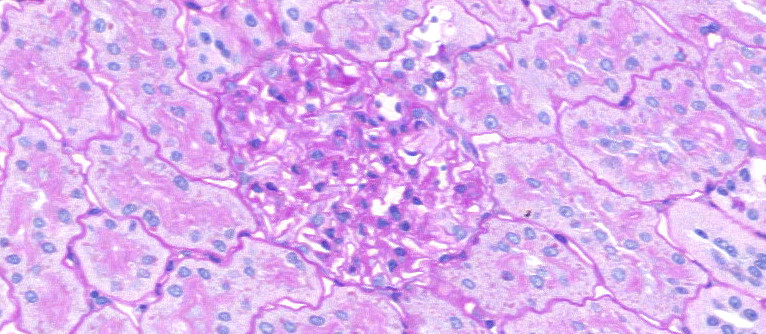

Supplement: Supplementary file 7 [file DataSheet13.ZIP › DKD/Fig 1D-PAS-DKD-24/24-6.jpeg]

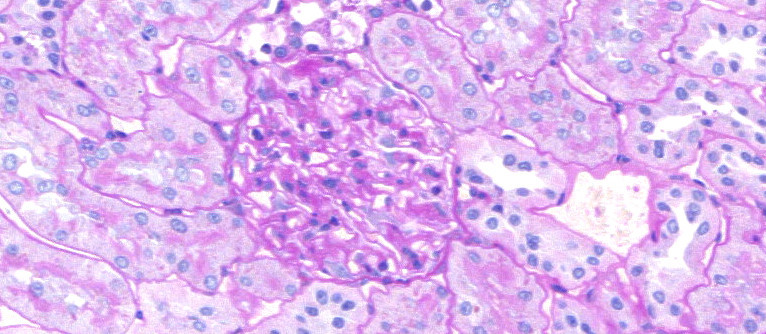

Supplement: Supplementary file 7 [file DataSheet13.ZIP › DKD/Fig 1D-PAS-DKD-24/24-7.jpeg]

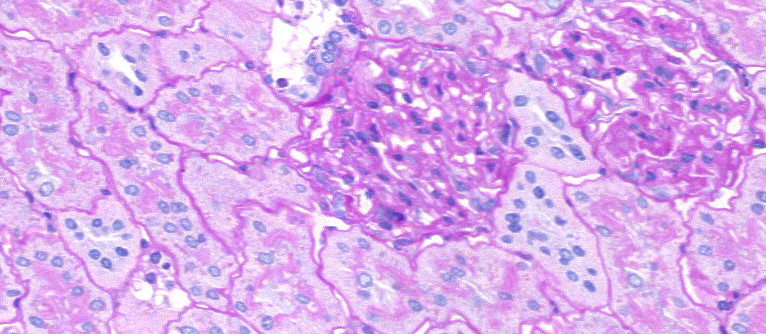

Supplement: Supplementary file 7 [file DataSheet13.ZIP › DKD/Fig 1D-PAS-DKD-24/24-8.jpeg]

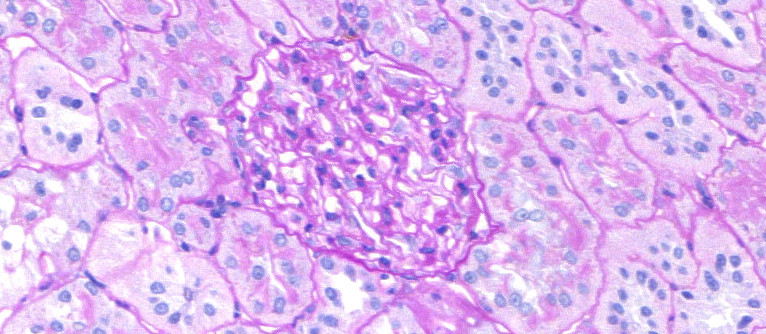

Supplement: Supplementary file 7 [file DataSheet13.ZIP › DKD/Fig 1D-PAS-DKD-24/24-9.jpeg]

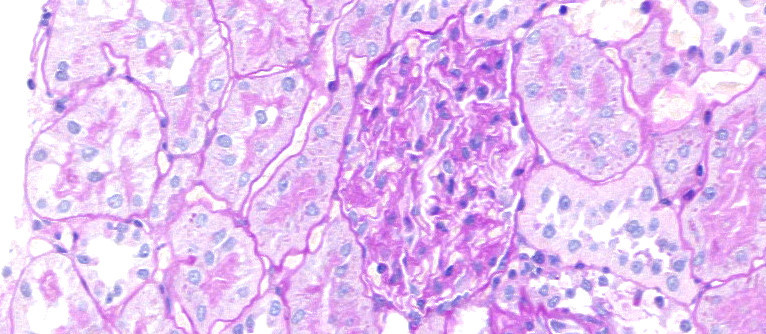

Supplement: Supplementary file 7 [file DataSheet13.ZIP › DKD/Fig 1D-PAS-DKD-25/25-1.jpeg]

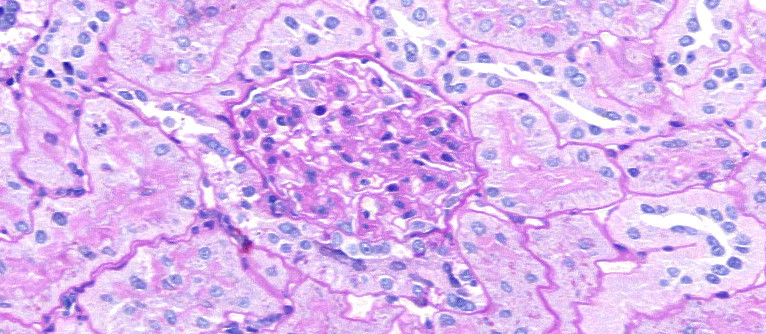

Supplement: Supplementary file 7 [file DataSheet13.ZIP › DKD/Fig 1D-PAS-DKD-25/25-10.jpeg]

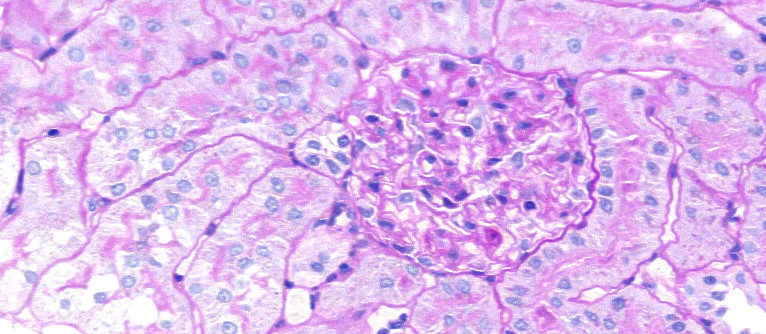

Supplement: Supplementary file 7 [file DataSheet13.ZIP › DKD/Fig 1D-PAS-DKD-25/25-11.jpeg]

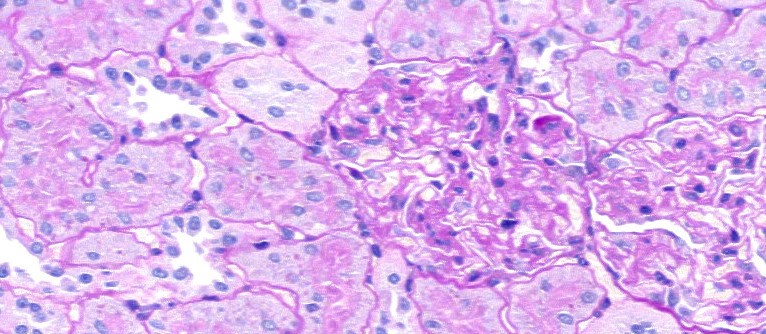

Supplement: Supplementary file 7 [file DataSheet13.ZIP › DKD/Fig 1D-PAS-DKD-25/25-12.jpeg]

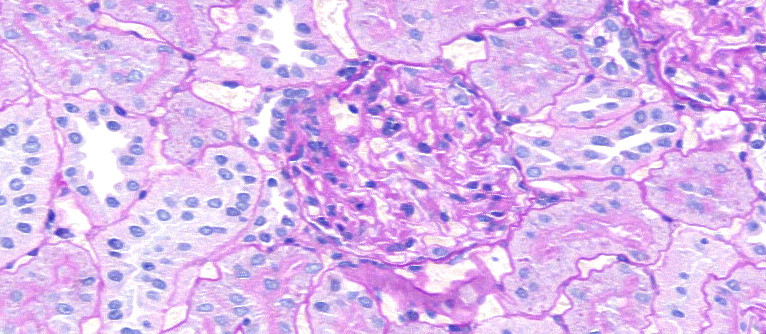

Supplement: Supplementary file 7 [file DataSheet13.ZIP › DKD/Fig 1D-PAS-DKD-25/25-13.jpeg]

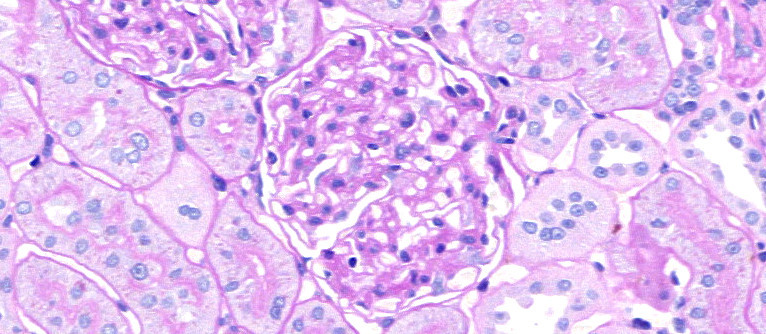

Supplement: Supplementary file 7 [file DataSheet13.ZIP › DKD/Fig 1D-PAS-DKD-25/25-14.jpeg]

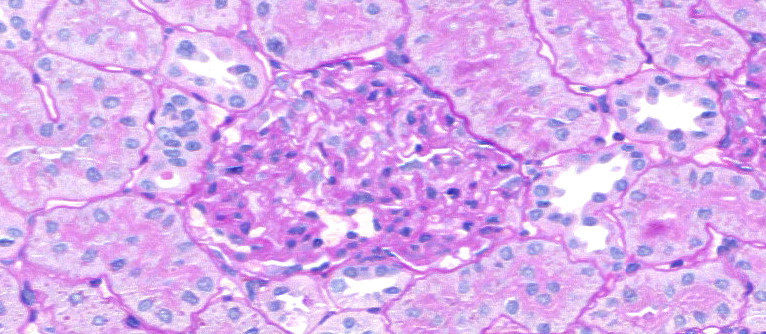

Supplement: Supplementary file 7 [file DataSheet13.ZIP › DKD/Fig 1D-PAS-DKD-25/25-15.jpeg]

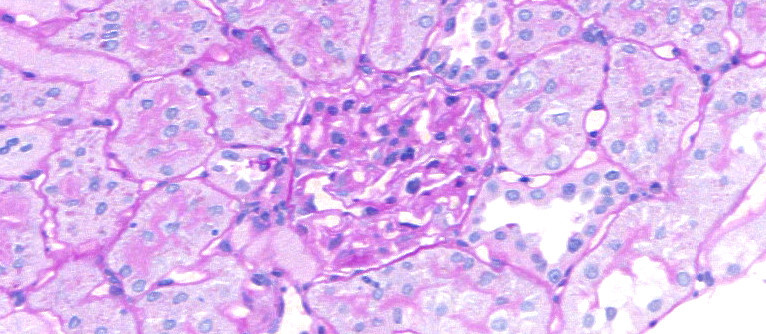

Supplement: Supplementary file 7 [file DataSheet13.ZIP › DKD/Fig 1D-PAS-DKD-25/25-16.jpeg]

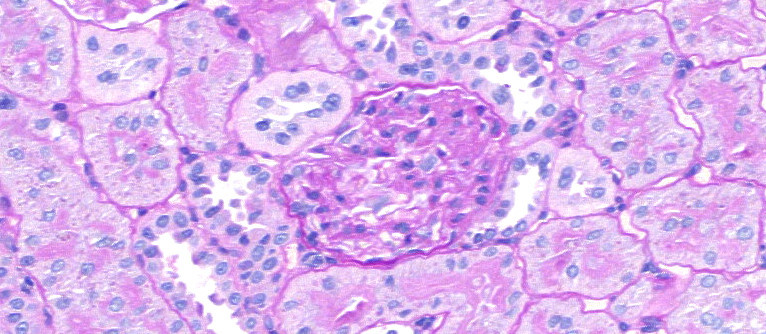

Supplement: Supplementary file 7 [file DataSheet13.ZIP › DKD/Fig 1D-PAS-DKD-25/25-17.jpeg]

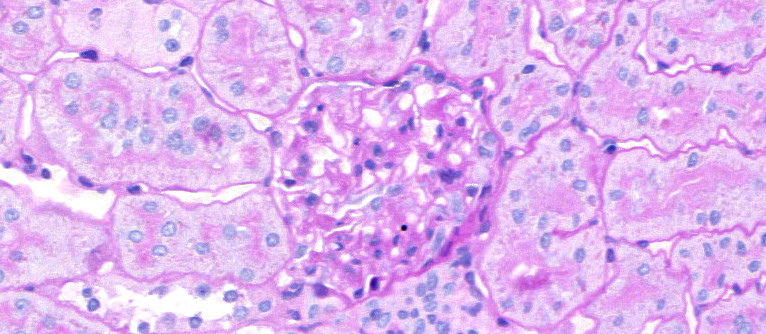

Supplement: Supplementary file 7 [file DataSheet13.ZIP › DKD/Fig 1D-PAS-DKD-25/25-18.jpeg]

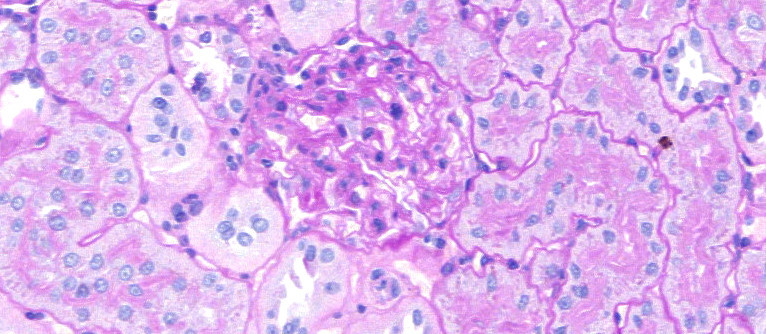

Supplement: Supplementary file 7 [file DataSheet13.ZIP › DKD/Fig 1D-PAS-DKD-25/25-19.jpeg]

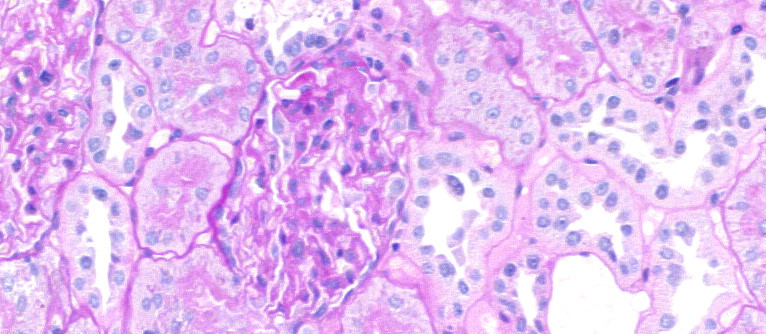

Supplement: Supplementary file 7 [file DataSheet13.ZIP › DKD/Fig 1D-PAS-DKD-25/25-2.jpeg]

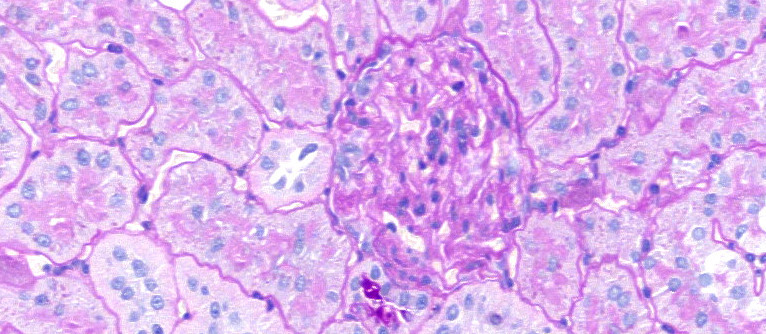

Supplement: Supplementary file 7 [file DataSheet13.ZIP › DKD/Fig 1D-PAS-DKD-25/25-20.jpeg]

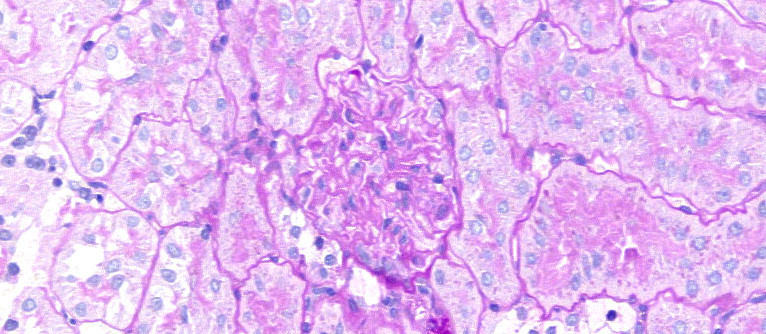

Supplement: Supplementary file 7 [file DataSheet13.ZIP › DKD/Fig 1D-PAS-DKD-25/25-3.jpeg]

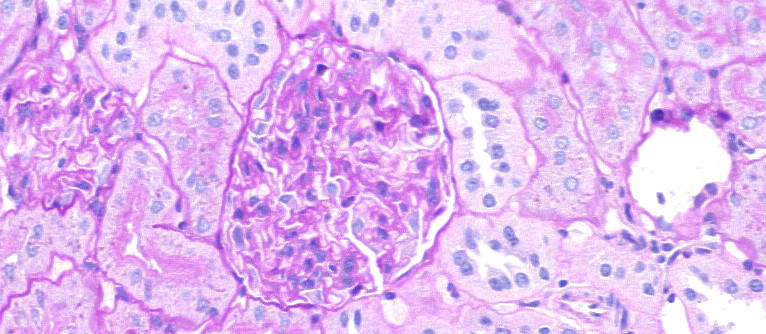

Supplement: Supplementary file 7 [file DataSheet13.ZIP › DKD/Fig 1D-PAS-DKD-25/25-4.jpeg]

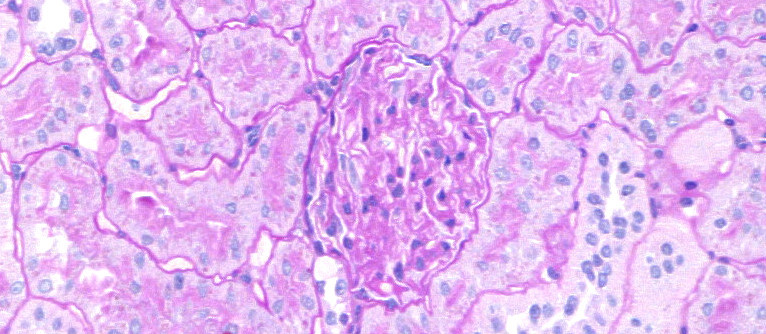

Supplement: Supplementary file 7 [file DataSheet13.ZIP › DKD/Fig 1D-PAS-DKD-25/25-5.jpeg]

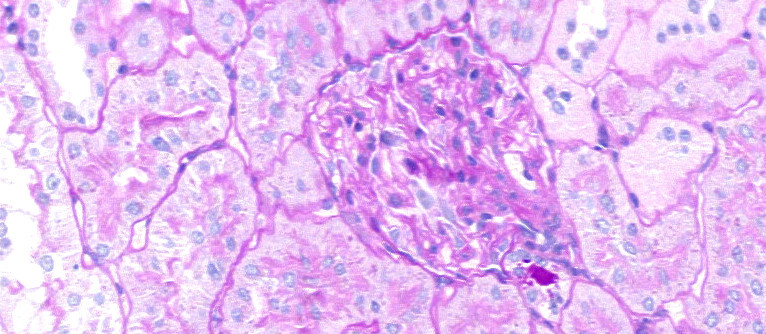

Supplement: Supplementary file 7 [file DataSheet13.ZIP › DKD/Fig 1D-PAS-DKD-25/25-6.jpeg]

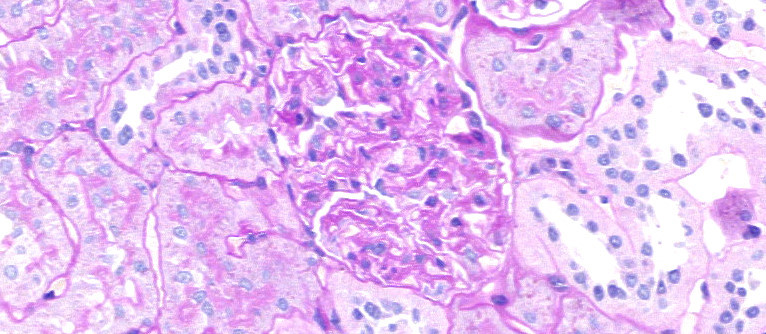

Supplement: Supplementary file 7 [file DataSheet13.ZIP › DKD/Fig 1D-PAS-DKD-25/25-7.jpeg]

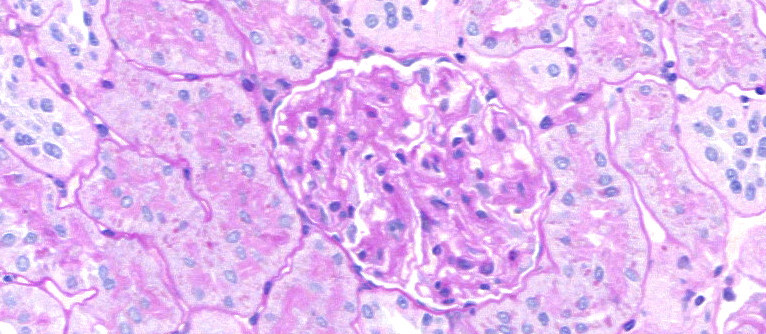

Supplement: Supplementary file 7 [file DataSheet13.ZIP › DKD/Fig 1D-PAS-DKD-25/25-8.jpeg]

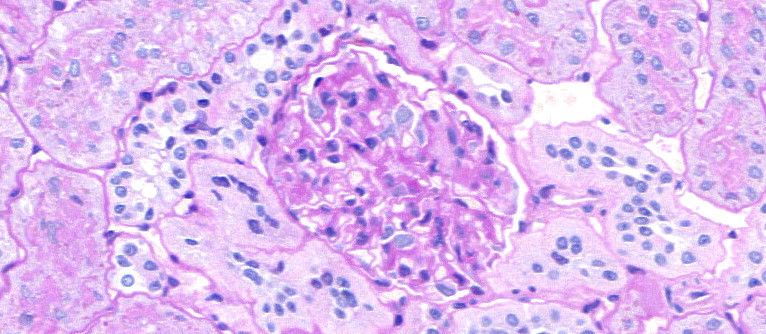

Supplement: Supplementary file 7 [file DataSheet13.ZIP › DKD/Fig 1D-PAS-DKD-25/25-9.jpeg]

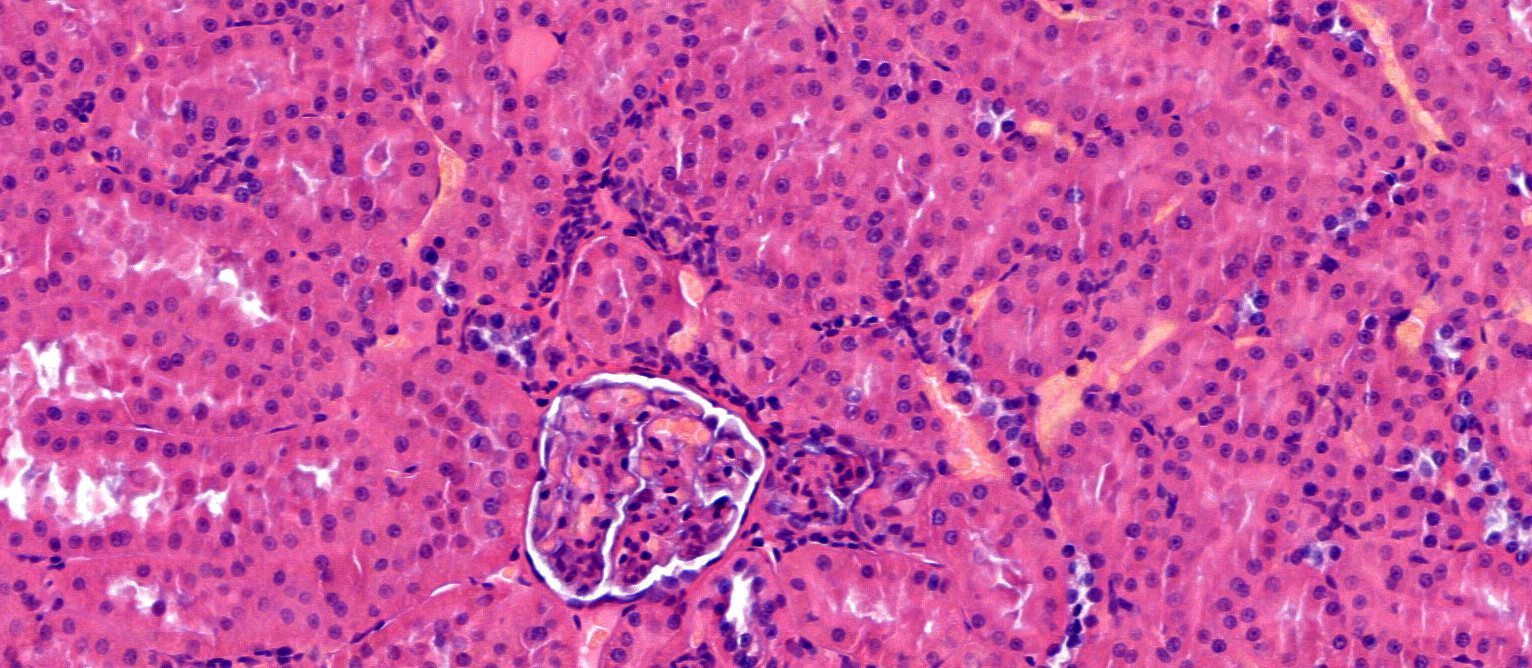

Supplement: Supplementary file 8 [file DataSheet1.ZIP › Fig 1D-HE-sham-1/1-1.jpeg]

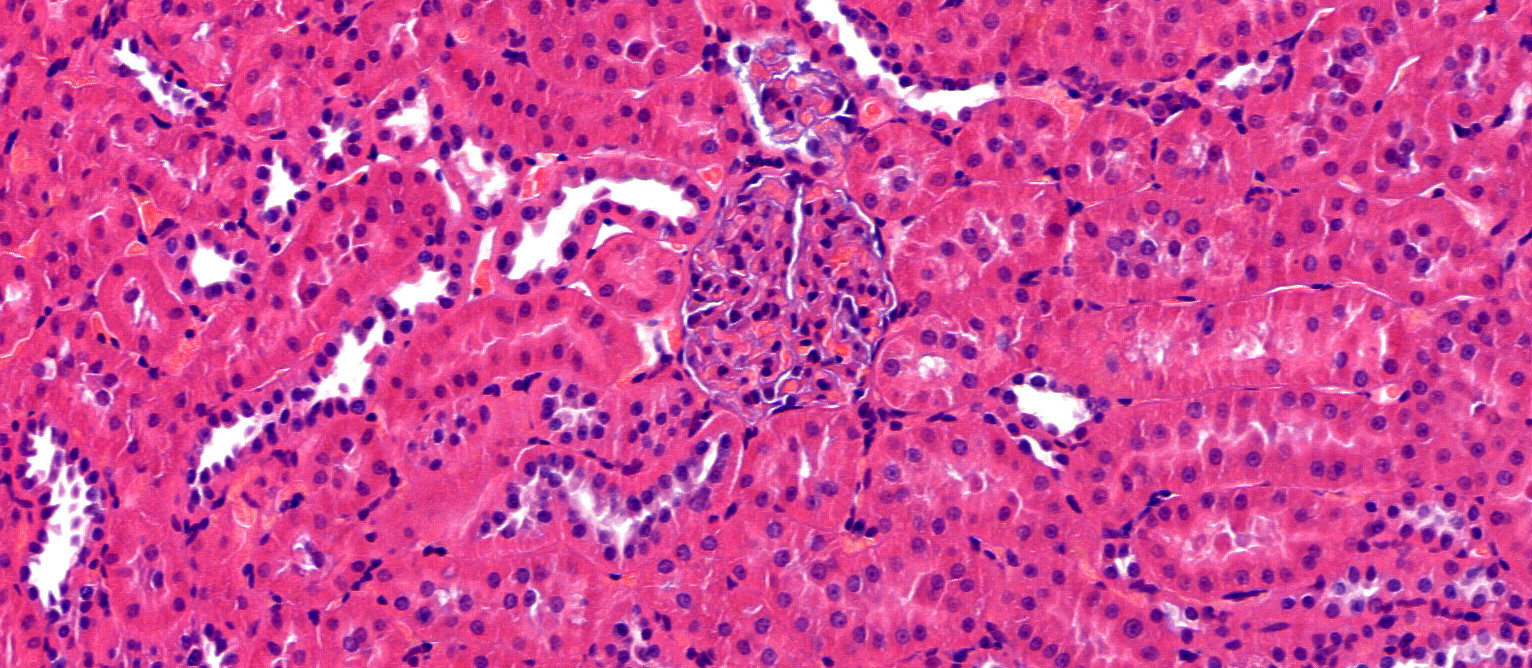

Supplement: Supplementary file 8 [file DataSheet1.ZIP › Fig 1D-HE-sham-1/1-10.jpeg]

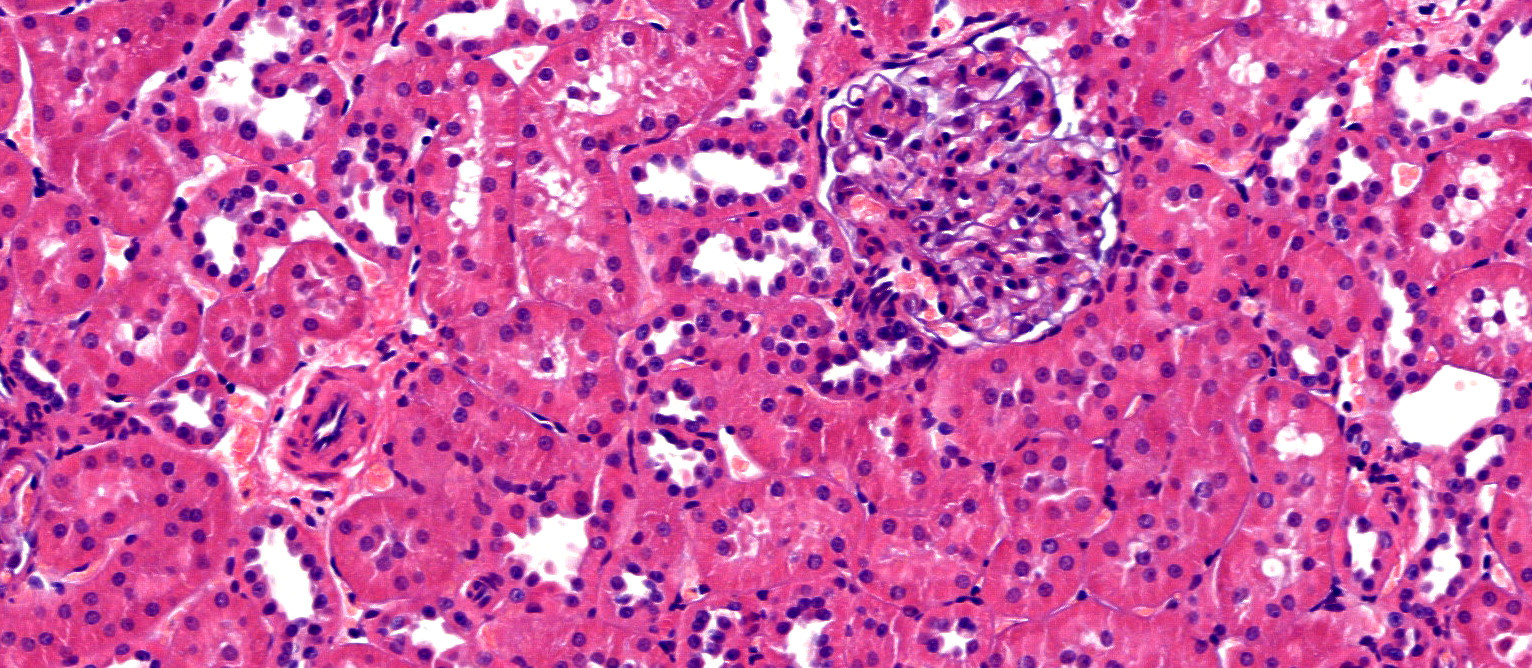

Supplement: Supplementary file 8 [file DataSheet1.ZIP › Fig 1D-HE-sham-1/1-2.jpeg]

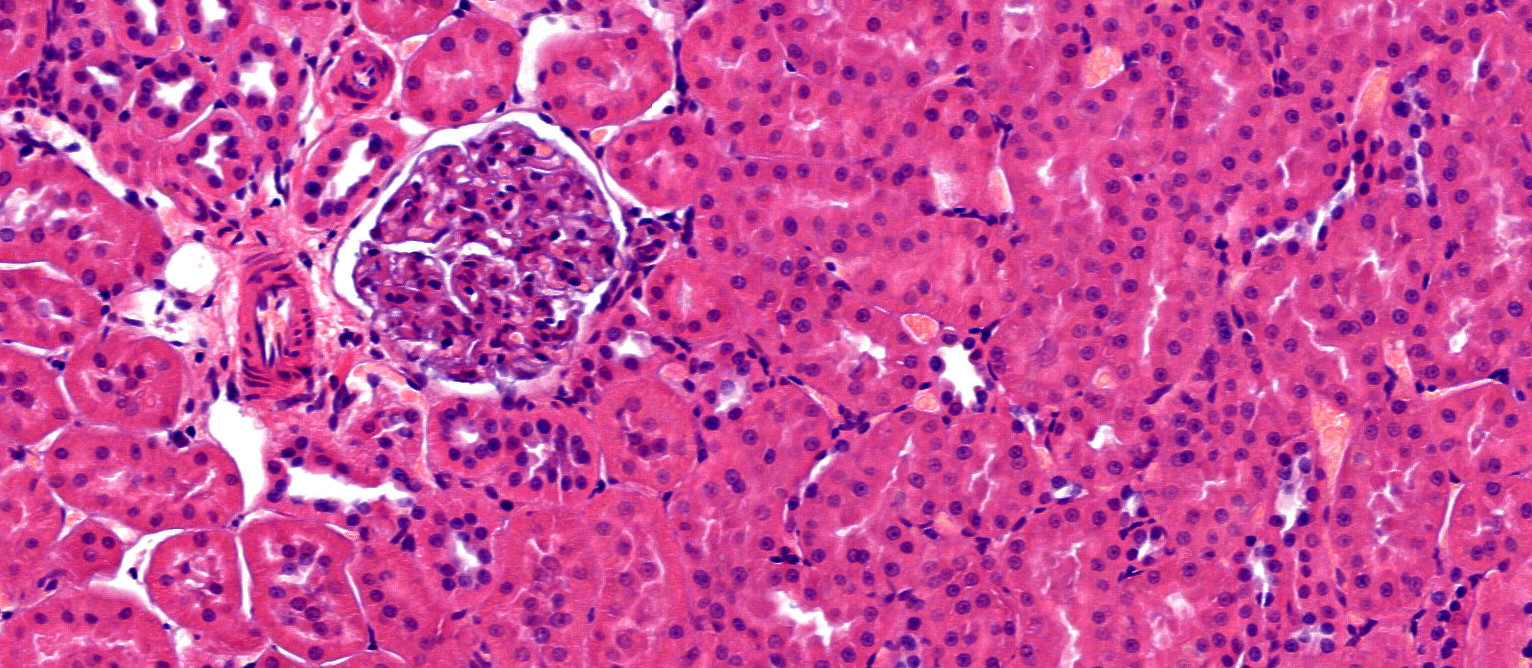

Supplement: Supplementary file 8 [file DataSheet1.ZIP › Fig 1D-HE-sham-1/1-3.jpeg]

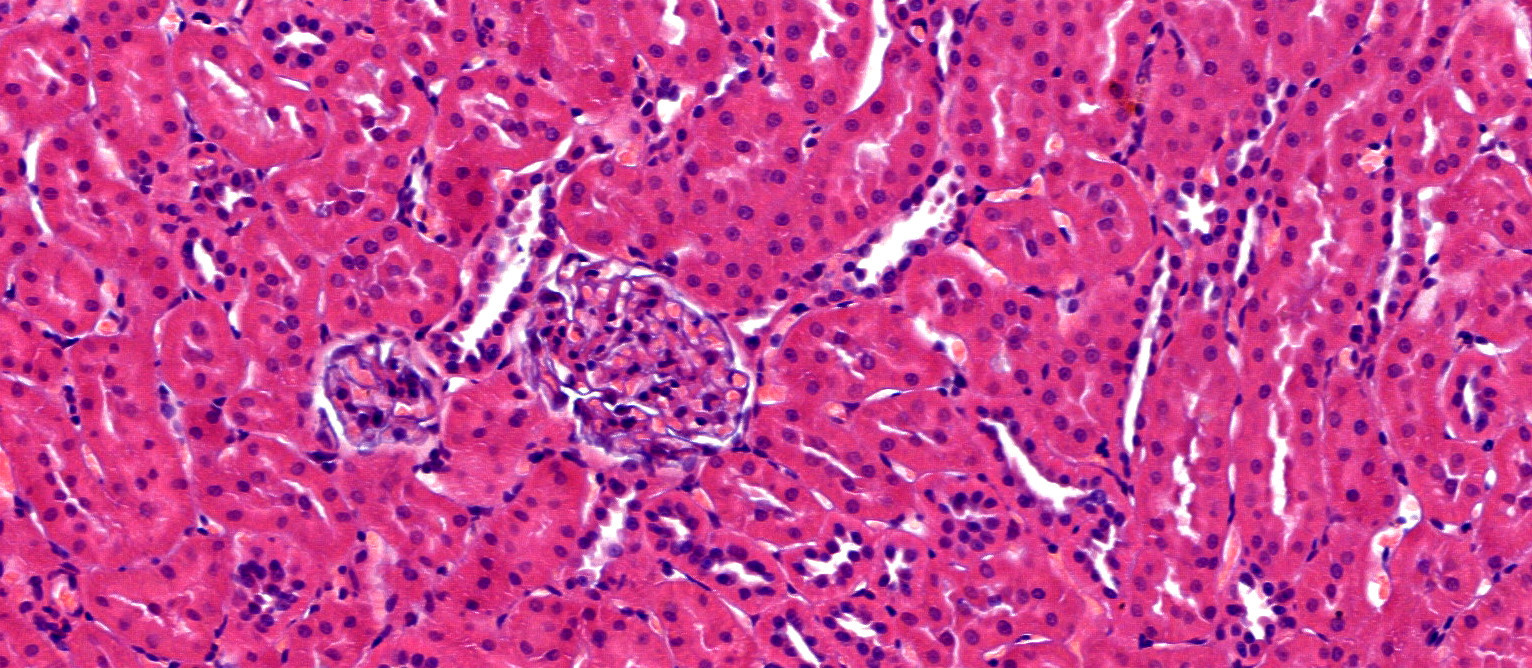

Supplement: Supplementary file 8 [file DataSheet1.ZIP › Fig 1D-HE-sham-1/1-4.jpeg]

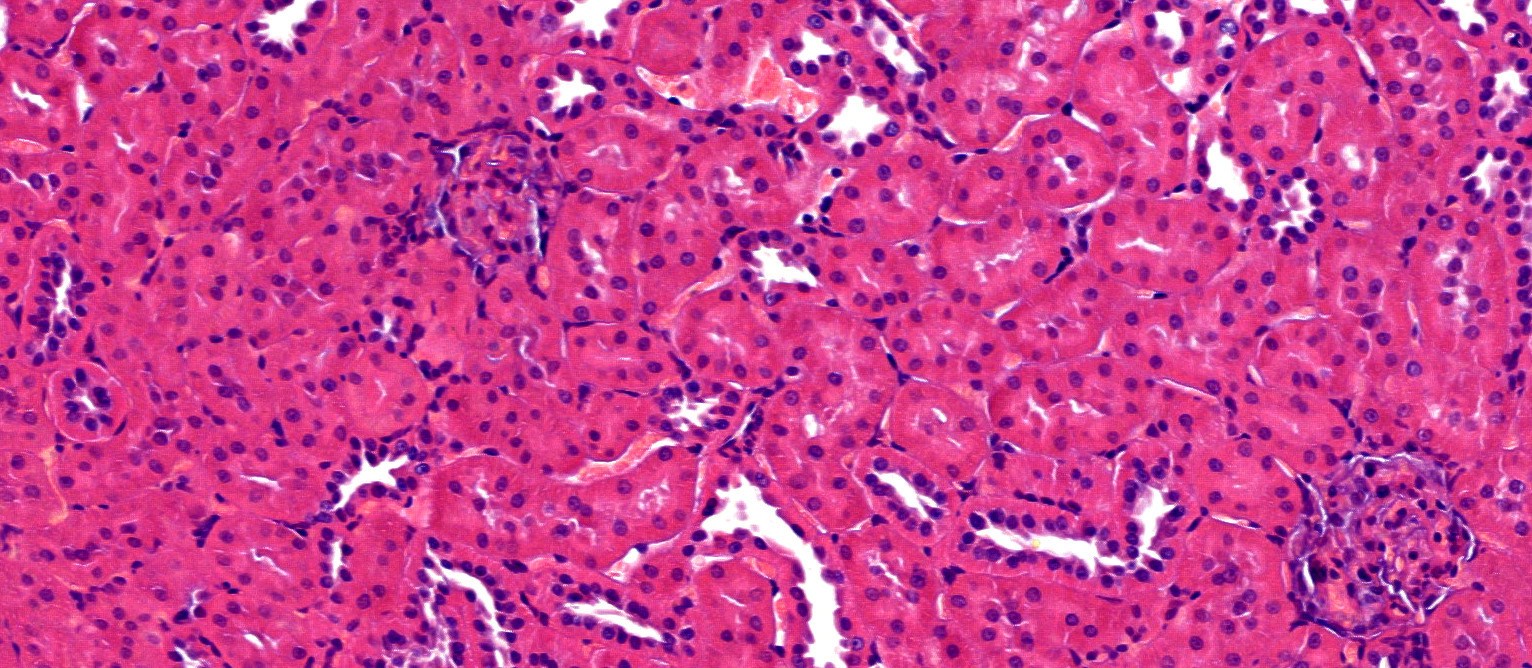

Supplement: Supplementary file 8 [file DataSheet1.ZIP › Fig 1D-HE-sham-1/1-5.jpeg]

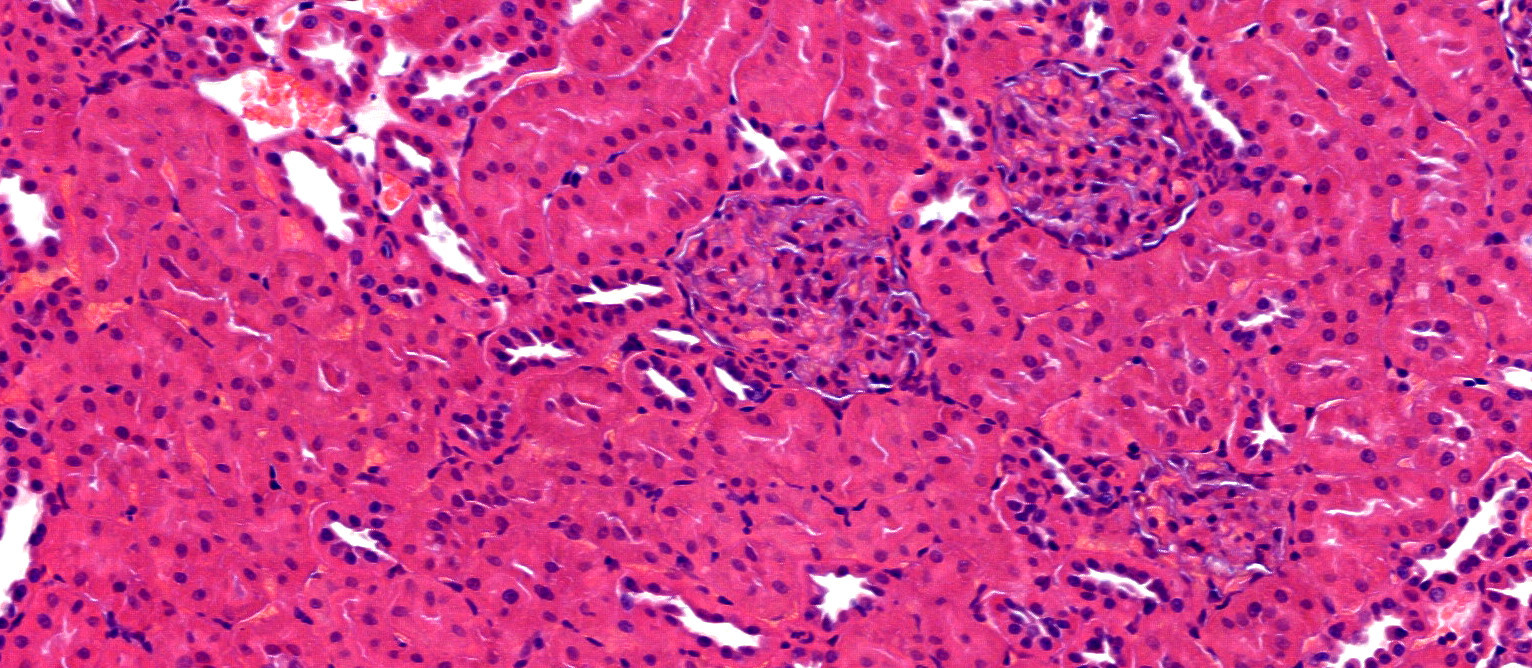

Supplement: Supplementary file 8 [file DataSheet1.ZIP › Fig 1D-HE-sham-1/1-6.jpeg]

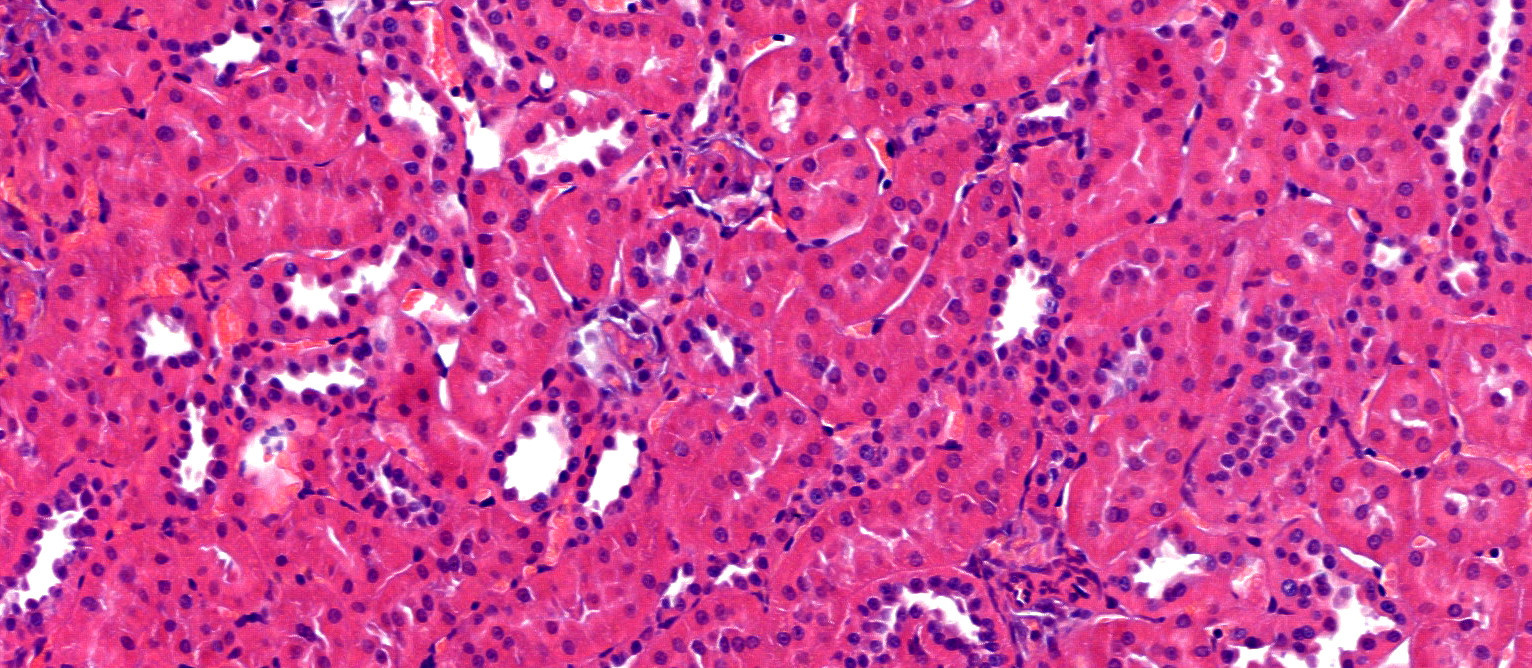

Supplement: Supplementary file 8 [file DataSheet1.ZIP › Fig 1D-HE-sham-1/1-7.jpeg]

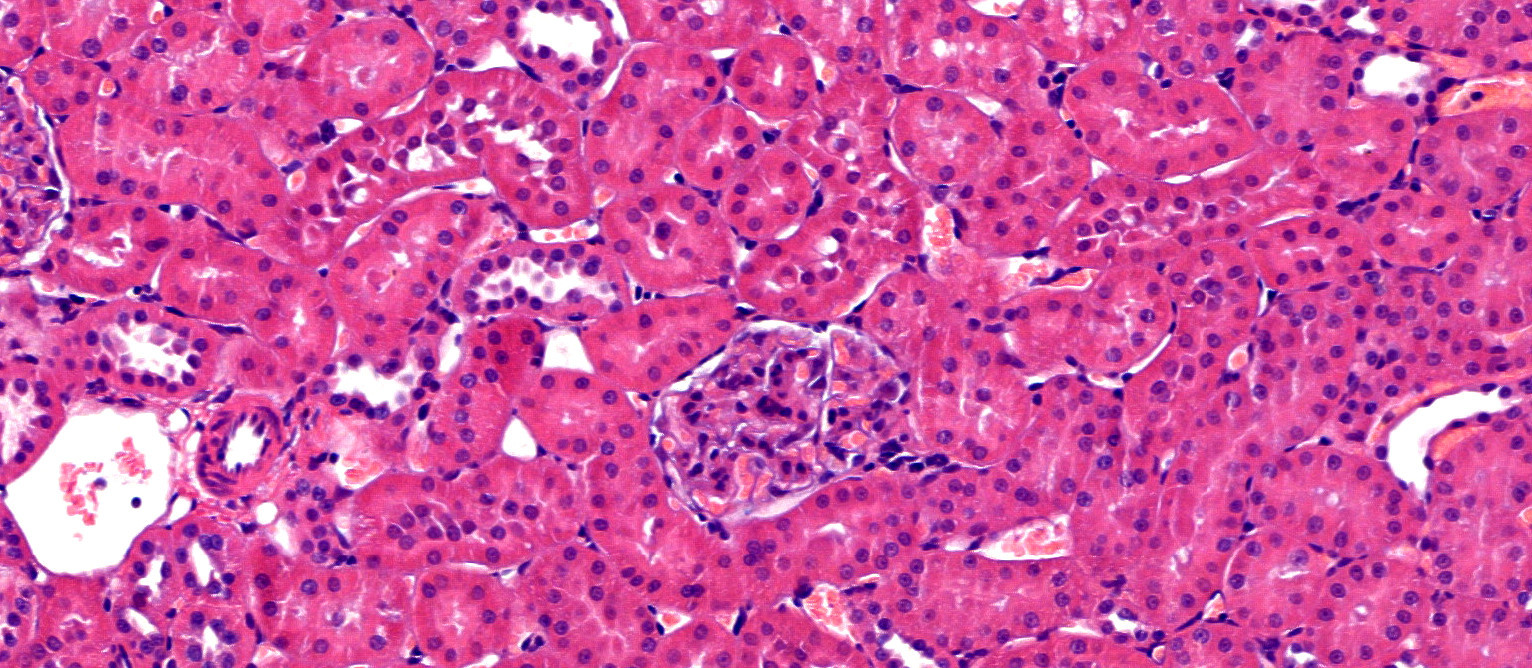

Supplement: Supplementary file 8 [file DataSheet1.ZIP › Fig 1D-HE-sham-1/1-8.jpeg]

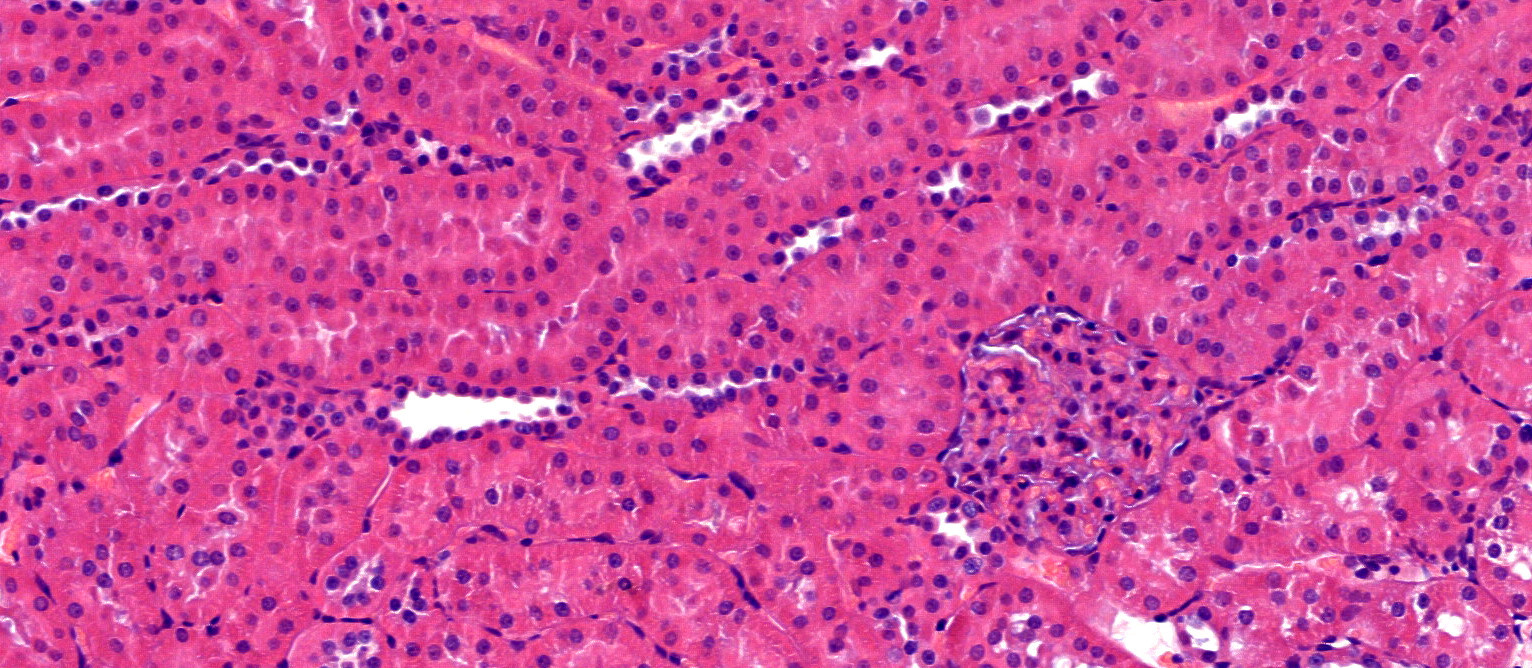

Supplement: Supplementary file 8 [file DataSheet1.ZIP › Fig 1D-HE-sham-1/1-9.jpeg]

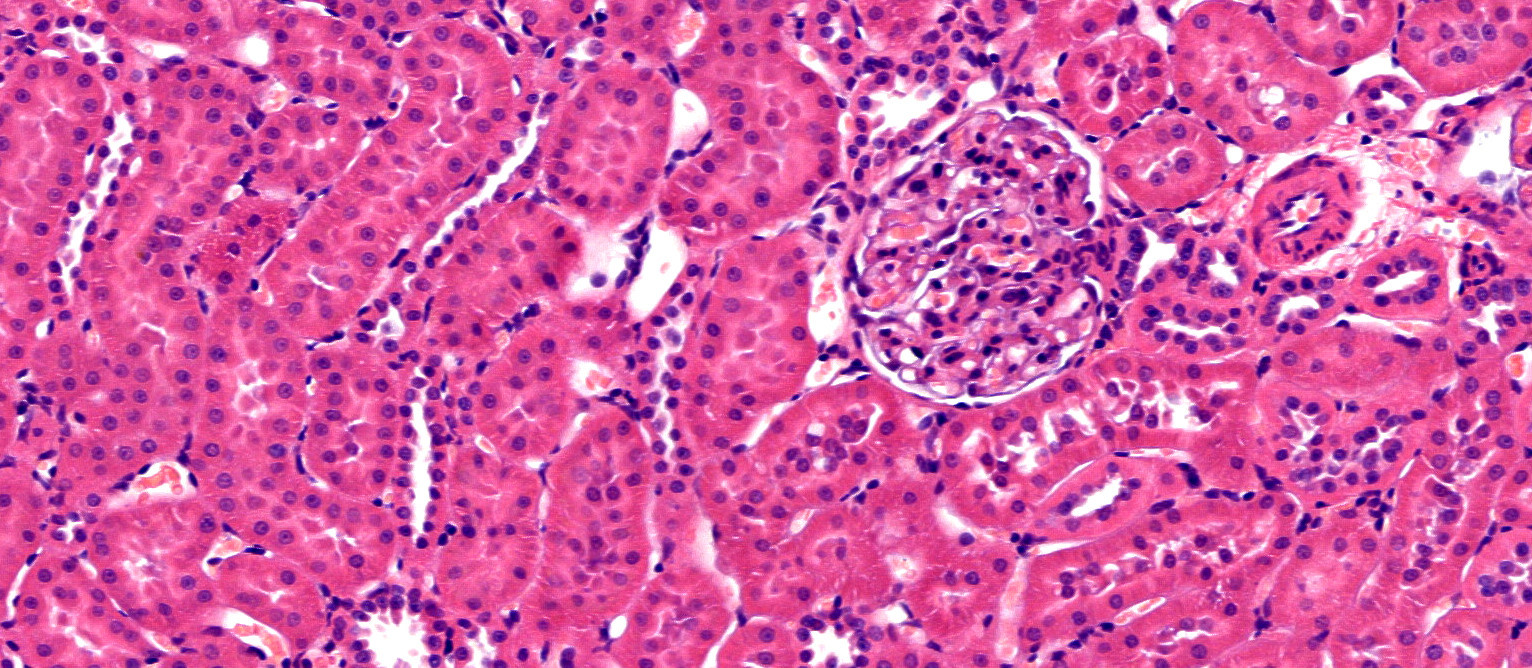

Supplement: Supplementary file 8 [file DataSheet1.ZIP › Fig 1D-HE-sham-2/2-1.jpeg]

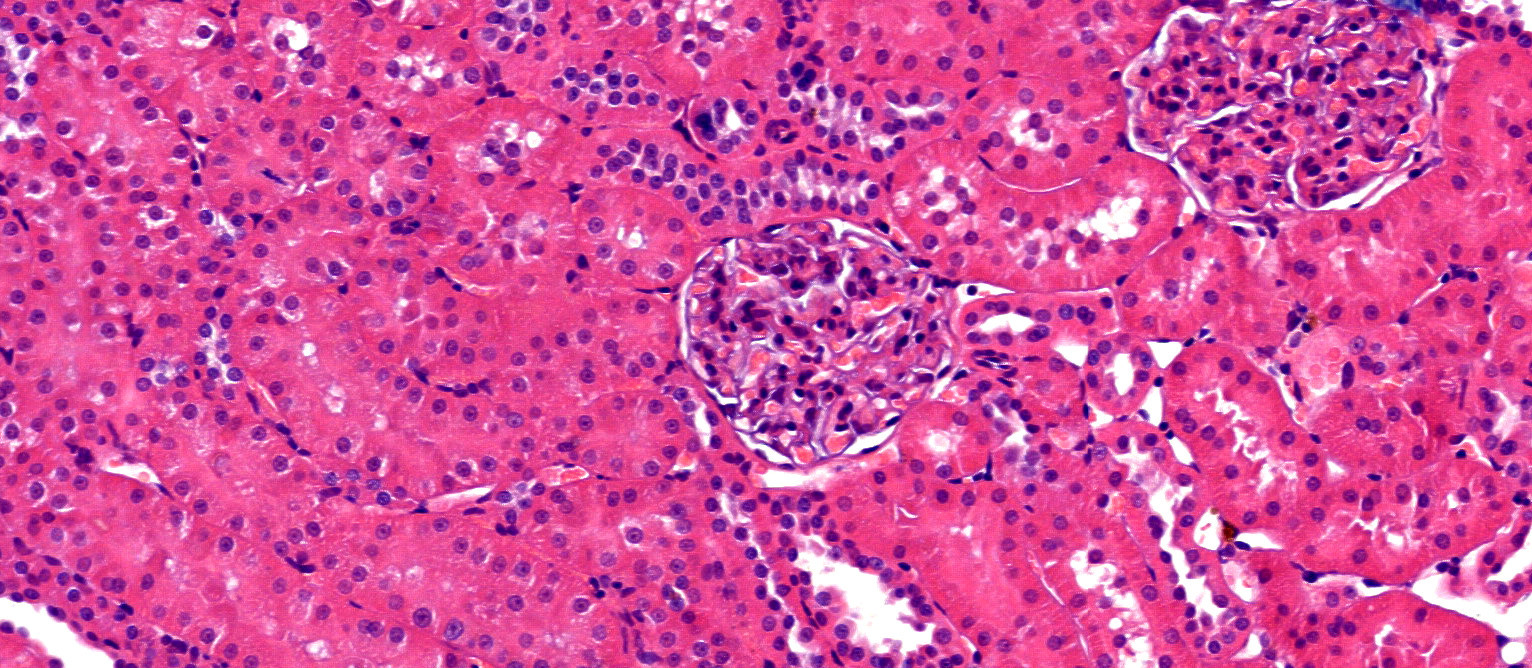

Supplement: Supplementary file 8 [file DataSheet1.ZIP › Fig 1D-HE-sham-2/2-10.jpeg]

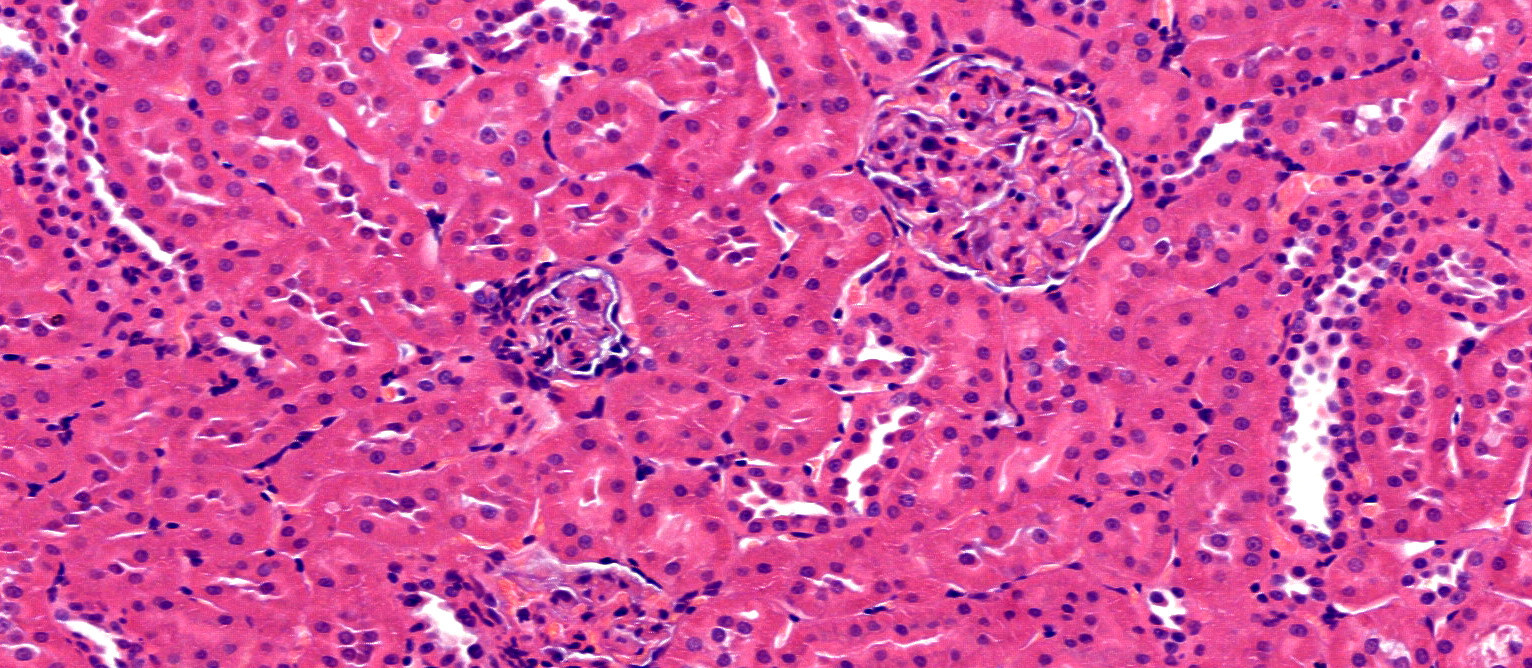

Supplement: Supplementary file 8 [file DataSheet1.ZIP › Fig 1D-HE-sham-2/2-2.jpeg]

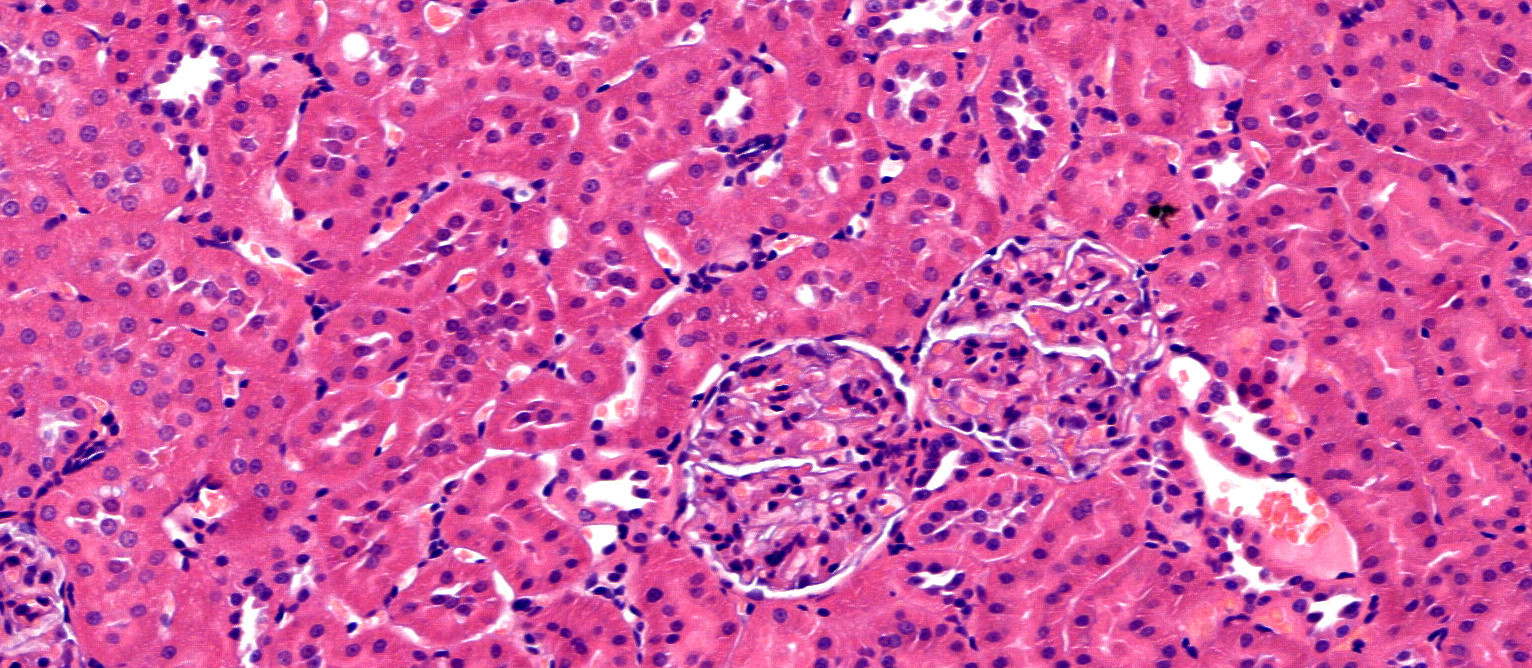

Supplement: Supplementary file 8 [file DataSheet1.ZIP › Fig 1D-HE-sham-2/2-3.jpeg]

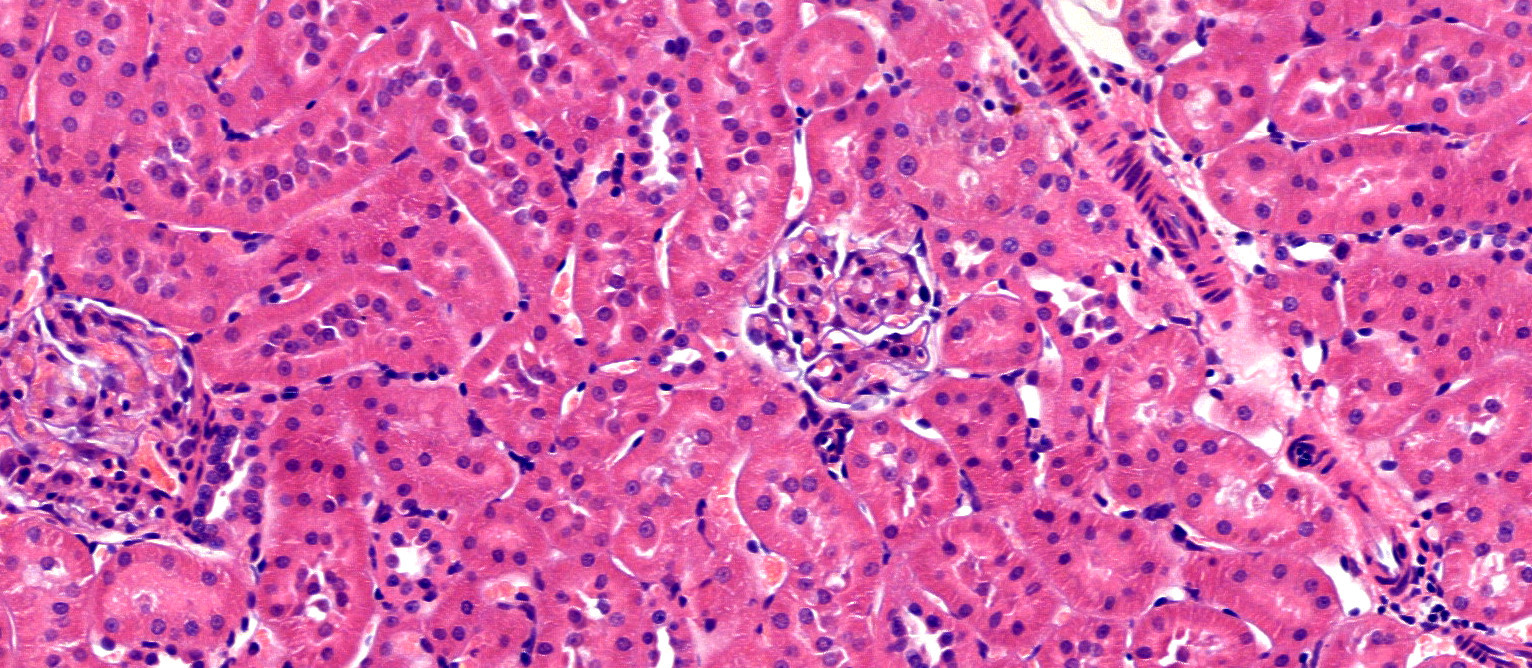

Supplement: Supplementary file 8 [file DataSheet1.ZIP › Fig 1D-HE-sham-2/2-4.jpeg]

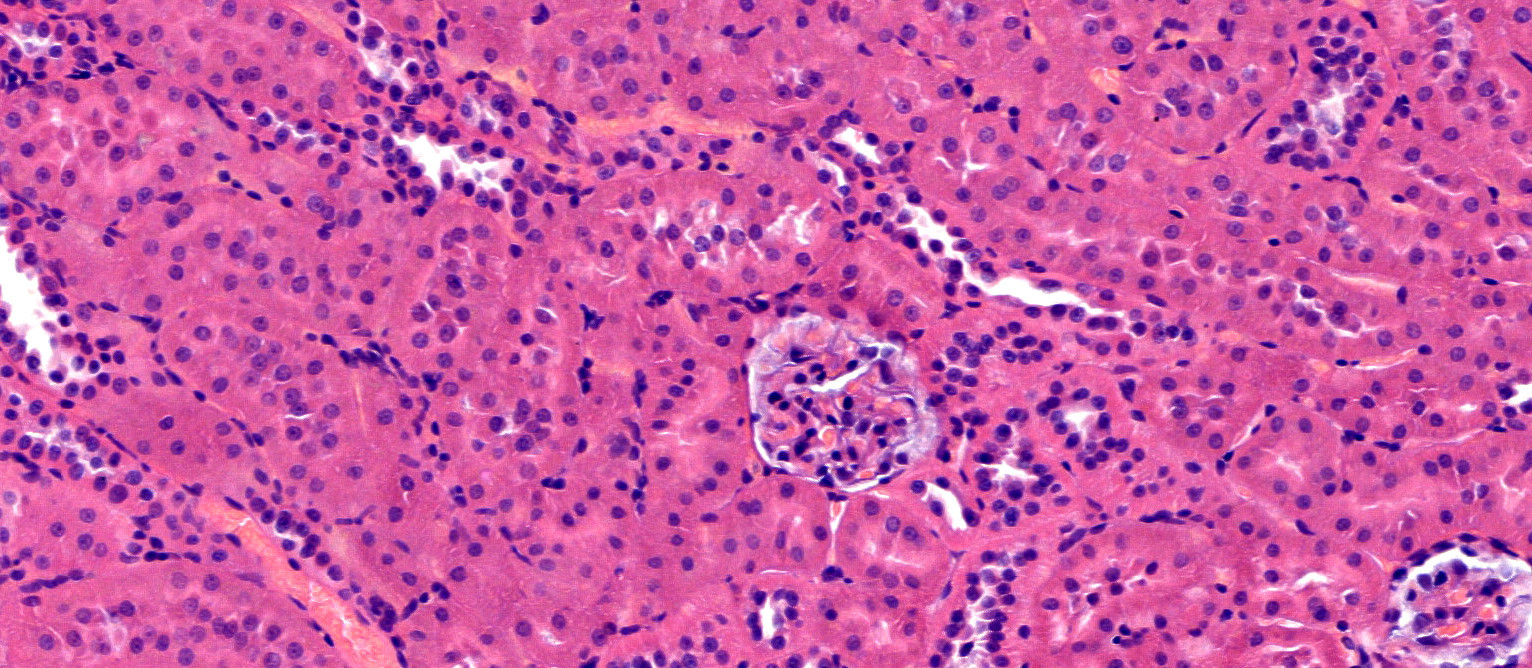

Supplement: Supplementary file 8 [file DataSheet1.ZIP › Fig 1D-HE-sham-2/2-5.jpeg]

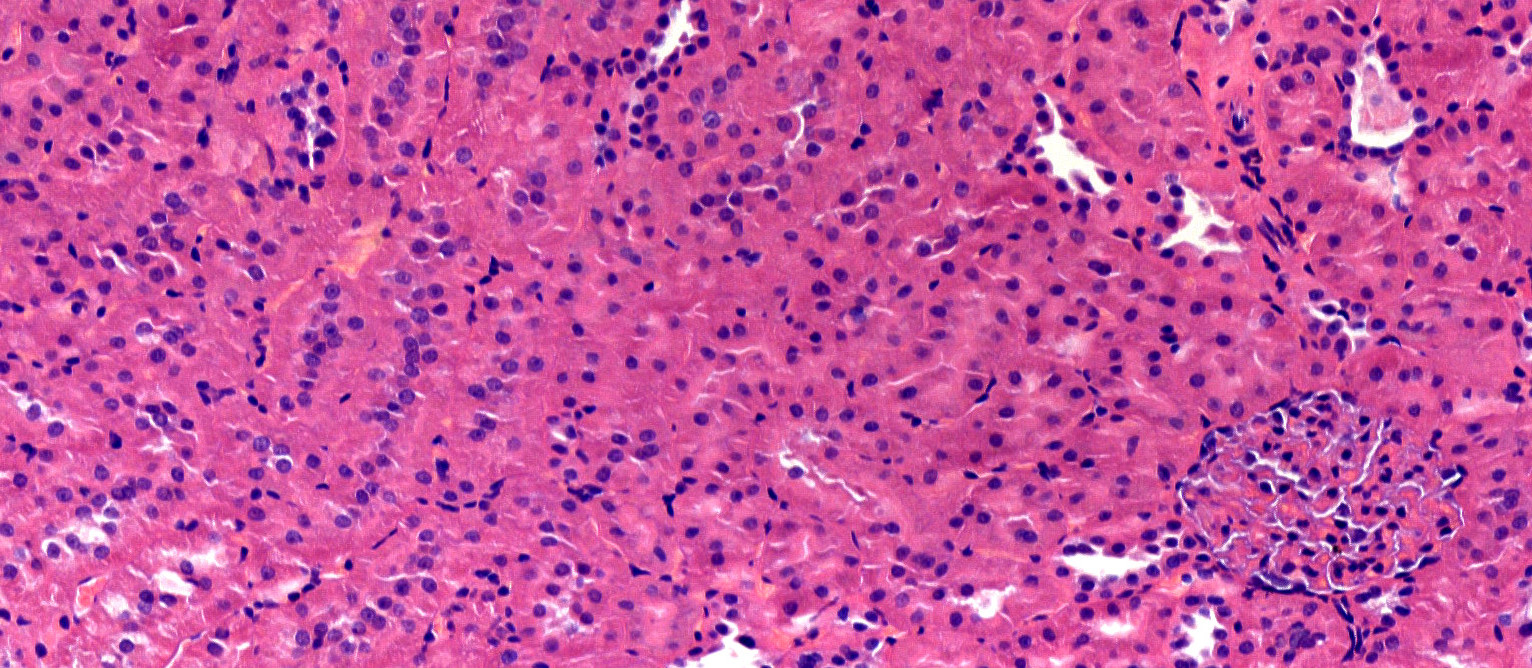

Supplement: Supplementary file 8 [file DataSheet1.ZIP › Fig 1D-HE-sham-2/2-6.jpeg]

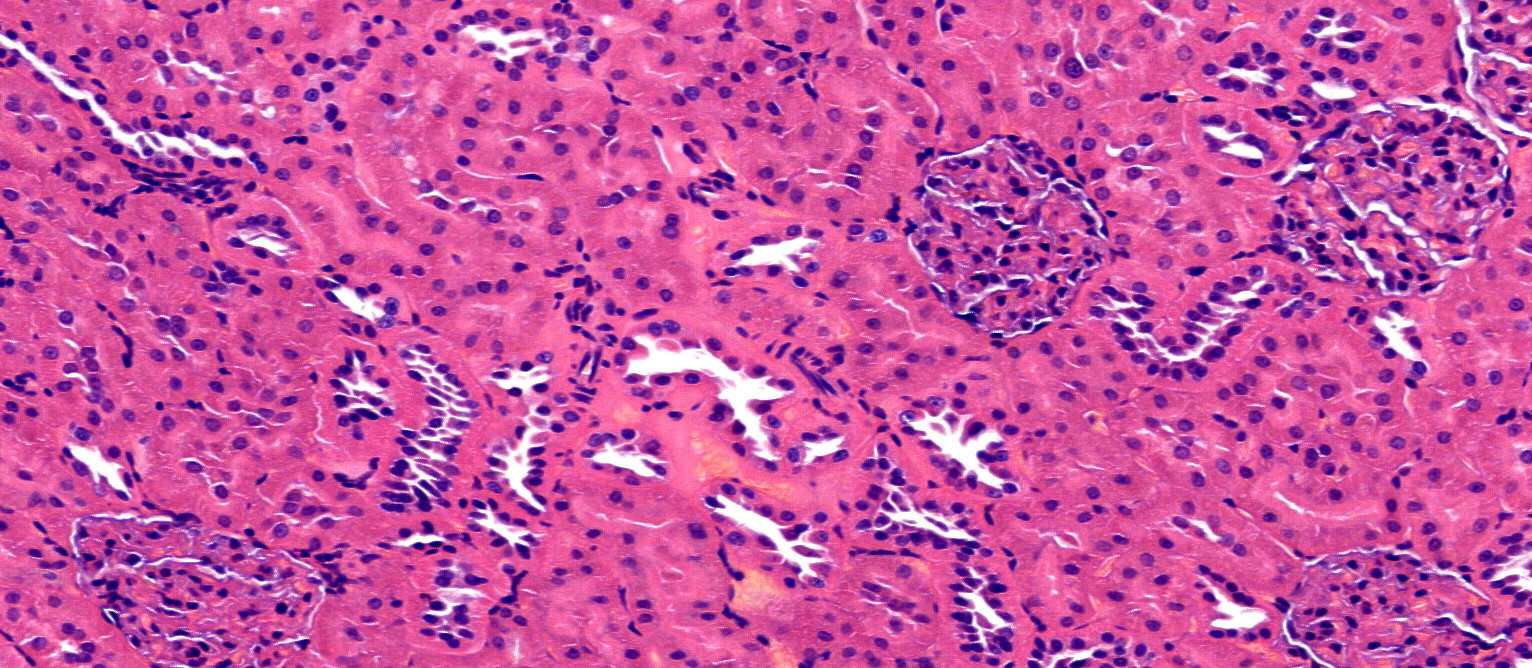

Supplement: Supplementary file 8 [file DataSheet1.ZIP › Fig 1D-HE-sham-2/2-7.jpeg]

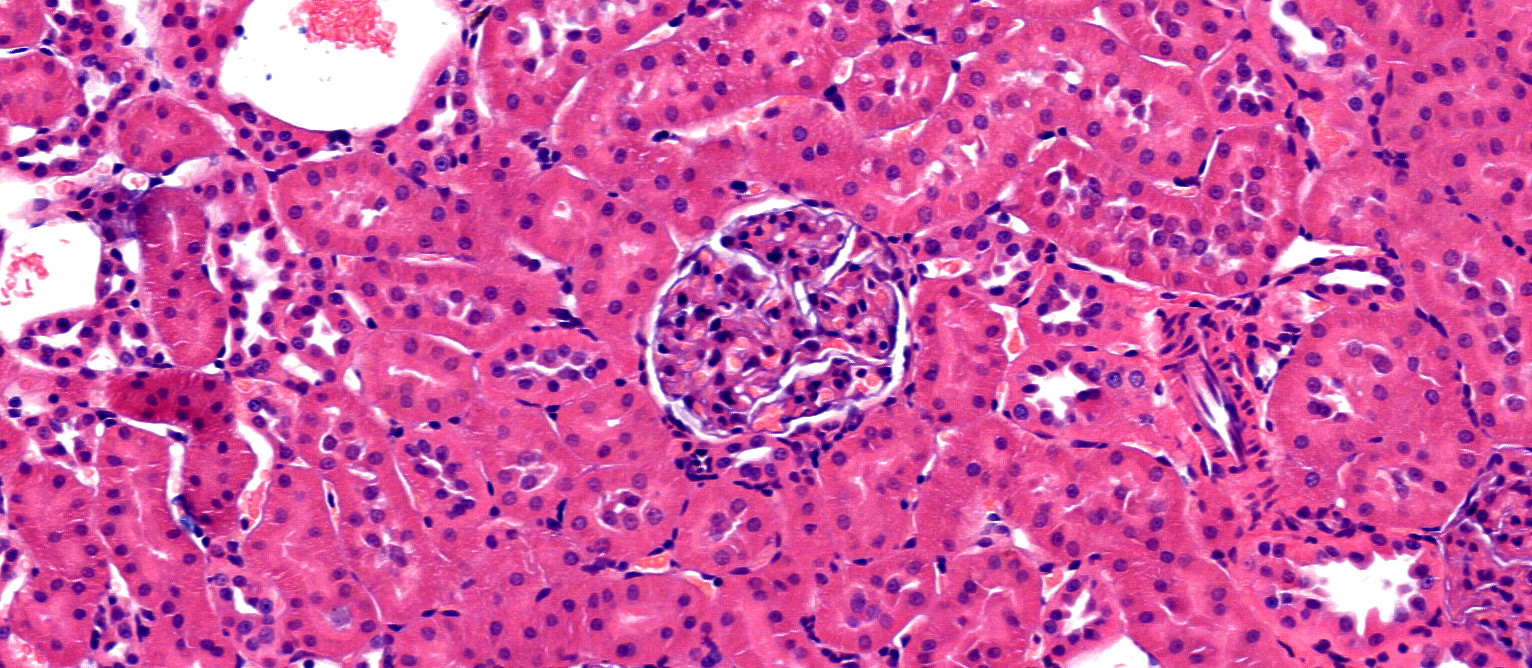

Supplement: Supplementary file 8 [file DataSheet1.ZIP › Fig 1D-HE-sham-2/2-8.jpeg]

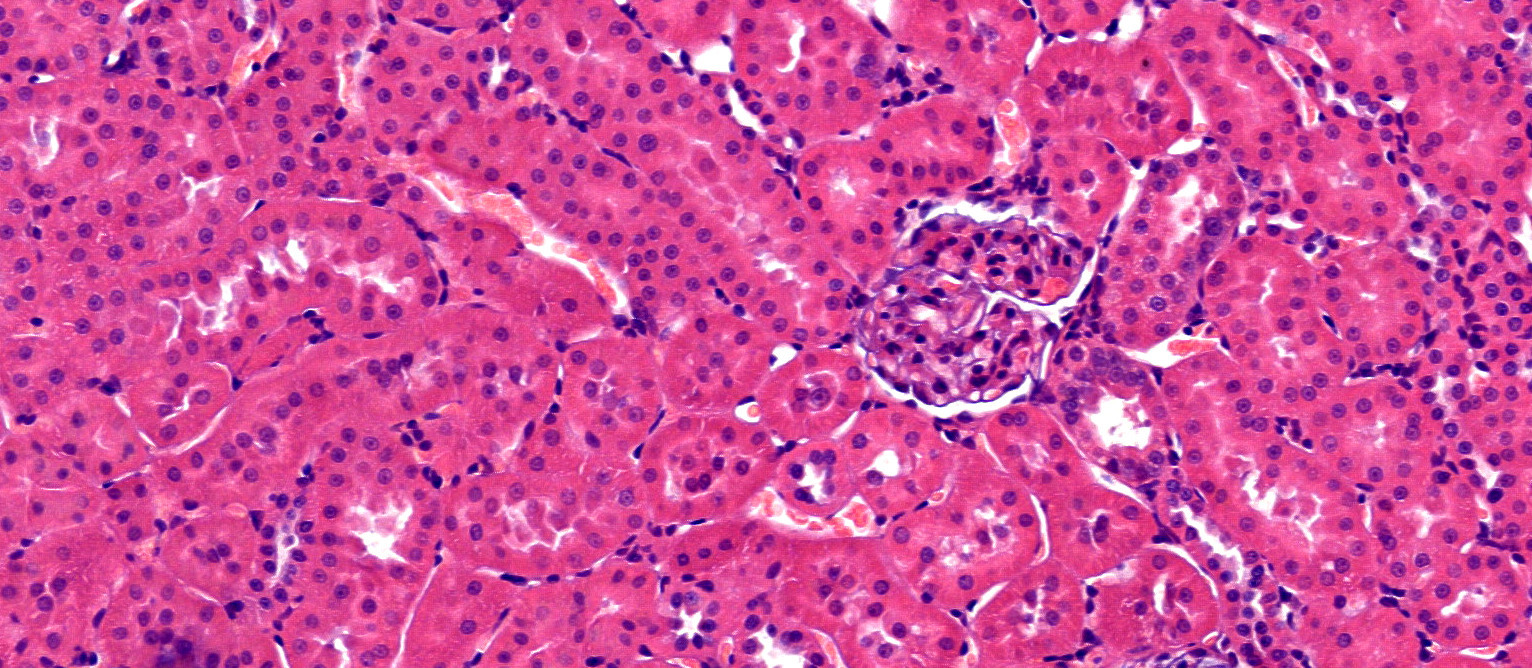

Supplement: Supplementary file 8 [file DataSheet1.ZIP › Fig 1D-HE-sham-2/2-9.jpeg]

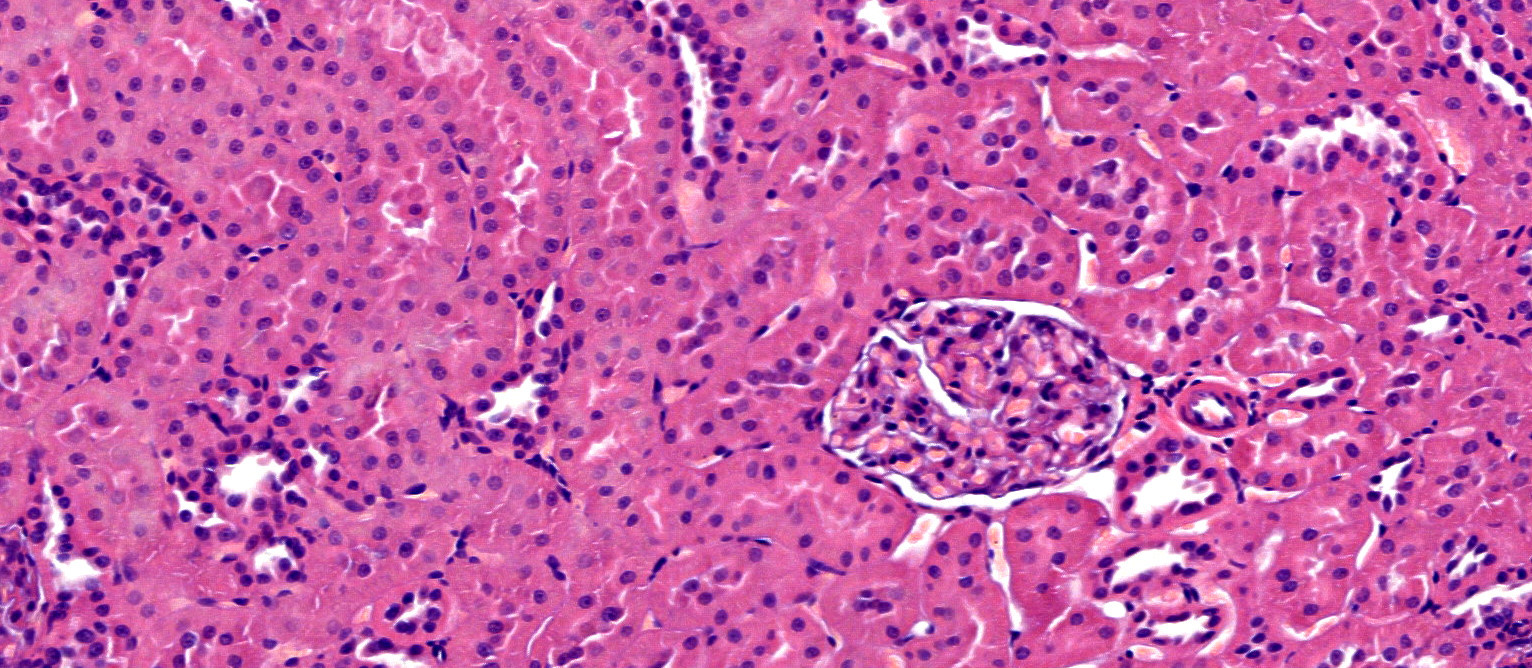

Supplement: Supplementary file 8 [file DataSheet1.ZIP › Fig 1D-HE-sham-3/3-1.jpeg]

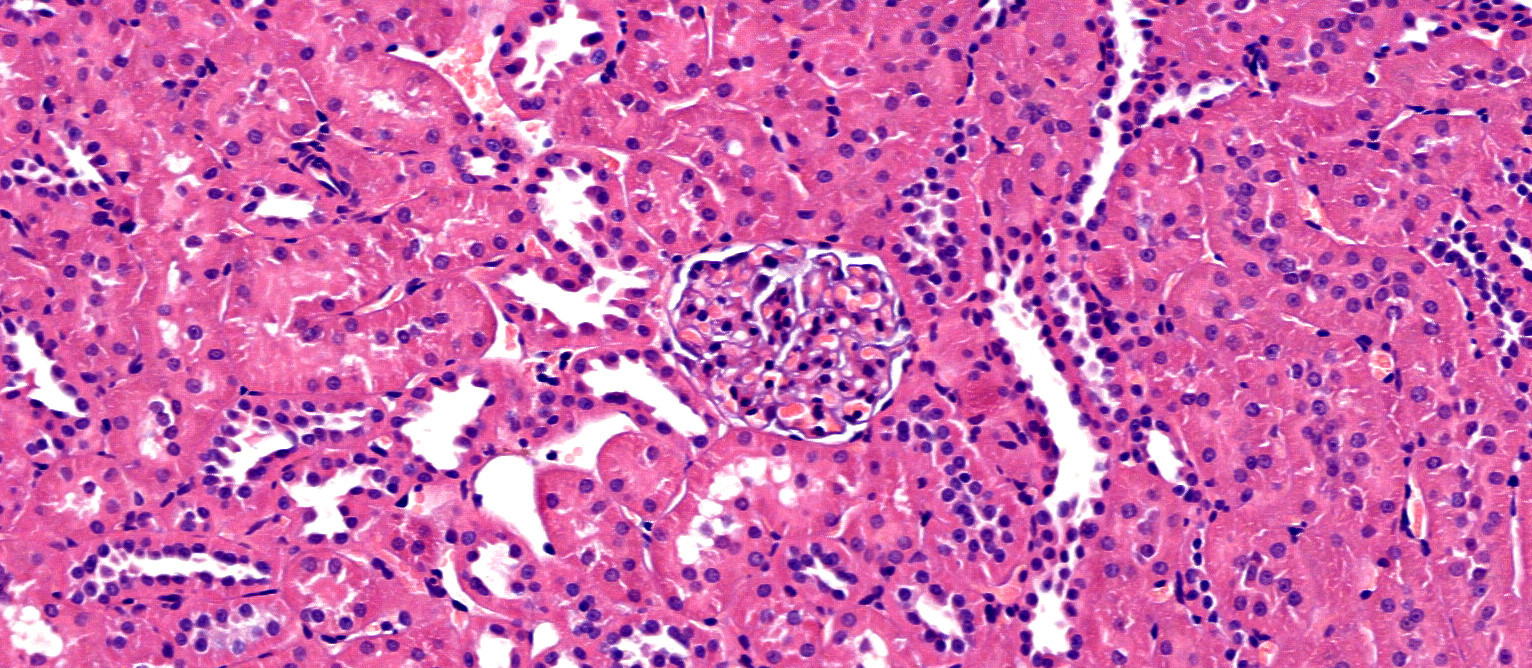

Supplement: Supplementary file 8 [file DataSheet1.ZIP › Fig 1D-HE-sham-3/3-10.jpeg]

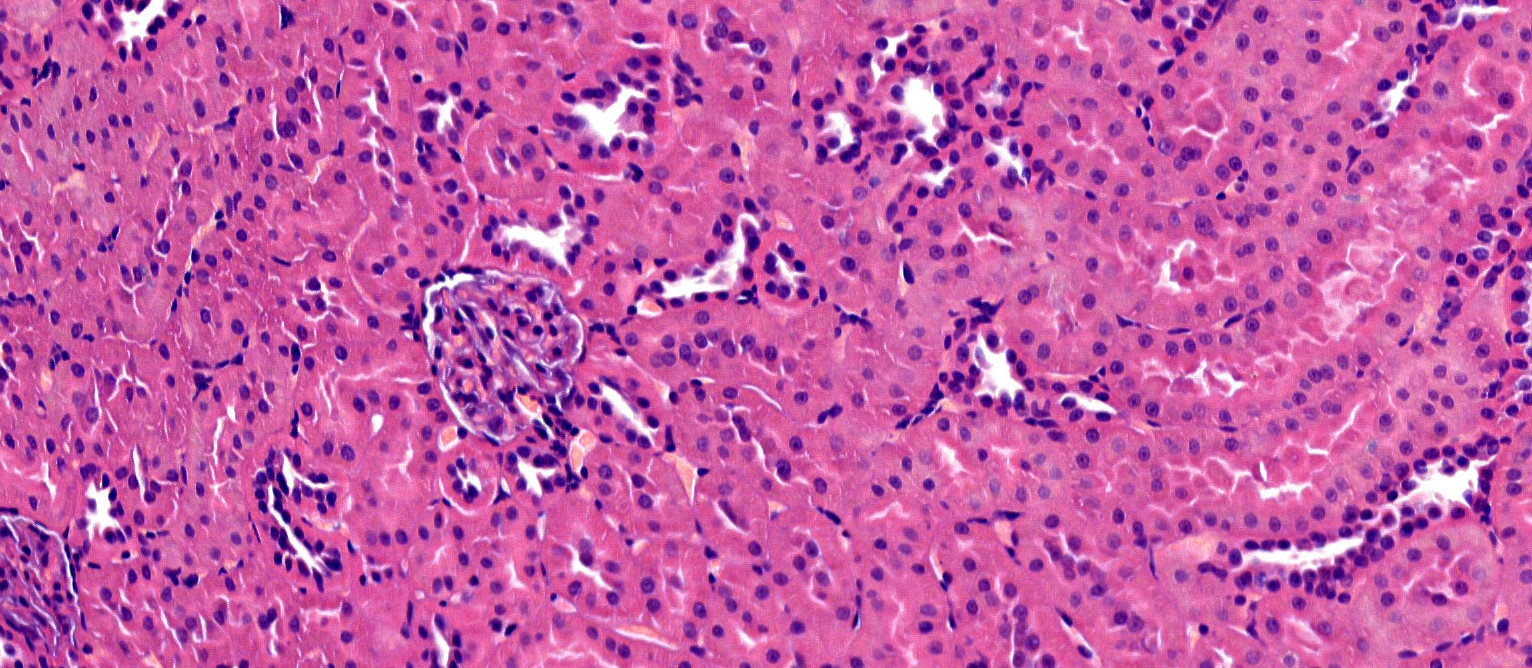

Supplement: Supplementary file 8 [file DataSheet1.ZIP › Fig 1D-HE-sham-3/3-2.jpeg]

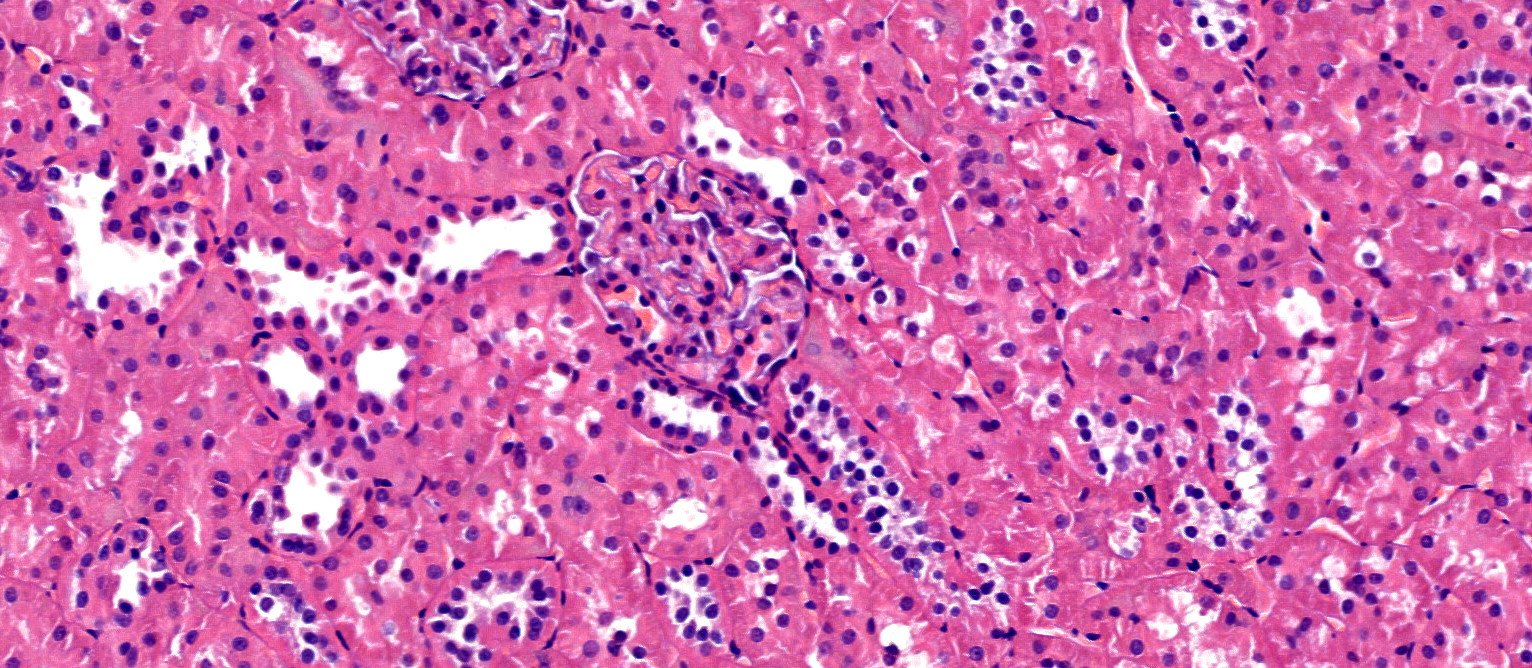

Supplement: Supplementary file 8 [file DataSheet1.ZIP › Fig 1D-HE-sham-3/3-3.jpeg]

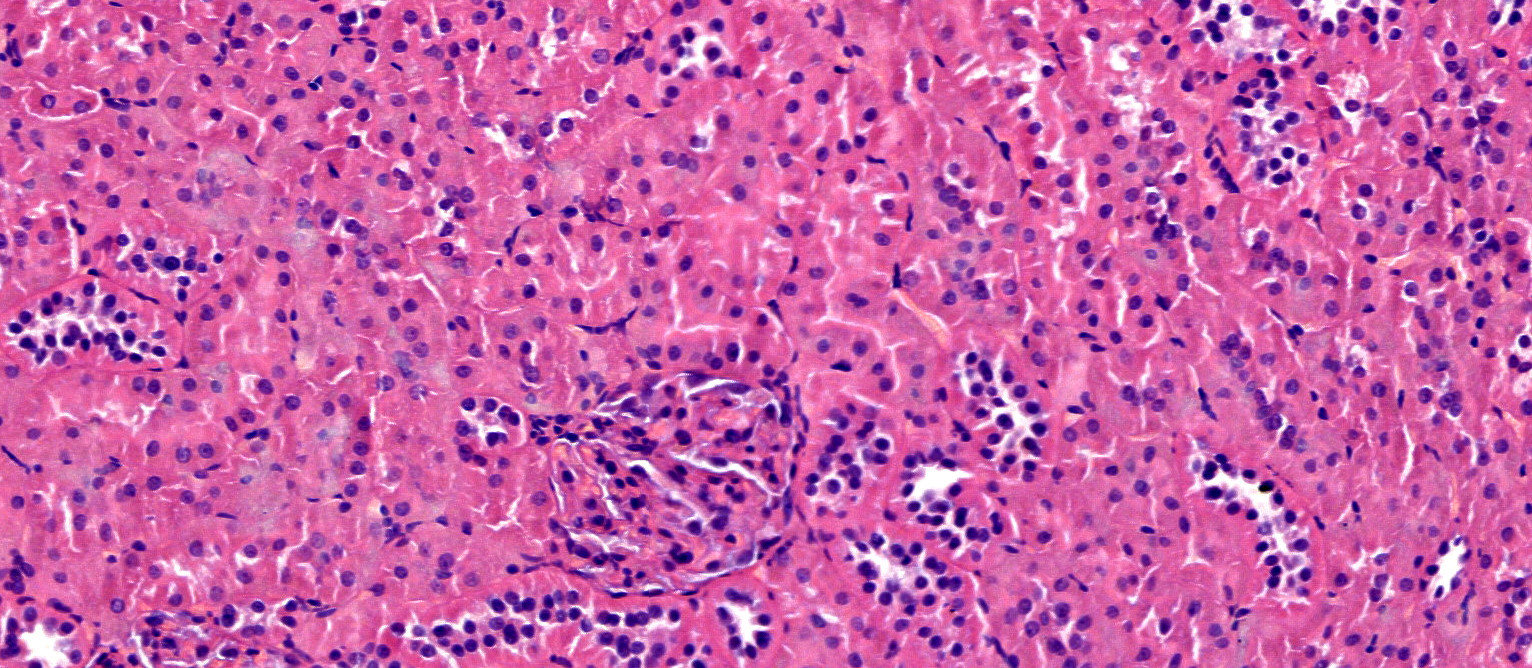

Supplement: Supplementary file 8 [file DataSheet1.ZIP › Fig 1D-HE-sham-3/3-4.jpeg]

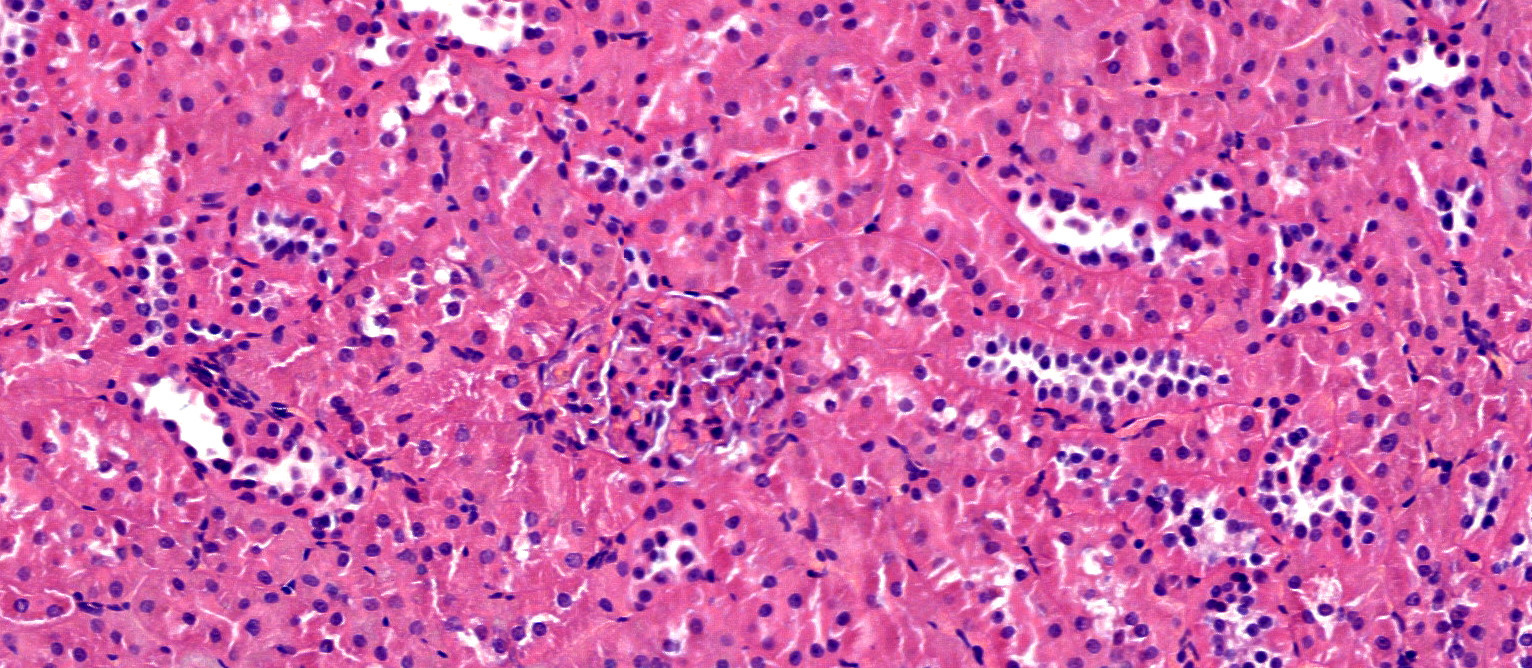

Supplement: Supplementary file 8 [file DataSheet1.ZIP › Fig 1D-HE-sham-3/3-5.jpeg]

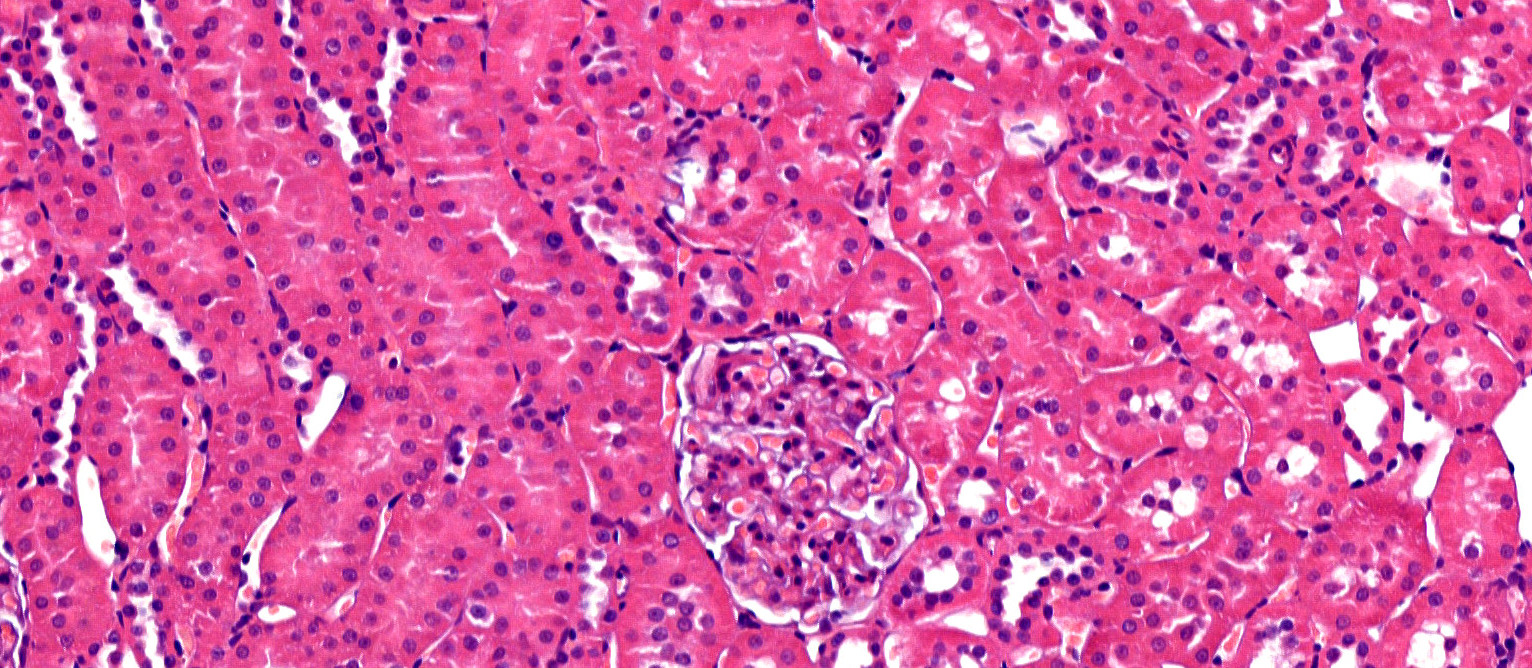

Supplement: Supplementary file 8 [file DataSheet1.ZIP › Fig 1D-HE-sham-3/3-6.jpeg]

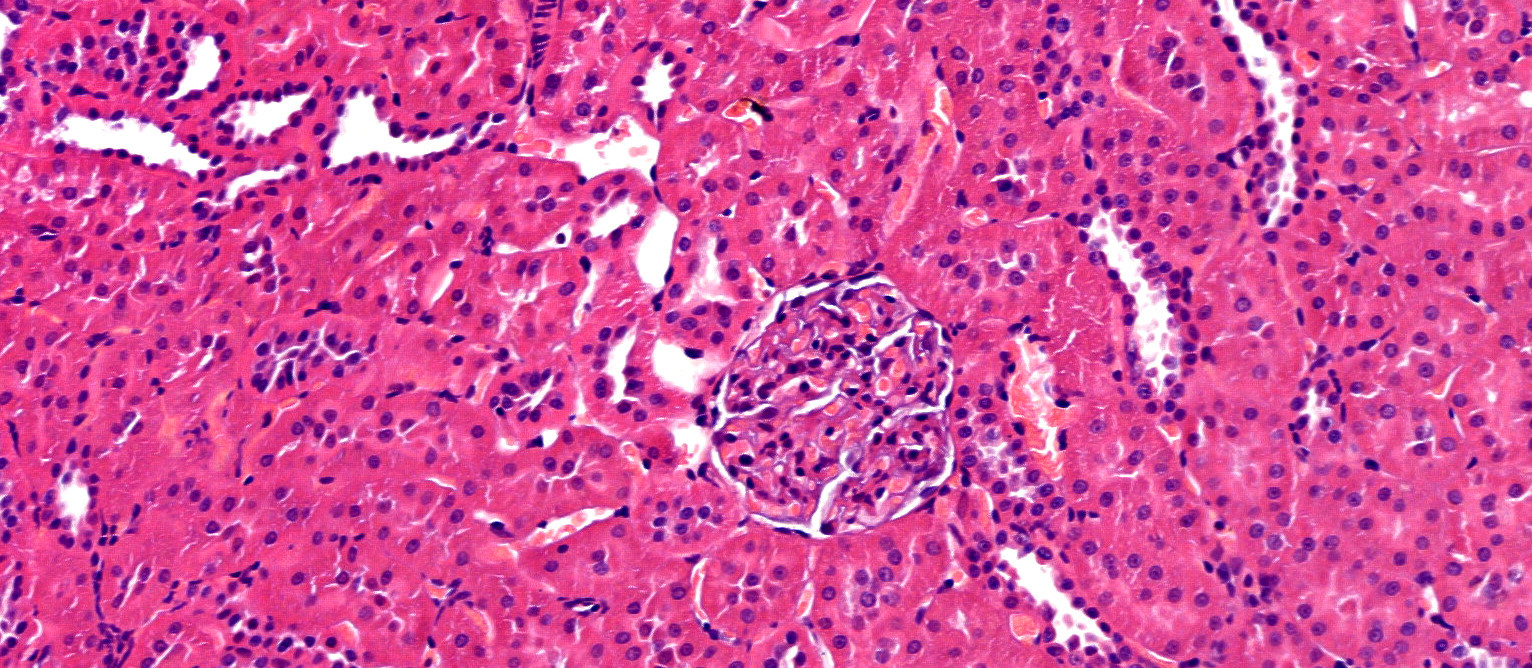

Supplement: Supplementary file 8 [file DataSheet1.ZIP › Fig 1D-HE-sham-3/3-7.jpeg]

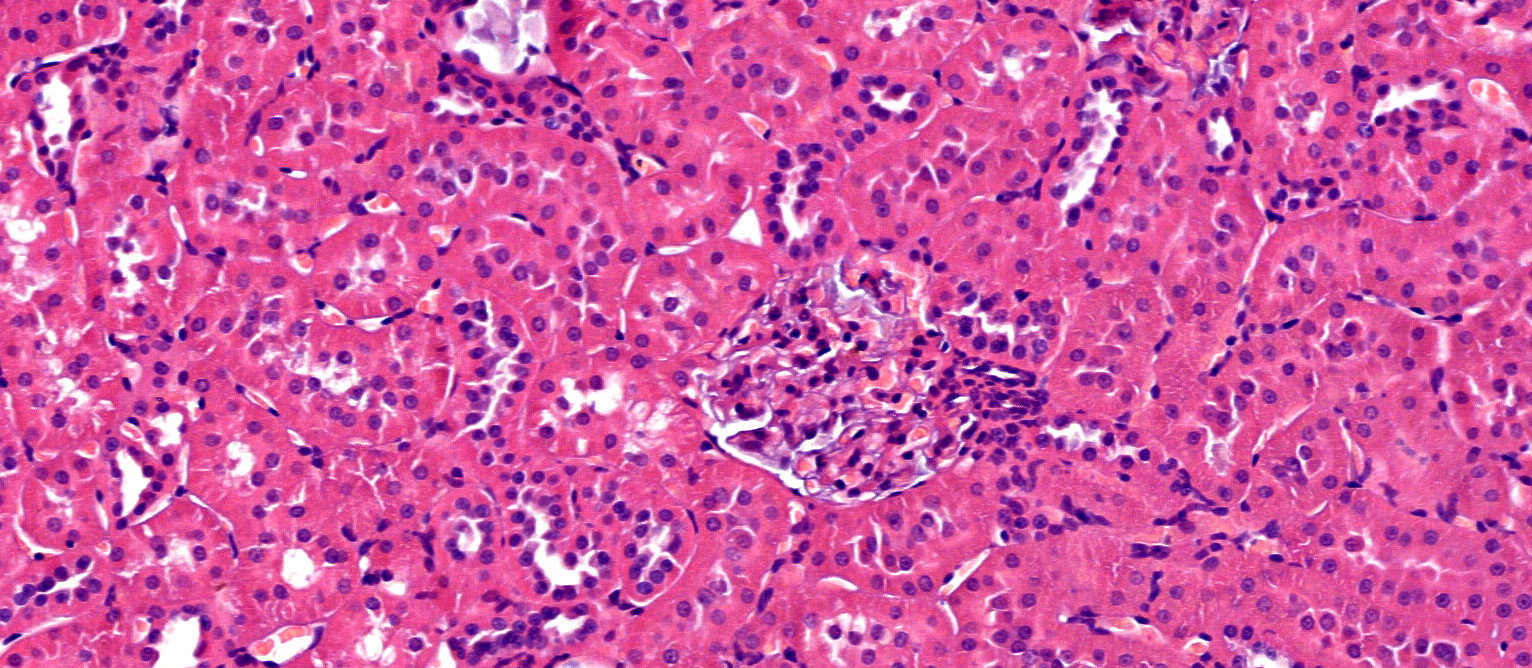

Supplement: Supplementary file 8 [file DataSheet1.ZIP › Fig 1D-HE-sham-3/3-8.jpeg]

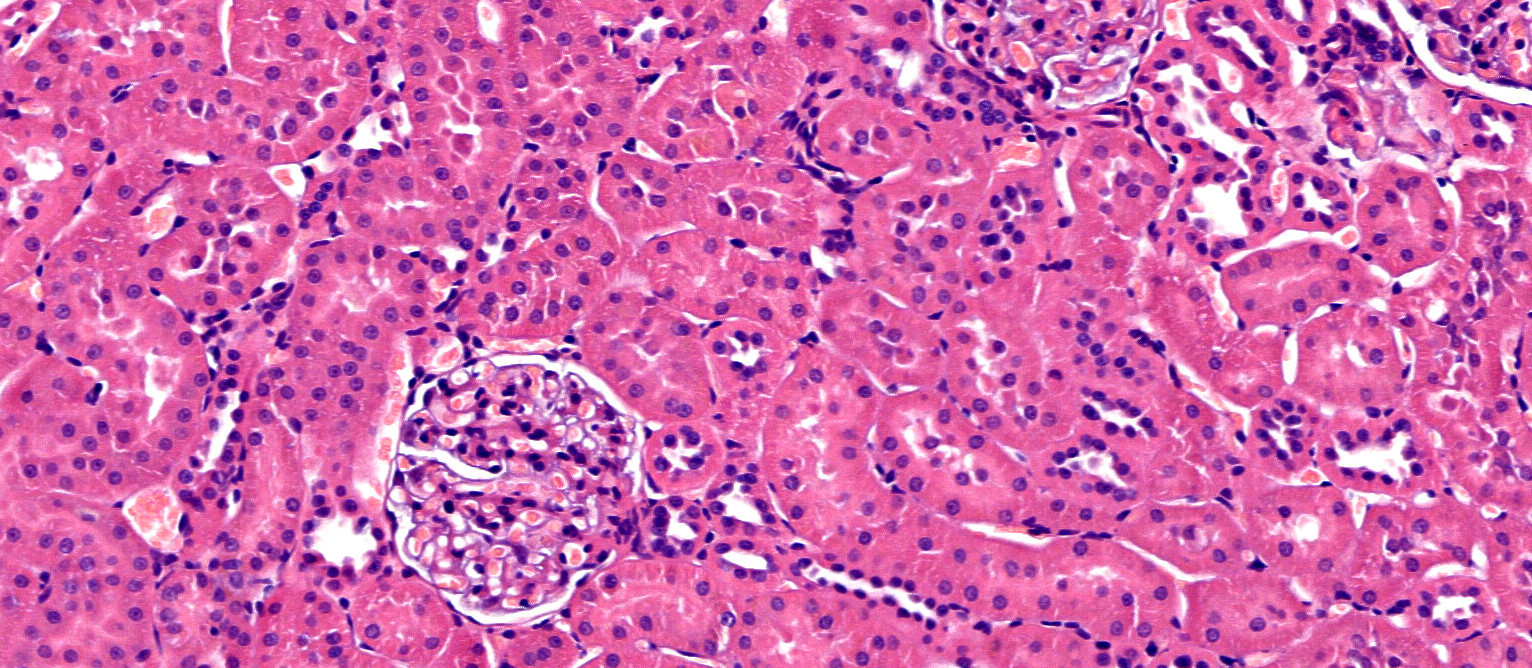

Supplement: Supplementary file 8 [file DataSheet1.ZIP › Fig 1D-HE-sham-3/3-9.jpeg]

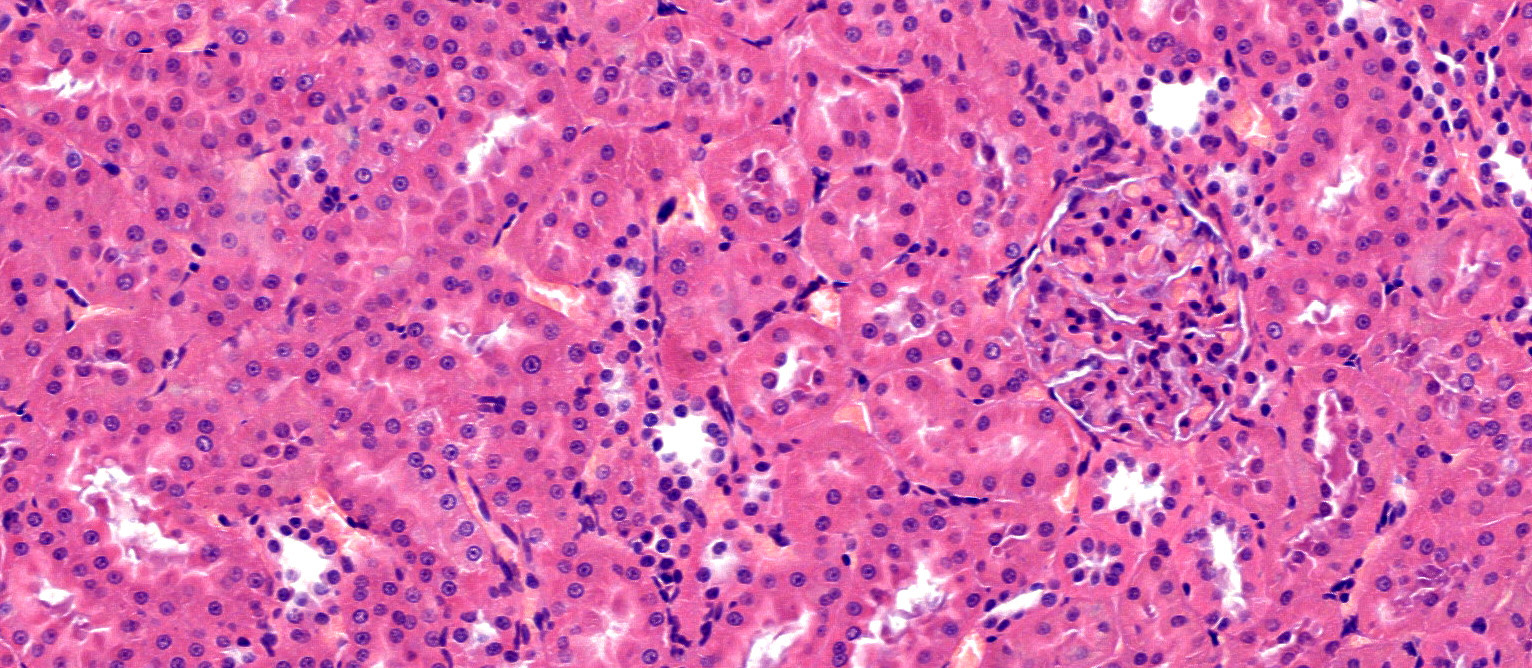

Supplement: Supplementary file 8 [file DataSheet1.ZIP › Fig 1D-HE-sham-4/4-1.jpeg]

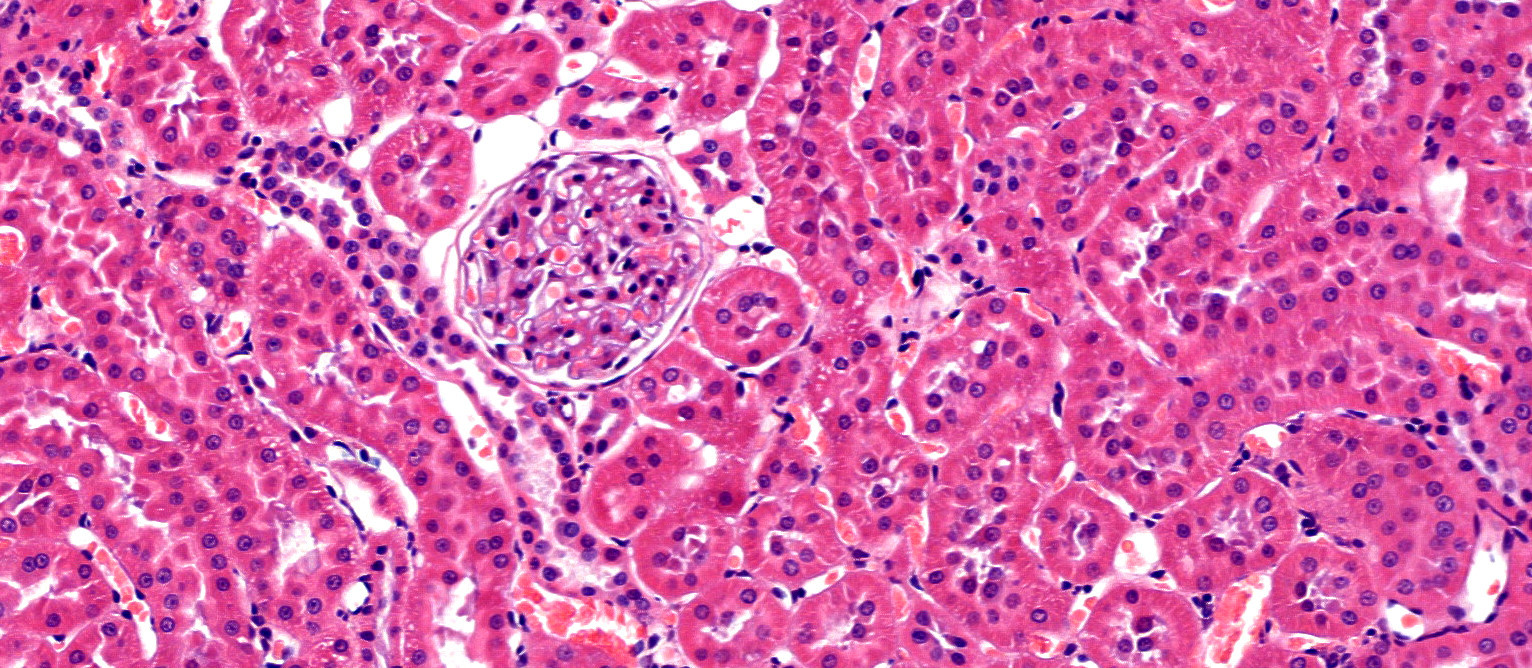

Supplement: Supplementary file 8 [file DataSheet1.ZIP › Fig 1D-HE-sham-4/4-10.jpeg]

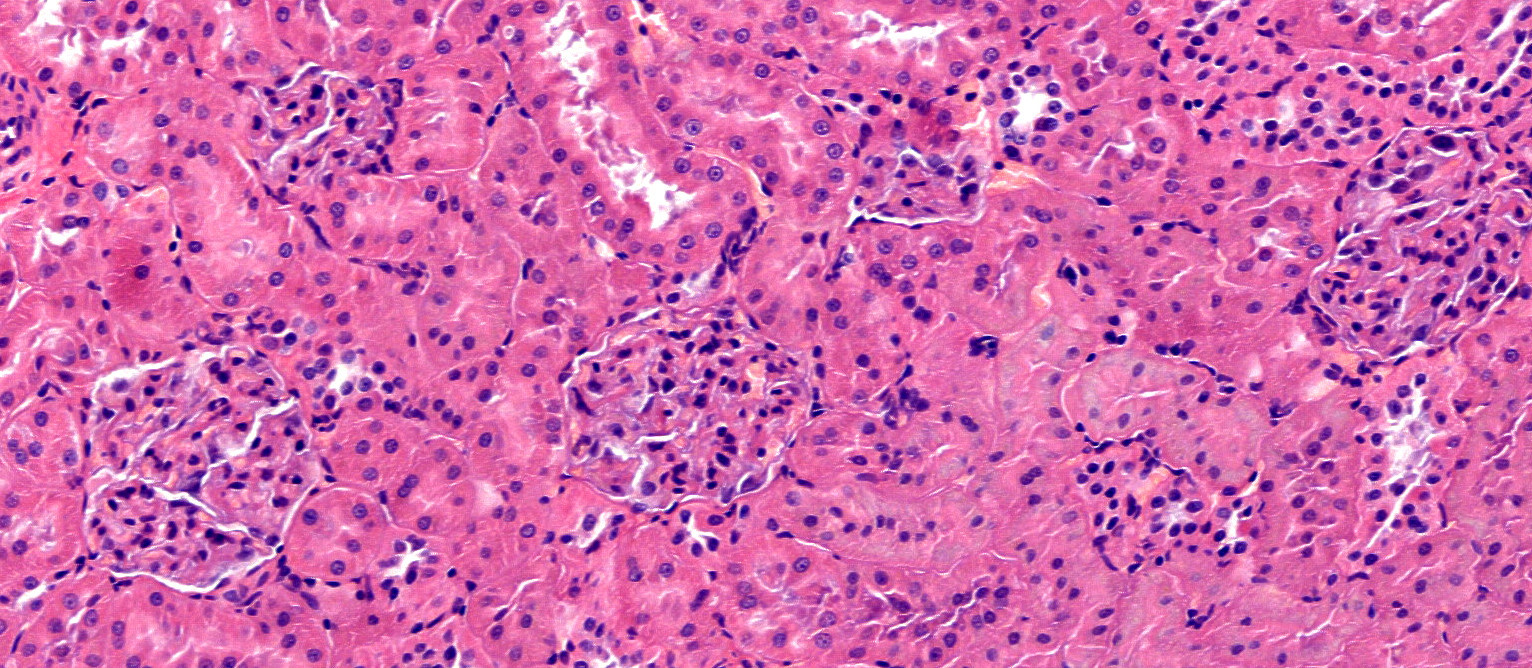

Supplement: Supplementary file 8 [file DataSheet1.ZIP › Fig 1D-HE-sham-4/4-2.jpeg]

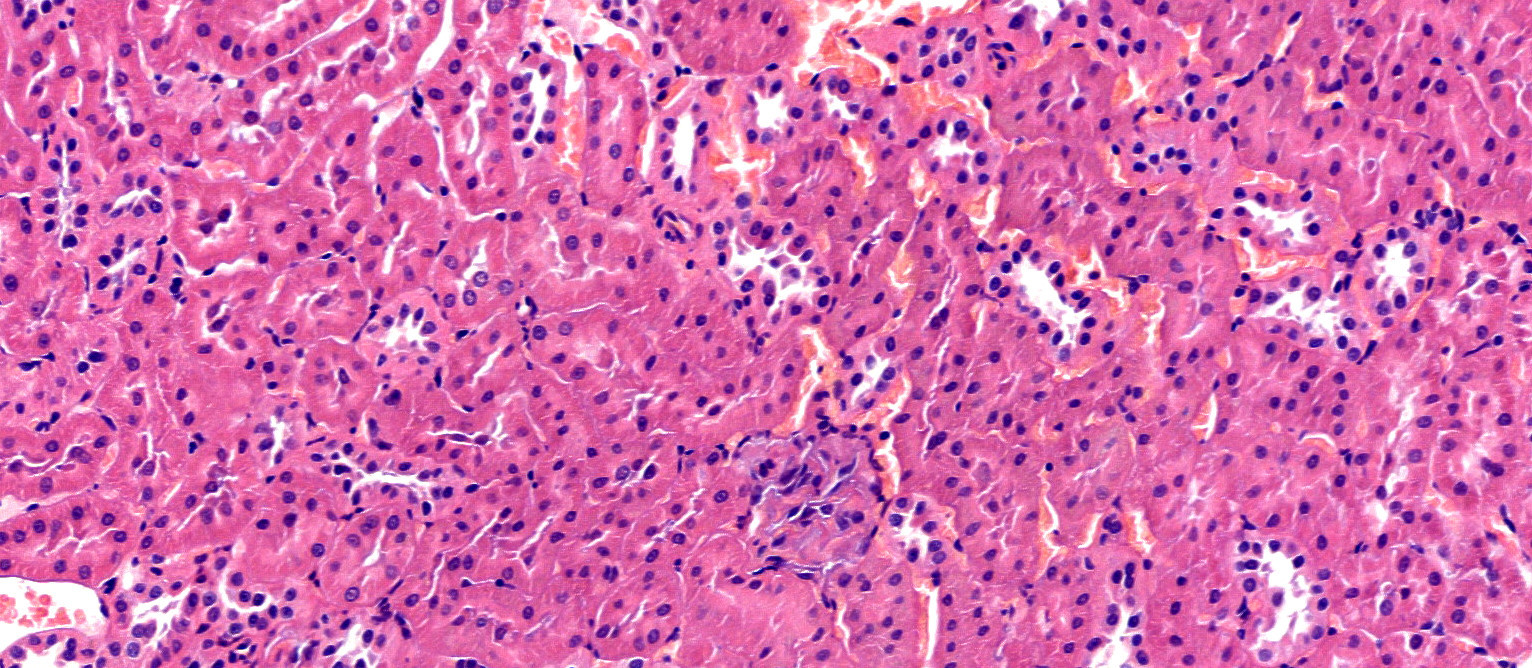

Supplement: Supplementary file 8 [file DataSheet1.ZIP › Fig 1D-HE-sham-4/4-3.jpeg]

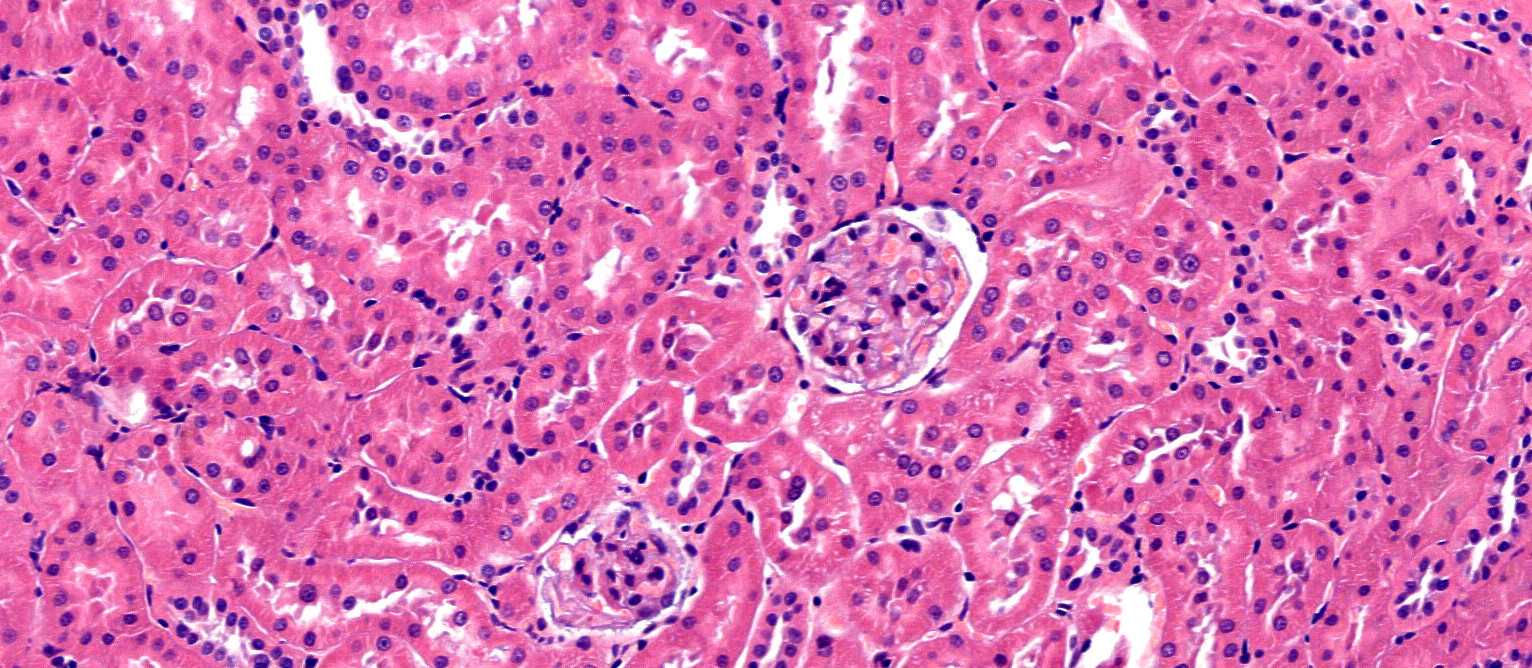

Supplement: Supplementary file 8 [file DataSheet1.ZIP › Fig 1D-HE-sham-4/4-4.jpeg]

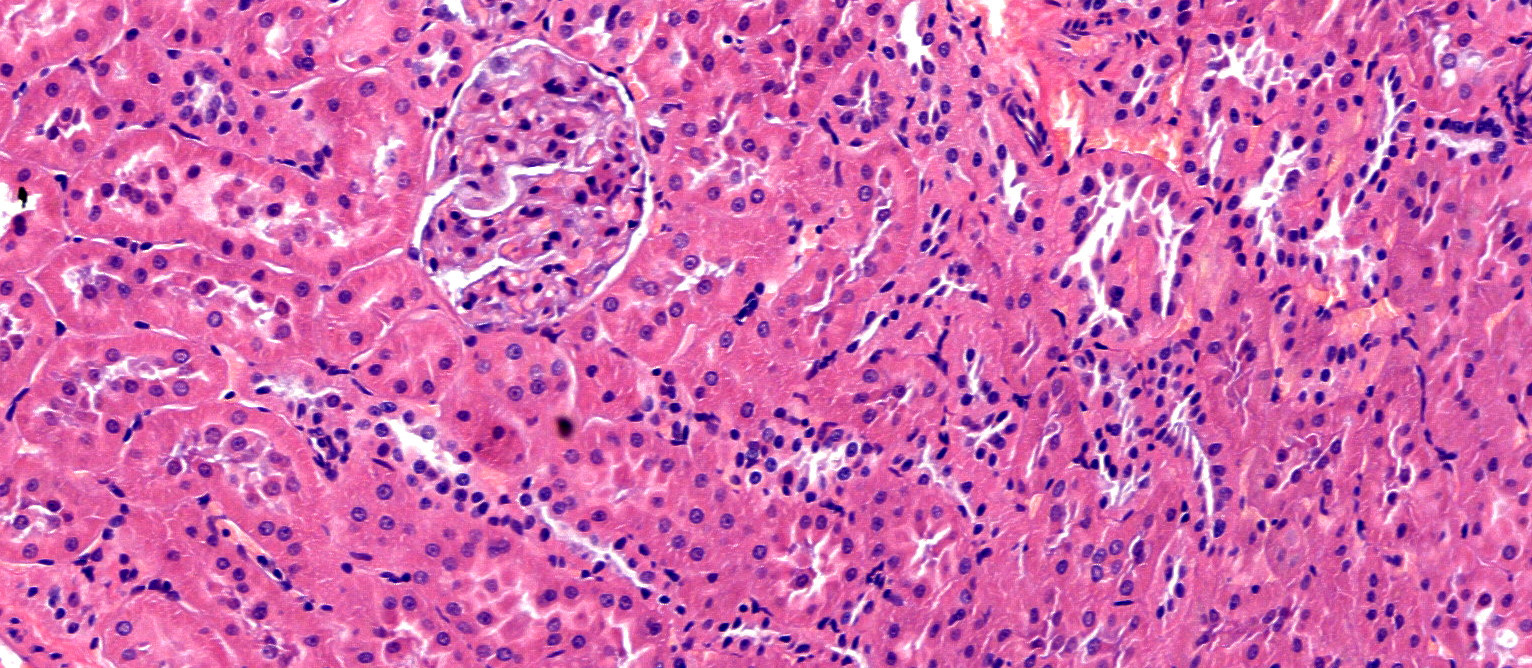

Supplement: Supplementary file 8 [file DataSheet1.ZIP › Fig 1D-HE-sham-4/4-5.jpeg]

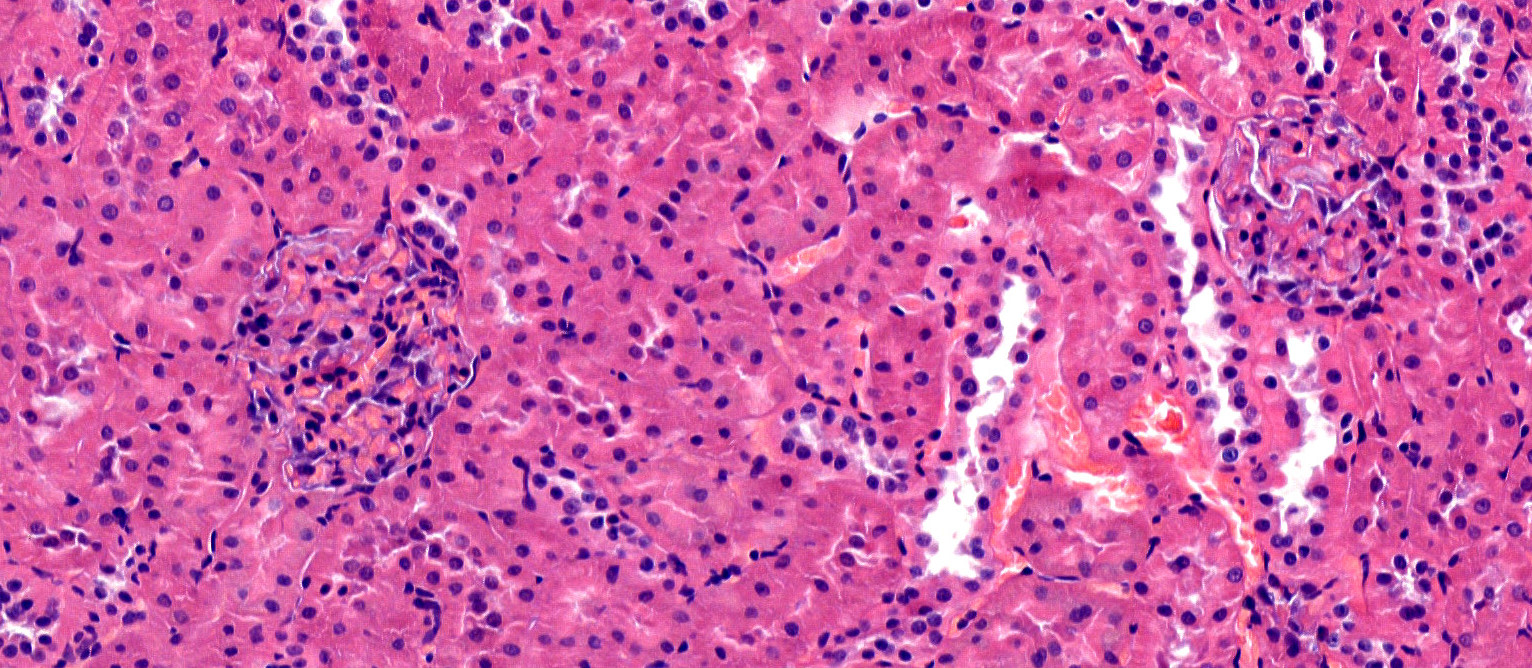

Supplement: Supplementary file 8 [file DataSheet1.ZIP › Fig 1D-HE-sham-4/4-6.jpeg]

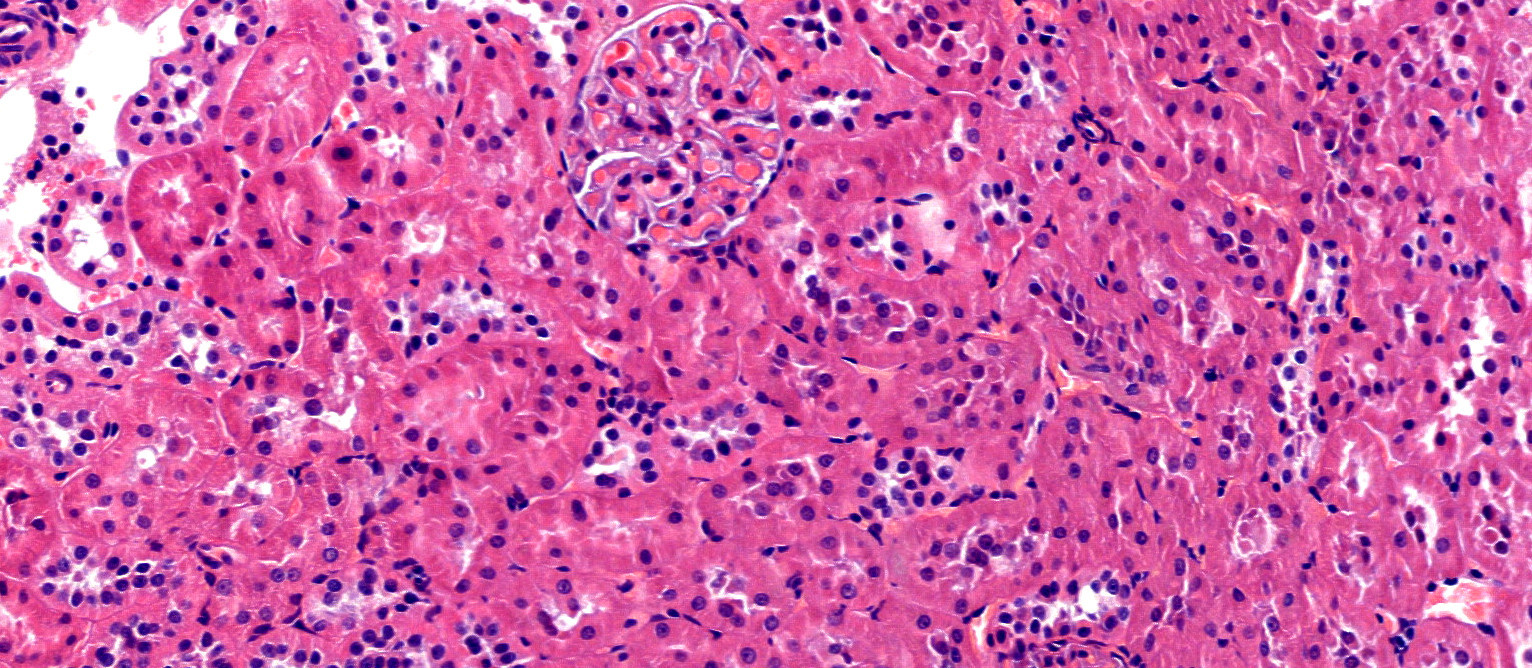

Supplement: Supplementary file 8 [file DataSheet1.ZIP › Fig 1D-HE-sham-4/4-7.jpeg]

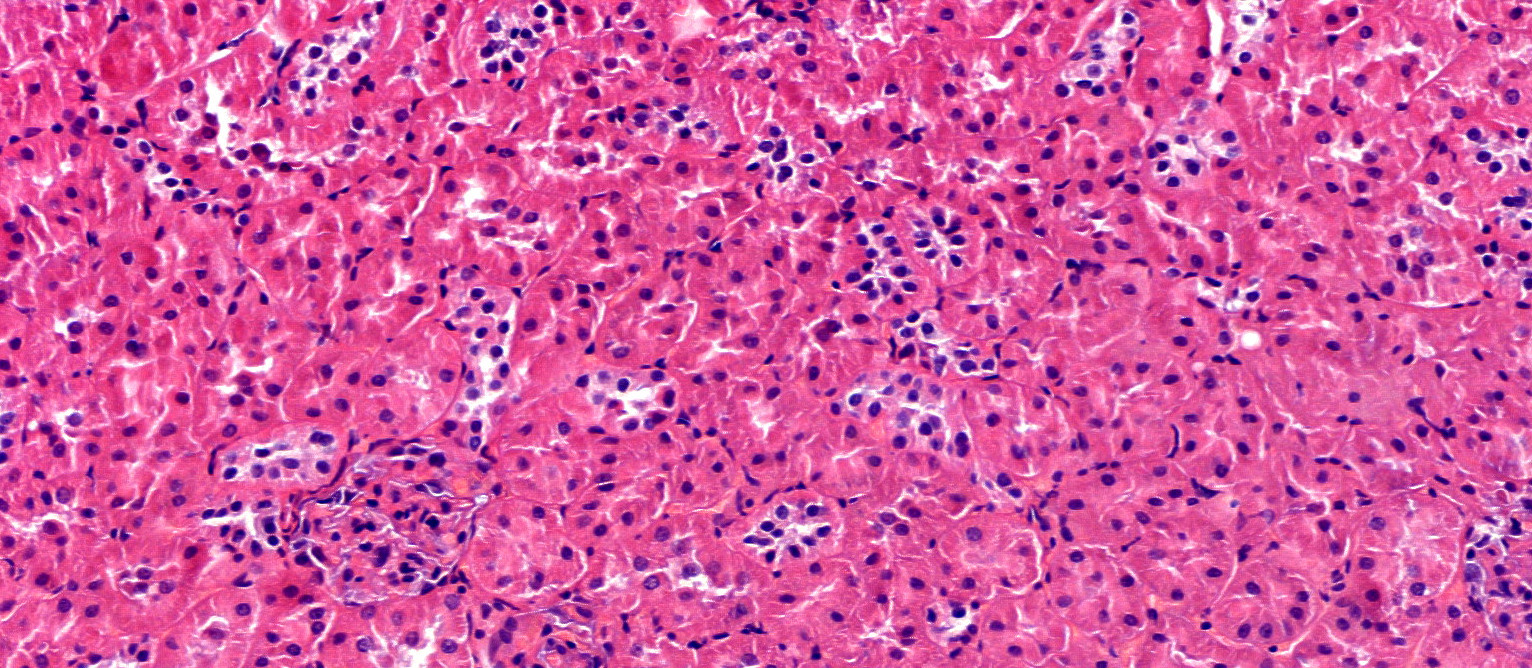

Supplement: Supplementary file 8 [file DataSheet1.ZIP › Fig 1D-HE-sham-4/4-8.jpeg]

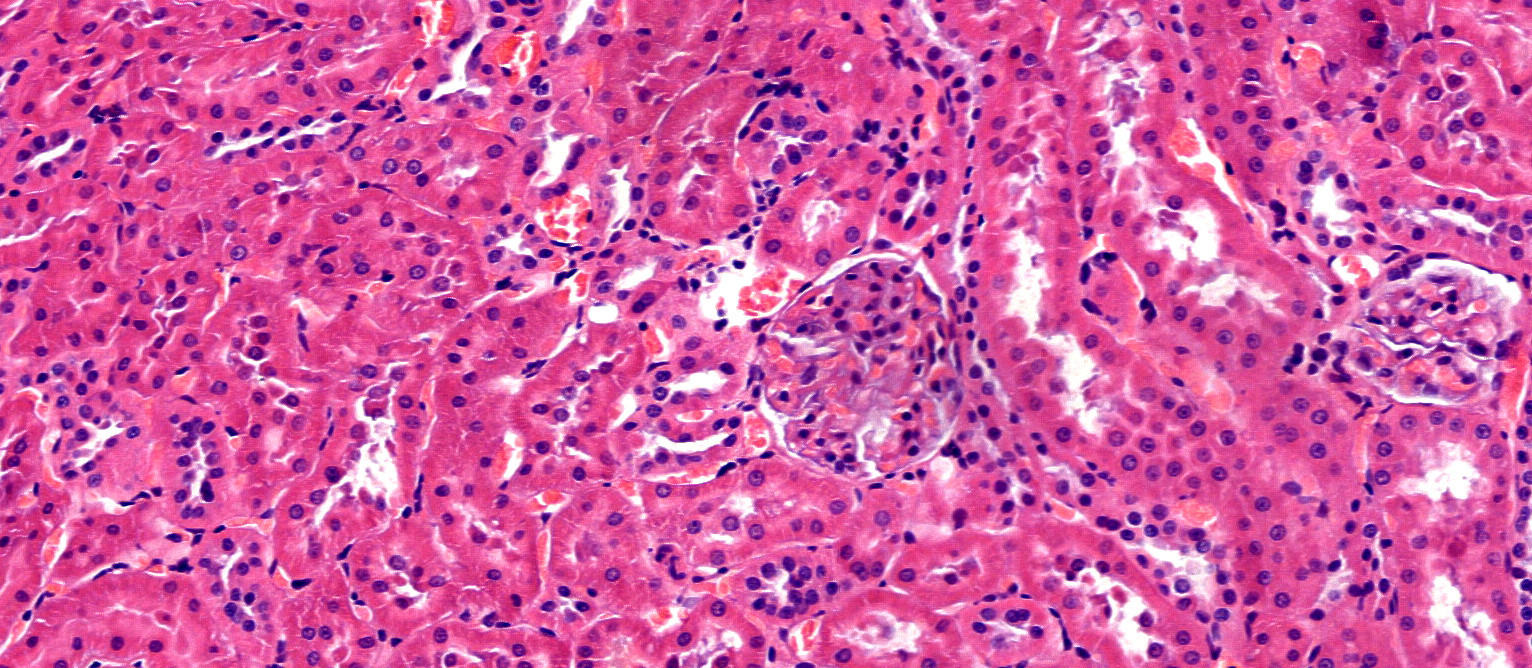

Supplement: Supplementary file 8 [file DataSheet1.ZIP › Fig 1D-HE-sham-4/4-9.jpeg]

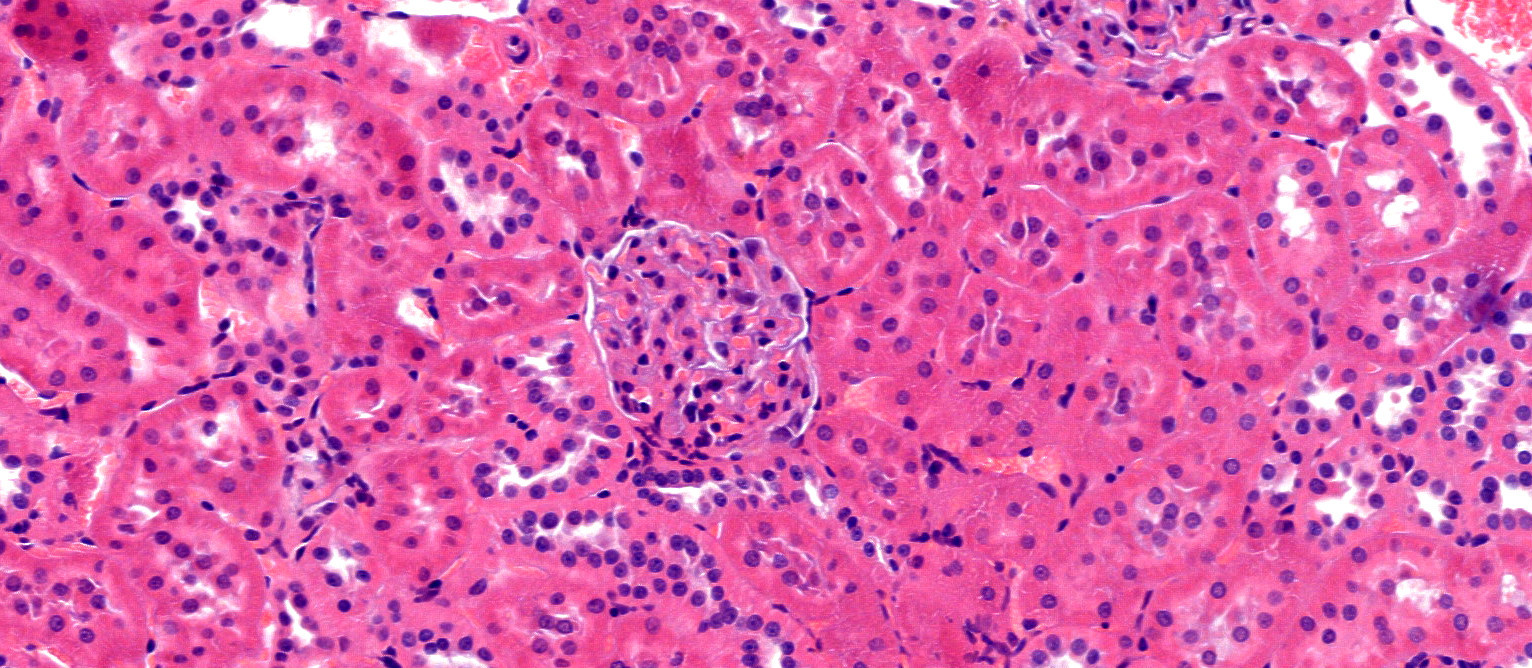

Supplement: Supplementary file 8 [file DataSheet1.ZIP › Fig 1D-HE-sham-5/5-1.jpeg]
