# Supplementary figures and images for: Characteristics of Serum Metabolites and Gut Microbiota in Diabetic Kidney Disease (part 6 of 13)
Source: Front Pharmacol. 2022 Apr 14;13:872988. doi: 10.3389/fphar.2022.872988 (PMC9084235; doi:10.3389/fphar.2022.872988)

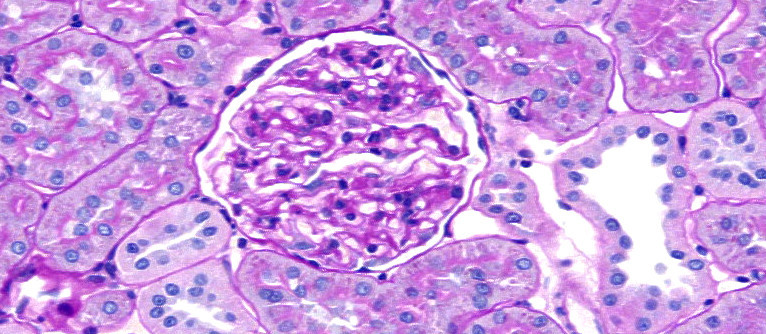

Supplement: Supplementary file 7 [file DataSheet13.ZIP › DKD/Fig 1D-PAS-DKD-17/17-10.jpeg]

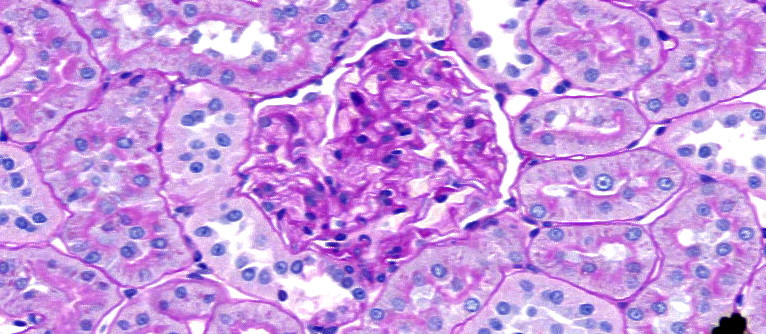

Supplement: Supplementary file 7 [file DataSheet13.ZIP › DKD/Fig 1D-PAS-DKD-17/17-11.jpeg]

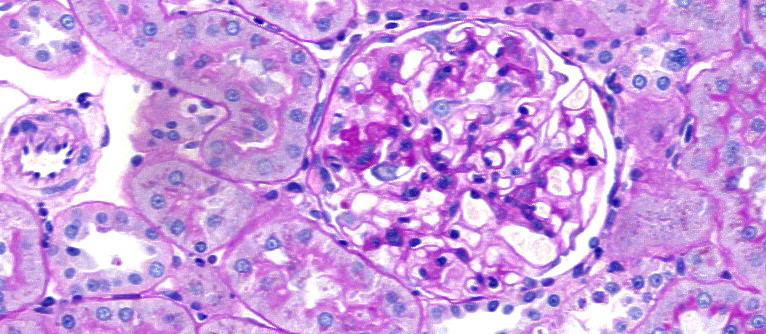

Supplement: Supplementary file 7 [file DataSheet13.ZIP › DKD/Fig 1D-PAS-DKD-17/17-12.jpeg]

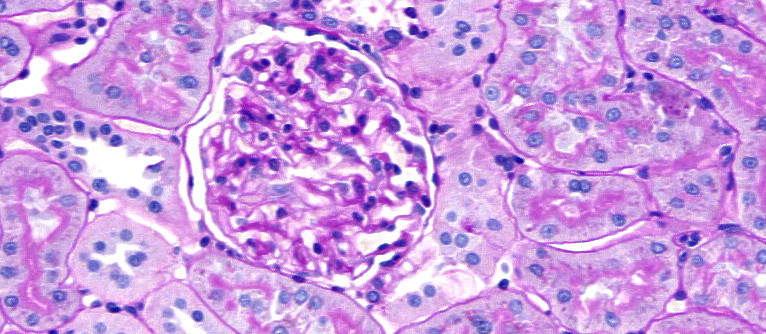

Supplement: Supplementary file 7 [file DataSheet13.ZIP › DKD/Fig 1D-PAS-DKD-17/17-13.jpeg]

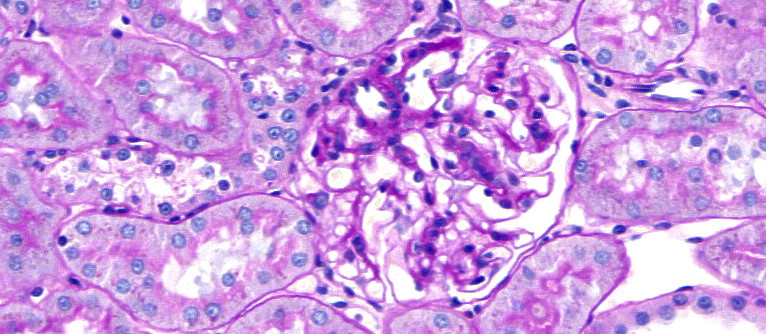

Supplement: Supplementary file 7 [file DataSheet13.ZIP › DKD/Fig 1D-PAS-DKD-17/17-14.jpeg]

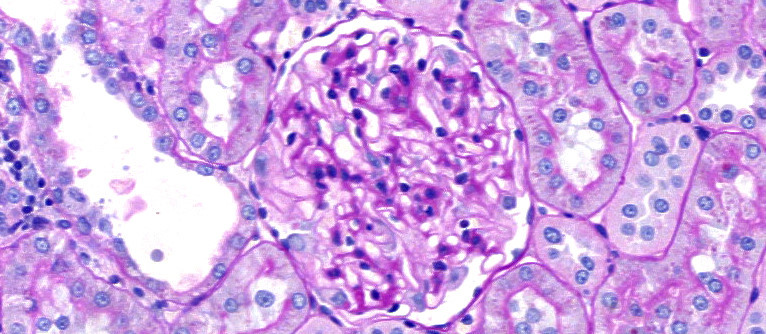

Supplement: Supplementary file 7 [file DataSheet13.ZIP › DKD/Fig 1D-PAS-DKD-17/17-15.jpeg]

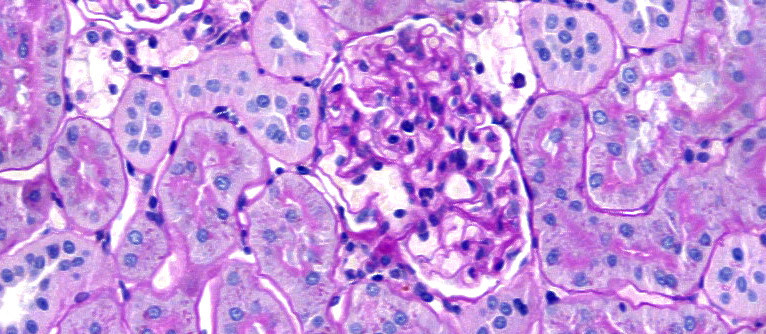

Supplement: Supplementary file 7 [file DataSheet13.ZIP › DKD/Fig 1D-PAS-DKD-17/17-16.jpeg]

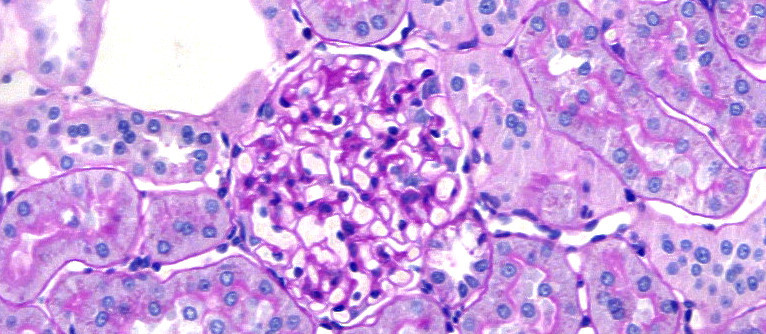

Supplement: Supplementary file 7 [file DataSheet13.ZIP › DKD/Fig 1D-PAS-DKD-17/17-17.jpeg]

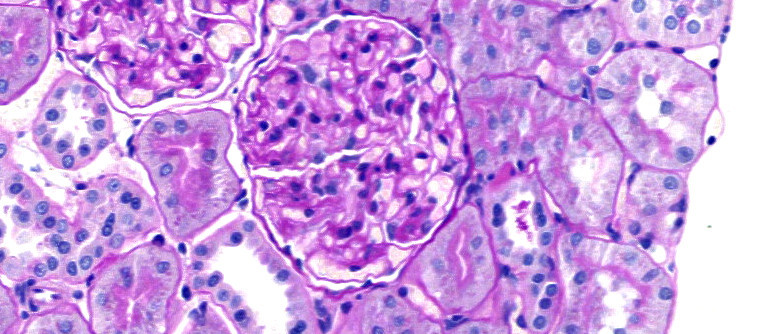

Supplement: Supplementary file 7 [file DataSheet13.ZIP › DKD/Fig 1D-PAS-DKD-17/17-18.jpeg]

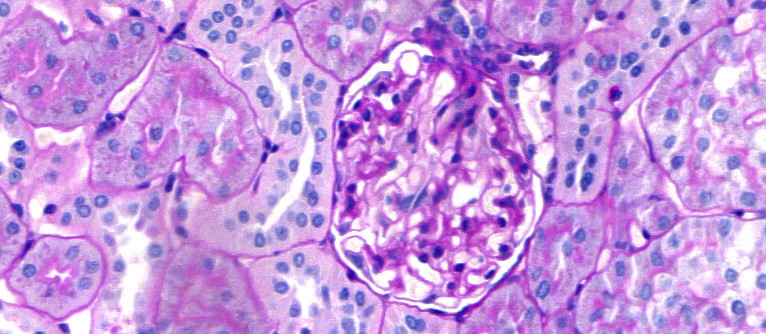

Supplement: Supplementary file 7 [file DataSheet13.ZIP › DKD/Fig 1D-PAS-DKD-17/17-19.jpeg]

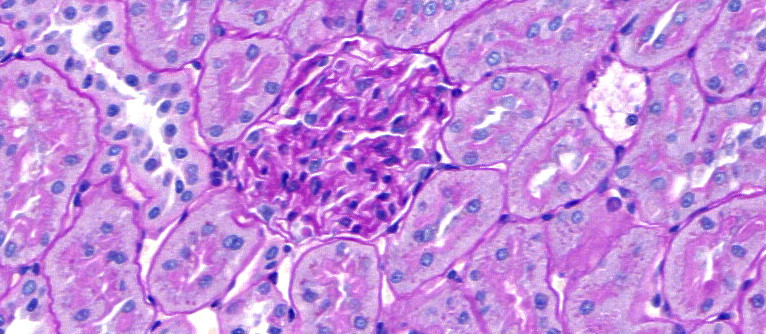

Supplement: Supplementary file 7 [file DataSheet13.ZIP › DKD/Fig 1D-PAS-DKD-17/17-2.jpeg]

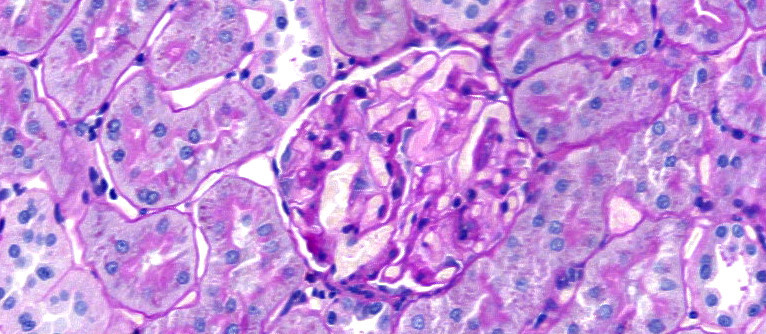

Supplement: Supplementary file 7 [file DataSheet13.ZIP › DKD/Fig 1D-PAS-DKD-17/17-20.jpeg]

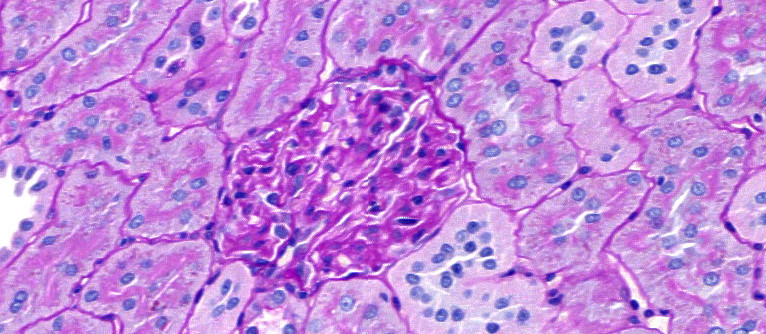

Supplement: Supplementary file 7 [file DataSheet13.ZIP › DKD/Fig 1D-PAS-DKD-17/17-3.jpeg]

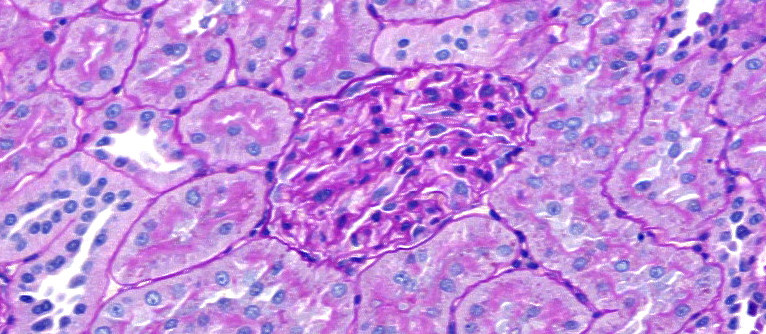

Supplement: Supplementary file 7 [file DataSheet13.ZIP › DKD/Fig 1D-PAS-DKD-17/17-4.jpeg]

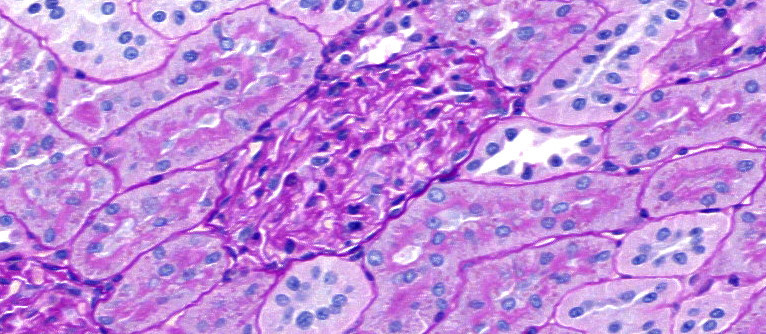

Supplement: Supplementary file 7 [file DataSheet13.ZIP › DKD/Fig 1D-PAS-DKD-17/17-5.jpeg]

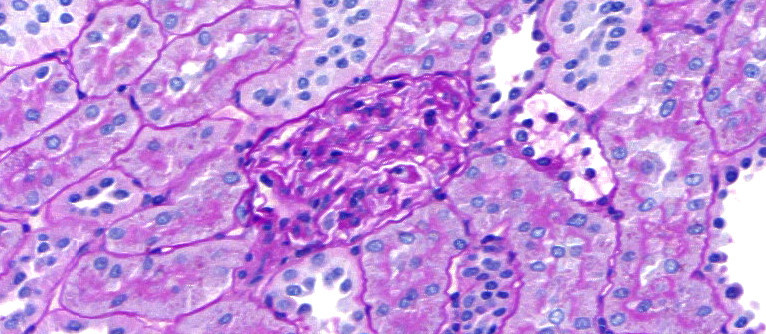

Supplement: Supplementary file 7 [file DataSheet13.ZIP › DKD/Fig 1D-PAS-DKD-17/17-6.jpeg]

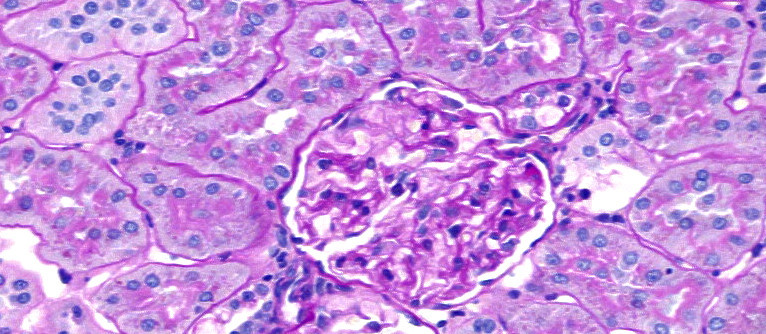

Supplement: Supplementary file 7 [file DataSheet13.ZIP › DKD/Fig 1D-PAS-DKD-17/17-7.jpeg]

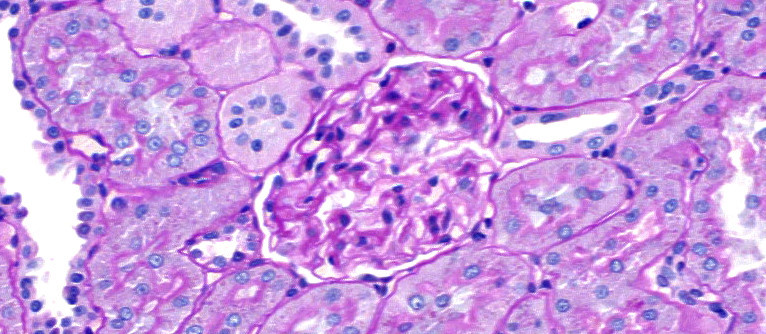

Supplement: Supplementary file 7 [file DataSheet13.ZIP › DKD/Fig 1D-PAS-DKD-17/17-8.jpeg]

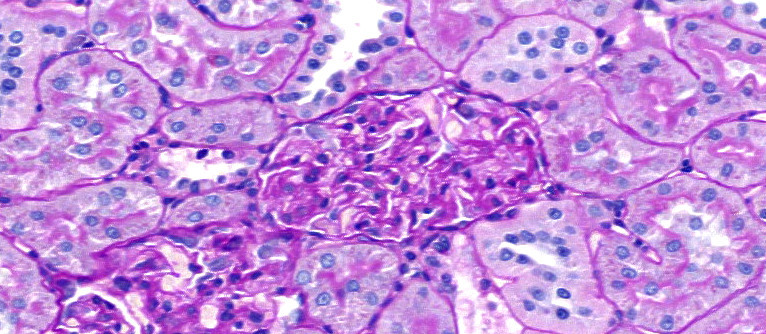

Supplement: Supplementary file 7 [file DataSheet13.ZIP › DKD/Fig 1D-PAS-DKD-17/17-9.jpeg]

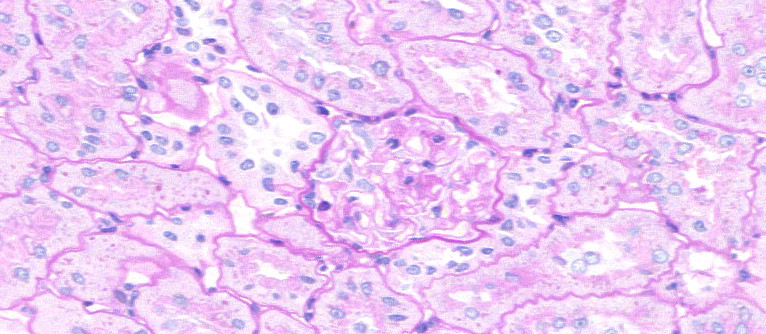

Supplement: Supplementary file 7 [file DataSheet13.ZIP › DKD/Fig 1D-PAS-DKD-18/18-1.jpeg]

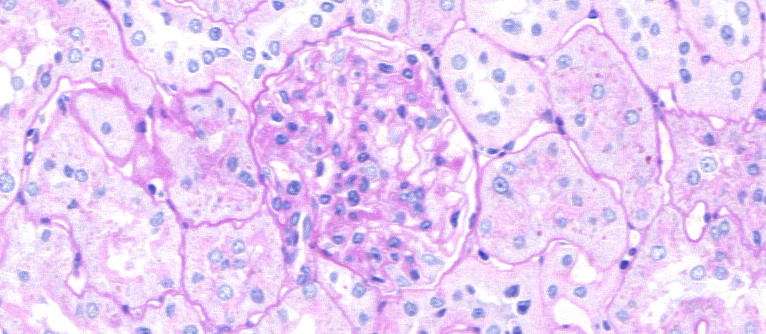

Supplement: Supplementary file 7 [file DataSheet13.ZIP › DKD/Fig 1D-PAS-DKD-18/18-10.jpeg]

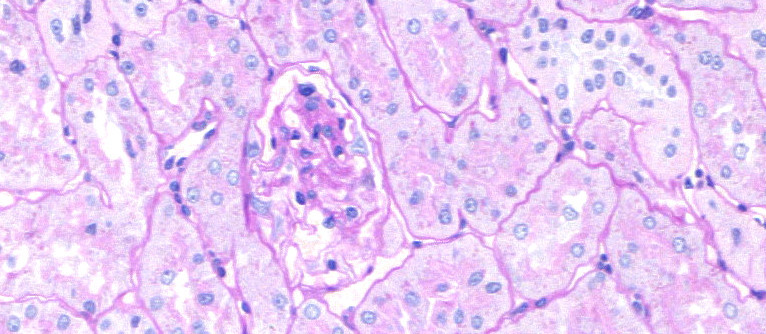

Supplement: Supplementary file 7 [file DataSheet13.ZIP › DKD/Fig 1D-PAS-DKD-18/18-11.jpeg]

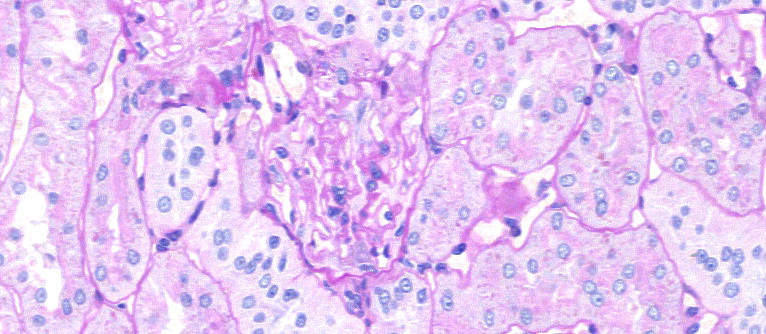

Supplement: Supplementary file 7 [file DataSheet13.ZIP › DKD/Fig 1D-PAS-DKD-18/18-12.jpeg]

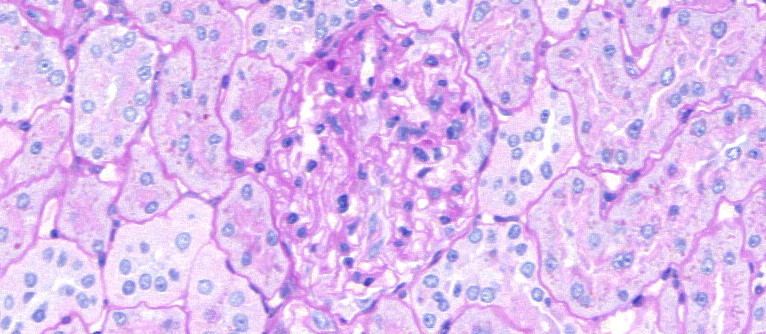

Supplement: Supplementary file 7 [file DataSheet13.ZIP › DKD/Fig 1D-PAS-DKD-18/18-13.jpeg]

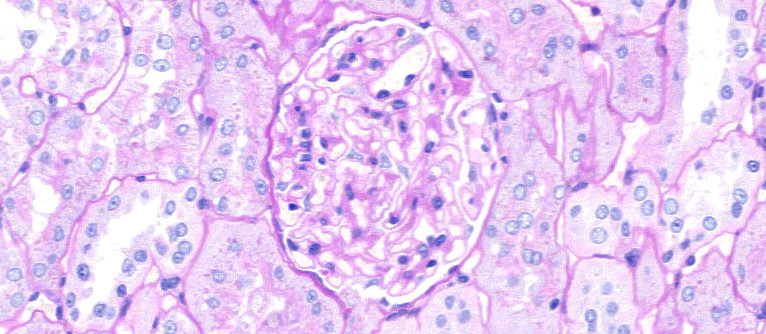

Supplement: Supplementary file 7 [file DataSheet13.ZIP › DKD/Fig 1D-PAS-DKD-18/18-14.jpeg]

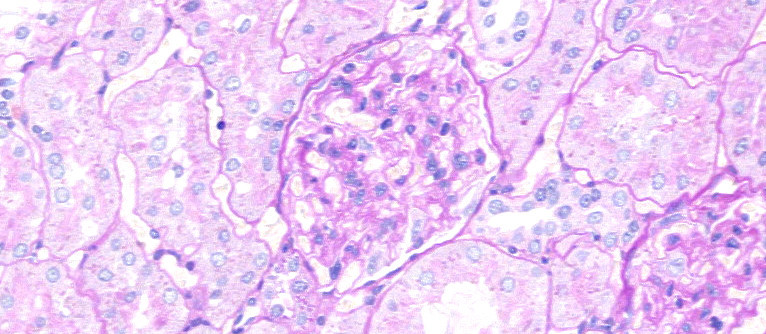

Supplement: Supplementary file 7 [file DataSheet13.ZIP › DKD/Fig 1D-PAS-DKD-18/18-15.jpeg]

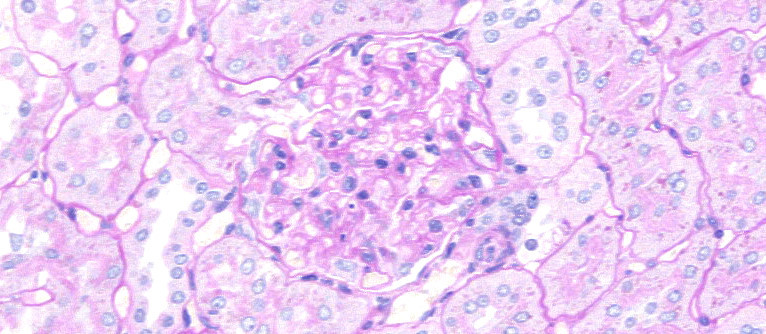

Supplement: Supplementary file 7 [file DataSheet13.ZIP › DKD/Fig 1D-PAS-DKD-18/18-16.jpeg]

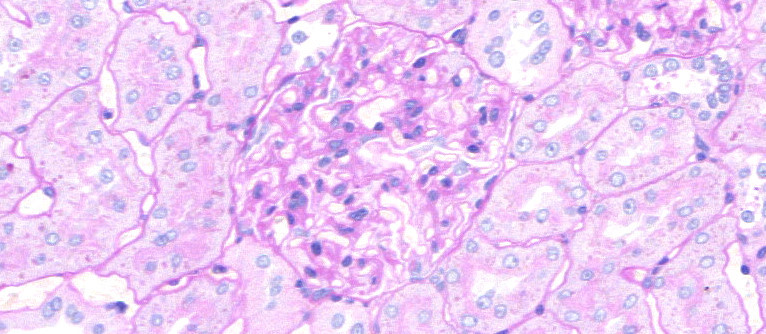

Supplement: Supplementary file 7 [file DataSheet13.ZIP › DKD/Fig 1D-PAS-DKD-18/18-17.jpeg]

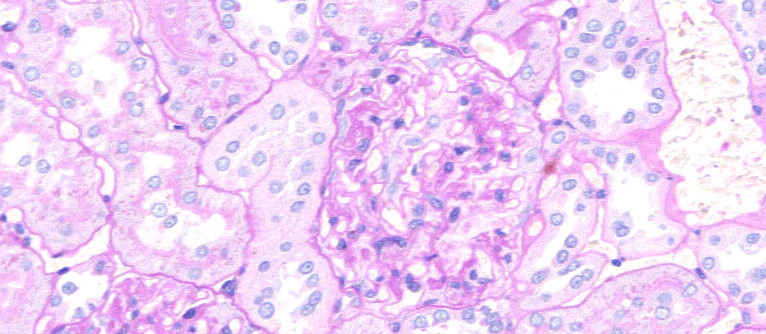

Supplement: Supplementary file 7 [file DataSheet13.ZIP › DKD/Fig 1D-PAS-DKD-18/18-18.jpeg]

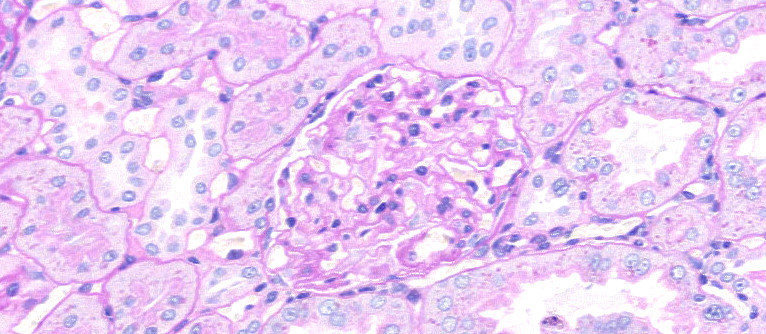

Supplement: Supplementary file 7 [file DataSheet13.ZIP › DKD/Fig 1D-PAS-DKD-18/18-19.jpeg]

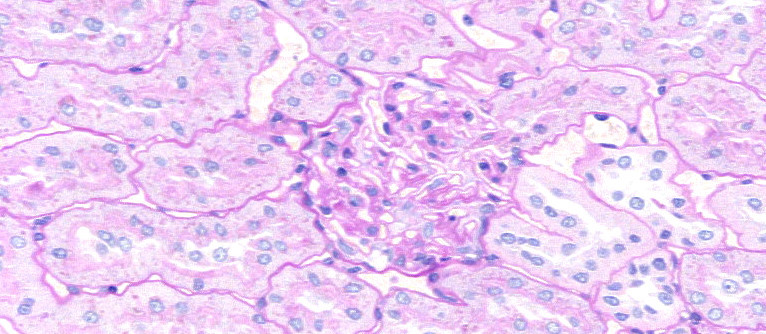

Supplement: Supplementary file 7 [file DataSheet13.ZIP › DKD/Fig 1D-PAS-DKD-18/18-2.jpeg]

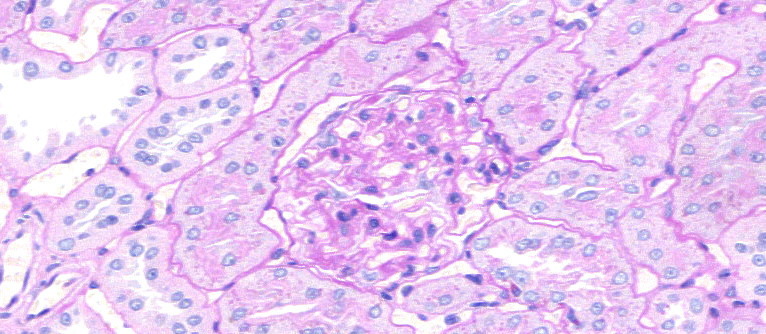

Supplement: Supplementary file 7 [file DataSheet13.ZIP › DKD/Fig 1D-PAS-DKD-18/18-20.jpeg]

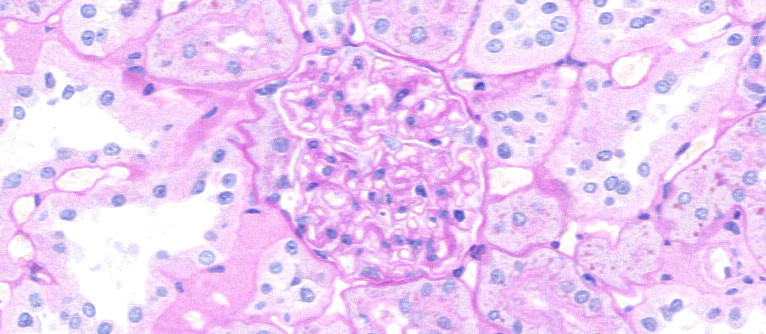

Supplement: Supplementary file 7 [file DataSheet13.ZIP › DKD/Fig 1D-PAS-DKD-18/18-3.jpeg]

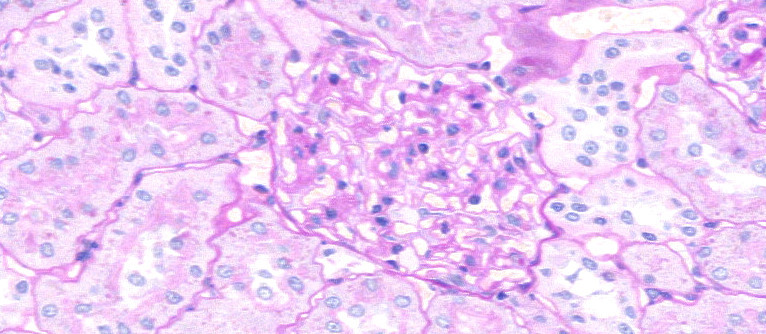

Supplement: Supplementary file 7 [file DataSheet13.ZIP › DKD/Fig 1D-PAS-DKD-18/18-4.jpeg]

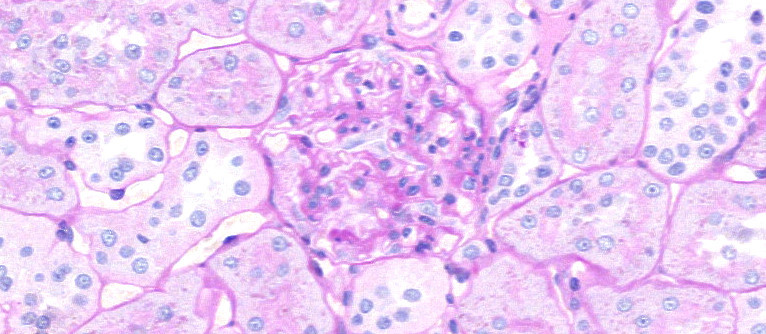

Supplement: Supplementary file 7 [file DataSheet13.ZIP › DKD/Fig 1D-PAS-DKD-18/18-5.jpeg]

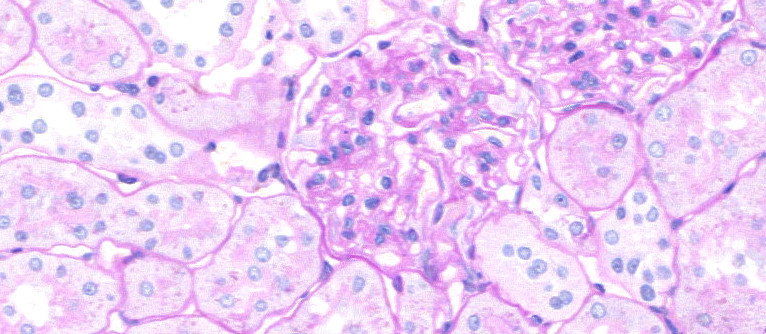

Supplement: Supplementary file 7 [file DataSheet13.ZIP › DKD/Fig 1D-PAS-DKD-18/18-6.jpeg]

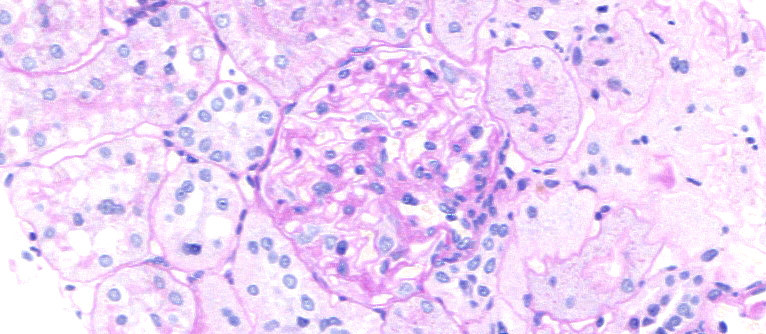

Supplement: Supplementary file 7 [file DataSheet13.ZIP › DKD/Fig 1D-PAS-DKD-18/18-7.jpeg]

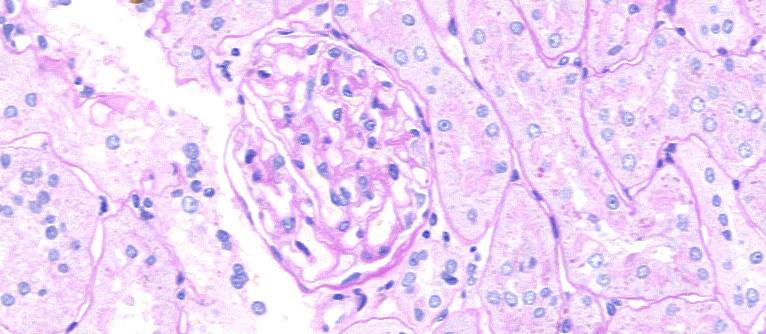

Supplement: Supplementary file 7 [file DataSheet13.ZIP › DKD/Fig 1D-PAS-DKD-18/18-8.jpeg]

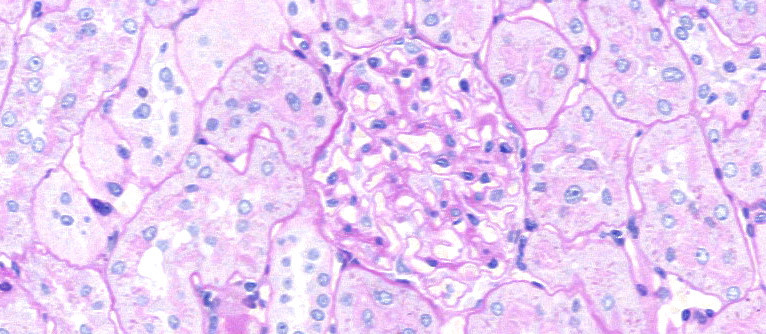

Supplement: Supplementary file 7 [file DataSheet13.ZIP › DKD/Fig 1D-PAS-DKD-18/18-9.jpeg]

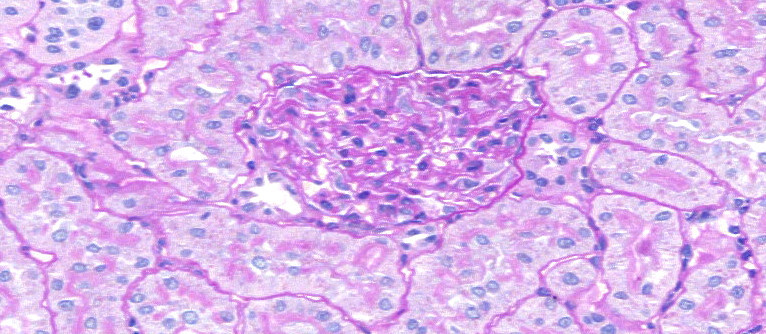

Supplement: Supplementary file 7 [file DataSheet13.ZIP › DKD/Fig 1D-PAS-DKD-20/20-1.jpeg]

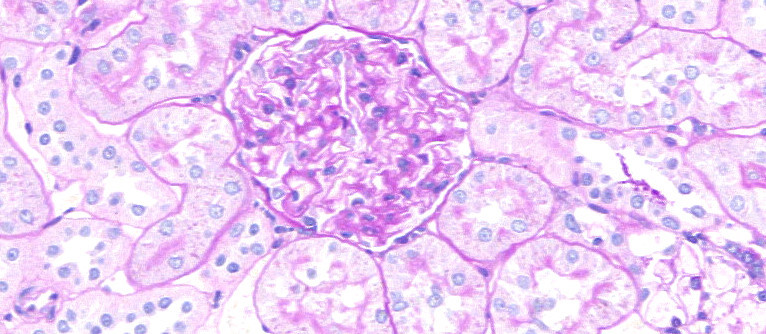

Supplement: Supplementary file 7 [file DataSheet13.ZIP › DKD/Fig 1D-PAS-DKD-20/20-10.jpeg]

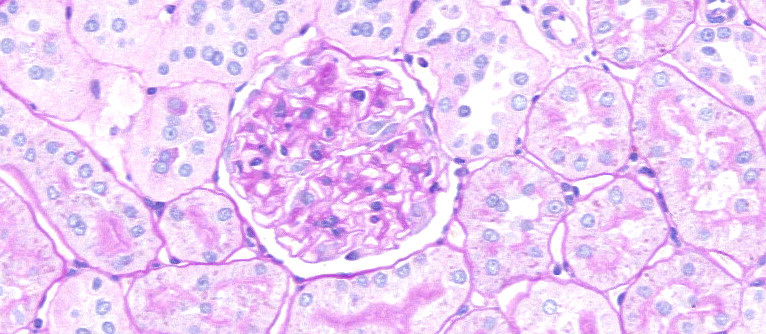

Supplement: Supplementary file 7 [file DataSheet13.ZIP › DKD/Fig 1D-PAS-DKD-20/20-11.jpeg]

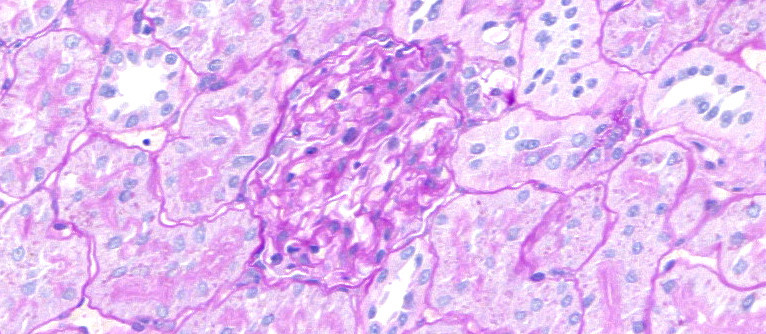

Supplement: Supplementary file 7 [file DataSheet13.ZIP › DKD/Fig 1D-PAS-DKD-20/20-12.jpeg]

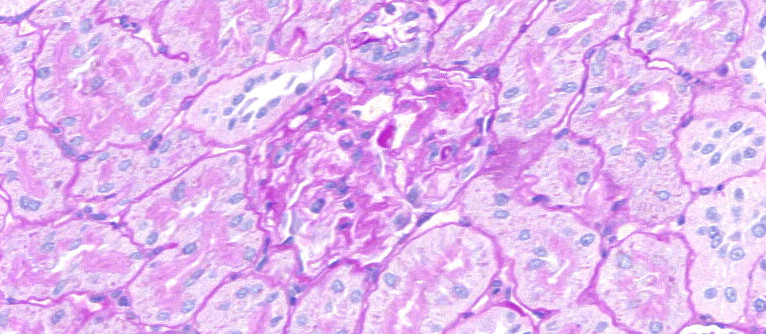

Supplement: Supplementary file 7 [file DataSheet13.ZIP › DKD/Fig 1D-PAS-DKD-20/20-13.jpeg]

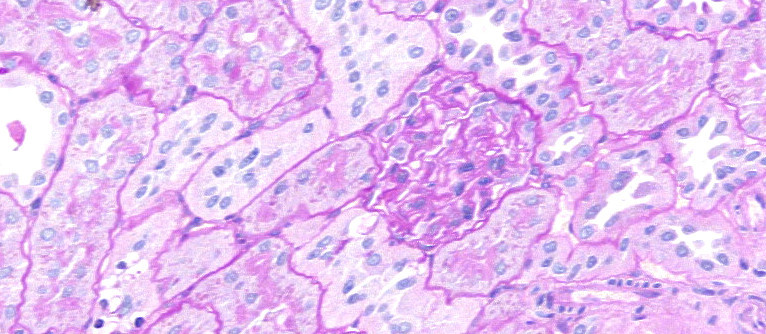

Supplement: Supplementary file 7 [file DataSheet13.ZIP › DKD/Fig 1D-PAS-DKD-20/20-14.jpeg]

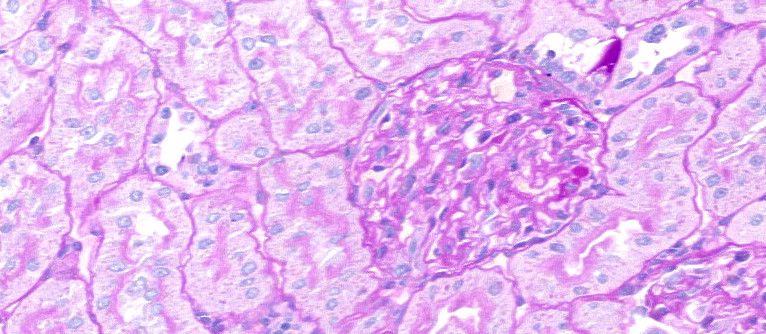

Supplement: Supplementary file 7 [file DataSheet13.ZIP › DKD/Fig 1D-PAS-DKD-20/20-15.jpeg]

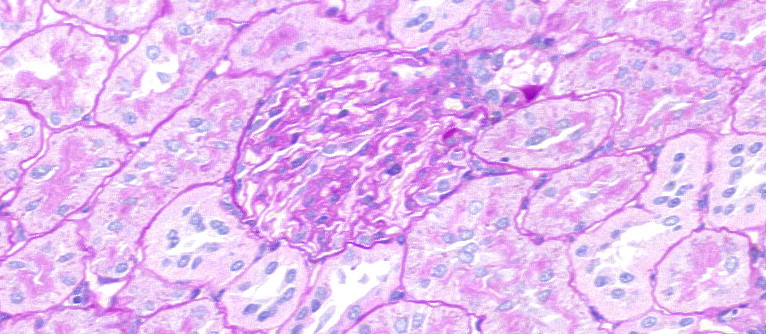

Supplement: Supplementary file 7 [file DataSheet13.ZIP › DKD/Fig 1D-PAS-DKD-20/20-16.jpeg]

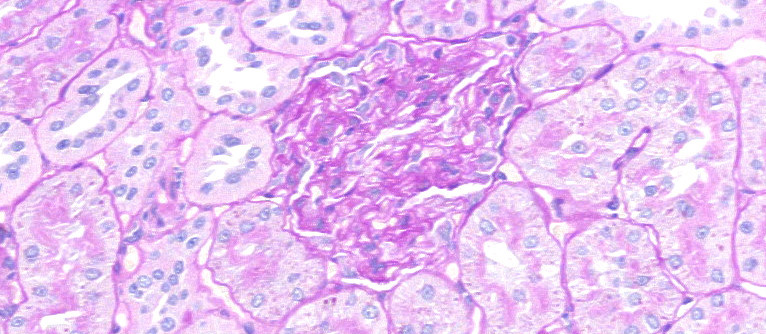

Supplement: Supplementary file 7 [file DataSheet13.ZIP › DKD/Fig 1D-PAS-DKD-20/20-17.jpeg]

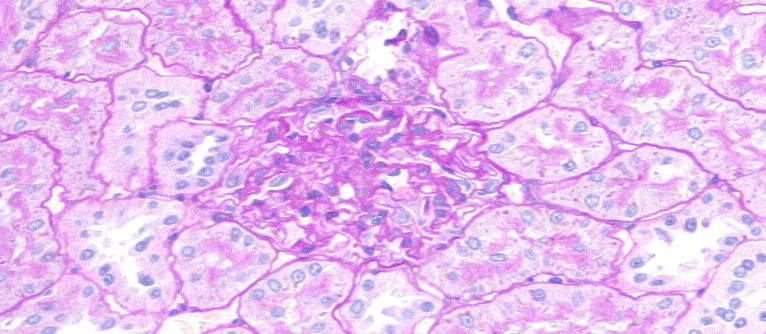

Supplement: Supplementary file 7 [file DataSheet13.ZIP › DKD/Fig 1D-PAS-DKD-20/20-18.jpeg]

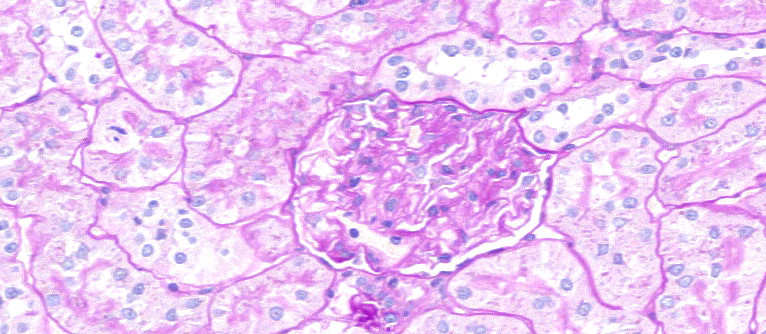

Supplement: Supplementary file 7 [file DataSheet13.ZIP › DKD/Fig 1D-PAS-DKD-20/20-19.jpeg]

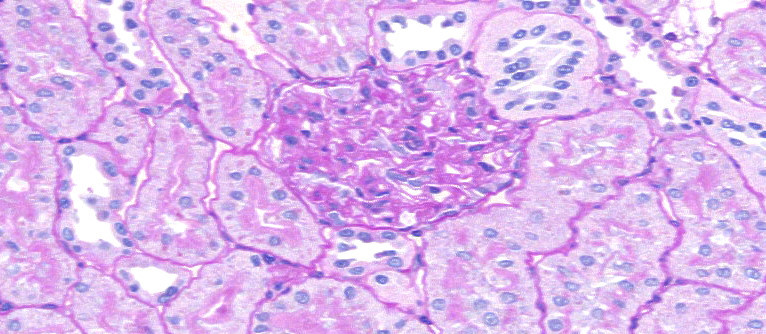

Supplement: Supplementary file 7 [file DataSheet13.ZIP › DKD/Fig 1D-PAS-DKD-20/20-2.jpeg]

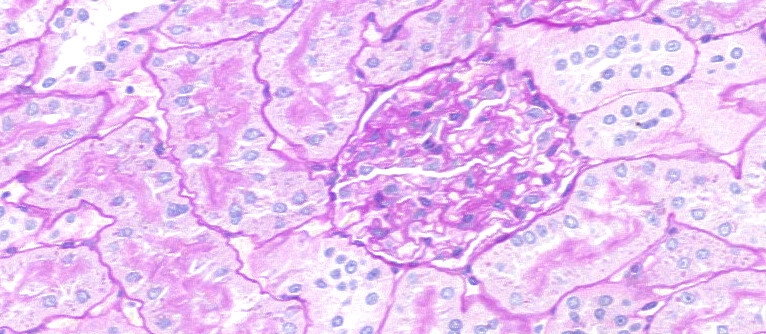

Supplement: Supplementary file 7 [file DataSheet13.ZIP › DKD/Fig 1D-PAS-DKD-20/20-20.jpeg]

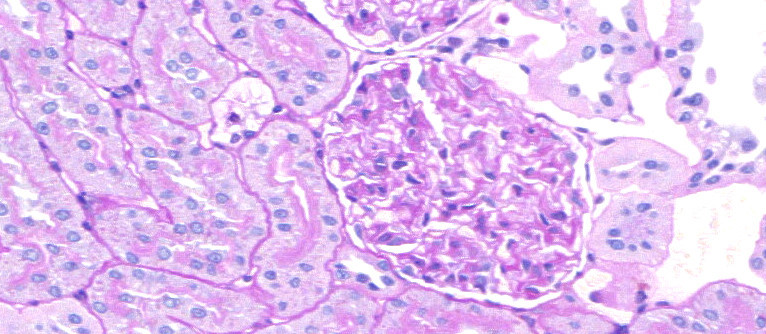

Supplement: Supplementary file 7 [file DataSheet13.ZIP › DKD/Fig 1D-PAS-DKD-20/20-3.jpeg]

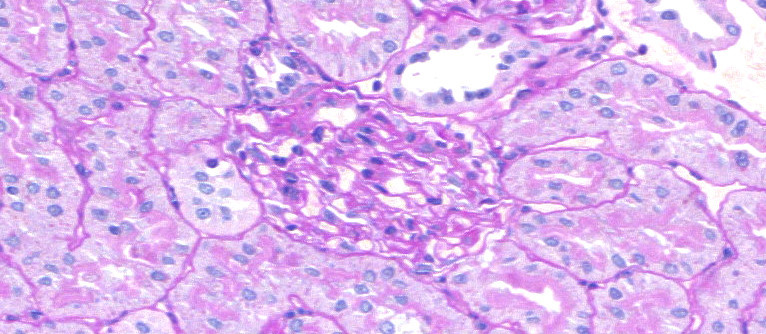

Supplement: Supplementary file 7 [file DataSheet13.ZIP › DKD/Fig 1D-PAS-DKD-20/20-4.jpeg]

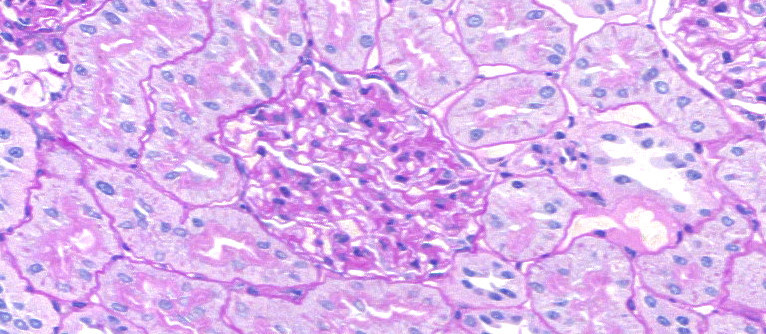

Supplement: Supplementary file 7 [file DataSheet13.ZIP › DKD/Fig 1D-PAS-DKD-20/20-5.jpeg]

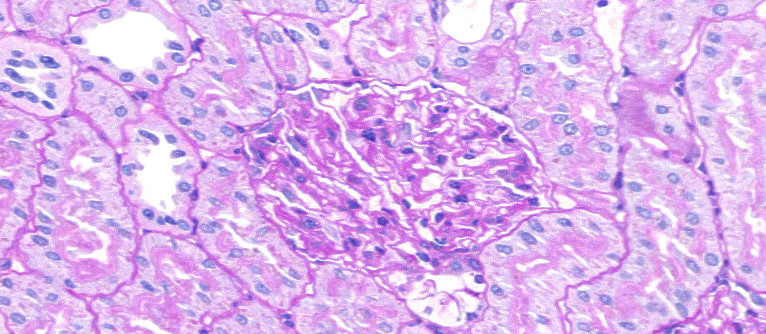

Supplement: Supplementary file 7 [file DataSheet13.ZIP › DKD/Fig 1D-PAS-DKD-20/20-6.jpeg]

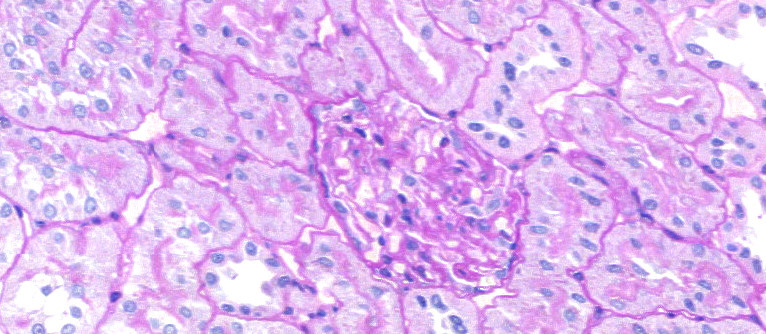

Supplement: Supplementary file 7 [file DataSheet13.ZIP › DKD/Fig 1D-PAS-DKD-20/20-7.jpeg]

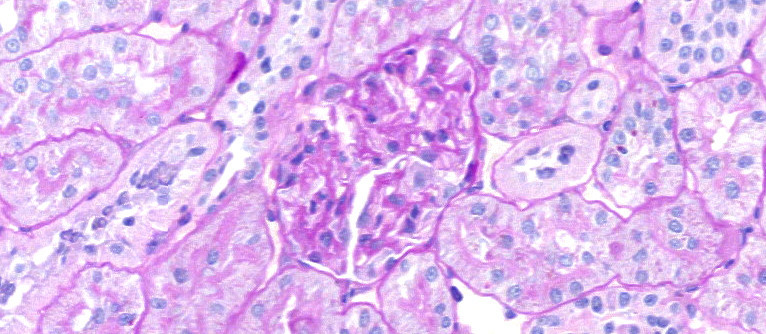

Supplement: Supplementary file 7 [file DataSheet13.ZIP › DKD/Fig 1D-PAS-DKD-20/20-8.jpeg]

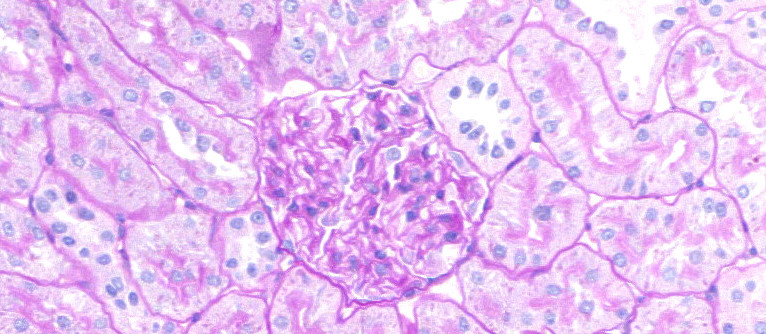

Supplement: Supplementary file 7 [file DataSheet13.ZIP › DKD/Fig 1D-PAS-DKD-20/20-9.jpeg]

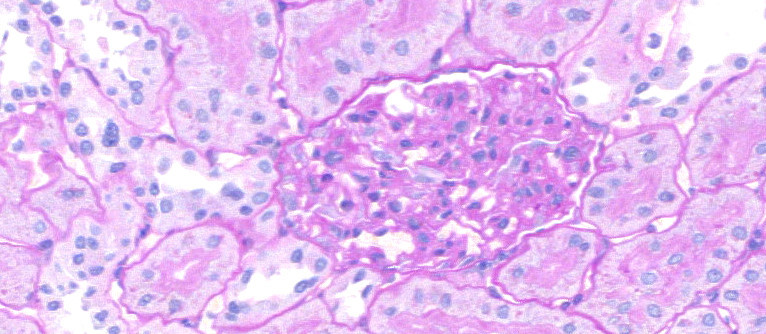

Supplement: Supplementary file 7 [file DataSheet13.ZIP › DKD/Fig 1D-PAS-DKD-21/21-1.jpeg]

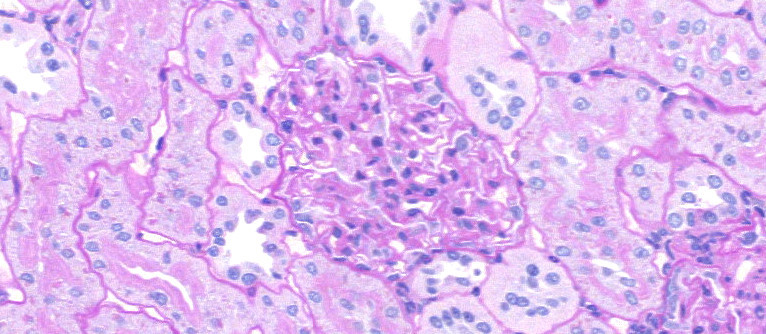

Supplement: Supplementary file 7 [file DataSheet13.ZIP › DKD/Fig 1D-PAS-DKD-21/21-10.jpeg]

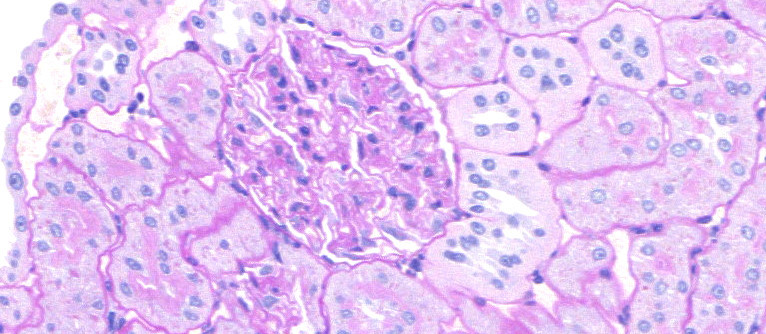

Supplement: Supplementary file 7 [file DataSheet13.ZIP › DKD/Fig 1D-PAS-DKD-21/21-11.jpeg]

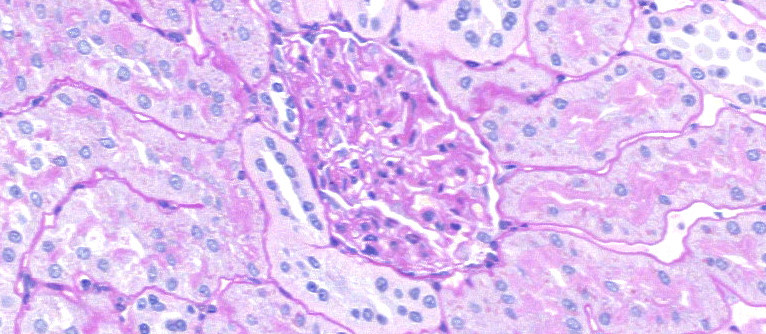

Supplement: Supplementary file 7 [file DataSheet13.ZIP › DKD/Fig 1D-PAS-DKD-21/21-12.jpeg]

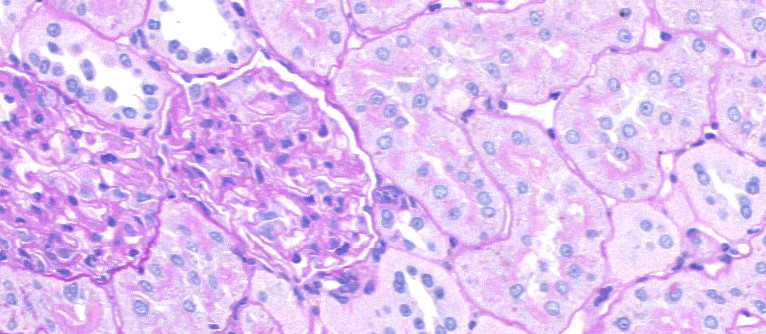

Supplement: Supplementary file 7 [file DataSheet13.ZIP › DKD/Fig 1D-PAS-DKD-21/21-13.jpeg]

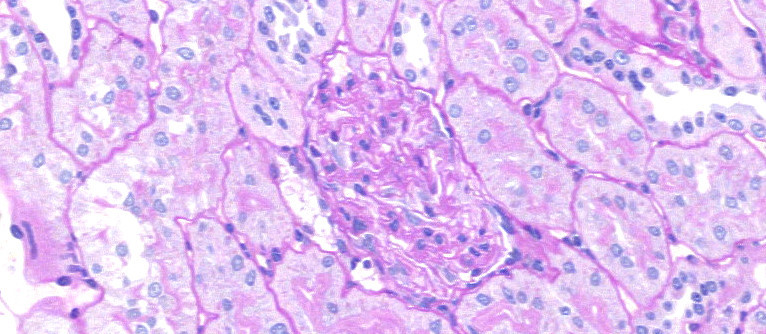

Supplement: Supplementary file 7 [file DataSheet13.ZIP › DKD/Fig 1D-PAS-DKD-21/21-14.jpeg]

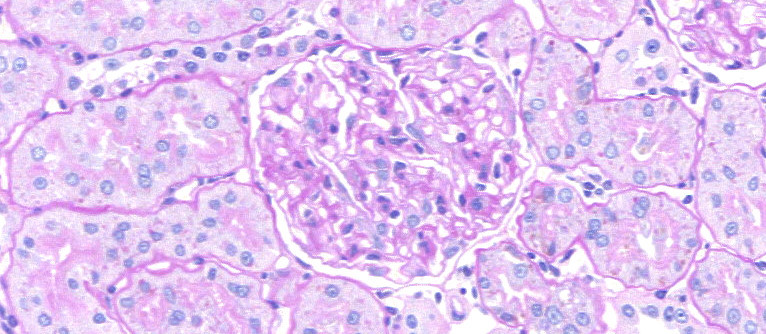

Supplement: Supplementary file 7 [file DataSheet13.ZIP › DKD/Fig 1D-PAS-DKD-21/21-15.jpeg]

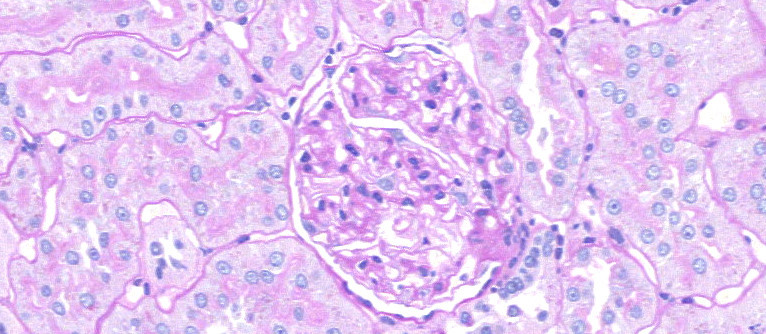

Supplement: Supplementary file 7 [file DataSheet13.ZIP › DKD/Fig 1D-PAS-DKD-21/21-16.jpeg]

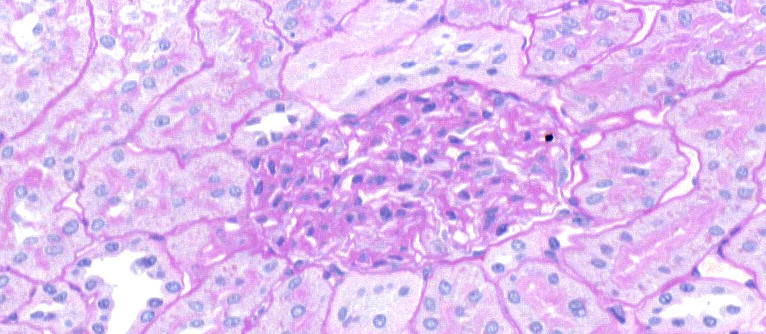

Supplement: Supplementary file 7 [file DataSheet13.ZIP › DKD/Fig 1D-PAS-DKD-21/21-17.jpeg]

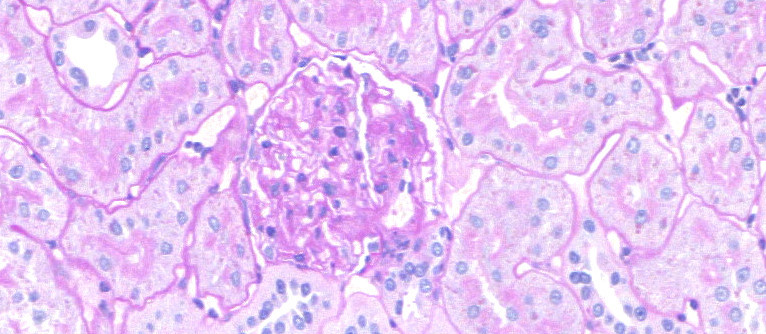

Supplement: Supplementary file 7 [file DataSheet13.ZIP › DKD/Fig 1D-PAS-DKD-21/21-18.jpeg]

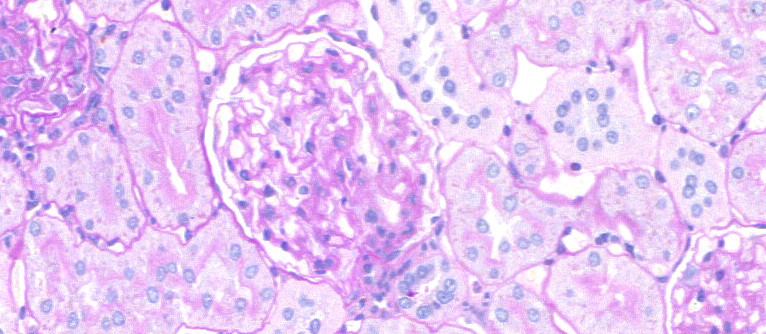

Supplement: Supplementary file 7 [file DataSheet13.ZIP › DKD/Fig 1D-PAS-DKD-21/21-19.jpeg]

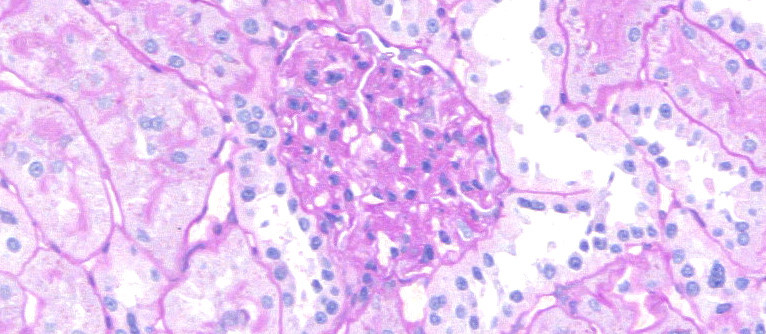

Supplement: Supplementary file 7 [file DataSheet13.ZIP › DKD/Fig 1D-PAS-DKD-21/21-2.jpeg]

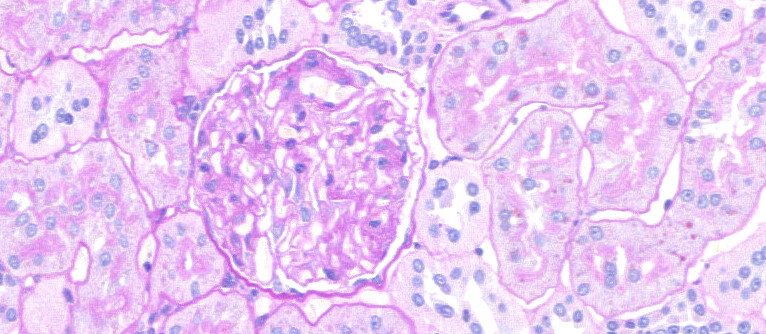

Supplement: Supplementary file 7 [file DataSheet13.ZIP › DKD/Fig 1D-PAS-DKD-21/21-20.jpeg]

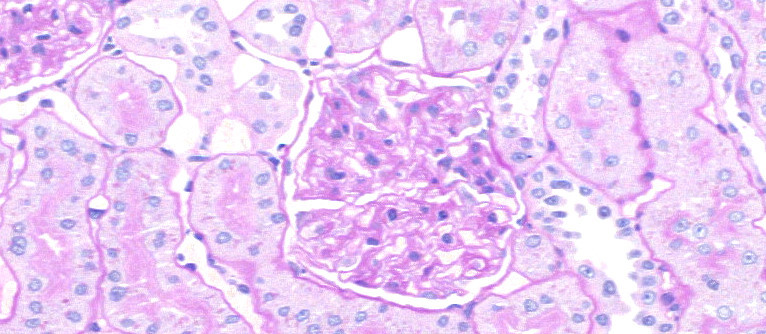

Supplement: Supplementary file 7 [file DataSheet13.ZIP › DKD/Fig 1D-PAS-DKD-21/21-3.jpeg]

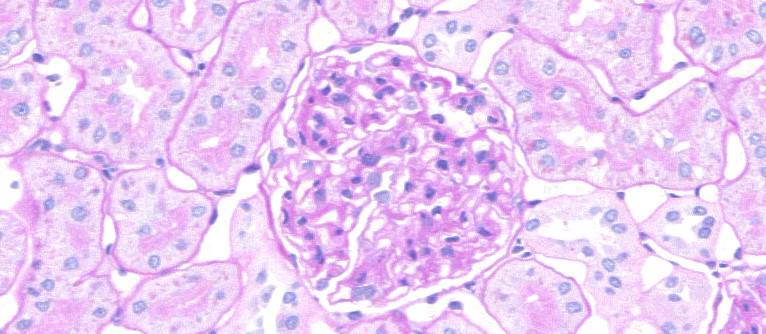

Supplement: Supplementary file 7 [file DataSheet13.ZIP › DKD/Fig 1D-PAS-DKD-21/21-4.jpeg]

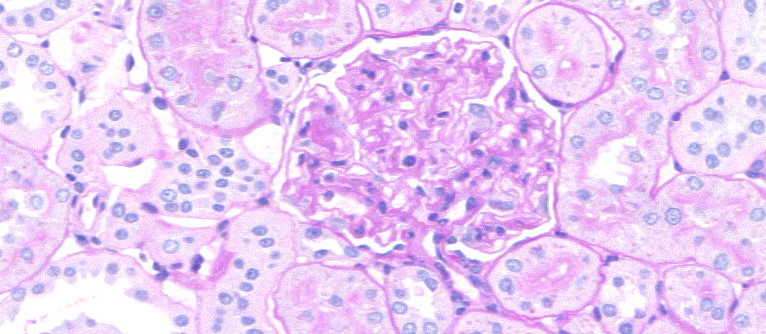

Supplement: Supplementary file 7 [file DataSheet13.ZIP › DKD/Fig 1D-PAS-DKD-21/21-5.jpeg]

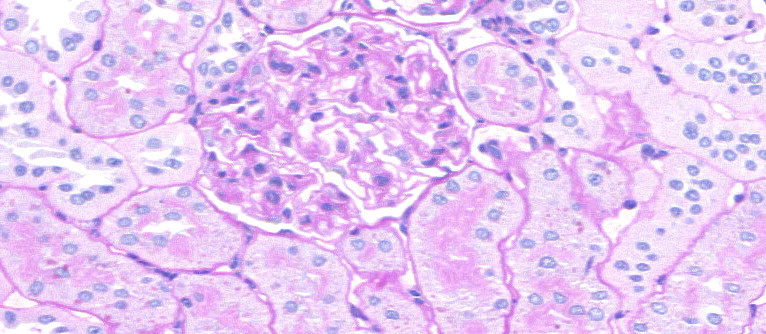

Supplement: Supplementary file 7 [file DataSheet13.ZIP › DKD/Fig 1D-PAS-DKD-21/21-6.jpeg]

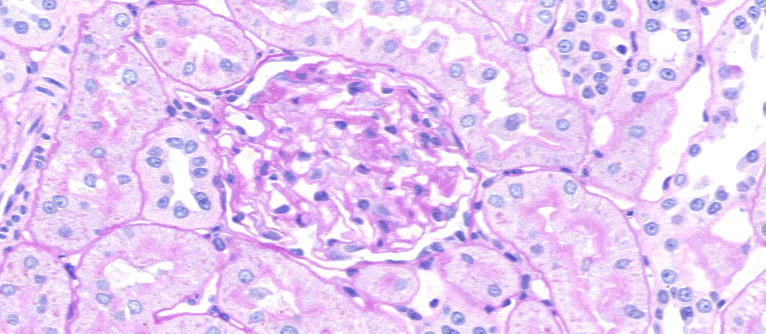

Supplement: Supplementary file 7 [file DataSheet13.ZIP › DKD/Fig 1D-PAS-DKD-21/21-7.jpeg]

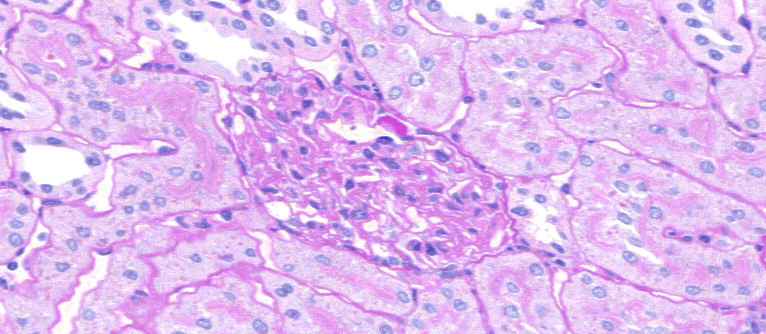

Supplement: Supplementary file 7 [file DataSheet13.ZIP › DKD/Fig 1D-PAS-DKD-21/21-8.jpeg]

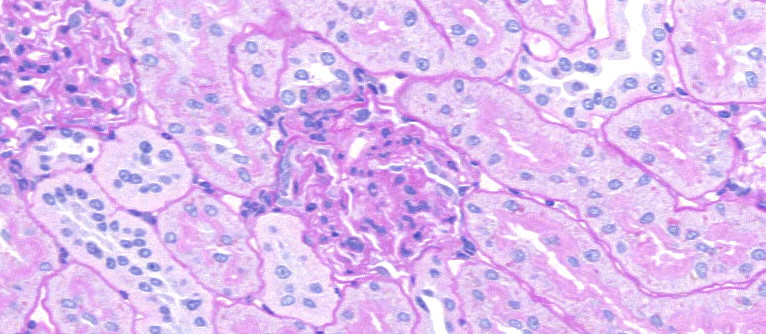

Supplement: Supplementary file 7 [file DataSheet13.ZIP › DKD/Fig 1D-PAS-DKD-21/21-9.jpeg]

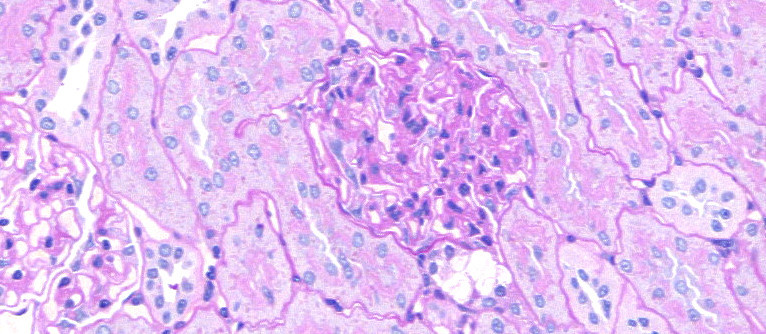

Supplement: Supplementary file 7 [file DataSheet13.ZIP › DKD/Fig 1D-PAS-DKD-22/22-1.jpeg]

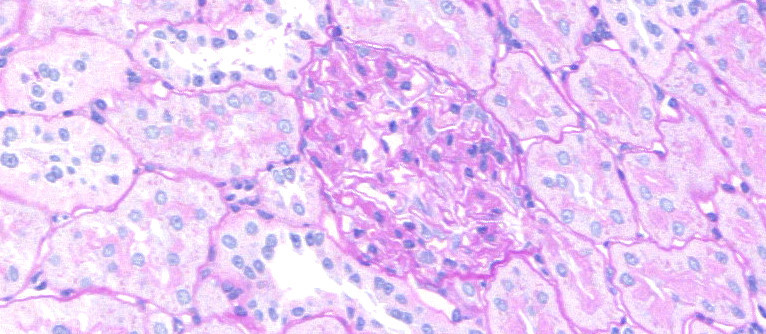

Supplement: Supplementary file 7 [file DataSheet13.ZIP › DKD/Fig 1D-PAS-DKD-22/22-10.jpeg]

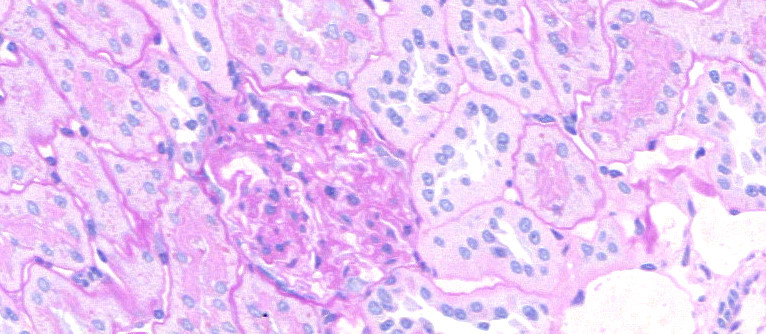

Supplement: Supplementary file 7 [file DataSheet13.ZIP › DKD/Fig 1D-PAS-DKD-22/22-11.jpeg]

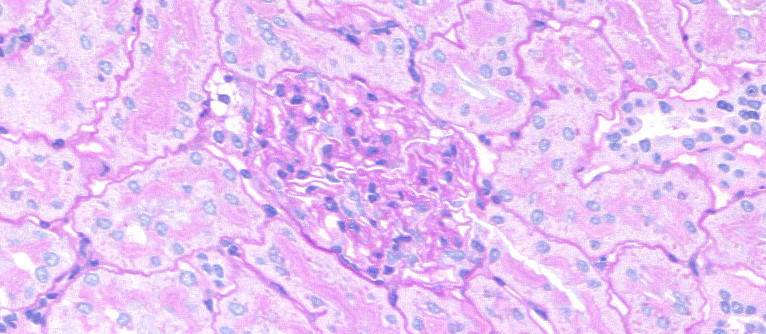

Supplement: Supplementary file 7 [file DataSheet13.ZIP › DKD/Fig 1D-PAS-DKD-22/22-12.jpeg]

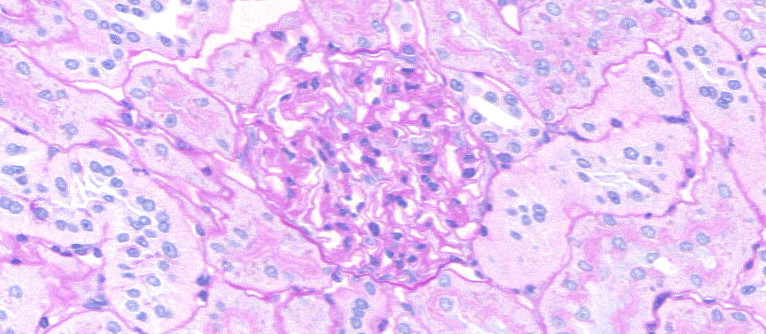

Supplement: Supplementary file 7 [file DataSheet13.ZIP › DKD/Fig 1D-PAS-DKD-22/22-13.jpeg]

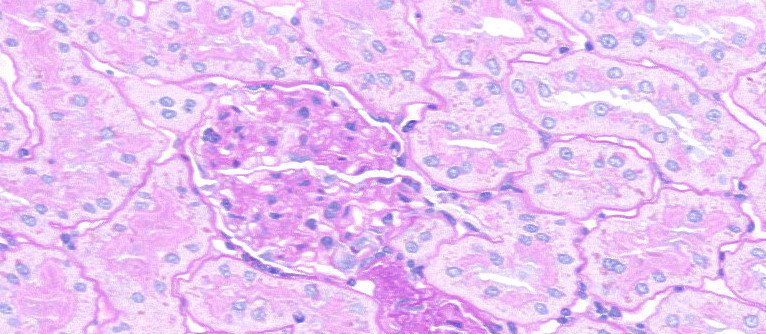

Supplement: Supplementary file 7 [file DataSheet13.ZIP › DKD/Fig 1D-PAS-DKD-22/22-14.jpeg]

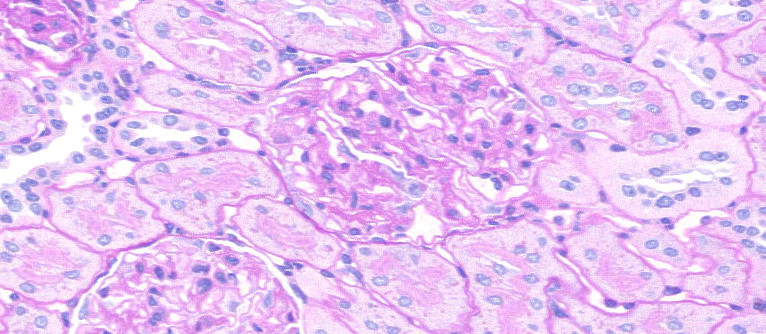

Supplement: Supplementary file 7 [file DataSheet13.ZIP › DKD/Fig 1D-PAS-DKD-22/22-15.jpeg]

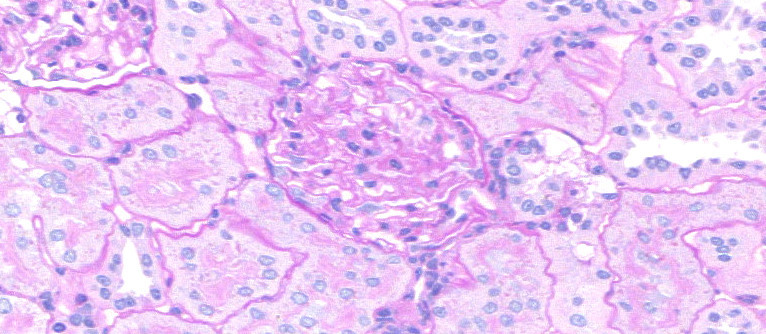

Supplement: Supplementary file 7 [file DataSheet13.ZIP › DKD/Fig 1D-PAS-DKD-22/22-16.jpeg]

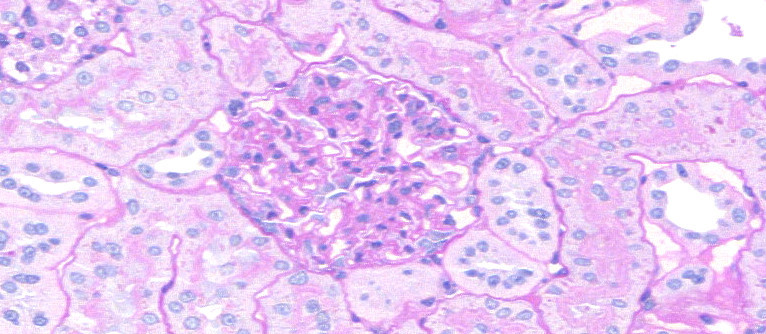

Supplement: Supplementary file 7 [file DataSheet13.ZIP › DKD/Fig 1D-PAS-DKD-22/22-17.jpeg]

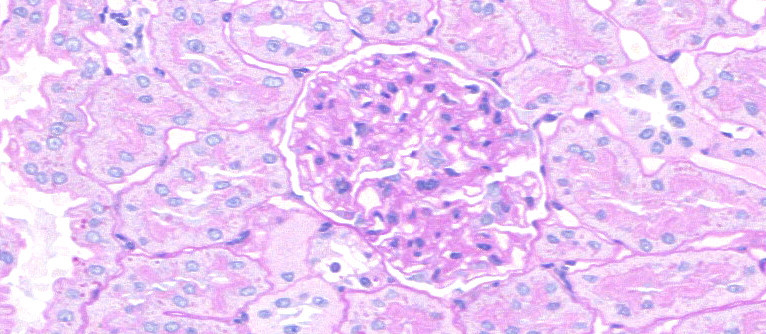

Supplement: Supplementary file 7 [file DataSheet13.ZIP › DKD/Fig 1D-PAS-DKD-22/22-18.jpeg]

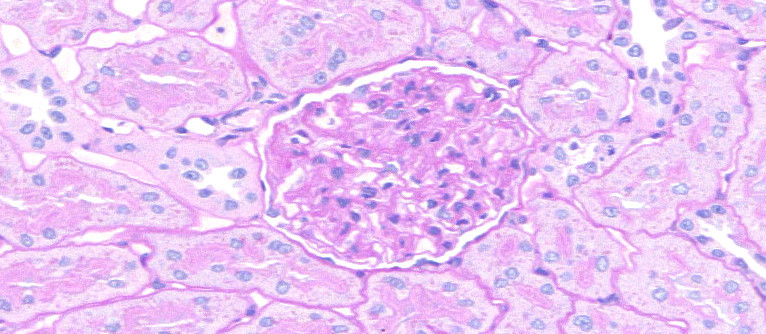

Supplement: Supplementary file 7 [file DataSheet13.ZIP › DKD/Fig 1D-PAS-DKD-22/22-19.jpeg]

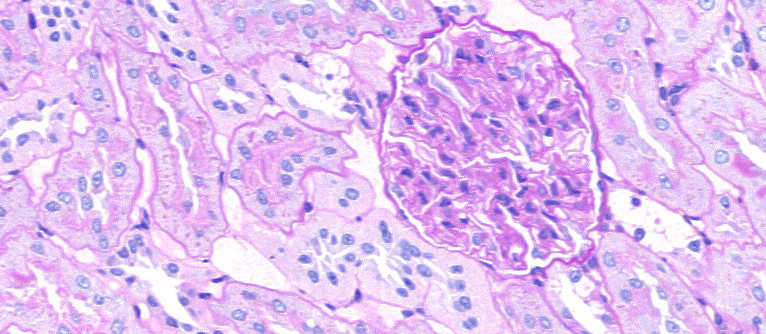

Supplement: Supplementary file 7 [file DataSheet13.ZIP › DKD/Fig 1D-PAS-DKD-22/22-2.jpeg]

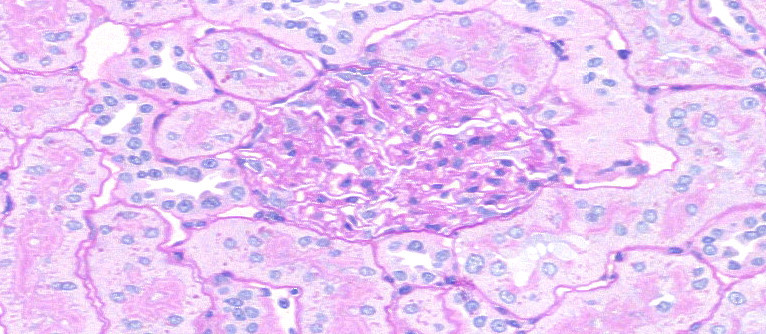

Supplement: Supplementary file 7 [file DataSheet13.ZIP › DKD/Fig 1D-PAS-DKD-22/22-20.jpeg]

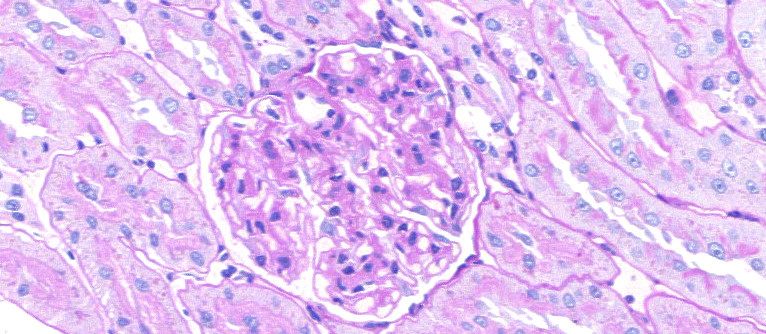

Supplement: Supplementary file 7 [file DataSheet13.ZIP › DKD/Fig 1D-PAS-DKD-22/22-3.jpeg]

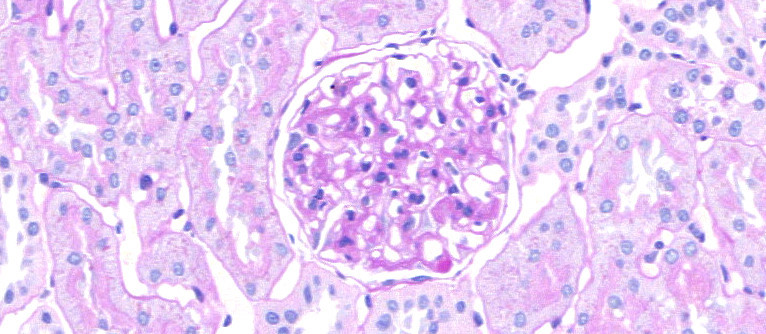

Supplement: Supplementary file 7 [file DataSheet13.ZIP › DKD/Fig 1D-PAS-DKD-22/22-4.jpeg]

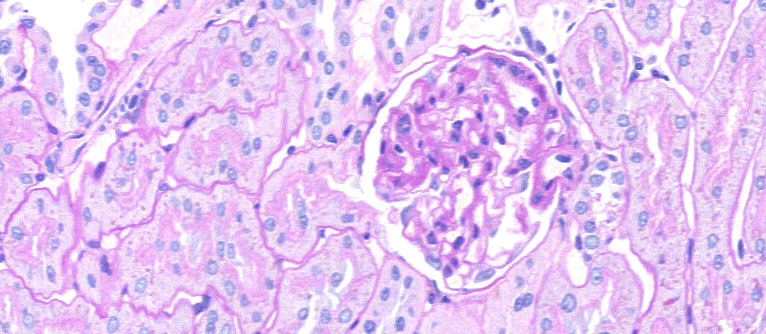

Supplement: Supplementary file 7 [file DataSheet13.ZIP › DKD/Fig 1D-PAS-DKD-22/22-5.jpeg]

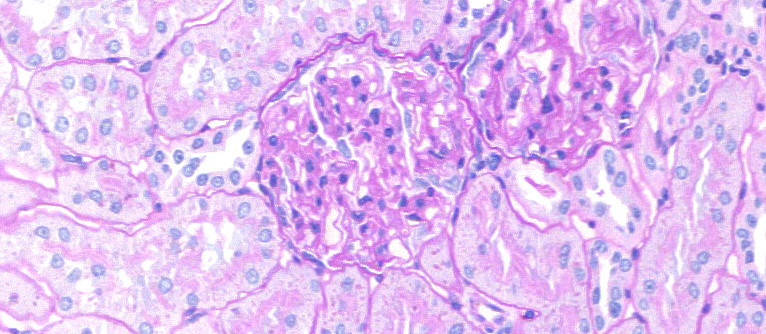

Supplement: Supplementary file 7 [file DataSheet13.ZIP › DKD/Fig 1D-PAS-DKD-22/22-6.jpeg]

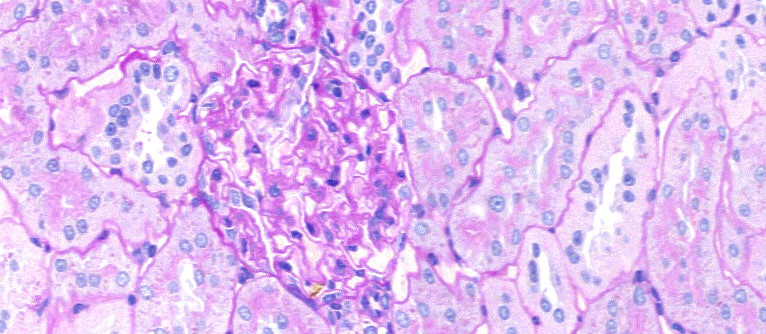

Supplement: Supplementary file 7 [file DataSheet13.ZIP › DKD/Fig 1D-PAS-DKD-22/22-7.jpeg]

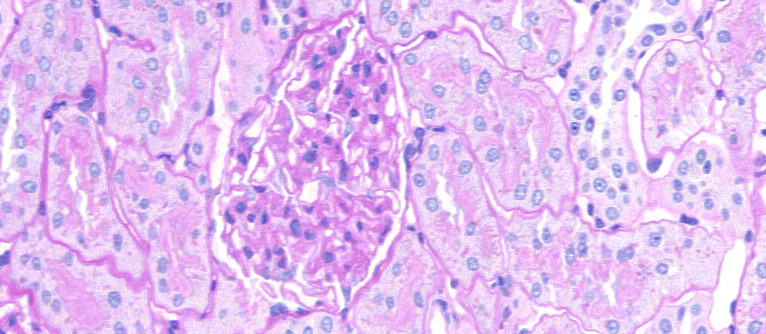

Supplement: Supplementary file 7 [file DataSheet13.ZIP › DKD/Fig 1D-PAS-DKD-22/22-8.jpeg]

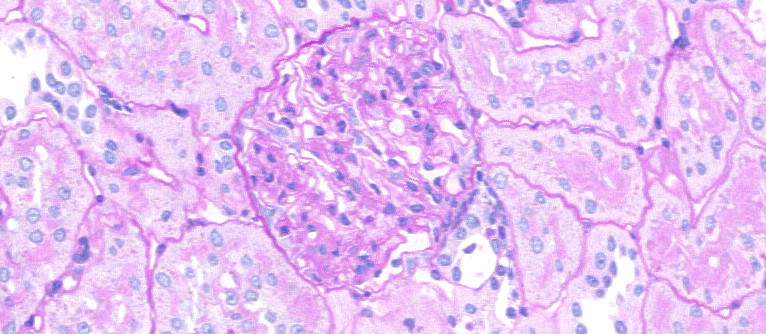

Supplement: Supplementary file 7 [file DataSheet13.ZIP › DKD/Fig 1D-PAS-DKD-22/22-9.jpeg]

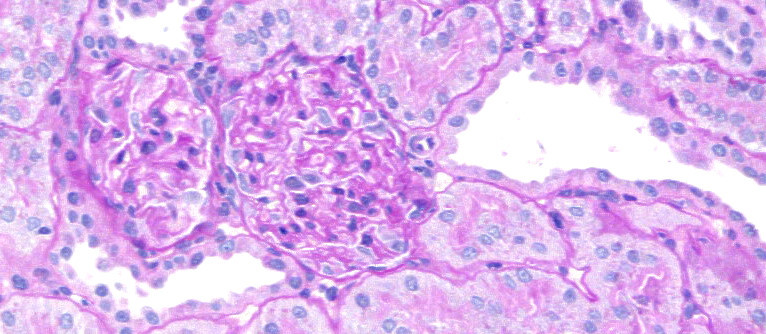

Supplement: Supplementary file 7 [file DataSheet13.ZIP › DKD/Fig 1D-PAS-DKD-23/23-1.jpeg]
