# Supplementary figures and images for: Characteristics of Serum Metabolites and Gut Microbiota in Diabetic Kidney Disease (part 8 of 13)
Source: Front Pharmacol. 2022 Apr 14;13:872988. doi: 10.3389/fphar.2022.872988 (PMC9084235; doi:10.3389/fphar.2022.872988)

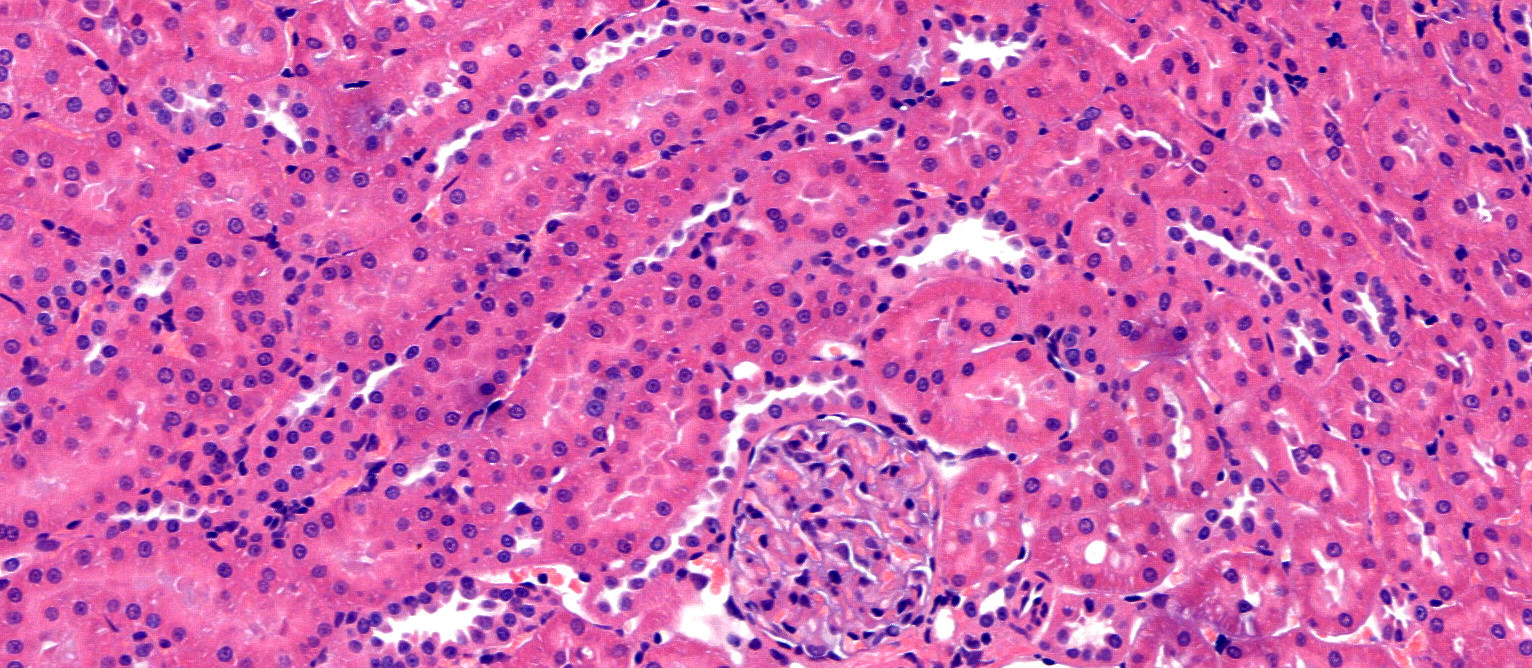

Supplement: Supplementary file 8 [file DataSheet1.ZIP › Fig 1D-HE-sham-5/5-10.jpeg]

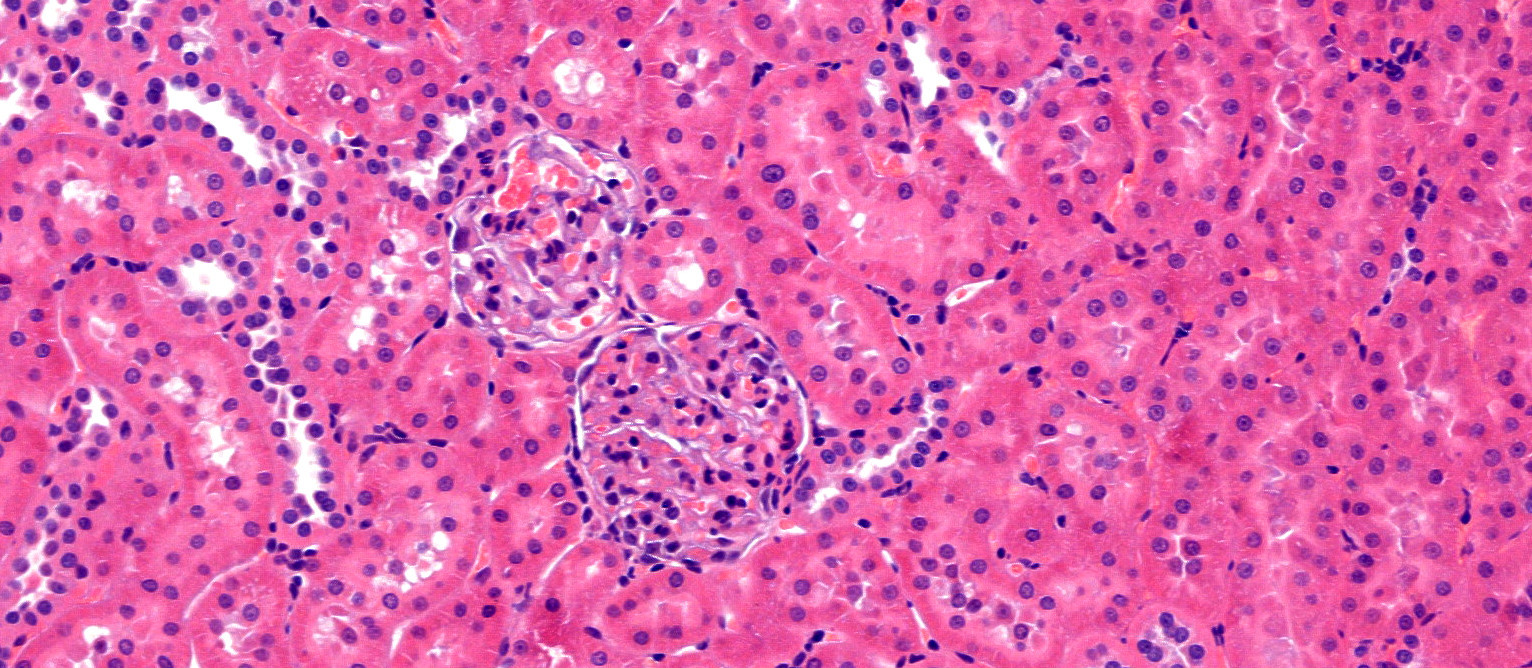

Supplement: Supplementary file 8 [file DataSheet1.ZIP › Fig 1D-HE-sham-5/5-2.jpeg]

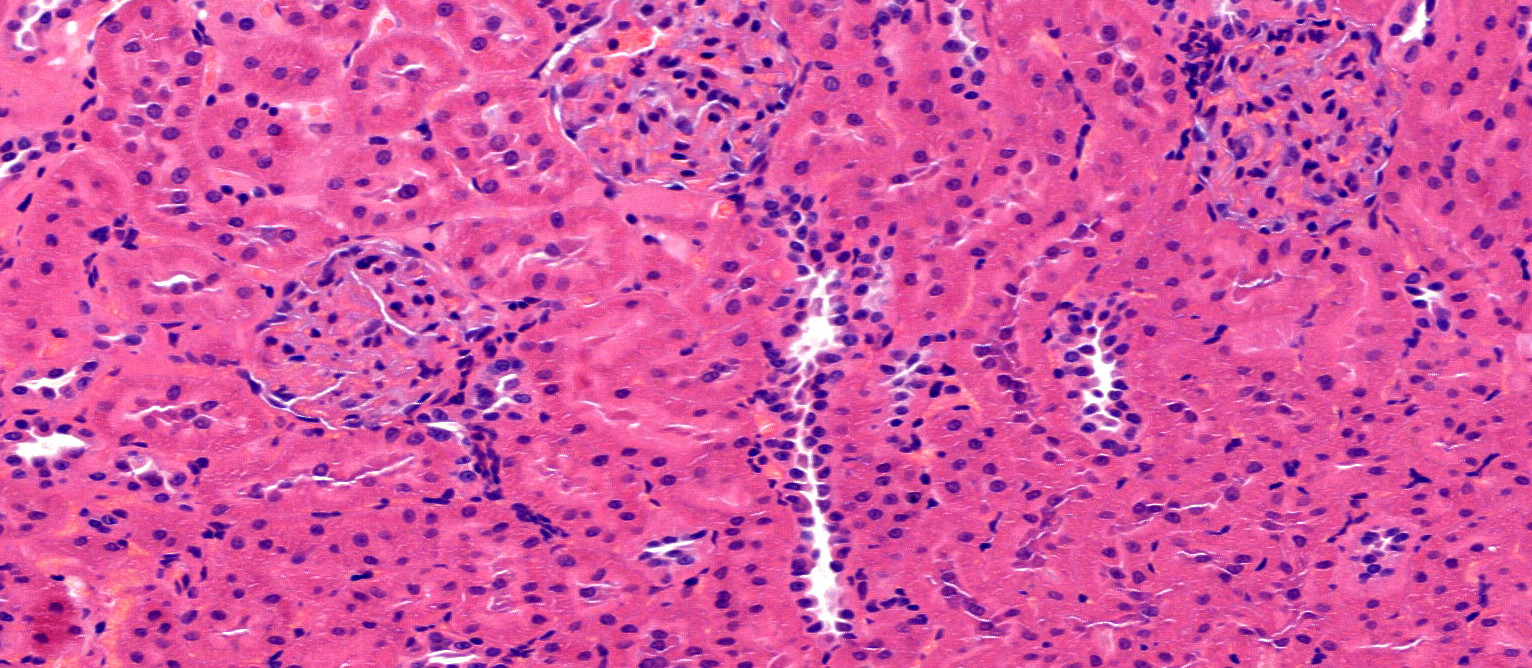

Supplement: Supplementary file 8 [file DataSheet1.ZIP › Fig 1D-HE-sham-5/5-3.jpeg]

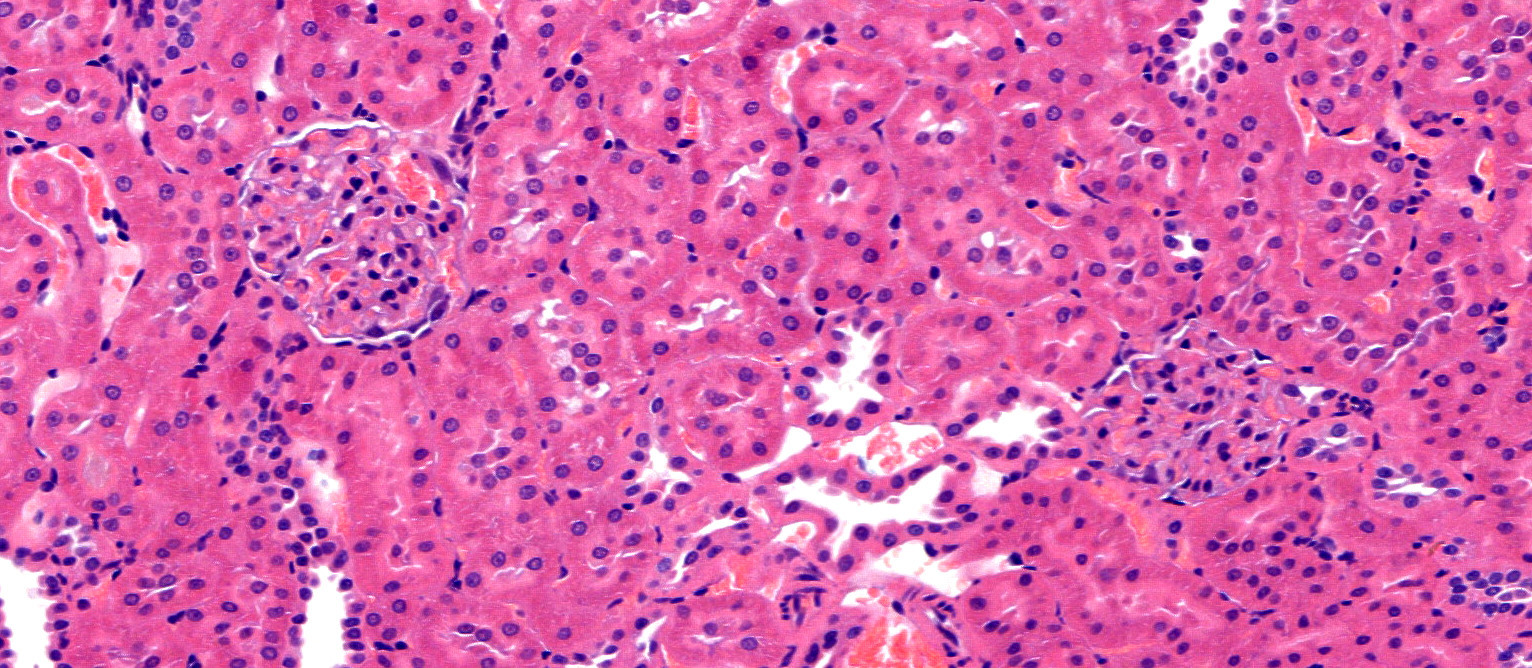

Supplement: Supplementary file 8 [file DataSheet1.ZIP › Fig 1D-HE-sham-5/5-4.jpeg]

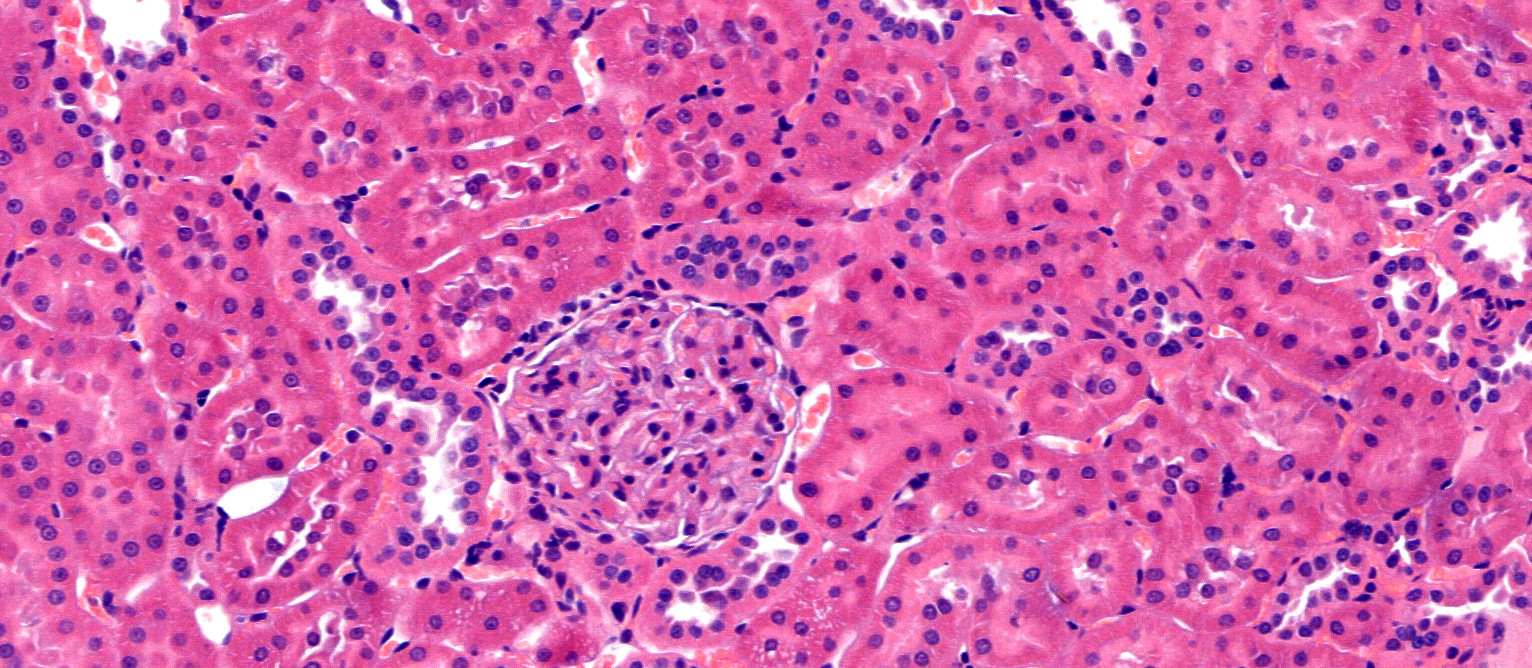

Supplement: Supplementary file 8 [file DataSheet1.ZIP › Fig 1D-HE-sham-5/5-5.jpeg]

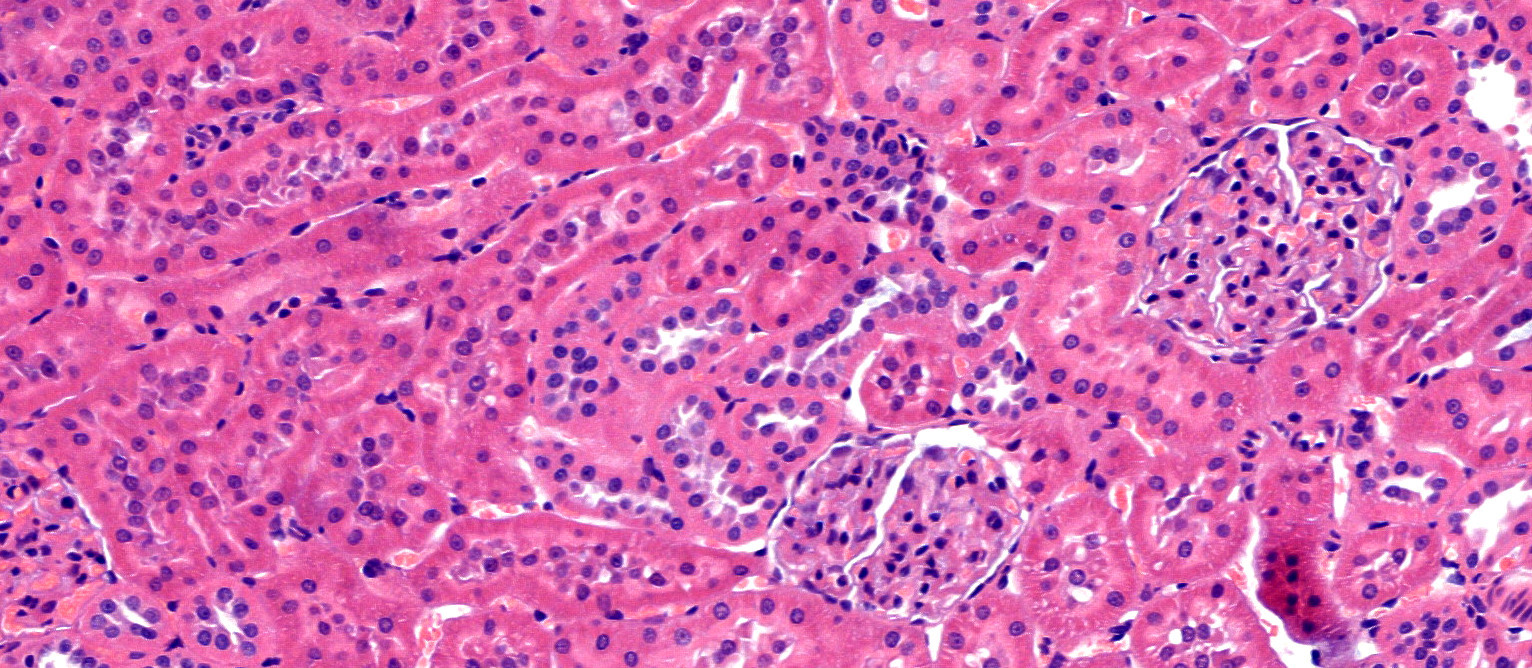

Supplement: Supplementary file 8 [file DataSheet1.ZIP › Fig 1D-HE-sham-5/5-6.jpeg]

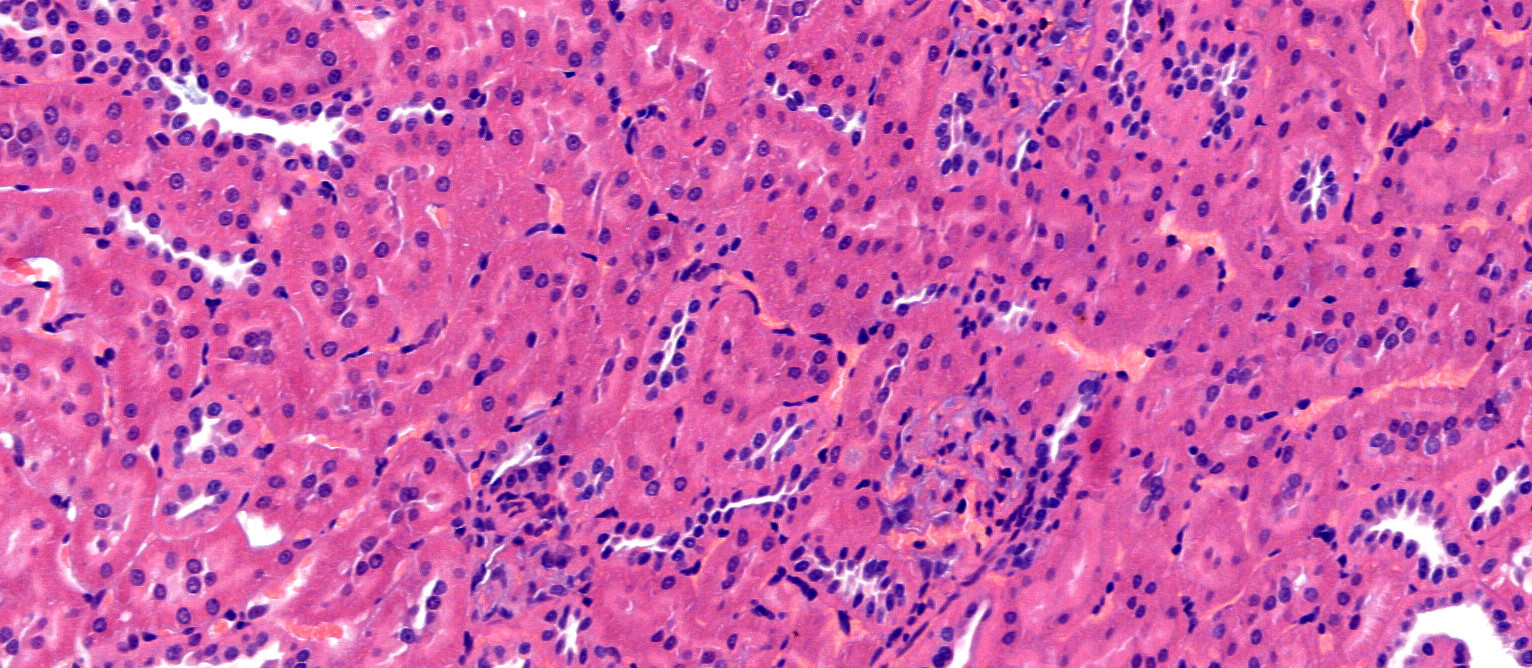

Supplement: Supplementary file 8 [file DataSheet1.ZIP › Fig 1D-HE-sham-5/5-7.jpeg]

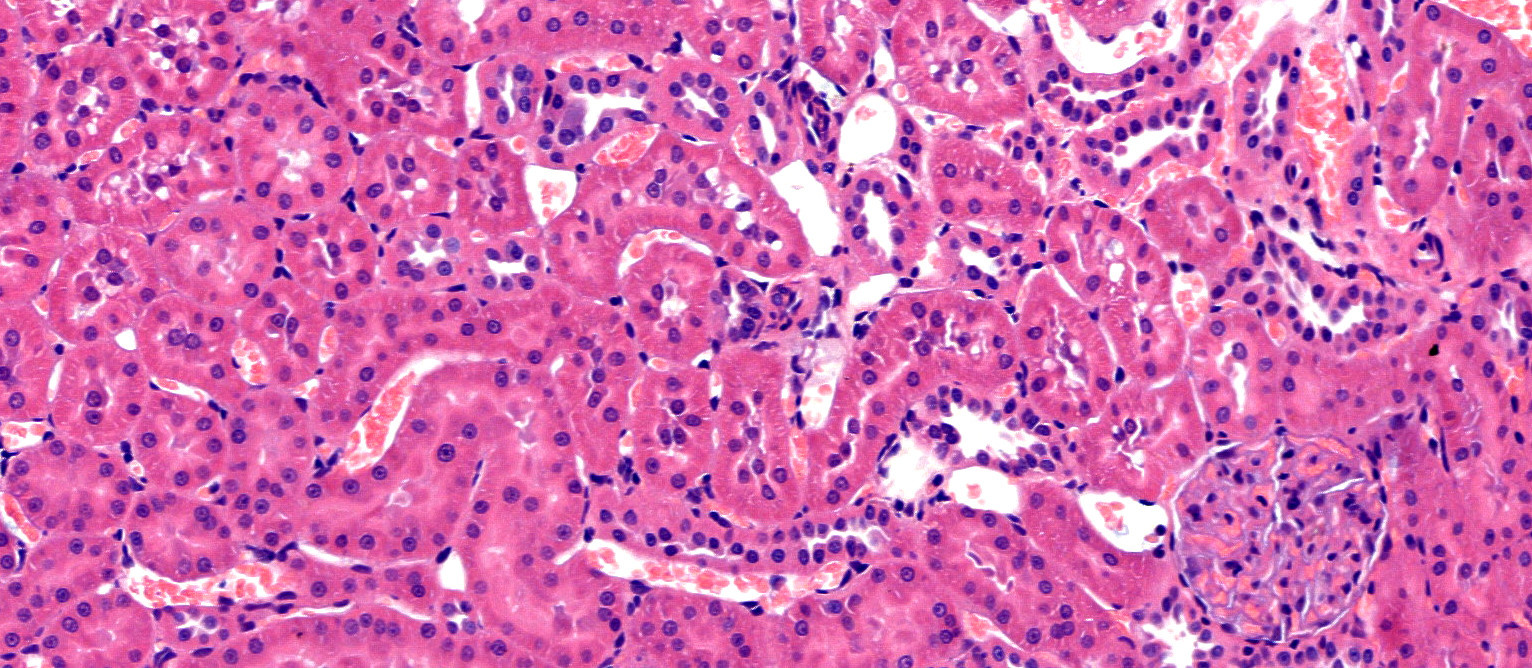

Supplement: Supplementary file 8 [file DataSheet1.ZIP › Fig 1D-HE-sham-5/5-8.jpeg]

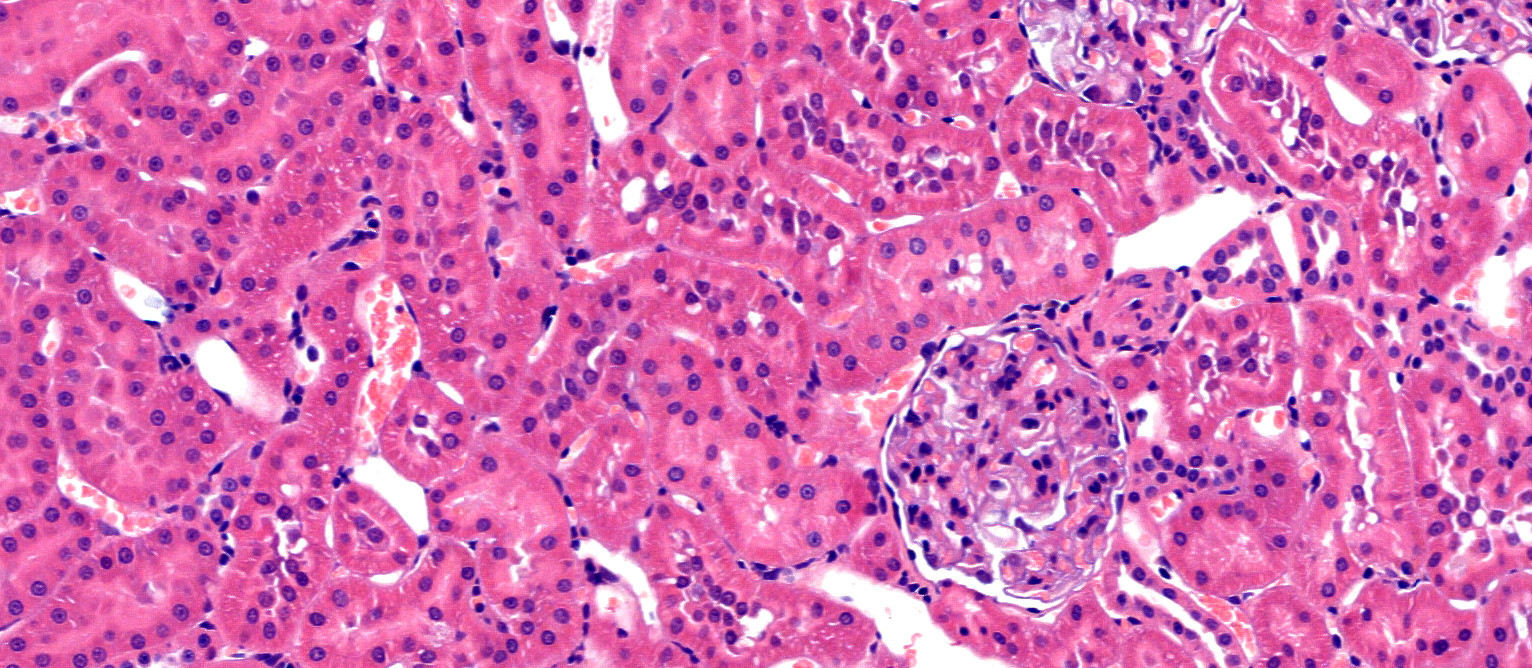

Supplement: Supplementary file 8 [file DataSheet1.ZIP › Fig 1D-HE-sham-5/5-9.jpeg]

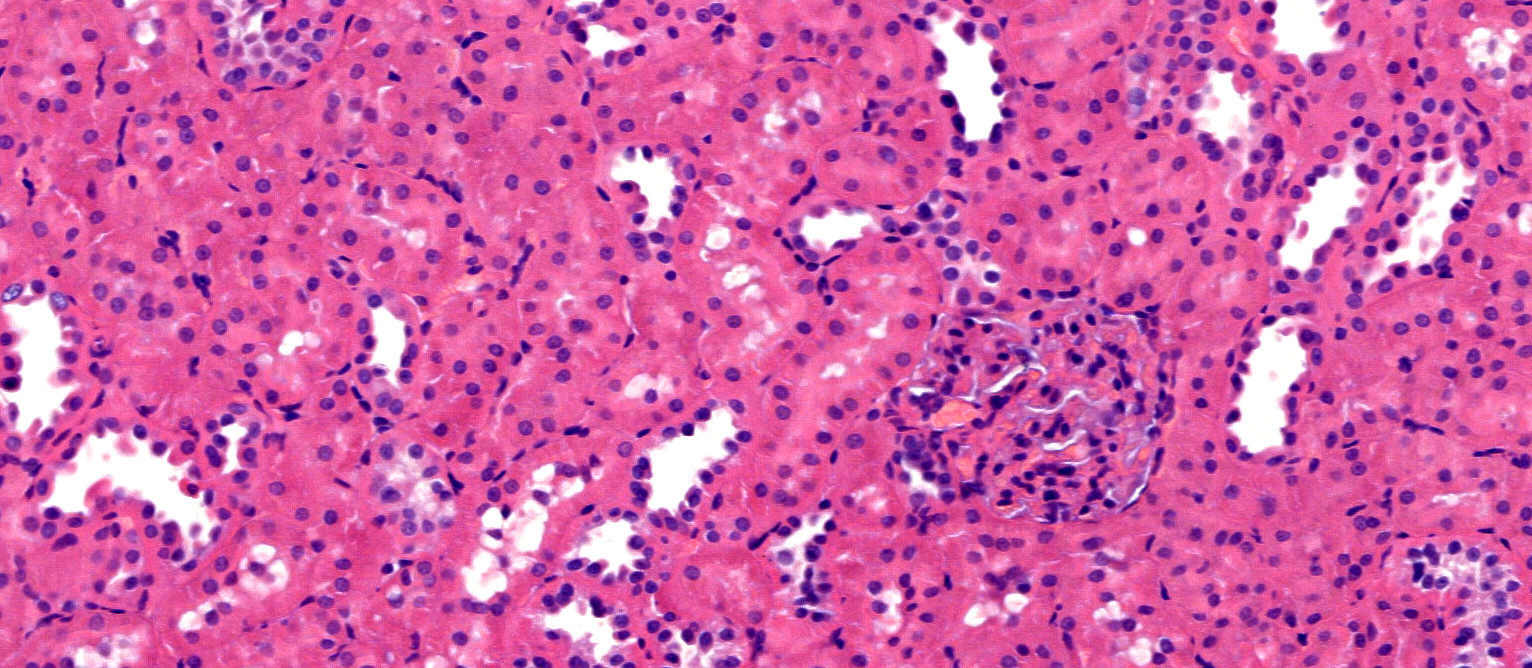

Supplement: Supplementary file 8 [file DataSheet1.ZIP › Fig 1D-HE-sham-6(1)/6-1.jpeg]

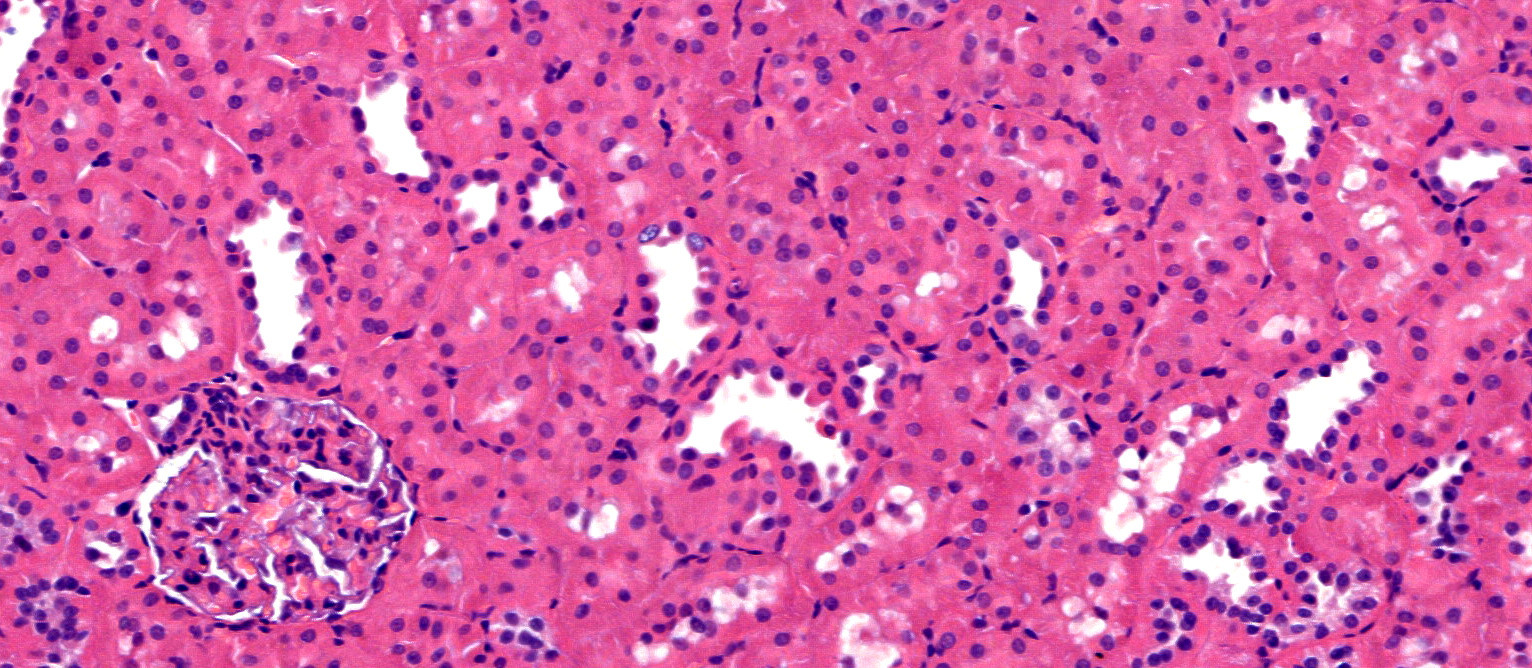

Supplement: Supplementary file 8 [file DataSheet1.ZIP › Fig 1D-HE-sham-6(1)/6-2.jpeg]

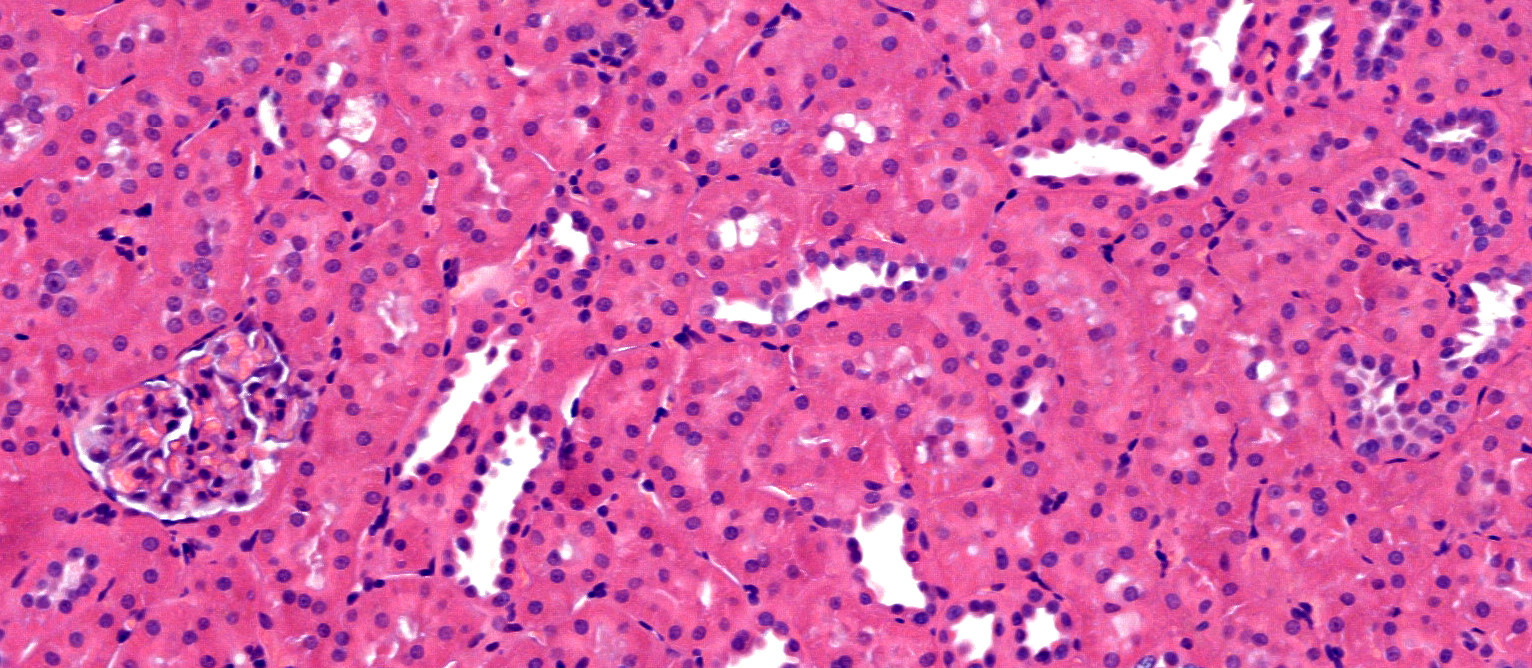

Supplement: Supplementary file 8 [file DataSheet1.ZIP › Fig 1D-HE-sham-6(1)/6-3.jpeg]

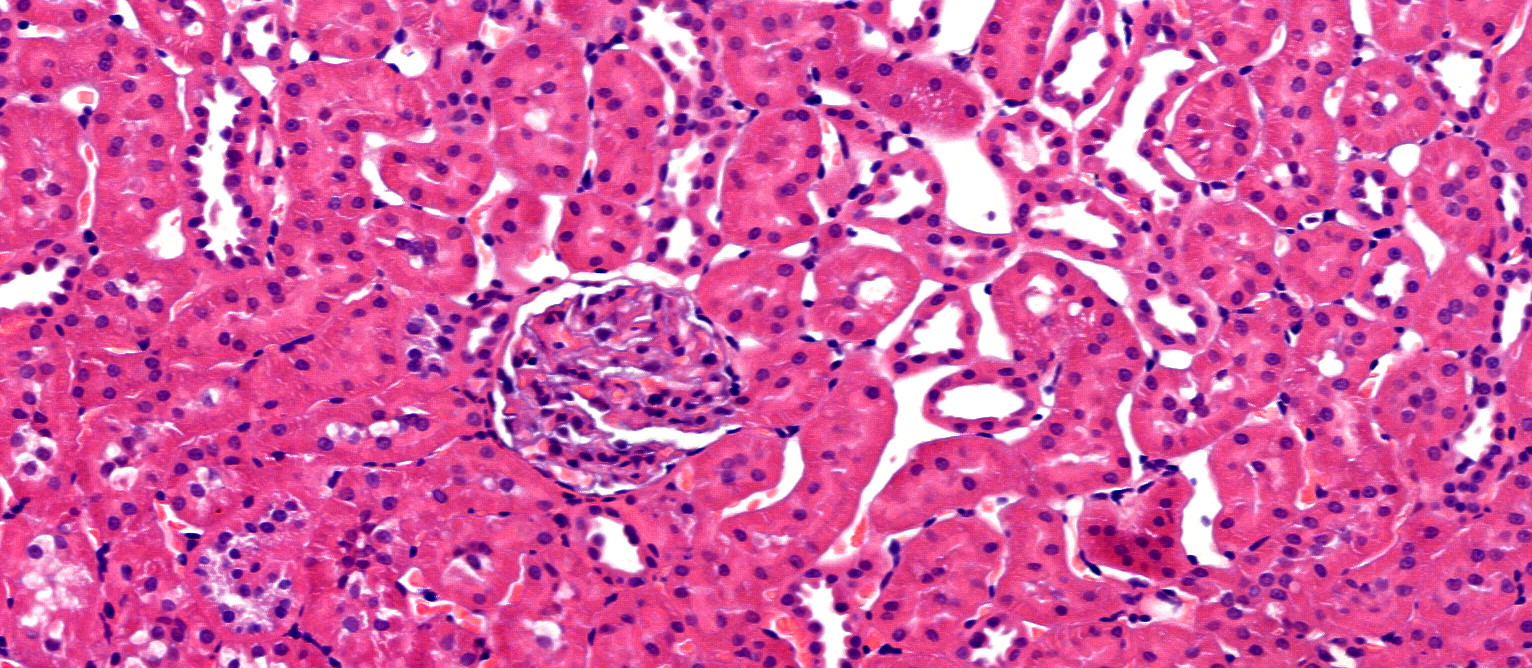

Supplement: Supplementary file 8 [file DataSheet1.ZIP › Fig 1D-HE-sham-6(1)/6-4.jpeg]

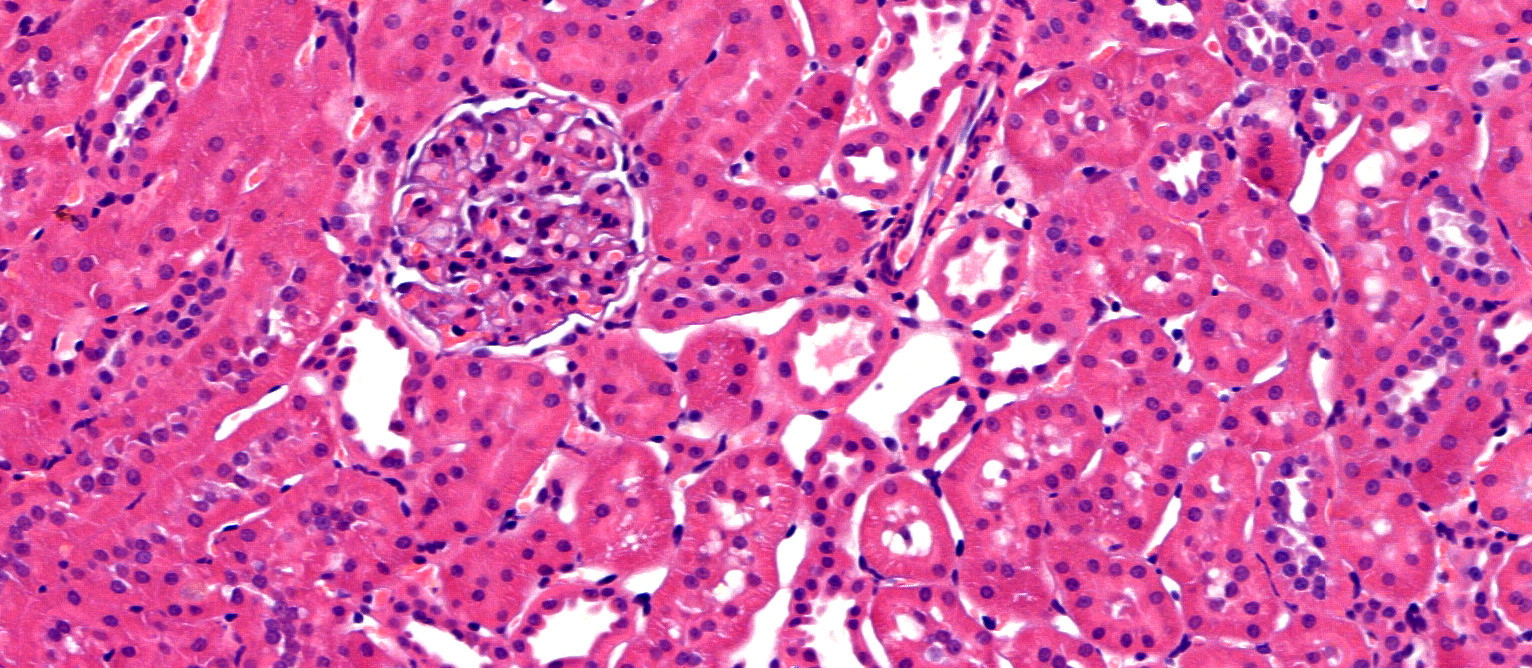

Supplement: Supplementary file 8 [file DataSheet1.ZIP › Fig 1D-HE-sham-6(1)/6-5.jpeg]

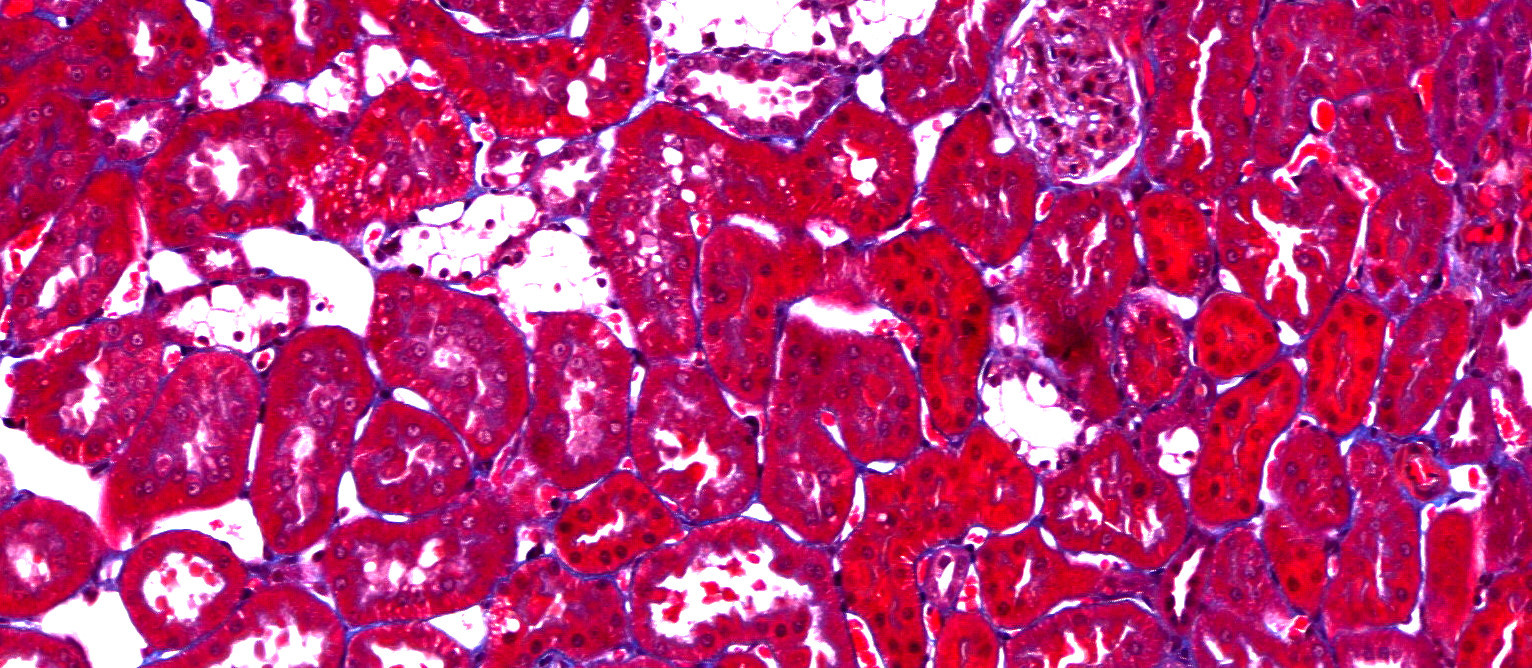

Supplement: Supplementary file 9 [file DataSheet10.ZIP › Fig 1D-masson-DKD-14/14-1.jpeg]

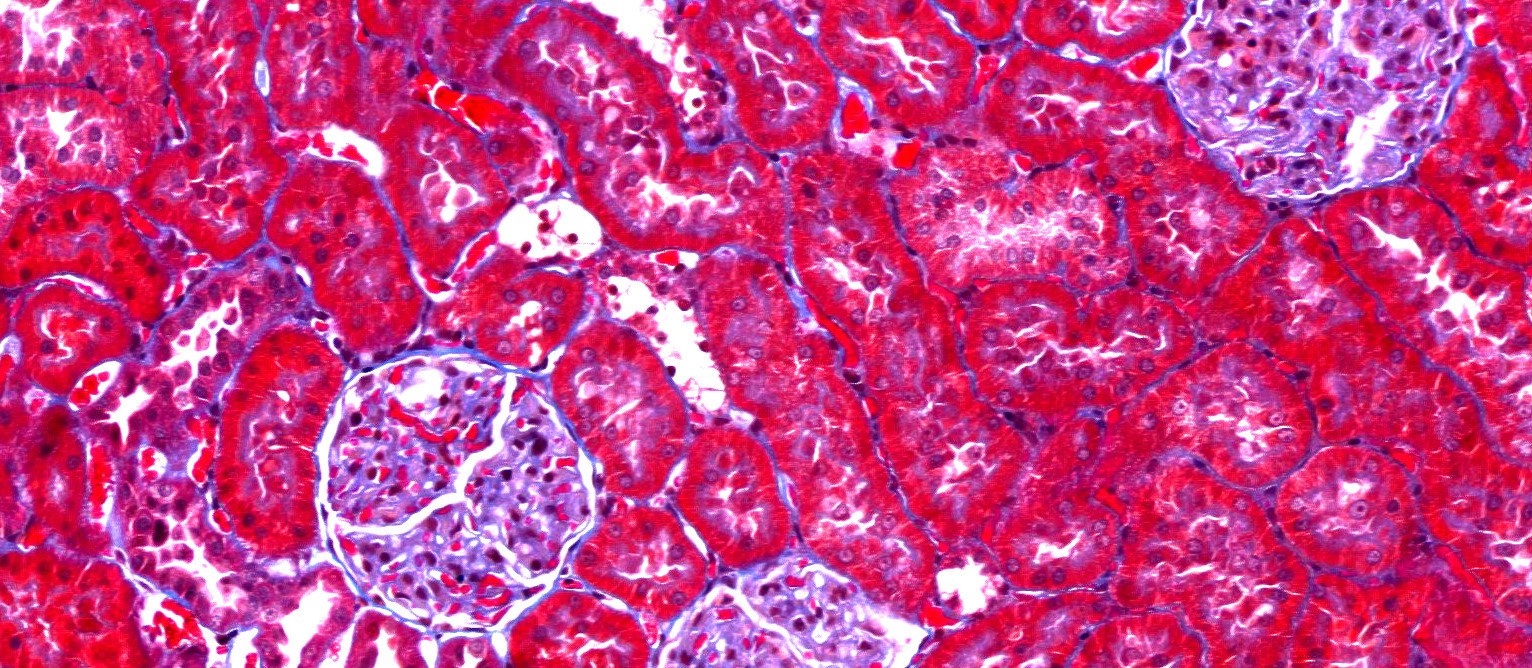

Supplement: Supplementary file 9 [file DataSheet10.ZIP › Fig 1D-masson-DKD-14/14-10.jpeg]

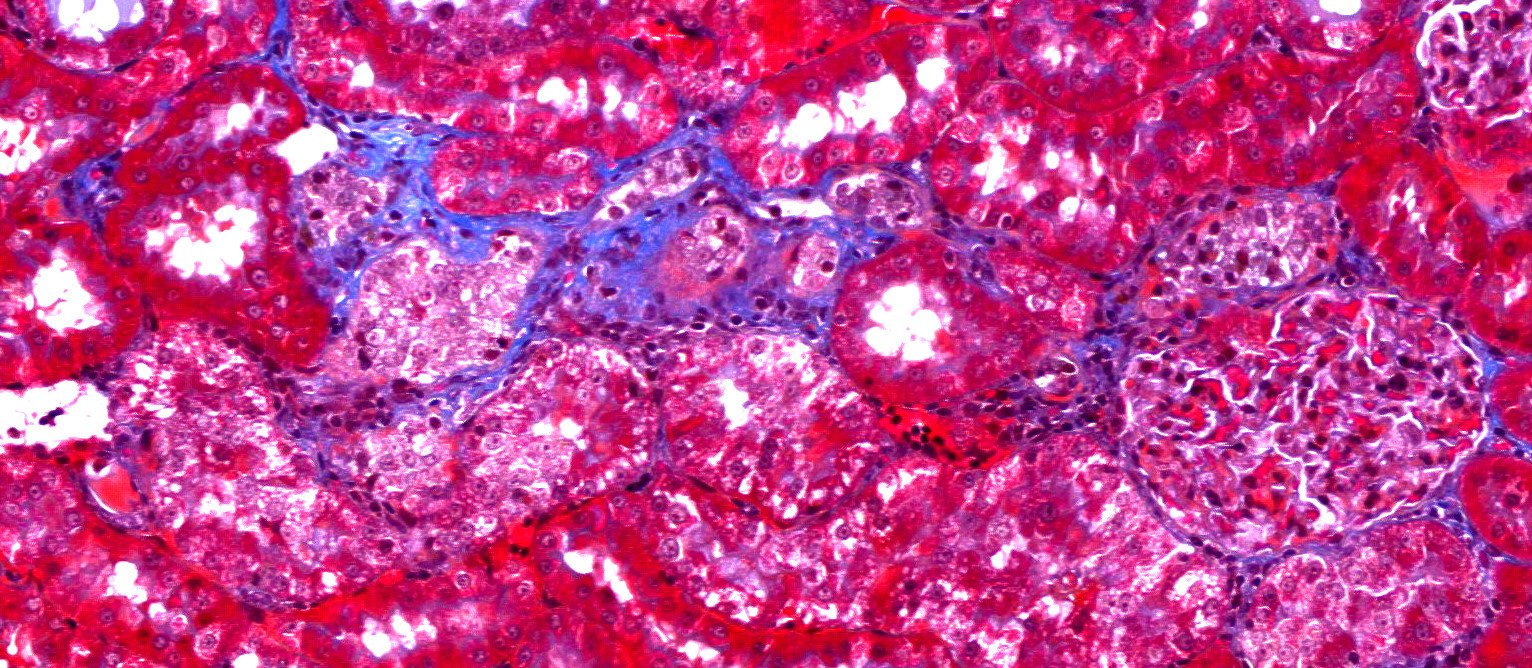

Supplement: Supplementary file 9 [file DataSheet10.ZIP › Fig 1D-masson-DKD-14/14-2.jpeg]

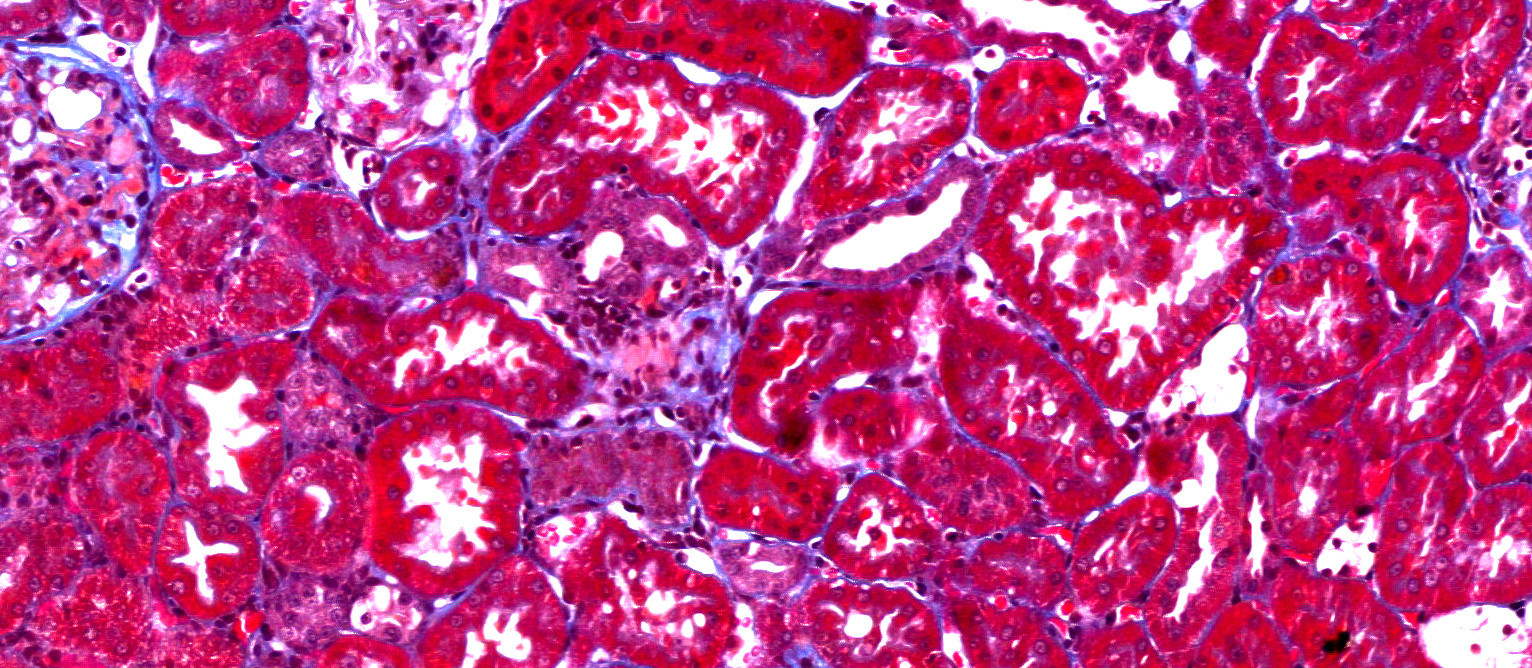

Supplement: Supplementary file 9 [file DataSheet10.ZIP › Fig 1D-masson-DKD-14/14-3.jpeg]

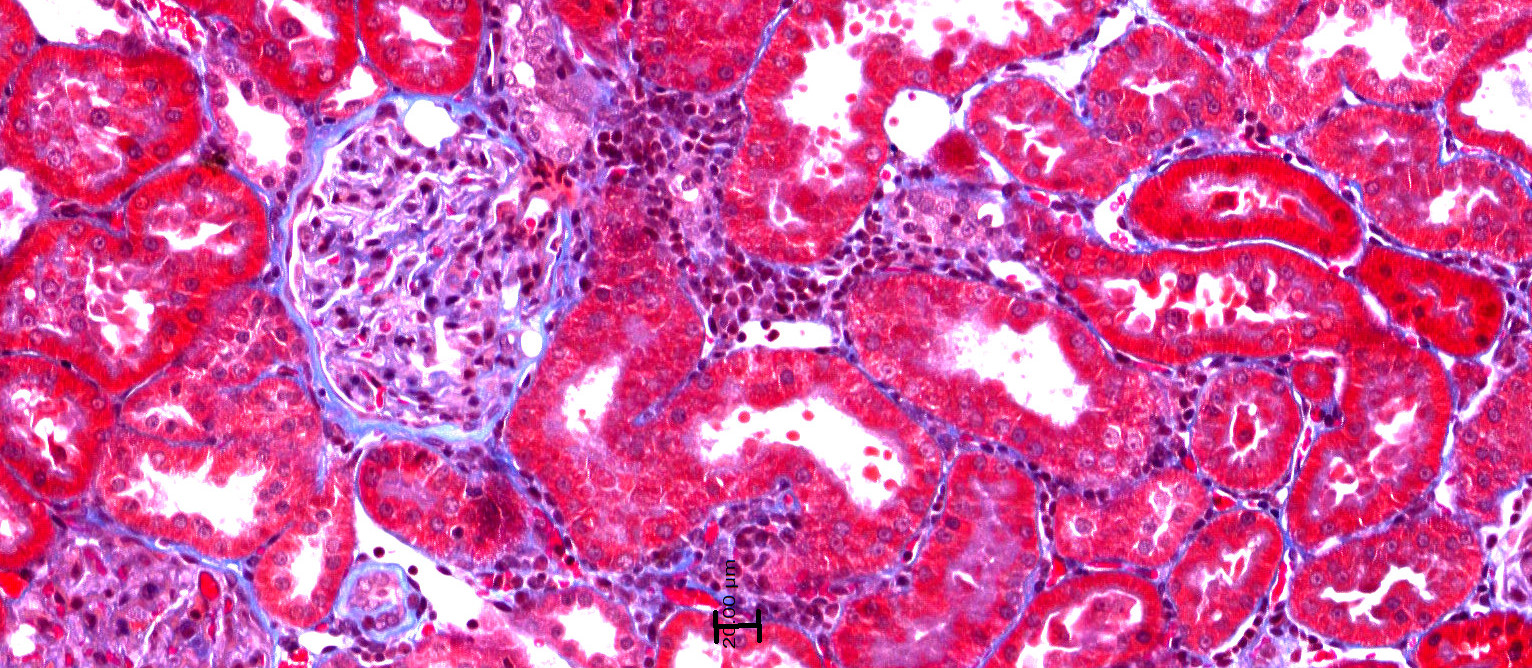

Supplement: Supplementary file 9 [file DataSheet10.ZIP › Fig 1D-masson-DKD-14/14-4.jpeg]

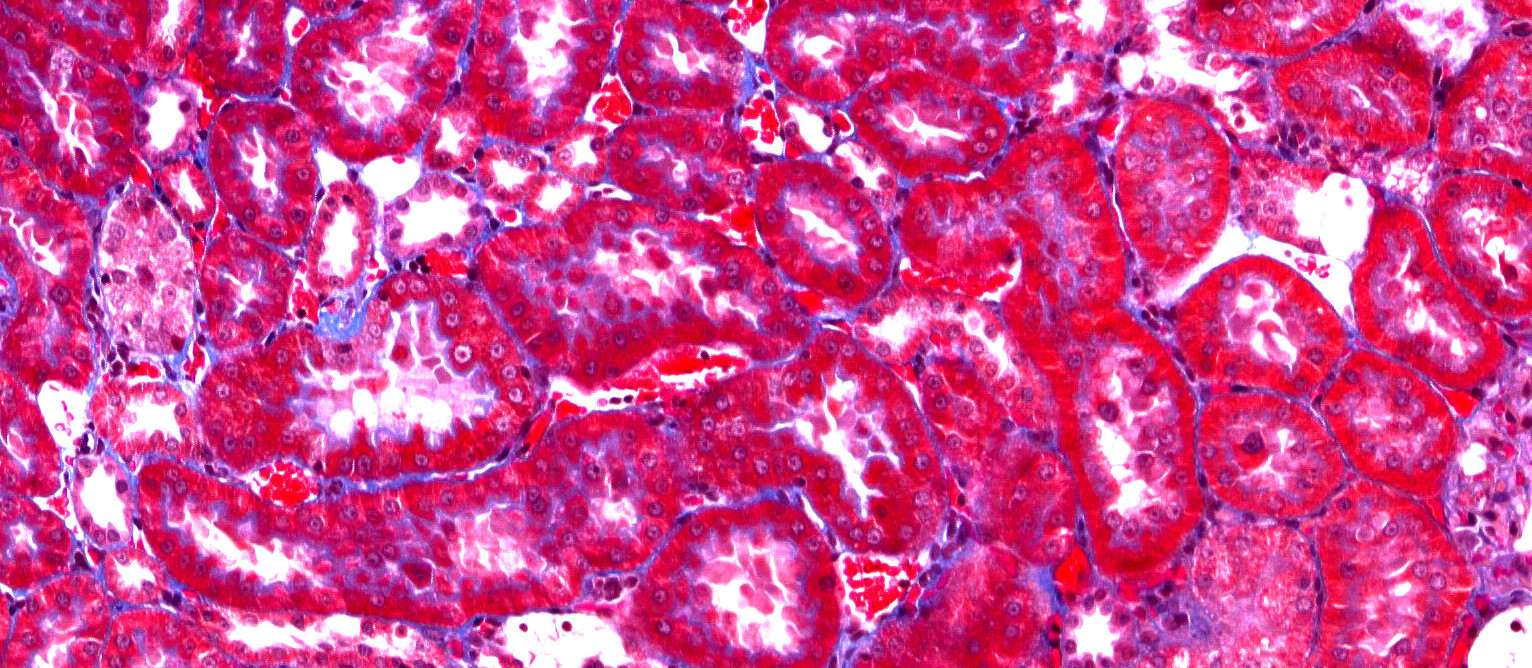

Supplement: Supplementary file 9 [file DataSheet10.ZIP › Fig 1D-masson-DKD-14/14-5.jpeg]

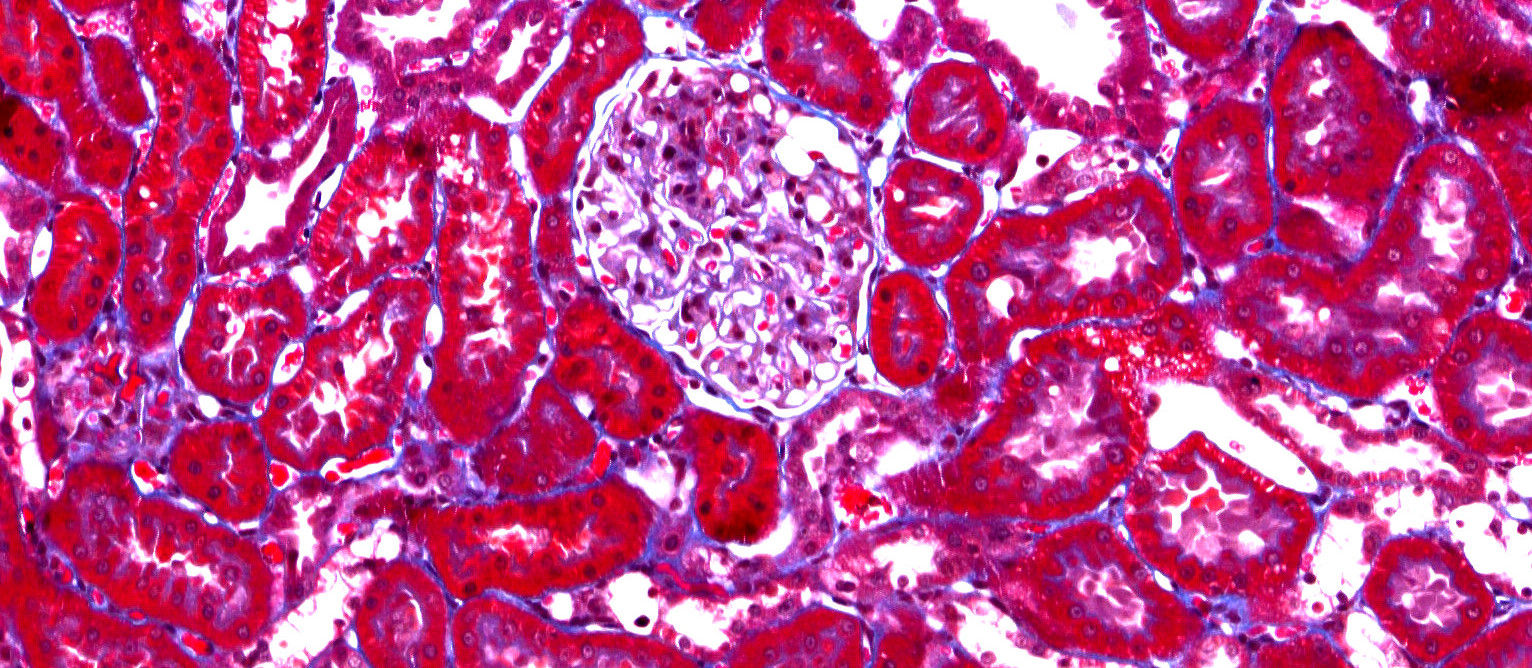

Supplement: Supplementary file 9 [file DataSheet10.ZIP › Fig 1D-masson-DKD-14/14-6.jpeg]

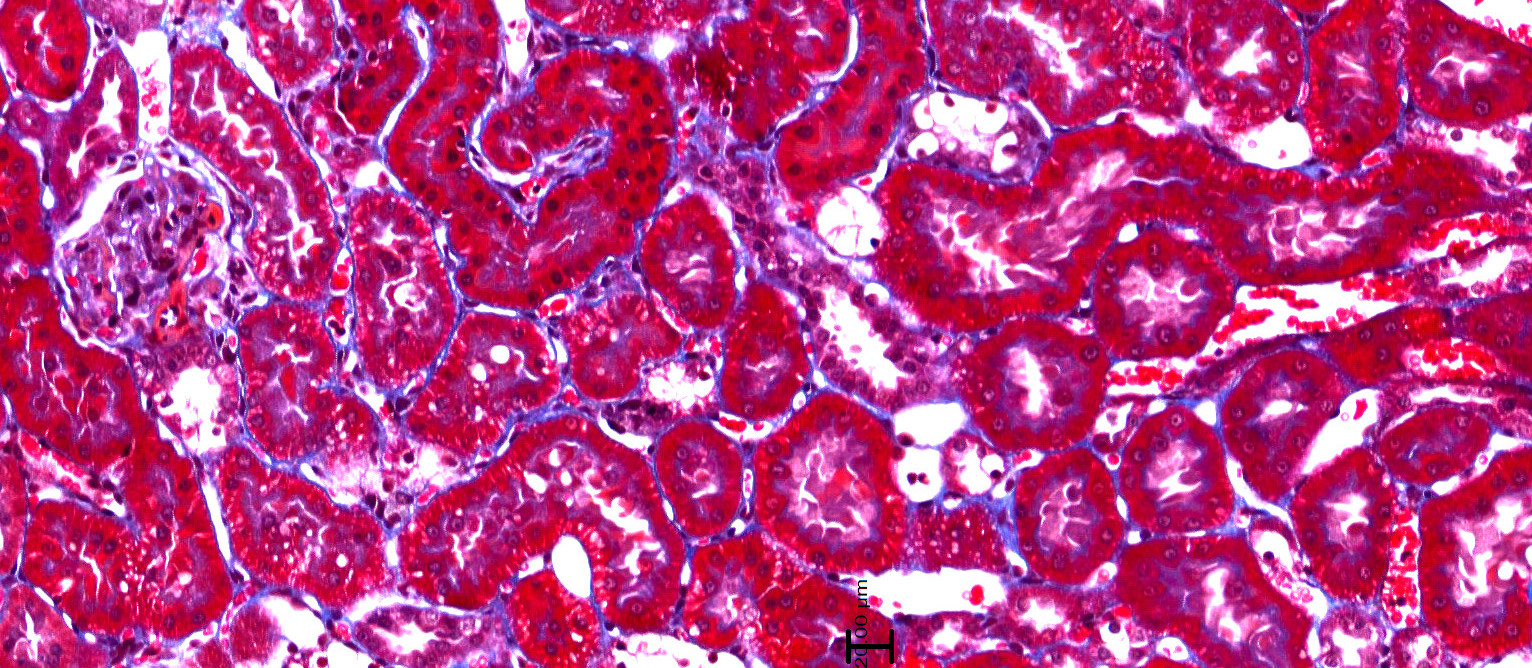

Supplement: Supplementary file 9 [file DataSheet10.ZIP › Fig 1D-masson-DKD-14/14-7.jpeg]

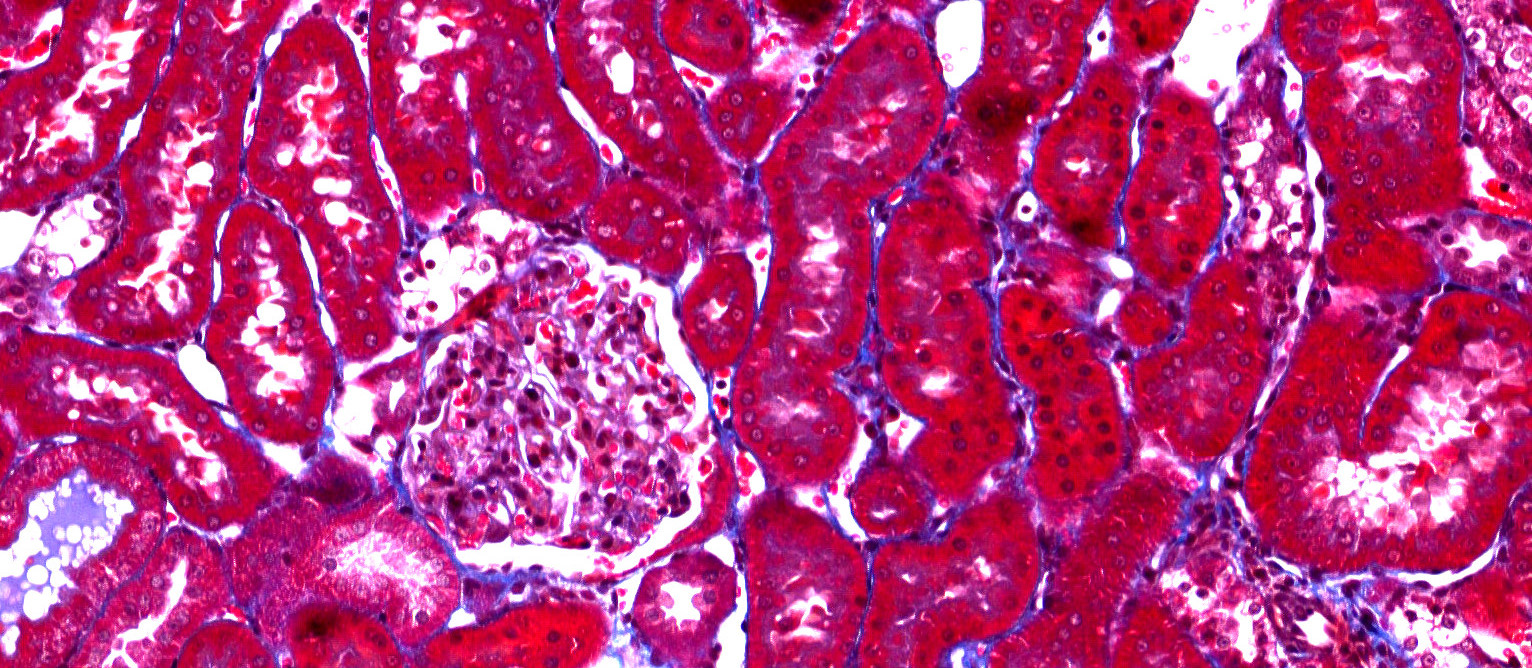

Supplement: Supplementary file 9 [file DataSheet10.ZIP › Fig 1D-masson-DKD-14/14-8.jpeg]

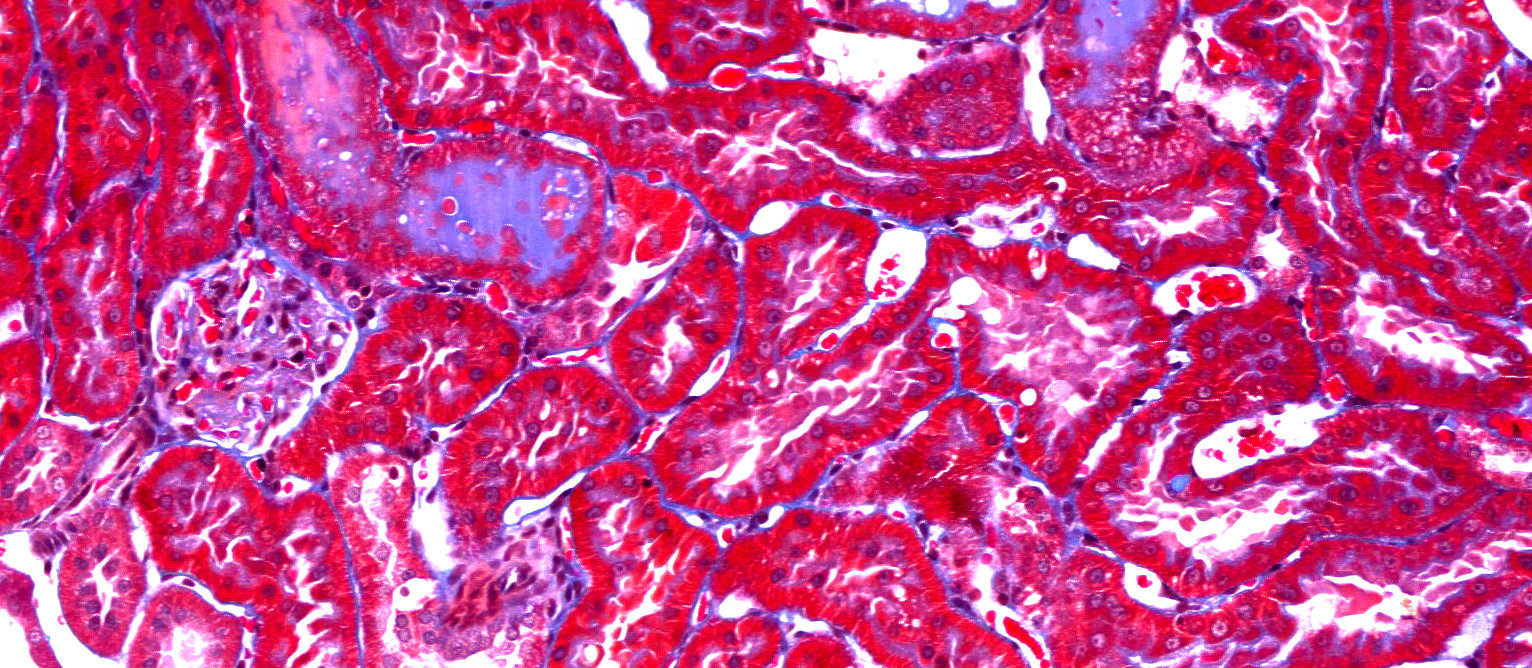

Supplement: Supplementary file 9 [file DataSheet10.ZIP › Fig 1D-masson-DKD-14/14-9.jpeg]

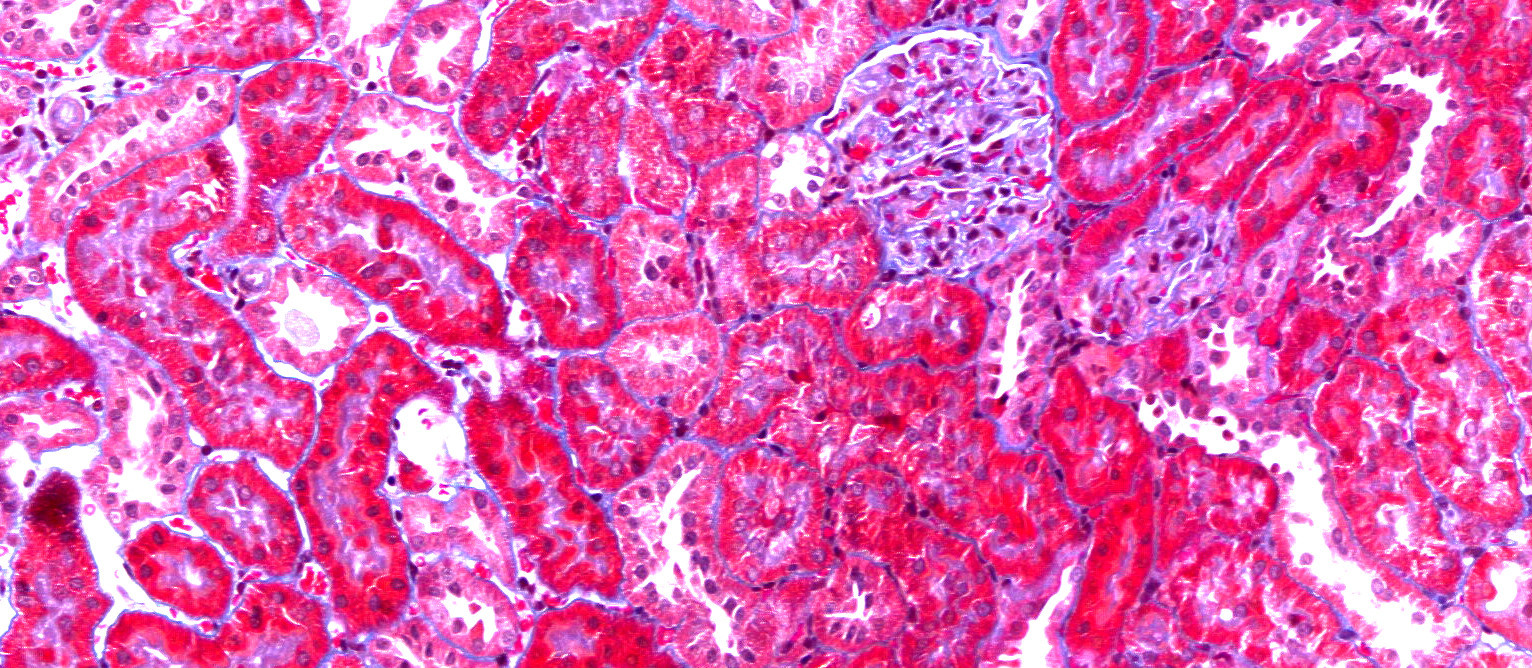

Supplement: Supplementary file 9 [file DataSheet10.ZIP › Fig 1D-masson-DKD-15/15-1.jpeg]

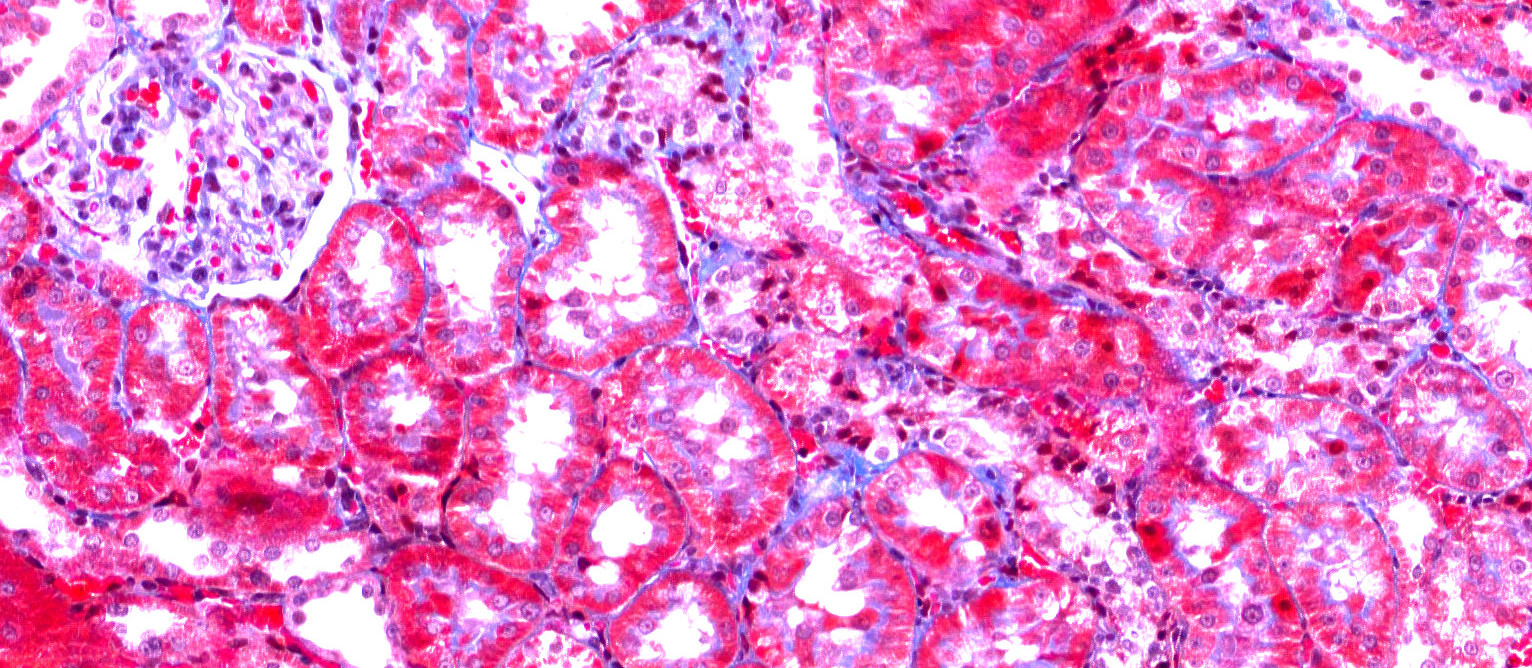

Supplement: Supplementary file 9 [file DataSheet10.ZIP › Fig 1D-masson-DKD-15/15-10.jpeg]

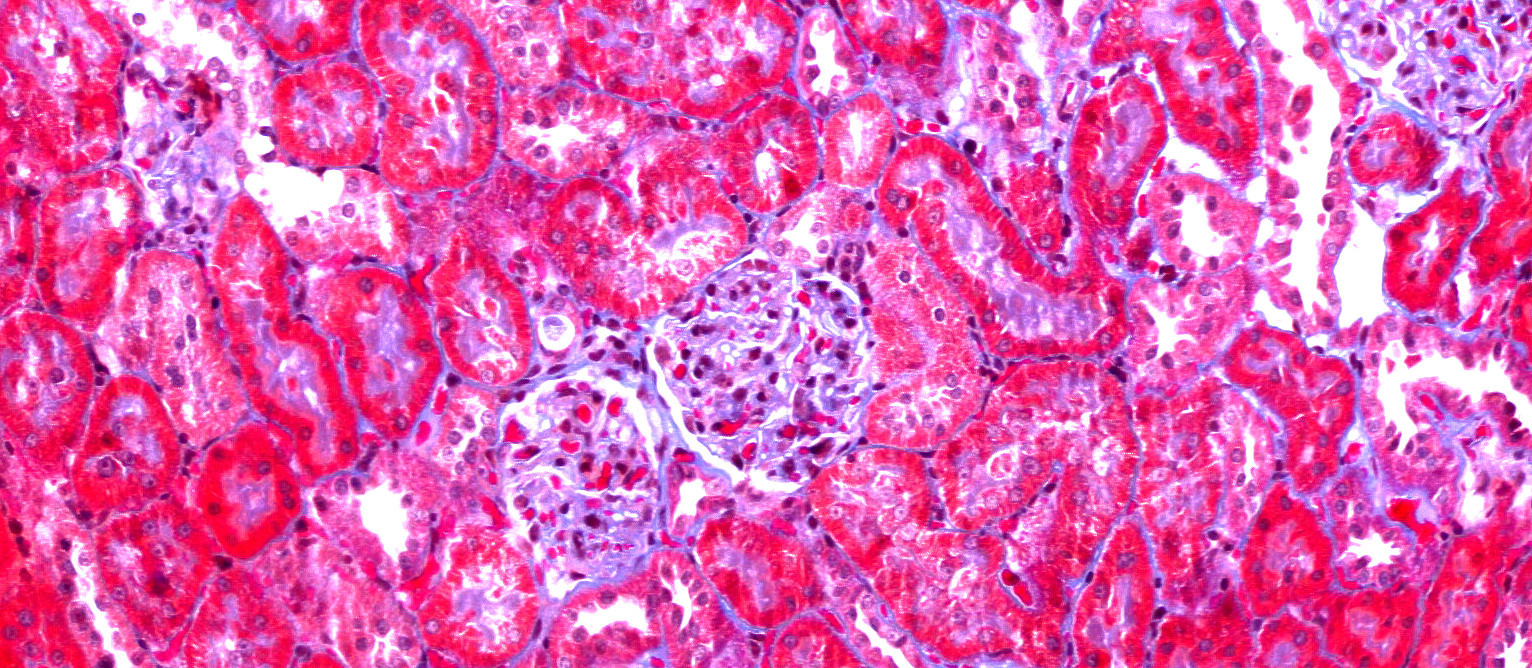

Supplement: Supplementary file 9 [file DataSheet10.ZIP › Fig 1D-masson-DKD-15/15-2.jpeg]

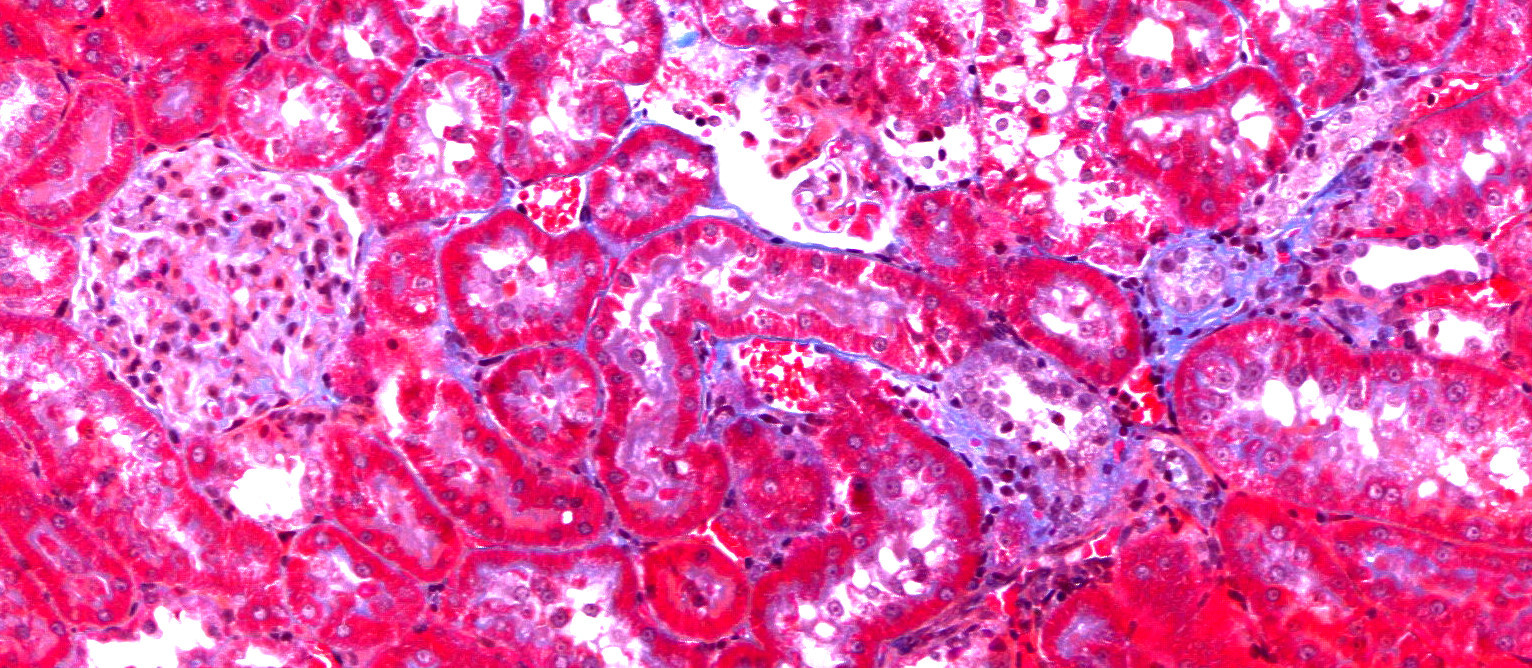

Supplement: Supplementary file 9 [file DataSheet10.ZIP › Fig 1D-masson-DKD-15/15-3.jpeg]

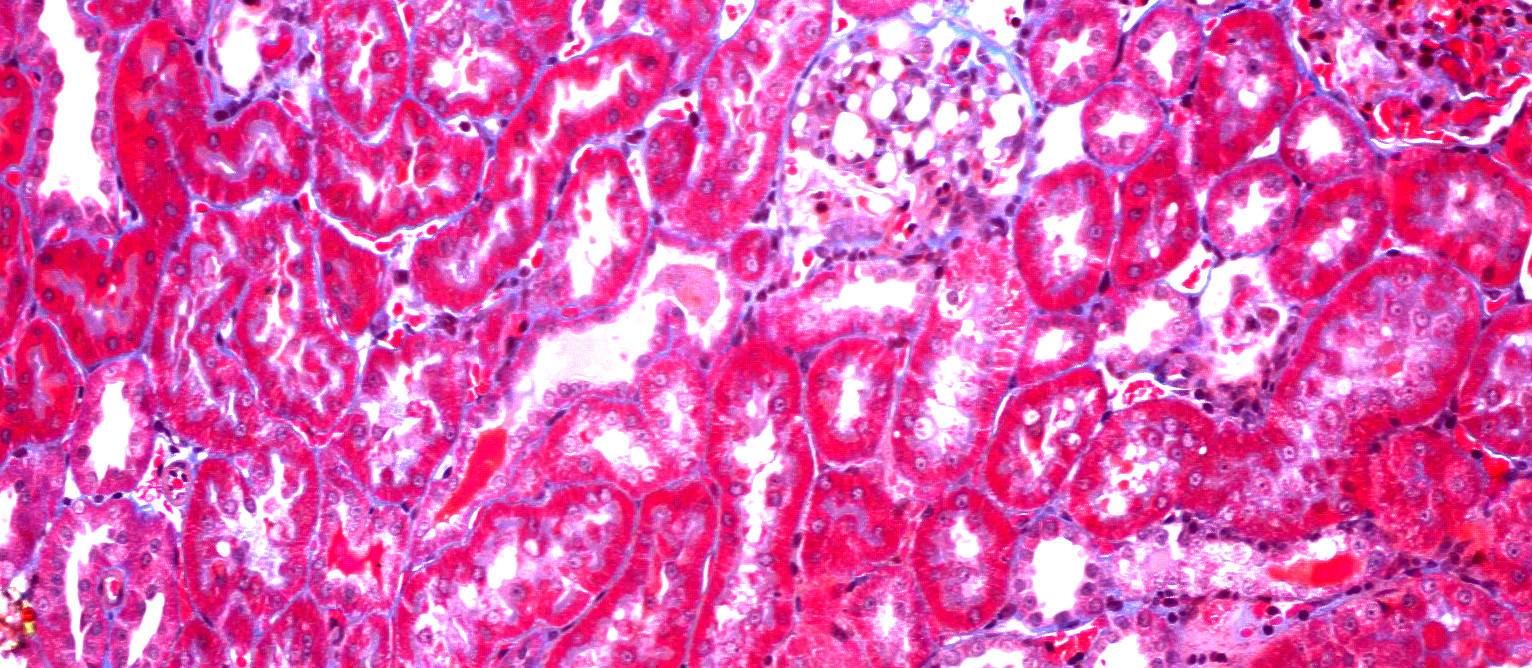

Supplement: Supplementary file 9 [file DataSheet10.ZIP › Fig 1D-masson-DKD-15/15-4.jpeg]

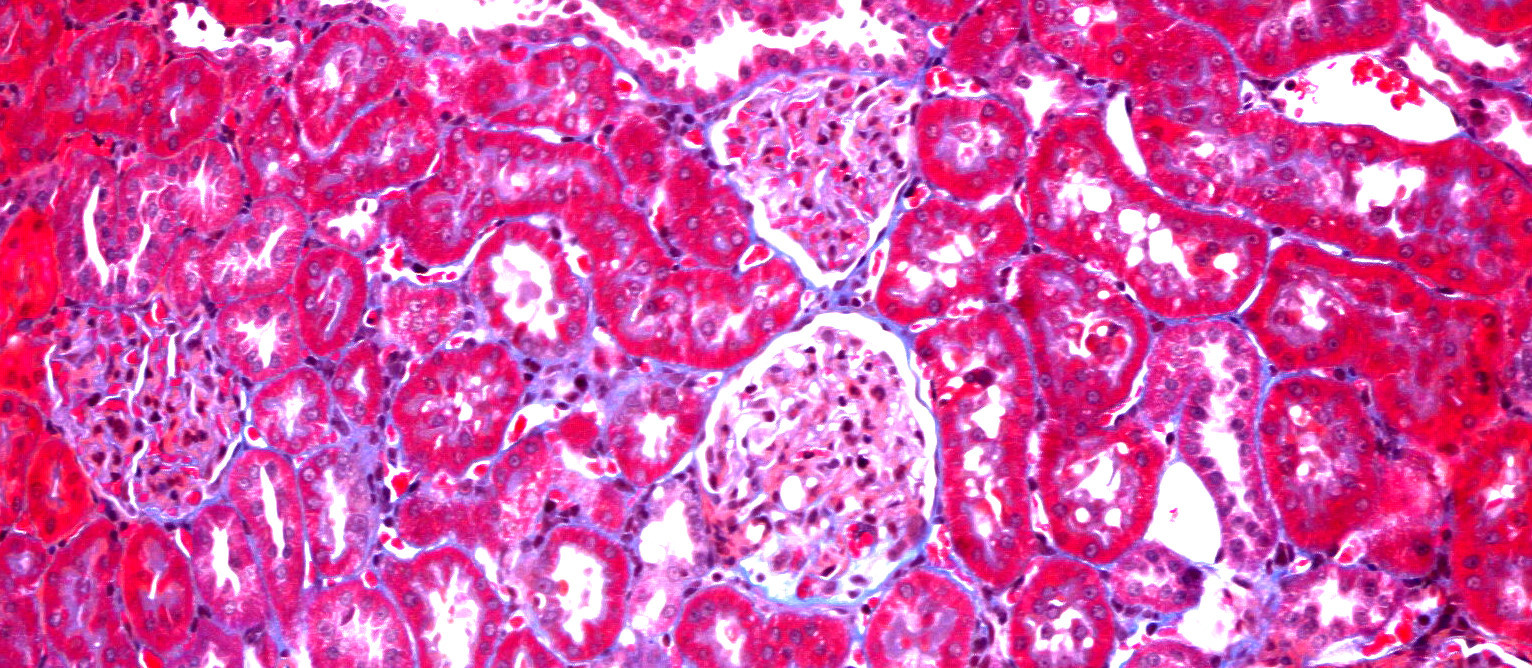

Supplement: Supplementary file 9 [file DataSheet10.ZIP › Fig 1D-masson-DKD-15/15-5.jpeg]

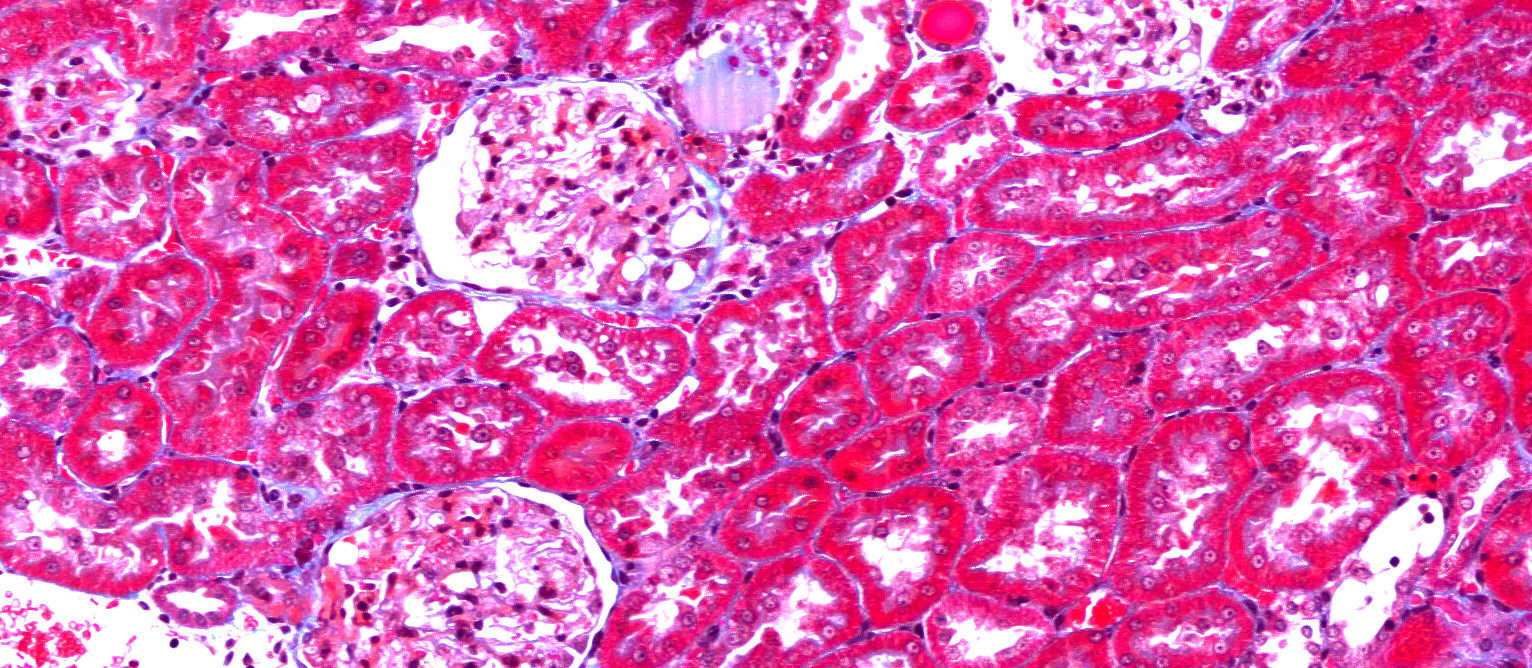

Supplement: Supplementary file 9 [file DataSheet10.ZIP › Fig 1D-masson-DKD-15/15-6.jpeg]

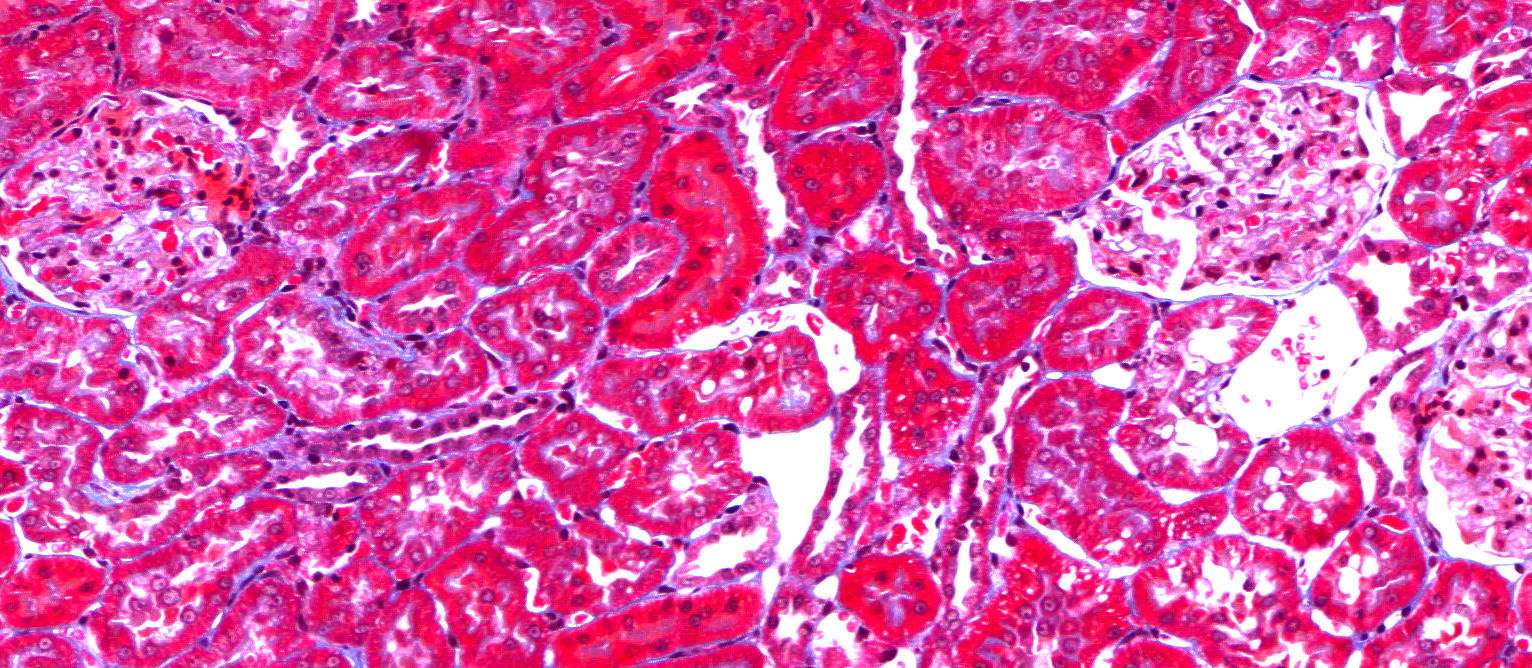

Supplement: Supplementary file 9 [file DataSheet10.ZIP › Fig 1D-masson-DKD-15/15-7.jpeg]

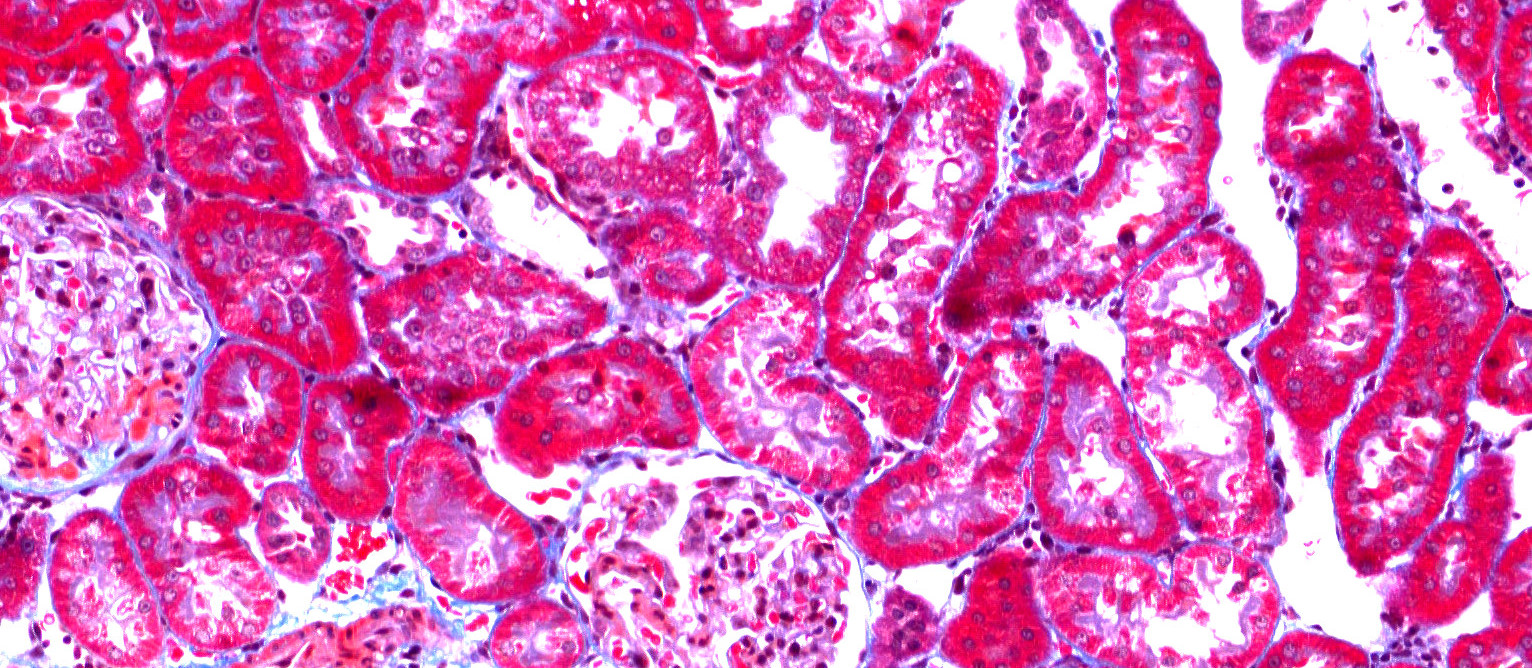

Supplement: Supplementary file 9 [file DataSheet10.ZIP › Fig 1D-masson-DKD-15/15-8.jpeg]

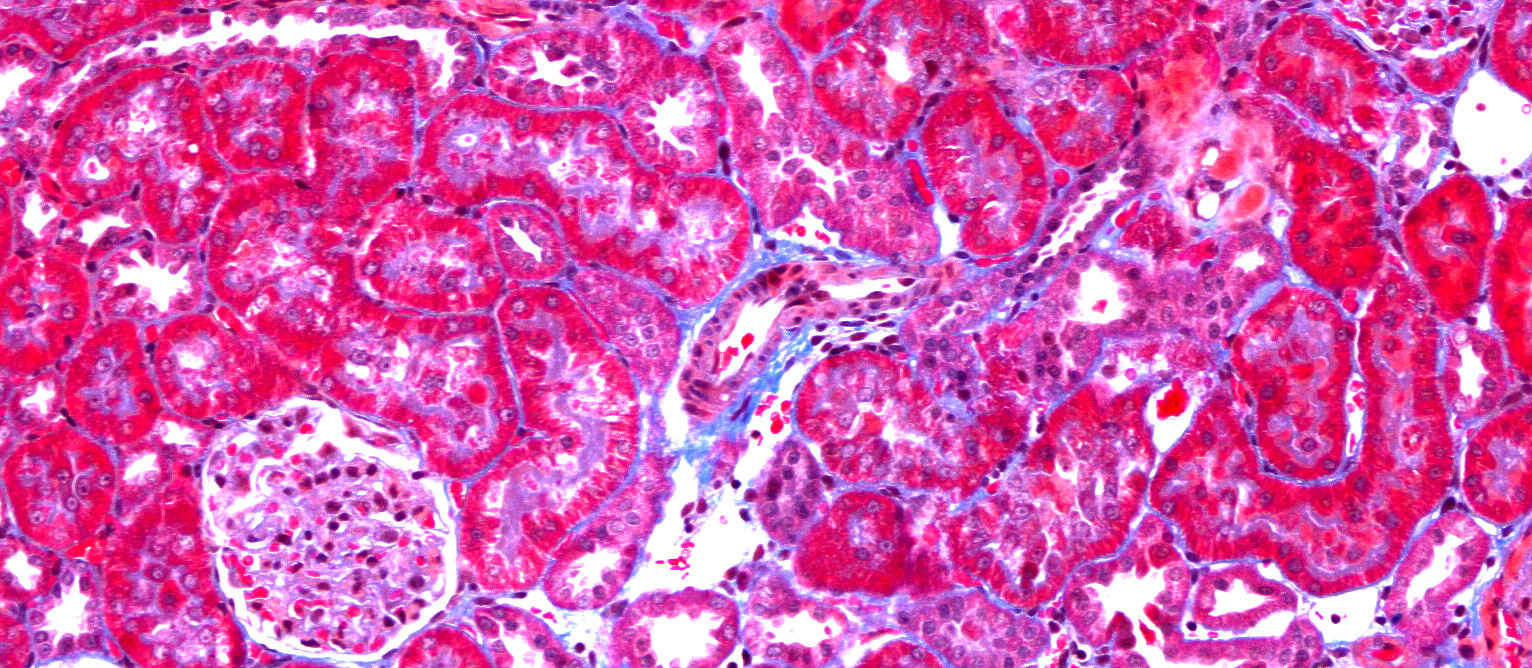

Supplement: Supplementary file 9 [file DataSheet10.ZIP › Fig 1D-masson-DKD-15/15-9.jpeg]

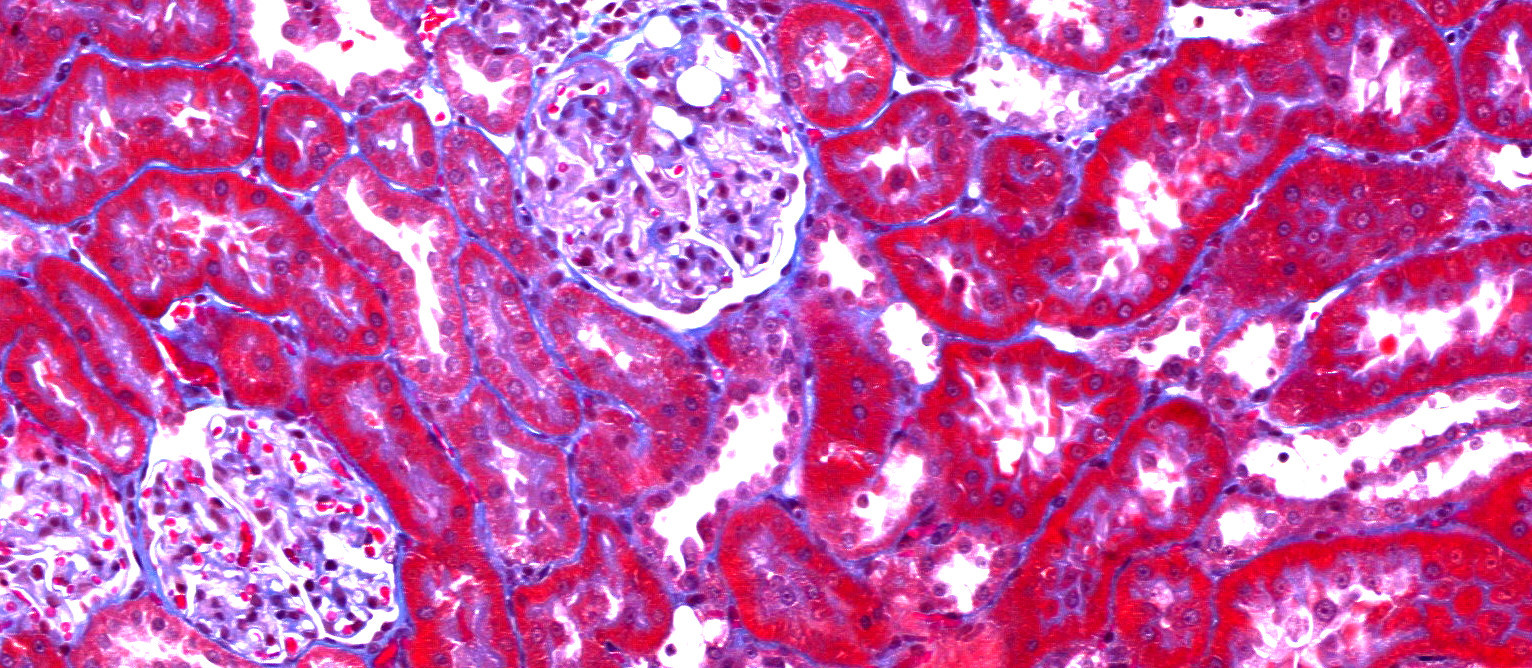

Supplement: Supplementary file 9 [file DataSheet10.ZIP › Fig 1D-masson-DKD-17/17-1.jpeg]

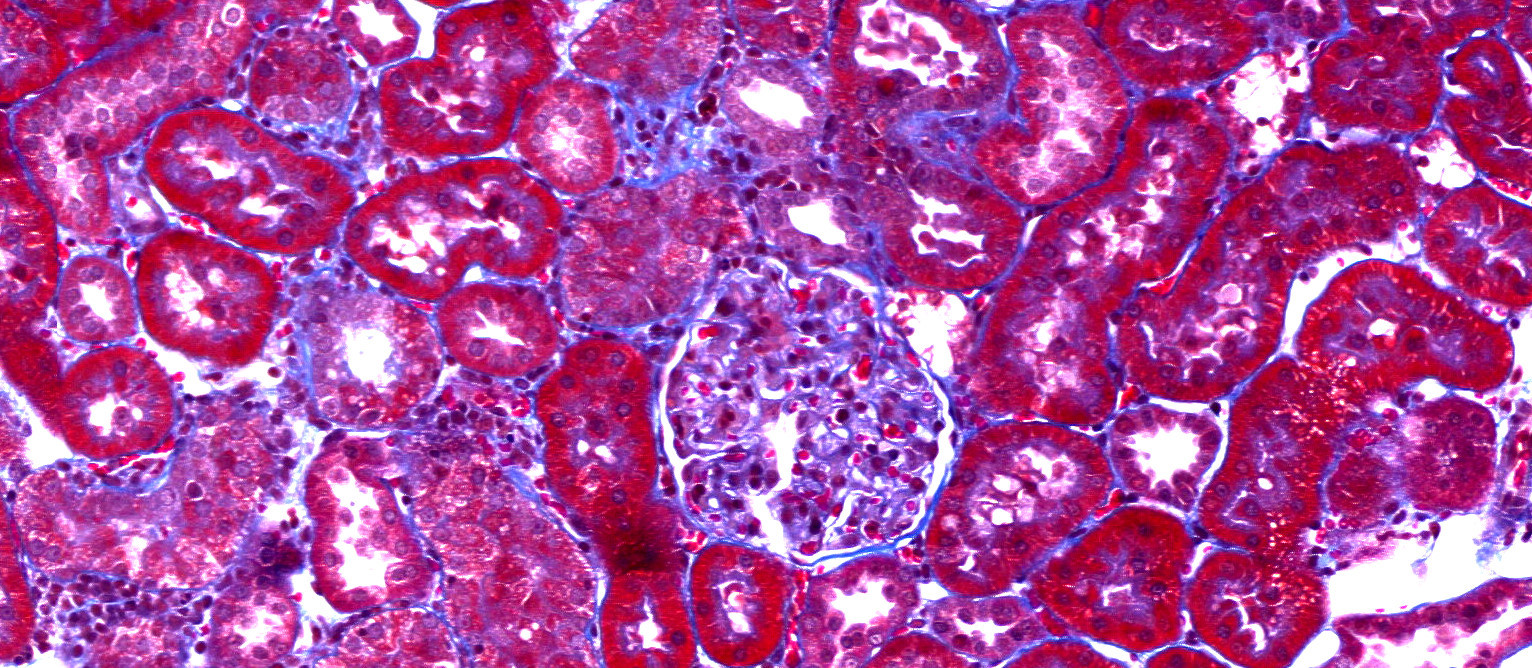

Supplement: Supplementary file 9 [file DataSheet10.ZIP › Fig 1D-masson-DKD-17/17-10.jpeg]

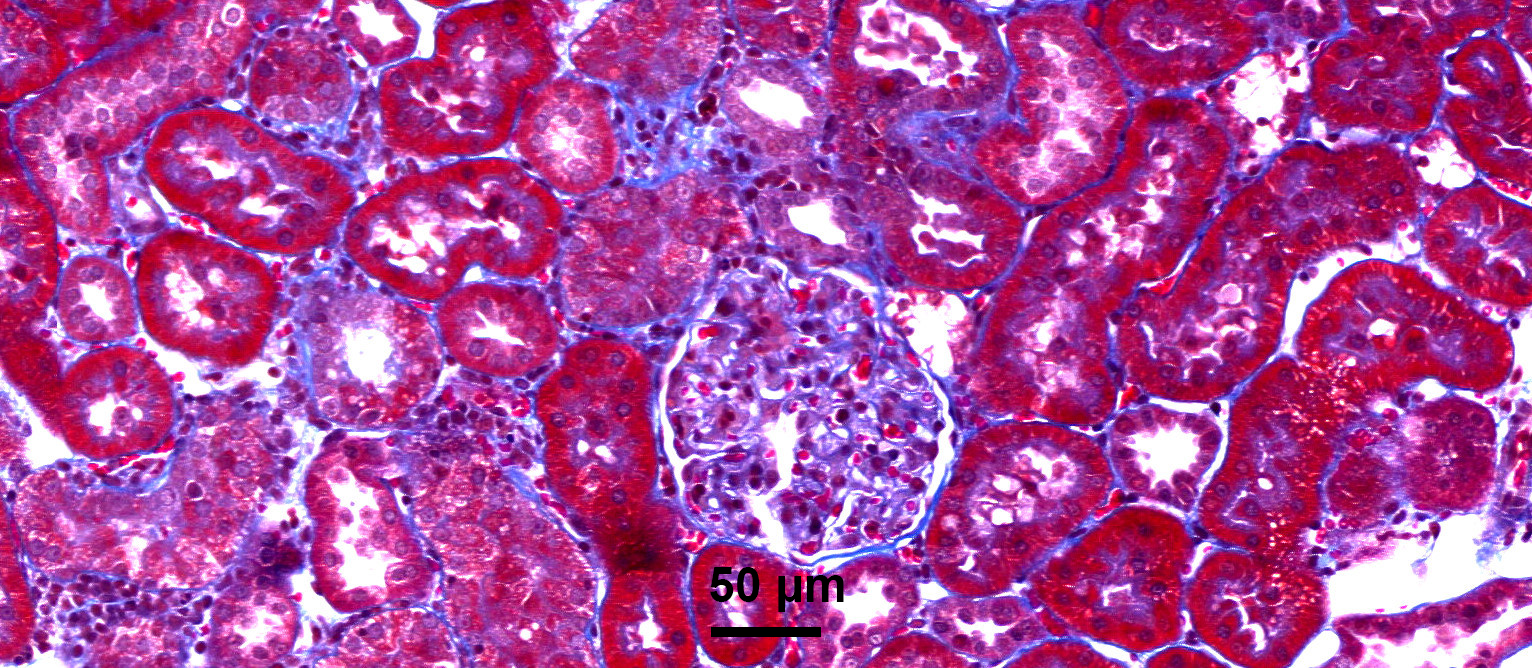

Supplement: Supplementary file 9 [file DataSheet10.ZIP › Fig 1D-masson-DKD-17/17-10-1.png]

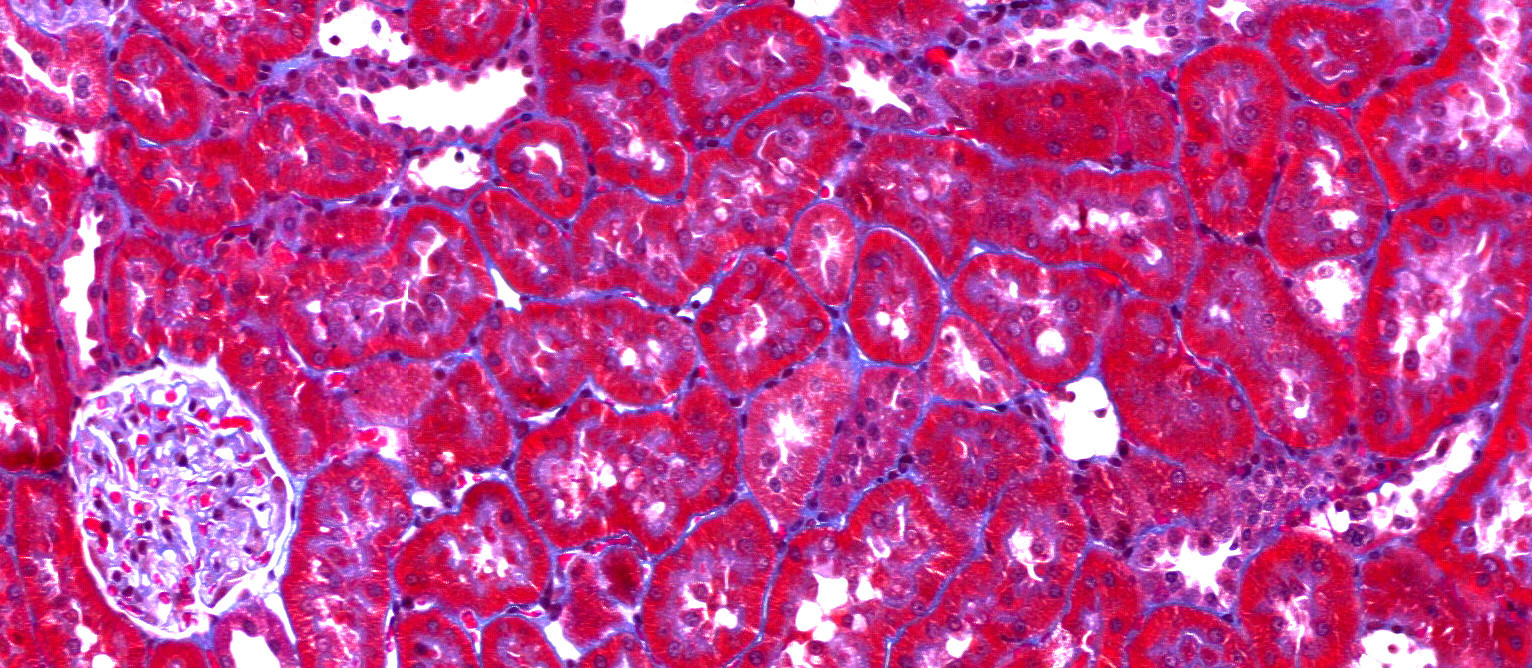

Supplement: Supplementary file 9 [file DataSheet10.ZIP › Fig 1D-masson-DKD-17/17-2.jpeg]

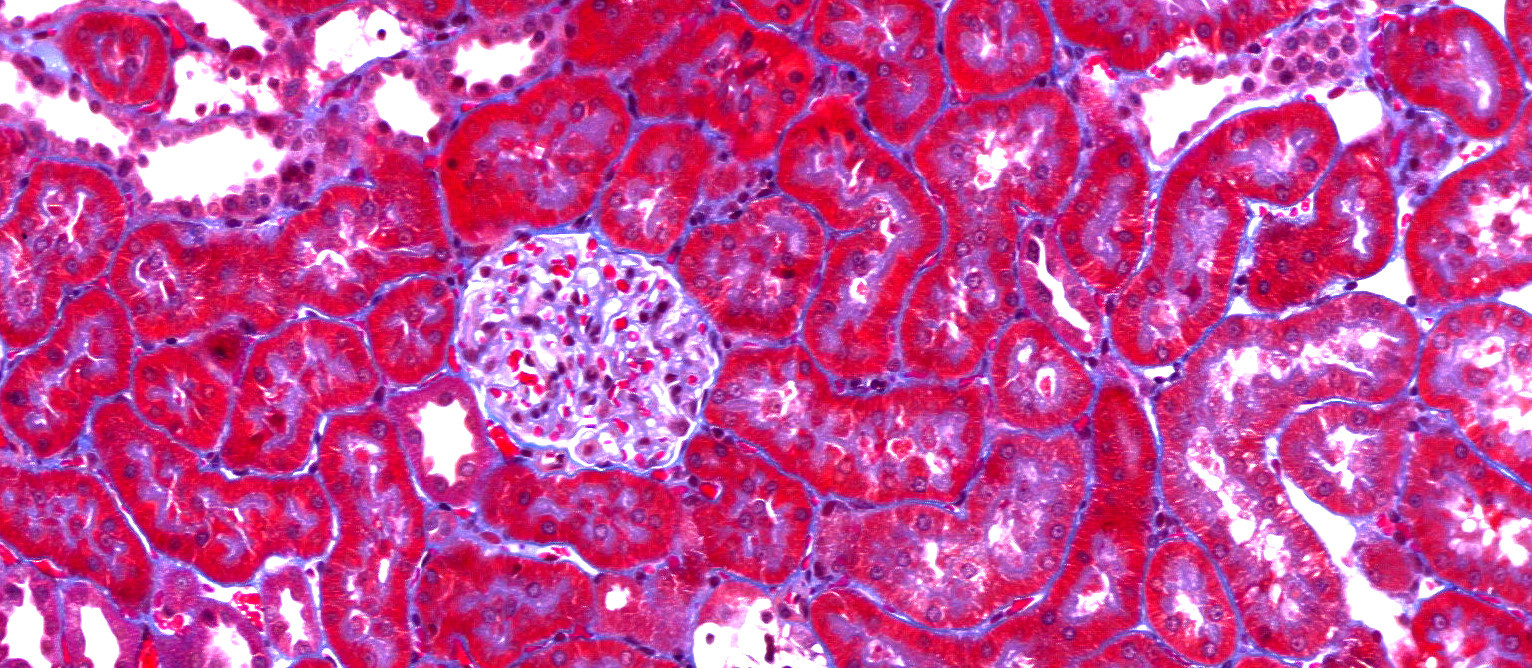

Supplement: Supplementary file 9 [file DataSheet10.ZIP › Fig 1D-masson-DKD-17/17-3.jpeg]

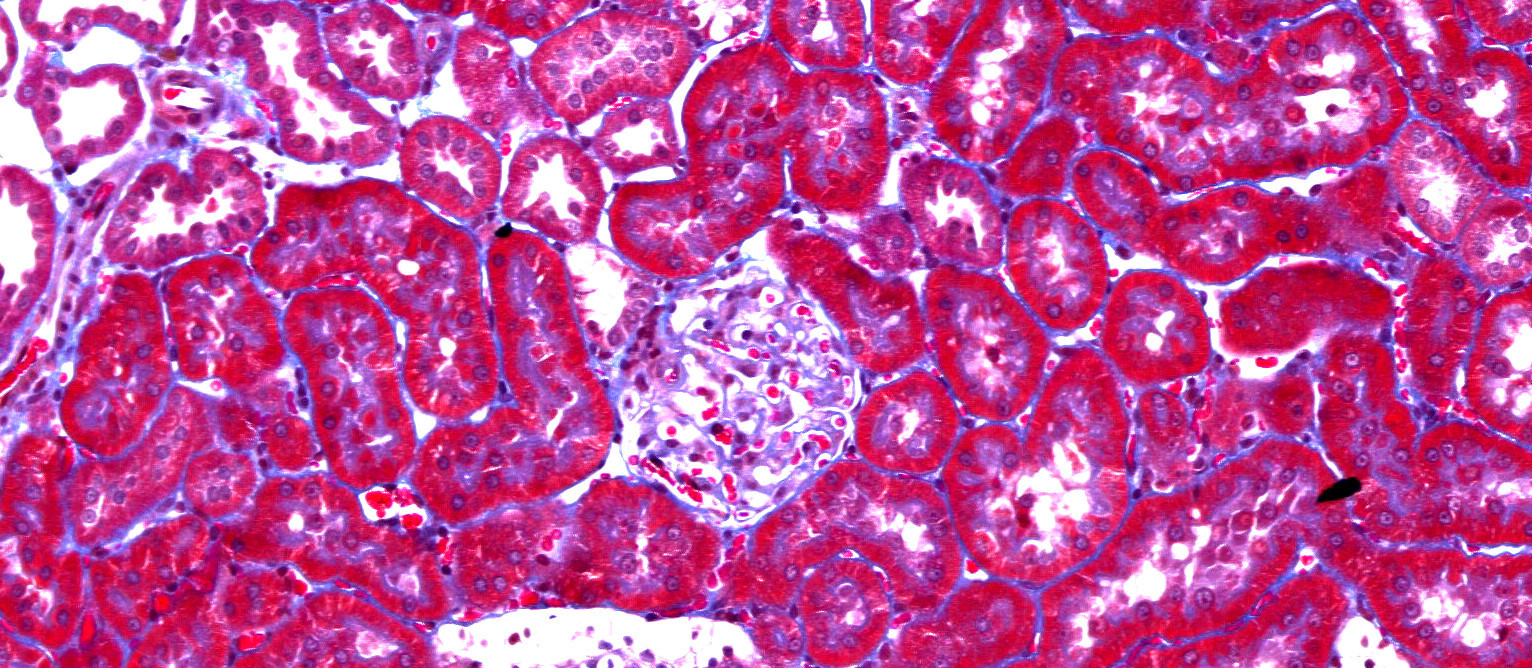

Supplement: Supplementary file 9 [file DataSheet10.ZIP › Fig 1D-masson-DKD-17/17-4.jpeg]

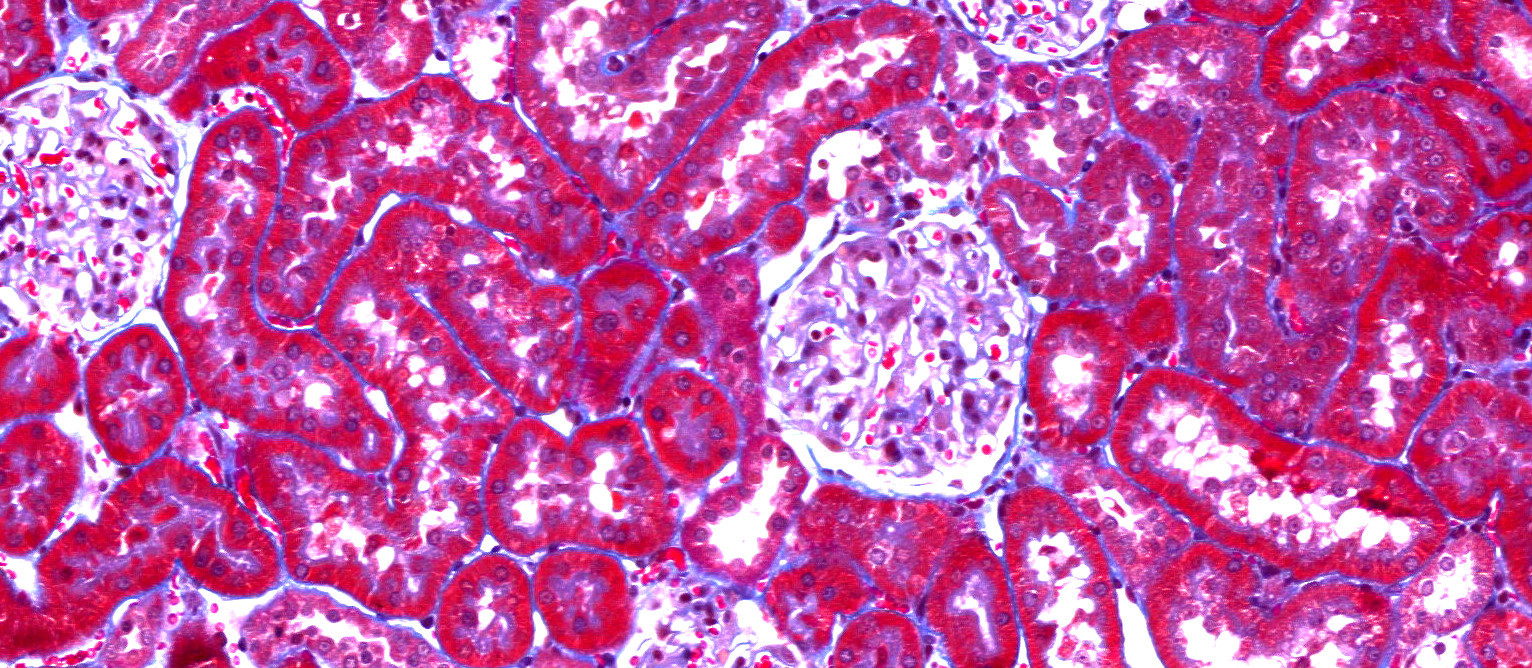

Supplement: Supplementary file 9 [file DataSheet10.ZIP › Fig 1D-masson-DKD-17/17-5.jpeg]

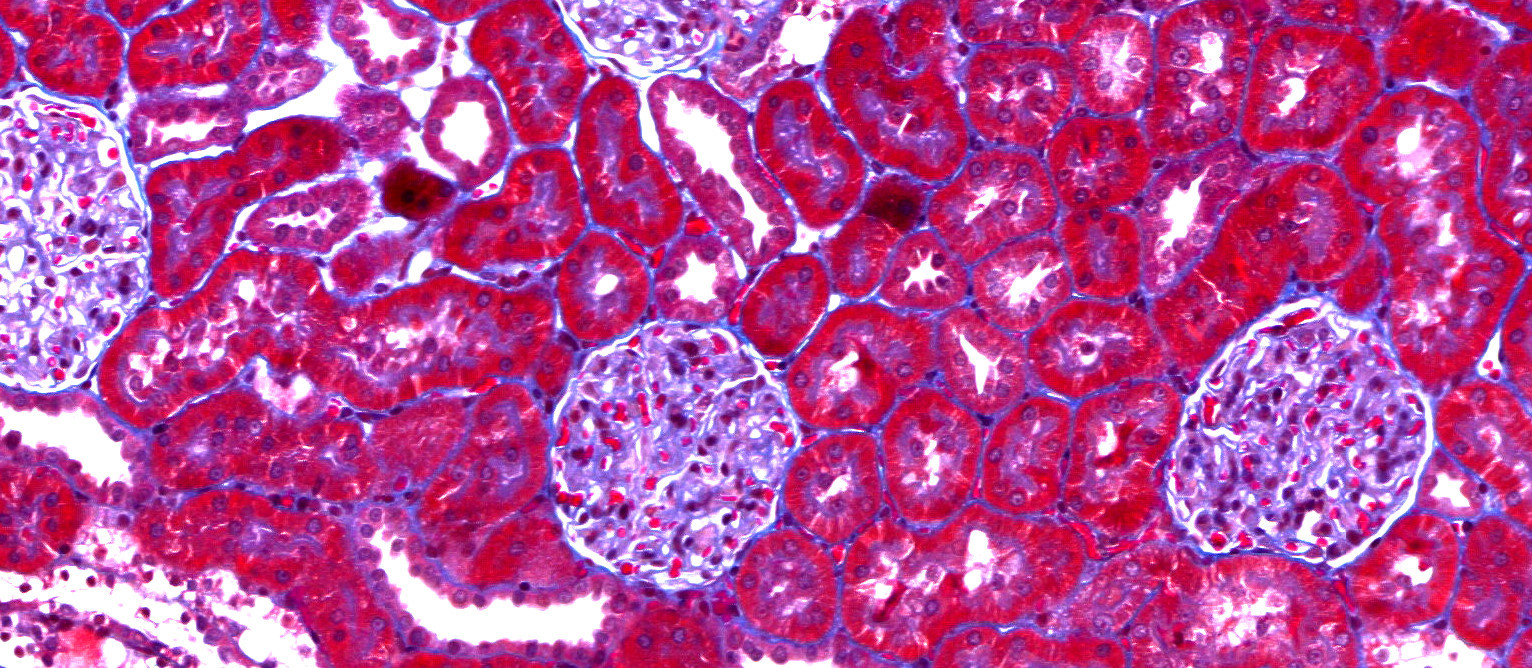

Supplement: Supplementary file 9 [file DataSheet10.ZIP › Fig 1D-masson-DKD-17/17-6.jpeg]

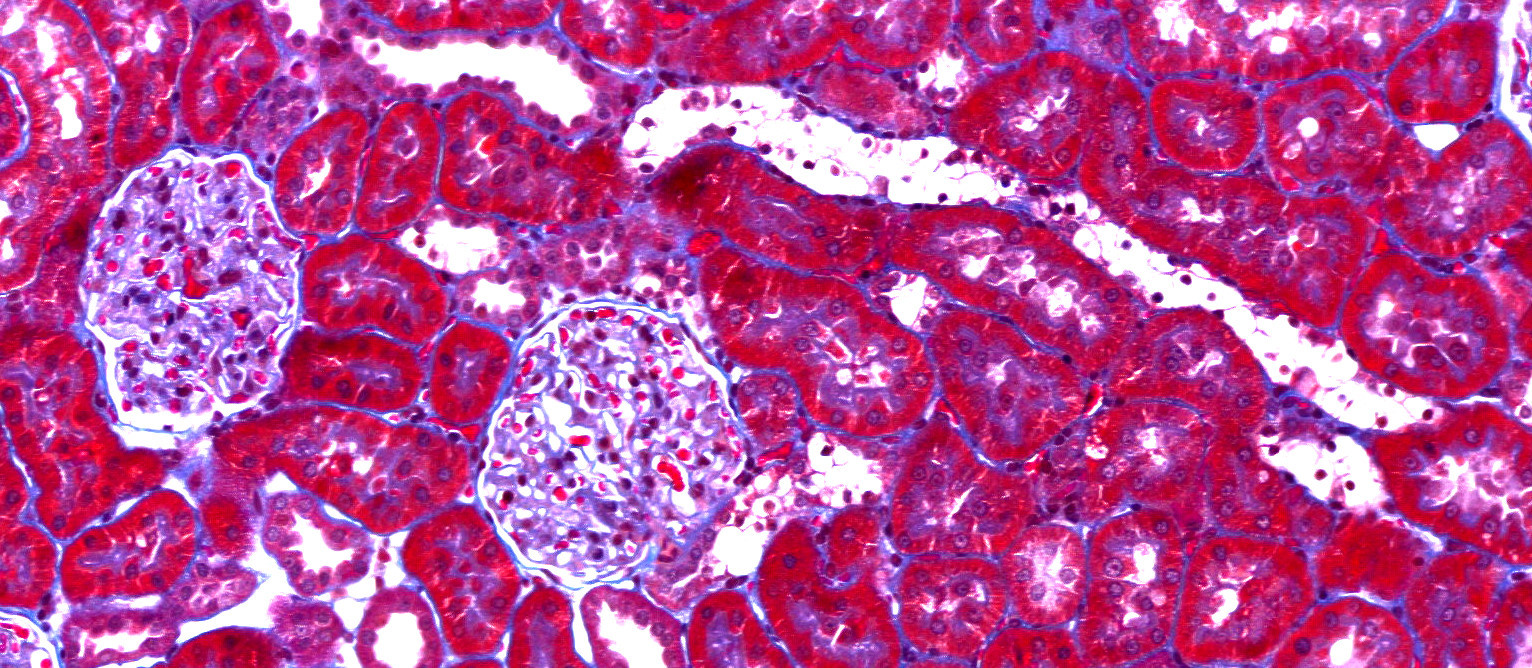

Supplement: Supplementary file 9 [file DataSheet10.ZIP › Fig 1D-masson-DKD-17/17-7.jpeg]

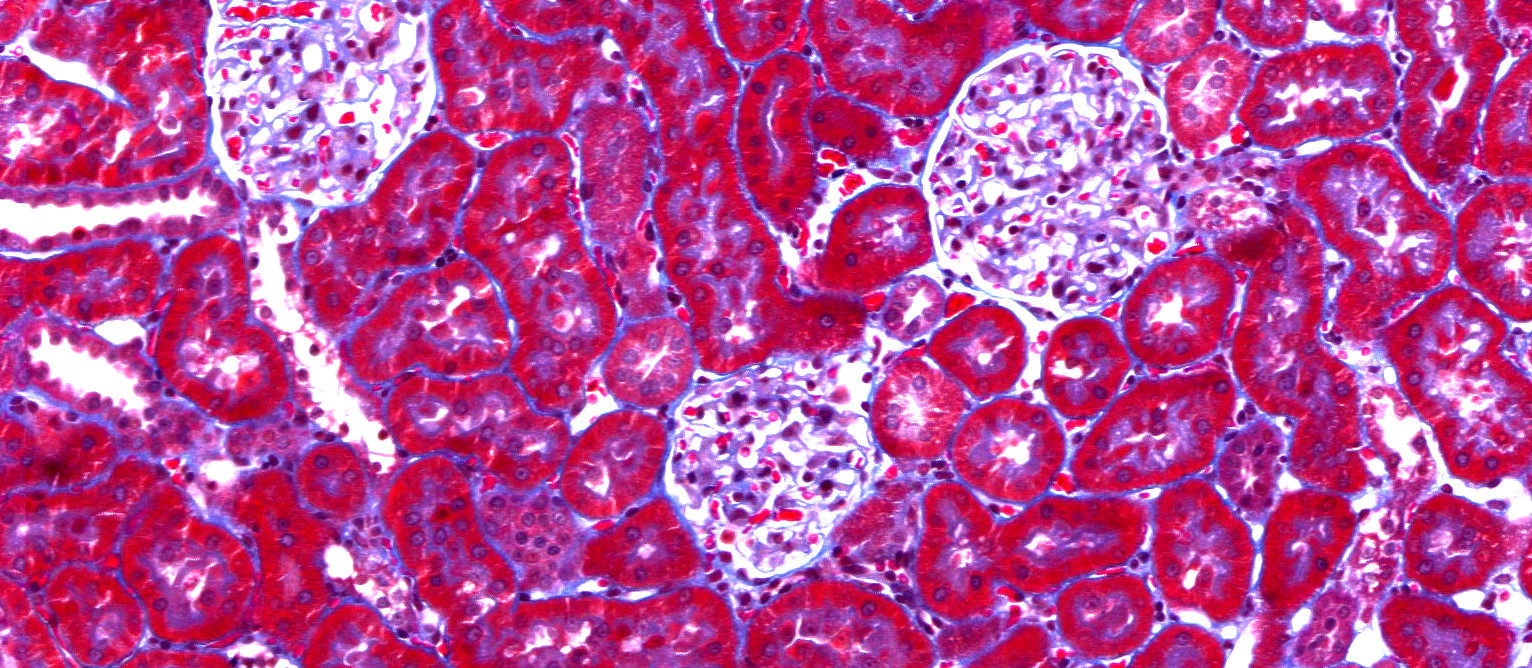

Supplement: Supplementary file 9 [file DataSheet10.ZIP › Fig 1D-masson-DKD-17/17-8.jpeg]

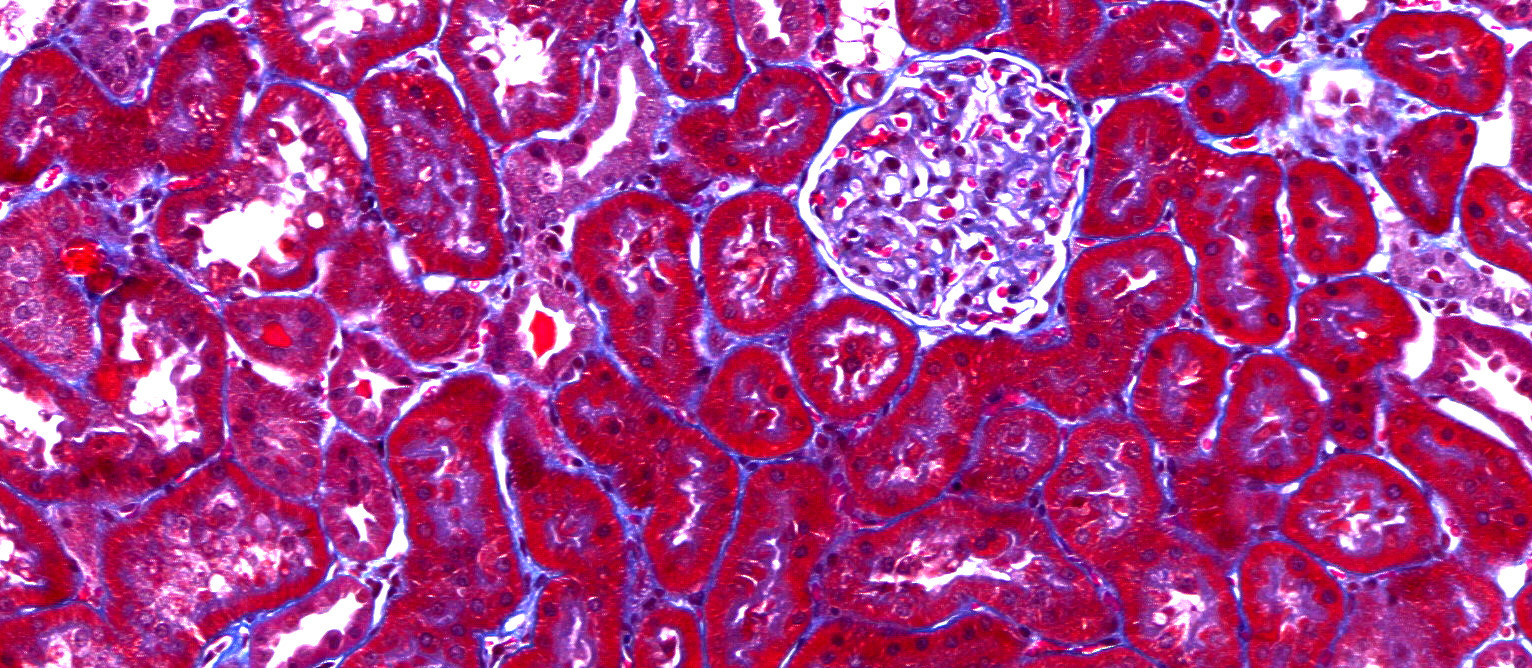

Supplement: Supplementary file 9 [file DataSheet10.ZIP › Fig 1D-masson-DKD-17/17-9.jpeg]

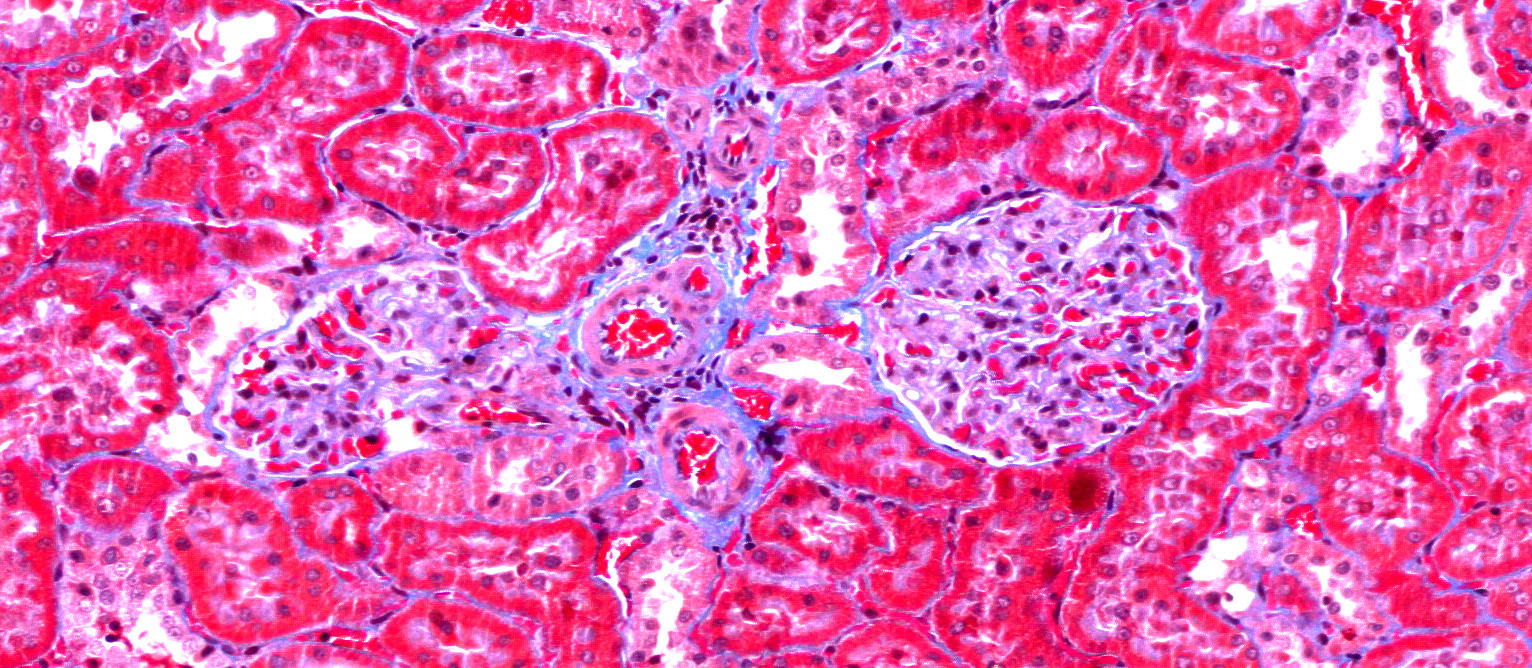

Supplement: Supplementary file 9 [file DataSheet10.ZIP › Fig 1D-masson-DKD-18(1)/18-1.jpeg]

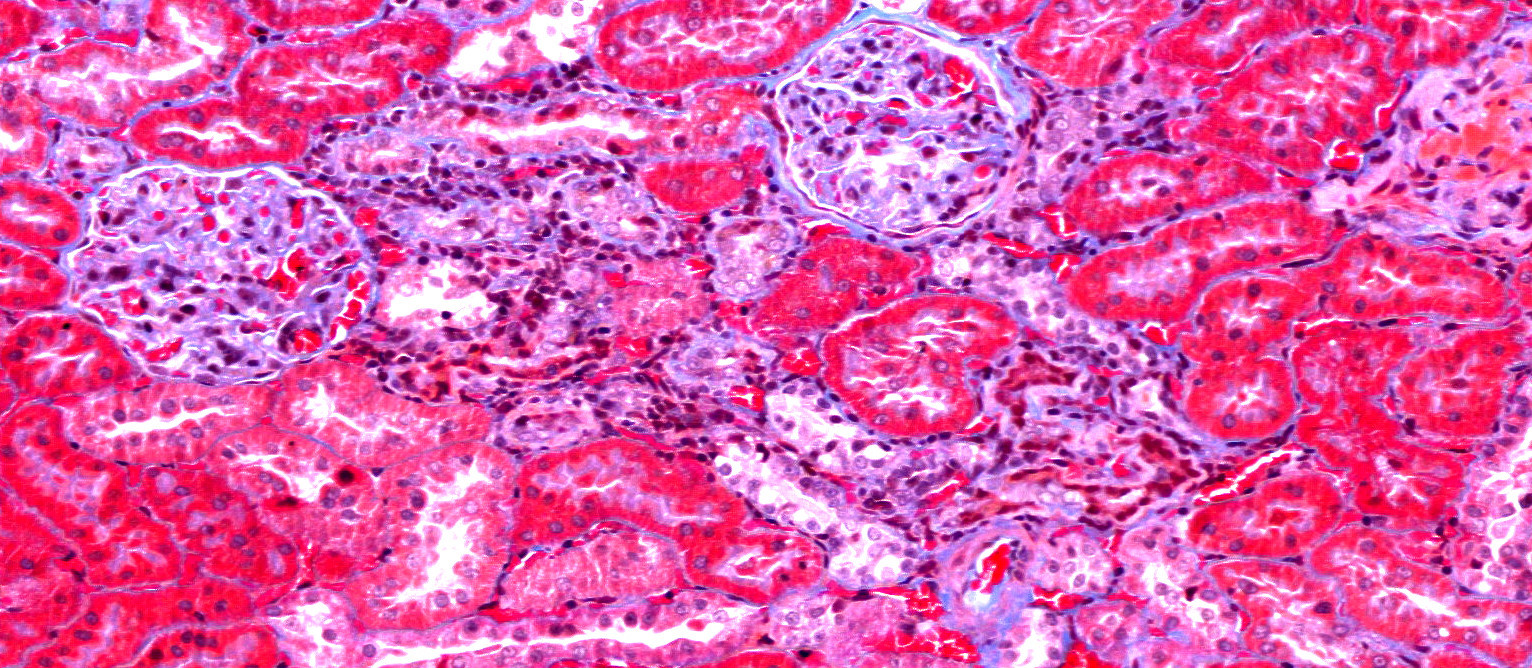

Supplement: Supplementary file 9 [file DataSheet10.ZIP › Fig 1D-masson-DKD-18(1)/18-2.jpeg]

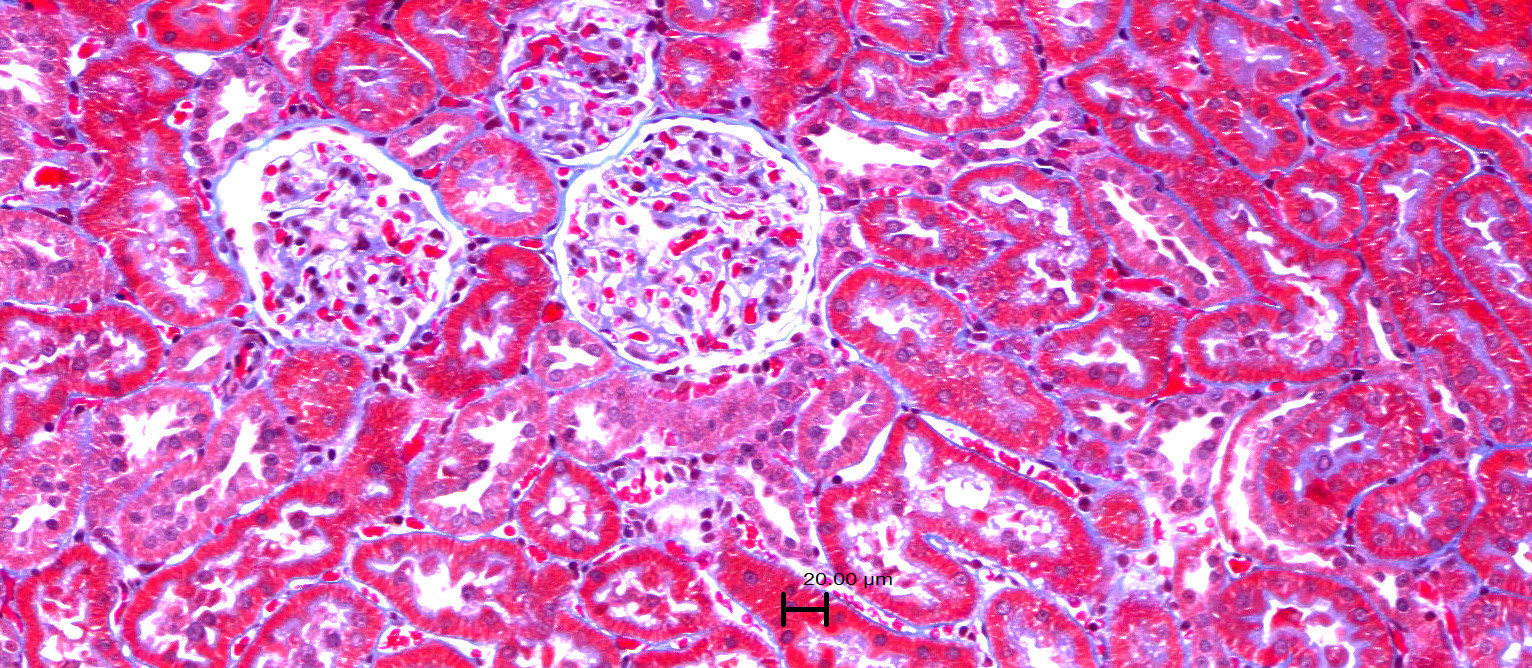

Supplement: Supplementary file 9 [file DataSheet10.ZIP › Fig 1D-masson-sham-10/10-1.jpeg]

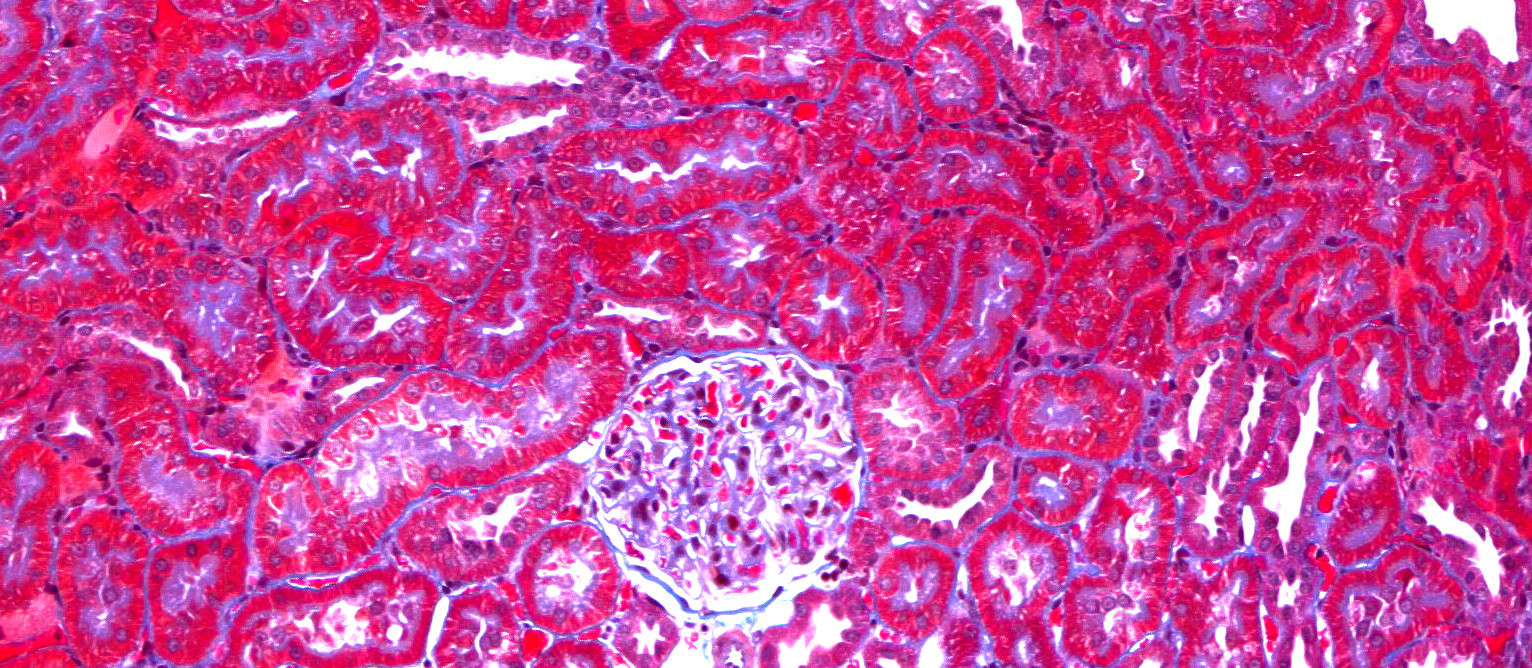

Supplement: Supplementary file 9 [file DataSheet10.ZIP › Fig 1D-masson-sham-10/10-10.jpeg]

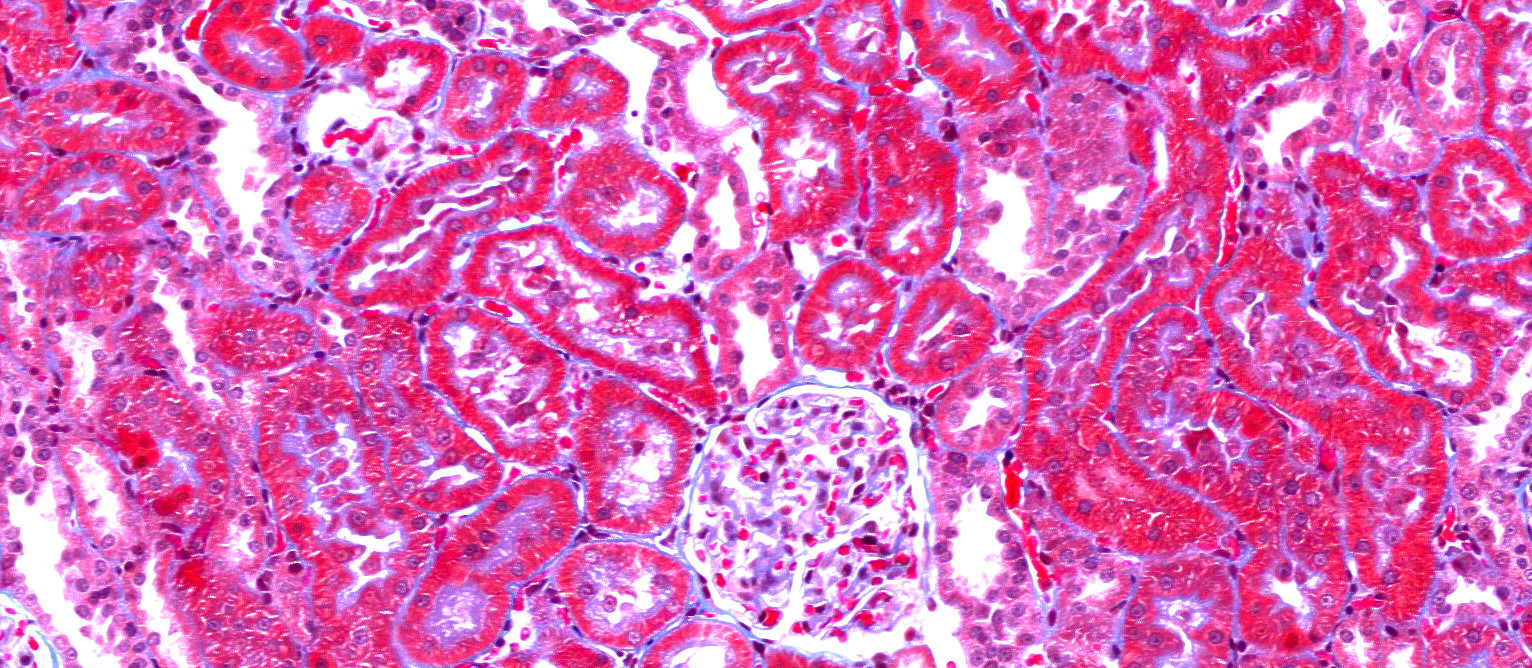

Supplement: Supplementary file 9 [file DataSheet10.ZIP › Fig 1D-masson-sham-10/10-2.jpeg]

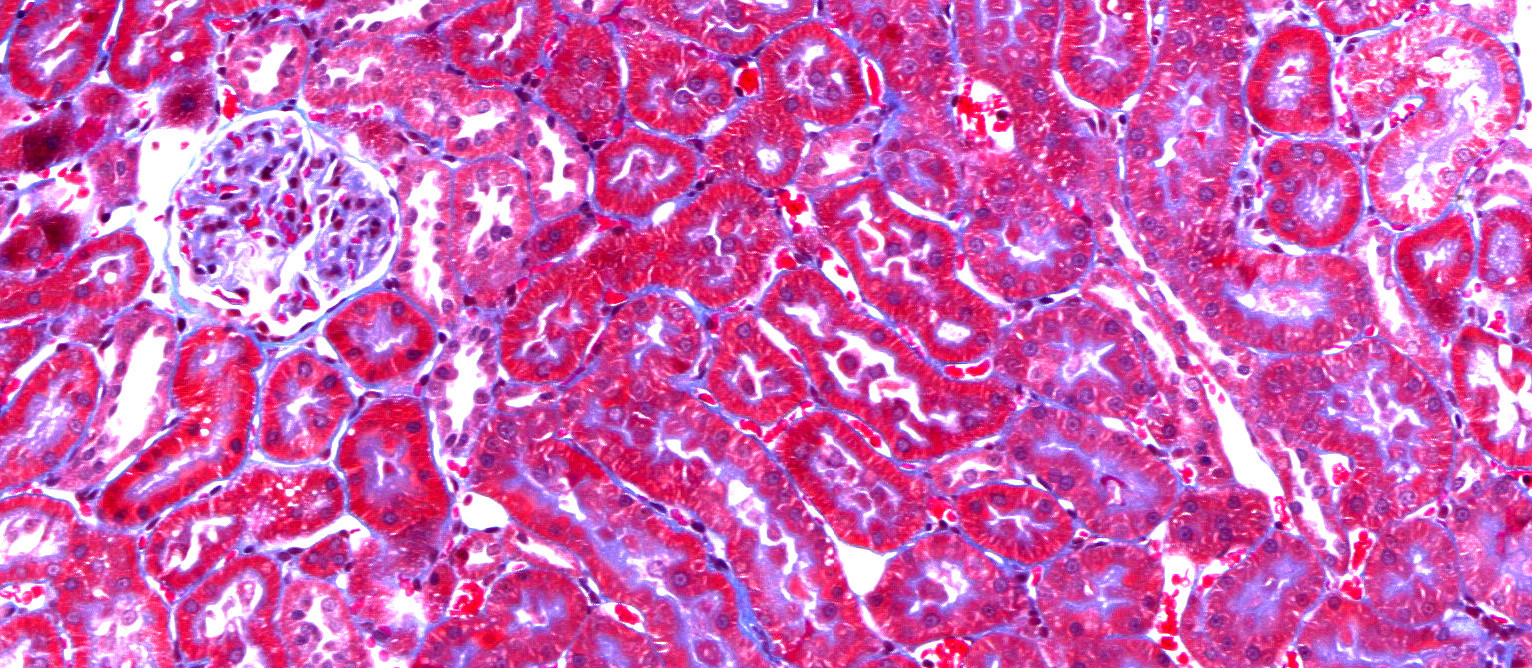

Supplement: Supplementary file 9 [file DataSheet10.ZIP › Fig 1D-masson-sham-10/10-3.jpeg]

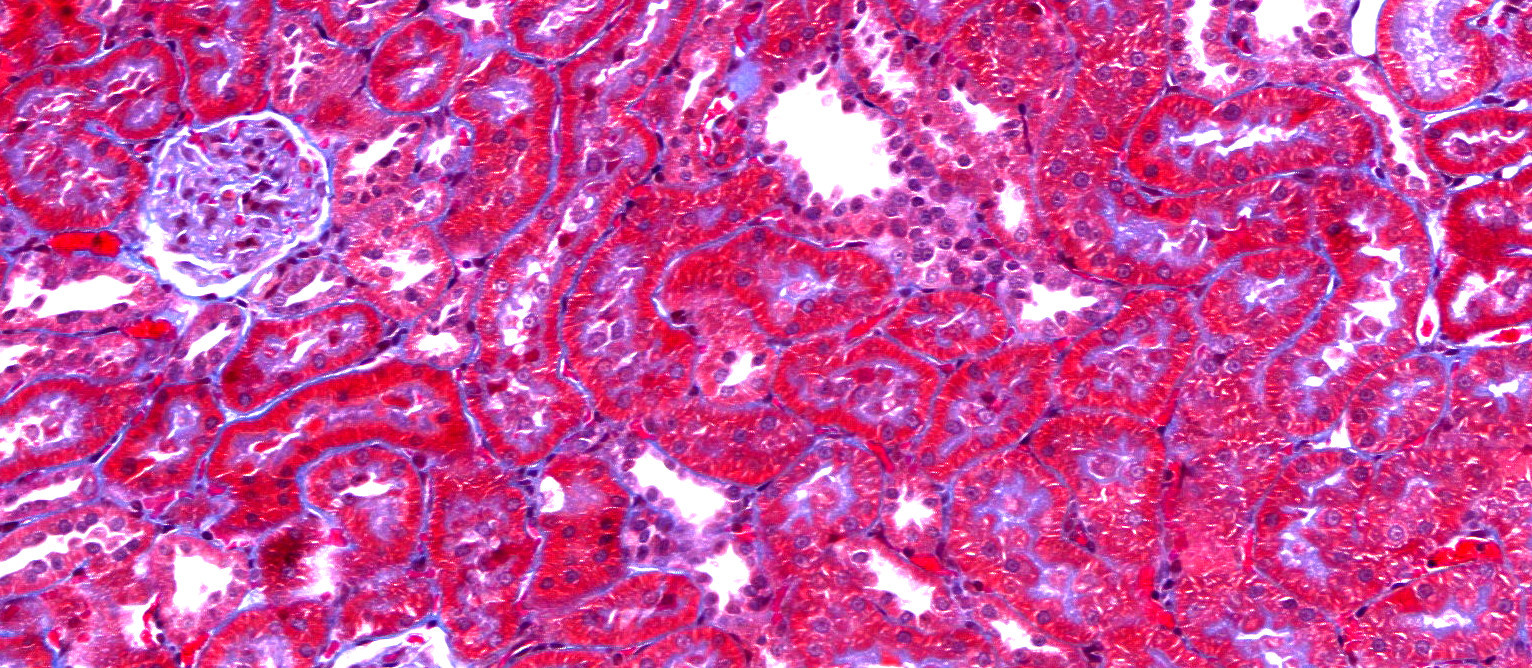

Supplement: Supplementary file 9 [file DataSheet10.ZIP › Fig 1D-masson-sham-10/10-4.jpeg]

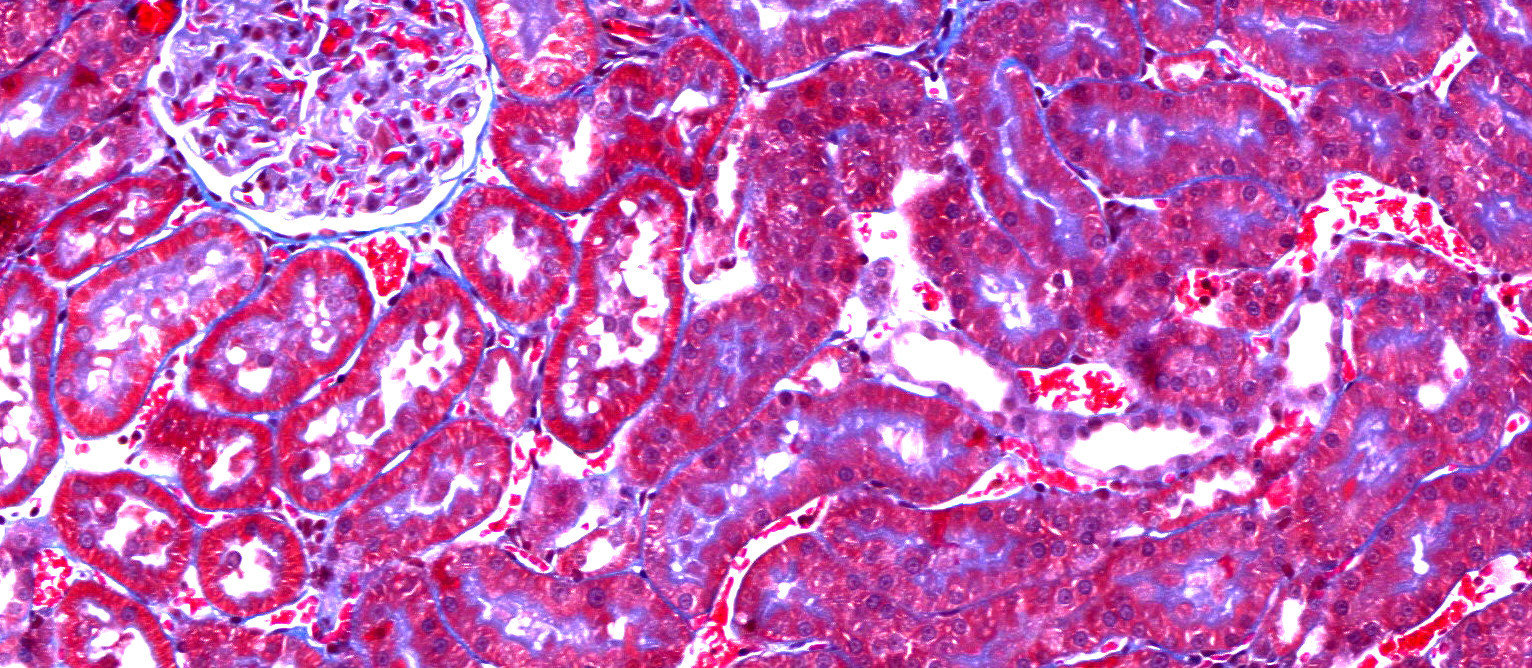

Supplement: Supplementary file 9 [file DataSheet10.ZIP › Fig 1D-masson-sham-10/10-5.jpeg]

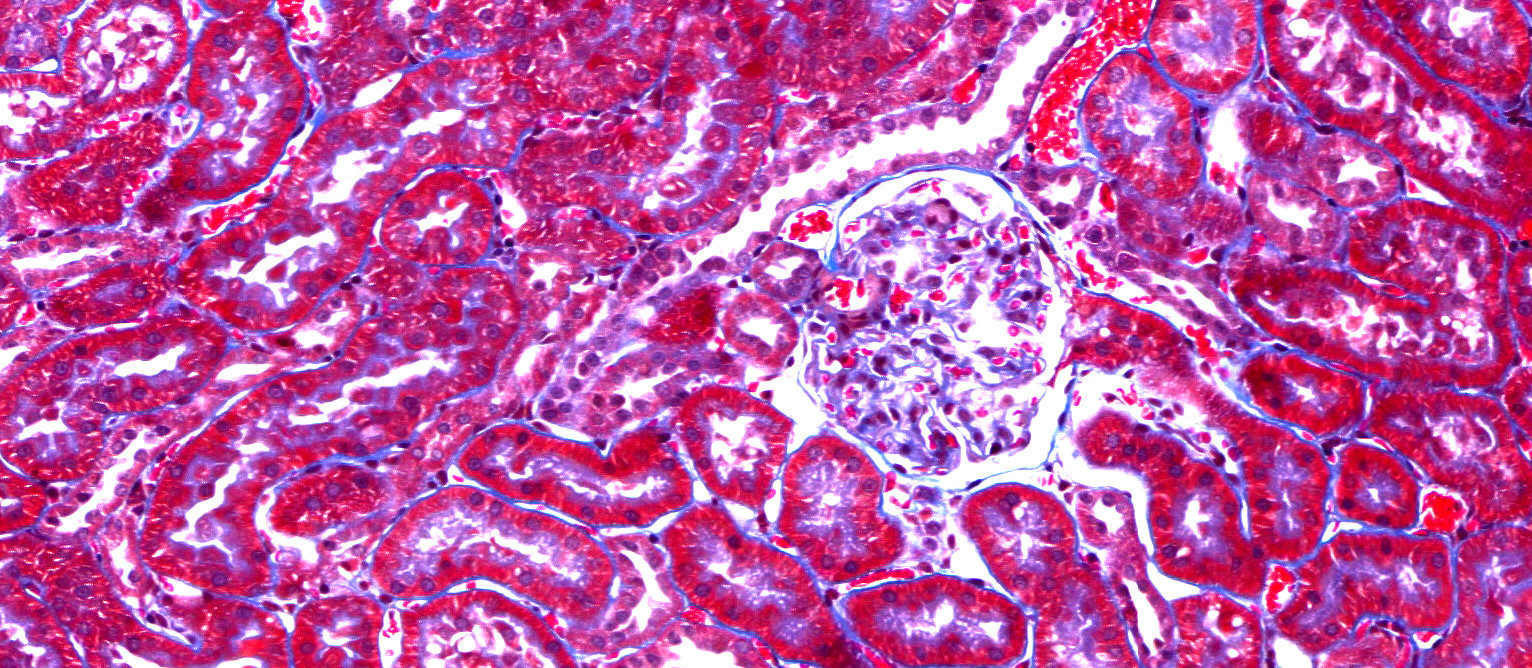

Supplement: Supplementary file 9 [file DataSheet10.ZIP › Fig 1D-masson-sham-10/10-6.jpeg]

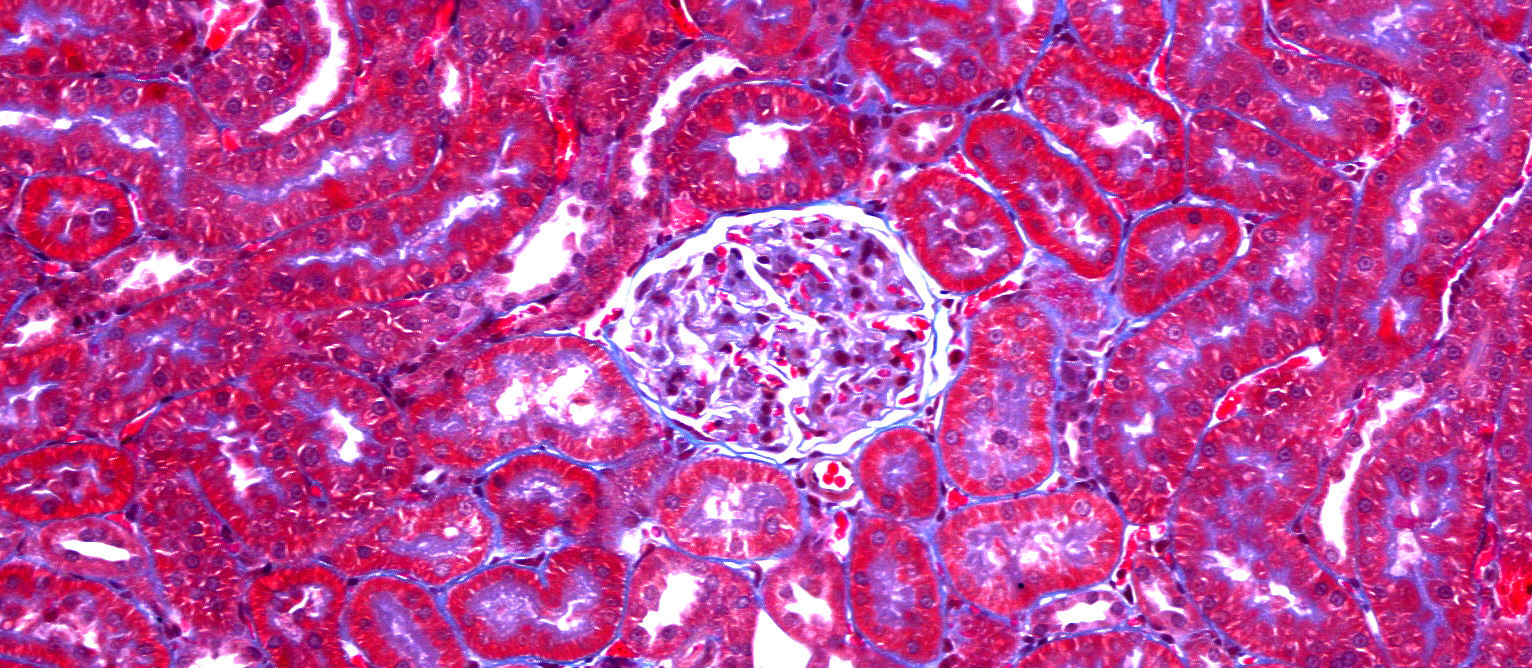

Supplement: Supplementary file 9 [file DataSheet10.ZIP › Fig 1D-masson-sham-10/10-7.jpeg]

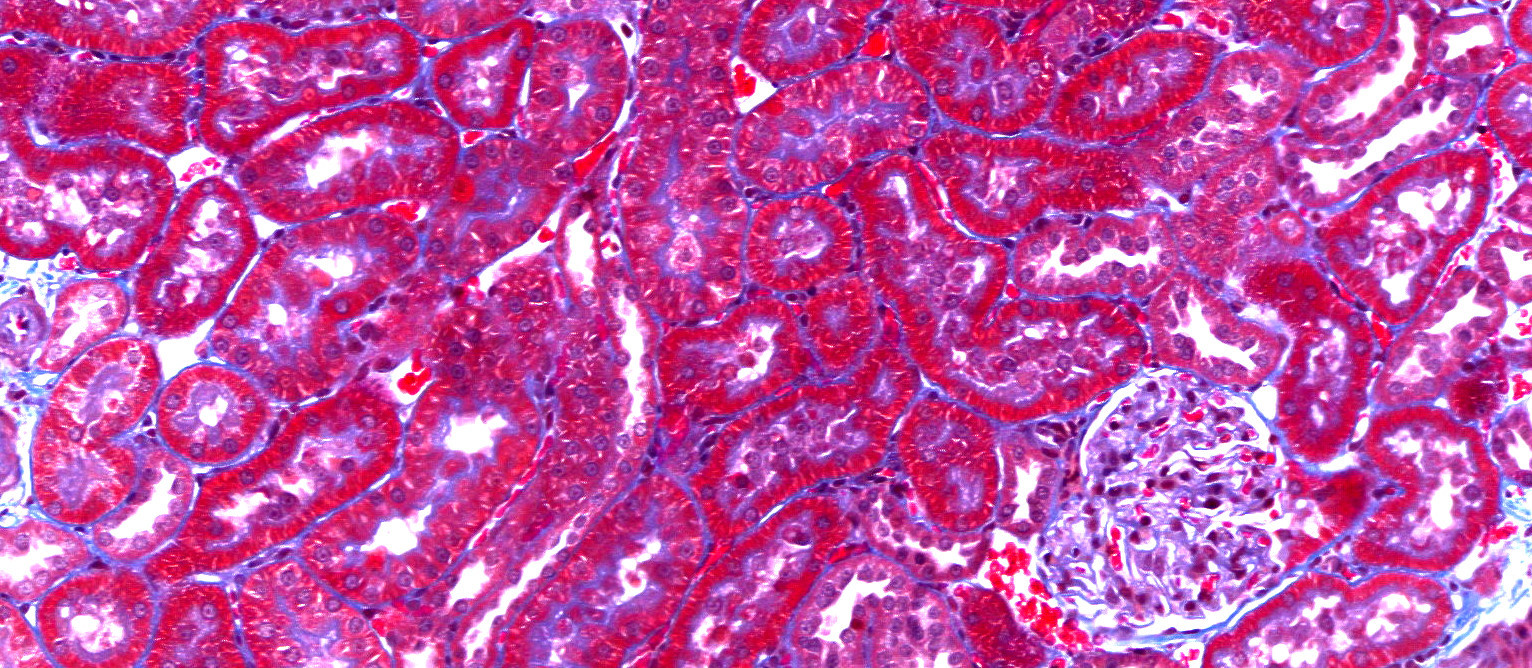

Supplement: Supplementary file 9 [file DataSheet10.ZIP › Fig 1D-masson-sham-10/10-8.jpeg]

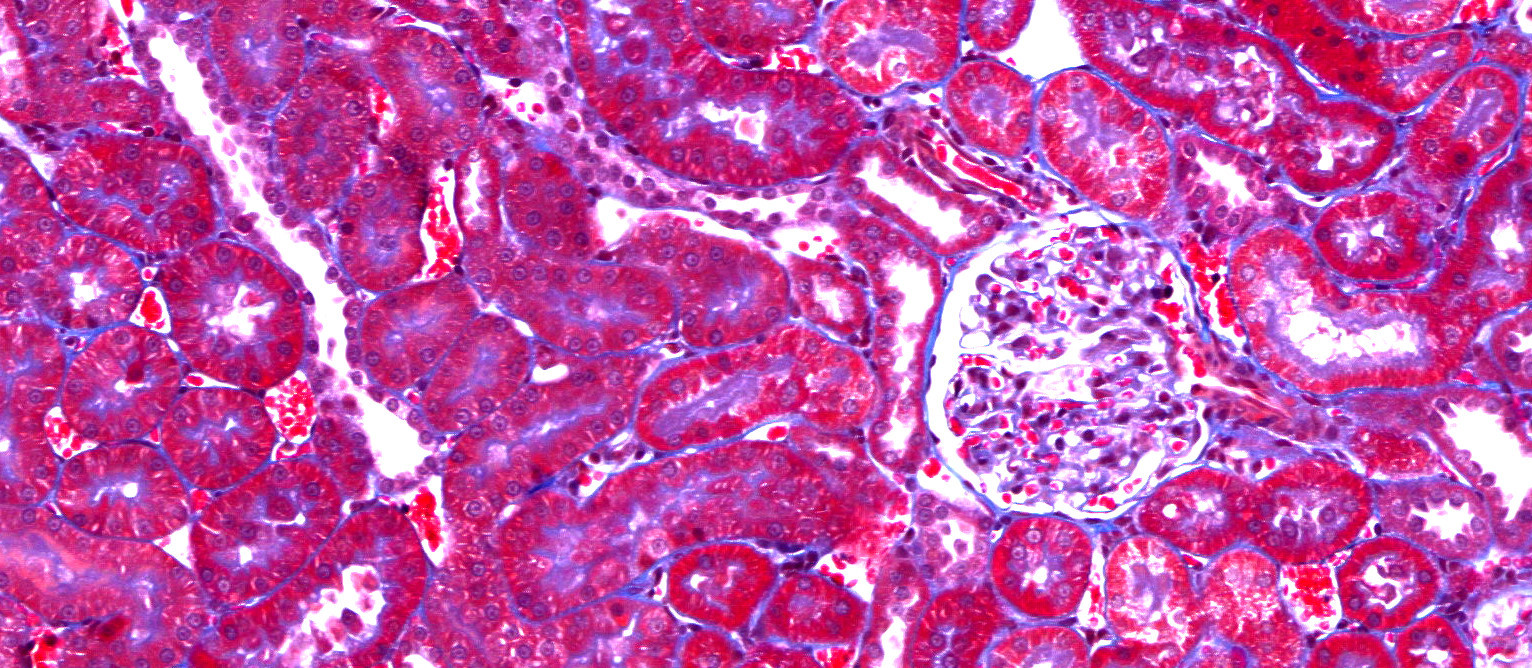

Supplement: Supplementary file 9 [file DataSheet10.ZIP › Fig 1D-masson-sham-10/10-9.jpeg]

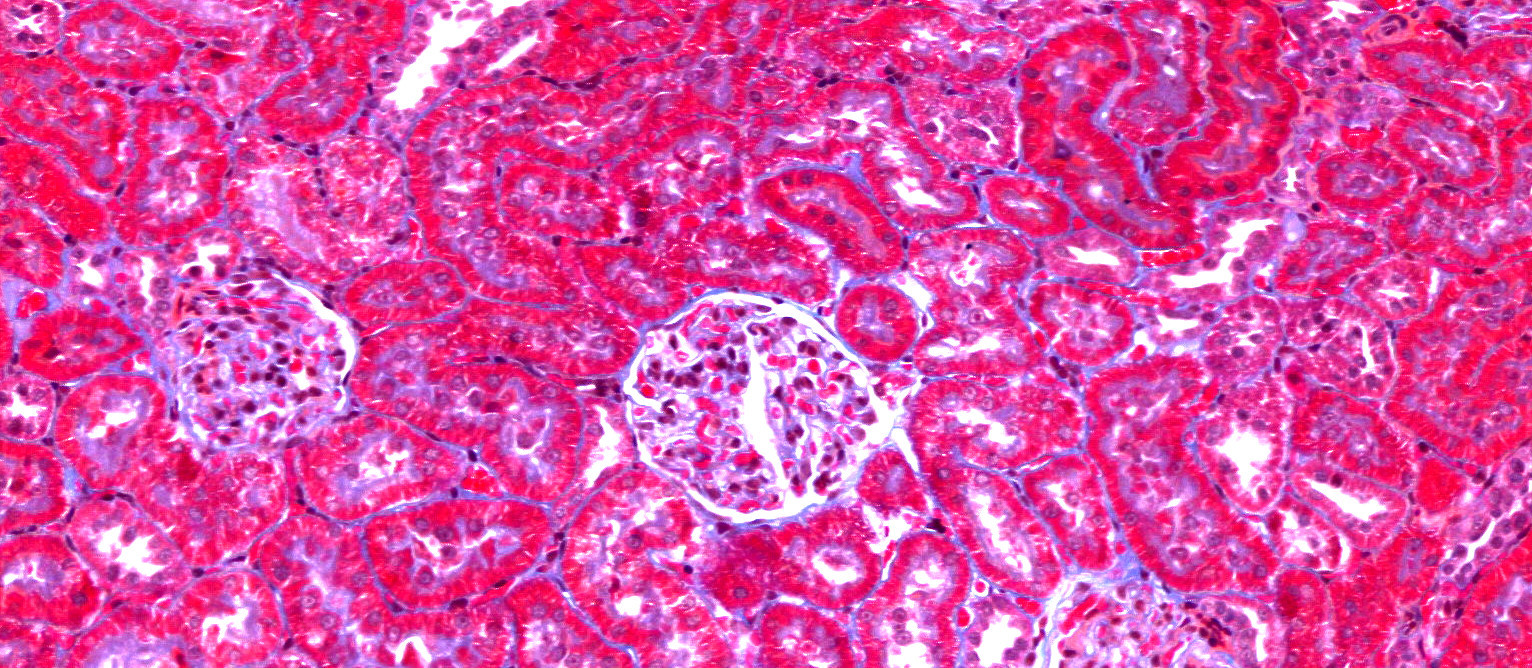

Supplement: Supplementary file 9 [file DataSheet10.ZIP › Fig 1D-masson-sham-9(2)/9-10.jpeg]

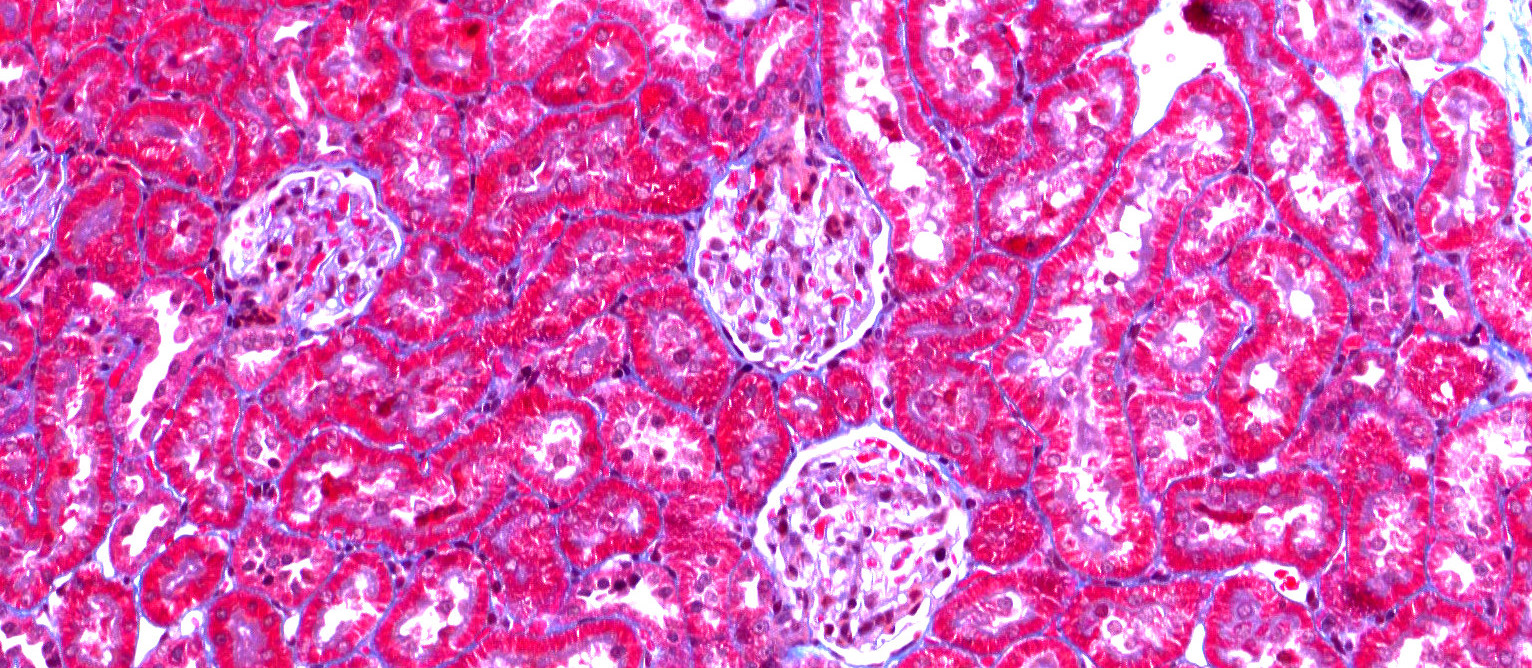

Supplement: Supplementary file 9 [file DataSheet10.ZIP › Fig 1D-masson-sham-9(2)/9-9.jpeg]

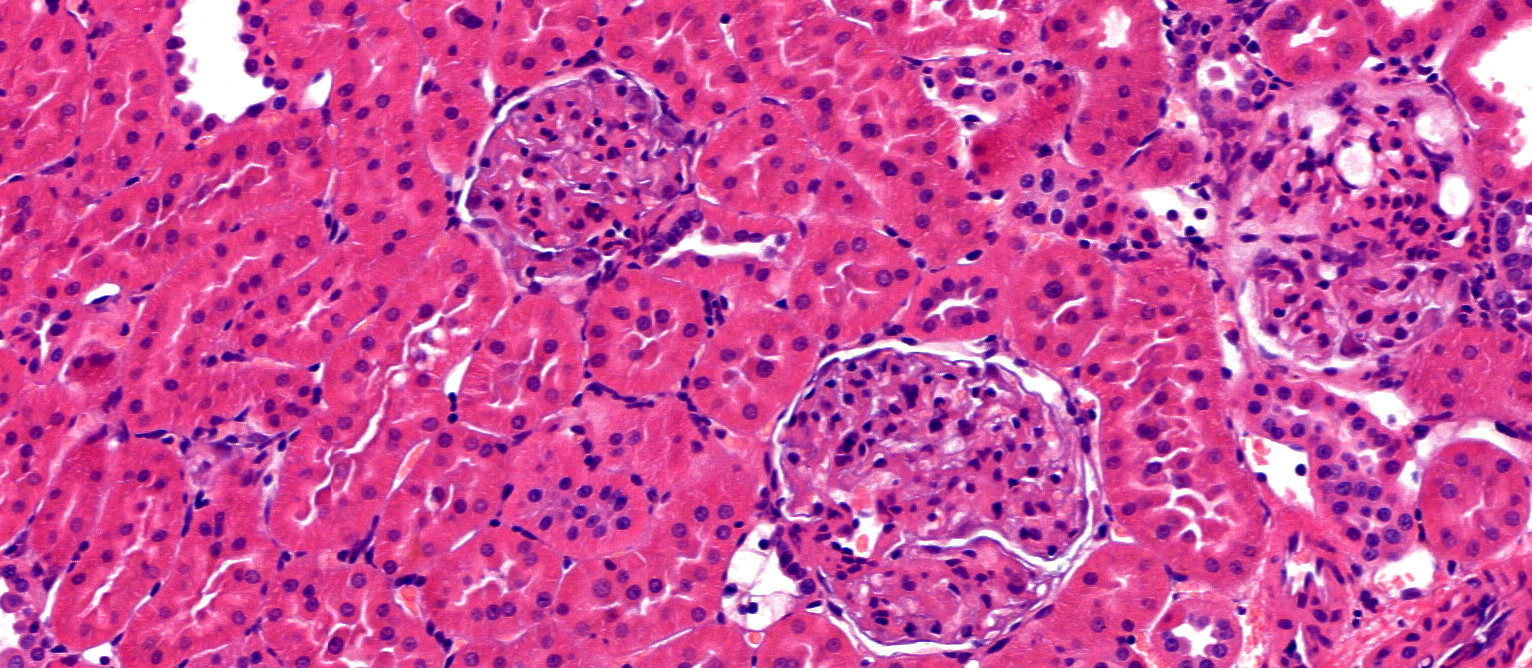

Supplement: Supplementary file 10 [file DataSheet6.ZIP › Fid 1D-HE-TSF-63(2)/63-10.jpeg]

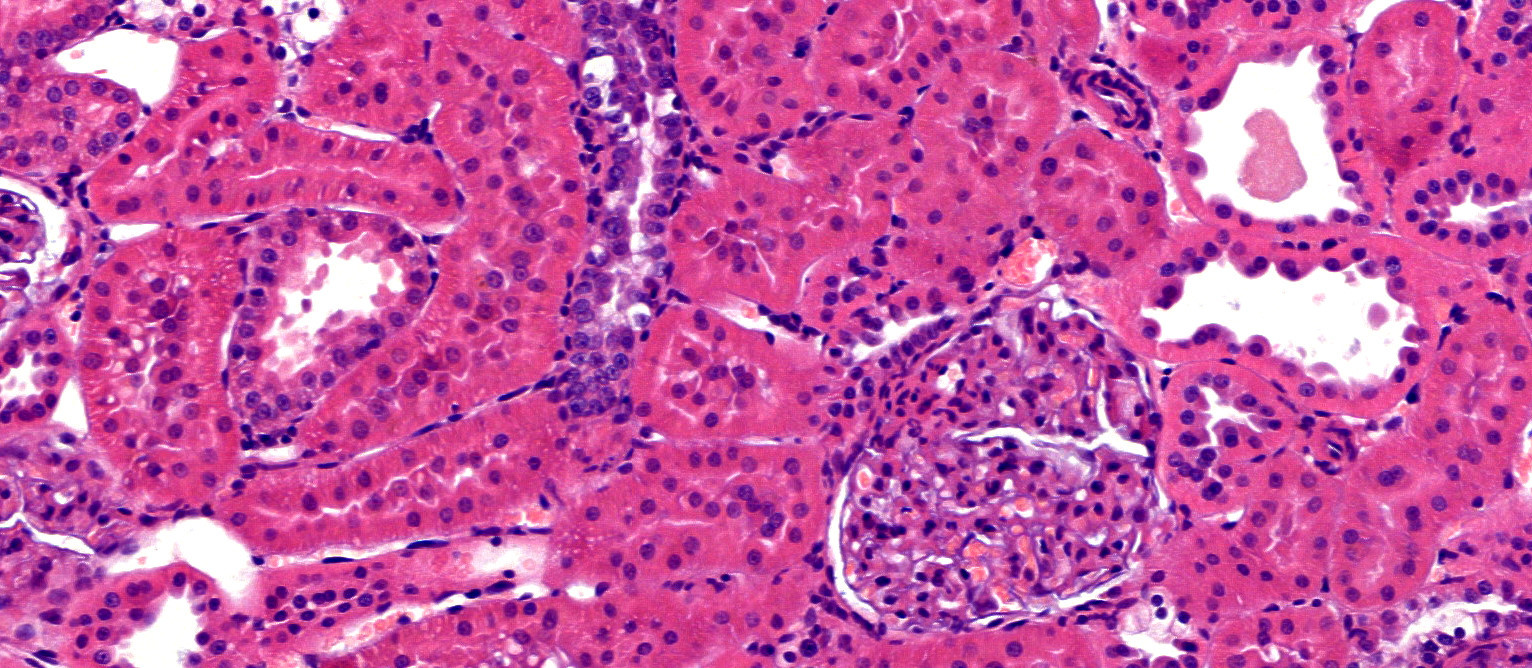

Supplement: Supplementary file 10 [file DataSheet6.ZIP › Fid 1D-HE-TSF-63(2)/63-9.jpeg]

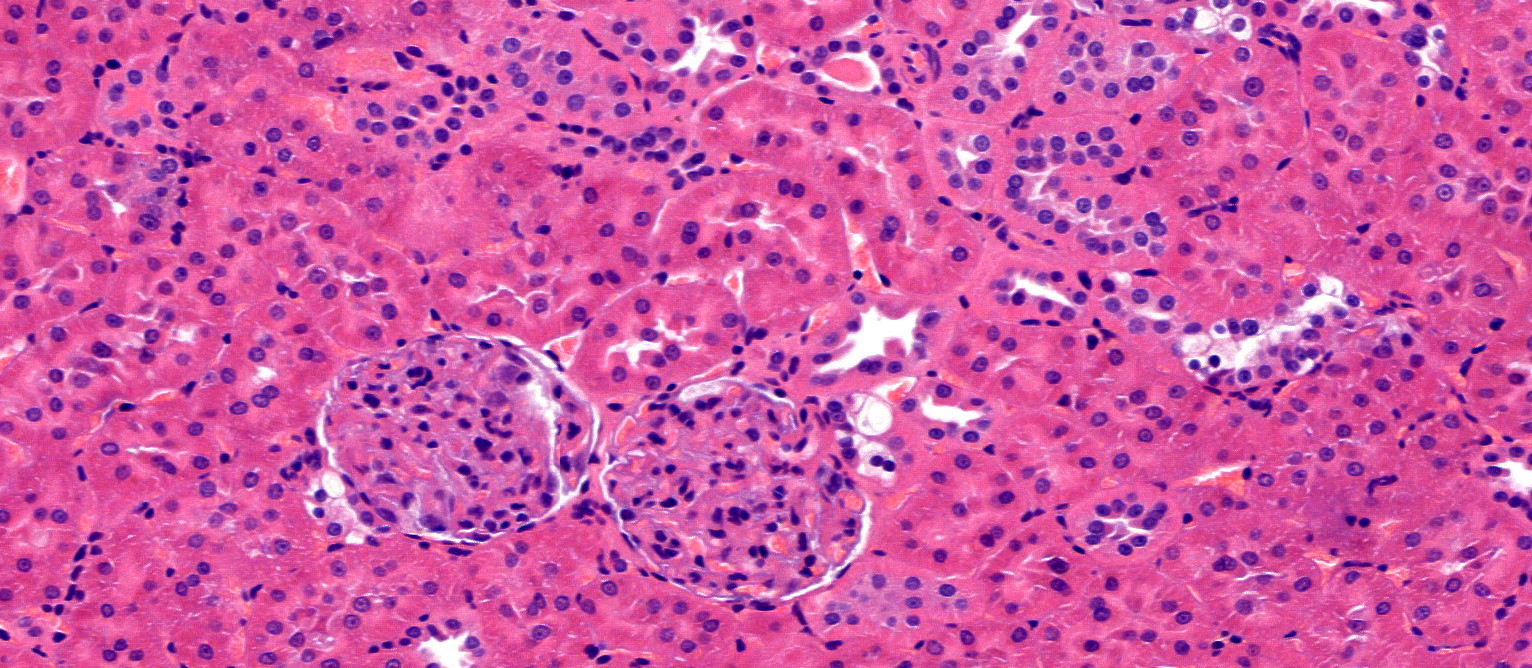

Supplement: Supplementary file 10 [file DataSheet6.ZIP › Fig 1D-HE-TSF-65/65-1.jpeg]

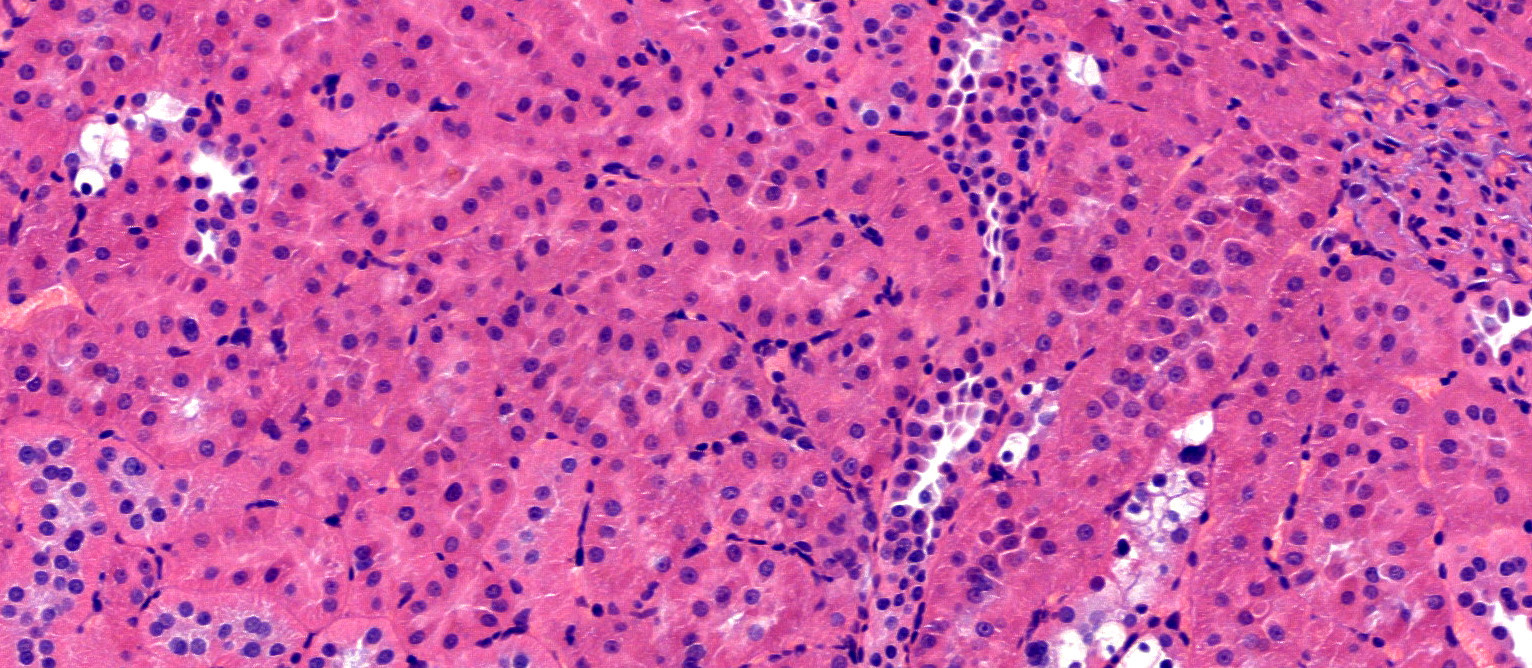

Supplement: Supplementary file 10 [file DataSheet6.ZIP › Fig 1D-HE-TSF-65/65-10.jpeg]

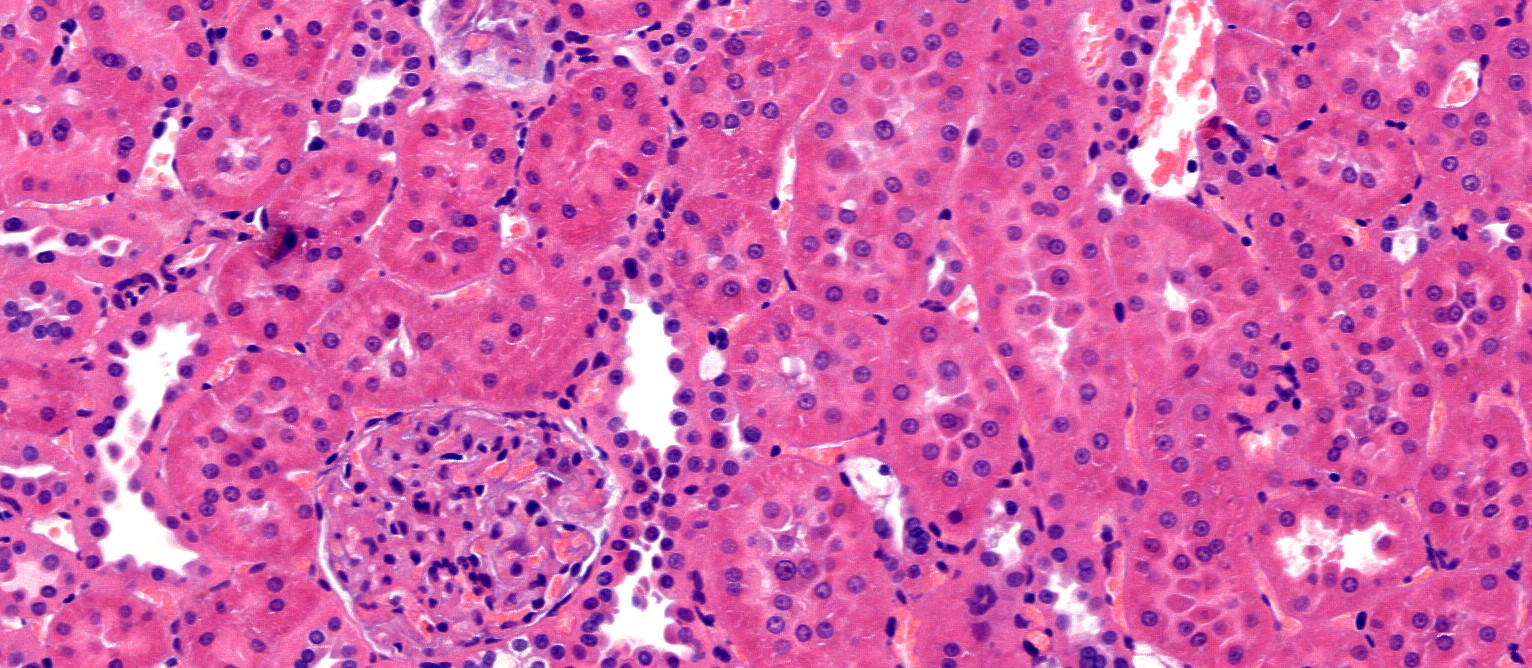

Supplement: Supplementary file 10 [file DataSheet6.ZIP › Fig 1D-HE-TSF-65/65-2.jpeg]

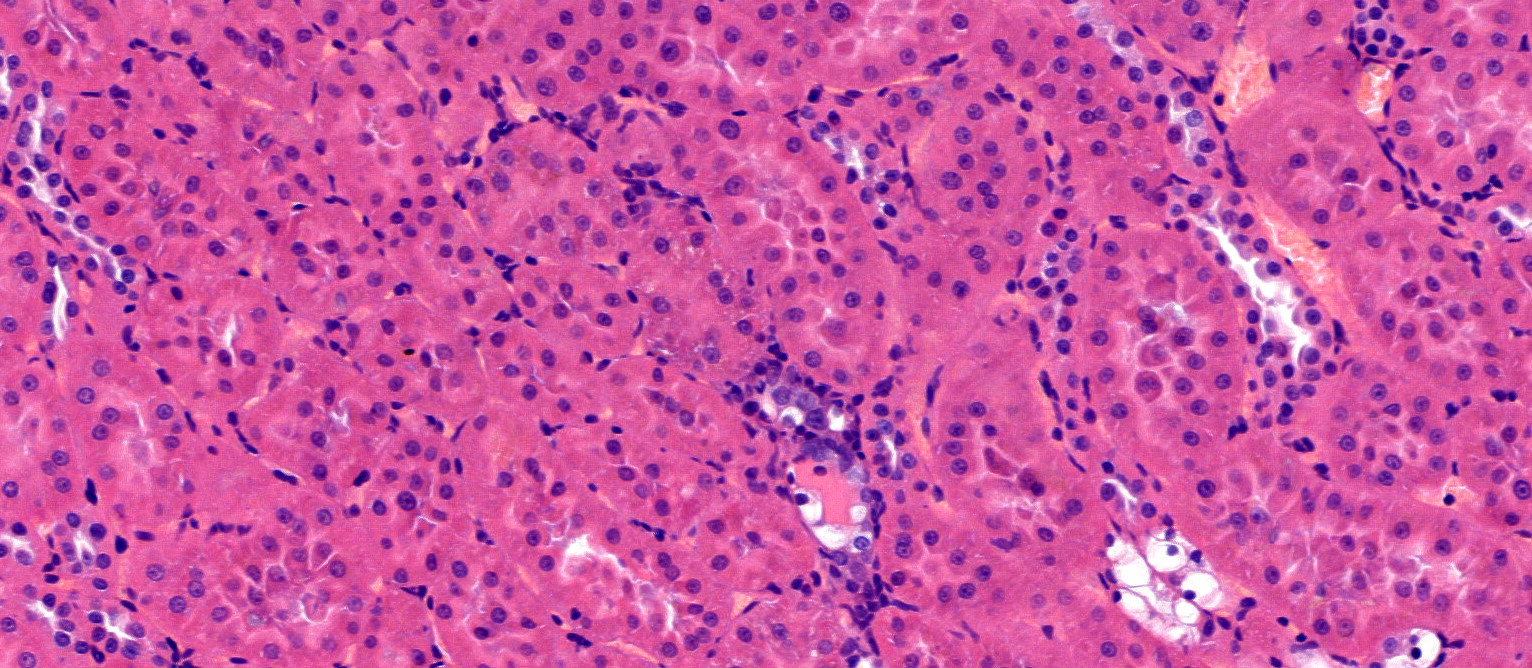

Supplement: Supplementary file 10 [file DataSheet6.ZIP › Fig 1D-HE-TSF-65/65-3.jpeg]

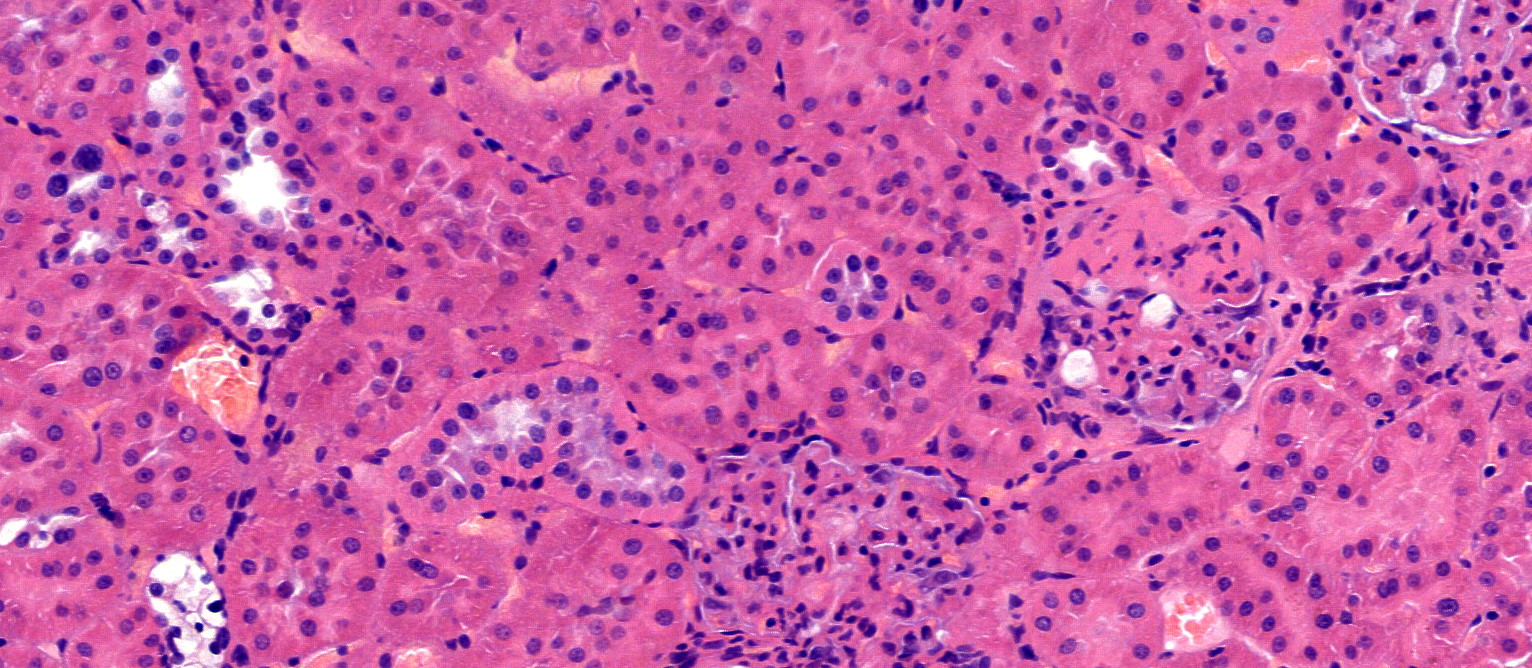

Supplement: Supplementary file 10 [file DataSheet6.ZIP › Fig 1D-HE-TSF-65/65-4.jpeg]

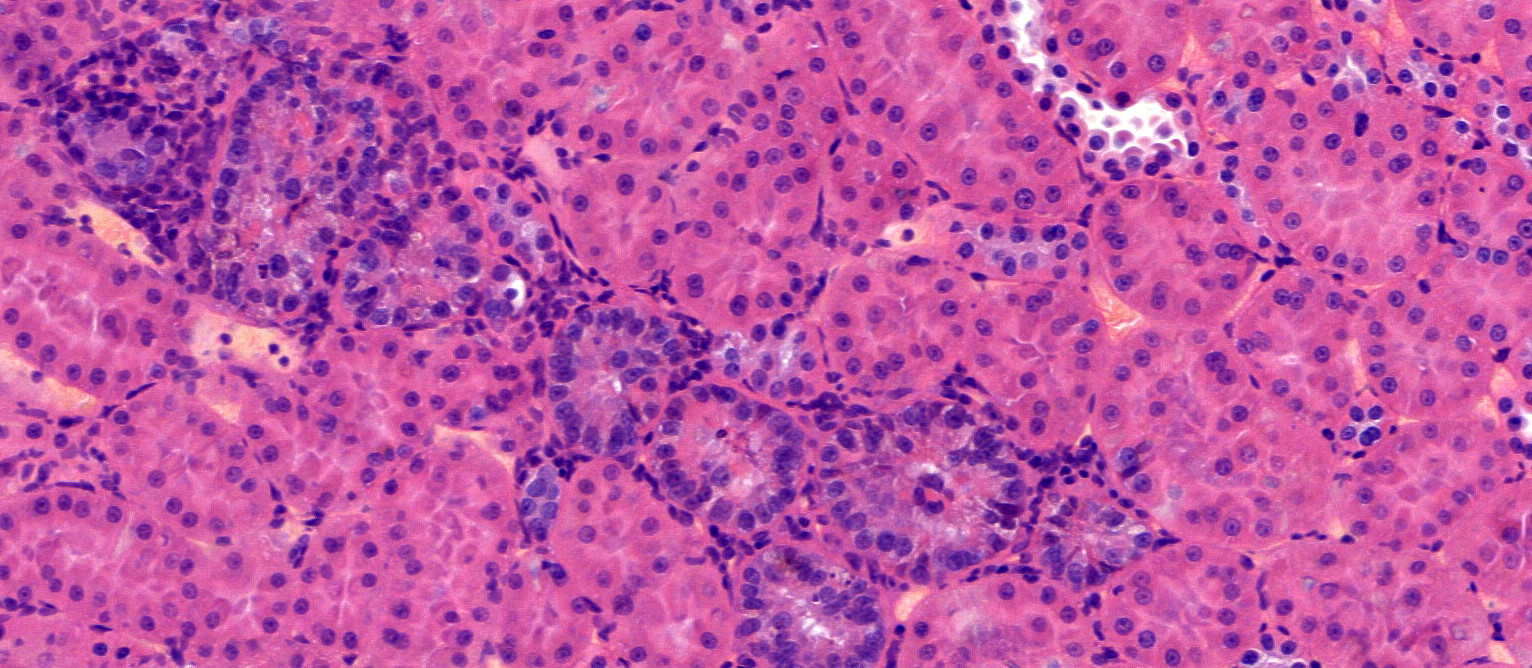

Supplement: Supplementary file 10 [file DataSheet6.ZIP › Fig 1D-HE-TSF-65/65-5.jpeg]

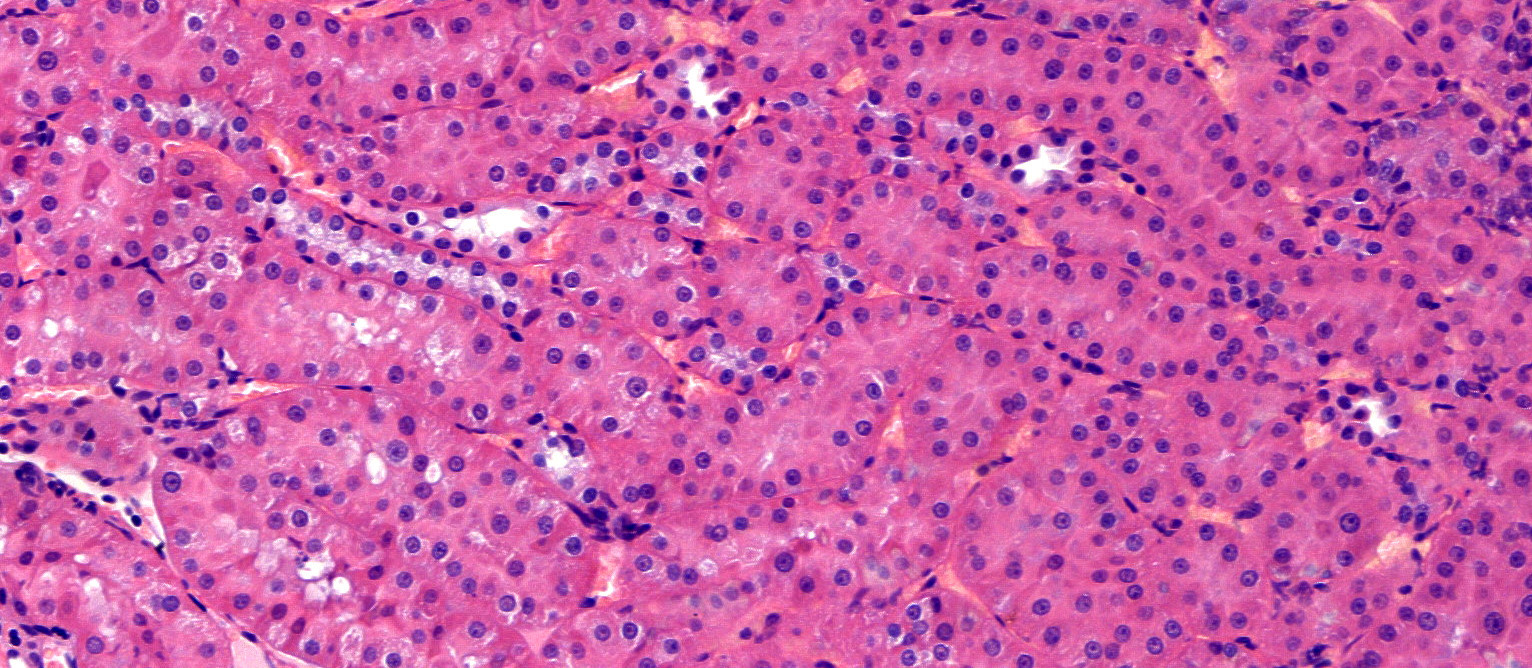

Supplement: Supplementary file 10 [file DataSheet6.ZIP › Fig 1D-HE-TSF-65/65-6.jpeg]

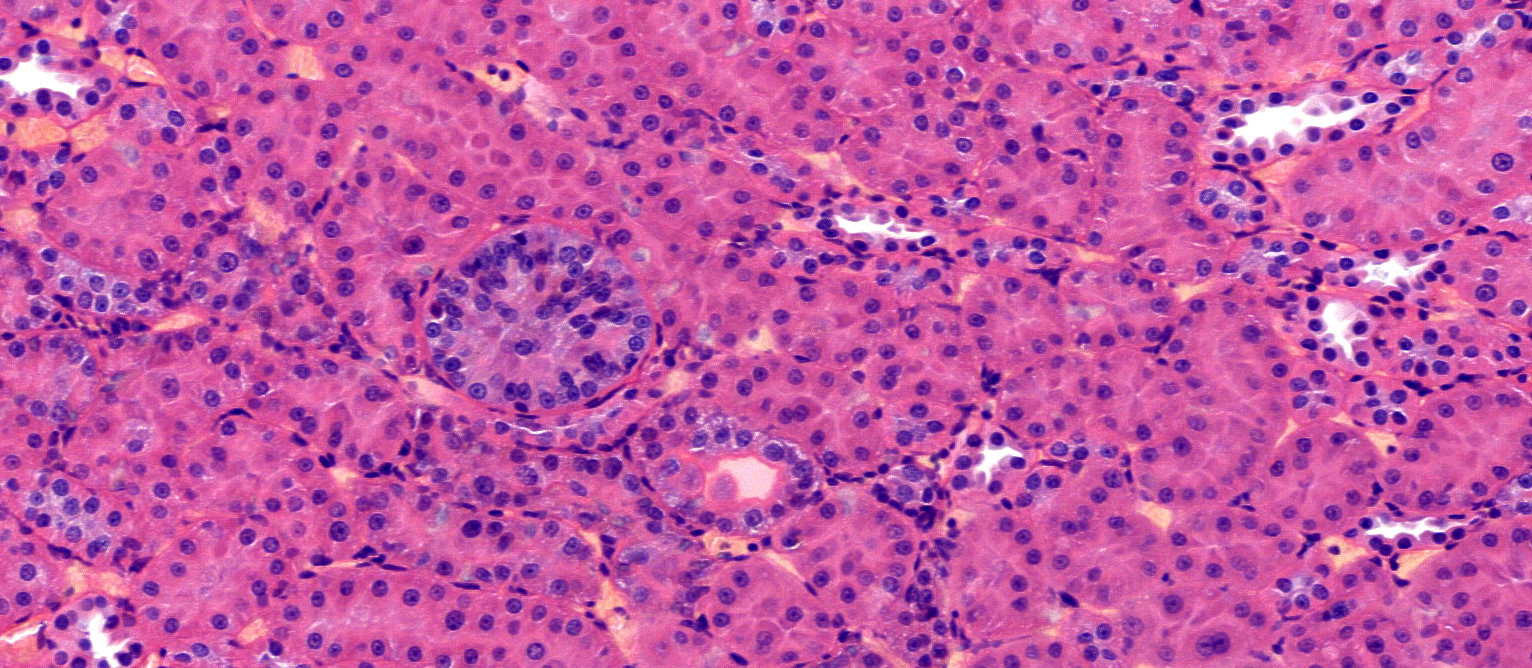

Supplement: Supplementary file 10 [file DataSheet6.ZIP › Fig 1D-HE-TSF-65/65-7.jpeg]

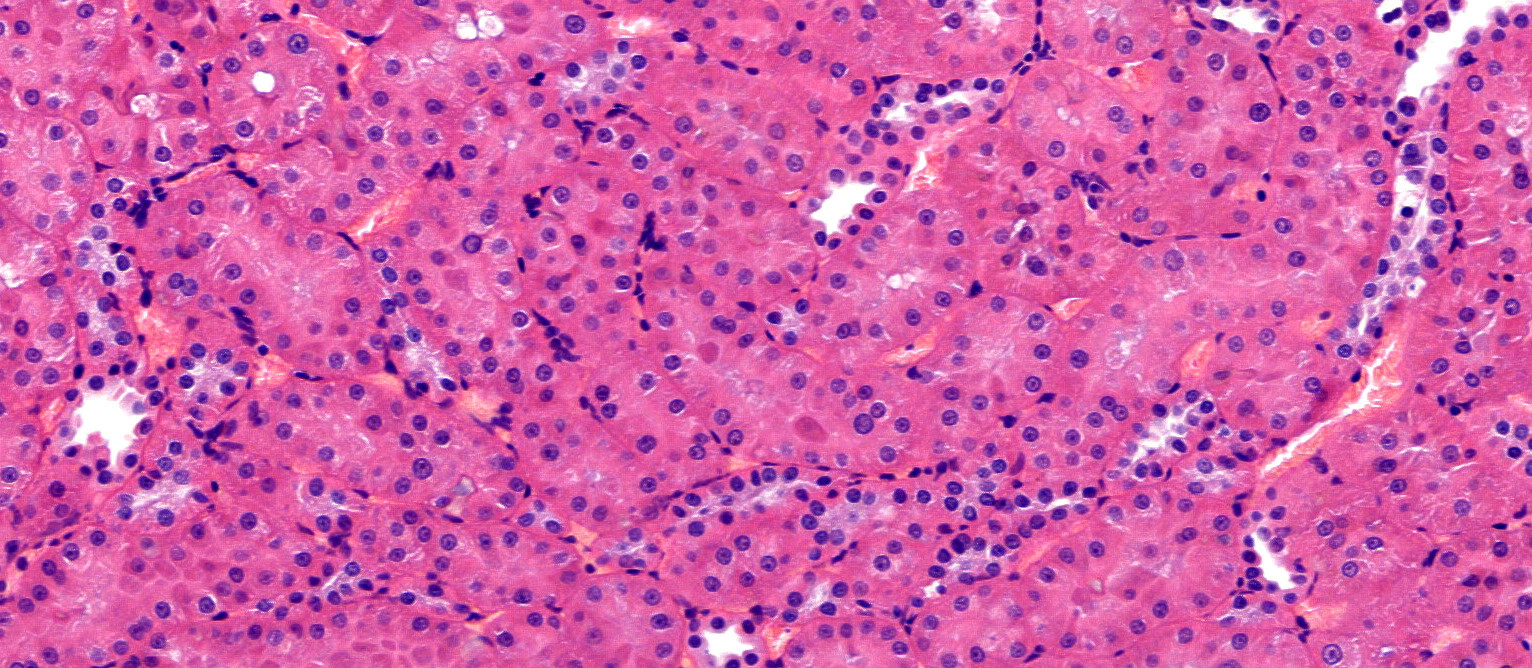

Supplement: Supplementary file 10 [file DataSheet6.ZIP › Fig 1D-HE-TSF-65/65-8.jpeg]

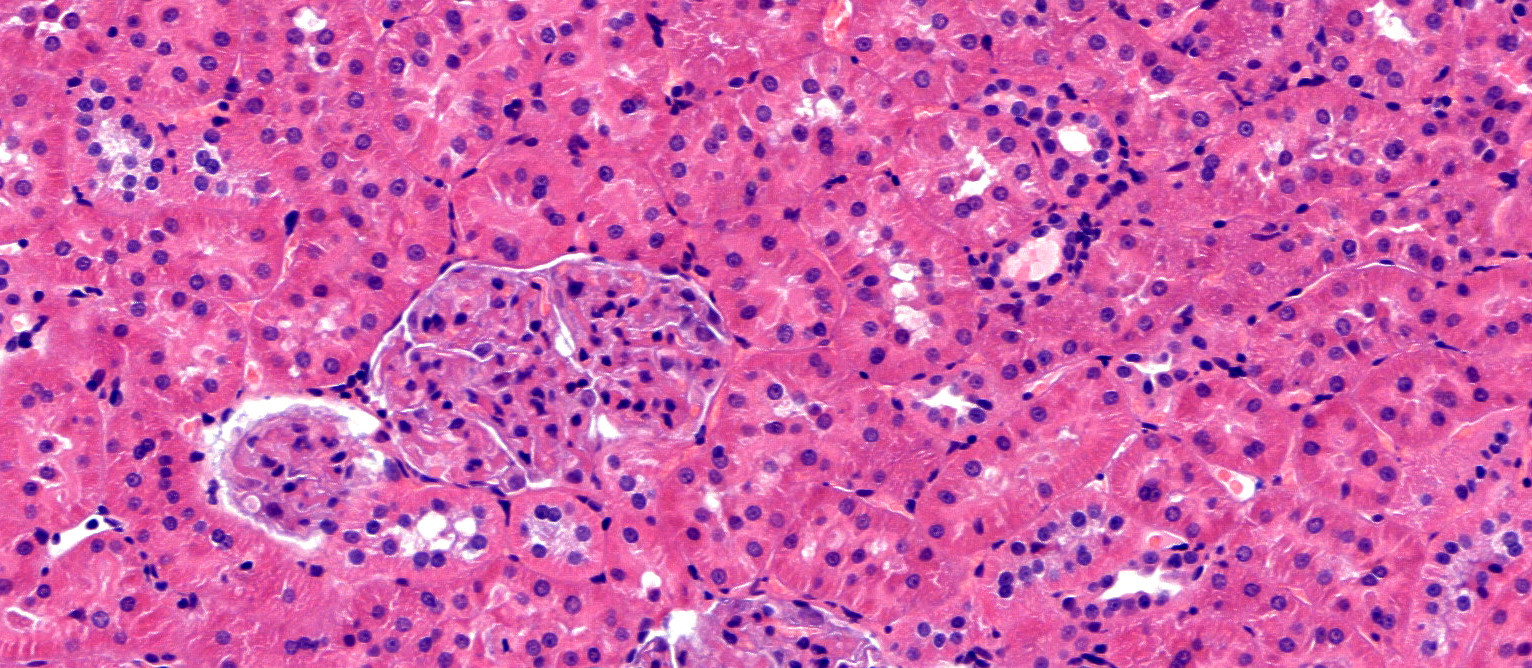

Supplement: Supplementary file 10 [file DataSheet6.ZIP › Fig 1D-HE-TSF-65/65-9.jpeg]

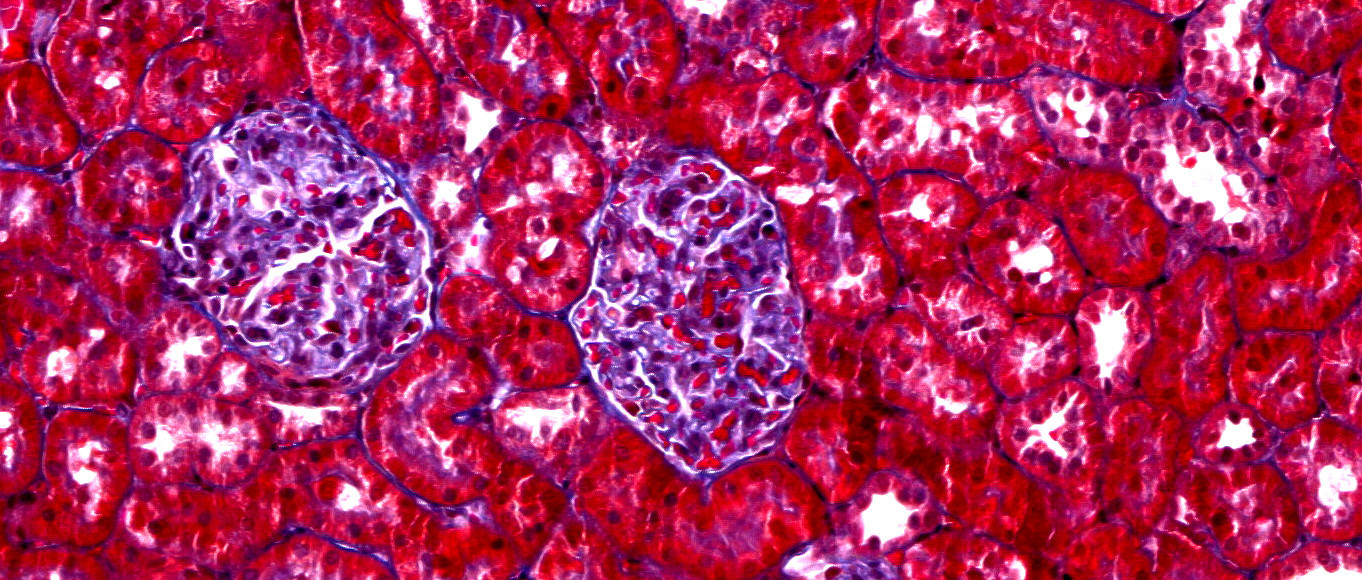

Supplement: Supplementary file 10 [file DataSheet6.ZIP › Fig 1D-masson-sham-1/1-1.jpeg]

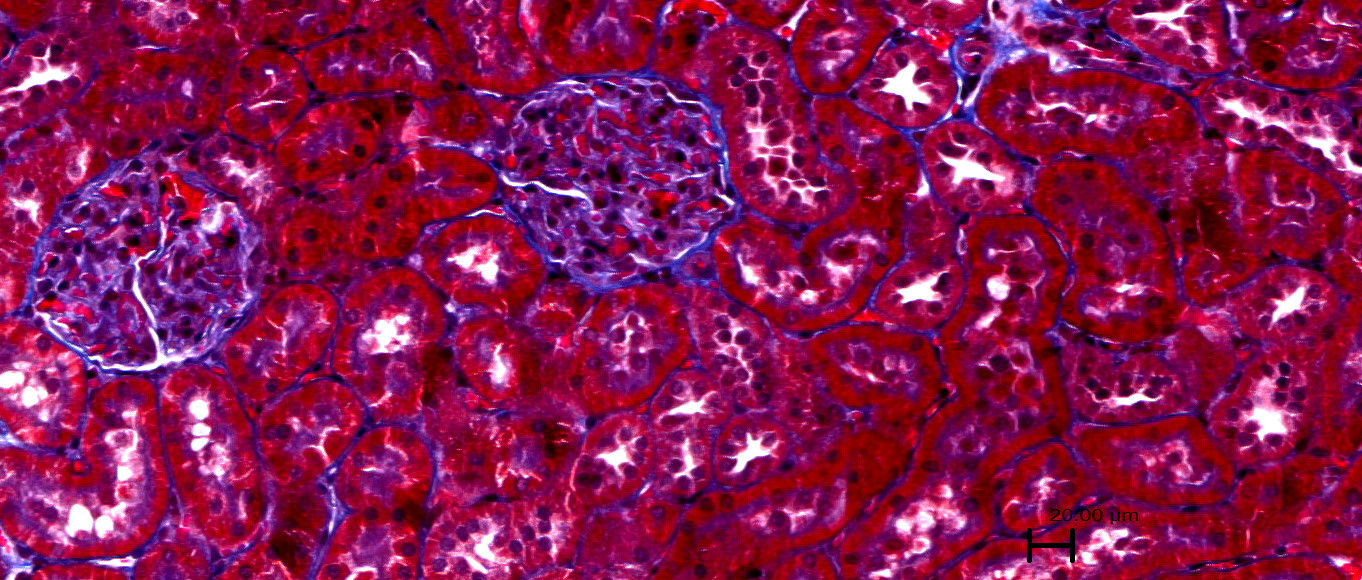

Supplement: Supplementary file 10 [file DataSheet6.ZIP › Fig 1D-masson-sham-1/1-10.jpeg]

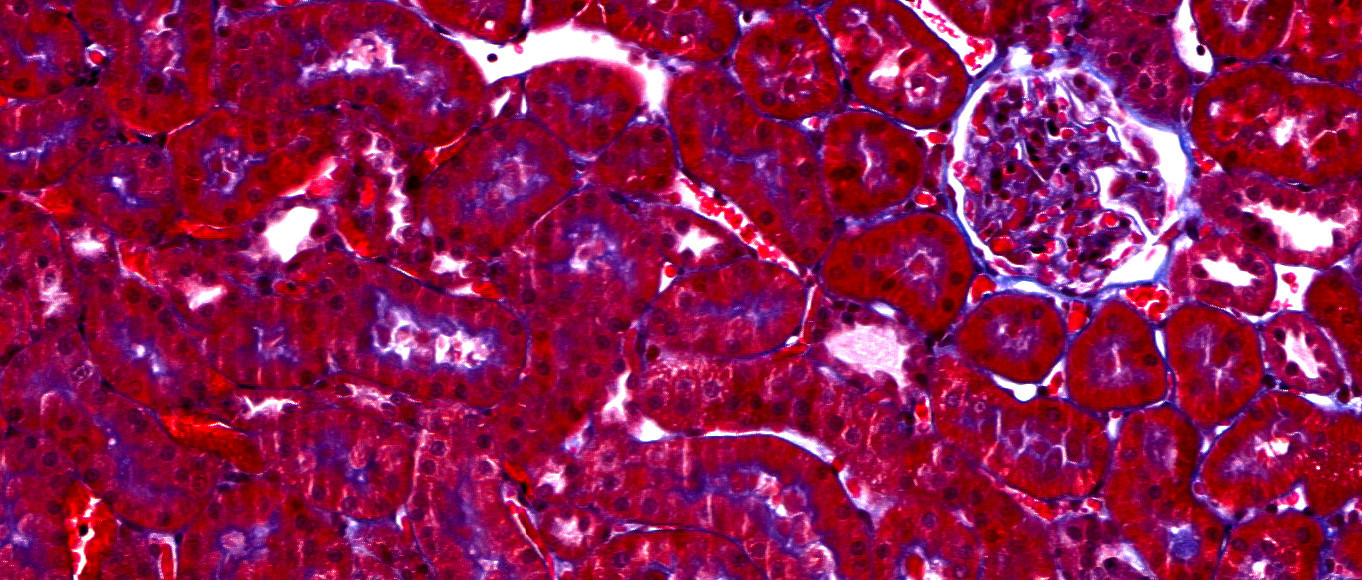

Supplement: Supplementary file 10 [file DataSheet6.ZIP › Fig 1D-masson-sham-1/1-2.jpeg]

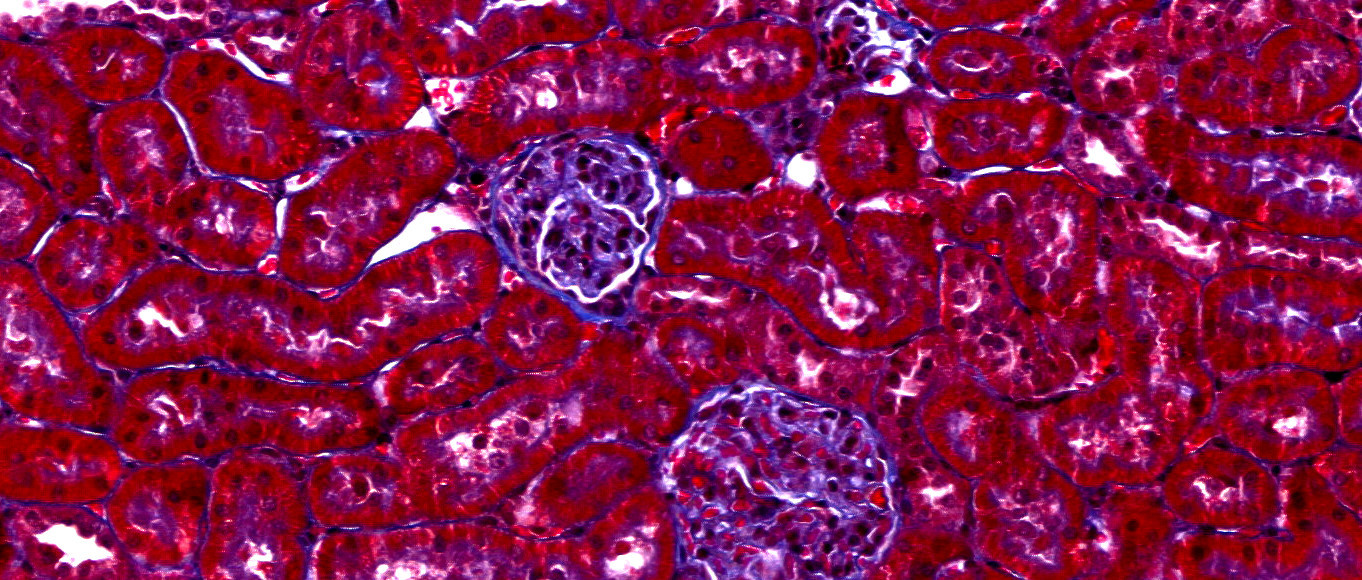

Supplement: Supplementary file 10 [file DataSheet6.ZIP › Fig 1D-masson-sham-1/1-3.jpeg]

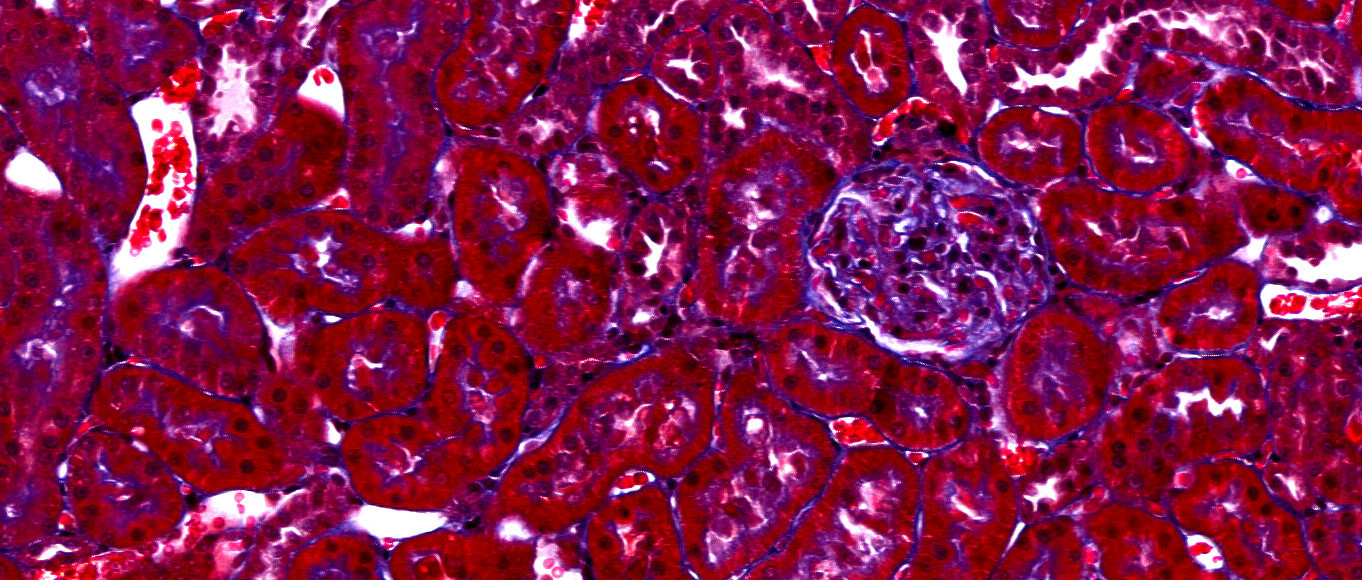

Supplement: Supplementary file 10 [file DataSheet6.ZIP › Fig 1D-masson-sham-1/1-4.jpeg]

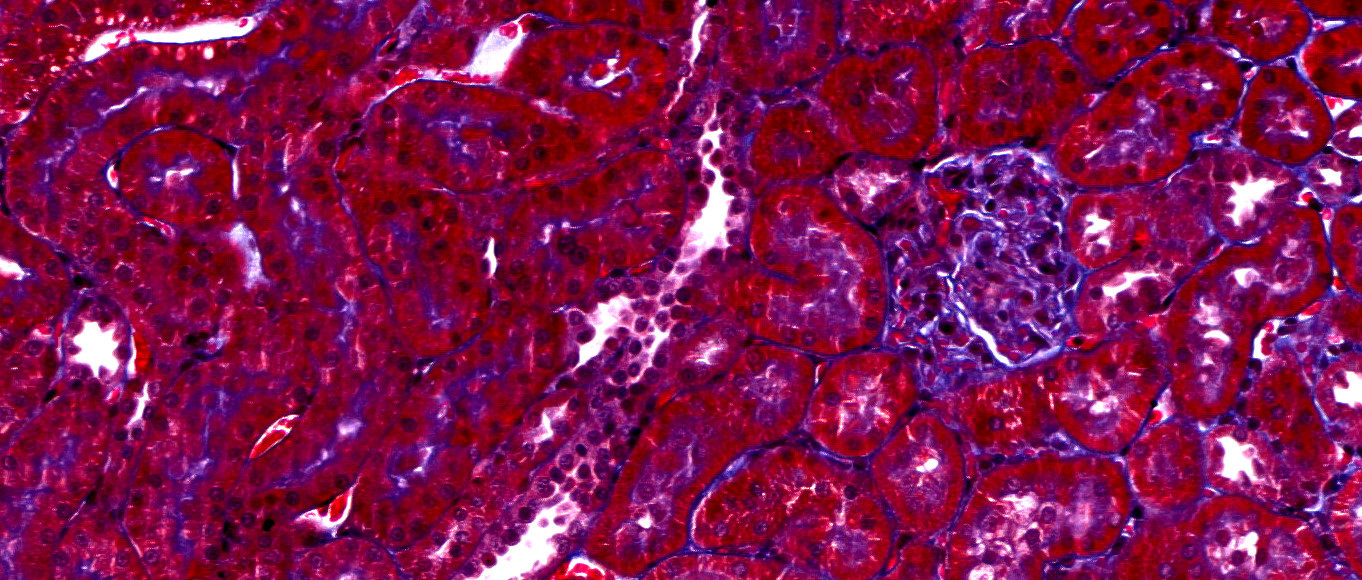

Supplement: Supplementary file 10 [file DataSheet6.ZIP › Fig 1D-masson-sham-1/1-5.jpeg]

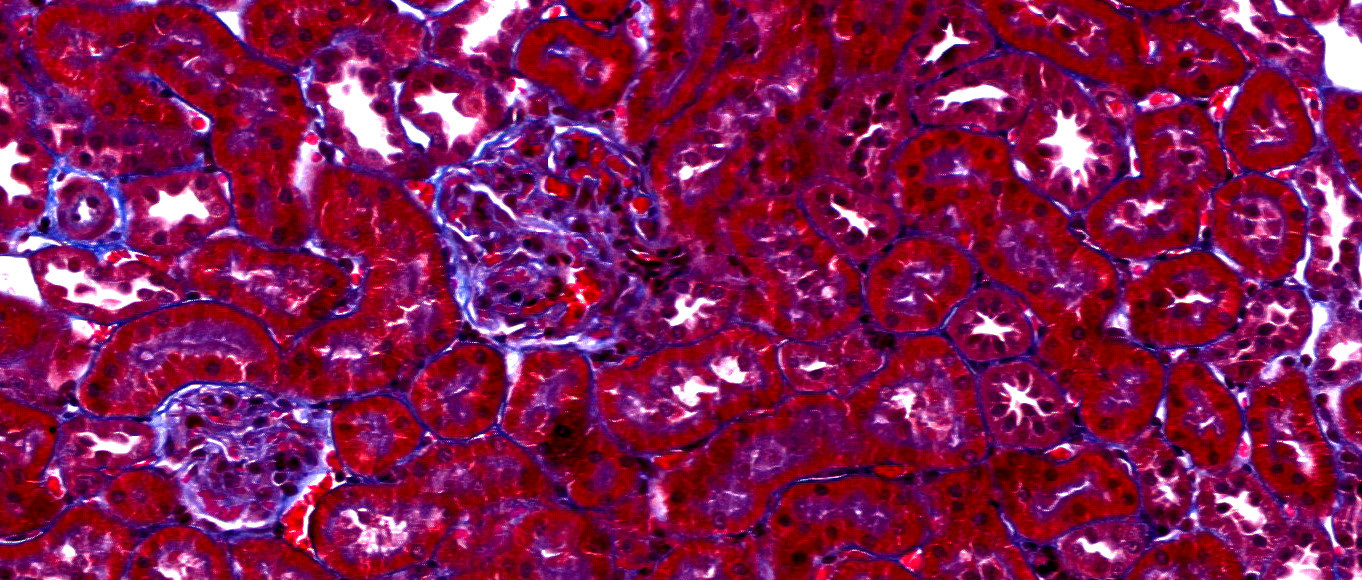

Supplement: Supplementary file 10 [file DataSheet6.ZIP › Fig 1D-masson-sham-1/1-6.jpeg]

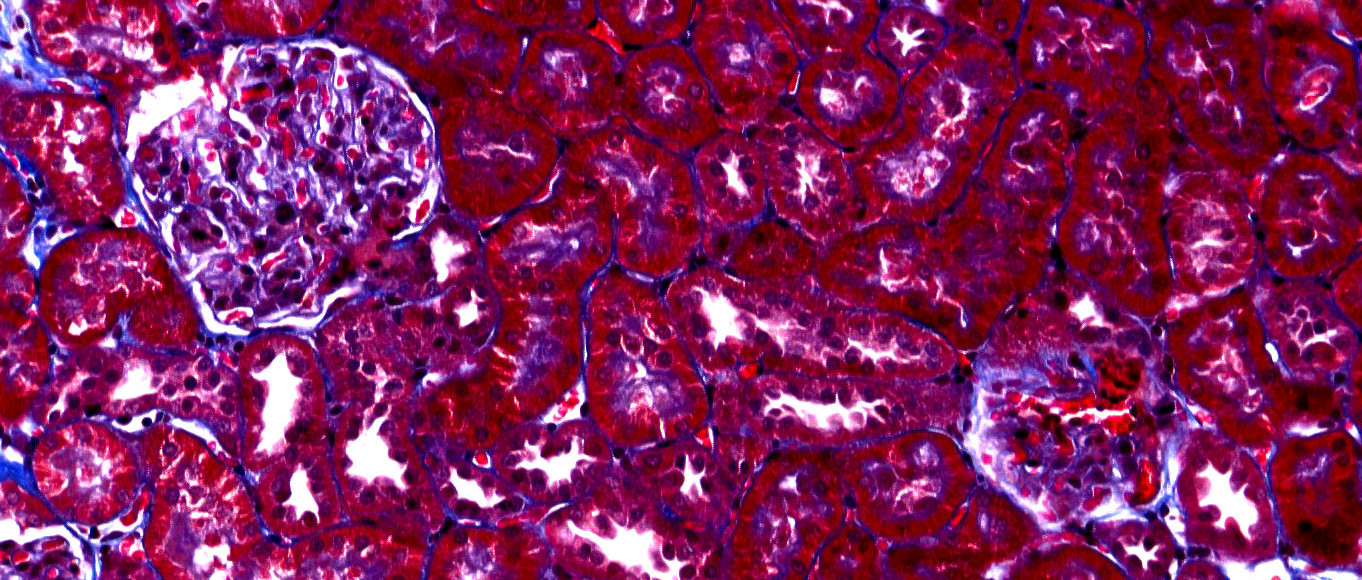

Supplement: Supplementary file 10 [file DataSheet6.ZIP › Fig 1D-masson-sham-1/1-7.jpeg]

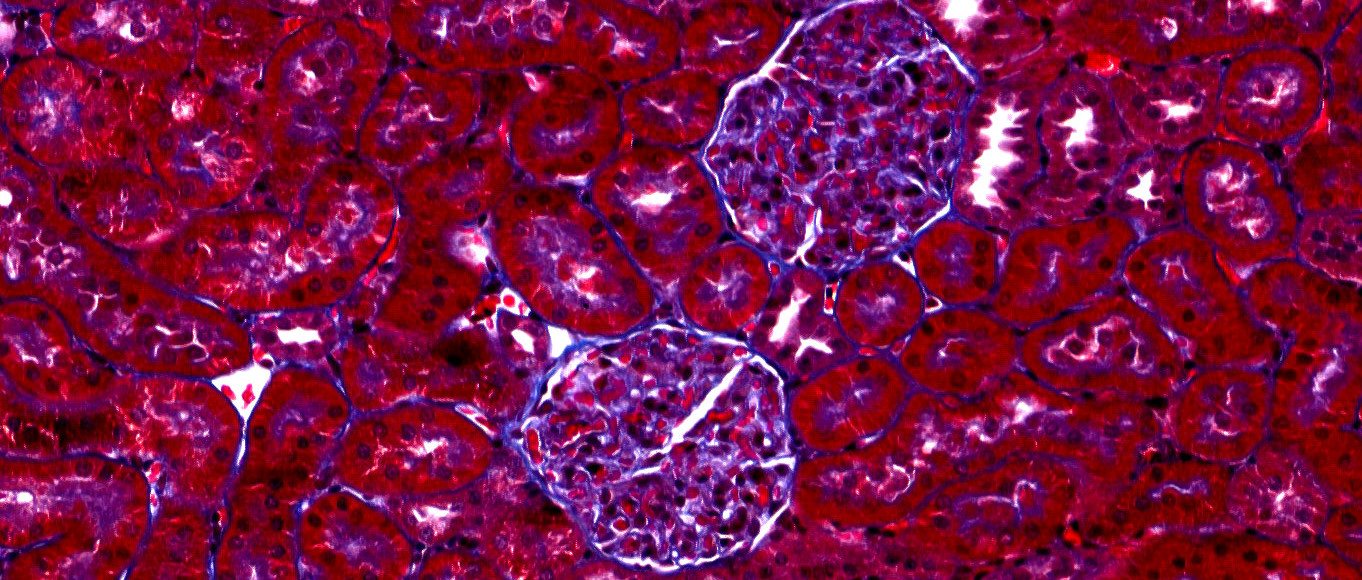

Supplement: Supplementary file 10 [file DataSheet6.ZIP › Fig 1D-masson-sham-1/1-8.jpeg]

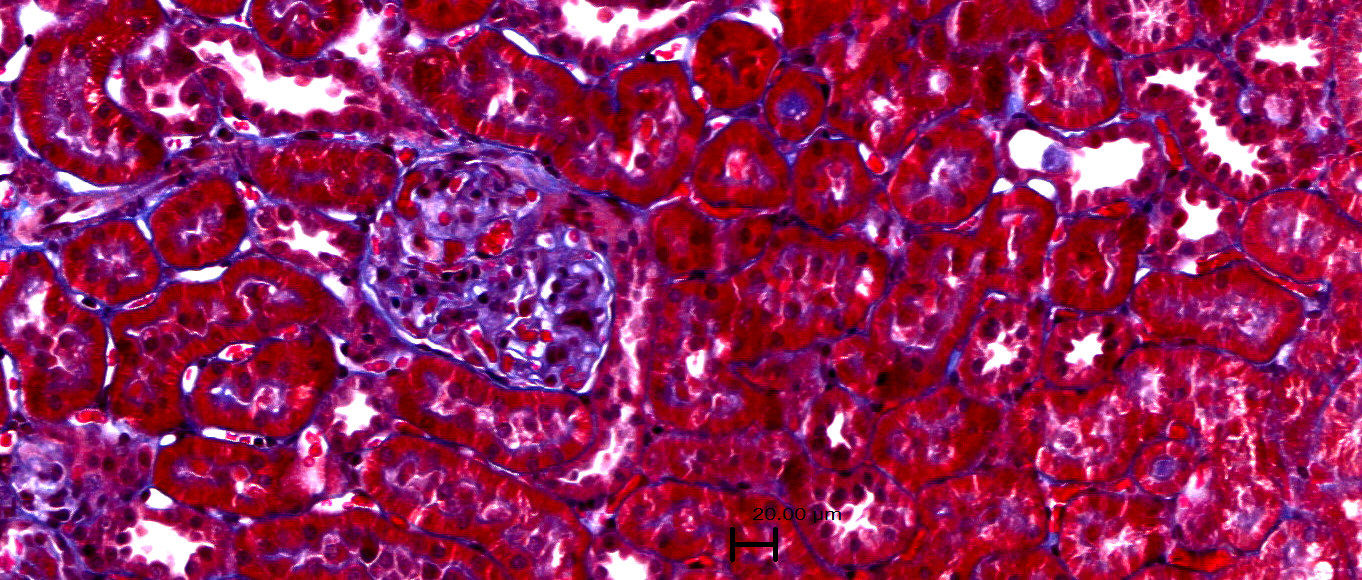

Supplement: Supplementary file 10 [file DataSheet6.ZIP › Fig 1D-masson-sham-1/1-9.jpeg]

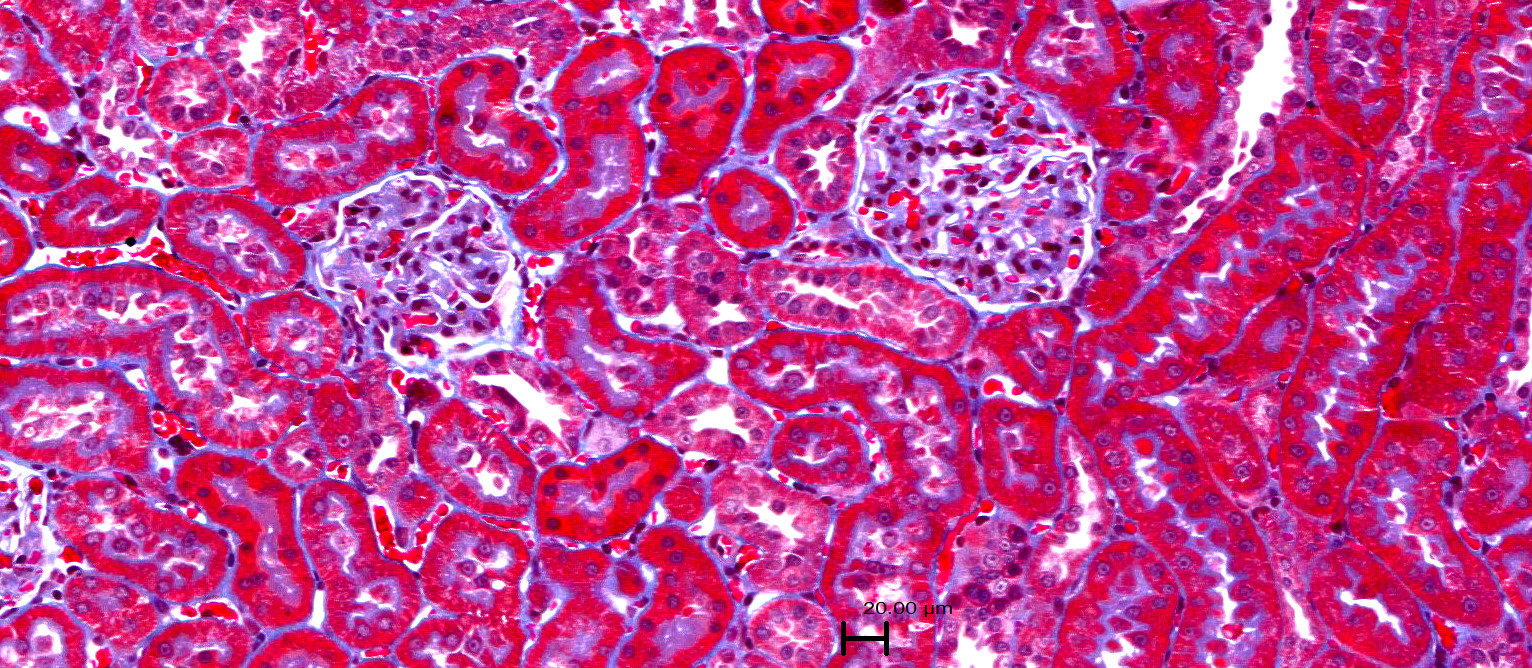

Supplement: Supplementary file 10 [file DataSheet6.ZIP › Fig 1D-masson-sham-2/2-1.jpeg]

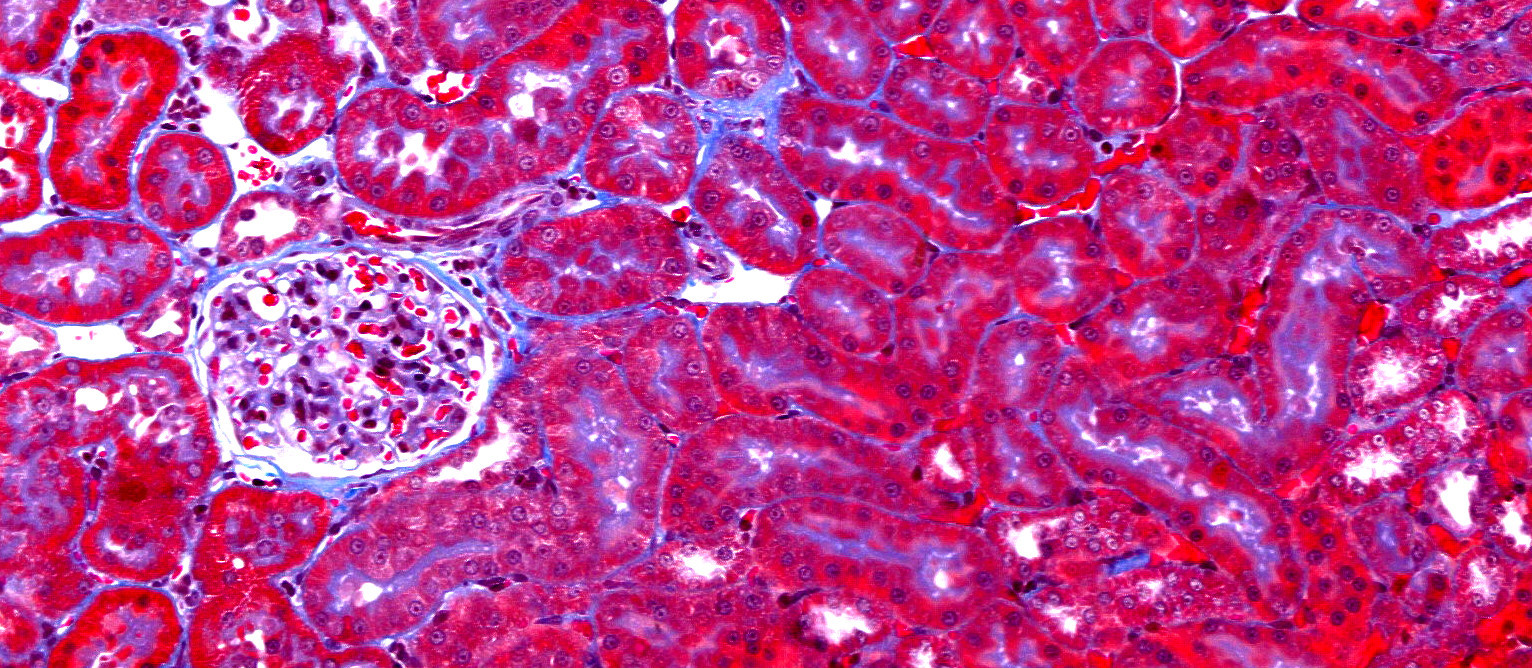

Supplement: Supplementary file 10 [file DataSheet6.ZIP › Fig 1D-masson-sham-2/2-10.jpeg]

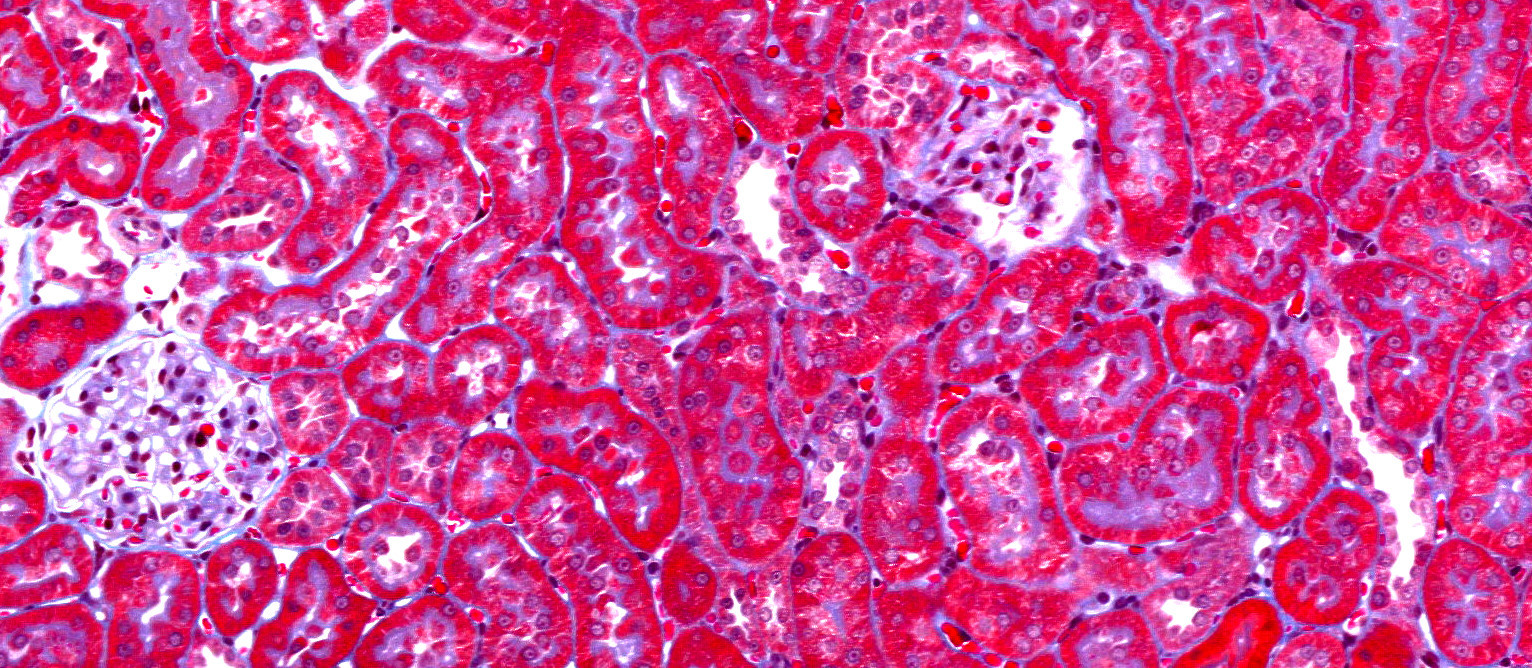

Supplement: Supplementary file 10 [file DataSheet6.ZIP › Fig 1D-masson-sham-2/2-2.jpeg]

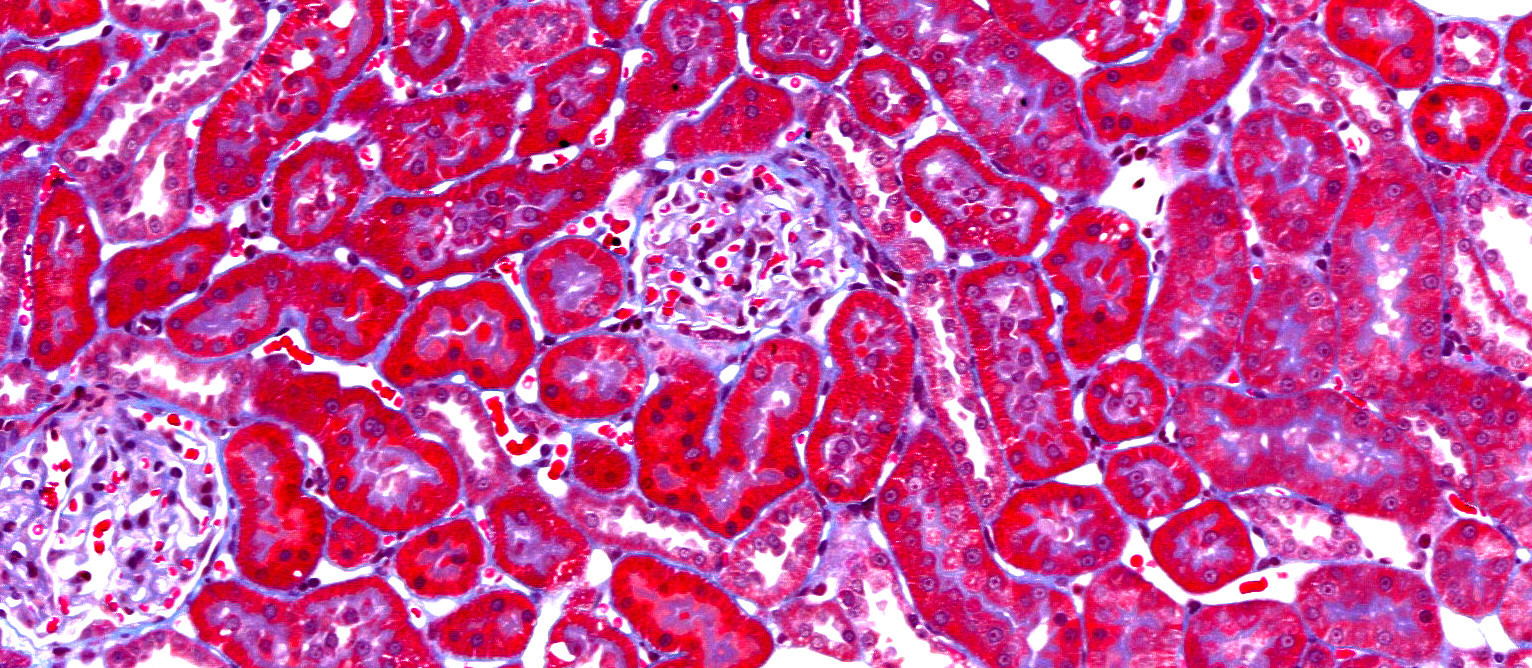

Supplement: Supplementary file 10 [file DataSheet6.ZIP › Fig 1D-masson-sham-2/2-3.jpeg]

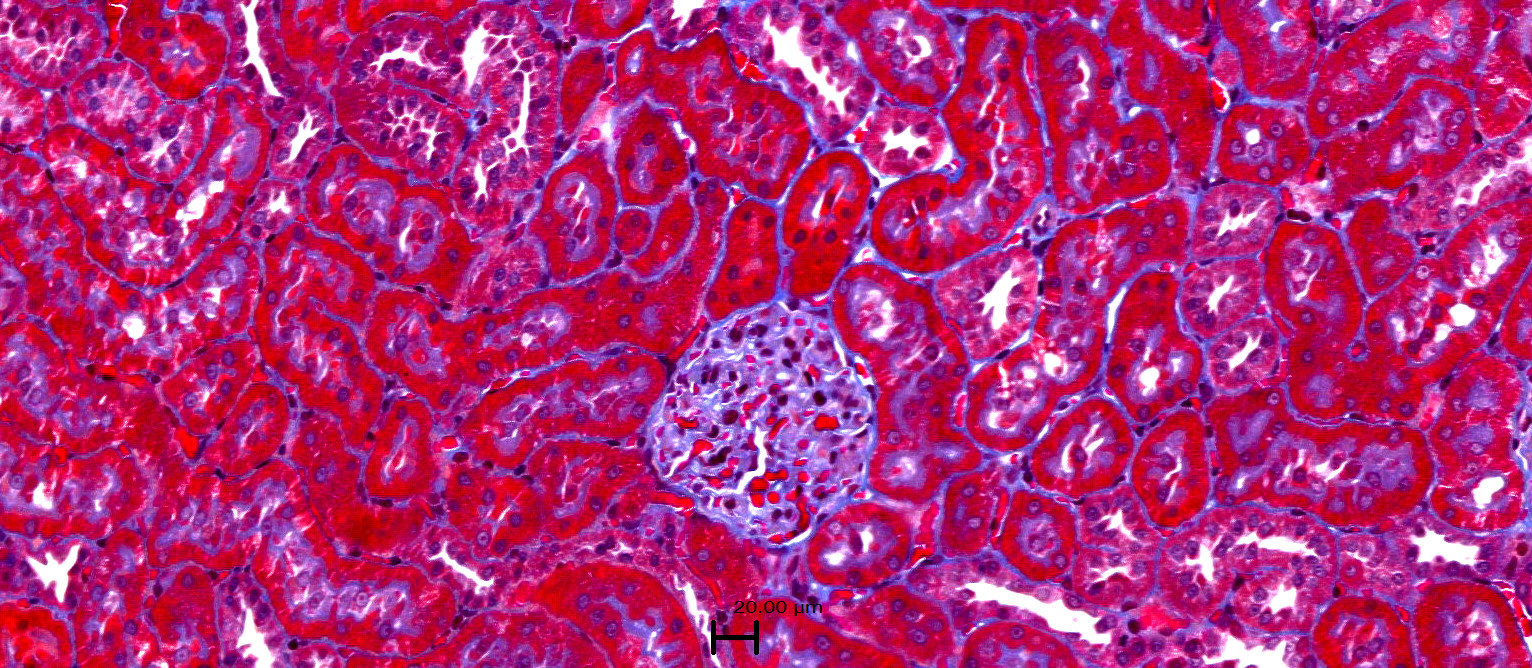

Supplement: Supplementary file 10 [file DataSheet6.ZIP › Fig 1D-masson-sham-2/2-4.jpeg]

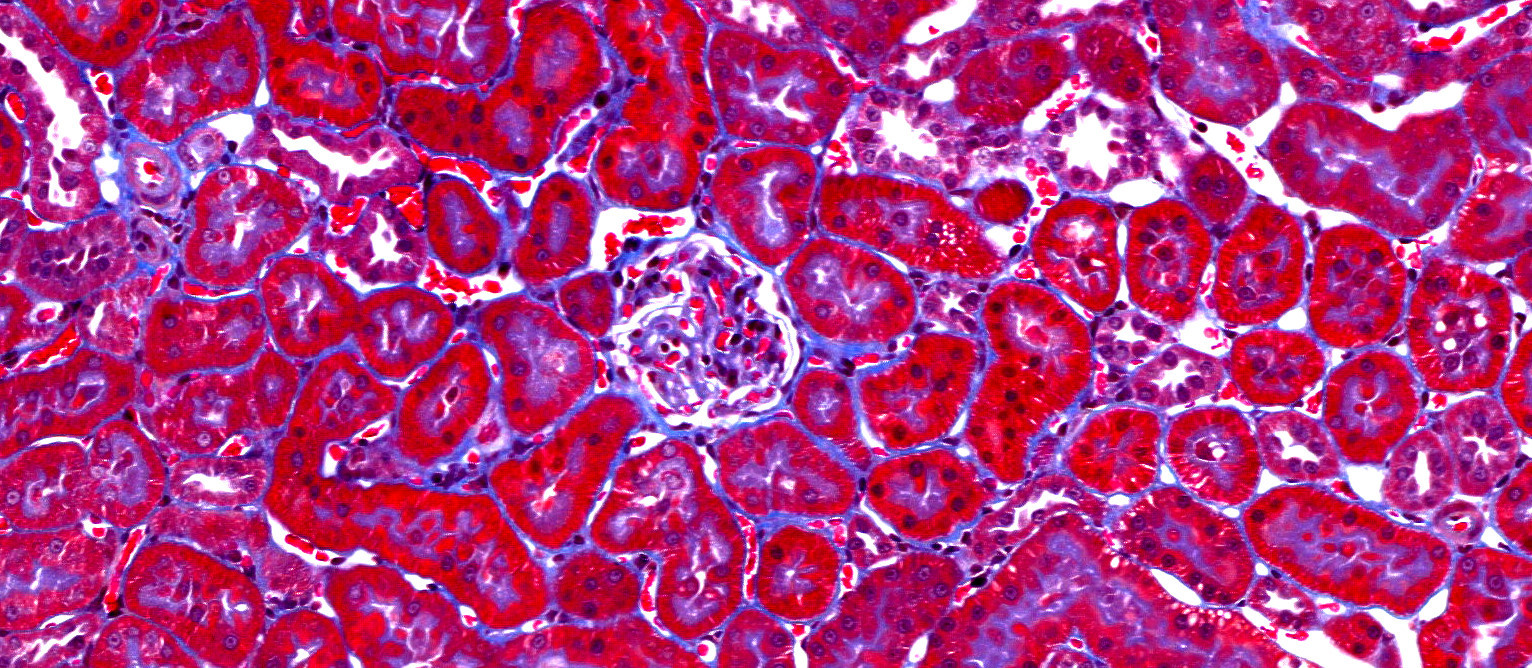

Supplement: Supplementary file 10 [file DataSheet6.ZIP › Fig 1D-masson-sham-2/2-5.jpeg]

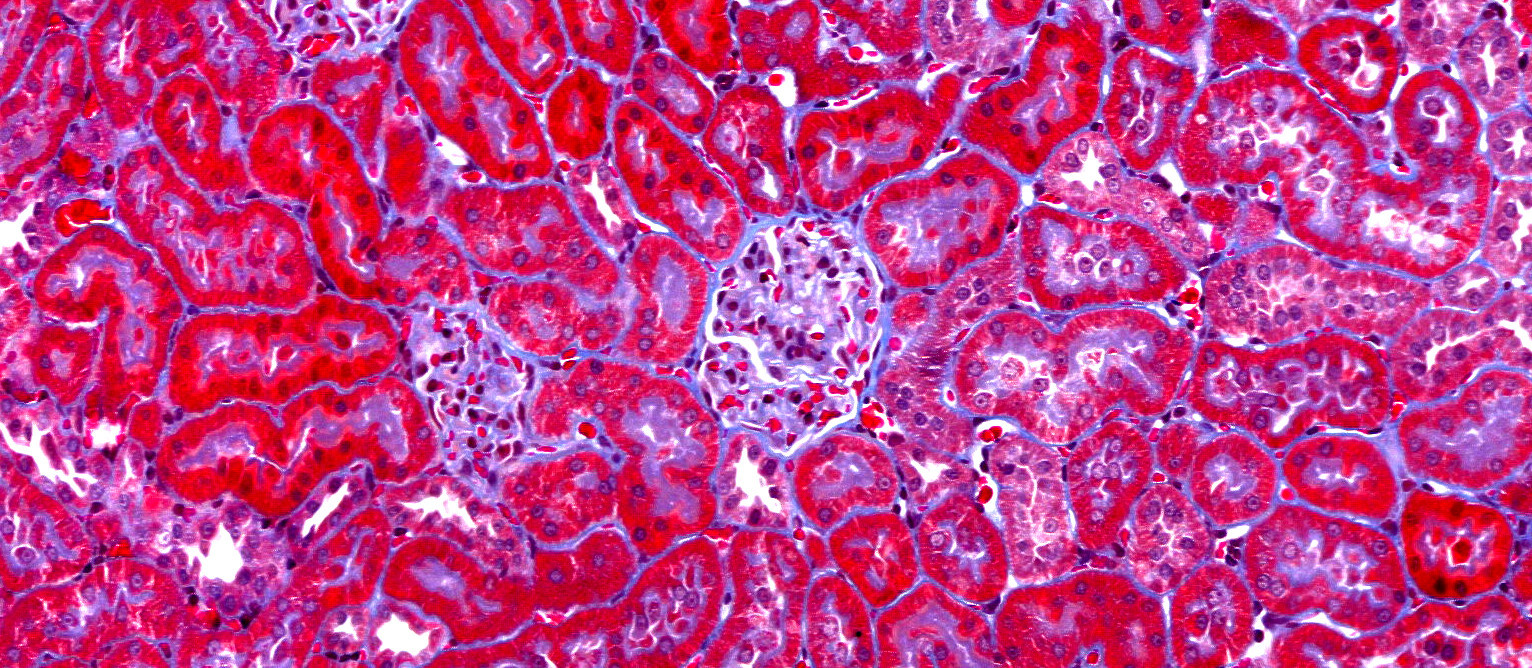

Supplement: Supplementary file 10 [file DataSheet6.ZIP › Fig 1D-masson-sham-2/2-6.jpeg]

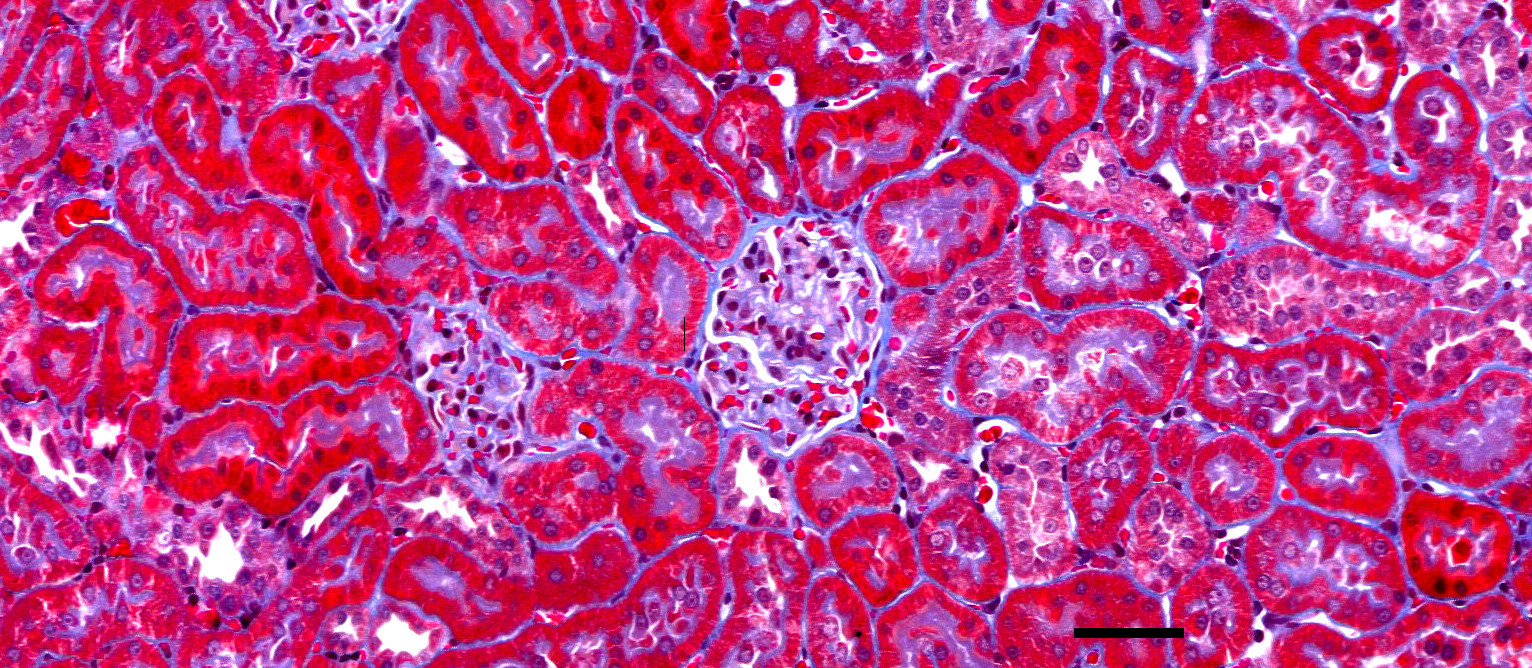

Supplement: Supplementary file 10 [file DataSheet6.ZIP › Fig 1D-masson-sham-2/2-6-1.png]

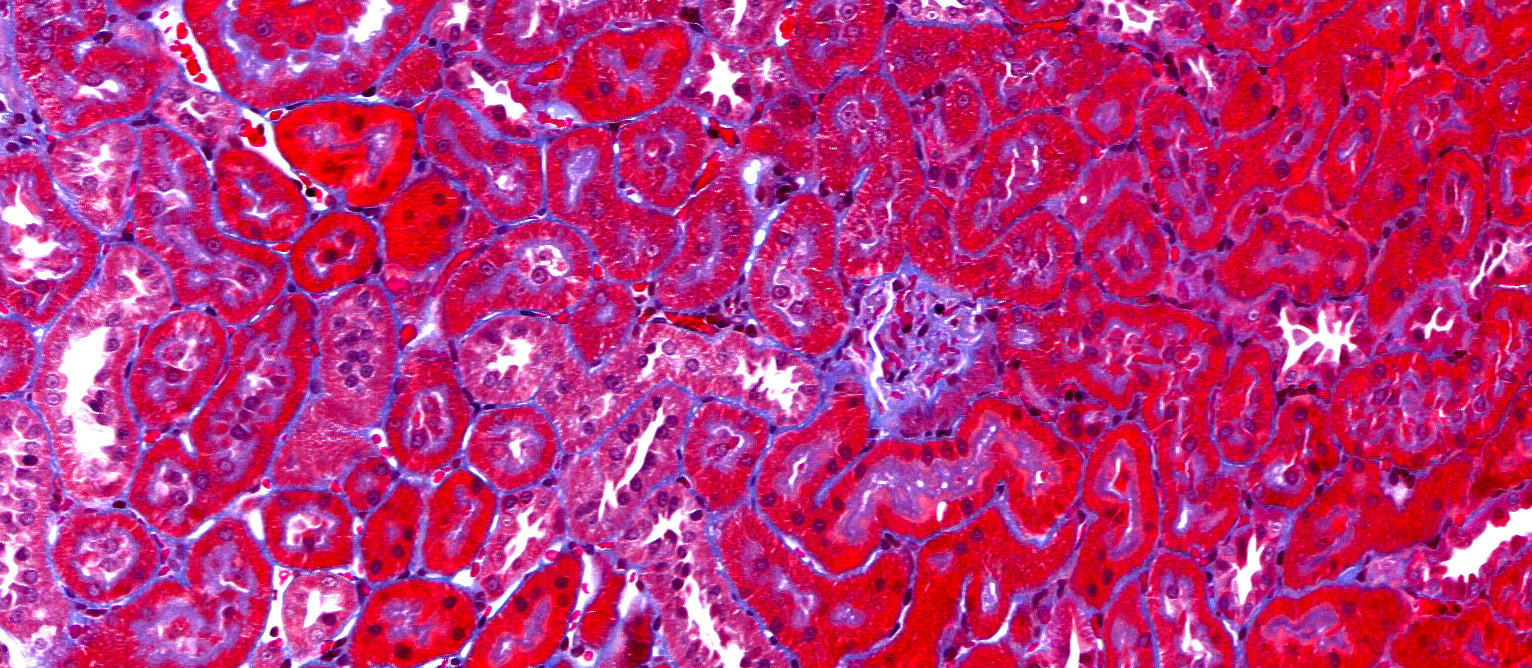

Supplement: Supplementary file 10 [file DataSheet6.ZIP › Fig 1D-masson-sham-2/2-7.jpeg]

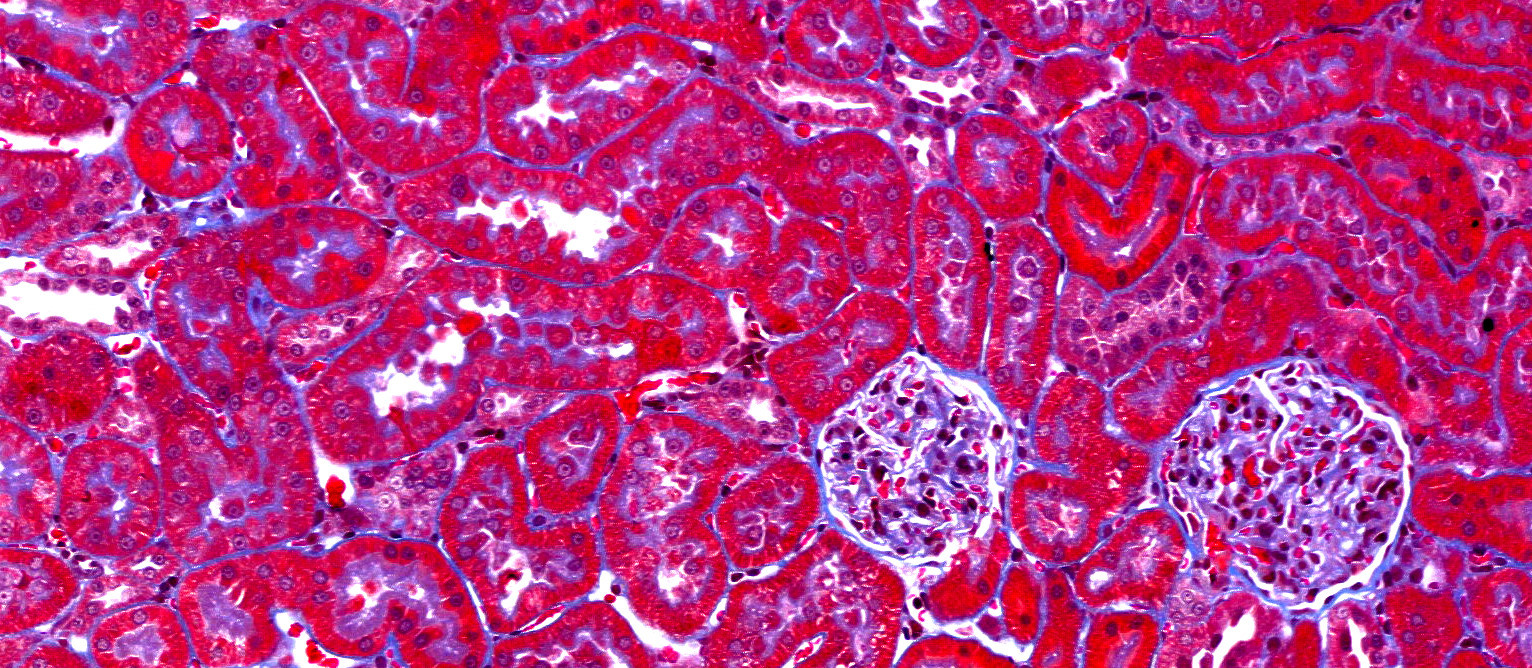

Supplement: Supplementary file 10 [file DataSheet6.ZIP › Fig 1D-masson-sham-2/2-8.jpeg]

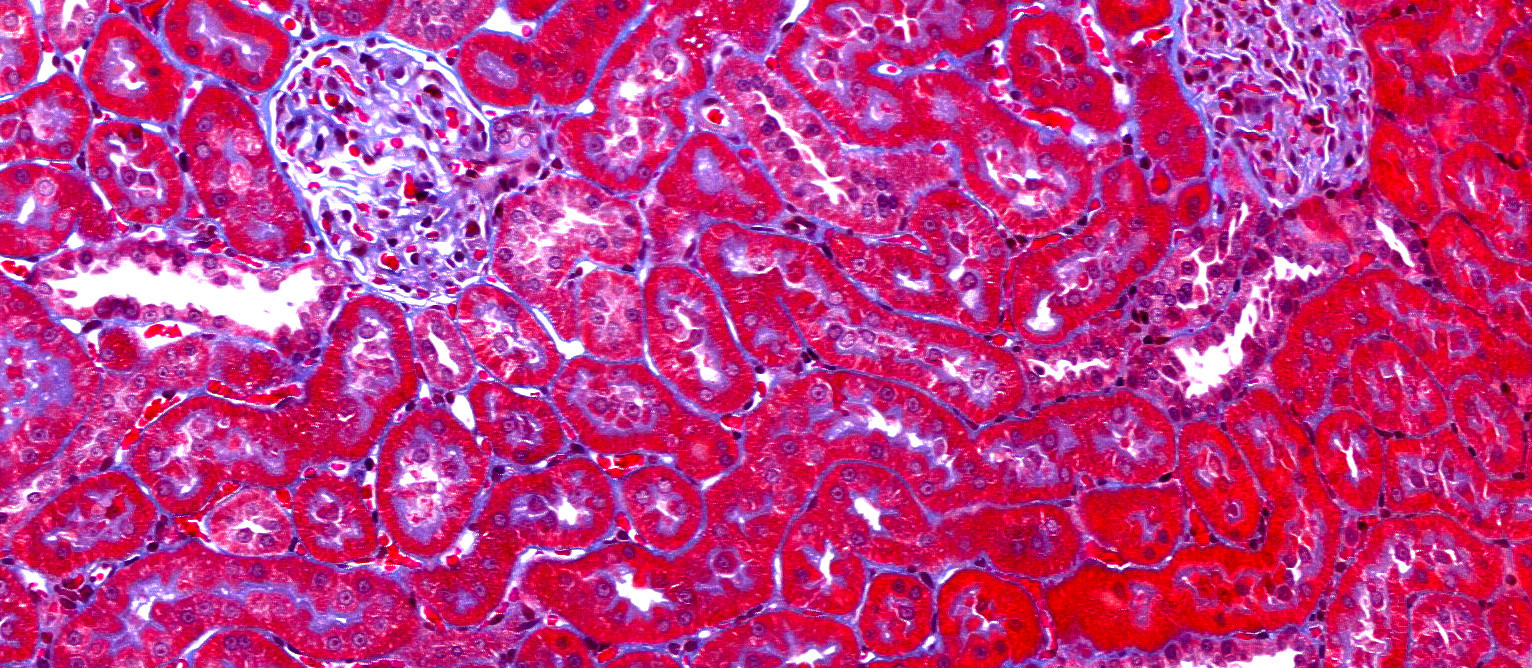

Supplement: Supplementary file 10 [file DataSheet6.ZIP › Fig 1D-masson-sham-2/2-9.jpeg]

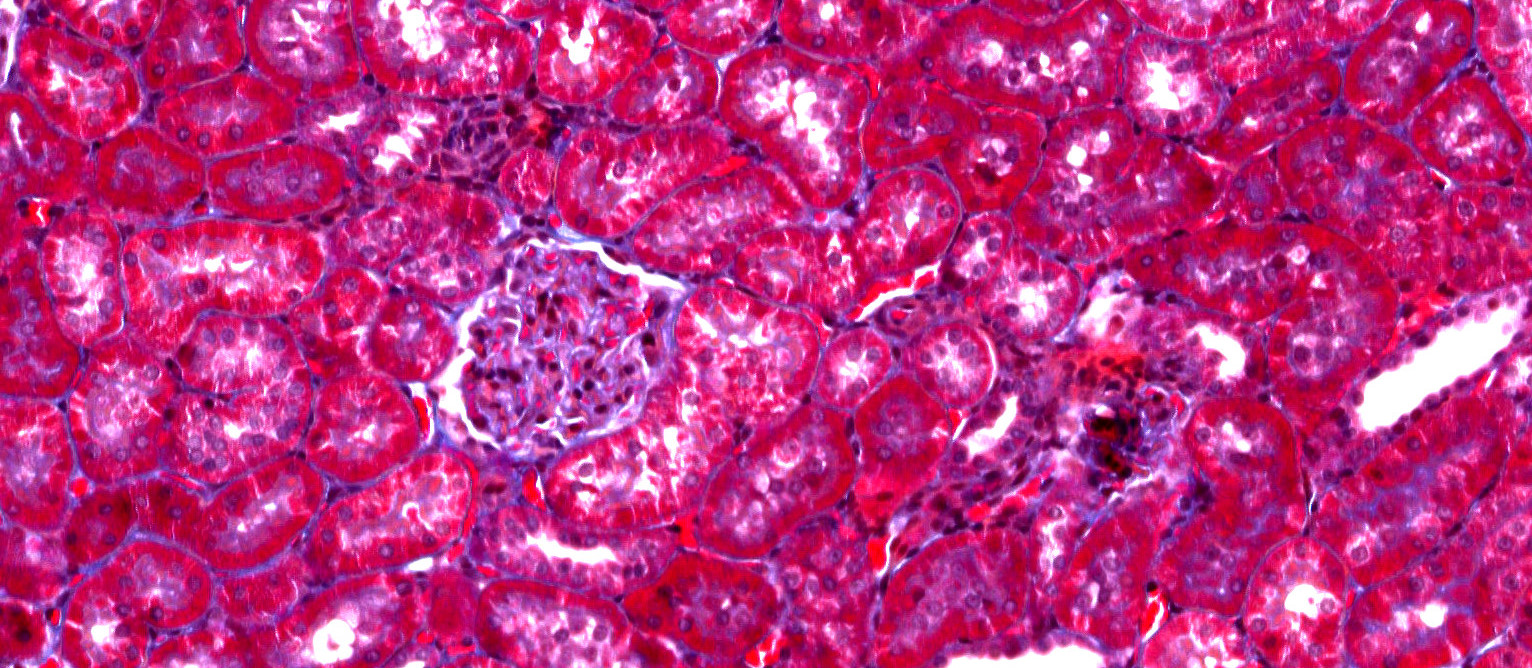

Supplement: Supplementary file 10 [file DataSheet6.ZIP › Fig 1D-masson-sham-3/3-1.jpeg]

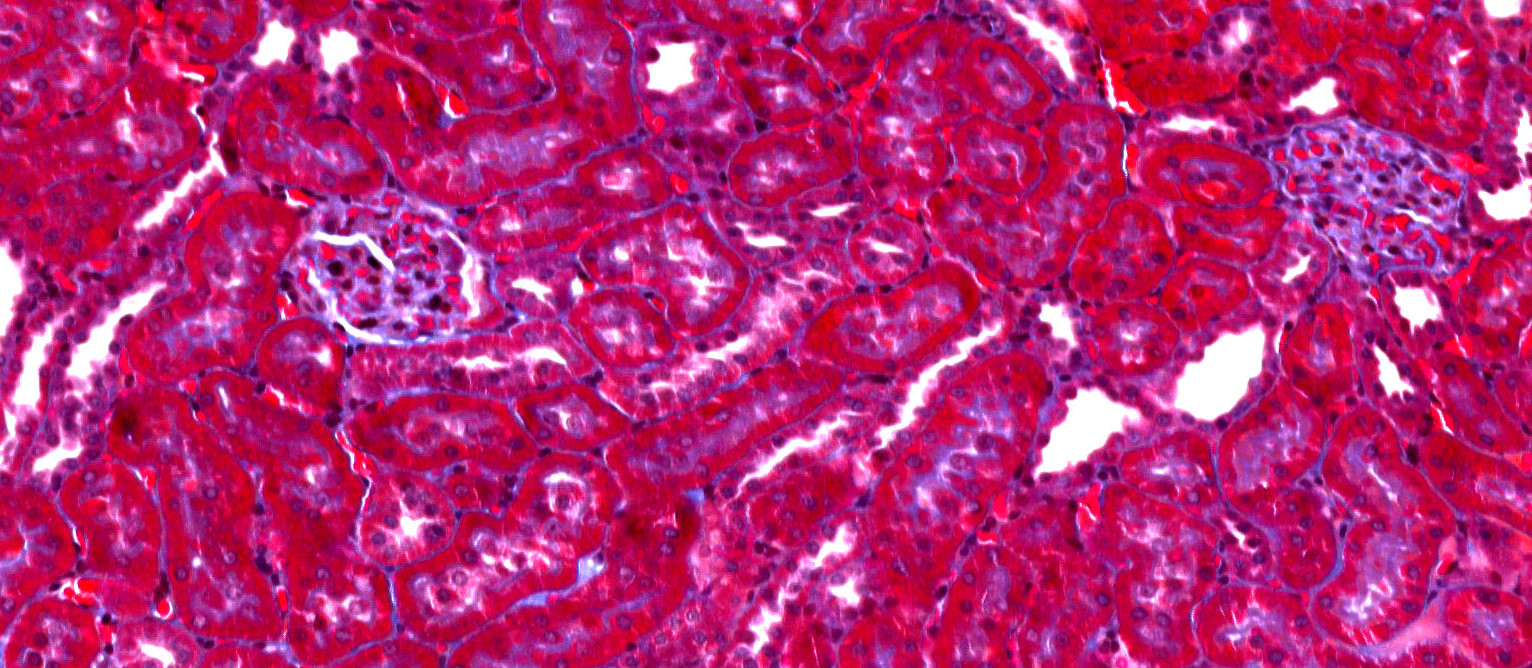

Supplement: Supplementary file 10 [file DataSheet6.ZIP › Fig 1D-masson-sham-3/3-10.jpeg]

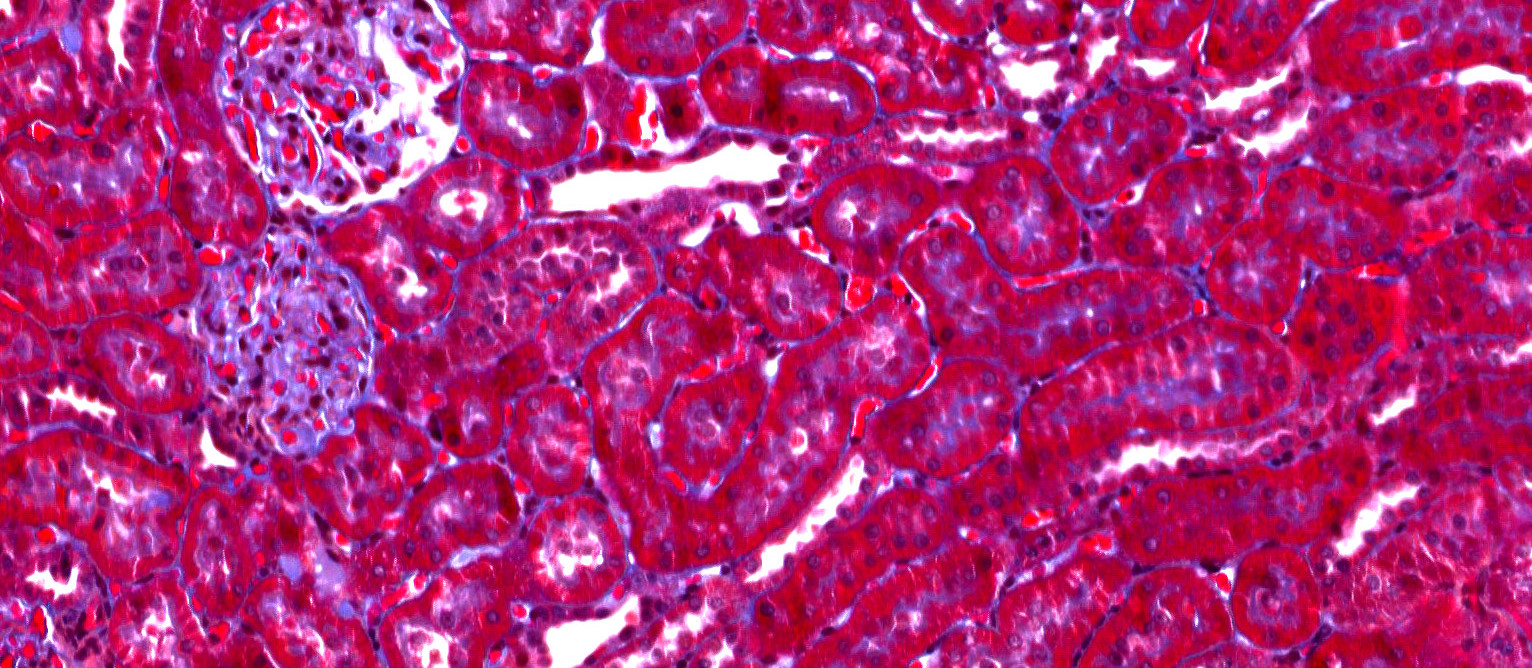

Supplement: Supplementary file 10 [file DataSheet6.ZIP › Fig 1D-masson-sham-3/3-2.jpeg]

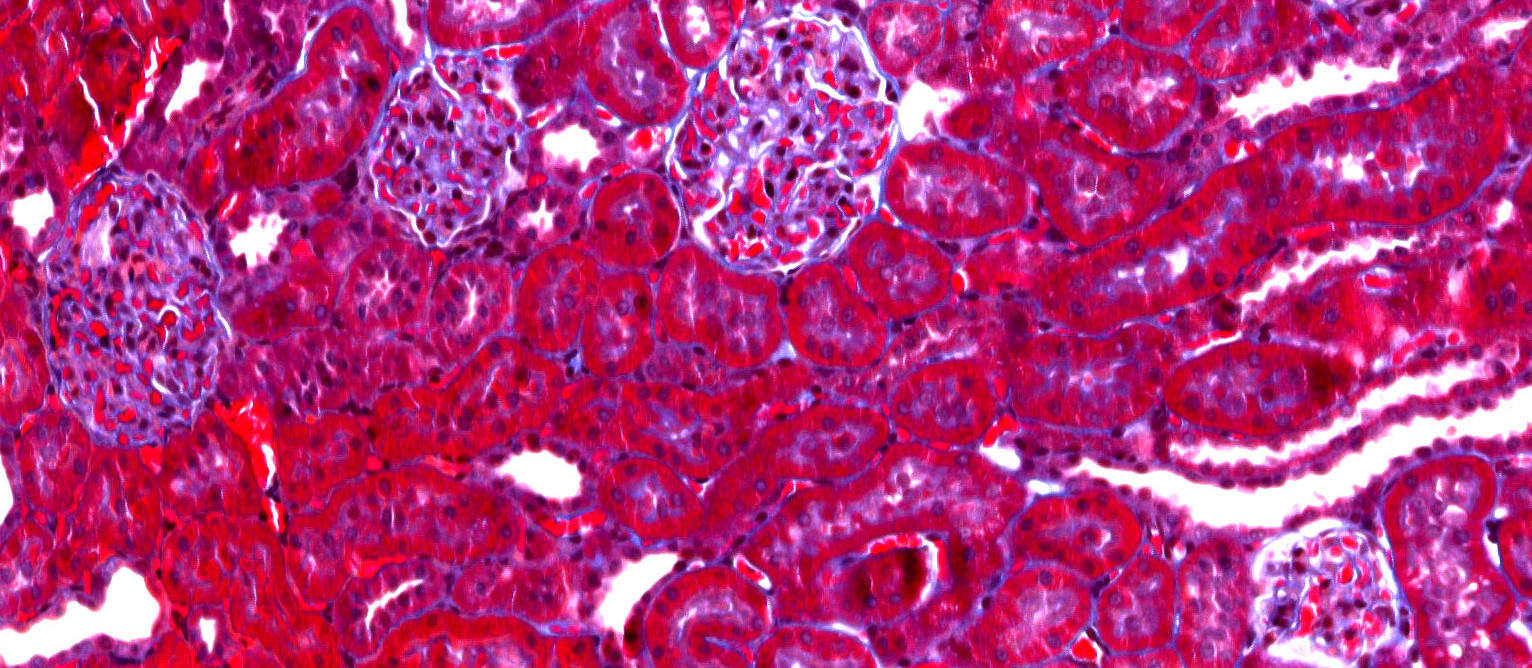

Supplement: Supplementary file 10 [file DataSheet6.ZIP › Fig 1D-masson-sham-3/3-3.jpeg]

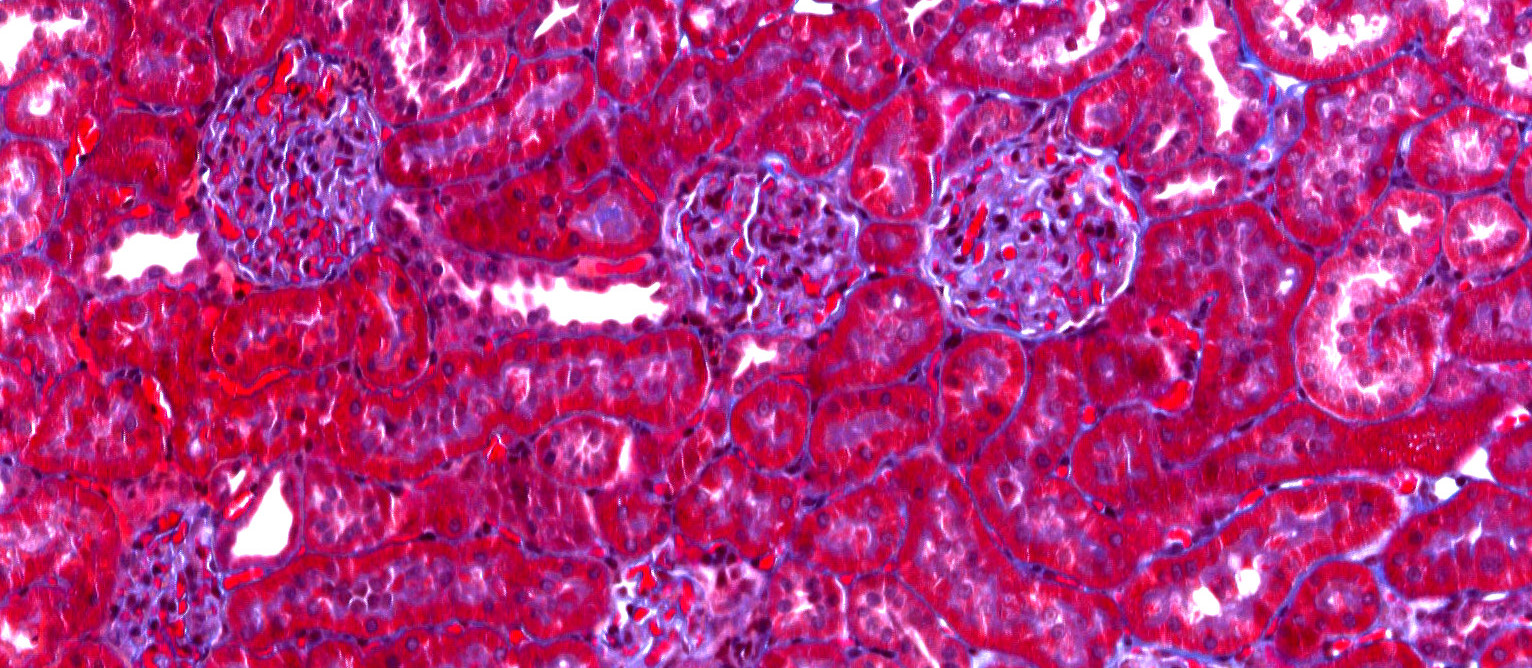

Supplement: Supplementary file 10 [file DataSheet6.ZIP › Fig 1D-masson-sham-3/3-4.jpeg]

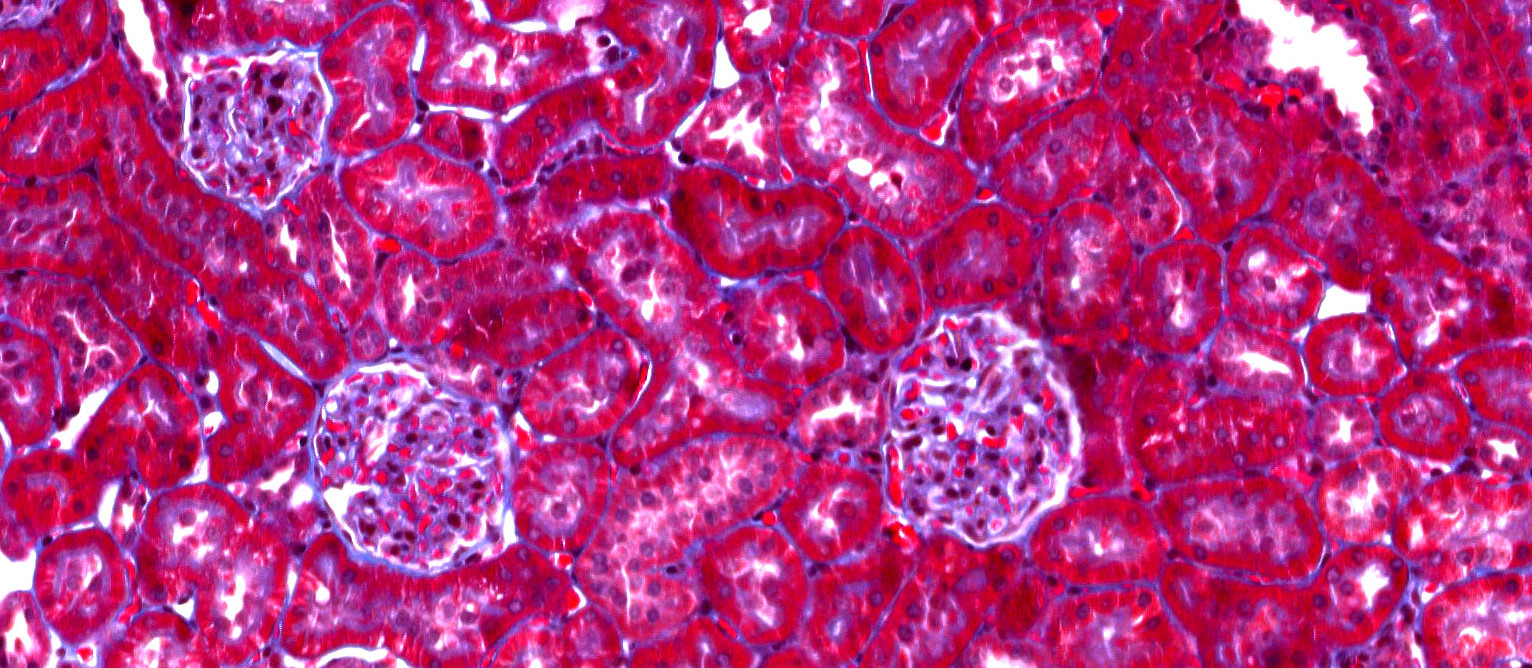

Supplement: Supplementary file 10 [file DataSheet6.ZIP › Fig 1D-masson-sham-3/3-5.jpeg]

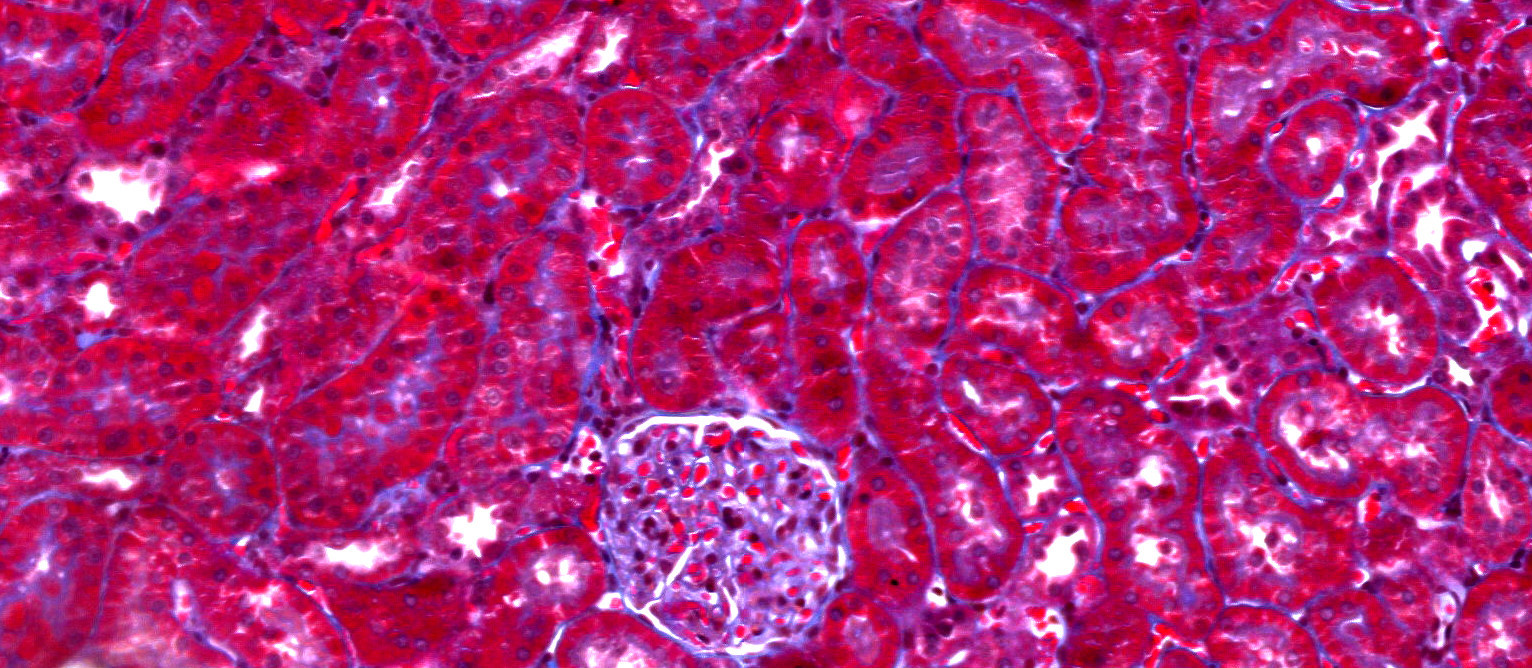

Supplement: Supplementary file 10 [file DataSheet6.ZIP › Fig 1D-masson-sham-3/3-6.jpeg]

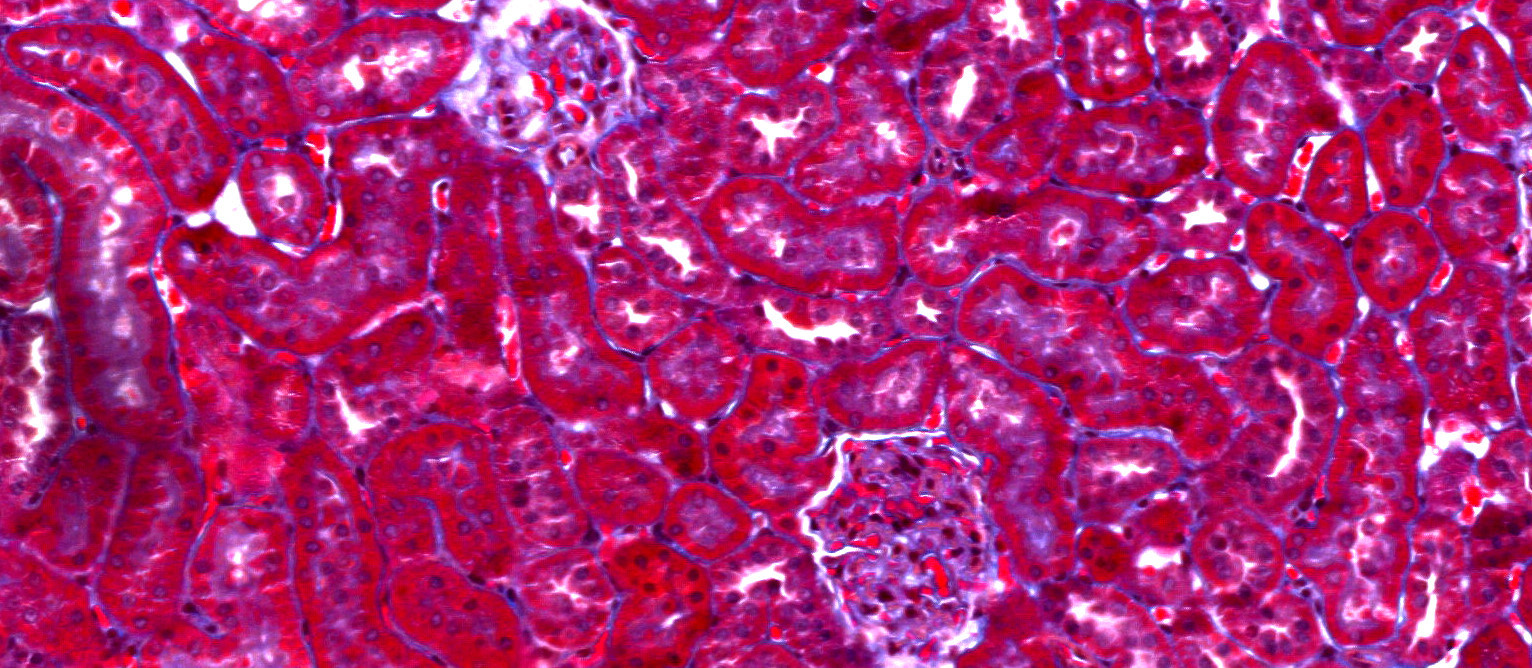

Supplement: Supplementary file 10 [file DataSheet6.ZIP › Fig 1D-masson-sham-3/3-7.jpeg]
