# Supplementary figures and images for: Characteristics of Serum Metabolites and Gut Microbiota in Diabetic Kidney Disease (part 9 of 13)
Source: Front Pharmacol. 2022 Apr 14;13:872988. doi: 10.3389/fphar.2022.872988 (PMC9084235; doi:10.3389/fphar.2022.872988)

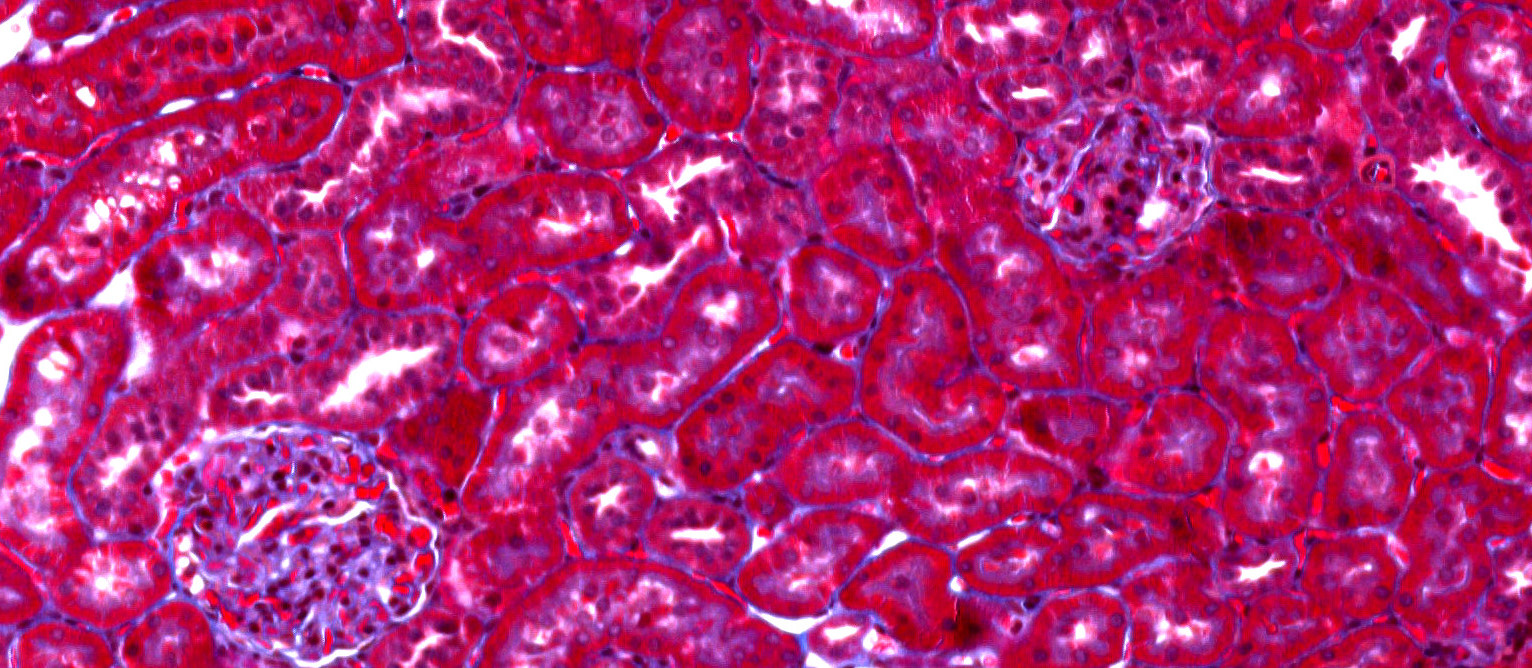

Supplement: Supplementary file 10 [file DataSheet6.ZIP › Fig 1D-masson-sham-3/3-8.jpeg]

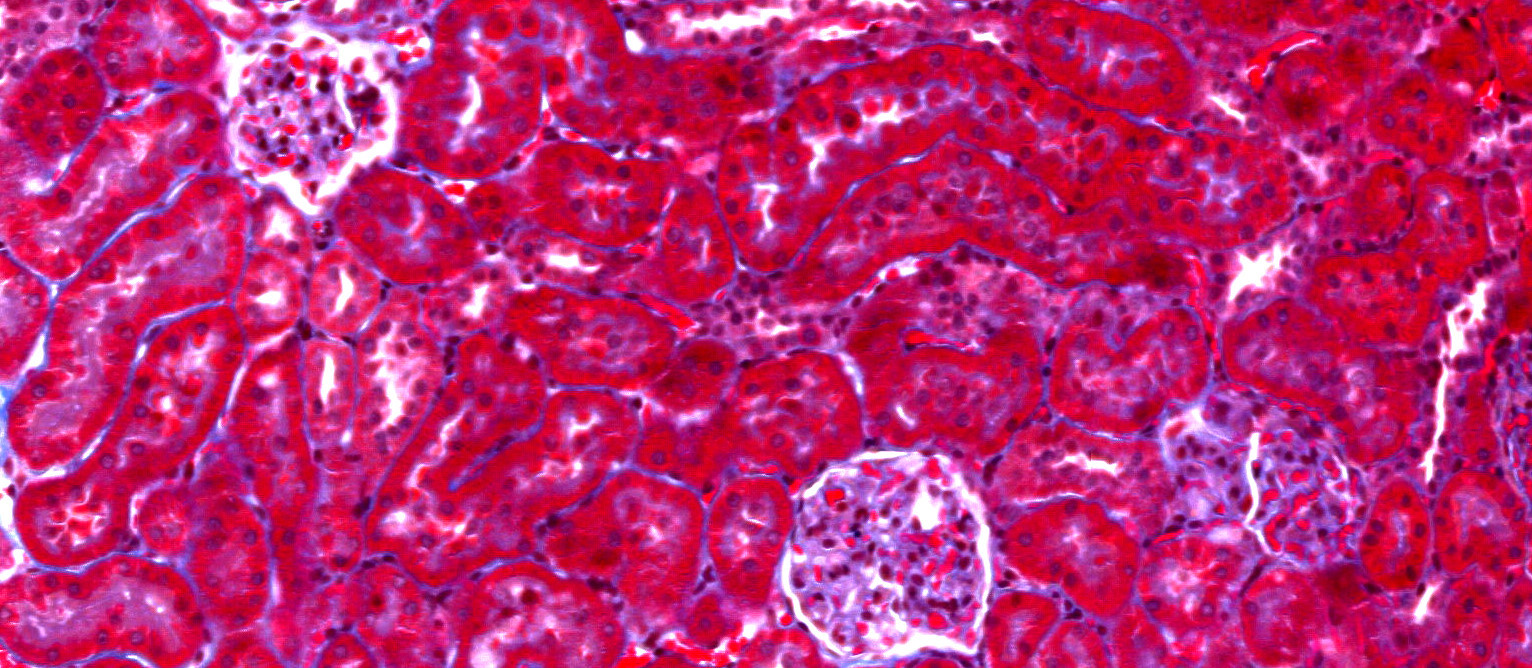

Supplement: Supplementary file 10 [file DataSheet6.ZIP › Fig 1D-masson-sham-3/3-9.jpeg]

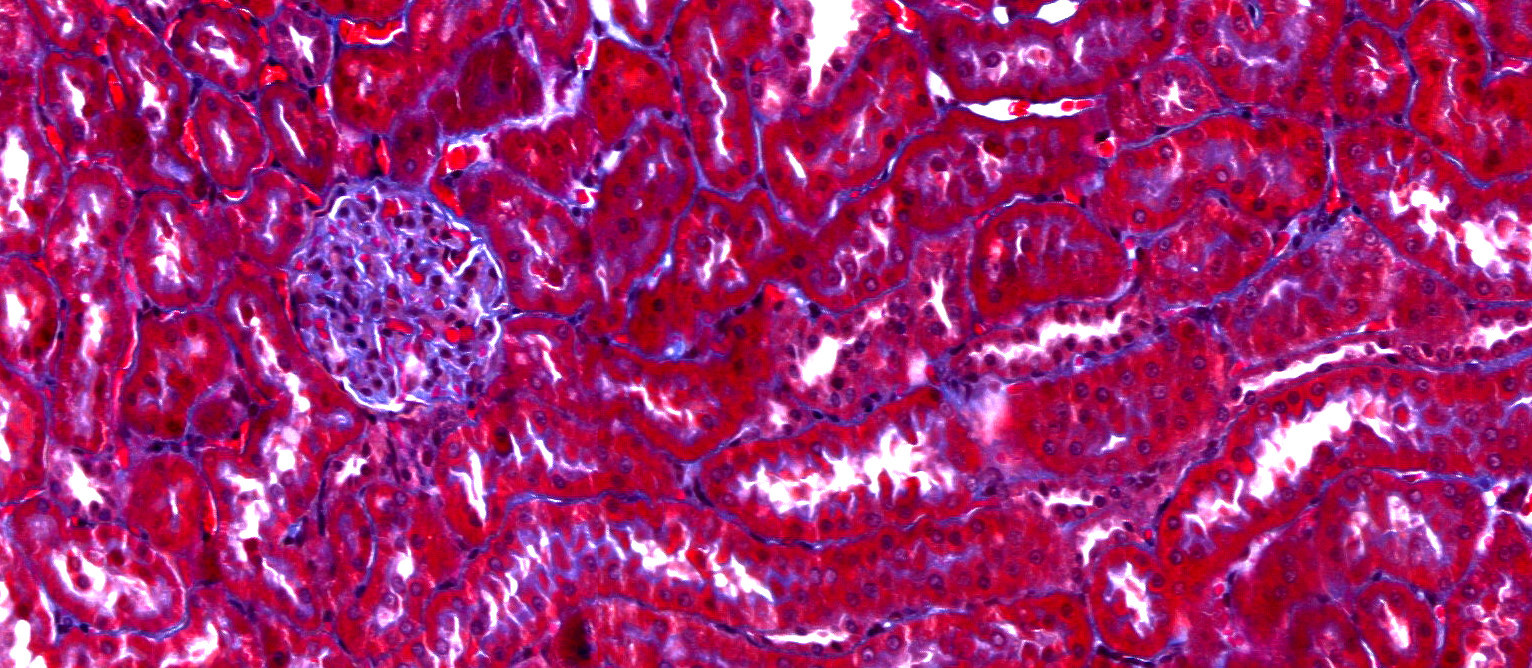

Supplement: Supplementary file 10 [file DataSheet6.ZIP › Fig 1D-masson-sham-4/4-1.jpeg]

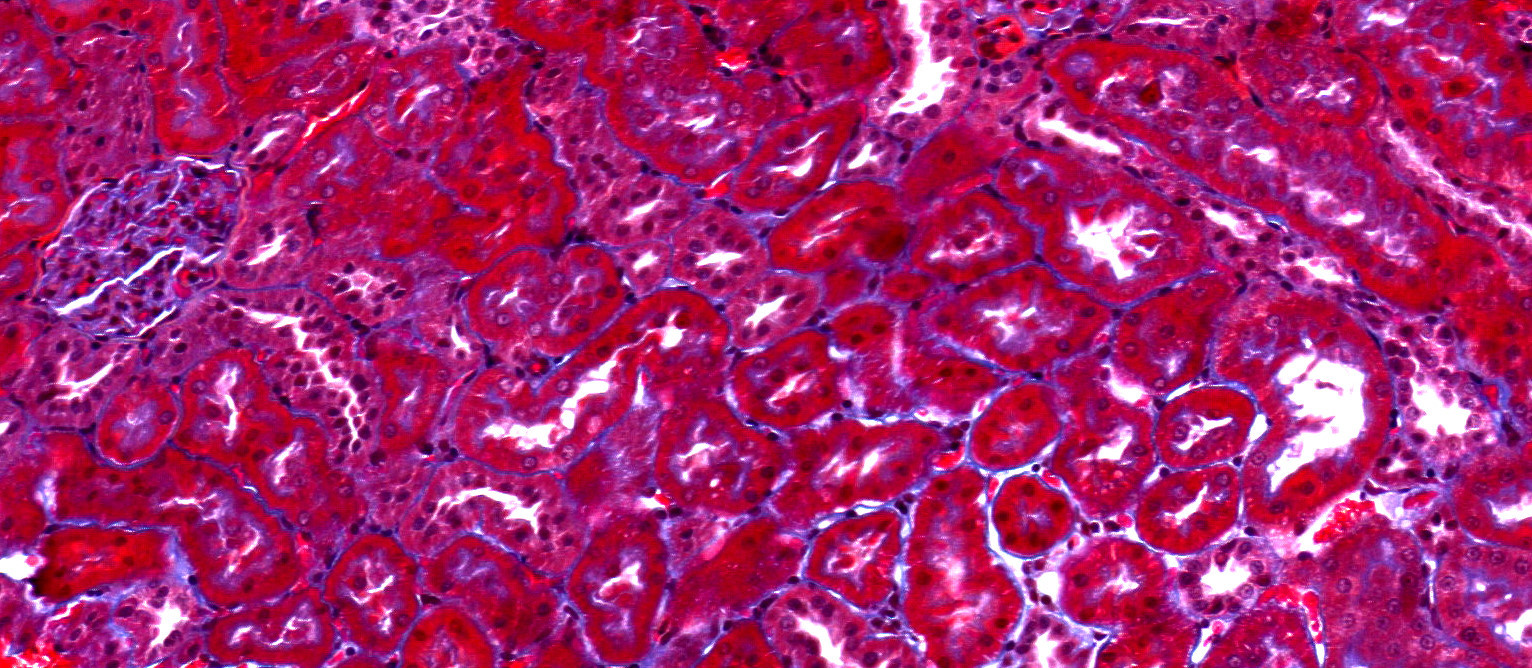

Supplement: Supplementary file 10 [file DataSheet6.ZIP › Fig 1D-masson-sham-4/4-10.jpeg]

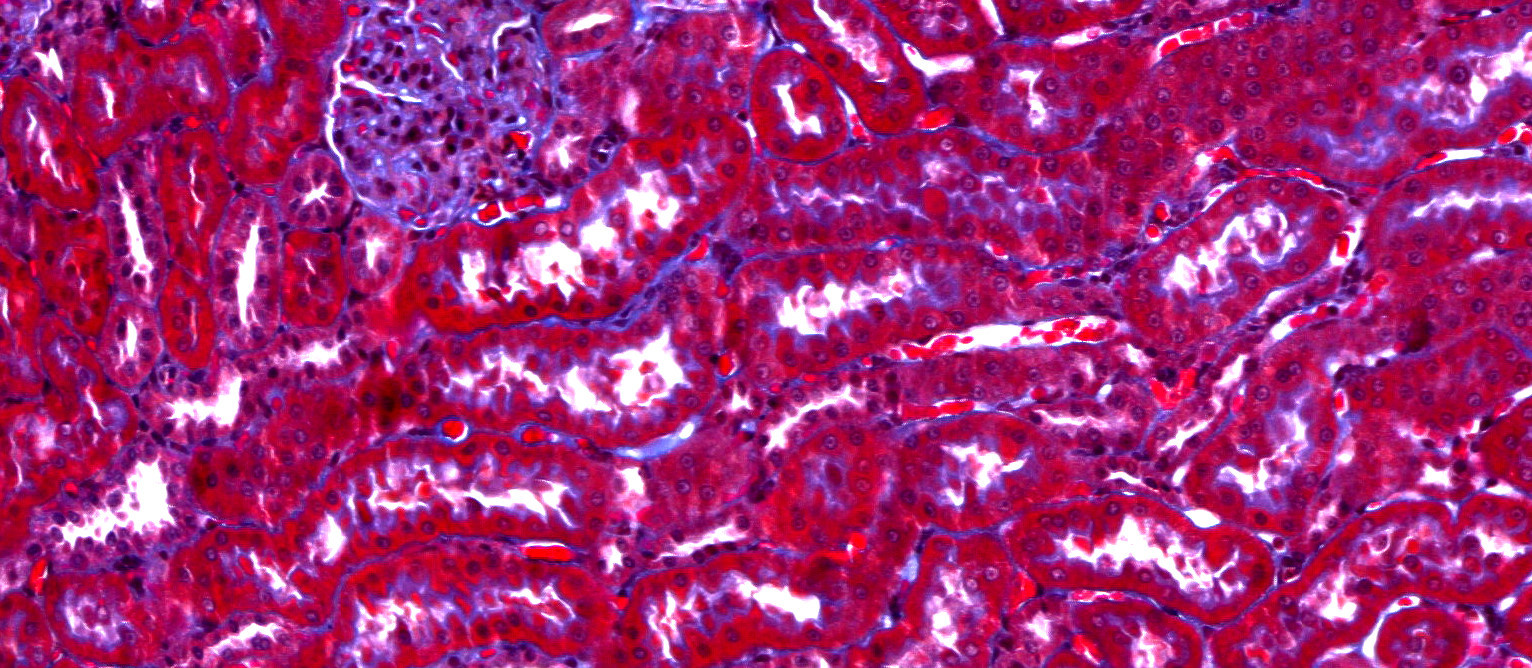

Supplement: Supplementary file 10 [file DataSheet6.ZIP › Fig 1D-masson-sham-4/4-2.jpeg]

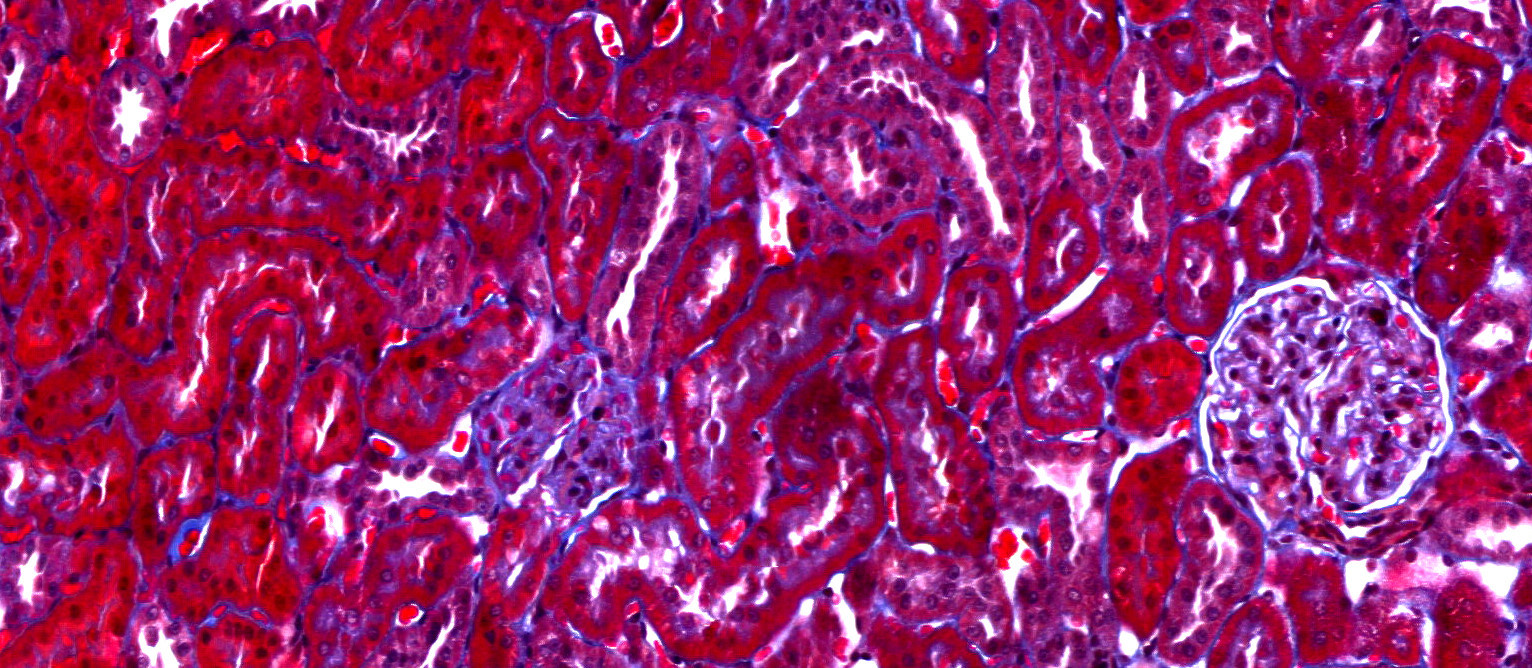

Supplement: Supplementary file 10 [file DataSheet6.ZIP › Fig 1D-masson-sham-4/4-3.jpeg]

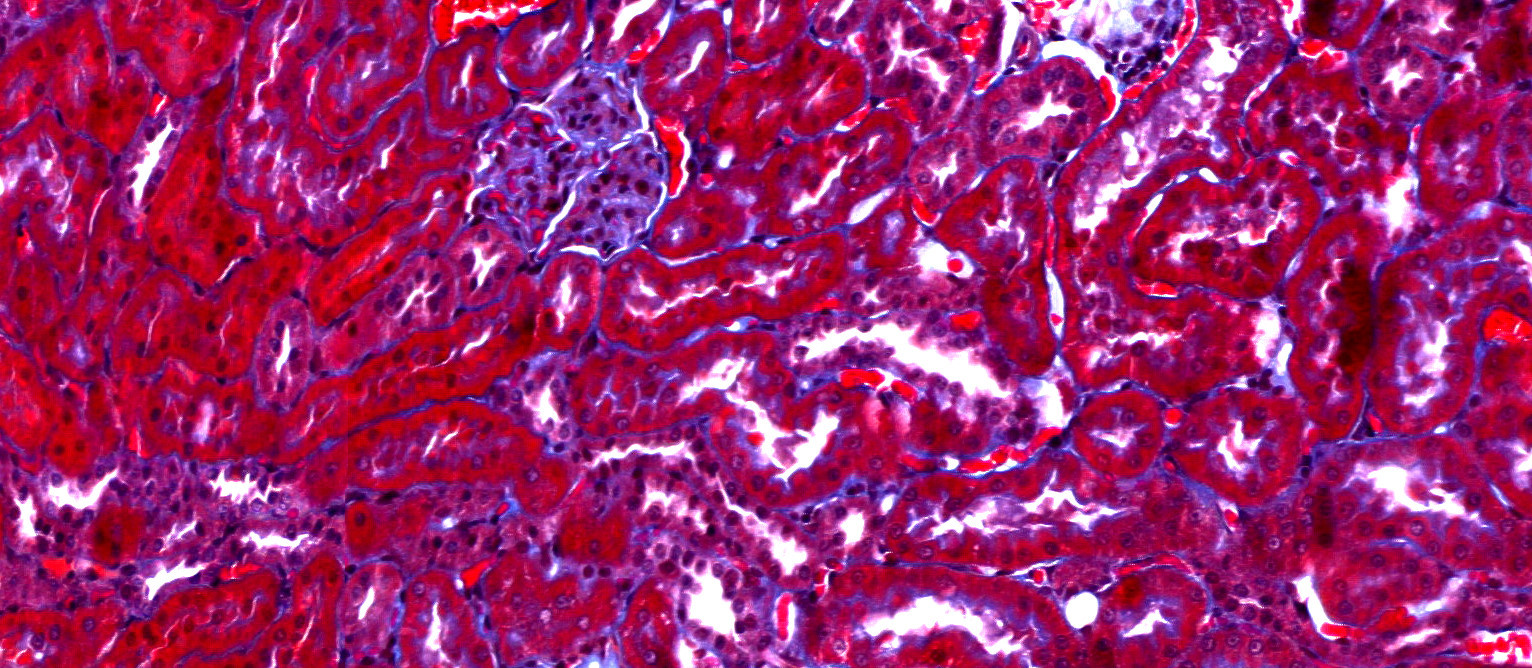

Supplement: Supplementary file 10 [file DataSheet6.ZIP › Fig 1D-masson-sham-4/4-4.jpeg]

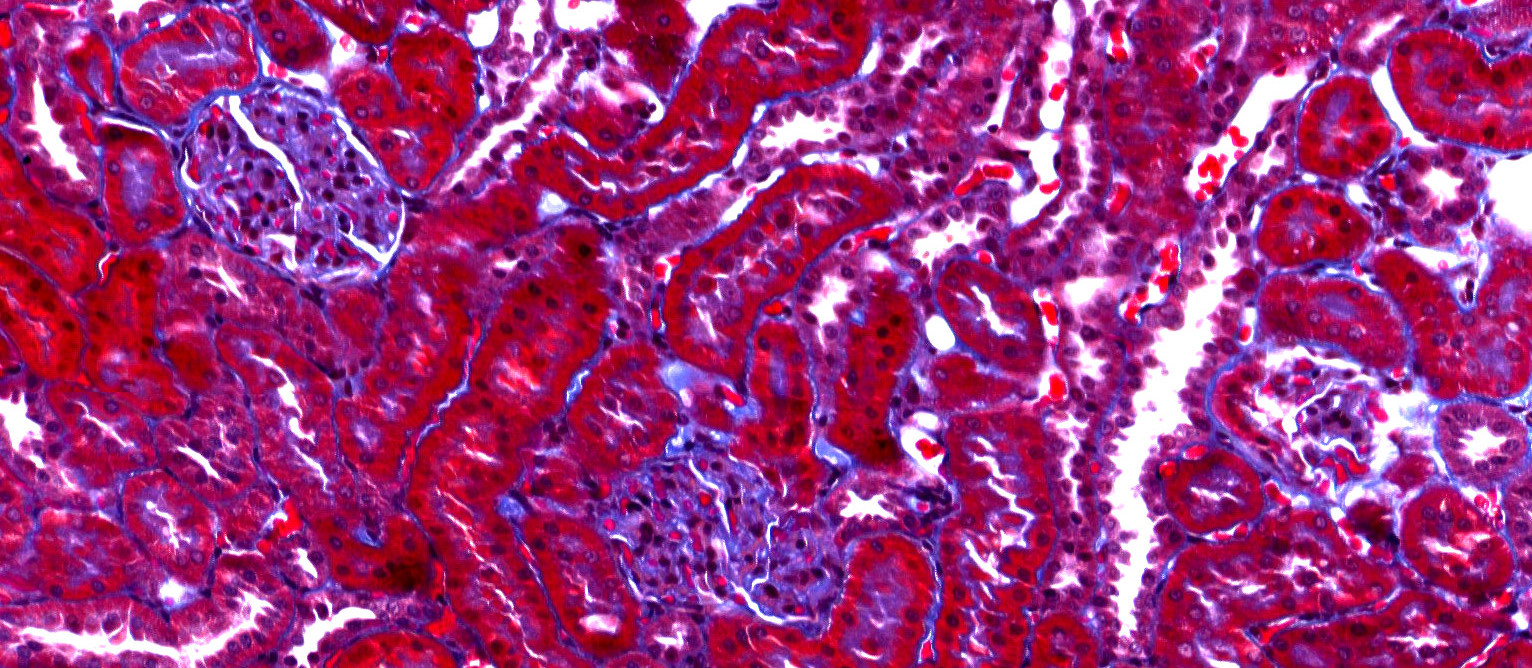

Supplement: Supplementary file 10 [file DataSheet6.ZIP › Fig 1D-masson-sham-4/4-5.jpeg]

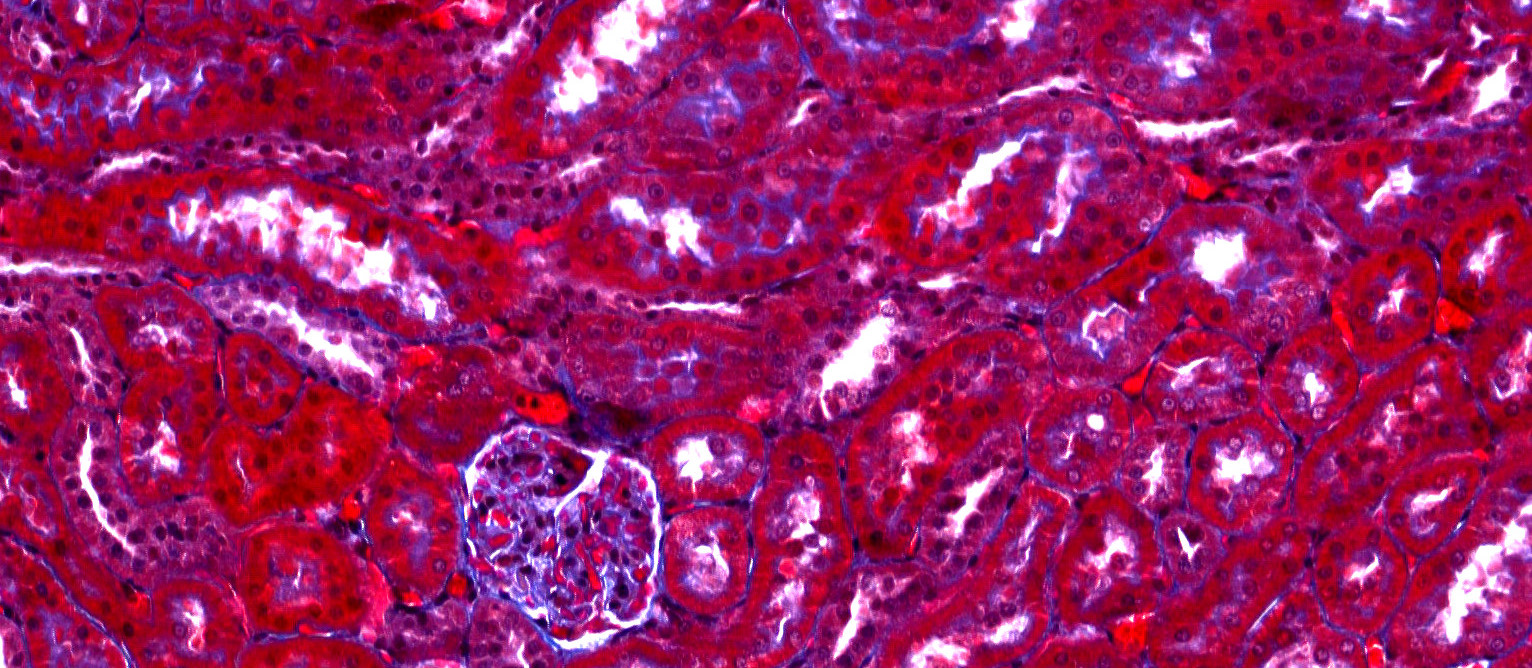

Supplement: Supplementary file 10 [file DataSheet6.ZIP › Fig 1D-masson-sham-4/4-6.jpeg]

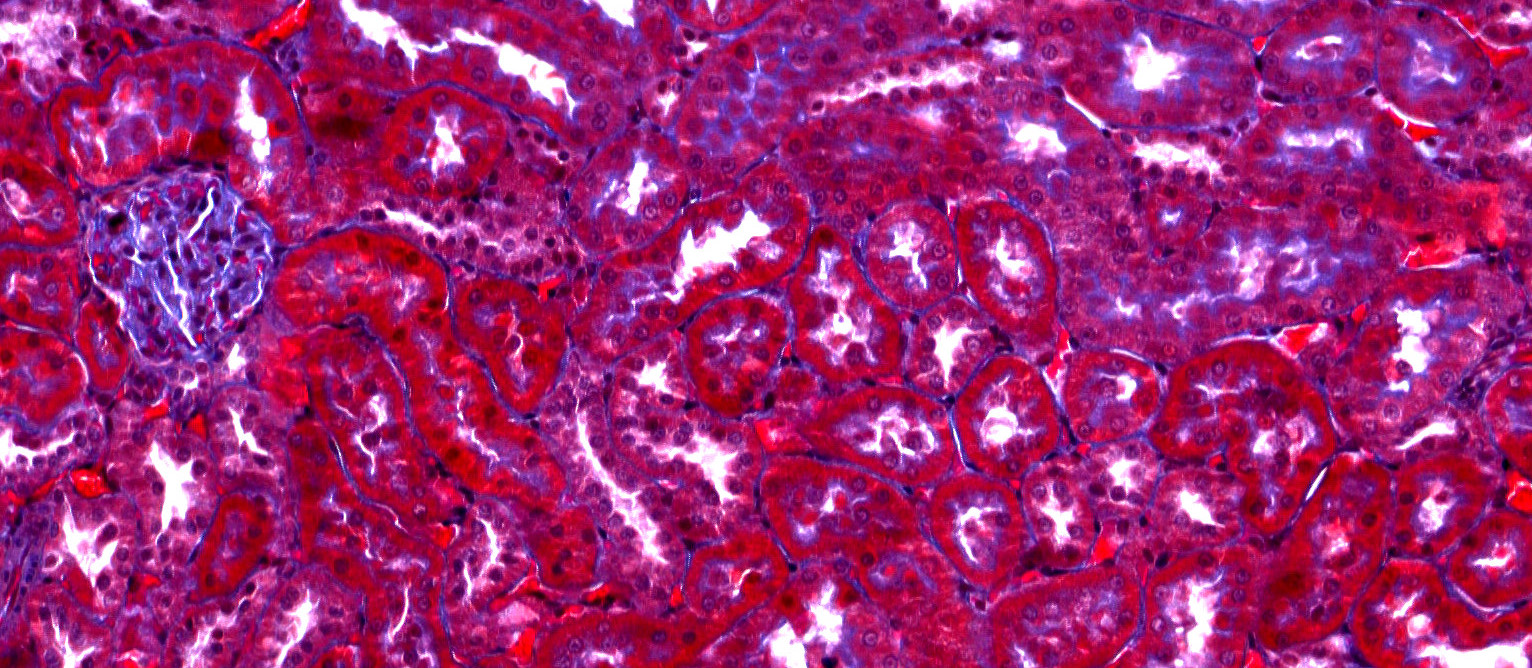

Supplement: Supplementary file 10 [file DataSheet6.ZIP › Fig 1D-masson-sham-4/4-7.jpeg]

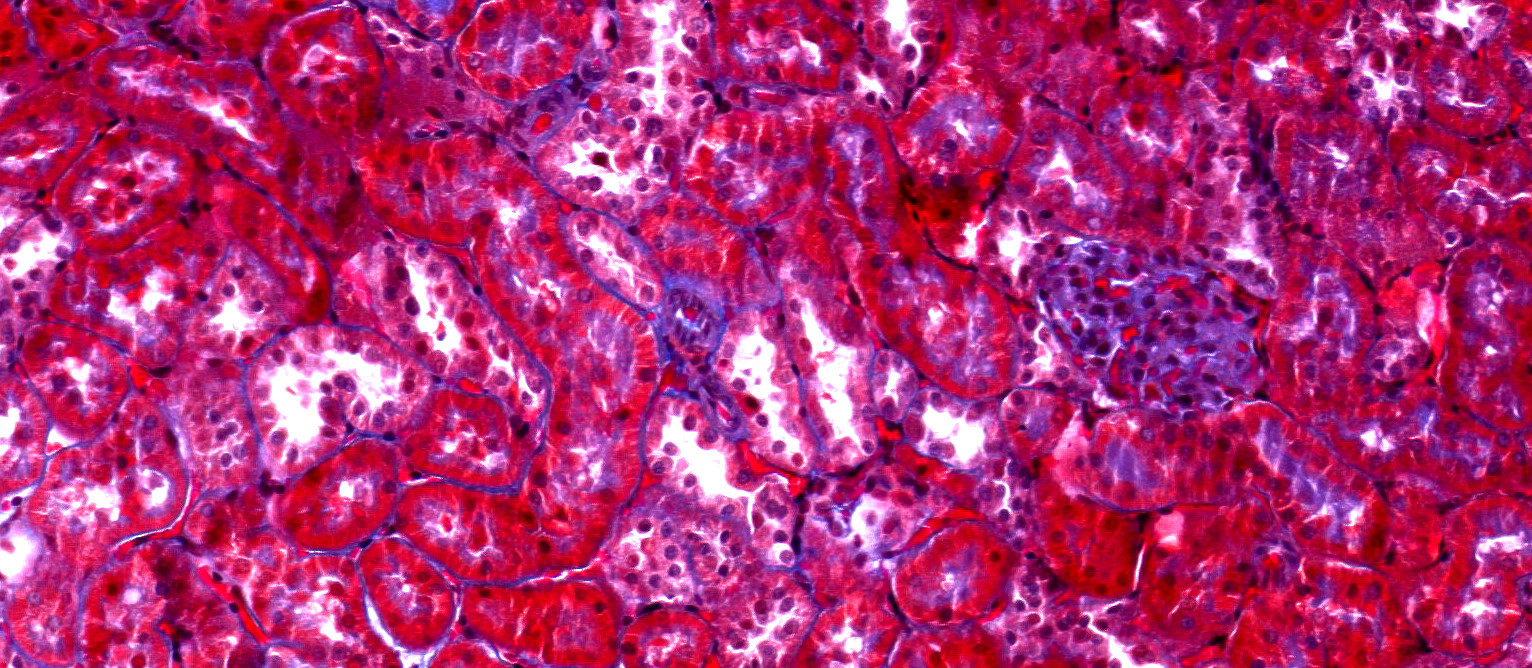

Supplement: Supplementary file 10 [file DataSheet6.ZIP › Fig 1D-masson-sham-4/4-8.jpeg]

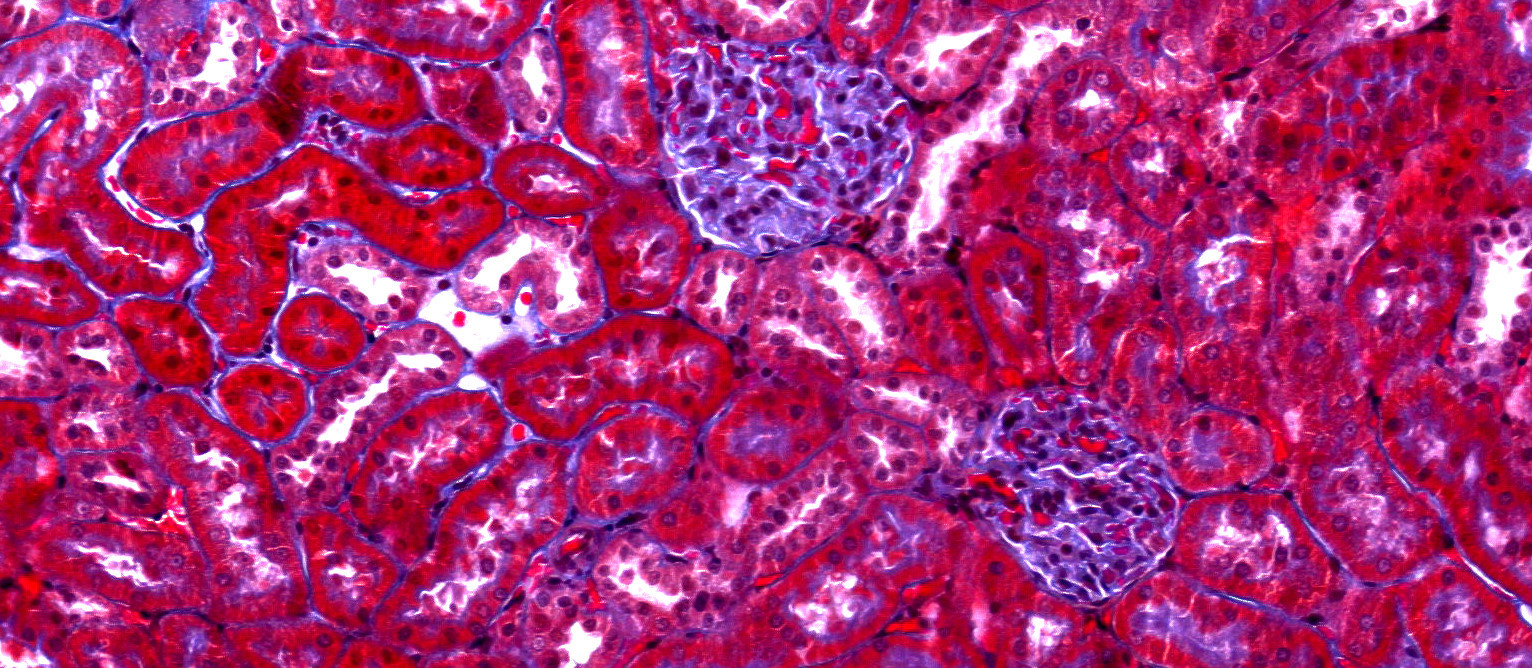

Supplement: Supplementary file 10 [file DataSheet6.ZIP › Fig 1D-masson-sham-4/4-9.jpeg]

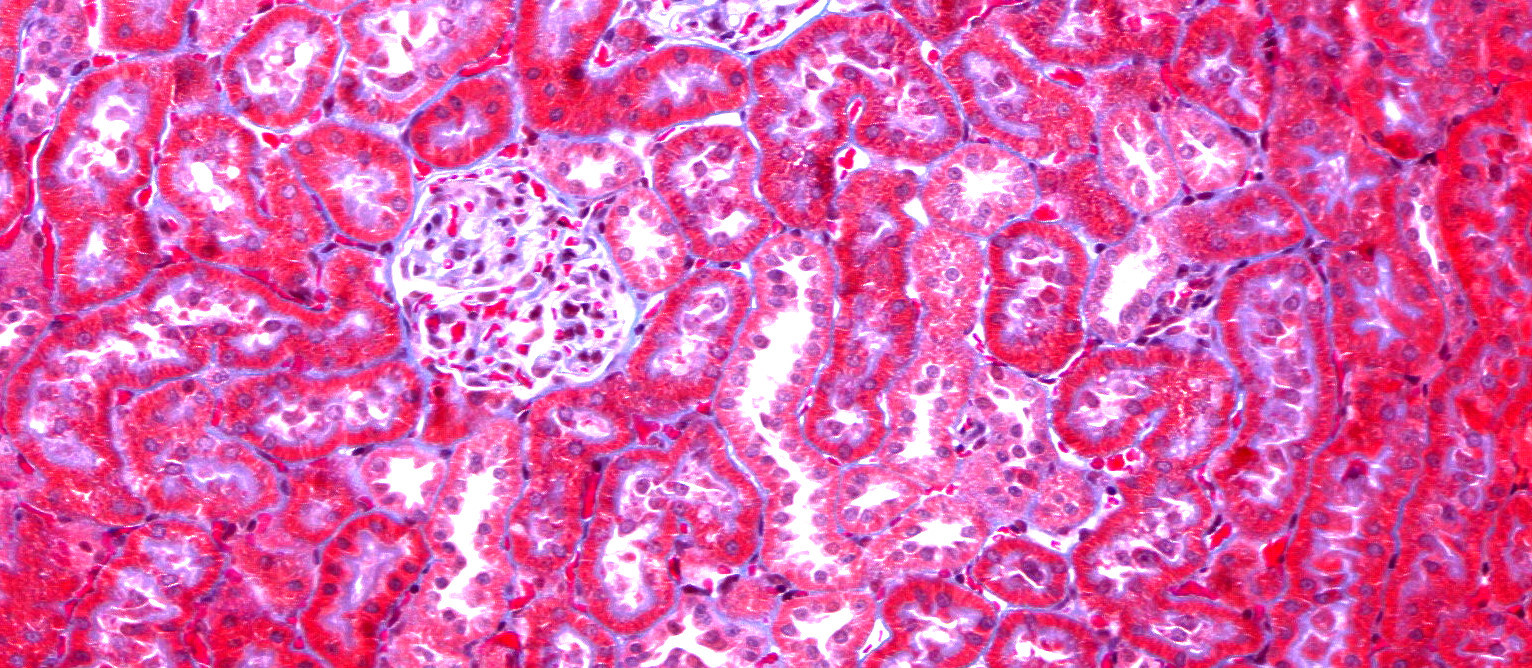

Supplement: Supplementary file 10 [file DataSheet6.ZIP › Fig 1D-masson-sham-5(1)/5-1.jpeg]

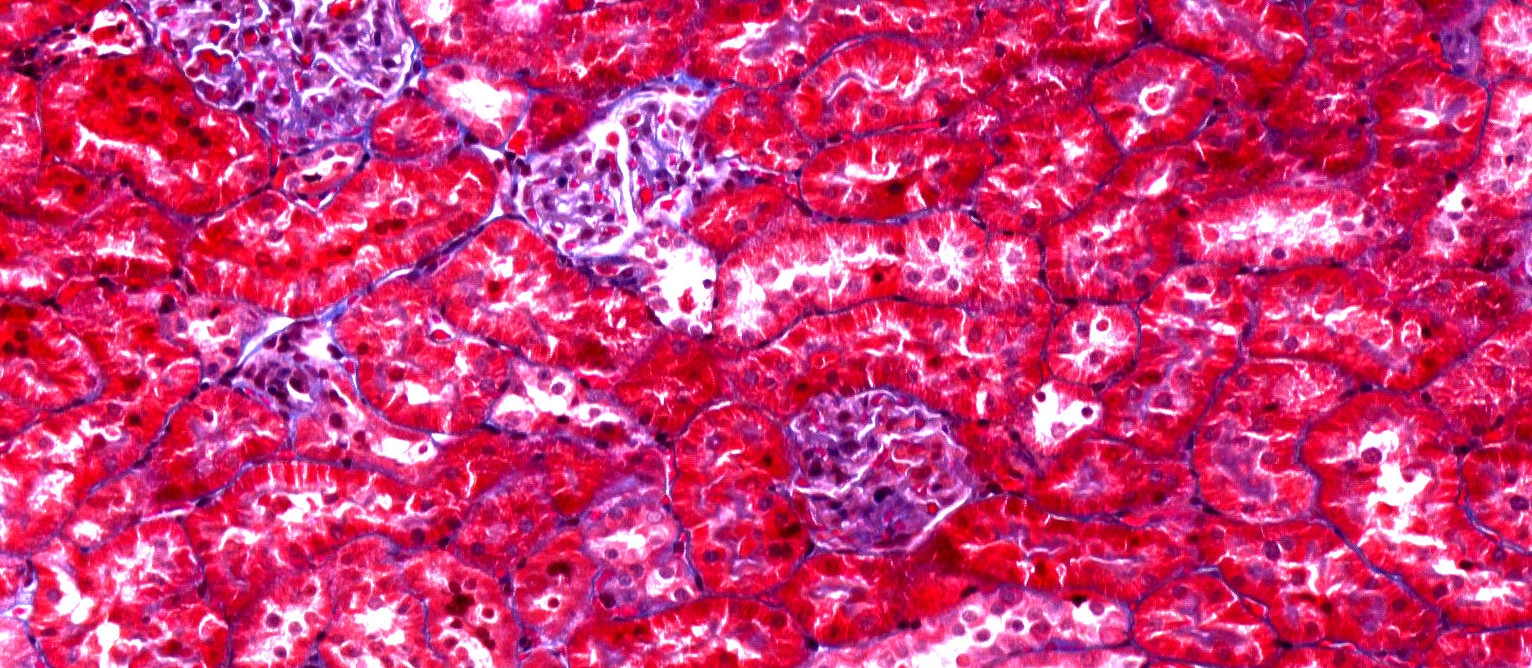

Supplement: Supplementary file 11 [file DataSheet12.ZIP › Fig 1D-masson-TSF-62(2)/62-10.jpeg]

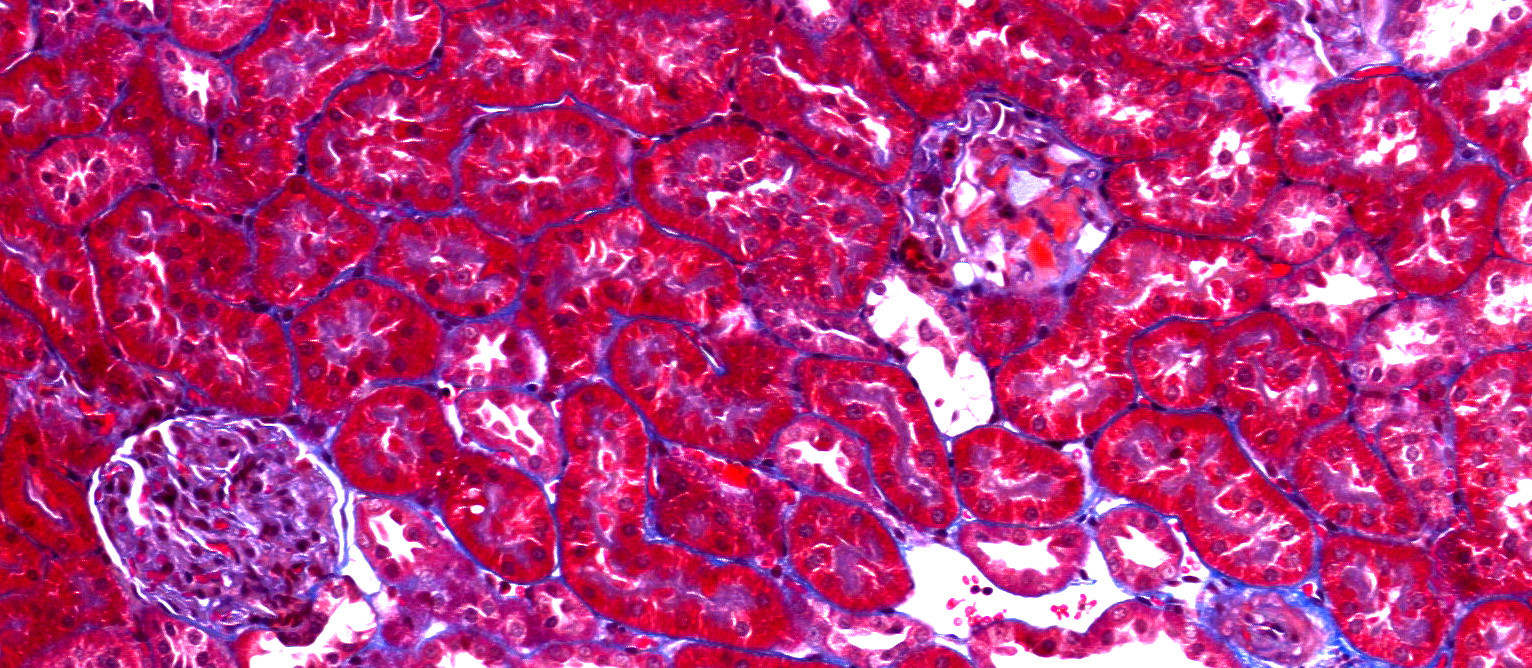

Supplement: Supplementary file 11 [file DataSheet12.ZIP › Fig 1D-masson-TSF-62(2)/62-4.jpeg]

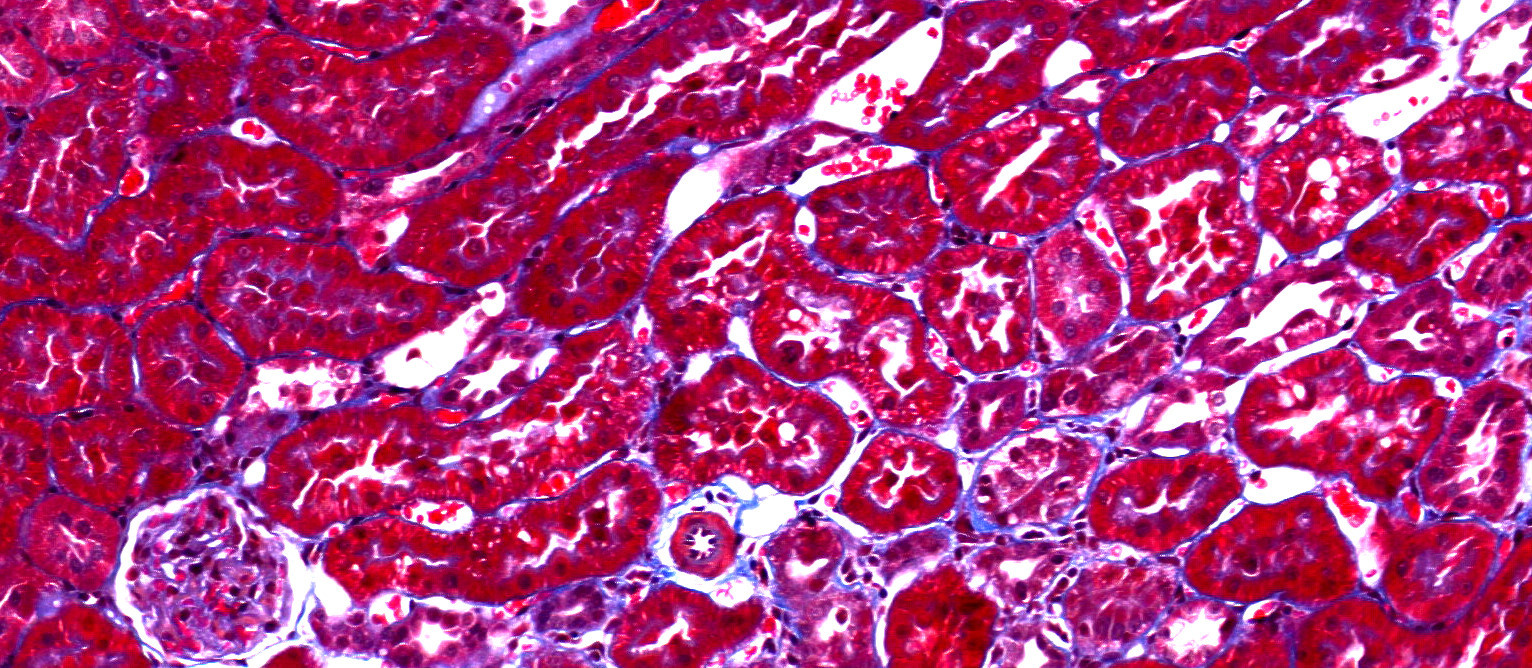

Supplement: Supplementary file 11 [file DataSheet12.ZIP › Fig 1D-masson-TSF-62(2)/62-5.jpeg]

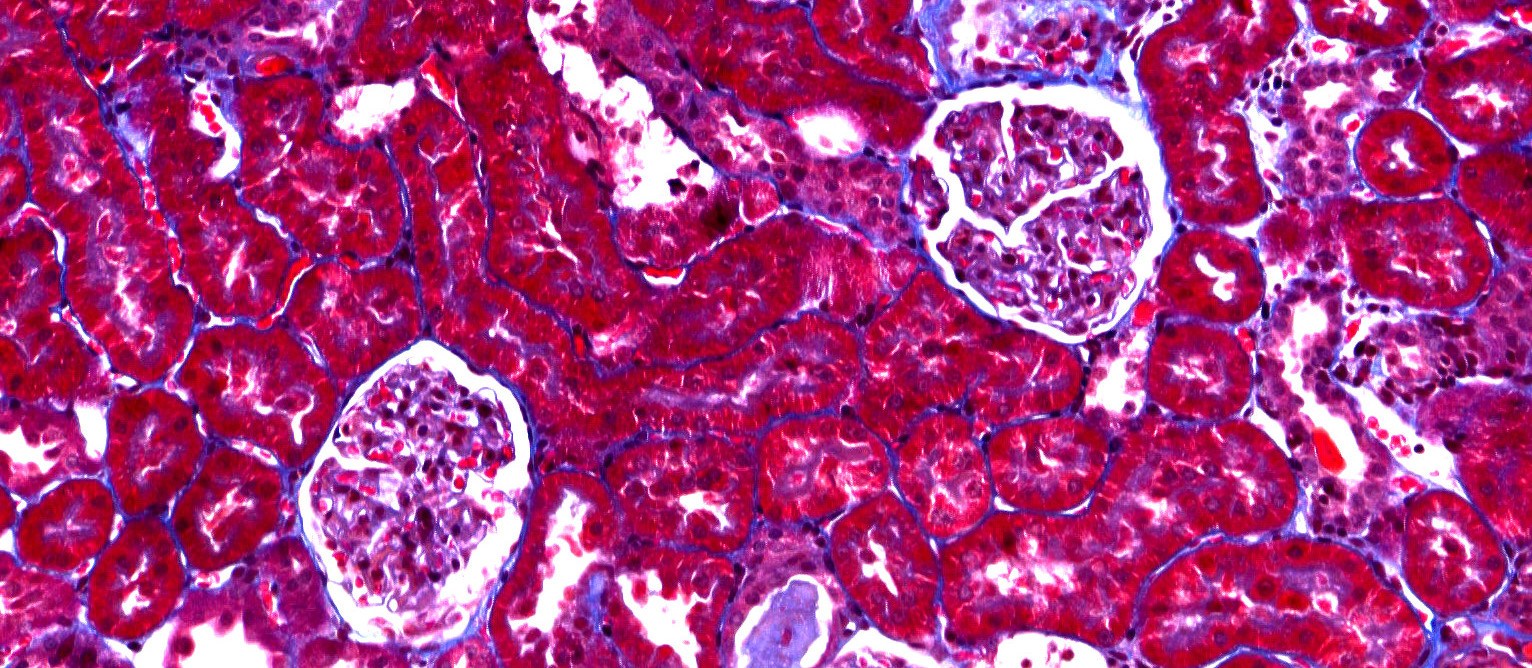

Supplement: Supplementary file 11 [file DataSheet12.ZIP › Fig 1D-masson-TSF-62(2)/62-6.jpeg]

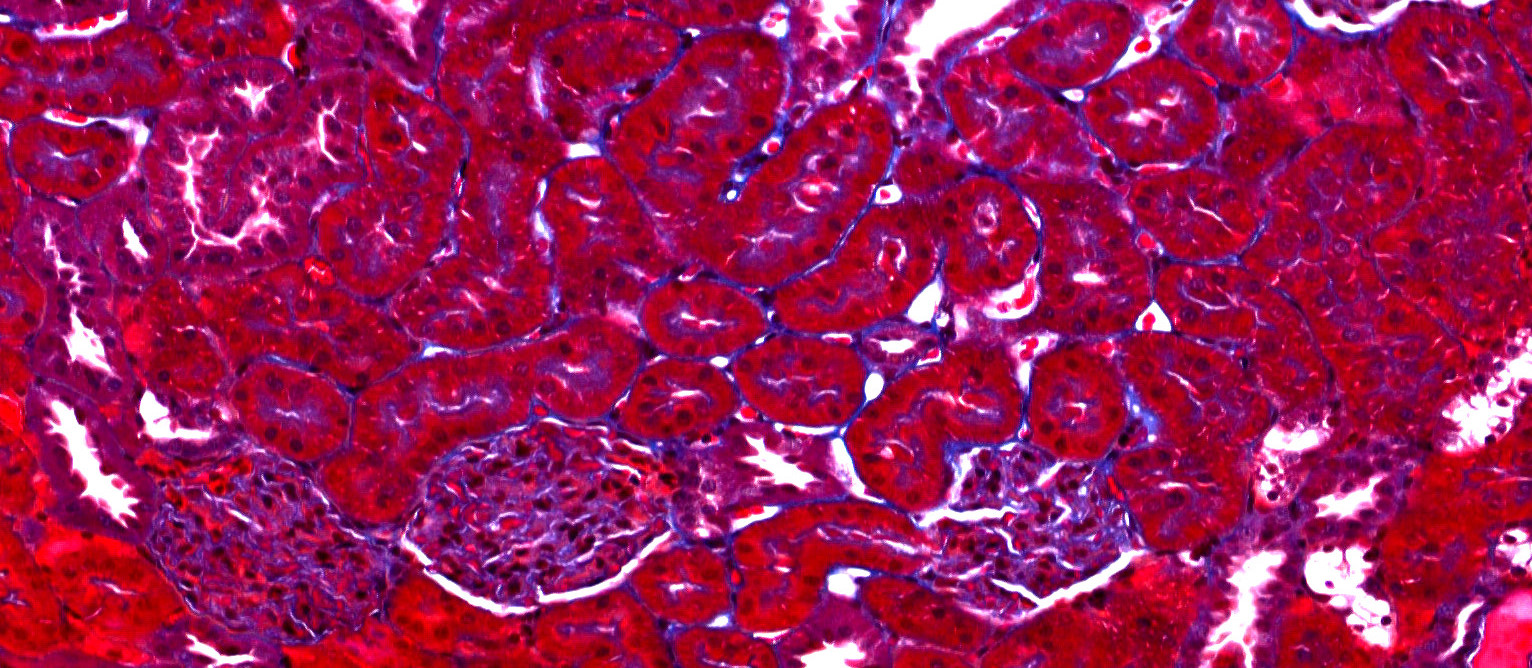

Supplement: Supplementary file 11 [file DataSheet12.ZIP › Fig 1D-masson-TSF-62(2)/62-7.jpeg]

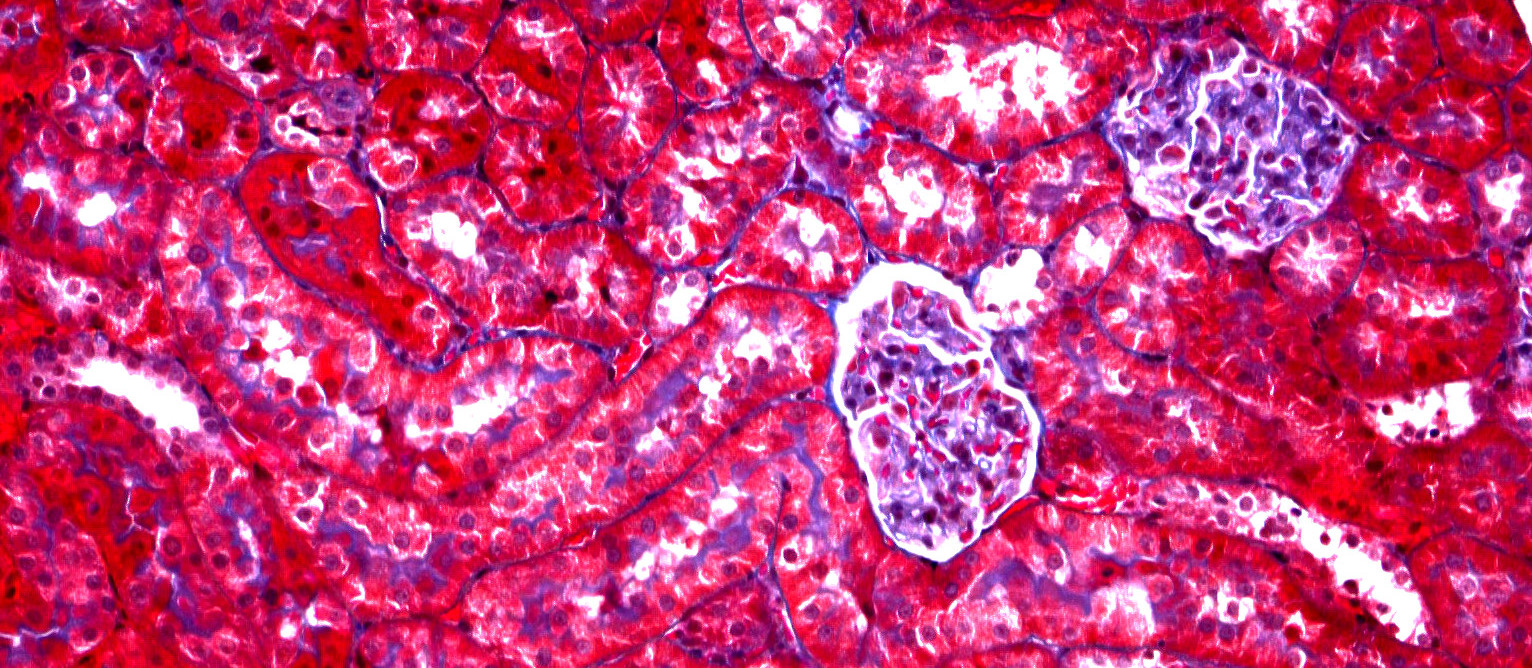

Supplement: Supplementary file 11 [file DataSheet12.ZIP › Fig 1D-masson-TSF-62(2)/62-8.jpeg]

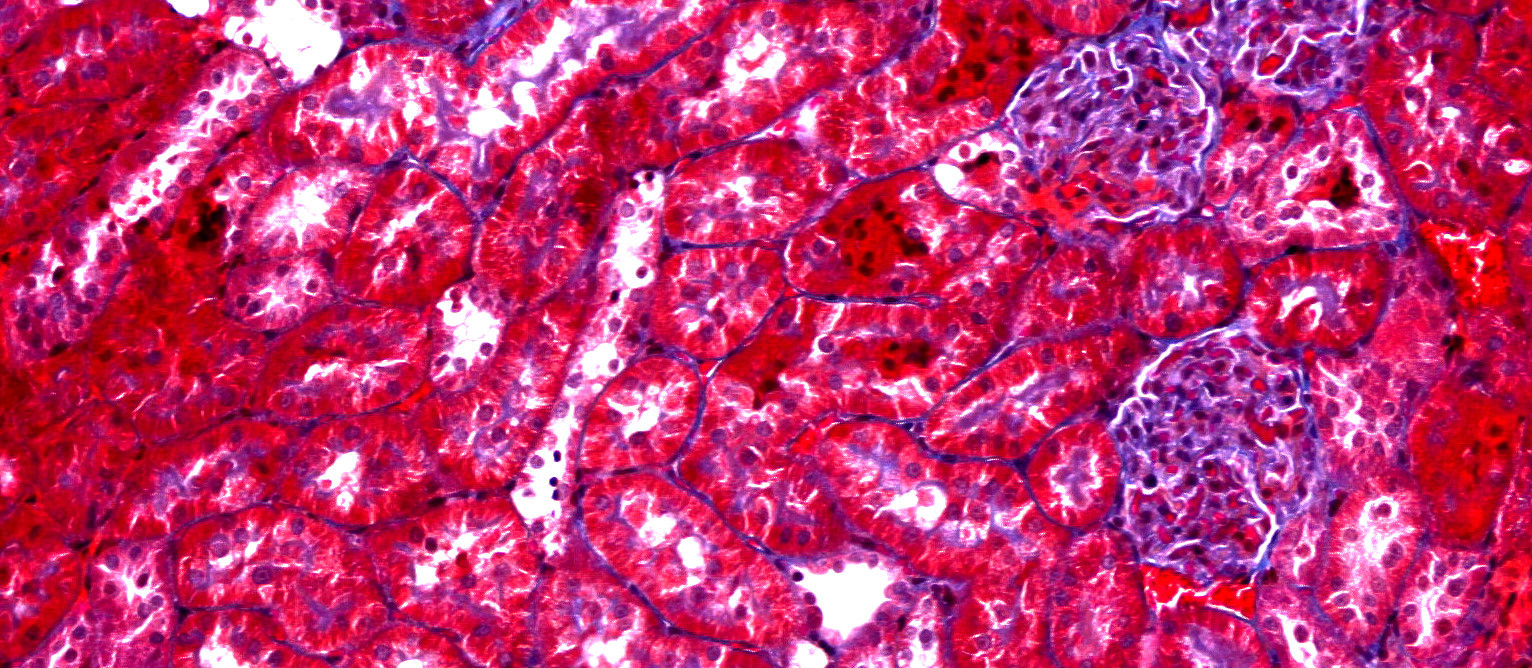

Supplement: Supplementary file 11 [file DataSheet12.ZIP › Fig 1D-masson-TSF-62(2)/62-9.jpeg]

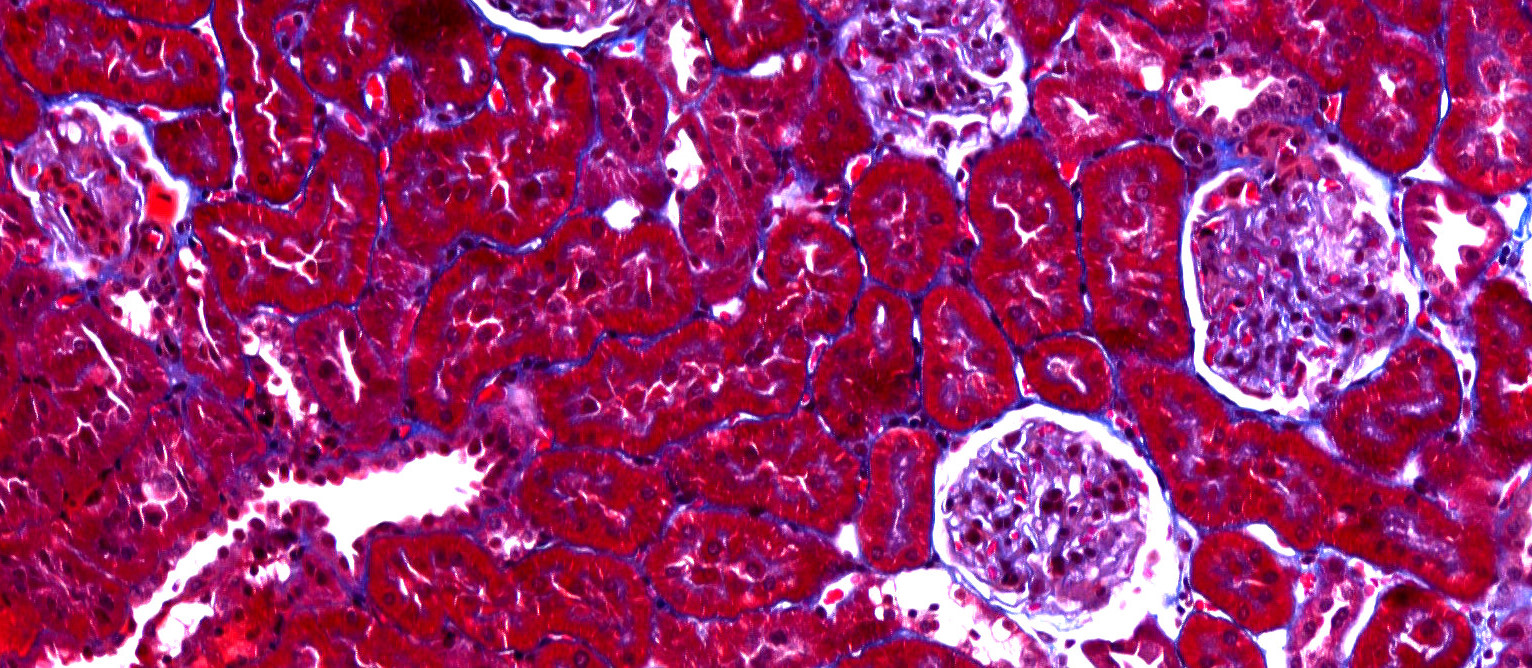

Supplement: Supplementary file 11 [file DataSheet12.ZIP › Fig 1D-masson-TSF-63/63-1.jpeg]

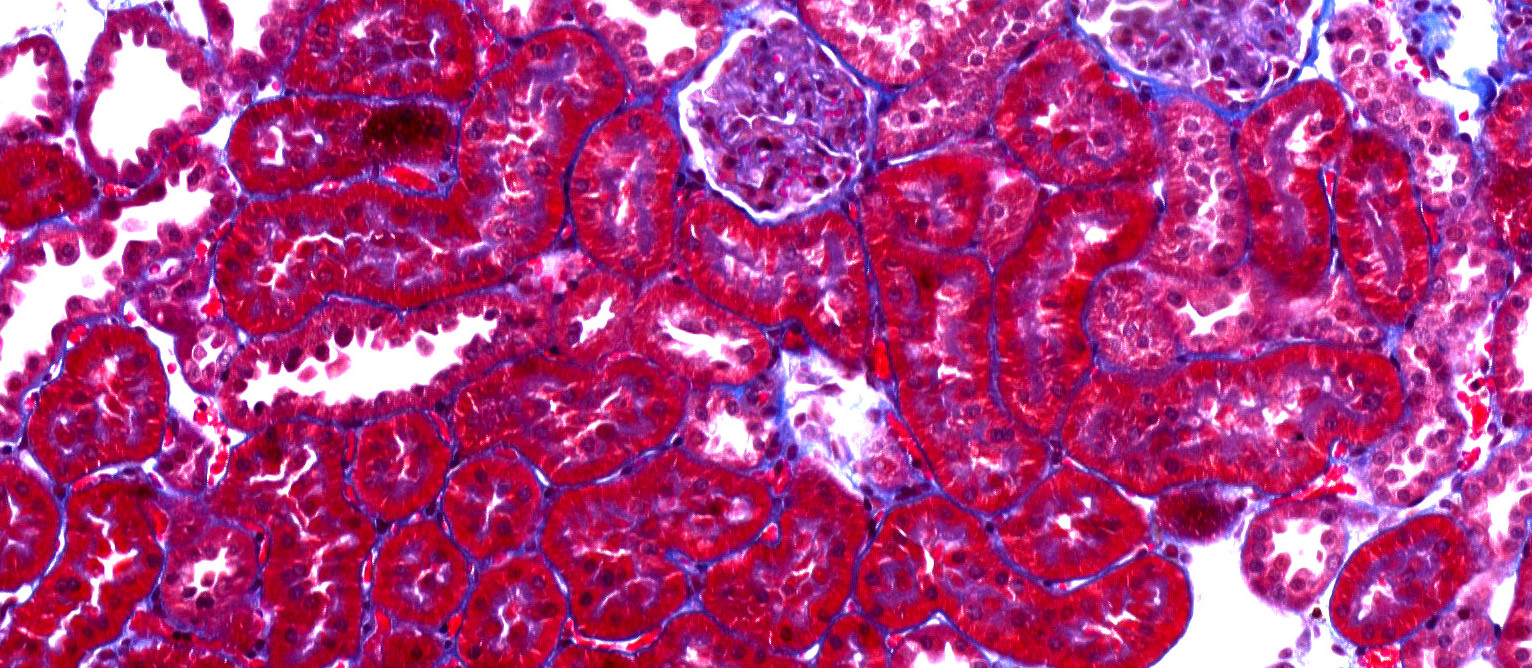

Supplement: Supplementary file 11 [file DataSheet12.ZIP › Fig 1D-masson-TSF-63/63-10.jpeg]

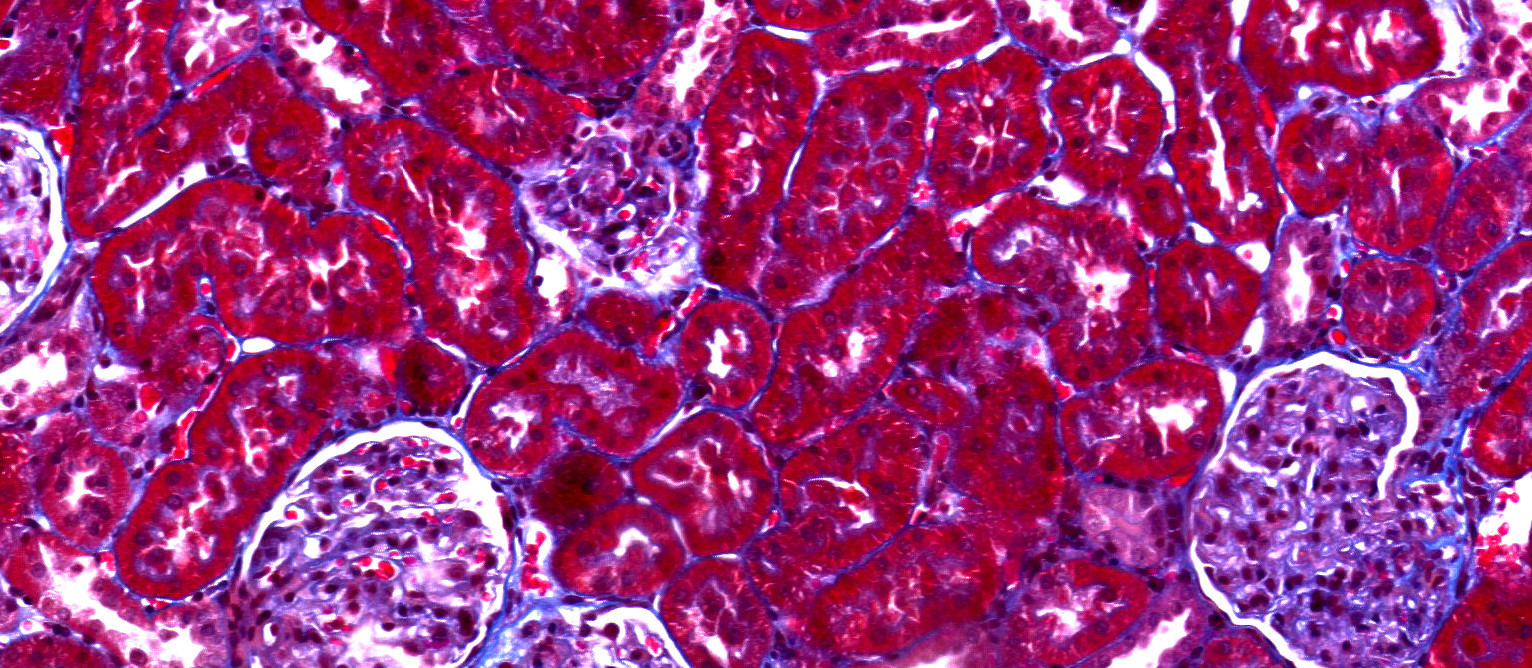

Supplement: Supplementary file 11 [file DataSheet12.ZIP › Fig 1D-masson-TSF-63/63-2.jpeg]

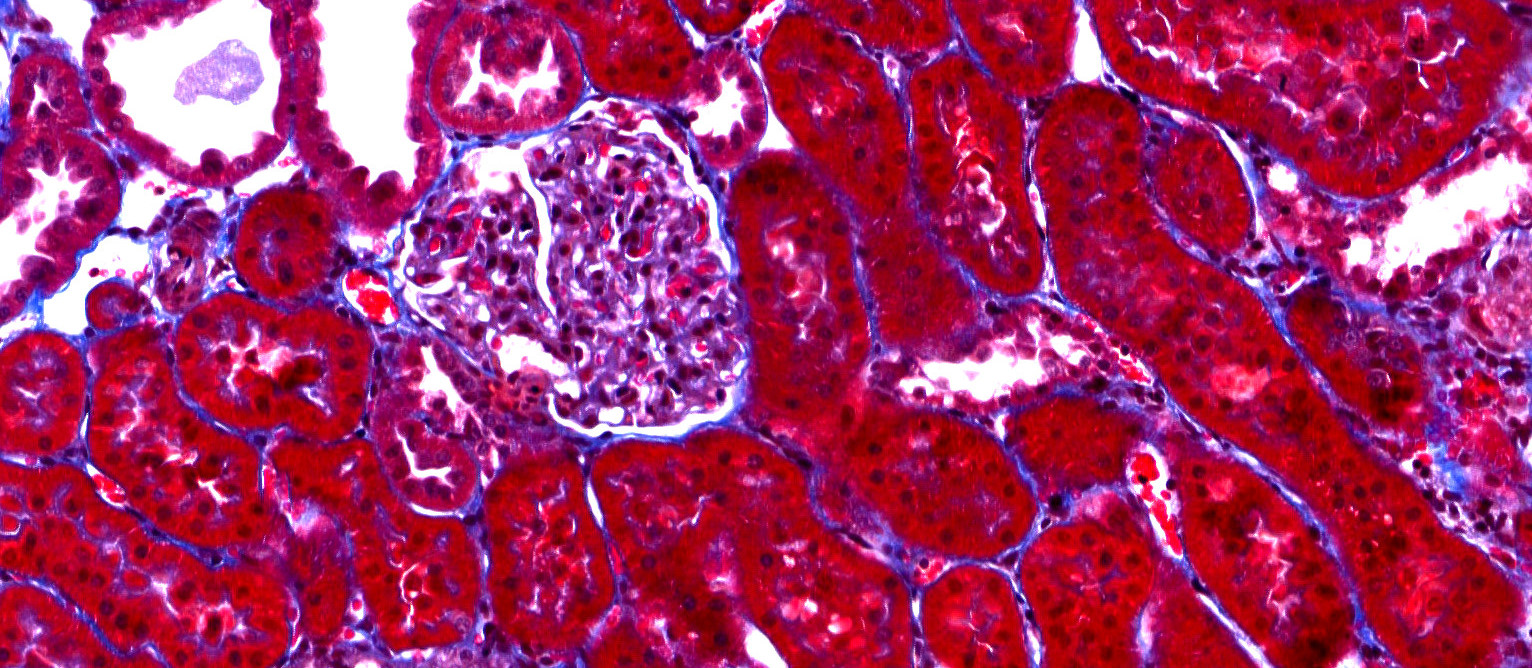

Supplement: Supplementary file 11 [file DataSheet12.ZIP › Fig 1D-masson-TSF-63/63-3.jpeg]

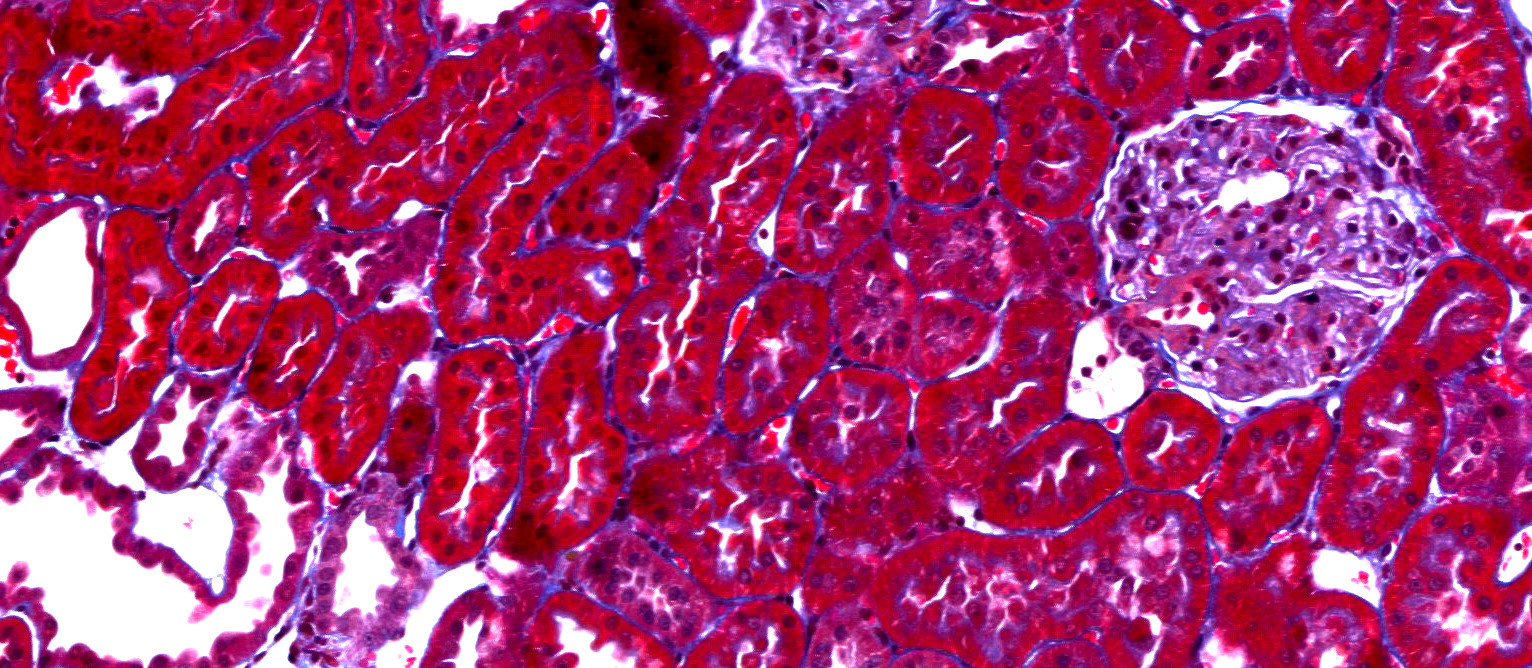

Supplement: Supplementary file 11 [file DataSheet12.ZIP › Fig 1D-masson-TSF-63/63-4.jpeg]

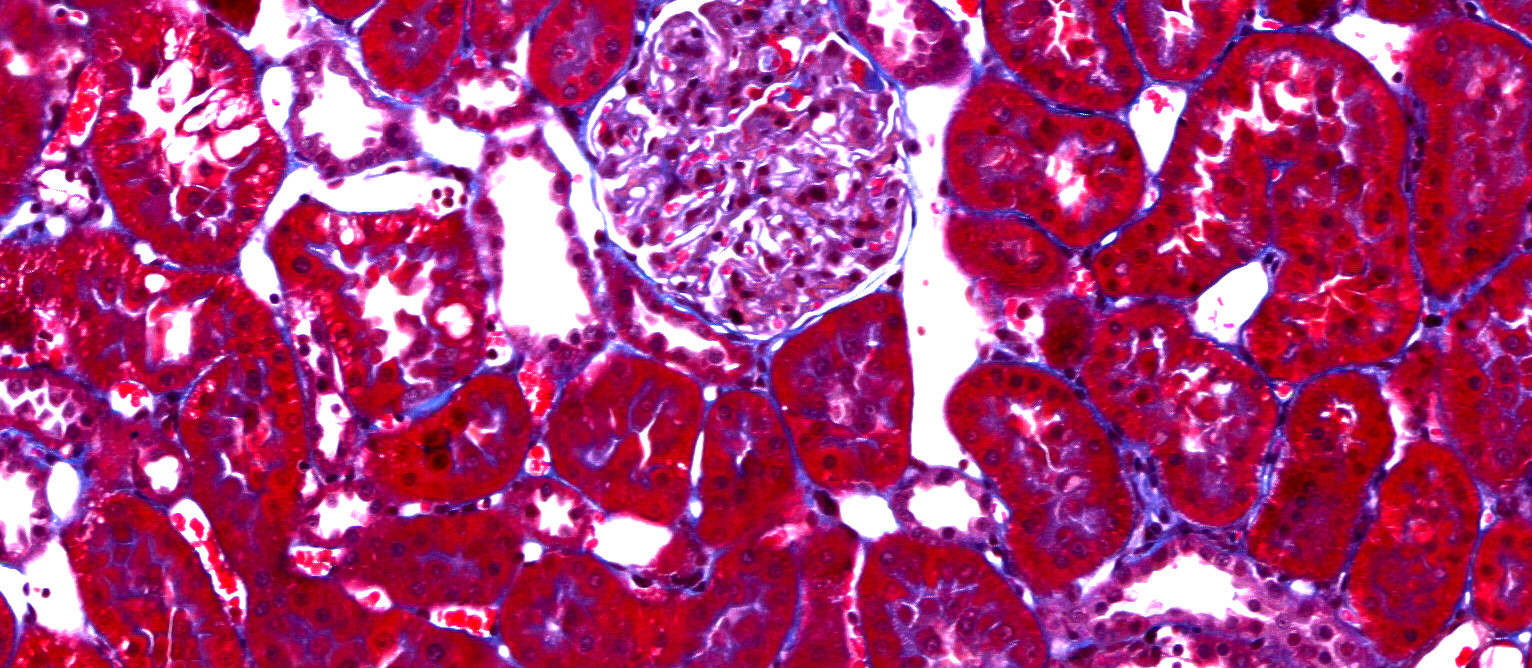

Supplement: Supplementary file 11 [file DataSheet12.ZIP › Fig 1D-masson-TSF-63/63-5.jpeg]

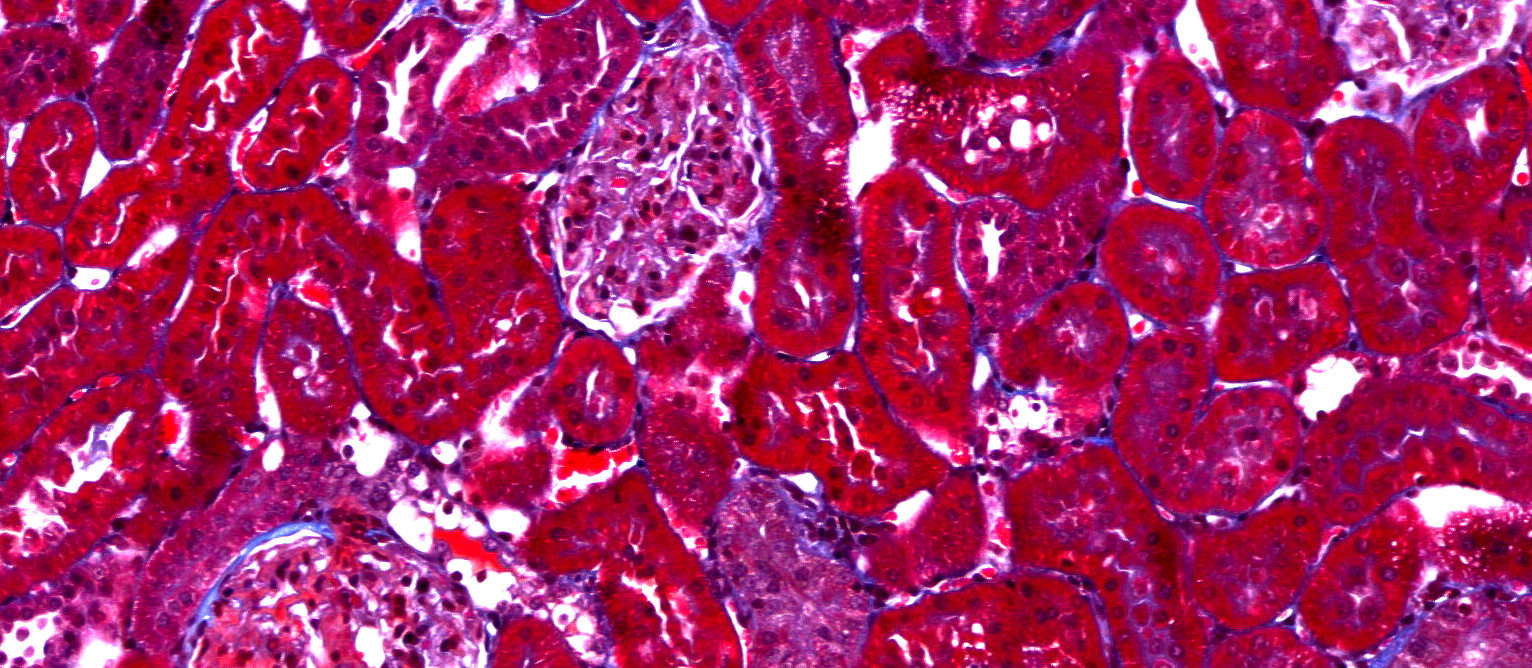

Supplement: Supplementary file 11 [file DataSheet12.ZIP › Fig 1D-masson-TSF-63/63-6.jpeg]

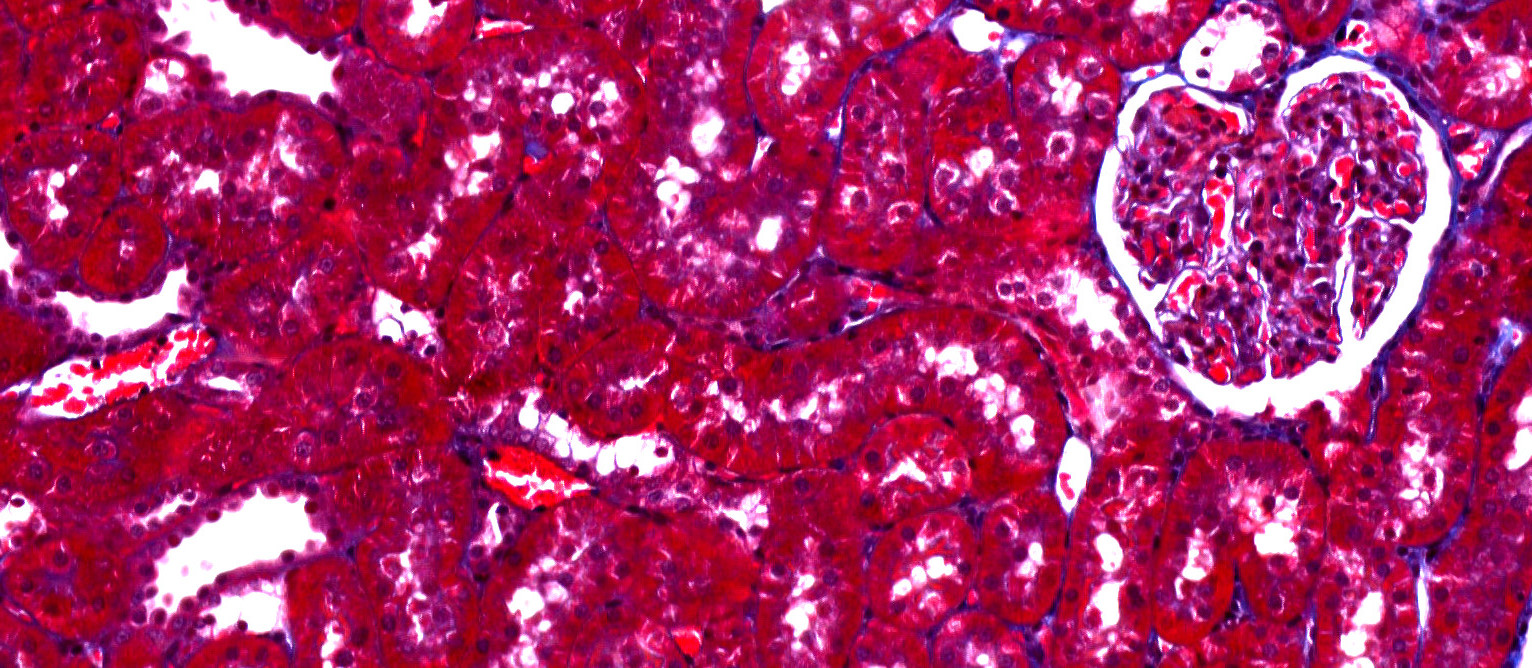

Supplement: Supplementary file 11 [file DataSheet12.ZIP › Fig 1D-masson-TSF-63/63-7.jpeg]

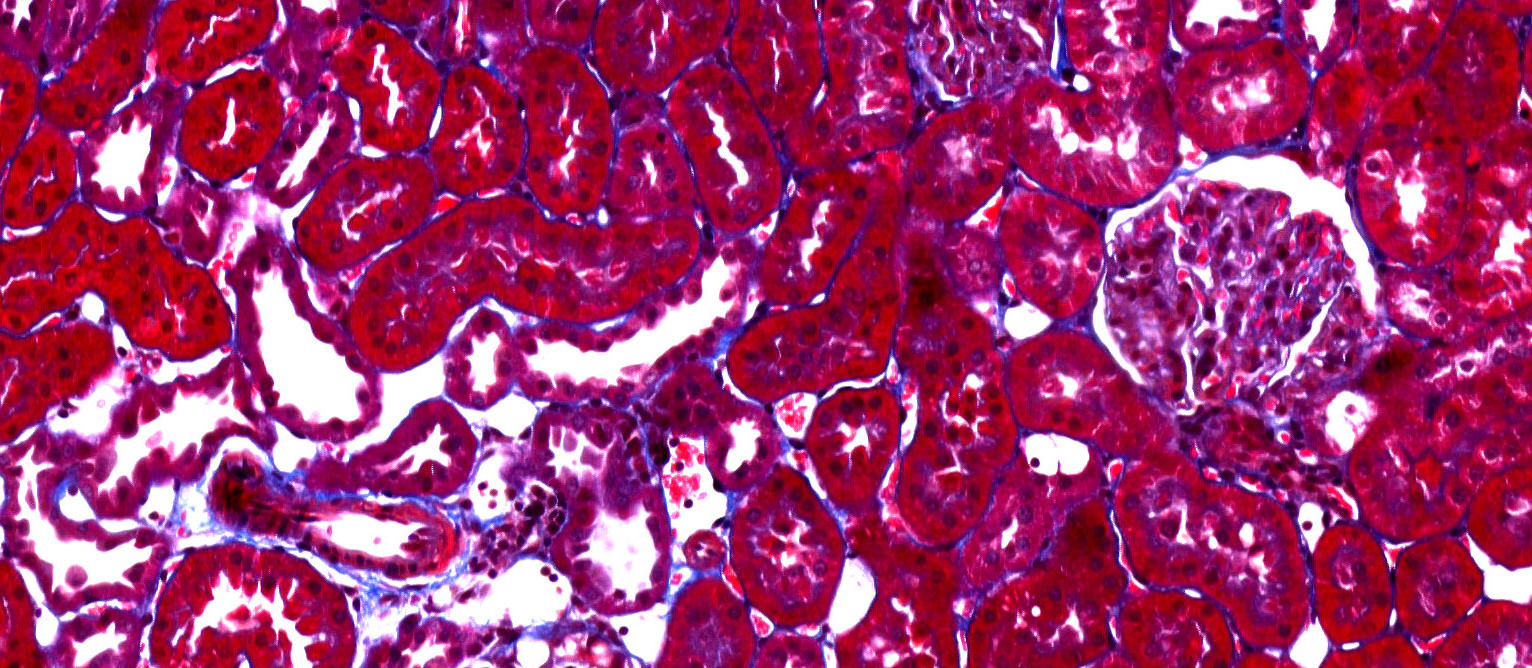

Supplement: Supplementary file 11 [file DataSheet12.ZIP › Fig 1D-masson-TSF-63/63-8.jpeg]

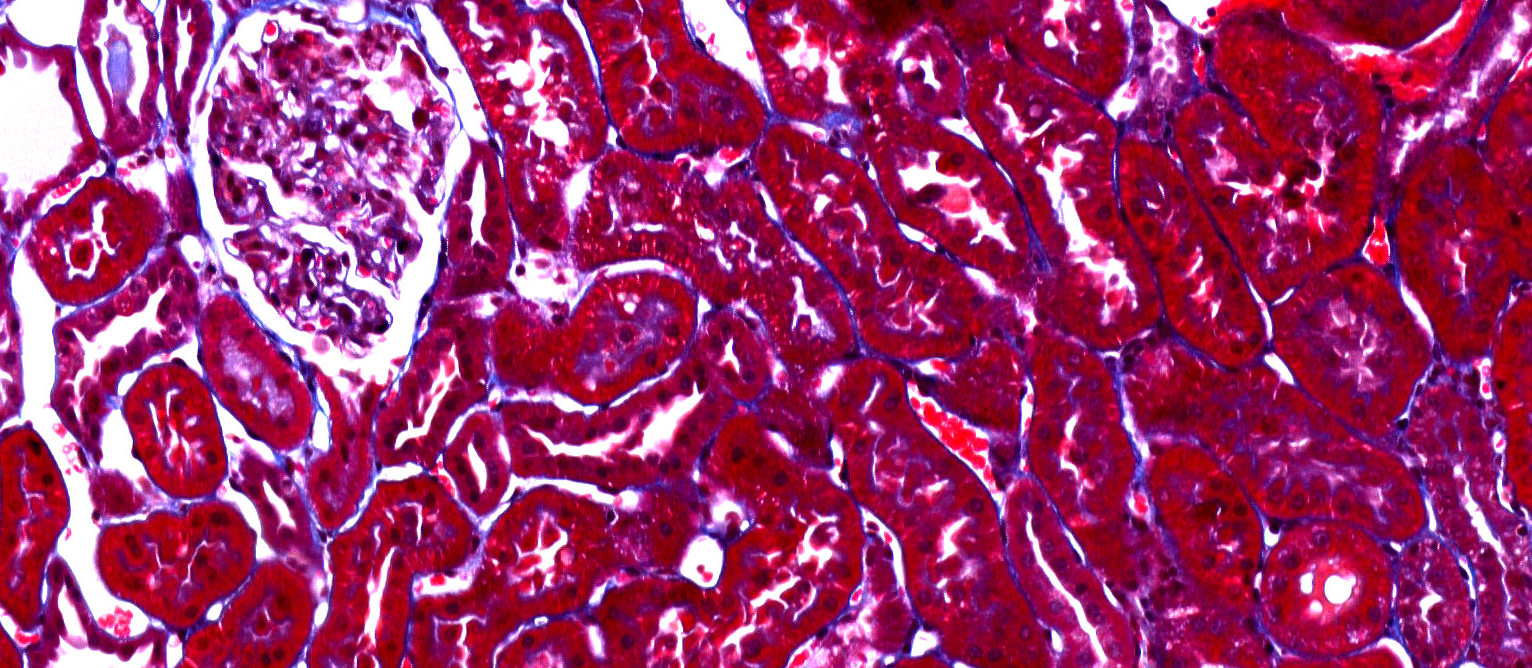

Supplement: Supplementary file 11 [file DataSheet12.ZIP › Fig 1D-masson-TSF-63/63-9.jpeg]

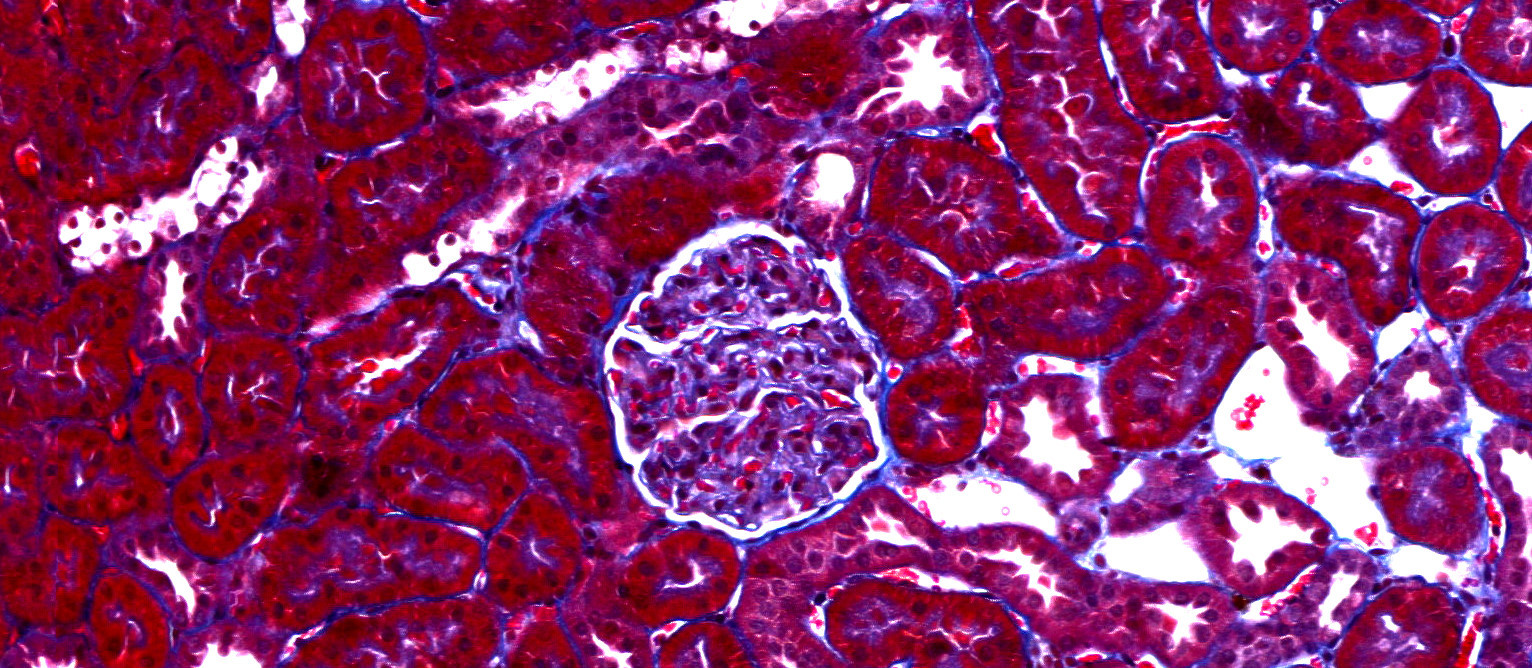

Supplement: Supplementary file 11 [file DataSheet12.ZIP › Fig 1D-masson-TSF-65/65-1.jpeg]

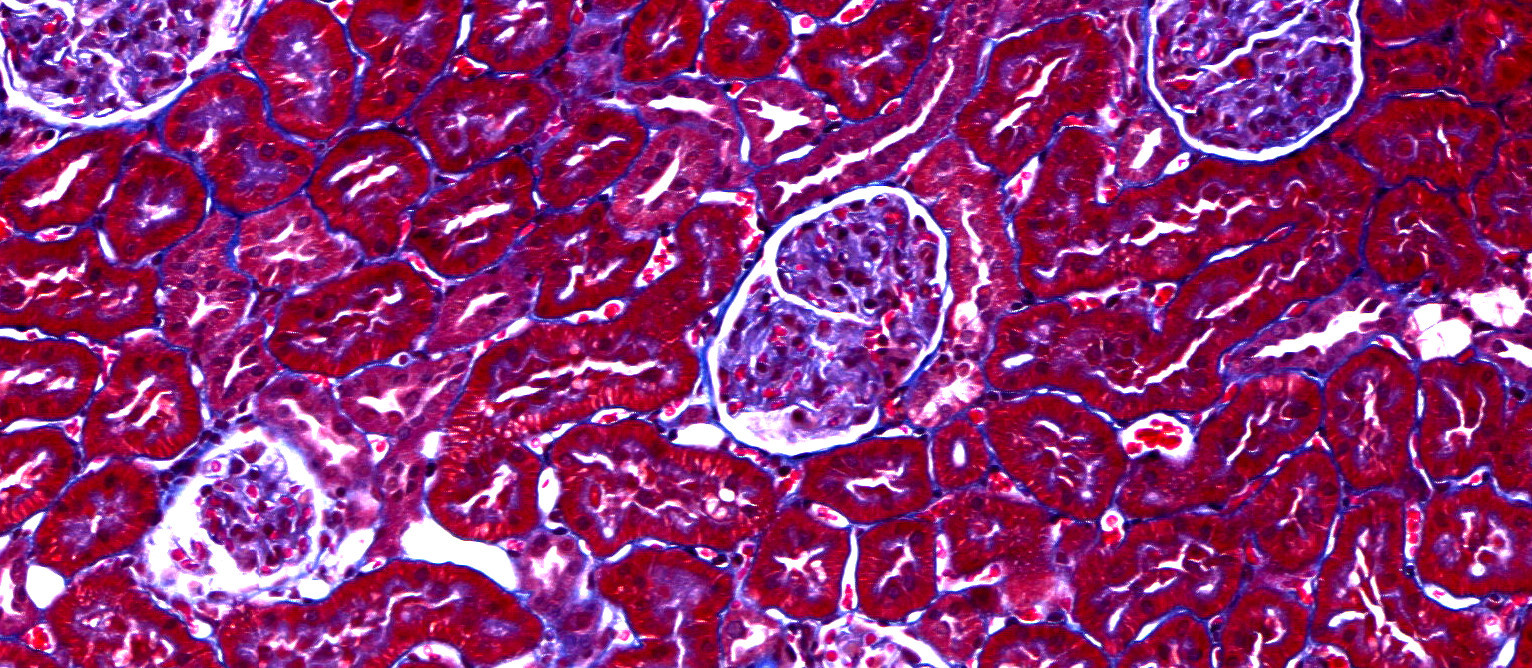

Supplement: Supplementary file 11 [file DataSheet12.ZIP › Fig 1D-masson-TSF-65/65-10.jpeg]

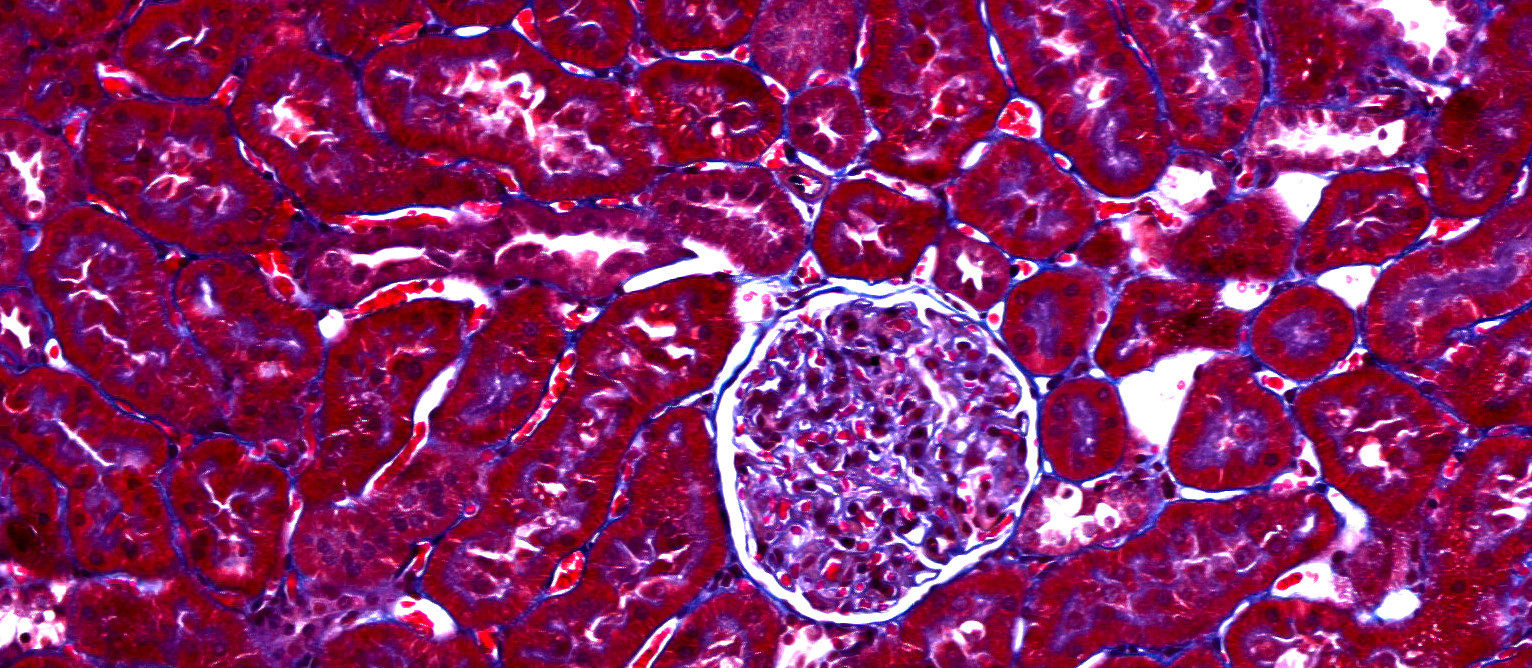

Supplement: Supplementary file 11 [file DataSheet12.ZIP › Fig 1D-masson-TSF-65/65-2.jpeg]

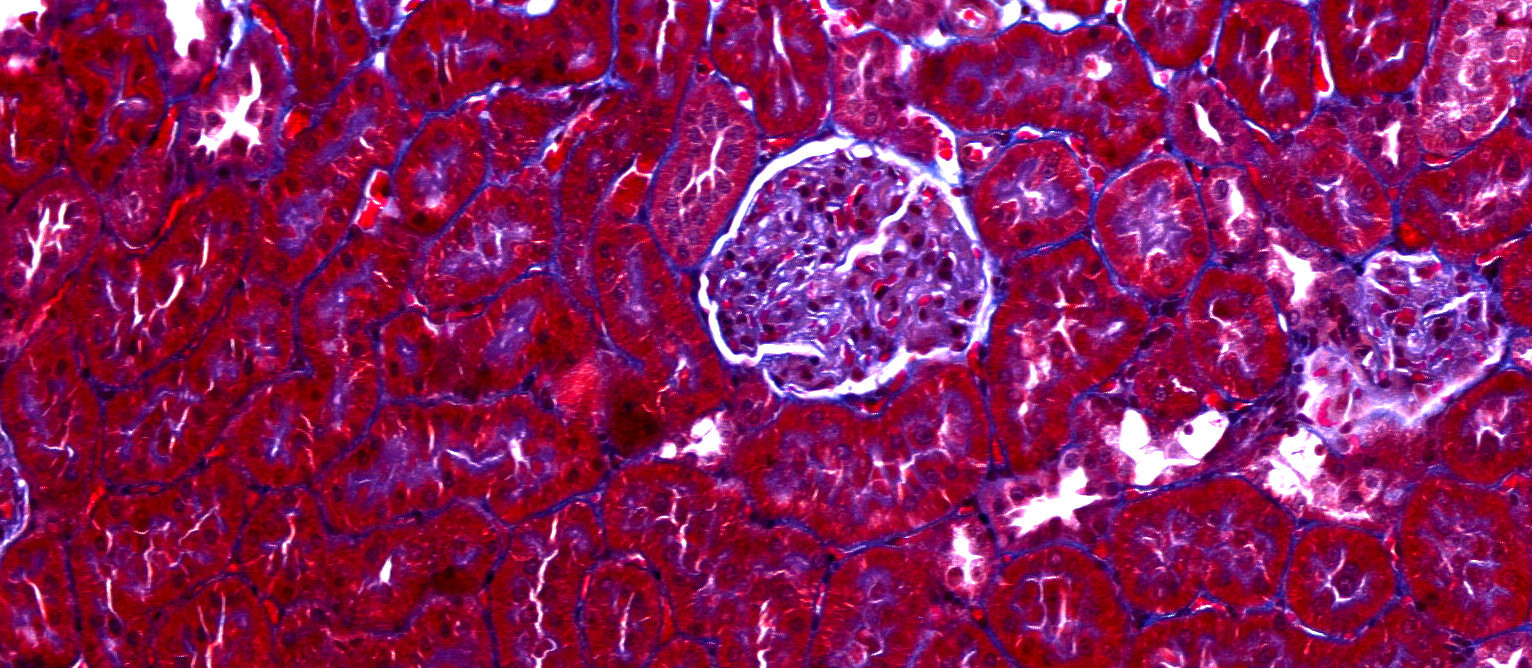

Supplement: Supplementary file 11 [file DataSheet12.ZIP › Fig 1D-masson-TSF-65/65-3.jpeg]

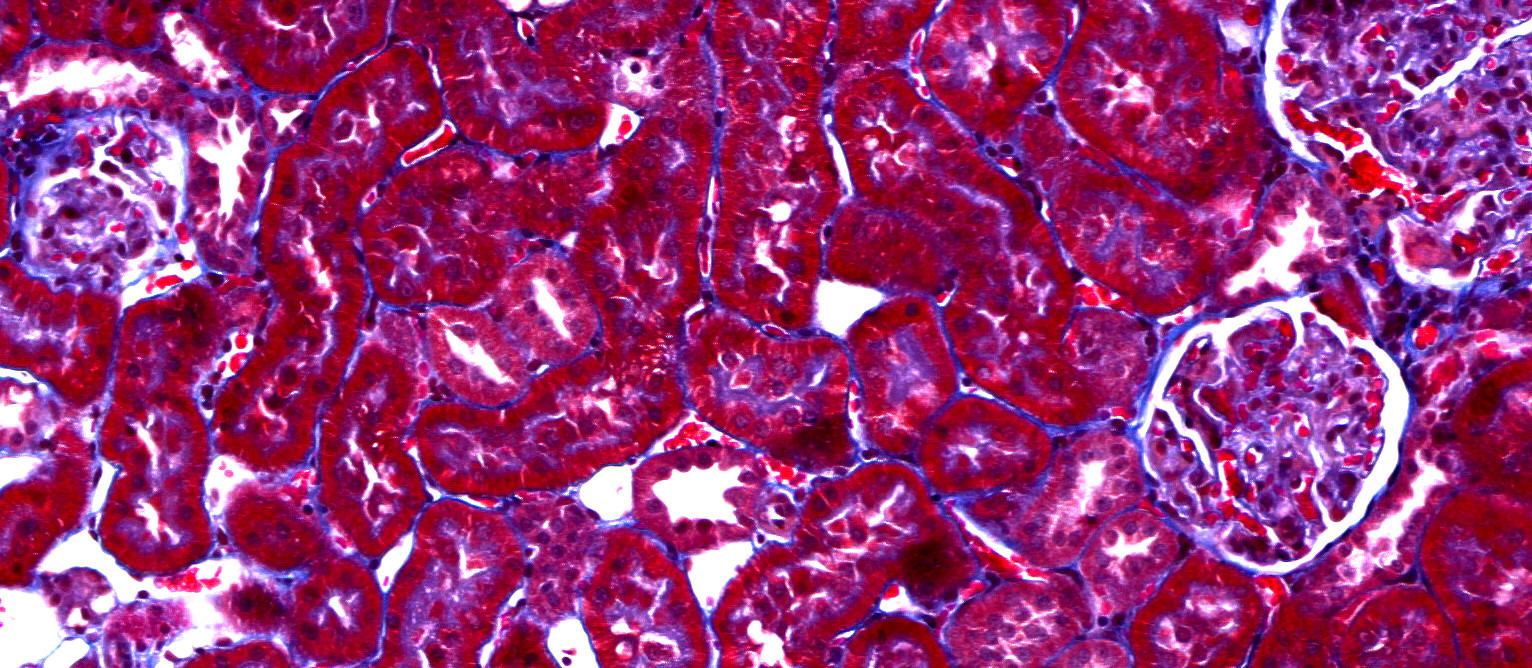

Supplement: Supplementary file 11 [file DataSheet12.ZIP › Fig 1D-masson-TSF-65/65-4.jpeg]

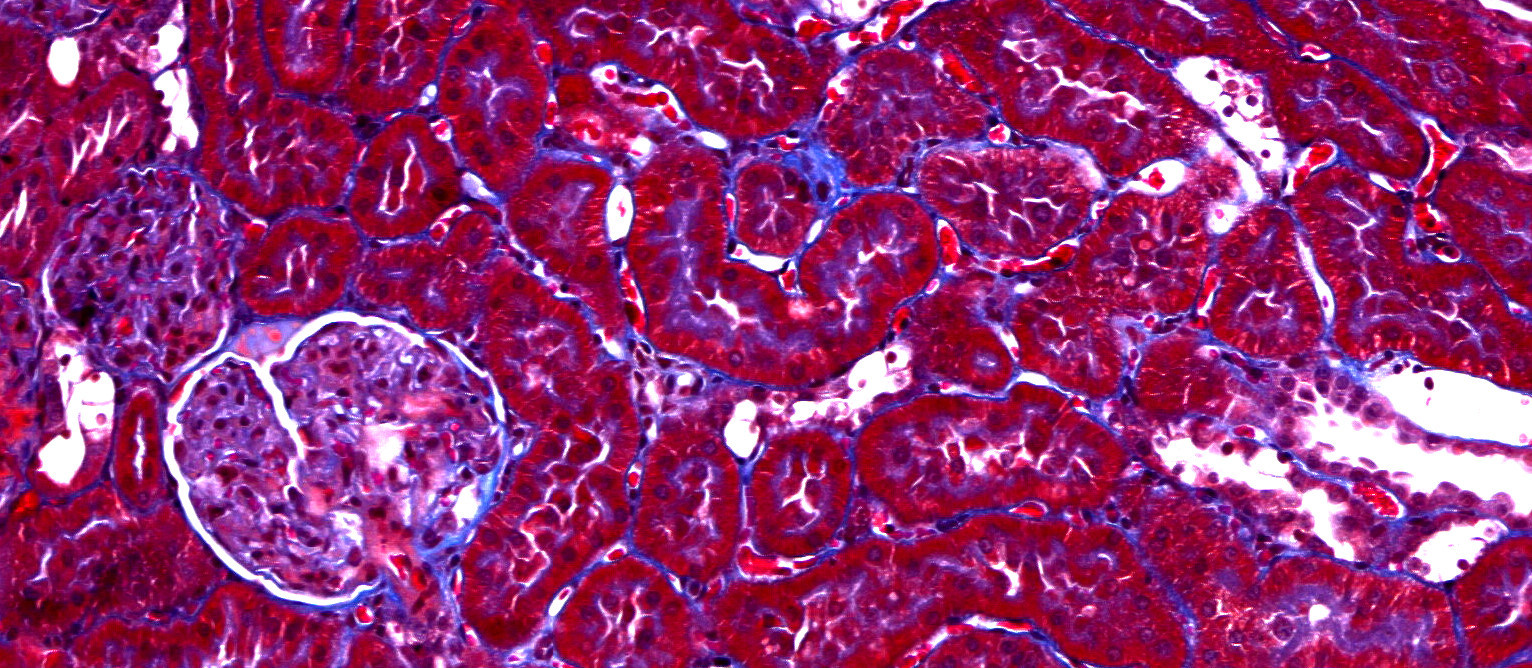

Supplement: Supplementary file 11 [file DataSheet12.ZIP › Fig 1D-masson-TSF-65/65-5.jpeg]

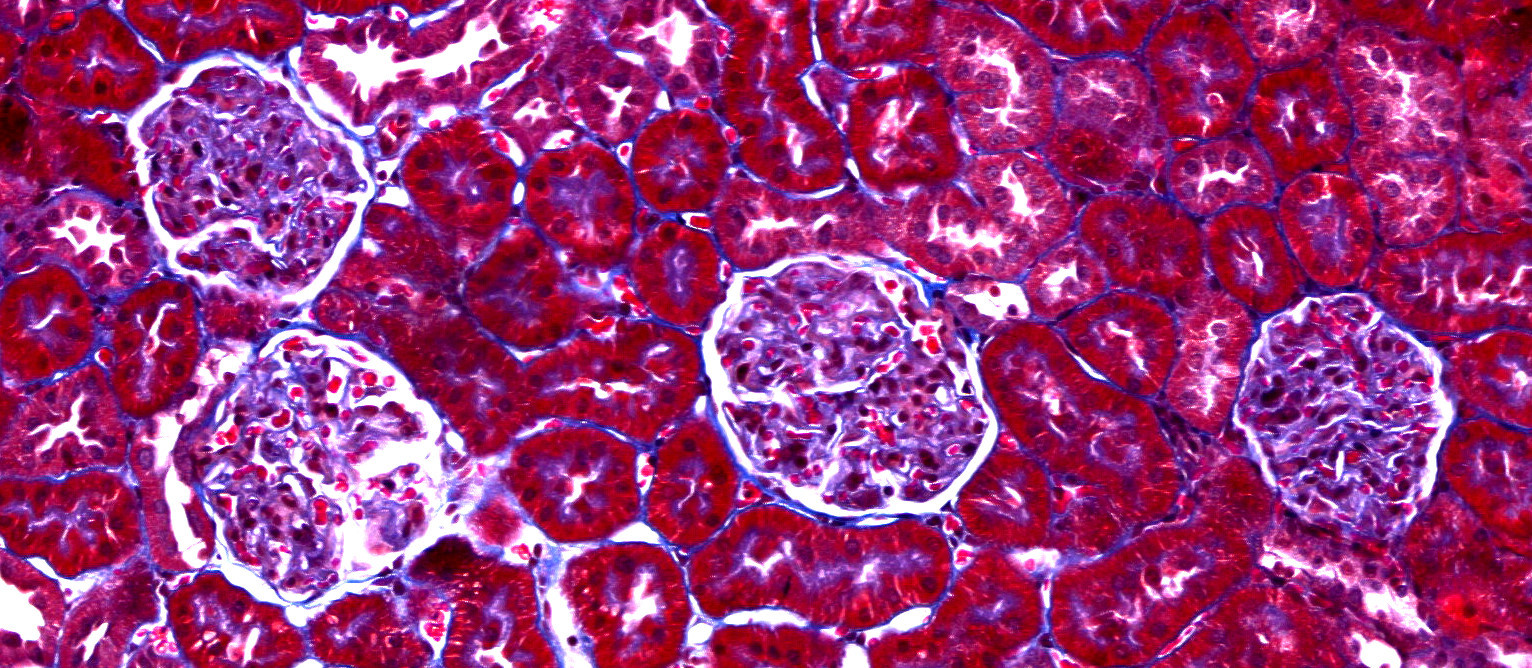

Supplement: Supplementary file 11 [file DataSheet12.ZIP › Fig 1D-masson-TSF-65/65-6.jpeg]

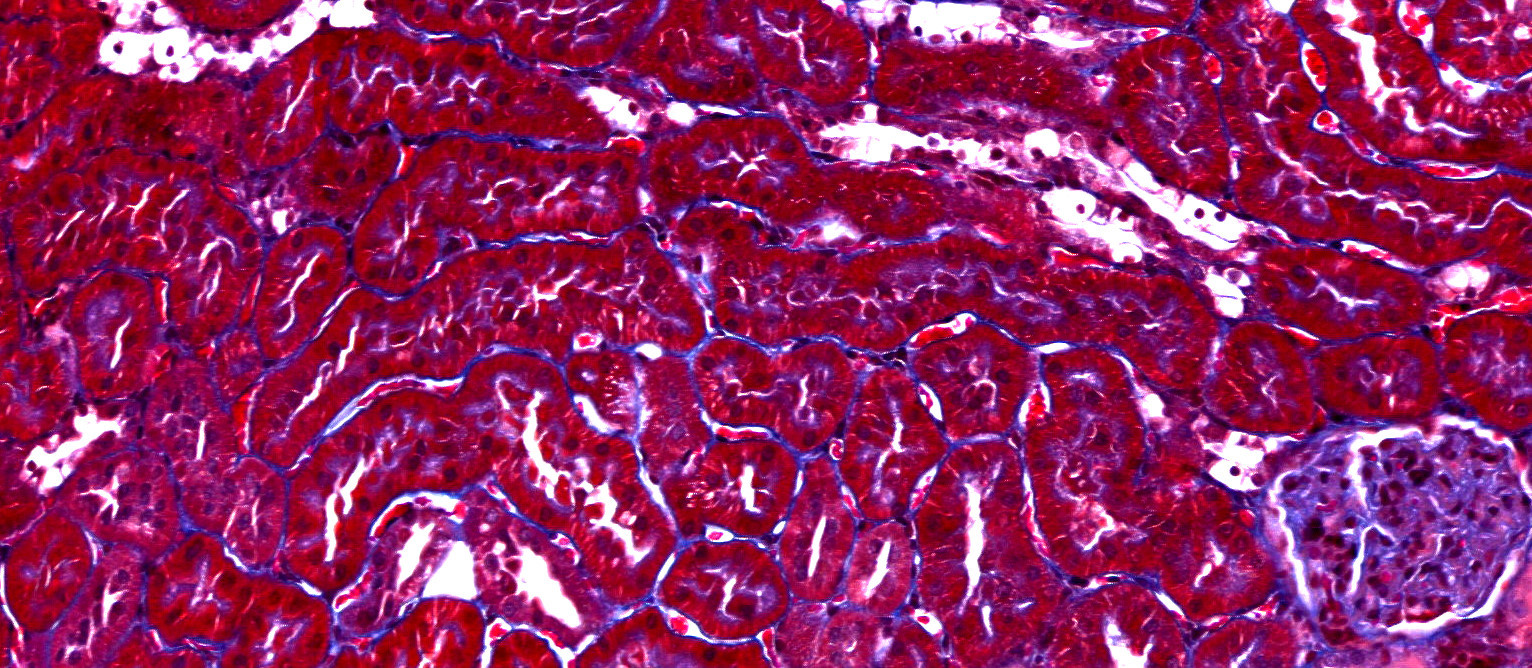

Supplement: Supplementary file 11 [file DataSheet12.ZIP › Fig 1D-masson-TSF-65/65-7.jpeg]

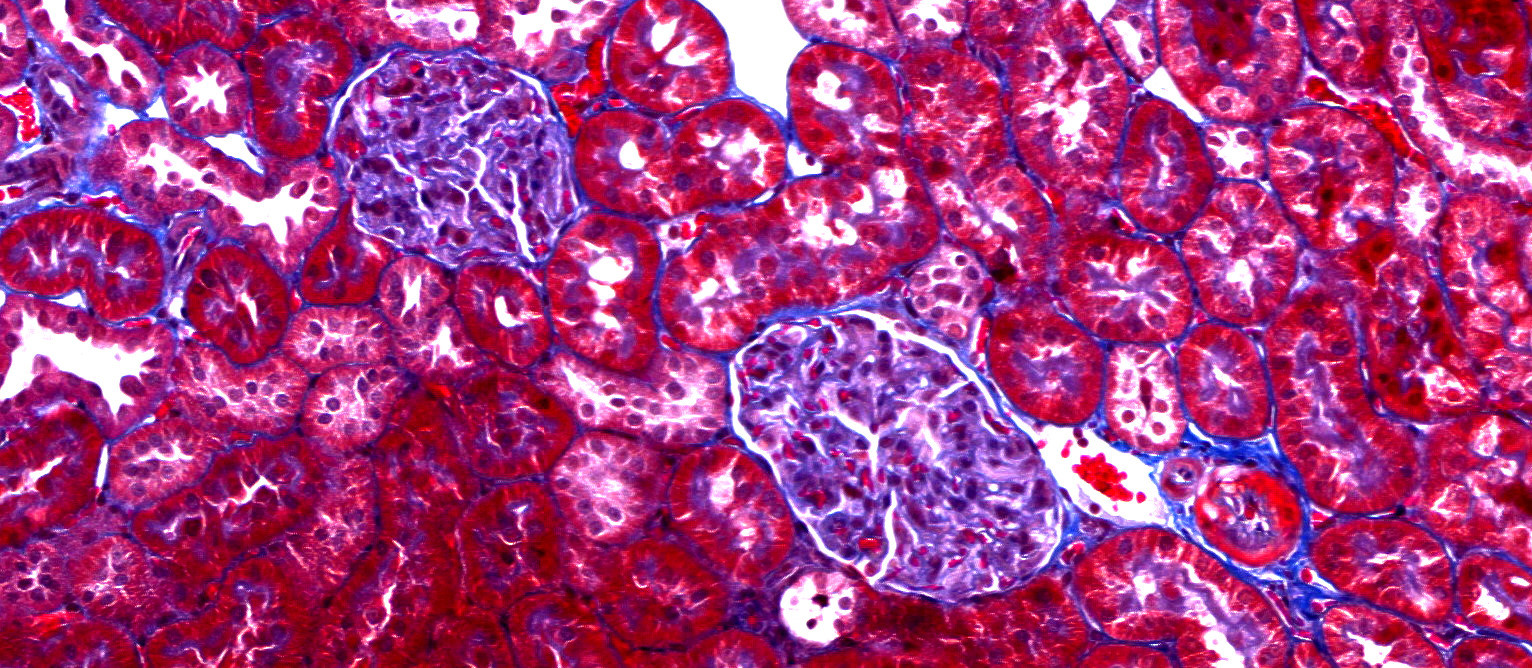

Supplement: Supplementary file 11 [file DataSheet12.ZIP › Fig 1D-masson-TSF-65/65-8.jpeg]

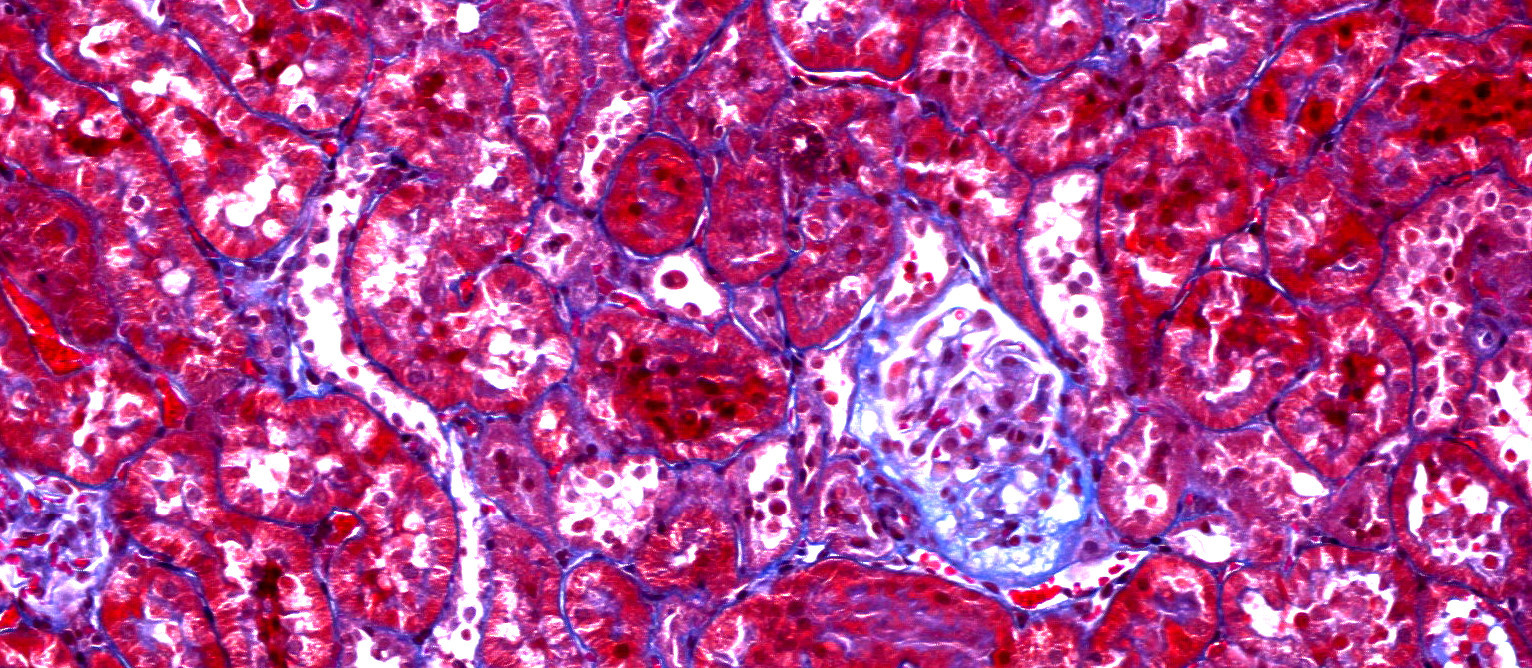

Supplement: Supplementary file 11 [file DataSheet12.ZIP › Fig 1D-masson-TSF-65/65-9.jpeg]

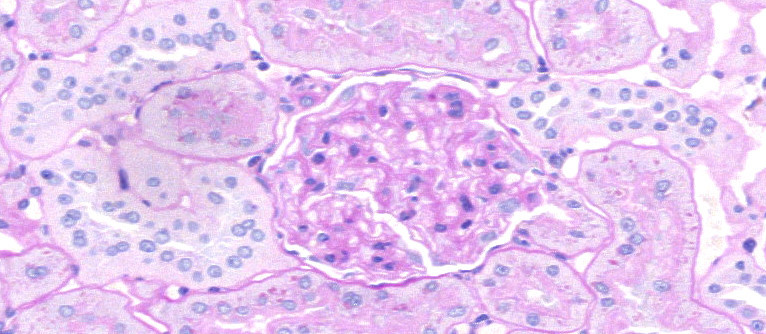

Supplement: Supplementary file 11 [file DataSheet12.ZIP › Fig 1D-PAS-TSF-53/53-1.jpeg]

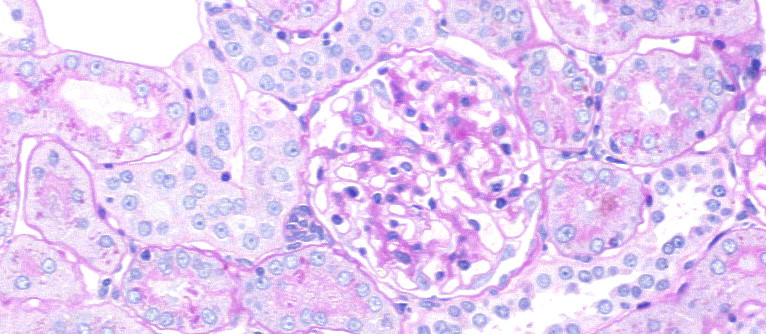

Supplement: Supplementary file 11 [file DataSheet12.ZIP › Fig 1D-PAS-TSF-53/53-10.jpeg]

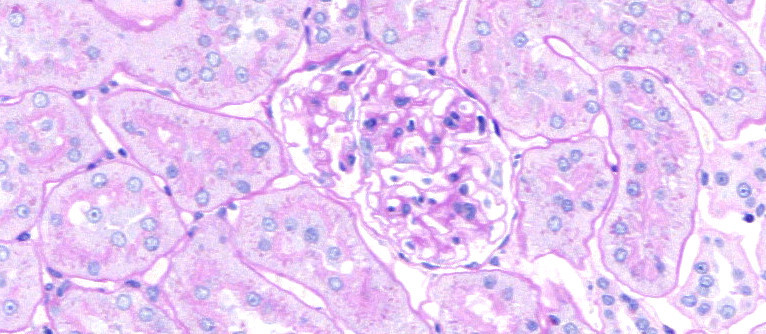

Supplement: Supplementary file 11 [file DataSheet12.ZIP › Fig 1D-PAS-TSF-53/53-11.jpeg]

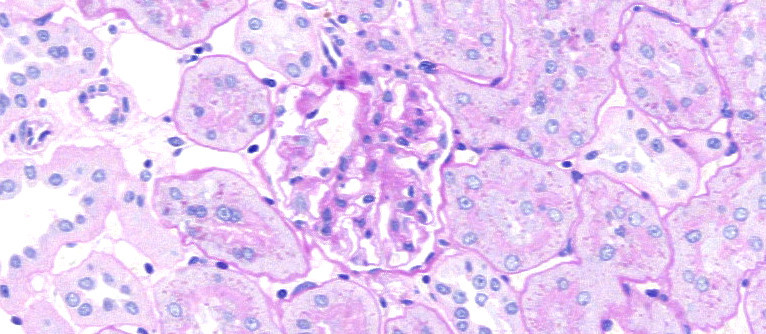

Supplement: Supplementary file 11 [file DataSheet12.ZIP › Fig 1D-PAS-TSF-53/53-12.jpeg]

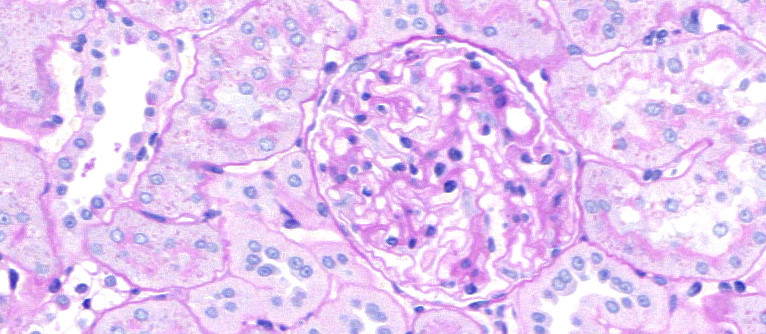

Supplement: Supplementary file 11 [file DataSheet12.ZIP › Fig 1D-PAS-TSF-53/53-13.jpeg]

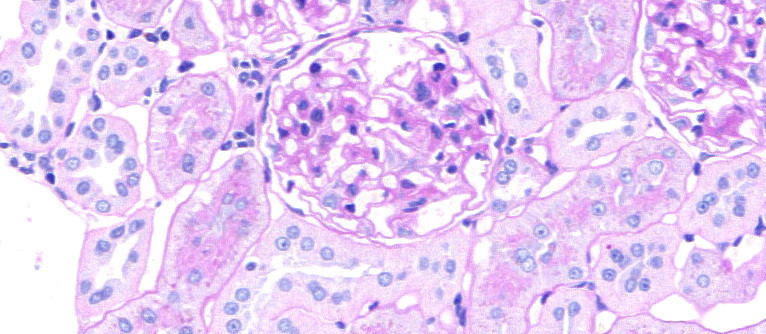

Supplement: Supplementary file 11 [file DataSheet12.ZIP › Fig 1D-PAS-TSF-53/53-14.jpeg]

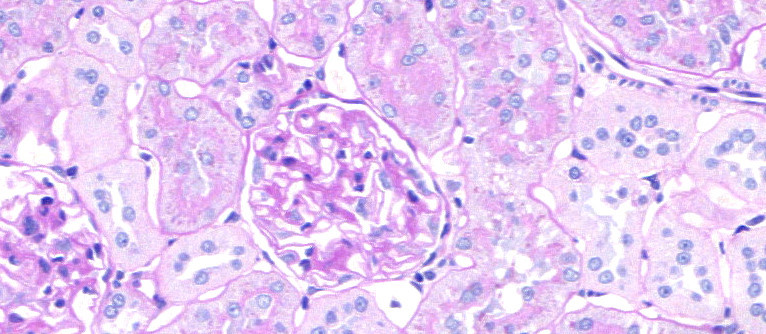

Supplement: Supplementary file 11 [file DataSheet12.ZIP › Fig 1D-PAS-TSF-53/53-15.jpeg]

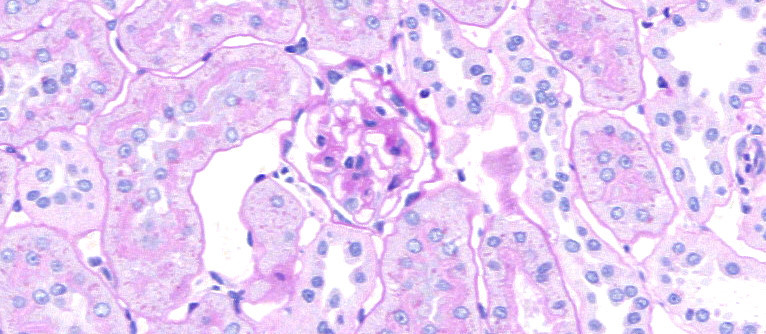

Supplement: Supplementary file 11 [file DataSheet12.ZIP › Fig 1D-PAS-TSF-53/53-16.jpeg]

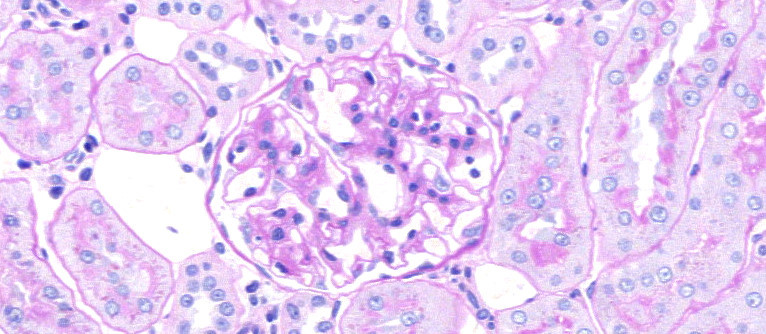

Supplement: Supplementary file 11 [file DataSheet12.ZIP › Fig 1D-PAS-TSF-53/53-17.jpeg]

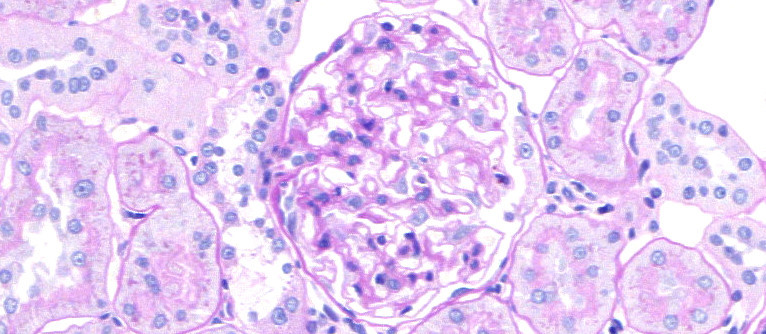

Supplement: Supplementary file 11 [file DataSheet12.ZIP › Fig 1D-PAS-TSF-53/53-18.jpeg]

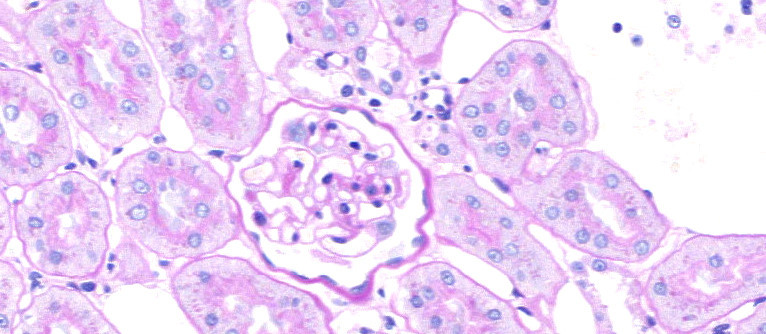

Supplement: Supplementary file 11 [file DataSheet12.ZIP › Fig 1D-PAS-TSF-53/53-19.jpeg]

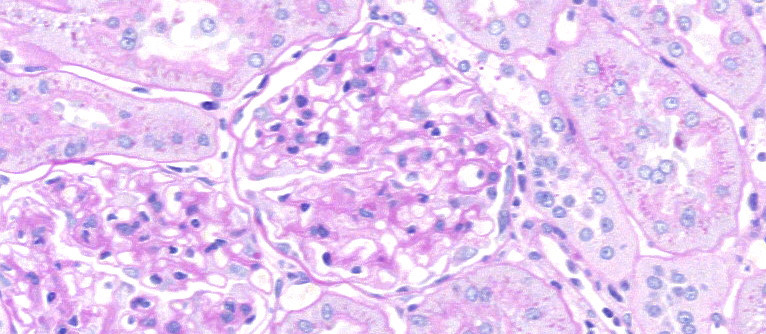

Supplement: Supplementary file 11 [file DataSheet12.ZIP › Fig 1D-PAS-TSF-53/53-2.jpeg]

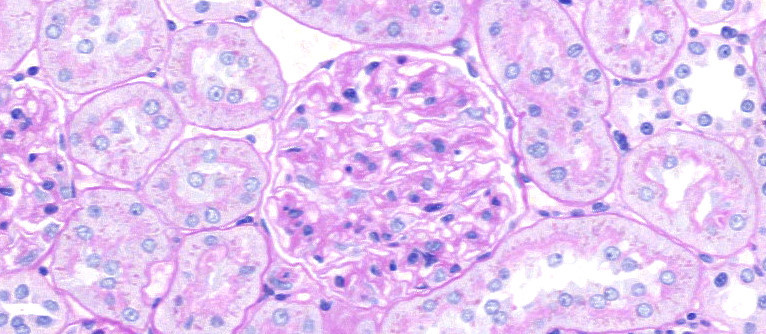

Supplement: Supplementary file 11 [file DataSheet12.ZIP › Fig 1D-PAS-TSF-53/53-20.jpeg]

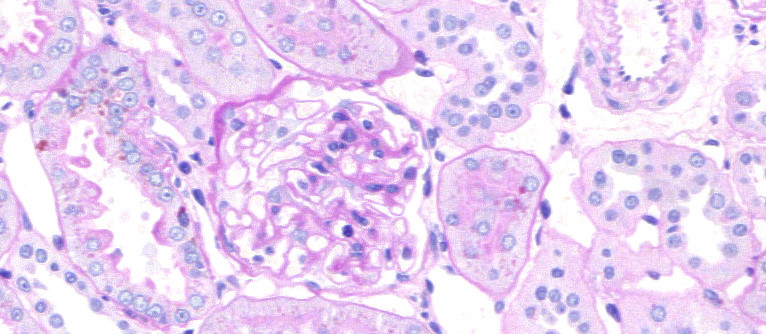

Supplement: Supplementary file 11 [file DataSheet12.ZIP › Fig 1D-PAS-TSF-53/53-3.jpeg]

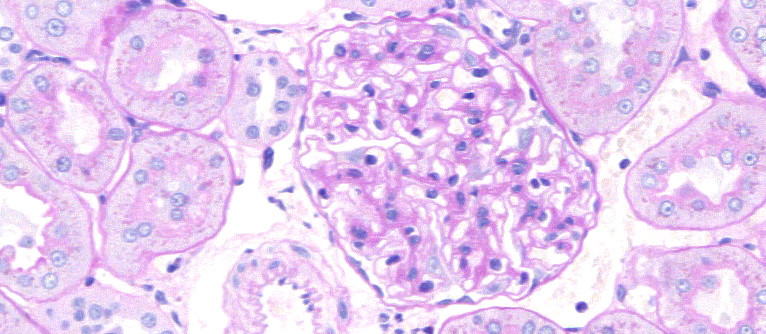

Supplement: Supplementary file 11 [file DataSheet12.ZIP › Fig 1D-PAS-TSF-53/53-4.jpeg]

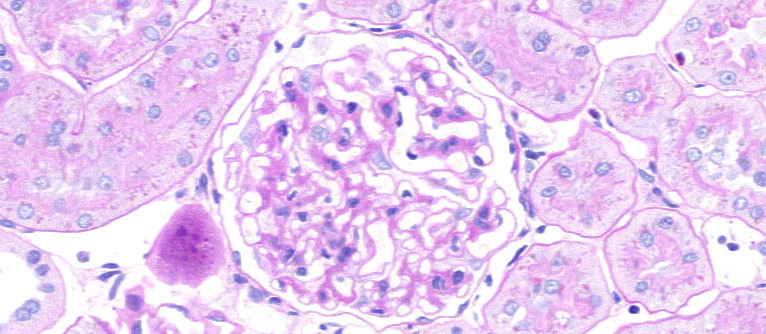

Supplement: Supplementary file 11 [file DataSheet12.ZIP › Fig 1D-PAS-TSF-53/53-5.jpeg]

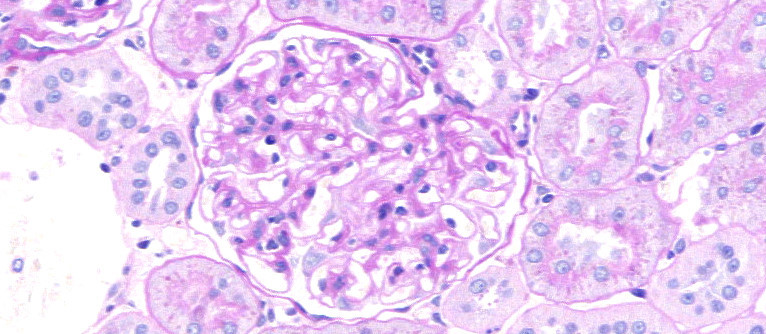

Supplement: Supplementary file 11 [file DataSheet12.ZIP › Fig 1D-PAS-TSF-53/53-6.jpeg]

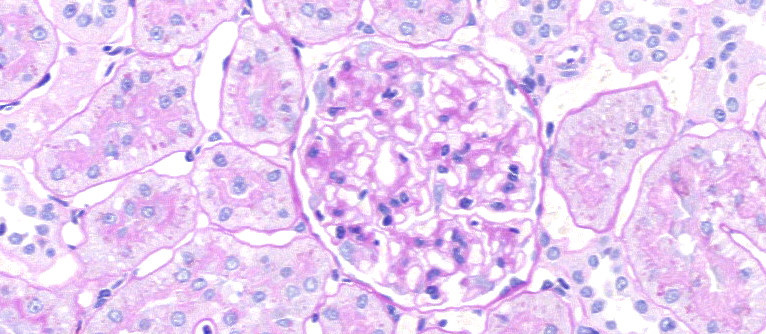

Supplement: Supplementary file 11 [file DataSheet12.ZIP › Fig 1D-PAS-TSF-53/53-7.jpeg]

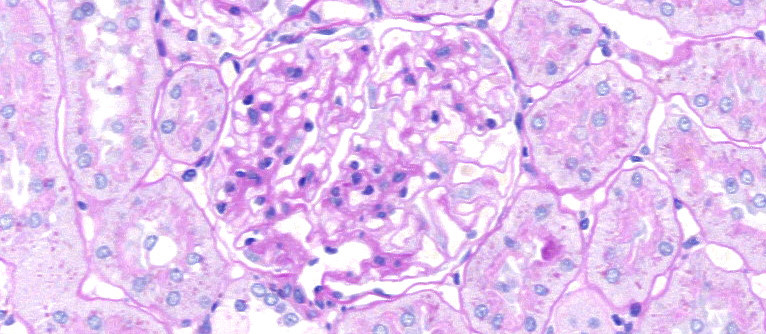

Supplement: Supplementary file 11 [file DataSheet12.ZIP › Fig 1D-PAS-TSF-53/53-8.jpeg]

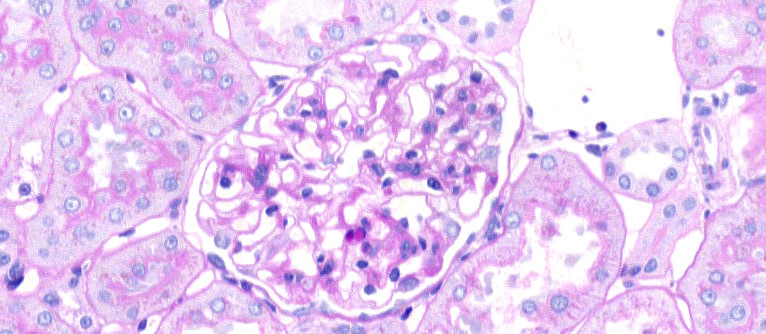

Supplement: Supplementary file 11 [file DataSheet12.ZIP › Fig 1D-PAS-TSF-53/53-9.jpeg]

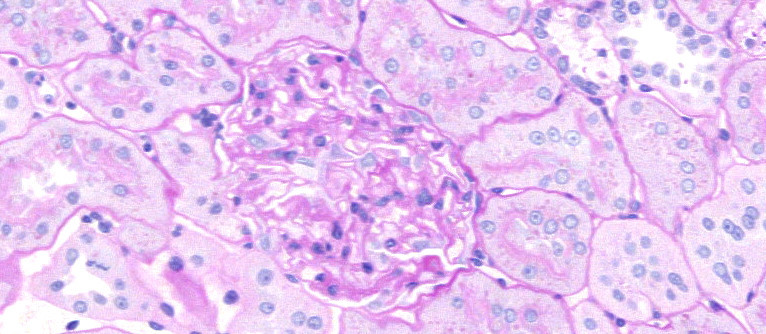

Supplement: Supplementary file 11 [file DataSheet12.ZIP › Fig 1D-PAS-TSF-56/56-1.jpeg]

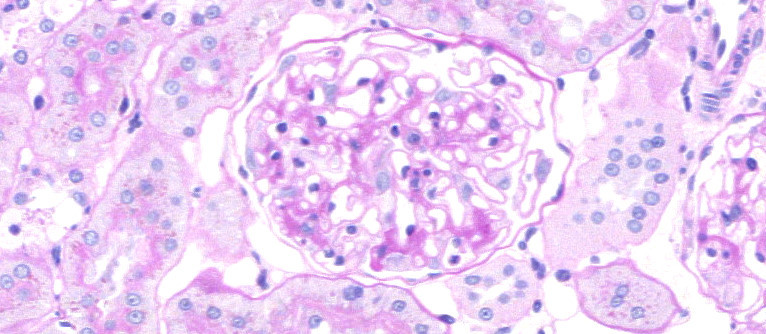

Supplement: Supplementary file 11 [file DataSheet12.ZIP › Fig 1D-PAS-TSF-56/56-10.jpeg]

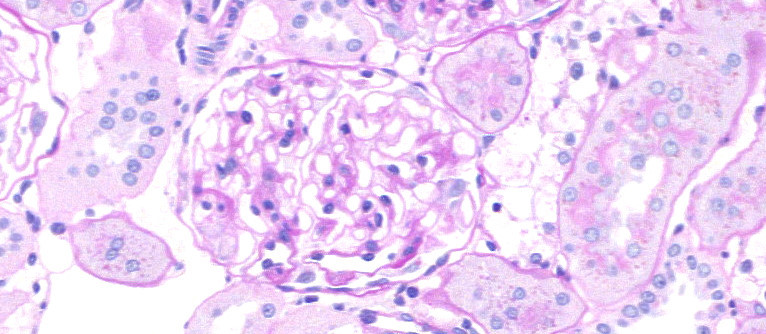

Supplement: Supplementary file 11 [file DataSheet12.ZIP › Fig 1D-PAS-TSF-56/56-11.jpeg]

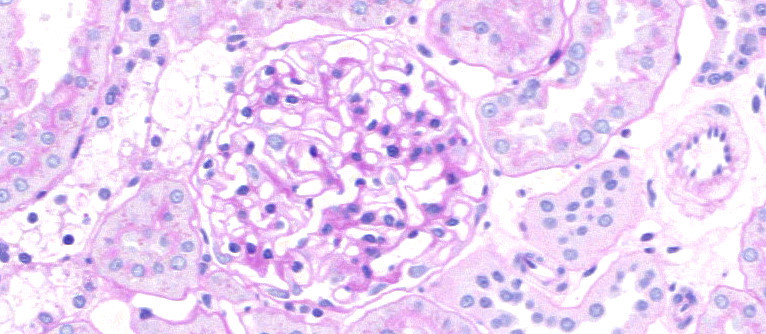

Supplement: Supplementary file 11 [file DataSheet12.ZIP › Fig 1D-PAS-TSF-56/56-12.jpeg]

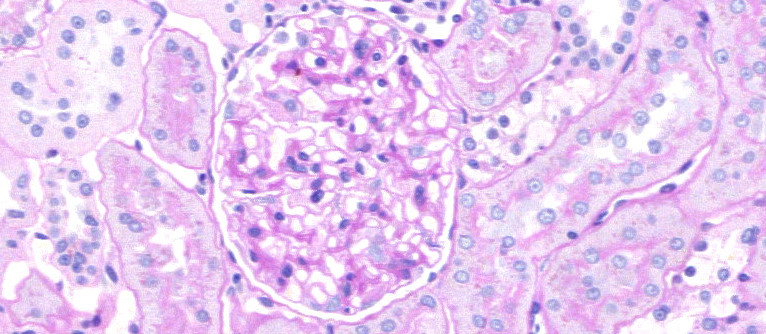

Supplement: Supplementary file 11 [file DataSheet12.ZIP › Fig 1D-PAS-TSF-56/56-13.jpeg]

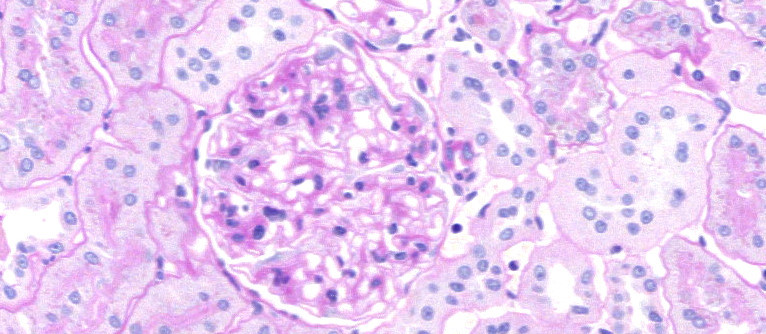

Supplement: Supplementary file 11 [file DataSheet12.ZIP › Fig 1D-PAS-TSF-56/56-14.jpeg]

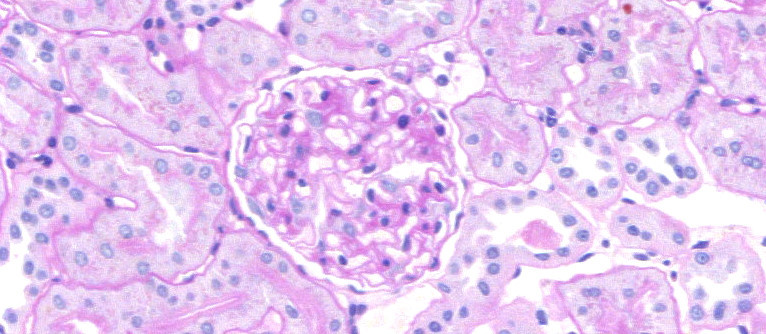

Supplement: Supplementary file 11 [file DataSheet12.ZIP › Fig 1D-PAS-TSF-56/56-15.jpeg]

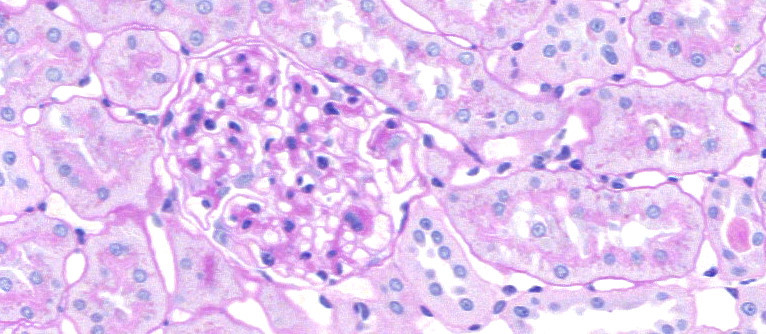

Supplement: Supplementary file 11 [file DataSheet12.ZIP › Fig 1D-PAS-TSF-56/56-16.jpeg]

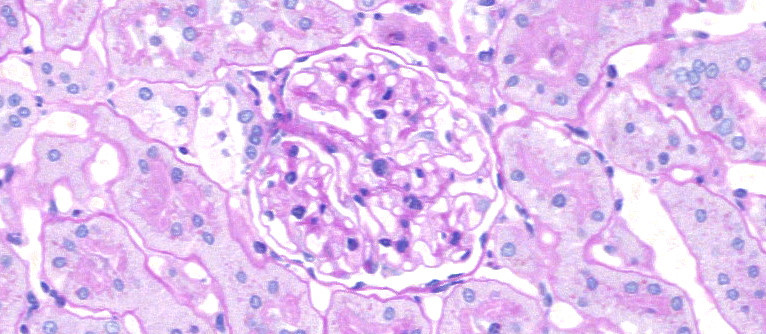

Supplement: Supplementary file 11 [file DataSheet12.ZIP › Fig 1D-PAS-TSF-56/56-17.jpeg]

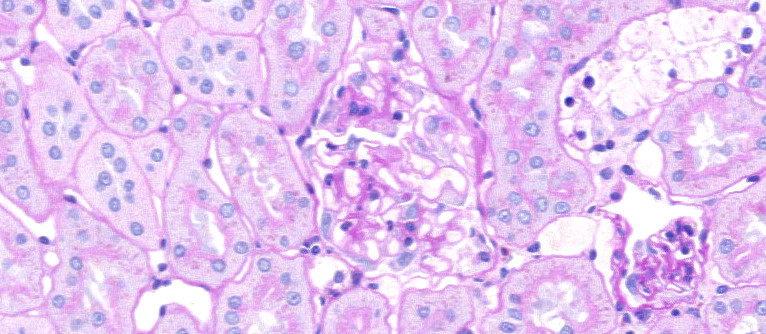

Supplement: Supplementary file 11 [file DataSheet12.ZIP › Fig 1D-PAS-TSF-56/56-18.jpeg]

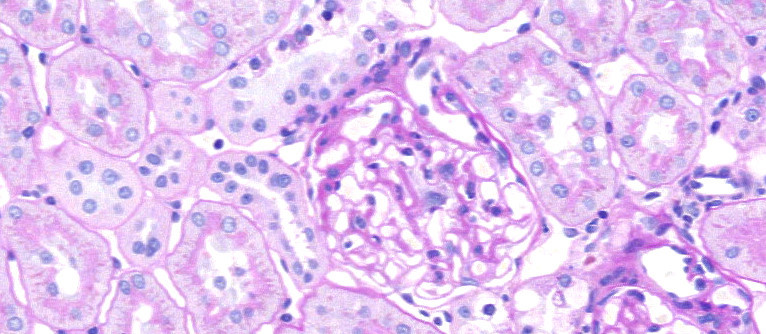

Supplement: Supplementary file 11 [file DataSheet12.ZIP › Fig 1D-PAS-TSF-56/56-19.jpeg]

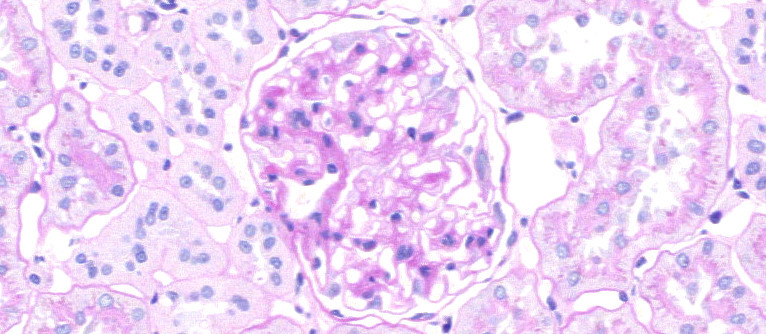

Supplement: Supplementary file 11 [file DataSheet12.ZIP › Fig 1D-PAS-TSF-56/56-2.jpeg]

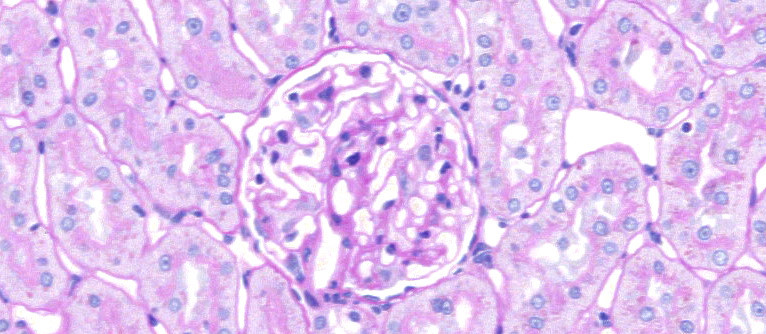

Supplement: Supplementary file 11 [file DataSheet12.ZIP › Fig 1D-PAS-TSF-56/56-20.jpeg]

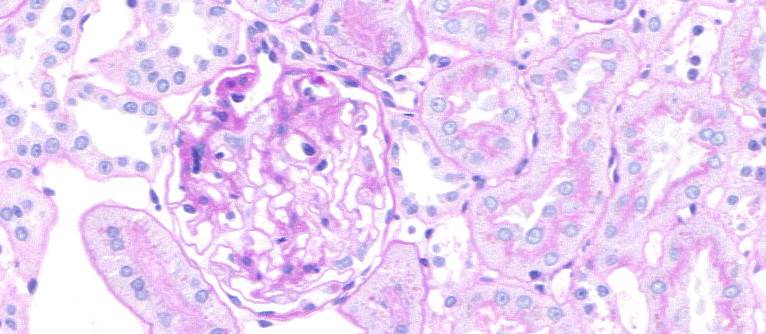

Supplement: Supplementary file 11 [file DataSheet12.ZIP › Fig 1D-PAS-TSF-56/56-3.jpeg]

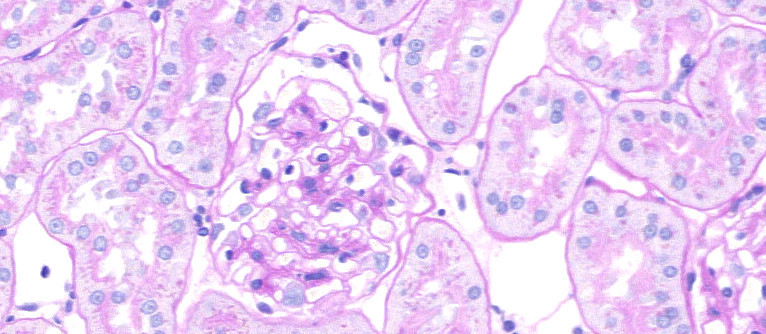

Supplement: Supplementary file 11 [file DataSheet12.ZIP › Fig 1D-PAS-TSF-56/56-4.jpeg]

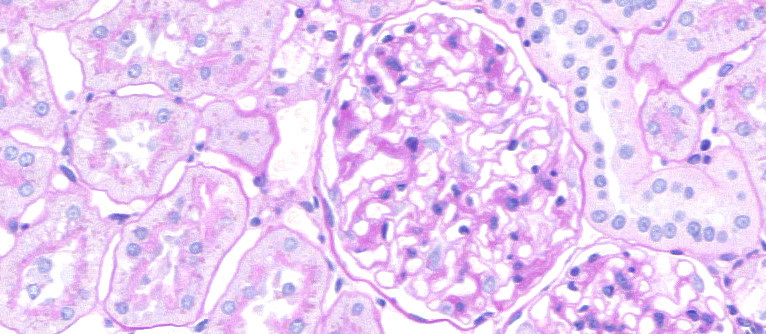

Supplement: Supplementary file 11 [file DataSheet12.ZIP › Fig 1D-PAS-TSF-56/56-5.jpeg]

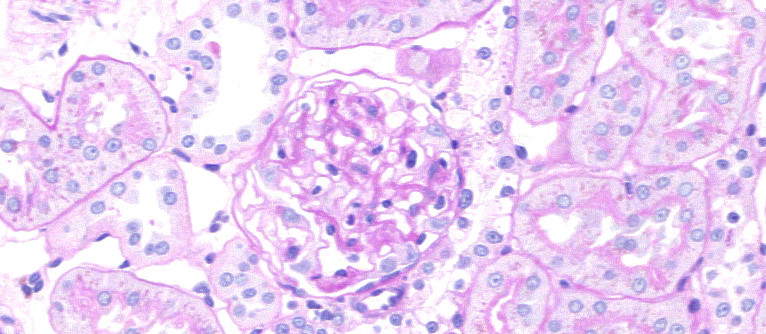

Supplement: Supplementary file 11 [file DataSheet12.ZIP › Fig 1D-PAS-TSF-56/56-6.jpeg]

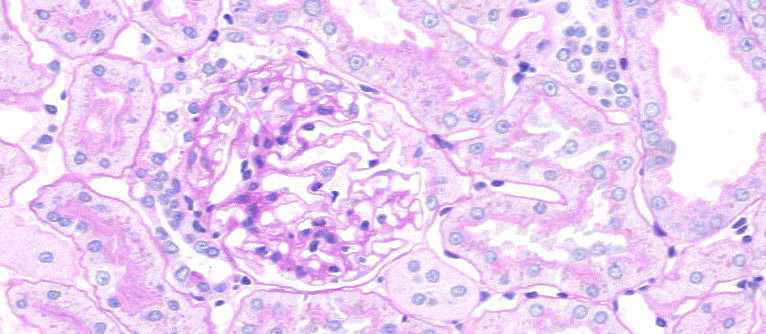

Supplement: Supplementary file 11 [file DataSheet12.ZIP › Fig 1D-PAS-TSF-56/56-7.jpeg]

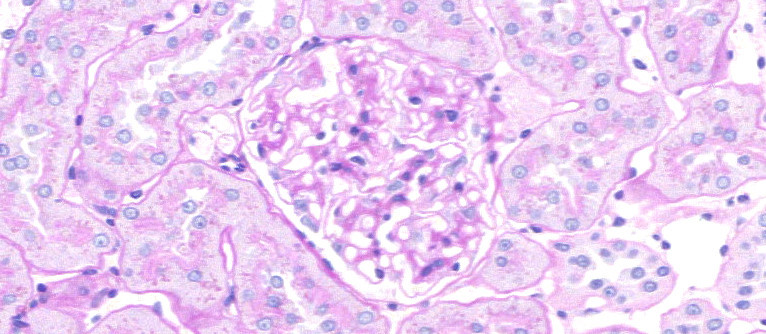

Supplement: Supplementary file 11 [file DataSheet12.ZIP › Fig 1D-PAS-TSF-56/56-8.jpeg]

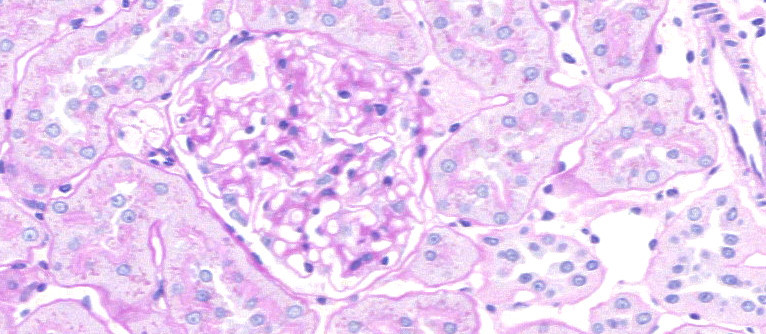

Supplement: Supplementary file 11 [file DataSheet12.ZIP › Fig 1D-PAS-TSF-56/56-9.jpeg]

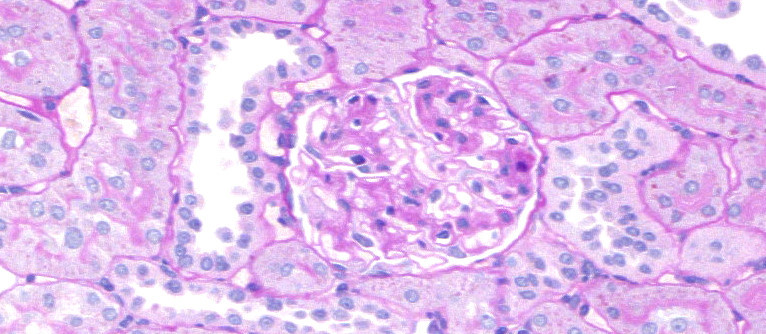

Supplement: Supplementary file 11 [file DataSheet12.ZIP › Fig 1D-PAS-TSF-57/57-1.jpeg]

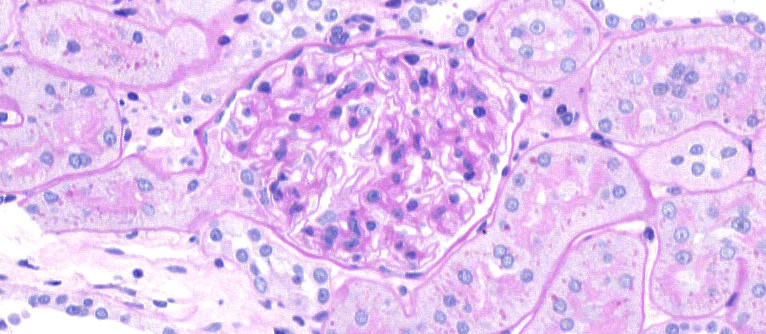

Supplement: Supplementary file 11 [file DataSheet12.ZIP › Fig 1D-PAS-TSF-57/57-10.jpeg]

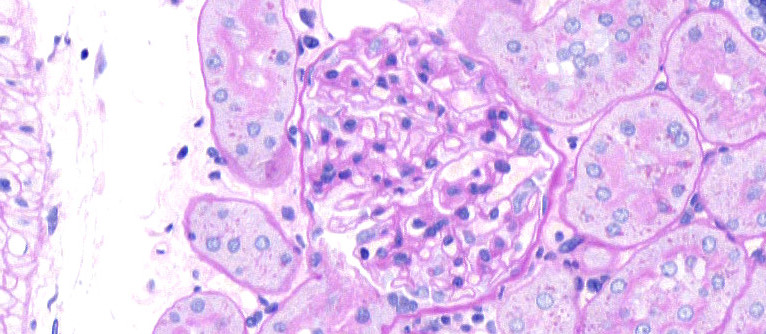

Supplement: Supplementary file 11 [file DataSheet12.ZIP › Fig 1D-PAS-TSF-57/57-11.jpeg]

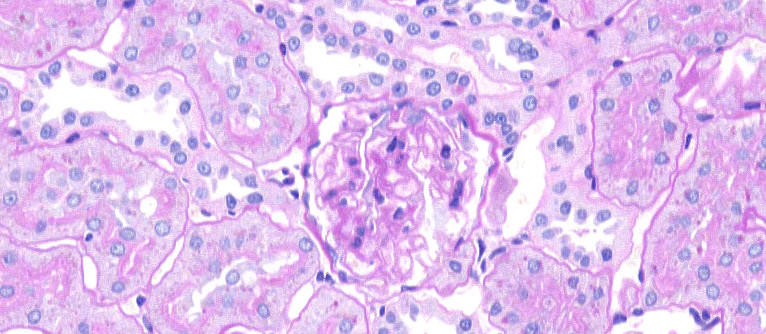

Supplement: Supplementary file 11 [file DataSheet12.ZIP › Fig 1D-PAS-TSF-57/57-12.jpeg]

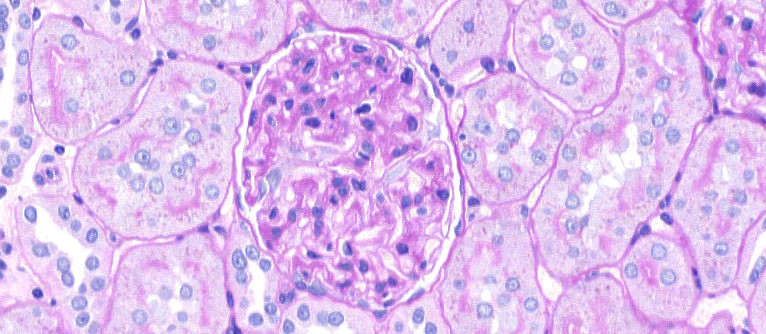

Supplement: Supplementary file 11 [file DataSheet12.ZIP › Fig 1D-PAS-TSF-57/57-13.jpeg]

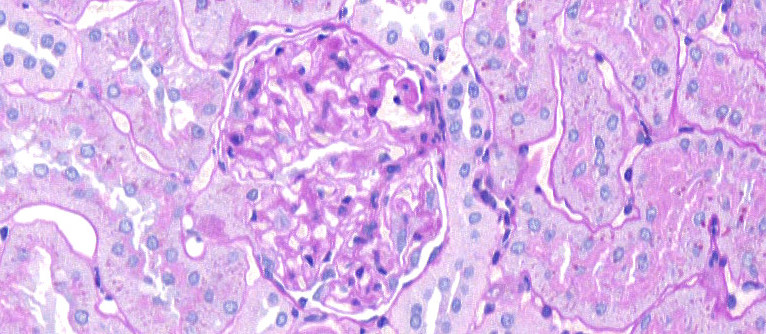

Supplement: Supplementary file 11 [file DataSheet12.ZIP › Fig 1D-PAS-TSF-57/57-14.jpeg]

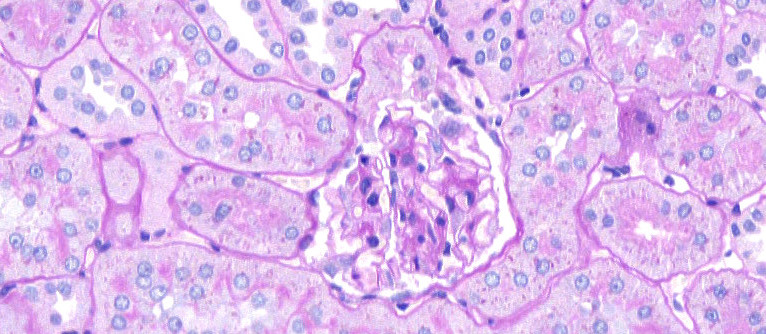

Supplement: Supplementary file 11 [file DataSheet12.ZIP › Fig 1D-PAS-TSF-57/57-15.jpeg]

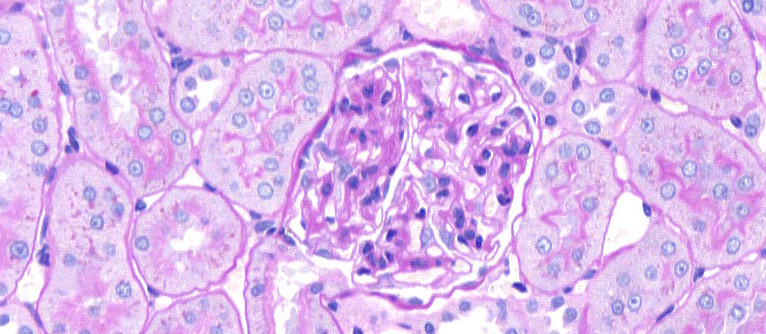

Supplement: Supplementary file 11 [file DataSheet12.ZIP › Fig 1D-PAS-TSF-57/57-16.jpeg]

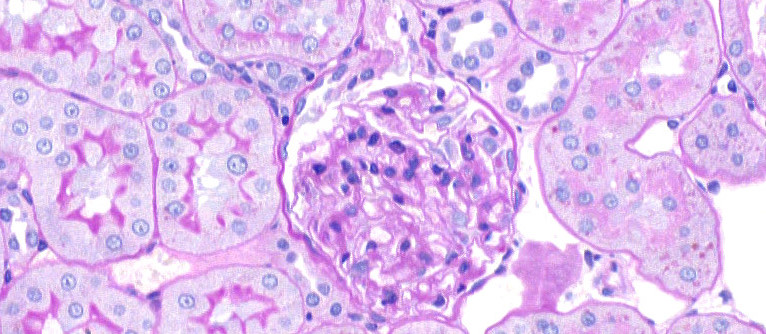

Supplement: Supplementary file 11 [file DataSheet12.ZIP › Fig 1D-PAS-TSF-57/57-17.jpeg]

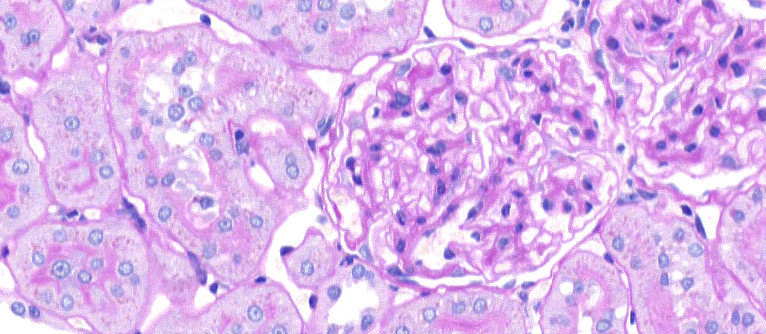

Supplement: Supplementary file 11 [file DataSheet12.ZIP › Fig 1D-PAS-TSF-57/57-18.jpeg]

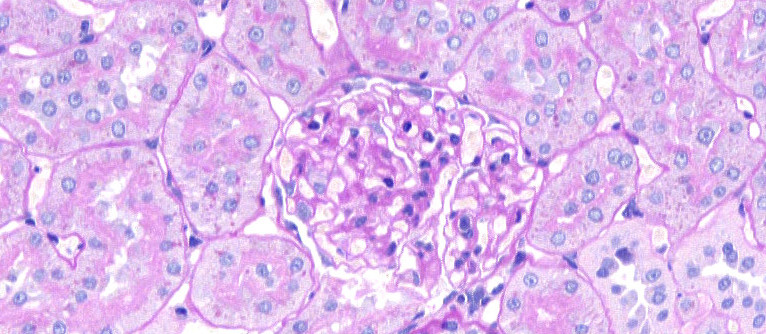

Supplement: Supplementary file 11 [file DataSheet12.ZIP › Fig 1D-PAS-TSF-57/57-19.jpeg]

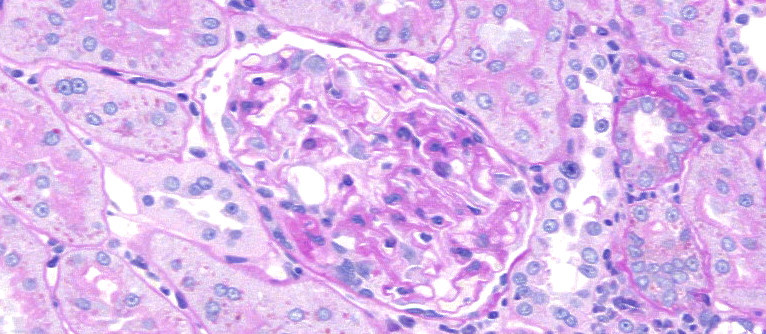

Supplement: Supplementary file 11 [file DataSheet12.ZIP › Fig 1D-PAS-TSF-57/57-2.jpeg]

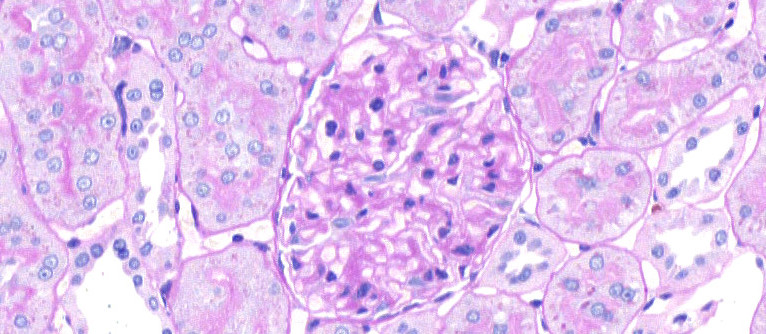

Supplement: Supplementary file 11 [file DataSheet12.ZIP › Fig 1D-PAS-TSF-57/57-20.jpeg]

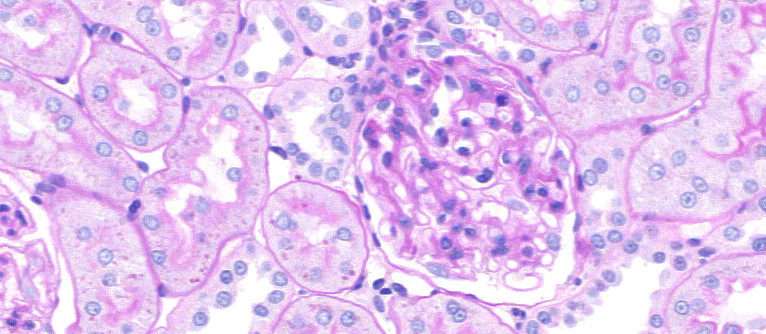

Supplement: Supplementary file 11 [file DataSheet12.ZIP › Fig 1D-PAS-TSF-57/57-3.jpeg]

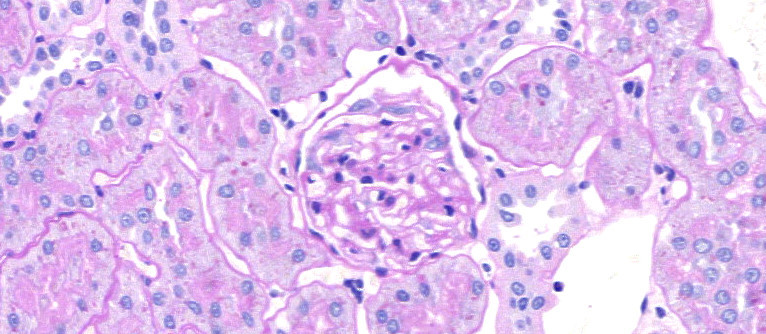

Supplement: Supplementary file 11 [file DataSheet12.ZIP › Fig 1D-PAS-TSF-57/57-4.jpeg]

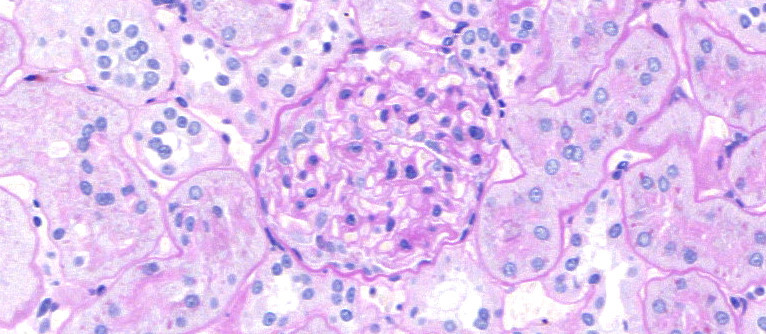

Supplement: Supplementary file 11 [file DataSheet12.ZIP › Fig 1D-PAS-TSF-57/57-5.jpeg]

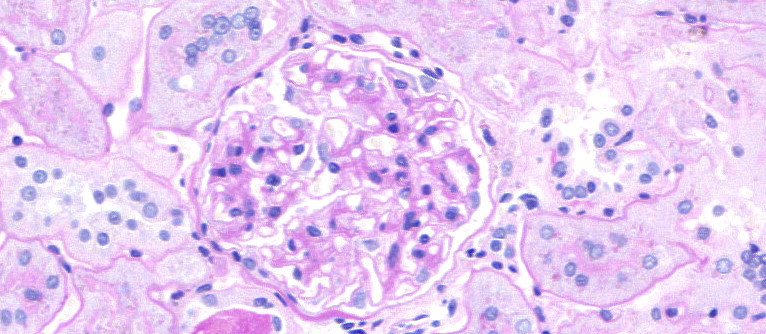

Supplement: Supplementary file 11 [file DataSheet12.ZIP › Fig 1D-PAS-TSF-57/57-6.jpeg]

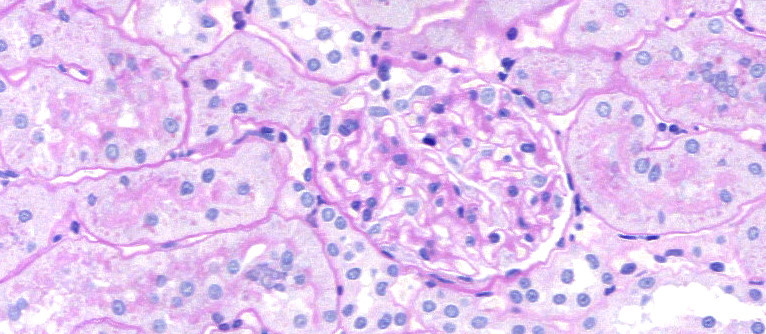

Supplement: Supplementary file 11 [file DataSheet12.ZIP › Fig 1D-PAS-TSF-57/57-7.jpeg]

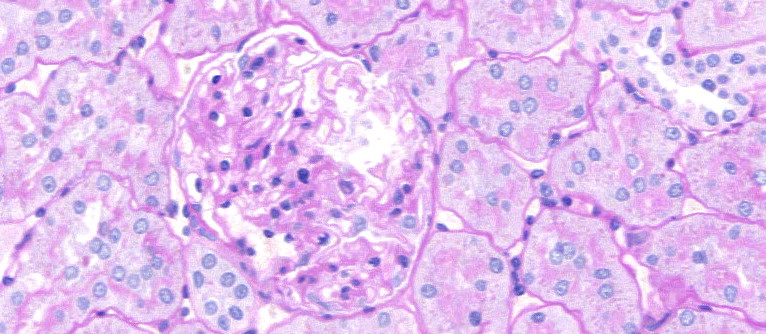

Supplement: Supplementary file 11 [file DataSheet12.ZIP › Fig 1D-PAS-TSF-57/57-8.jpeg]

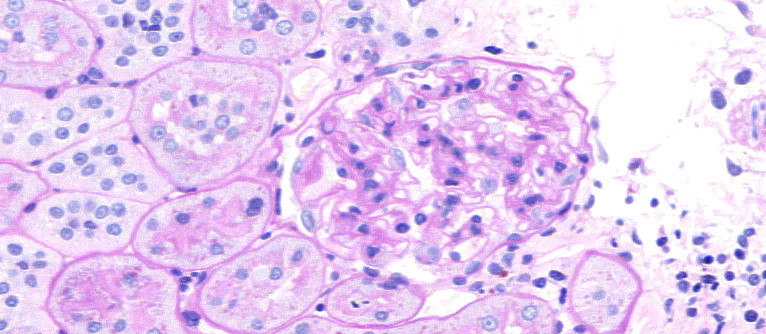

Supplement: Supplementary file 11 [file DataSheet12.ZIP › Fig 1D-PAS-TSF-57/57-9.jpeg]
