# Supplementary figures and images for: Characteristics of Serum Metabolites and Gut Microbiota in Diabetic Kidney Disease (part 10 of 13)
Source: Front Pharmacol. 2022 Apr 14;13:872988. doi: 10.3389/fphar.2022.872988 (PMC9084235; doi:10.3389/fphar.2022.872988)

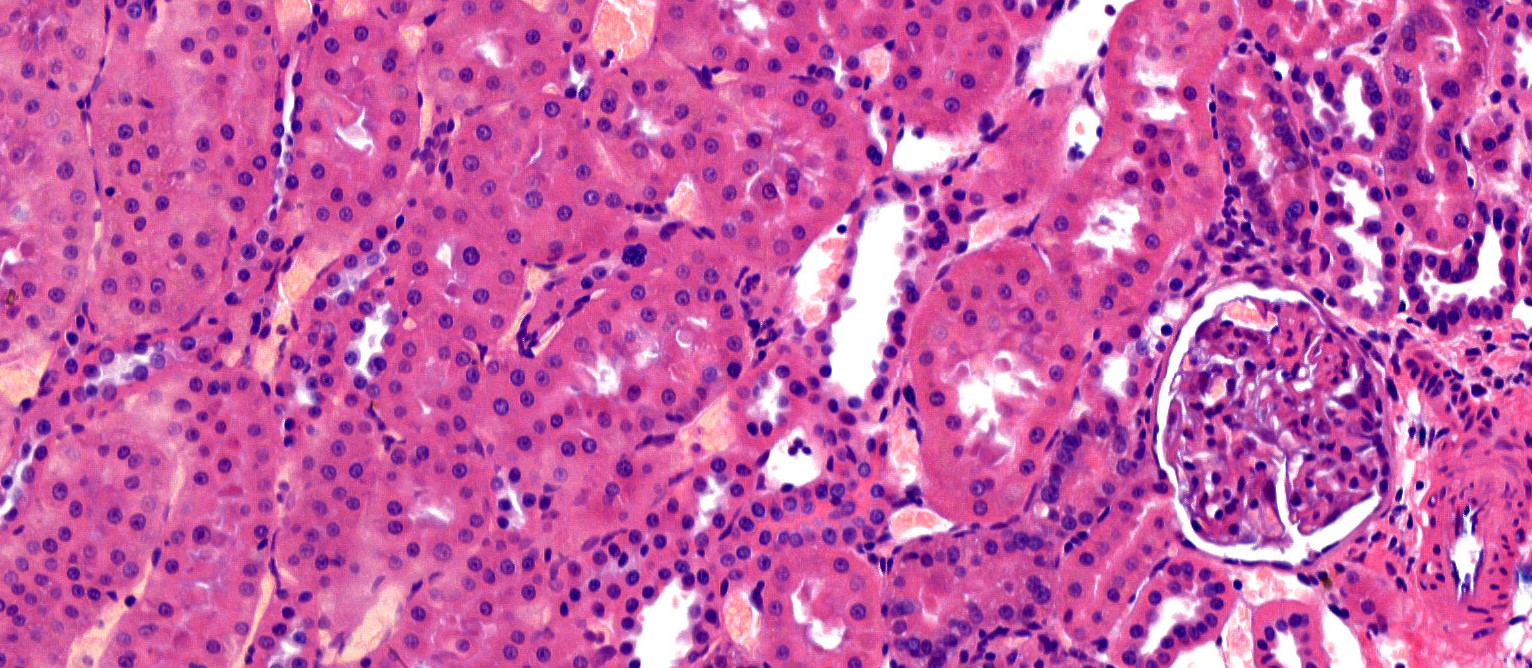

Supplement: Supplementary file 12 [file DataSheet2.ZIP › Fig 1D-HE-DKD-15(2)/15-10.jpeg]

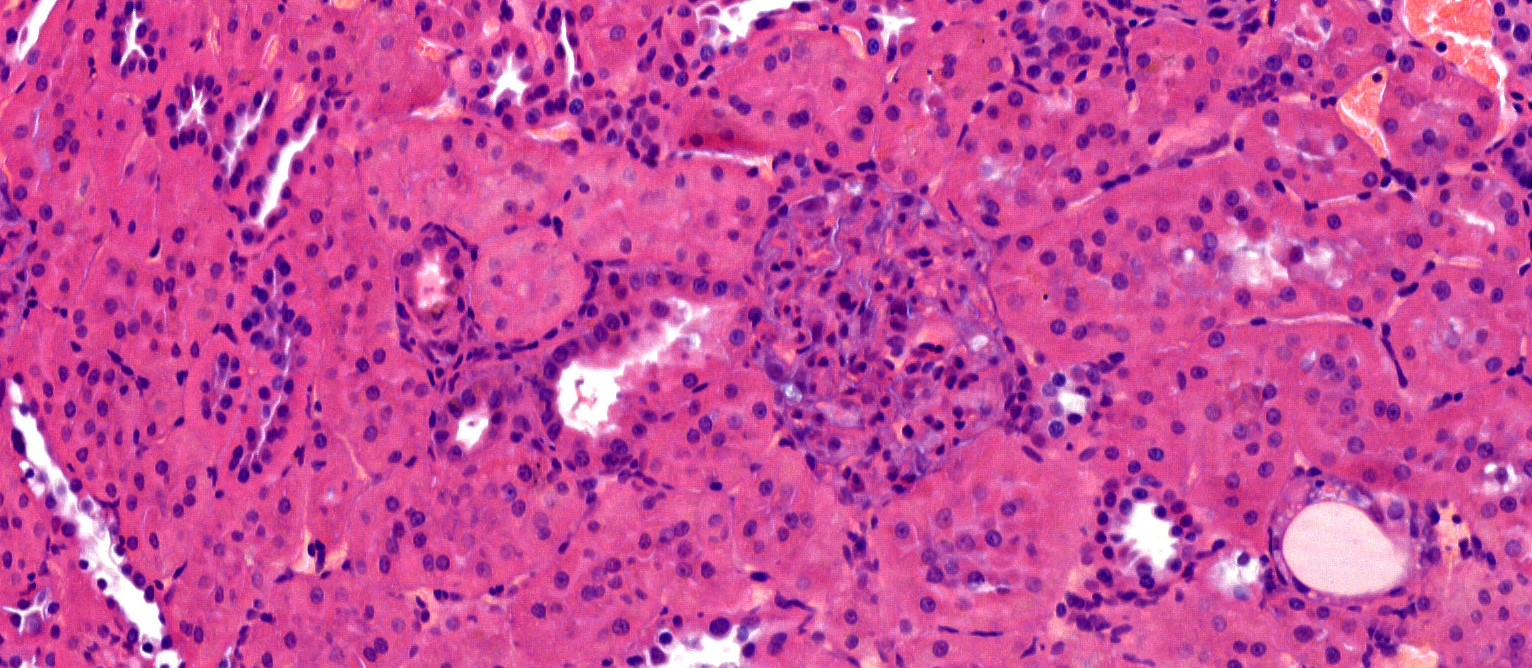

Supplement: Supplementary file 12 [file DataSheet2.ZIP › Fig 1D-HE-DKD-15(2)/15-5.jpeg]

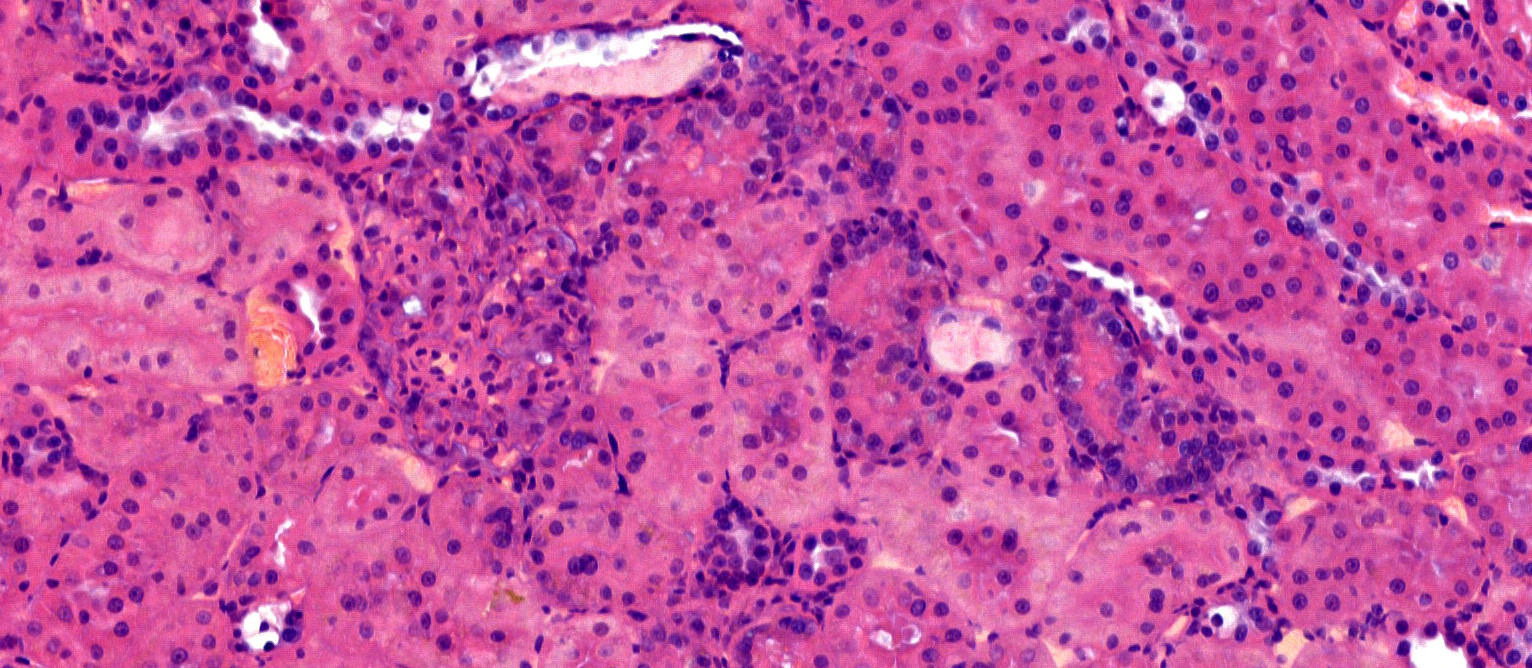

Supplement: Supplementary file 12 [file DataSheet2.ZIP › Fig 1D-HE-DKD-15(2)/15-6.jpeg]

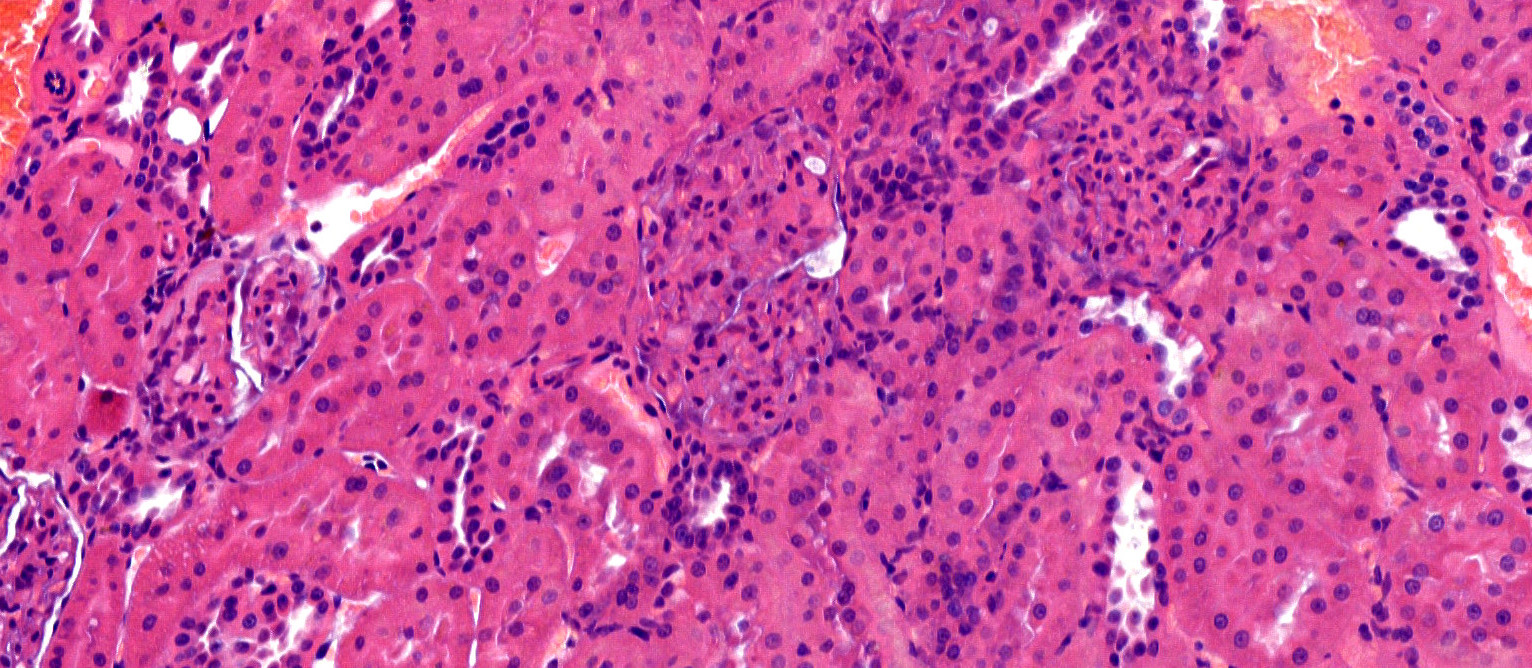

Supplement: Supplementary file 12 [file DataSheet2.ZIP › Fig 1D-HE-DKD-15(2)/15-7.jpeg]

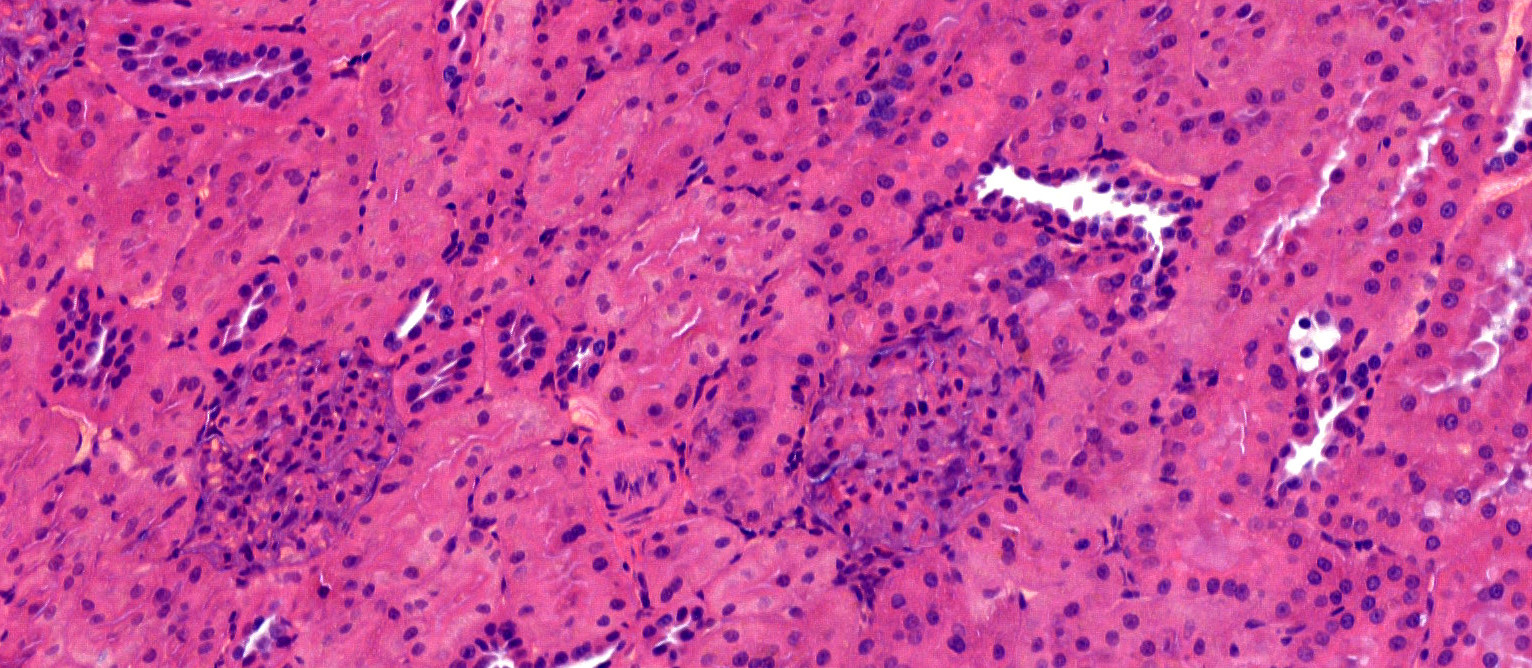

Supplement: Supplementary file 12 [file DataSheet2.ZIP › Fig 1D-HE-DKD-15(2)/15-8.jpeg]

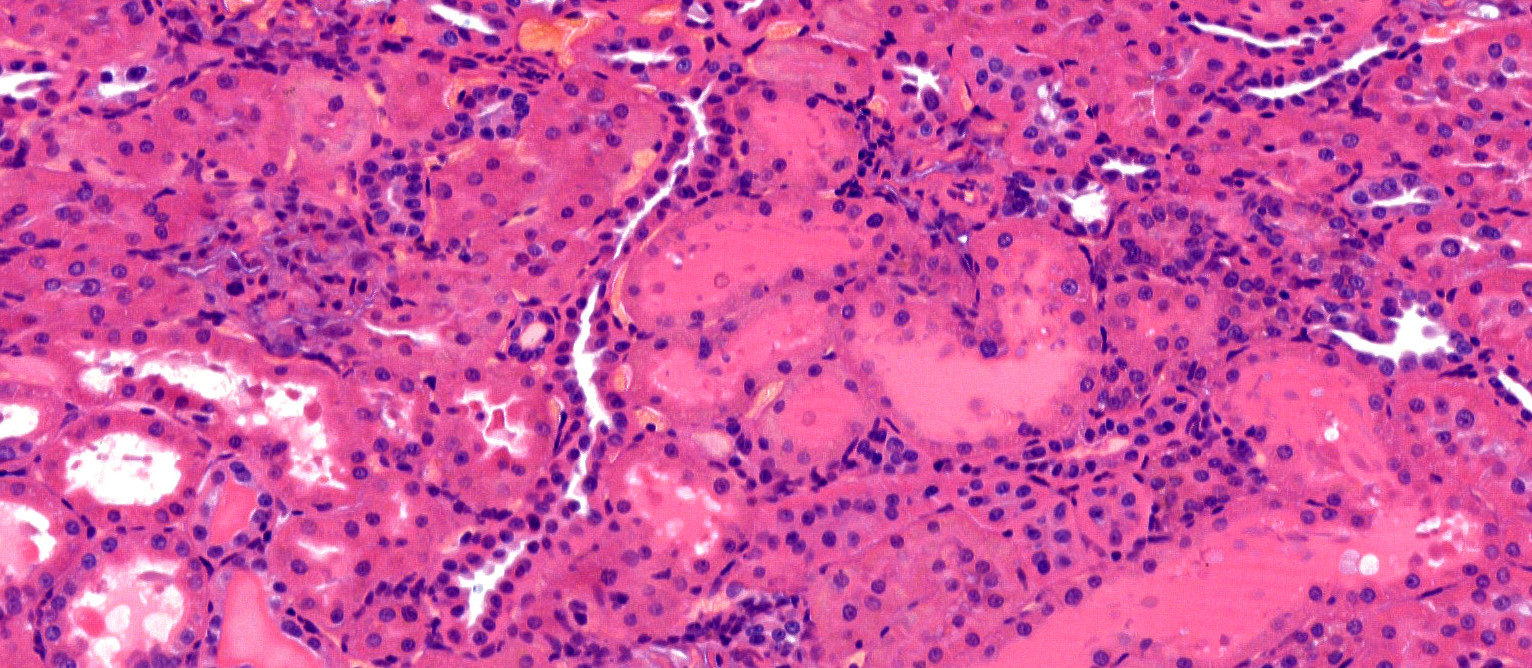

Supplement: Supplementary file 12 [file DataSheet2.ZIP › Fig 1D-HE-DKD-15(2)/15-9.jpeg]

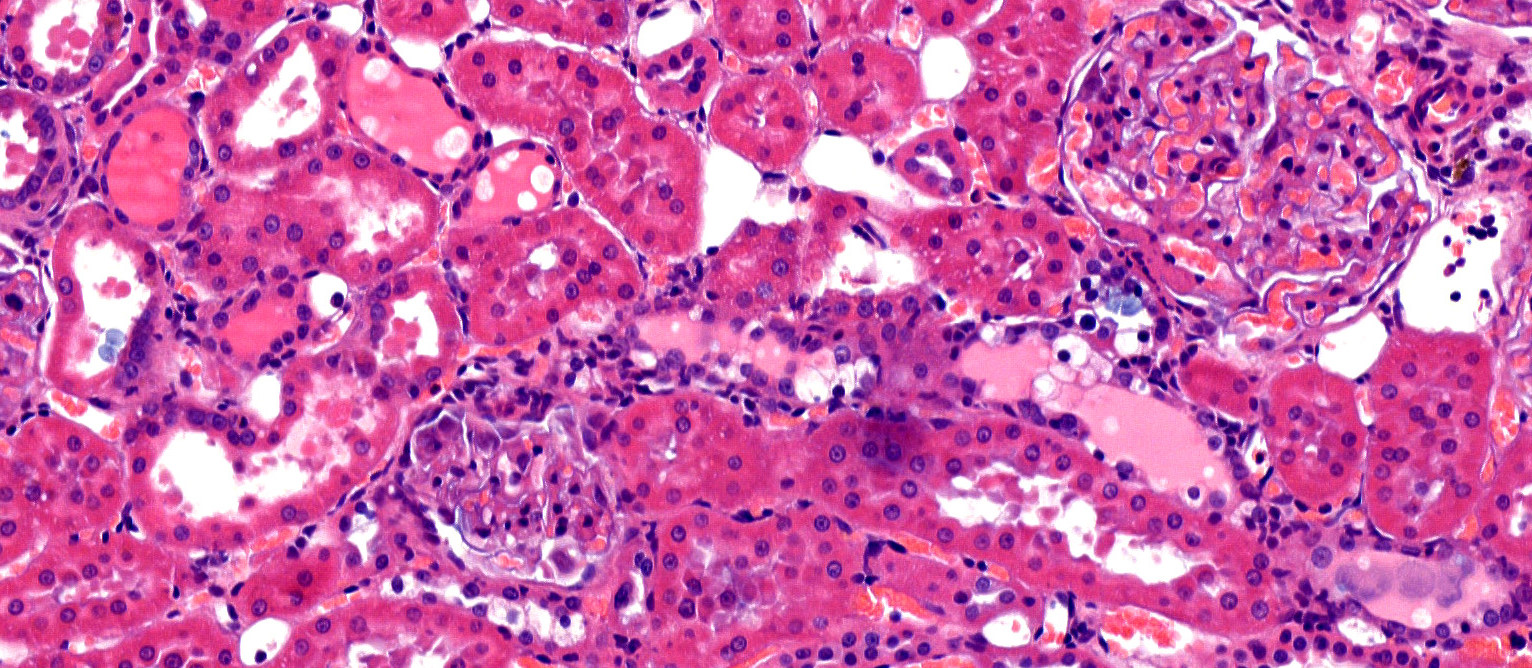

Supplement: Supplementary file 12 [file DataSheet2.ZIP › Fig 1D-HE-DKD-17/17 (1).jpeg]

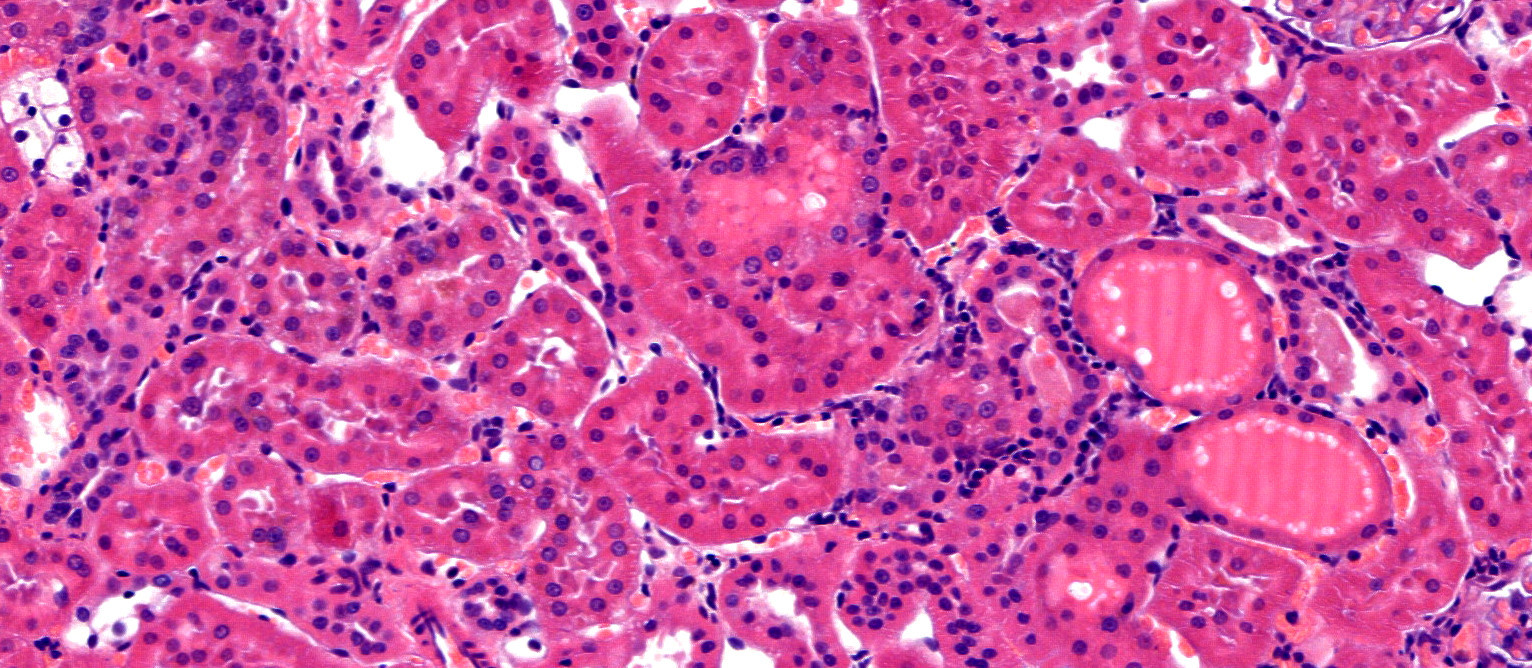

Supplement: Supplementary file 12 [file DataSheet2.ZIP › Fig 1D-HE-DKD-17/17 (10).jpeg]

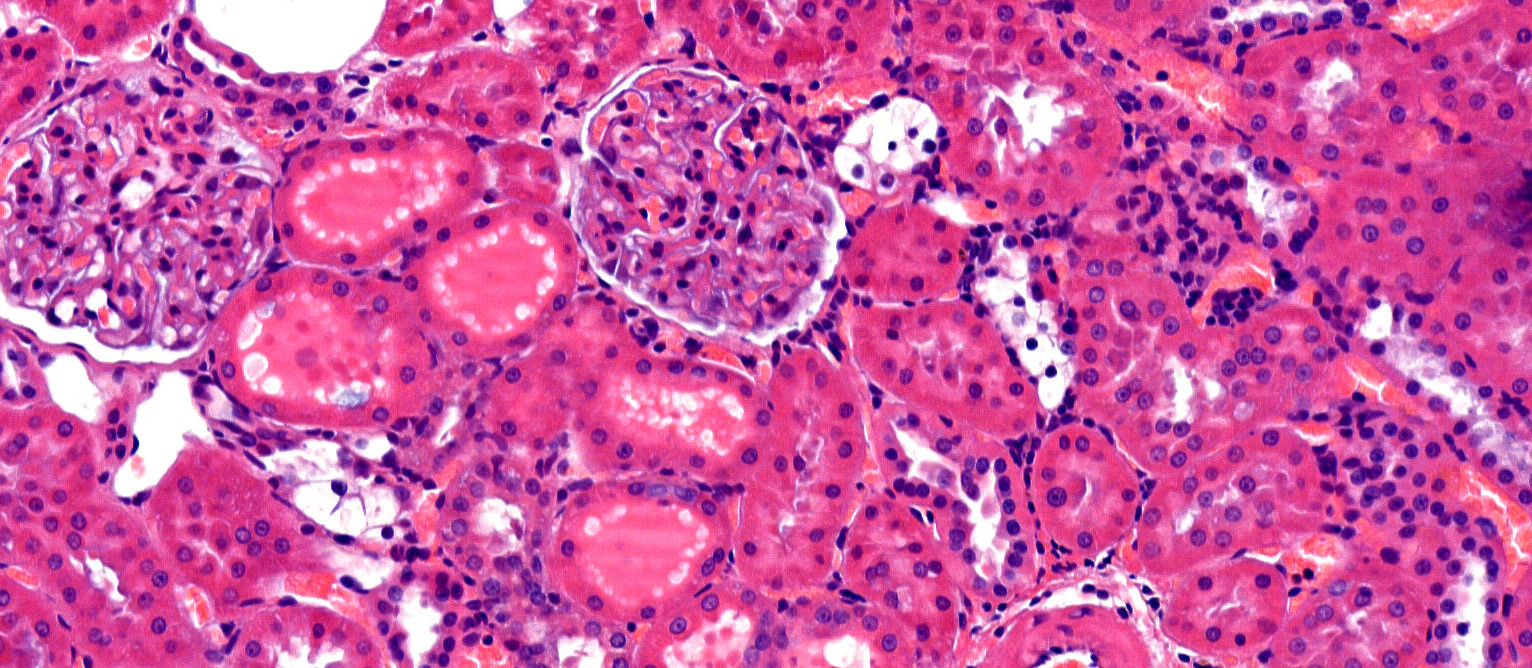

Supplement: Supplementary file 12 [file DataSheet2.ZIP › Fig 1D-HE-DKD-17/17 (2).jpeg]

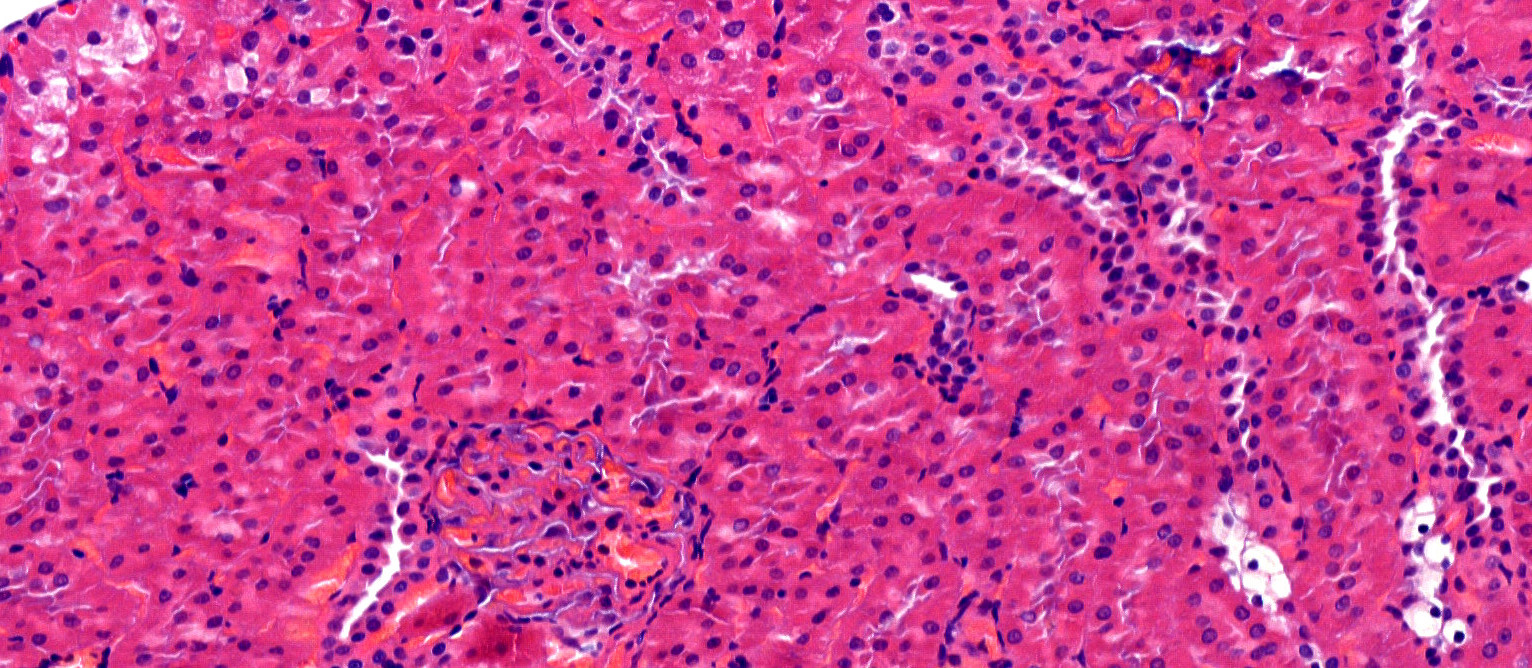

Supplement: Supplementary file 12 [file DataSheet2.ZIP › Fig 1D-HE-DKD-17/17 (3).jpeg]

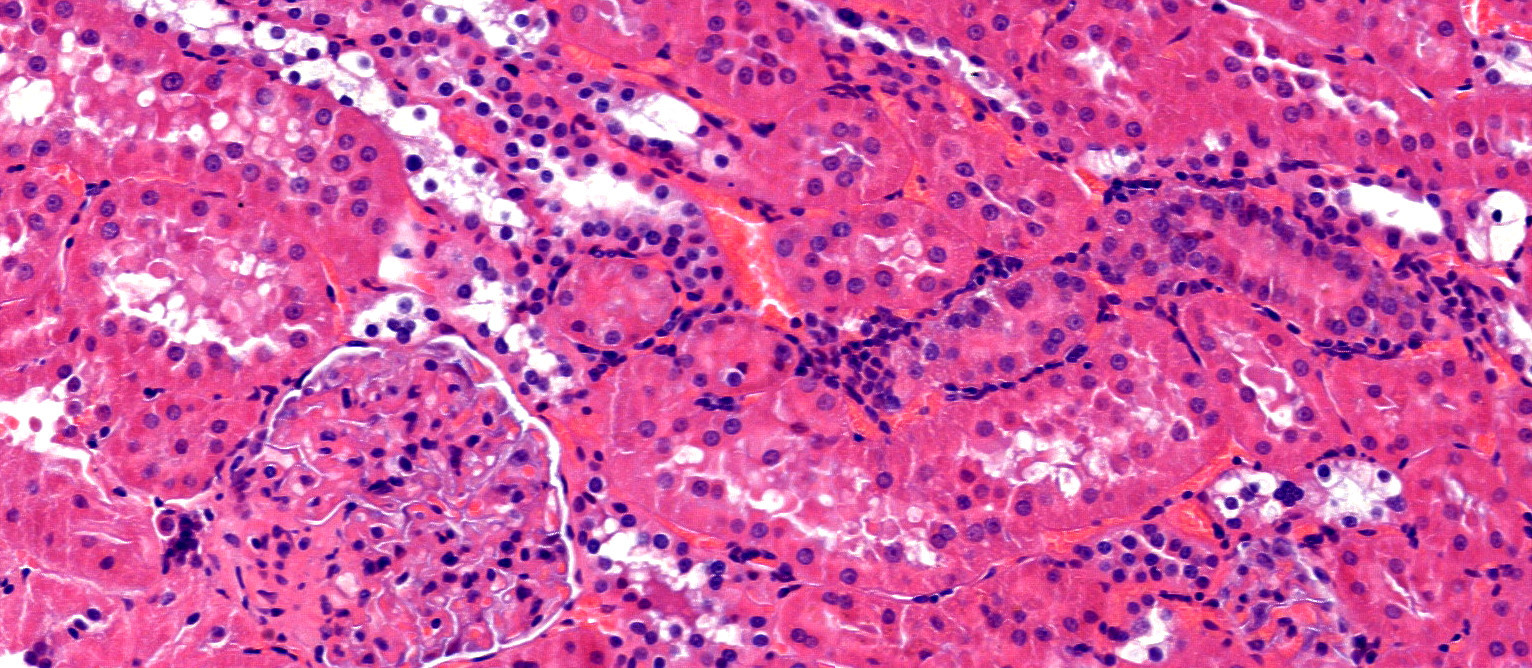

Supplement: Supplementary file 12 [file DataSheet2.ZIP › Fig 1D-HE-DKD-17/17 (4).jpeg]

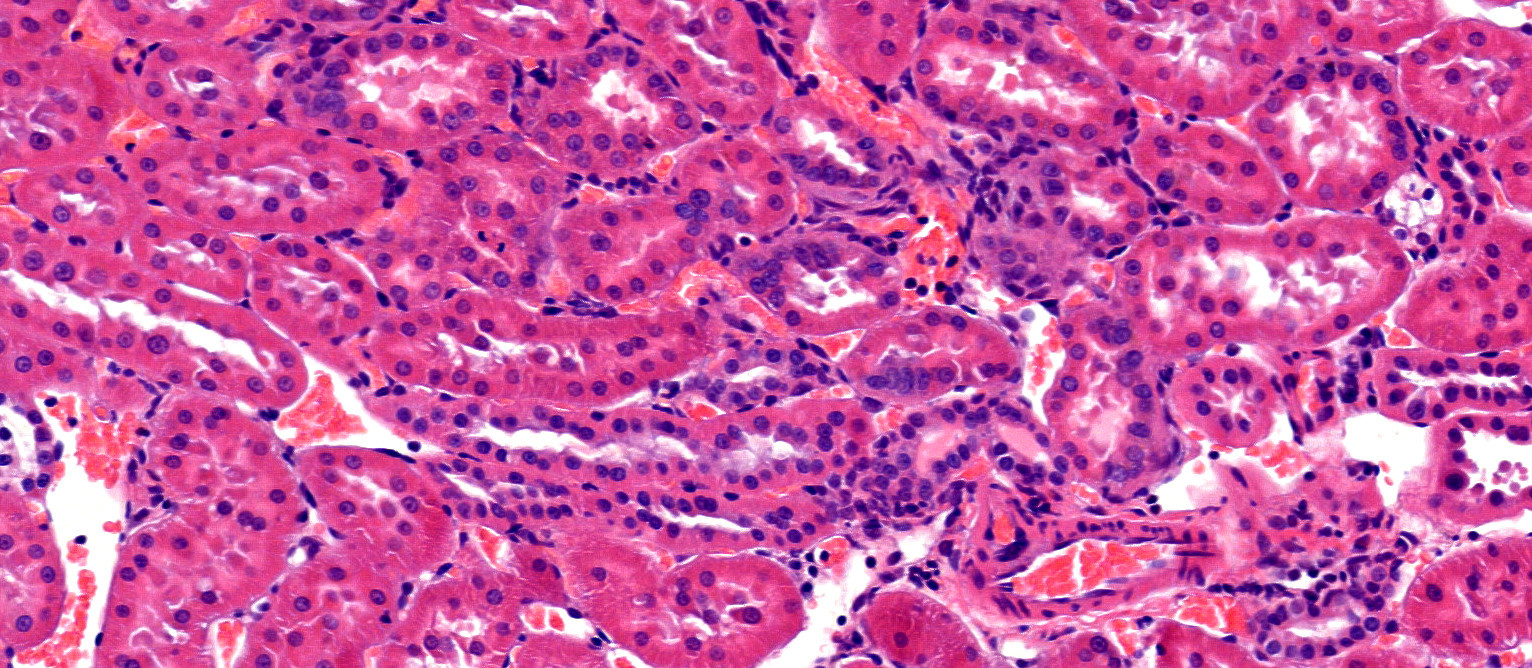

Supplement: Supplementary file 12 [file DataSheet2.ZIP › Fig 1D-HE-DKD-17/17 (5).jpeg]

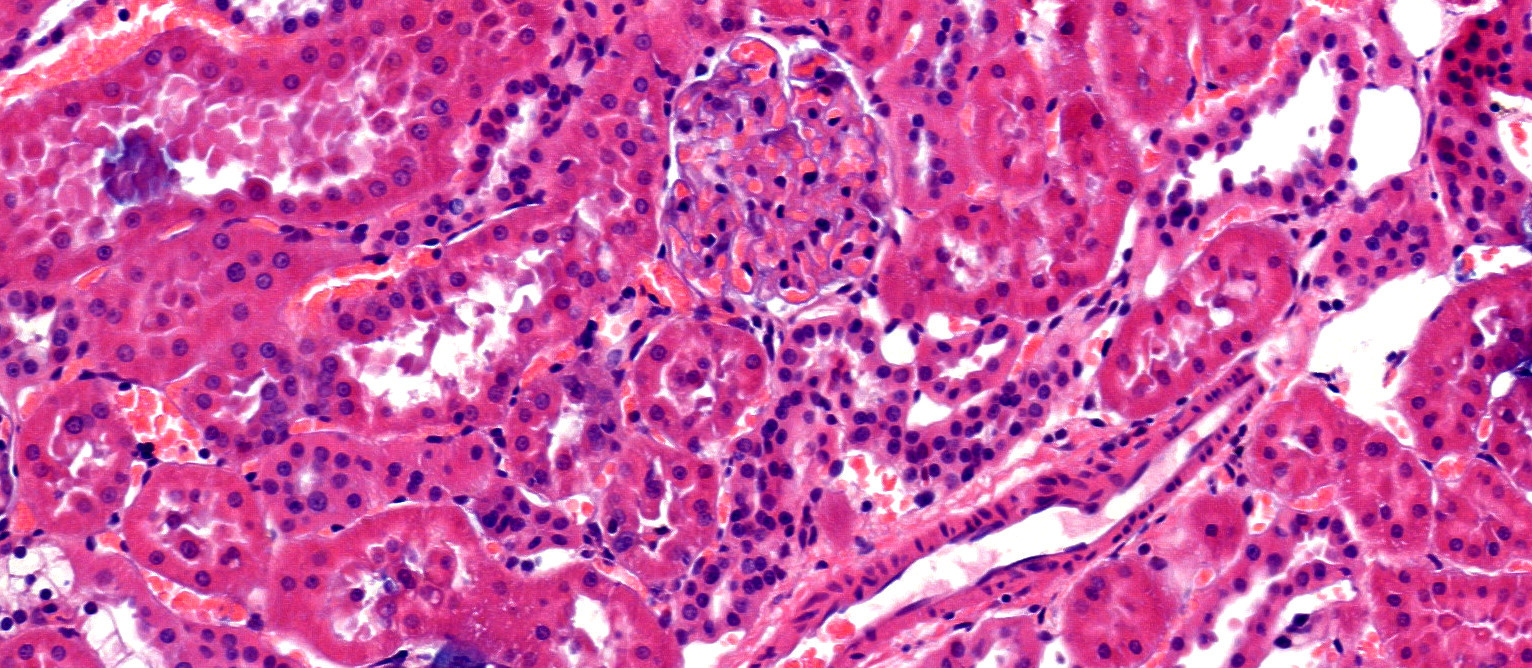

Supplement: Supplementary file 12 [file DataSheet2.ZIP › Fig 1D-HE-DKD-17/17 (6).jpeg]

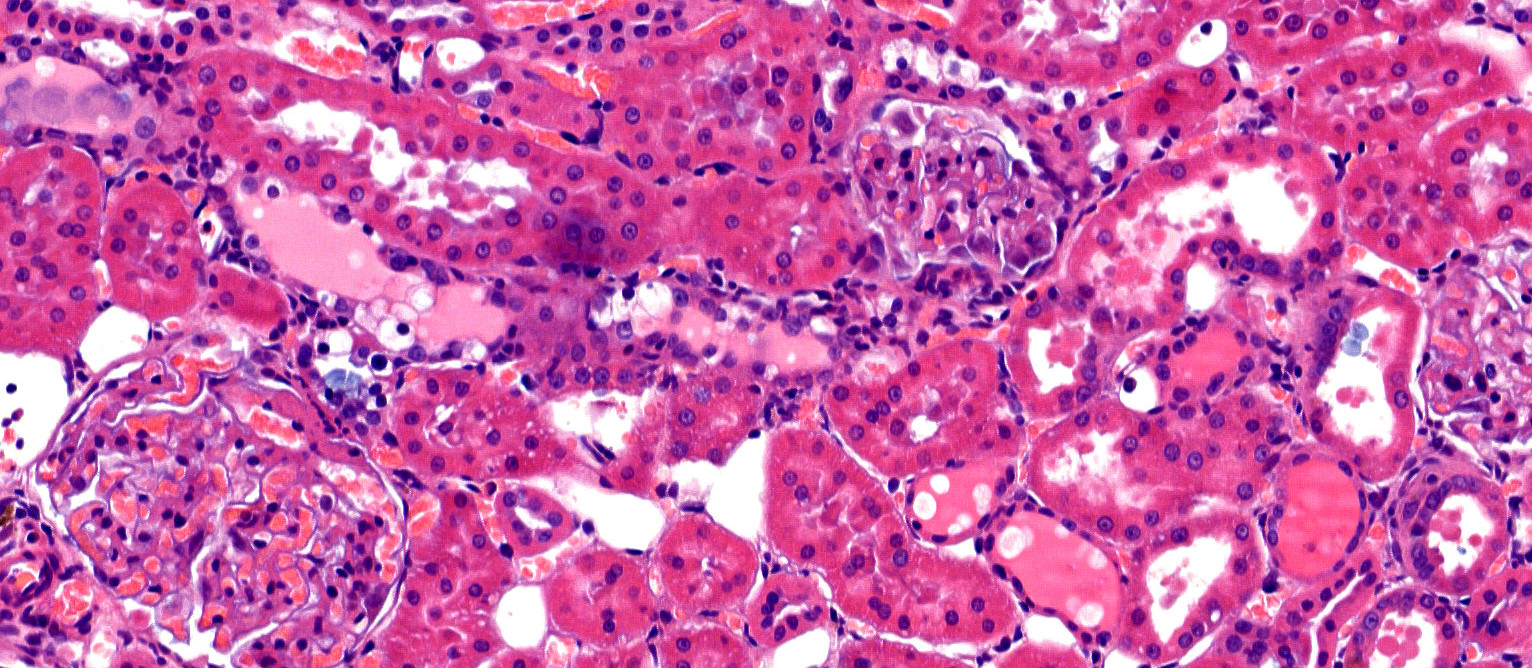

Supplement: Supplementary file 12 [file DataSheet2.ZIP › Fig 1D-HE-DKD-17/17 (7).jpeg]

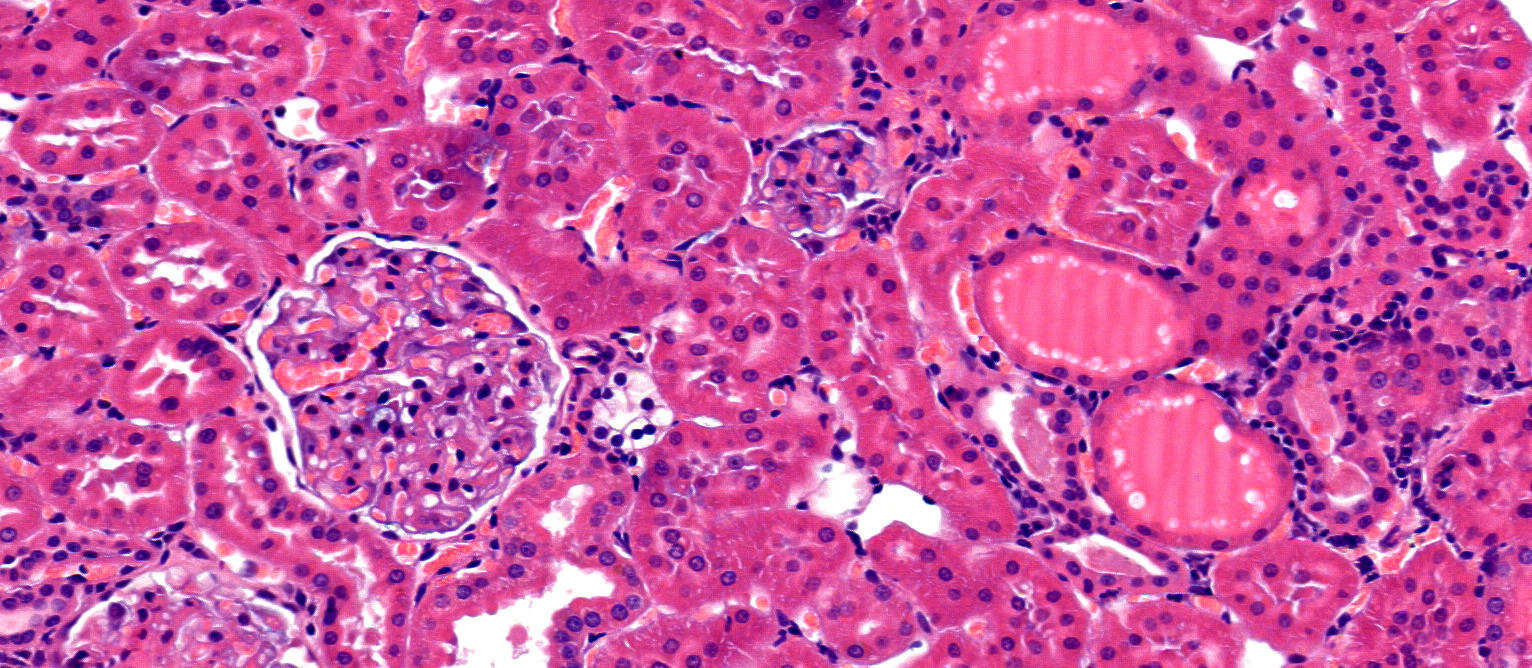

Supplement: Supplementary file 12 [file DataSheet2.ZIP › Fig 1D-HE-DKD-17/17 (8).jpeg]

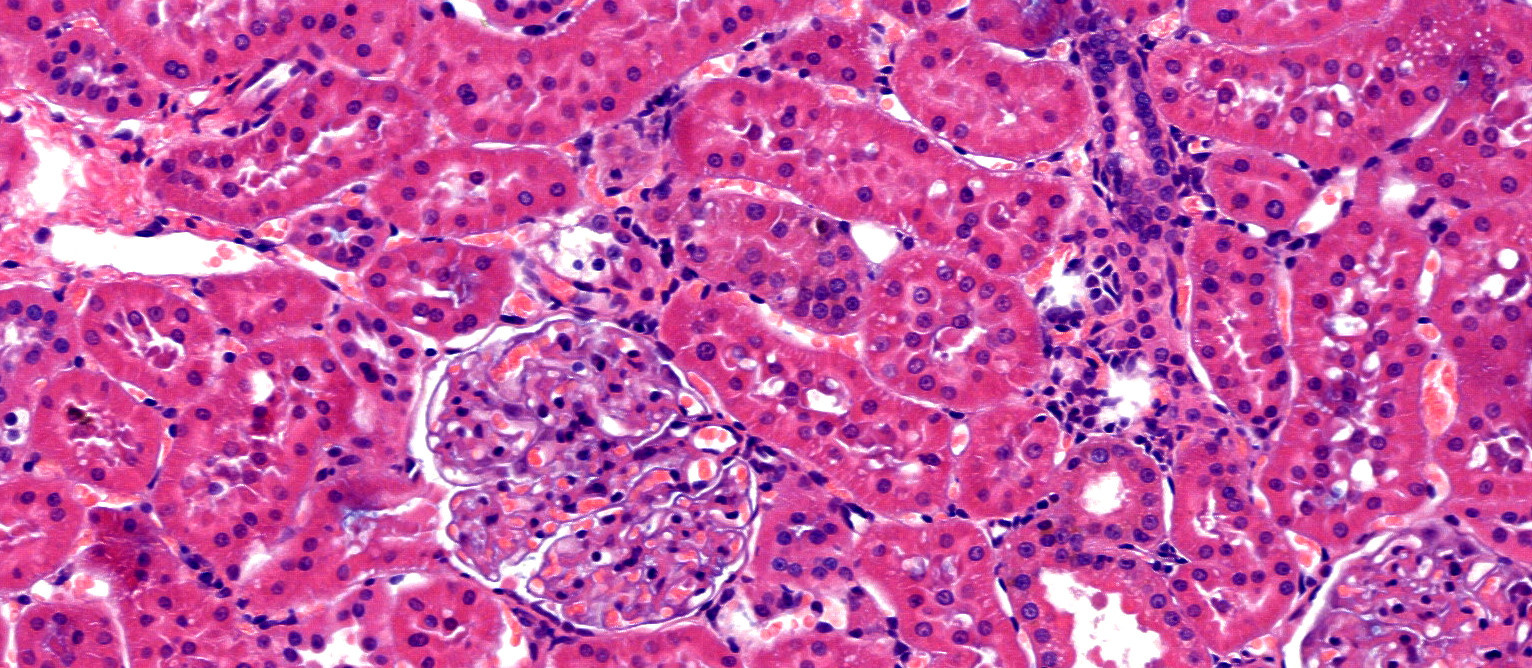

Supplement: Supplementary file 12 [file DataSheet2.ZIP › Fig 1D-HE-DKD-17/17 (9).jpeg]

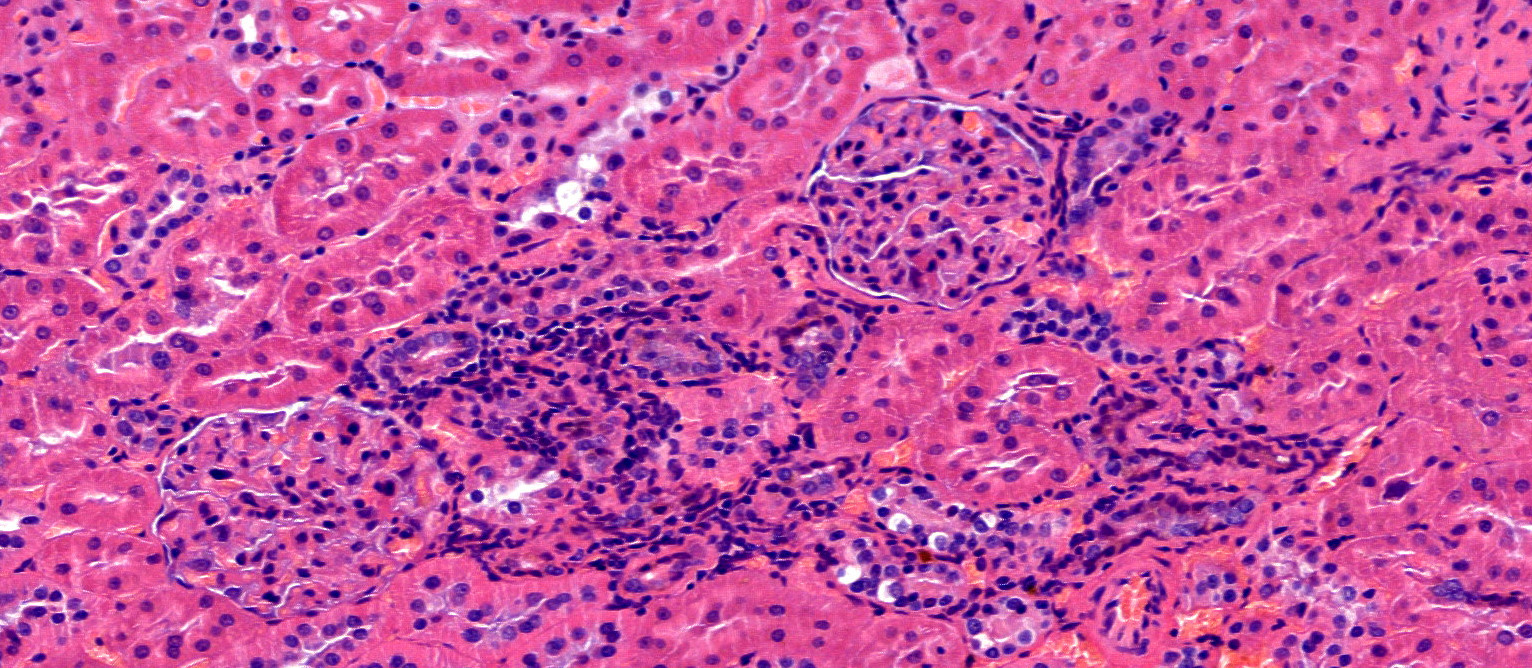

Supplement: Supplementary file 12 [file DataSheet2.ZIP › Fig 1D-HE-DKD-18/18-1.jpeg]

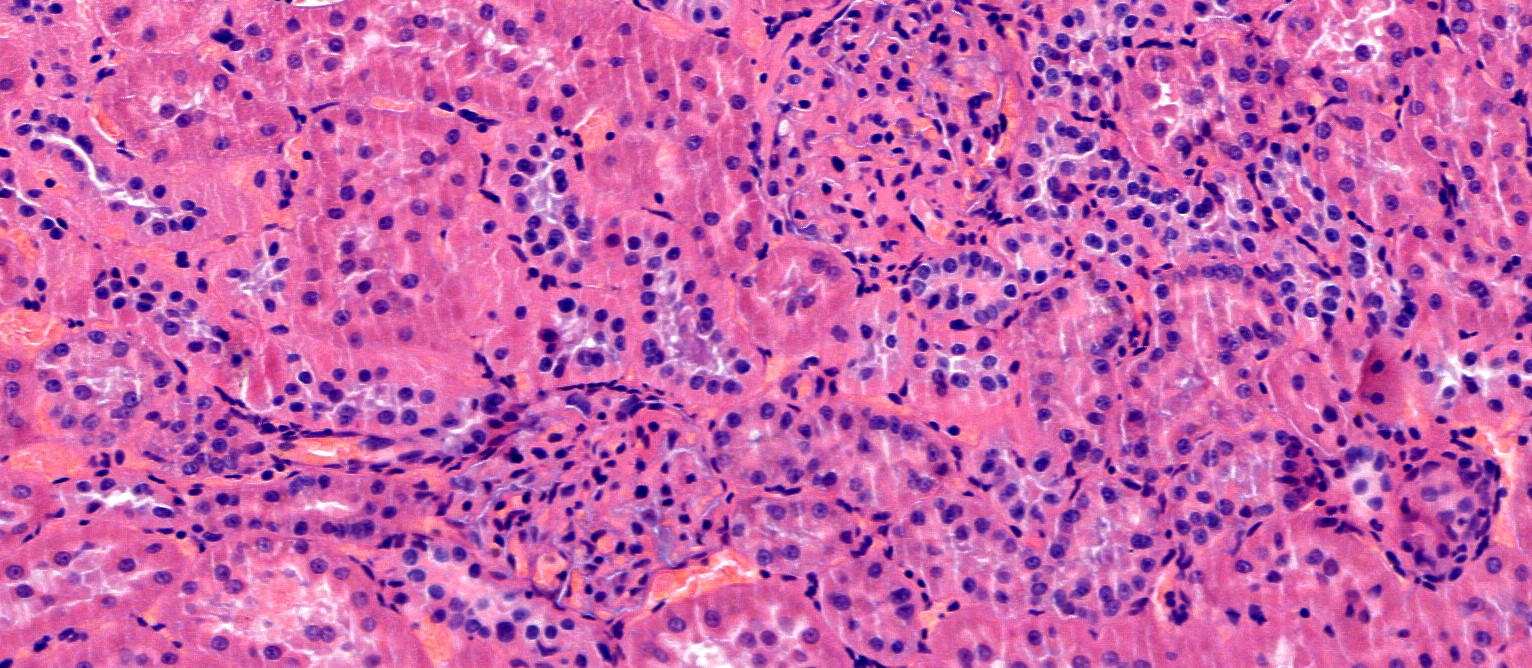

Supplement: Supplementary file 12 [file DataSheet2.ZIP › Fig 1D-HE-DKD-18/18-10.jpeg]

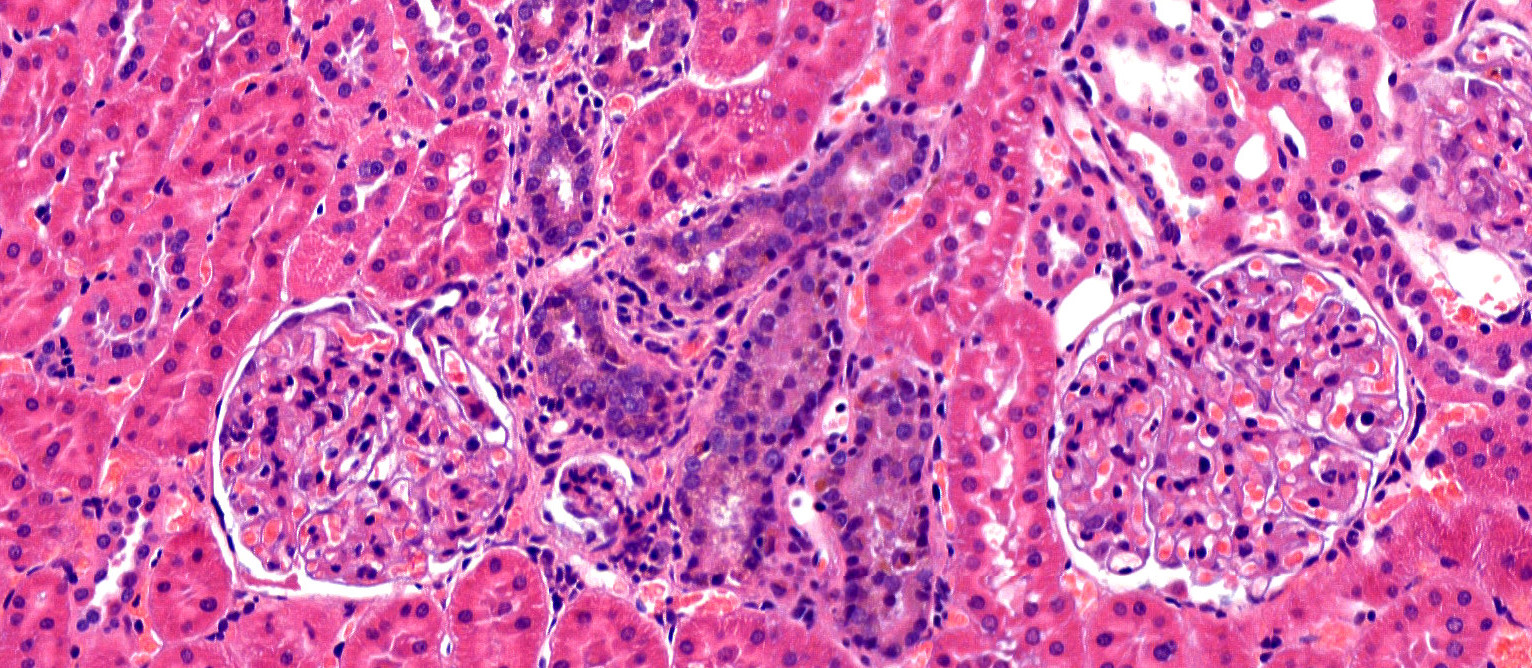

Supplement: Supplementary file 12 [file DataSheet2.ZIP › Fig 1D-HE-DKD-18/18-2.jpeg]

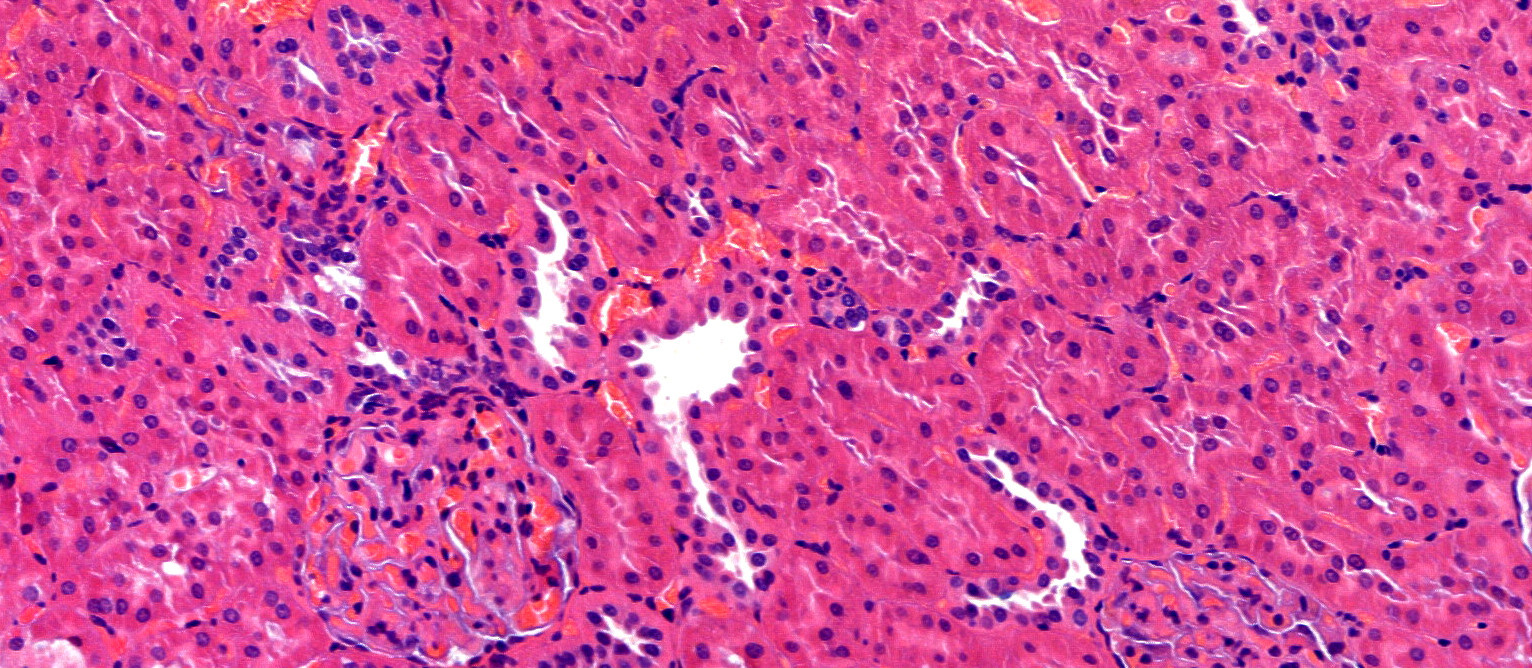

Supplement: Supplementary file 12 [file DataSheet2.ZIP › Fig 1D-HE-DKD-18/18-3.jpeg]

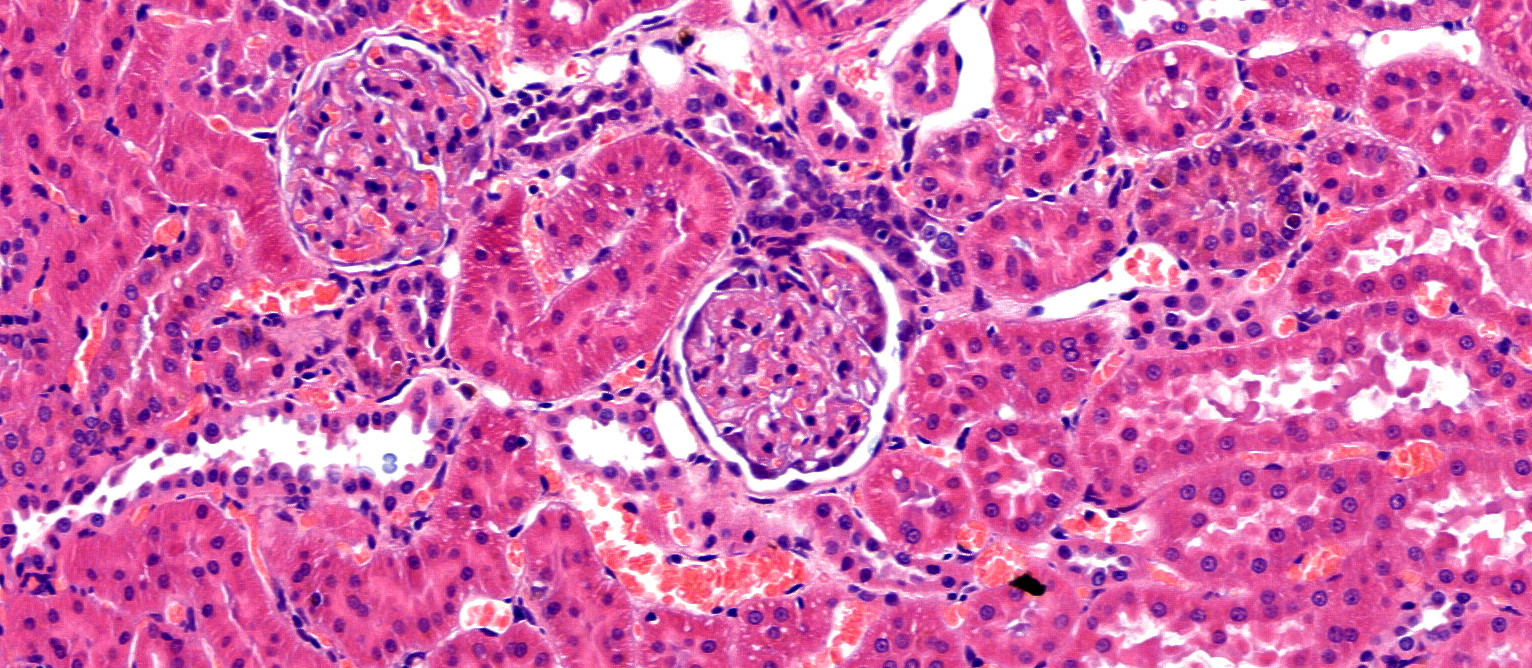

Supplement: Supplementary file 12 [file DataSheet2.ZIP › Fig 1D-HE-DKD-18/18-4.jpeg]

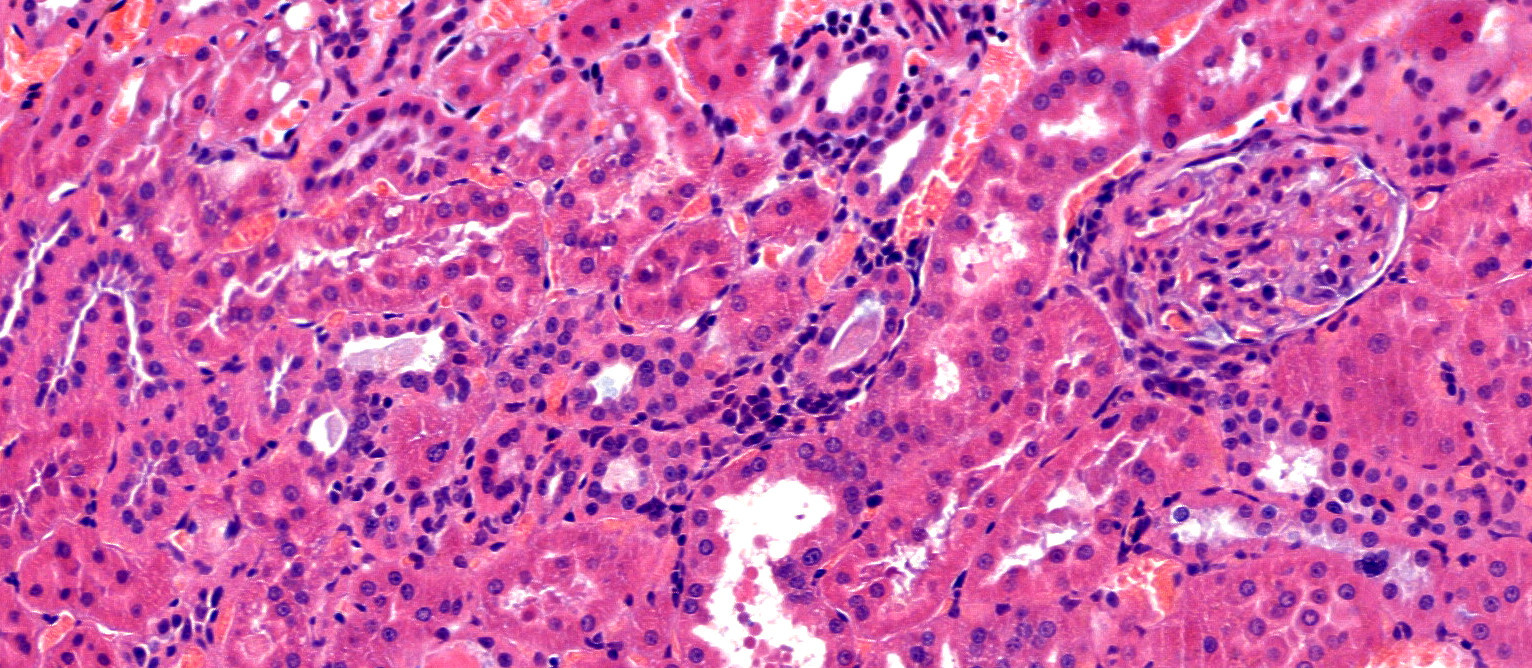

Supplement: Supplementary file 12 [file DataSheet2.ZIP › Fig 1D-HE-DKD-18/18-5.jpeg]

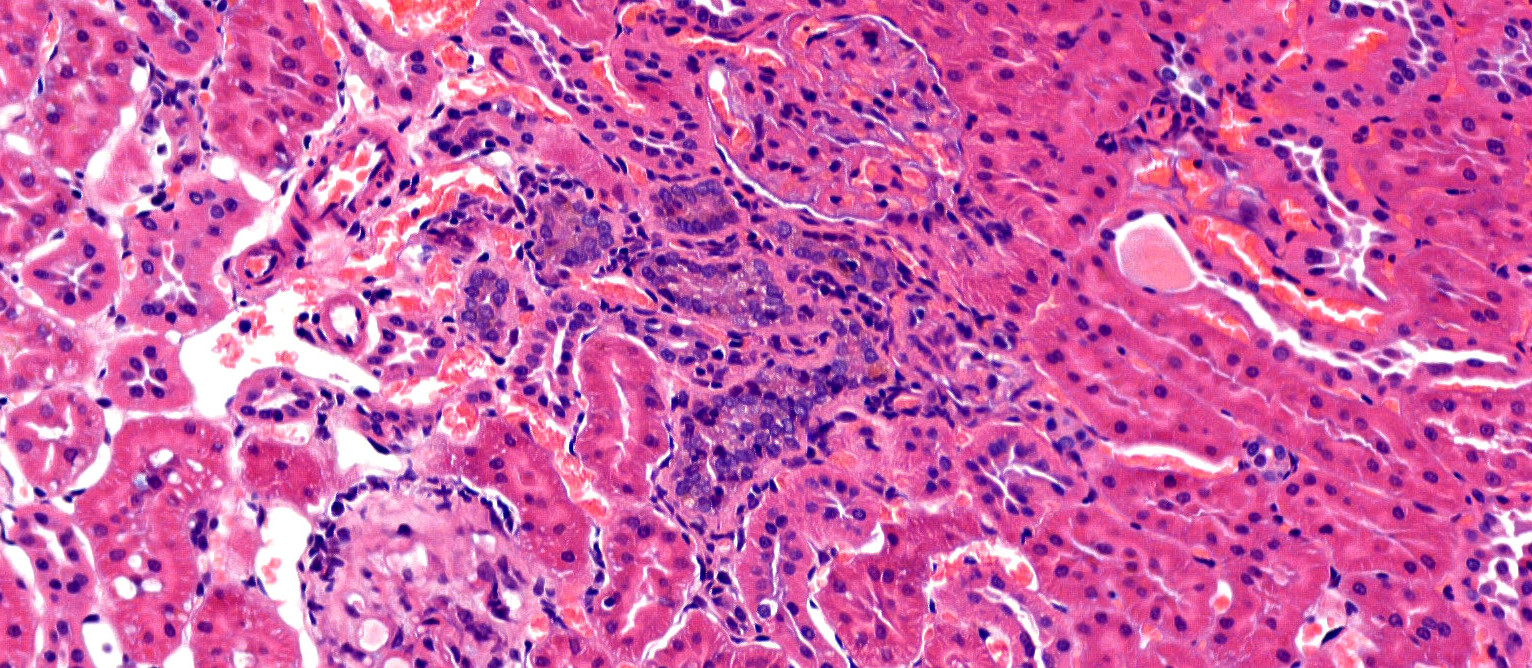

Supplement: Supplementary file 12 [file DataSheet2.ZIP › Fig 1D-HE-DKD-18/18-6.jpeg]

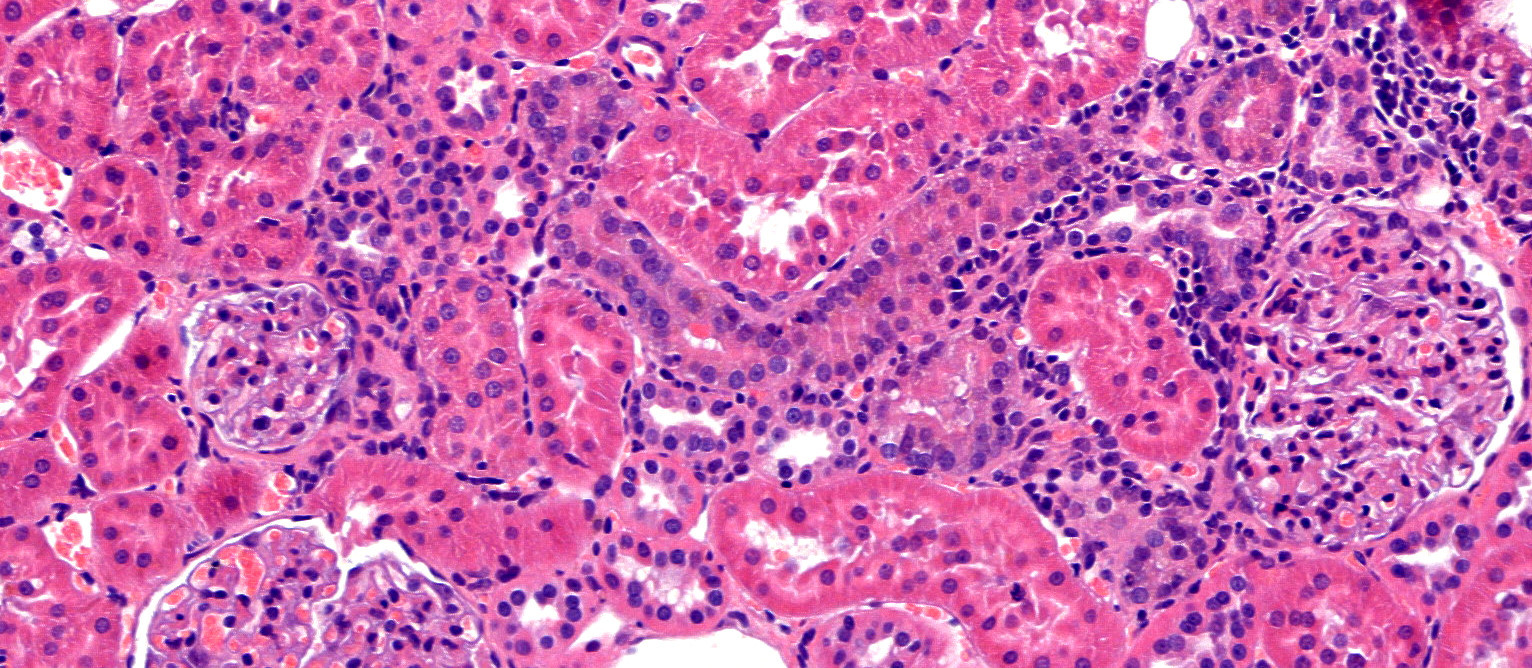

Supplement: Supplementary file 12 [file DataSheet2.ZIP › Fig 1D-HE-DKD-18/18-7.jpeg]

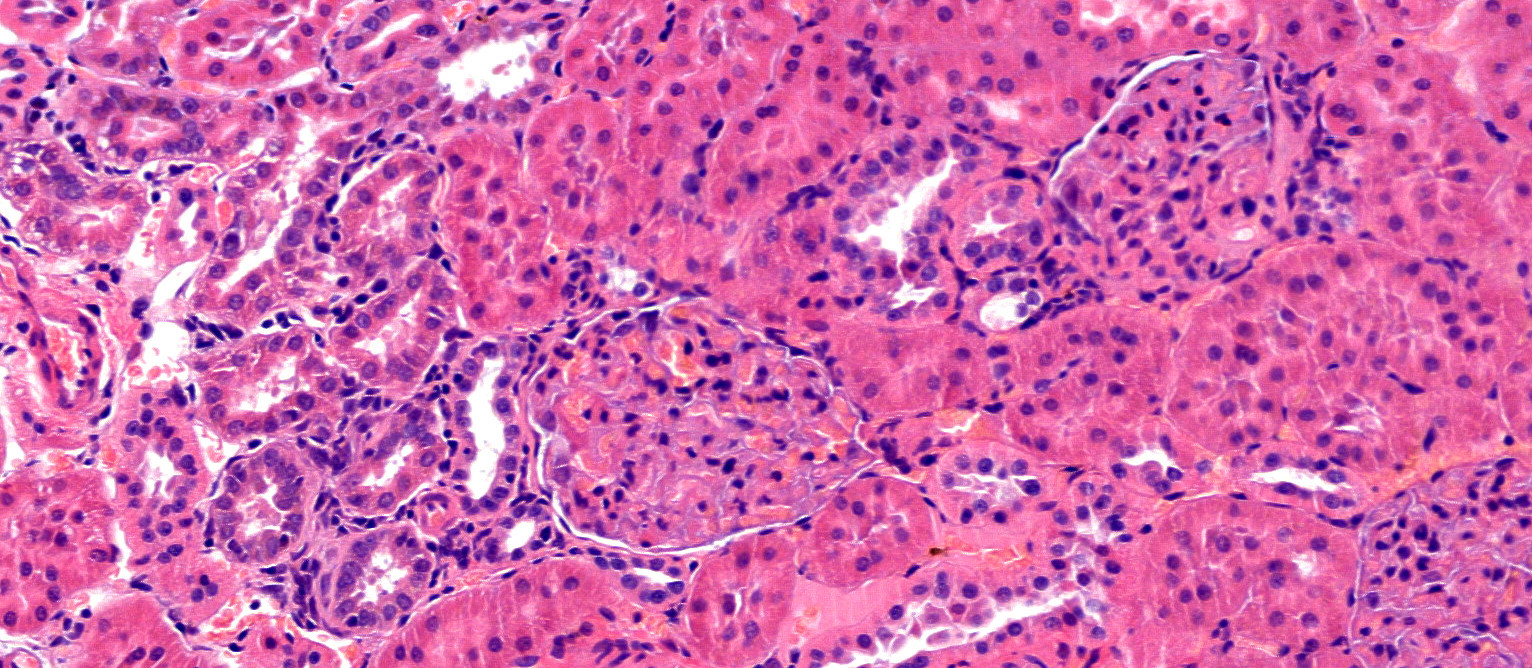

Supplement: Supplementary file 12 [file DataSheet2.ZIP › Fig 1D-HE-DKD-18/18-8.jpeg]

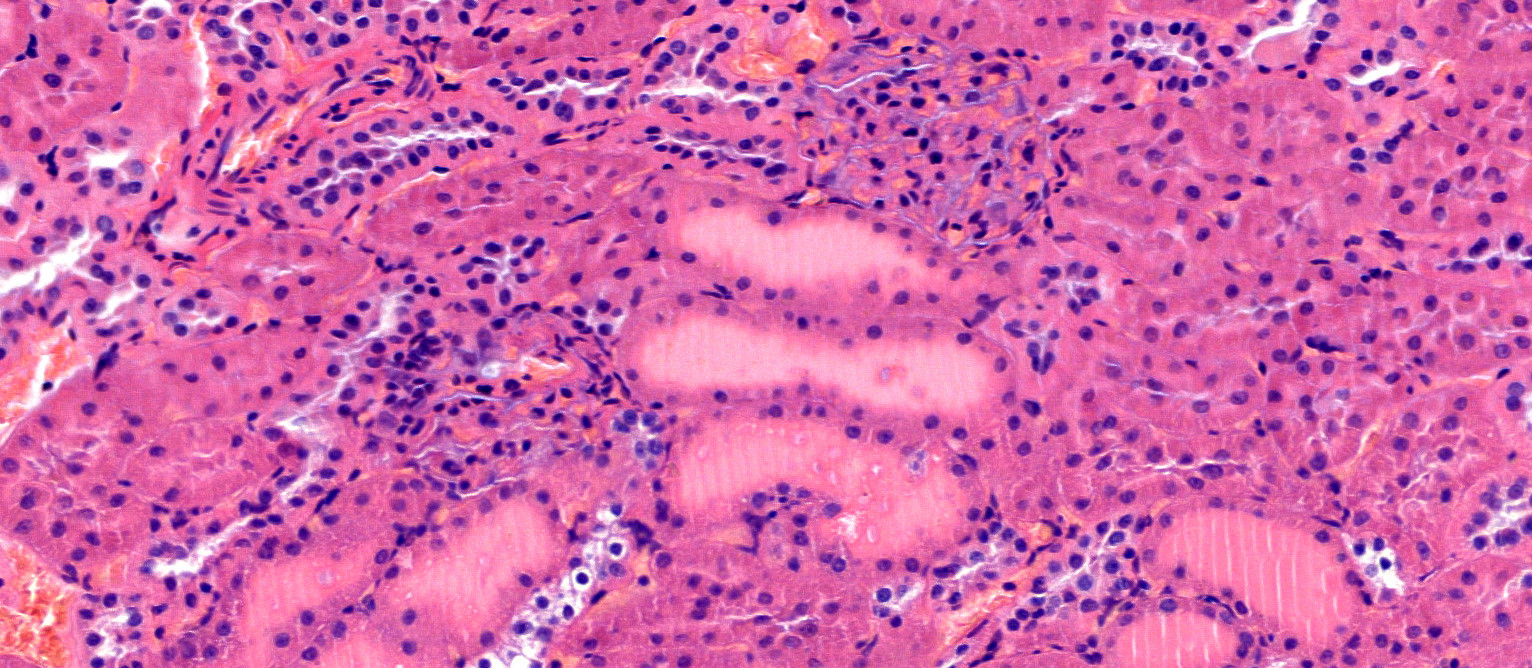

Supplement: Supplementary file 12 [file DataSheet2.ZIP › Fig 1D-HE-DKD-18/18-9.jpeg]

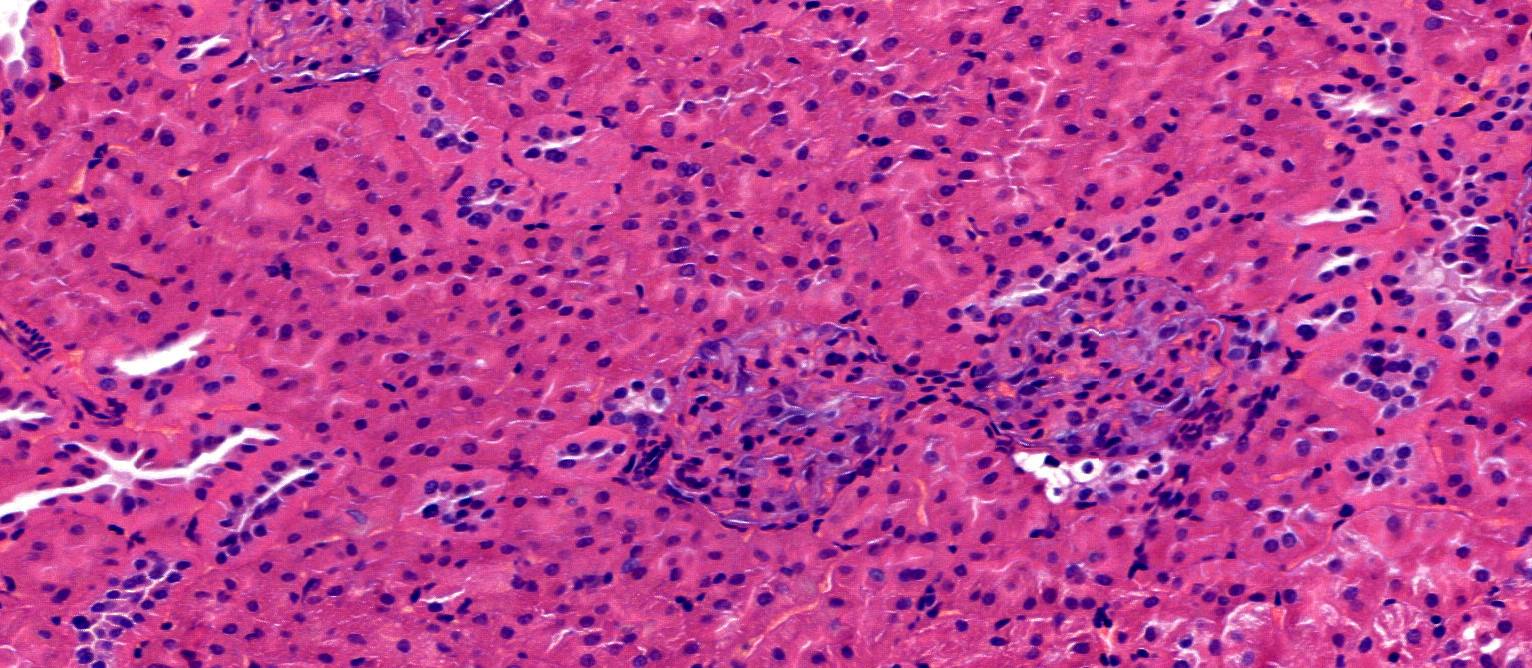

Supplement: Supplementary file 12 [file DataSheet2.ZIP › Fig 1D-HE-DKD-20/20-1.jpeg]

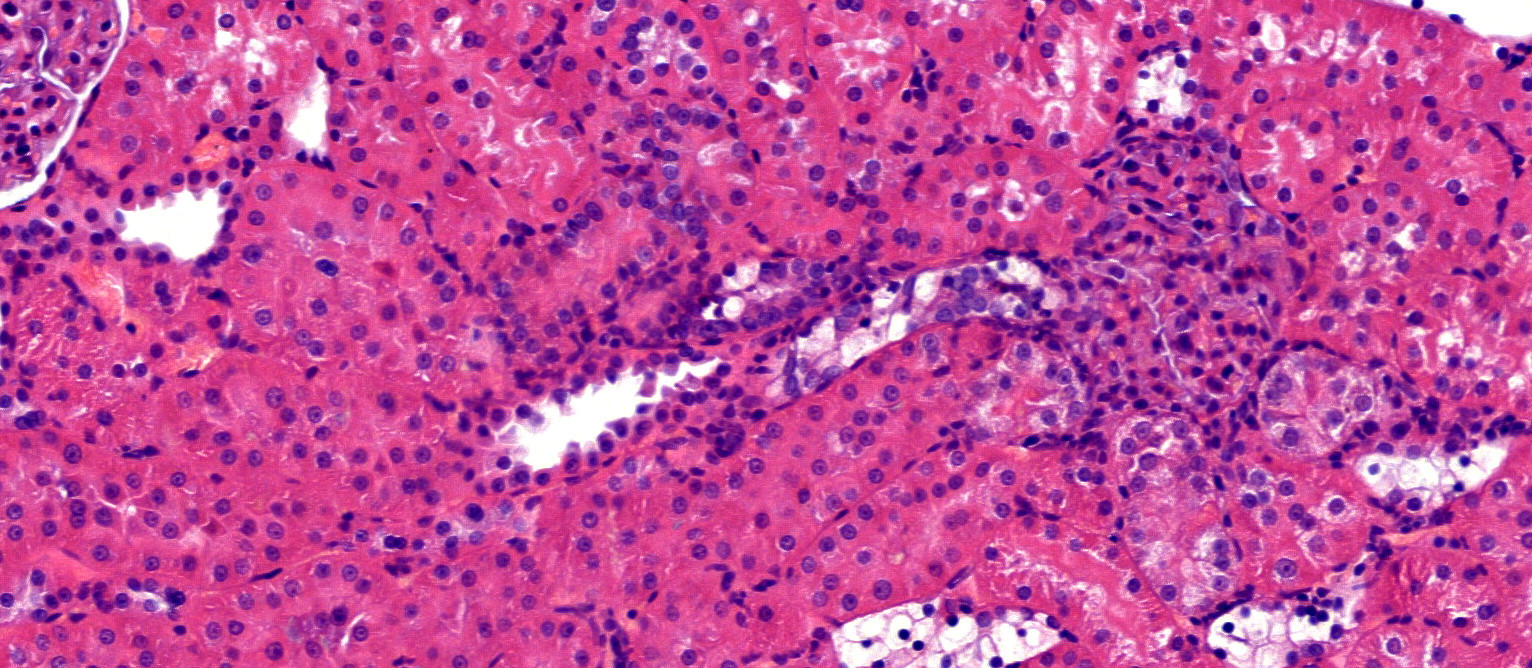

Supplement: Supplementary file 12 [file DataSheet2.ZIP › Fig 1D-HE-DKD-20/20-10.jpeg]

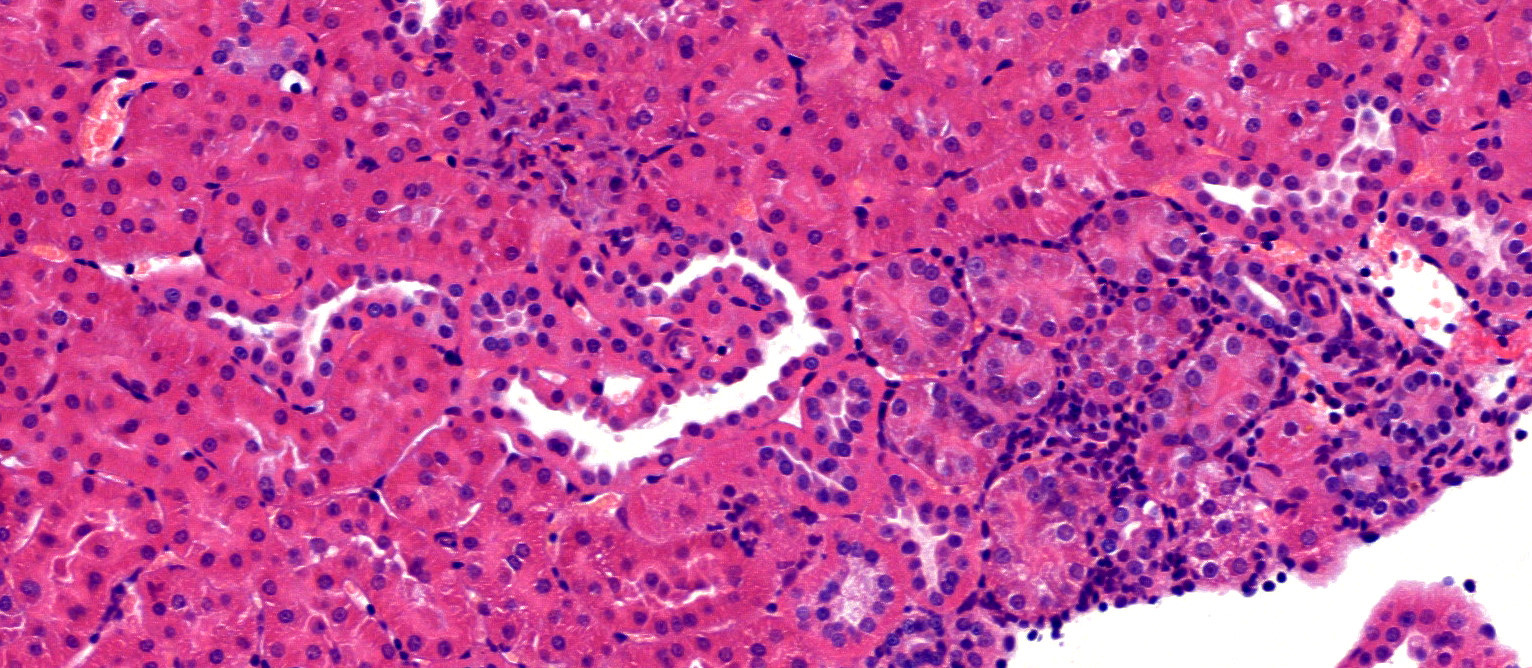

Supplement: Supplementary file 12 [file DataSheet2.ZIP › Fig 1D-HE-DKD-20/20-2.jpeg]

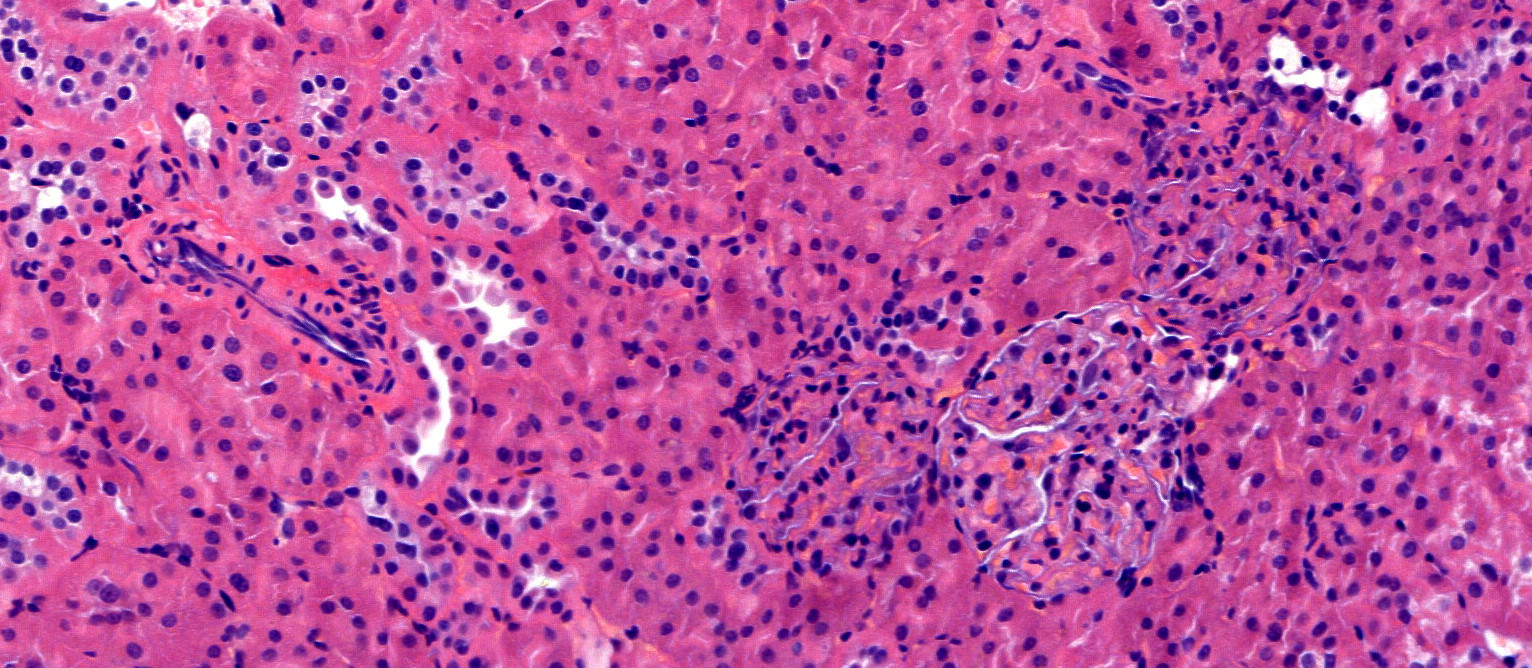

Supplement: Supplementary file 12 [file DataSheet2.ZIP › Fig 1D-HE-DKD-20/20-3.jpeg]

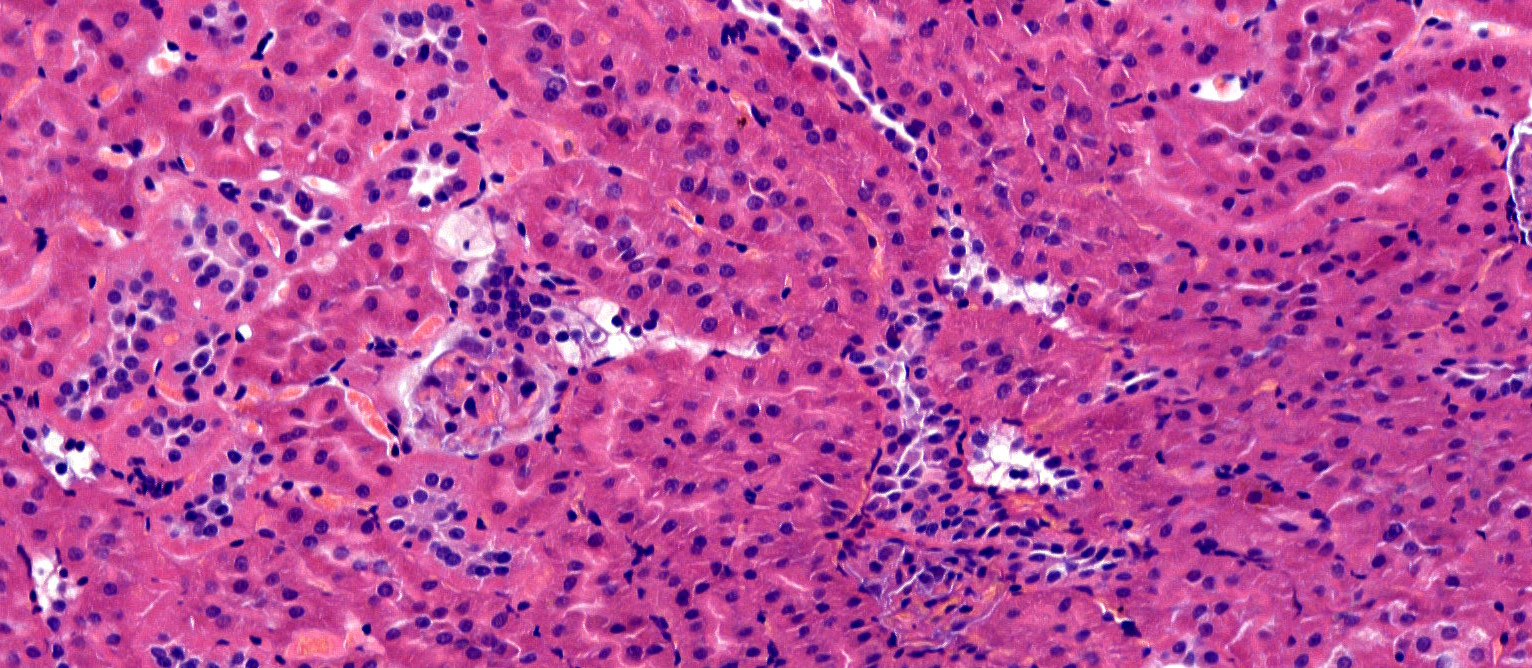

Supplement: Supplementary file 12 [file DataSheet2.ZIP › Fig 1D-HE-DKD-20/20-4.jpeg]

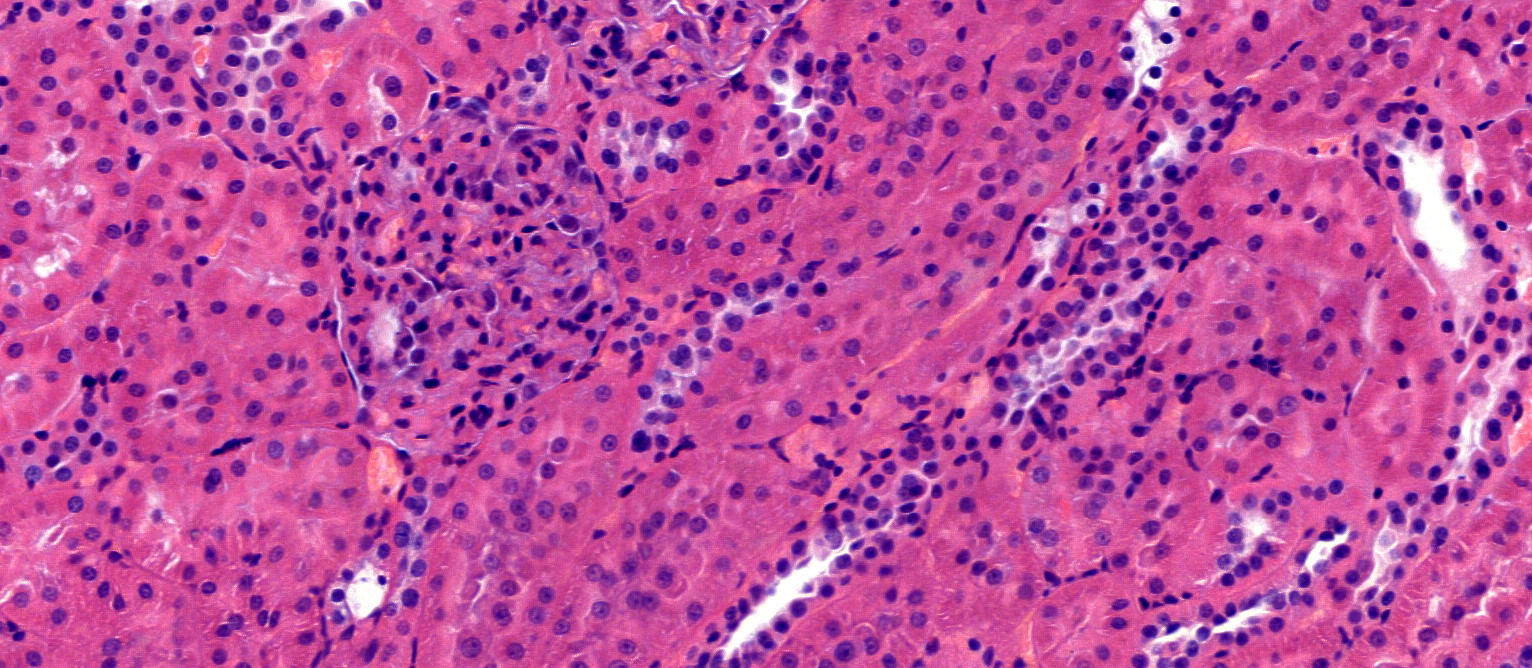

Supplement: Supplementary file 12 [file DataSheet2.ZIP › Fig 1D-HE-DKD-20/20-5.jpeg]

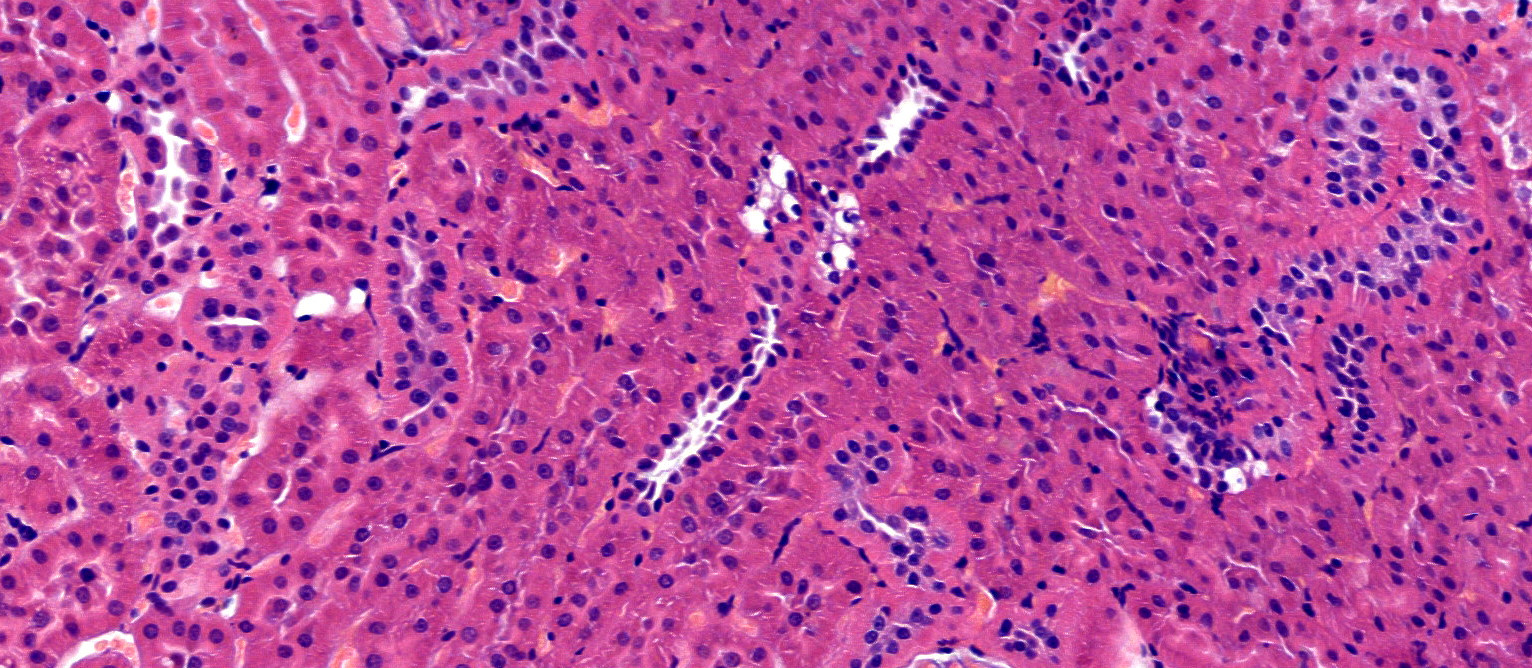

Supplement: Supplementary file 12 [file DataSheet2.ZIP › Fig 1D-HE-DKD-20/20-6.jpeg]

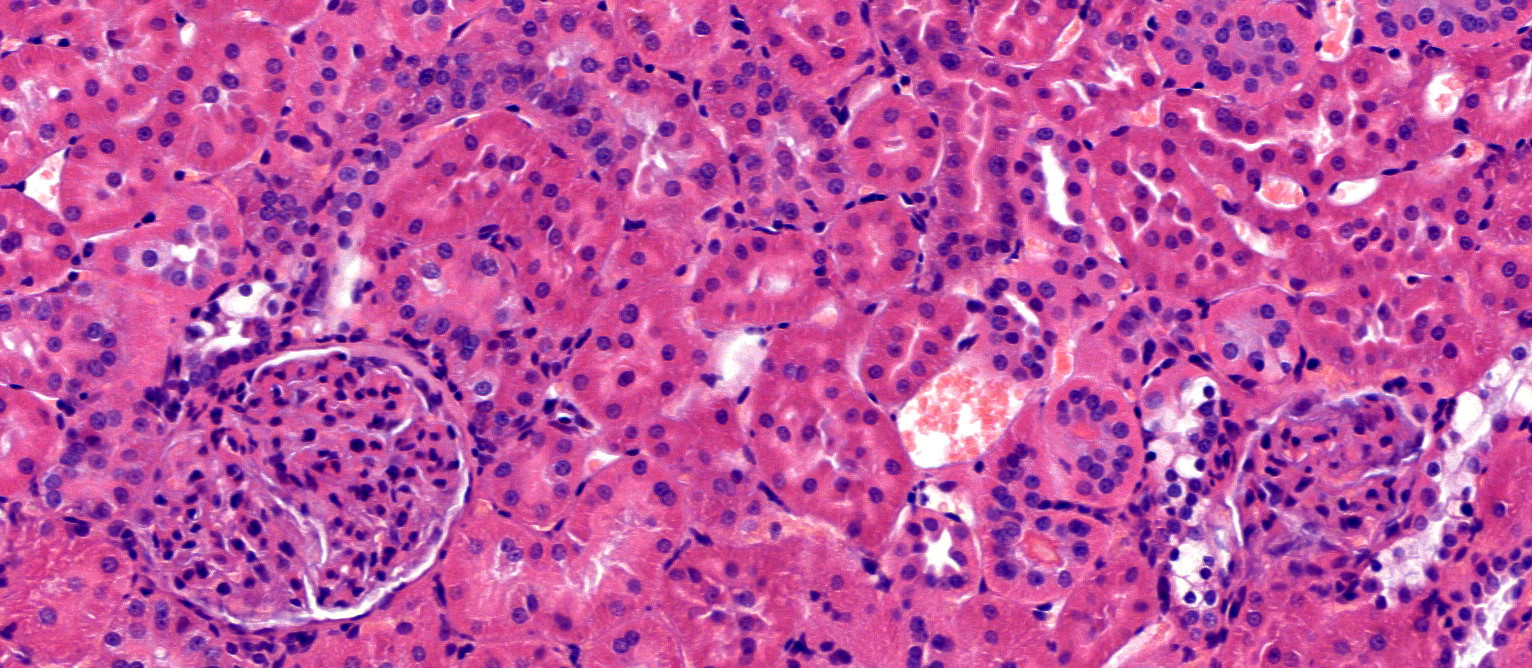

Supplement: Supplementary file 12 [file DataSheet2.ZIP › Fig 1D-HE-DKD-20/20-7.jpeg]

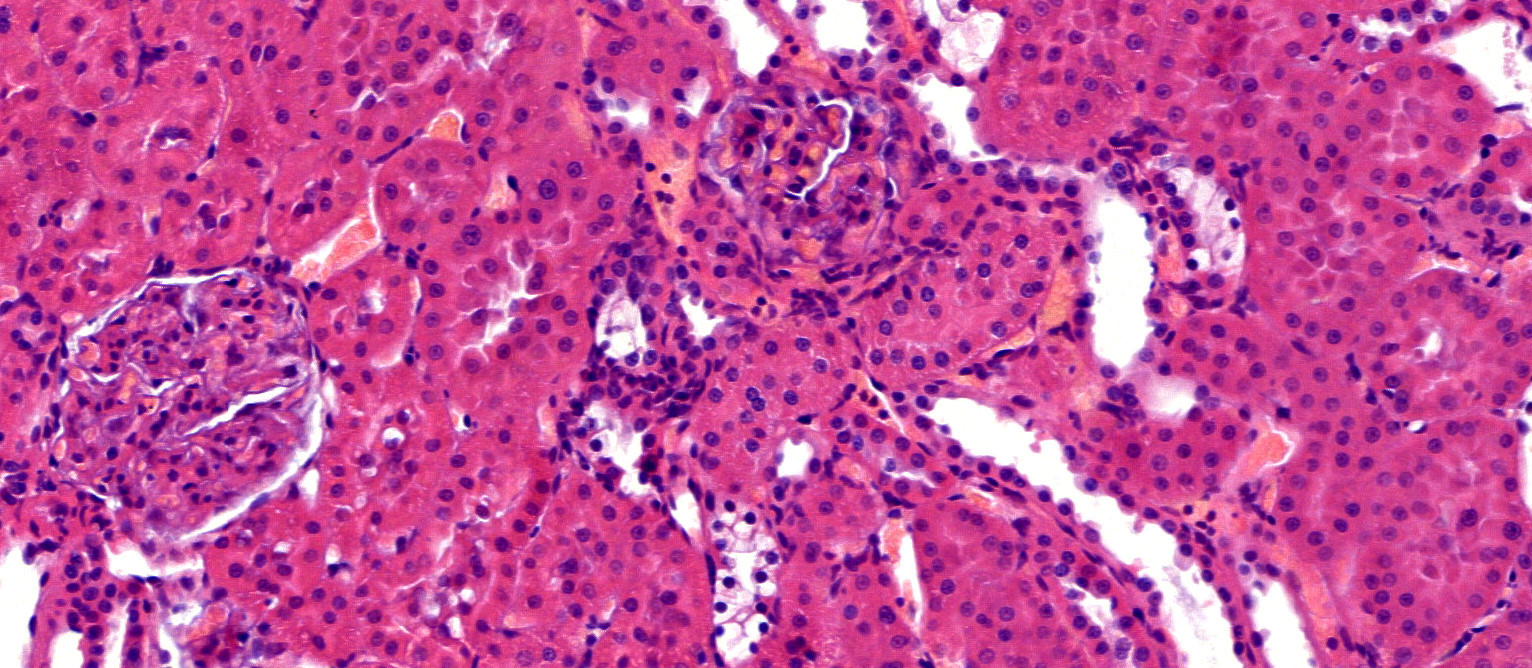

Supplement: Supplementary file 12 [file DataSheet2.ZIP › Fig 1D-HE-DKD-20/20-8.jpeg]

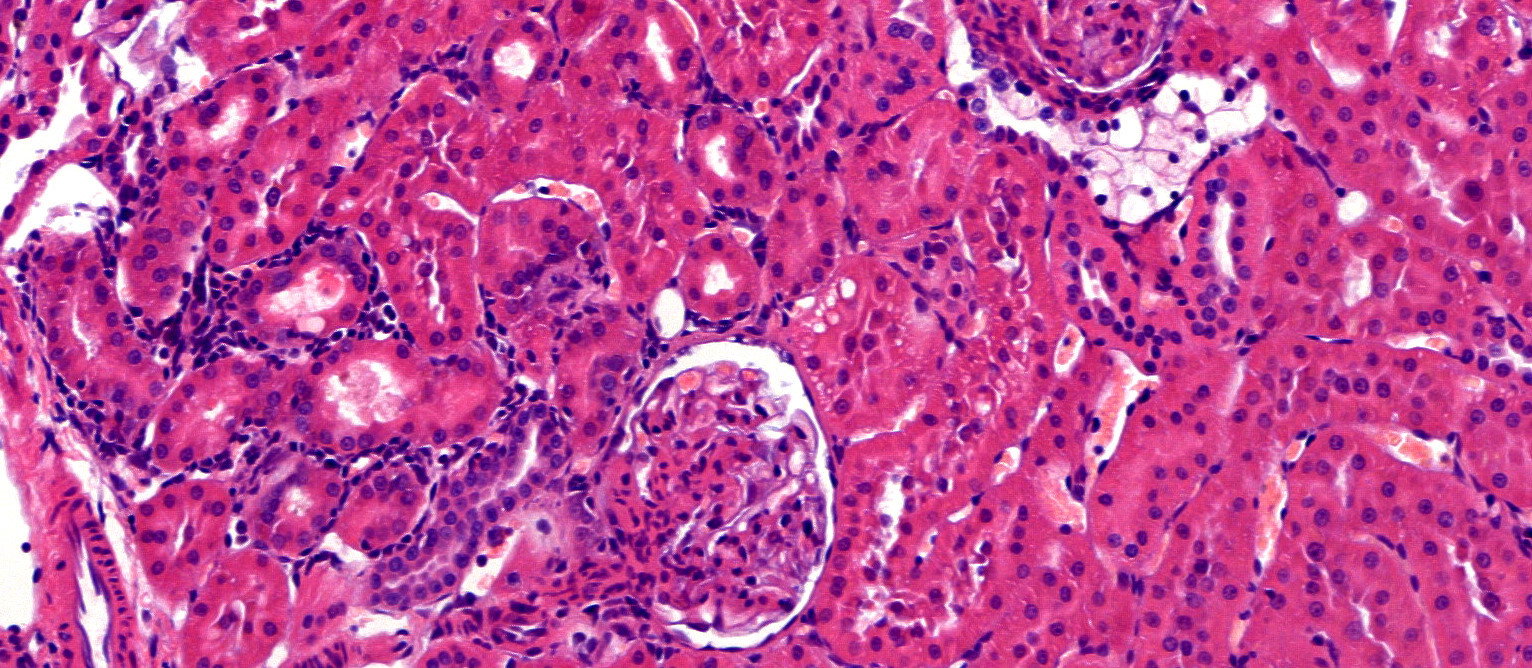

Supplement: Supplementary file 12 [file DataSheet2.ZIP › Fig 1D-HE-DKD-20/20-9.jpeg]

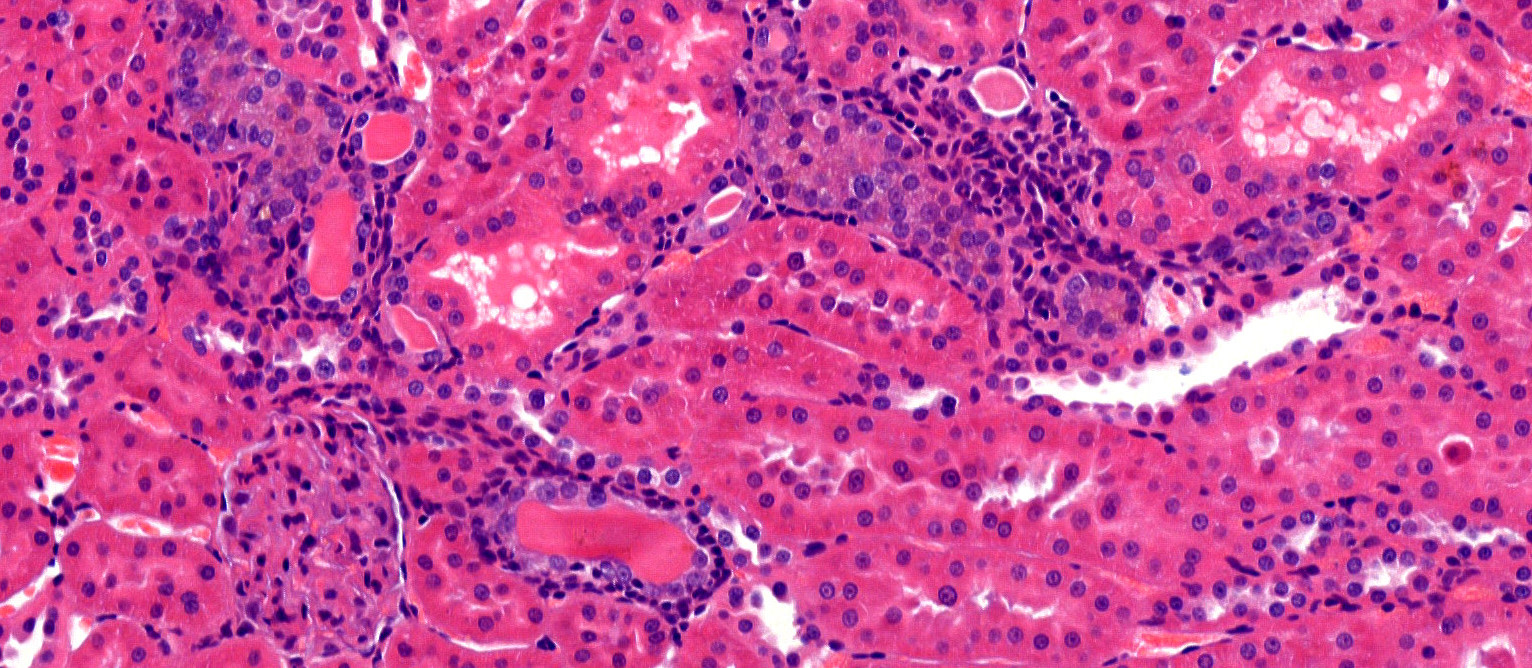

Supplement: Supplementary file 12 [file DataSheet2.ZIP › Fig 1D-HE-DKD-21/21-1.jpeg]

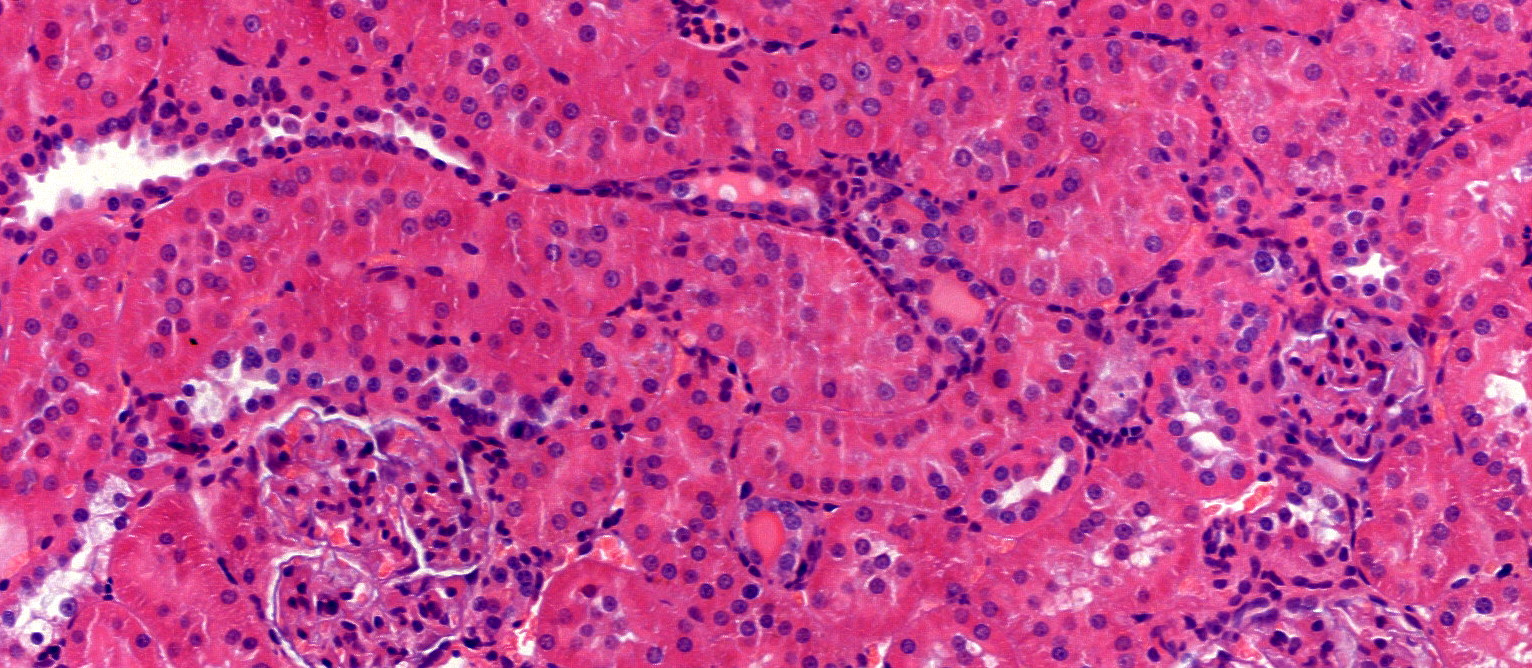

Supplement: Supplementary file 12 [file DataSheet2.ZIP › Fig 1D-HE-DKD-21/21-10.jpeg]

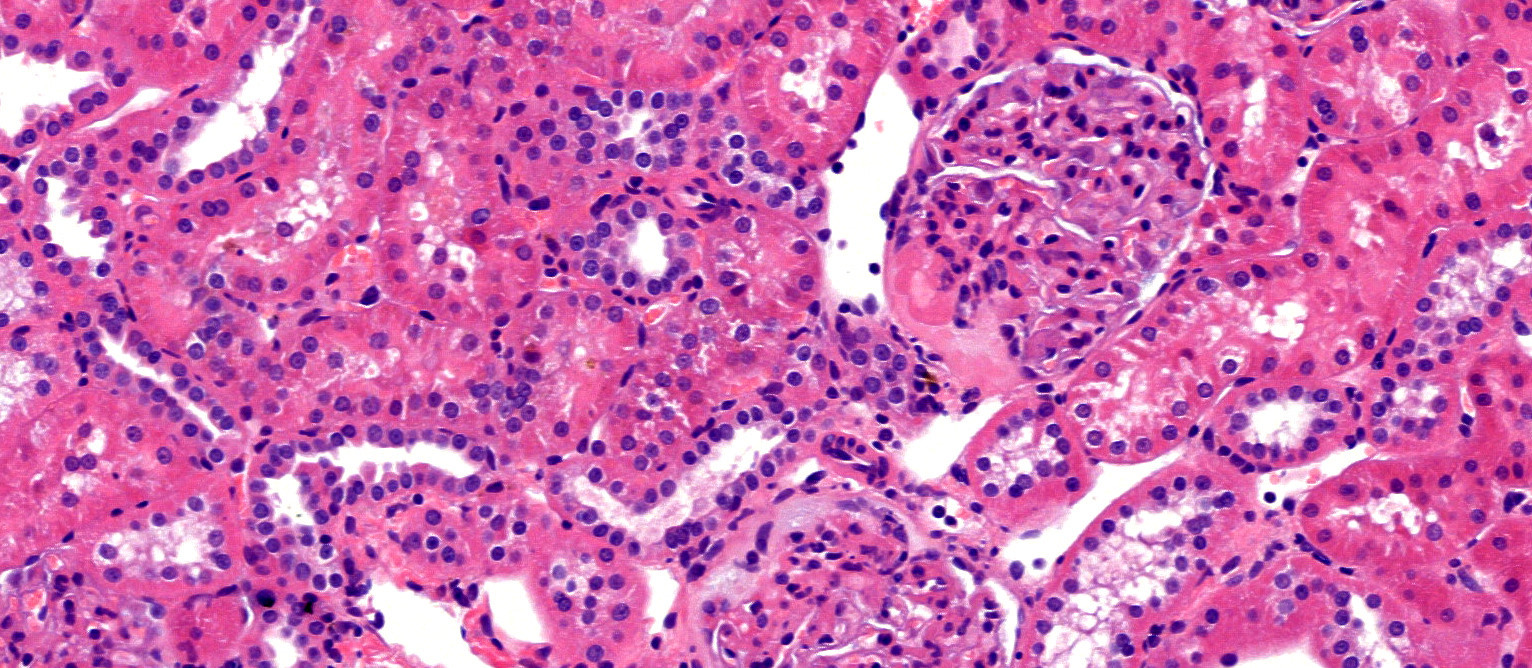

Supplement: Supplementary file 12 [file DataSheet2.ZIP › Fig 1D-HE-DKD-21/21-2.jpeg]

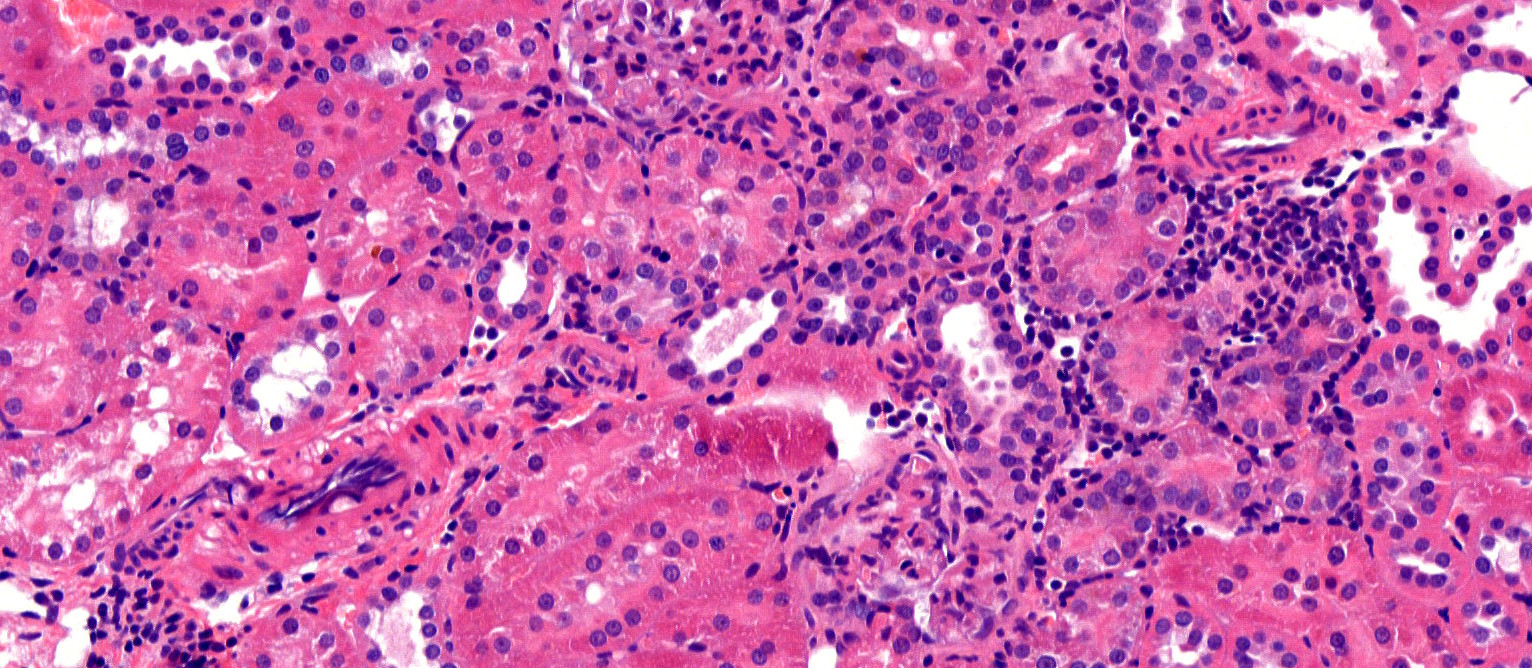

Supplement: Supplementary file 12 [file DataSheet2.ZIP › Fig 1D-HE-DKD-21/21-3.jpeg]

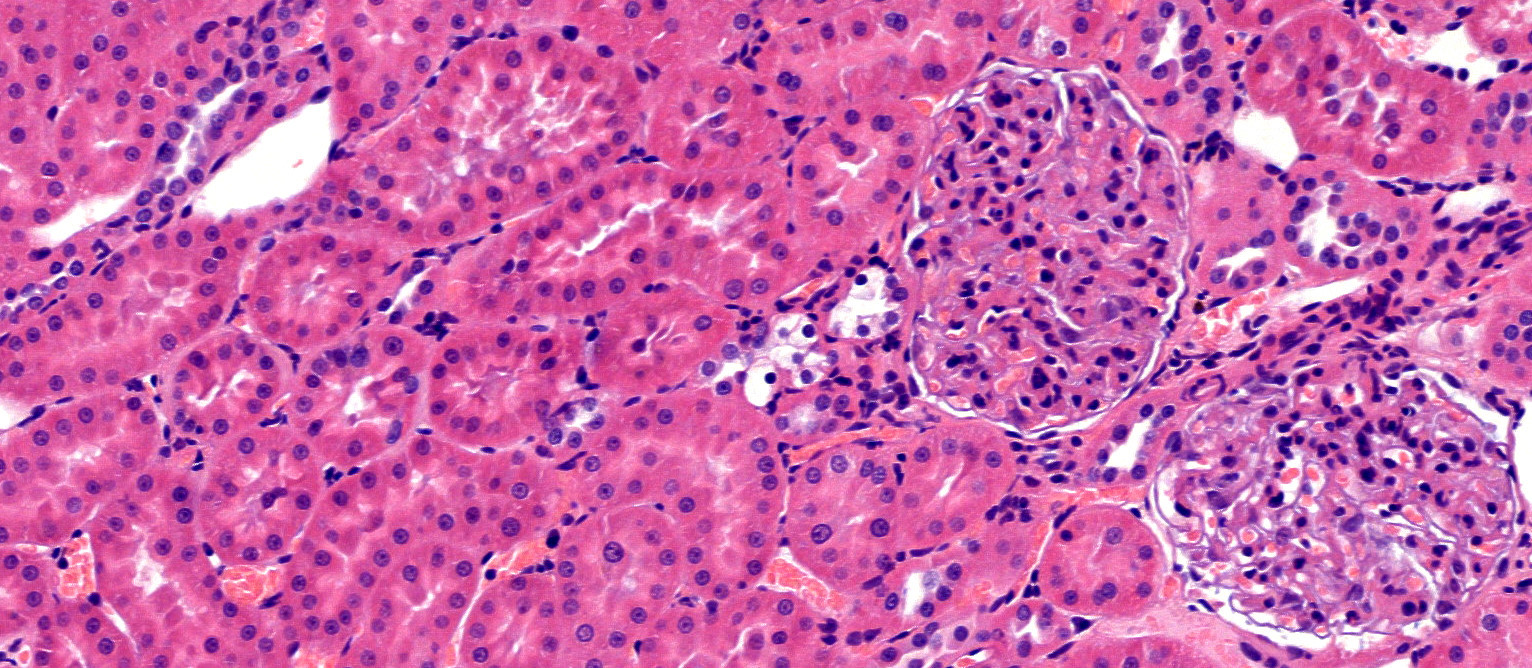

Supplement: Supplementary file 12 [file DataSheet2.ZIP › Fig 1D-HE-DKD-21/21-4.jpeg]

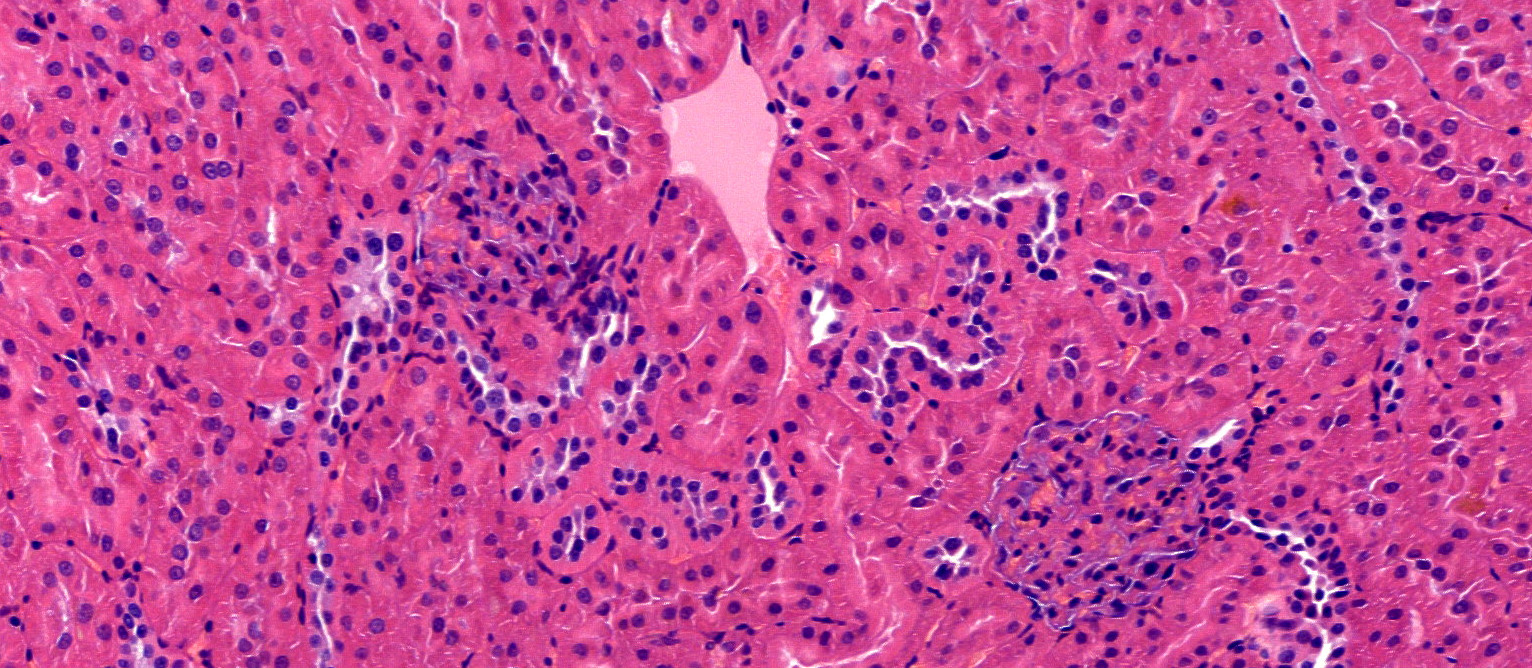

Supplement: Supplementary file 12 [file DataSheet2.ZIP › Fig 1D-HE-DKD-21/21-5.jpeg]

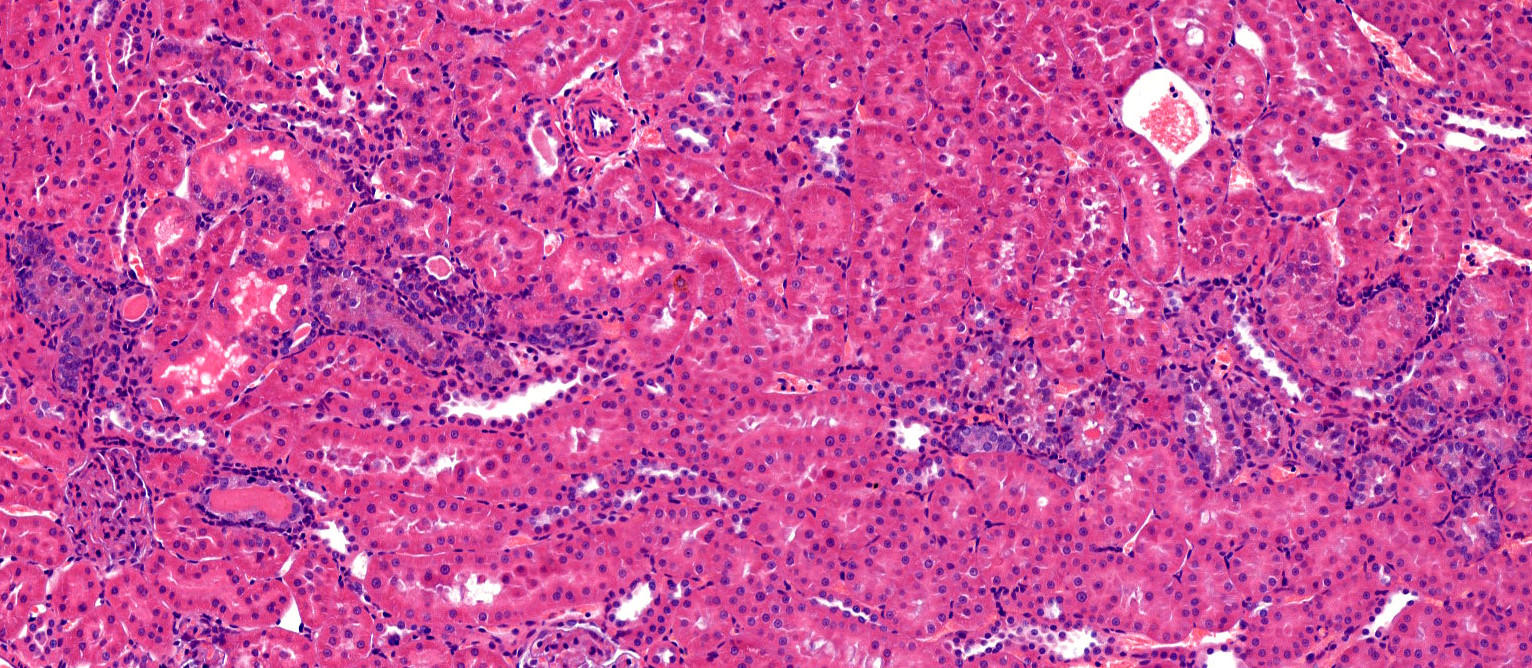

Supplement: Supplementary file 12 [file DataSheet2.ZIP › Fig 1D-HE-DKD-21/21-6.jpeg]

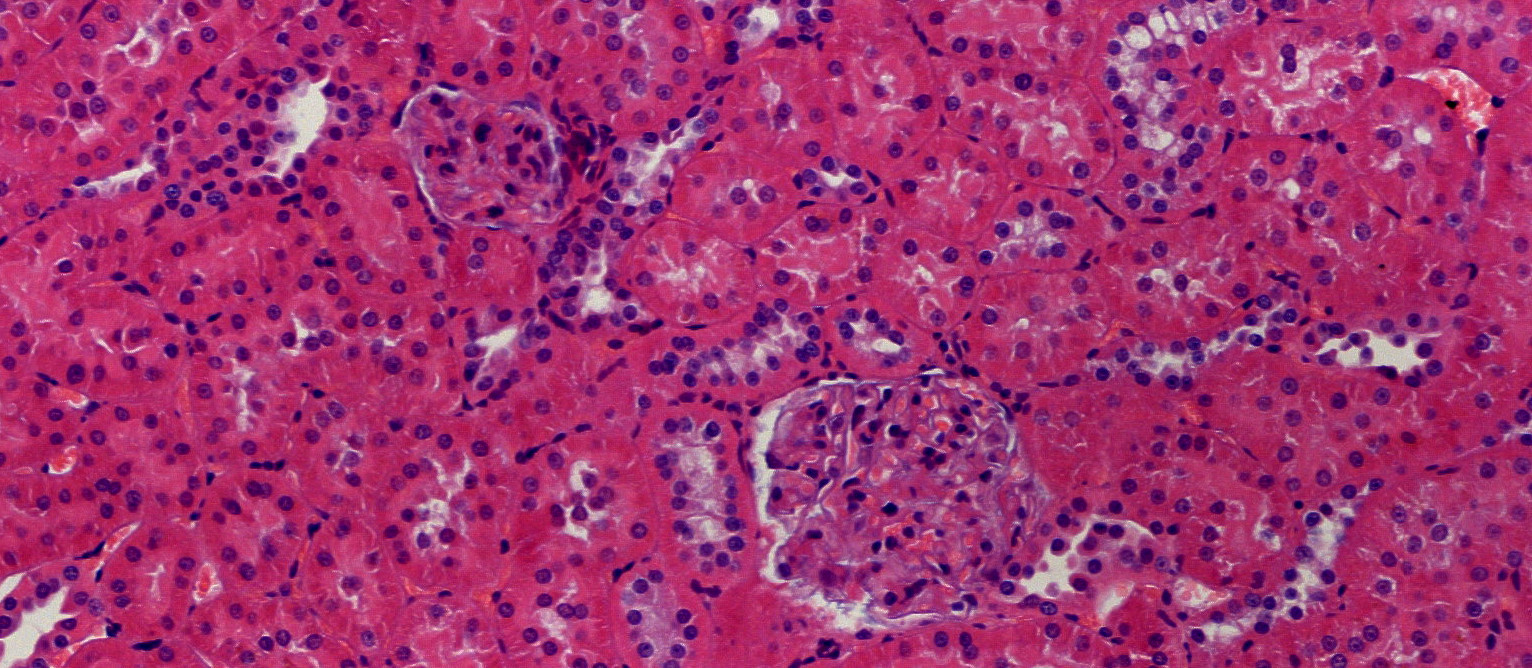

Supplement: Supplementary file 12 [file DataSheet2.ZIP › Fig 1D-HE-DKD-21/21-7.jpeg]

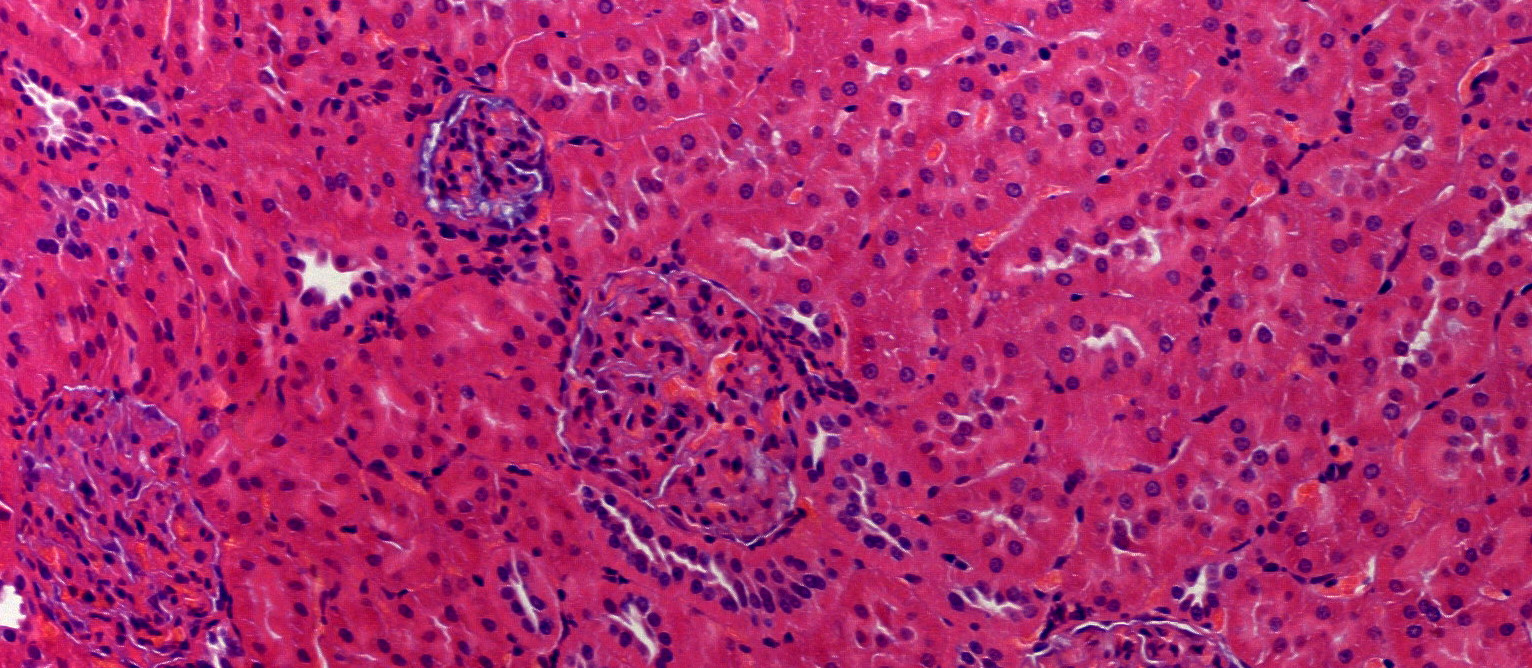

Supplement: Supplementary file 12 [file DataSheet2.ZIP › Fig 1D-HE-DKD-21/21-8.jpeg]

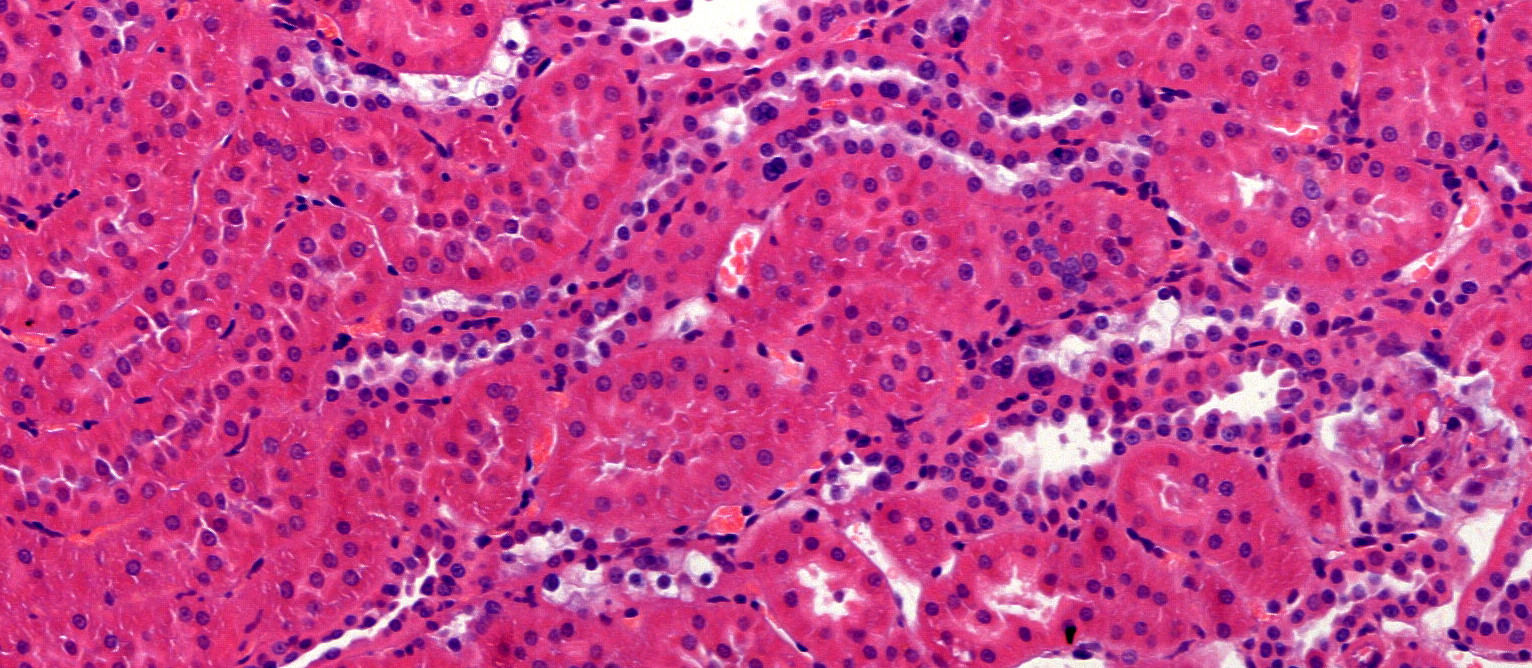

Supplement: Supplementary file 12 [file DataSheet2.ZIP › Fig 1D-HE-DKD-21/21-9.jpeg]

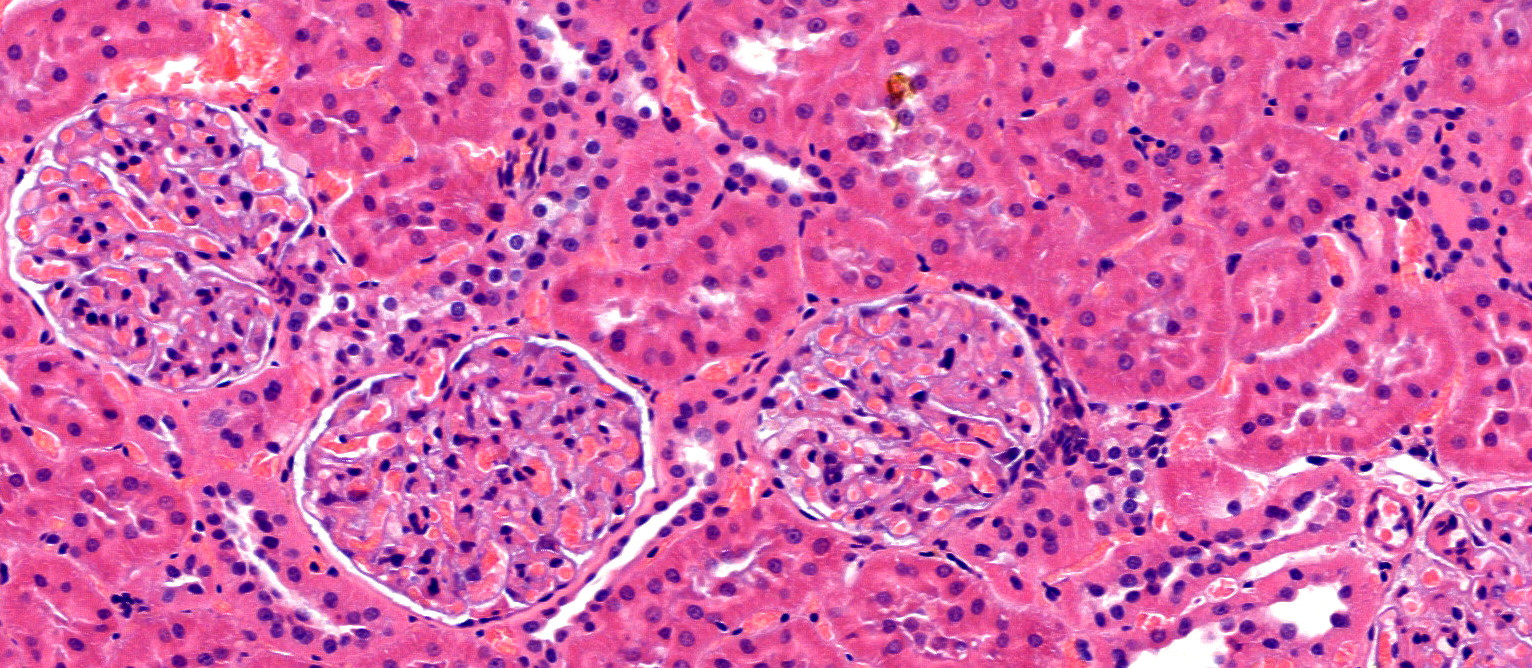

Supplement: Supplementary file 12 [file DataSheet2.ZIP › Fig 1D-HE-DKD-22/22-1.jpeg]

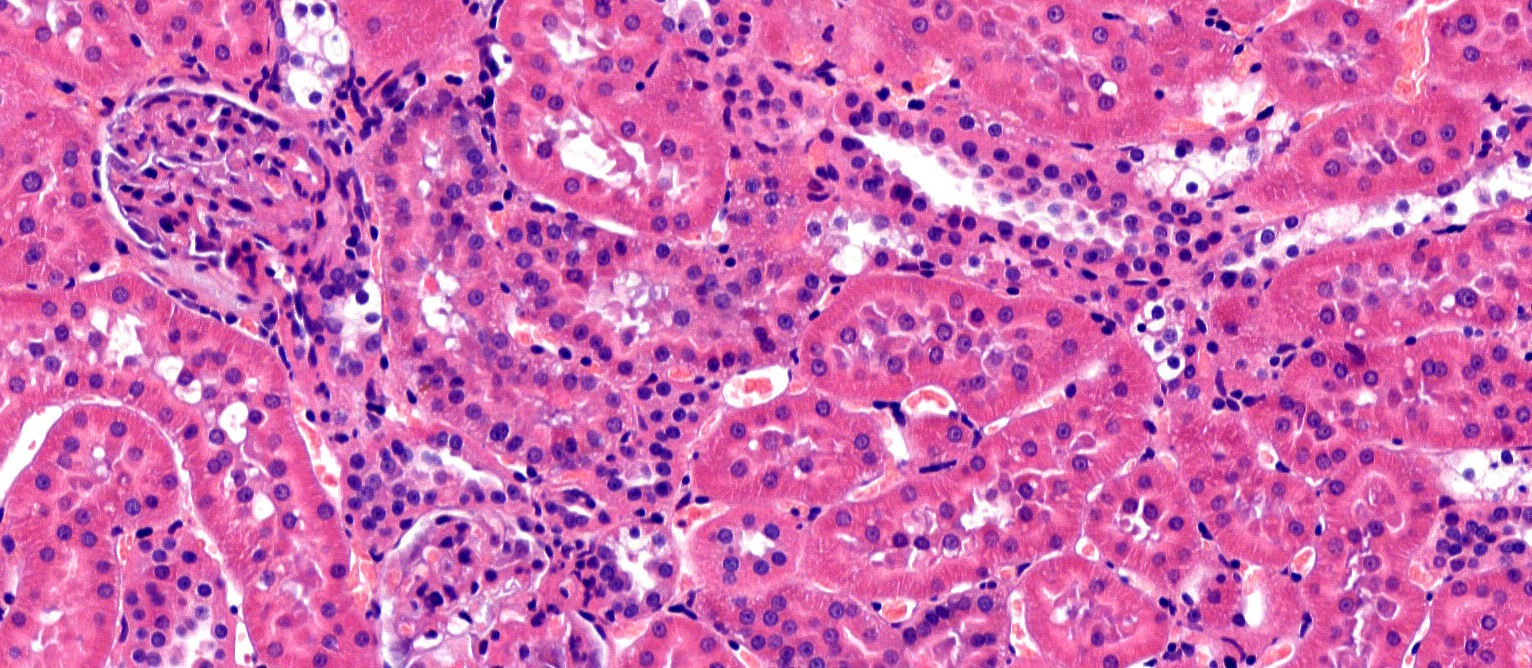

Supplement: Supplementary file 12 [file DataSheet2.ZIP › Fig 1D-HE-DKD-22/22-10.jpeg]

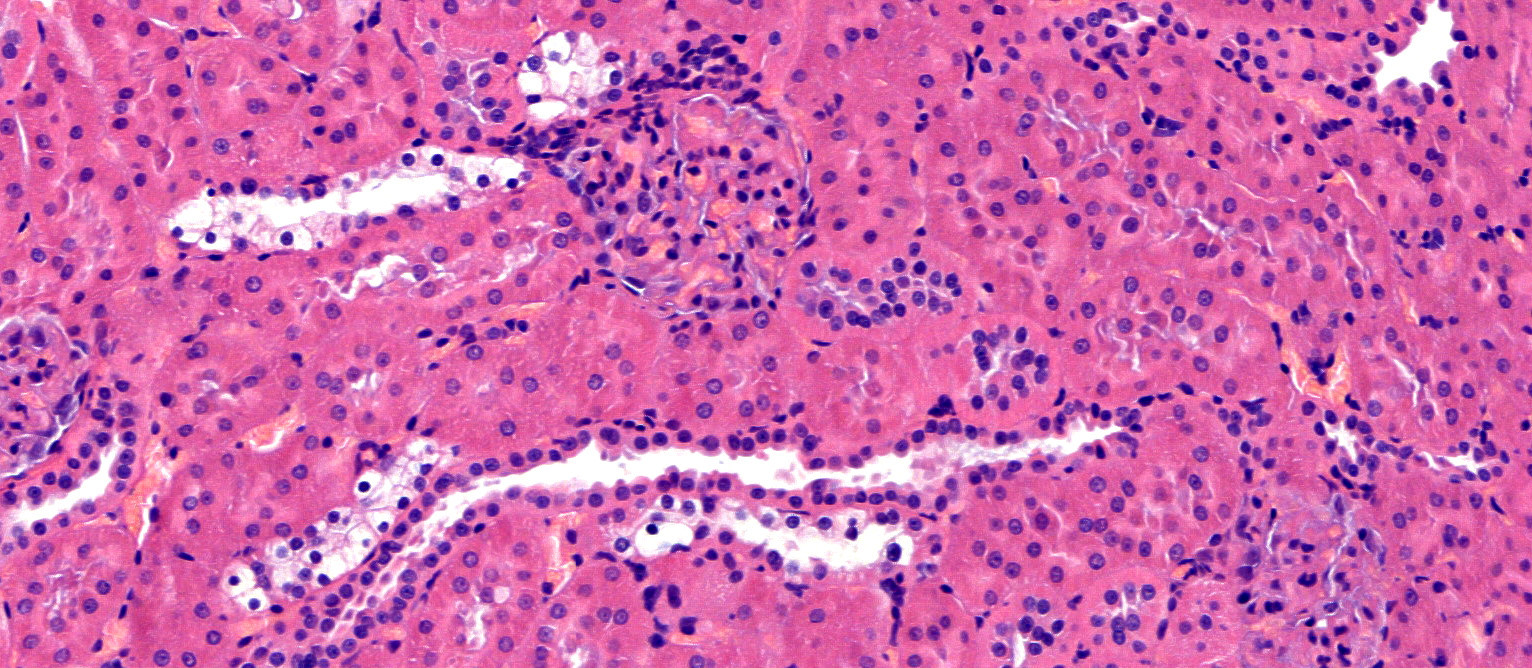

Supplement: Supplementary file 12 [file DataSheet2.ZIP › Fig 1D-HE-DKD-22/22-2.jpeg]

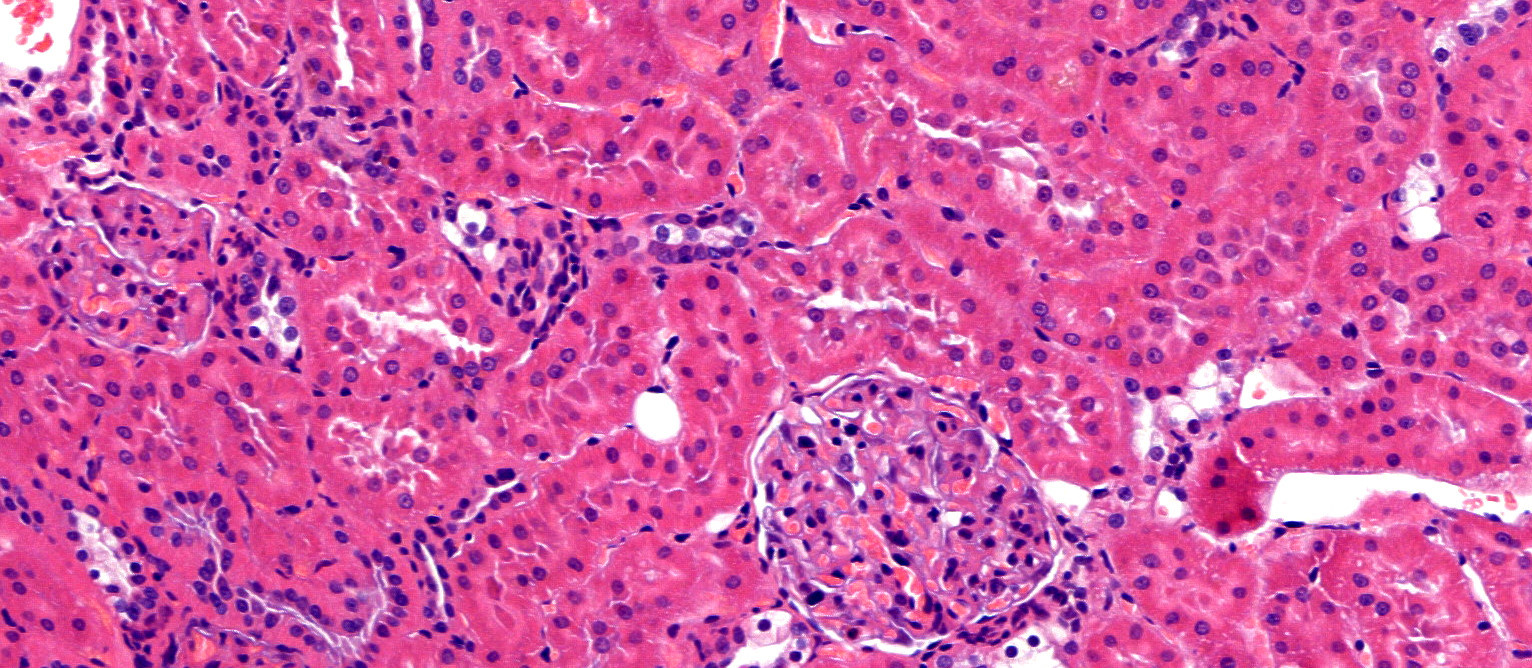

Supplement: Supplementary file 12 [file DataSheet2.ZIP › Fig 1D-HE-DKD-22/22-3.jpeg]

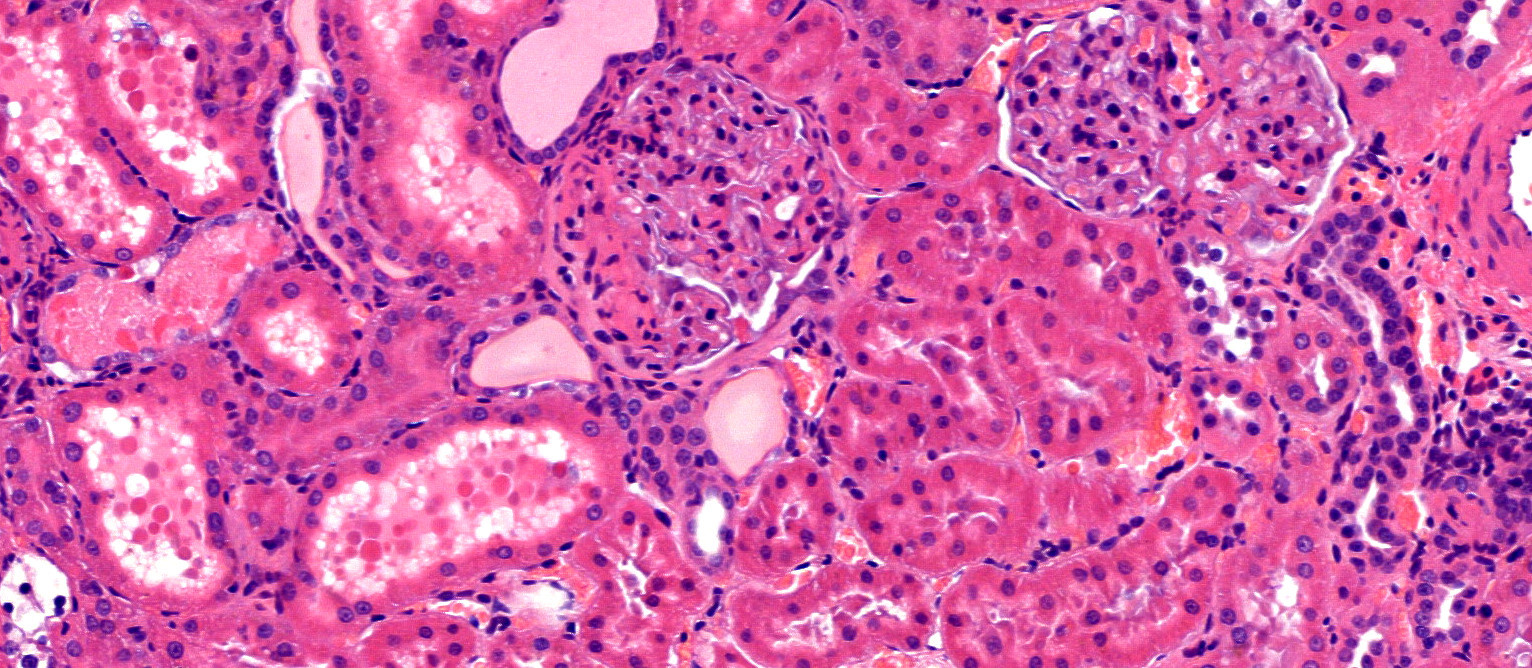

Supplement: Supplementary file 12 [file DataSheet2.ZIP › Fig 1D-HE-DKD-22/22-4.jpeg]

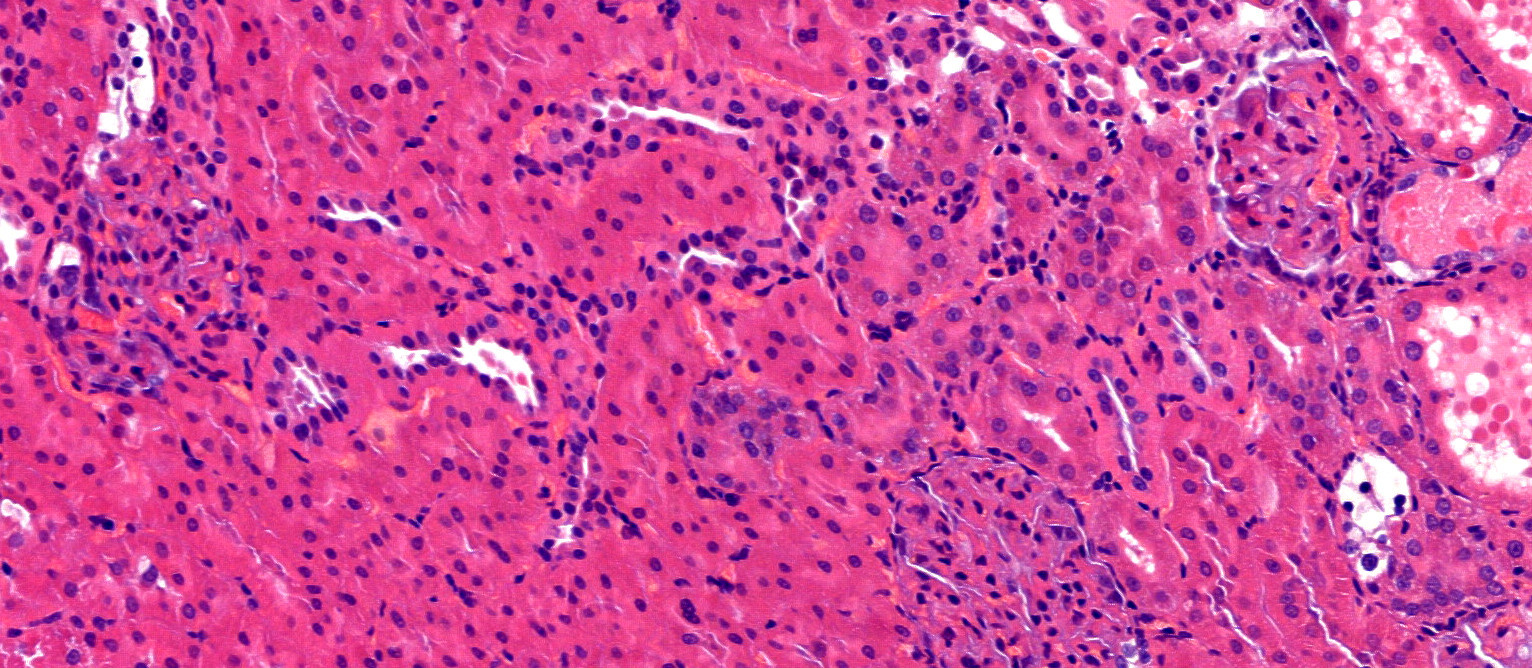

Supplement: Supplementary file 12 [file DataSheet2.ZIP › Fig 1D-HE-DKD-22/22-5.jpeg]

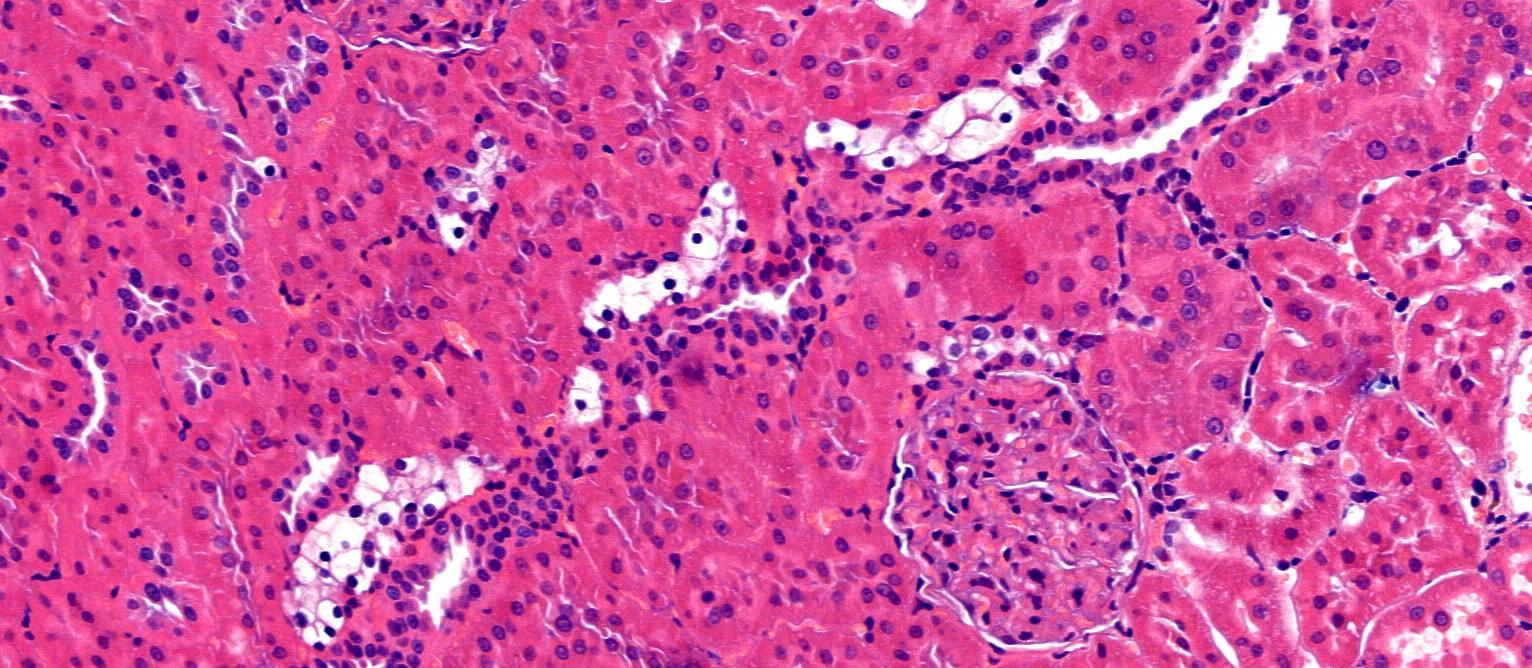

Supplement: Supplementary file 12 [file DataSheet2.ZIP › Fig 1D-HE-DKD-22/22-6.jpeg]

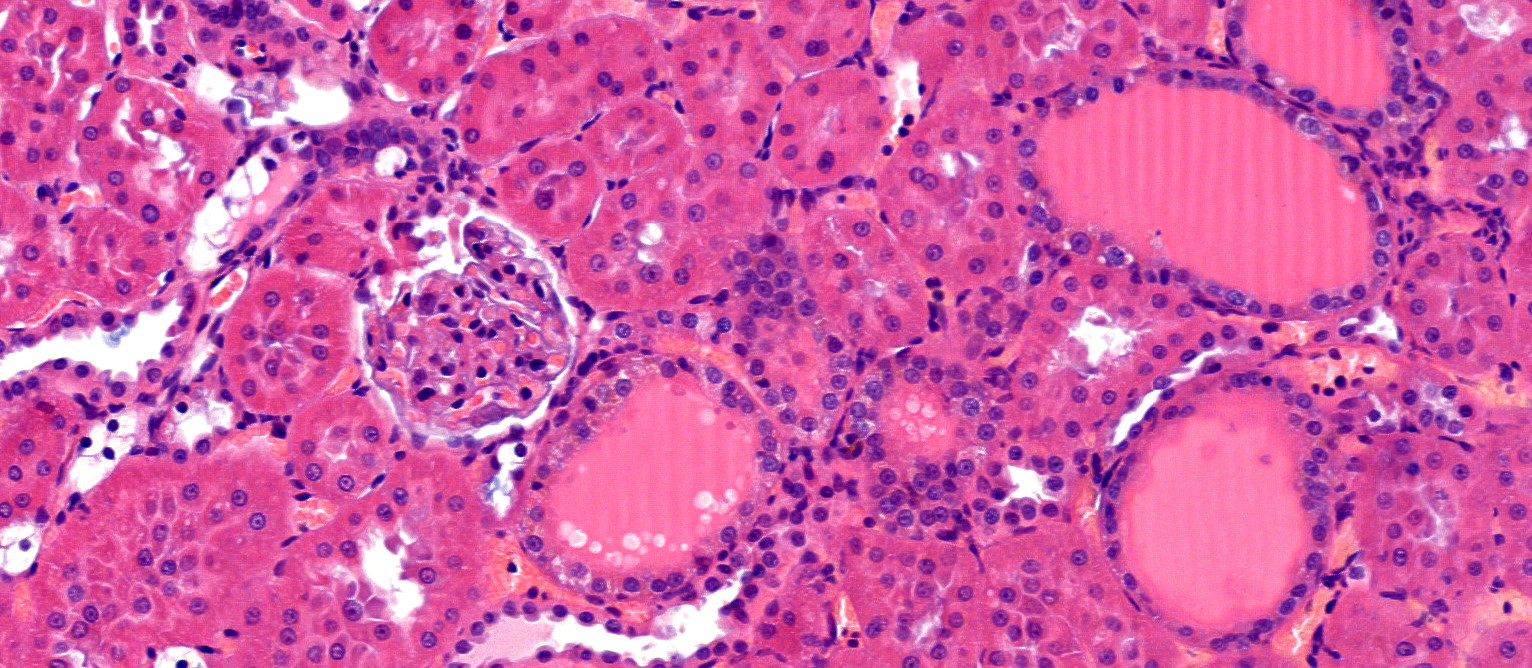

Supplement: Supplementary file 12 [file DataSheet2.ZIP › Fig 1D-HE-DKD-22/22-7.jpeg]

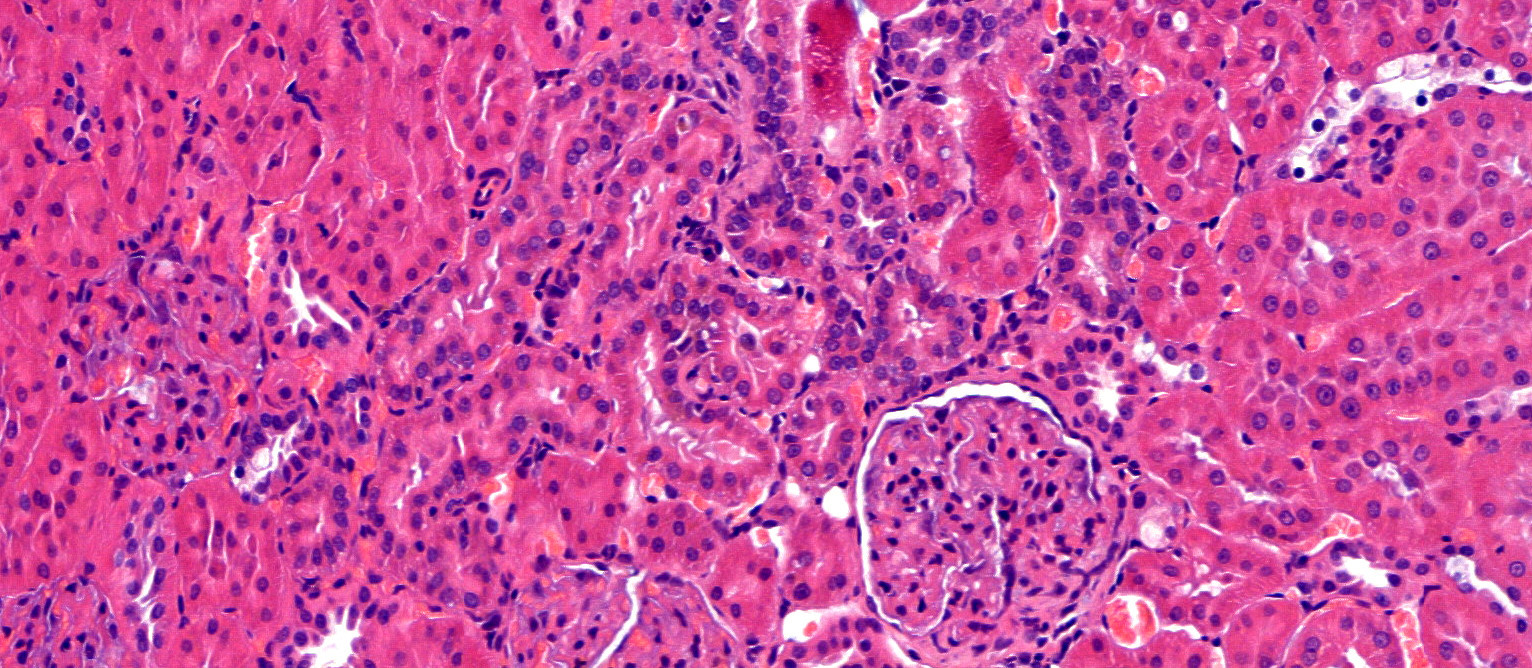

Supplement: Supplementary file 12 [file DataSheet2.ZIP › Fig 1D-HE-DKD-22/22-8.jpeg]

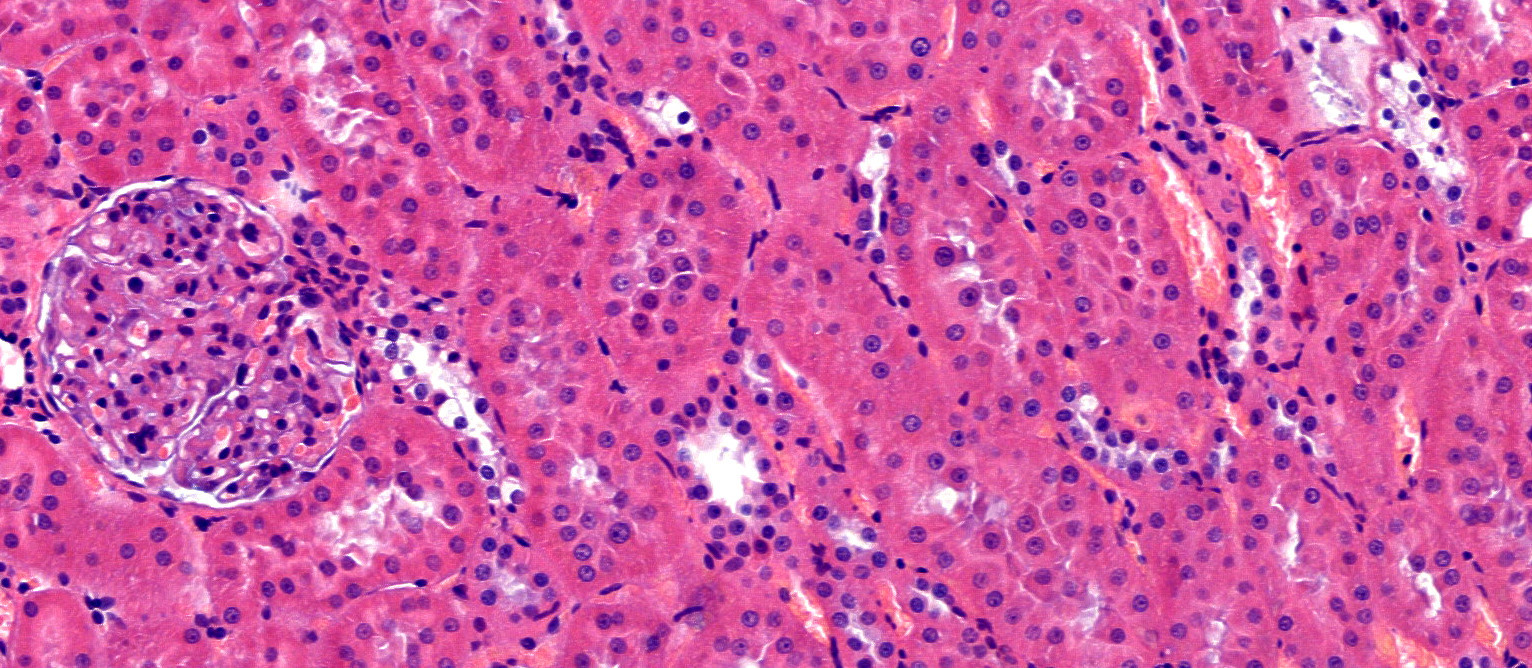

Supplement: Supplementary file 12 [file DataSheet2.ZIP › Fig 1D-HE-DKD-22/22-9.jpeg]

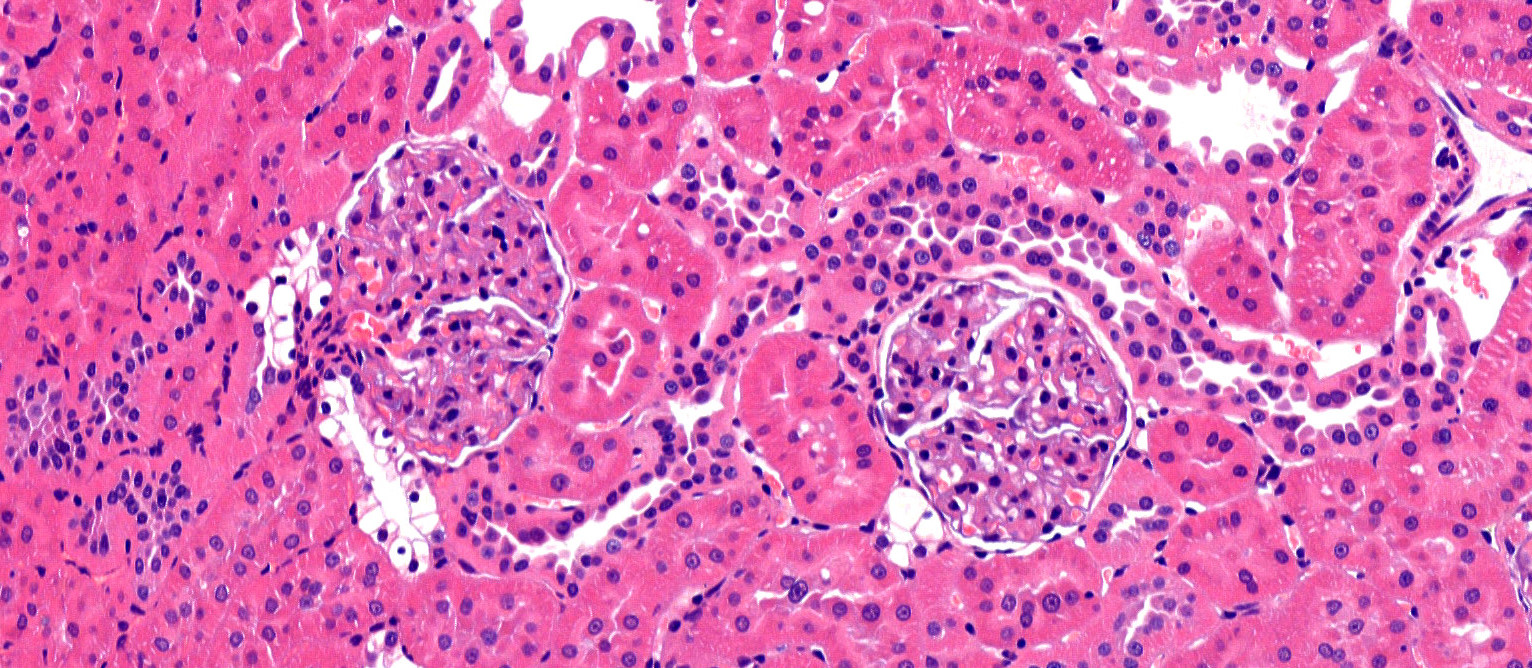

Supplement: Supplementary file 12 [file DataSheet2.ZIP › Fig 1D-HE-DKD-23(1)/23-1.jpeg]

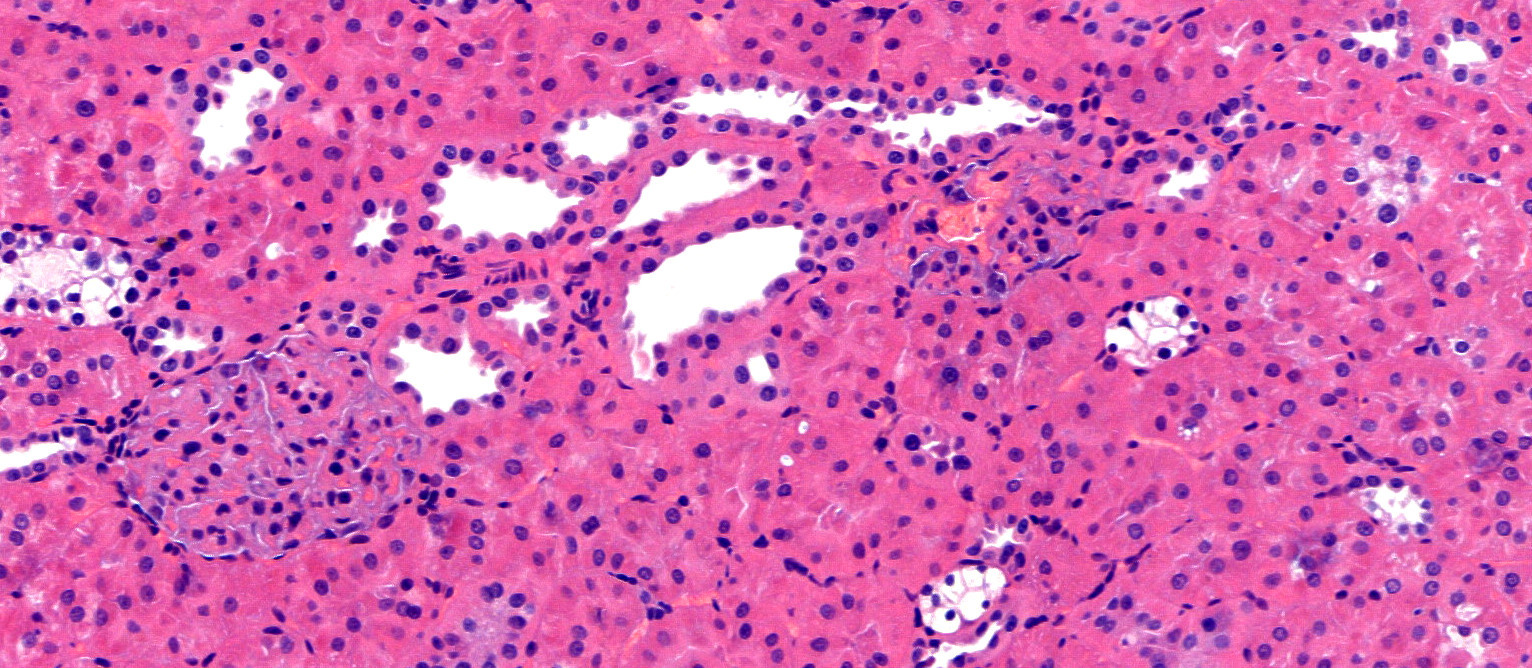

Supplement: Supplementary file 12 [file DataSheet2.ZIP › Fig 1D-HE-DKD-23(1)/23-2.jpeg]

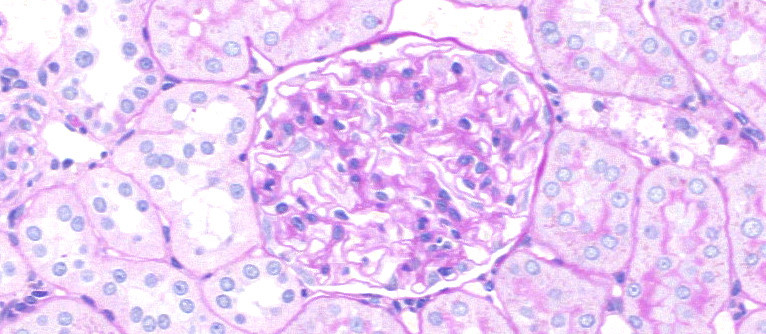

Supplement: Supplementary file 13 [file DataSheet15.ZIP › Fig 1D-PAS-TSF-58/58-1.jpeg]

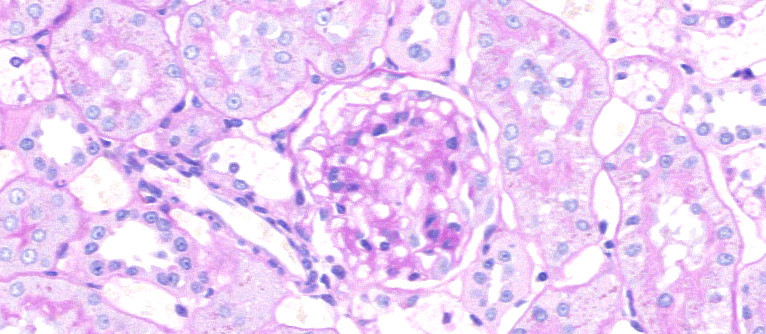

Supplement: Supplementary file 13 [file DataSheet15.ZIP › Fig 1D-PAS-TSF-58/58-10.jpeg]

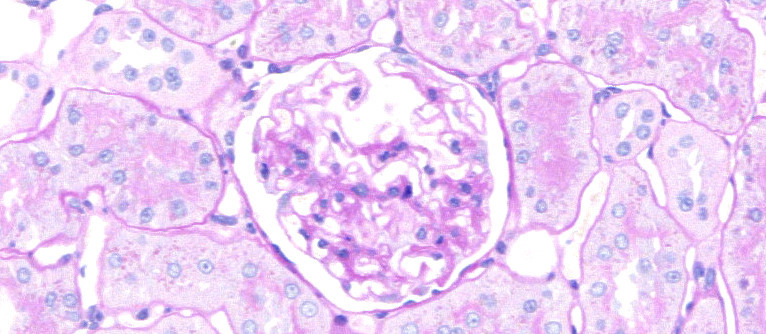

Supplement: Supplementary file 13 [file DataSheet15.ZIP › Fig 1D-PAS-TSF-58/58-11.jpeg]

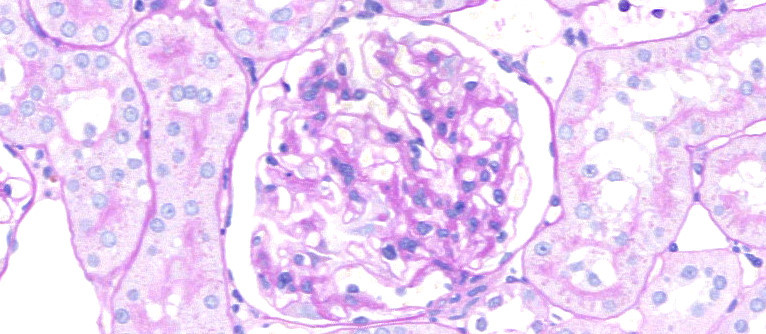

Supplement: Supplementary file 13 [file DataSheet15.ZIP › Fig 1D-PAS-TSF-58/58-12.jpeg]

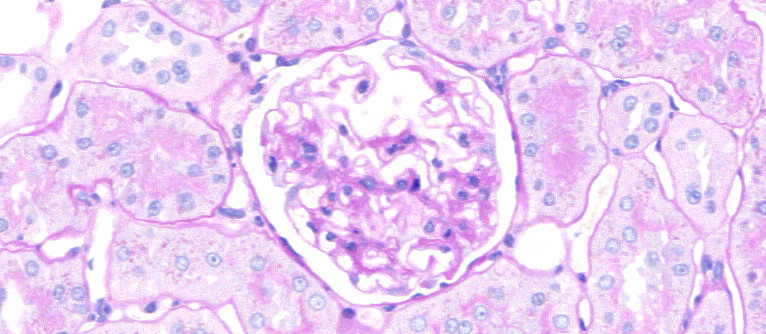

Supplement: Supplementary file 13 [file DataSheet15.ZIP › Fig 1D-PAS-TSF-58/58-13.jpeg]

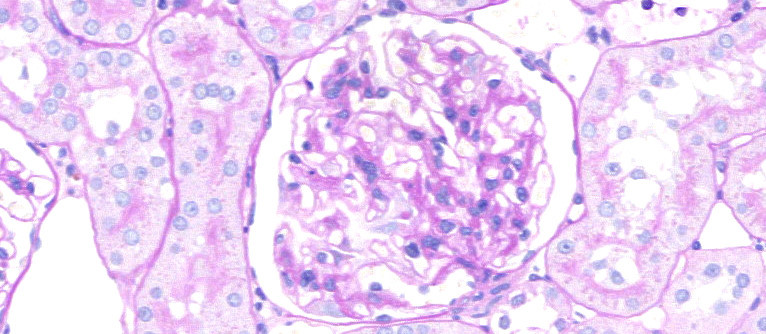

Supplement: Supplementary file 13 [file DataSheet15.ZIP › Fig 1D-PAS-TSF-58/58-14.jpeg]

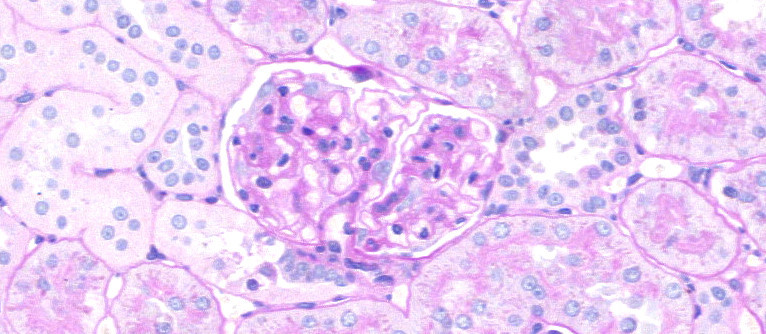

Supplement: Supplementary file 13 [file DataSheet15.ZIP › Fig 1D-PAS-TSF-58/58-15.jpeg]

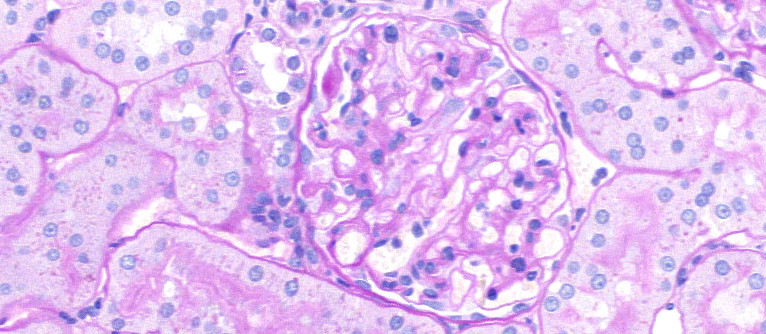

Supplement: Supplementary file 13 [file DataSheet15.ZIP › Fig 1D-PAS-TSF-58/58-16.jpeg]

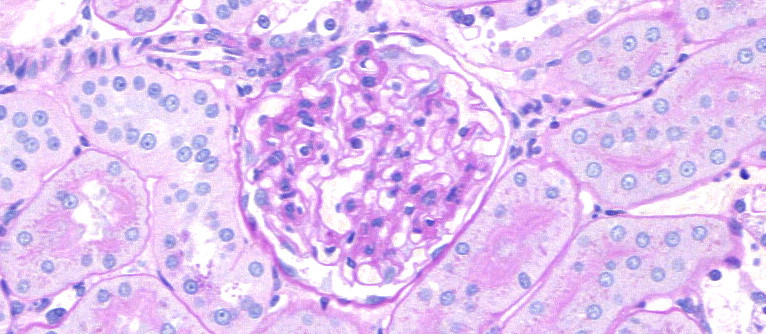

Supplement: Supplementary file 13 [file DataSheet15.ZIP › Fig 1D-PAS-TSF-58/58-17.jpeg]

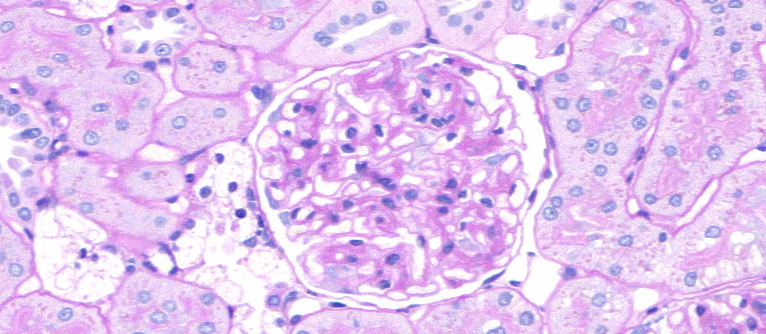

Supplement: Supplementary file 13 [file DataSheet15.ZIP › Fig 1D-PAS-TSF-58/58-18.jpeg]

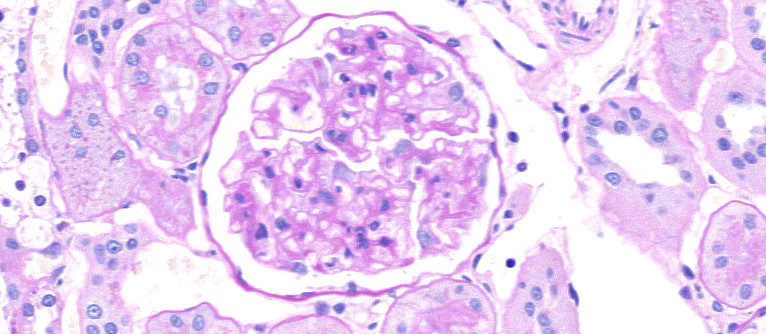

Supplement: Supplementary file 13 [file DataSheet15.ZIP › Fig 1D-PAS-TSF-58/58-19.jpeg]

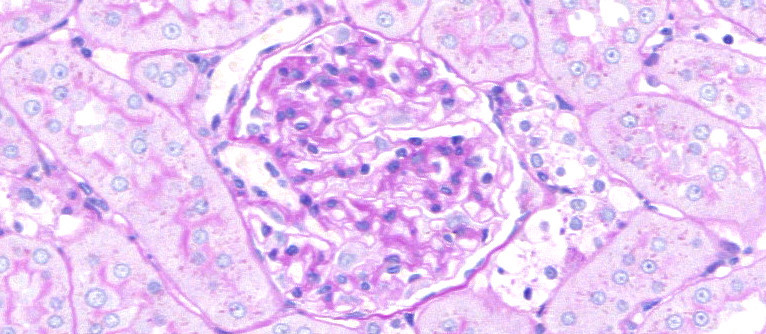

Supplement: Supplementary file 13 [file DataSheet15.ZIP › Fig 1D-PAS-TSF-58/58-2.jpeg]

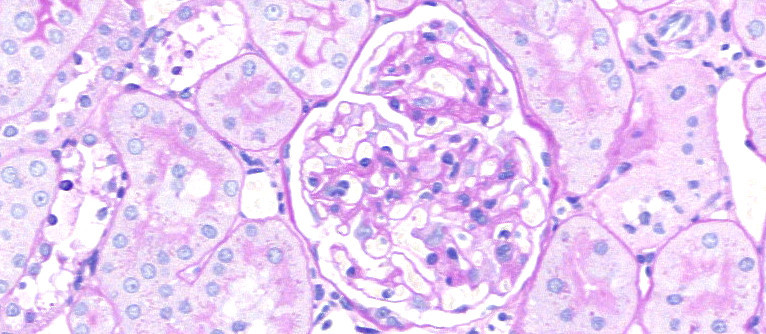

Supplement: Supplementary file 13 [file DataSheet15.ZIP › Fig 1D-PAS-TSF-58/58-20.jpeg]

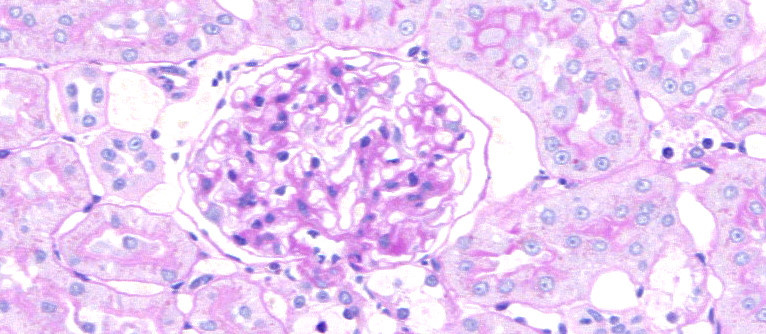

Supplement: Supplementary file 13 [file DataSheet15.ZIP › Fig 1D-PAS-TSF-58/58-3.jpeg]

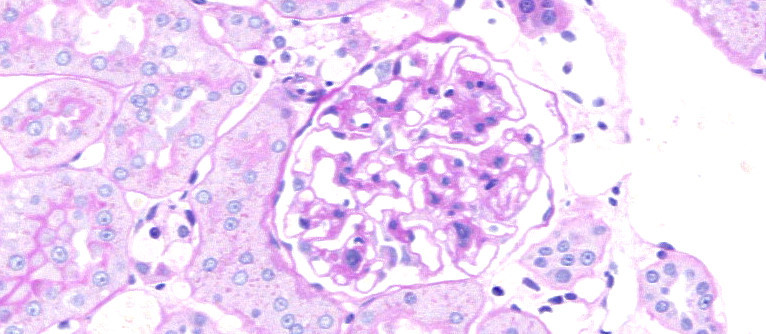

Supplement: Supplementary file 13 [file DataSheet15.ZIP › Fig 1D-PAS-TSF-58/58-4.jpeg]

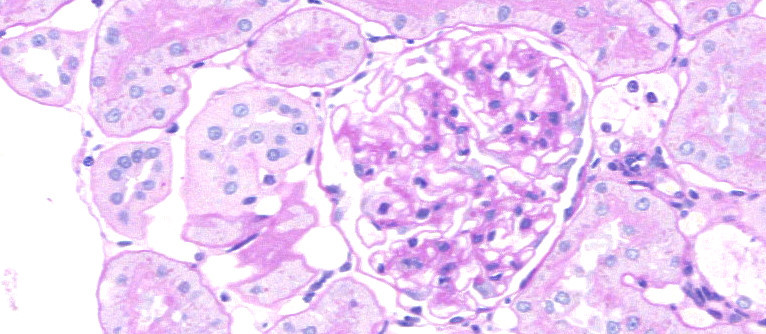

Supplement: Supplementary file 13 [file DataSheet15.ZIP › Fig 1D-PAS-TSF-58/58-5.jpeg]

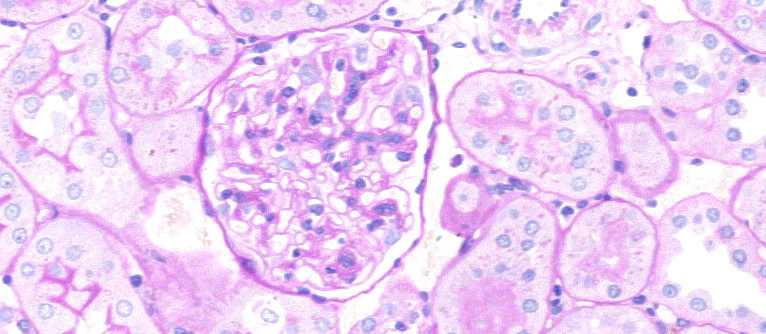

Supplement: Supplementary file 13 [file DataSheet15.ZIP › Fig 1D-PAS-TSF-58/58-6.jpeg]

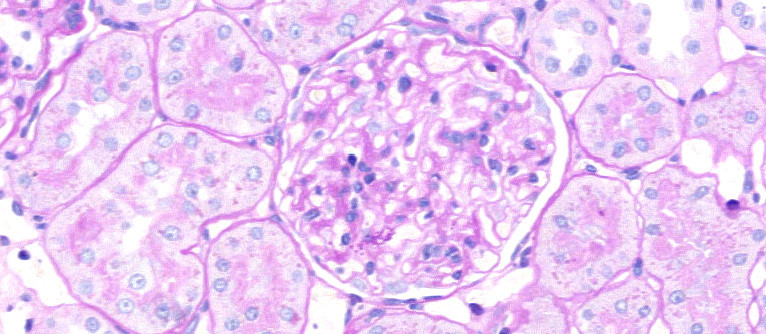

Supplement: Supplementary file 13 [file DataSheet15.ZIP › Fig 1D-PAS-TSF-58/58-7.jpeg]

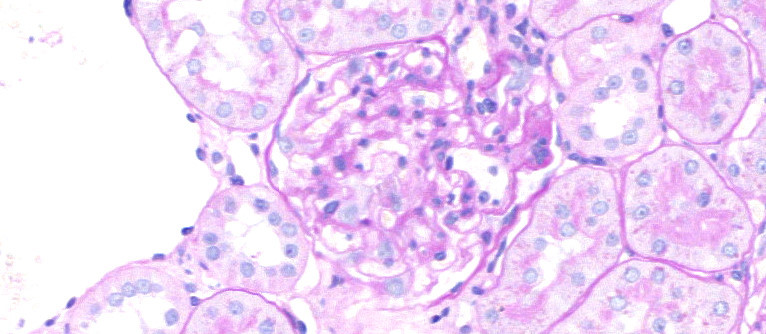

Supplement: Supplementary file 13 [file DataSheet15.ZIP › Fig 1D-PAS-TSF-58/58-8.jpeg]

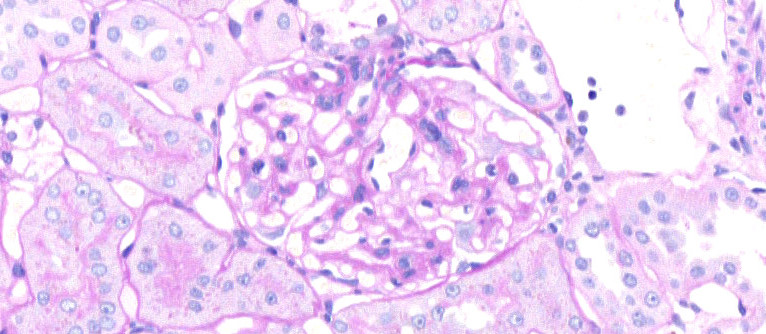

Supplement: Supplementary file 13 [file DataSheet15.ZIP › Fig 1D-PAS-TSF-58/58-9.jpeg]

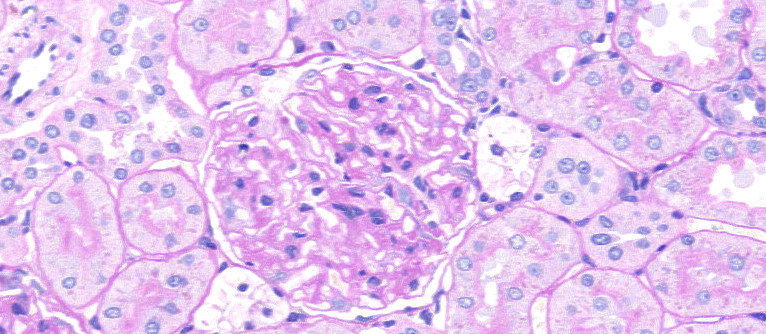

Supplement: Supplementary file 13 [file DataSheet15.ZIP › Fig 1D-PAS-TSF-59/59-1.jpeg]

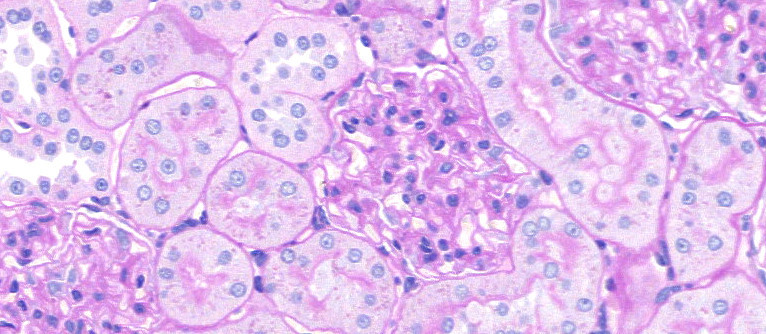

Supplement: Supplementary file 13 [file DataSheet15.ZIP › Fig 1D-PAS-TSF-59/59-10.jpeg]

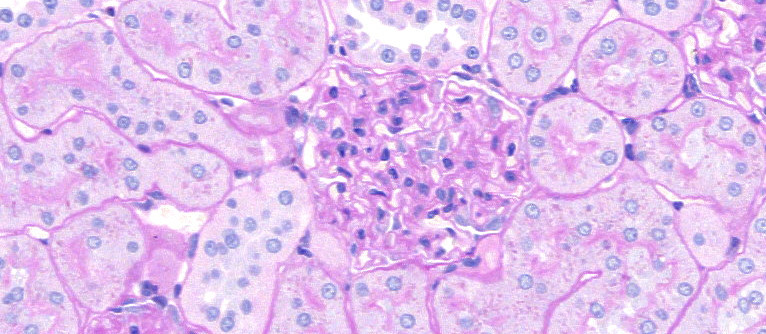

Supplement: Supplementary file 13 [file DataSheet15.ZIP › Fig 1D-PAS-TSF-59/59-11.jpeg]

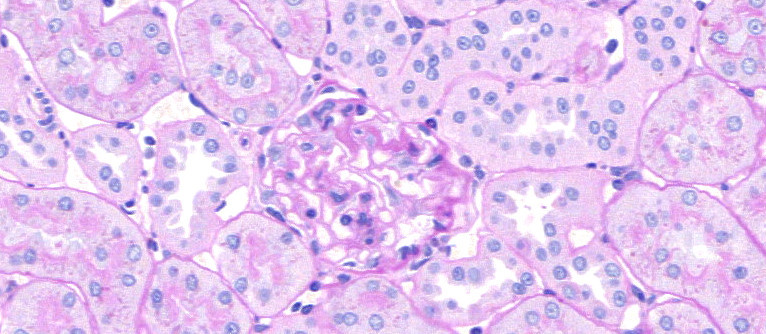

Supplement: Supplementary file 13 [file DataSheet15.ZIP › Fig 1D-PAS-TSF-59/59-12.jpeg]

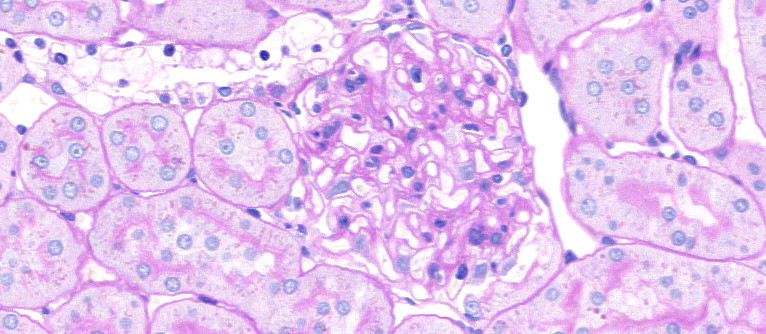

Supplement: Supplementary file 13 [file DataSheet15.ZIP › Fig 1D-PAS-TSF-59/59-13.jpeg]

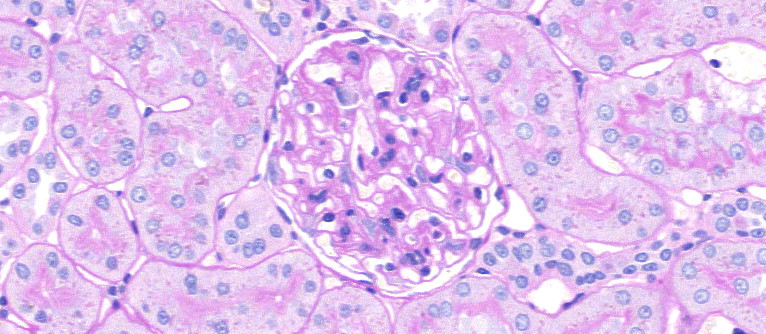

Supplement: Supplementary file 13 [file DataSheet15.ZIP › Fig 1D-PAS-TSF-59/59-14.jpeg]

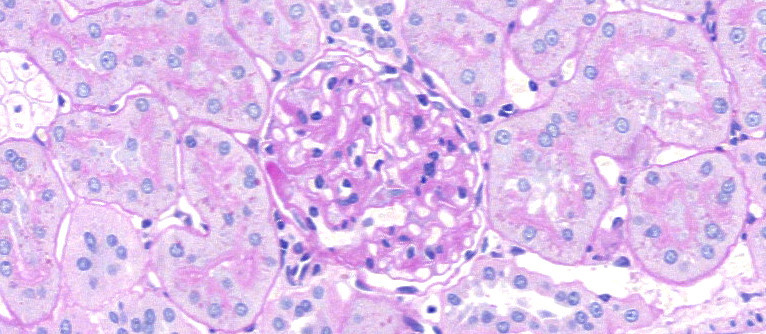

Supplement: Supplementary file 13 [file DataSheet15.ZIP › Fig 1D-PAS-TSF-59/59-15.jpeg]

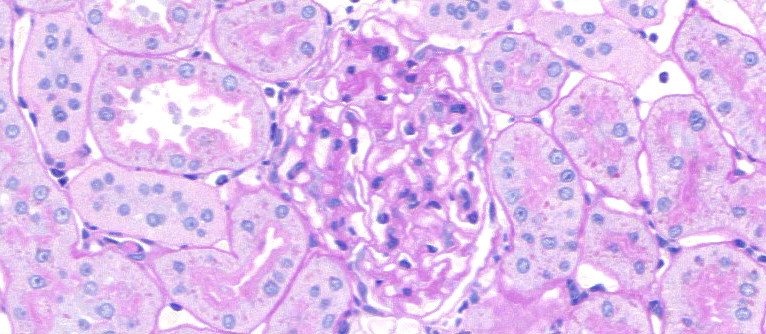

Supplement: Supplementary file 13 [file DataSheet15.ZIP › Fig 1D-PAS-TSF-59/59-16.jpeg]

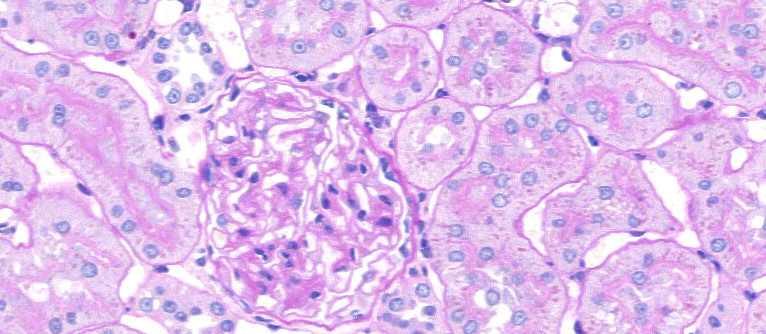

Supplement: Supplementary file 13 [file DataSheet15.ZIP › Fig 1D-PAS-TSF-59/59-17.jpeg]

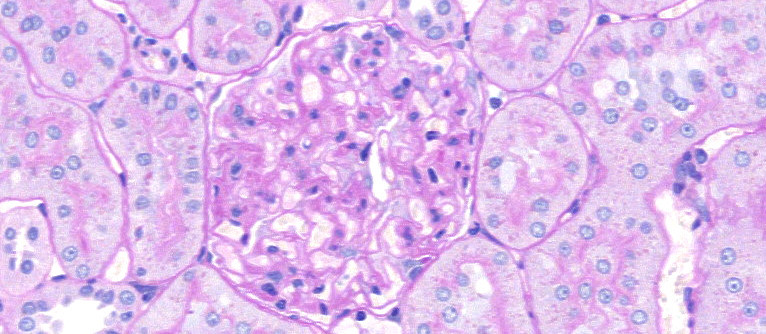

Supplement: Supplementary file 13 [file DataSheet15.ZIP › Fig 1D-PAS-TSF-59/59-18.jpeg]

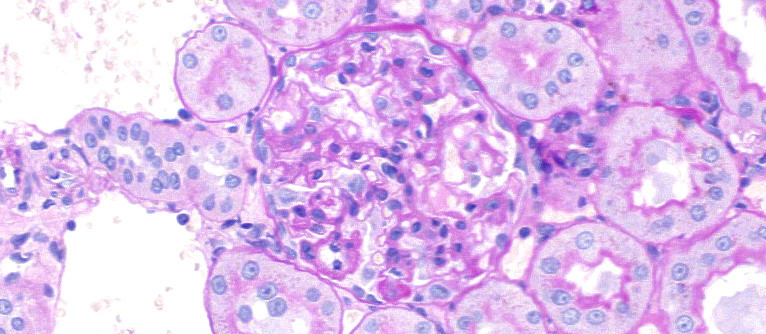

Supplement: Supplementary file 13 [file DataSheet15.ZIP › Fig 1D-PAS-TSF-59/59-19.jpeg]

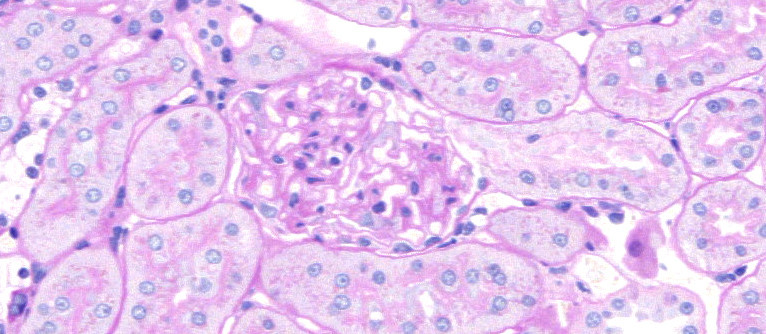

Supplement: Supplementary file 13 [file DataSheet15.ZIP › Fig 1D-PAS-TSF-59/59-2.jpeg]

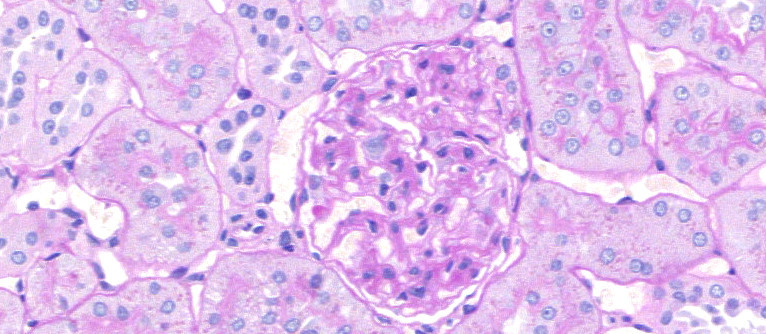

Supplement: Supplementary file 13 [file DataSheet15.ZIP › Fig 1D-PAS-TSF-59/59-20.jpeg]

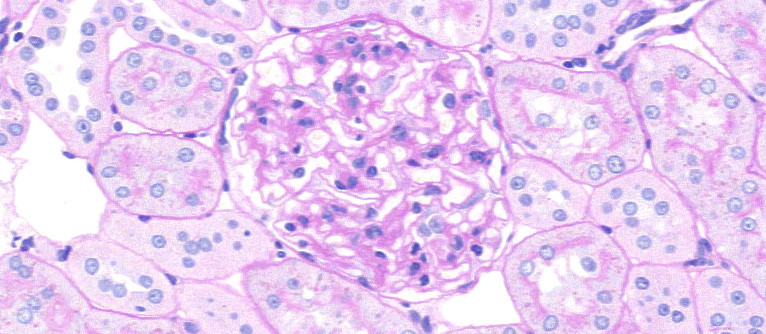

Supplement: Supplementary file 13 [file DataSheet15.ZIP › Fig 1D-PAS-TSF-59/59-3.jpeg]

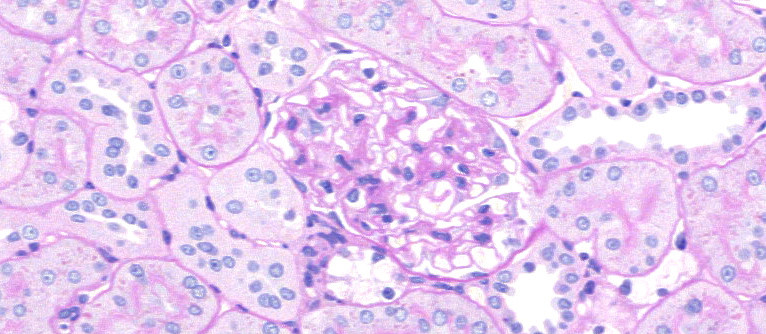

Supplement: Supplementary file 13 [file DataSheet15.ZIP › Fig 1D-PAS-TSF-59/59-4.jpeg]

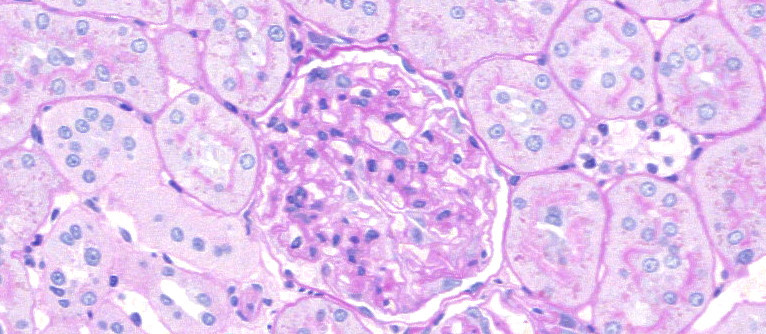

Supplement: Supplementary file 13 [file DataSheet15.ZIP › Fig 1D-PAS-TSF-59/59-5.jpeg]

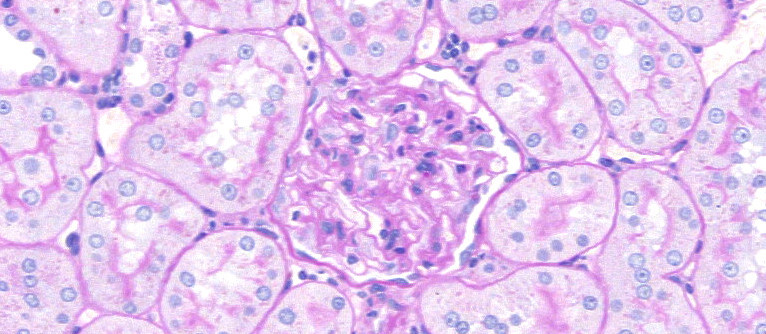

Supplement: Supplementary file 13 [file DataSheet15.ZIP › Fig 1D-PAS-TSF-59/59-6.jpeg]

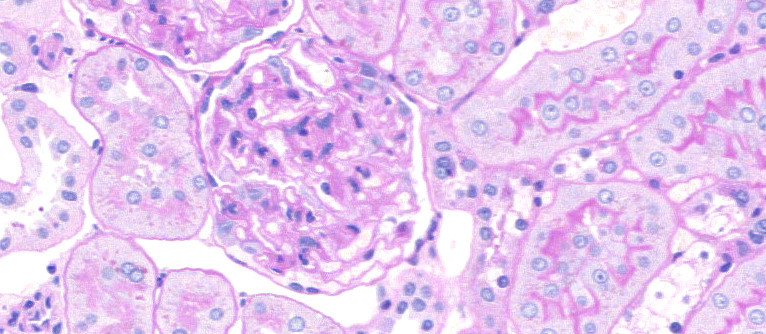

Supplement: Supplementary file 13 [file DataSheet15.ZIP › Fig 1D-PAS-TSF-59/59-7.jpeg]

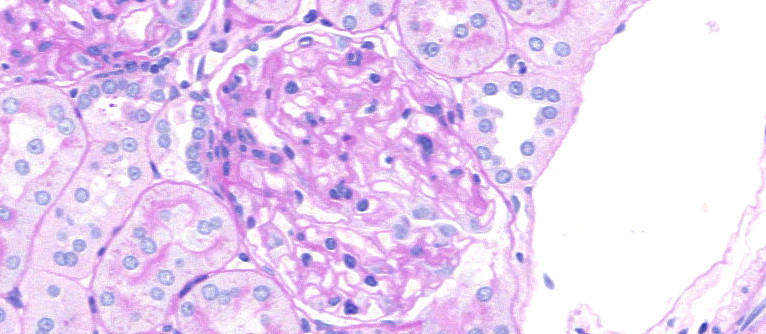

Supplement: Supplementary file 13 [file DataSheet15.ZIP › Fig 1D-PAS-TSF-59/59-8.jpeg]

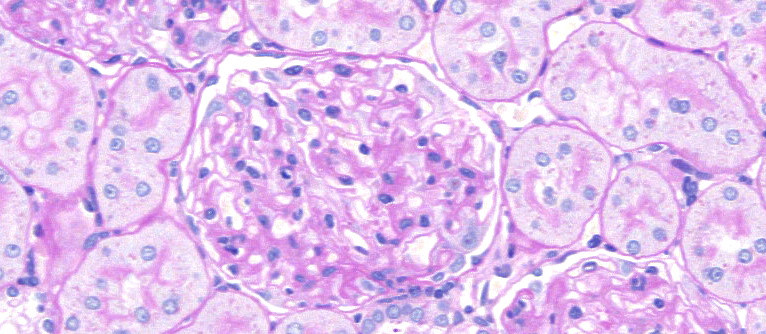

Supplement: Supplementary file 13 [file DataSheet15.ZIP › Fig 1D-PAS-TSF-59/59-9.jpeg]

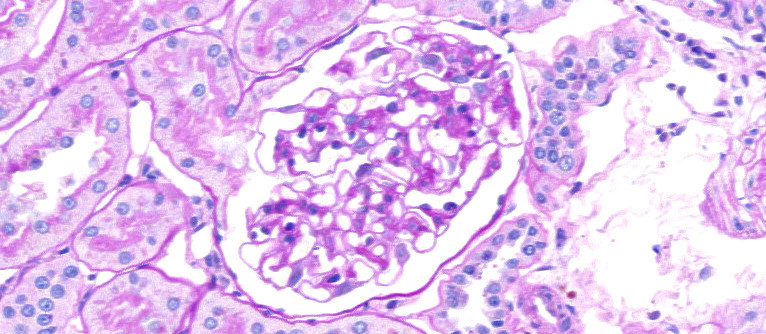

Supplement: Supplementary file 13 [file DataSheet15.ZIP › Fig 1D-PAS-TSF-60/60-1.jpeg]

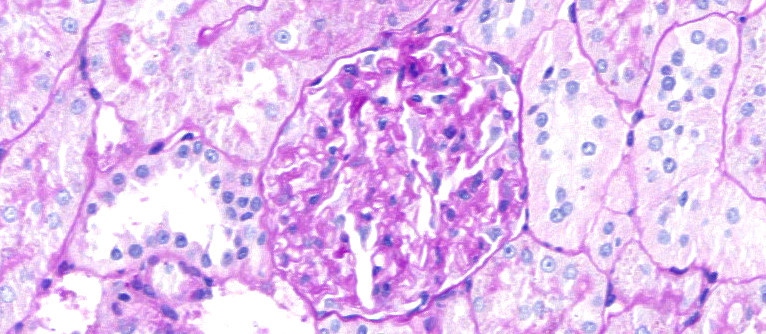

Supplement: Supplementary file 13 [file DataSheet15.ZIP › Fig 1D-PAS-TSF-60/60-10.jpeg]
