# Supplementary figures and images for: Characteristics of Serum Metabolites and Gut Microbiota in Diabetic Kidney Disease (part 11 of 13)
Source: Front Pharmacol. 2022 Apr 14;13:872988. doi: 10.3389/fphar.2022.872988 (PMC9084235; doi:10.3389/fphar.2022.872988)

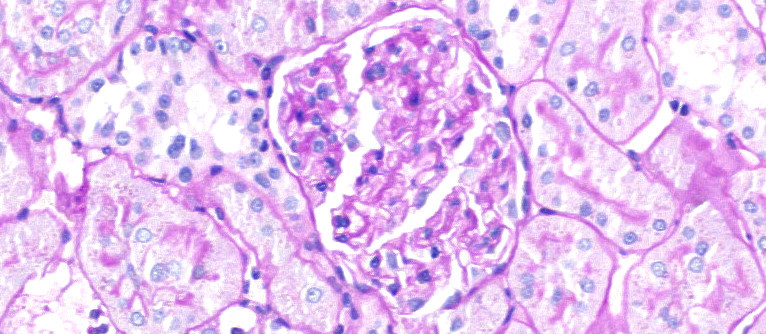

Supplement: Supplementary file 13 [file DataSheet15.ZIP › Fig 1D-PAS-TSF-60/60-11.jpeg]

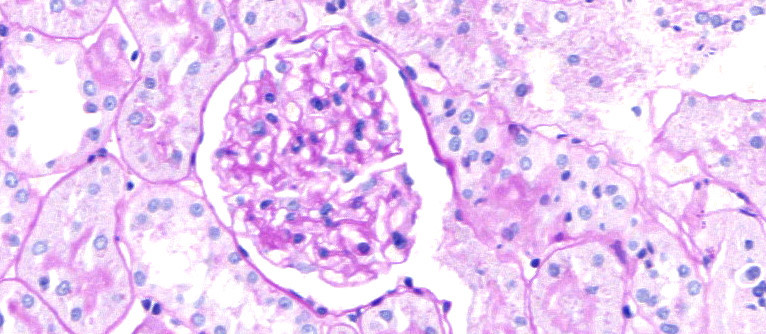

Supplement: Supplementary file 13 [file DataSheet15.ZIP › Fig 1D-PAS-TSF-60/60-12.jpeg]

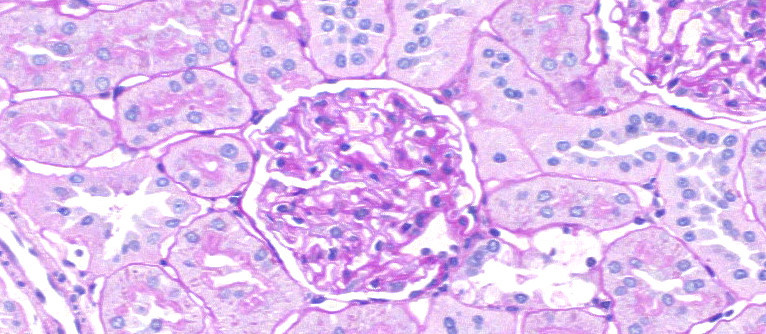

Supplement: Supplementary file 13 [file DataSheet15.ZIP › Fig 1D-PAS-TSF-60/60-13.jpeg]

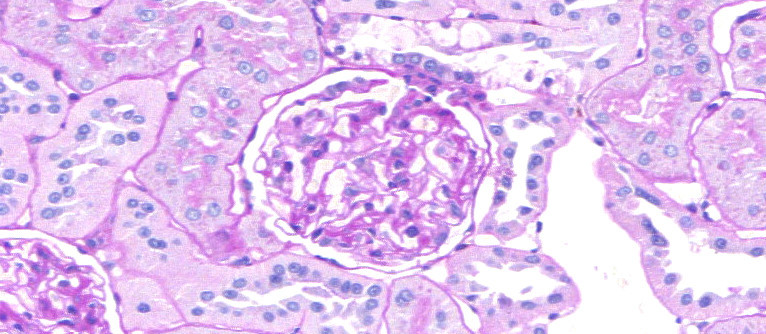

Supplement: Supplementary file 13 [file DataSheet15.ZIP › Fig 1D-PAS-TSF-60/60-14.jpeg]

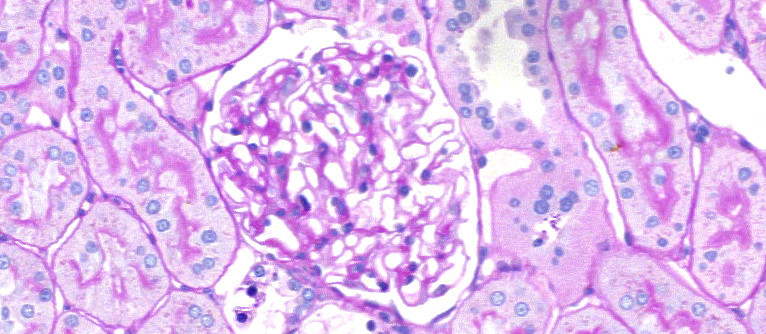

Supplement: Supplementary file 13 [file DataSheet15.ZIP › Fig 1D-PAS-TSF-60/60-15.jpeg]

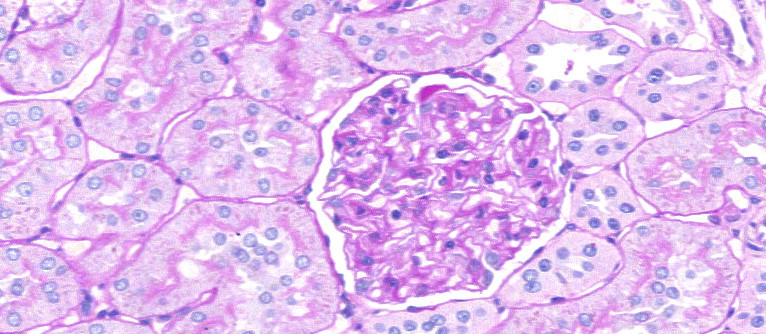

Supplement: Supplementary file 13 [file DataSheet15.ZIP › Fig 1D-PAS-TSF-60/60-16.jpeg]

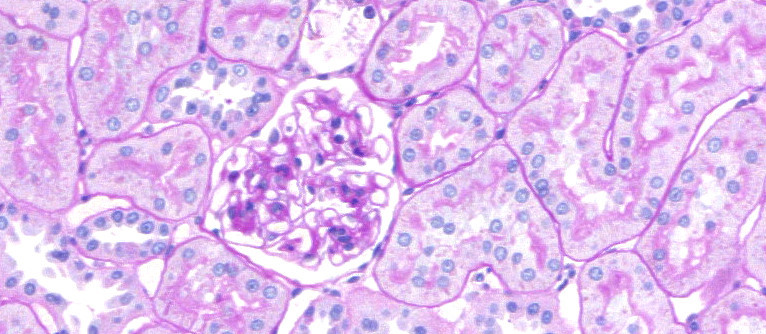

Supplement: Supplementary file 13 [file DataSheet15.ZIP › Fig 1D-PAS-TSF-60/60-17.jpeg]

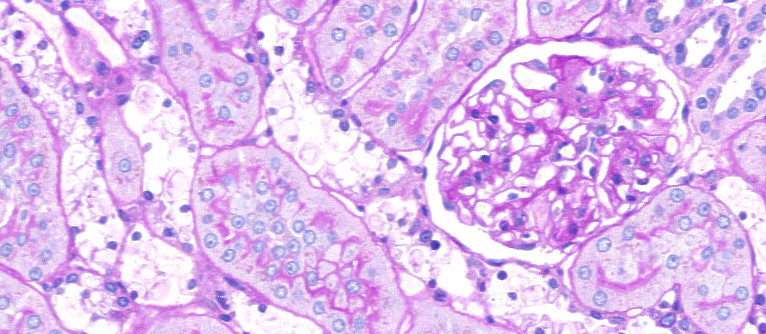

Supplement: Supplementary file 13 [file DataSheet15.ZIP › Fig 1D-PAS-TSF-60/60-18.jpeg]

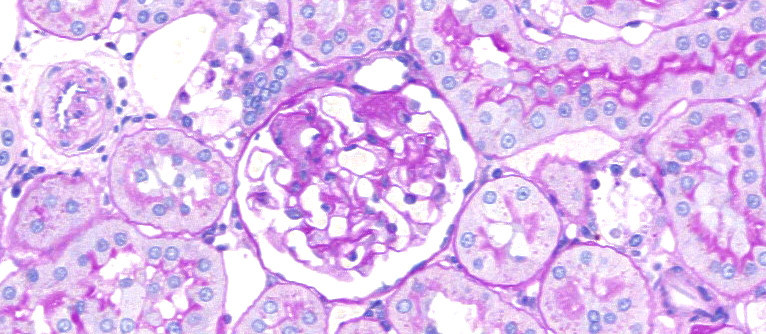

Supplement: Supplementary file 13 [file DataSheet15.ZIP › Fig 1D-PAS-TSF-60/60-19.jpeg]

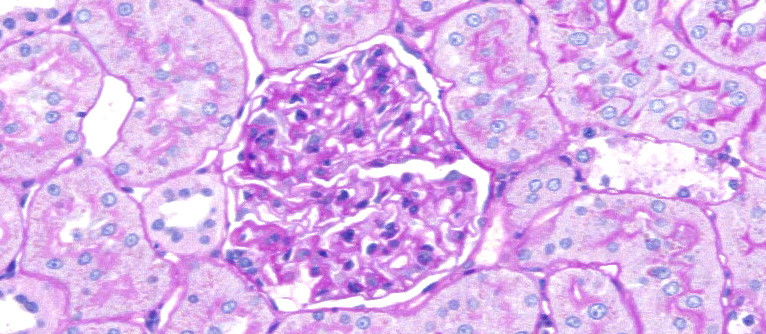

Supplement: Supplementary file 13 [file DataSheet15.ZIP › Fig 1D-PAS-TSF-60/60-2.jpeg]

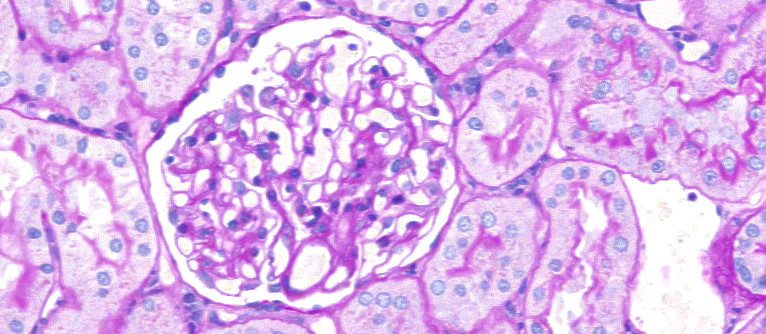

Supplement: Supplementary file 13 [file DataSheet15.ZIP › Fig 1D-PAS-TSF-60/60-20.jpeg]

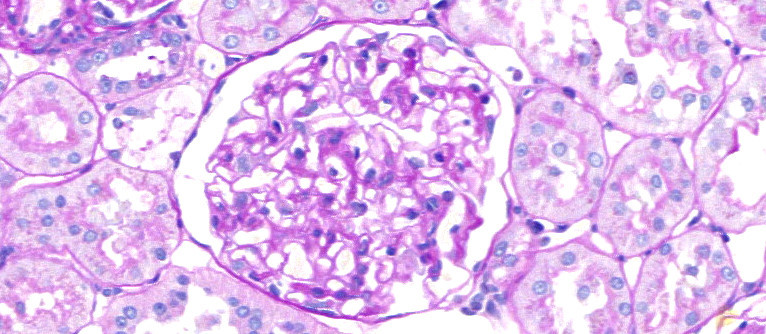

Supplement: Supplementary file 13 [file DataSheet15.ZIP › Fig 1D-PAS-TSF-60/60-3.jpeg]

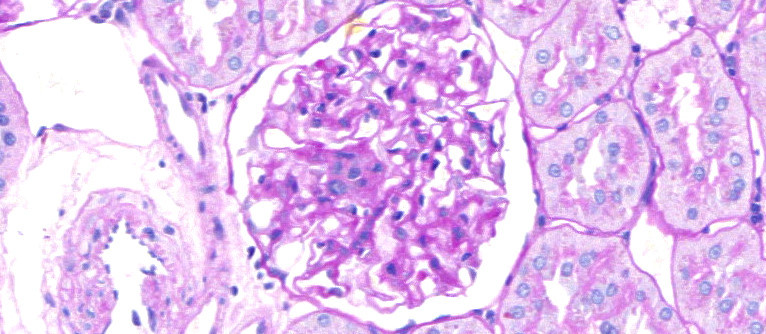

Supplement: Supplementary file 13 [file DataSheet15.ZIP › Fig 1D-PAS-TSF-60/60-4.jpeg]

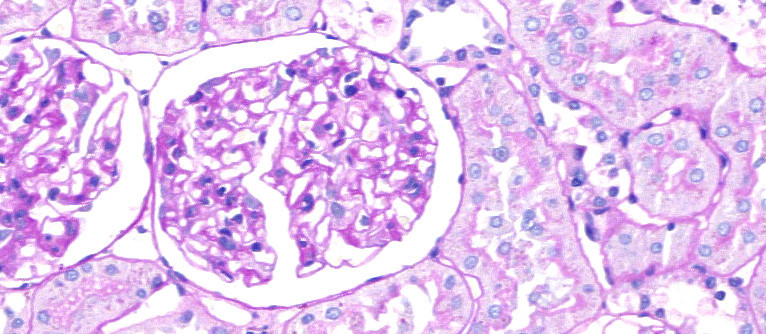

Supplement: Supplementary file 13 [file DataSheet15.ZIP › Fig 1D-PAS-TSF-60/60-5.jpeg]

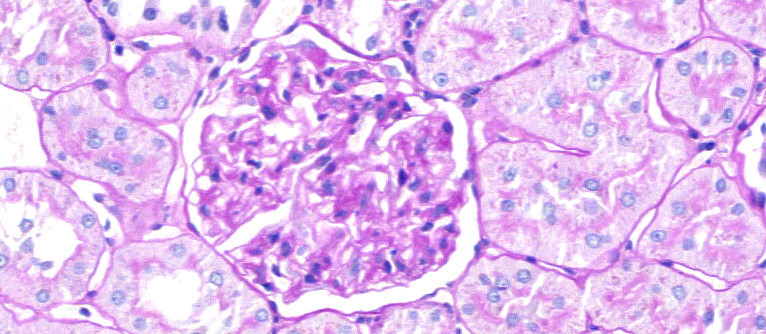

Supplement: Supplementary file 13 [file DataSheet15.ZIP › Fig 1D-PAS-TSF-60/60-6.jpeg]

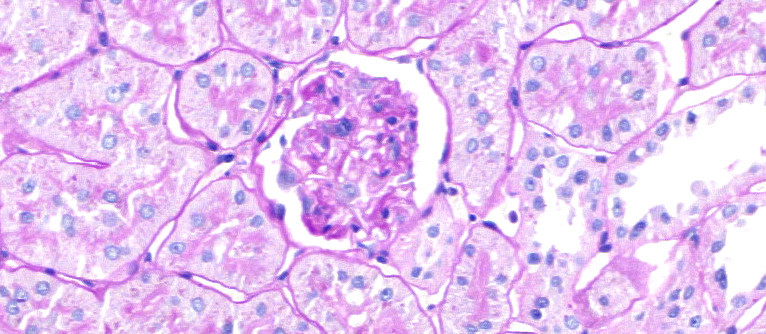

Supplement: Supplementary file 13 [file DataSheet15.ZIP › Fig 1D-PAS-TSF-60/60-7.jpeg]

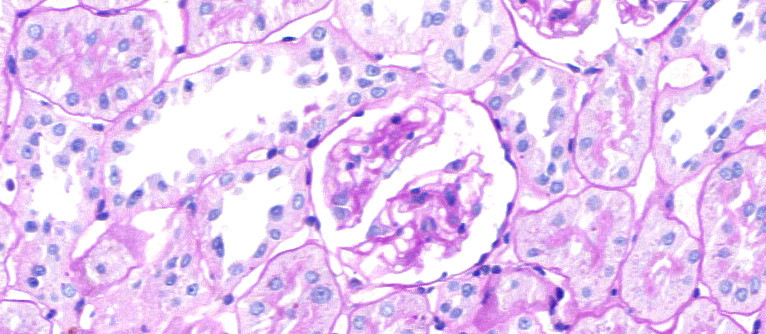

Supplement: Supplementary file 13 [file DataSheet15.ZIP › Fig 1D-PAS-TSF-60/60-8.jpeg]

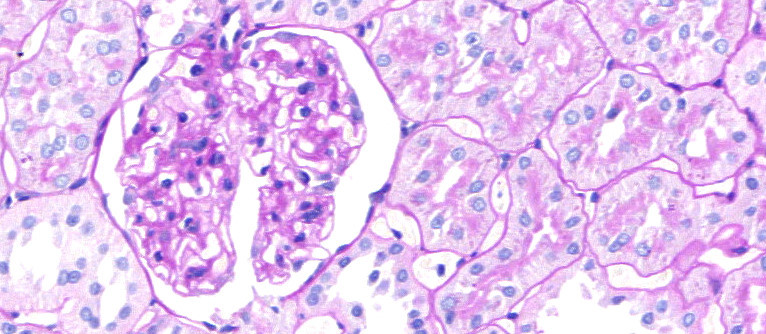

Supplement: Supplementary file 13 [file DataSheet15.ZIP › Fig 1D-PAS-TSF-60/60-9.jpeg]

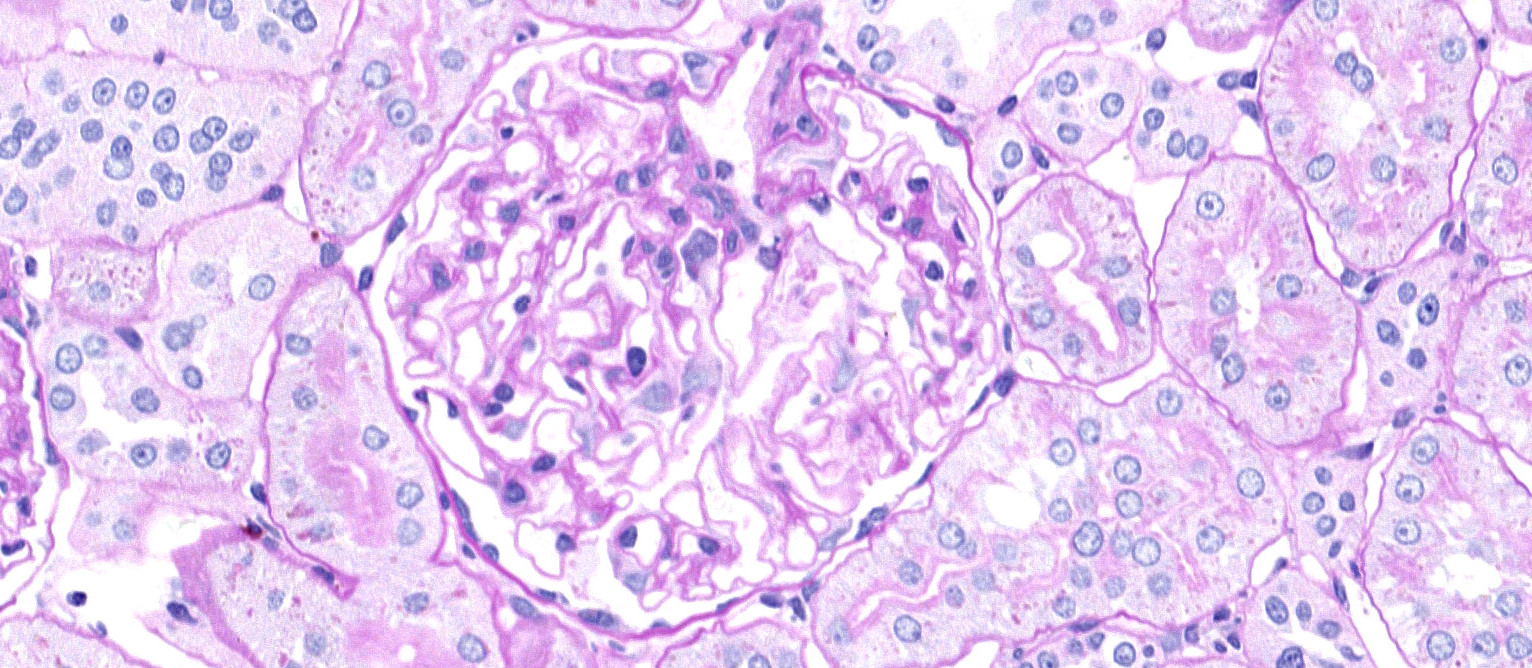

Supplement: Supplementary file 13 [file DataSheet15.ZIP › Fig 1D-PAS-TSF-61/61-1.jpeg]

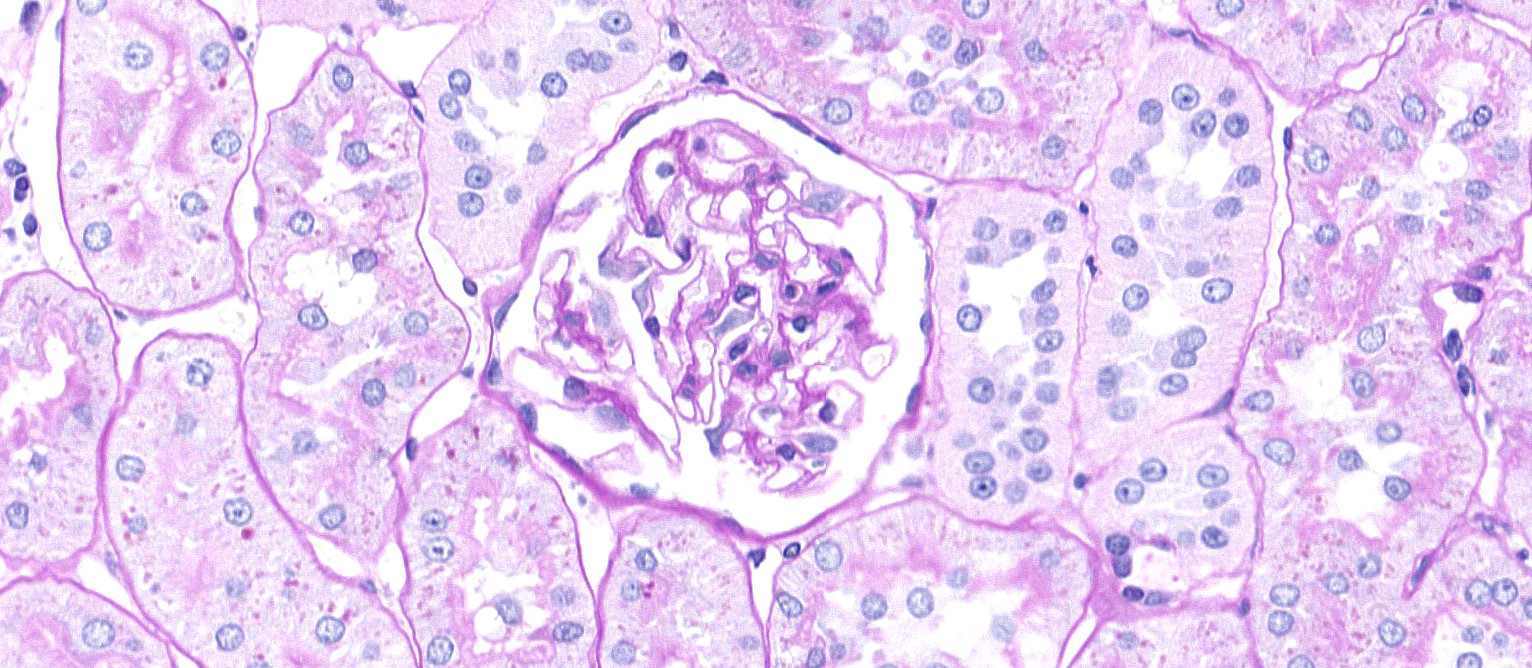

Supplement: Supplementary file 13 [file DataSheet15.ZIP › Fig 1D-PAS-TSF-61/61-10.jpeg]

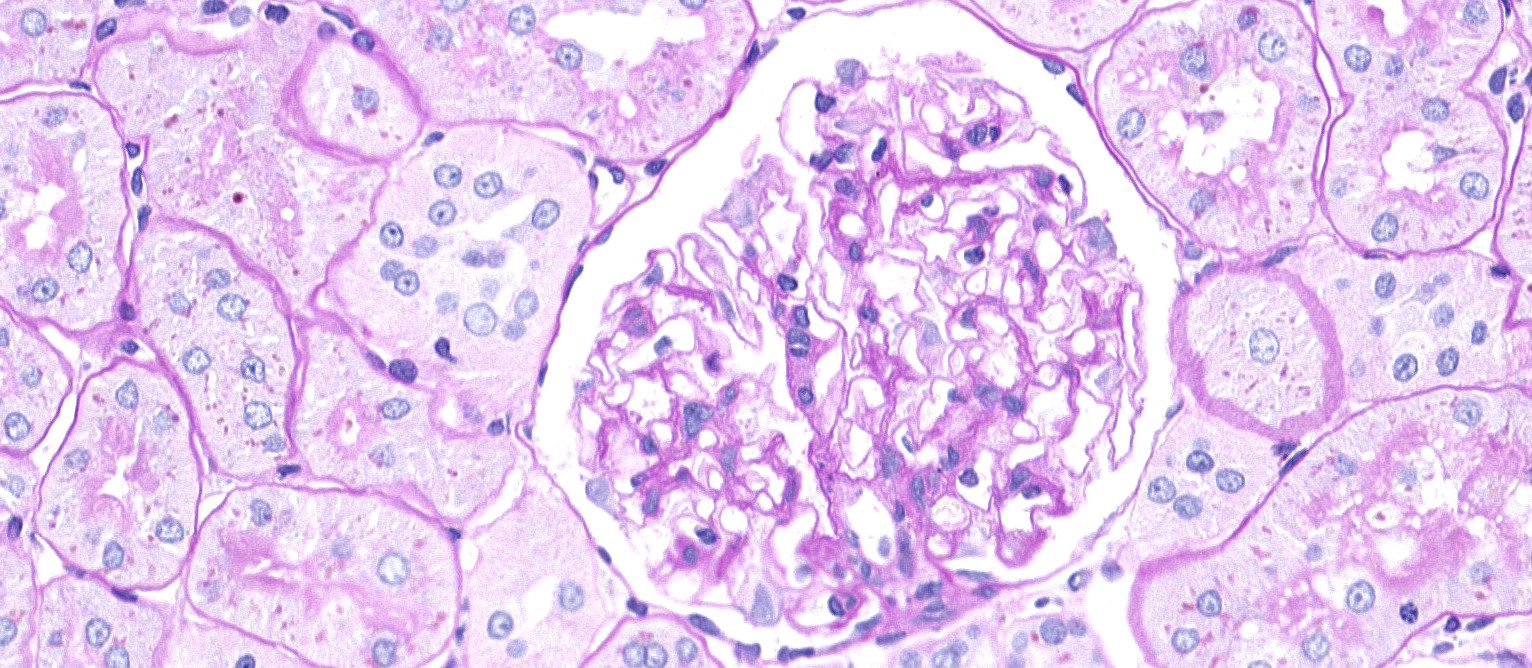

Supplement: Supplementary file 13 [file DataSheet15.ZIP › Fig 1D-PAS-TSF-61/61-11.jpeg]

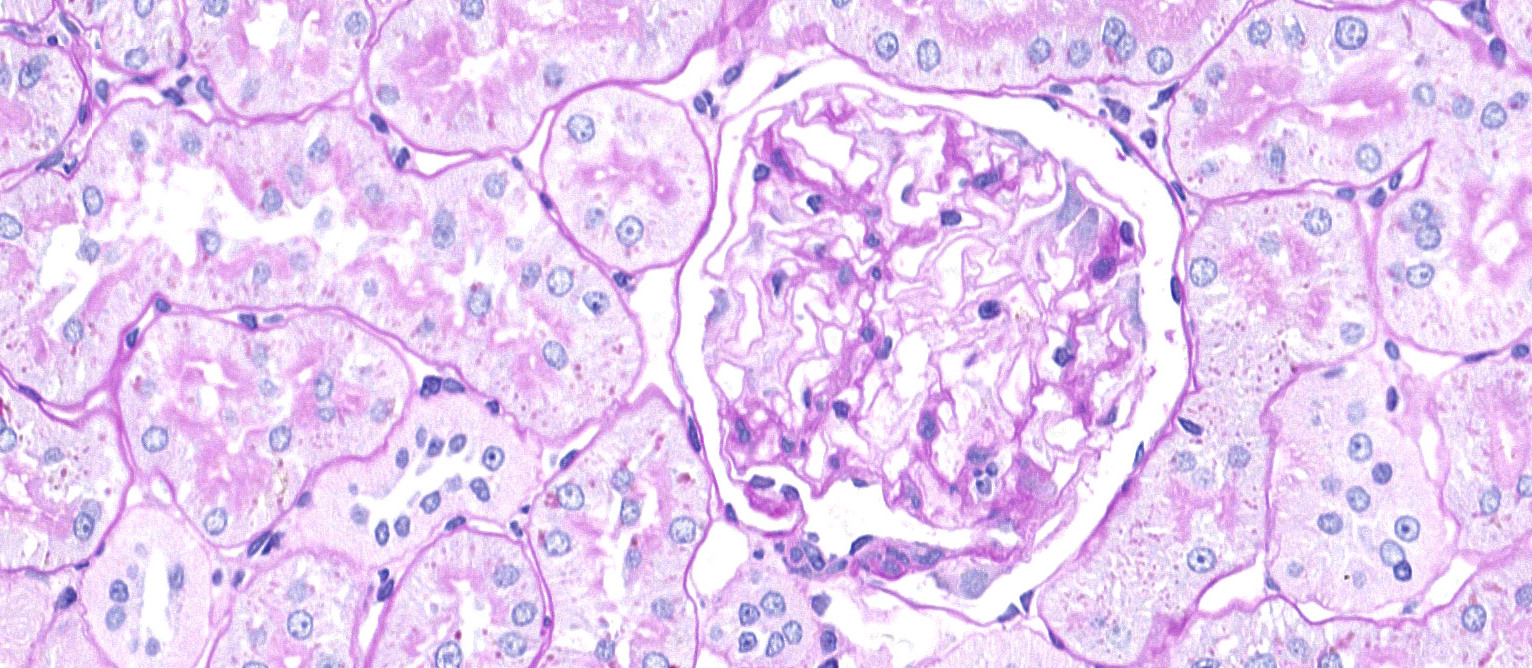

Supplement: Supplementary file 13 [file DataSheet15.ZIP › Fig 1D-PAS-TSF-61/61-12.jpeg]

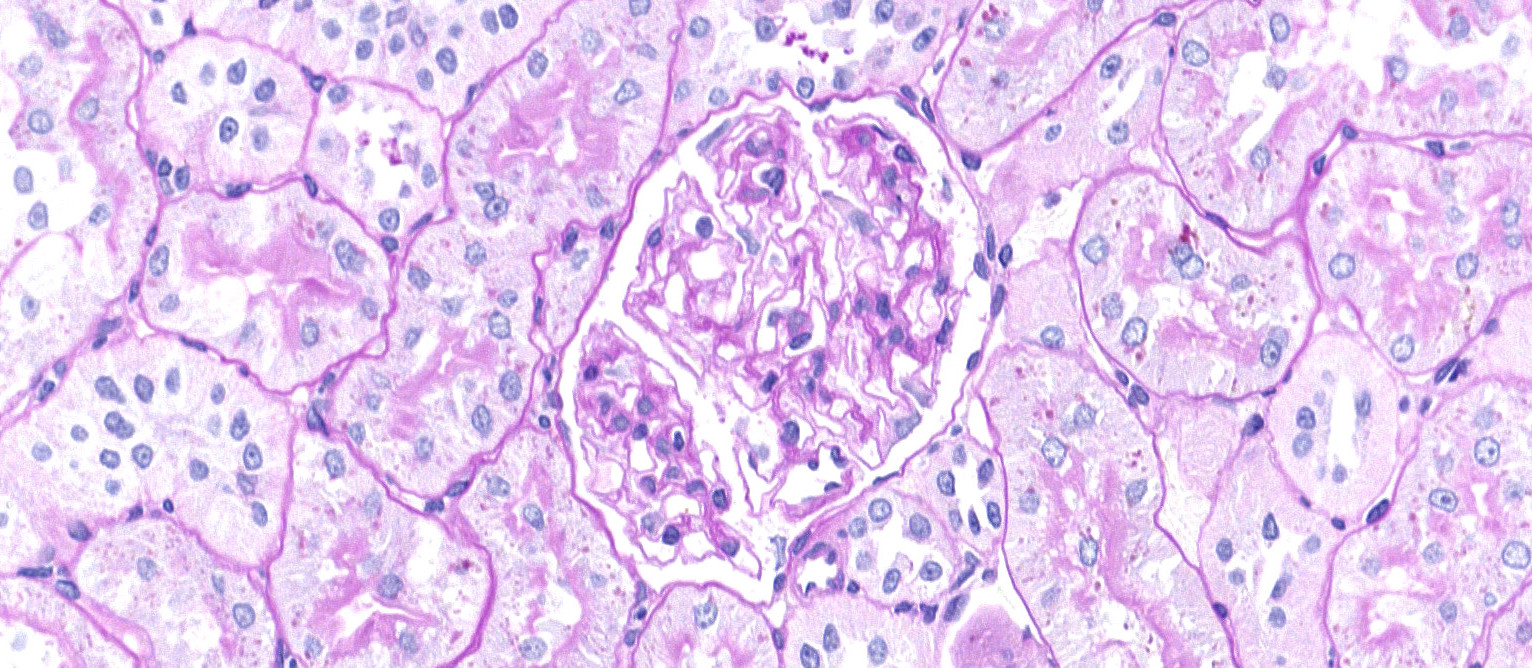

Supplement: Supplementary file 13 [file DataSheet15.ZIP › Fig 1D-PAS-TSF-61/61-13.jpeg]

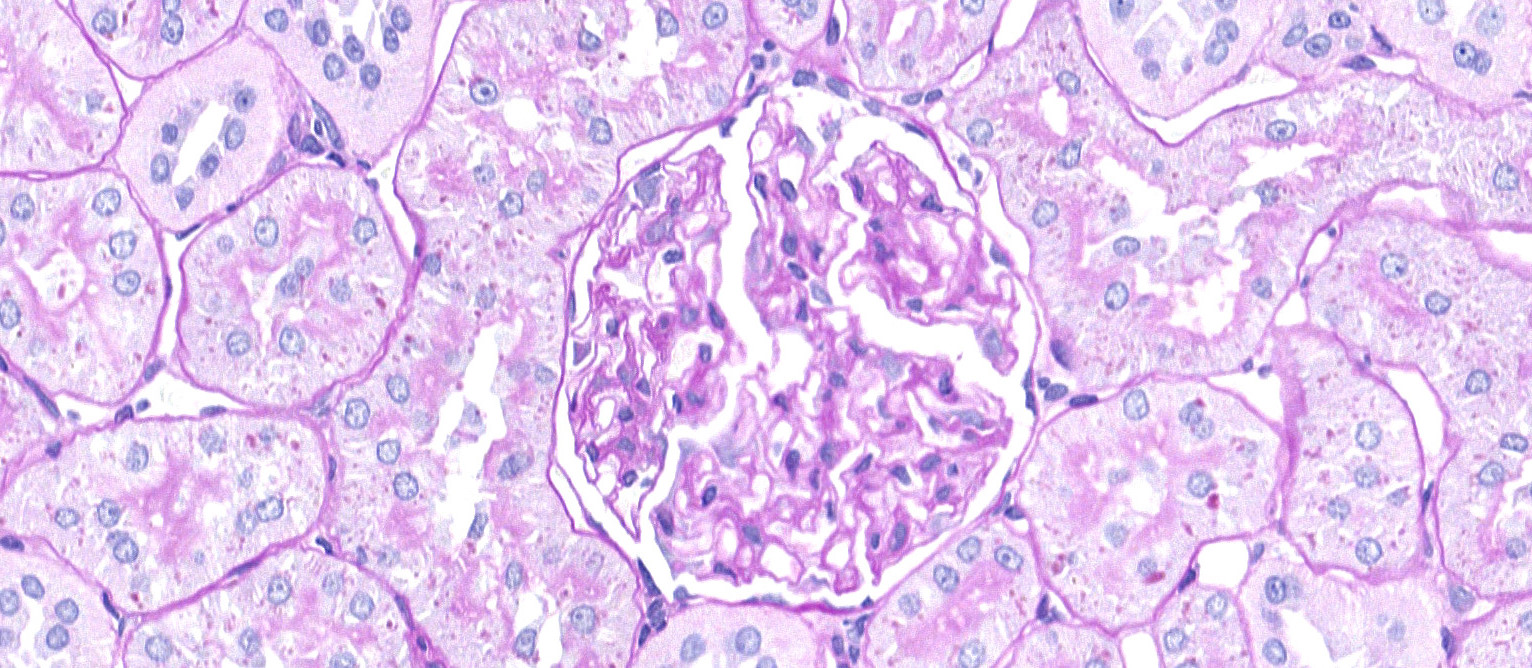

Supplement: Supplementary file 13 [file DataSheet15.ZIP › Fig 1D-PAS-TSF-61/61-14.jpeg]

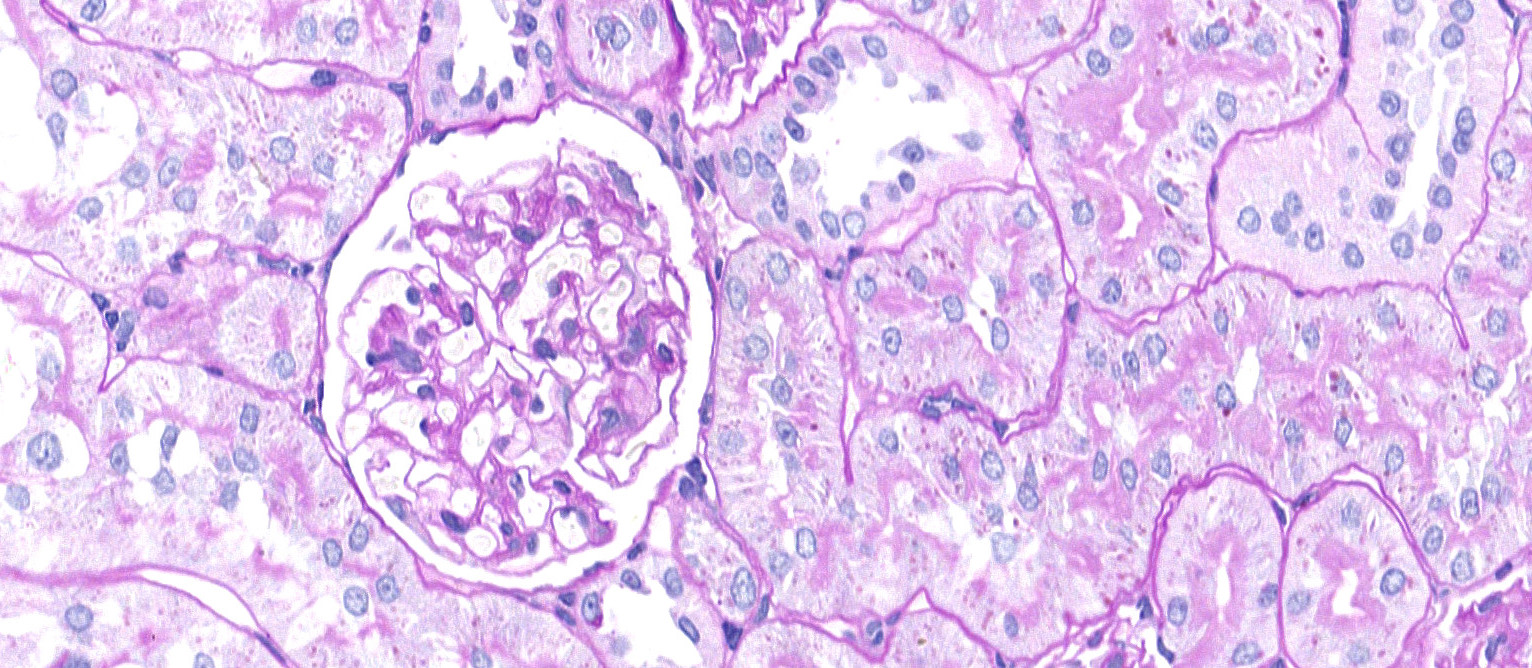

Supplement: Supplementary file 13 [file DataSheet15.ZIP › Fig 1D-PAS-TSF-61/61-15.jpeg]

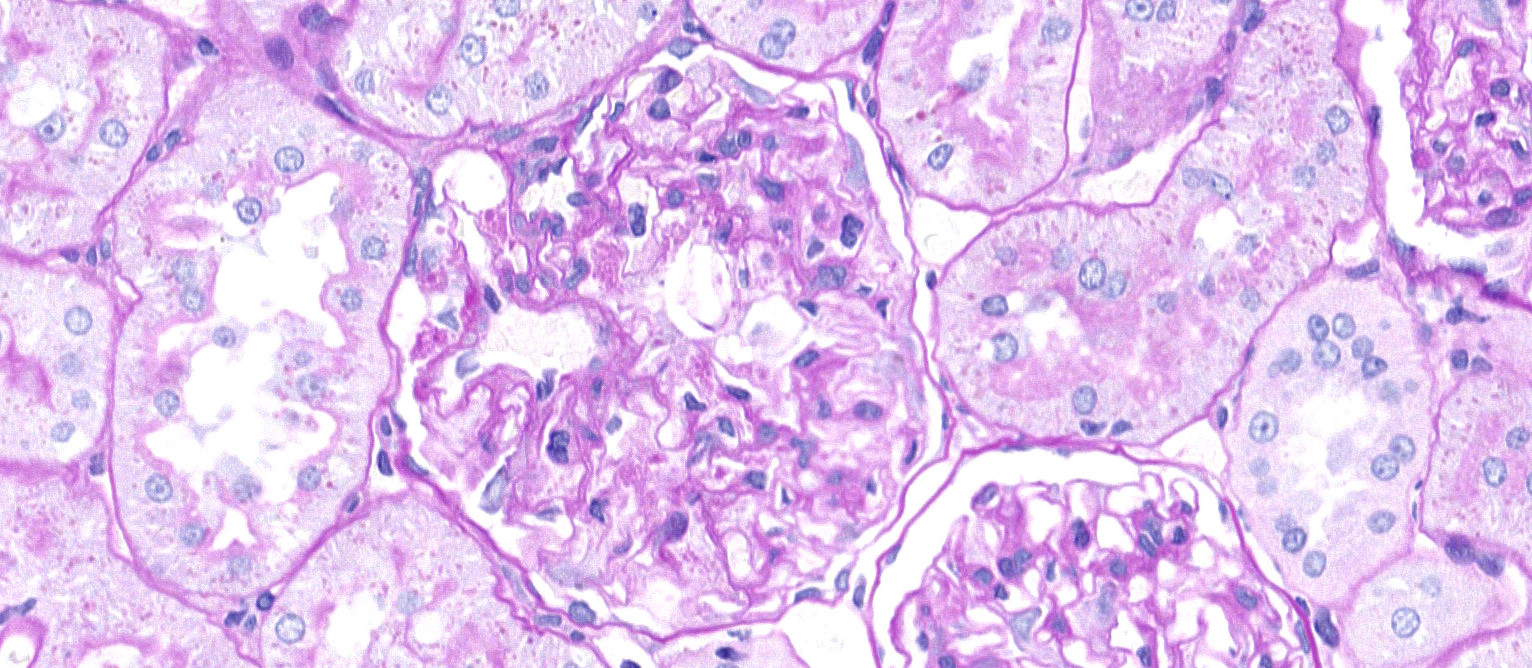

Supplement: Supplementary file 13 [file DataSheet15.ZIP › Fig 1D-PAS-TSF-61/61-16.jpeg]

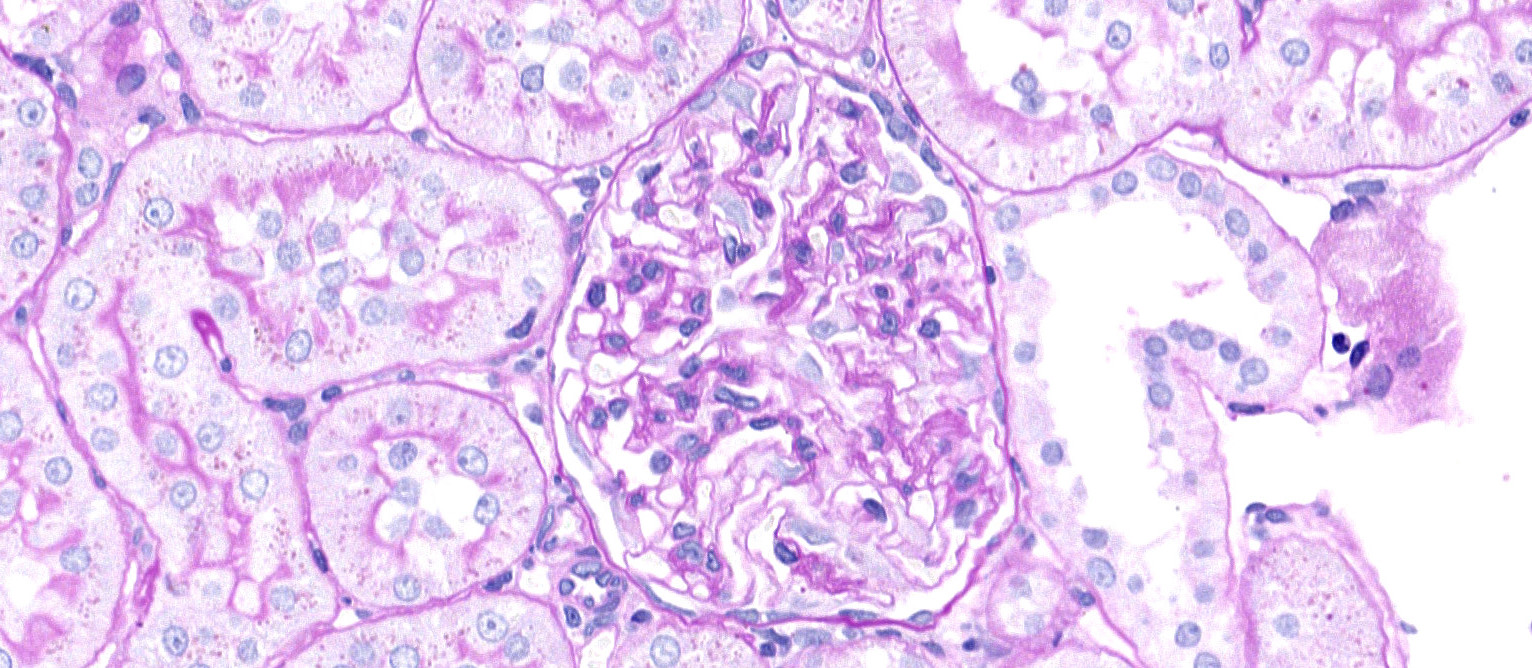

Supplement: Supplementary file 13 [file DataSheet15.ZIP › Fig 1D-PAS-TSF-61/61-17.jpeg]

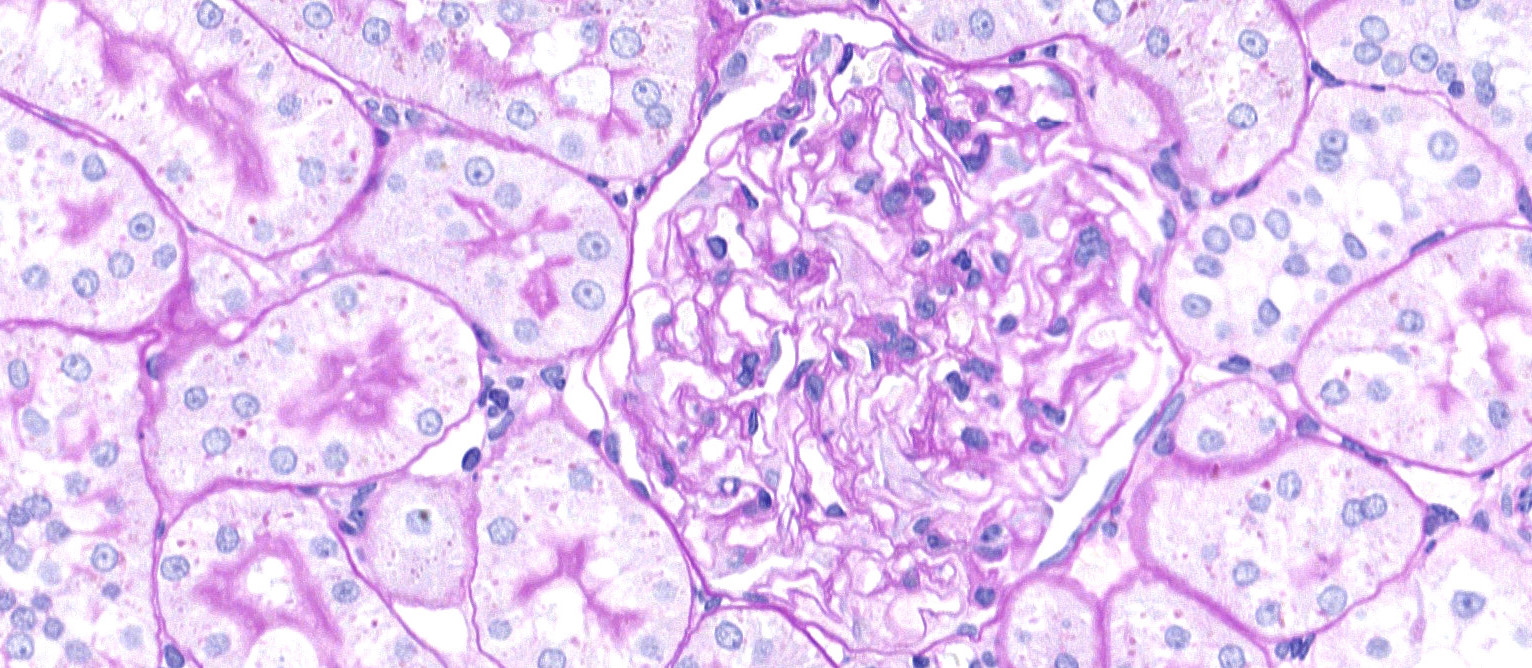

Supplement: Supplementary file 13 [file DataSheet15.ZIP › Fig 1D-PAS-TSF-61/61-18.jpeg]

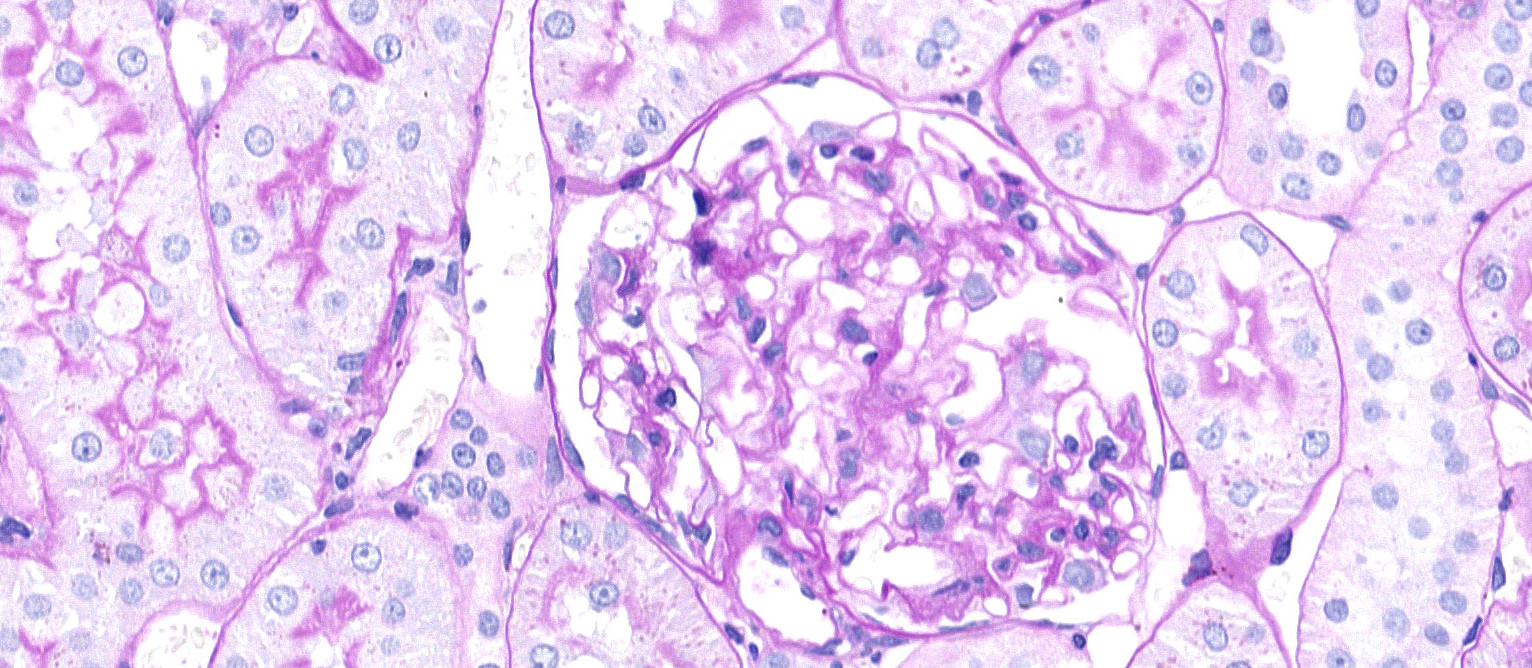

Supplement: Supplementary file 13 [file DataSheet15.ZIP › Fig 1D-PAS-TSF-61/61-19.jpeg]

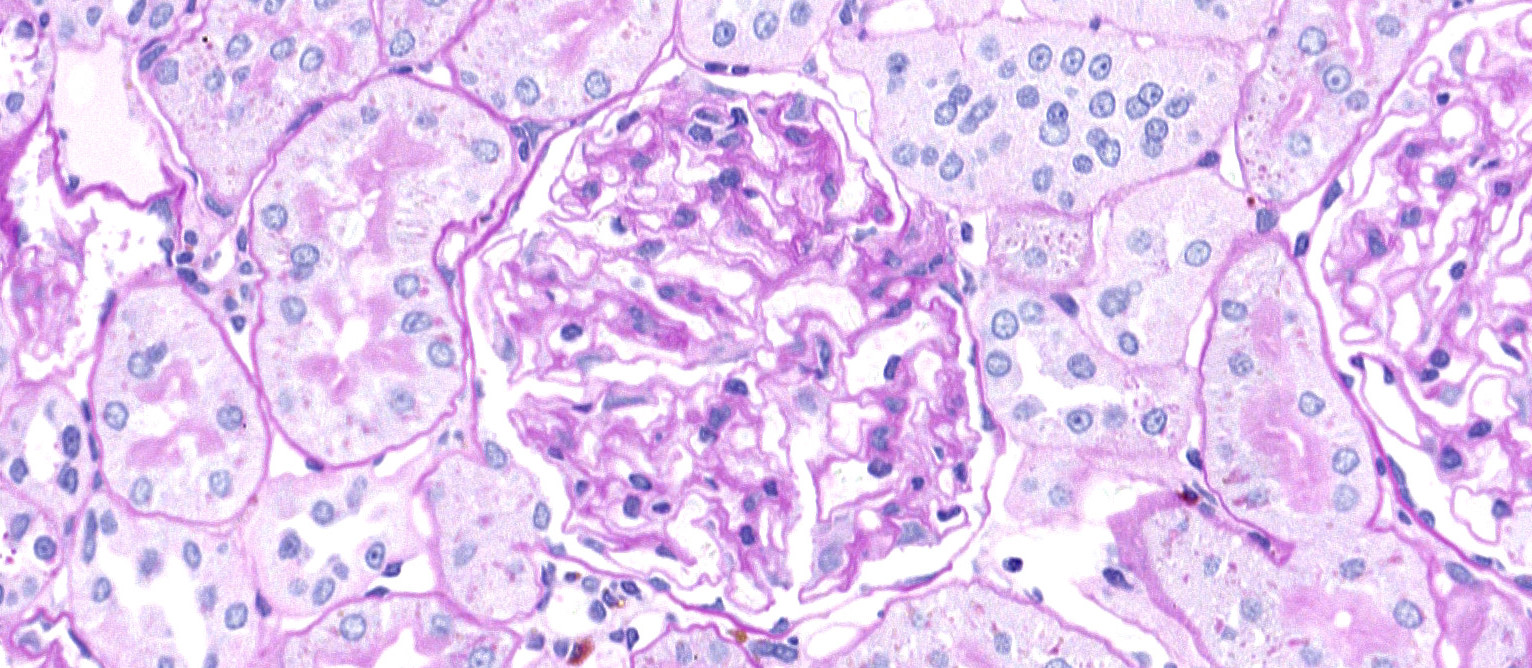

Supplement: Supplementary file 13 [file DataSheet15.ZIP › Fig 1D-PAS-TSF-61/61-2.jpeg]

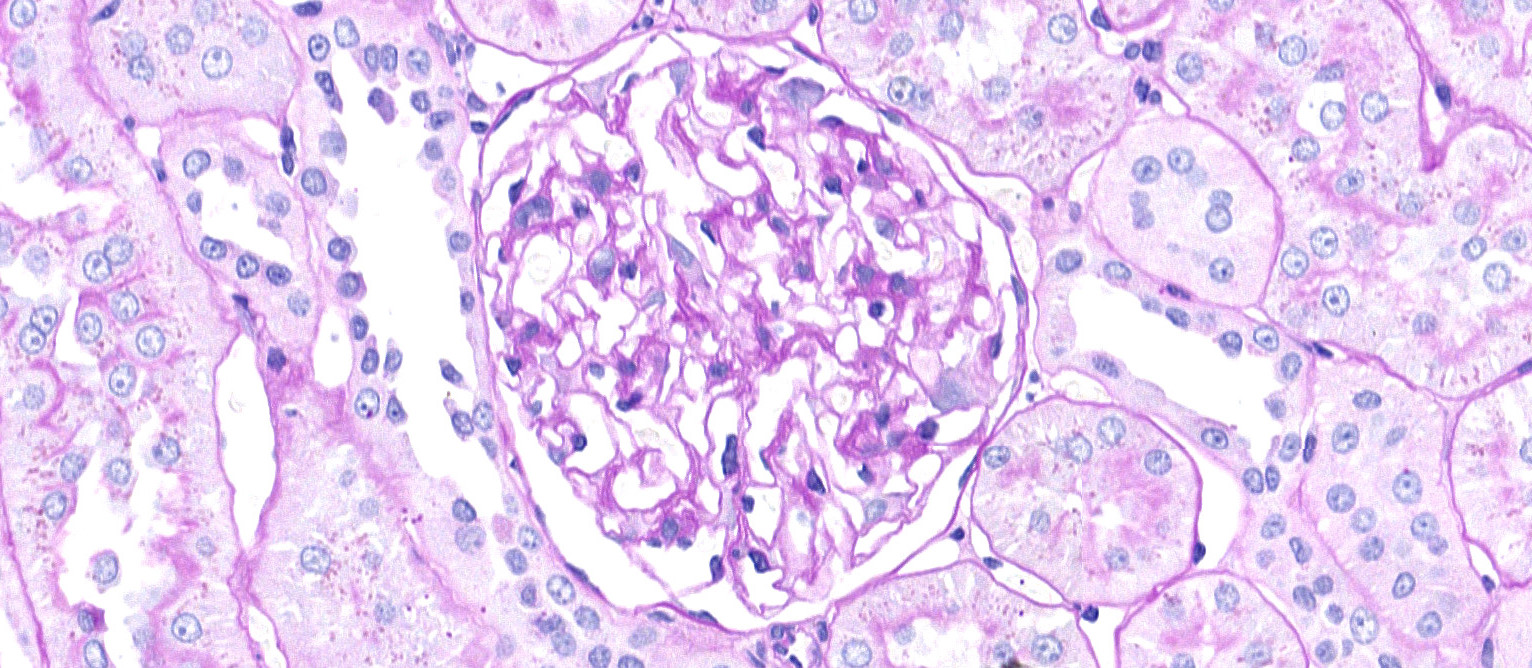

Supplement: Supplementary file 13 [file DataSheet15.ZIP › Fig 1D-PAS-TSF-61/61-20.jpeg]

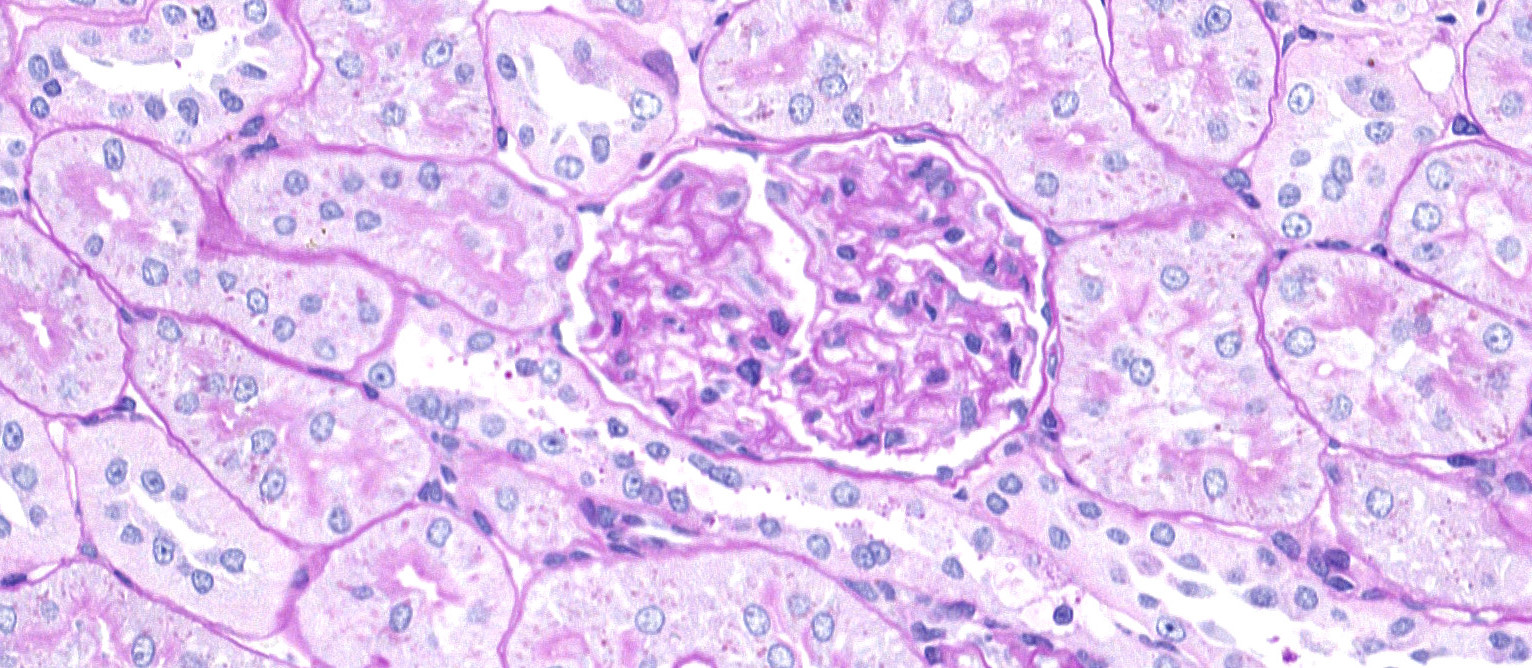

Supplement: Supplementary file 13 [file DataSheet15.ZIP › Fig 1D-PAS-TSF-61/61-3.jpeg]

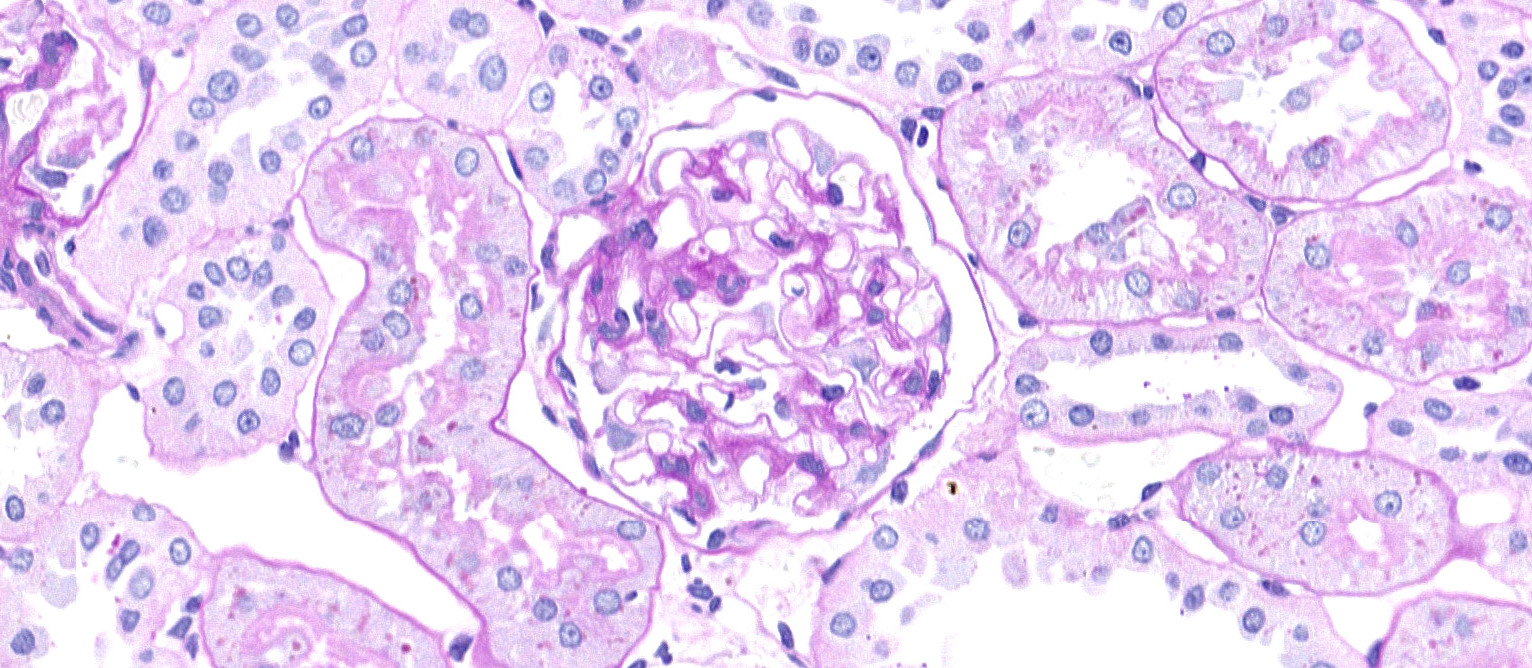

Supplement: Supplementary file 13 [file DataSheet15.ZIP › Fig 1D-PAS-TSF-61/61-4.jpeg]

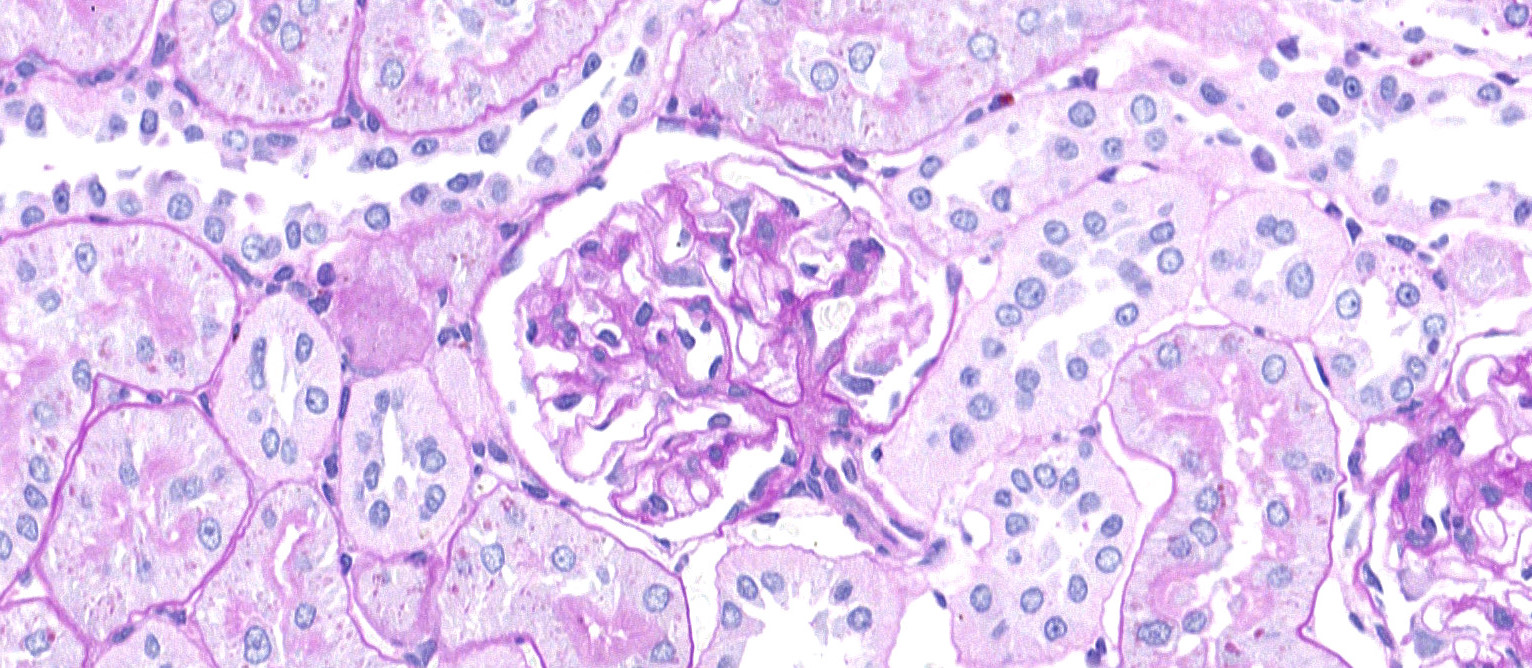

Supplement: Supplementary file 13 [file DataSheet15.ZIP › Fig 1D-PAS-TSF-61/61-5.jpeg]

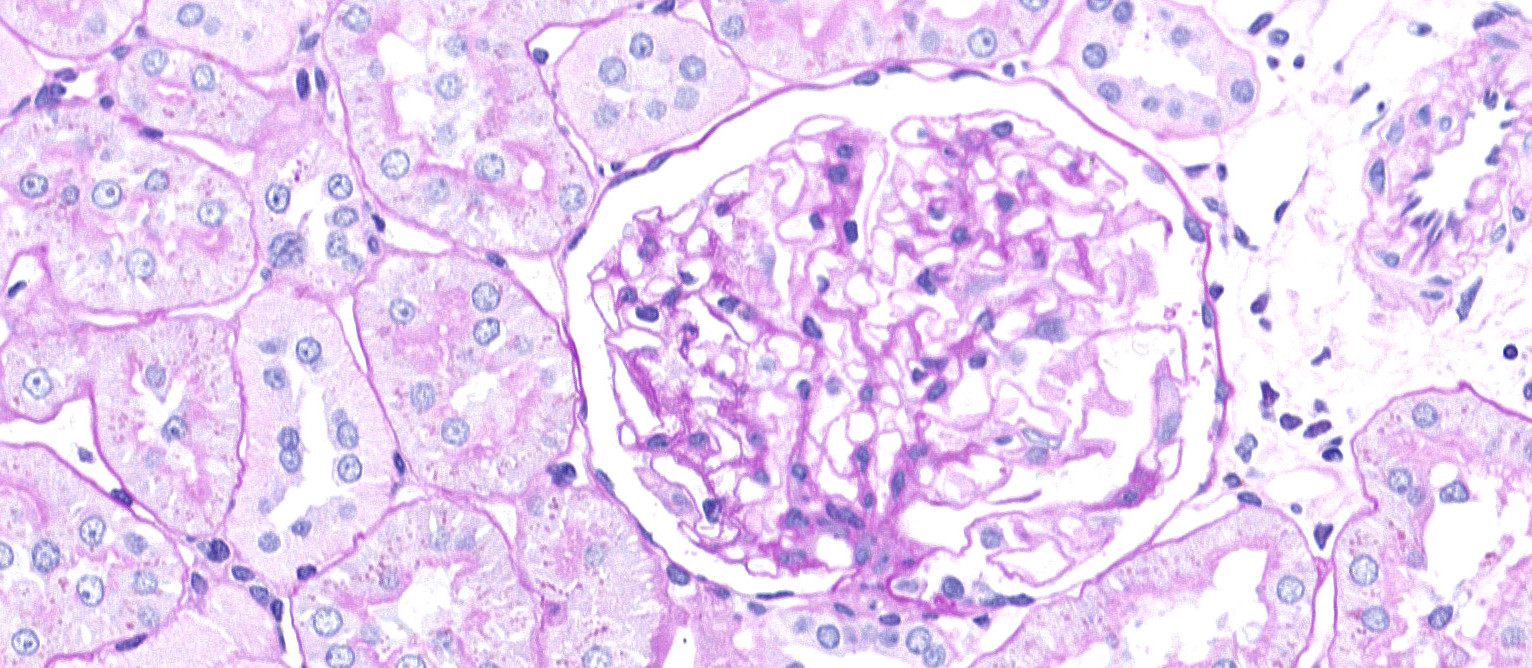

Supplement: Supplementary file 13 [file DataSheet15.ZIP › Fig 1D-PAS-TSF-61/61-6.jpeg]

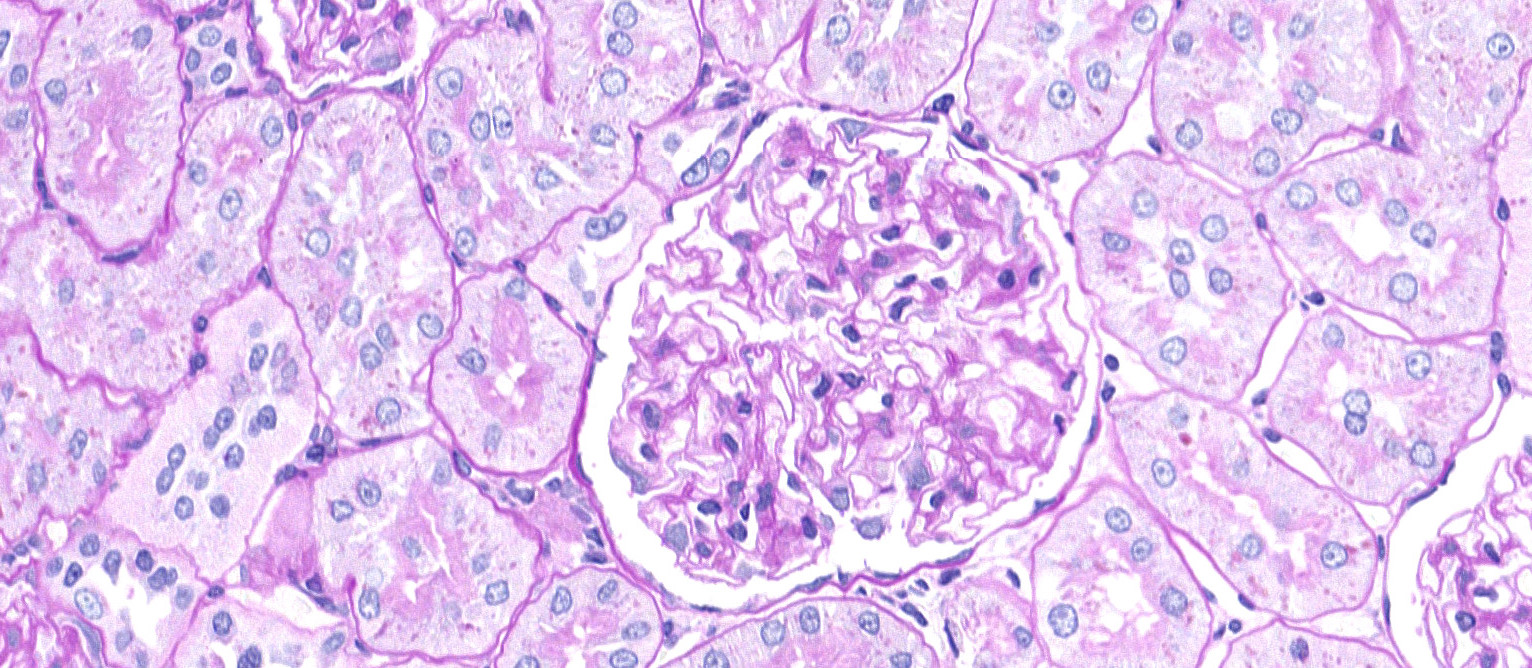

Supplement: Supplementary file 13 [file DataSheet15.ZIP › Fig 1D-PAS-TSF-61/61-7.jpeg]

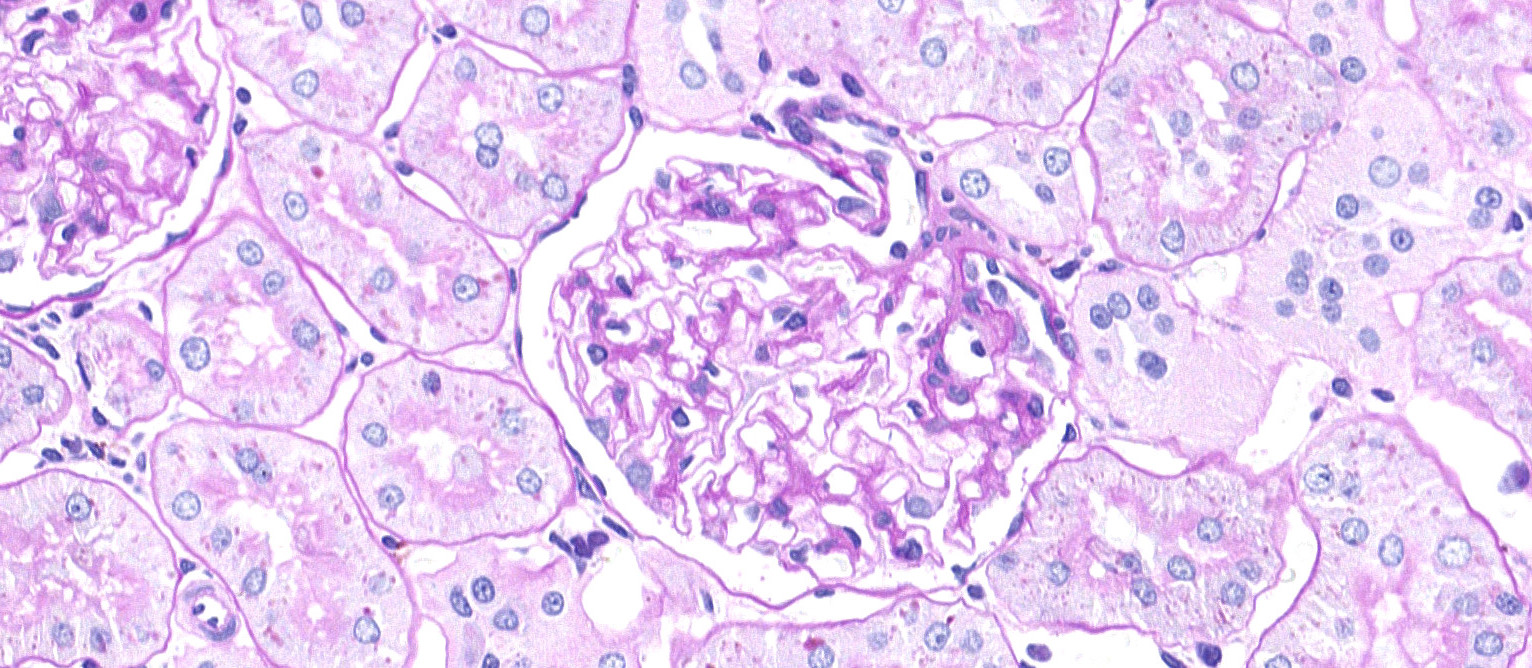

Supplement: Supplementary file 13 [file DataSheet15.ZIP › Fig 1D-PAS-TSF-61/61-8.jpeg]

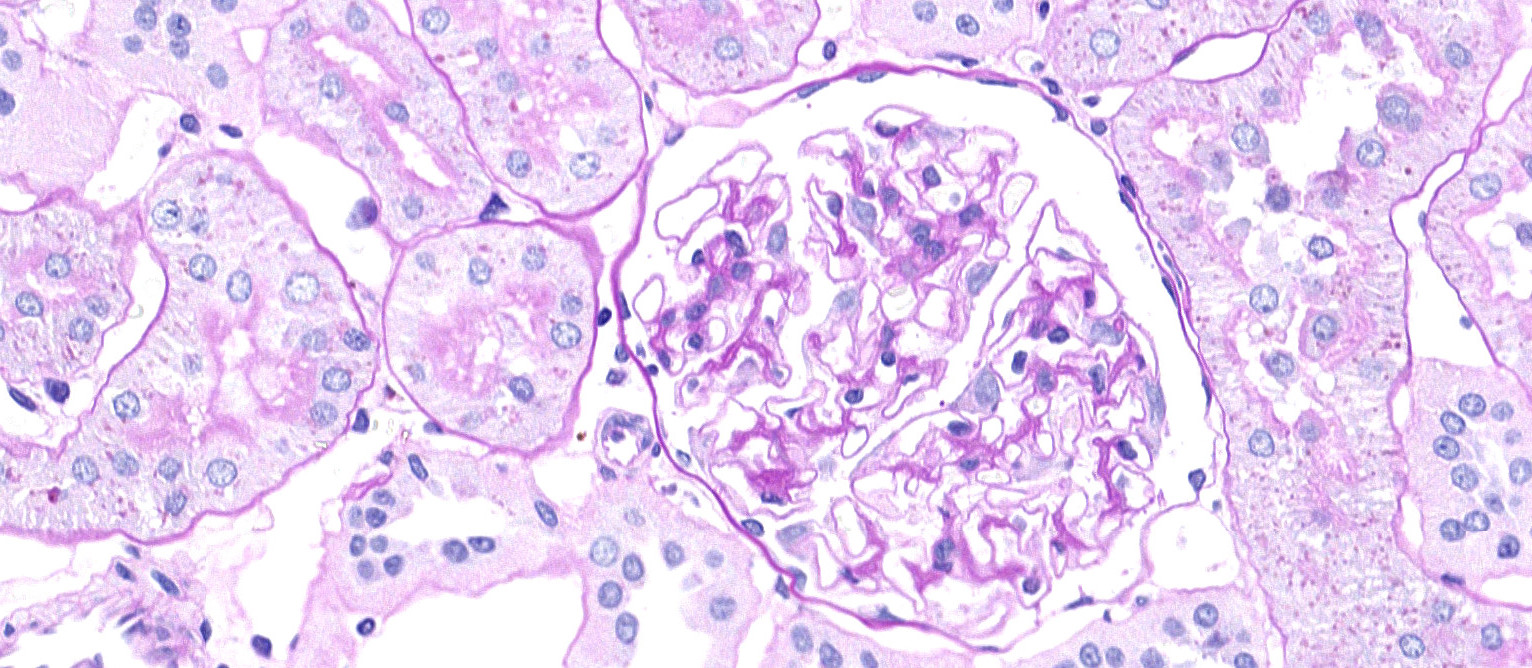

Supplement: Supplementary file 13 [file DataSheet15.ZIP › Fig 1D-PAS-TSF-61/61-9.jpeg]

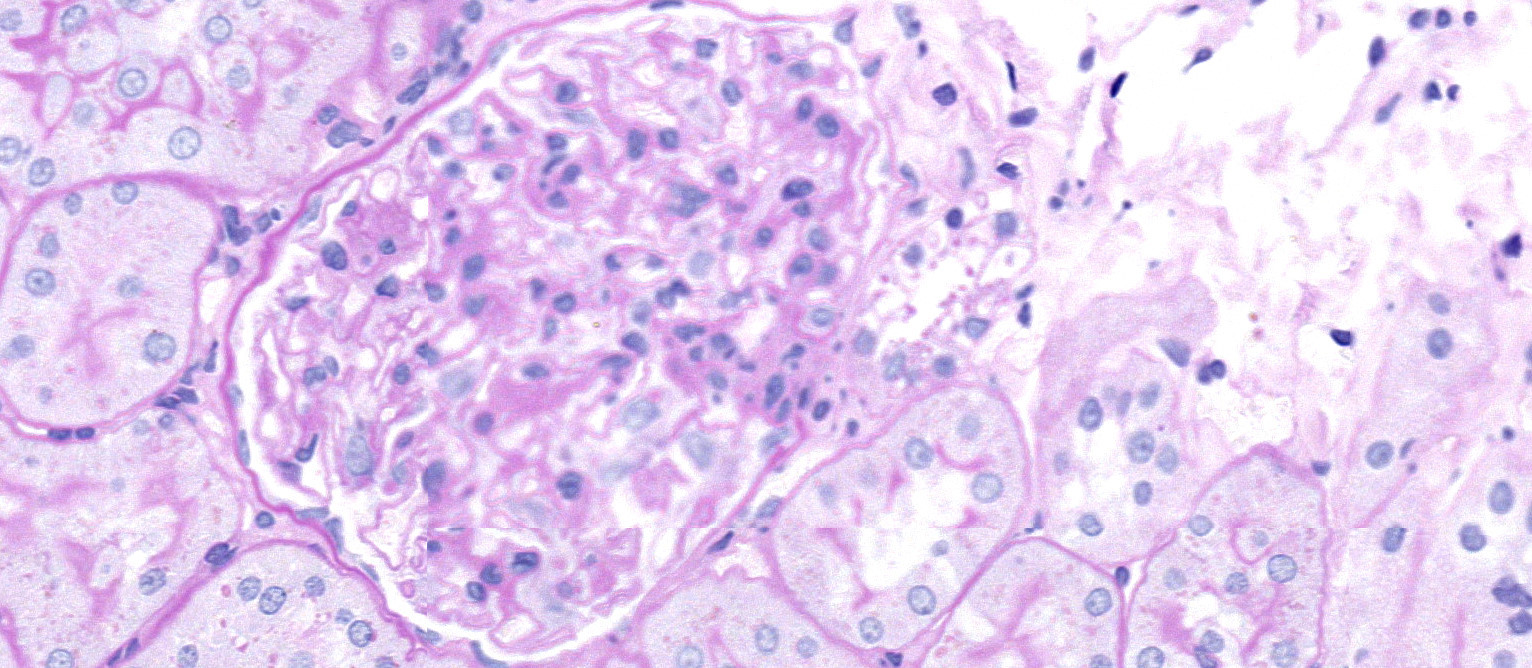

Supplement: Supplementary file 13 [file DataSheet15.ZIP › Fig 1D-PAS-TSF-62/62-1.jpeg]

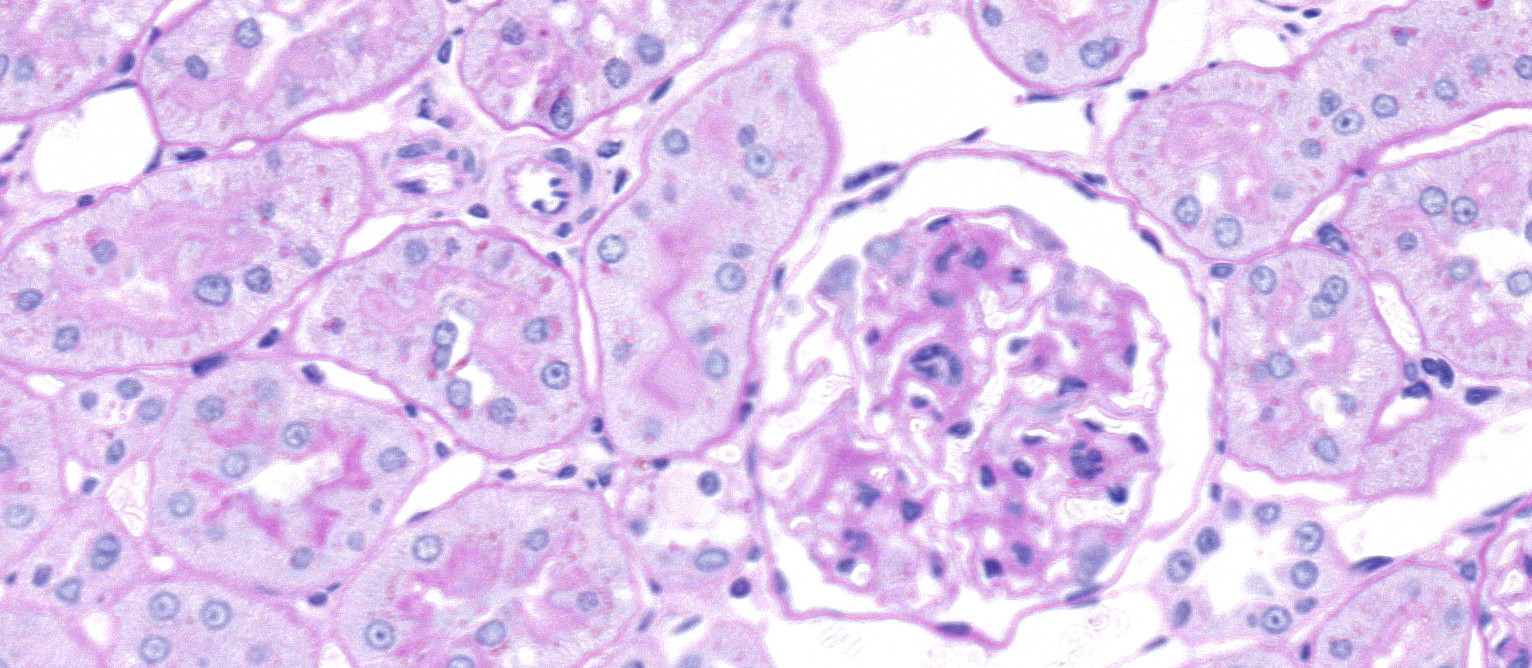

Supplement: Supplementary file 13 [file DataSheet15.ZIP › Fig 1D-PAS-TSF-62/62-10.jpeg]

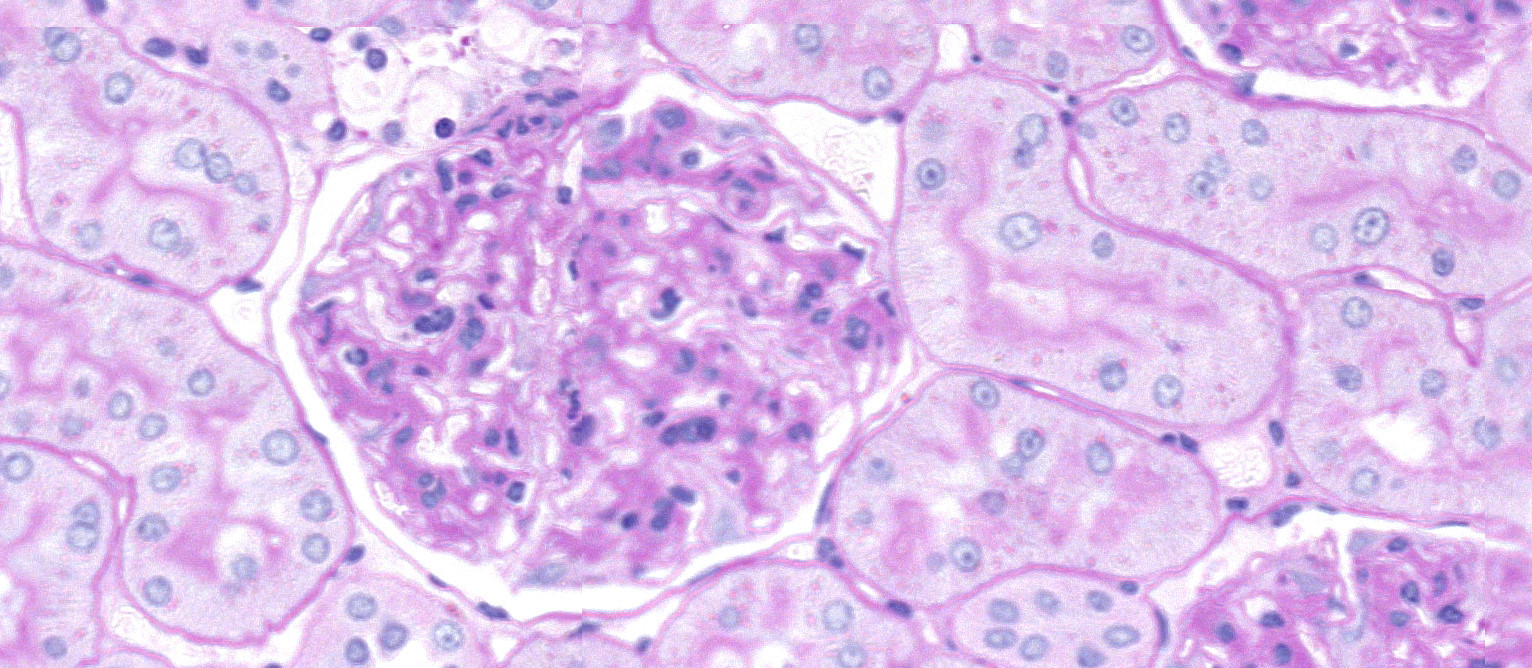

Supplement: Supplementary file 13 [file DataSheet15.ZIP › Fig 1D-PAS-TSF-62/62-11.jpeg]

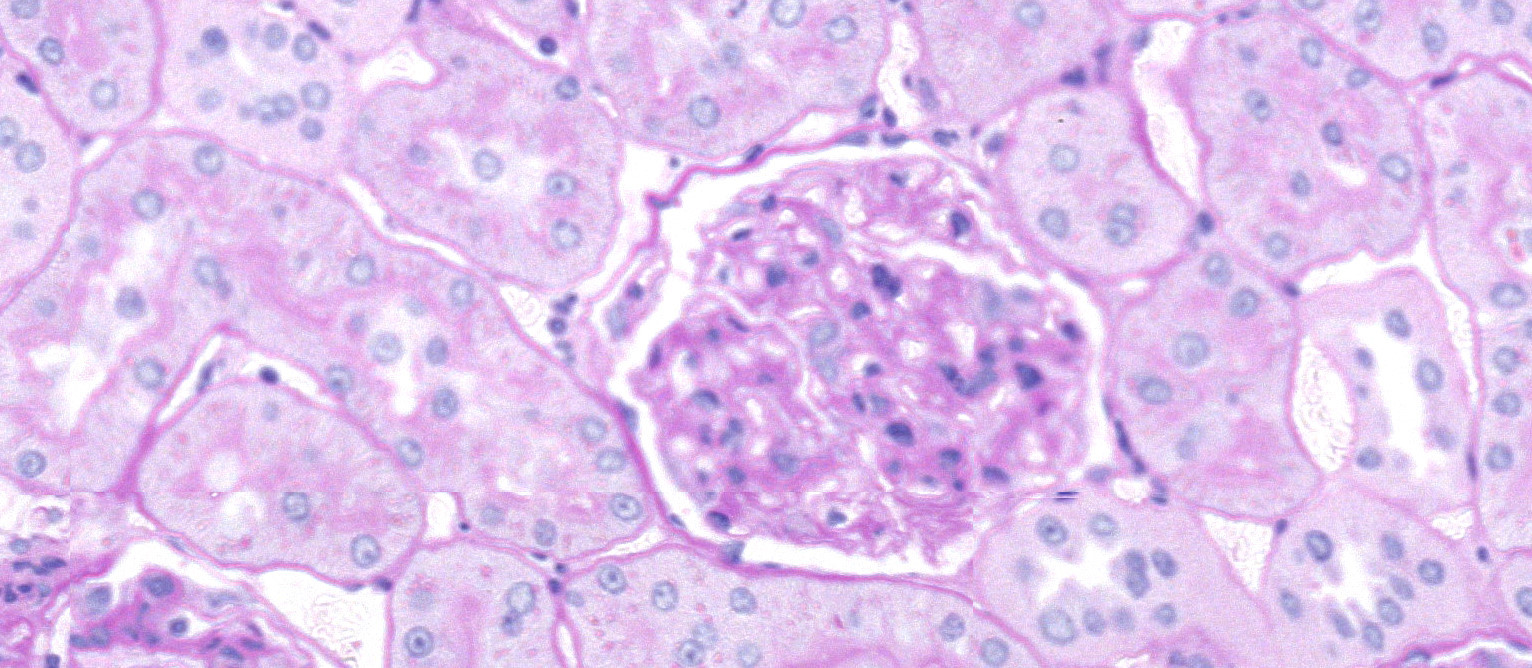

Supplement: Supplementary file 13 [file DataSheet15.ZIP › Fig 1D-PAS-TSF-62/62-12.jpeg]

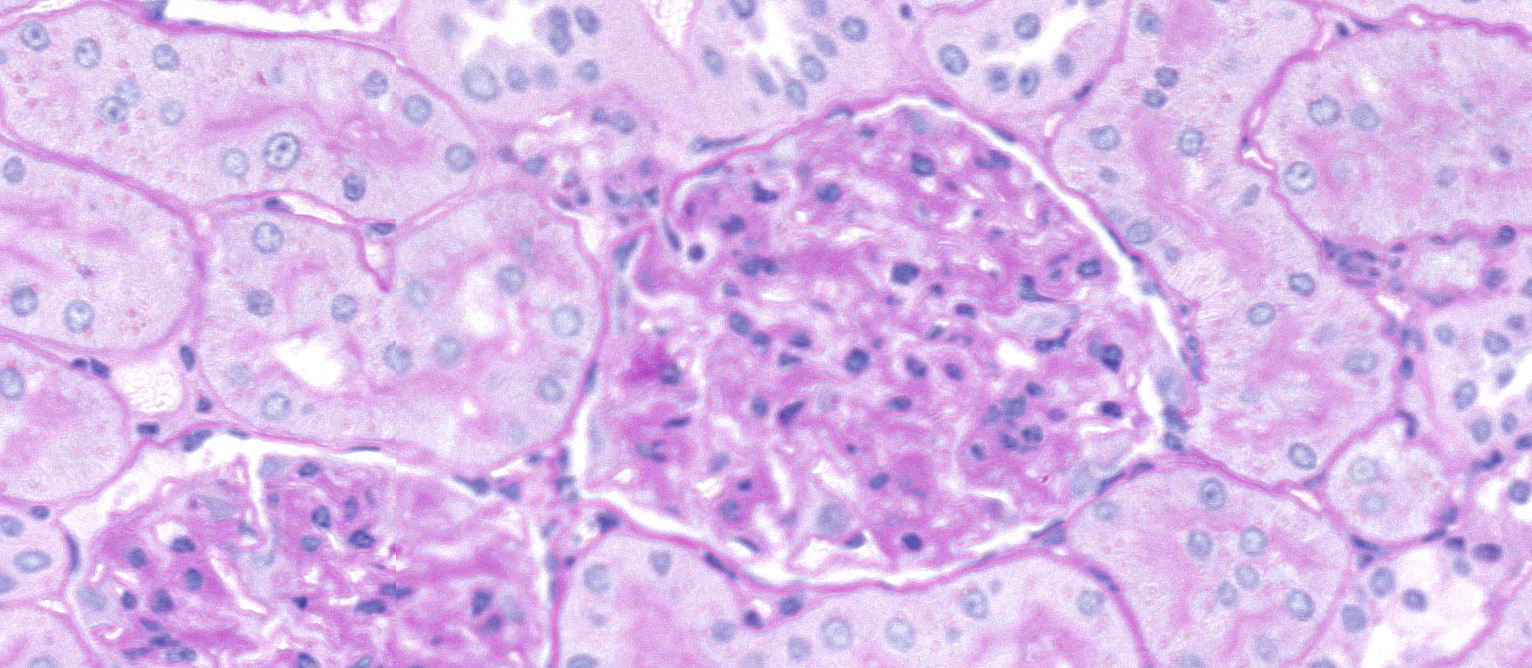

Supplement: Supplementary file 13 [file DataSheet15.ZIP › Fig 1D-PAS-TSF-62/62-13.jpeg]

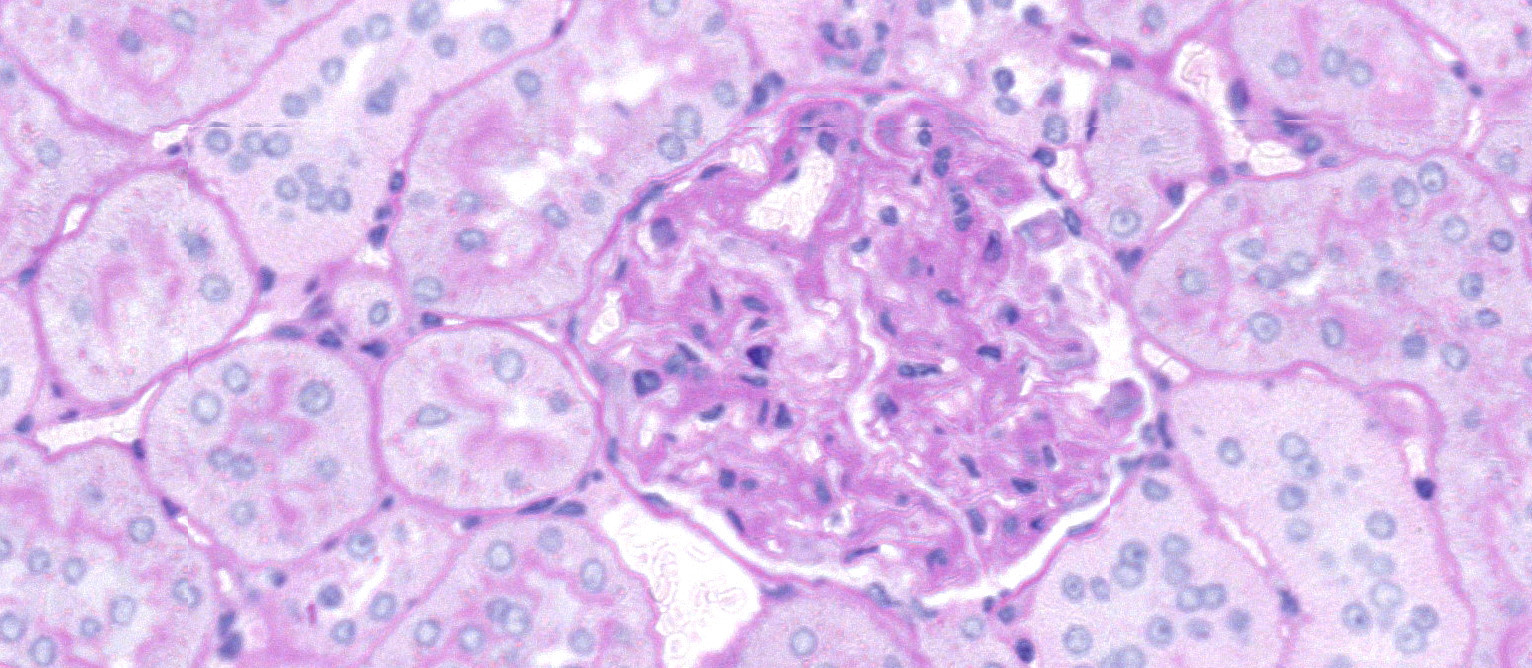

Supplement: Supplementary file 13 [file DataSheet15.ZIP › Fig 1D-PAS-TSF-62/62-14.jpeg]

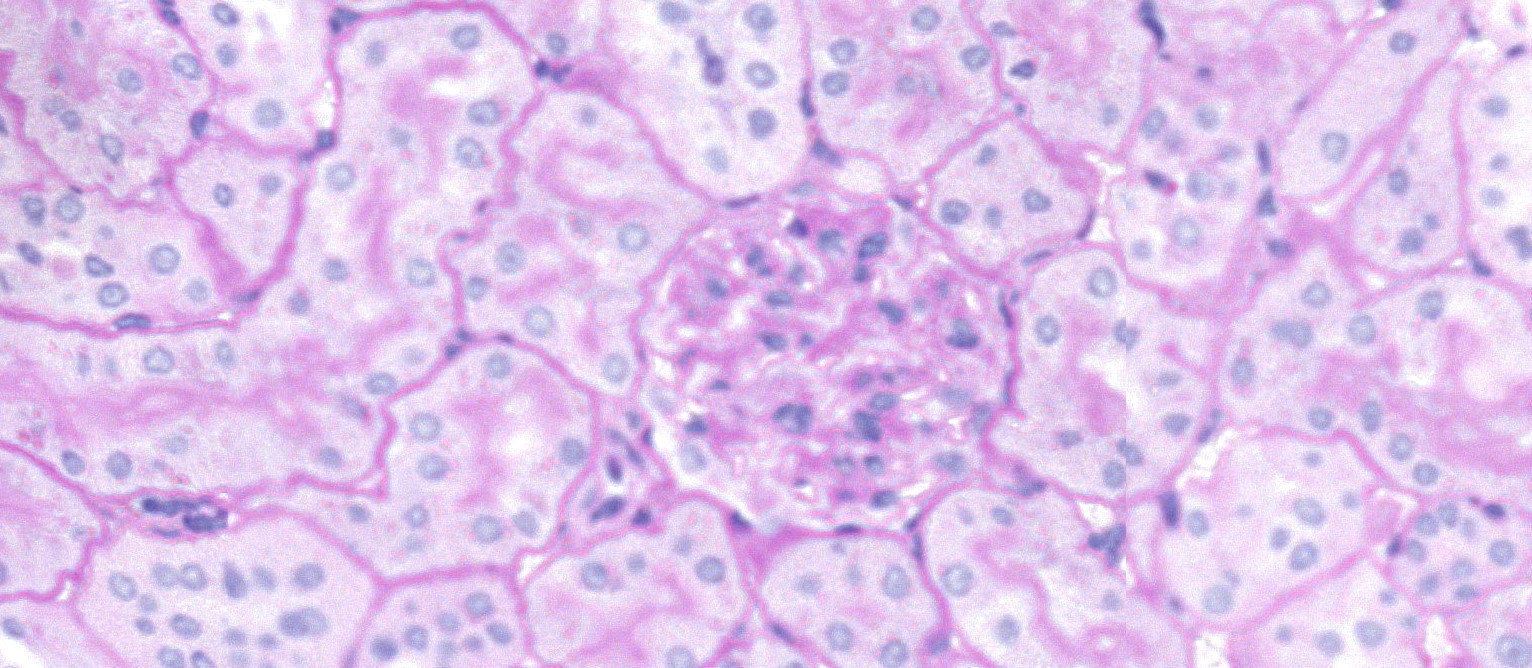

Supplement: Supplementary file 13 [file DataSheet15.ZIP › Fig 1D-PAS-TSF-62/62-15.jpeg]

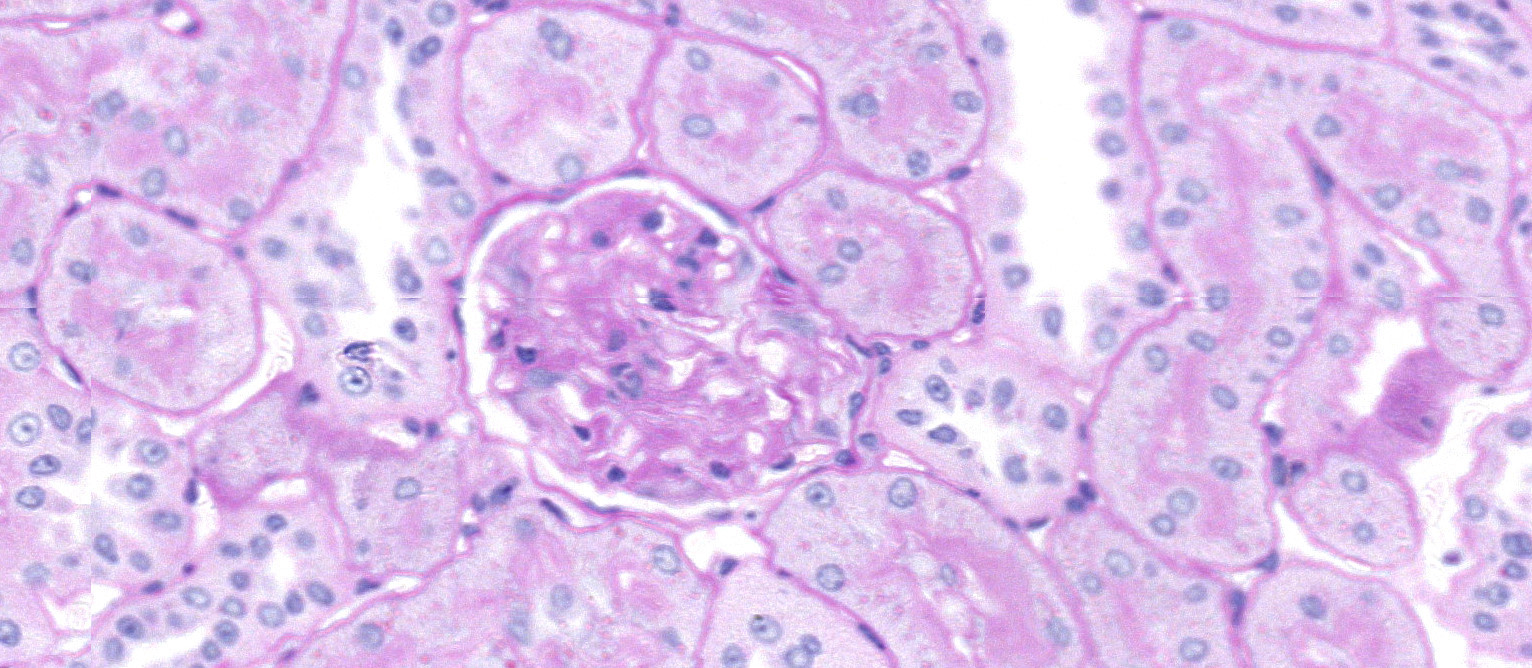

Supplement: Supplementary file 13 [file DataSheet15.ZIP › Fig 1D-PAS-TSF-62/62-16.jpeg]

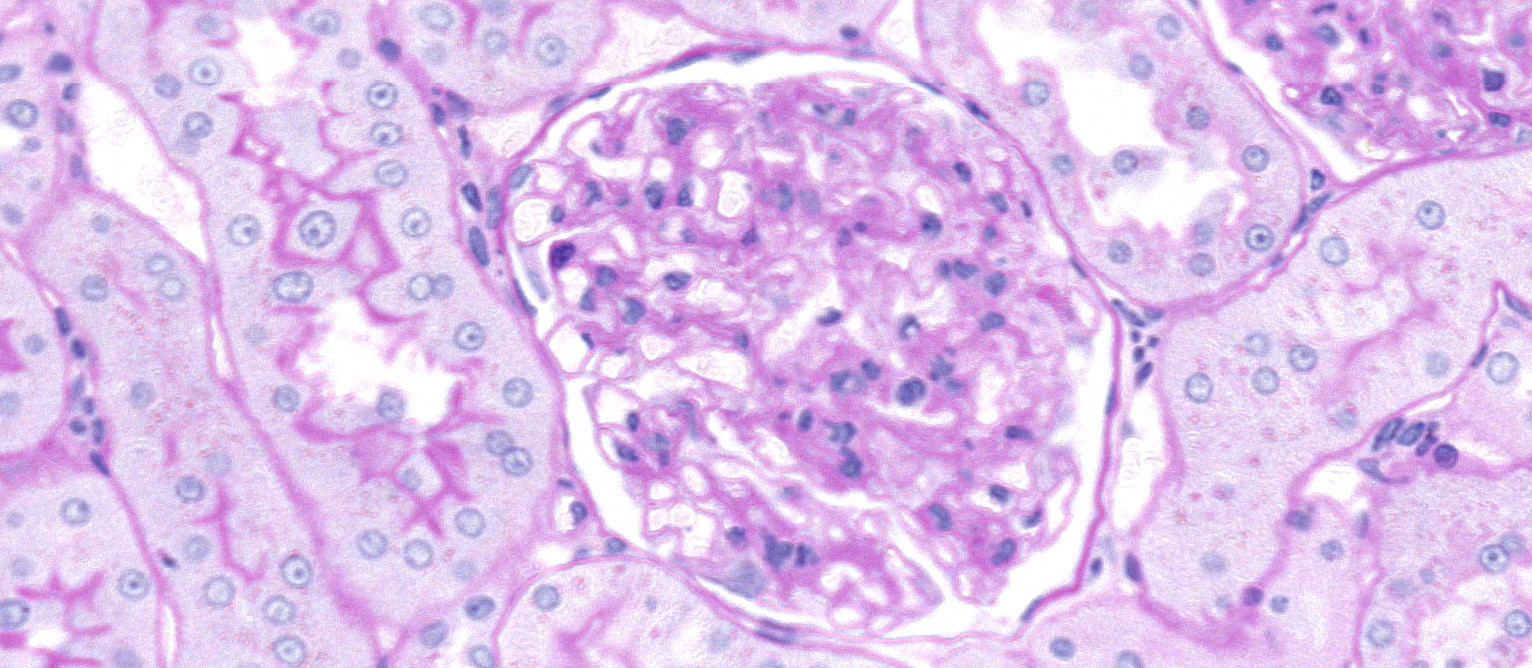

Supplement: Supplementary file 13 [file DataSheet15.ZIP › Fig 1D-PAS-TSF-62/62-17.jpeg]

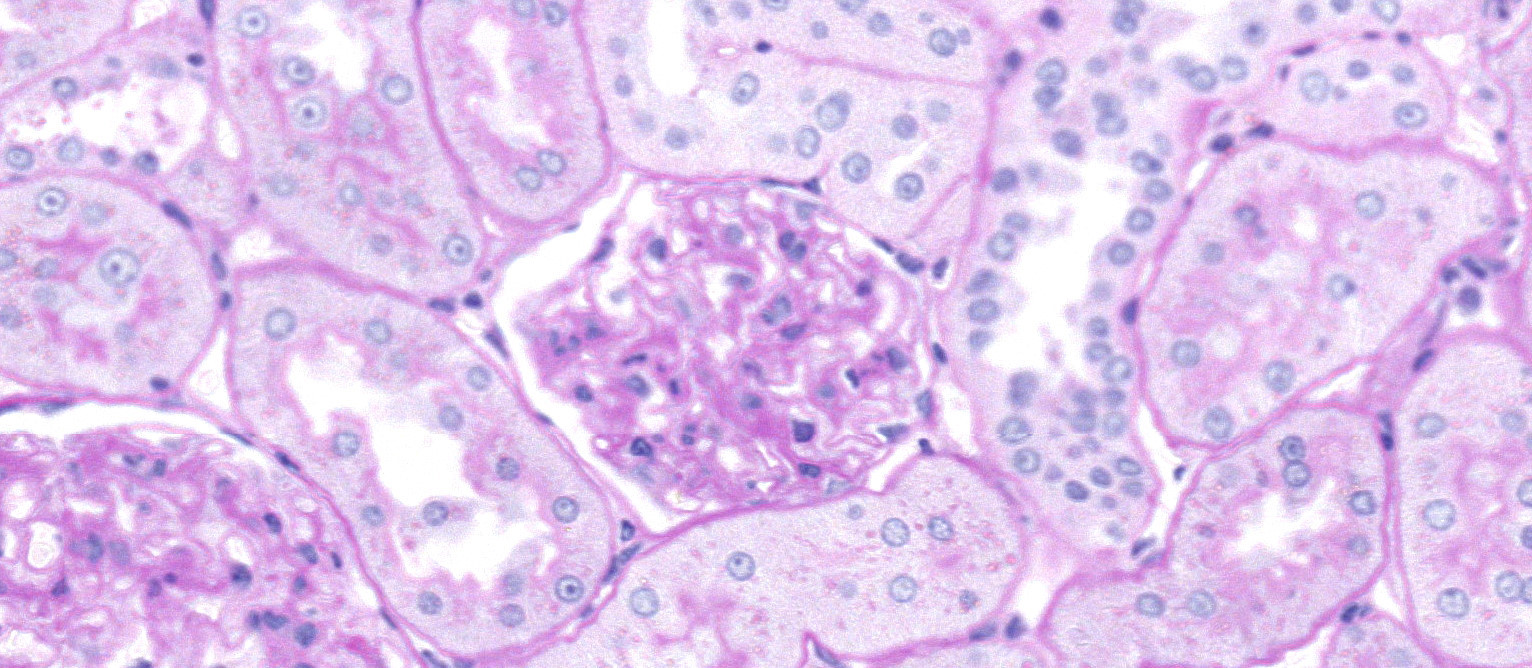

Supplement: Supplementary file 13 [file DataSheet15.ZIP › Fig 1D-PAS-TSF-62/62-18.jpeg]

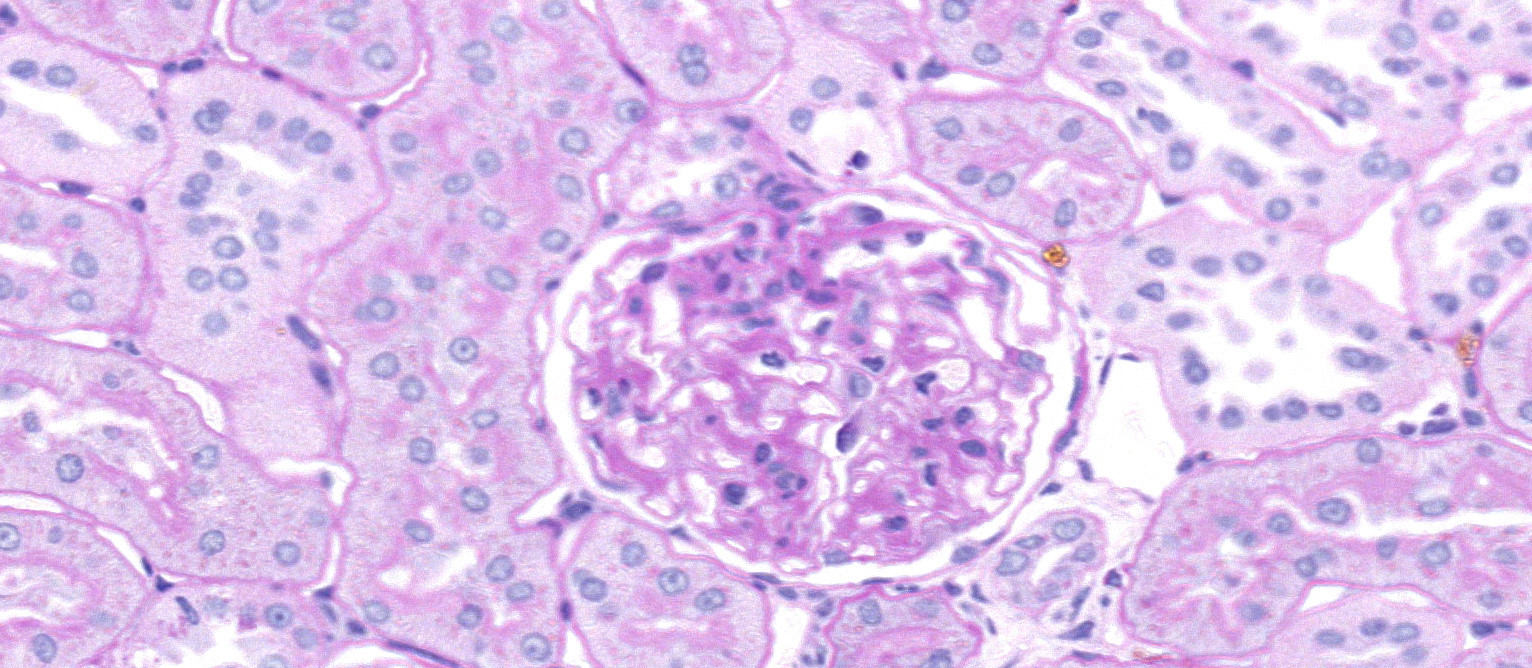

Supplement: Supplementary file 13 [file DataSheet15.ZIP › Fig 1D-PAS-TSF-62/62-19.jpeg]

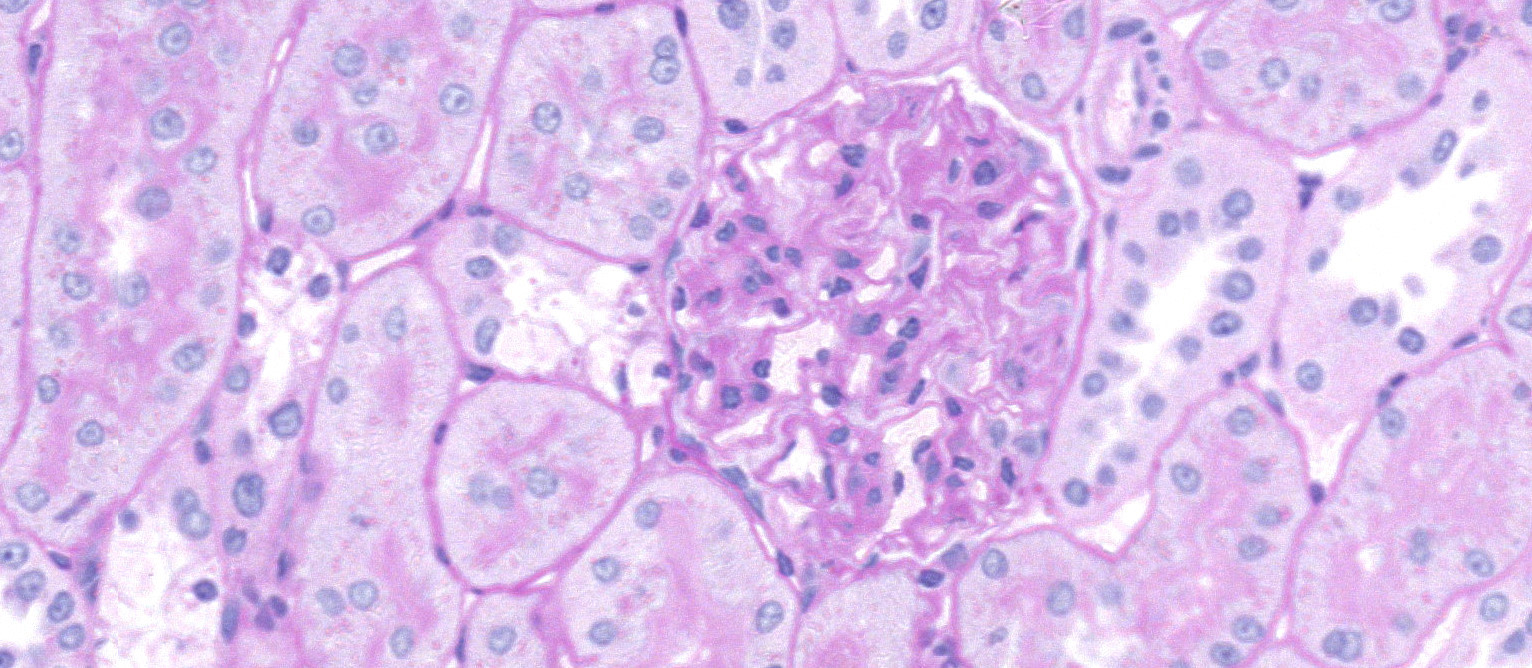

Supplement: Supplementary file 13 [file DataSheet15.ZIP › Fig 1D-PAS-TSF-62/62-2.jpeg]

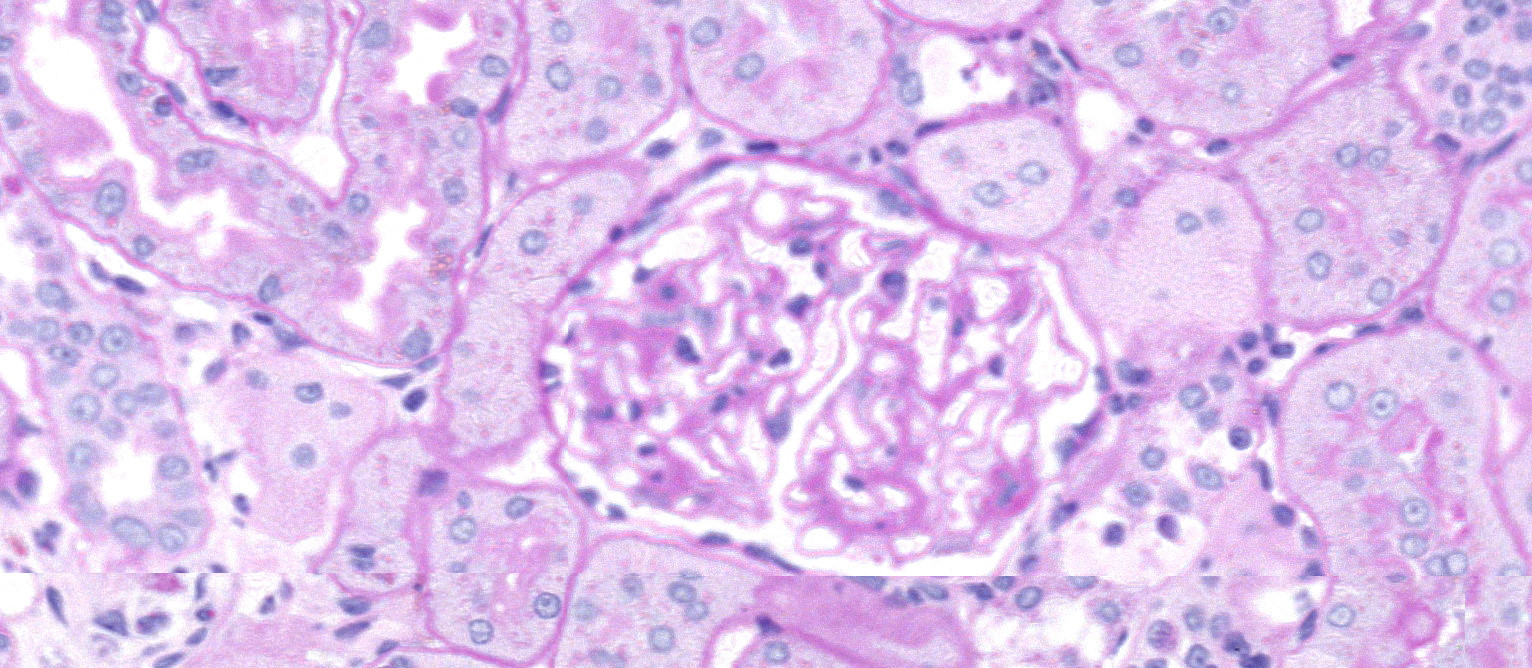

Supplement: Supplementary file 13 [file DataSheet15.ZIP › Fig 1D-PAS-TSF-62/62-20.jpeg]

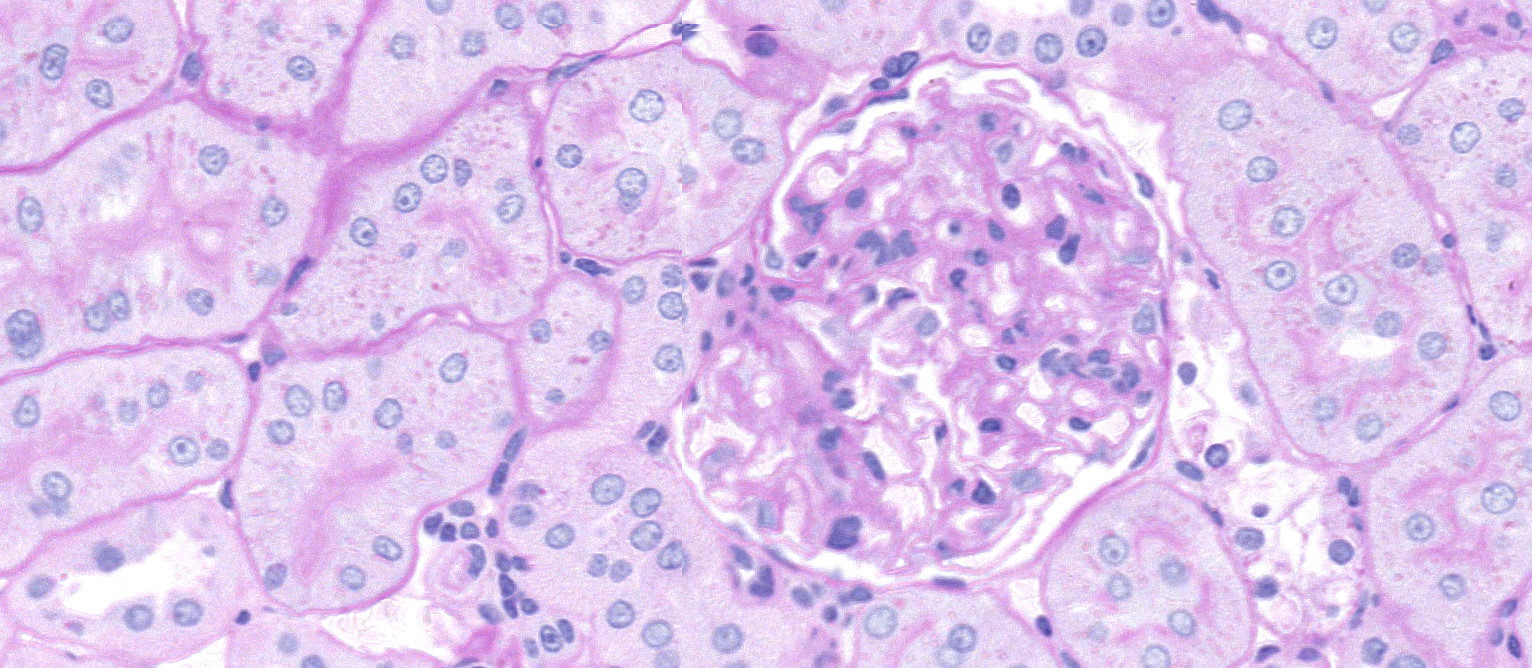

Supplement: Supplementary file 13 [file DataSheet15.ZIP › Fig 1D-PAS-TSF-62/62-3.jpeg]

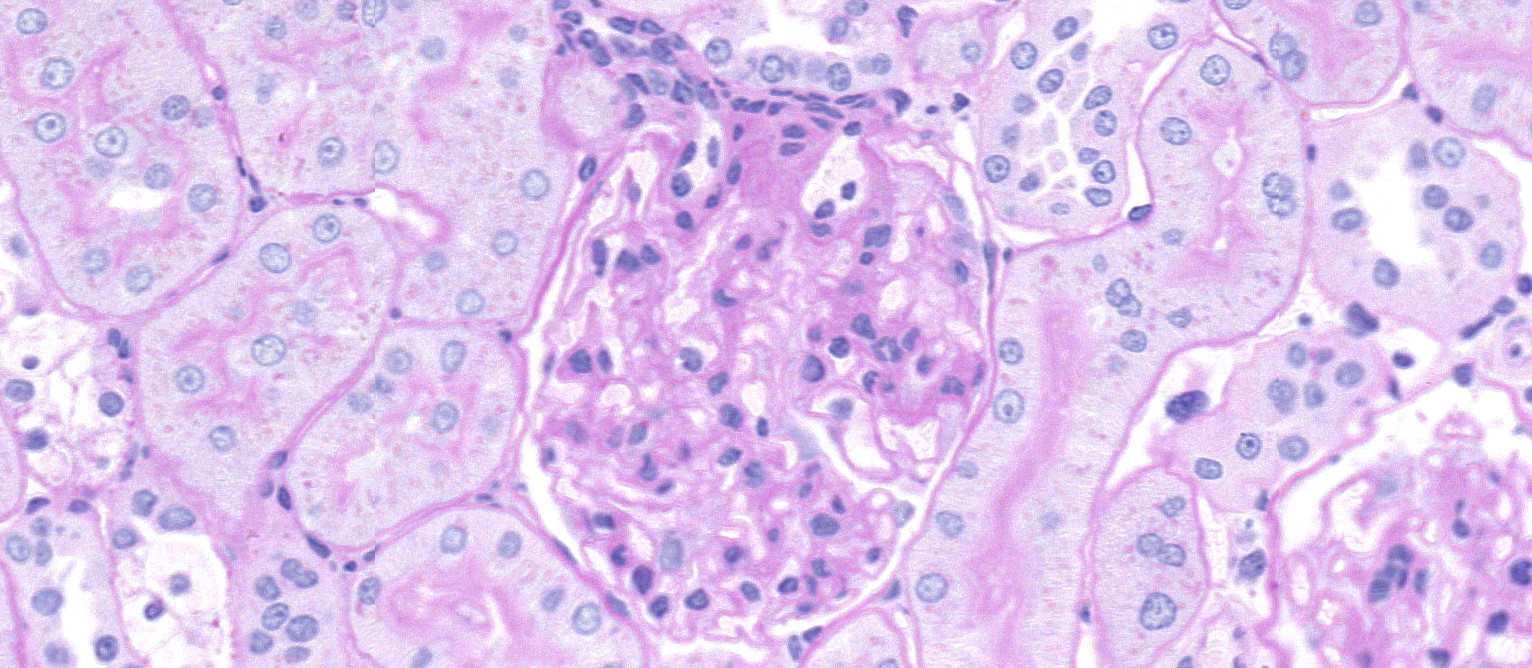

Supplement: Supplementary file 13 [file DataSheet15.ZIP › Fig 1D-PAS-TSF-62/62-4.jpeg]

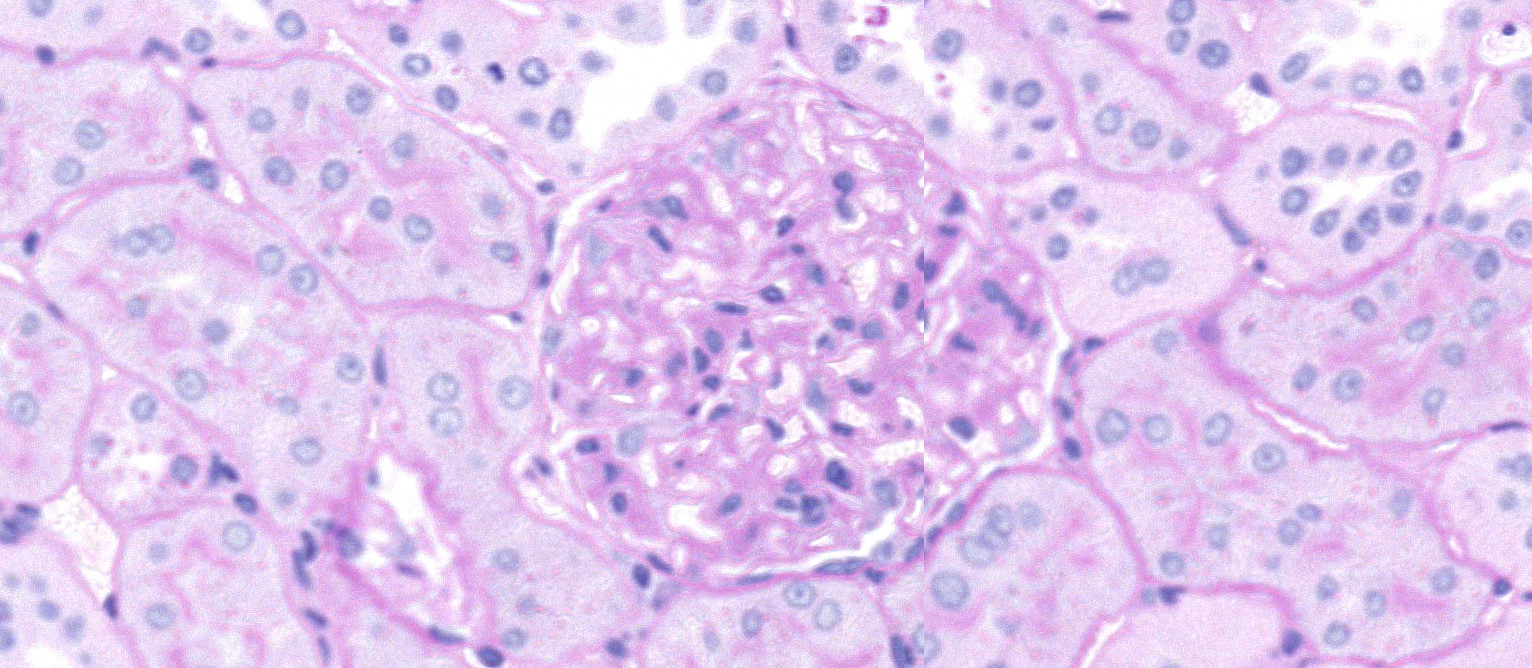

Supplement: Supplementary file 13 [file DataSheet15.ZIP › Fig 1D-PAS-TSF-62/62-5.jpeg]

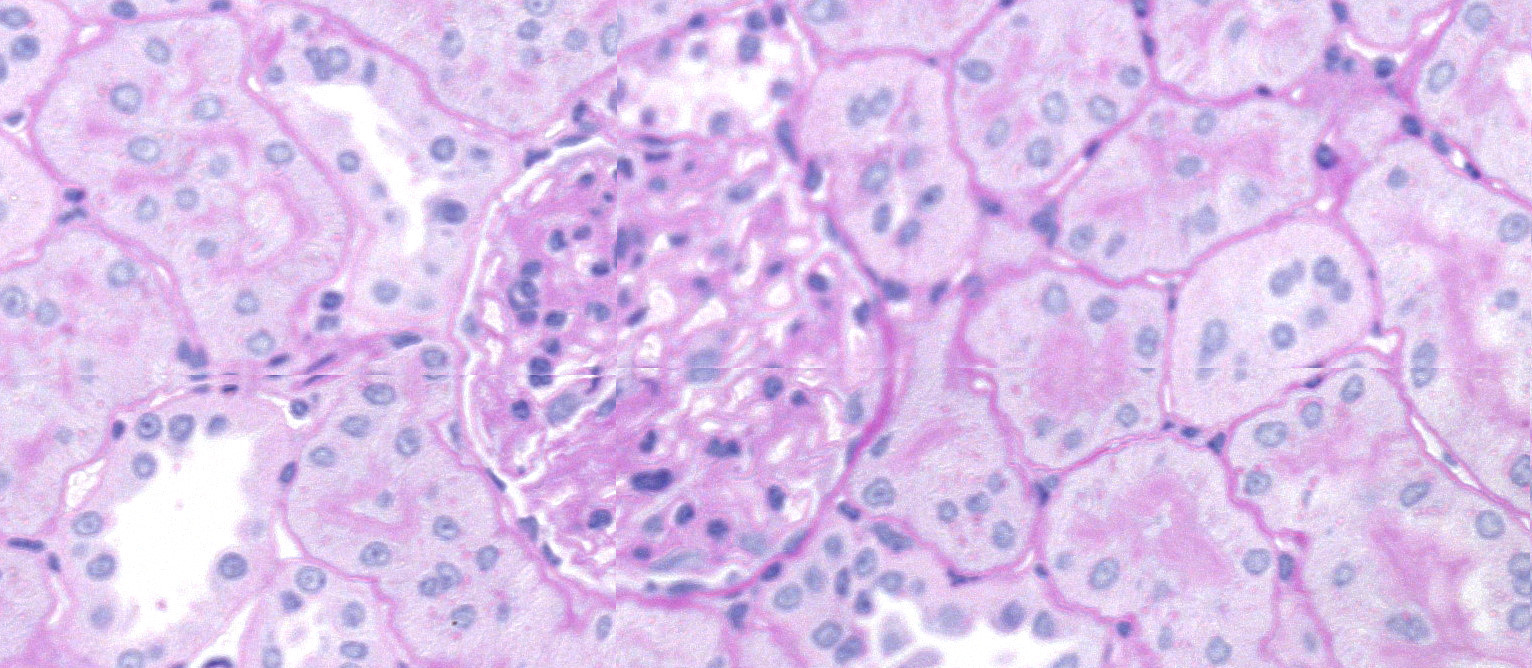

Supplement: Supplementary file 13 [file DataSheet15.ZIP › Fig 1D-PAS-TSF-62/62-6.jpeg]

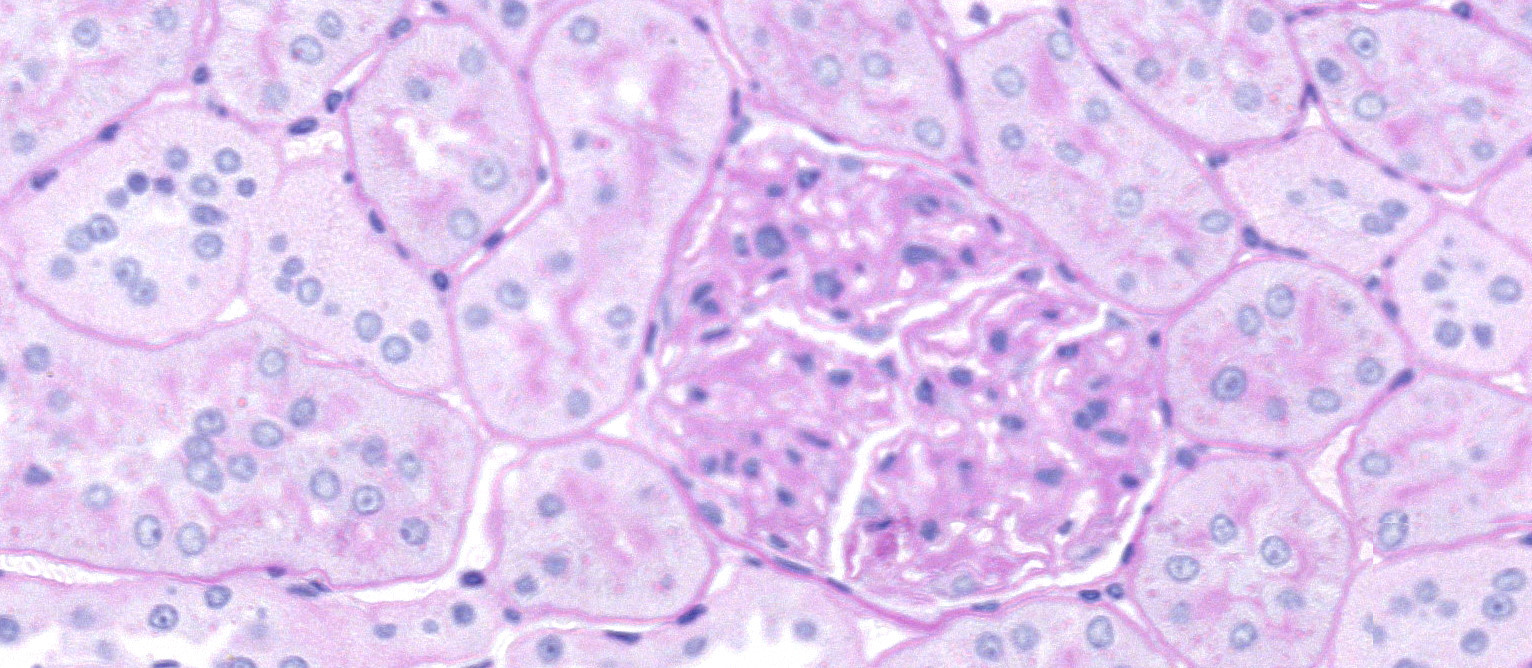

Supplement: Supplementary file 13 [file DataSheet15.ZIP › Fig 1D-PAS-TSF-62/62-7.jpeg]

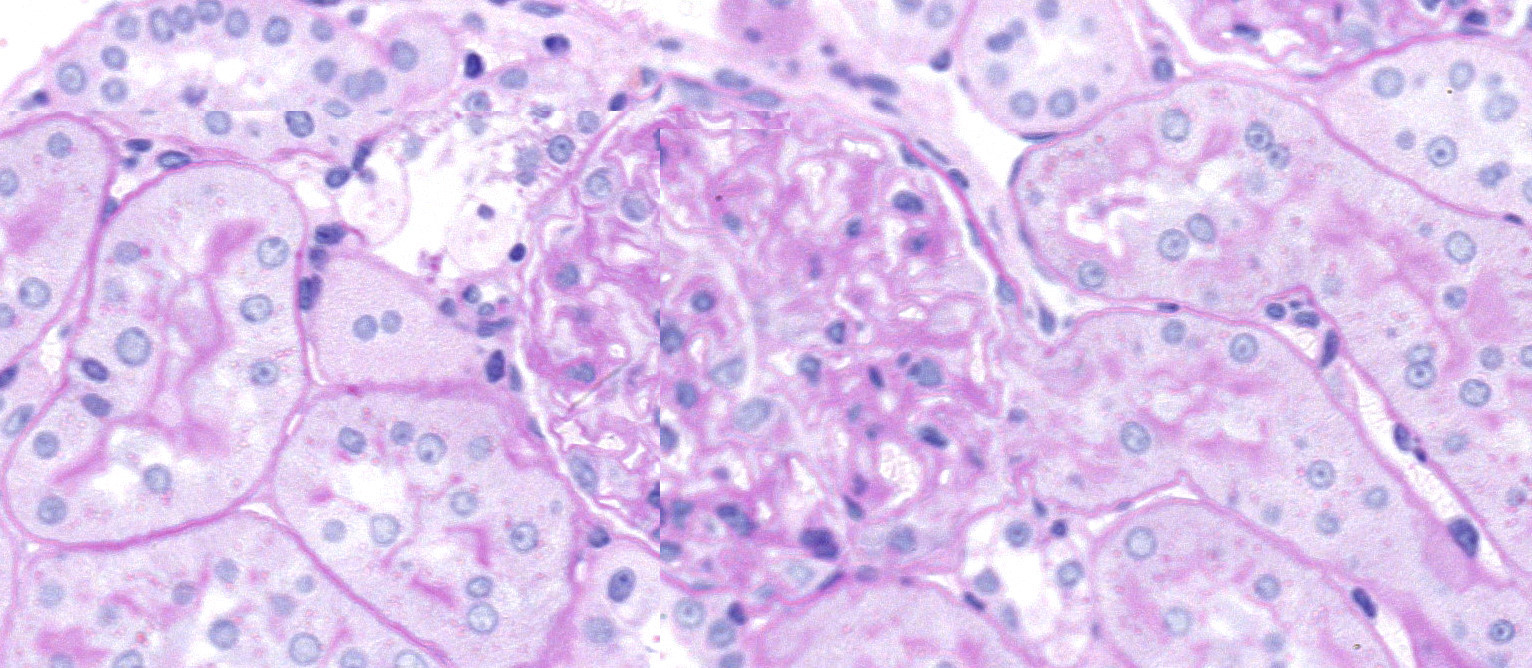

Supplement: Supplementary file 13 [file DataSheet15.ZIP › Fig 1D-PAS-TSF-62/62-8.jpeg]

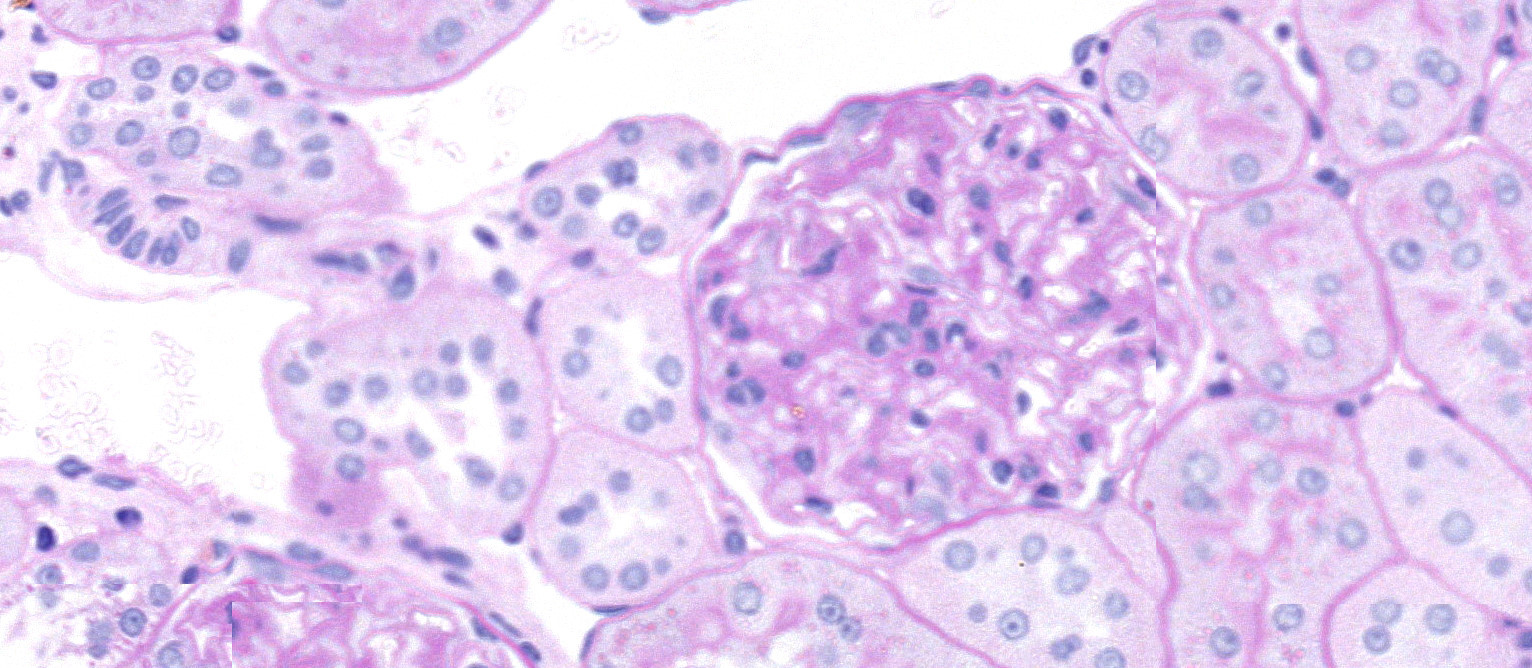

Supplement: Supplementary file 13 [file DataSheet15.ZIP › Fig 1D-PAS-TSF-62/62-9.jpeg]

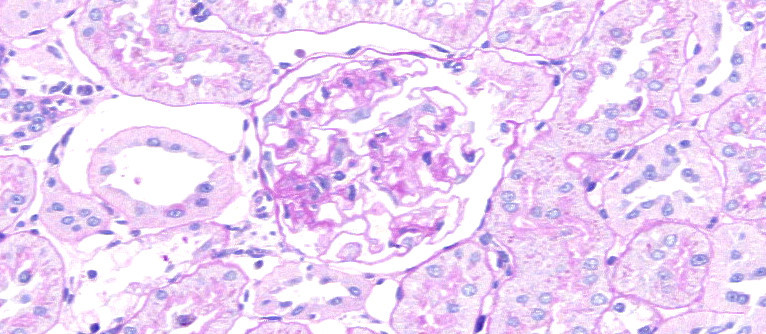

Supplement: Supplementary file 13 [file DataSheet15.ZIP › Fig 1D-PAS-TSF-63/63-1.jpeg]

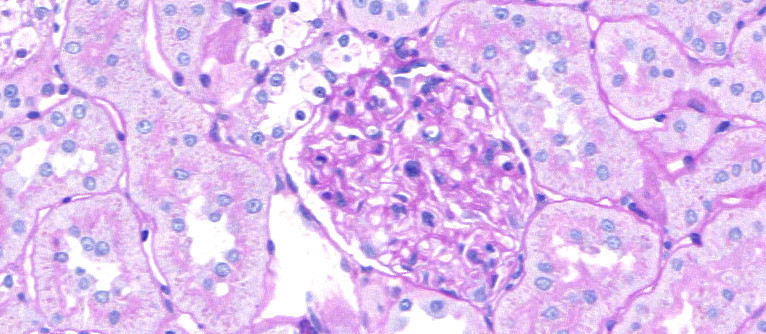

Supplement: Supplementary file 13 [file DataSheet15.ZIP › Fig 1D-PAS-TSF-63/63-10.jpeg]

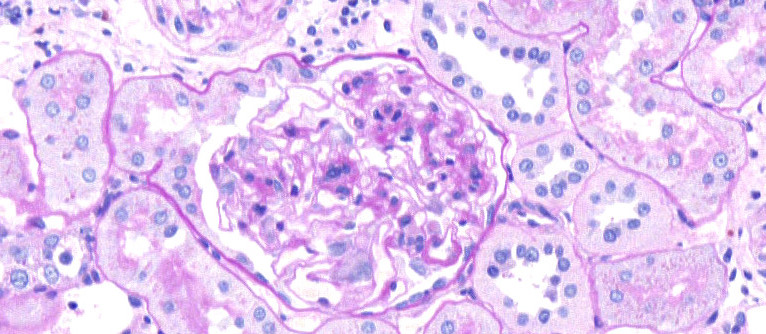

Supplement: Supplementary file 13 [file DataSheet15.ZIP › Fig 1D-PAS-TSF-63/63-11.jpeg]

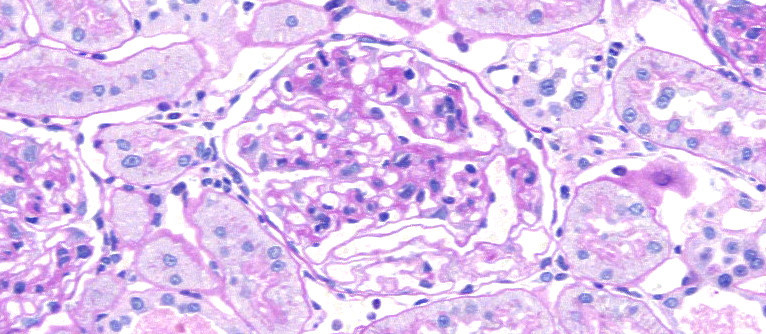

Supplement: Supplementary file 13 [file DataSheet15.ZIP › Fig 1D-PAS-TSF-63/63-12.jpeg]

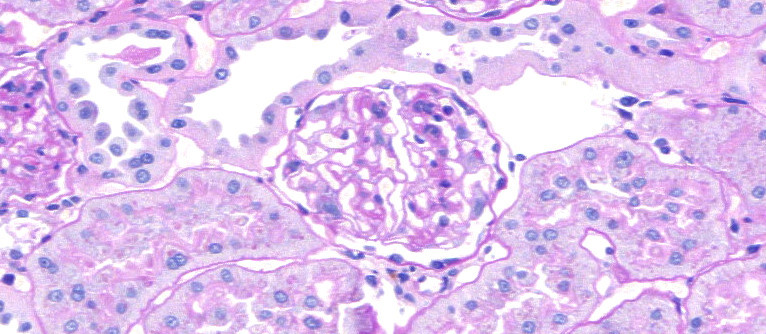

Supplement: Supplementary file 13 [file DataSheet15.ZIP › Fig 1D-PAS-TSF-63/63-13.jpeg]

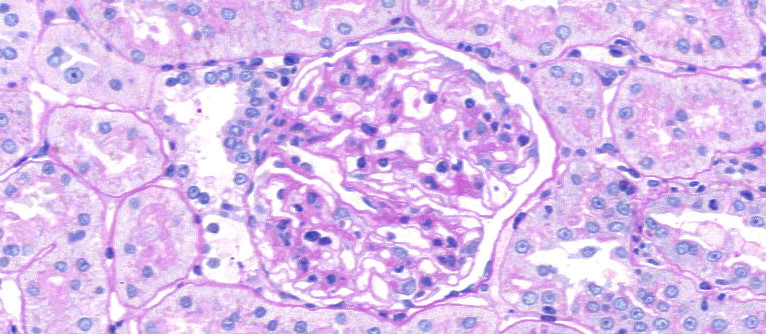

Supplement: Supplementary file 13 [file DataSheet15.ZIP › Fig 1D-PAS-TSF-63/63-14.jpeg]

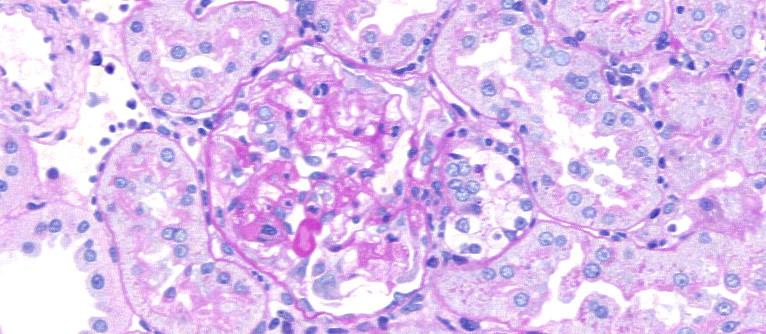

Supplement: Supplementary file 13 [file DataSheet15.ZIP › Fig 1D-PAS-TSF-63/63-15.jpeg]

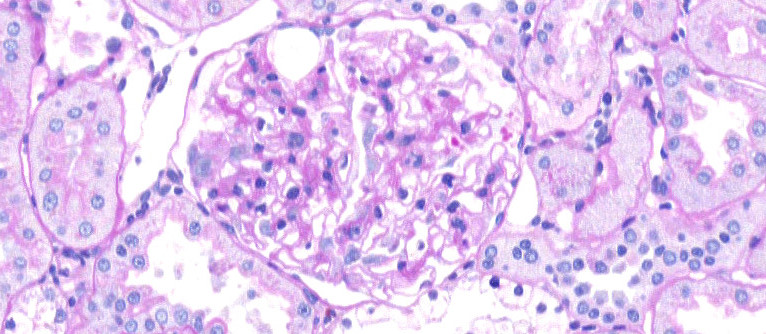

Supplement: Supplementary file 13 [file DataSheet15.ZIP › Fig 1D-PAS-TSF-63/63-16.jpeg]

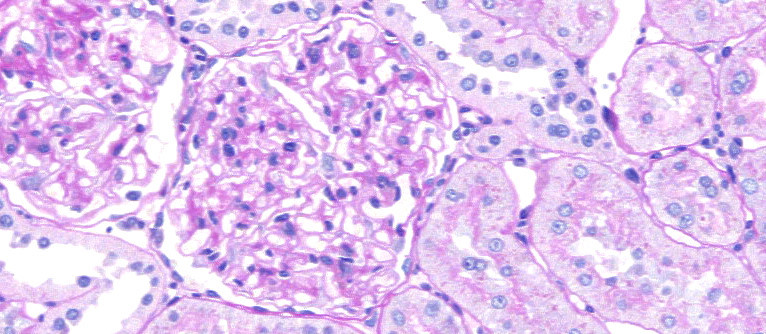

Supplement: Supplementary file 13 [file DataSheet15.ZIP › Fig 1D-PAS-TSF-63/63-17.jpeg]

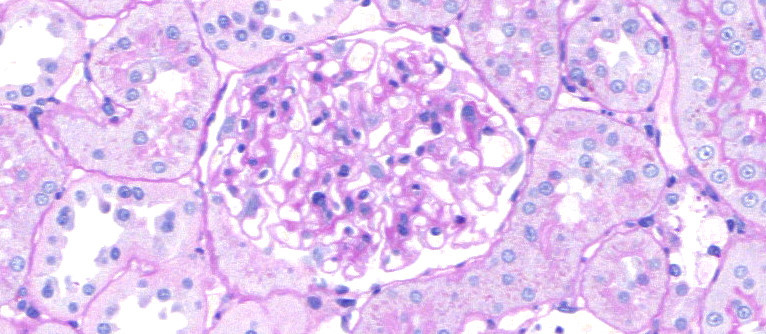

Supplement: Supplementary file 13 [file DataSheet15.ZIP › Fig 1D-PAS-TSF-63/63-18.jpeg]

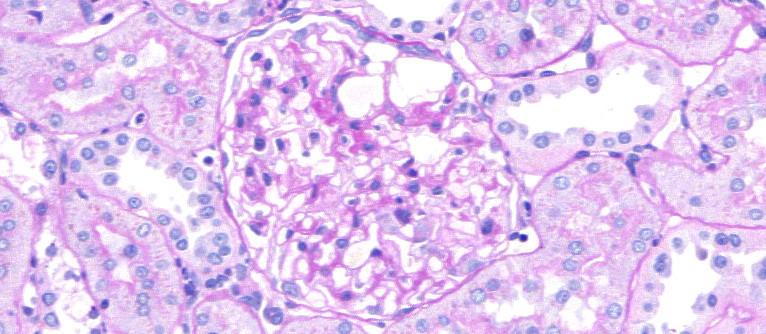

Supplement: Supplementary file 13 [file DataSheet15.ZIP › Fig 1D-PAS-TSF-63/63-19.jpeg]

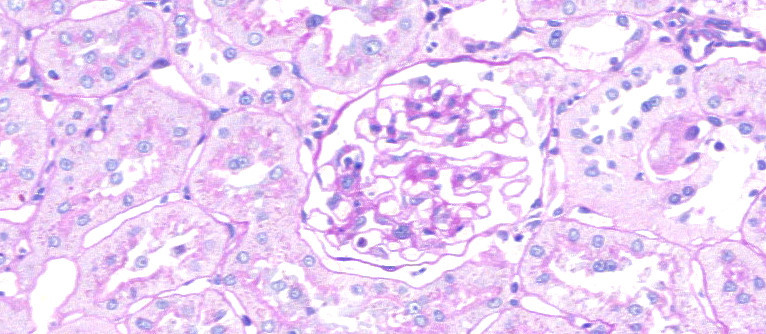

Supplement: Supplementary file 13 [file DataSheet15.ZIP › Fig 1D-PAS-TSF-63/63-2.jpeg]

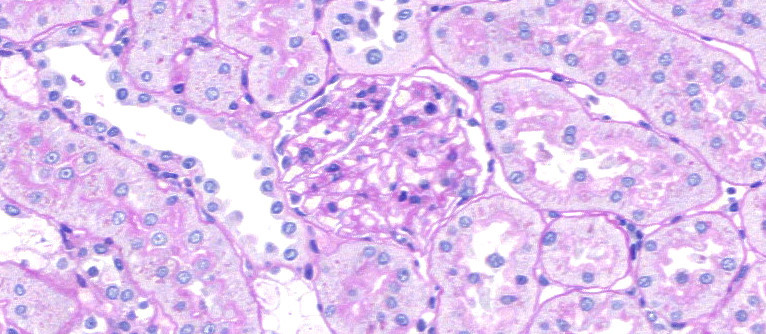

Supplement: Supplementary file 13 [file DataSheet15.ZIP › Fig 1D-PAS-TSF-63/63-20.jpeg]

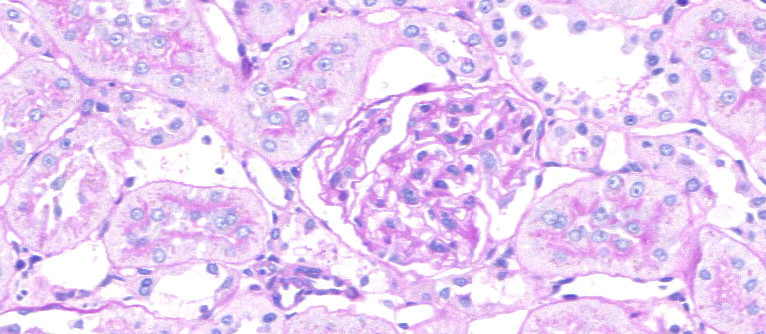

Supplement: Supplementary file 13 [file DataSheet15.ZIP › Fig 1D-PAS-TSF-63/63-3.jpeg]

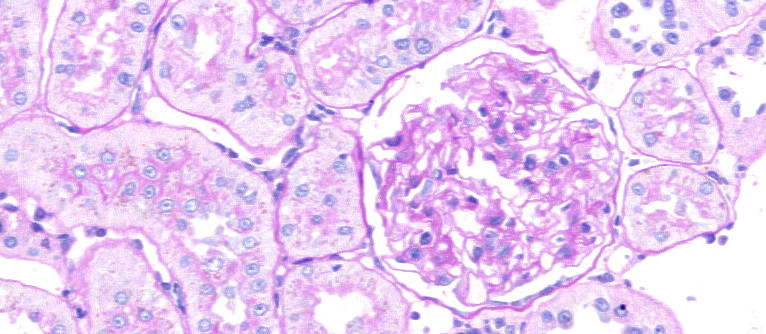

Supplement: Supplementary file 13 [file DataSheet15.ZIP › Fig 1D-PAS-TSF-63/63-4.jpeg]

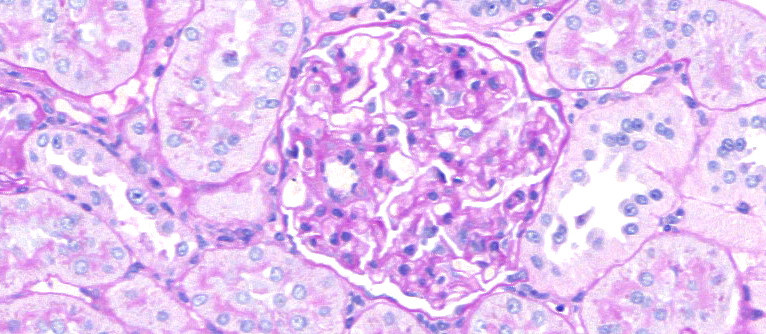

Supplement: Supplementary file 13 [file DataSheet15.ZIP › Fig 1D-PAS-TSF-63/63-5.jpeg]

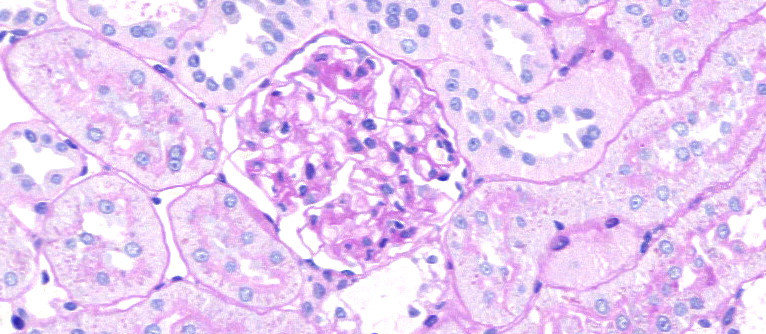

Supplement: Supplementary file 13 [file DataSheet15.ZIP › Fig 1D-PAS-TSF-63/63-6.jpeg]

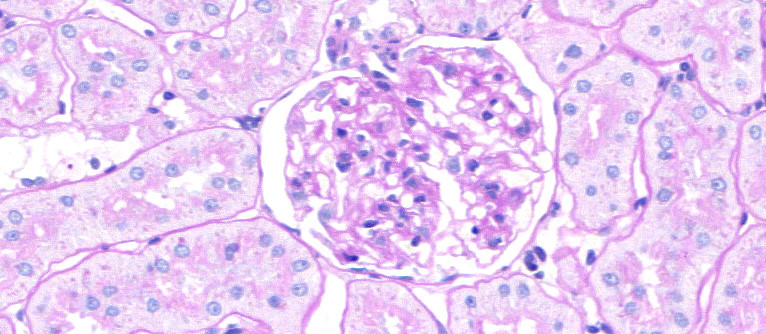

Supplement: Supplementary file 13 [file DataSheet15.ZIP › Fig 1D-PAS-TSF-63/63-7.jpeg]

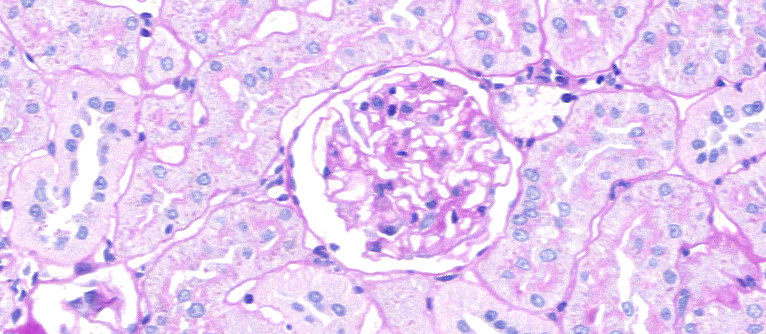

Supplement: Supplementary file 13 [file DataSheet15.ZIP › Fig 1D-PAS-TSF-63/63-8.jpeg]

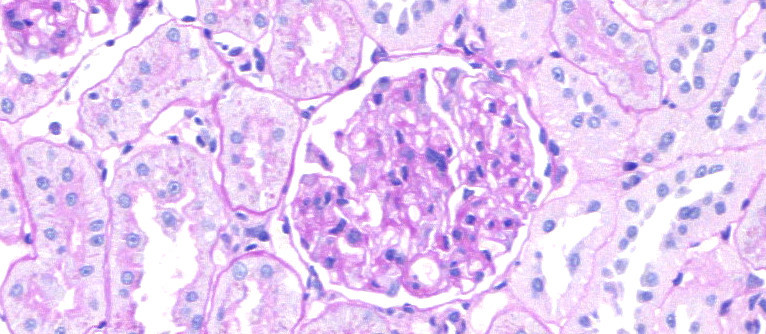

Supplement: Supplementary file 13 [file DataSheet15.ZIP › Fig 1D-PAS-TSF-63/63-9.jpeg]

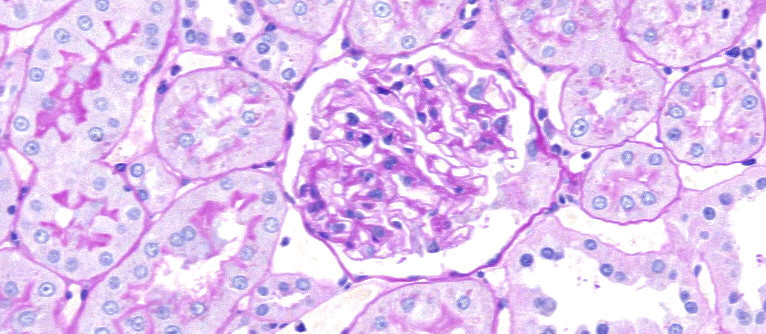

Supplement: Supplementary file 13 [file DataSheet15.ZIP › Fig 1D-PAS-TSF-65/65-1.jpeg]

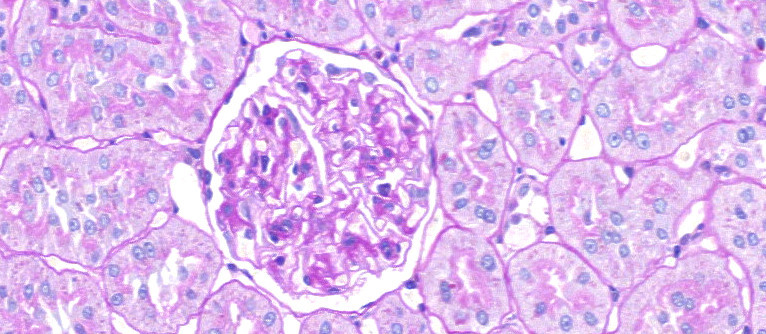

Supplement: Supplementary file 13 [file DataSheet15.ZIP › Fig 1D-PAS-TSF-65/65-10.jpeg]

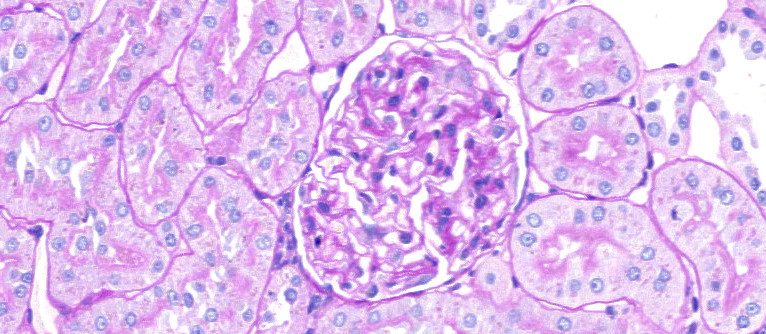

Supplement: Supplementary file 13 [file DataSheet15.ZIP › Fig 1D-PAS-TSF-65/65-11.jpeg]

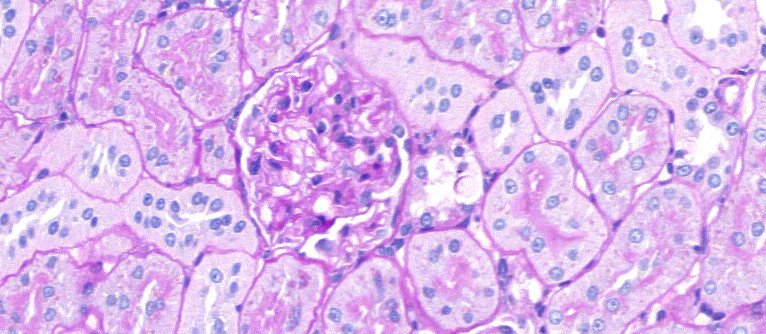

Supplement: Supplementary file 13 [file DataSheet15.ZIP › Fig 1D-PAS-TSF-65/65-12.jpeg]

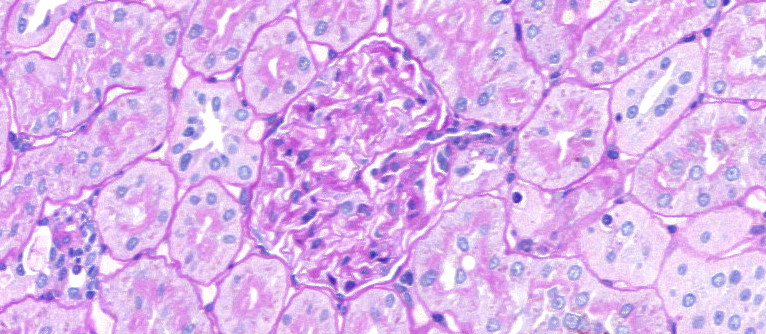

Supplement: Supplementary file 13 [file DataSheet15.ZIP › Fig 1D-PAS-TSF-65/65-13.jpeg]

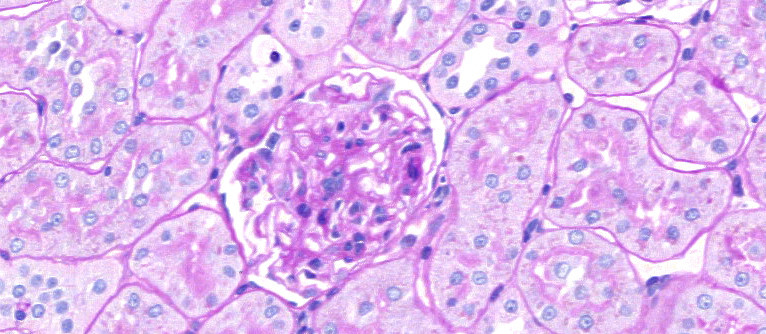

Supplement: Supplementary file 13 [file DataSheet15.ZIP › Fig 1D-PAS-TSF-65/65-14.jpeg]

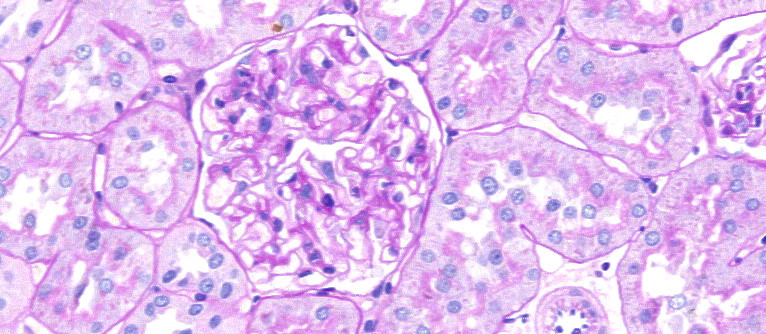

Supplement: Supplementary file 13 [file DataSheet15.ZIP › Fig 1D-PAS-TSF-65/65-15.jpeg]

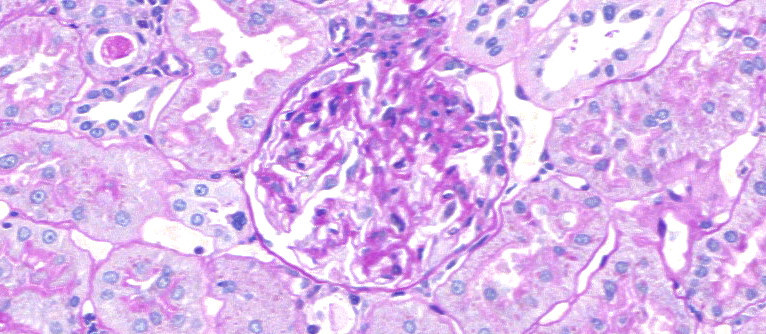

Supplement: Supplementary file 13 [file DataSheet15.ZIP › Fig 1D-PAS-TSF-65/65-16.jpeg]

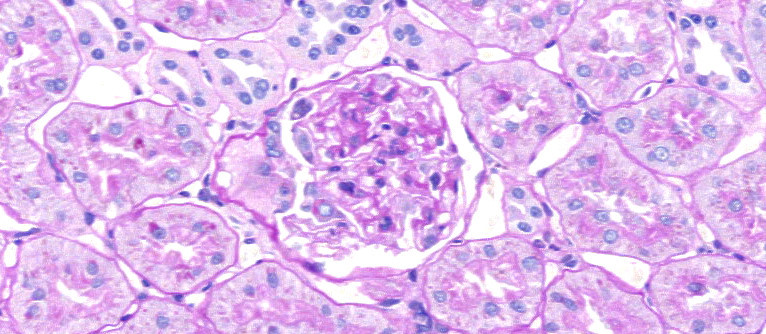

Supplement: Supplementary file 13 [file DataSheet15.ZIP › Fig 1D-PAS-TSF-65/65-17.jpeg]

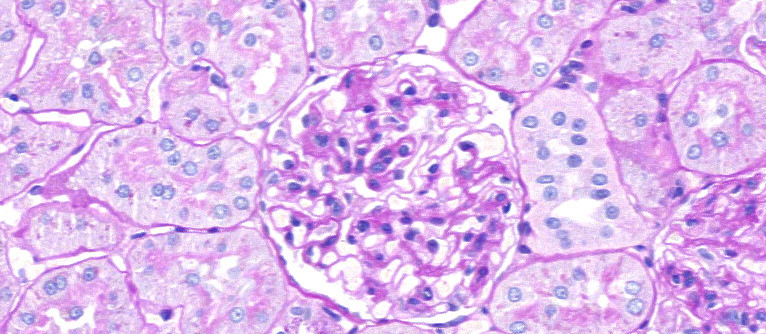

Supplement: Supplementary file 13 [file DataSheet15.ZIP › Fig 1D-PAS-TSF-65/65-18.jpeg]

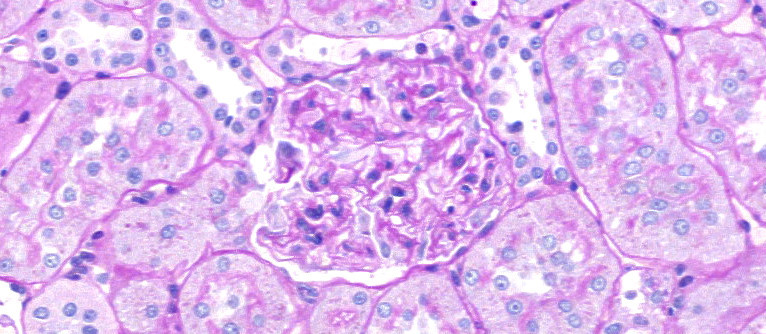

Supplement: Supplementary file 13 [file DataSheet15.ZIP › Fig 1D-PAS-TSF-65/65-19.jpeg]

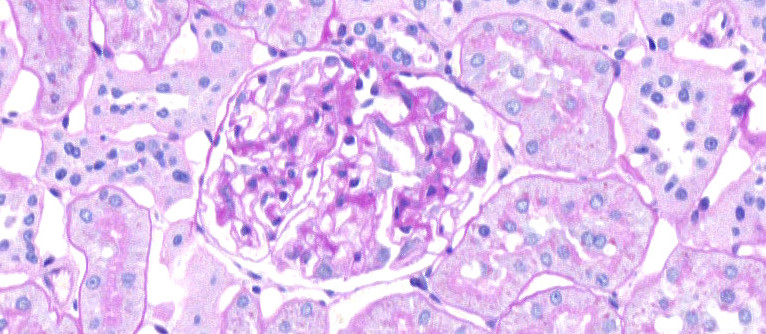

Supplement: Supplementary file 13 [file DataSheet15.ZIP › Fig 1D-PAS-TSF-65/65-2.jpeg]

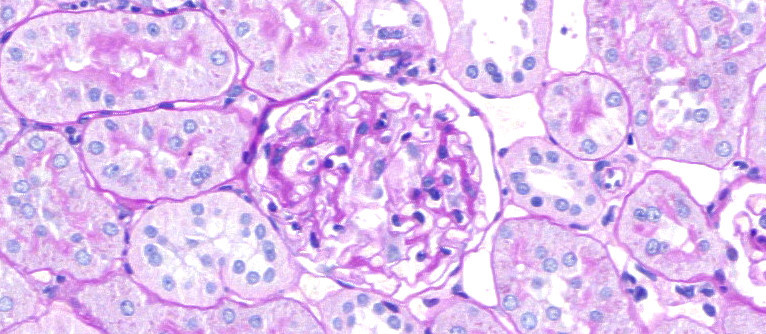

Supplement: Supplementary file 13 [file DataSheet15.ZIP › Fig 1D-PAS-TSF-65/65-20.jpeg]

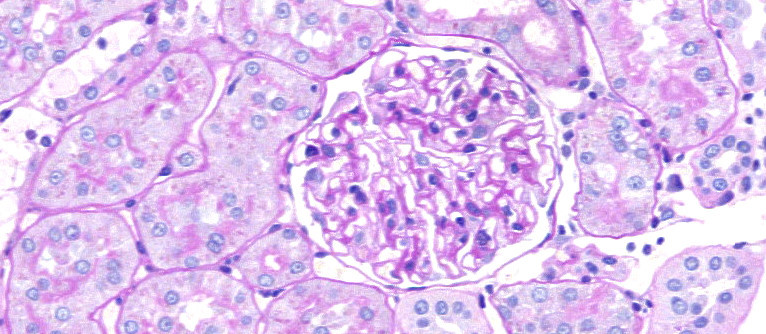

Supplement: Supplementary file 13 [file DataSheet15.ZIP › Fig 1D-PAS-TSF-65/65-3.jpeg]

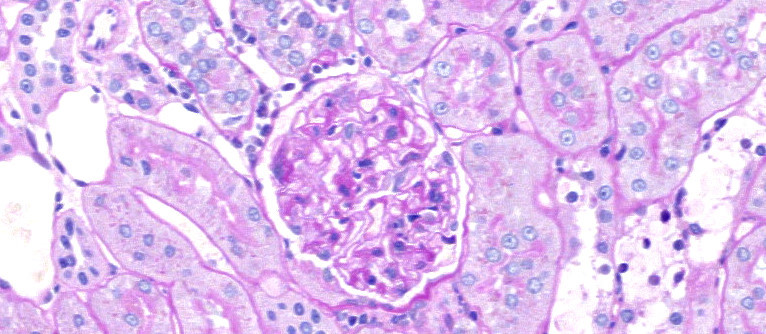

Supplement: Supplementary file 13 [file DataSheet15.ZIP › Fig 1D-PAS-TSF-65/65-4.jpeg]

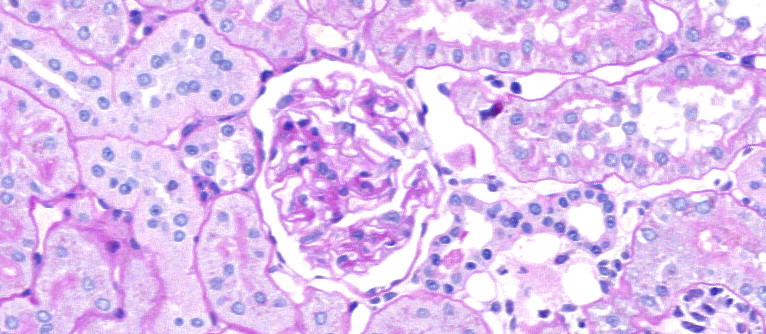

Supplement: Supplementary file 13 [file DataSheet15.ZIP › Fig 1D-PAS-TSF-65/65-5.jpeg]

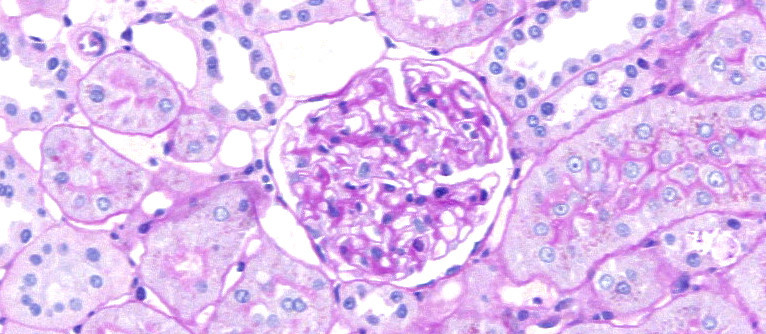

Supplement: Supplementary file 13 [file DataSheet15.ZIP › Fig 1D-PAS-TSF-65/65-6.jpeg]

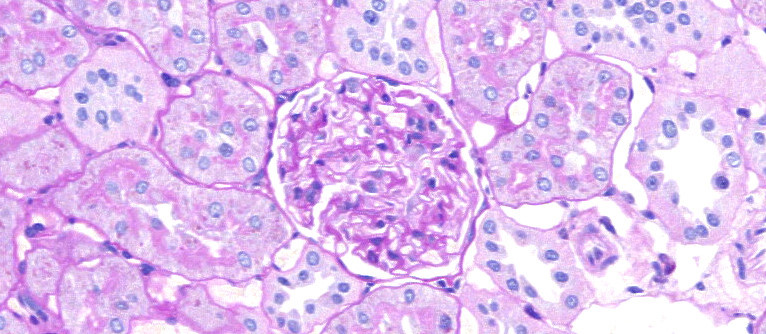

Supplement: Supplementary file 13 [file DataSheet15.ZIP › Fig 1D-PAS-TSF-65/65-7.jpeg]

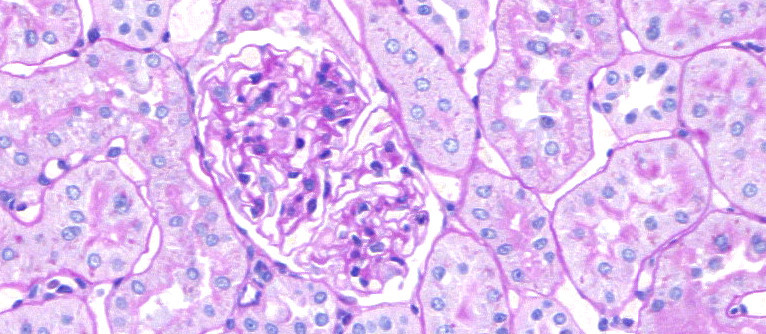

Supplement: Supplementary file 13 [file DataSheet15.ZIP › Fig 1D-PAS-TSF-65/65-8.jpeg]

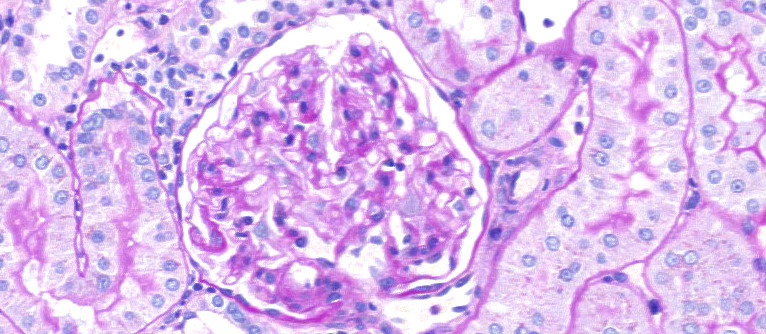

Supplement: Supplementary file 13 [file DataSheet15.ZIP › Fig 1D-PAS-TSF-65/65-9.jpeg]

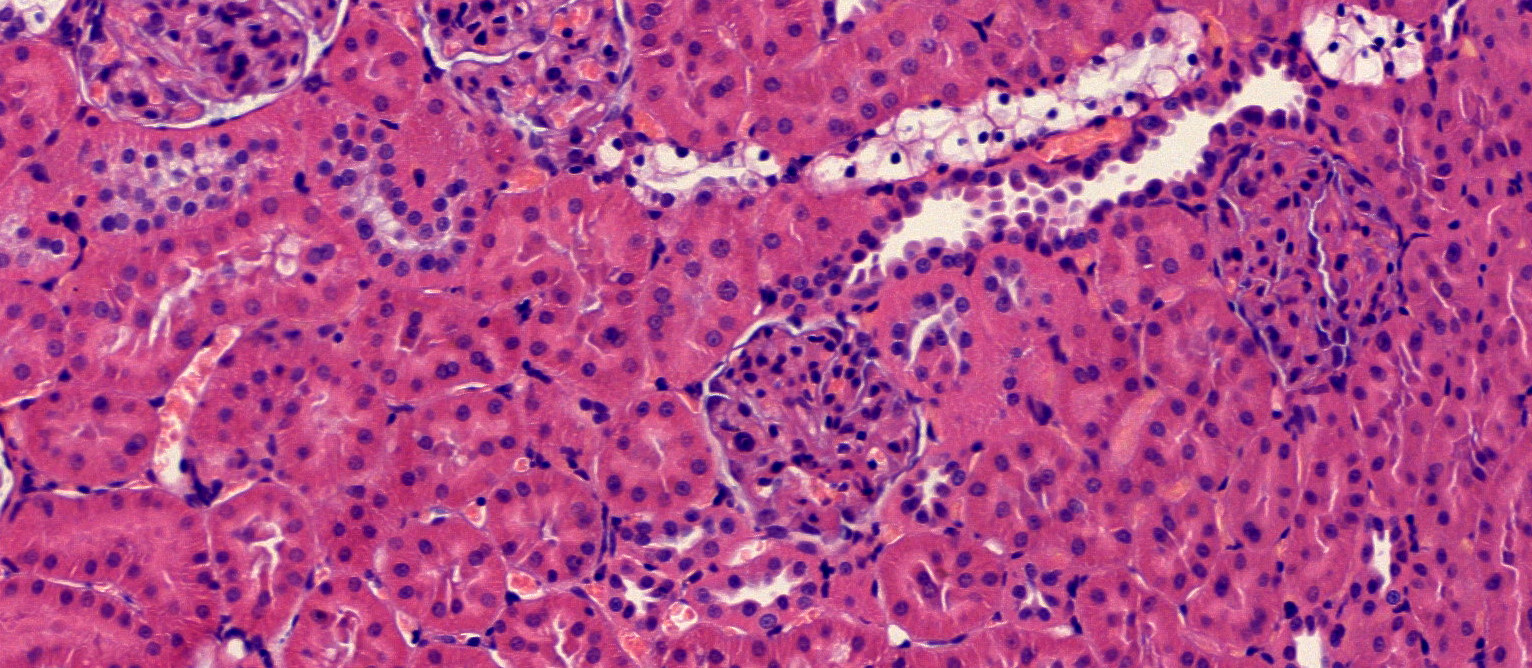

Supplement: Supplementary file 14 [file DataSheet5.ZIP › Fig 1D-HE-TSF-58/58-1.jpeg]

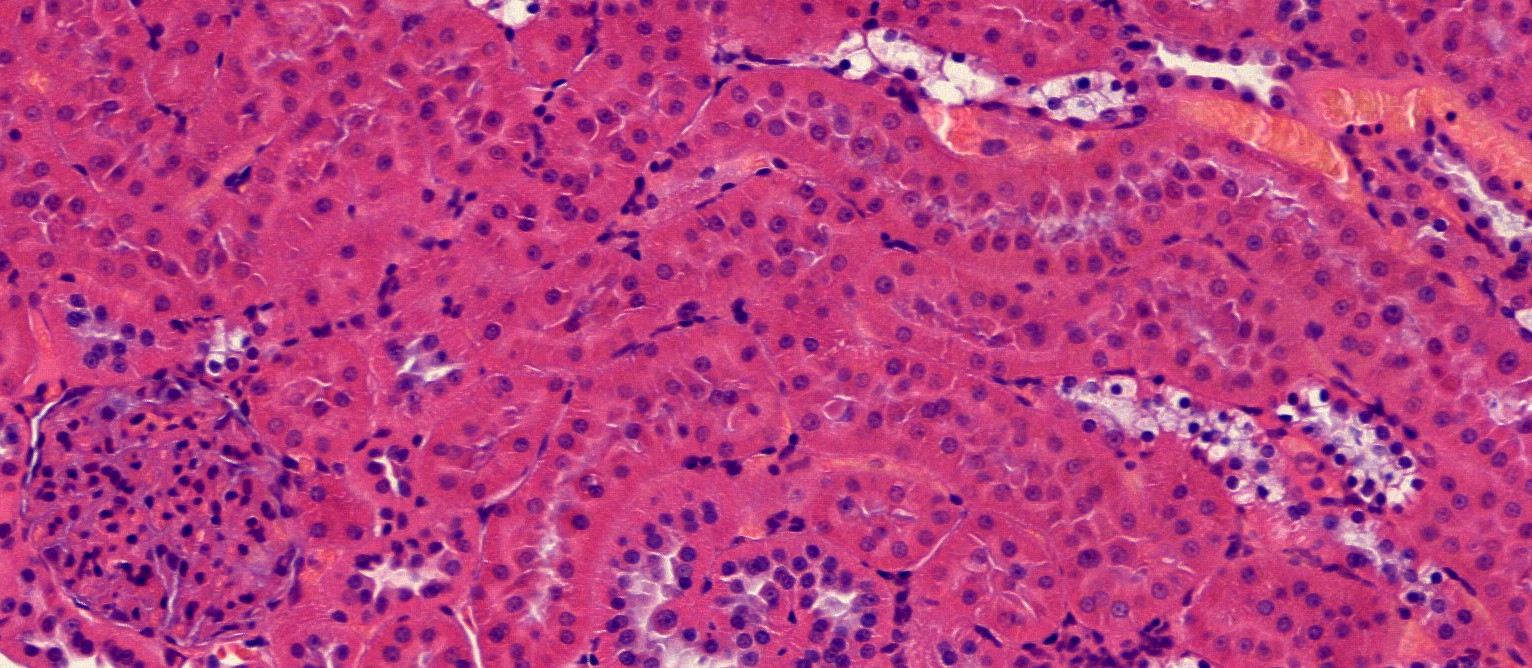

Supplement: Supplementary file 14 [file DataSheet5.ZIP › Fig 1D-HE-TSF-58/58-10.jpeg]
