# Supplementary figures and images for: Characteristics of Serum Metabolites and Gut Microbiota in Diabetic Kidney Disease (part 12 of 13)
Source: Front Pharmacol. 2022 Apr 14;13:872988. doi: 10.3389/fphar.2022.872988 (PMC9084235; doi:10.3389/fphar.2022.872988)

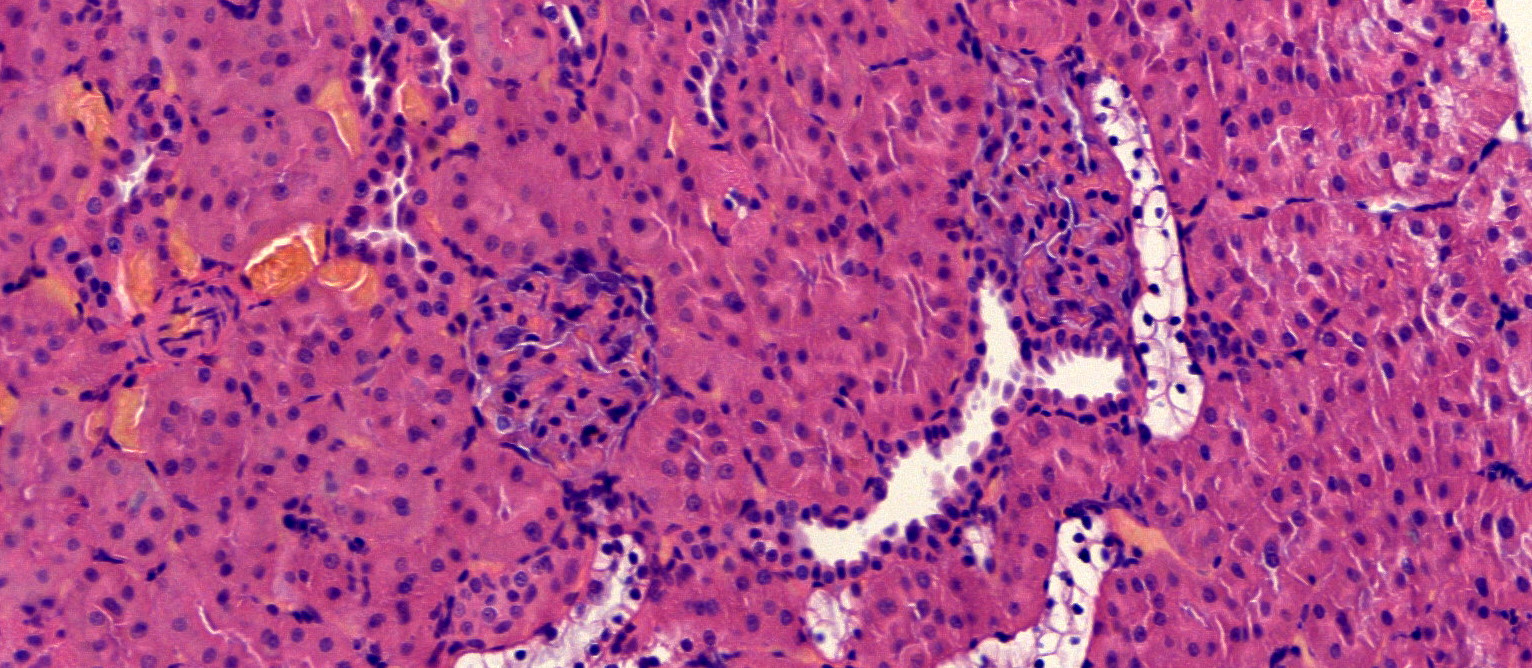

Supplement: Supplementary file 14 [file DataSheet5.ZIP › Fig 1D-HE-TSF-58/58-2.jpeg]

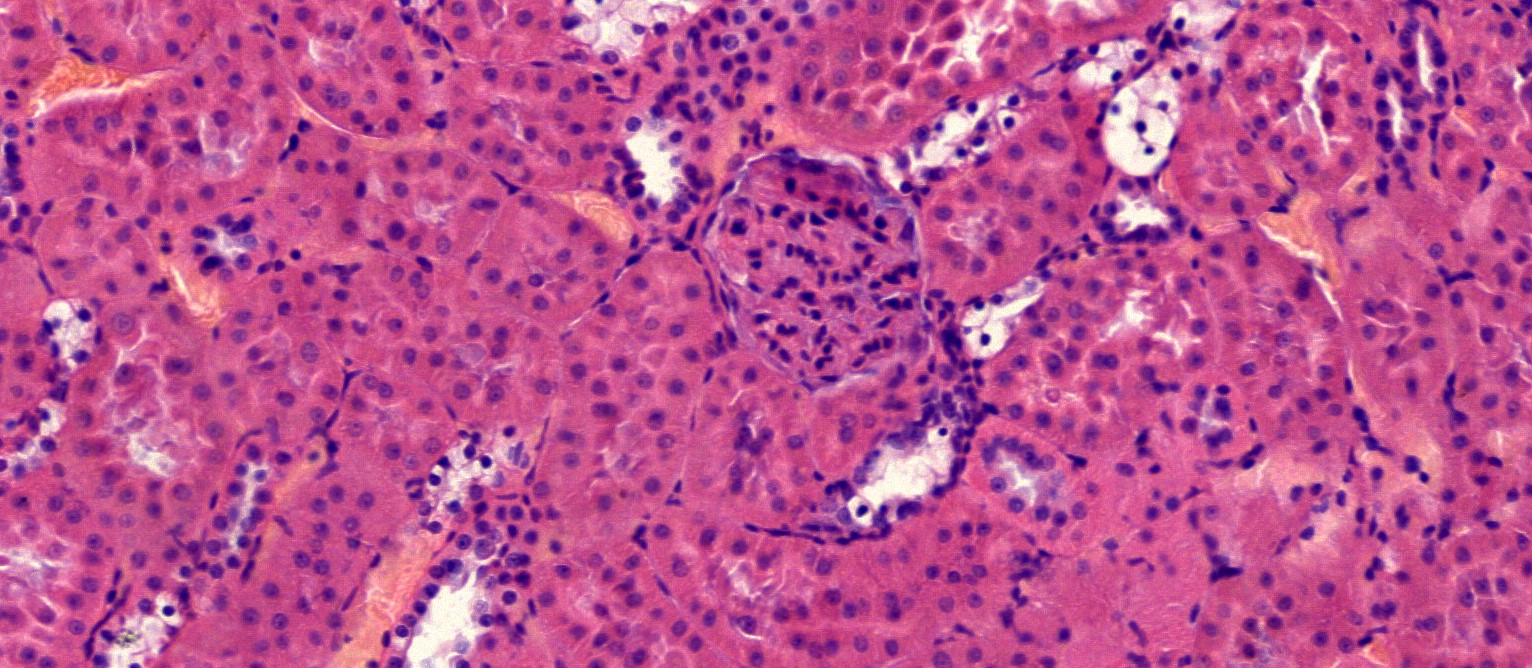

Supplement: Supplementary file 14 [file DataSheet5.ZIP › Fig 1D-HE-TSF-58/58-3.jpeg]

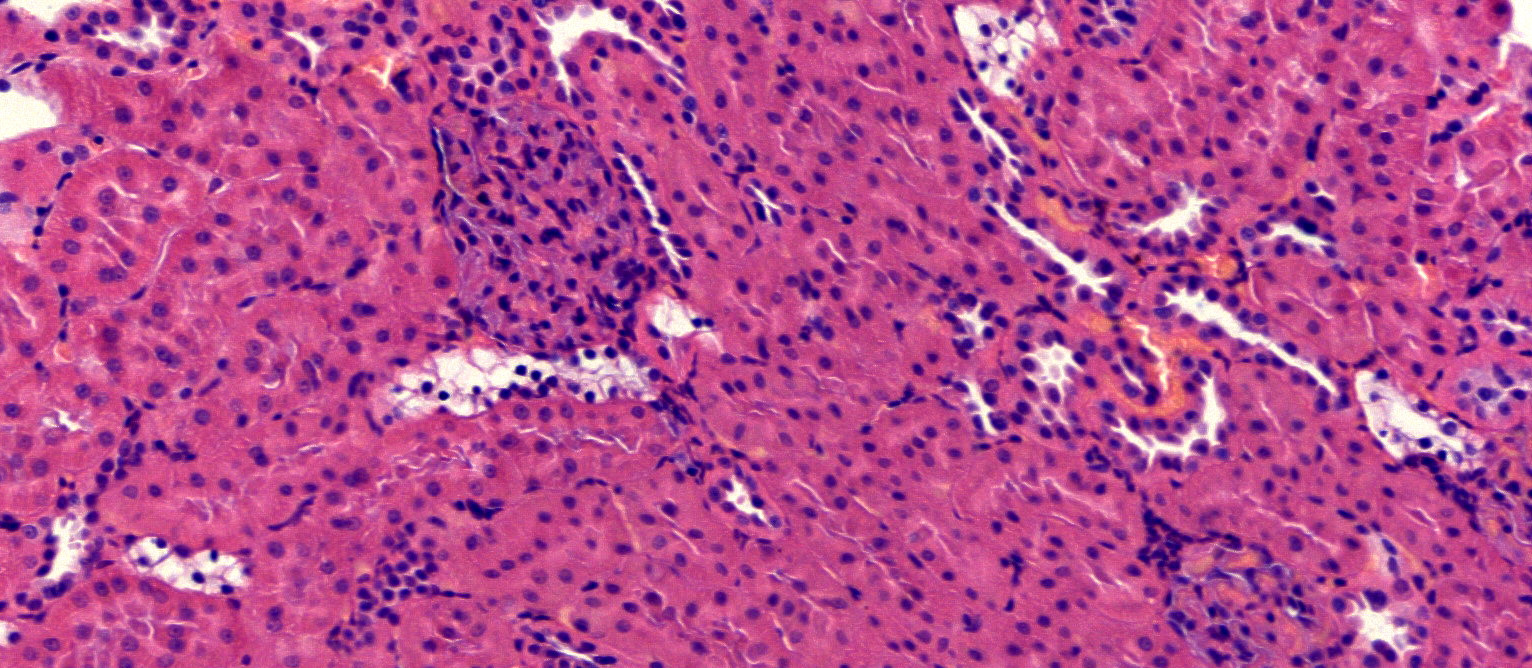

Supplement: Supplementary file 14 [file DataSheet5.ZIP › Fig 1D-HE-TSF-58/58-4.jpeg]

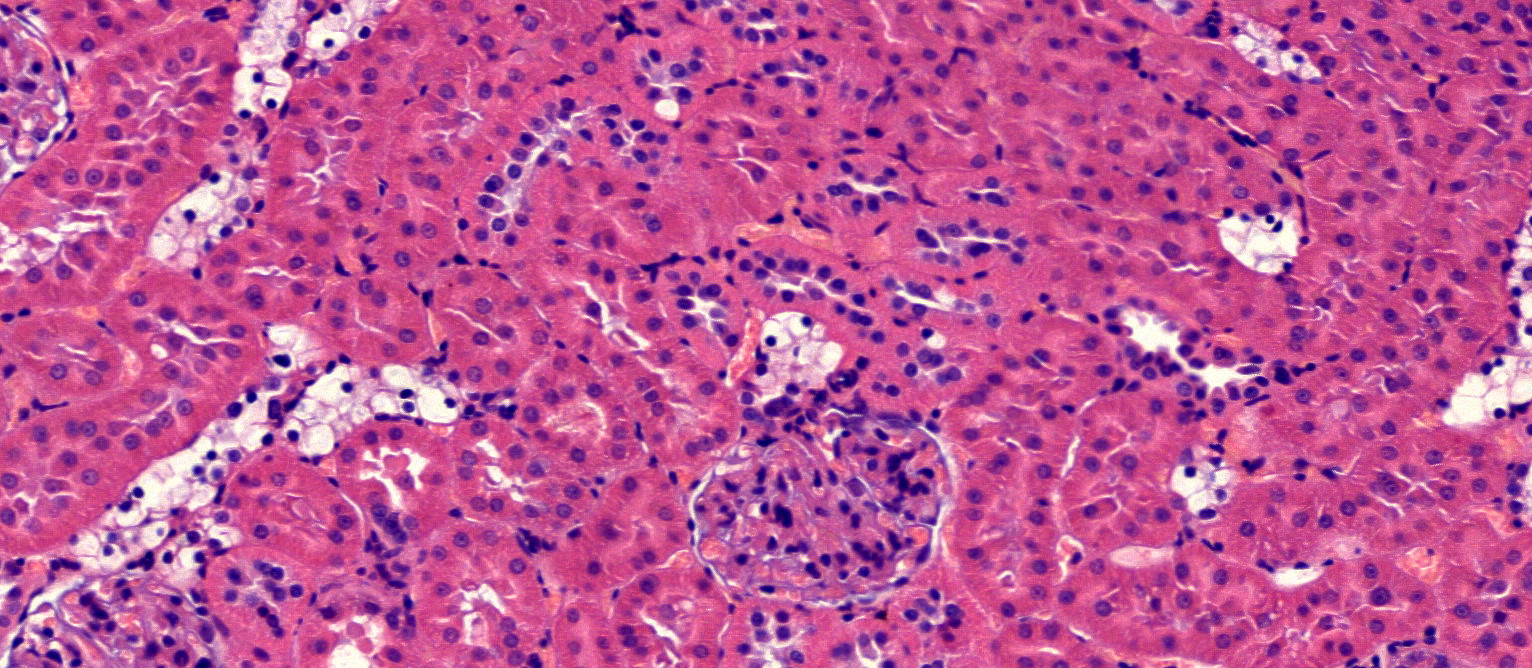

Supplement: Supplementary file 14 [file DataSheet5.ZIP › Fig 1D-HE-TSF-58/58-5.jpeg]

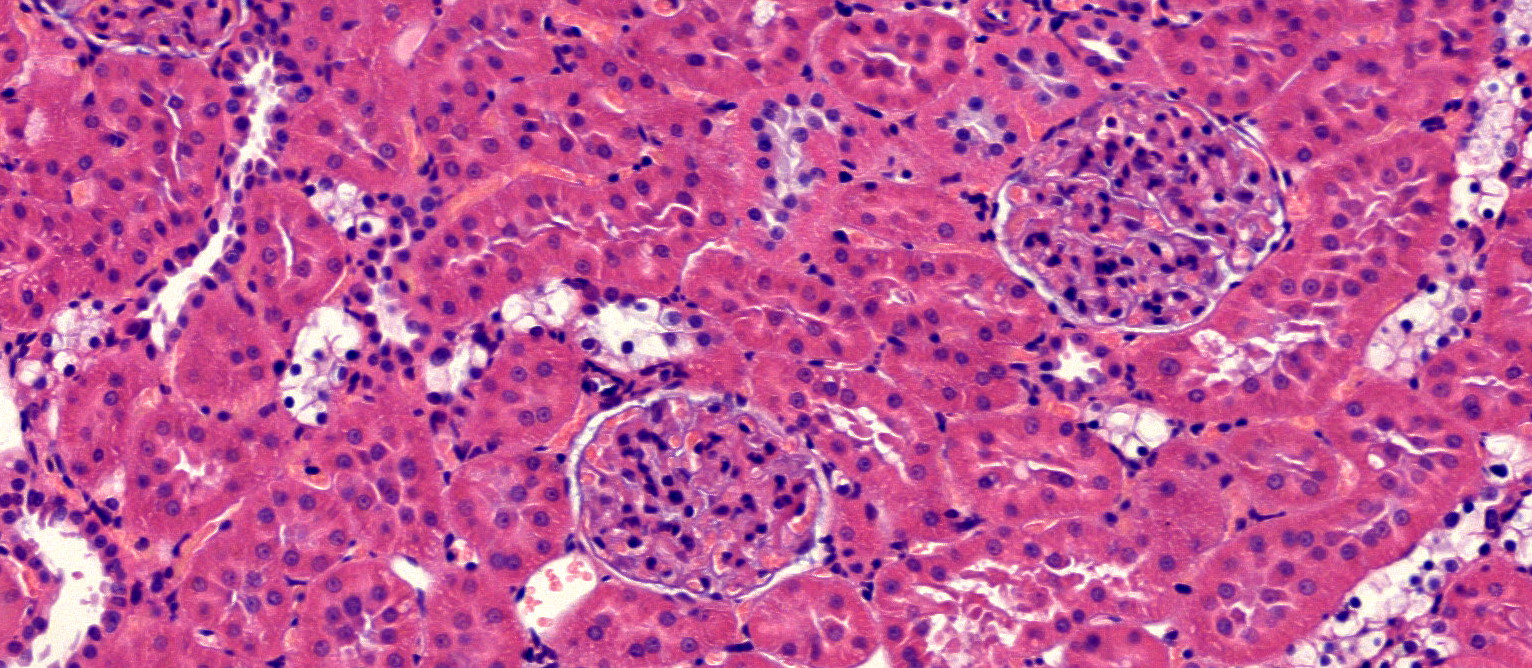

Supplement: Supplementary file 14 [file DataSheet5.ZIP › Fig 1D-HE-TSF-58/58-6.jpeg]

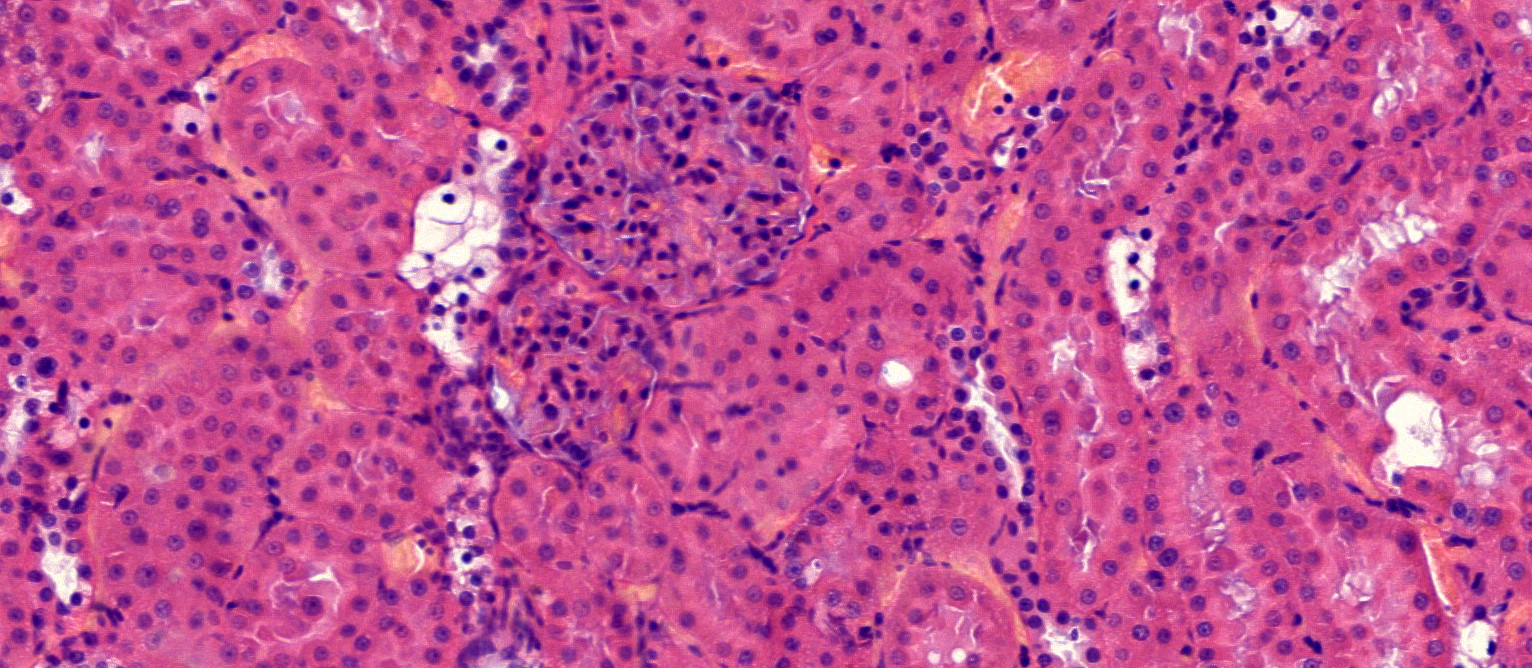

Supplement: Supplementary file 14 [file DataSheet5.ZIP › Fig 1D-HE-TSF-58/58-7.jpeg]

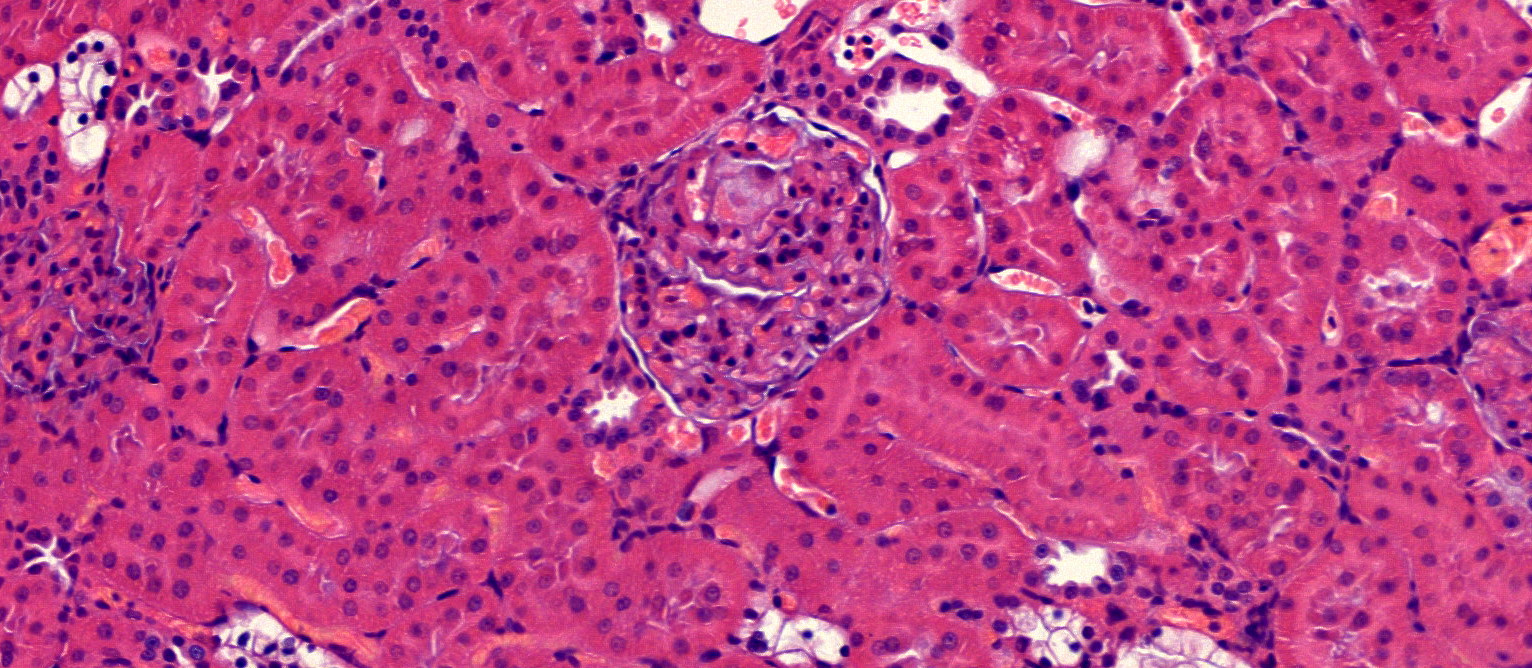

Supplement: Supplementary file 14 [file DataSheet5.ZIP › Fig 1D-HE-TSF-58/58-8.jpeg]

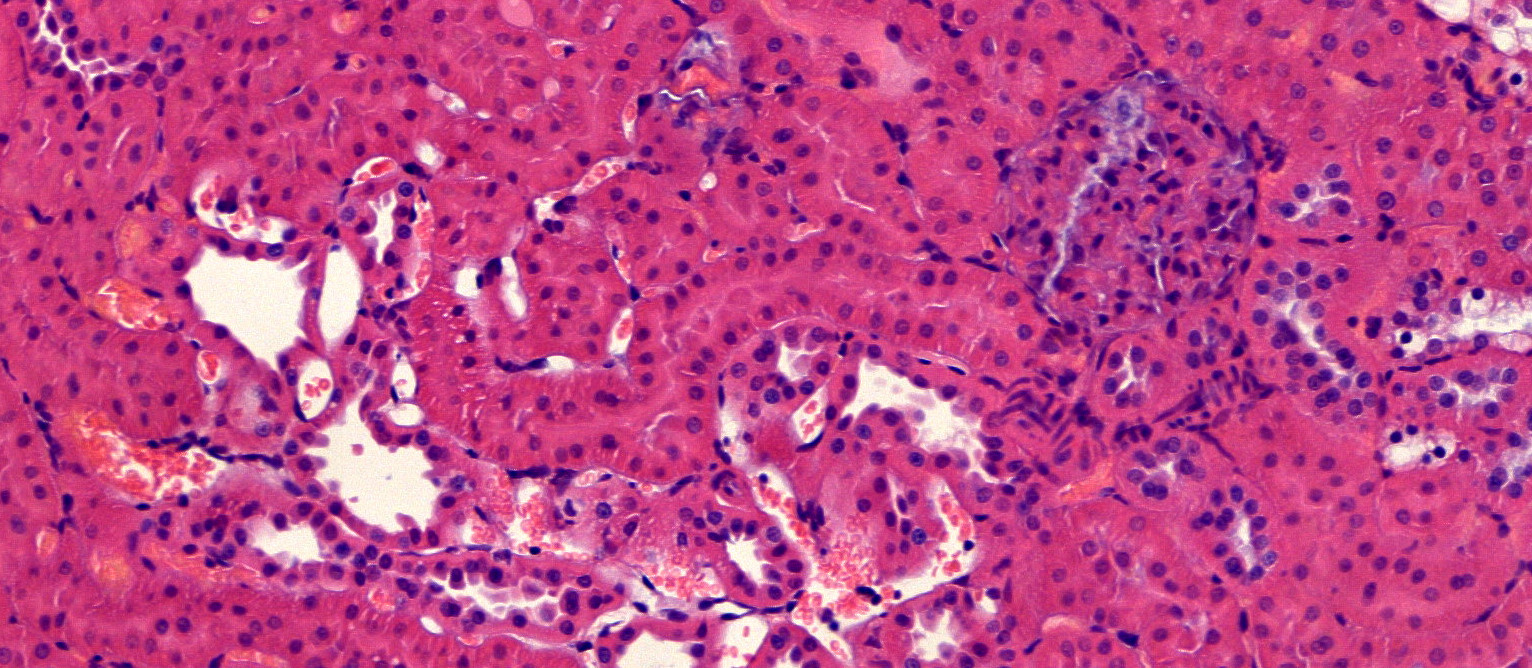

Supplement: Supplementary file 14 [file DataSheet5.ZIP › Fig 1D-HE-TSF-58/58-9.jpeg]

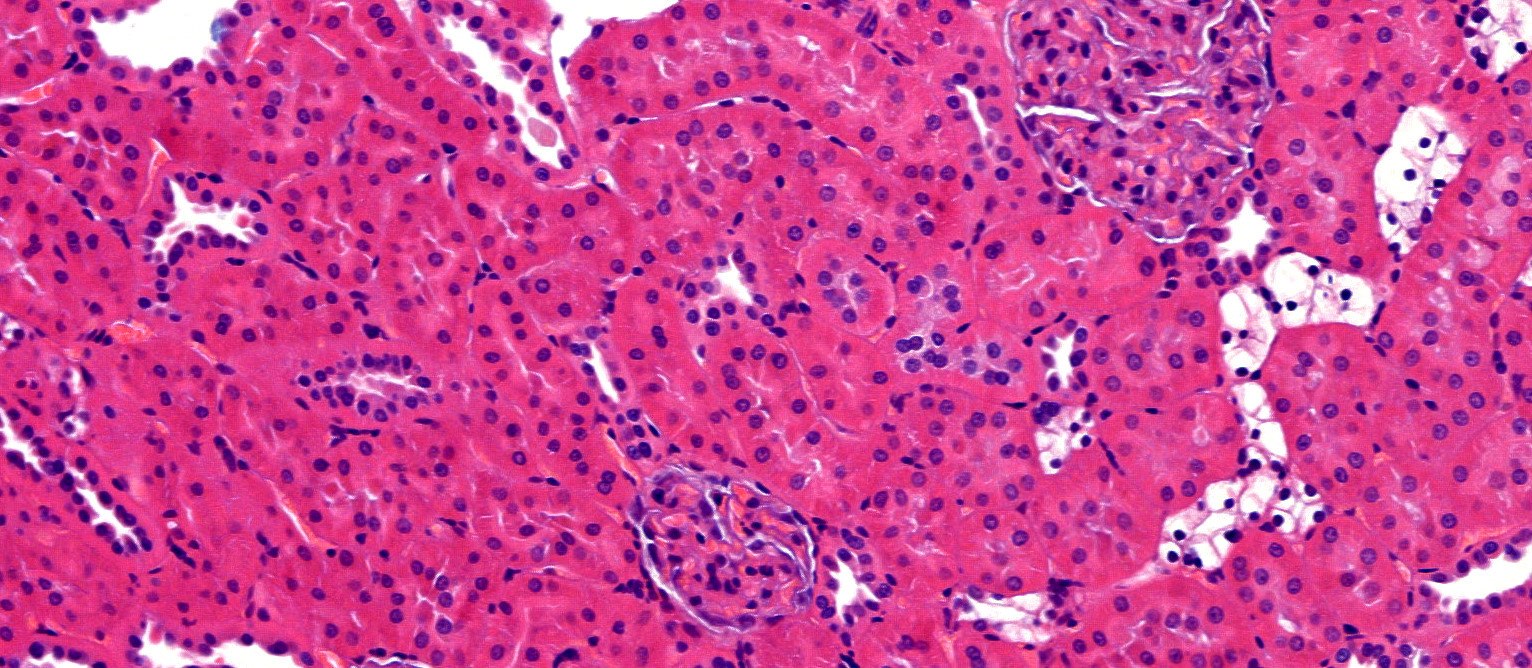

Supplement: Supplementary file 14 [file DataSheet5.ZIP › Fig 1D-HE-TSF-59/59-1.jpeg]

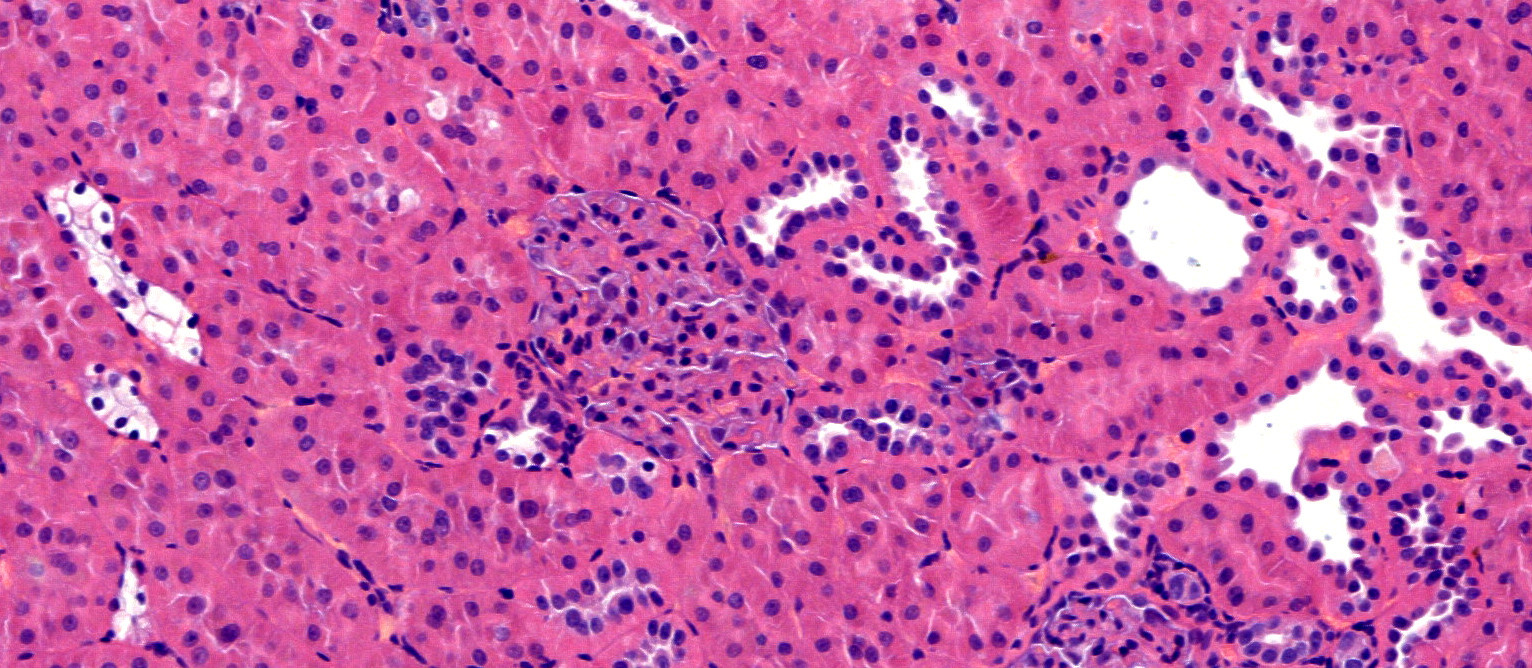

Supplement: Supplementary file 14 [file DataSheet5.ZIP › Fig 1D-HE-TSF-59/59-10.jpeg]

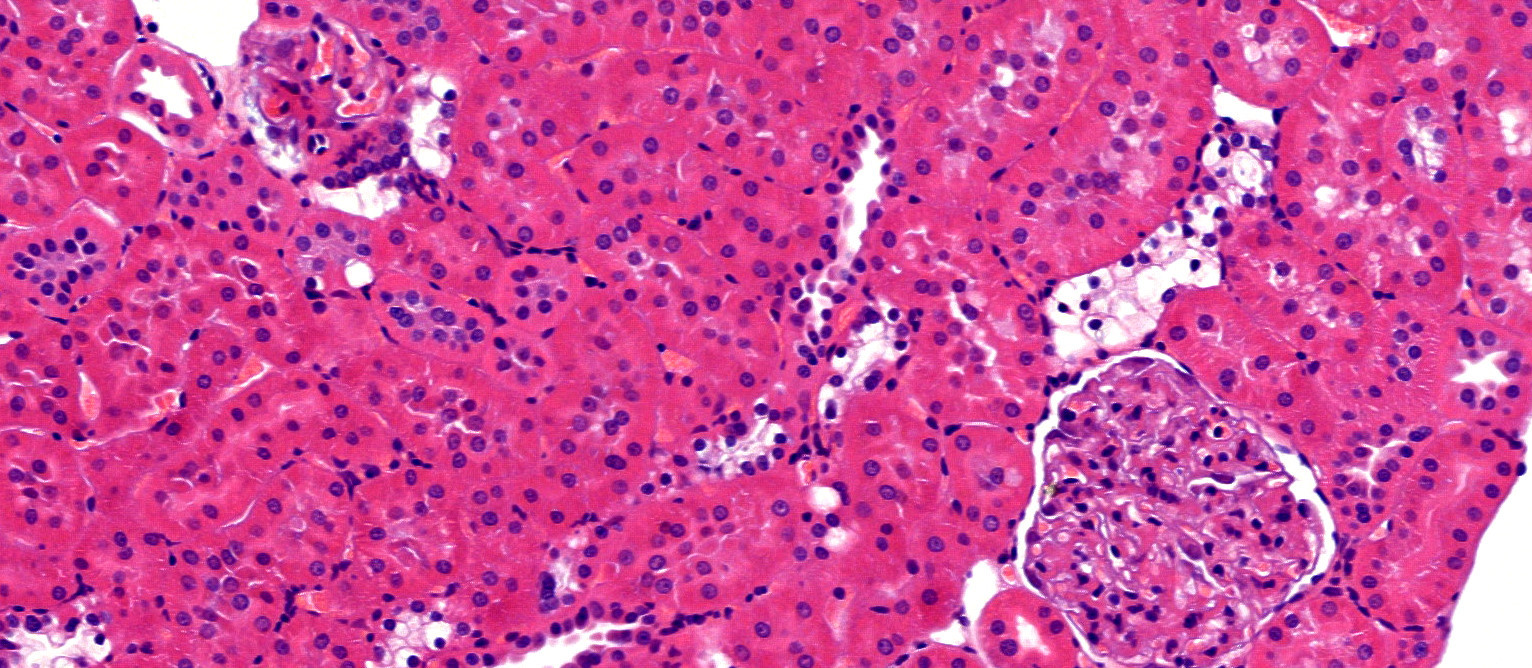

Supplement: Supplementary file 14 [file DataSheet5.ZIP › Fig 1D-HE-TSF-59/59-2.jpeg]

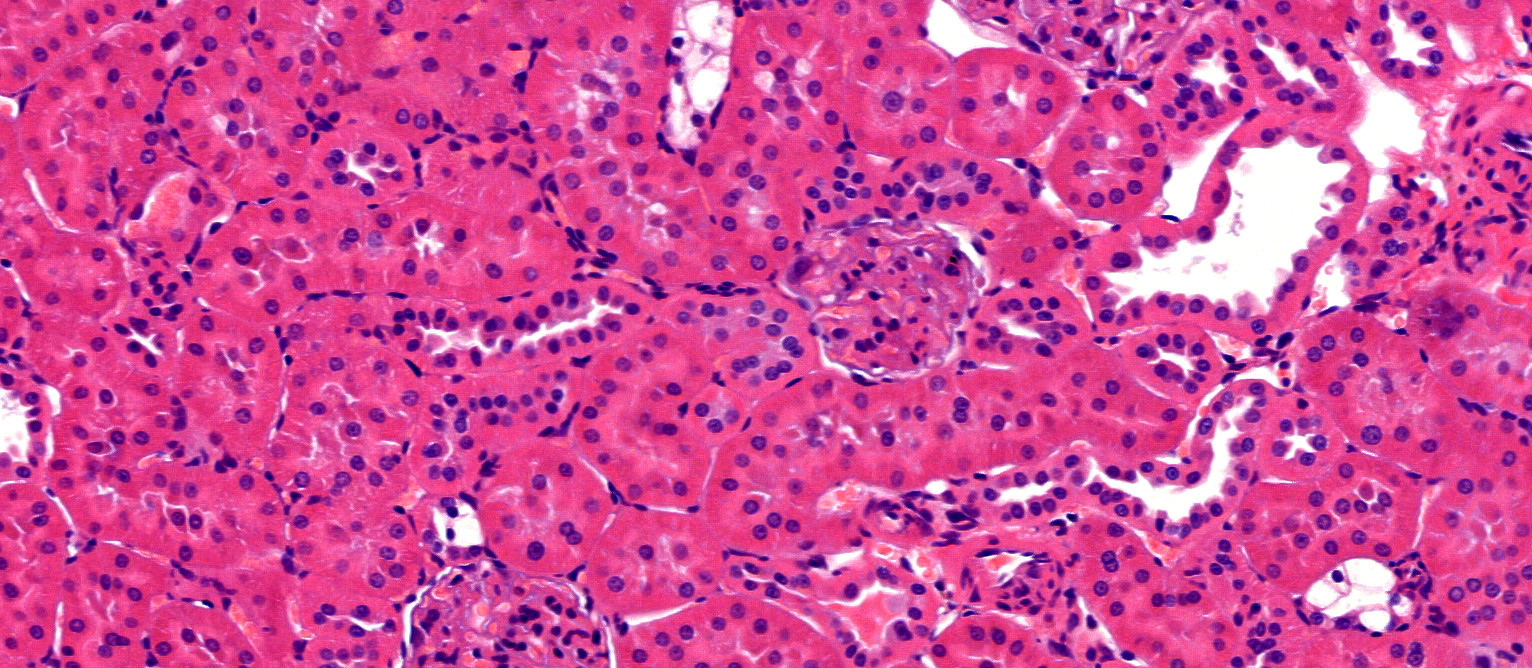

Supplement: Supplementary file 14 [file DataSheet5.ZIP › Fig 1D-HE-TSF-59/59-3.jpeg]

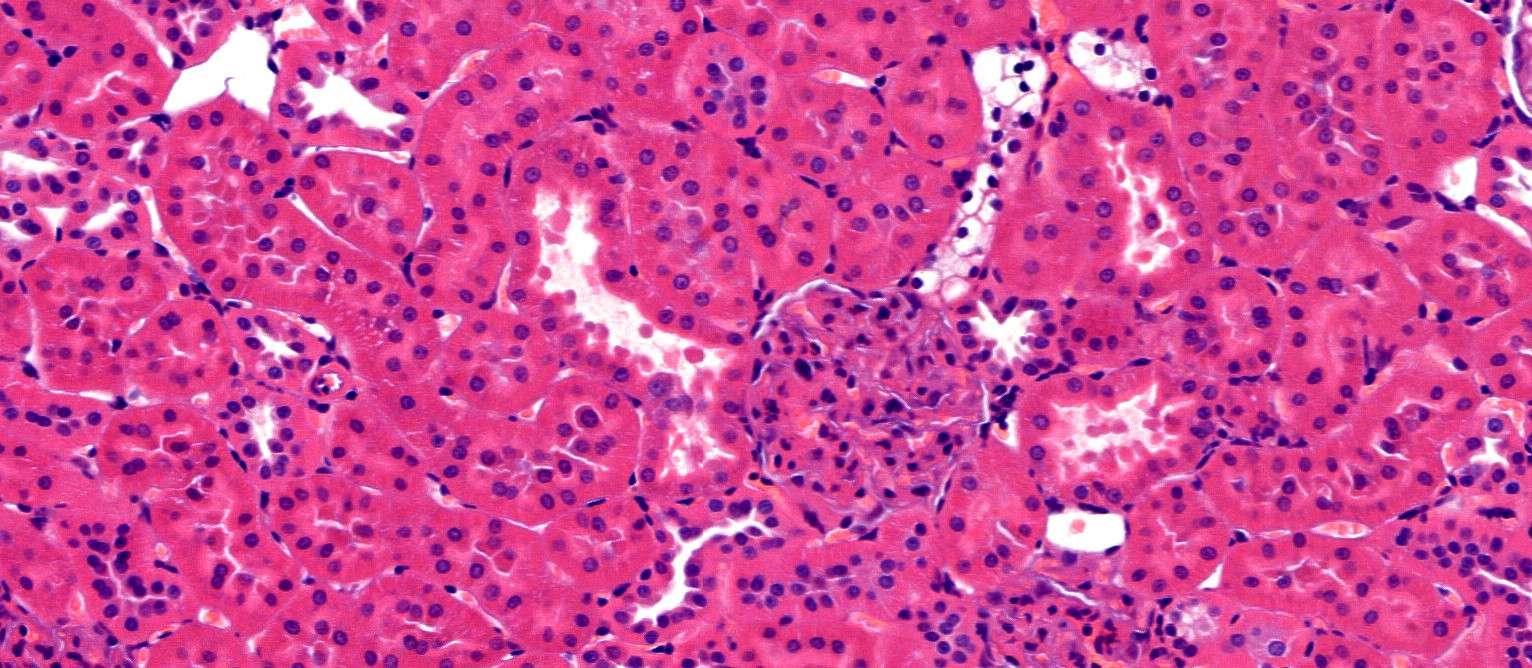

Supplement: Supplementary file 14 [file DataSheet5.ZIP › Fig 1D-HE-TSF-59/59-4.jpeg]

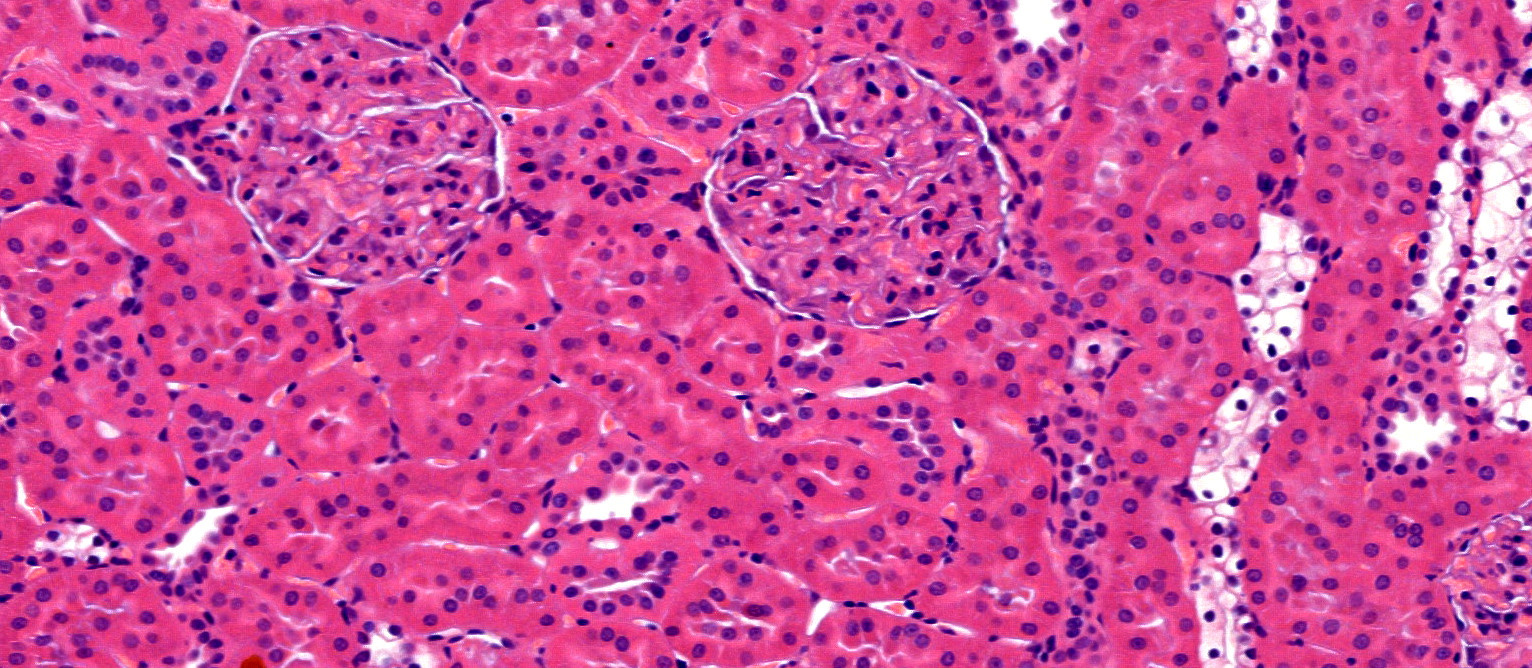

Supplement: Supplementary file 14 [file DataSheet5.ZIP › Fig 1D-HE-TSF-59/59-5.jpeg]

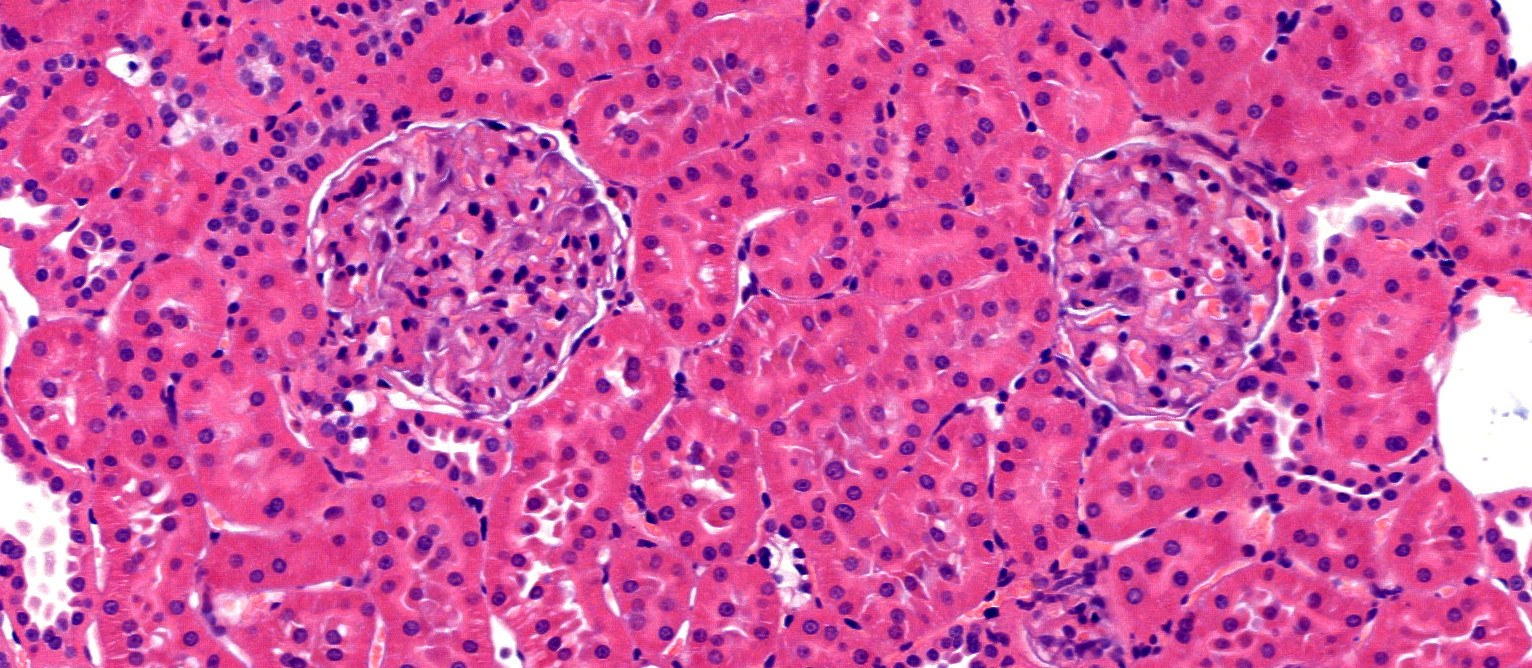

Supplement: Supplementary file 14 [file DataSheet5.ZIP › Fig 1D-HE-TSF-59/59-6.jpeg]

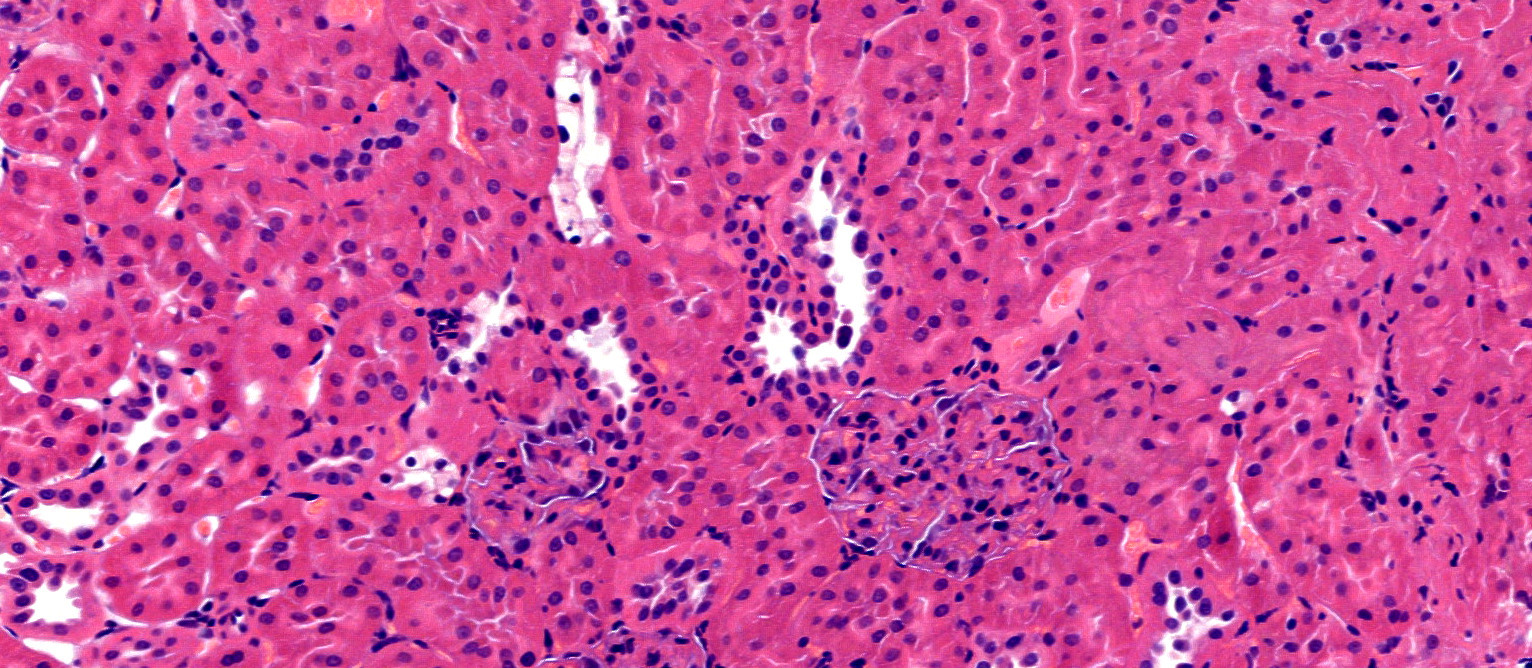

Supplement: Supplementary file 14 [file DataSheet5.ZIP › Fig 1D-HE-TSF-59/59-7.jpeg]

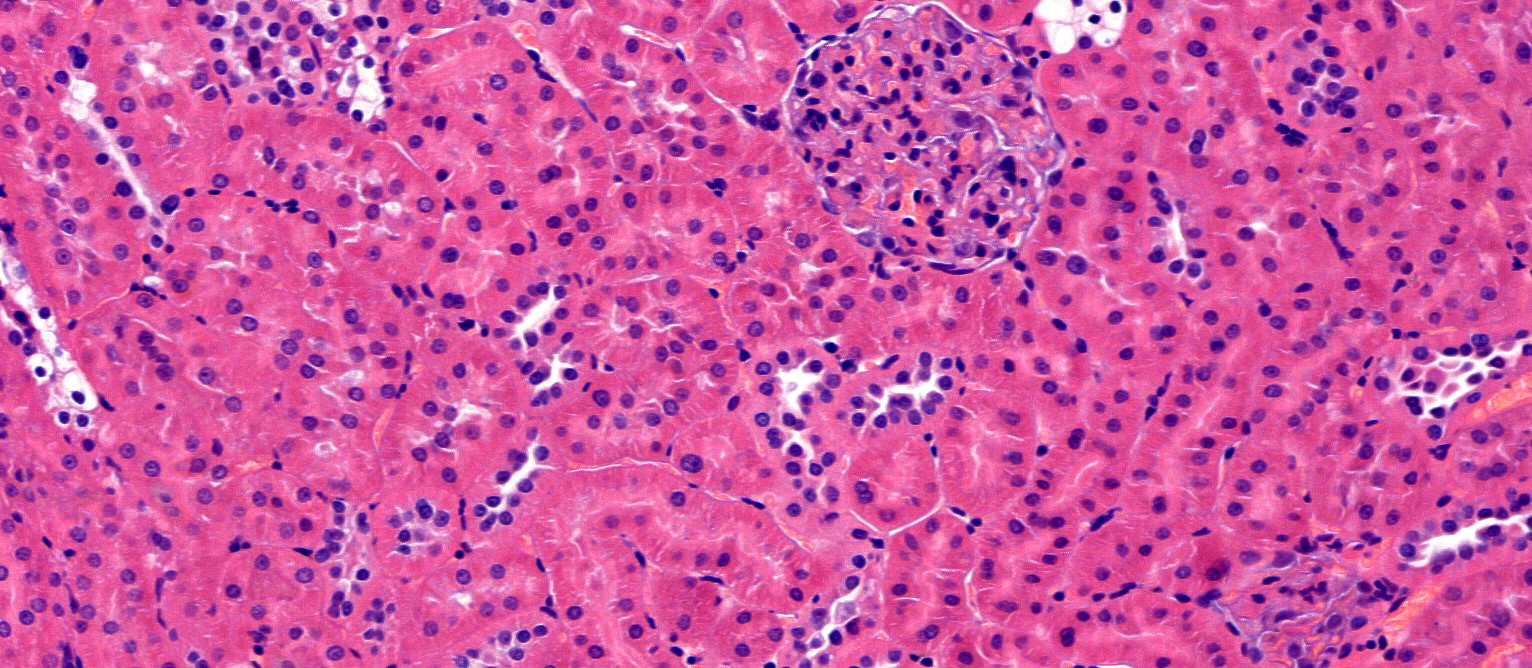

Supplement: Supplementary file 14 [file DataSheet5.ZIP › Fig 1D-HE-TSF-59/59-8.jpeg]

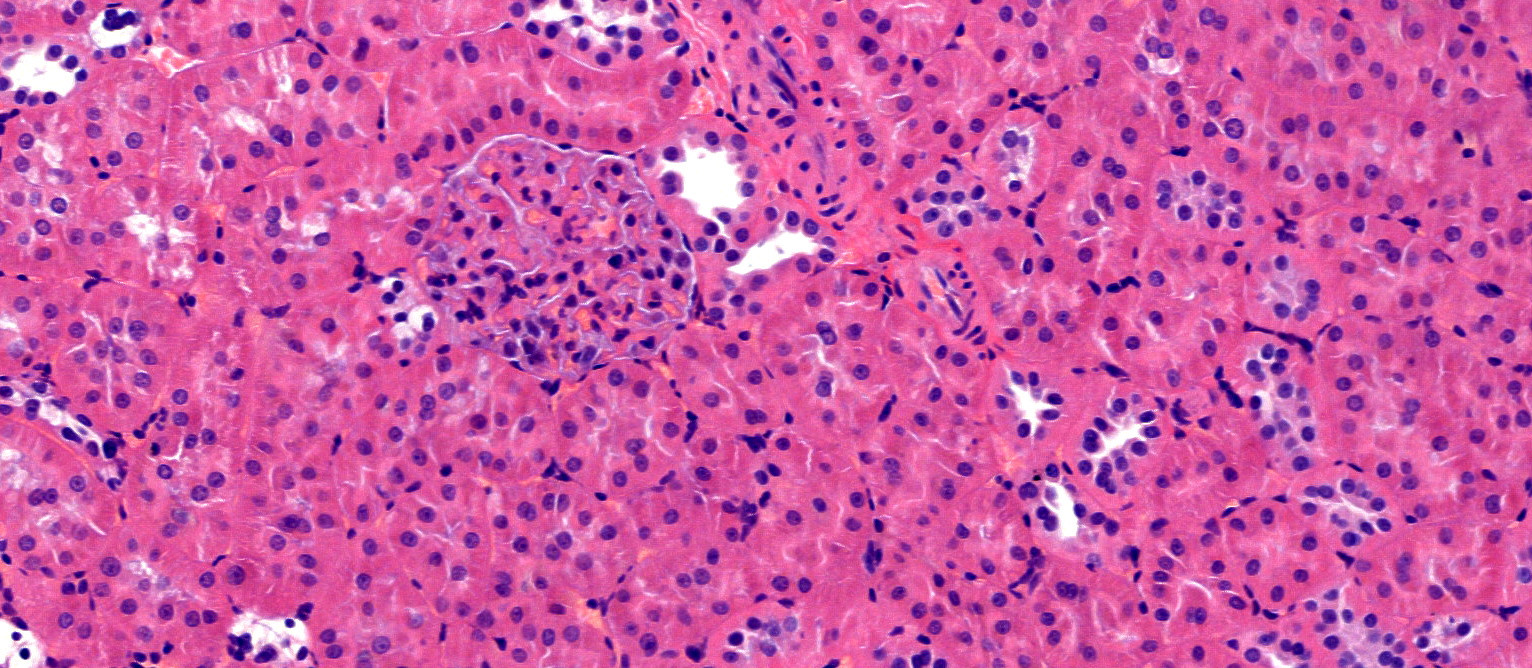

Supplement: Supplementary file 14 [file DataSheet5.ZIP › Fig 1D-HE-TSF-59/59-9.jpeg]

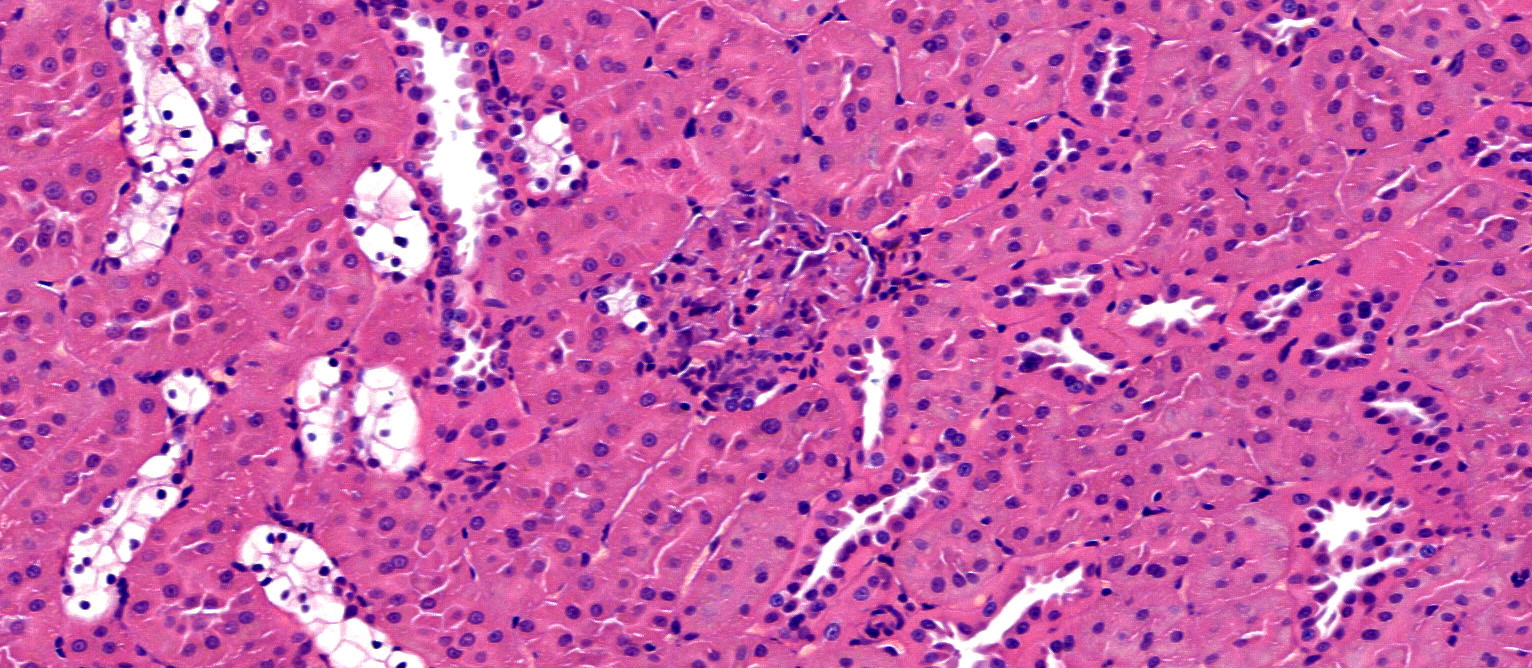

Supplement: Supplementary file 14 [file DataSheet5.ZIP › Fig 1D-HE-TSF-60/60-1.jpeg]

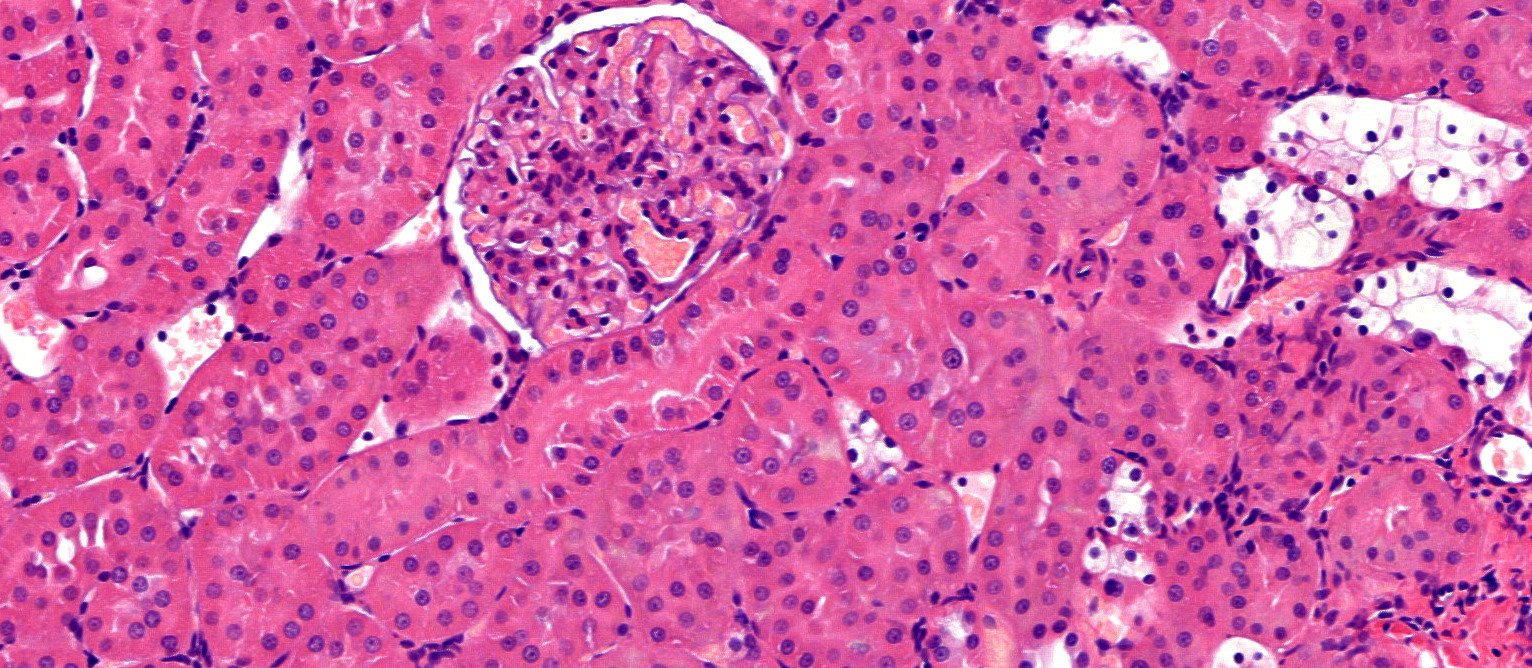

Supplement: Supplementary file 14 [file DataSheet5.ZIP › Fig 1D-HE-TSF-60/60-10.jpeg]

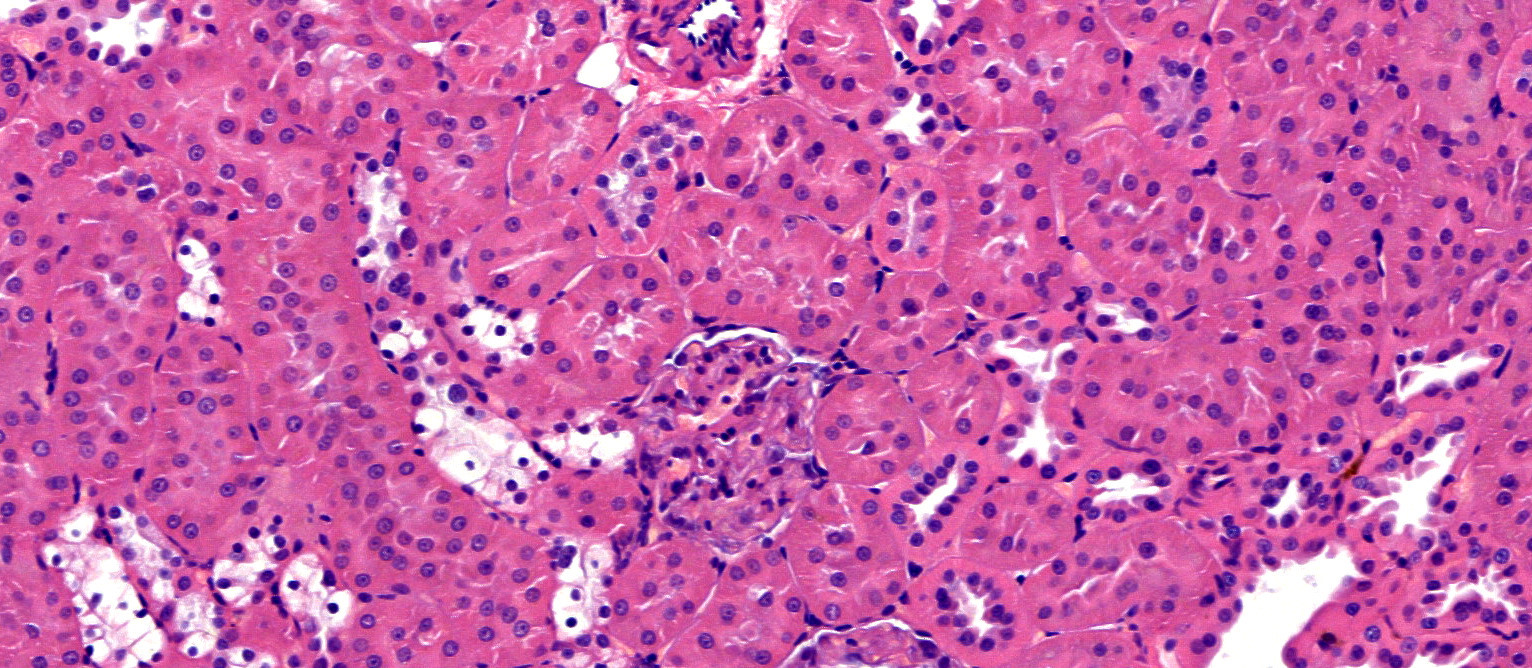

Supplement: Supplementary file 14 [file DataSheet5.ZIP › Fig 1D-HE-TSF-60/60-2.jpeg]

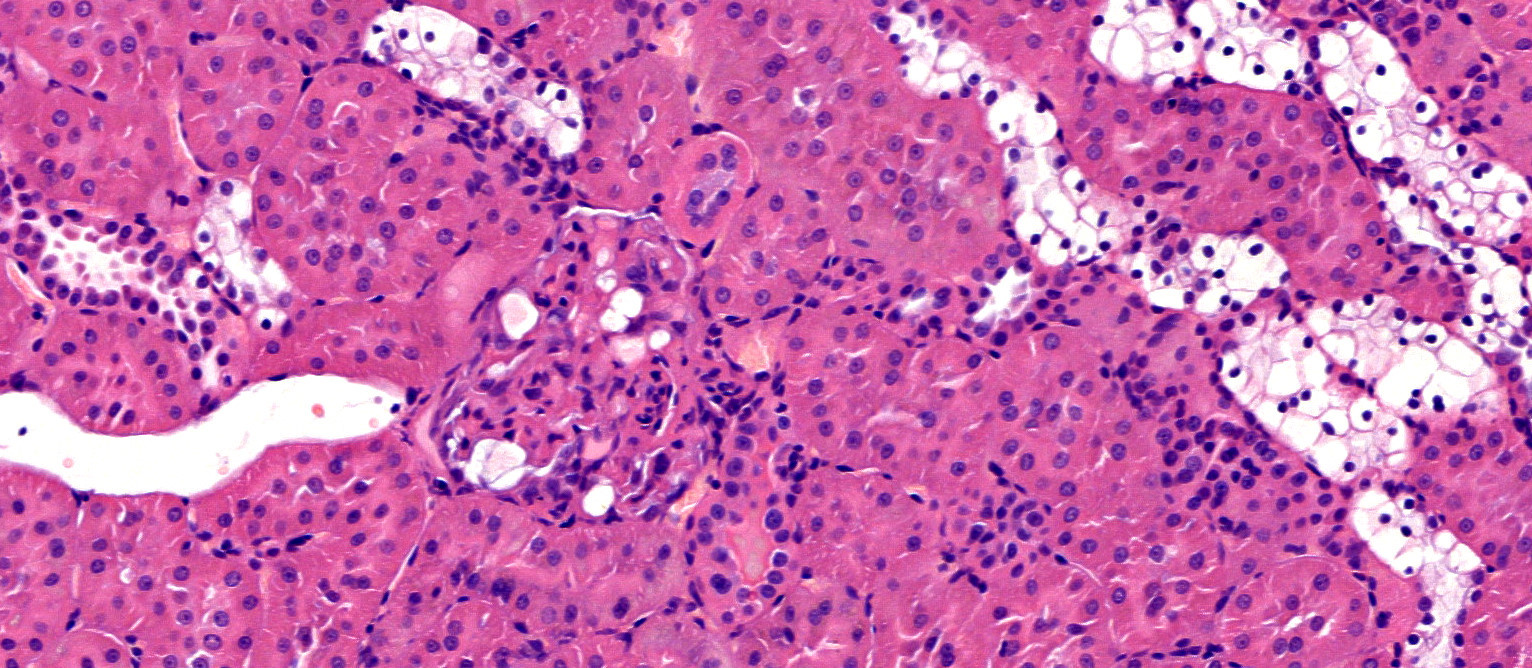

Supplement: Supplementary file 14 [file DataSheet5.ZIP › Fig 1D-HE-TSF-60/60-3.jpeg]

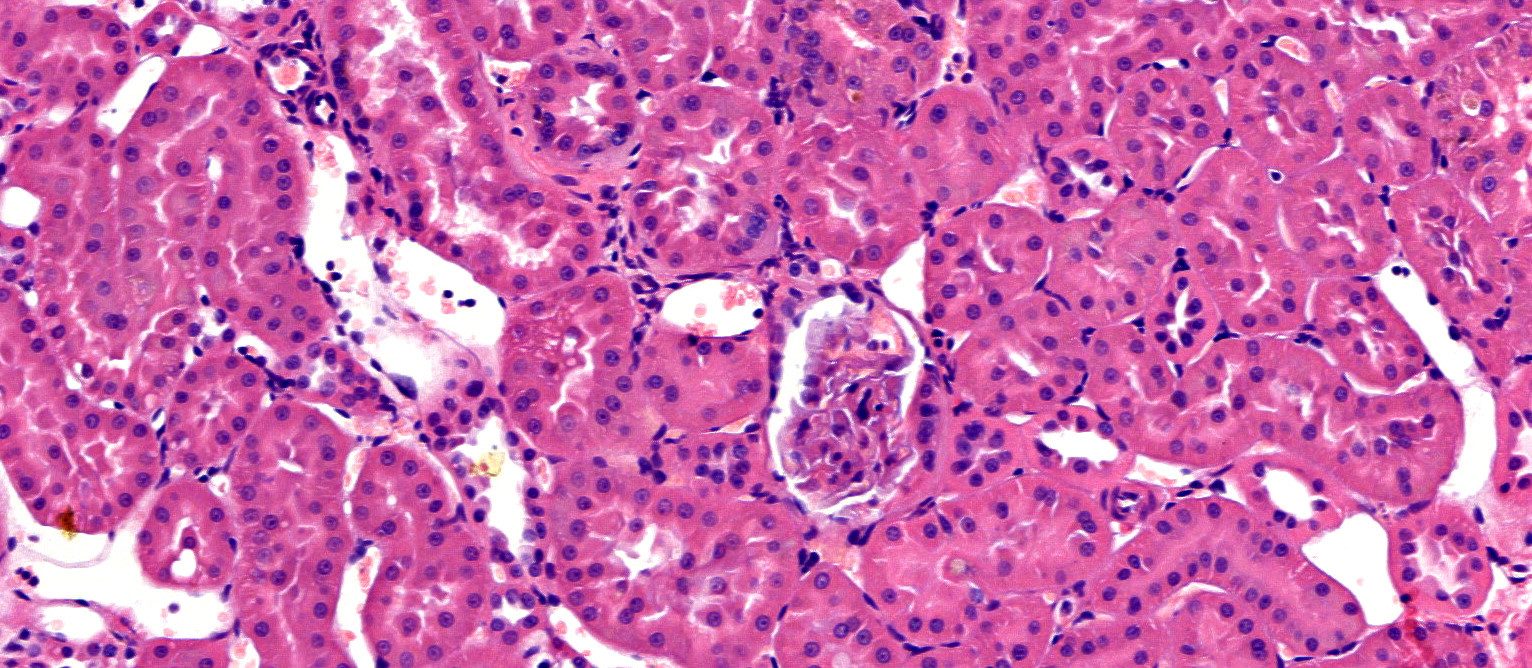

Supplement: Supplementary file 14 [file DataSheet5.ZIP › Fig 1D-HE-TSF-60/60-4.jpeg]

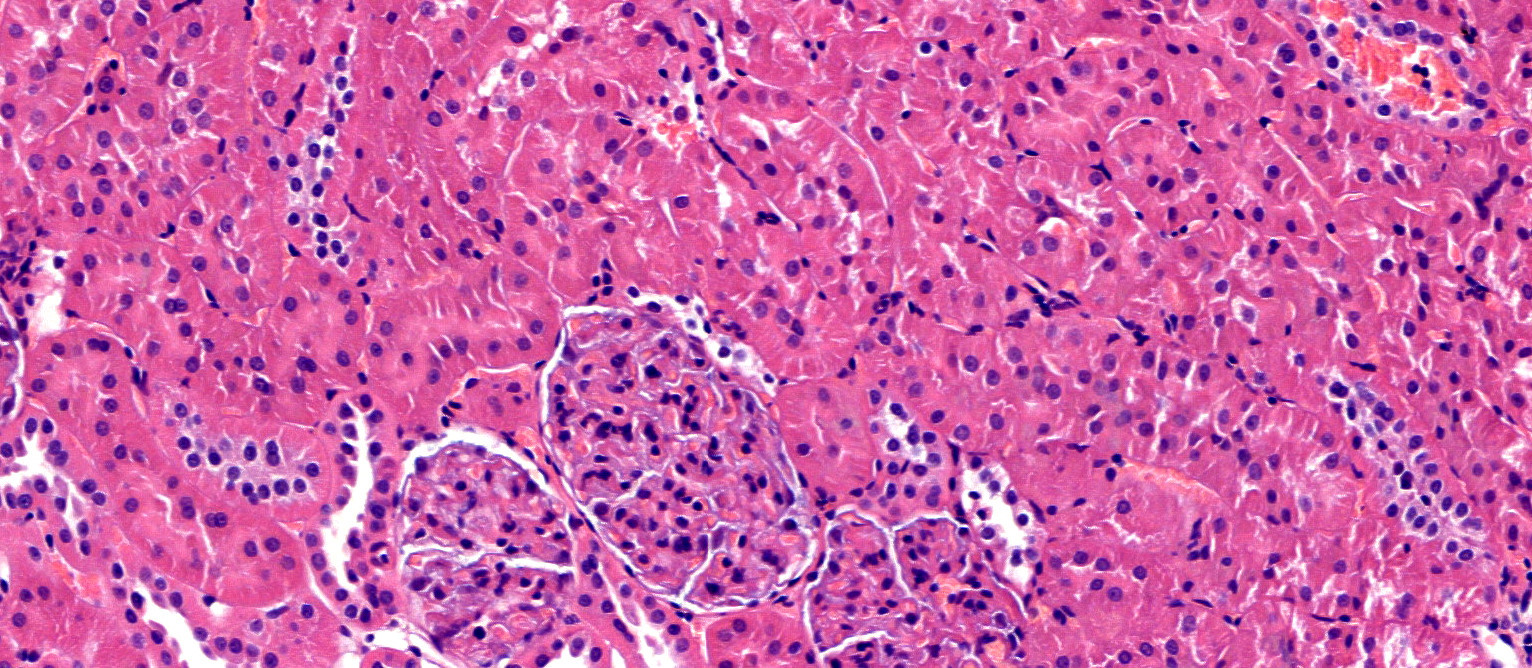

Supplement: Supplementary file 14 [file DataSheet5.ZIP › Fig 1D-HE-TSF-60/60-5.jpeg]

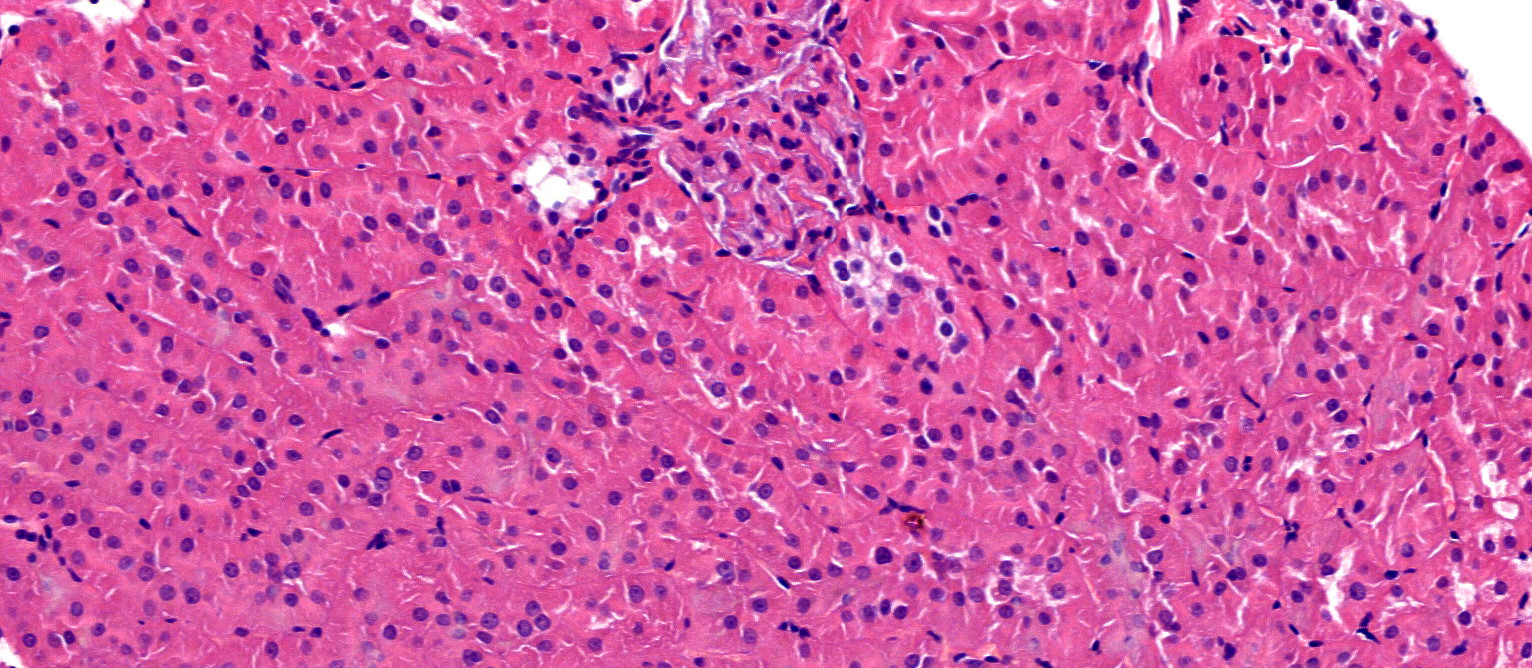

Supplement: Supplementary file 14 [file DataSheet5.ZIP › Fig 1D-HE-TSF-60/60-6.jpeg]

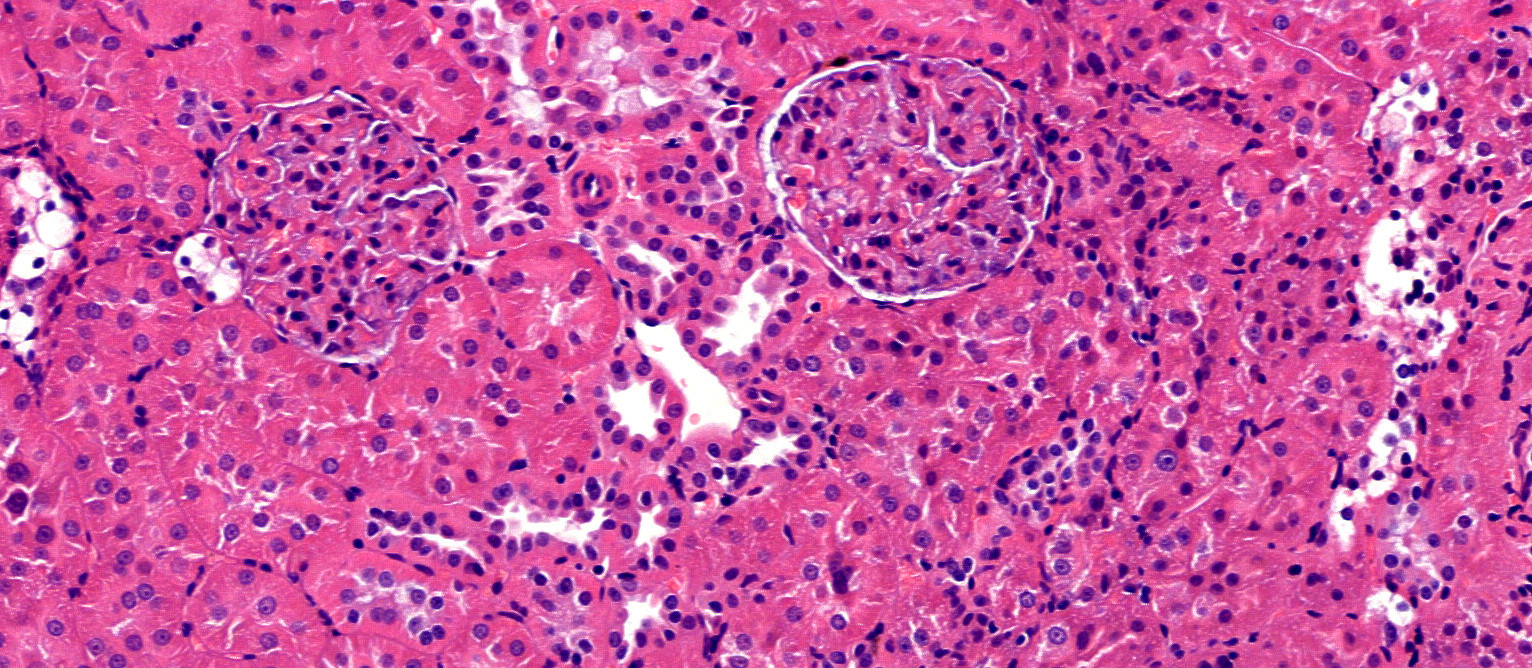

Supplement: Supplementary file 14 [file DataSheet5.ZIP › Fig 1D-HE-TSF-60/60-7.jpeg]

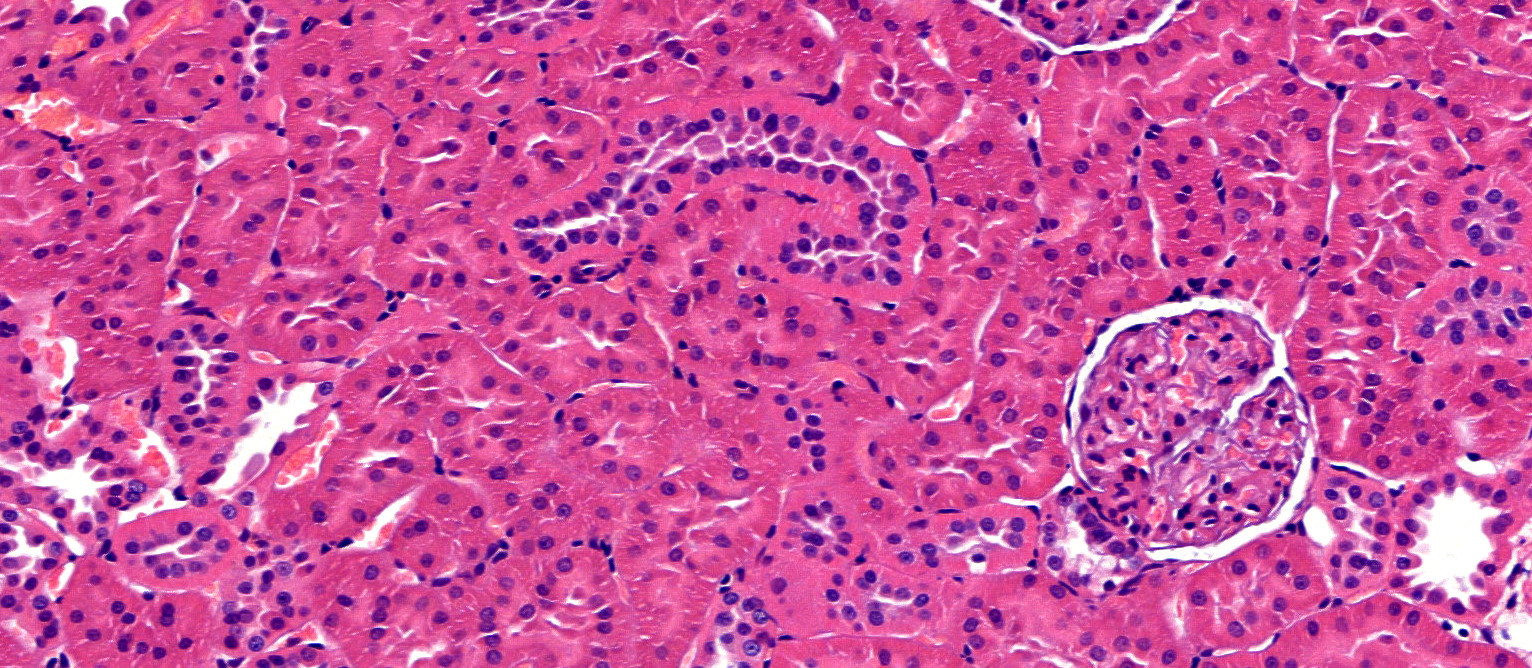

Supplement: Supplementary file 14 [file DataSheet5.ZIP › Fig 1D-HE-TSF-60/60-8.jpeg]

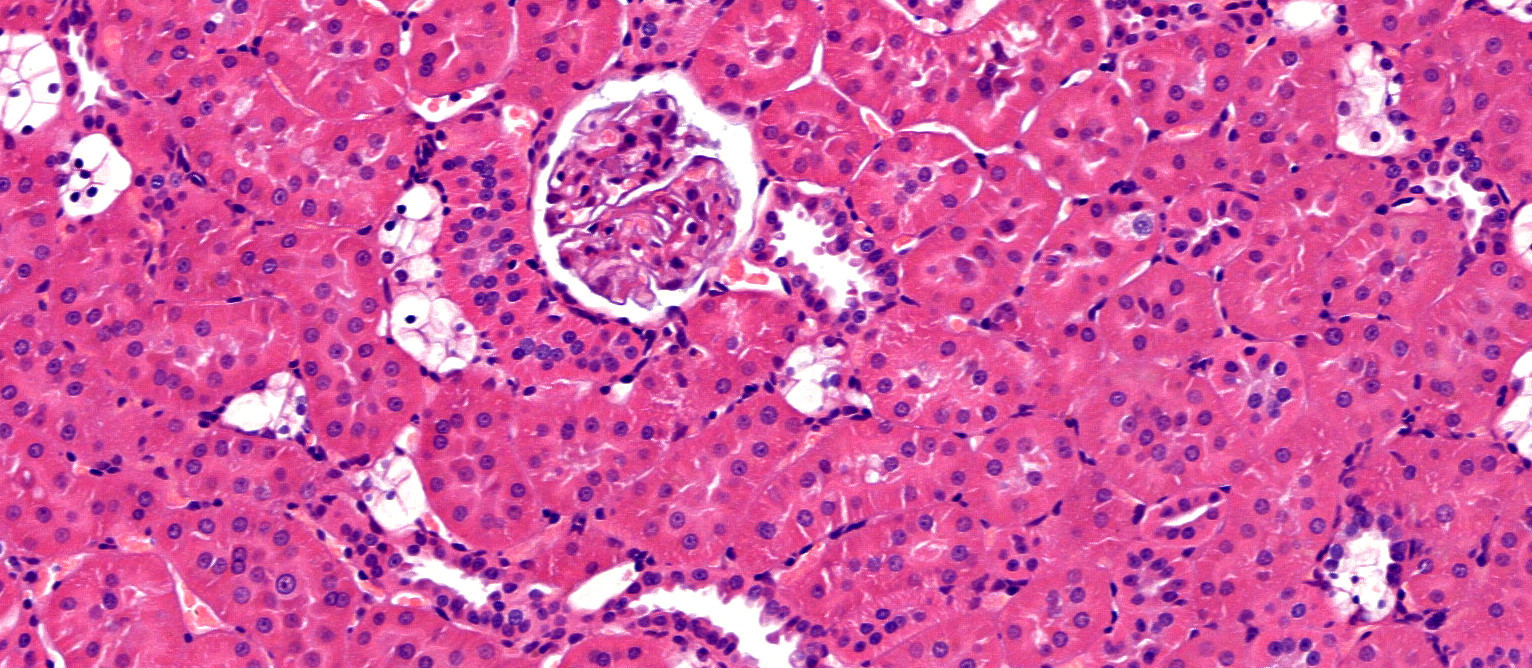

Supplement: Supplementary file 14 [file DataSheet5.ZIP › Fig 1D-HE-TSF-60/60-9.jpeg]

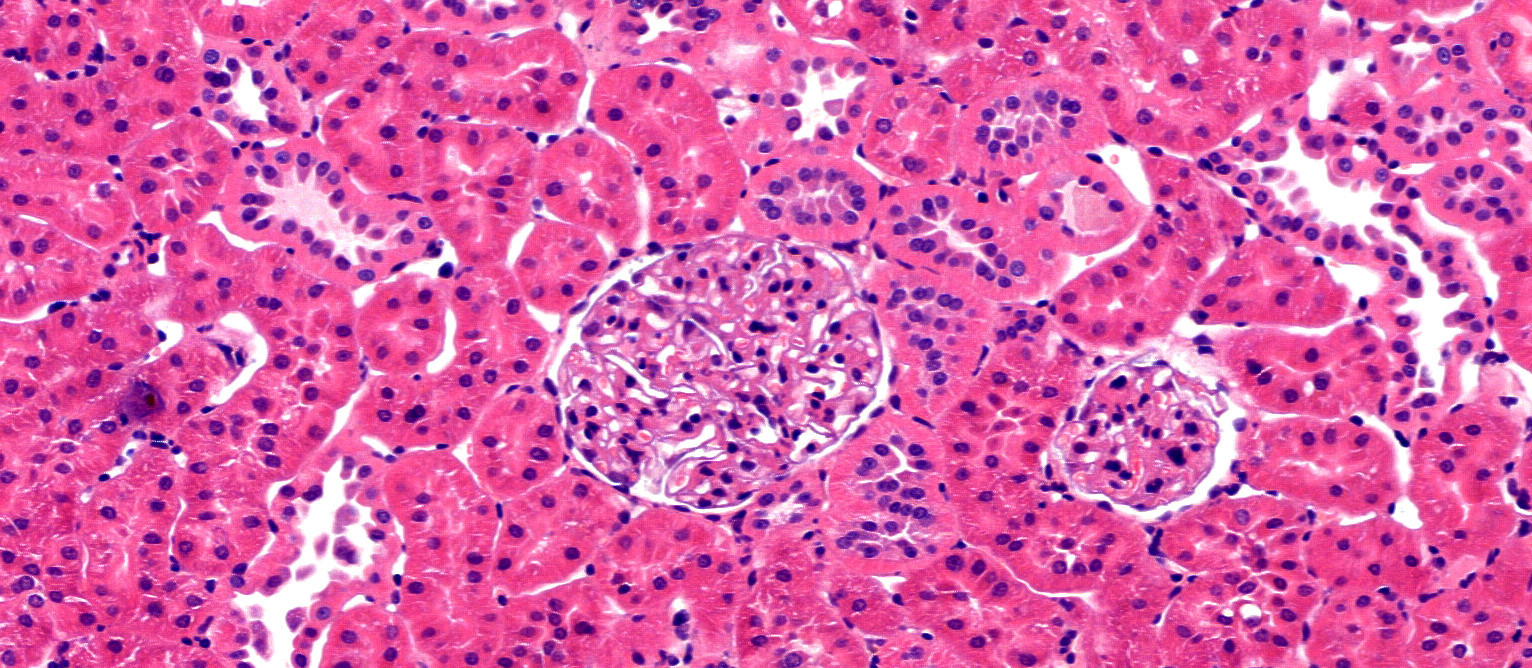

Supplement: Supplementary file 14 [file DataSheet5.ZIP › Fig 1D-HE-TSF-61/61-1.jpeg]

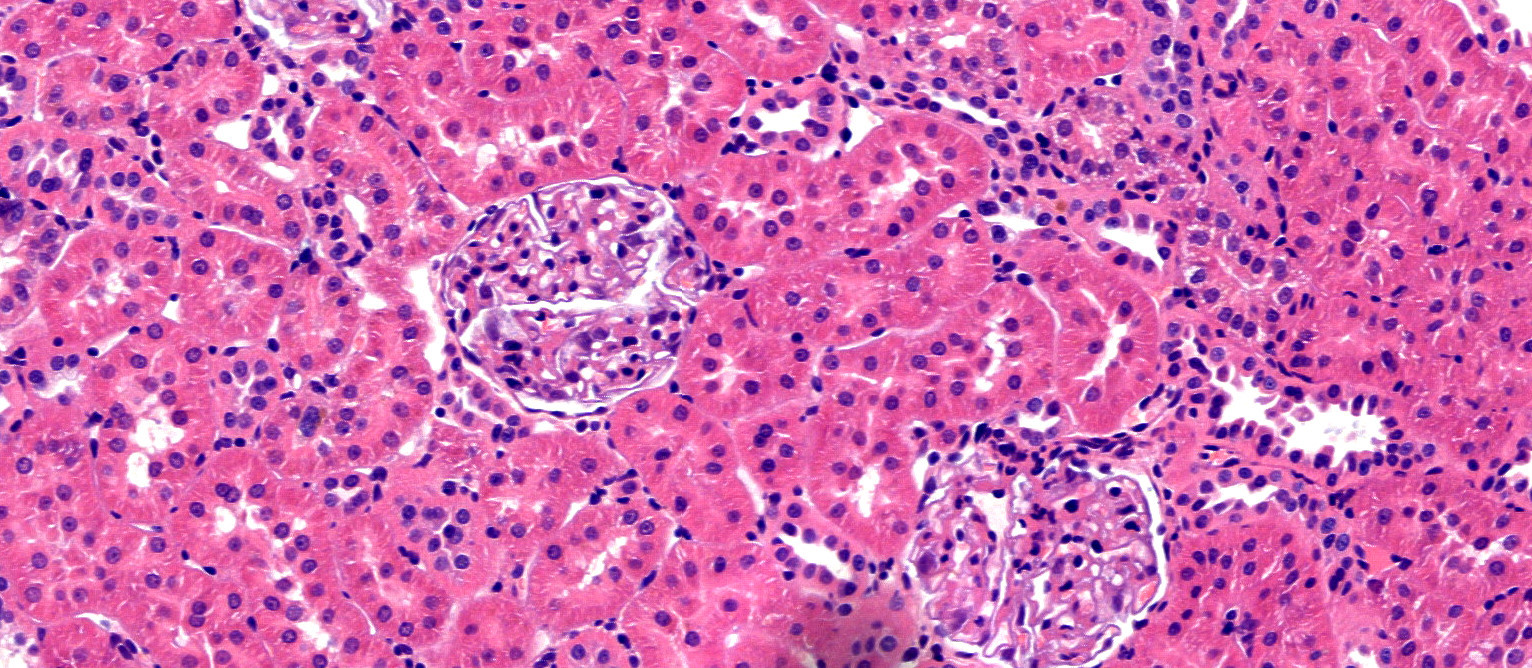

Supplement: Supplementary file 14 [file DataSheet5.ZIP › Fig 1D-HE-TSF-61/61-10.jpeg]

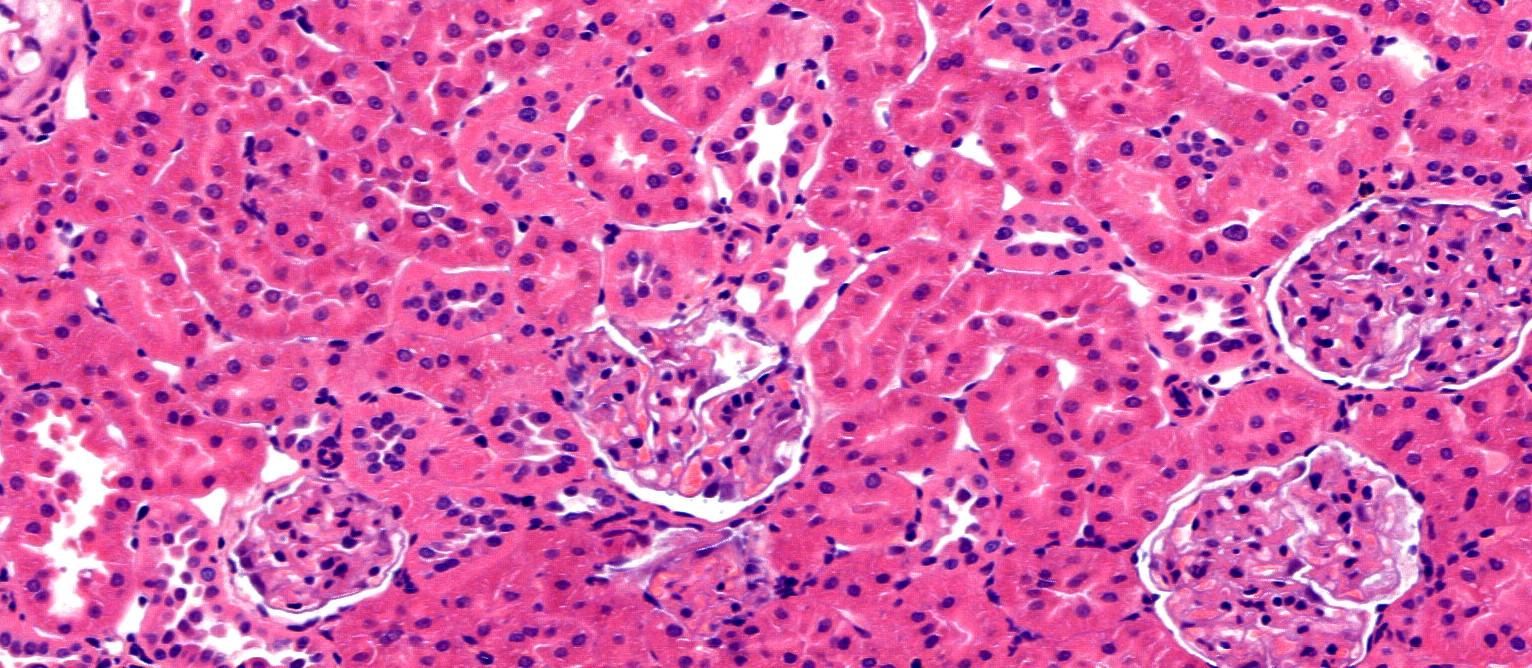

Supplement: Supplementary file 14 [file DataSheet5.ZIP › Fig 1D-HE-TSF-61/61-2.jpeg]

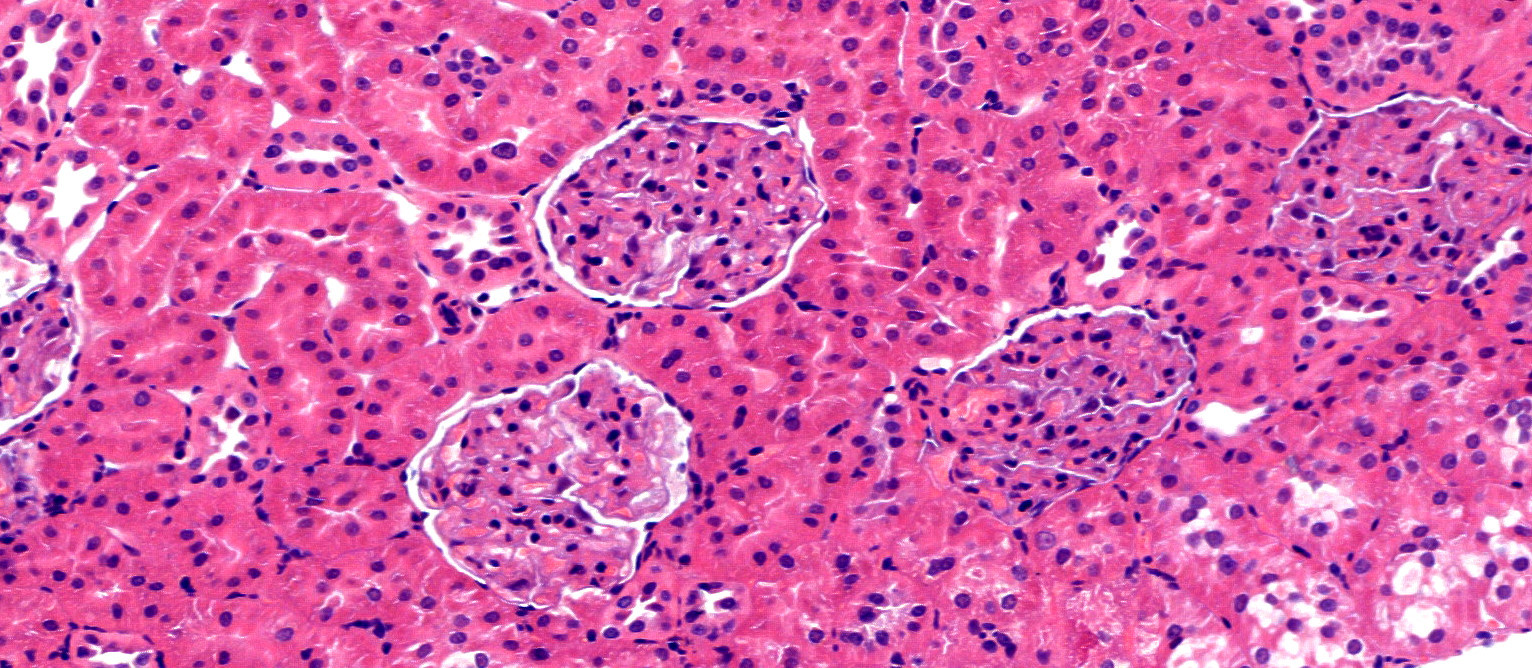

Supplement: Supplementary file 14 [file DataSheet5.ZIP › Fig 1D-HE-TSF-61/61-3.jpeg]

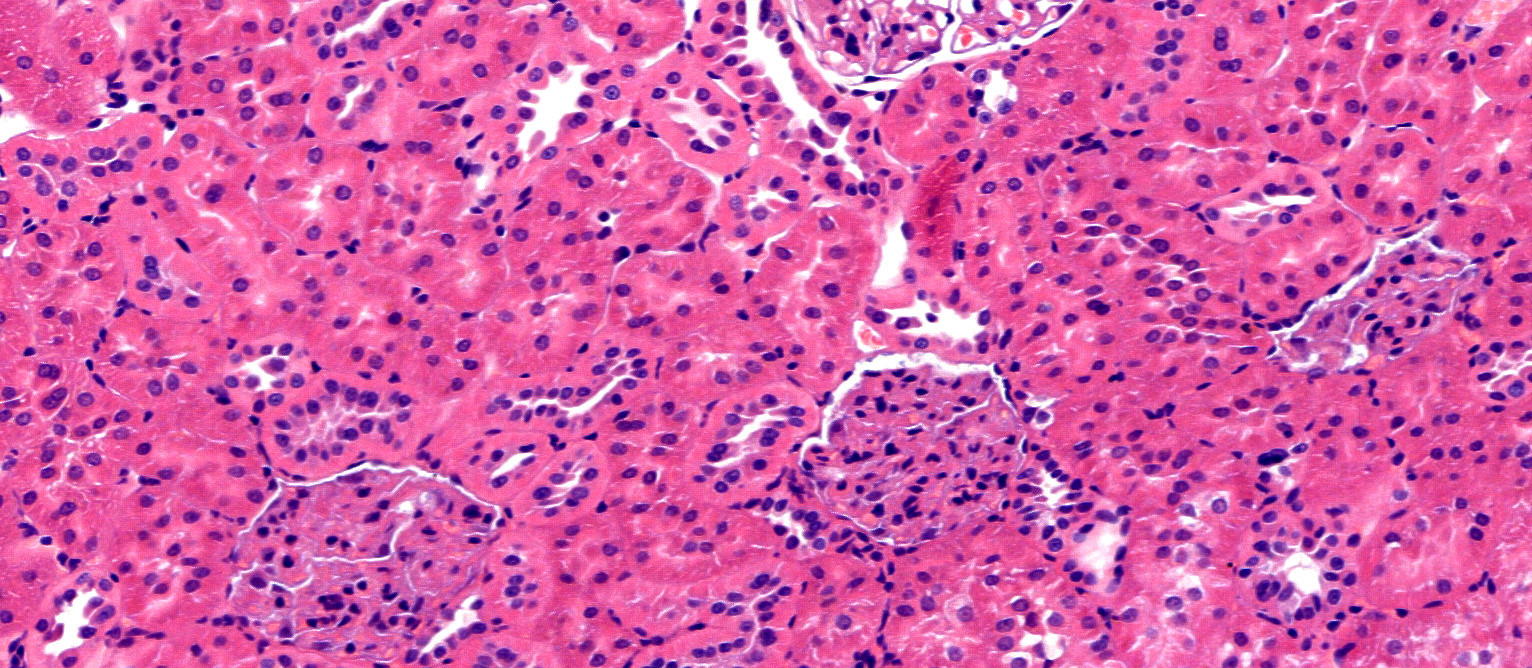

Supplement: Supplementary file 14 [file DataSheet5.ZIP › Fig 1D-HE-TSF-61/61-4.jpeg]

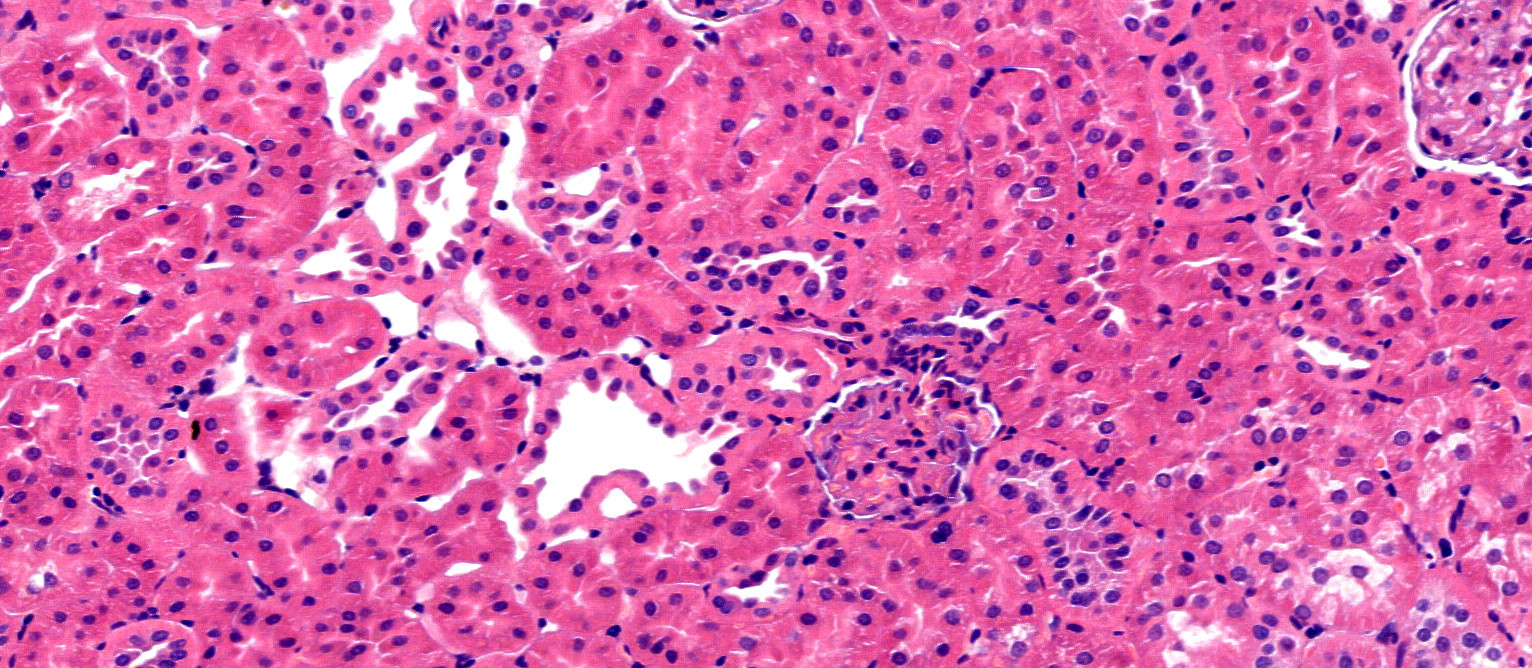

Supplement: Supplementary file 14 [file DataSheet5.ZIP › Fig 1D-HE-TSF-61/61-5.jpeg]

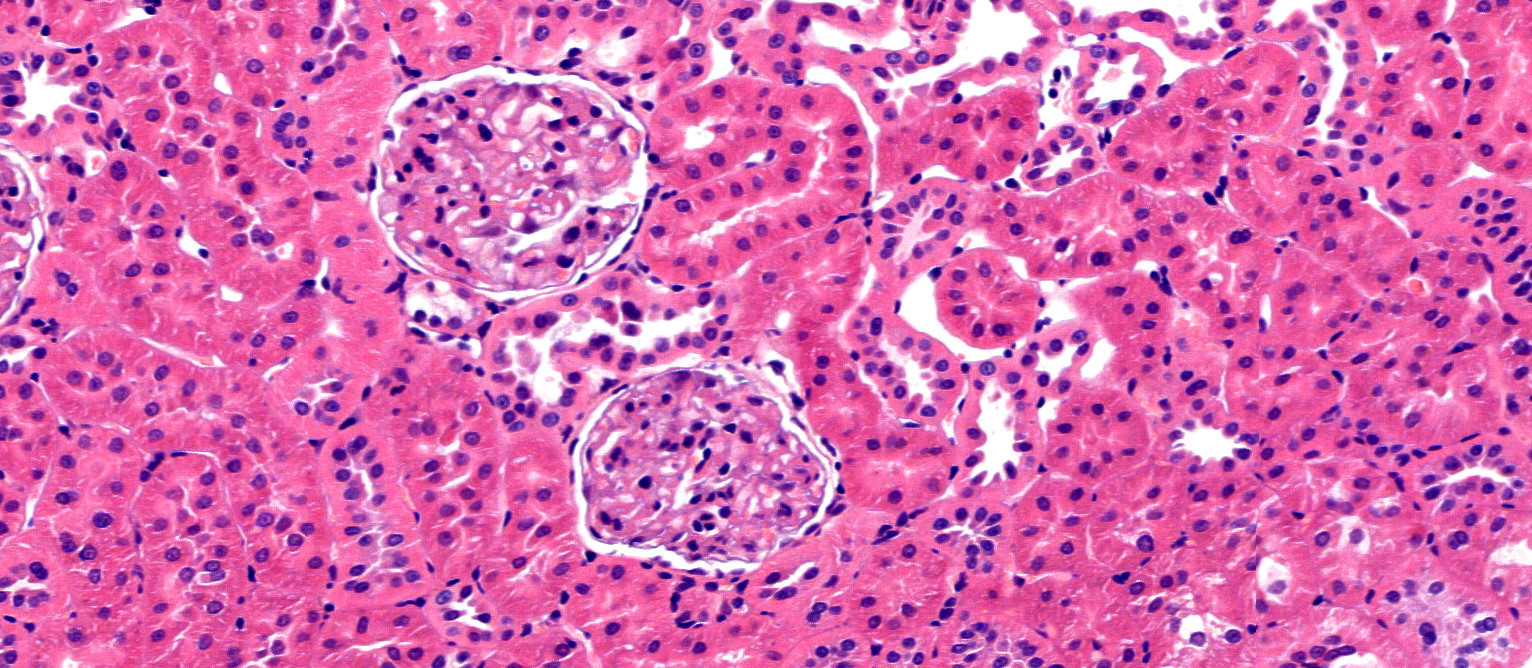

Supplement: Supplementary file 14 [file DataSheet5.ZIP › Fig 1D-HE-TSF-61/61-6.jpeg]

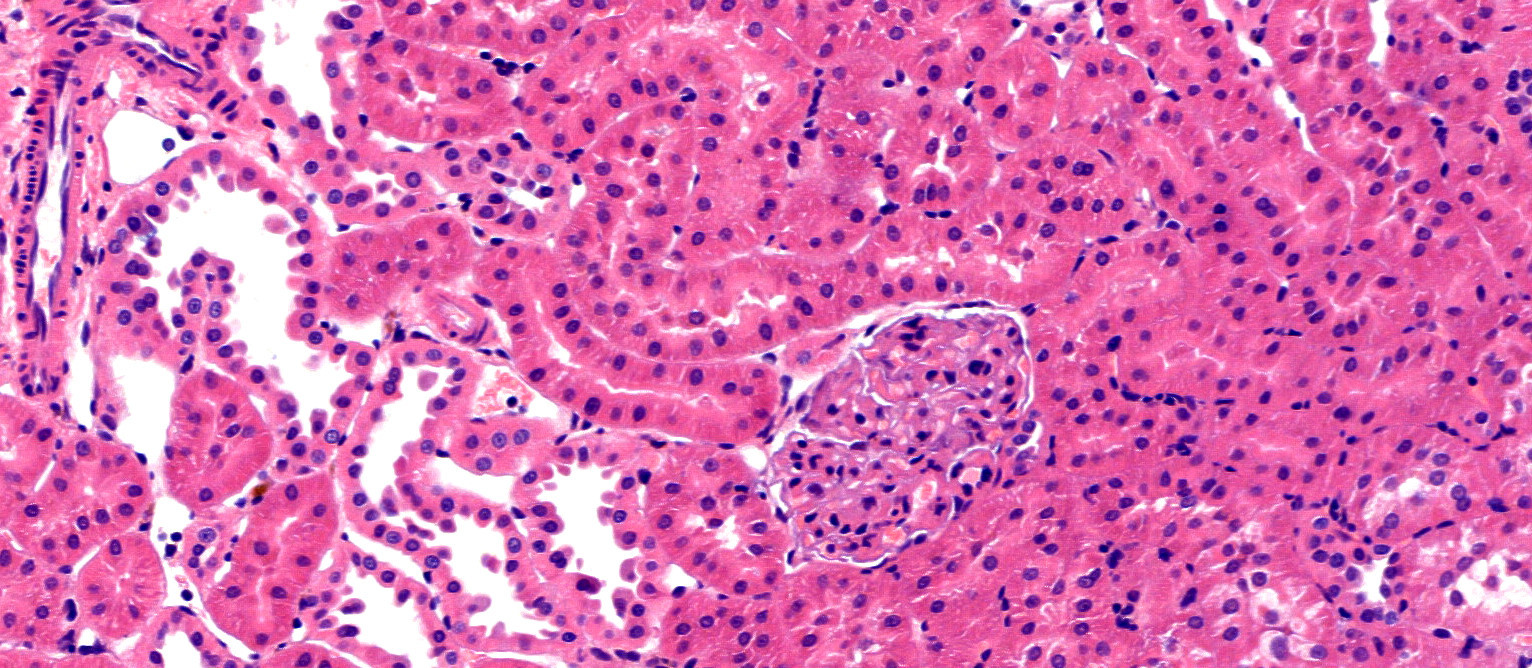

Supplement: Supplementary file 14 [file DataSheet5.ZIP › Fig 1D-HE-TSF-61/61-7.jpeg]

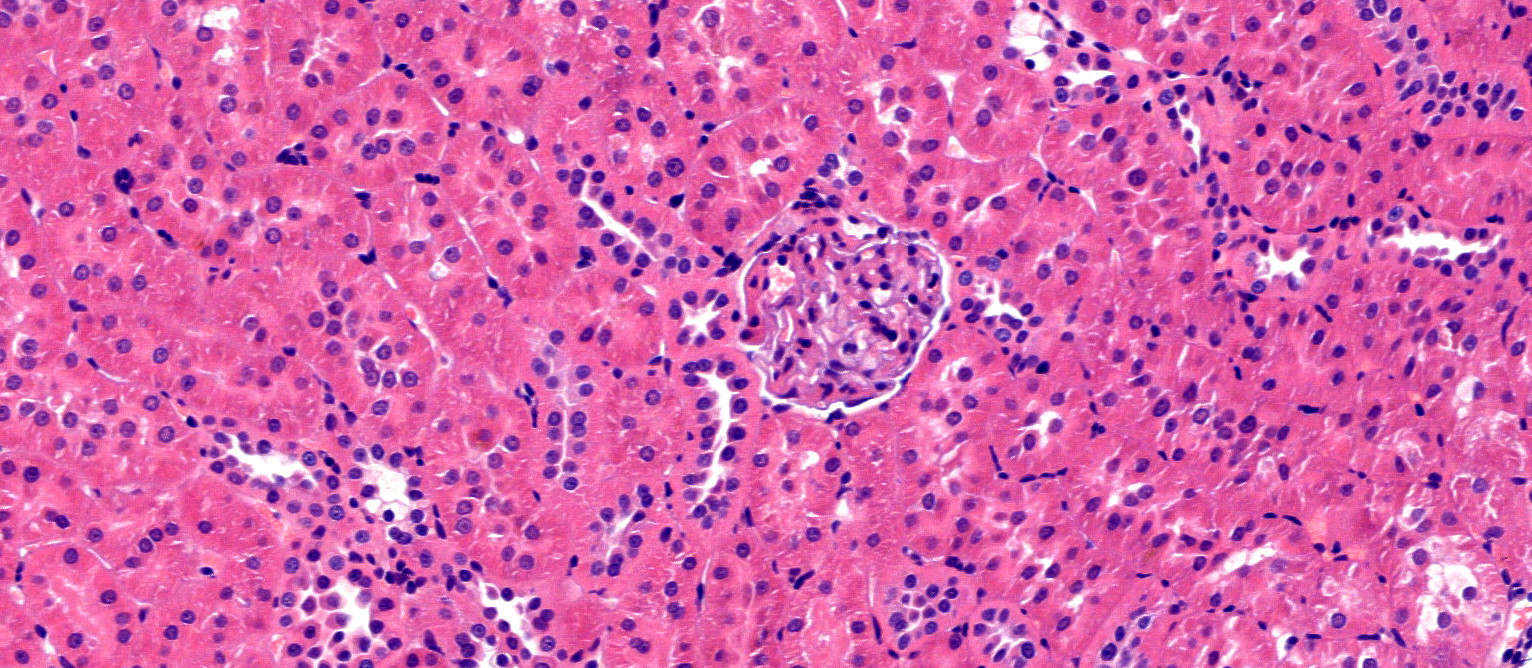

Supplement: Supplementary file 14 [file DataSheet5.ZIP › Fig 1D-HE-TSF-61/61-8.jpeg]

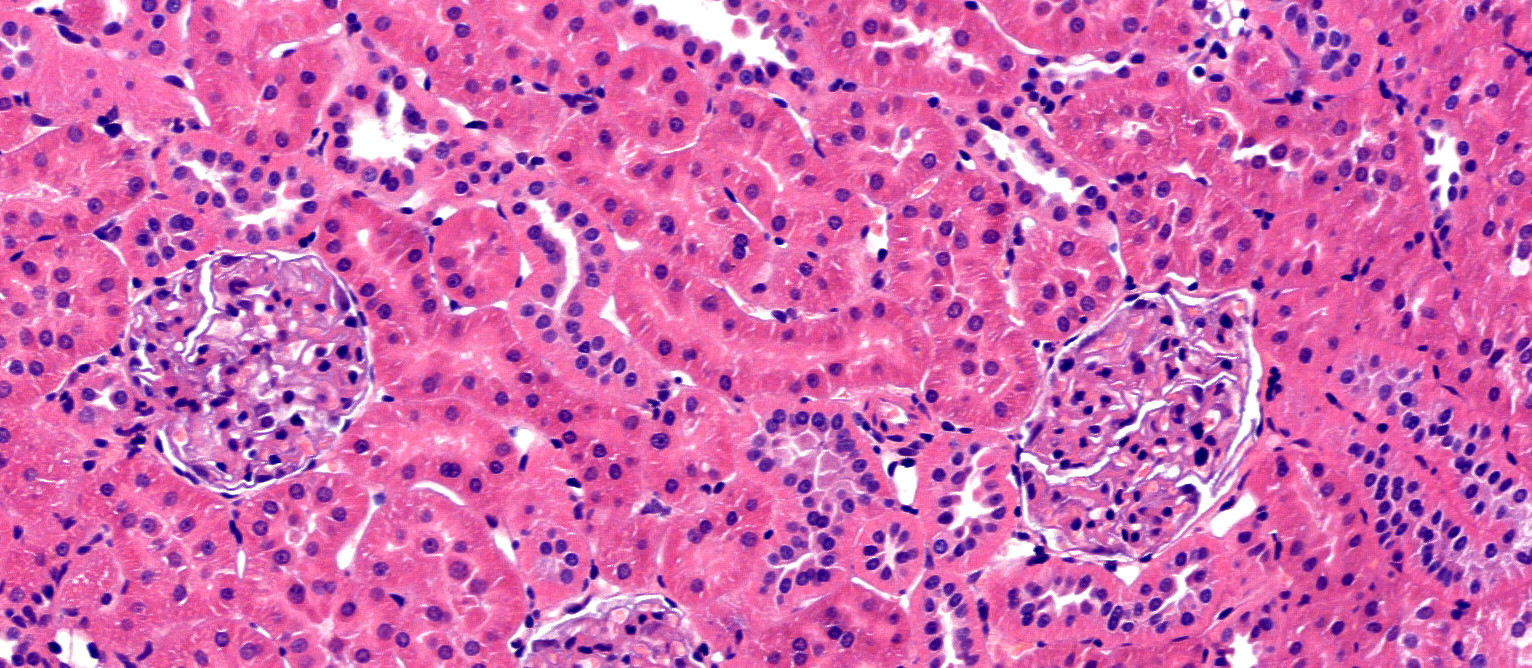

Supplement: Supplementary file 14 [file DataSheet5.ZIP › Fig 1D-HE-TSF-61/61-9.jpeg]

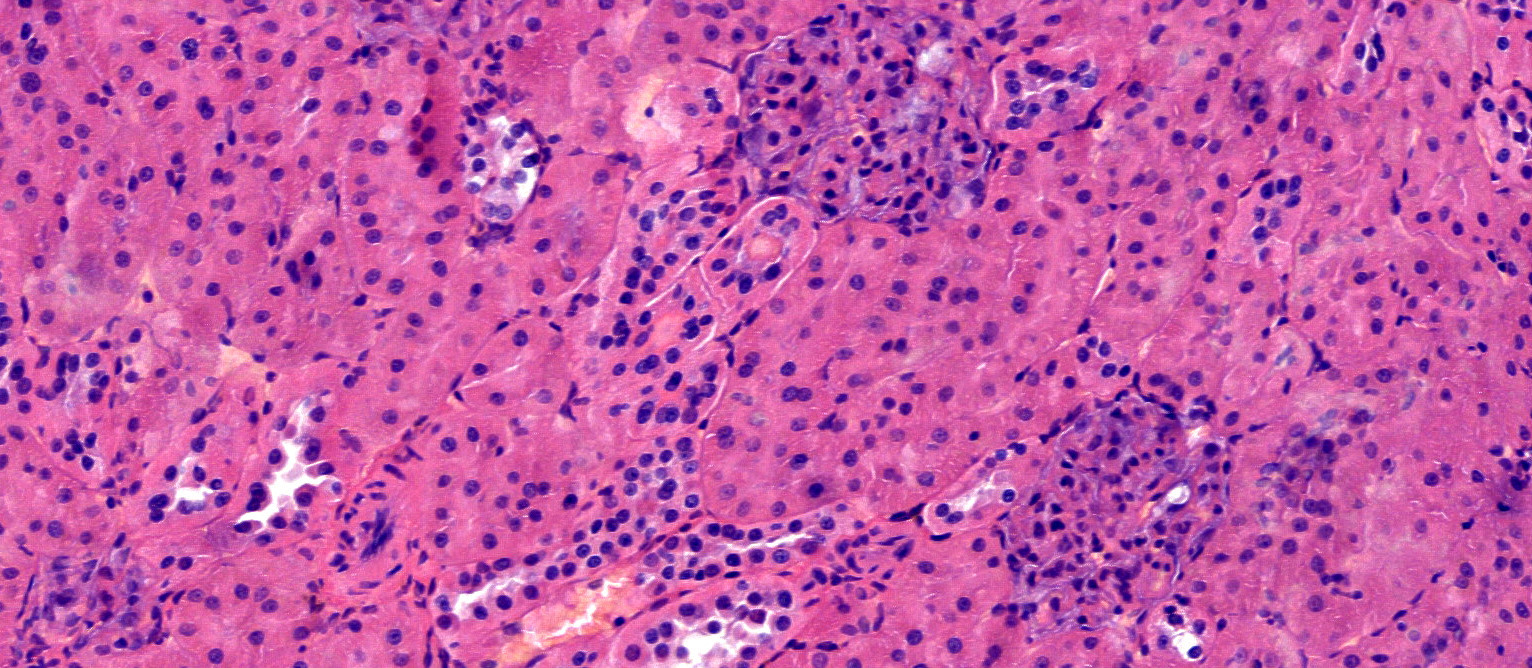

Supplement: Supplementary file 14 [file DataSheet5.ZIP › Fig 1D-HE-TSF-62/62-1.jpeg]

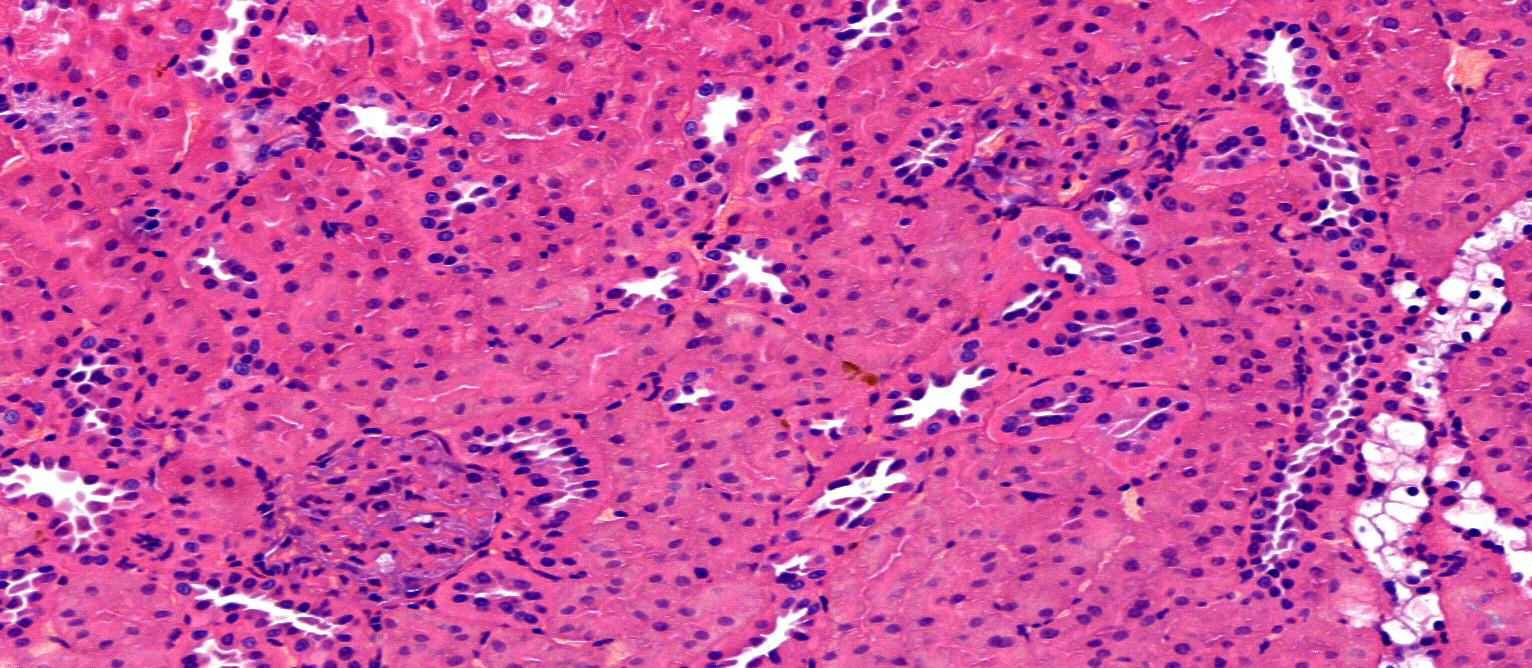

Supplement: Supplementary file 14 [file DataSheet5.ZIP › Fig 1D-HE-TSF-62/62-10.jpeg]

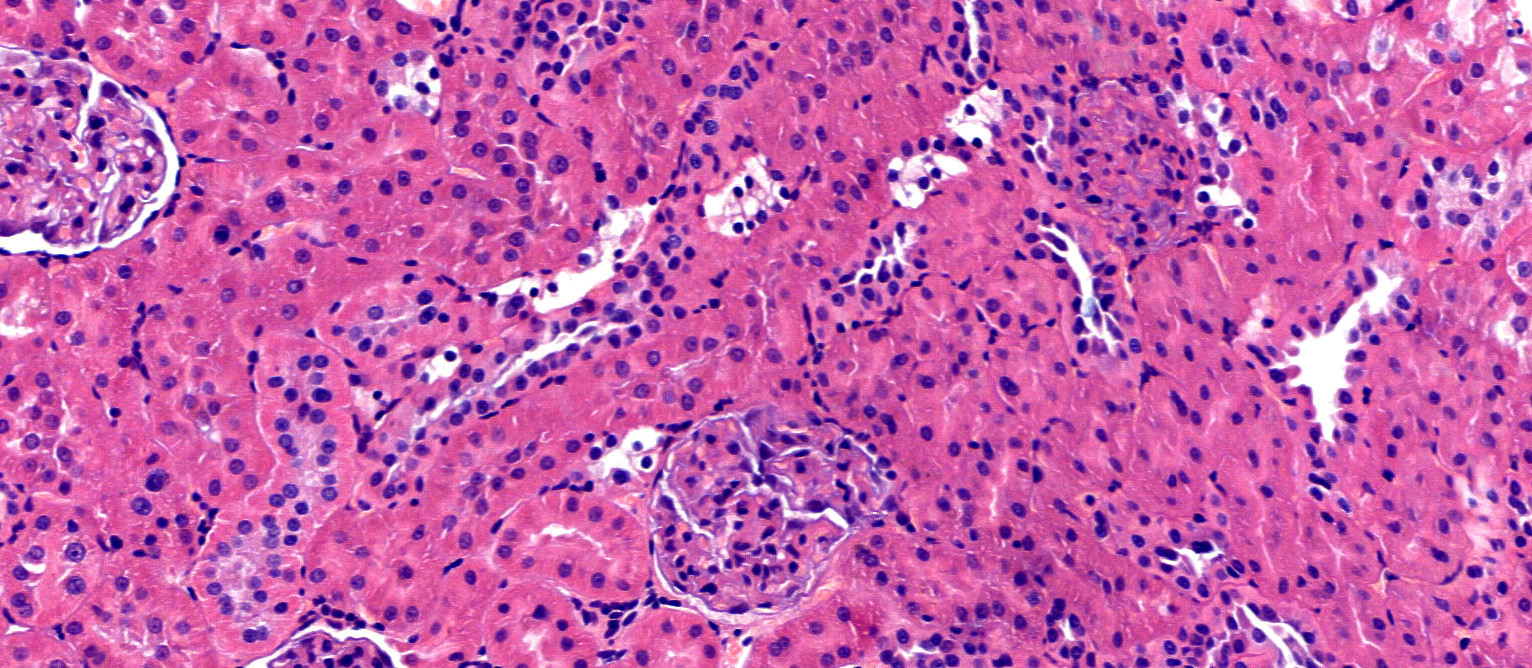

Supplement: Supplementary file 14 [file DataSheet5.ZIP › Fig 1D-HE-TSF-62/62-2.jpeg]

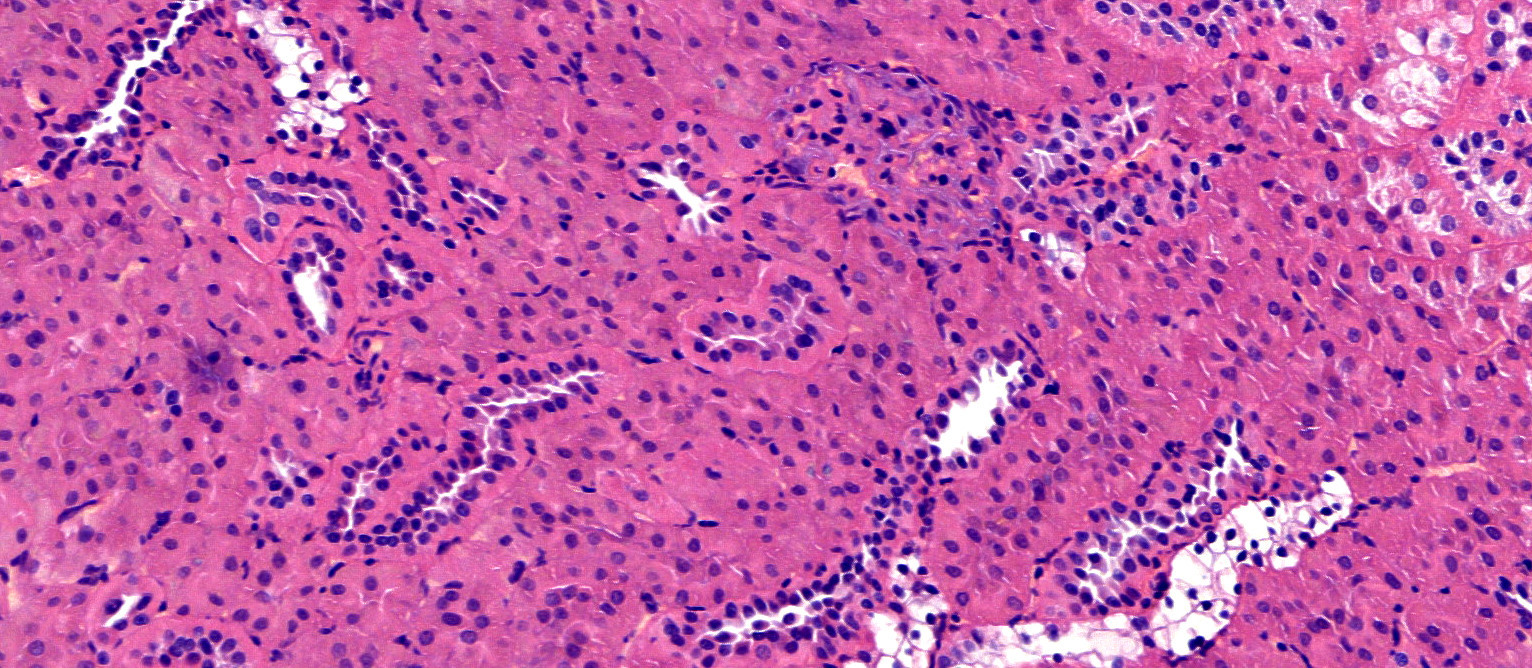

Supplement: Supplementary file 14 [file DataSheet5.ZIP › Fig 1D-HE-TSF-62/62-3.jpeg]

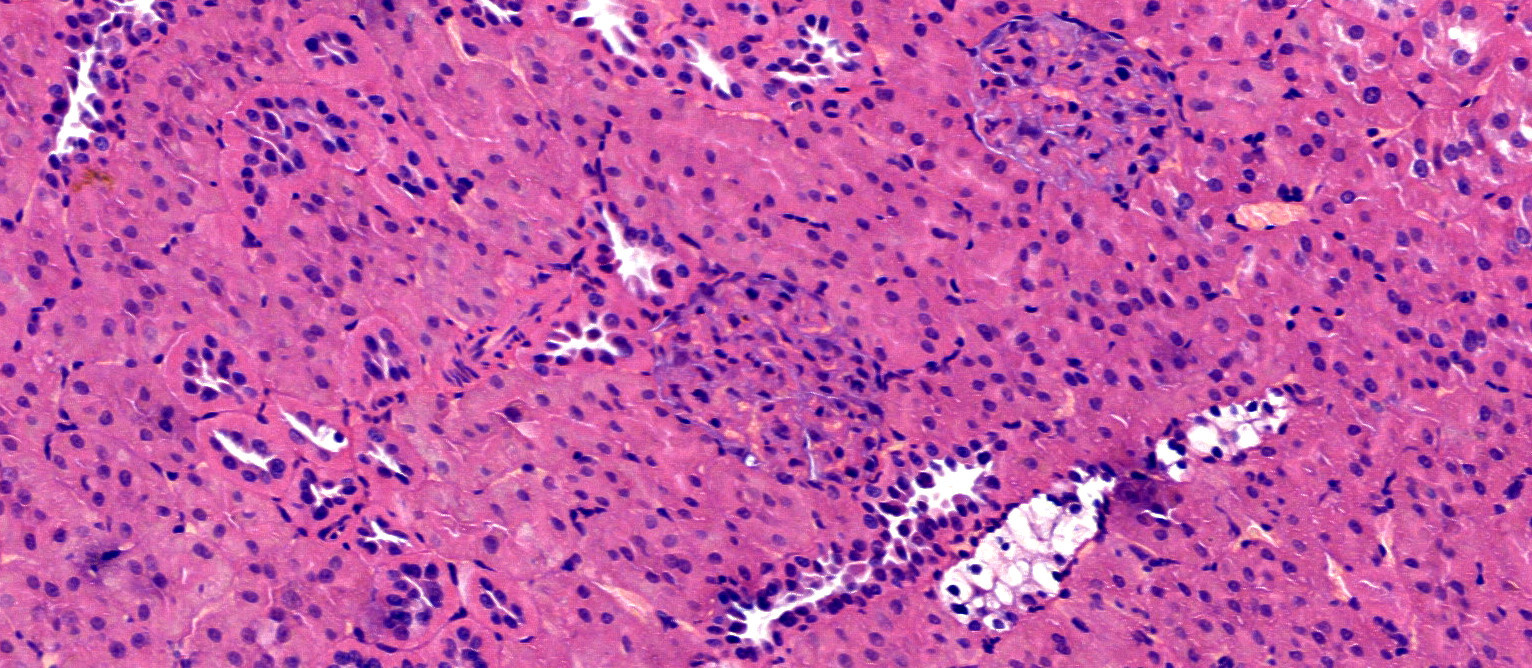

Supplement: Supplementary file 14 [file DataSheet5.ZIP › Fig 1D-HE-TSF-62/62-4.jpeg]

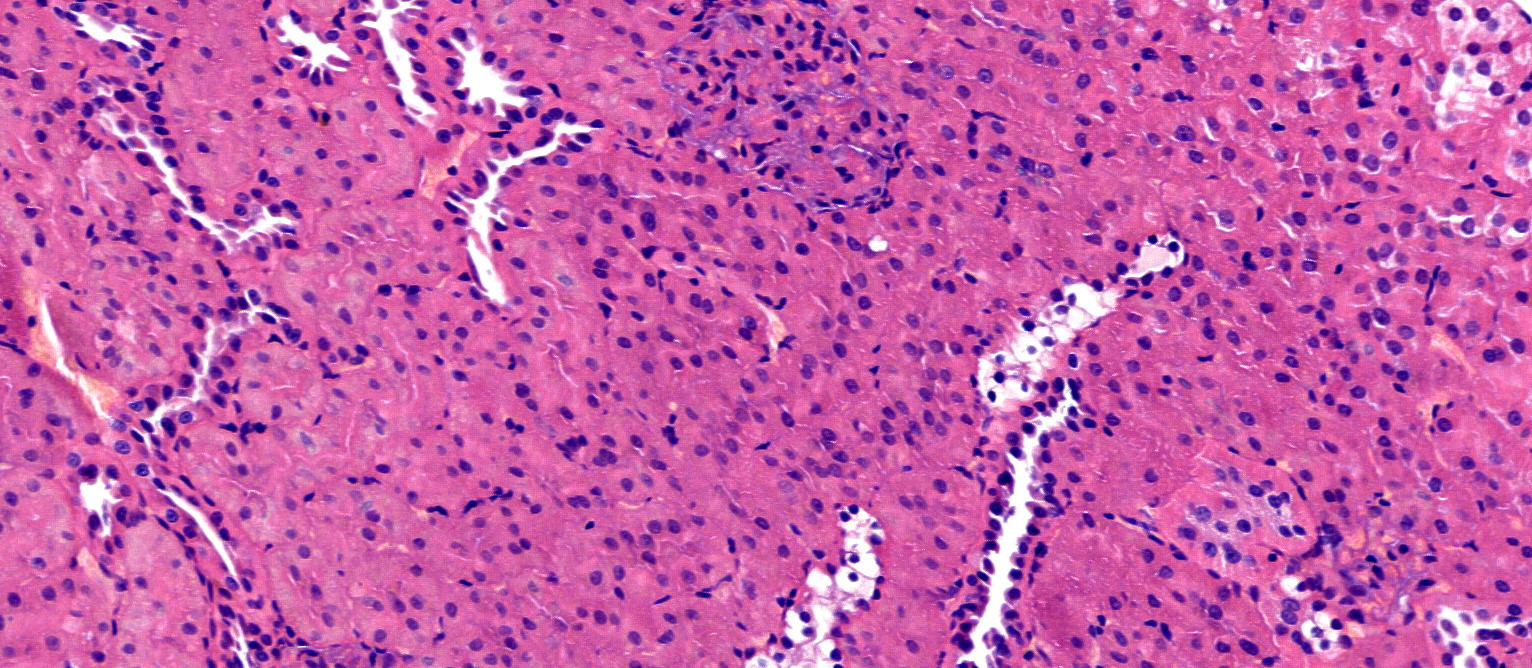

Supplement: Supplementary file 14 [file DataSheet5.ZIP › Fig 1D-HE-TSF-62/62-5.jpeg]

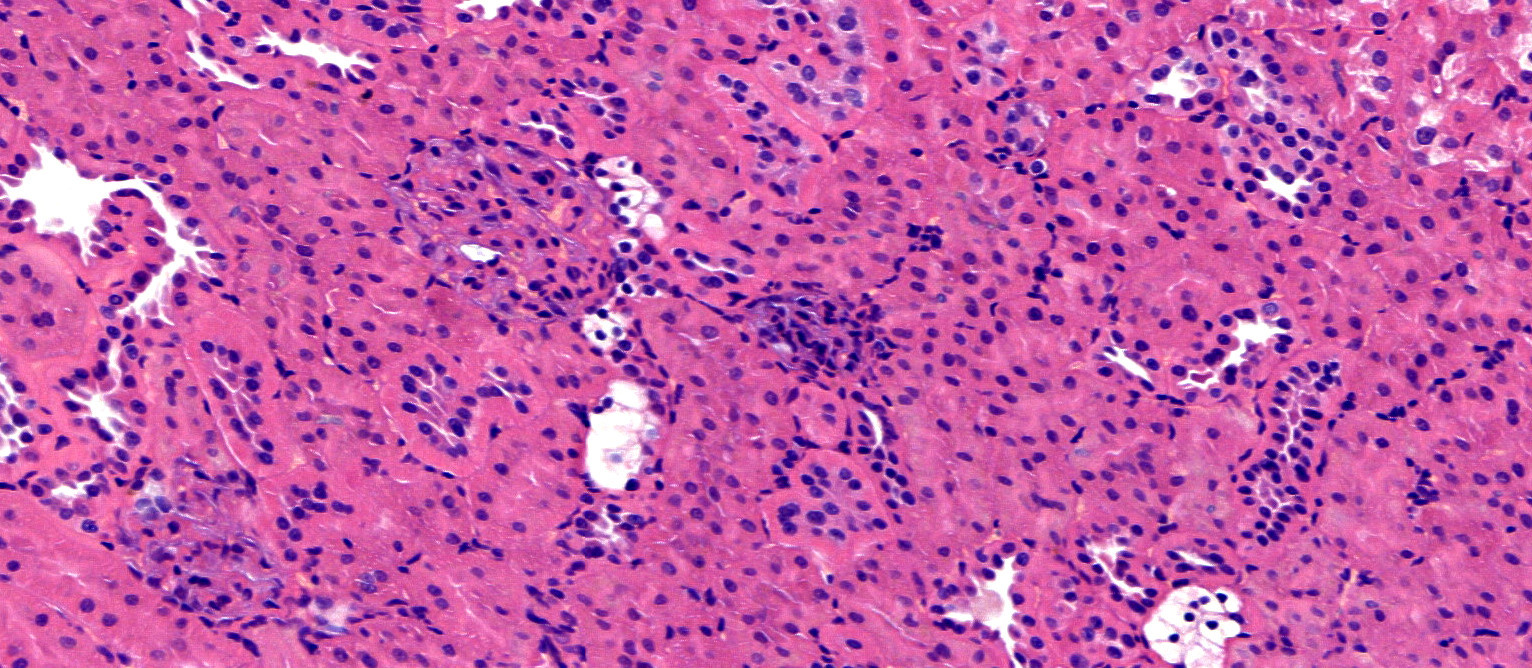

Supplement: Supplementary file 14 [file DataSheet5.ZIP › Fig 1D-HE-TSF-62/62-6.jpeg]

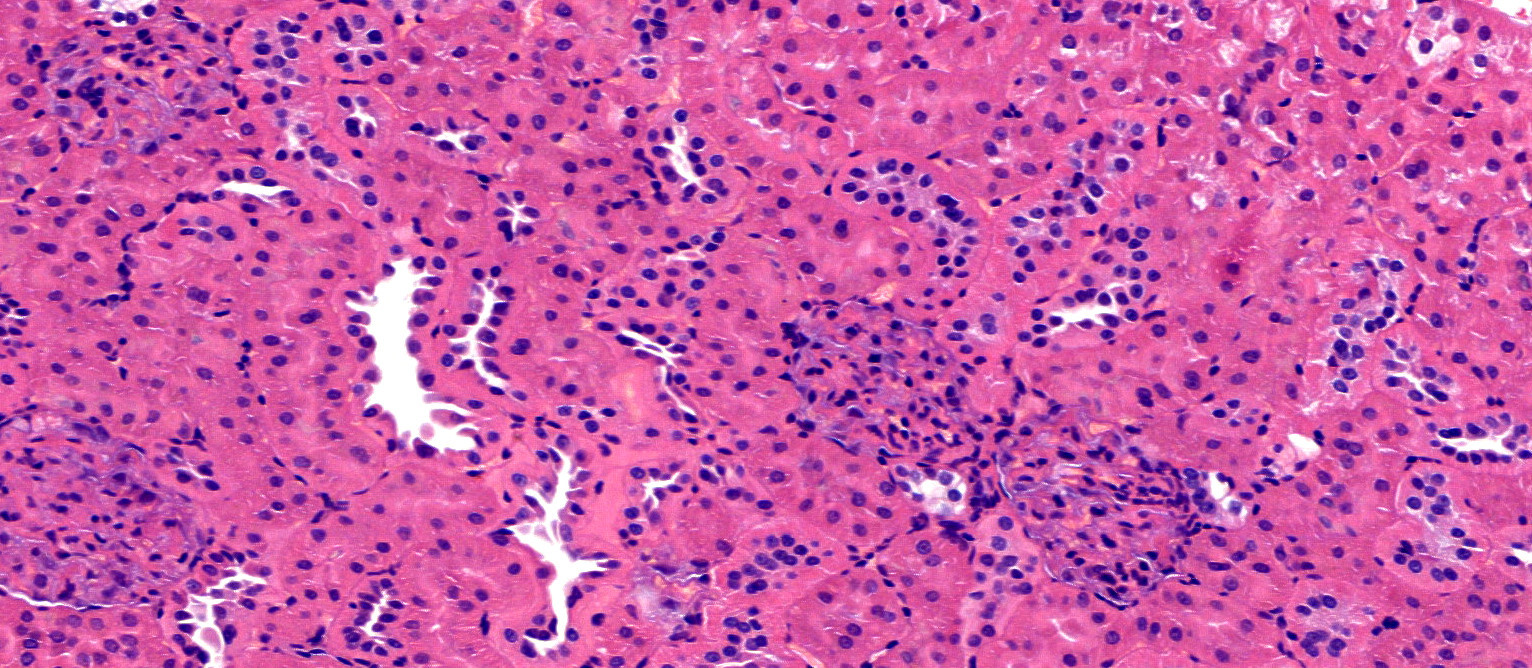

Supplement: Supplementary file 14 [file DataSheet5.ZIP › Fig 1D-HE-TSF-62/62-7.jpeg]

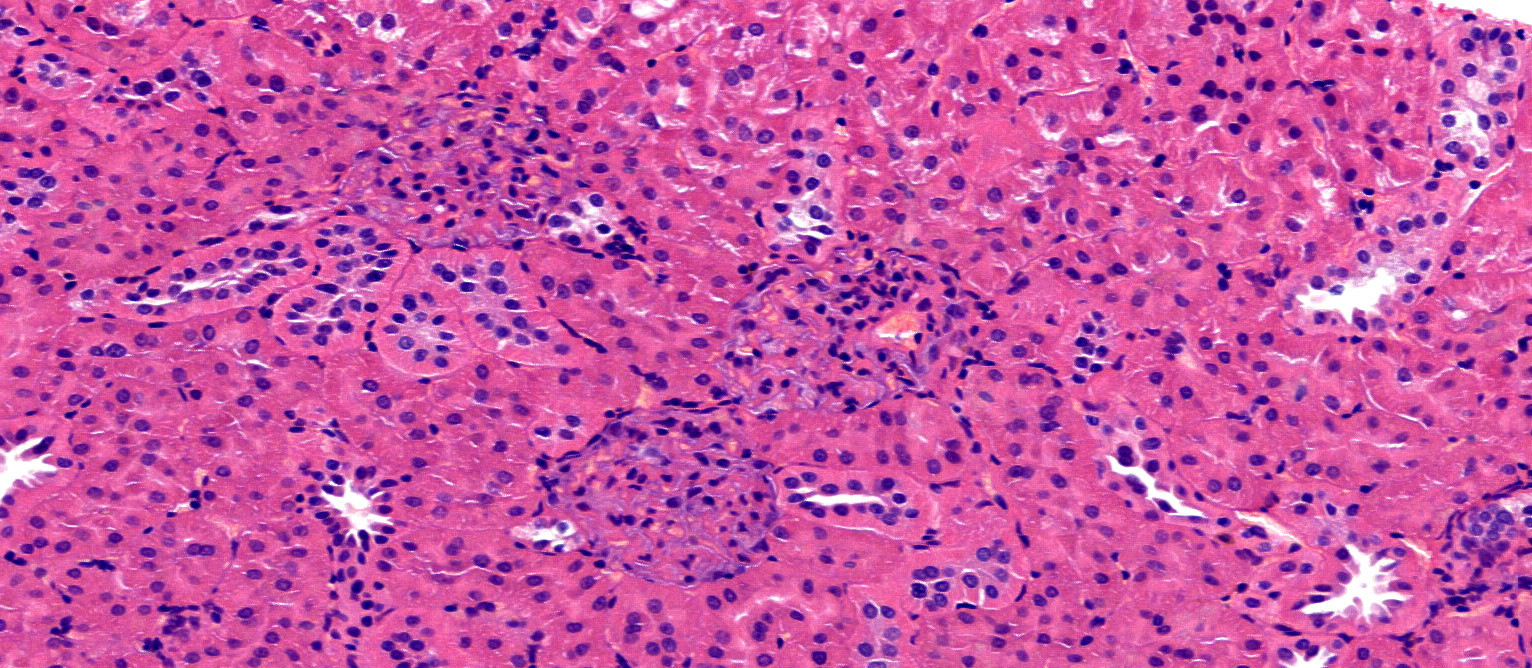

Supplement: Supplementary file 14 [file DataSheet5.ZIP › Fig 1D-HE-TSF-62/62-8.jpeg]

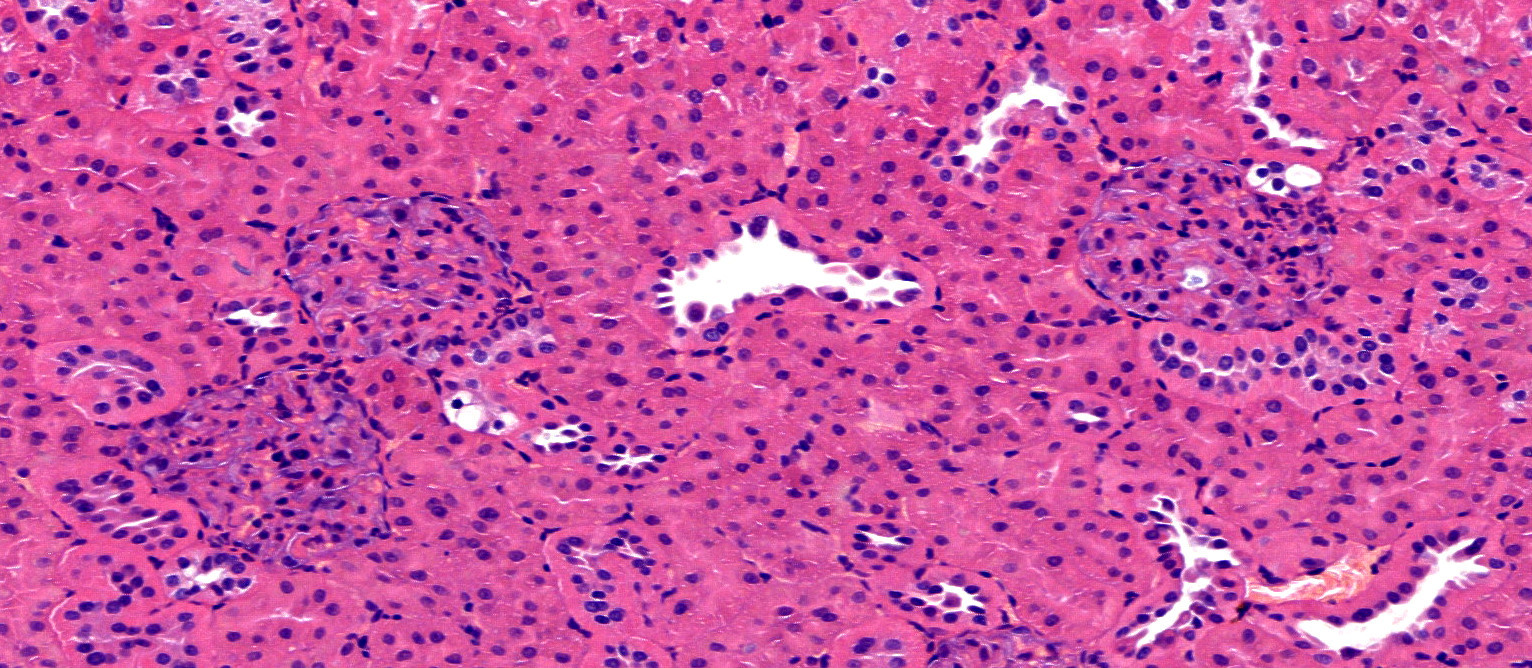

Supplement: Supplementary file 14 [file DataSheet5.ZIP › Fig 1D-HE-TSF-62/62-9.jpeg]

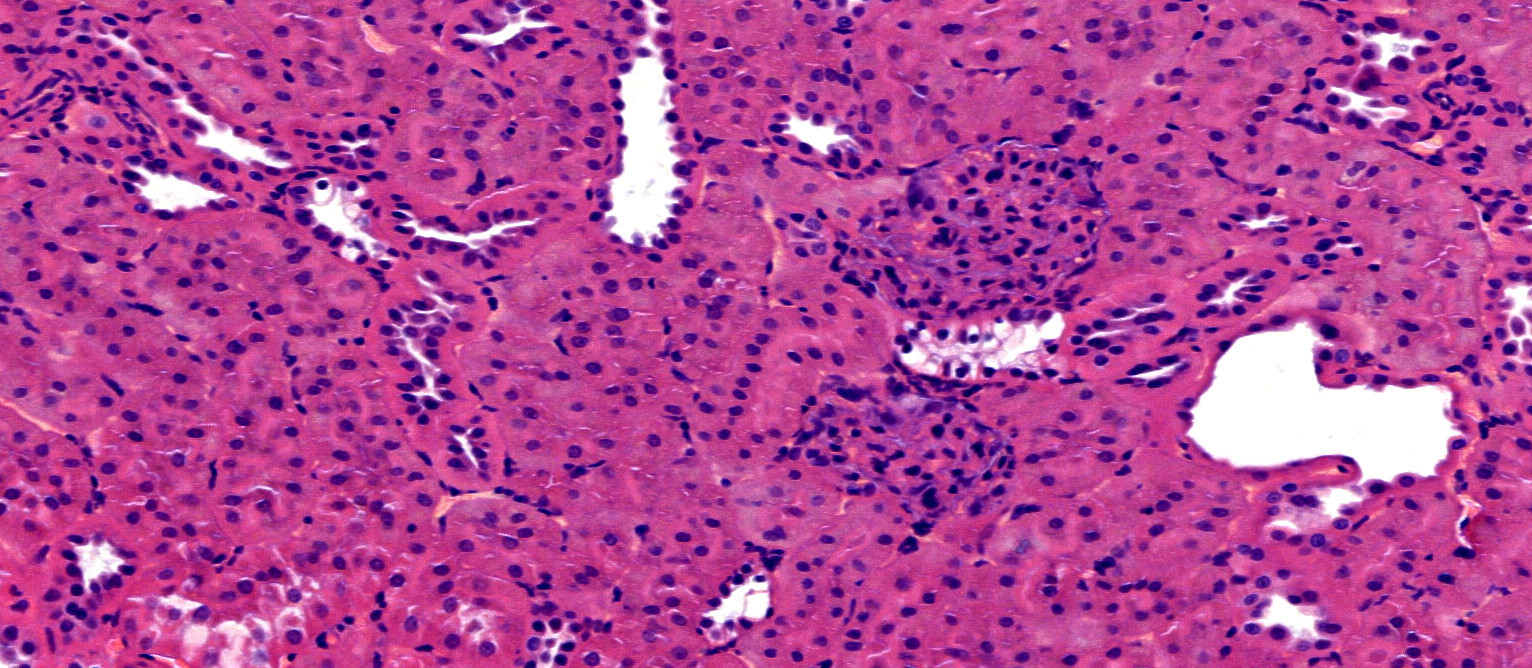

Supplement: Supplementary file 14 [file DataSheet5.ZIP › Fig 1D-HE-TSF-63(1)/63-1.jpeg]

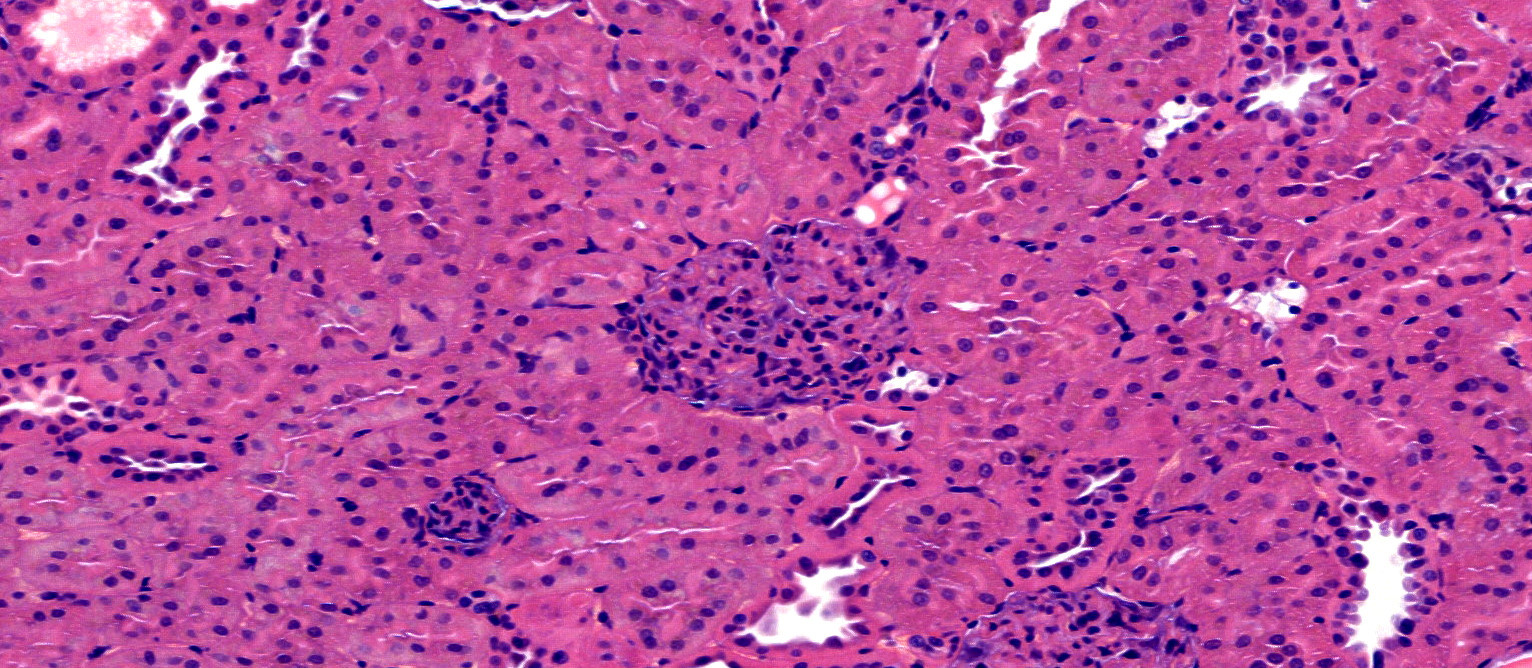

Supplement: Supplementary file 14 [file DataSheet5.ZIP › Fig 1D-HE-TSF-63(1)/63-2.jpeg]

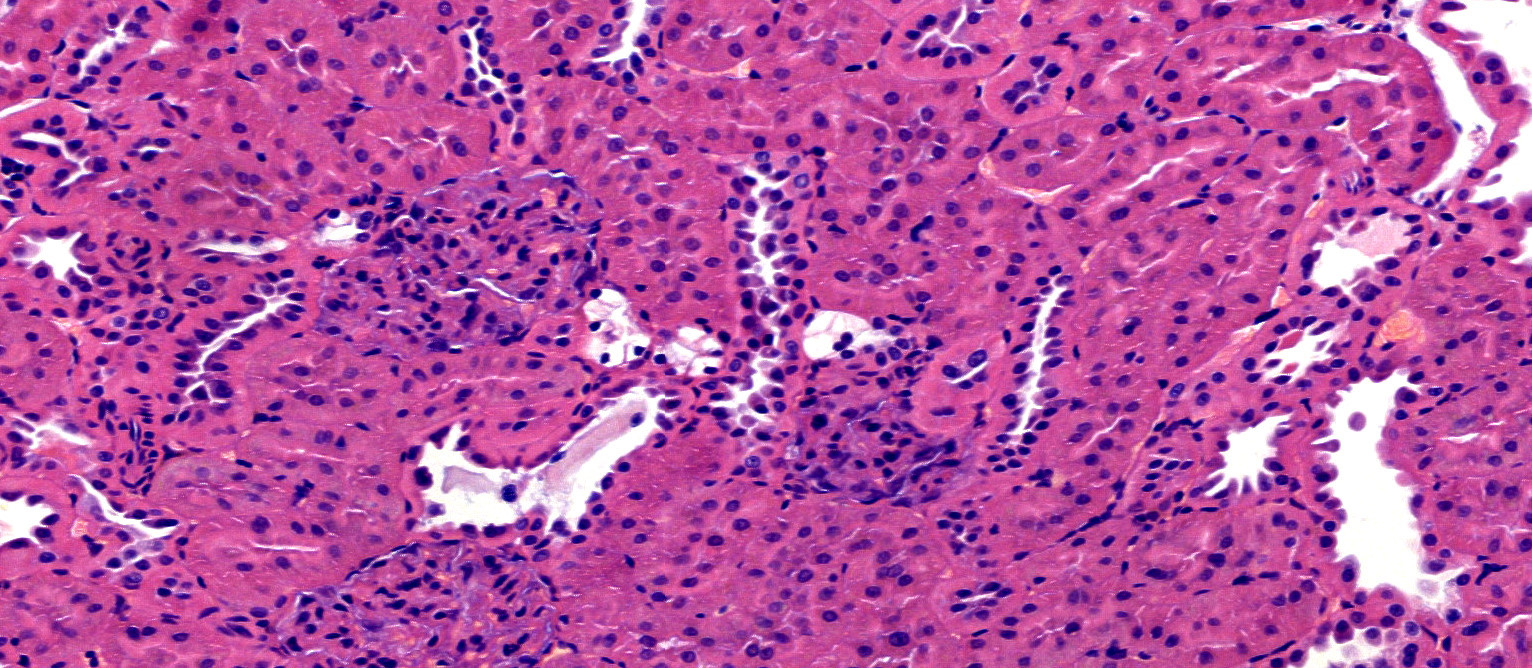

Supplement: Supplementary file 14 [file DataSheet5.ZIP › Fig 1D-HE-TSF-63(1)/63-3.jpeg]

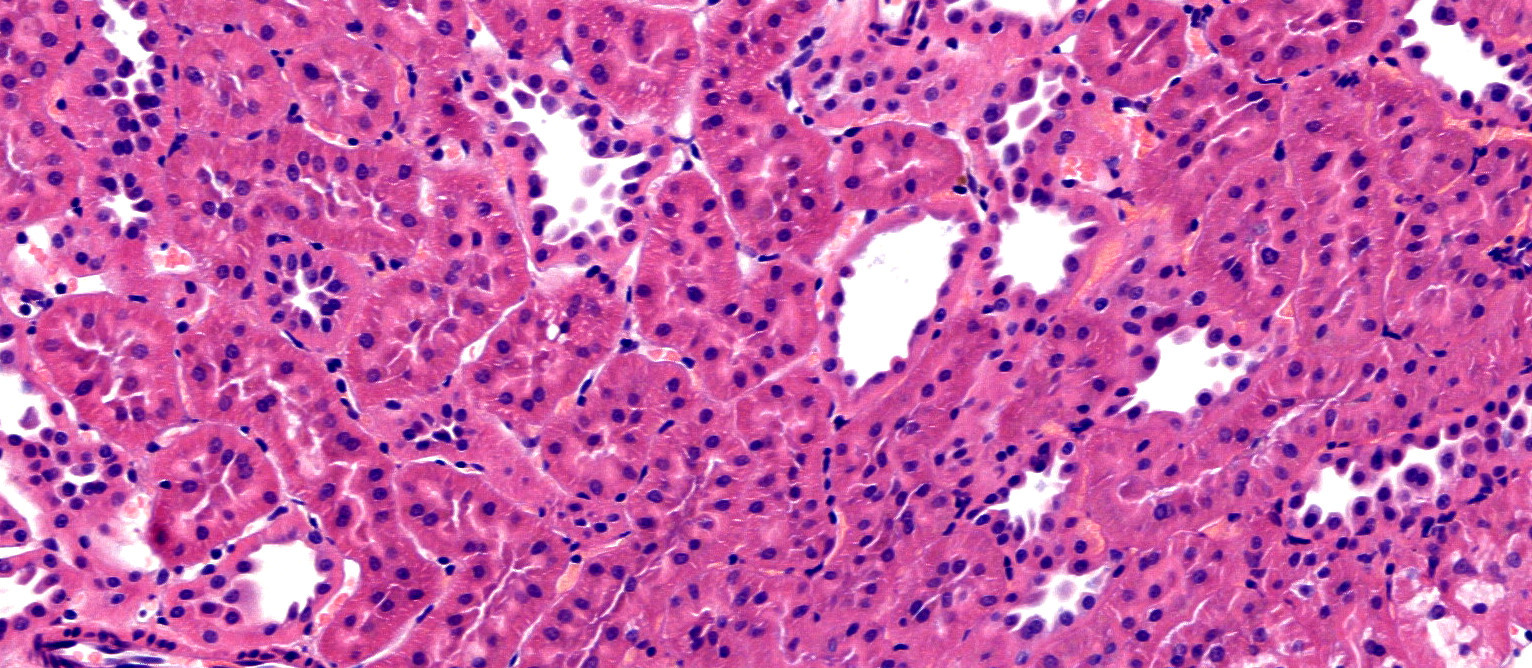

Supplement: Supplementary file 14 [file DataSheet5.ZIP › Fig 1D-HE-TSF-63(1)/63-4.jpeg]

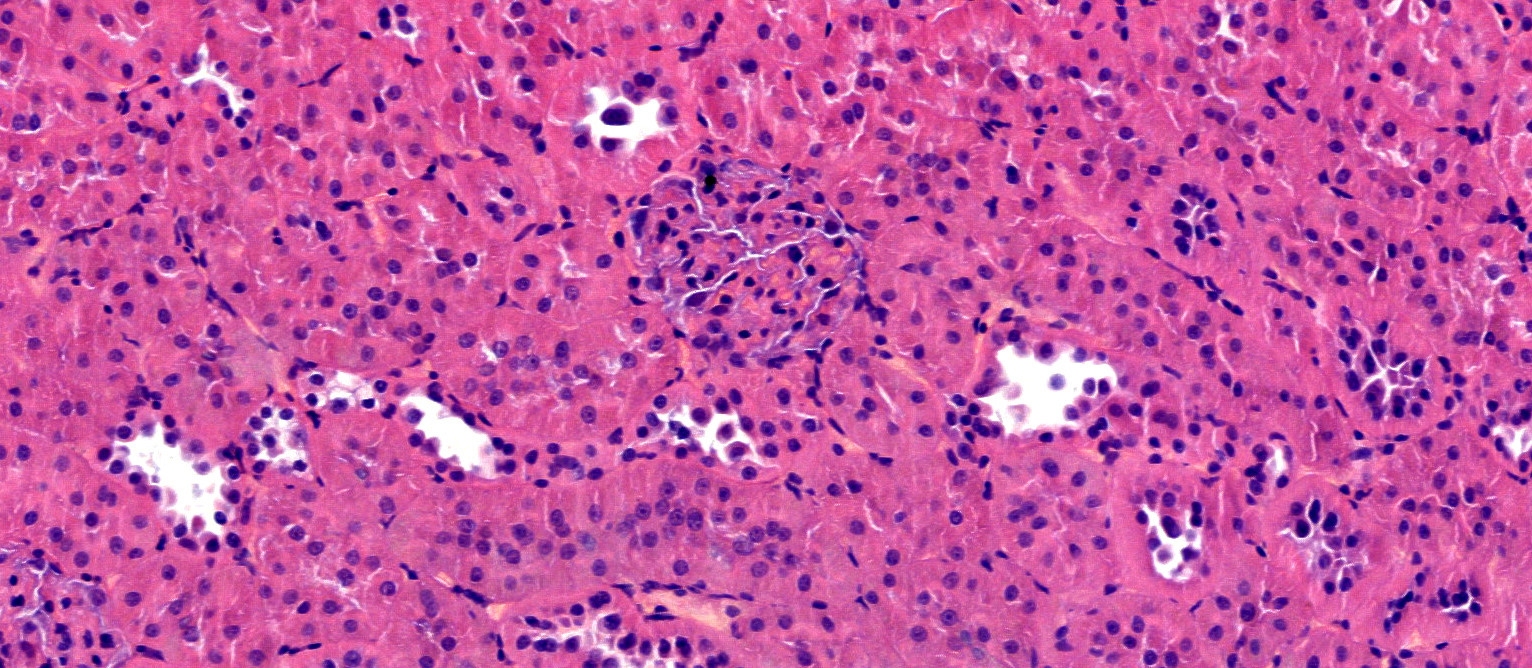

Supplement: Supplementary file 14 [file DataSheet5.ZIP › Fig 1D-HE-TSF-63(1)/63-5.jpeg]

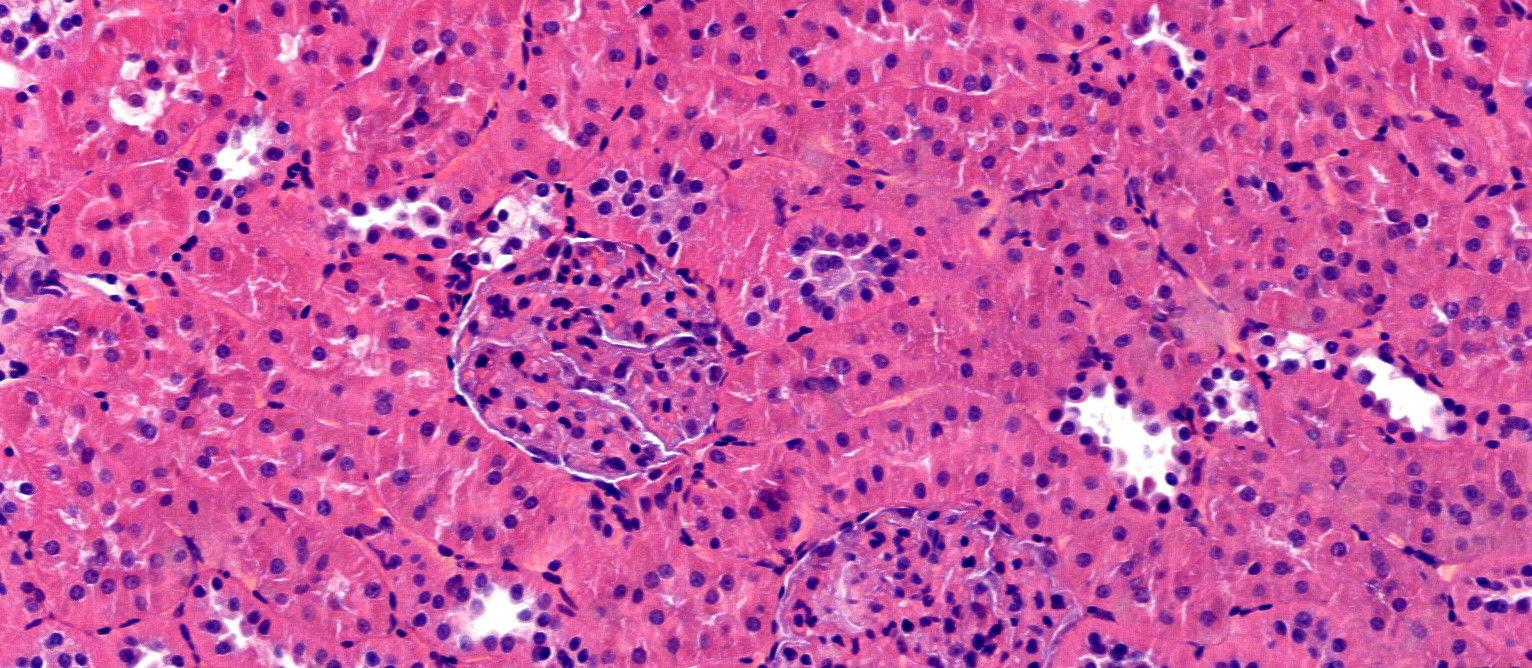

Supplement: Supplementary file 14 [file DataSheet5.ZIP › Fig 1D-HE-TSF-63(1)/63-6.jpeg]

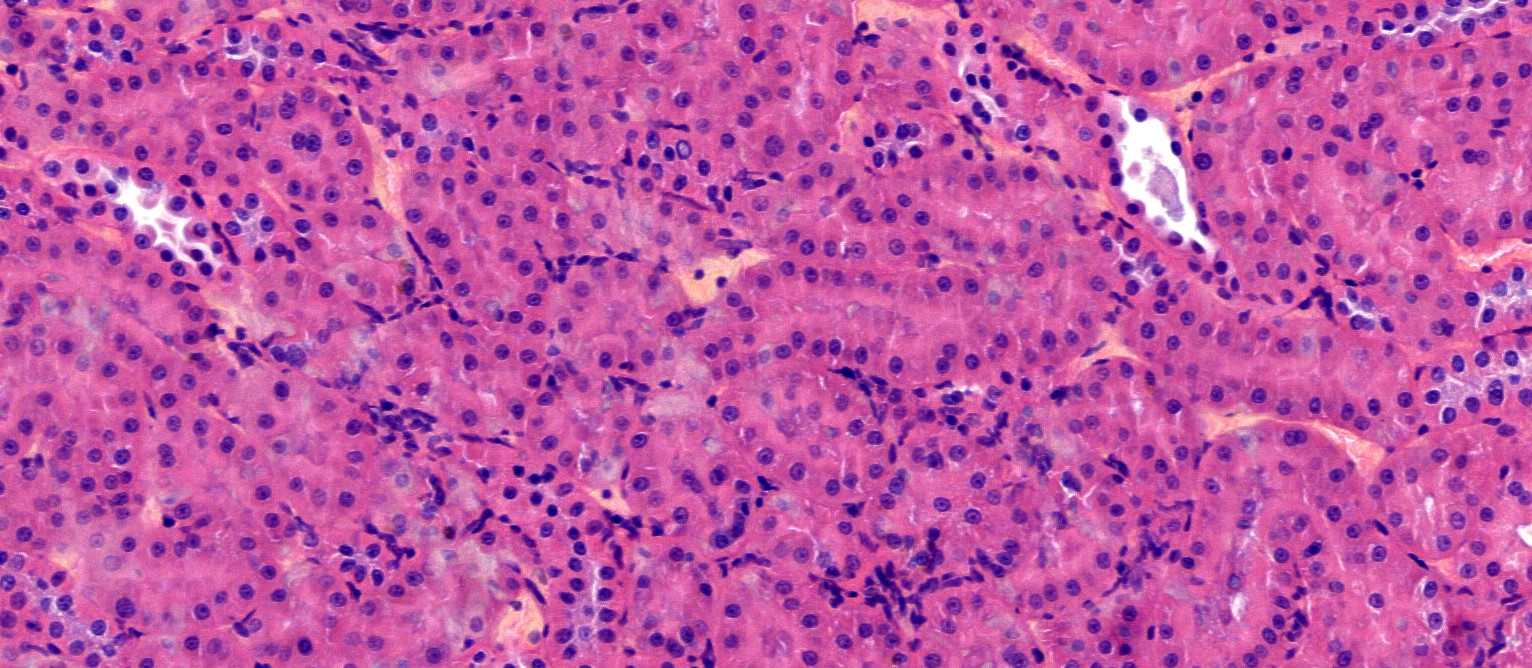

Supplement: Supplementary file 14 [file DataSheet5.ZIP › Fig 1D-HE-TSF-63(1)/63-7.jpeg]

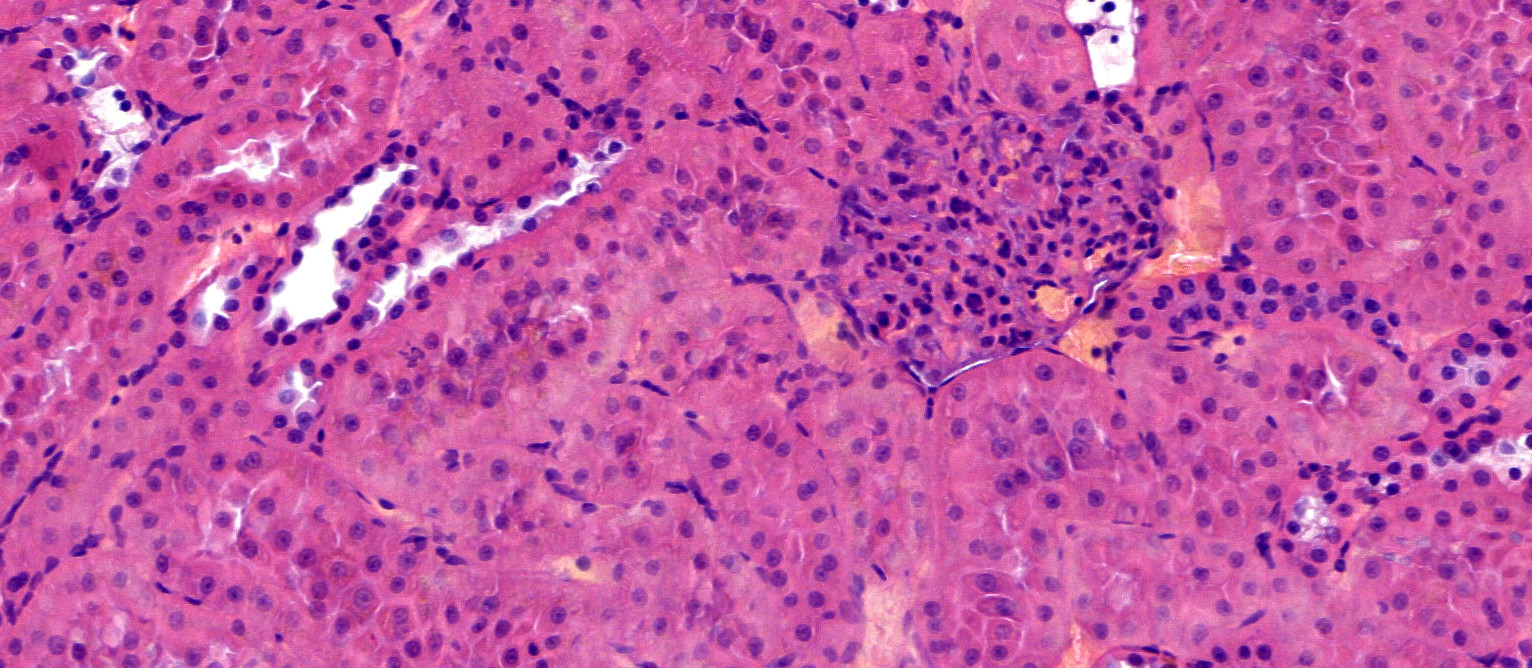

Supplement: Supplementary file 14 [file DataSheet5.ZIP › Fig 1D-HE-TSF-63(1)/63-8.jpeg]

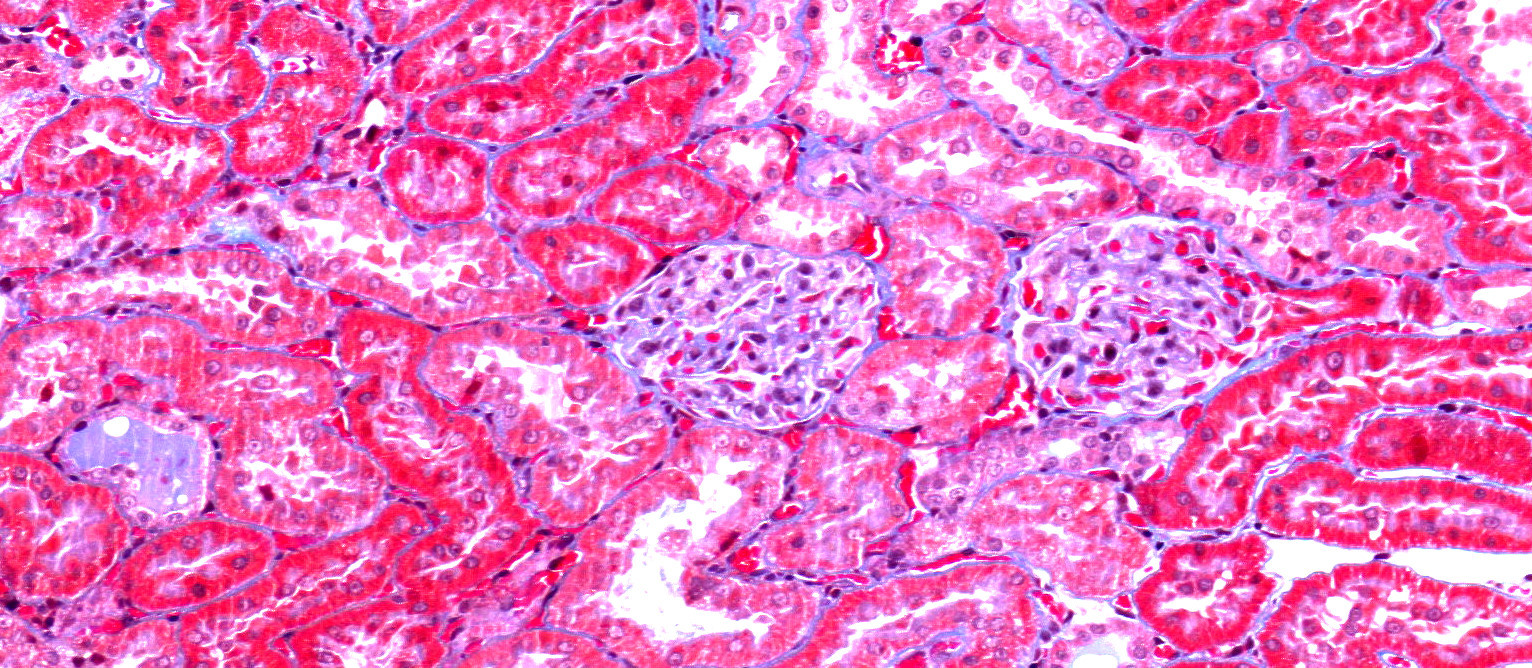

Supplement: Supplementary file 15 [file DataSheet7.ZIP › Fig 1D-masson-DKD-18(2)/18-10.jpeg]

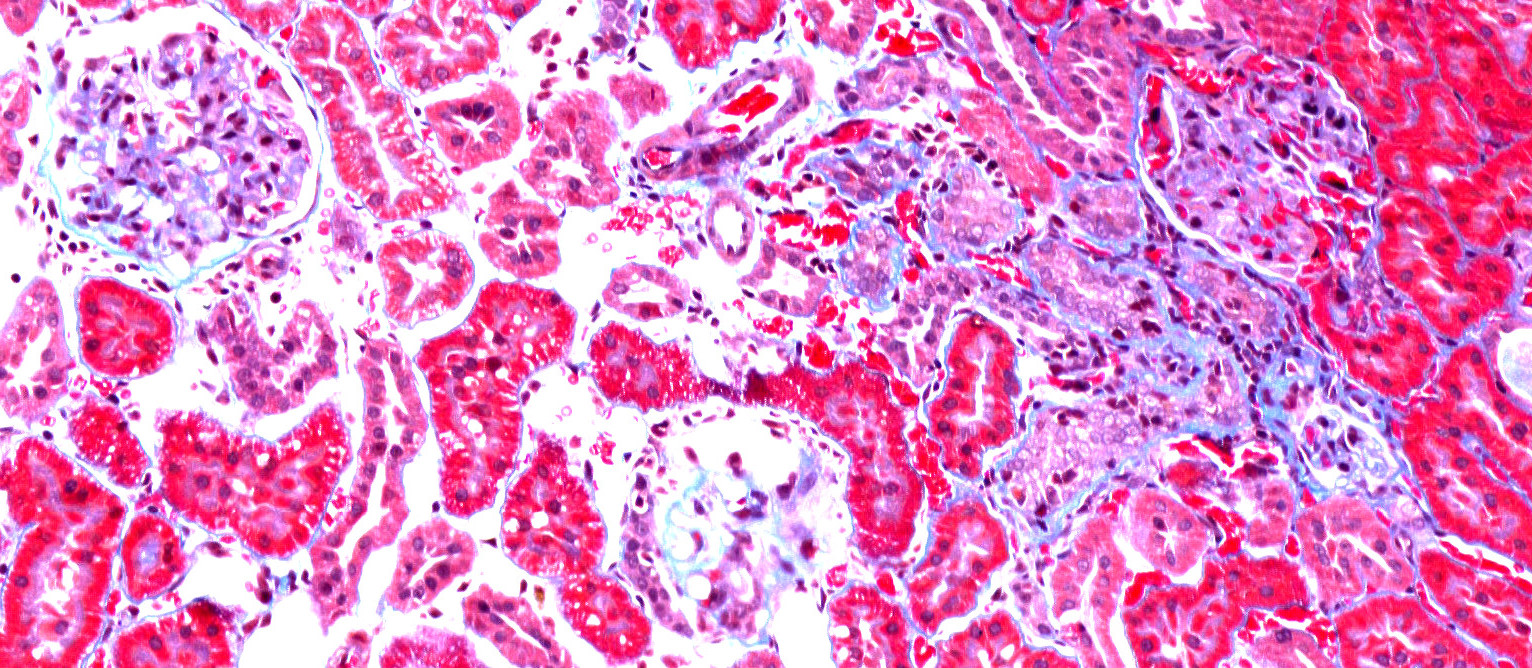

Supplement: Supplementary file 15 [file DataSheet7.ZIP › Fig 1D-masson-DKD-18(2)/18-3.jpeg]

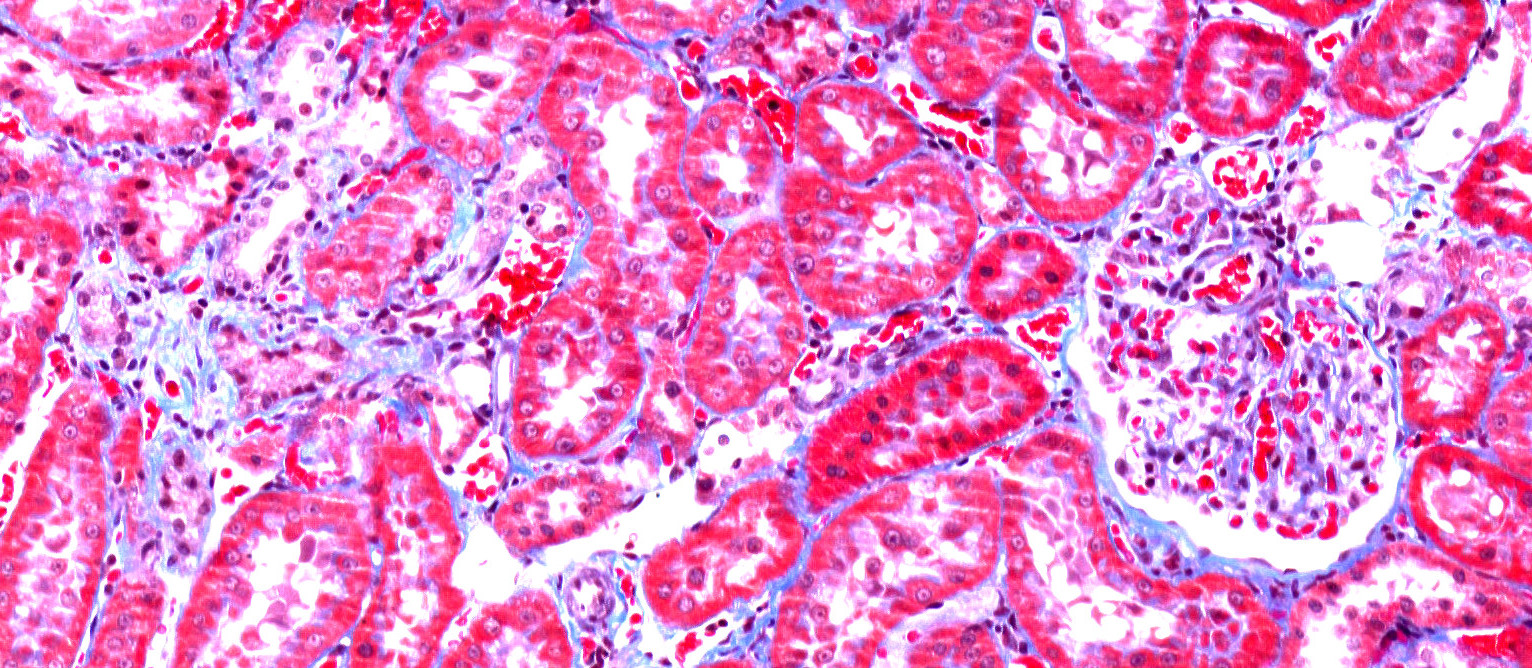

Supplement: Supplementary file 15 [file DataSheet7.ZIP › Fig 1D-masson-DKD-18(2)/18-4.jpeg]

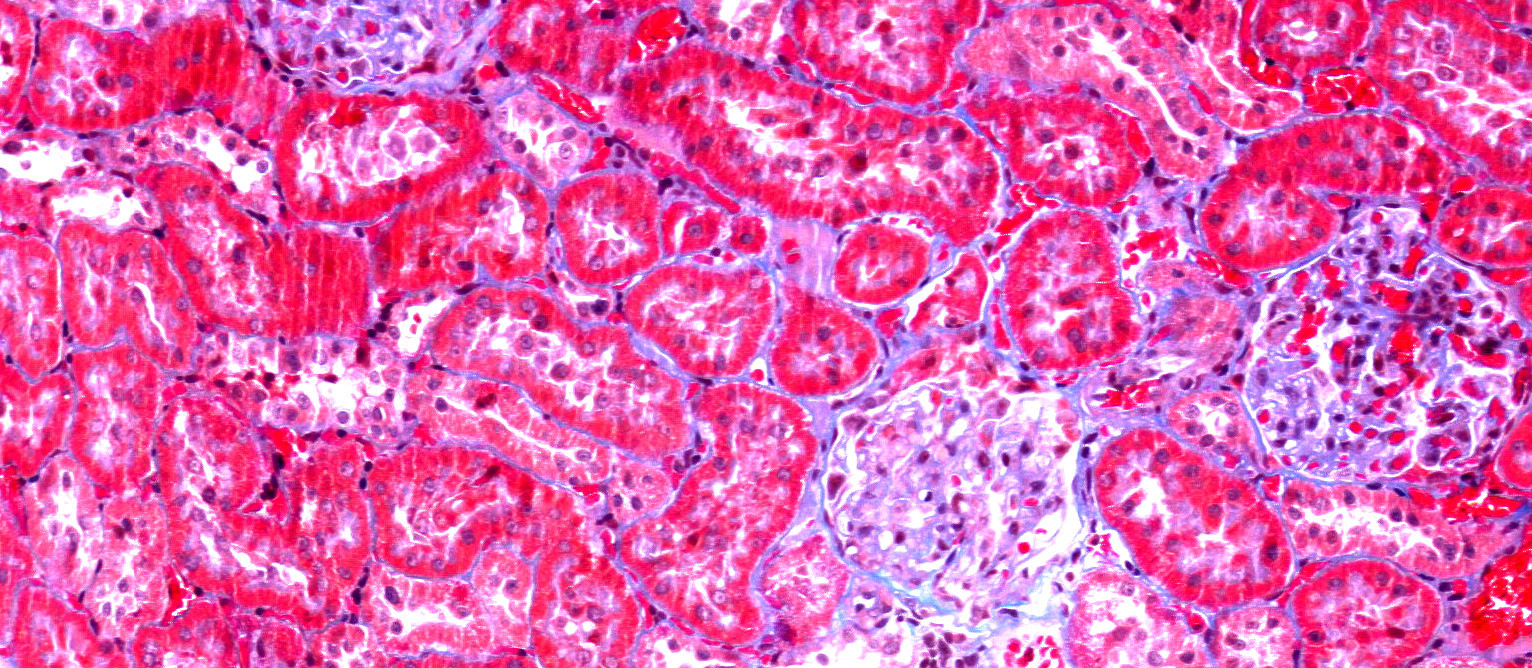

Supplement: Supplementary file 15 [file DataSheet7.ZIP › Fig 1D-masson-DKD-18(2)/18-5.jpeg]

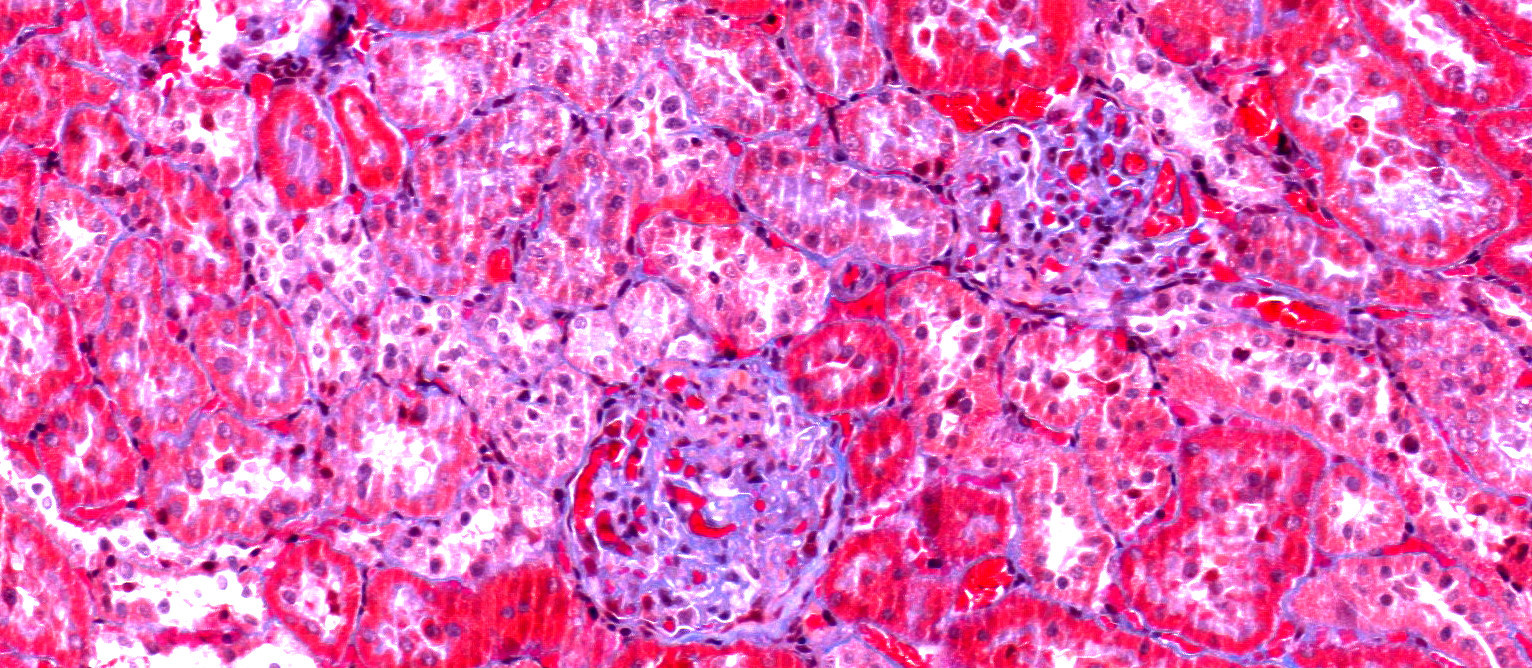

Supplement: Supplementary file 15 [file DataSheet7.ZIP › Fig 1D-masson-DKD-18(2)/18-6.jpeg]

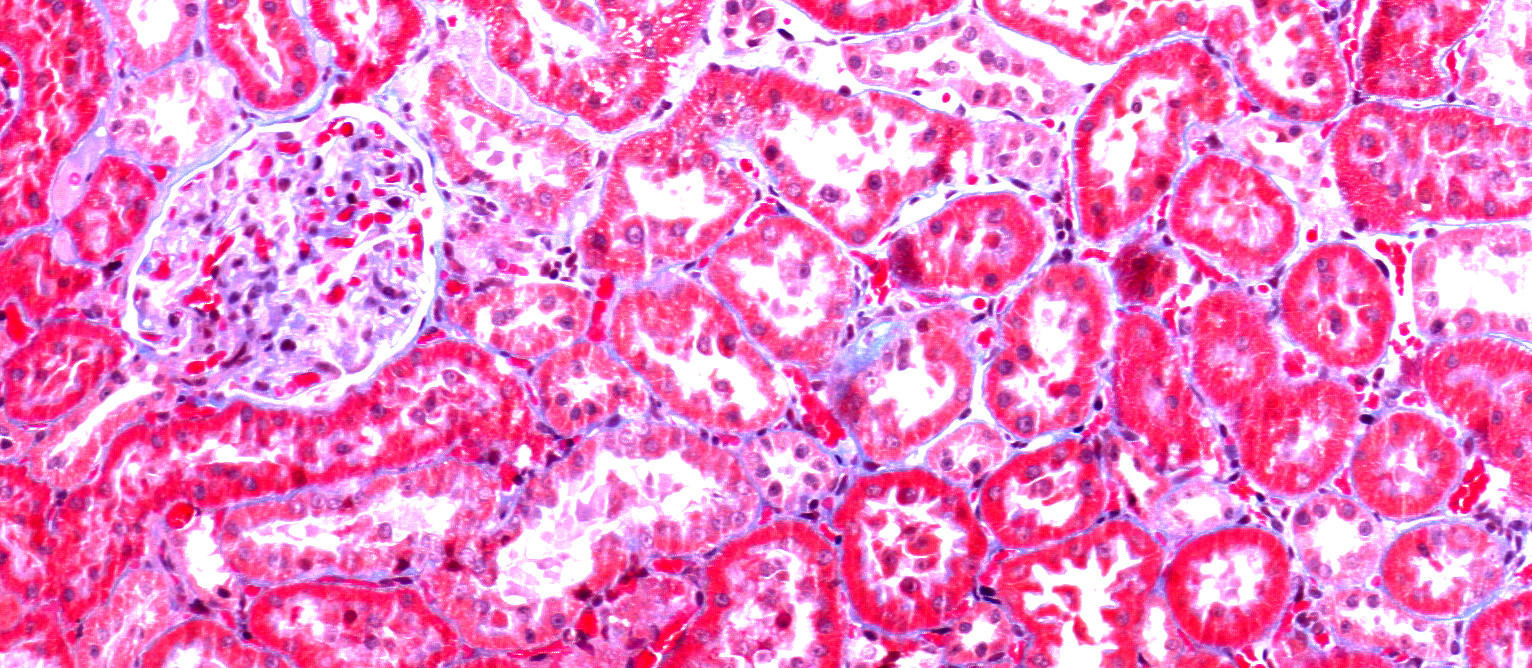

Supplement: Supplementary file 15 [file DataSheet7.ZIP › Fig 1D-masson-DKD-18(2)/18-7.jpeg]

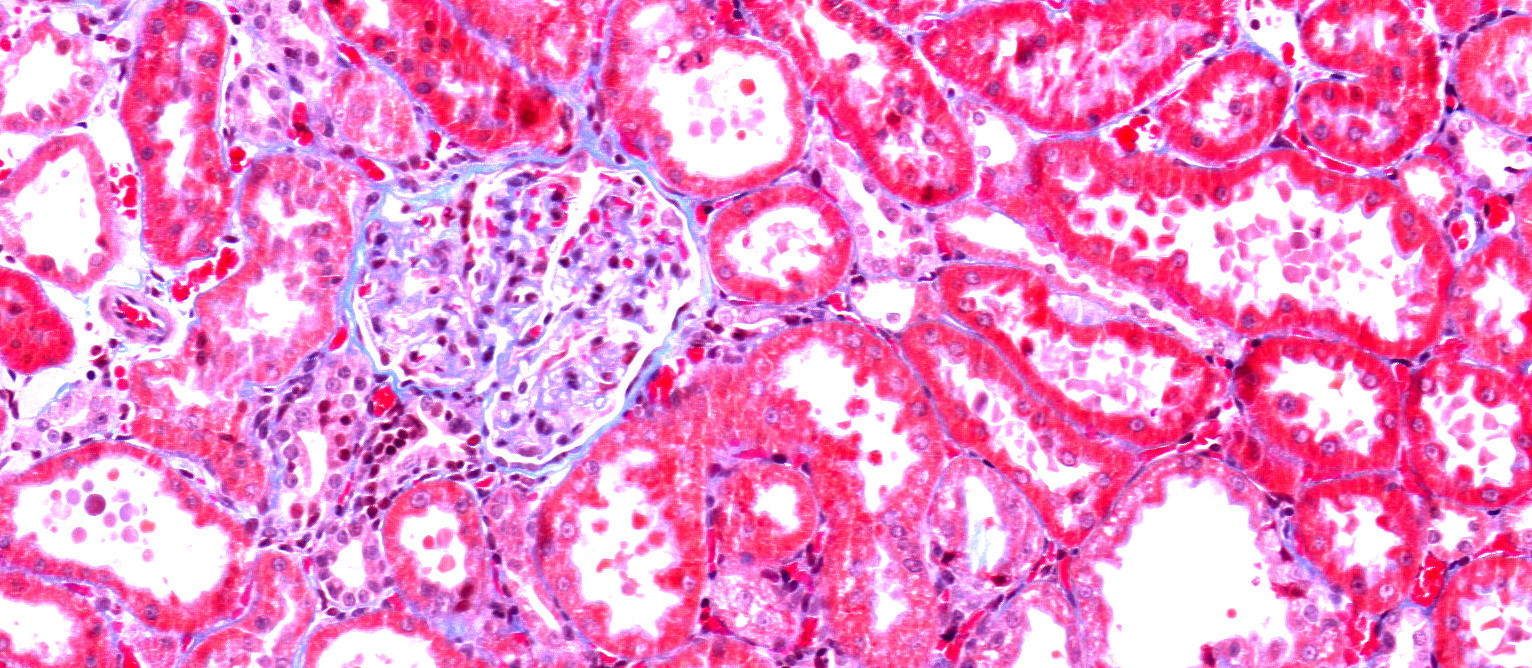

Supplement: Supplementary file 15 [file DataSheet7.ZIP › Fig 1D-masson-DKD-18(2)/18-8.jpeg]

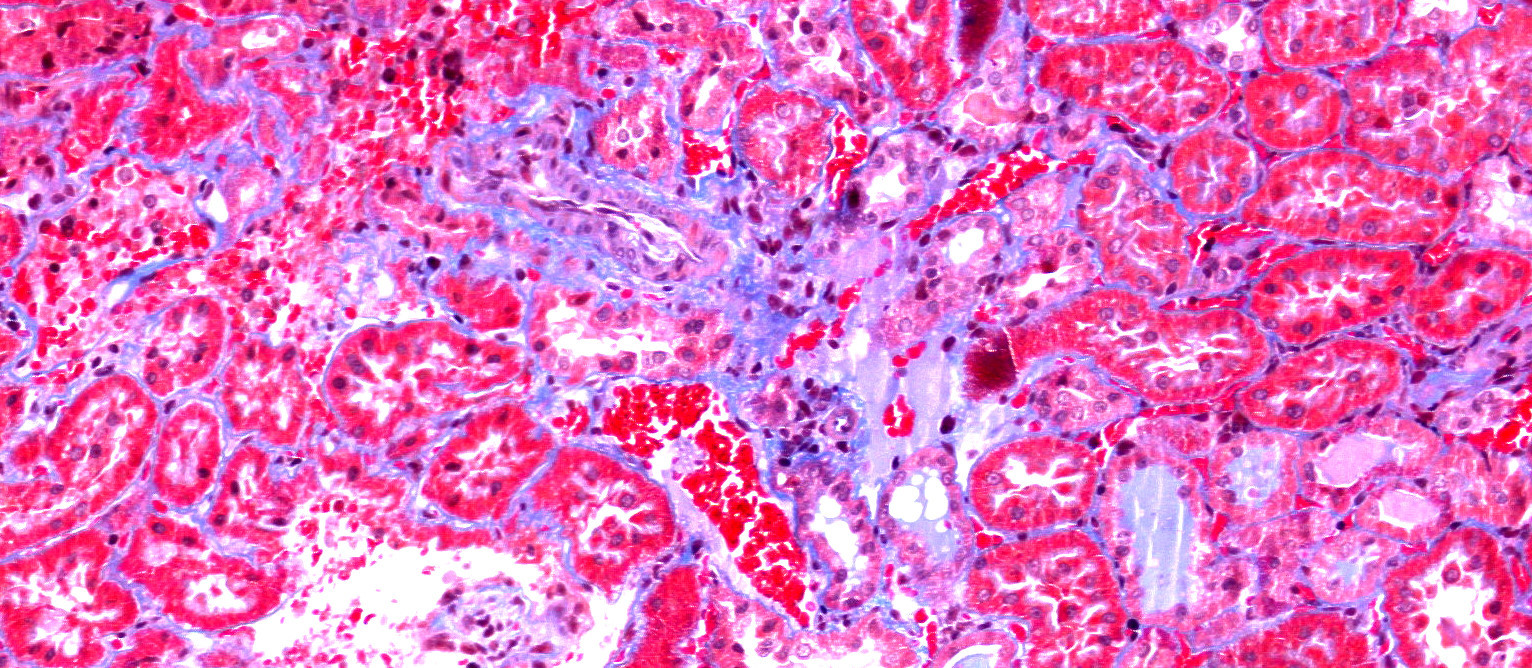

Supplement: Supplementary file 15 [file DataSheet7.ZIP › Fig 1D-masson-DKD-18(2)/18-9.jpeg]

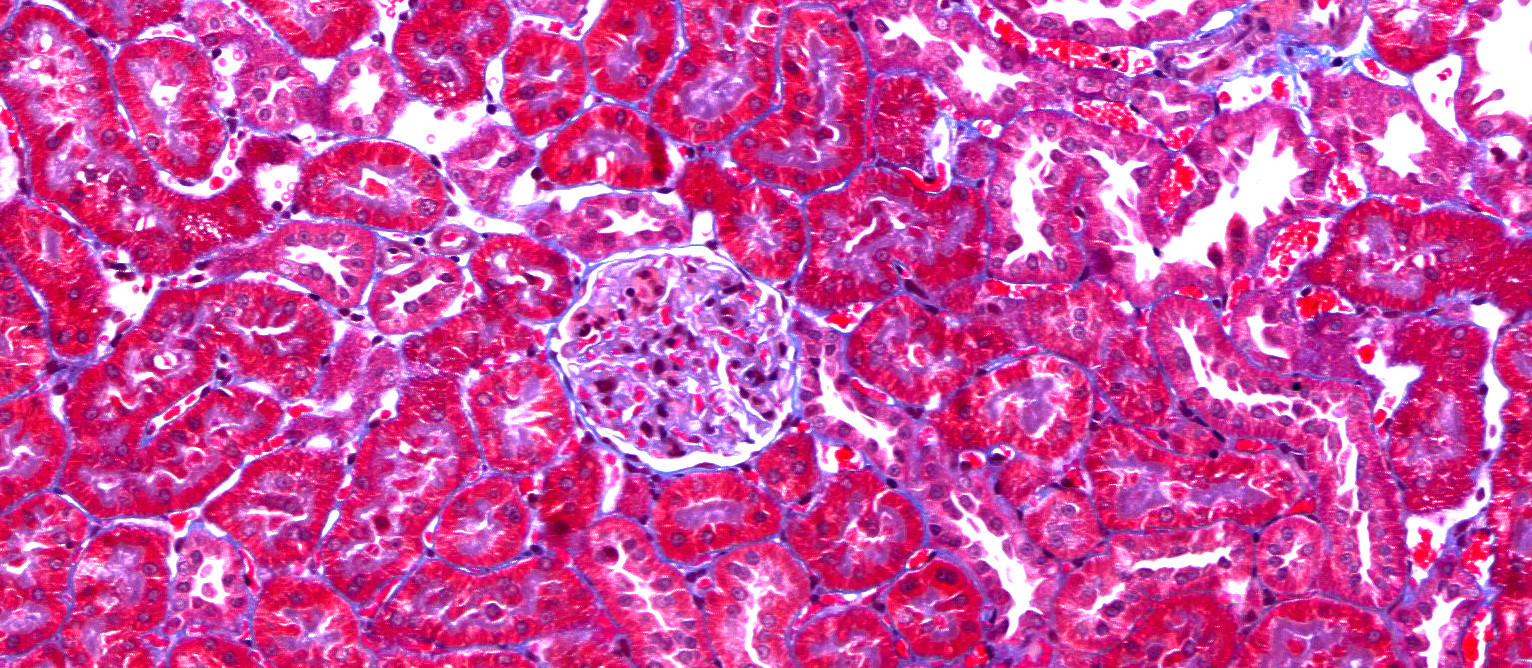

Supplement: Supplementary file 15 [file DataSheet7.ZIP › Fig 1D-masson-DKD-20/20-1.jpeg]

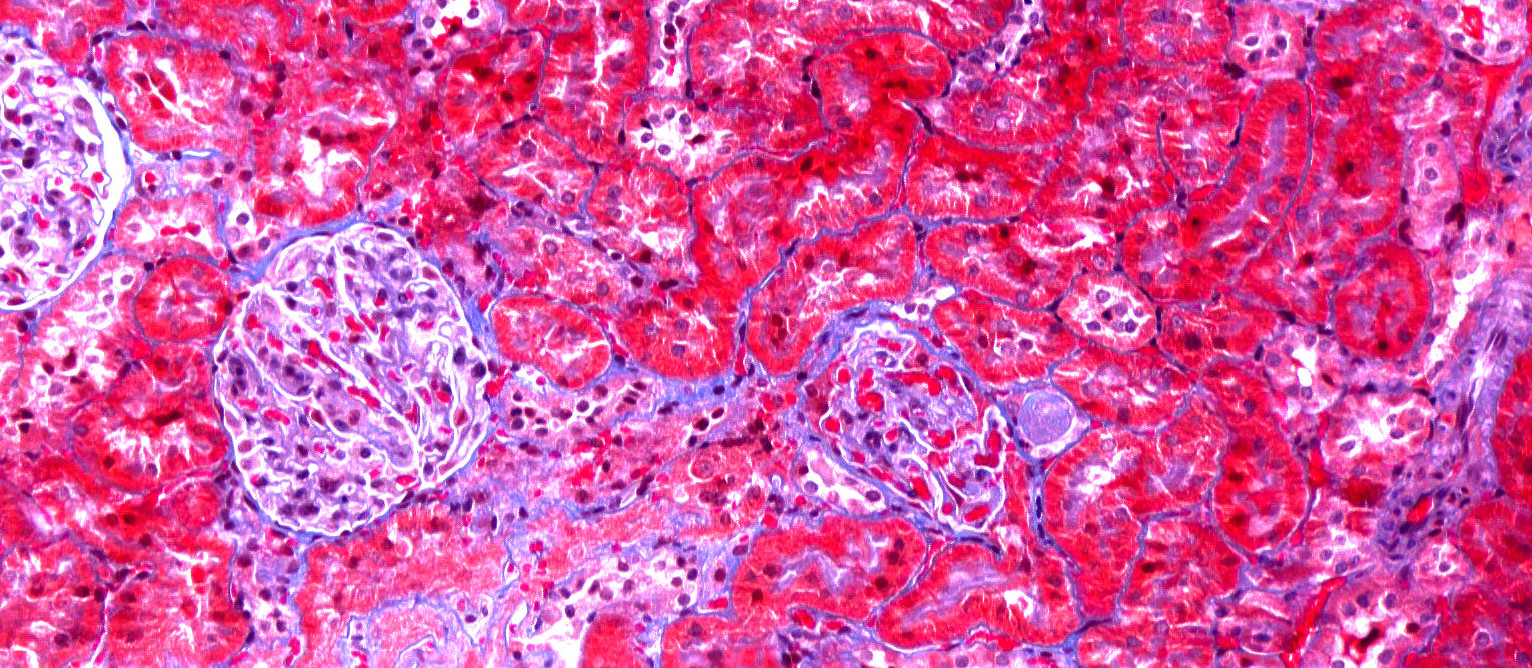

Supplement: Supplementary file 15 [file DataSheet7.ZIP › Fig 1D-masson-DKD-20/20-10.jpeg]

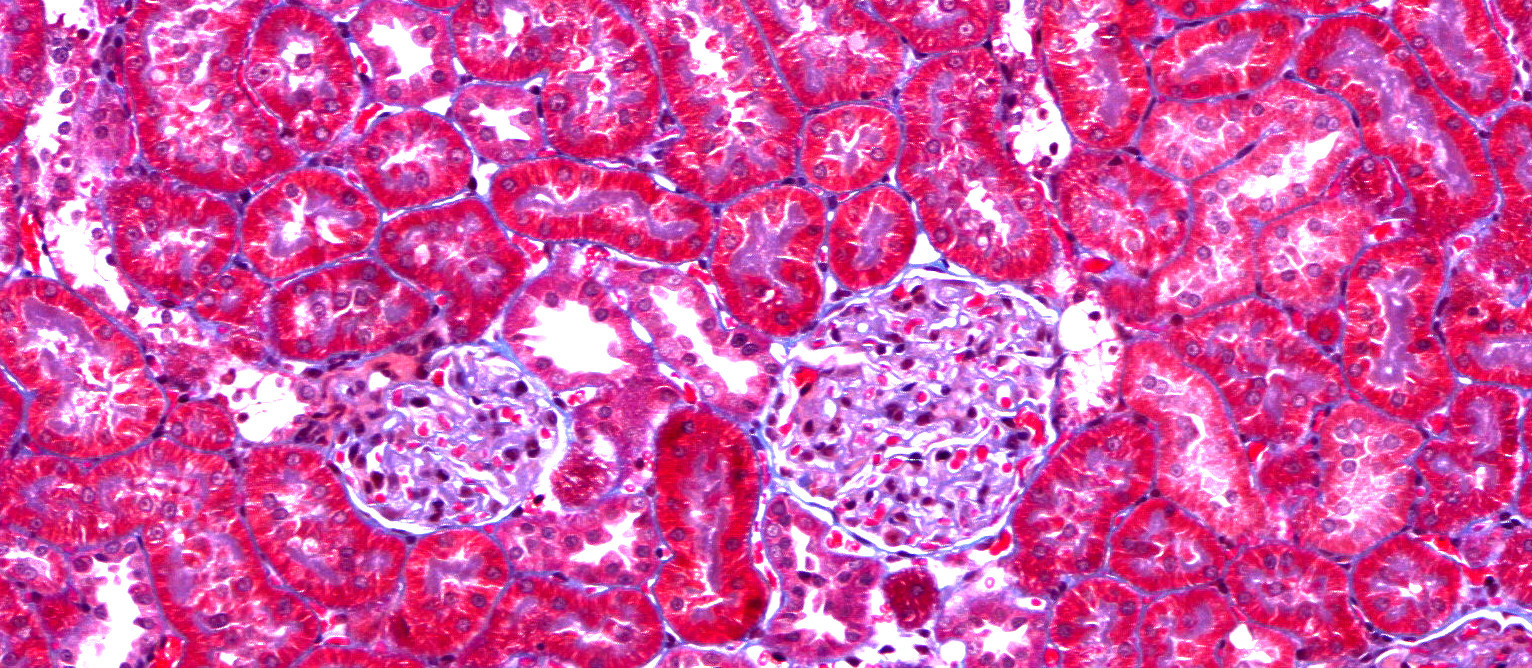

Supplement: Supplementary file 15 [file DataSheet7.ZIP › Fig 1D-masson-DKD-20/20-2.jpeg]

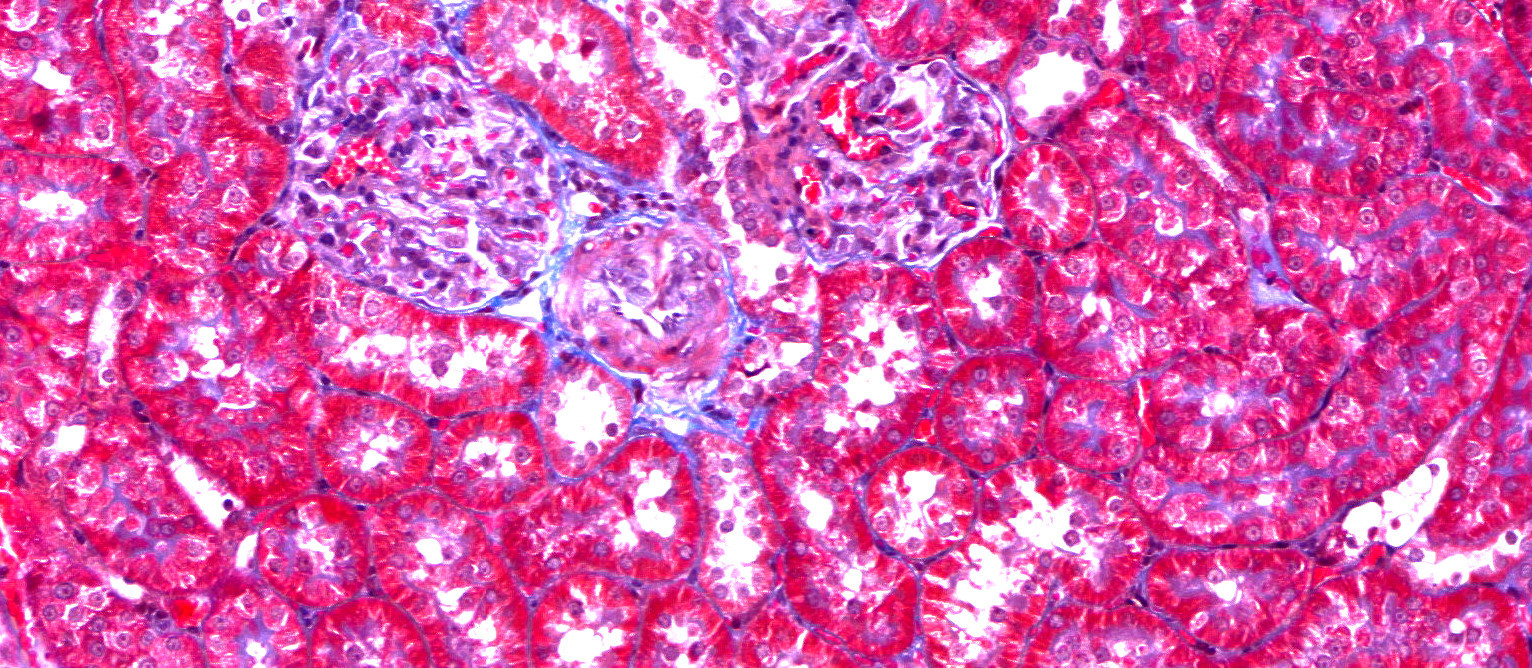

Supplement: Supplementary file 15 [file DataSheet7.ZIP › Fig 1D-masson-DKD-20/20-3.jpeg]

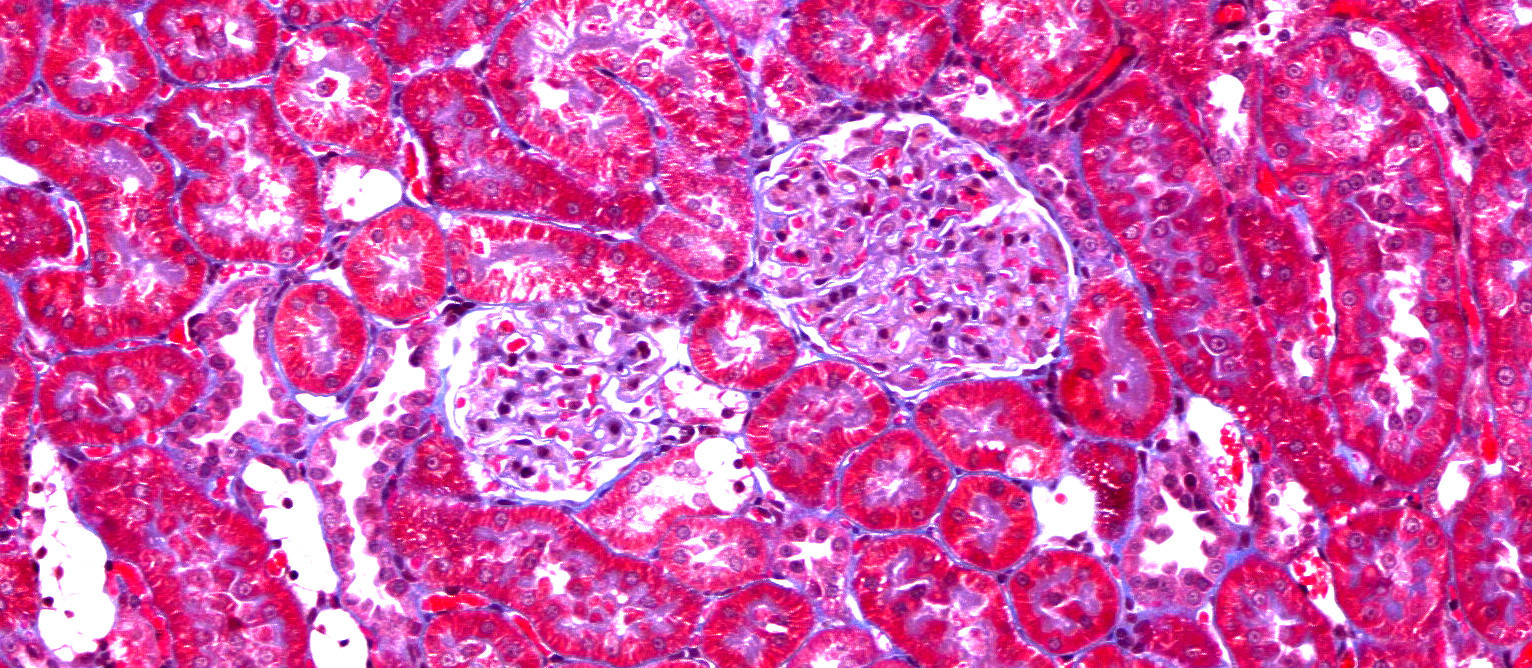

Supplement: Supplementary file 15 [file DataSheet7.ZIP › Fig 1D-masson-DKD-20/20-4.jpeg]

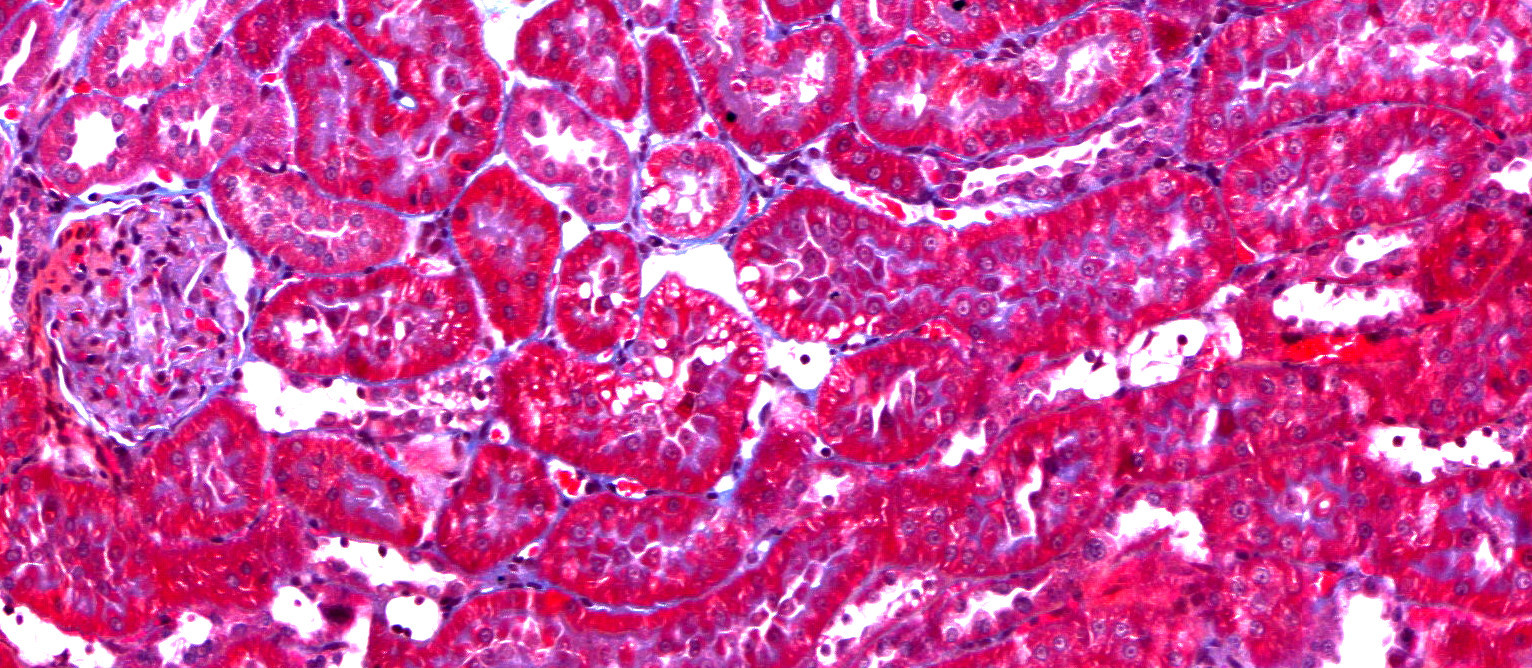

Supplement: Supplementary file 15 [file DataSheet7.ZIP › Fig 1D-masson-DKD-20/20-5.jpeg]

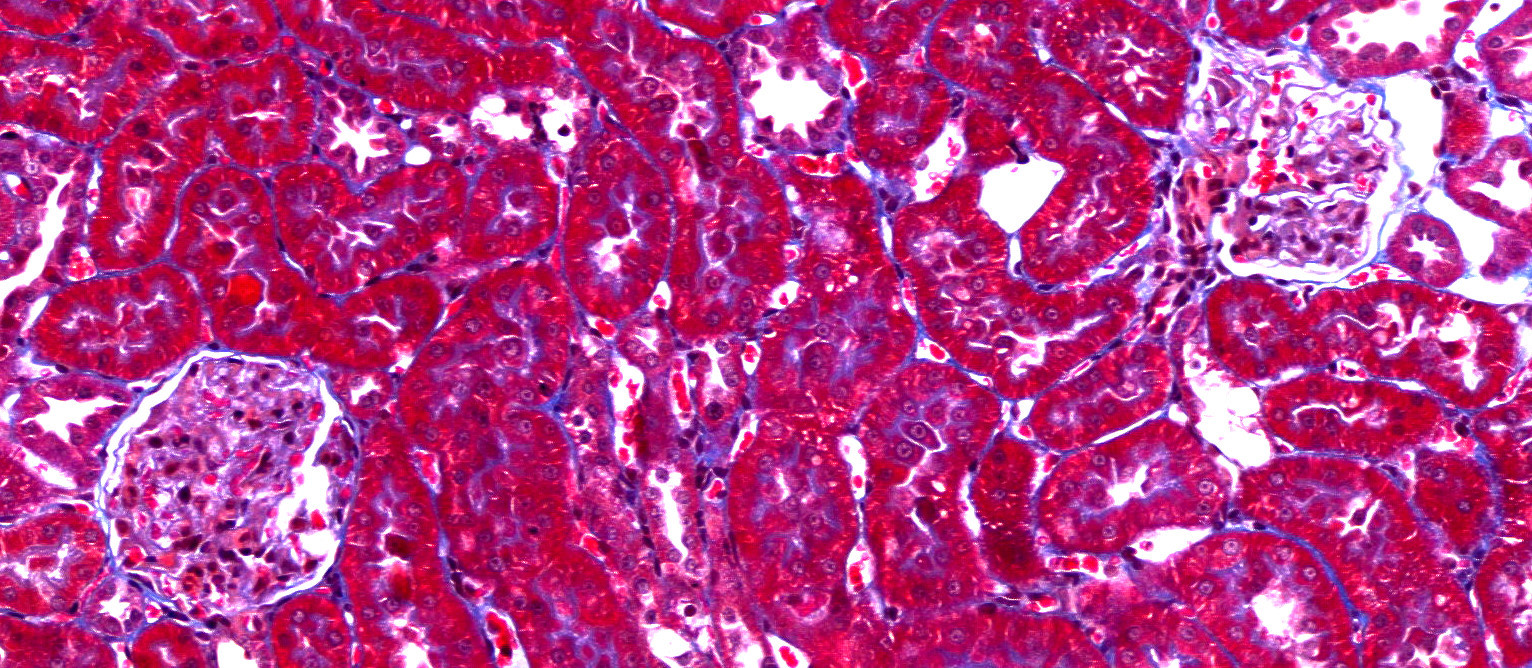

Supplement: Supplementary file 15 [file DataSheet7.ZIP › Fig 1D-masson-DKD-20/20-6.jpeg]

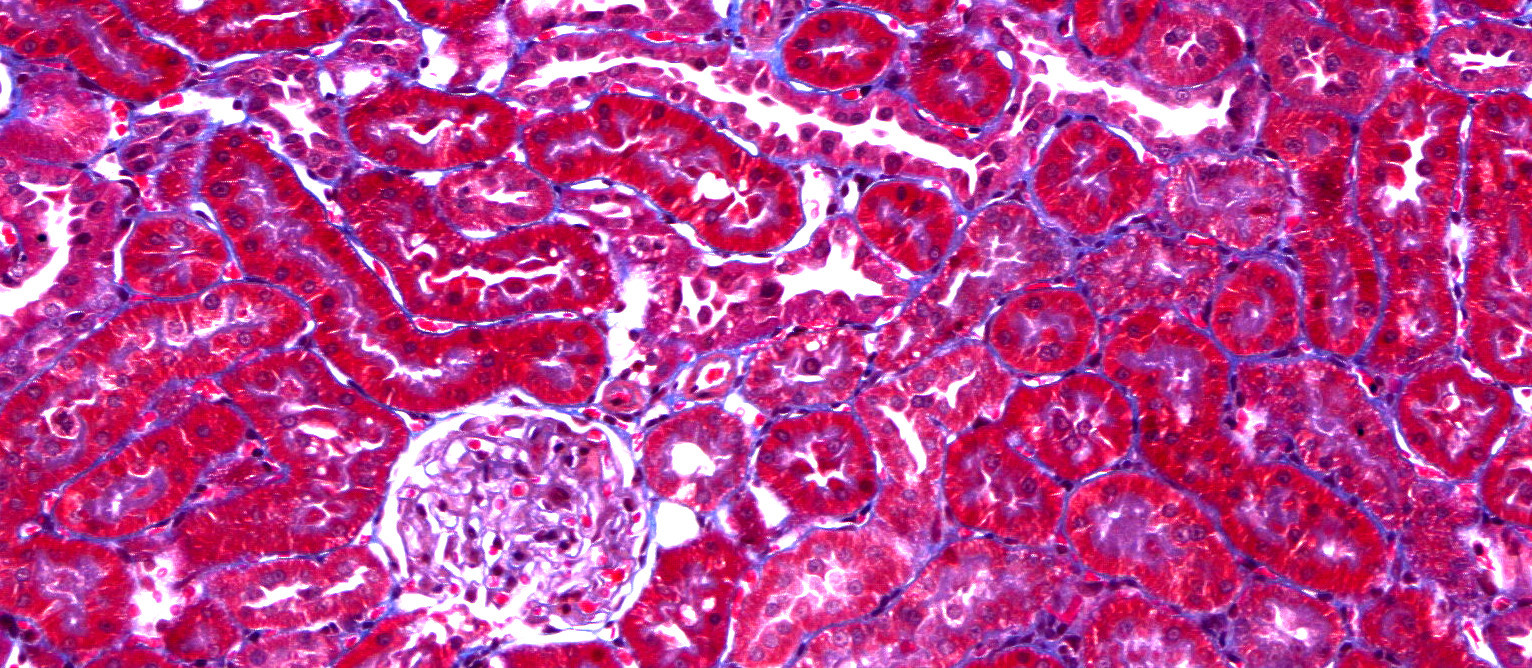

Supplement: Supplementary file 15 [file DataSheet7.ZIP › Fig 1D-masson-DKD-20/20-7.jpeg]

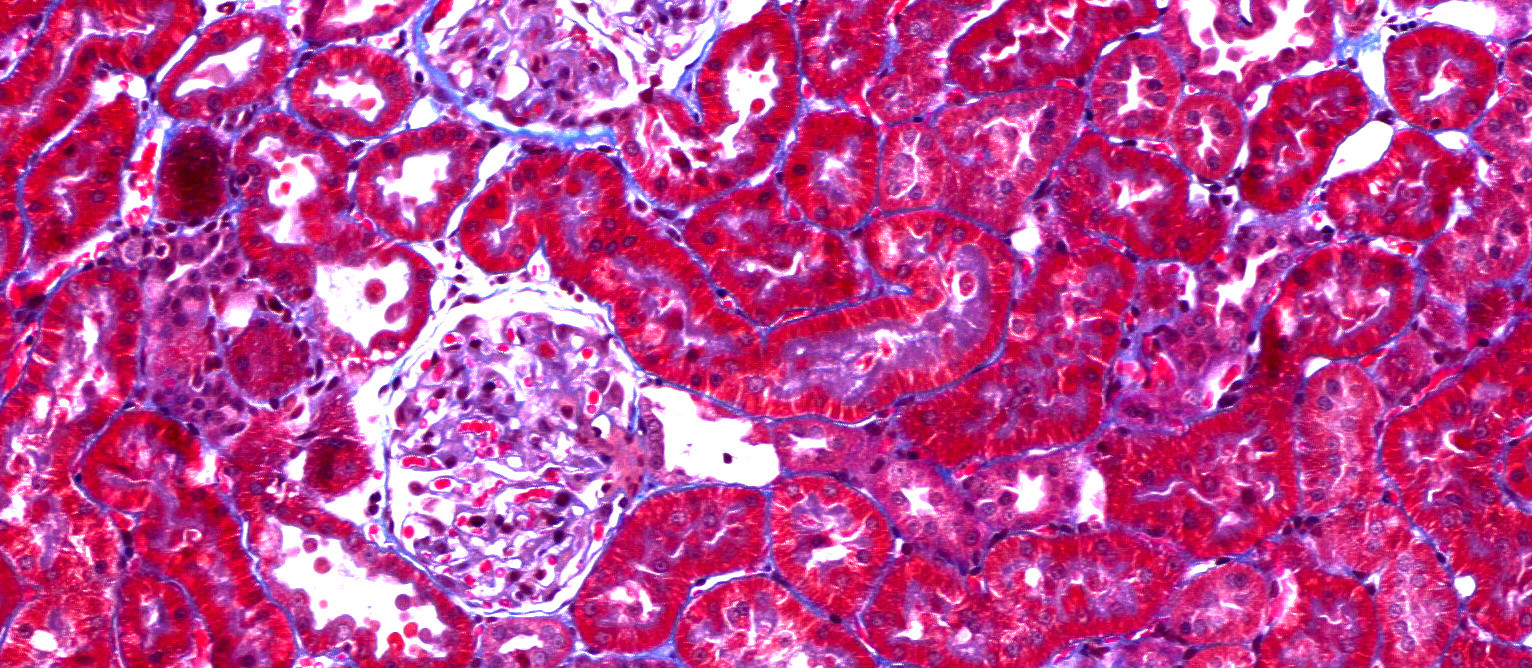

Supplement: Supplementary file 15 [file DataSheet7.ZIP › Fig 1D-masson-DKD-20/20-8.jpeg]

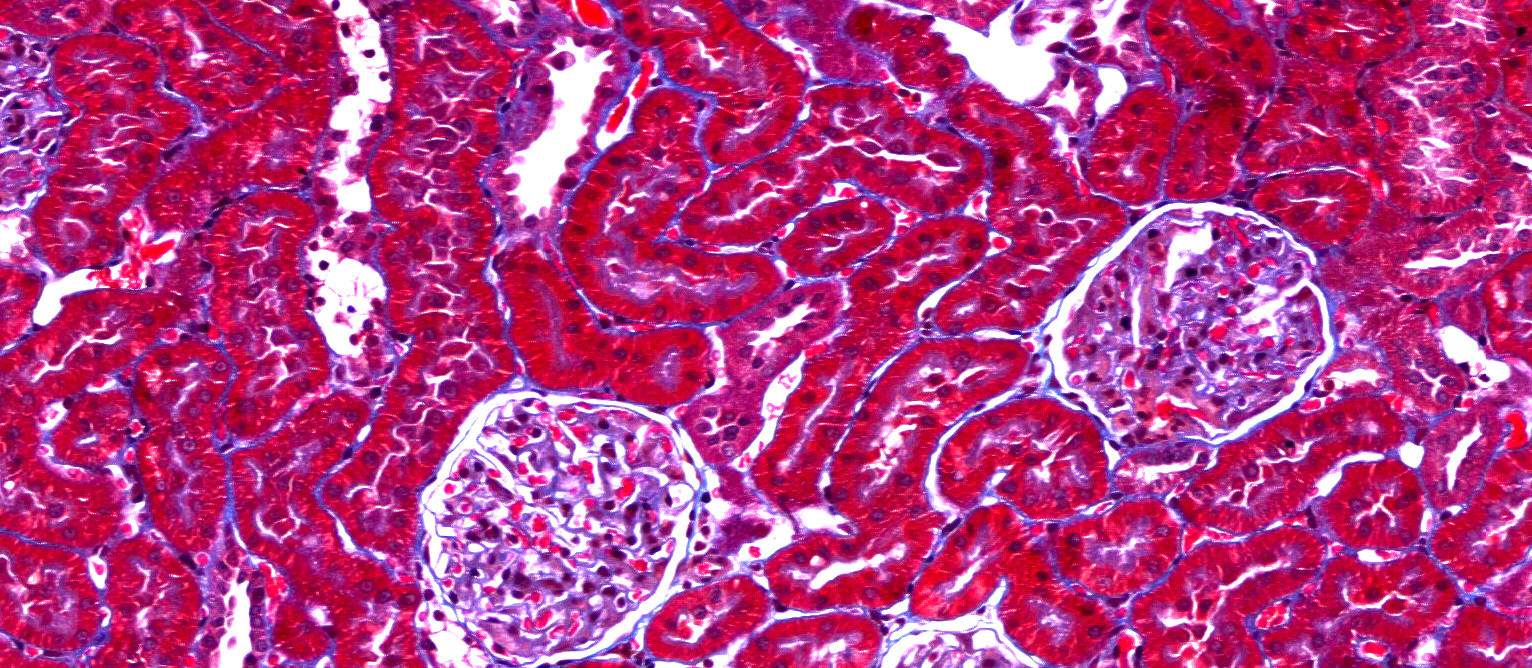

Supplement: Supplementary file 15 [file DataSheet7.ZIP › Fig 1D-masson-DKD-20/20-9.jpeg]

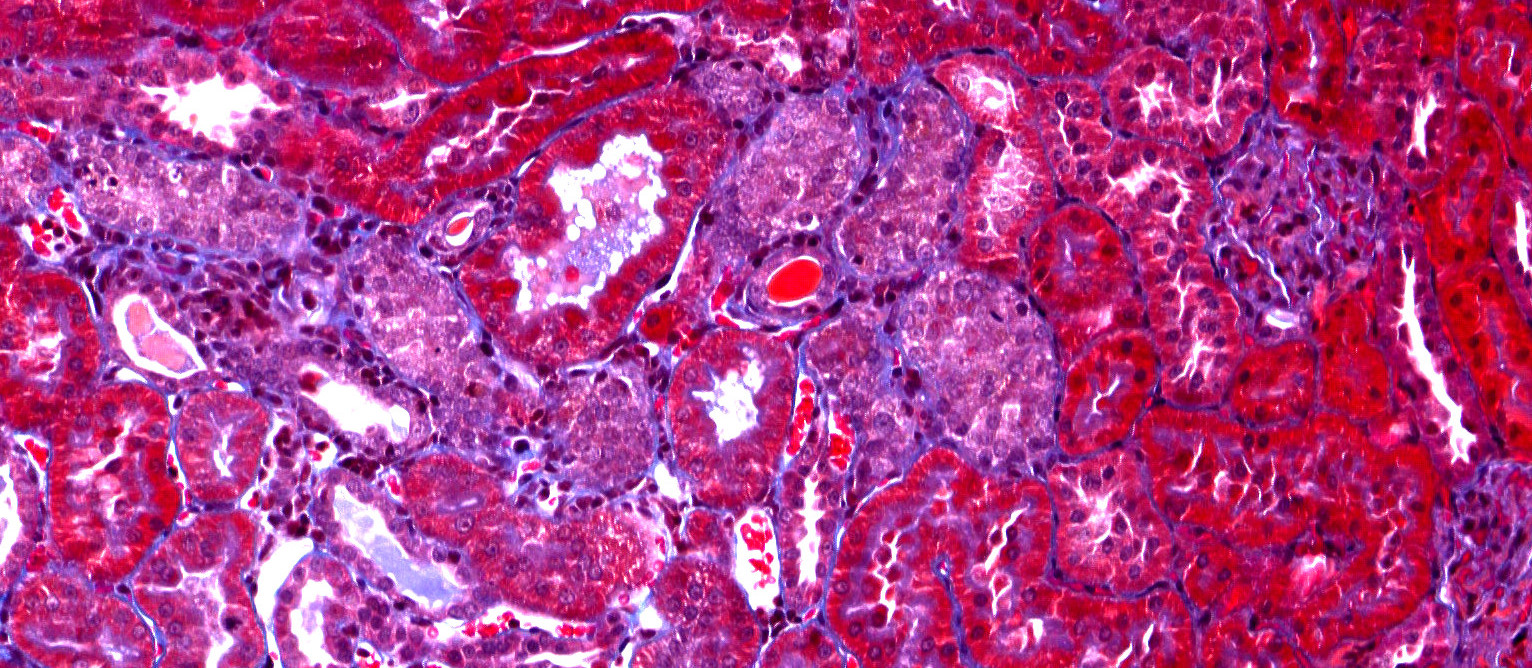

Supplement: Supplementary file 15 [file DataSheet7.ZIP › Fig 1D-masson-DKD-21/21-1.jpeg]

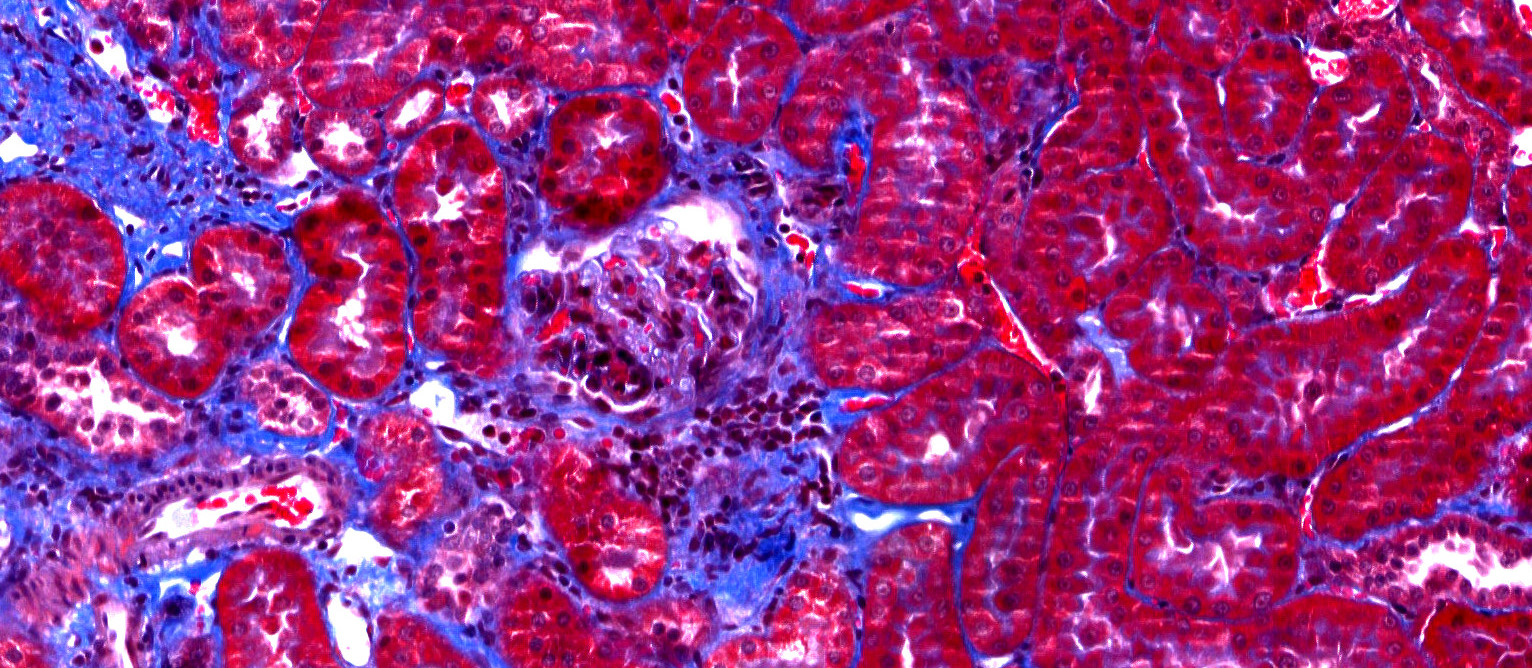

Supplement: Supplementary file 15 [file DataSheet7.ZIP › Fig 1D-masson-DKD-21/21-10.jpeg]

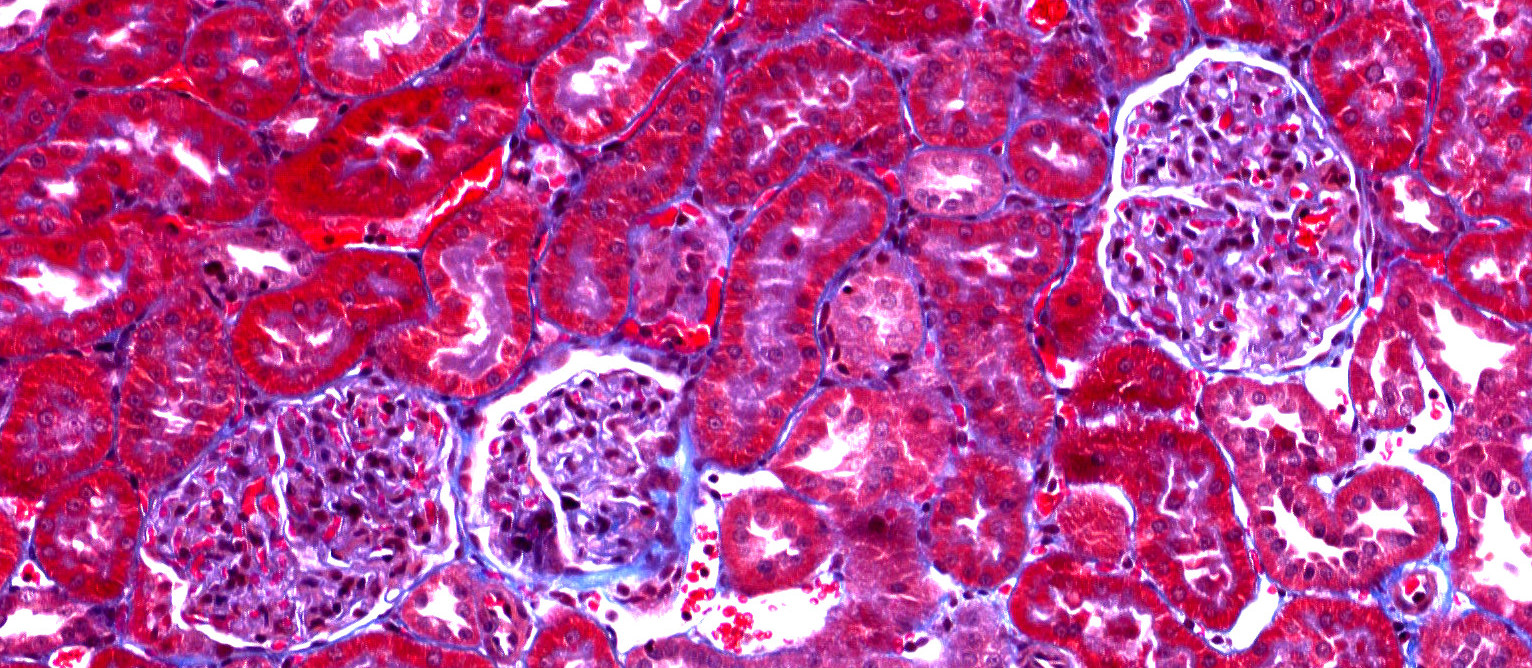

Supplement: Supplementary file 15 [file DataSheet7.ZIP › Fig 1D-masson-DKD-21/21-2.jpeg]

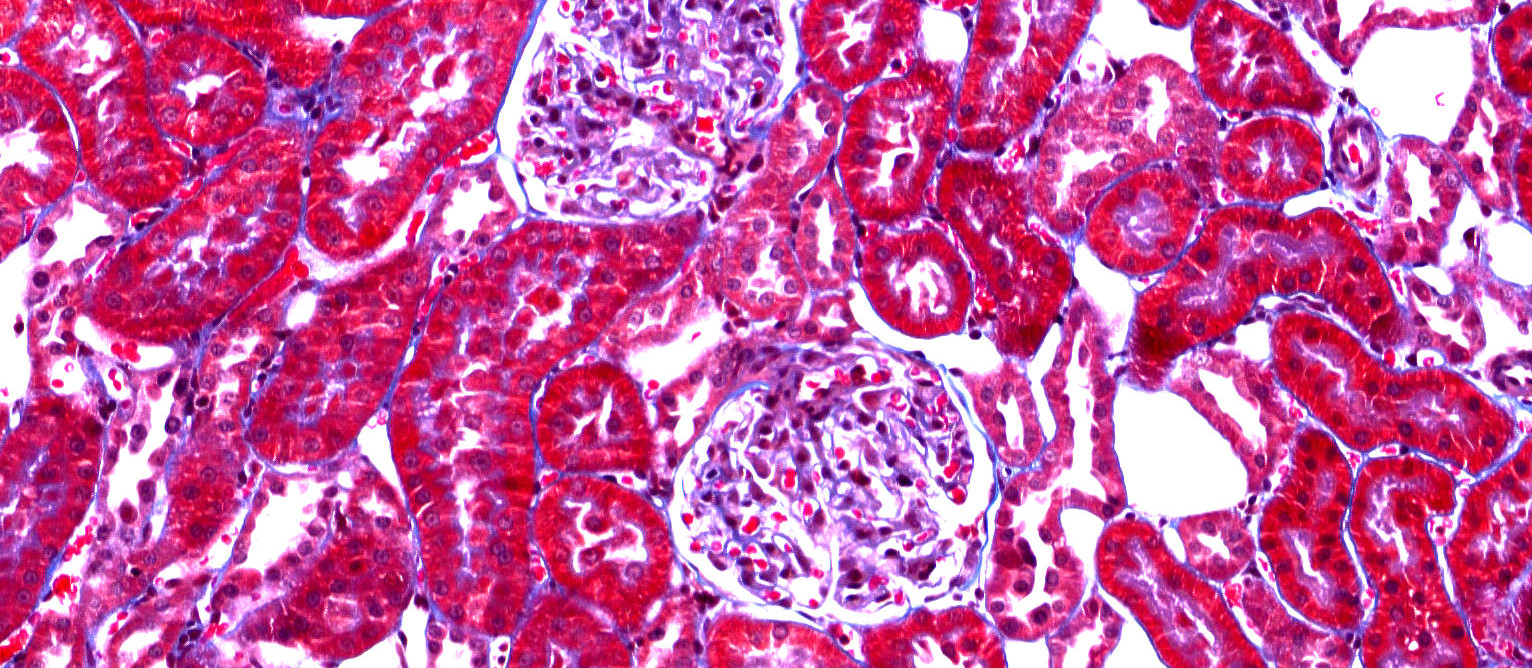

Supplement: Supplementary file 15 [file DataSheet7.ZIP › Fig 1D-masson-DKD-21/21-3.jpeg]

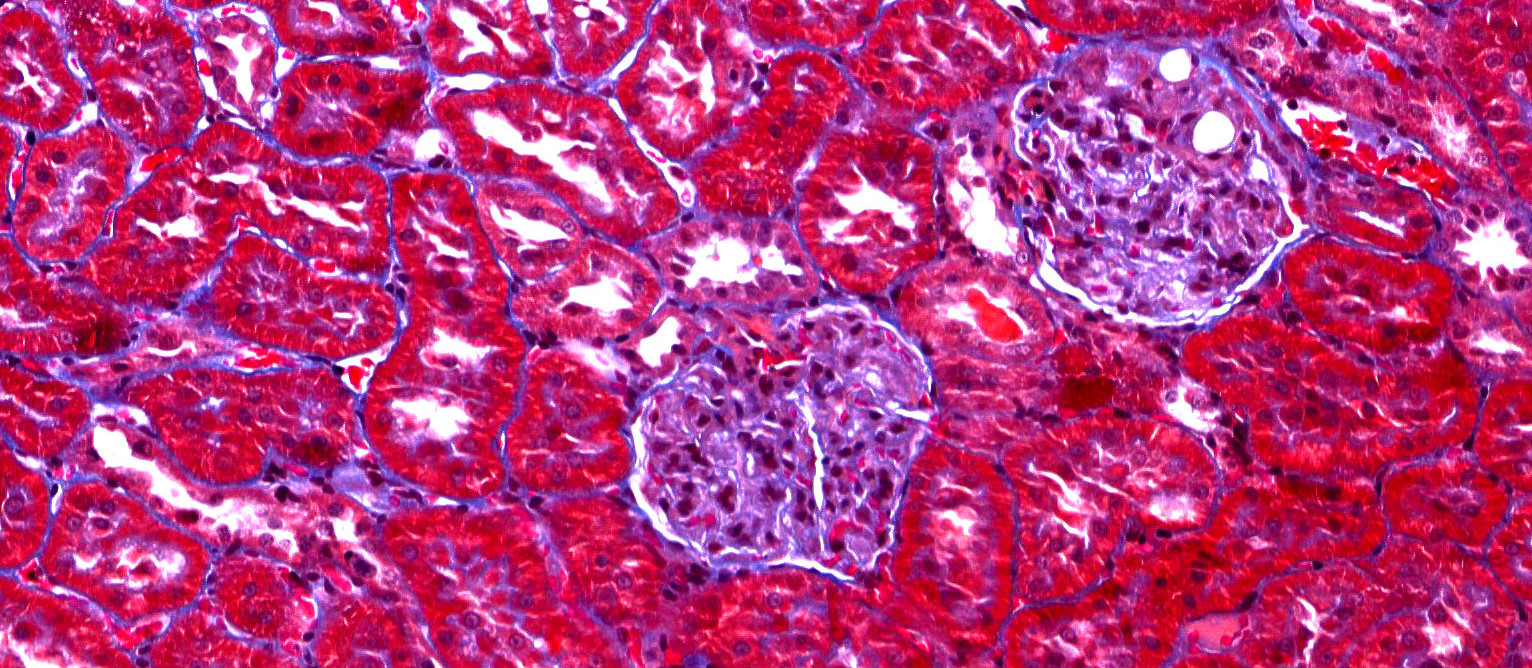

Supplement: Supplementary file 15 [file DataSheet7.ZIP › Fig 1D-masson-DKD-21/21-4.jpeg]

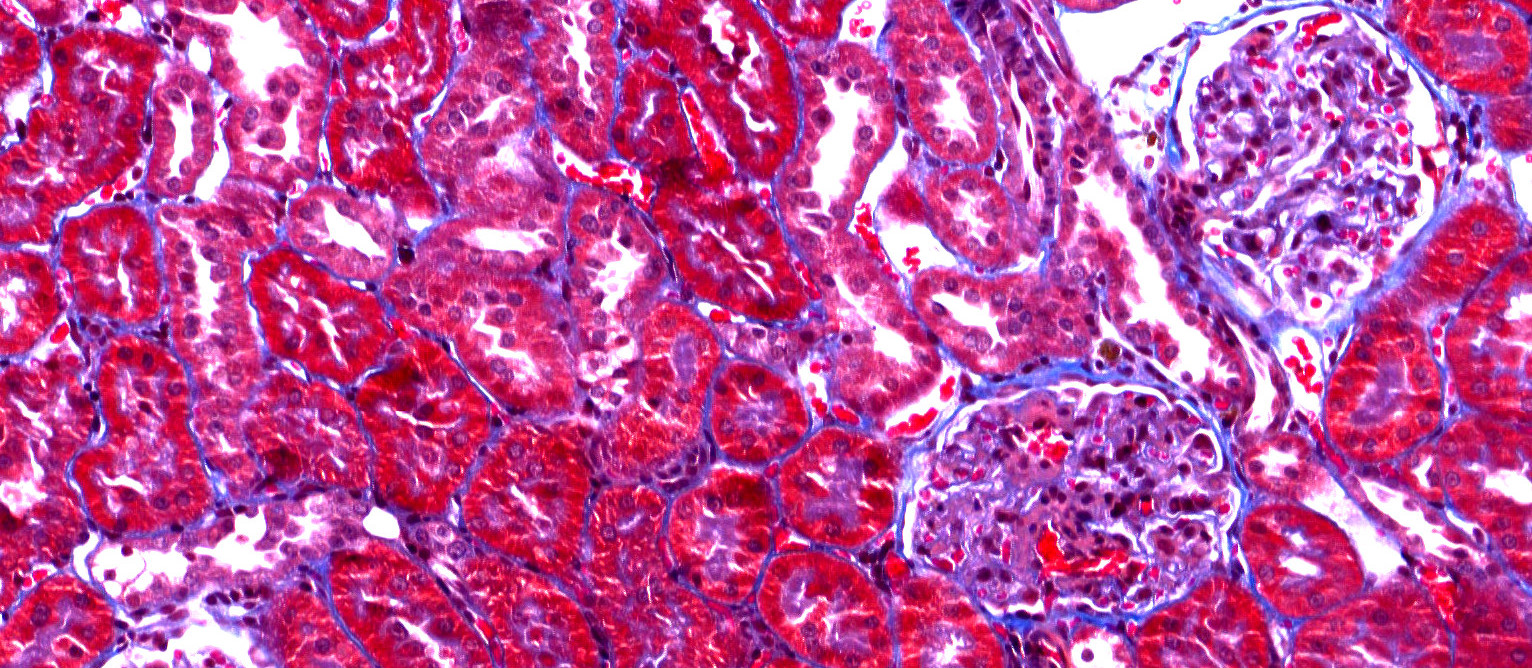

Supplement: Supplementary file 15 [file DataSheet7.ZIP › Fig 1D-masson-DKD-21/21-5.jpeg]

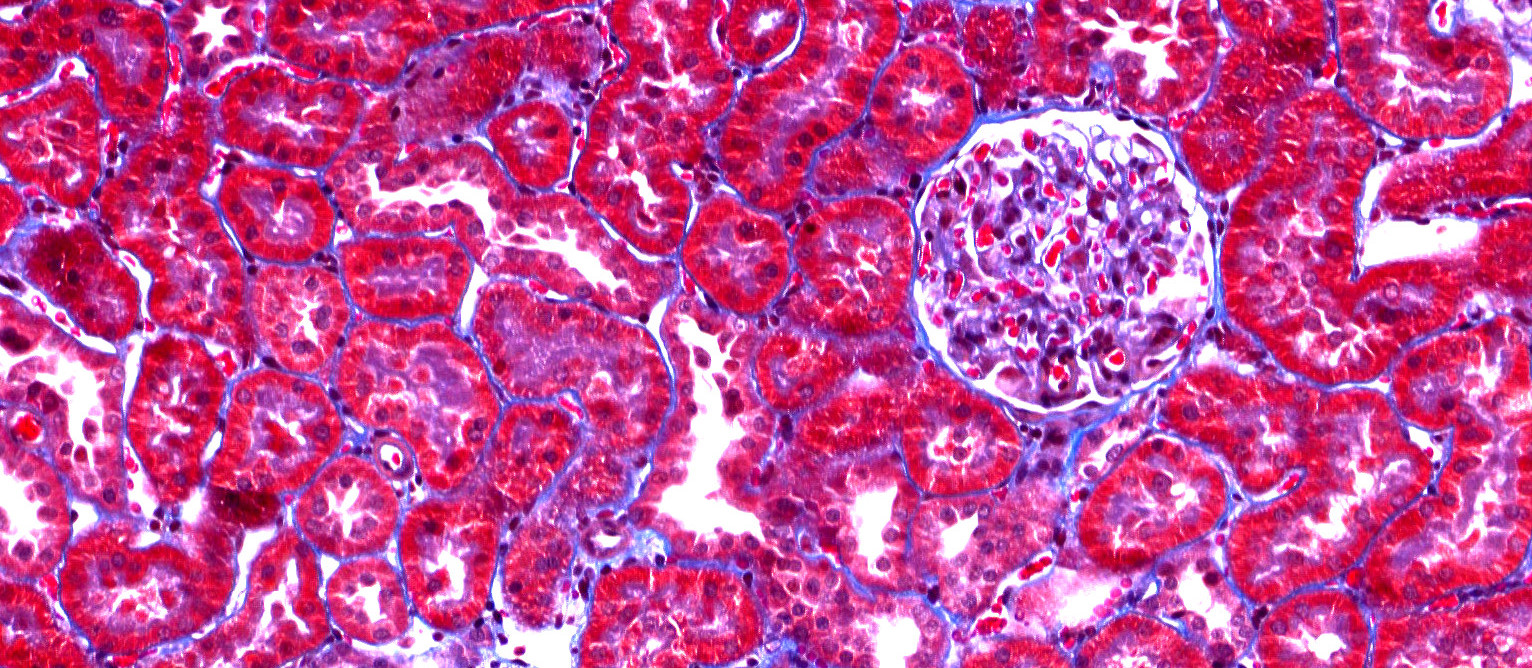

Supplement: Supplementary file 15 [file DataSheet7.ZIP › Fig 1D-masson-DKD-21/21-6.jpeg]

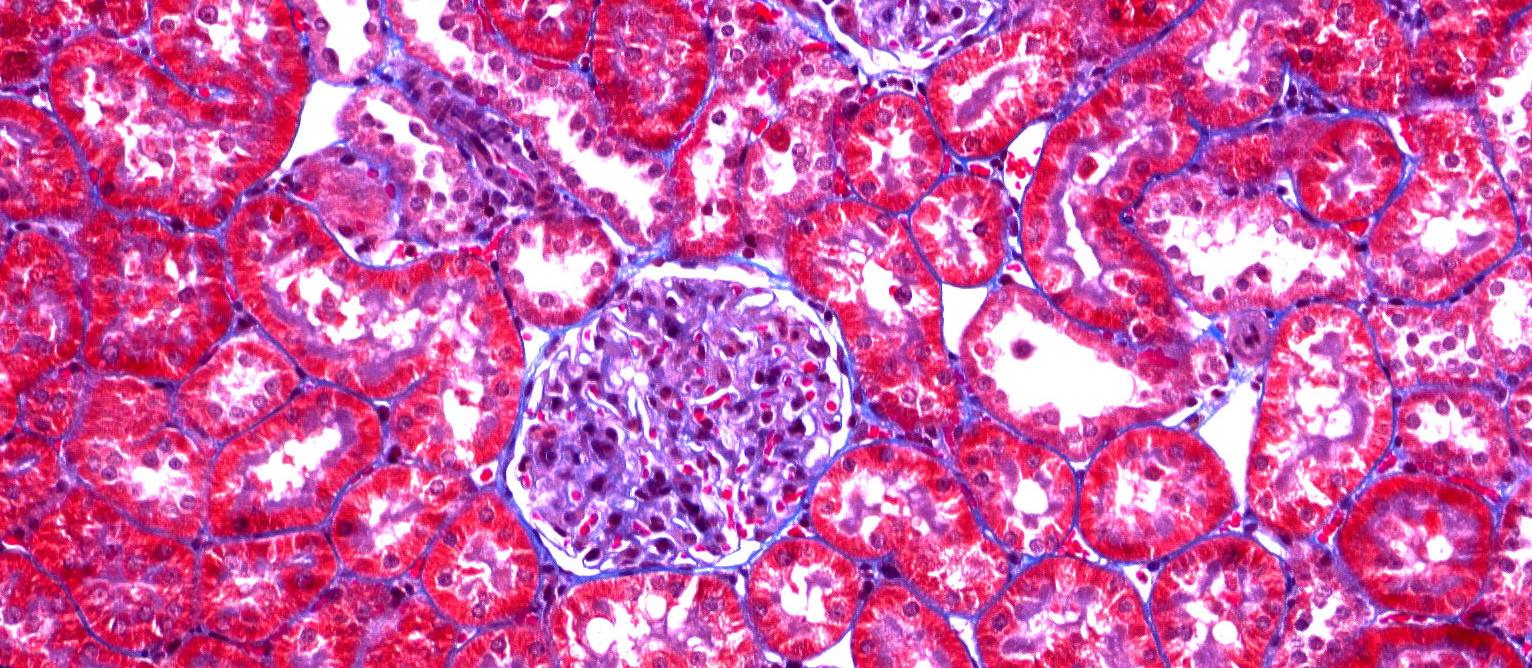

Supplement: Supplementary file 15 [file DataSheet7.ZIP › Fig 1D-masson-DKD-21/21-7.jpeg]

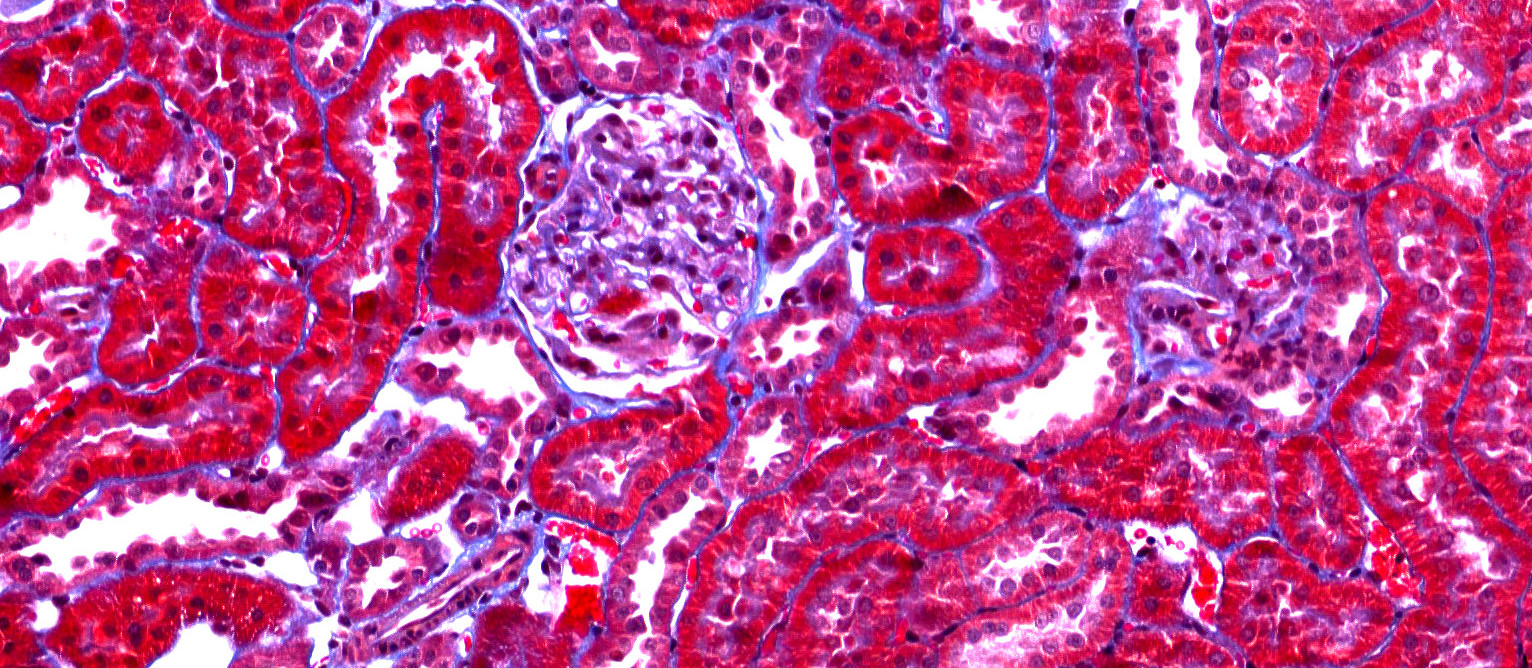

Supplement: Supplementary file 15 [file DataSheet7.ZIP › Fig 1D-masson-DKD-21/21-8.jpeg]

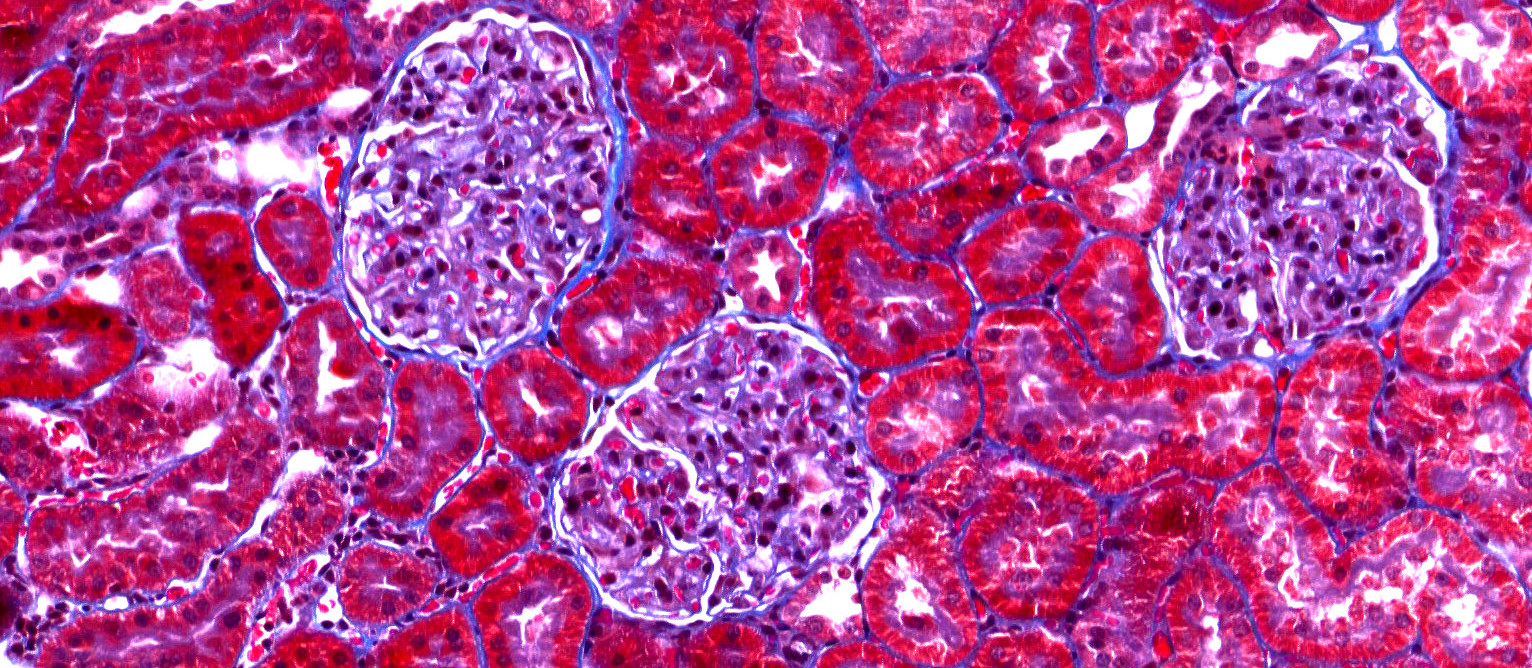

Supplement: Supplementary file 15 [file DataSheet7.ZIP › Fig 1D-masson-DKD-21/21-9.jpeg]

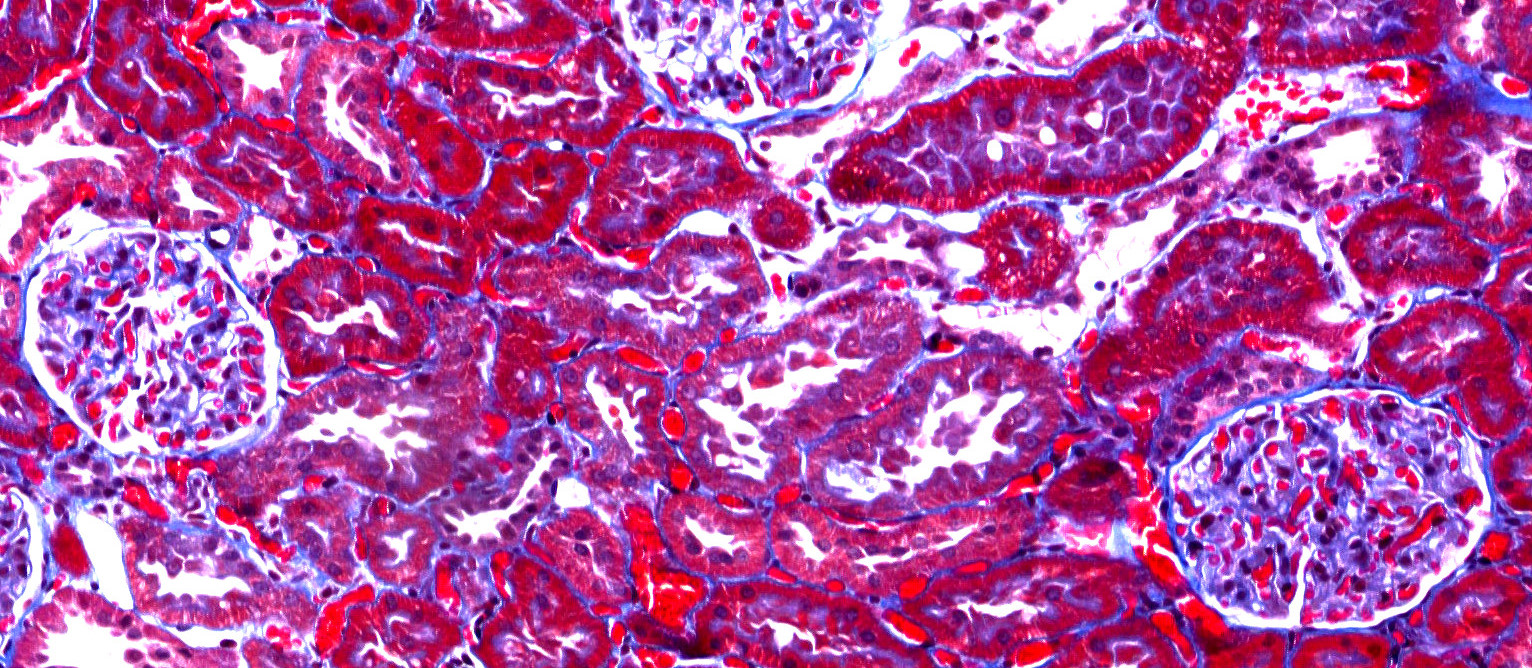

Supplement: Supplementary file 15 [file DataSheet7.ZIP › Fig 1D-masson-DKD-22/22-1.jpeg]

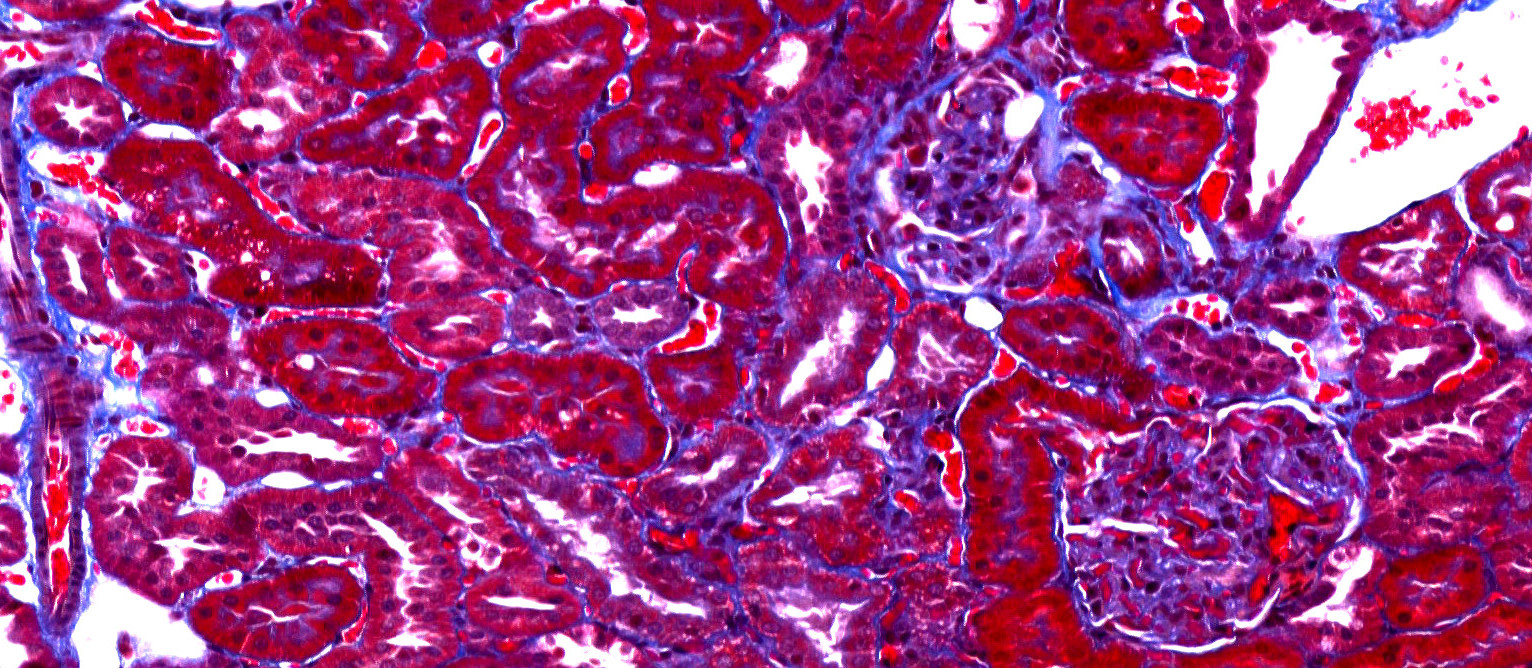

Supplement: Supplementary file 15 [file DataSheet7.ZIP › Fig 1D-masson-DKD-22/22-10.jpeg]

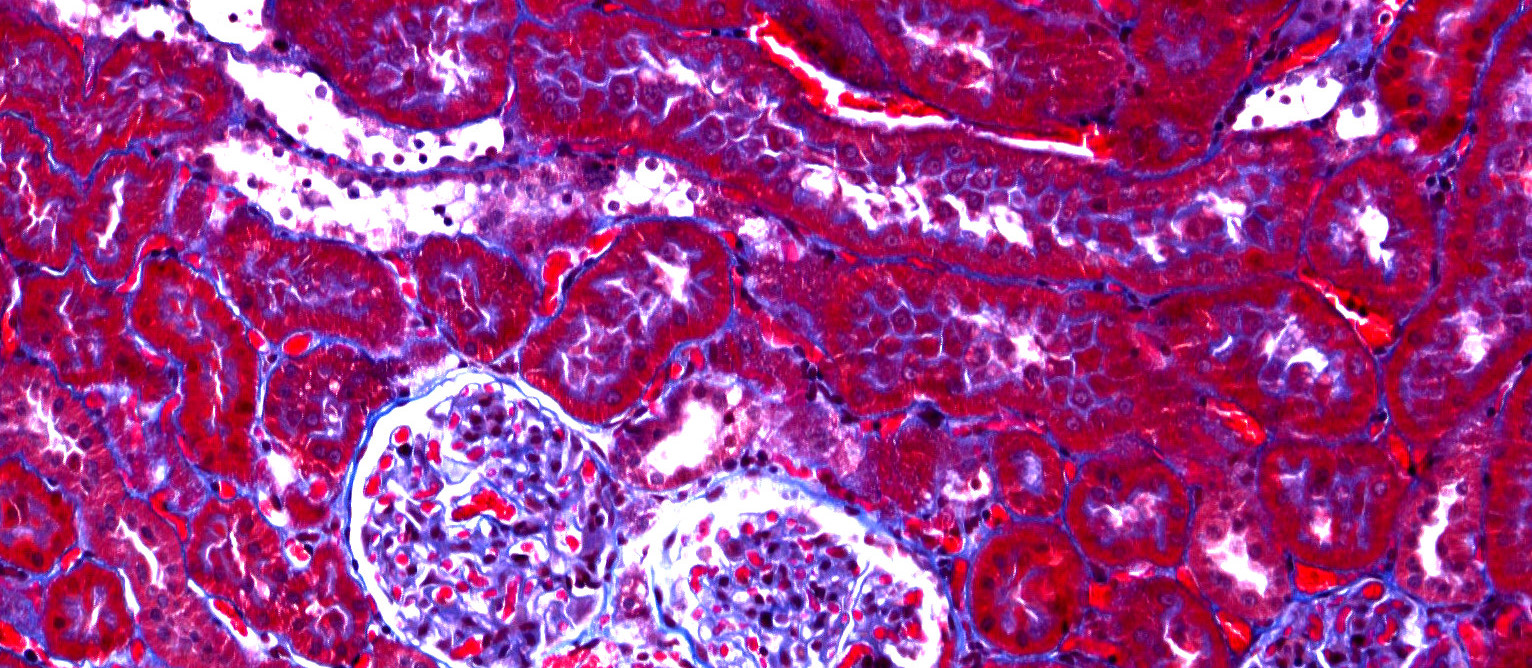

Supplement: Supplementary file 15 [file DataSheet7.ZIP › Fig 1D-masson-DKD-22/22-2.jpeg]

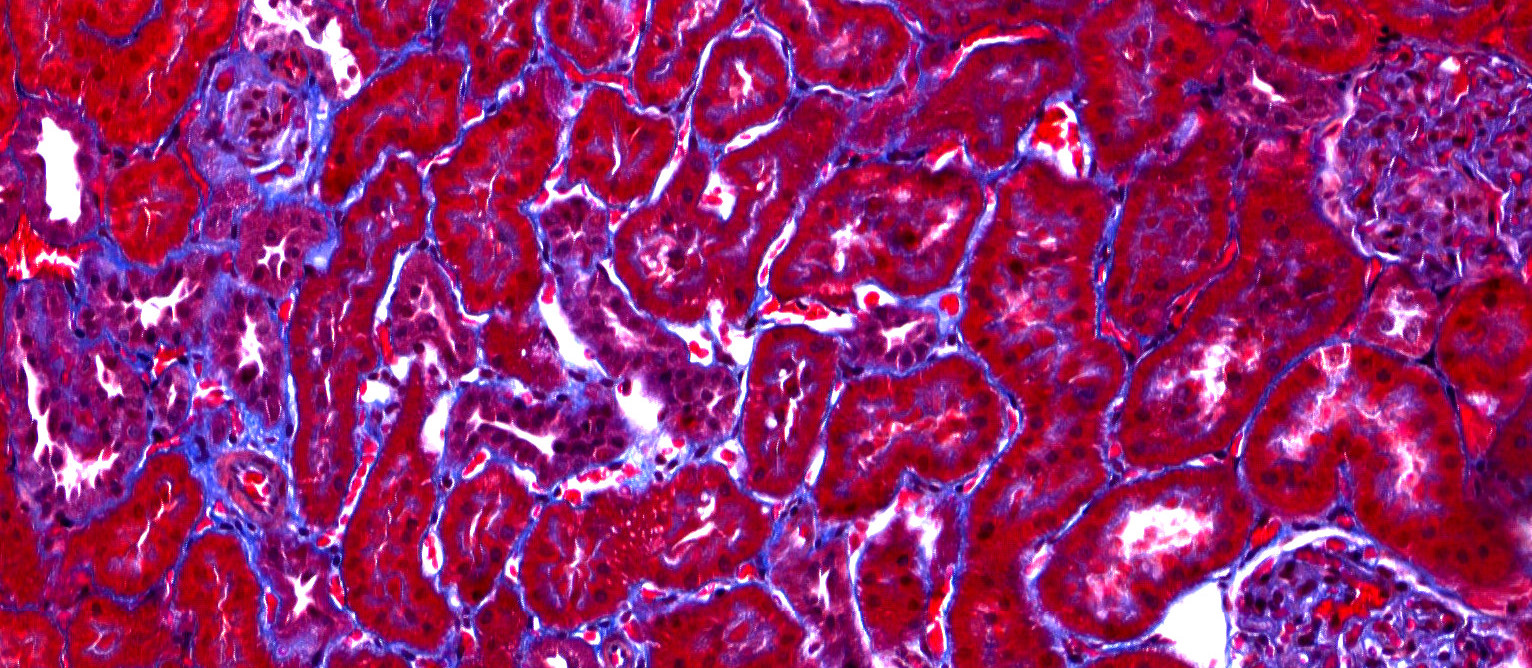

Supplement: Supplementary file 15 [file DataSheet7.ZIP › Fig 1D-masson-DKD-22/22-3.jpeg]

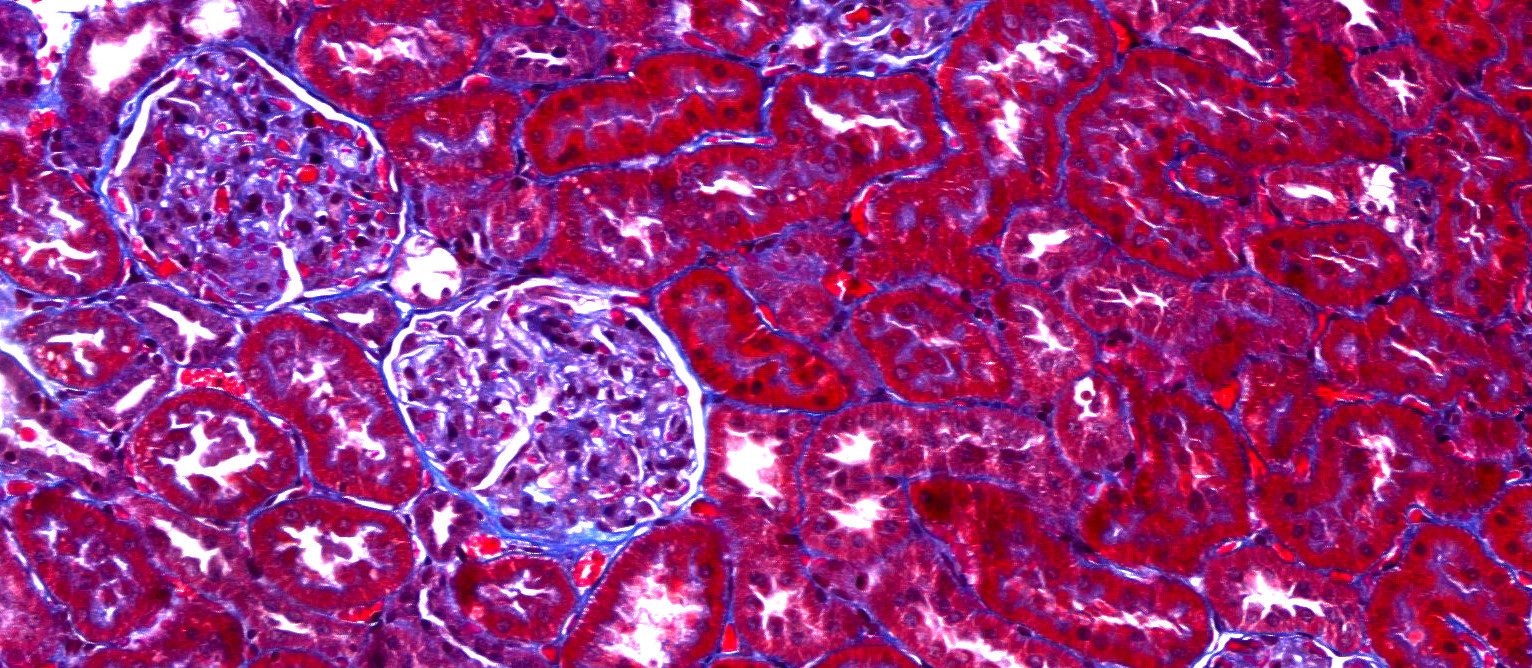

Supplement: Supplementary file 15 [file DataSheet7.ZIP › Fig 1D-masson-DKD-22/22-4.jpeg]

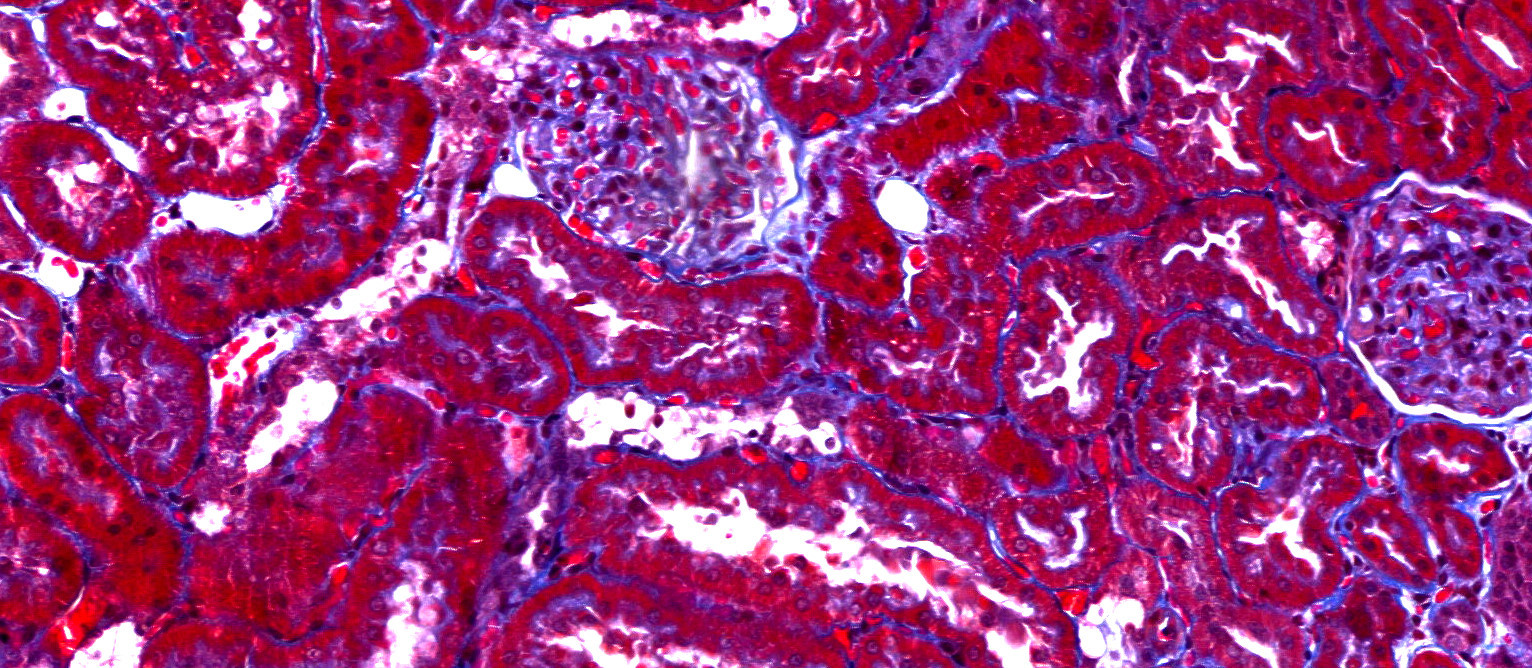

Supplement: Supplementary file 15 [file DataSheet7.ZIP › Fig 1D-masson-DKD-22/22-5.jpeg]

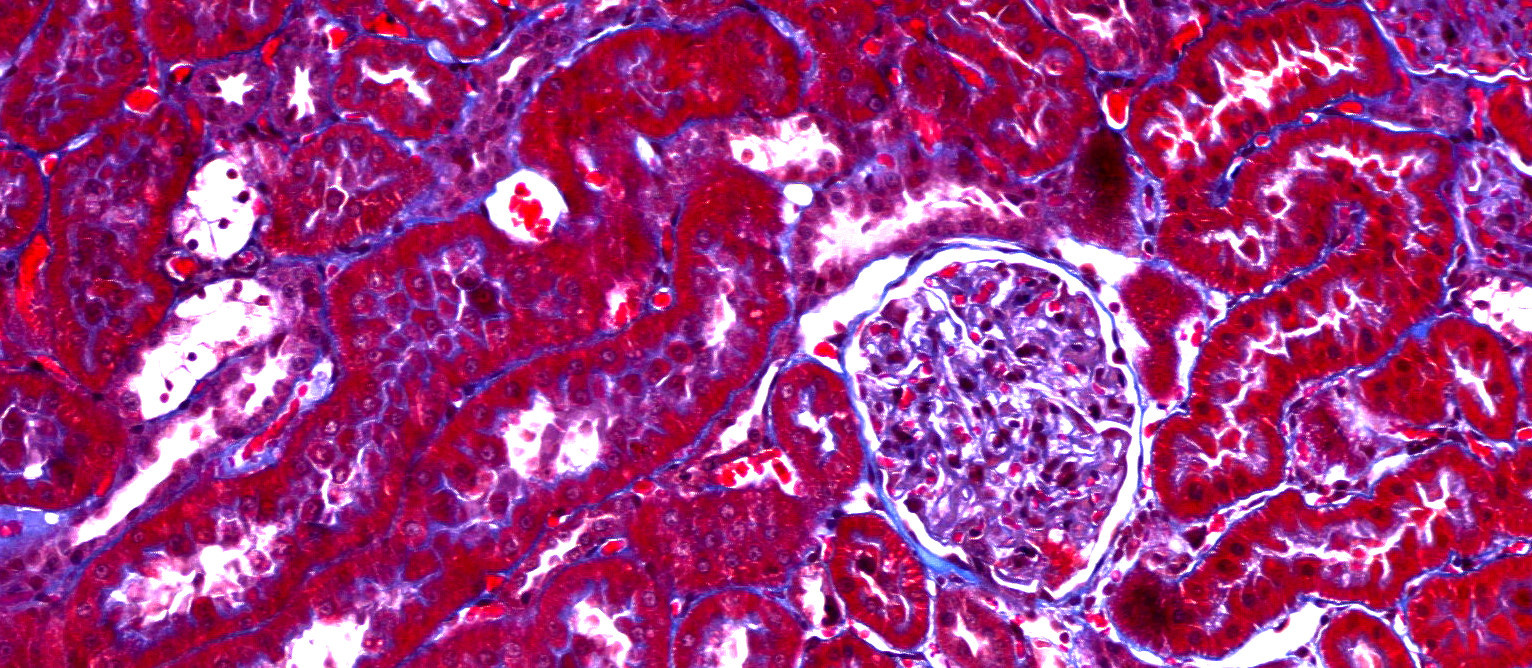

Supplement: Supplementary file 15 [file DataSheet7.ZIP › Fig 1D-masson-DKD-22/22-6.jpeg]

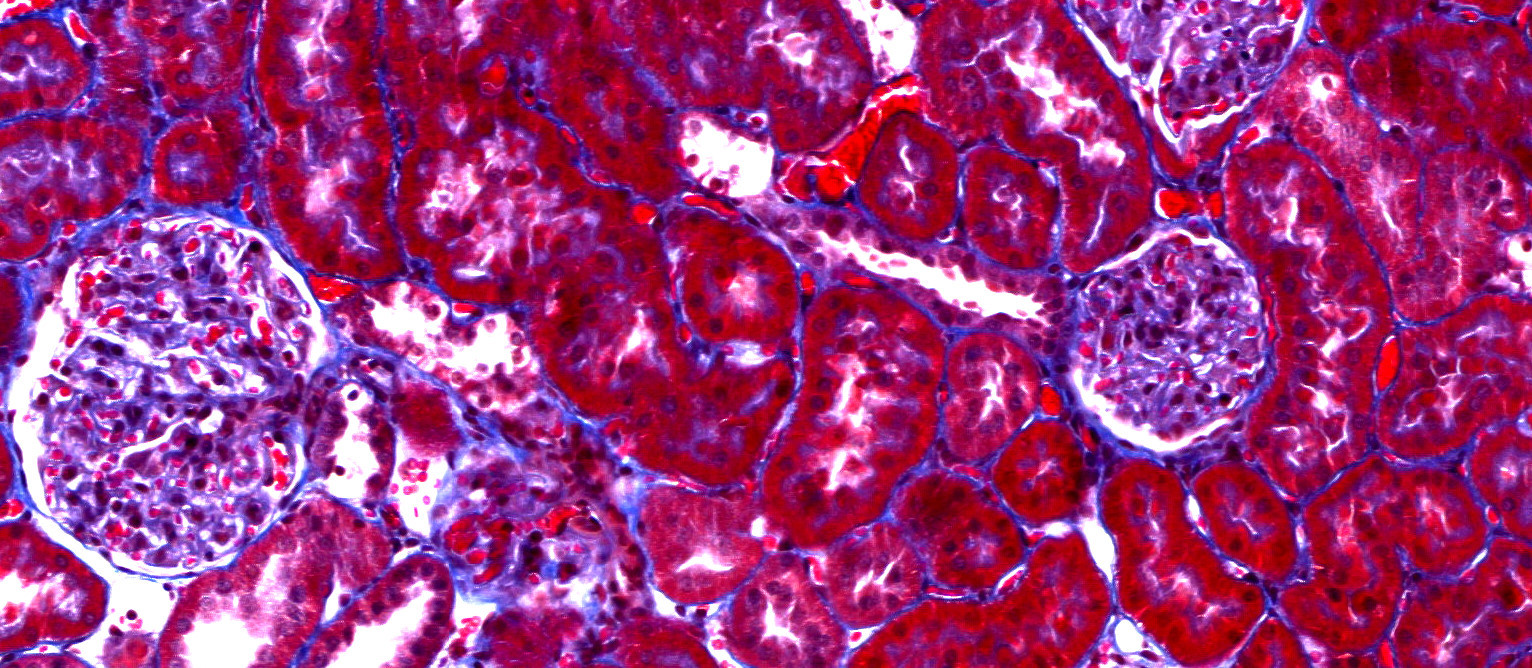

Supplement: Supplementary file 15 [file DataSheet7.ZIP › Fig 1D-masson-DKD-22/22-7.jpeg]

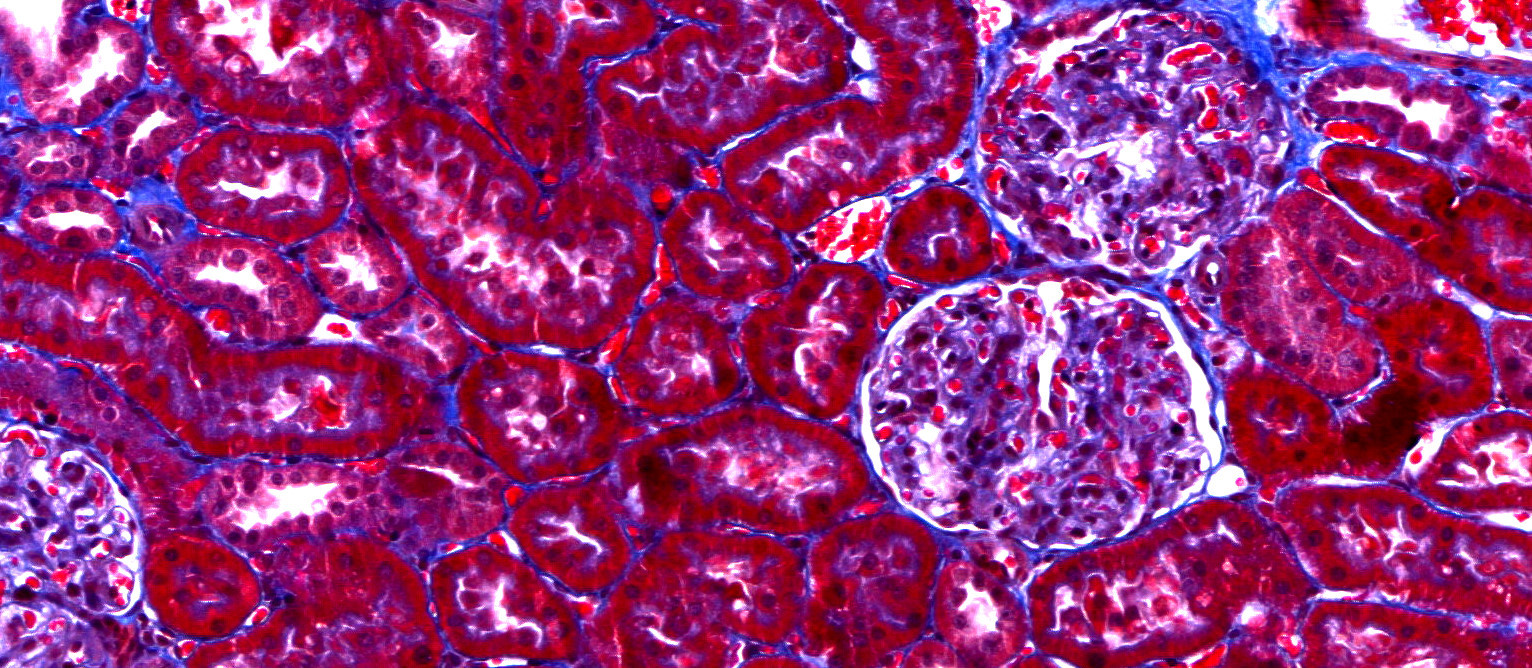

Supplement: Supplementary file 15 [file DataSheet7.ZIP › Fig 1D-masson-DKD-22/22-8.jpeg]

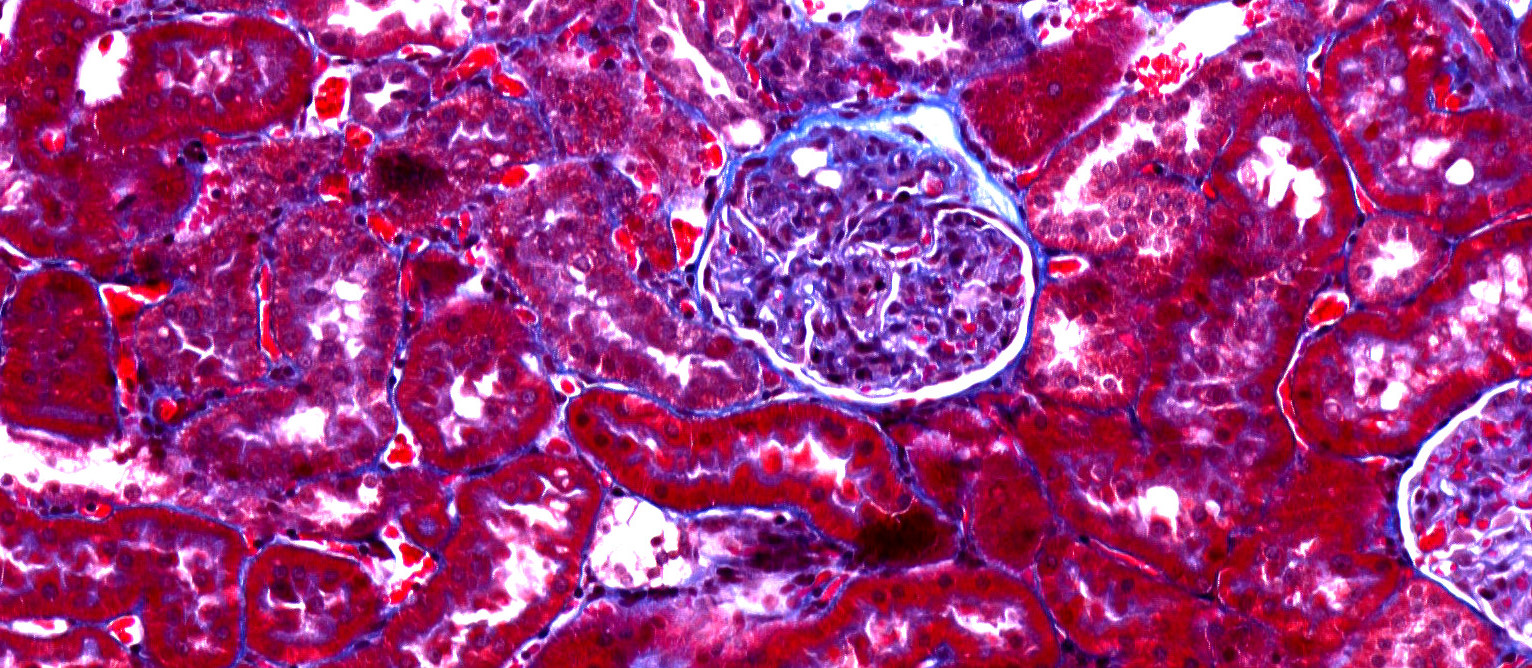

Supplement: Supplementary file 15 [file DataSheet7.ZIP › Fig 1D-masson-DKD-22/22-9.jpeg]

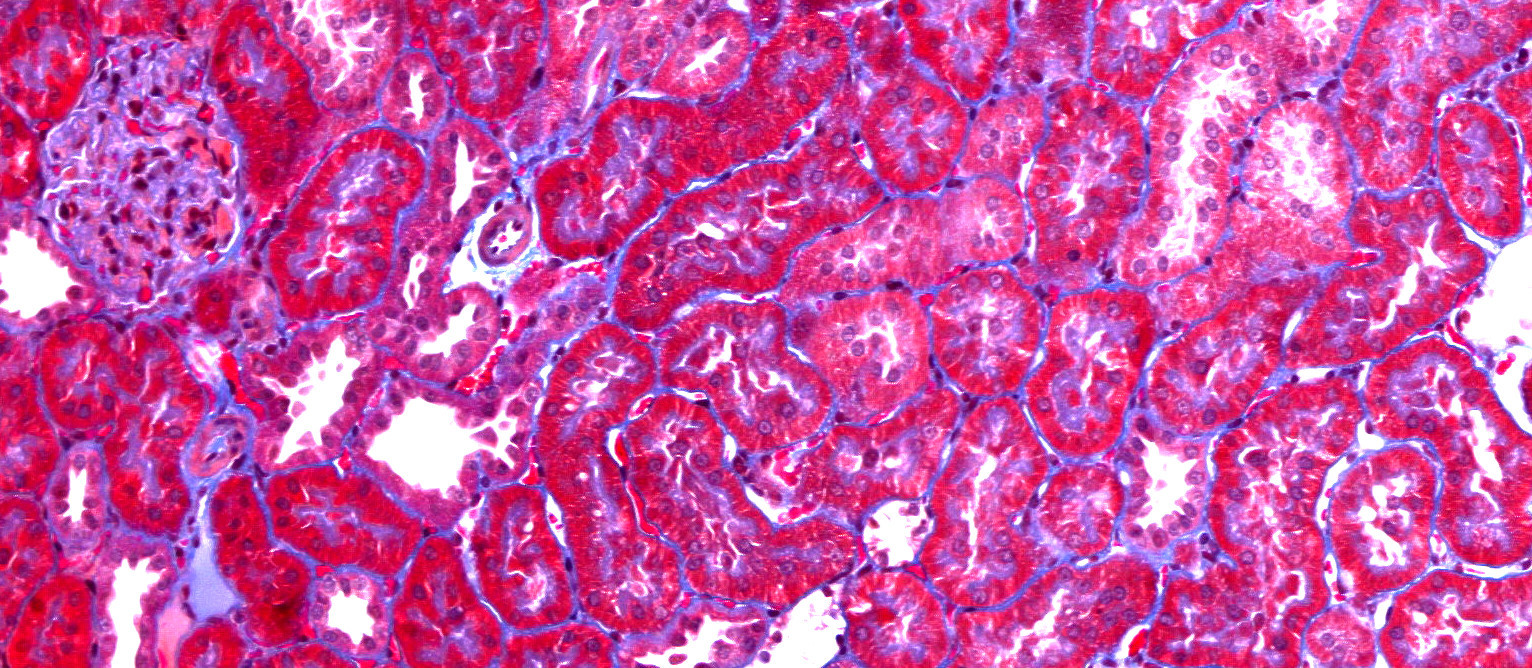

Supplement: Supplementary file 15 [file DataSheet7.ZIP › Fig 1D-masson-DKD-23/23-1.jpeg]

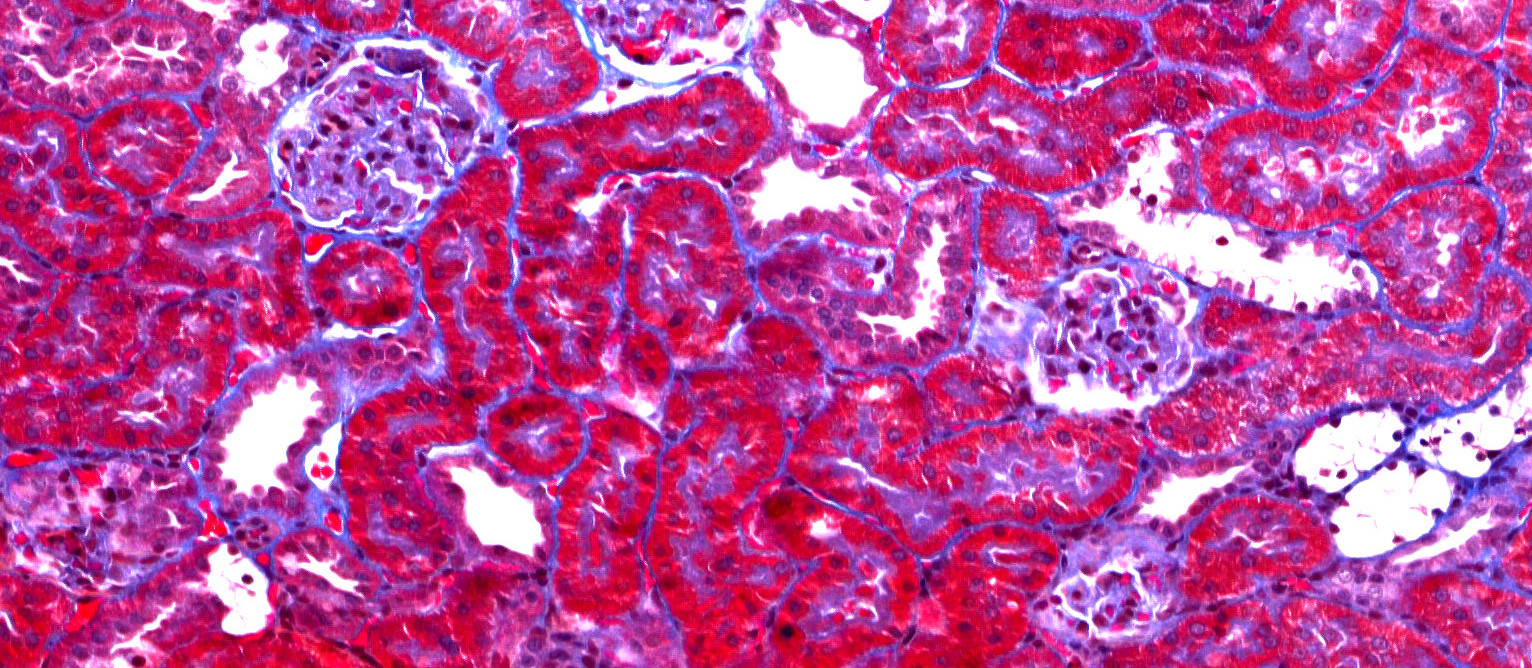

Supplement: Supplementary file 15 [file DataSheet7.ZIP › Fig 1D-masson-DKD-23/23-10.jpeg]

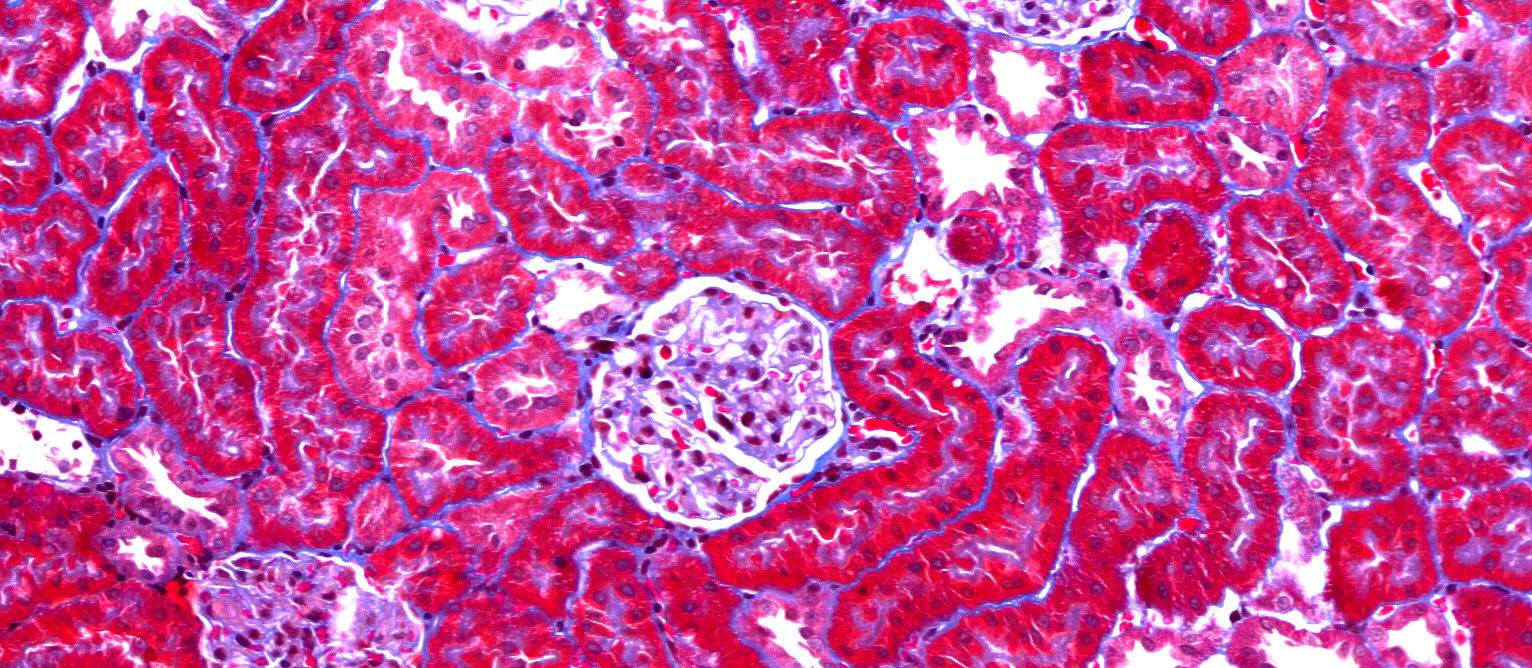

Supplement: Supplementary file 15 [file DataSheet7.ZIP › Fig 1D-masson-DKD-23/23-2.jpeg]

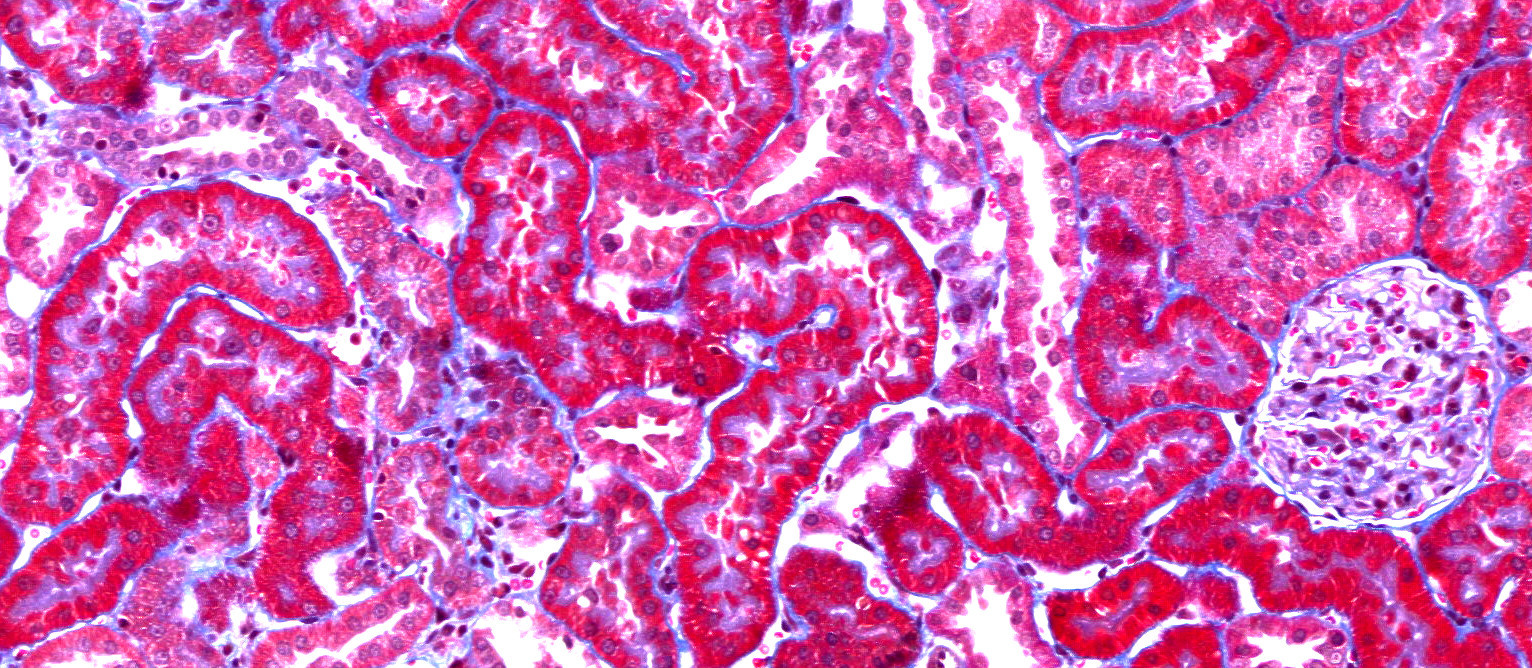

Supplement: Supplementary file 15 [file DataSheet7.ZIP › Fig 1D-masson-DKD-23/23-3.jpeg]

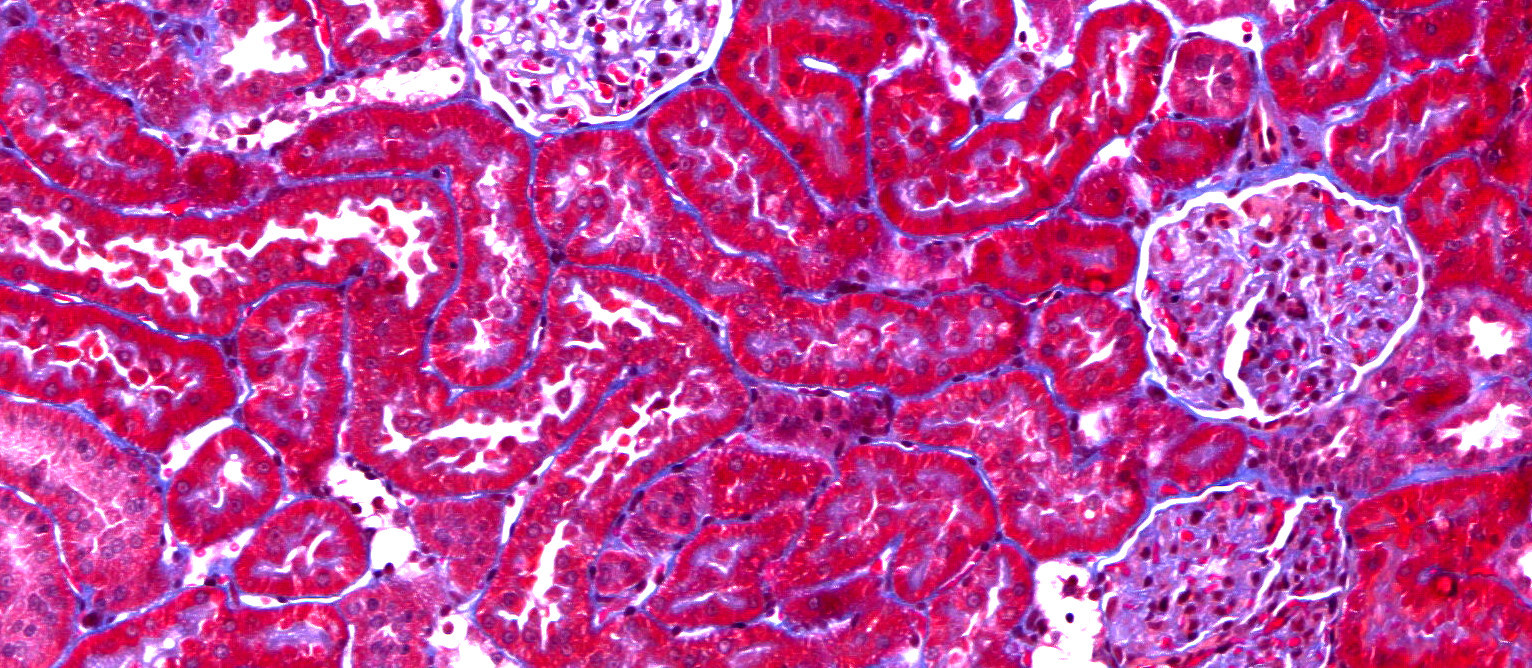

Supplement: Supplementary file 15 [file DataSheet7.ZIP › Fig 1D-masson-DKD-23/23-4.jpeg]

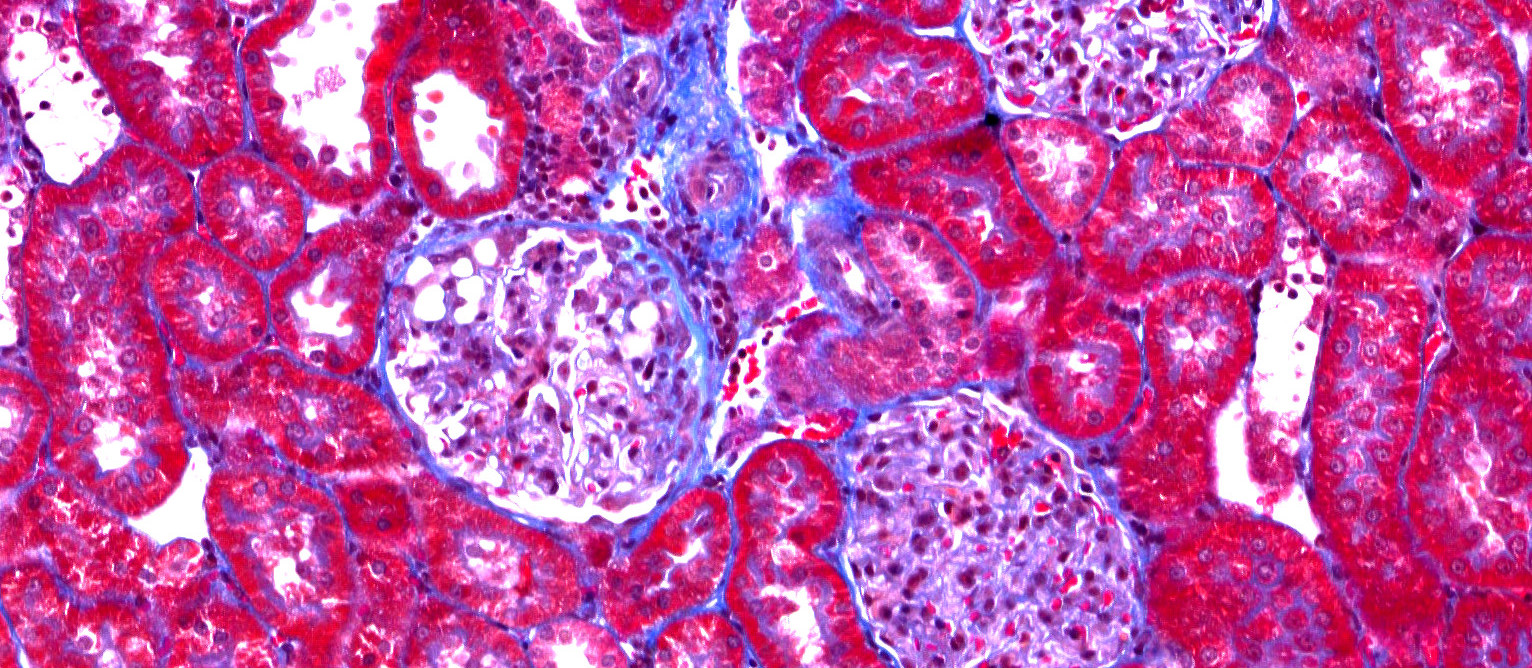

Supplement: Supplementary file 15 [file DataSheet7.ZIP › Fig 1D-masson-DKD-23/23-5.jpeg]
