# Supplementary figures and images for: Characteristics of Serum Metabolites and Gut Microbiota in Diabetic Kidney Disease (part 13 of 13)
Source: Front Pharmacol. 2022 Apr 14;13:872988. doi: 10.3389/fphar.2022.872988 (PMC9084235; doi:10.3389/fphar.2022.872988)

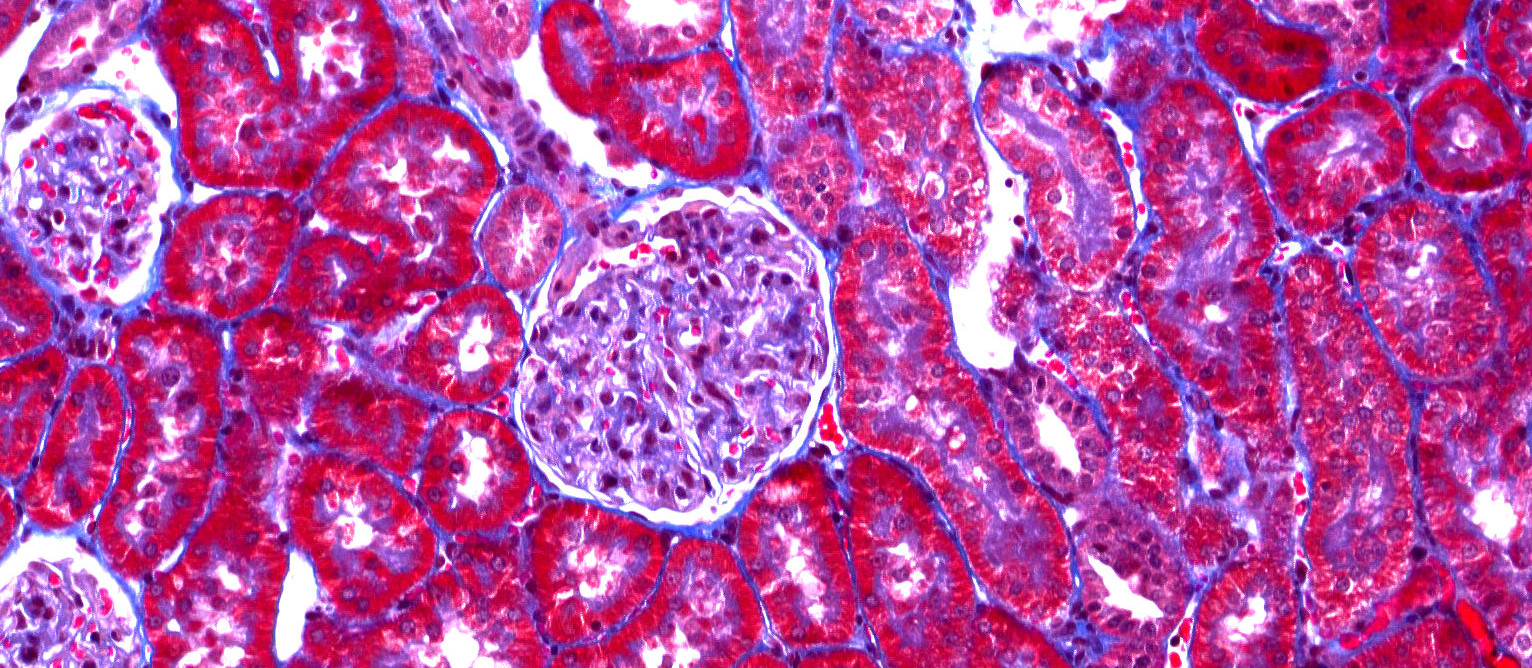

Supplement: Supplementary file 15 [file DataSheet7.ZIP › Fig 1D-masson-DKD-23/23-6.jpeg]

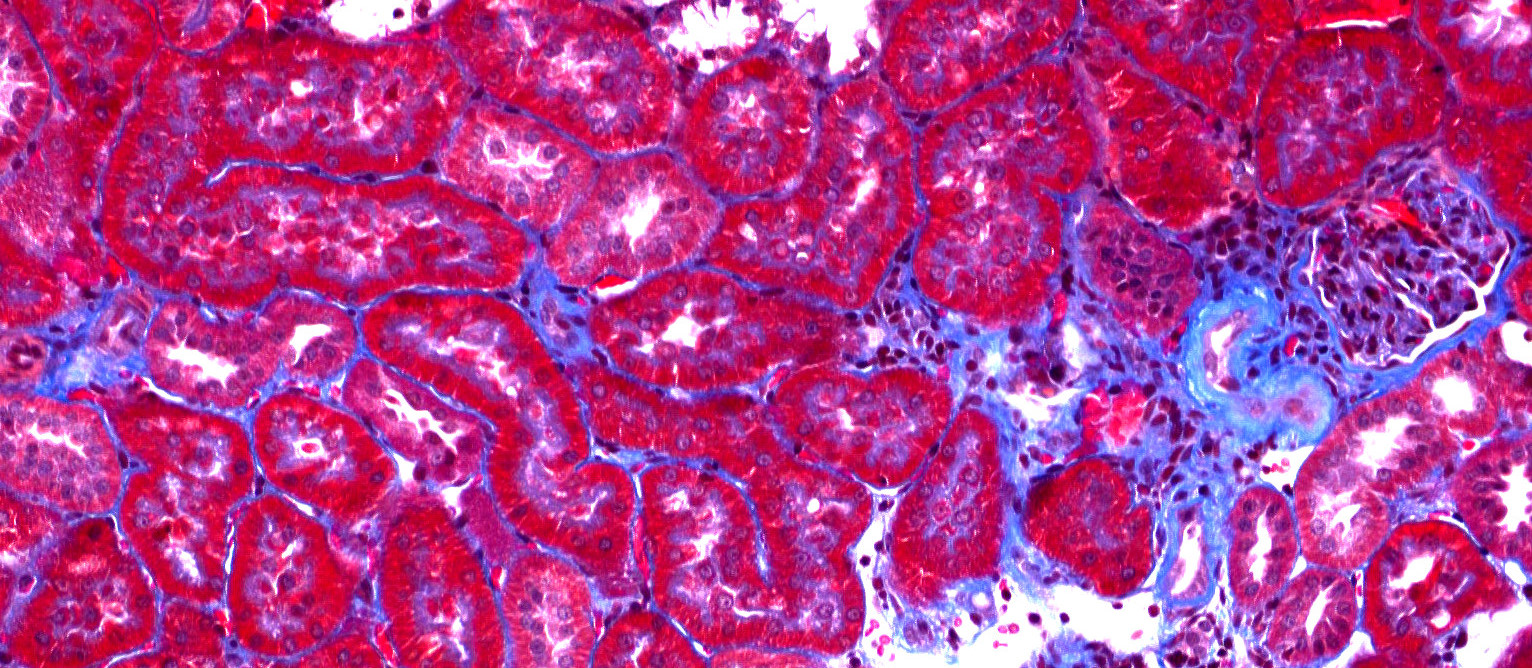

Supplement: Supplementary file 15 [file DataSheet7.ZIP › Fig 1D-masson-DKD-23/23-7.jpeg]

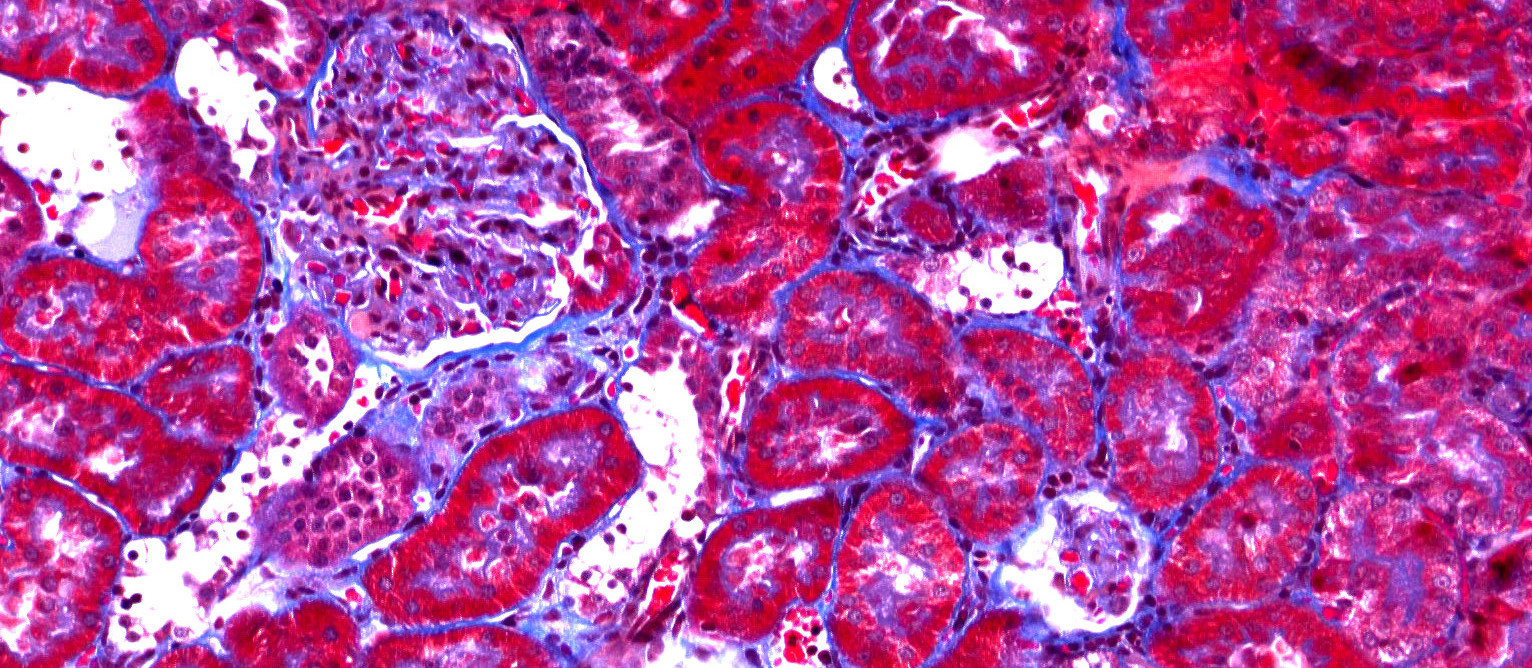

Supplement: Supplementary file 15 [file DataSheet7.ZIP › Fig 1D-masson-DKD-23/23-8.jpeg]

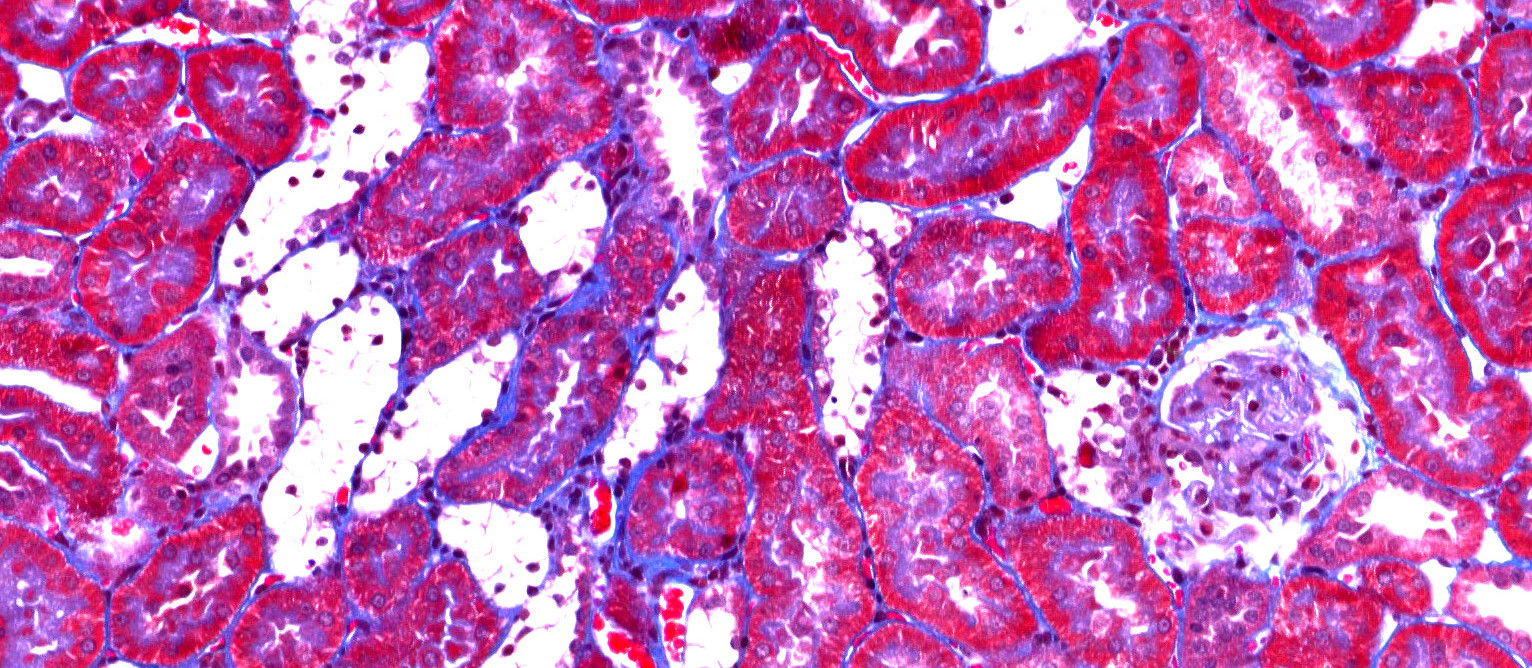

Supplement: Supplementary file 15 [file DataSheet7.ZIP › Fig 1D-masson-DKD-23/23-9.jpeg]
